# Inter-tissue coexpression network analysis reveals *DPP4* as an important gene in heart to blood communication

**DOI:** 10.1186/s13073-016-0268-1

**Published:** 2016-02-09

**Authors:** Quan Long, Carmen Argmann, Sander M. Houten, Tao Huang, Siwu Peng, Yong Zhao, Zhidong Tu, The GTEx Consortium, Jun Zhu

**Affiliations:** Department of Genetics and Genomic Sciences, Icahn School of Medicine at Mount Sinai, New York, NY 10029 USA; Icahn Institute of Genomics and Multiscale Biology, Icahn School of Medicine at Mount Sinai, New York, NY 10029 USA

## Abstract

**Background:**

Inter-tissue molecular interactions are critical to the function and behavior of biological systems in multicellular organisms, but systematic studies of interactions between tissues are lacking. Also, existing studies of inter-tissue interactions are based on direct gene expression correlations, which can’t distinguish correlations due to common genetic architectures versus biochemical or molecular signal exchange between tissues.

**Methods:**

We developed a novel strategy to study inter-tissue interaction by removing effects of genetic regulation of gene expression (genetic decorrelation). We applied our method to the comprehensive atlas of gene expression across nine human tissues in the Genotype-Tissue Expression (GTEx) project to generate novel genetically decorrelated inter-tissue networks. From this we derived modules of genes important in inter-tissue interactions that are likely driven by biological signal exchange instead of their common genetic basis. Importantly we highlighted communication between tissues and elucidated gene activities in one tissue inducing gene expression changes in others.

**Results:**

We reveal global unidirectional inter-tissue coordination of specific biological pathways such as protein synthesis. Using our data, we highlighted a clinically relevant example whereby heart expression of *DPP4* was coordinated with a gene expression signature characteristic for whole blood proliferation, potentially impacting peripheral stem cell mobilization. We also showed that expression of the poorly characterized *FOCAD* in heart correlated with protein biosynthetic processes in the lung.

**Conclusions:**

In summary, this is the first resource of human multi-tissue networks enabling the investigation of molecular inter-tissue interactions. With the networks in hand, we may systematically design combination therapies that simultaneously target multiple tissues or pinpoint potential side effects of a drug in other tissues.

**Electronic supplementary material:**

The online version of this article (doi:10.1186/s13073-016-0268-1) contains supplementary material, which is available to authorized users.

## Background

Tissues in multicellular organisms do not operate in isolation, but interact with other tissues and organ systems. Examples include the control of adrenal glucocorticoid secretion by the hypothalamic-pituitary-adrenal axis and the regulation of glucose homeostasis by the endocrine pancreas. Although abundant large-scale data on protein–protein interactions and gene–gene interactions [1–3] in single tissues have been reported, large scale unbiased interactions across tissues are currently less well characterized. An unbiased picture of interactions between tissues in humans will provide essential insights into human biology in health and disease and further assist in the development of treatments for complex disease. For example, therapeutically targeting a gene in one tissue may cause side effects or beneficial effects in distant tissues. Therefore, a systematic method of uncovering tissue–tissue interactions in an unbiased way is urgently needed.

Previously we reported an inter-tissue view of obesity in mice with respect to molecular states that are associated with physiological states using gene expression in adipose, liver and hypothalamus from an F2 progeny [4]. Currently, the overall picture of tissue–tissue interactions at the transcriptional level in healthy humans remains unknown. The Genotype-Tissue Expression (GTEx) project [5] aims to create a comprehensive public atlas of gene expression and its regulation across multiple human tissues. This project aims to release genotype and transcriptome data generated by RNA-Seq in more than 30 tissues of approximately 900 post-mortem donors [6]. In its pilot phase, expression data for nine tissues from 185 subjects are available. In this dataset, multiple tissues have been profiled within each subject, enabling us to perform an inter-tissue interaction analysis (Additional file 1). To our knowledge this is the first comprehensive resource of multi-tissue human expression data enabling the investigation of molecular tissue–tissue interactions in healthy people.

**Fig. 1:**
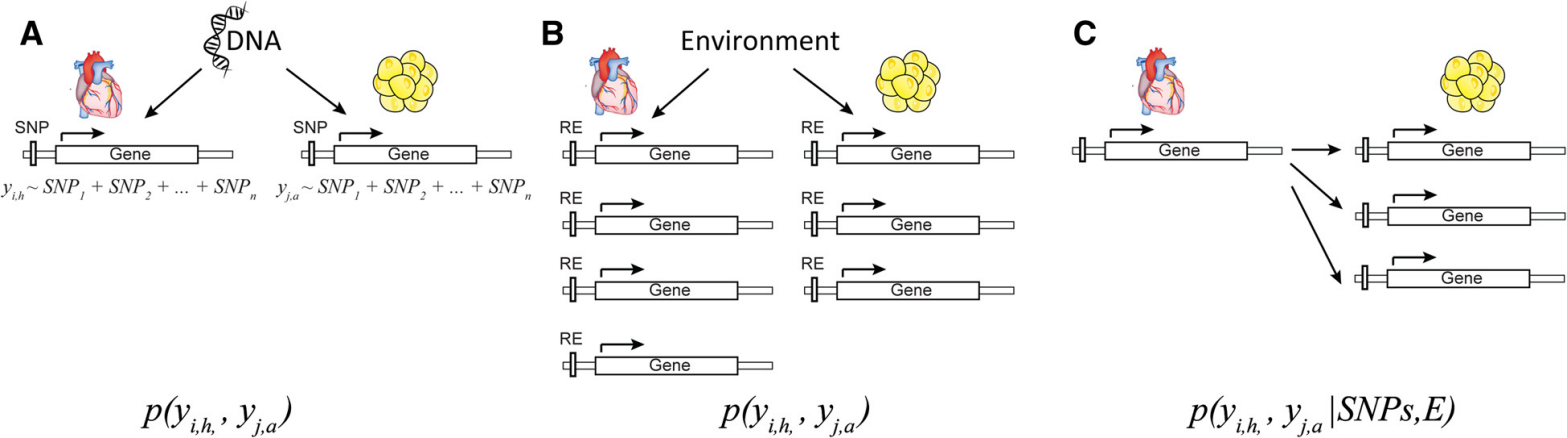
Inter-tissue correlation of expression levels of two genes in different tissues due to responding to the same genetic variation (**a**); responding to the same environmental variation (**b**); or biological signal exchanges between two tissues (**c**)

**Fig. 2:**
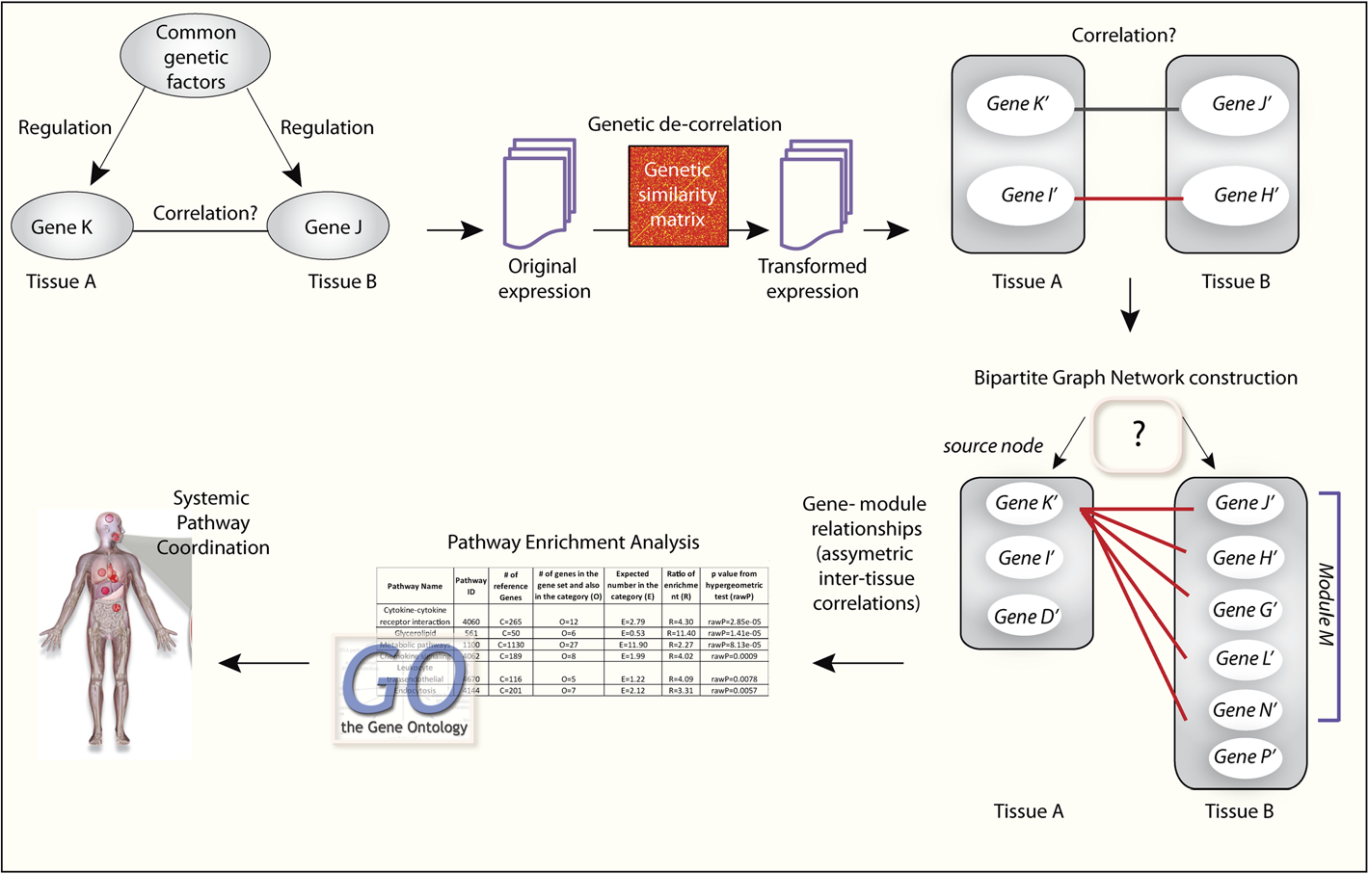
Schema depicting the novel strategy for generating genetically decorrelated inter-tissue networks and deriving modules of genes important in inter-tissue interactions that are driven by biological signal exchanges instead of their common genetic basis. The *black line* between gene *K’* and *J’* denotes the undesired correlation due to shared genetic basis, which will be removed after decorrelation. *Red lines* denote genuine correlations due to non-genetic biological interactions. A gene module detection strategy is used to claim a set of genes, namely module *M*, in tissue *B* is correlated with a gene, namely *K’* in tissue *A*. We hypothesize that asymmetric inter-tissue correlations, whereby a gene in one tissue is asymmetrically correlated to a group of genes in a second tissue, may indicate interesting unidirectional interactions between tissues. If gene *K’* is correlated with module *M* while the average number of correlations between members in *M* and other genes in tissue *A* is much lower than the links between *K’* and *M*, we report this asymmetric association. Biological function associated with these unidirectional interactions is determined by gene ontology enrichment analysis, which provides insight into systemic pathway coordination

In this study, we aim to distinguish between inter-tissue interactions caused by different factors (Fig. 1). Expression levels of two genes in two tissues, e.g., *y*_*i*,*h*_ for gene *i* in the heart, and *y*_*j*,*a*_ for gene *j* in adipose tissue, are correlated because they are regulated independently by the same genetic locus (Fig. 1a), or they respond independently to the same environmental cues (Fig. 1b), or gene *i* in the heart signals to the adipose and regulates expression of gene *j* (Fig. 1c). Transcriptional regulation of gene expression in different tissues by common genetic or environmental perturbations has been well studied [7]. A comparison of co-expression modules of genes within individual tissues has revealed conservation of biological pathways responding to common genetic or environmental signals [8–10]. However, signaling between tissues via biological signals that regulate transcription has not been extensively studied. Here we developed a novel strategy for generating a global view of tissue–tissue interactions at the transcriptional level (Fig. 2). To derive modules of genes important in inter-tissue interactions that are driven by biological signal exchanges instead of their common genetic basis, we performed a *genetic-decorrelated* tissue–tissue coexpression (*gd*TTC) network analysis for all pairs of tissues. Genetic decorrelation is a method to remove all genetic contribution from *y*_*i*,*a*_ by regression *y*_*i*,*a*_ ∼ *SNP*_*S*_ + *y*_*i*,a_^*^. The resulting *y*_*i*,a_^*^ is expected to be independent from genetic regulation. Following the genetic decorrelation of all gene expression data, we performed standard bipartite clustering in order to identify clusters of genes and derived biological insight related to the inter-tissue correlations. To dissect sub-clusters within a cluster, we developed a selection algorithm for identifying asymmetric inter-tissue interactions, called gene-to-module detection, to find sub-clusters regulated by a few genes noted as key regulators and their biological function.

Here we provide an unbiased global view of the asymmetric molecular interactions between pairs of nine human tissues, which reflect biological processes that are being communicated and coordinated amongst tissues rather than common responses to genetic variations. We show that some pathways, such as the “establishment of protein localization to organelle” and “translational initiation”, are the most prevalent pathways observed amongst all asymmetric inter-tissue coexpression networks identified. On the other extreme is the observation that a wide variety of pathways are found enriched only in asymmetric inter-tissue coexpression networks between a single pair of tissues. Our tissue–tissue coexpression network analysis revealed novel asymmetric inter-tissue interactions of which we highlight two. As a clinically relevant example, we show an asymmetric inter-tissue sub-network whereby heart *DPP4* is coexpressed with a module of cell cycle-related genes in whole blood, suggesting *DPP4* expression in the heart coordinates whole blood proliferation, thereby potentially regulating stem cell trafficking and mobilization to peripheral tissues. As a second example, we show that expression of the poorly characterized *FOCAD* in the heart correlates with protein biosynthetic processes in the lung.

## Methods

### Biospecimens

Tissue samples collected in the GTEx pilot study [5] were from 237 post-mortem donors. A full description can be found in the Supplementary Materials of the GTEx pilot study [5]. Briefly, sampled tissues were preserved in PAXgene® tissue kits (PreAnalytiX®). Samples were fixed for a minimum of 2–4 hours, and then placed in the stabilizer buffer for shipping to the GTEx Comprehensive Biospecimen Resource (CBR). The mean sample ischemic time was around 420 minutes [5]. Supporting quality documents and workflows for the project are available at http://biospecimens.cancer.gov/resources/sops/default.asp.

Note that tissues collected in the GTEx study are postmortem. Melé et al. [11] show that gene expression profiles in the GTEx study are similar to the profiles of corresponding tissues from living donors, and tissue profile classifiers based on profiles in the GTEx study can accurately classify expression profiles of tissues from living donors in multiple independent studies. Melé et al. [11] also show that sample ischemic time in the GTEx study has a small impact on gene expression but a larger impact on splicing.

### RNA sequencing

Total RNA was isolated from PAXgene® tubes using proper Qiagen kits following the manufacture's specification. Detailed experimental procedures for blood, PAXGene-preserved, and frozen tissue samples can be found in the Supplementary Materials of the GTEx pilot study [5]. All samples sequenced had a RNA integrity number (RIN) value of 6.0 or higher and at least 1 μg of total RNA.

RNA sequencing was performed using a standard non-strand specific protocol with poly-A selection of mRNA. Non-strand-specific RNA sequencing was performed at the Broad Institute using a large-scale, automated variant of the Illumina Tru Seq™ RNA Sample Preparation protocol (Illumina: TruSeq Protocol Info). Detailed experimental procedures can be found in the Supplementary Materials of the GTEx pilot study [5].

RNA-seq data were aligned with TopHat version v1.4.1 to the UCSC human genome release version hg19 (Genome Reference Consortium GRCh37). Gencode version 12 was used as a transcriptome model for the alignment as well as all gene and isoform quantifications. Unaligned reads were merged back in to create a final bam. Gencode v12 annotates a total of 53,934 genes, including 20,110 protein coding genes, 11,790 long non-coding RNAs (lncRNAs), and 12,869 pseudogenes. Expression levels were produced at the gene level in RPKM units using RNA-SeQC. Samples with fewer than 10 million mapped reads were removed.

### Data availability

All primary sequence and clinical data files, and any other protected data, are deposited in and available from dbGaP (http://www.ncbi.nlm.nih.gov/gap) (phs000424).

### RNAseq data preprocessing

The RNAseq data used for the analyses described in this manuscript were obtained from dbGaP (accession number phs000424.v2.p1) in Feb 2014. It is common practice to adjust known and hidden confounding factors in gene expression data before further analysis [12]. We used PEER factors [13] to represent hidden confounding factors. While removing too many PEER factors increases the risk of filtering out genuine biological signals, adjusting too few PEER factors will obscure interesting discoveries with experimental covariates. To address this problem, we developed a systematic method to quantify the reasonable numbers of PEER factors to be adjusted in each tissue with respect to the optimal gene ontology (GO) enrichment (Notes and Figure S1 in Additional file 2). Many genes had low expression levels and low variances due to zero-valued expression levels in many subjects. As these genes may introduce artificially high correlations between them we removed genes in a tissue expression data set if their standard deviations were smaller than 20 % of the difference between their maximal and minimal expression levels.

### Decorrelating genetic components of gene expression

Using the standard mixed model notations, a gene expression level (e.g., gene *i*) in a given tissue (e.g., tissue *A*) can be modeled as Y_i,A_ = β_0_ + u_g_ + ε, where u_g_ is the random term representing the contribution from a large number of loci of small effects and ε is the residual representing other contributions. It is assumed that the total variance was normalized to be one, and both terms follow multivariate normal distribution. More specifically, we assume that ε ~ N(0, (1 − h_i,A_)I), and u_g_ ~ N(0, h_i,A_K_g_), where I is the identity matrix and *K*_*g*_ the kinship matrix estimated from the genotypes using standard procedure calculating Realized Relationship Matrix [14, 15]: the *r*th column and *s*th row of the matrix (that is, the similarity of *r*th and *s*th subject) is calculated by the formula ${K}_{g\left(r,s\right)}=\frac{1}{L}{\displaystyle \sum_{l=1}^L\frac{\left({g}_{l,r}-{p}_l\right)\left({g}_{l,s}-{p}_l\right)}{\left(1-{p}_l\right){p}_l}}$, where *L* is the number of available SNPs, *p*_*l*_ is the allele frequency of the SNP *l*, and *g*_*l*,*r*_ is the genotype coded as 0, 1, and 2, corresponding to homozygote, heterozygote, and the other homozygote. Here ${h}_{i,A}=\frac{Var\left({\mu}_g\right)}{Var\left({\mu}_g\right)+Var\left(\varepsilon \right)}$ is the variance component of expression level of gene *i* in tissue *A* that can be explained by genotype, a parameter referred to as “pseudo-heritability” in mixed model literals. We used FaST-LMM method to estimate *h*_*i,A*_ [14]. After the key parameter *h*_*i,A*_ has been estimated, we defined the Cholesky decomposition matrix *D* in the same way as Kang et al. [16]: $\mathrm{D}={\mathrm{U}}^{-\frac{1}{2}}\left({\mathrm{h}}_{\mathrm{i},\mathrm{A}}\mathrm{S}+\mathrm{I}\right)$ , where *U* is the eigenvector matrix of *K*_*g*_, and *S* is the diagnose matrix formed by eigenvalues of *K*_*g*_. Applying this matrix on the expression data yields transformed expression data for which the genetic contributions are decorrelated. More precisely, we calculate the transformed data by applying transformation Y_i,A_^*^ = DY_i,A_. Similarly, we generated Y*_j,B_ following the same procedure.

As *Y*_*i*,*A*_ is adjusted for all potential environmental covariants, and Y*_i,A_ and Y*_j,B_ contain no contribution from covariance of genome similarity, the inter-tissue interaction represented as a Spearman correlation Corr(Y*_i,A_, Y*_j,B_*)* is likely driven by biological regulation.

### *P* values and false discovery rate estimation of the correlations

Following the standard method, for any given Spearman correlation *r* with sample size *n*, we calculated a *t*-statistic using the formula $\mathrm{t}=\mathrm{r}\sqrt{\left(\mathrm{n}-2\right)/\left(1-{\mathrm{r}}^2\right)}$. Assuming that it follows a t-distribution with degree of freedom *df = n*-2, we obtained the *p* value of the correlation. A pair of genes is defined as being significantly correlated if and only if their correlations meet the following conditions: (1) the *p* values of correlations based on genetically decorrelated data are <10^−3^; (2) as transformation may introduce some artificial correlations, we set the *p* values of correlations based on the original data <1 × 10^−3.5^. False discovery rates (FDRs) were estimated by permutation tests. We randomly permute the sample labels of gene expression data, then re-calculated transformation matrix *D* and genetically decorrelated data. After calculating correlations based on original and genetically decorrelated data, we counted the number of pairs of genes that were significantly correlated as defined above. For each tissue pair, we performed a permutation test five times and estimated the average FDR for the tissue pair. FDRs for most tissue pairs (Table S2a in Additional file 3) are <0.004 except that for the lung-skin pair, which is 42 %.

### Inter-tissue network generation

A natural way to find clusters in two groups of nodes is to use bipartite clustering [17], an established technique in the field of machine learning. We implemented a standard bipartite clustering algorithm using singular values decomposition [17]. However, when applying GO enrichment analysis for the genes in the partitioned clusters, we found no significant enrichment. A bipartite clustering method aims to find balanced clusters in the two groups of nodes. However, inter-tissue interactions may not be bi-directional, where a large number of genes in tissue A correlate with a group of genes in tissue B, but rather unidirectional, where a small number of genes in tissue A interact with a large number of genes in tissue B. We developed the following procedure to identify unidirectional interactions.

### Gene-to-module interactions

Given a tissue pair *A* and *B*, we define a set of genes for each gene *i*_*A*_ in tissue A, namely ${M}_{i_A,B}$, representing genes in tissue *B* that are correlated with the gene *i*_*A*_ in tissue A. Then, we define significant asymmetry correlation sets as follows. First, we calculate the total number of genes in tissue *A* that are correlated with any of the members in *M* (in tissue *B*) as ${\displaystyle \sum_{J_B\in {M}_{i_A,B}}{M}_{J_B,A}}$ and the average number of genes for members in ${M}_{i_A,B}$ as $A{M}_{i_A,B}=\frac{{\displaystyle \sum_{J_B\in {M}_{i_A,B}}{M}_{J_B,A}}}{M_{i_A,B}}$. If ${M}_{i_A,B}$ is less than one-tenth of ${M}_{i_A,B}$, we define the set ${M}_{i_A,B}$ as a candidate asymmetry set. Second, we check whether candidate asymmetric inter-tissue correlations ${M}_{i_A,B}$ are due to potential common regulations in a single tissue. For the same gene *i* in tissue *B*, we define ${M}_{i_A,B}$ as an asymmetric set if ${M}_{i_A,B}$ is less than one-tenth of the size of ${M}_{i_A,B}$. The significance of a module of size $\left|{M}_{i_A,B}\right|$ can be approximately estimated using binomial models. For each pair of tissues, we calculate the average number genes that each gene was correlated with. Then, according to the size of a module, we estimate the *p* value of observing a module of the same size by chance. The *p* values for modules of size *m* in each tissue pair is listed in Table S2b in Additional file 3.

### Enrichment analysis

We used Bioconductor [18] to carry out the GO biological process (GOBP) enrichment analysis. We applied the hypergeometric test using the annotation database “org.Hs.eg.db”. The *p* value cutoff used was 0.05 dividing the number of GOBPs tested in the corresponding tissue pairs. Since the GOBPs are hierarchically organized in a tree-like structure, terms at different levels of the tree are not comparable. We counted the number of terms in the path from the root as the “level” of each GO term and used only level 3 GO terms. We also applied the hypergeometric test for enrichment analysis using the disease GWAS candidate gene catalog (http://www.genome.gov/gwastudies/) and disease signature database. MSigDB (http://www.broadinstitute.org/gsea/msigdb/index.jsp) and multiple Human tissue atlas expression profile datasets were used to generate the heat map of the expression levels of the genes in whole blood that correlated with heart *DPP4* expression levels [19, 20].

## Results and discussion

### Genetic decorrelation of GTEx data and construction of inter-tissue coexpression networks

All gene expression data used in this analysis were preprocessed to correct for common confounders such as batch effects and experimental artifacts by adjusting for factors estimated using the probabilistic estimation of expression residuals (PEER) method [13]. After correcting for the PEER factors and filtering lowly expressed genes, we calculated the Spearman correlation for each gene pair between the nine surveyed tissues. In total, there are 9 × 8⁄2 = 36 tissue pairs, and for each tissue pair we calculated around 20,000 × 20,000 correlations between gene pairs. An inter-tissue interaction was defined as a pair of genes whose correlations both before and after genetic decorrelation are significant (see "Methods" for details).

The major goal and novelty of this study is to explore biological interactions between tissues, in the absence of the potentially confounding common genetic contributions to different genes [4] due to common regulatory elements or shared expression quantitative trait loci (eQTL), which were reported in the GTEx paper [5]. A high correlation between two genes in two different tissues can be due to their shared genetic contribution, which obscures tissue–tissue correlations related to biological communication. In order to dissect out these shared genetic contributions, a straightforward procedure would be to remove common genetic effects by conditioning on shared eQTLs. However, this procedure relies on many parameters, such as the number of eQTLs to include, and therefore may suffer from over-fitting, especially for genes whose expression levels have complicated genetic architectures. In general, for any two individuals, the probability of sharing genetic alleles, regardless of how complicated their genetic architecture is, is proportional to their genomic similarity. Motivated by this observation, we developed a procedure to decorrelate genetic effects using the identity-by-state (IBS) matrix as estimated by the genotypes. We first estimated the pseudo-heritability [21], which is the expression variance component explained by the genome similarities [22, 23]. Then we used the estimated heritability to create a transformation matrix for removing genetic regulation from the expression data so that, in theory, the data, after transformation, contain minimal genetic contributions. This procedure is commonly used in mixed models [16, 24] and is described in detail in the "Methods" section.

**Fig. 3:**
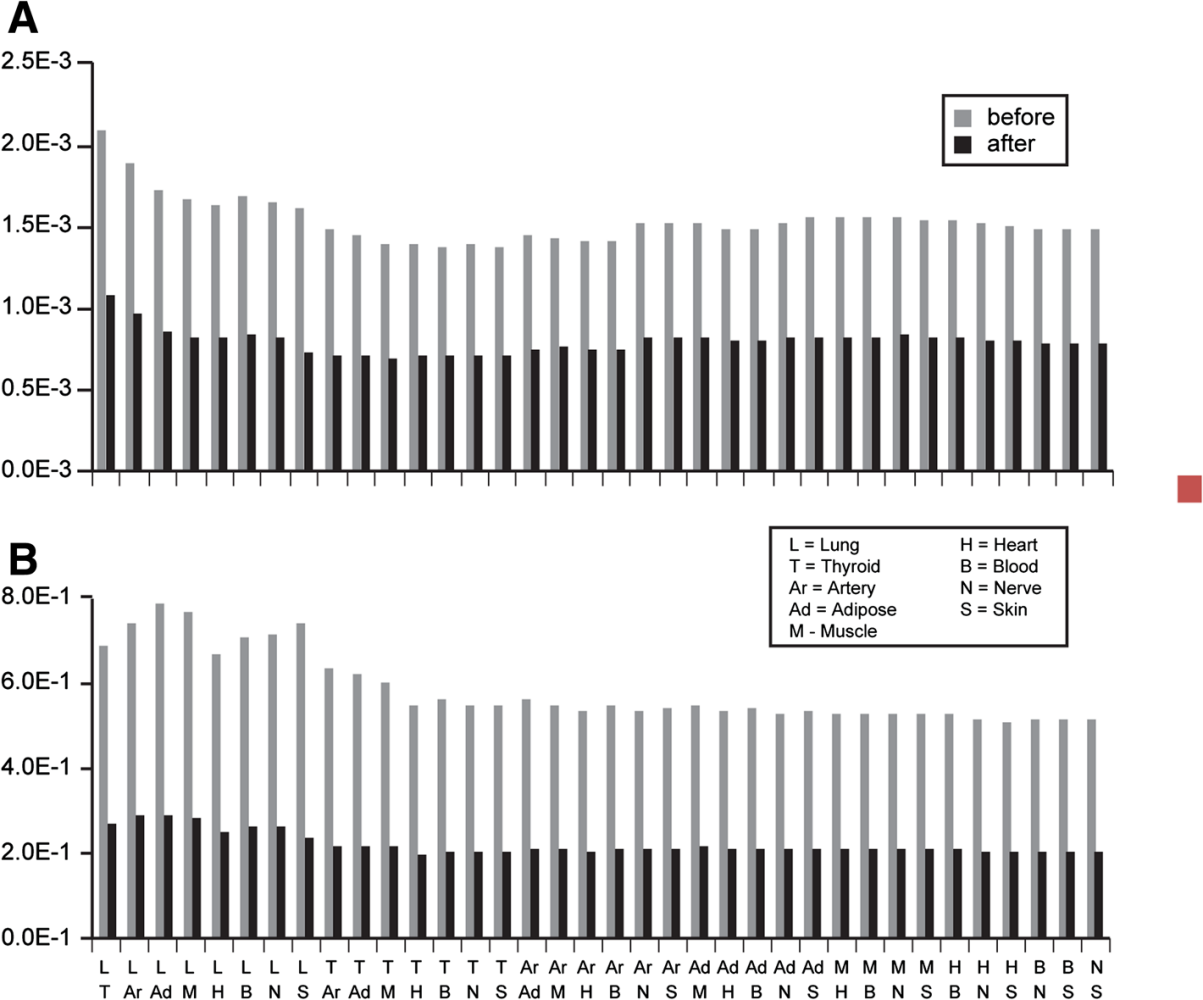
Assessing the indication of genetic contribution to the inter-tissue correlations. **a** Average number of shared eQTLs per pair of significantly correlated genes. **b** Proportion of significant correlations due to *cis* correlation of genes (i.e., expression levels of the same gene in two different tissues correlate with each other)

After genetic decorrelation, cross-tissue gene–gene correlations were calculated again following the same procedure described above. We used *R* and *R*^∗^ to represent correlation matrices before and after genetic decorrelation. To evaluate whether the genetic decorrelation procedure effectively removed the genetic component from the data we applied two different tests. First, we compared the shared eQTLs between cross-tissue correlated gene pairs. Given a pair of tissues, we calculated the average number of shared eQTLs (as described in the GTEx pilot analysis [5]) of all pairs of genes that were significantly correlated. By comparing this indicator before and after decorrelating genetic effects, we observed that the average number of shared eQTLs decreased dramatically after transformation (Fig. 3a). Secondly, we compared the correlation between pairs of identical genes in two tissues before and after genetic decorrelation. The genetic architecture of transcriptional regulators for the same gene in different tissues is similar such that common genetic regulation likely contributes to the correlation of expression of the same gene in different tissues. We observed that this proportion was also significantly reduced after applying the transformation (Fig. 3b). Thus, we conclude that our transformation procedure removed a significant portion of the genetic contribution to the correlation of gene expression between tissues.

In some cases, the mixed model-based transformation can also introduce artifacts such as when the pseudo-heritability of two genes is similar. In these instances, their transformation matrix will be alike and could therefore generate artificial correlations. Also, since the sample size of the shared tissue pairs is low (Additional file 1), the variance of the estimate of pseudo-heritability, which is proportional to the sample size, can be large. In order to filter out potential artificial correlations introduced by genetic decorrelation, we adopted the following conservative rule: two genes from two tissues are defined as inter-tissue correlated if and only if they are significantly correlated with each another before and after the transformation. These inter-tissue correlations are most likely due to biological signals instead of common genetics.

### A global overview of the patterns of inter-tissue coexpressed genes

**Fig. 4:**
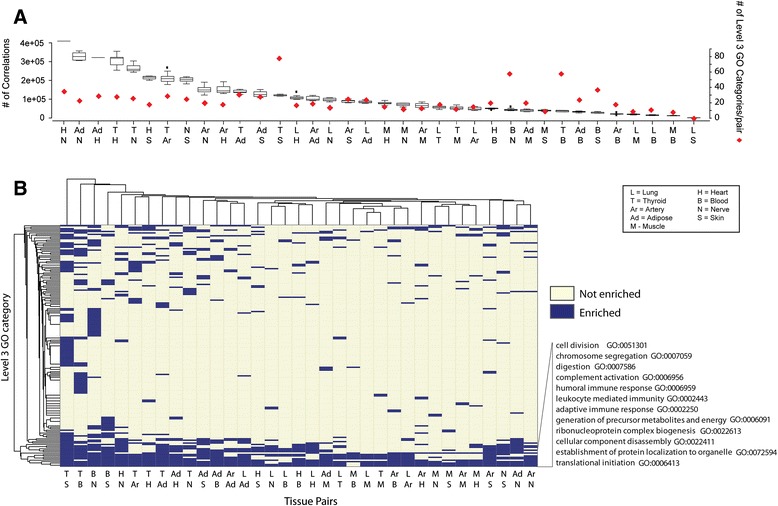
Functional annotation of inter-tissue correlations. **a** Summary of the number of correlations between tissue pairs (left axis) and summary of the number of significant GOBPs associated with the asymmetric inter-tissue correlations (gene-to-module relationships) per tissue pair (right axis). **b** Heat map transformation summarizing the GO enrichment analysis results for each tissue pair. The GO categories used are listed in Additional file 6

Given the correlation matrix *R*^∗^ calculated above, for each pair of tissues we constructed inter-tissue coexpression networks as bipartite graphs. In these graphs, the nodes in two columns are the genes in the two tissues, and an edge between two nodes is added if they are inter-tissue correlated. To assess a global pattern of inter-tissue coexpressed genes we examined the total number of significant gene–gene correlations between each tissue pair, which provided an estimate of the degree of potential biological interactions between tissues (Fig. 4a). Among the top five pairs of tissue were nerve and heart, with nerve in combination with heart, adipose and thyroid, and heart in combination with nerve, thyroid and adipose. Given that the function of nerves within the nervous system is to send signals from one part of the body to another and to receive feedback in order to coordinate motor and sensory responses, it is not surprising to observe a significant number of inter-tissue correlations involving the tibial nerve. Similarly, given the intimate connection between the heart and the circulation, which delivers blood to all parts of the body, observing a significant number of inter-tissue coexpressions is expected. In contrast, the lung was the most prominent in tissue pairs scoring the lowest number of inter-tissue correlations in combination with skin, whole blood and muscle.

### Identification of asymmetric inter-tissue correlation patterns

Gene clusters derived using a standard bipartite clustering algorithm [17] were not significantly enriched in any GOBP. A standard bipartite clustering aims to find a cluster of genes interacting with another cluster of genes in the second tissue. However, cross-tissue interactions may be unidirectional, e.g., a few genes in one tissue regulate many genes in another tissue. Thus, we developed a selection strategy for identifying asymmetric inter-tissue correlation patterns. For each individual gene we assessed whether there was a large cluster of genes in another tissue asymmetrically linked with it, which were referred to as gene-to-module relationships (Fig. 2). The numbers of gene-to-module relationships identified in each pair of tissues are listed in Additional file 4. We then performed GO enrichment analysis on the top 20 most significant gene-to-module relationships to infer which biological pathways are involved in inter-tissue interactions. The complete set of significantly enriched pathways is listed in Additional file 5. It is worth noting that the total number of inter-tissue relationships and the number of biological pathways enriched among genes involved in gene-to-module relationships (Fig. 4a) were not related. The tissue pairs with the largest number of enriched level 3 GOBPs, such as thyroid–skin and thyroid–blood, had small numbers of total cross-tissue correlations (Fig. 4a). Thyroid is the tissue where gene expression is most likely to be regulated by genetic variation [5] so that genetic decorrelation has the largest effect for thyroid expression data. These results indicate that decorrelating genetic effects may facilitate the identification of biological connections between tissues.

The GOBPs associated with the top 20 most significant gene-to-module relationships according to the tissue pairs they were identified in are summarized in Fig. 4b (GO terms used in the analysis are listed in Additional file 6). Some pathways associated with unidirectional interactions between tissues were enriched in many tissue pairs while other pathways were only enriched in specific tissue pairs (Fig. 4b). A closer look at the extremes revealed some interesting observations. Most strikingly, four pathways, including establishment of protein localization to organelle, translational initiation, cellular component disassembly and ribonucleoprotein complex biogenesis, were identified in almost all tissue pairs, revealing global synchronization of these processes by unidirectional inter-tissue interactions. Since these pathways are related to protein synthesis, our result likely reveals the importance of coordination in growth processes within the human body and that this coordination can be achieved via asymmetric molecular interactions. The joint coordination of protein biology across tissues via asymmetric interactions implies the expression of one gene in one tissue is sufficient to impact clusters of genes related to protein biology in another.

Other pathways that were involved in inter-tissue interactions in most tissue pairs included different aspects of the immune responses (complement activation, adaptive immune response, humoral immune response, leukocyte-mediated immunity), metabolism (generation of precursor metabolites and energy) and cell division.

**Fig. 5:**
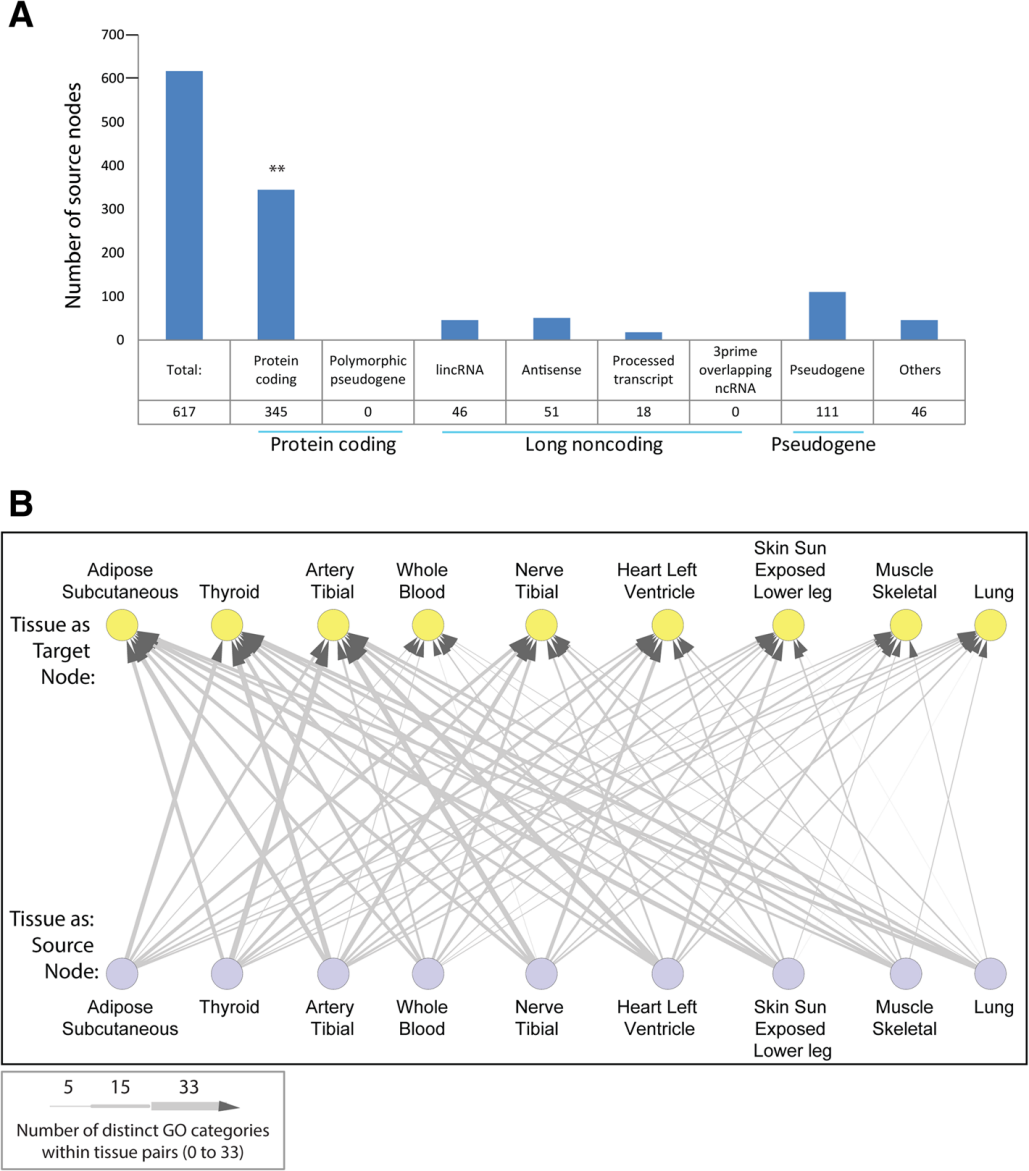
Characteristics of inter-tissue unidirectional relationships. **a** Gene types of source nodes in the top ten most significant gene-to-module relationships in each tissue pair. Protein coding genes were enriched among source nodes (*p* value = 1.6 × 10^−21^). **b** Numbers of unique GOBPs that are inter-tissue regulated in a unidirectional way. For each source node we therefore picked only the top significantly enriched GOBP associated with its gene cluster (listed in Additional file 7). The most biologically diverse interactions are from thyroid to artery. *lincRNA* long intergenic non-coding RNA

While these analyses provide information on the number of inter-tissue correlations and the associated GOBPs, they do not specify the regulators or source nodes (as shown in Fig. 2) of these interactions. We collected source nodes of the top ten most significant gene-to-module relationships in each tissue pair, and characterized them according to gene types (Fig. 5a). There were a total of 617 source nodes, among which 345 (56 %) source nodes were protein coding genes, significantly higher than the 37 % expected (*p* value = 1.6 × 10^−21^). Even though there are more transcripts mapped to pseudogenes than to lncRNAs, there were more lncRNAs than pseudogenes among source nodes. These results suggest that protein coding genes and lncRNAs play a significant role in inter-tissue communication.

Instead of counting which GOBPs are inter-tissue regulated as shown in Fig. 4, we examined which GOBPs are inter-tissue regulated in a unidirectional way. For each source node we therefore picked only the top significantly enriched GOBP associated with its gene cluster (listed in Additional file 7). We then counted the number of unique GOBPs for each possible tissue pair (Fig. 5b). This revealed that the most biologically diverse interactions are from thyroid to artery. One example of such interactions is the correlation of *HEBP2* in thyroid with genes involved in translational elongation in the artery. HEBP2 is a small heme binding protein that potentially regulates necrotic cell death and mitochondrial permeability [25]. *HEBP2* is universally expressed in all tissues. However, HEBP2 protein expression is high only in thyroid tissue except for male reproductive tissues (http://www.proteinatlas.org/ENSG00000051620-HEBP2/tissue), suggesting a potential role of *HEBP2* in thyroid. Other prominent interactions are from heart to artery, artery to thyroid and nerve to artery. An example of the former interaction is the correlation of *SRPX2* in heart to genes associated with the respiratory electron transport chain in artery. *SRPX2* encodes a secreted protein containing sushi repeat domains that can mediate angiogenesis [26]. *SRPX2* also plays a role in synapse formation and vocalization in mice and mutations in *SRPX2* have been identified in disorders of language cortex and cognition [27, 28].

These results suggest that multiple biological processes are systemically coordinated via unidirectional interactions between tissues. In an effort to demonstrate the utility of inter-tissue coexpression in yielding novel biological insights, we highlight in the following section two asymmetric inter-tissue correlation patterns, one related to a prominent anti-diabetic drug target and another to protein synthesis.

### *DPP4* in the heart is coexpressed with a cell cycle module in whole blood

**Fig. 6:**
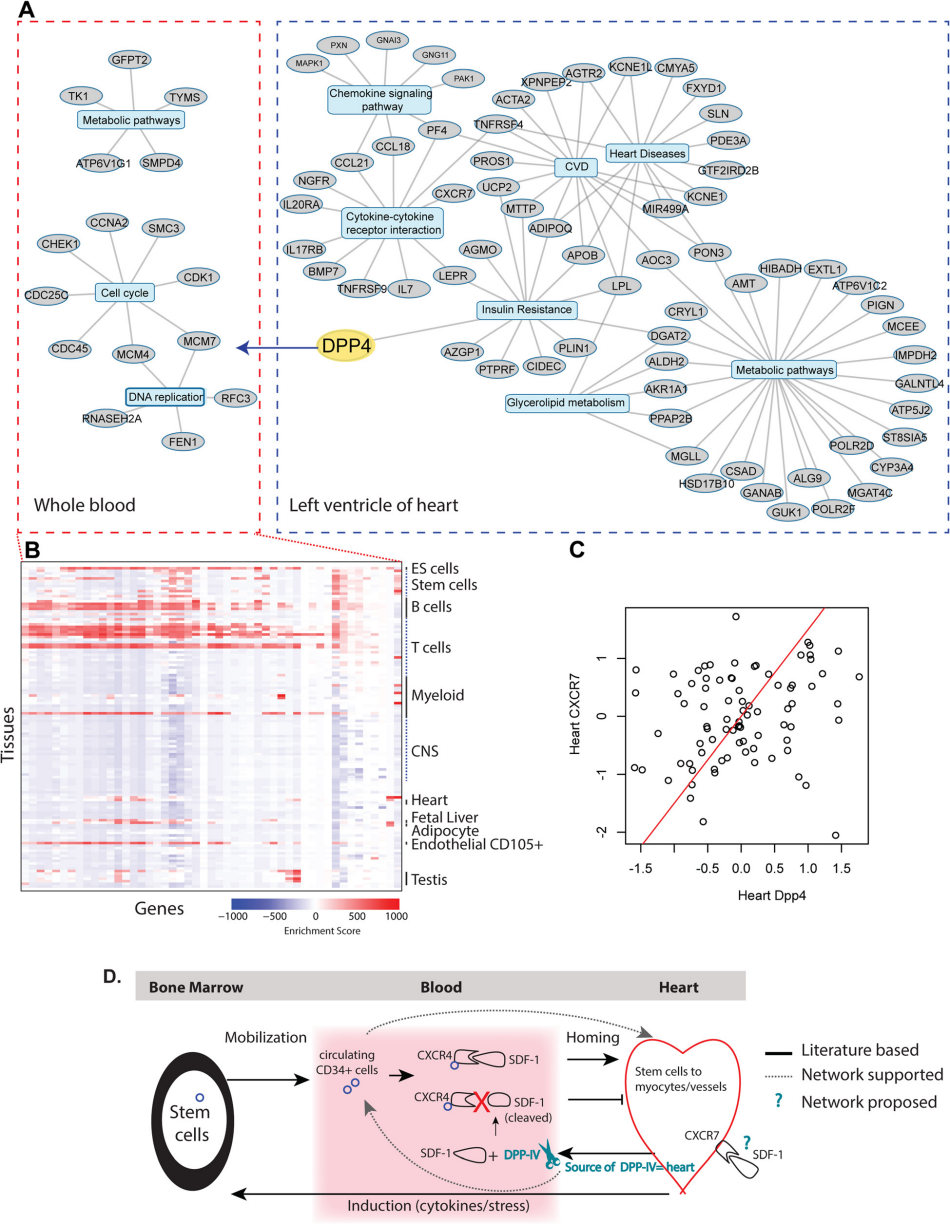
Cardiac DPP4 controls a set of cell proliferation related genes in whole blood. **a** A network visualization of a subset of genes in either whole blood (*left panel*) or heart (*right panel*) that are co-correlating with heart *DPP4*. Central nodes reflect the names of the top scoring canonical pathways (full list in Additional file 9) with the associated nodes being the genes co-correlating with heart *DPP4* that are found in those pathways. Heart *DPP4* is in general negatively correlated with nodes in the whole blood cross-tissue network. **b** Heat map displaying the enrichment scores obtained from gene enrichment profiler (http://xavierlab2.mgh.harvard.edu/EnrichmentProfiler/enrichmentMaps.html) for the whole blood geneset that correlated with heart DPP4 levels. Only a subset of 126 tissues are annotated (for full results see Additional file 10). **c** A scatter plot of expression levels of *CXCR7* versus *DPP4* in the heart (correlation coefficient *r* = 0.224, *p* value = 0.02). Full enrichment analyses are summarized in Additional file 11. **d** Literature-based and network-supported associations between DPP4 and SDF-1 with respect to blood and heart. Network-proposed insights are highlighted in *blue*. The SDF-1/CXCR4 axis has been shown to be critical in tissue repair, including in the heart, as SDF-1 is well known as a key regulator of stem cell migration to sites of tissue injury. A major enzyme mediating the degradation of SDF-1 is DPP4. Suppression of DPP4 enzymatic activities by pharmacological inhibitors preserves SDF-1, which results in enhanced homing of CXCR4^+^ progenitor cells from bone marrow to infarcted tissues. *CNS* central nervous system, *ES* embryonic stem

One example for illustrating inter-tissue communication is between heart and whole blood. *DPP4* in the heart was found significantly coexpressed with a set of genes in peripheral whole blood (Additional file 8), which are enriched for genes involved in cell cycle control and DNA replication (Fig. 6a; detailed in Additional file 9). Interestingly, the majority of the correlations between heart *DPP4* and the whole blood genes were negative (Additional file 8), suggesting an inverse relationship between heart *DPP4* and these processes. To determine the most representative cell populations associated with the *DPP4*–whole blood gene set, we compared expression levels of these genes with expression profiles assayed across 126 primary human tissues and cell types in the Gene Enrichment Profiler [29]. Tissue-specific gene expression is summarized by an enrichment score where high enrichment scores mean higher specificity. A heat map of the enrichment scores for the *DPP4*-correlating whole blood gene set reveals that this set of genes is most highly expressed in many proliferating blood cell types, including embryonic and CD34+ hematopoietic stem cells, preB cells, thymic CD34+ T cells and CD105+ endothelial cells (Fig. 6b; listed in Additional file 10). As correlations between *DPP4* in heart and the whole blood gene set are mostly negative (Additional file 8), it suggests that cardiac *DPP4* expression is negatively correlated with cell cycle events of multipotent precursor type cells, such as populations of embryonic stem cells, which can differentiate into myeloid progenitor or endothelial progenitor subtypes.

*DPP4*, also known as CD26, is a serine protease that cleaves selected N-terminal penultimate amino acids and thereby potentially alters the function of a wide number of substrates [30]. DPP4 exists both as a cell surface protein (mDPP4) with fairly ubiquitous expression as well as a soluble form (sDPP4) in body fluids such as plasma, both having enzymatic capabilities. sDPP4 has been mainly linked to the proteolytic cleavage of DPP4 from the cell membrane [31]. About 90–95 % of serum DPP4 activity is associated with circulating DPP4 levels, but the kinetics and regulation of circulating DPP4 levels remain unclear [32]. Therefore, investigating cross-tissue *DPP4* molecular associations could provide valuable insight into DPP4 function in physiology and disease. Thus, *DPP4* is an excellent example for investigating inter-tissue coexpression networks, as it provides the biological context to investigate such complex associations.

To further investigate the inter-tissue communication being coordinated by cardiac *DPP4* expression and the whole blood compartment, we assessed the coexpression network associated with *DPP4 within* the heart. This *within*-tissue coexpression network analysis revealed a set of genes that are highly enriched for cytokine–cytokine receptor interactions as well as lipid-related pathways (Fig. 6c; detailed in Additional file 11). Importantly, these genes are also enriched in diseases such as insulin resistance, metabolic syndrome and obesity (Fig. 6c). Thus, heart expression of *DPP4* appears to be associating its molecular metabolic state with decreased proliferative events in subsets of whole blood cells. We hypothesize that this reflects altered mobilization of stem cells to the heart required for vascular repair in response to metabolic stresses such as diabetes.

Extensive literature exists on DPP4, mainly due to DPP4 inhibitors or gliptins, a class of oral hypoglycemics commonly used to treat type 2 diabetes [33]. These compounds lower blood glucose via enhancing circulating levels of the incretin GLP-1. Thus, we looked to validate our inter-tissue network-driven DPP4 hypothesis using existing knowledge. Many studies have demonstrated that pharmacological inhibition of DPP4 is associated with a beneficial effect on the incidence of cardiovascular events [30]. Multiple mechanisms have been attributed to this phenomenon, including glucose-lowering, but also other direct cardiovascular effects such as anti-thrombotic as well as cardiac remodeling and inflammation control [34, 35]. This is in part because DPP4 is known to cleave a wide array of bioactive peptides in addition to GLP-1, such as chemokines [36]. Relevant for the validation of our results is the known link between DPP4 activity and molecular signals of hematopoiesis in whole blood, namely that DPP4 is known to cleave and inactivate the stromal cell-derived factor SDF-1 [33, 37].

SDF-1 is a major chemokine regulating stem/progenitor cell trafficking in the bone marrow and tissues [34]. Diabetes and other metabolic disturbances threaten the endothelial layer of the heart, which is critical to maintain cardiac function since endothelial cells have limited proliferative capacity and low proliferation rates trigger atherogenesis. Endothelial restoration depends on the coordinated contribution of local endothelial cells and a subset of bone-marrow progenitor cells (BM-CD34+ or endothelial progenitor cells) that secrete soluble mediators that also stimulate blood vessel growth and re-endothelialization. Diabetes affects the ability of endothelial progenitor cells to migrate to the target tissue because it reduces the activity of the chemotractant molecule SDF-1 through maladaptive DPP4-mediated cleavage, thereby blunting the ability of SDF-1 to trigger CXCR4 or CXCR7 downstream signaling [34, 38]. Our studies suggest that a key ‘source’ of the SDF-1 signal modulating the kinetics of stem/progenitor cells is the expression and activity of DPP4 in the heart. Indeed, body atlas data from BioGPS (http://biogps.org/#goto=genereport&id=1803) showed that the expression level of *DPP4* in smooth muscle, cardiac myocytes as well as immune cell types is much higher than that in the whole blood.

Although our study cannot determine whether it is sDPP4 and/or mDPP4 responsible for mediating the cross-tissue coordination, overall our data are supported by observations in the literature [39]. Firstly, diabetes induction in a rat model promoted the activation of the membrane-bound form of cardiac DPP4, which resulted in reduced myocardial SDF-1 levels and impaired angiogenesis, a result that could be reversed by genetic and pharmacological ablation of DPP4 [39]. Secondly, in humans, it has been shown that myocardium-derived sDPP4 activity can be assessed in the coronary sinus which also correlates well with DPP4 activity measured in the peripheral vein. Importantly, in patients with diastolic hear failure (DHF), peripheral vein activity of DPP4 was associated with measurements of DPP4 in the coronary sinus as well as with indices representing DHF, such that circulating DPP4 may potentially serve as a biomarker for monitoring DHF [39]. Finally, circulating DPP4 activities in both peripheral and coronary sinus in patients with comorbid diabetes were also increased, thus further supporting the importance of DPP4, of which some proportion is cardiac derived.

Interestingly, our data demonstrated a significant correlation (*p* value = 0.02; Fig. 6c) between *DPP4* expression in heart and heart expression of *CXCR7*, a SDF-1 receptor [40] whose expression on the endothelium has been linked to regulation of systemic levels of SDF-1 [38], suggesting that mDPP4 is likely mediating this cross-tissue communication via SDF-1. Thus, overall, our inter-tissue data-driven network approach could recapitulate the known biology, namely that metabolic disturbances of the heart are negatively associated with progenitor cell populations in the blood via DPP4 expression [33] (solid and dashed arrows in Fig. 6d), thus in part validating this novel methodology. Importantly, our network approach supports that a major contributor to the DPP4 pool is heart, especially under metabolic stress, and that there may be an important role for CXCR7 in co-modulating SDF-1 function (Fig. 6d).

### Cardiac expression of *FOCAD* correlated with lung protein synthetic processes

One example of asymmetric inter-tissue correlations related to protein synthesis is observed for cardiac expression of the poorly characterized *FOCAD* (*KIAA1797* or focadhesin) and the gene set it correlated with in the lung. This set of genes in lung coordinated by *FOCAD* was enriched for GOBPs related to protein synthesis, such as translational termination, translational elongation and SRP-dependent cotranslational protein targeting to membrane.

Focadhesin is a ubiquitously expressed gene with highest levels in brain, but appreciable levels in other tissues such as heart. Focadhesin was recently described as a tumor suppressor in glioblastoma, polyposis and colorectal cancer [41–43]. The focadhesin protein localizes to the end of actin filaments, where it colocalizes with vinculin, a major component of focal adhesions [41]. Moreover, focadhesin physically interacts with vinculin [41, 44]. No function for this gene has been established in the heart.

Focal adhesions are the sites where cells and extracellular matrix physically interact. Many proteins constitute the focal adhesion, with the integrins being the link between the cytosol and the extracellular matrix. Integrins function as cell surface receptors, and their interaction with the extracellular matrix enables them to transduce signals from the outside to the cell (“outside-in” signaling). In the heart focal adhesions play an important role in the response to biomechanical stress, enabling the myocardium to undergo structural changes [45]. In the GTEx dataset, *FOCAD* expression in the heart correlates with integrin α5 (*ITGA5*, *r=* 0.44, *p* = 1.34 × 10^−5^) and paxillin (*PXN*, *r=* 0.37, *p* = 3.12 × 10^−4^), further signifying its role in focal adhesion. Our data suggest that signaling at the focal adhesion not only has effects within the tissue, but can also affect other tissues, in this case the lung. From a mechanistic point of view, it is known that integrins can also signal to the extracellular matrix (“inside-out” signaling) [46], but how such a signal may be propagated from the heart to the lung is at present unclear.

Our observations are consistent with the close interaction between heart and lung as both organs share the same restricted space in the chest. Moreover, heart and lung are also functionally connected, such as that the lungs are responsible for the exchange of CO_2_ for O_2_ in blood, while the heart circulates this blood. This tight connection is illustrated by several pathophysiological conditions whereby one organ can develop a problem that influences the efficiency of the other. For example, in pulmonary arterial hypertension there is an increase in pulmonary vascular resistance directly impacting on the right ventricle [47]. Chronic heart failure is associated with mild to moderate changes in pulmonary function [48].

Other evidence supporting a role for *FOCAD* in heart and lung comes from several genome-wide association studies (GWAS) since variants in *FOCAD* have been associated with cardiac as well as pulmonary traits. SNPs in *FOCAD* have been associated with heart rate in American Indians [49], and heart failure in the STAMPEED study [50]. A SNP in *FOCAD* also associated with a pulmonary function measure (percentage predicted forced expiratory flow from the 25^th^ to 75^th^ percentile/forced vital capacity for latest exam) in the Framingham Heart GWAS [51]. Together these findings implicate focadhesin in inter-tissue communication from heart to lung.

### Inter-tissue module and disease GWAS candidate genes

Although our *DPP4* and *FOCAD* examples above have relevancy to disease, as *DPP4* is a target of current diabetes therapy and the other gene is a GWAS hit, to generalize the utility of our cross-tissue network analysis on informing on disease association we annotated significant inter-tissue gene modules against disease or drug signatures in MSigDB and the catalogue of genes implicated in various GWAS (results listed in Additional file 7). If an inter-tissue gene module is enriched for a GOBP, it is likely enriched for a signature in MSigDB. We also observed 17 gene module pairs from various tissue pairs that were significantly enriched (at Fisher’s exact test *p* value <0.05/number disease categories) in genes genetically associated with disease. For example, in one adipose to artery gene module where the source node was *ANKRD36B* (ankyrin repeat domain-containing protein 36B) we found that the genes it correlates with in the artery were significantly enriched in genes reported to have relevance to Gaucher disease severity in humans. Gaucher disease is associated with a genetic defect in breakdown of complex glycolipids and causes a lysosomal storage disorder. Interestingly, although the function *of ANKRD36B* is not well defined, it has been identified as a tumor-associated antigen in chronic lymphocytic leukemia (CLL) [52]. Multiple myeloma risk is significantly increased in Gaucher patients, but CLL has also been reported in patients and perhaps our data shed light on some of the biology associated with enhanced cancer risk [53]. Another significant GWAS candidate gene enrichment was found between the artery–whole blood tissue pair, with the source node being *CPLX2* (Complexin 2) and the associated GWAS for conduct disorder (interaction). Conduct disorder is a prevalent childhood psychiatric condition including a persistent pattern of rule-breaking and aggressive behavior [54]. Interestingly, *CPLX2* is a known modulator of neurotransmitter release and has been shown in humans to be decreased in expression in animal models of depression and in humans suffering from depression. *CPLX2* knockout mice also have significant abnormalities in cognitive function and synaptic plasticity [55]. Furthermore, variants of this gene have been found associated with attention deficient hyperactivity disorder [56] and schizophrenia [57] and the module of genes it associated with in blood are enriched for the GO pathway related to transmission of nerve impulses. Although *CPLX2* expression is considerably high in the brain, bioGPS does suggest ubiquitous expression and one hypothesis from our dataset that could be tested is whether Clpx2 modulates release of a signal in the blood that in turn impacts on the brain.

### Gene expression profiles and sample ischemic time

It is known that sample ischemic time impacts on RNA quality, which in turn affects gene expression profiles. RNA quality is quantified as the RNA integrity number (RIN). The RNAseq data preprocessing process removed confounding factors, including sample ischemic time and RINs represented as PEER factors. To specifically check residual effect of sample ischemic time and RINs on the data (after correcting PEER factors) used in this study, we correlated sample ischemic time and RINs with gene expression profiles, and the correlation coefficient distributions were found to be similar to those of the permuted data (Figure S2 in Additional file 2). More specifically, the correlation coefficients between sample ischemic time and heart *DPP4* and *FOCAD* expression levels are 0.07 and 0.04, with corresponding *p* values of 0.28 and 0.35, suggesting that the processes discussed above are not due to ischemic time.

### Potential shortcoming

It is worth noting that an asymmetric inter-tissue correlation suggests but is not equivalent to a causal–reactive relationship between tissues. Even though in the example of heart *DPP4* and whole blood expression changes we highlighted substantial evidence supporting a causal role of heart DPP4, we need to be cautious when assuming causal–reactive relationships in general. Second, the inter-tissue relationships are based on a healthy cohort, which are useful for understanding general communication between tissues. However, the inter-tissue relationships for a specific disease may be different. Multi-tissue profiles from a disease cohort are needed to construct inter-tissue relationships under a disease state. Inter-tissue relationship differences between healthy and disease states may shed light on how multiple tissues together contribute to disease pathogenesis. Third, the accuracy of the transformation matrix *D* used in genetic decorrelation depends on the sample size. A large number of samples are needed to robustly estimate the matrix *D* and to use our inter-tissue analysis based on genetic decorrelation.

## Conclusions

We have developed an effective strategy to generate genetically decorrelated inter-tissue networks that have the power to highlight communication between tissues and elucidate genes active in one tissue inducing gene expression changes in another tissue. This analysis revealed global unidirectional inter-tissue coordination of certain biological pathways, such as protein synthesis. We highlighted cardiac *FOCAD* as one potential key mediator of this process between the heart and lung. Beyond the conserved pathways, we also uncovered a clinically relevant example whereby expression levels of *DPP4* in the heart are coordinated with whole blood proliferation, thereby potentially regulating stem cell proliferation, trafficking and mobilization to peripheral tissues, an observation important for regenerative medicine. More broadly, this is the first resource of human multi-tissue networks enabling the investigation of molecular inter-tissue interactions. With the networks in hand, we may systematically design combination therapies that simultaneously target multiple tissues or pinpoint potential side effects of a drug in other tissues.

## Additional files

Additional file 1: Table S1. Sample sizes of shared subjects for each pair of tissues. (PDF 6 kb) Additional file 2:
**Supporting notes. Figure S1.** The optimal numbers of principal components (PCs) to correct in each tissue. **Figure S2.** Histograms of correlation coefficients between sample ischemic time and RINs with gene expression profiles in nine tissues. *Red lines* are for correlation with RINs, and *blue lines* are for correlation with sample ischemic time. *Solid lines* are for empirical gene expression profiles in the study, *dashed lines* are for permuted data. (DOCX 500 kb) Additional file 3: Table S2. Significant scores based on permutation tests. **a** Estimated FDRs of inter-tissue connection for each tissue pair. **b** Empirical *p* value for significant gene-to-module interactions in each tissue pair. (XLSX 129 kb) Additional file 4: Table S3. Number of significant gene-modules identified for each tissue pair. (PDF 42 kb) Additional file 5: Table S4. Top 20 significant modules in terms of *p* value of GO enrichment analysis in all tissue pairs. (PDF 2423 kb) Additional file 6: Table S5. Names and IDs of GO terms used in Fig. 4b. (PDF 11 kb) Additional file 7: Table S6. Functional annotation of significant inter-tissue gene modules against GO biological processes, disease candidate genes in GWAS catalog, and the molecular signature database (MSigDB). (CSV 387 kb) Additional file 8: Table S7. A list of genes whose expression in whole blood significantly correlated with cardiac *DPP4* expression. (XLSX 15 kb) Additional file 9: Table S8. Full list of KEGG enrichment analysis results for the set of genes that heart DPP4 correlates with in whole blood. (XLSX 10 kb) Additional file 10: Table S9. The enrichment scores for each of the DPP4-correlating whole blood genes in primary human tissues and cell types. (XLSX 64 kb) Additional file 11: Table S10. KEGG and disease pathway enrichment analysis for genes that correlate with DPP4 within the heart. (XLSX 16 kb)
