# Supplementary material for: Inter-tissue coexpression network analysis reveals DPP4 as an important gene in heart to blood communication
Source: Genome Med. 2016 Feb 9;8:15. doi: 10.1186/s13073-016-0268-1 (PMC4746932; doi:10.1186/s13073-016-0268-1)
Supplement: Additional file 1: Table S1. — Sample sizes of shared subjects for each pair of tissues. (PDF 6 kb) [file 13073_2016_268_MOESM1_ESM.pdf]

tissue pair	sample size
Lung.Thyroid	84
Lung.Artery_Tibial	83
Lung.Adipose_Subcutaneous	68
Lung.Muscle_Skeletal	100
Lung.Heart_Left_Ventricle	66
Lung.Whole_Blood	104
Lung.Nerve_Tibial	70
Lung.Skin_Sun_Exposed_Lower_leg	71
Thyroid.Artery_Tibial	73
Thyroid.Adipose_Subcutaneous	65
Thyroid.Muscle_Skeletal	88
Thyroid.Heart_Left_Ventricle	58
Thyroid.Whole_Blood	90
Thyroid.Nerve_Tibial	56
Thyroid.Skin_Sun_Exposed_Lower_leg	60
Artery_Tibial.Adipose_Subcutaneous	68
Artery_Tibial.Muscle_Skeletal	101
Artery_Tibial.Heart_Left_Ventricle	62
Artery_Tibial.Whole_Blood	100
Artery_Tibial.Nerve_Tibial	68
Artery_Tibial.Skin_Sun_Exposed_Lower_leg	66
Adipose_Subcutaneous.Muscle_Skeletal	85
Adipose_Subcutaneous.Heart_Left_Ventricle	48
Adipose_Subcutaneous.Whole_Blood	83
Adipose_Subcutaneous.Nerve_Tibial	53
Adipose_Subcutaneous.Skin_Sun_Exposed_Lower_	60
Muscle_Skeletal.Heart_Left_Ventricle	69
Muscle_Skeletal.Whole_Blood	122
Muscle_Skeletal.Nerve_Tibial	77
Muscle_Skeletal.Skin_Sun_Exposed_Lower_leg	85
Heart_Left_Ventricle.Whole_Blood	74
Heart_Left_Ventricle.Nerve_Tibial	48
Heart_Left_Ventricle.Skin_Sun_Exposed_Lower_leg	51
Whole_Blood.Nerve_Tibial	79
Whole_Blood.Skin_Sun_Exposed_Lower_leg	84
Nerve_Tibial.Skin_Sun_Exposed_Lower_leg	56
