# Supplementary material for: Inter-tissue coexpression network analysis reveals DPP4 as an important gene in heart to blood communication
Source: Genome Med. 2016 Feb 9;8:15. doi: 10.1186/s13073-016-0268-1 (PMC4746932; doi:10.1186/s13073-016-0268-1)

**Supplementary Notes for**

**Inter-tissue coexpression network analysis reveals *DPP4* as an important gene in heart to blood communication**

Quan Long^1,2^,*, Carmen Argmann^1,2^,*, Sander M. Houten^1^, Tao Huang^1,2^, Siwu Peng^1^, Yong Zhao^1,2^, Zhidong Tu^1,2^, The GTEx Consortium, Jun Zhu^1,2,†^

^1^Department of Genetics and Genomic Sciences, Icahn School of Medicine at Mount Sinai, New York, NY, 10029, USA; ^2^Icahn Institute of Genomics and Multiscale Biology, Icahn School of Medicine at Mount Sinai, New York, NY, 10029, USA

* Equal contribution

† Correspondence should be addressed to

Jun Zhu, Ph.D

Icahn School of Medicine at Mount Sinai

One Gustave L. Levy Place, Box 1498

New York, NY 10029-6574

Fax: 646-537-8660

Telephone: 212-659-8942

Email: [jun.zhu@mssm.edu](mailto:jun.zhu@mssm.edu)

**Determining the optimal number of principal components to correct**

The gene expression levels sometimes may be disrupted by technical or biological reasons. If these technical and biological factors are not the study’s research interests, their effects on gene expression level should be removed. When the technical and biological co-factors are not clear or have too many missing values, the principal component (PC) of the gene expression matrix will be used to represent the covariates[^1^](#_ENREF_1). But how many PCs should be used is still an open question. If too few PCs are corrected, the results may be misled by unwanted factors; if too many PCs are corrected, there will no signal left. It is a dilemma to choose the optimal number of PCs to correct. Some empirical studies suggested “rule of ten” [^2^](#_ENREF_2) for regression analysis. It has not been studied in co-expression network field.

To balance between removing the irrelevant covariates and reserving co-expression structure, the optimal number of principal components to correct in each tissue was chosen based on Mantel test[^3^](#_ENREF_3),[^4^](#_ENREF_4) of the gene expression similarity matrix and the GO based gene similarity matrix.

We used the gene similarity matrix based on Gene Ontology (GO) as the benchmark and then compared the gene expression similarity with the GO based gene similarity matrix. If the gene expression similarity is most similar to the GO based gene similarity matrix, the number of PCs used to correct this data is considered as optimal.

***The construction of GO based gene similarity matrix***

To evaluate the gene similarity based on GO annotation, two methods were applied: the Jaccard similarity coefficient and Pearson correlation coefficient. The Jaccard similarity coefficient was defined as following:

Supposing the two genes $G_{1}$ and $G_{2}$ were annotated onto two sets of GO terms $GO_{1}=\{go_{1}^{1},go_{1}^{2},\cdots,go_{1}^{m}\}$ and$GO_{2}=\{go_{2}^{1},go_{2}^{2},\cdots,go_{2}^{n}\}$, the Jaccard functional similarity[^5^](#_ENREF_5),[^6^](#_ENREF_6) was computed by equation (1):

$Jaccard\text{ }Similarity(G_{1},G_{2})=\frac{|GO_{1}\cap GO_{2}|}{|GO_{1}\cup GO_{2}|}$ (1)

The correlation similarity was defined as following:

Supposing there were $N$ GO terms, each gene can be represented with a $N$dimensional vector. If gene$G_{i}$ was annotated onto GO term$j$, then the $j$element of the vector for gene$G_{i}$was 1; otherwise, it was 0. The Pearson functional similarity between two genes was the absolute value of Pearson correlation coefficient of the GO binary vector of these two genes.

***The construction of gene expression similarity matrix***

Beside gender, we used 1, 2, 3, 4, 5, 6, 7, 8, 9, 10, 15 PCs as covariates. These covariates were corrected with linear regression method. The gene expression residual of gene $i$in individual $j$was computed as following:

$R_{i}^{j}=E_{i}^{j}-\sum_{k=1}^{N} \beta_{ik}PC_{k}^{j}-\beta_{i\text{ }Gender}Gender^{j}-\varepsilon_{i}$ (2)

where $E_{i}^{j}$is the expression level of gene $i$in individual $j$,$PC_{k}^{j}$ is the k-th PC in individual $j$, $N$is the number of PCs considered as covariates, $\beta_{ik}$is the regression coefficient of gene $i$on PC $k$, $Gender^{j}$ is the gender of individual $j$, $\beta_{i\text{ }Gender}$ is the regression coefficient of gene $i$on gender, $\varepsilon_{i}$is the intercept from the regression model of gene $i$.

The gene expression residual was used to compute the gene expression similarity matrix. To be comparable with Jaccard similarity, the absolute values of the Pearson correlation coefficient in the gene expression similarity matrix were used.

***Comparing the gene expression similarity with the GO based gene similarity matrix***

Mantel test [^3^](#_ENREF_3) was applied to compare the gene expression similarity with the GO based gene similarity matrix. Mantel test calculates the correlation coefficient $r$ of $\text{N}\times\left(\text{N}-1\right)/2$pairwise relations for all entries in two $\text{N}\times\text{N}$matrixes. Its significance was evaluated by permuting the matrices, calculating the permuted $r_{permute}$and comparing the original $r$to$r_{permute}$.

The Mantel test correlation $r$is calculated based on the $(i,j)$elements of two $\text{N}\times\text{N}$matrixes with Pearson correlation statistic[^4^](#_ENREF_4)

$r(M_{Expression},M_{GO})=\frac{\sum_{i<j} (e_{ij}^{Expression}-\overline{e^{Expression}})(e_{ij}^{GO}-\overline{e^{GO}})}{\sqrt{\sum_{i<j} (e_{ij}^{Expression}-\overline{e^{Expression}})^{2}}\sqrt{\sum_{i<j} (e_{ij}^{GO}-\overline{e^{GO}})^{2}}}$ (3)

where $e_{ij}^{Expression}$ and$e_{ij}^{GO}$are the expression and GO based similarity between gene$i$and $j$, i.e. the $(i,j)$elements from expression and GO based similarity matrix$M_{Expression}$and $M_{GO}$, $\overline{e^{Expression}}$and $\overline{e^{GO}}$are the mean of all pairs$i<j$in$M_{Expression}$and $M_{GO}$.

A positive $r$indicates that$M_{Expression}$and $M_{GO}$are more consistent, i.e. if the gene expression profiles are similar, the GO annotation will be similar, too.

***Selection of correction principal components***

Two methods, Jaccard and correlation, were applied to evaluate GO based gene similarity. The Mantel tests of these two GO based gene similarity methods were averaged and divided by the r of correcting one PC to get the relative mean Mantel test r. In **Figure S1**, the Mantel test r between GO based gene similarity matrix and gene expression similarity matrix with different number of PCs corrected were shown. The star indicates the chosen optimal number of PCs to correct. Usually, it is the peak of r. But since the curve is irregular, sometimes it is second highest points by considering both r value and the trend of r. The optimal numbers of PCs to correct in adipose, artery, heart, lung, muscle, nerve, skin, thyroid and whole blood were 5, 3, 5, 8, 4, 4, 5, 4 and 7, respectively.

The curves in Figure S1 represent the relative mean Mantel test r between GO based Jaccard and correlation similarity matrix and gene expression similarity matrix with different number of PCs corrected. The optimal numbers of PCs to correct in adipose, artery, heart, lung, muscle, nerve, skin, thyroid and whole blood were 5, 3, 5, 8, 4, 4, 5, 4 and 7, respectively.

**References**

1 Pickrell, J. K. *et al.* Understanding mechanisms underlying human gene expression variation with RNA sequencing. *Nature* **464**, 768-772, doi:10.1038/nature08872 (2010).

2 Peduzzi, P., Concato, J., Kemper, E., Holford, T. R. & Feinstein, A. R. A simulation study of the number of events per variable in logistic regression analysis. *Journal of clinical epidemiology* **49**, 1373-1379 (1996).

3 Mantel, N. The detection of disease clustering and a generalized regression approach. *Cancer research* **27**, 209-220 (1967).

4 Shannon, W. D., Watson, M. A., Perry, A. & Rich, K. Mantel statistics to correlate gene expression levels from microarrays with clinical covariates. *Genetic epidemiology* **23**, 87-96, doi:10.1002/gepi.1115 (2002).

5 Wang, J. Z., Du, Z., Payattakool, R., Yu, P. S. & Chen, C. F. A new method to measure the semantic similarity of GO terms. *Bioinformatics* **23**, 1274-1281, doi:10.1093/bioinformatics/btm087 (2007).

6 Du, Z., Li, L., Chen, C. F., Yu, P. S. & Wang, J. Z. G-SESAME: web tools for GO-term-based gene similarity analysis and knowledge discovery. *Nucleic acids research* **37**, W345-349, doi:10.1093/nar/gkp463 (2009).

**Figure S1** The optimal numbers of PCs to correct in each tissue


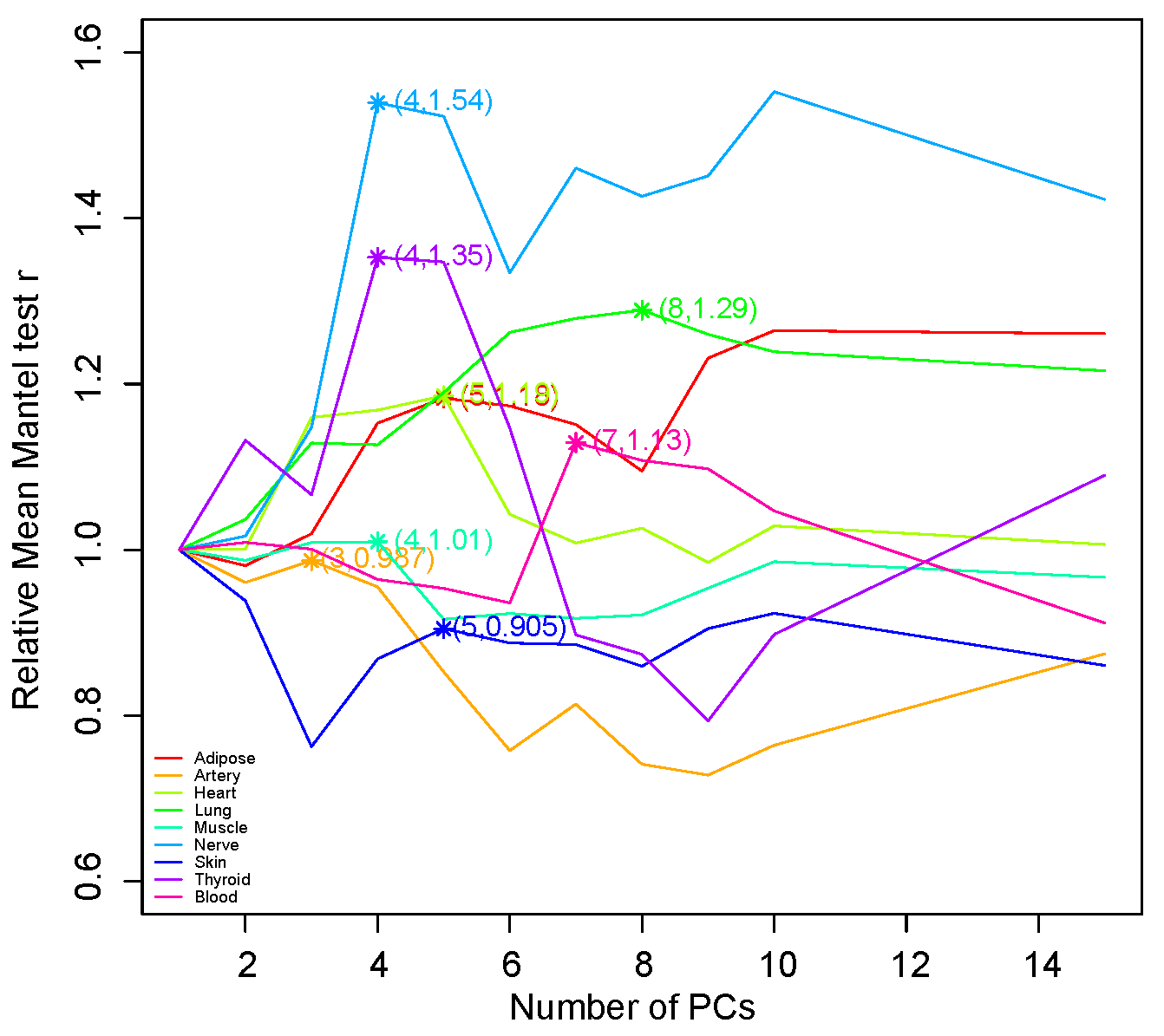


**Figure S2** Histograms of correlation coefficients between sample ischemic time and RINs with gene expression profiles in 9 tissues. Red lines are for correlation with RINs, and blue lines are for correlation with sample ischemic time. Solid lines are for empirical gene expression profiles in the study, dash lines are for permuted data.


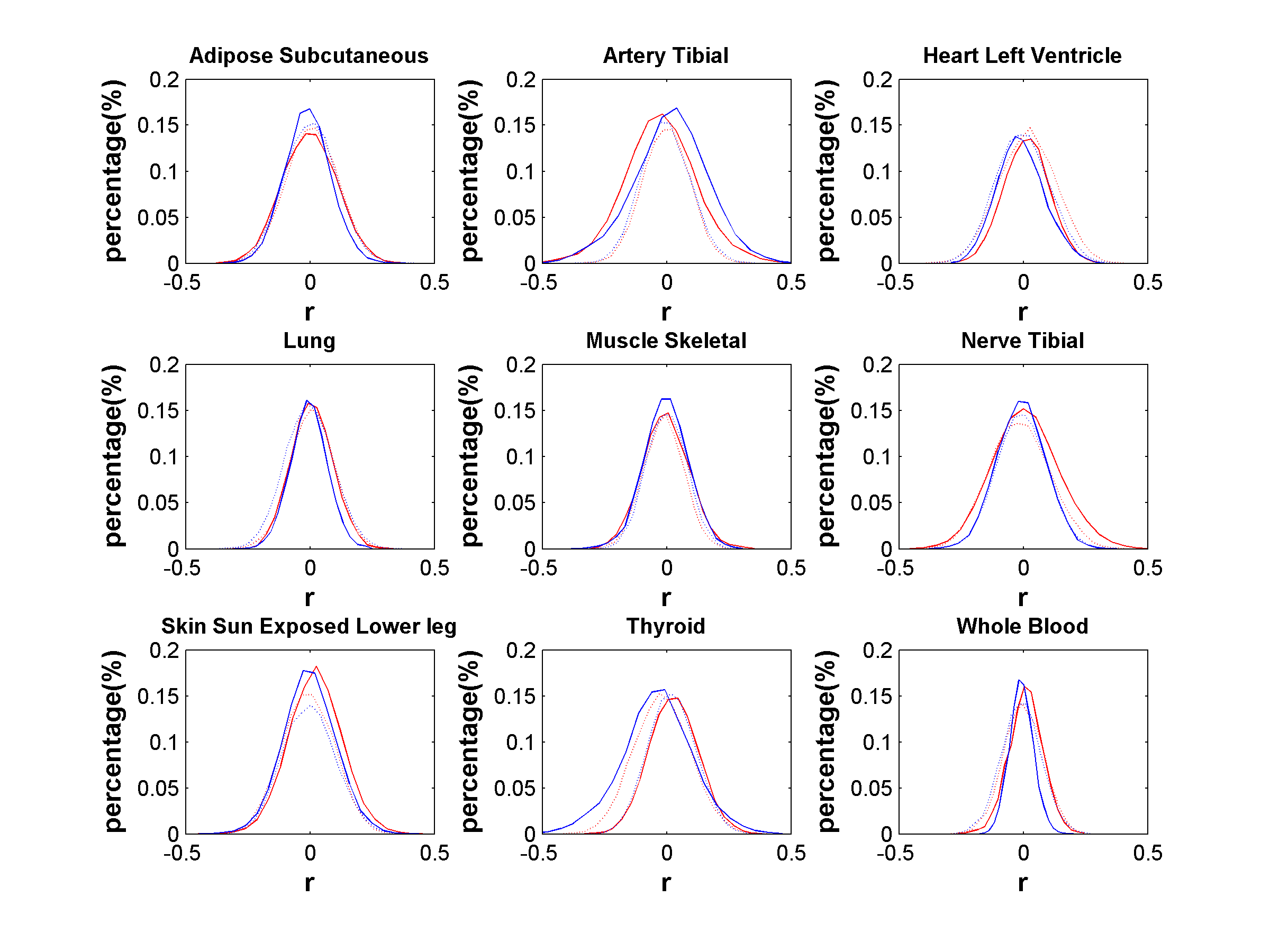

Supplement: Additional file 2: — Supporting notes. Figure S1. The optimal numbers of principal components (PCs) to correct in each tissue. Figure S2. Histograms of correlation coefficients between sample ischemic time and RINs with gene expression profiles in nine tissues. Red lines are for correlation with RINs, and blue lines are for correlation with sample ischemic time. Solid lines are for empirical gene expression profiles in the study, dashed lines are for permuted data. (DOCX 500 kb) [file 13073_2016_268_MOESM2_ESM.docx]
