# Supplementary material for: Inter-tissue coexpression network analysis reveals DPP4 as an important gene in heart to blood communication
Source: Genome Med. 2016 Feb 9;8:15. doi: 10.1186/s13073-016-0268-1 (PMC4746932; doi:10.1186/s13073-016-0268-1)
Supplement: Additional file 4: Table S3. — Number of significant gene-modules identified for each tissue pair. (PDF 42 kb) [file 13073_2016_268_MOESM4_ESM.pdf]

Tissue	# of modules
Adipose_Subcutaneous.Heart_Left_Ventricle	609
Adipose_Subcutaneous.Muscle_Skeletal	507
Adipose_Subcutaneous.Nerve_Tibial	745
Adipose_Subcutaneous.Skin_Sun_Exposed_Lower_leg	756
Adipose_Subcutaneous.Whole_Blood	418
Artery_Tibial.Adipose_Subcutaneous	724
Artery_Tibial.Heart_Left_Ventricle	1034
Artery_Tibial.Muscle_Skeletal	685
Artery_Tibial.Nerve_Tibial	1032
Artery_Tibial.Skin_Sun_Exposed_Lower_leg	719
Artery_Tibial.Whole_Blood	380
Heart_Left_Ventricle.Nerve_Tibial	1126
Heart_Left_Ventricle.Skin_Sun_Exposed_Lower_leg	576
Heart_Left_Ventricle.Whole_Blood	304
Lung.Adipose_Subcutaneous	437
Lung.Artery_Tibial	513
Lung.Heart_Left_Ventricle	504
Lung.Muscle_Skeletal	208
Lung.Nerve_Tibial	522
Lung.Skin_Sun_Exposed_Lower_leg	9
Lung.Thyroid	445
Lung.Whole_Blood	105
Muscle_Skeletal.Heart_Left_Ventricle	512
Muscle_Skeletal.Nerve_Tibial	541
Muscle_Skeletal.Skin_Sun_Exposed_Lower_leg	344
Muscle_Skeletal.Whole_Blood	164
Nerve_Tibial.Skin_Sun_Exposed_Lower_leg	514
Thyroid.Adipose_Subcutaneous	957
Thyroid.Artery_Tibial	1513
Thyroid.Heart_Left_Ventricle	1050
Thyroid.Muscle_Skeletal	635
Thyroid.Nerve_Tibial	908
Thyroid.Skin_Sun_Exposed_Lower_leg	589
Thyroid.Whole_Blood	391
Whole_Blood.Nerve_Tibial	423
Whole_Blood.Skin_Sun_Exposed_Lower_leg	248
.	21147 total
