# Supplementary material for: Inter-tissue coexpression network analysis reveals DPP4 as an important gene in heart to blood communication
Source: Genome Med. 2016 Feb 9;8:15. doi: 10.1186/s13073-016-0268-1 (PMC4746932; doi:10.1186/s13073-016-0268-1)
Supplement: Additional file 6: Table S5. — Names and IDs of GO terms used in Fig. 4b. (PDF 11 kb) [file 13073_2016_268_MOESM6_ESM.pdf]

Supplemental Table 5

index	GO Level 3 category	G O annotation
1	GO:0030029	actin filament-based process
2	GO:0002504	antigen processing and presentation of peptide or polysaccharide antigen via MHC class II
3	GO:0031295	T cell costimulation
4	GO:0035966	response to topologically incorrect protein
5	GO:0060041	retina development in camera-type eye
6	GO:0006323	DNA packaging
7	GO:0009582	detection of abiotic stimulus
8	GO:0009581	detection of external stimulus
9	GO:0006903	vesicle targeting
10	GO:0051650	establishment of vesicle localization
11	GO:0001505	regulation of neurotransmitter levels
12	GO:0003001	generation of a signal involved in cell-cell signaling
13	GO:0061061	muscle structure development
14	GO:0007507	heart development
15	GO:0050879	multicellular organismal movement
16	GO:0045061	thymic T cell selection
17	GO:0006403	RNA localization
18	GO:0043414	macromolecule methylation
19	GO:0048514	blood vessel morphogenesis
20	GO:0001568	blood vessel development
21	GO:0001525	angiogenesis
22	GO:0050808	synapse organization
23	GO:0010324	membrane invagination
24	GO:0051607	defense response to virus
25	GO:0035383	thioester metabolic process
26	GO:0031589	cell-substrate adhesion
27	GO:0001101	response to acid
28	GO:0003206	cardiac chamber morphogenesis
29	GO:0003205	cardiac chamber development

30	GO:0007423	sensory organ development
31	GO:0071216	cellular response to biotic stimulus
32	GO:0006099	tricarboxylic acid cycle
33	GO:0030595	leukocyte chemotaxis
34	GO:0051303	establishment of chromosome localization
35	GO:0045123	cellular extravasation
36	GO:0007585	respiratory gaseous exchange
37	GO:0009595	detection of biotic stimulus
38	GO:0002238	response to molecule of fungal origin
39	GO:0021700	developmental maturation
40	GO:0033002	muscle cell proliferation
41	GO:0009612	response to mechanical stimulus
42	GO:0003407	neural retina development
43	GO:0001754	eye photoreceptor cell differentiation
44	GO:0044236	multicellular organismal metabolic process
45	GO:0040012	regulation of locomotion
46	GO:0042391	regulation of membrane potential
47	GO:0043299	leukocyte degranulation
48	GO:0045730	respiratory burst
49	GO:0043383	negative T cell selection
50	GO:0043900	regulation of multi-organism process
51	GO:0044364	disruption of cells of other organism
52	GO:0043954	cellular component maintenance
53	GO:0050803	regulation of synapse structure and activity
54	GO:0048546	digestive tract morphogenesis
55	GO:0006790	sulfur compound metabolic process
56	GO:0001503	ossification
57	GO:0001709	cell fate determination
58	GO:0060021	palate development
59	GO:0048729	tissue morphogenesis
60	GO:0010927	cellular component assembly involved in morphogenesis
61	GO:0034330	cell junction organization
62	GO:0071705	nitrogen compound transport
63	GO:0010243	response to organonitrogen compound

64	GO:0060323	head morphogenesis
65	GO:0060325	face morphogenesis
66	GO:0016337	cell-cell adhesion
67	GO:0042267	natural killer cell mediated cytotoxicity
68	GO:0048598	embryonic morphogenesis
69	GO:0060560	developmental growth involved in morphogenesis
70	GO:0097194	execution phase of apoptosis
71	GO:0010817	regulation of hormone levels
72	GO:0006725	cellular aromatic compound metabolic process
73	GO:0055094	response to lipoprotein particle stimulus
74	GO:0007420	brain development
75	GO:0007618	mating
76	GO:0021915	neural tube development
77	GO:0006081	cellular aldehyde metabolic process
78	GO:0048588	developmental cell growth
79	GO:0061024	membrane organization
80	GO:0002237	response to molecule of bacterial origin
81	GO:0010035	response to inorganic substance
82	GO:0031341	regulation of cell killing
83	GO:0002218	activation of innate immune response
84	GO:0046148	pigment biosynthetic process
85	GO:0010171	body morphogenesis
86	GO:0048736	appendage development
87	GO:0050673	epithelial cell proliferation
88	GO:0035295	tube development
89	GO:0060322	head development
90	GO:0060324	face development
91	GO:0001822	kidney development
92	GO:0035107	appendage morphogenesis
93	GO:0021510	spinal cord development
94	GO:0048771	tissue remodeling
95	GO:0060349	bone morphogenesis
96	GO:0042440	pigment metabolic process
97	GO:0030324	lung development

98	GO:0034101	erythrocyte homeostasis
99	GO:0072593	reactive oxygen species metabolic process
100	GO:0045103	intermediate filament-based process
101	GO:0006970	response to osmotic stress
102	GO:0001553	luteinization
103	GO:0090150	establishment of protein localization to membrane
104	GO:0001666	response to hypoxia
105	GO:0007163	establishment or maintenance of cell polarity
106	GO:0003002	regionalization
107	GO:0007272	ensheathment of neurons
108	GO:0061351	neural precursor cell proliferation
109	GO:0070482	response to oxygen levels
110	GO:0061053	somite development
111	GO:0048286	lung alveolus development
112	GO:0051098	regulation of binding
113	GO:0048568	embryonic organ development
114	GO:0009991	response to extracellular stimulus
115	GO:0001889	liver development
116	GO:0009636	response to toxic substance
117	GO:0022406	membrane docking
118	GO:0060135	maternal process involved in female pregnancy
119	GO:0071496	cellular response to external stimulus
120	GO:0008217	regulation of blood pressure
121	GO:0060004	reflex
122	GO:0002377	immunoglobulin production
123	GO:0061439	kidney vasculature morphogenesis
124	GO:0061438	renal system vasculature morphogenesis
125	GO:0001763	morphogenesis of a branching structure
126	GO:0031099	regeneration
127	GO:0032196	transposition
128	GO:0040008	regulation of growth
129	GO:0001964	startle response
130	GO:0042303	molting cycle
131	GO:0002088	lens development in camera-type eye

132	GO:0021542	dentate gyrus development
133	GO:0060042	retina morphogenesis in camera-type eye
134	GO:0032835	glomerulus development
135	GO:0043449	cellular alkene metabolic process
136	GO:1900673	olefin metabolic process
137	GO:0097006	regulation of plasma lipoprotein particle levels
138	GO:0022612	gland morphogenesis
139	GO:0034381	plasma lipoprotein particle clearance
140	GO:0071827	plasma lipoprotein particle organization
141	GO:0045494	photoreceptor cell maintenance
142	GO:0010876	lipid localization
143	GO:0051235	maintenance of location
144	GO:0051186	cofactor metabolic process
145	GO:0006730	one-carbon metabolic process
146	GO:0051651	maintenance of location in cell
147	GO:0071214	cellular response to abiotic stimulus
148	GO:0009314	response to radiation
149	GO:0042493	response to drug
150	GO:0001816	cytokine production
151	GO:0002757	immune response-activating signal transduction
152	GO:0070661	leukocyte proliferation
153	GO:0048534	hematopoietic or lymphoid organ development
154	GO:0043062	extracellular structure organization
155	GO:0048002	antigen processing and presentation of peptide antigen
156	GO:0019884	antigen processing and presentation of exogenous antigen
157	GO:0007017	microtubule-based process
158	GO:0051301	cell division
159	GO:0007059	chromosome segregation
160	GO:0007586	digestion
161	GO:0006956	complement activation
162	GO:0006959	humoral immune response
163	GO:0002443	leukocyte mediated immunity
164	GO:0002250	adaptive immune response
165	GO:0006091	generation of precursor metabolites and energy

166	GO:0022613	ribonucleoprotein complex biogenesis
167	GO:0022411	cellular component disassembly
168	GO:0072594	establishment of protein localization to organelle
169	GO:0006413	translational initiation
