# Supplementary material for: Inter-tissue coexpression network analysis reveals DPP4 as an important gene in heart to blood communication
Source: Genome Med. 2016 Feb 9;8:15. doi: 10.1186/s13073-016-0268-1 (PMC4746932; doi:10.1186/s13073-016-0268-1)
Supplement: Additional file 5: Table S4. — Top 20 significant modules in terms of p value of GO enrichment analysis in all tissue pairs. (PDF 2423 kb) [file 13073_2016_268_MOESM5_ESM.pdf]

Tissue: Heart_Left_Ventricle=>Adipose_Subcutaneous Type: asymmetric

SourceGene: CRABP1(ENSG00000166426.7)

TargetGeneSet: RP11 SRGAP2P2 CDC42SE1 PMVK PTPN7 MRPL33
SGOL2 PHLDB2 COX17 PSAPL1 C4orf3 NDUFC1 NDUFS4 COX7C FAM65B RPS6KA2
MRPS24 TP53TG1 SGK196 TMEM67 COX6C DEPTOR FAM27E2 CNTRL QSOX2 FBXW5
ZNF215 TBC1D10C TMEM106C DNAJC14 TMEM198B PARP4 ELF1
FLVCR2 NDUFB1 MOK NDUFB10 GABARAPL2 MAP1LC3B IL17C SCIMP
NLRP1 CBX1 UQCR11 S1PR4 PRR22 HSH2D FKBP8 C19orf54 SAMHD1
NCOA3 ZBP1 RP4 ATP5J HSF2BP TTC38

GOBPID	Pvalue	OddsRatio	ExpCount	Count	Size	Term
GO:0022904	5.98E-10	34.25119617	0.305584389	8	96	respiratory electron transport chain
GO:0022900	8.59E-09	23.85797828	0.426544876	8	134	electron transport chain
GO:0045333	1.36E-08	22.42105263	0.452010242	8	142	cellular respiration
GO:0015980	4.90E-06	9.932054216	0.977233409	8	307	energy derivation by oxidation of organic compounds
GO:0006120	4.91E-06	42.77678571	0.114594146	4	36	"mitochondrial electron transport, NADH to ubiquinone"

GO:0006091 6.71E-06 8.119331742 1.362397066 9 428 generation of precursor metabolites and energy

GO:0042773 1.45E-05 31.80952381 0.149609024 4 47 ATP synthesis coupled electron transport

GO:0042775 1.45E-05 31.80952381 0.149609024 4 47 mitochondrial ATP synthesis coupled electron transport

Tissue: Adipose_Subcutaneous=>Heart_Left_Ventricle Type: asymmetric

SourceGene: CTD-2184D3.7(ENSG00000259709.1) Novel antisense

TargetGeneSet: RPL11 IPO9-AS1 RP11 RAB1A FAHD2B KANSL1L RPL37A MME-AS1 RPL39P3 PDAP1 TAS2R5 RPL30 PTCH1 RPL35 TOR1B EIF5AL1 RPLP2 RPS25 ERH SLIRP SRP14 MAP2K5 WHAMM MYL12B RPL36 CDC37 EEF1B2P3 6-Sep

GOBPID	Pvalue	OddsRatio	ExpCount	Count	Size	Term
GO:0006414	1.09E-14	111.984375	0.152584596	9	105	translational elongation
GO:0006415	3.44E-13	106.3725672	0.132239983	8	91	translational termination
GO:0006612	3.48E-13	74.40625	0.222337554	9	153	protein targeting to membrane
GO:0006614	1.11E-12	90.93100714	0.152584596	8	105	SRP-dependent cotranslational protein targeting to membrane

GO:0006613	1.20E-12	89.99686028	0.154037783	8	106
cotranslational protein targeting to membrane					
GO:0045047	1.20E-12	89.99686028	0.154037783	8	106
protein targeting to ER					
GO:0072599	1.20E-12	89.99686028	0.154037783	8	106
establishment of protein localization to endoplasmic reticulum					
GO:0070972	2.89E-12	80.11188811	0.171476022	8	118
protein localization to endoplasmic reticulum					
GO:0043624	2.38E-11	60.62599469	0.222337554	8	153
cellular protein complex disassembly					
GO:0043241	3.08E-11	58.58461538	0.229603488	8	158
protein complex disassembly					
GO:0034623	6.69E-11	52.87859129	0.252854474	8	174
cellular macromolecular complex disassembly					
GO:0032984	8.40E-11	51.31443995	0.260120407	8	179
macromolecular complex disassembly					
GO:0000184	2.27E-10	63.91964286	0.172929209	7	119
"nuclear-transcribed mRNA catabolic process, nonsense-mediated decay"					
GO:0072594	2.39E-10	44.69073783	0.296450073	8	204
establishment of protein localization to organelle					
GO:0006605	3.35E-10	27.36285266	0.688810463	10	474
protein targeting					
GO:0019080	1.22E-09	49.60416667	0.219431181	7	151
viral genome expression					
GO:0019083	1.22E-09	49.60416667	0.219431181	7	151

viral transcription

GO:0006413	1.27E-09	49.25862069	0.220884368	7	152
translational initiation

GO:0000956	2.68E-09	44.03703704	0.245588541	7	169
nuclear-transcribed mRNA catabolic process

GO:0071845	3.29E-09	31.55852843	0.412705003	8	284
cellular component disassembly at cellular level

GO:0022411	3.67E-09	31.0989011	0.41851775	8	288
cellular component disassembly

GO:0006402	3.84E-09	41.69298246	0.25866722	7	178	mRNA
catabolic process

GO:0006401	1.03E-08	35.93939394	0.297903259	7	205	RNA
catabolic process

GO:0019058	2.34E-08	31.70982143	0.335686112	7	231
viral infectious cycle

GO:0022415	5.28E-08	28.01778656	0.377828524	7	260
viral reproductive process

GO:0033365	1.74E-07	18.48138958	0.687357276	8	473
protein localization to organelle

GO:0016032	2.19E-06	15.75	0.65538717	7	451	viral
reproduction

Tissue: Heart_Left_Ventricle=>Adipose_Subcutaneous	Type:	asymmetric

SourceGene: CTD-2290C23.1(ENSG00000224282.3)

TargetGeneSet: MTND1P23 RP11 TNFRSF4 KCNAB2 C1QC C1QB ARID1A C1orf38 CSF3R C1orf162 ADORA3 RP4 SRGAP2P2 C1orf56 SLAMF8 PIGM CD84 CEP350 PTPRC BIRC6 ITGB6 PDCD1 BRK1 UBE2E2 STAB1 FAM208A PHC3 MFSD7 SH3TC1 BOD1L TXK PDGFRA BMP2K RPS3A FYB EMB SKP1 DDX46 CSF1R DOCK2 ATF6B GPSM3 CUTA FGD2 WDR11 RP3 MPLKIP CCL24 IFRD1 TFEC TBXAS1 PRSS1 SLA SYK WDFY4 RPS3AP5 PNLIPRP2 MS4A6A MS4A7 FERMT3 BAD RNASEH2C BCRP1 RHOD AIP SLCO2B1 MYO7A ATM UBE4A KCNJ5 CD4 PTPN6 CD163 C3AR1 ITPR2 ALG10B MLL4 NCKAP1L RBMS1P1 SELPLG GIT2 MED13L LACC1 MYCBP2-AS1 SLC7A7 FANCM VTI1B SIVA1 FMN1 USP8 STUB1 MEFV SLC7A6 ARRB2 POLR2A TMEM106A TMEM106A-AS1 RHBDF2 TNRC6C C19orf35 CLEC4G CD209 EIF3G GMIP TYROBP RASGRP4 JOSD2 LILRA1 SIGLEC1 ADAMTS1 TRPM2 MCM3AP EP300 C22orf32 NFAM1 WAS CACNA1F VSIG4 BTK DDX26B MPP1

GOBPID	Pvalue	OddsRatio	ExpCount	Count	Size	Term
GO:0002253	1.53E-08	8.129535604	2.048370355	14	299	activation of immune response
GO:0046649	3.70E-08	6.441882638	2.966369109	16	433	lymphocyte activation

GO:0051249	6.51E-08	7.883979328	1.938758563	13	283
regulation of lymphocyte activation					
GO:0050864	7.76E-08	17.19584525	0.554909695	8	81
regulation of B cell activation					
GO:0045577	7.90E-08	63.56382979	0.116462529	5	17
regulation of B cell differentiation					
GO:0050865	8.49E-08	7.020275344	2.349802782	14	343
regulation of cell activation					
GO:0050851	1.59E-07	12.48947368	0.842640648	9	123
antigen receptor-mediated signaling pathway					

GO:0050778	1.76E-07	6.589176471	2.493668258	14	364	
positive regulation of immune response						
GO:0045579	2.54E-07	120.8168421	0.061656633	4	9	
positive regulation of B cell differentiation						
GO:0042110	2.61E-07	6.938668491	2.185385094	13	319	T
cell activation						
GO:0002694	2.80E-07	6.892630625	2.199086568	13	321	
regulation of leukocyte activation						
GO:0002429	3.10E-07	11.47419355	0.911148017	9	133	
immune response-activating cell surface receptor signaling pathway						

GO:0002768	6.09E-07	10.53111111	0.986506124	9	144	
immune response-regulating cell surface receptor signaling pathway						
GO:0002521	1.02E-06	6.6882283	2.068922566	12	302	
leukocyte differentiation						
GO:0030098	1.32E-06	8.199930499	1.397550343	10	204	
lymphocyte differentiation						
GO:0050853	1.42E-06	31.75531915	0.198671372	5	29	B
cell receptor signaling pathway						

GO:0050871	1.76E-06	19.22580645	0.369939797	6	54	positive regulation of B cell activation
GO:0002757	4.69E-06	7.054681648	1.609923189	10	235	immune response-activating signal transduction
GO:0050900	5.87E-06	6.868524734	1.651027611	10	241	leukocyte migration
GO:0001817	6.09E-06	5.566928351	2.459414573	12	359	regulation of cytokine production
GO:0030183	6.89E-06	14.86992716	0.465850114	6	68	B cell differentiation

GO:0002764	7.55E-06	6.663204608	1.698982769	10	248	
immune response-regulating signaling pathway						
GO:0042113	9.97E-06	8.613565745	1.048162757	8	153	B
cell activation						
GO:0002252	1.27E-05	5.155080214	2.644384472	12	386	
immune effector process						
GO:0006954	1.56E-05	4.680659864	3.165040482	13	462	
inflammatory response						
GO:0030097	1.59E-05	4.669922481	3.171891219	13	463	
hemopoiesis						

GO:0001816 1.59E-05 5.03070136 2.706041104 12 395
cytokine production

GO:0042098 1.62E-05 9.761750881 0.808386963 7 118 T
cell proliferation

GO:0051251 1.64E-05 6.866990291 1.472908449 9 215
positive regulation of lymphocyte activation

Tissue: Heart_Left_Ventricle=>Adipose_Subcutaneous Type: cluster

SourceGene: CTD-2290C23.1(ENSG00000224282.3)

TargetGeneSet: MTND1P23 RP11 TNFRSF4 KCNAB2 C1QC C1QB ARID1A
C1orf38 CSF3R C1orf162 ADORA3 RP4 SRGAP2P2 C1orf56 SLAMF8 PIGM
CD84 CEP350 PTPRC BIRC6 ITGB6 PDCD1 BRK1 UBE2E2 STAB1 FAM208A PHC3
MFSD7 SH3TC1 BOD1L TXK PDGFRA BMP2K RPS3A FYB EMB SKP1
DDX46 CSF1R DOCK2 ATF6B GPSM3 CUTA FGD2 WDR11 RP3 MPLKIP
CCL24 IFRD1 TFEC TBXAS1 PRSS1 SLA SYK WDFY4 RPS3AP5 PNLIPRP2
MS4A6A MS4A7 FERMT3 BAD RNASEH2C BCRP1 RHOD AIP SLCO2B1
MYO7A ATM UBE4A KCNJ5 CD4 PTPN6 CD163 C3AR1 ITPR2 ALG10B MLL4
NCKAP1L RBMS1P1 SELPLG GIT2 MED13L LACC1 MYCBP2-AS1 SLC7A7 FANCM
VTI1B SIVA1 FMN1 USP8 STUB1 MEFV SLC7A6 ARRB2 POLR2A TMEM106A
TMEM106A-AS1 RHBDF2 TNRC6C C19orf35 CLEC4G CD209 EIF3G GMIP
TYROBP RASGRP4 JOSD2 LILRA1 SIGLEC1 ADAMTS1 TRPM2 MCM3AP EP300 C22orf32
NFAM1 WAS CACNA1F VSIG4 BTK DDX26B MPP1

GOBPID	Pvalue	OddsRatio	ExpCount	Count	Size	Term
GO:0002253	1.53E-08	8.129535604	2.048370355	14	299	activation of immune response
GO:0046649	3.70E-08	6.441882638	2.966369109	16	433	lymphocyte activation
GO:0051249	6.51E-08	7.883979328	1.938758563	13	283	regulation of lymphocyte activation

GO:0050864	7.76E-08	17.19584525	0.554909695	8	81
regulation of B cell activation					
GO:0045577	7.90E-08	63.56382979	0.116462529	5	17
regulation of B cell differentiation					
GO:0050865	8.49E-08	7.020275344	2.349802782	14	343
regulation of cell activation					
GO:0050851	1.59E-07	12.48947368	0.842640648	9	123
antigen receptor-mediated signaling pathway					

GO:0050778	1.76E-07	6.589176471	2.493668258	14	364	positive regulation of immune response
GO:0045579	2.54E-07	120.8168421	0.061656633	4	9	positive regulation of B cell differentiation
GO:0042110	2.61E-07	6.938668491	2.185385094	13	319	T cell activation
GO:0002694	2.80E-07	6.892630625	2.199086568	13	321	regulation of leukocyte activation
GO:0002429	3.10E-07	11.47419355	0.911148017	9	133	immune response-activating cell surface receptor signaling pathway

GO:0002768	6.09E-07	10.53111111	0.986506124	9	144	
immune response-regulating cell surface receptor signaling pathway						
GO:0002521	1.02E-06	6.6882283	2.068922566	12	302	
leukocyte differentiation						
GO:0030098	1.32E-06	8.199930499	1.397550343	10	204	
lymphocyte differentiation						
GO:0050853	1.42E-06	31.75531915	0.198671372	5	29	B
cell receptor signaling pathway						
GO:0050871	1.76E-06	19.22580645	0.369939797	6	54	
positive regulation of B cell activation						

GO:0002757	4.69E-06	7.054681648	1.609923189	10	235	
immune response-activating signal transduction						
GO:0050900	5.87E-06	6.868524734	1.651027611	10	241	
leukocyte migration						
GO:0001817	6.09E-06	5.566928351	2.459414573	12	359	
regulation of cytokine production						
GO:0030183	6.89E-06	14.86992716	0.465850114	6	68	B
cell differentiation						
GO:0002764	7.55E-06	6.663204608	1.698982769	10	248	
immune response-regulating signaling pathway						

GO:0042113	9.97E-06	8.613565745	1.048162757	8	153	B
cell activation						
GO:0002252	1.27E-05	5.155080214	2.644384472	12	386	
immune effector process						
GO:0006954	1.56E-05	4.680659864	3.165040482	13	462	
inflammatory response						
GO:0030097	1.59E-05	4.669922481	3.171891219	13	463	
hemopoiesis						
GO:0001816	1.59E-05	5.03070136	2.706041104	12	395	
cytokine production						

GO:0042098 1.62E-05 9.761750881 0.808386963 7 118 T
cell proliferation

GO:0051251 1.64E-05 6.866990291 1.472908449 9 215
positive regulation of lymphocyte activation

Tissue: Heart_Left_Ventricle=>Adipose_Subcutaneous Type: asymmetric

SourceGene: ENSG00000228649.1

TargetGeneSet: TRIM63 ACTA1 NEB XIRP2 KBTBD10 TTN MYL1 WDR48 ODZ1
RP11 CMYA5 SRPK1 LMOD2 ANKRD2 NRAP RPL23AP64 RP3 POU5F1P3
MYL2 OAS3 MYH7 ATP2A1 MYLPF ENO3 MYH2 MYH1 SPAG9 CACNG1
FOXJ1 CKM TNNT1 C20orf85 EEF1A2 PVALB APOBEC3H

GOBPID	Pvalue	OddsRatio	ExpCount	Count	Size	Term
GO:0006936	2.68E-16	51.03650442	0.479620787	13	239	muscle contraction
GO:0003012	1.08E-15	45.50321146	0.533803889	13	266	muscle system process

GO:0030049	5.39E-15	176.8479263	0.07826448	8	39
muscle filament sliding					
GO:0033275	5.39E-15	176.8479263	0.07826448	8	39
actin-myosin filament sliding					
GO:0070252	1.03E-14	161.210084	0.084284825	8	42
actin-mediated cell contraction					
GO:0030048	1.89E-13	107.3464052	0.118400111	8	59
actin filament-based movement					
GO:0006941	1.08E-10	67.1644385	0.150508615	7	75
striated muscle contraction					
GO:0030029	6.19E-09	17.46071339	0.866929624	10	432
actin filament-based process					
GO:0003010	2.90E-08	1663.961538	0.008027126	3	4
voluntary skeletal muscle contraction					
GO:0014721	2.90E-08	1663.961538	0.008027126	3	4
twitch skeletal muscle contraction					
GO:0061061	6.47E-08	15.77475	0.820773649	9	409
muscle structure development					
GO:0007517	9.97E-08	18.24309927	0.608054806	8	303
muscle organ development					
GO:0010927	4.15E-06	25.25423729	0.246834129	5	123
cellular component assembly involved in morphogenesis					
GO:0003009	6.89E-06	103.8894231	0.038128849	3	19
skeletal muscle contraction					

GO:0045071 1.09E-05 87.46761134 0.044149194 3 22
negative regulation of viral genome replication

GO:0048525 1.09E-05 87.46761134 0.044149194 3 22
negative regulation of viral reproduction

GO:0050879 1.83E-05 72.23578595 0.05217632 3 26
multicellular organismal movement

GO:0050881 1.83E-05 72.23578595 0.05217632 3 26
musculoskeletal movement

Tissue: Heart_Left_Ventricle=>Adipose_Subcutaneous Type: asymmetric

SourceGene: ENSG00000231536.1 antisense RNA

TargetGeneSet: RPS8 ADORA3 FCGR2A NEURL1B RP11 CTB RP4 RSPH9 STK3 RPL8 RP1 PNLIPRP3 NCKAP1L RPL18AP3 TERF1P5 RPL4 RPL13 RPS15 ELAVL1 RPL18A SSBP4 ETV2 RPL18 LILRA1 RPS4X

GOBPID Pvalue OddsRatio ExpCount Count Size Term

GO:0006415 3.44E-13 106.3725672 0.132239983 8 91
translational termination

GO:0006414 1.11E-12 90.93100714 0.152584596 8 105
translational elongation

GO:0006614 1.11E-12 90.93100714 0.152584596 8 105
SRP-dependent cotranslational protein targeting to membrane

GO:0006613 1.20E-12 89.99686028 0.154037783 8 106
cotranslational protein targeting to membrane

GO:0045047 1.20E-12 89.99686028 0.154037783 8 106

protein targeting to ER

GO:0072599	1.20E-12	89.99686028	0.154037783	8	106	
establishment of protein localization to endoplasmic reticulum						
GO:0070972	2.89E-12	80.11188811	0.171476022	8	118	
protein localization to endoplasmic reticulum						
GO:0000184	3.10E-12	79.38461538	0.172929209	8	119	
"nuclear-transcribed mRNA catabolic process, nonsense-mediated decay"						
GO:0019080	2.14E-11	61.48251748	0.219431181	8	151	
viral genome expression						
GO:0019083	2.14E-11	61.48251748	0.219431181	8	151	
viral transcription						
GO:0006413	2.26E-11	61.05128205	0.220884368	8	152	
translational initiation						
GO:0006612	2.38E-11	60.62599469	0.222337554	8	153	
protein targeting to membrane						
GO:0043624	2.38E-11	60.62599469	0.222337554	8	153	
cellular protein complex disassembly						
GO:0043241	3.08E-11	58.58461538	0.229603488	8	158	
protein complex disassembly						
GO:0000956	5.30E-11	54.53989489	0.245588541	8	169	
nuclear-transcribed mRNA catabolic process						
GO:0034623	6.69E-11	52.87859129	0.252854474	8	174	
cellular macromolecular complex disassembly						
GO:0006402	8.03E-11	51.6199095	0.25866722	8	178	mRNA
catabolic process						
GO:0032984	8.40E-11	51.31443995	0.260120407	8	179	
macromolecular complex disassembly						

GO:0072594 2.39E-10 44.69073783 0.296450073 8 204
establishment of protein localization to organelle

GO:0006401 2.49E-10 44.46075752 0.297903259 8 205 RNA
catabolic process

GO:0019058 6.43E-10 39.20524319 0.335686112 8 231
viral infectious cycle

GO:0022415 1.64E-09 34.62271062 0.377828524 8 260
viral reproductive process

GO:0071845 3.29E-09 31.55852843 0.412705003 8 284
cellular component disassembly at cellular level

GO:0022411 3.67E-09 31.0989011 0.41851775 8 288
cellular component disassembly

GO:0016032 1.20E-07 19.42976211 0.65538717 8 451
viral reproduction

GO:0033365 1.74E-07 18.48138958 0.687357276 8 473
protein localization to organelle

GO:0006605 1.76E-07 18.44040938 0.688810463 8 474
protein targeting

Tissue: Heart_Left_Ventricle=>Adipose_Subcutaneous Type: asymmetric

SourceGene: FAM124B(ENSG00000124019.9)

TargetGeneSet: WASF2 RP4 KIF2C DDAH1 RP11 UGGT1 CDCA7 KLF7
ARPC4 FANCD2 CENPE BRCC3 KIF20A ANLN PBK PTP4A3 NAPRT1 MELK E2F7
BUB1B OIP5 CCNB2 PLK1 HIC1 BIRC5 TYMS NDC80 LIG1 E2F1
C22orf29 MT

GOBPID Pvalue OddsRatio ExpCount Count Size Term

GO:0000087	4.56E-11	24.64231137	0.722441354	11	360	M phase of mitotic cell cycle
GO:0051301	5.17E-10	19.37402368	0.907065255	11	452	cell division
GO:0000280	8.13E-10	21.79876161	0.702373538	10	350	nuclear division
GO:0007067	8.13E-10	21.79876161	0.702373538	10	350	mitosis
GO:0048285	1.39E-09	20.55847953	0.742509169	10	370	organelle fission
GO:0000236	1.48E-08	47.36268575	0.170576431	6	85	mitotic prometaphase
GO:0051329	7.65E-06	12.01735093	0.760570203	7	379	interphase of mitotic cell cycle
GO:0051325	8.77E-06	11.7576555	0.776624455	7	387	interphase

Tissue: Heart_Left_Ventricle=>Adipose_Subcutaneous Type: cluster

SourceGene: GABRA1(ENSG00000022355.10)

TargetGeneSet: PANK4 KCNAB2 ESPN C1QC C1QB FUCA1 UBXN11 CD52 FGR
C1orf38 RAB42 LAPTM5 BRD8 SLC6A9 CD53 C1orf88 C1orf162 ADORA3
APH1A CTSS ARHGAP30 FCER1G FAM20B NPL PRG4 PTPRC RP11 CR1
RCOR3 CDC42BPA DISC1 NID1 GPR137B VAMP8 BDP1 KYNU RPE
C2orf62 SLC11A1 ARL4C FANCD2 CMTM7 HCLS1 CD86 CTBP1 RBPJ TBC1D1
CCDC109B FYB ITGA1 HEXB PAPD4 CTNNA1 GPX3 DOCK2 MGAT1
KIF13A C2 UNC5CL ZC3H12D WTAP AVL9 PILRA PMPCB TFEC TBXAS1 CTSB
MSR1 PREX2 SLA KIAA1432 TLN1 FAM27E2 SYK COL15A1 DAB2IP RSU1
VSTM4 HPSE2 COMMD9 PRG2 SERPING1 LPXN MS4A6A MS4A7 UNC93B1
ALDH3B1 ANO1 ANO1-AS1 NEU3 IL10RA PTPN6 C1S CD163 DAZAP2 BIN2
PRR13 IL23A TMEM55B SLC7A7 LTB4R NPC2 FLVCR2 GALC FAM98B UBR1
UNC13C FAM96A HEXA ADPGK SLC5A11 APOBR ZDHHC7 CYBA ARRB2 SCIMP CD68
PIK3R5 PMP22 MPRIP ABHD15 SSH2 RNF135 EVI2B EVI2A FMNL1 RP13
MPPE1 CABYR TCF4 STXBP2 NOTCH3 IFI30 TYROBP ZNF527 PSG5 PLAUR
C5AR1 CA11 SIGLEC9 SIGLEC7 OSCAR LILRB2 SIRPG SIRPB1 HCK C20orf111
TTPAL PLTP DNAJC28 ARVCF HPS4 GPR143 TLR8 PIGA WAS VSIG4 RP1
SASH3 RAP2C

GOBPID	Pvalue	OddsRatio	ExpCount	Count	Size	Term
GO:0050778	1.46E-11	7.708769994	3.324891011	21	364	positive regulation of immune response
GO:0002252	4.38E-11	7.232728619	3.525845962	21	386	immune effector process

GO:0002443	8.56E-11	9.990450928	1.927340668	16	211
leukocyte mediated immunity					
GO:0002253	1.87E-09	7.358279371	2.731160473	17	299
activation of immune response					
GO:0002250	3.44E-09	9.114314665	1.808594561	14	198
adaptive immune response					

GO:0002449	4.02E-09	10.04829204	1.525430766	13	167	lymphocyte mediated immunity
GO:0002697	4.18E-09	8.966192332	1.835997509	14	201	regulation of immune effector process
GO:0002460	9.78E-08	8.372781065	1.65331119	12	181	adaptive immune response based on somatic recombination of immune receptors built from immunoglobulin superfamily domains
GO:0002703	1.54E-07	12.55010285	0.840357069	9	92	regulation of leukocyte mediated immunity

GO:0046479 8.09E-07 89.4625 0.082208844 4 9
glycosphingolipid catabolic process

GO:0006956 1.63E-06 14.263 0.575461906 7 63 complement
activation

GO:0002283 2.09E-06 63.89285714 0.100477476 4 11
neutrophil activation involved in immune response

GO:0002764 2.83E-06 5.967372881 2.265310359 12 248
immune response-regulating signaling pathway

GO:0006689	2.96E-06	332.9767442	0.036537264	3	4
ganglioside catabolic process					
GO:0019377	3.11E-06	55.90234375	0.109611792	4	12
glycolipid catabolic process					
GO:0006958	5.92E-06	15.4491342	0.456715798	6	50
"complement activation, classical pathway"					

GO:0016064	7.86E-06	8.904478547	1.013909072	8	111	
immunoglobulin mediated immune response						
GO:0019724	9.57E-06	8.650639075	1.04131202	8	114	B
cell mediated immunity						
GO:0072376	9.73E-06	10.63552	0.749013909	7	82	
protein activation cascade						
GO:0043304	1.11E-05	37.2578125	0.146149055	4	16	
regulation of mast cell degranulation						

GO:0042098	1.23E-05	8.33372434	1.077849284	8	118	T cell proliferation
GO:0033006	1.44E-05	34.38942308	0.155283371	4	17	regulation of mast cell activation involved in immune response
GO:0002263	1.67E-05	7.96858345	1.123520864	8	123	cell activation involved in immune response
GO:0002366	1.67E-05	7.96858345	1.123520864	8	123	leukocyte activation involved in immune response

GO:0031347 1.89E-05 4.248616618 3.681129334 14 403
regulation of defense response

Tissue: Adipose_Subcutaneous=>Heart_Left_Ventricle Type: asymmetric

SourceGene: HIRA(ENSG00000100084.9) histone cell cycle regulator

TargetGeneSet: TTLL10 C1QA C1QB E2F2 CLSPN CDCA8 TMEM125 CDC20
FAM72D PRPF3 GPR161 NEK2 C1orf148 FTH1P2 NCAPH CKAP2L PSD4
RABL2A MARCO HJURP OXTR KIF15 CAMK2N2 RP11 SLC39A8 CENPE BRCC3
CCNB1 HMMR RMND5B DHX16 SLC25A27 FKBP1C RNASET2 RPL23P8 CHN2 ANLN
MELK ZNF658B SAPCD2 CDK1 C10orf116 AGAP11 CEP55 PIK3AP1 MKI67
RIC8A EHF MPP2 EEF1A1P33 ESPL1 CSRP2 EP400NL DLGAP5 XRCC3
SIVA1 BUB1B NUSAP1 CCNB2 E4F1 FLYWCH1 CLDN9 ANKS3 CTRB2 AURKB
TOP2A KIF18B BRIP1 BIRC5 TYMS NDC80 ZNF562 ZNF653 ASF1B LRRC25 TPX2
PI3 UBE2C CTSZ RP1 APOBEC3B CTA TMEM27 KIF4A RP13
FAM199X

GOBPID Pvalue OddsRatio ExpCount Count Size Term

GO:0000280	2.14E-19	20.49376988	1.646944848	22	350	
nuclear division						
GO:0007067	2.14E-19	20.49376988	1.646944848	22	350	
mitosis						
GO:0000087	3.92E-19	19.8732956	1.694000415	22	360	M
phase of mitotic cell cycle						
GO:0048285	7.07E-19	19.28848076	1.741055982	22	370	
organelle fission						
GO:0051301	7.66E-16	14.46374093	2.126911632	21	452	cell
division						
GO:0010564	4.01E-13	12.71545455	1.948100477	18	414	
regulation of cell cycle process						

GO:0007059	1.94E-12	23.86439732	0.658777939	12	140
chromosome segregation					
GO:0000819	2.05E-12	50.87110761	0.244688949	9	52
sister chromatid segregation					
GO:0007346	7.69E-12	13.85121855	1.425783683	15	303
regulation of mitotic cell cycle					
GO:0007017	2.67E-11	11.4311365	1.849283787	16	393
microtubule-based process					
GO:0000070	6.08E-11	46.64065041	0.230572279	8	49
mitotic sister chromatid segregation					
GO:0007051	3.49E-09	26.50185185	0.376444537	8	80
spindle organization					

GO:0000236	5.68E-09	24.77229437	0.39997232	8	85	
mitotic prometaphase						
GO:0000086	2.25E-08	16.09943503	0.677600166	9	144	G2/M
transition of mitotic cell cycle						
GO:0000226	4.54E-08	10.56541548	1.265794755	11	269	
microtubule cytoskeleton organization						
GO:0007088	1.75E-07	20.26189031	0.41408899	7	88	
regulation of mitosis						
GO:0051783	1.75E-07	20.26189031	0.41408899	7	88	
regulation of nuclear division						
GO:0030261	2.58E-07	45.58095238	0.141166701	5	30	
chromosome condensation						
GO:0071156	4.00E-07	9.59068694	1.242266971	10	264	
regulation of cell cycle arrest						

GO:0051656	4.52E-07	17.44384374	0.475261228	7	101	establishment of organelle localization
GO:0008608	1.02E-06	69.08653846	0.079994464	4	17	attachment of spindle microtubules to kinetochore
GO:0007050	1.08E-06	7.560276389	1.736350426	11	369	cell cycle arrest
GO:0051329	1.40E-06	7.349590008	1.783405993	11	379	interphase of mitotic cell cycle
GO:0031577	1.48E-06	30.77220077	0.197633382	5	42	spindle checkpoint
GO:0051313	1.65E-06	59.86666667	0.089405577	4	19	attachment of spindle microtubules to chromosome

GO:0030071	1.67E-06	29.96031746	0.202338938	5	43	regulation of mitotic metaphase/anaphase transition
GO:0051325	1.72E-06	7.189109742	1.821050446	11	387	interphase
GO:0000075	1.86E-06	9.263839383	1.138744724	9	242	cell cycle checkpoint
GO:0034501	1.97E-06	221.2307692	0.02823334	3	6	protein localization to kinetochore
GO:0007091	2.10E-06	28.45833333	0.211750052	5	45	mitotic metaphase/anaphase transition
GO:0031145	2.46E-06	17.97989108	0.390561207	6	83	anaphase-promoting complex-dependent proteasomal ubiquitin-dependent protein catabolic process

GO:0050000	2.53E-06	52.81617647	0.098816691	4	21
chromosome localization					
GO:0051303	2.53E-06	52.81617647	0.098816691	4	21
establishment of chromosome localization					
GO:0007093	3.89E-06	12.38909588	0.654072383	7	139
mitotic cell cycle checkpoint					
GO:0051983	4.44E-06	44.884375	0.112933361	4	24
regulation of chromosome segregation					
GO:0000910	5.10E-06	15.72030792	0.442322331	6	94
cytokinesis					
GO:0045786	5.53E-06	6.322666996	2.056328282	11	437
negative regulation of cell cycle					

GO:0051640 6.44E-06 11.42726126 0.705833506 7 150 organelle localization

GO:0006323 7.34E-06 11.19009656 0.719950176 7 153 DNA packaging

GO:0045787 8.19E-06 14.40221774 0.479966784 6 102 positive regulation of cell cycle

GO:0007052 1.66E-05 30.93534483 0.155283371 4 33 mitotic spindle organization

Tissue: Adipose_Subcutaneous=>Heart_Left_Ventricle Type: asymmetric

SourceGene: HSPD1P16(ENSG00000218792.2) HSPD1P16 heat shock 60kDa protein 1 (chaperonin) pseudogene 16

TargetGeneSet: ARHGEF19 CAPZB RAB42 MPL RP11 MCOLN2 GORAB METTL13 TRMT1L IPO9-AS1 ARL8A ARHGEF33 RPL37A TMIE CTD PPP1R2P3 C6orf195 C8orf59 RPL30 RPL8 NOL8 RPLP2 RPL27A ZNF408 MEN1 RPS25 ANKRD13A MPHOSPH9 UFM1 ARHGEF40 RPL36A NDUFB1 CCDC78 FBXL19-AS1 RPL17 RPL19 HEXIM2 RPL38 SIRT7 CDC37 ATP13A1 IGSF23 LZTR1

GOBPID Pvalue OddsRatio ExpCount Count Size Term

GO:0006415	2.59E-13	68.41622481	0.201508546	9	91	translational termination
GO:0043624	6.95E-13	45.37825811	0.338800083	10	153	cellular protein complex disassembly
GO:0043241	9.61E-13	43.82985258	0.349871981	10	158	protein complex disassembly
GO:0006414	9.72E-13	58.38179348	0.232509861	9	105	translational elongation
GO:0006614	9.72E-13	58.38179348	0.232509861	9	105	SRP-dependent cotranslational protein targeting to membrane
GO:0006613	1.06E-12	57.77588525	0.234724241	9	106	cotranslational protein targeting to membrane
GO:0045047	1.06E-12	57.77588525	0.234724241	9	106	protein targeting to ER
GO:0072599	1.06E-12	57.77588525	0.234724241	9	106	establishment of protein localization to endoplasmic reticulum
GO:0034623	2.53E-12	39.5094235	0.385302055	10	174	cellular macromolecular complex disassembly
GO:0070972	2.84E-12	51.37215796	0.261296796	9	118	protein localization to endoplasmic reticulum

GO:0000184	3.07E-12	50.90158103	0.263511176	9	119	
"nuclear-transcribed mRNA catabolic process, nonsense-mediated decay"						
GO:0032984	3.37E-12	38.32705756	0.396373953	10	179	
macromolecular complex disassembly						
GO:0019080	2.67E-11	39.34262094	0.334371324	9	151	
viral genome expression						
GO:0019083	2.67E-11	39.34262094	0.334371324	9	151	
viral transcription						
GO:0006413	2.83E-11	39.06476133	0.336585703	9	152	
translational initiation						
GO:0006612	3.01E-11	38.79076087	0.338800083	9	153	
protein targeting to membrane						
GO:0000956	7.36E-11	34.87255435	0.374230157	9	169	
nuclear-transcribed mRNA catabolic process						
GO:0006402	1.17E-10	32.99459738	0.394159574	9	178	mRNA
catabolic process						
GO:0071845	3.23E-10	23.46549436	0.628883814	10	284	
cellular component disassembly at cellular level						
GO:0022411	3.71E-10	23.12132112	0.637741333	10	288	
cellular component disassembly						

GO:0072594	3.95E-10	28.54314381	0.451733444	9	204
establishment of protein localization to organelle

GO:0006401	4.13E-10	28.39551908	0.453947824	9	205	RNA
catabolic process

GO:0019058	1.19E-09	25.02408931	0.511521694	9	231
viral infectious cycle

GO:0022415	3.36E-09	22.0876494	0.575738703	9	260
viral reproductive process

GO:0006605	4.42E-08	13.67065047	1.049615944	10	474
protein targeting

GO:0016032	3.84E-07	12.37389337	0.998685212	9	451
viral reproduction

GO:0033365	5.73E-07	11.76864693	1.047401564	9	473
protein localization to organelle

Tissue: Heart_Left_Ventricle=>Adipose_Subcutaneous	Type:	asymmetric

SourceGene:	IFIT1(ENSG00000185745.8)	interferon-induced protein with tetratricopeptide repeats 1

TargetGeneSet: EIF1AX RCN3 SLC30A2 HMGN2 FGR CDC20 STIL RP11 MCL1 FCRLA RASAL2 KIF14 PPP2R5A ANGEL2 CENPF ODC1 RHOB XPO1 SH3RF3 LINC00116 BUB1 CCNT2 SGOL2 COPS8 SGOL1 LZTFL1 FAM116A PHLDB2 BOC BZW1P2 CCNL1 CCRL1 GUF1 CORIN PLK4 HMGB2 POLK GPR150 FSTL4 KIF20A PCYOX1L HMMR TRIM41 C6orf62 FKBP5 KCTD20 UBR2 SLC29A1 ANKRD6 ZNF259P1 MAN1A1 STEAP1B ANLN LAMB1 KCND2 FAM40B CDCA2 PBK TMEM67 MPDZ MELK KLF9 KLF4 GBGT1 SYT15 CEP55 PHBP9 LZTS2 ZNF215 KIF18A CAT RPLP0P2 PELI3 AASDHPPT MPP2 GRIN2B TMEM106C TROAP DNAJC14 TMEM198B SDSL CAMKK2 GTF2H3 DIAPH3 DLGAP5 RHOJ FCF1 OIP5 NUSAP1 ATP8B4 LARP6 PAQR4 HMOX2 PLK1 SHCBP1 ORC6 CDT1 ZBTB4 SPAG5 PNMT VAT1 MPP3 KIF18B H3F3B BIRC5 TYMS RNF125 SLC25A23 KANK2 ZNF441 FKBP8 CEACAM4 ZNF665 TPX2 SAMHD1 UBE2C TIAM1 PKNOX1 CDC45 ZNF74 SAMM50 ARHGAP6 FIGF

GOBPID	Pvalue	OddsRatio	ExpCount	Count	Size	Term
GO:0000087	5.68E-16	11.95300445	2.565912394	23	360	M phase of mitotic cell cycle
GO:0000280	3.75E-15	11.60945498	2.494637049	22	350	nuclear division
GO:0007067	3.75E-15	11.60945498	2.494637049	22	350	mitosis

GO:0048285	1.18E-14	10.92663545	2.637187738	22	370	organelle fission
GO:0051301	7.49E-14	9.328001166	3.221645561	23	452	cell division
GO:0010564	6.88E-10	7.460962567	2.950799253	18	414	regulation of cell cycle process
GO:0000236	8.75E-09	17.97984323	0.605840426	9	85	mitotic prometaphase
GO:0007059	5.78E-08	11.76013234	0.99785482	10	140	

chromosome segregation

GO:0007346	2.31E-07	7.002068966	2.159642931	13	303

regulation of mitotic cell cycle

GO:0030071	5.57E-07	23.92477013	0.30648398	6	43

regulation of mitotic metaphase/anaphase transition

GO:0007091	7.36E-07	22.69468676	0.320739049	6	45

mitotic metaphase/anaphase transition

GO:0071156	2.78E-06	6.661153119	1.881669089	11	264

regulation of cell cycle arrest

GO:0007088	3.01E-06	12.8432356	0.62722303	7	88
regulation of mitosis					
GO:0051783	3.01E-06	12.8432356	0.62722303	7	88
regulation of nuclear division					
GO:0007017	4.26E-06	5.309473684	2.80112103	13	393
microtubule-based process					
GO:0000910	4.69E-06	11.95246648	0.669988236	7	94
cytokinesis					
GO:0010948	5.39E-06	11.68223315	0.684243305	7	96
negative regulation of cell cycle process					

GO:0000086	8.57E-06	8.8	1.026364957	8	144	G2/M transition of mitotic cell cycle
GO:0000075	8.69E-06	6.542454579	1.724863331	10	242	cell cycle checkpoint
GO:0031577	1.15E-05	19.73386652	0.299356446	5	42	spindle checkpoint
GO:0051488	1.21E-05	107.58	0.049892741	3	7	activation of anaphase-promoting complex activity
GO:0050000	1.33E-05	34.06060606	0.149678223	4	21	chromosome localization

GO:0051303	1.33E-05	34.06060606	0.149678223	4	21

establishment of chromosome localization

GO:0051329	1.59E-05	5.023564991	2.701335548	12	379

interphase of mitotic cell cycle

GO:0051325	1.95E-05	4.913582418	2.758355823	12	387

interphase

Tissue: Heart_Left_Ventricle=>Adipose_Subcutaneous Type: asymmetric

SourceGene: LAD1(ENSG00000159166.9) ladinin 1

TargetGeneSet: CDCA8 PGCP1 ASPM KIF14 CENPF RAB3GAP2 TCEB1P21
BUB1 IHH PASK KIF15 RP11 TACC3 CENPE FBXO4 CTA C9orf174
ZWINT CEP55 HELLS MKI67 CDCA5 CDCA3 RNASE13 CBLN3 BUB1B KIAA0101
WDR73 PKMYT1 SHCBP1 ORC6 FAM64A AURKB TOP2A KIF18B PCTP NDC80
UHRF1 ZNF490 DHPS STX10 PARP4P3 CLTCL1 CDC45 GTSE1 MBTPS2 FAM122C MT

GOBPID	Pvalue	OddsRatio	ExpCount	Count	Size	Term
GO:0000280	2.36E-18	35.94249249	0.896131756	17	350	nuclear division
GO:0007067	2.36E-18	35.94249249	0.896131756	17	350	mitosis
GO:0000087	3.81E-18	34.86982507	0.92173552	17	360	M phase of mitotic cell cycle
GO:0048285	6.05E-18	33.85793201	0.947339284	17	370	organelle fission
GO:0051301	1.06E-13	21.80726025	1.157290153	15	452	cell division
GO:0000236	3.44E-11	51.36408419	0.217631998	8	85	mitotic prometaphase
GO:0007059	1.93E-09	29.84743992	0.358452702	8	140	chromosome segregation
GO:0010564	4.09E-09	14.70900935	1.059995848	11	414	regulation of cell cycle process

GO:0007017	3.57E-08	13.56832028	1.006227943	10	393
microtubule-based process

GO:0000075	1.40E-07	16.71676982	0.6196111	8	242	cell
cycle checkpoint

GO:0000819	1.98E-07	47.76263298	0.133139575	5	52
sister chromatid segregation

GO:0071156	2.72E-07	15.25646552	0.675939381	8	264
regulation of cell cycle arrest

GO:0007346	7.75E-07	13.20303916	0.775794063	8	303
regulation of mitotic cell cycle

GO:0007093	1.29E-06	20.78243997	0.355892326	6	139
mitotic cell cycle checkpoint

GO:0007051	1.74E-06	29.87291667	0.204830116	5	80
spindle organization

GO:0051640	2.02E-06	19.18010753	0.384056467	6	150
organelle localization

GO:0007050	3.38E-06	10.73875251	0.944778908	8	369	cell
cycle arrest

GO:0034508 5.52E-06 115.5320856 0.03584527 3 14
centromere complex assembly

GO:0000070 7.10E-06 38.7043771 0.125458446 4 49
mitotic sister chromatid segregation

GO:0051310 1.03E-05 90.75630252 0.0435264 3 17
metaphase plate congression

GO:0045786 1.17E-05 8.992846234 1.118884506 8 437
negative regulation of cell cycle

GO:0050000 1.99E-05 70.56862745 0.053767905 3 21
chromosome localization

GO:0051303 1.99E-05 70.56862745 0.053767905 3 21
establishment of chromosome localization

Tissue: Heart_Left_Ventricle=>Adipose_Subcutaneous Type: asymmetric

SourceGene: NT5C2(ENSG00000076685.13) "5'-nucleotidase, cytosolic II"

TargetGeneSet: PUSL1 MIB2 RP11 PGCP1 SLAMF8 DUSP27 NLRC4 FBXO48
TCEB1P21 RHOQP2 NEB XIRP2 KBTBD10 TTN MYL1 TMEM169 CMYA5
TMEM232 LMOD2 MYOM2 ENDOG TMEM141 FAS NRAP C11orf73 DDN
LINC00464 BUB1B RBFOX1 ATP2A1 MYLPF MYH2 MYH1 CCR10 PSMA8 RYR1
CKM SNORD33 MYBPC2 EEF1A2 PI4KAP2 SMPX HMGN1P26

GOBPID	Pvalue	OddsRatio	ExpCount	Count	Size	Term
GO:0006936	1.28E-11	34.43474042	0.46308214	10	239	muscle contraction
GO:0003012	3.70E-11	30.74435764	0.515396858	10	266	muscle system process
GO:0030049	1.03E-08	92.00127877	0.075565705	5	39	muscle filament sliding
GO:0033275	1.03E-08	92.00127877	0.075565705	5	39	actin-myosin filament sliding
GO:0070252	1.52E-08	84.52408931	0.081378451	5	42	actin-mediated cell contraction
GO:0030048	8.73E-08	57.84621578	0.114317348	5	59	actin filament-based movement
GO:0006941	2.95E-07	44.57453416	0.145318663	5	75	striated muscle contraction

Tissue: Adipose_Subcutaneous=>Heart_Left_Ventricle Type: asymmetric
SourceGene: RP11-439A17.9(ENSG00000233029.2)
TargetGeneSet: AURKAIP1 SPEN ARHGEF19 MINOS1 RP11 ZSCAN20 MRPS15 FAM151A RPL5 FAM212B LYSMD1 SNRPE SRGAP3 ZNF513 NFU1 COX5B TSPO SF3B3 EEF1B2 CAPN7 UBE2E1 RPL18AP7 GORASP1 LIMD1 ZNF717 YEATS2 MRAP CNO NDUFC1 COX7C ARAP3 NSD1 TMEM14B CCNC HEBP2 AGPAT4 MRPS24 SRI TECPR1 ZKSCAN5 FIS1 TNPO3 C7orf73 PRKDC RPS20 C8orf59 RPL30 COX6C TMEM65 SPATC1 RPL8 C9orf46 RPS6 ALDH1A1 POLR3A RPL27A NDUFS3 FAU RPS25 PFDN5 C12orf51 GCN1L1 GTF3A MED4 MRPL52 U2 SLIRP MGA TPM1 RPL4 MYO9A NDUFAB1 FAM65A 7SK WDR81 METTL16 PSMB6 GABARAP CTD RPL26 TMEM220 TRIM16 RPS7P1 RPL23A CCL2 RPL19 DCAF7 NDUFV2 TXNL4A ATP5D OAZ1 C19orf70 CDC37 C19orf53 NDUFB7 BRD4 MAST3 COX6B1 RPS11 ZNF808 ZNF341 PLCG1 NCOA3 APOL2 TNRC6B PPARA PNPLA4 EEF1B2P3 FUNDC1 COX7B BCORL1

GOBPID	Pvalue	OddsRatio	ExpCount	Count	Size	Term

GO:0006414 3.69274637378678e-15 28.1420118343195 0.668465850114179
14 105 translational elongation
GO:0006415 1.50203658113874e-14 30.1286919831224 0.579337070098955
13 91 translational termination
GO:0006614 1.01827296410455e-13 25.518849752339 0.668465850114179 13
105 SRP-dependent cotranslational protein targeting to membrane
GO:0006613 1.15469488928884e-13 25.2426840887437 0.674832191543838
13 106 cotranslational protein targeting to membrane
GO:0045047 1.15469488928884e-13 25.2426840887437 0.674832191543838
13 106 protein targeting to ER
GO:0072599 1.15469488928884e-13 25.2426840887437 0.674832191543838
13 106 establishment of protein localization to endoplasmic reticulum
GO:0070972 4.74744411074256e-13 22.3389993972272 0.751228288699744
13 118 protein localization to endoplasmic reticulum
GO:0000184 5.30249188814586e-13 22.1267016957249 0.757594630129403
13 119 nuclear-transcribed mRNA catabolic process, nonsense-mediated decay
GO:0019080 1.15437991848963e-11 16.9577141808842 0.961317555878486
13 151 viral genome expression
GO:0019083 1.15437991848963e-11 16.9577141808842 0.961317555878486
13 151 viral transcription
GO:0006413 1.25589680093365e-11 16.8345323741007 0.967683897308145
13 152 translational initiation
GO:0006612 1.36550618939728e-11 16.7131103074141 0.974050238737804
13 153 protein targeting to membrane
GO:0043624 1.36550618939728e-11 16.7131103074141 0.974050238737804
13 153 cellular protein complex disassembly
GO:0043241 2.05652439826081e-11 16.1311217808817 1.0058819458861 13
158 protein complex disassembly
GO:0022904 2.13412240890585e-11 22.8052287581699 0.611168777247249
11 96 respiratory electron transport chain
GO:0072594 3.82294072612714e-11 13.385020242915 1.2987336516504 14 204
establishment of protein localization to organelle
GO:0000956 4.82346741374753e-11 14.9820675105485 1.07591170161235
13 169 nuclear-transcribed mRNA catabolic process
GO:0034623 6.96470222143446e-11 14.5116754461829 1.10774340876064
13 174 cellular macromolecular complex disassembly
GO:0006402 9.26646282702262e-11 14.1558879938627 1.13320877447927
13 178 mRNA catabolic process
GO:0032984 9.94107155620554e-11 14.0696202531646 1.13957511590893
13 179 macromolecular complex disassembly
GO:0019058 2.00447753500612e-10 11.697270471464 1.47062487025119 14
231 viral infectious cycle
GO:0006401 5.37465029970302e-10 12.1420754219409 1.30509999308006
13 205 RNA catabolic process
GO:0022900 8.17322226875273e-10 15.7177556960755 0.853089751574286
11 134 electron transport chain
GO:0022415 9.47557506538276e-10 10.2971648947259 1.6552487717113 14
260 viral reproductive process
GO:0045333 1.52063803221109e-09 14.7495994722458 0.904020483011556
11 142 cellular respiration
GO:0016032 3.06268020579448e-09 7.2726574500768 2.87121998477614 17
451 viral reproduction
GO:0006605 6.45100649366348e-09 6.89522975929978 3.01764583765829
17 474 protein targeting
GO:0071845 2.76755982344779e-08 8.55453314026811 1.80804096602311
13 284 cellular component disassembly at cellular level
GO:0022411 3.26461497070382e-08 8.42771001150748 1.83350633174175
13 288 cellular component disassembly
GO:0033365 2.75047658881905e-07 5.91263539953496 3.01127949622863
15 473 protein localization to organelle
GO:0006119 1.7511410088196e-06 19.1954382826476 0.369247802920213
6 58 oxidative phosphorylation
GO:0006120 3.02300201070875e-06 26.5628476084538 0.229188291467718

5	36	mitochondrial electron transport, NADH to ubiquinone
GO:0015980	3.87136211202597e-06	6.45199366032699	1.95446681890527
11	307	energy derivation by oxidation of organic compounds
GO:0042773	1.16400533956989e-05	19.5908593322386	0.299218047193966
5	47	ATP synthesis coupled electron transport
GO:0042775	1.16400533956989e-05	19.5908593322386	0.299218047193966
5	47	mitochondrial ATP synthesis coupled electron transport
GO:0006091	1.66141261505467e-05	5.02752403846154	2.72479413189399
12	428	generation of precursor metabolites and energy
Tissue: Adipose_Subcutaneous=>Heart_Left_Ventricle	Type:	cluster
SourceGene:	RP11-439A17.9(ENSG00000233029.2)
TargetGeneSet:	AURKAIP1	SPEN	ARHGEF19	MINOS1	RP11	ZSCAN20
MRPS15	FAM151A	RPL5	FAM212B	LYSMD1	SNRPE	SRGAP3	ZNF513	NFU1	COX5B	TSPO
SF3B3	EEF1B2	CAPN7	UBE2E1	RPL18AP7	GORASP1	LIMD1	ZNF717	YEATS2	MRAP
CNO	NDUFC1	COX7C	ARAP3	NSD1	TMEM14B	CCNC	HEBP2	AGPAT4	MRPS24	SRI
TECPR1	ZKSCAN5	FIS1	TNPO3	C7orf73	PRKDC	RPS20	C8orf59	RPL30	COX6C
TMEM65	SPATC1	RPL8	C9orf46	RPS6	ALDH1A1	POLR3A	RPL27A	NDUFS3	FAU
RPS25	PFDN5	C12orf51	GCN1L1	GTF3A	MED4	MRPL52	U2	SLIRP	MGA
TPM1	RPL4	MYO9A	NDUFAB1	FAM65A	7SK	WDR81	METTL16	PSMB6	GABARAP	CTD
RPL26	TMEM220	TRIM16	RPS7P1	RPL23A	CCL2	RPL19	DCAF7	NDUFV2	TXNL4A
ATP5D	OAZ1	C19orf70	CDC37	C19orf53	NDUFB7	BRD4	MAST3
COX6B1	RPS11	ZNF808	ZNF341	PLCG1	NCOA3	APOL2	TNRC6B	PPARA	PNPLA4
EEF1B2P3	FUNDC1	COX7B	BCORL1
GOBPID	Pvalue	OddsRatio	ExpCount	Count	Size	Term
GO:0006414	3.69274637378678e-15	28.1420118343195	0.668465850114179
14	105	translational elongation
GO:0006415	1.50203658113874e-14	30.1286919831224	0.579337070098955
13	91	translational termination
GO:0006614	1.01827296410455e-13	25.518849752339	0.668465850114179	13
105	SRP-dependent cotranslational protein targeting to membrane
GO:0006613	1.15469488928884e-13	25.2426840887437	0.674832191543838
13	106	cotranslational protein targeting to membrane
GO:0045047	1.15469488928884e-13	25.2426840887437	0.674832191543838
13	106	protein targeting to ER
GO:0072599	1.15469488928884e-13	25.2426840887437	0.674832191543838
13	106	establishment of protein localization to endoplasmic reticulum
GO:0070972	4.74744411074256e-13	22.3389993972272	0.751228288699744
13	118	protein localization to endoplasmic reticulum
GO:0000184	5.30249188814586e-13	22.1267016957249	0.757594630129403
13	119	nuclear-transcribed mRNA catabolic process, nonsense-mediated decay
GO:0019080	1.15437991848963e-11	16.9577141808842	0.961317555878486
13	151	viral genome expression
GO:0019083	1.15437991848963e-11	16.9577141808842	0.961317555878486
13	151	viral transcription
GO:0006413	1.25589680093365e-11	16.8345323741007	0.967683897308145
13	152	translational initiation
GO:0006612	1.36550618939728e-11	16.7131103074141	0.974050238737804
13	153	protein targeting to membrane
GO:0043624	1.36550618939728e-11	16.7131103074141	0.974050238737804
13	153	cellular protein complex disassembly
GO:0043241	2.05652439826081e-11	16.1311217808817	1.0058819458861	13
158	protein complex disassembly
GO:0022904	2.13412240890585e-11	22.8052287581699	0.611168777247249
11	96	respiratory electron transport chain
GO:0072594	3.82294072612714e-11	13.385020242915	1.2987336516504	14	204
establishment of protein localization to organelle
GO:0000956	4.82346741374753e-11	14.9820675105485	1.07591170161235
13	169	nuclear-transcribed mRNA catabolic process
GO:0034623	6.96470222143446e-11	14.5116754461829	1.10774340876064
13	174	cellular macromolecular complex disassembly
GO:0006402	9.26646282702262e-11	14.1558879938627	1.13320877447927
13	178	mRNA catabolic process
GO:0032984	9.94107155620554e-11	14.0696202531646	1.13957511590893

13	179	macromolecular complex disassembly
GO:0019058	2.00447753500612e-10	11.697270471464 1.47062487025119	14
231	viral infectious cycle
GO:0006401	5.37465029970302e-10	12.1420754219409	1.30509999308006
13	205	RNA catabolic process
GO:0022900	8.17322226875273e-10	15.7177556960755	0.853089751574286
11	134	electron transport chain
GO:0022415	9.47557506538276e-10	10.2971648947259	1.6552487717113 14
260	viral reproductive process
GO:0045333	1.52063803221109e-09	14.7495994722458	0.904020483011556
11	142	cellular respiration
GO:0016032	3.06268020579448e-09	7.2726574500768 2.87121998477614	17
451	viral reproduction
GO:0006605	6.45100649366348e-09	6.89522975929978	3.01764583765829
17	474	protein targeting
GO:0071845	2.76755982344779e-08	8.55453314026811	1.80804096602311
13	284	cellular component disassembly at cellular level
GO:0022411	3.26461497070382e-08	8.42771001150748	1.83350633174175
13	288	cellular component disassembly
GO:0033365	2.75047658881905e-07	5.91263539953496	3.01127949622863
15	473	protein localization to organelle
GO:0006119	1.7511410088196e-06	19.1954382826476	0.369247802920213
6	58	oxidative phosphorylation
GO:0006120	3.02300201070875e-06	26.5628476084538	0.229188291467718
5	36	mitochondrial electron transport, NADH to ubiquinone
GO:0015980	3.87136211202597e-06	6.45199366032699	1.95446681890527
11	307	energy derivation by oxidation of organic compounds
GO:0042773	1.16400533956989e-05	19.5908593322386	0.299218047193966
5	47	ATP synthesis coupled electron transport
GO:0042775	1.16400533956989e-05	19.5908593322386	0.299218047193966
5	47	mitochondrial ATP synthesis coupled electron transport
GO:0006091	1.66141261505467e-05	5.02752403846154	2.72479413189399
12	428	generation of precursor metabolites and energy
Tissue: Heart_Left_Ventricle=>Adipose_Subcutaneous	Type:	asymmetric
SourceGene:	RP11-655M14.13(ENSG00000255318.1)
TargetGeneSet:	CSMD2	RPS8	RPL5	RP11	SRGAP2P2	PSMB4	RPS7	NFU1
ANKRD36 BRK1	RPL15	RAD54L2 KCTD6	ZBTB20	GYG1	MRPS18C ADH5	SMARCA5-AS1
RPS3A	CTD	BTF3	SKP1	PCDHGA4 CTB	MSX2	CUTA	WDR11	ABRACL
RPS3AP26	BRAF	ZNF484	C10orf31	ADRA2A	RPS24	RPS3AP5 DNMBP-AS1
TMEM126B	IFT46	PHB2	MLL4	RPL6	RPSAP54 STARD13-AS1	ESD	MED4
PSMA3	SQRDL	RPS3AP6 FAM108C1	ADCY7	KARS	RPL26	STX8	RPS7P1
RPS7P11 TUBD1	PSMG2	CHMP4B
GOBPID	Pvalue	OddsRatio	ExpCount	Count	Size	Term
GO:0006415	1.45666516821016e-10	41.8459291712304	0.258182824718013
8	91	translational termination
GO:0006414	4.64438735855612e-10	35.7713214620431	0.297903259290015
8	105	translational elongation
GO:0006614	4.64438735855612e-10	35.7713214620431	0.297903259290015
8	105	SRP-dependent cotranslational protein targeting to membrane
GO:0006613	5.01328235068794e-10	35.4038342609771	0.300740433188015
8	106	cotranslational protein targeting to membrane
GO:0045047	5.01328235068794e-10	35.4038342609771	0.300740433188015
8	106	protein targeting to ER
GO:0072599	5.01328235068794e-10	35.4038342609771	0.300740433188015
8	106	establishment of protein localization to endoplasmic reticulum
GO:0070972	1.18662231122188e-09	31.5151515151515	0.334786519964016
8	118	protein localization to endoplasmic reticulum
GO:0000184	1.26955061819192e-09	31.2290472290472	0.337623693862016
8	119	nuclear-transcribed mRNA catabolic process, nonsense-mediated decay
GO:0019080	8.41804379915956e-09	24.1864801864802	0.428413258598021
8	151	viral genome expression
GO:0019083	8.41804379915956e-09	24.1864801864802	0.428413258598021
8	151	viral transcription

GO:0006413 8.86757287242486e-09 24.016835016835 0.431250432496021 8
152 translational initiation
GO:0006612 9.33771581822956e-09 23.8495297805643 0.434087606394021
8 153 protein targeting to membrane
GO:0043624 9.33771581822956e-09 23.8495297805643 0.434087606394021
8 153 cellular protein complex disassembly
GO:0043241 1.20261549553799e-08 23.0464646464646 0.448273475884022
8 158 protein complex disassembly
GO:0000956 2.03875613063529e-08 21.4552983248635 0.479482388762023
8 169 nuclear-transcribed mRNA catabolic process
GO:0034623 2.56067776833948e-08 20.8017524644031 0.493668258252024
8 174 cellular macromolecular complex disassembly
GO:0006402 3.05762649345097e-08 20.3065953654189 0.505016953844025
8 178 mRNA catabolic process
GO:0032984 3.19413302749748e-08 20.1864256601099 0.507854127742025
8 179 macromolecular complex disassembly
GO:0016032 3.28582059199147e-08 11.6416666666667 1.27956542799806
11 451 viral reproduction
GO:0072594 8.80848819809488e-08 17.5807050092764 0.578783475192028
8 204 establishment of protein localization to organelle
GO:0006401 9.14744595825104e-08 17.4902322719582 0.581620649090028
8 205 RNA catabolic process
GO:0019058 2.29018273477018e-07 15.4227476559315 0.655387170438032
8 231 viral infectious cycle
GO:0022415 5.63551274443617e-07 13.6200096200096 0.737665213480036
8 260 viral reproductive process
GO:0071845 1.0973138698979e-06 12.4145805884936 0.805757387032039
8 284 cellular component disassembly at cellular level
GO:0022411 1.21888515040308e-06 12.2337662337662 0.81710608262404
8 288 cellular component disassembly
GO:0033365 5.50598763044479e-06 8.45325969827586 1.34198325375407
9 473 protein localization to organelle
GO:0006605 5.60114799230724e-06 8.43447580645161 1.34482042765207
9 474 protein targeting
GO:0006364 1.06890887301401e-05 20.0771604938272 0.295066085392014
5 104 rRNA processing
GO:0016072 1.40496412678571e-05 18.9219576719577 0.312089128780015
5 110 rRNA metabolic process
GO:0042274 1.67934151149249e-05 75.7631578947368 0.0510691301640025
3 18 ribosomal small subunit biogenesis
GO:0034470 1.95796569622906e-05 12.5620029455081 0.567434779600028
6 200 ncRNA processing
Tissue: Adipose_Subcutaneous=>Heart_Left_Ventricle Type: asymmetric

SourceGene: ST3GAL5(ENSG00000115525.11) "ST3 beta-galactoside alpha-2,3-sialyltransferase 5"

TargetGeneSet: RP11 MRPL20 DNAJC16 EIF1AX RPRD2 ILDR2 TGFB2 LYPLAL1
CICP5 RAB4A RPS7 NBAS MRPL33 MORN2 UBC CCDC142 SNRNP200
TNFAIP6 CIDEC HIGD1A ADIPOQ LETM1 MRAP RPS3A NDUFAF2 MTND5P11
TMEM14B RPS10 PGM3 HEBP2 MAGI2-AS3 ZKSCAN5 GS1 MEPCE PNPLA8
ADCK2 RPL30 FBXO32 FAM49B EEF1D RPL8 LURAP1L RPS6 DCTN3 HABP4
TRIM32 CDK5RAP2 RPL35 GTF3C4 UPF2 DCLRE1C TMEM180 RPLP2 RPL27A FAR1
RP4 HYLS1 CACNA1C-IT3 REP15 RAP1B GLIPR1 RPL18AP3 ANKRD13A
FAM179B MAPK1IP1L VTI1B PLDN ZFAND6 C17orf50 RPL19 C17orf53
SRSF1 SRSF2 CABYR MOB3A ALKBH7 ZNF490 ZNF829 SPRED3 AURKC CSRP2BP RBL1
PCK1 SMPX DYNLT3 ACSL4 FAM127A EMD

GOBPID	Pvalue	OddsRatio	ExpCount	Count	Size	Term
GO:0006414	1.47E-14	37.64845293	0.443221922	12	105	translational elongation
GO:0000184	6.85E-14	32.6904444	0.502318179	12	119	"nuclear-transcribed mRNA catabolic process, nonsense-mediated decay"
GO:0006415	1.07E-13	39.3525	0.384125666	11	91	translational termination
GO:0006614	5.39E-13	33.4587234	0.443221922	11	105	SRP-dependent cotranslational protein targeting to membrane

GO:0006613	6.00E-13	33.10421053	0.447443084	11	106	cotranslational protein targeting to membrane
GO:0045047	6.00E-13	33.10421053	0.447443084	11	106	protein targeting to ER
GO:0072599	6.00E-13	33.10421053	0.447443084	11	106	establishment of protein localization to endoplasmic reticulum
GO:0006413	1.32E-12	24.9271137	0.641616497	12	152	translational initiation
GO:0070972	1.99E-12	29.36691589	0.498097018	11	118	protein localization to endoplasmic reticulum
GO:0000956	4.68E-12	22.20148187	0.713376237	12	169	nuclear-transcribed mRNA catabolic process
GO:0006402	8.66E-12	20.98450947	0.751366687	12	178	mRNA catabolic process

GO:0019080	3.00E-11	22.39285714	0.637395336	11	151	viral genome expression
GO:0019083	3.00E-11	22.39285714	0.637395336	11	151	viral transcription
GO:0006612	3.46E-11	22.0743662	0.645837658	11	153	protein targeting to membrane
GO:0043624	3.46E-11	22.0743662	0.645837658	11	153	cellular protein complex disassembly
GO:0072594	4.30E-11	18.10969388	0.861116878	12	204	establishment of protein localization to organelle
GO:0006401	4.56E-11	18.01459237	0.865338039	12	205	RNA catabolic process

GO:0043241	4.91E-11	21.31605442	0.666943464	11	158	protein complex disassembly
GO:0034623	1.39E-10	19.20208589	0.734482043	11	174	cellular macromolecular complex disassembly
GO:0032984	1.89E-10	18.62404762	0.755587849	11	179	macromolecular complex disassembly
GO:0019058	2.84E-09	14.17	0.975088229	11	231	viral infectious cycle
GO:0033365	7.35E-09	9.040652668	1.996609231	14	473	protein localization to organelle
GO:0006605	7.56E-09	9.020351526	2.000830392	14	474	protein targeting

GO:0022415 9.77E-09 12.49405622 1.097501903 11 260
viral reproductive process

GO:0071845 2.43E-08 11.376337 1.198809771 11 284
cellular component disassembly at cellular level

GO:0022411 2.81E-08 11.20888087 1.215694416 11 288
cellular component disassembly

GO:0016032 3.21E-07 7.782622844 1.903743686 12 451
viral reproduction

Tissue: Heart_Left_Ventricle=>Adipose_Subcutaneous Type: asymmetric
SourceGene: VDAC1P2(ENSG00000213856.3)
TargetGeneSet: RP11 TCEB1P21 NEB XIRP2 KBTBD10 TTN MYL1
KBTBD5 CMYA5 HCG16 RP1 CTB LMOD2 YWHAZP3 DYDC2 OR52E8 CSRP3 MYL2
THSD1P1 MYH7 CTD ATP2A1 MYH2 MYH1 FOXJ1 TMEM161A CKM
COX6CP2 PVALB LGALS1 RP13
GOBPID Pvalue OddsRatio ExpCount Count Size Term
GO:0006936 6.37191302025124e-12 55.5730434782609 0.314234309044357
9 239 muscle contraction
GO:0030049 6.82931318946151e-12 201.384615384615 0.0512767282541001
6 39 muscle filament sliding
GO:0033275 6.82931318946151e-12 201.384615384615 0.0512767282541001
6 39 actin-myosin filament sliding
GO:0070252 1.09550346103916e-11 184.564102564103 0.0552210919659539
6 42 actin-mediated cell contraction
GO:0003012 1.66726778779246e-11 49.6400778210117 0.349733582451041
9 266 muscle system process
GO:0030048 9.28670025034424e-11 125.216255442671 0.0775724863331257
6 59 actin filament-based movement

GO:0006941	4.09911483040142e-10	96.0735785953177	0.0986090927963463
6	75	striated muscle contraction
GO:0030029	3.38449798682131e-08	24.0274442538593	0.567988374506954
8	432	actin filament-based process
GO:0061061	5.18891587400419e-07	20.3586235489221	0.537748252716075
7	409	muscle structure development
GO:0007517	1.74331969548227e-06	21.965811965812 0.398380734897239	6
303	muscle organ development
GO:0007507	3.51644406305201e-06	19.3626373626374	0.449657463151339
6	342	heart development
GO:0003010	9.81137925112146e-06	848.823529411765	0.00525915161580513
2	4	voluntary skeletal muscle contraction
GO:0014721	9.81137925112146e-06	848.823529411765	0.00525915161580513
2	4	twitch skeletal muscle contraction
GO:0048738	1.34512757015315e-05	34.4048048048048	0.151200608954398
4	115	cardiac muscle tissue development
GO:0003015	1.3920077546574e-05	34.0952380952381	0.152515396858349
4	116	heart process
GO:0060047	1.3920077546574e-05	34.0952380952381	0.152515396858349
4	116	heart contraction
Tissue: Muscle_Skeletal=>Adipose_Subcutaneous	Type:	asymmetric

SourceGene:	ARGLU1(ENSG00000134884.8)	arginine and glutamate rich 1

TargetGeneSet: RP11 CEP104 RPL22 VPS13D RP1 UBR4 HSPG2 RPL11
ARID1A EPB41 PUM1 KPNA7 ZBTB8OS 7SK MFI2 MACF1 SZT2 PTPRF
KDM4A DPH5 HIPK1 MRPS21 ADAR ASH1L ARHGEF11 DCAF8 EDEM3
LPGAT1 URB2 ADAM18 TTC32 ASXL2 SOS1-IT1 CCDC104 USP34 AAK1
ALMS1 TET3 RETSAT TMEM131 HOXD-AS2 NBEAL1 FARP2 BRK1 NR2C2
GOLGA4 ZNF621 USP19 USP4 RNF123 VPRBP RAD54L2 APPL1 PCNP ZBTB11
KPNA1 CNBP H1FX EIF4G1 CTBP1-AS1 NOP14 DANCR WDFY3-AS1
RPL34 MAML3 NIPBL CTD TAF9 BDP1 DMXL1 RAD50 AFF4 HSPA4
CTNNA1 RPS14 SLC2A3P1 HRH2 NSD1 DHX16 DDX39B SRPK1 ZFAND3 CUL9
XPO5 TMEM63B KIAA1919 RP3 C6orf99 TNRC18 FBXL18 PLEKHA8 TRRAP CUX1
COG5 TNPO3 MLL3 POLR3D KIAA1967 KAT6A RPS20 NIPAL2 VPS13B
EIF3H EIF2C2 HEATR7A KCNV2 KIAA2026 SNAPC3 BICD2 SETX BRD3
SEC16A NOTCH1 EHMT1 HK1 SEC24C FUT11 KIAA0913 TNKS2 IDE
DNMBP-AS1 CHUK PPRC1 GBF1 EIF6 PDDC1 CARS-AS1 RPL27A
DNAJC24 TNKS1BP1 KDM2A LRP5 PPP6R3 UBE4A DDX6 SRPR ERC1
SCAF11 CCDC65 MLL4 PFDN5 ANKRD52 PPTC7 ATXN2 C12orf47 MLXIP
SBNO1 EP400 ZDHHC20 DGKH MYCBP2-AS1 TEP1 ARHGEF40 HNRNPC
C14orf21 STRN3 NIN EXOC5 EXD2 CCNK DYNC1H1 TECPR2 CDC42BPB
KIAA0284 CYFIP1 HERC2 TYRO3 MGA GANC CCNDBP1 TP53BP1 USP8
CCPG1 RPS3AP6 MYO9A SIN3A SEC11A ZNF592 POLG NME3 ZNF598 TSC2 PKD1
PDPK1 SLX4 CREBBP RPS15A SMG1 GTF3C1 SRCAP RNF40 RAB43P1 VPS35
LONP2 ADCY7 AARS ZNF778 RP13 ZZEF1 RAI1 TOM1L2 LLGL1 FLII
KIAA0100 RPL23A NUFIP2 TAOK1 RPL19 CDK12 NKIRAS2 KPNB1 CLTC
DCAF7 ERN1 CEP95 UBE2O PGS1 CSNK1D FOXK2 ZNF236 NFIC ZBTB7A
PLIN4 RANBP3 KHSRP CARM1 SMARCA4 C19orf43 BRD4 UPF1 ARHGAP35
RPS11 PRR12 CNOT3 U2AF2 ATRN RNF24 RRBP1 NCOA6 SOGA1 RALGAPB
STAU1 ADRM1 RP4 ZGPAT DNAJC5 SCAF4 SON CBR3-AS1 AGPAT3
UBE2G2 MCM3AP DSTNP1 ZNF70 C22orf45 CTA SYN3 EIF3L SMCR7L
EP300 TCF20 ZBED4 SBF1 CDKL5 UXT KDM5C HUWE1 GNL3L RRAGB
FAM123B RPS4X RBM41

GOBPID	Pvalue	OddsRatio	ExpCount	Count	Size	Term

GO:0000956 6.11E-10 7.914936525 2.52605356 17 169
nuclear-transcribed mRNA catabolic process

GO:0006402 1.37E-09 7.467711227 2.660577123 17 178 mRNA
catabolic process

GO:0000184 2.70E-09 9.326732673 1.778700436 14 119
"nuclear-transcribed mRNA catabolic process, nonsense-mediated decay"

GO:0006614	5.39E-09	9.844666952	1.569441561	13	105

SRP-dependent cotranslational protein targeting to membrane

GO:0006613	6.06E-09	9.738121723	1.584388624	13	106

cotranslational protein targeting to membrane

GO:0045047	6.06E-09	9.738121723	1.584388624	13	106

protein targeting to ER

GO:0072599	6.06E-09	9.738121723	1.584388624	13	106

establishment of protein localization to endoplasmic reticulum

GO:0006413	8.40E-09	7.679485783	2.271953498	15	152	translational initiation
GO:0006415	1.02E-08	10.54058079	1.360182686	12	91	translational termination
GO:0006401	1.18E-08	6.382951994	3.06414781	17	205	RNA catabolic process

GO:0016570	1.35E-08	5.576771406	3.901183309	19	261
histone modification					
GO:0016569	1.95E-08	5.439516129	3.990865684	19	267
covalent chromatin modification					

GO:0070972	2.26E-08	8.617874736	1.763753373	13	118
protein localization to endoplasmic reticulum					
GO:0033365	4.25E-08	4.028082928	7.069960556	25	473
protein localization to organelle					

GO:0006605	4.43E-08	4.018820182	7.084907619	25	474
protein targeting					
GO:0006612	7.00E-08	7.028420828	2.286900561	14	153
protein targeting to membrane					
GO:0072594	7.04E-08	5.977446809	3.049200747	16	204
establishment of protein localization to organelle					

GO:0034623	3.47E-07	6.096844059	2.600788873	14	174
cellular macromolecular complex disassembly					
GO:0022415	3.76E-07	4.918915565	3.886236247	17	260
viral reproductive process					

GO:0019058	3.87E-07	5.216744186	3.452771435	16	231
viral infectious cycle					
GO:0019080	4.23E-07	6.541764832	2.257006436	13	151
viral genome expression					

GO:0019083	4.23E-07	6.541764832	2.257006436	13	151
viral transcription					
GO:0006414	4.70E-07	8.072184743	1.569441561	11	105
translational elongation					
GO:0032984	4.91E-07	5.909990999	2.675524185	14	179
macromolecular complex disassembly					

GO:0016568	1.30E-06	3.744066486	6.262819182	21	419	chromatin modification
GO:0006397	2.35E-06	3.730547628	5.963877932	20	399	mRNA processing

GO:0043624	3.18E-06	5.879849812	2.286900561	12	153
cellular protein complex disassembly					
GO:0043241	4.45E-06	5.676470588	2.361635873	12	158
protein complex disassembly					

GO:0071845	5.81E-06	4.169253731	4.244965746	16	284
cellular component disassembly at cellular level

GO:0022411	6.94E-06	4.106764706	4.304753996	16	288
cellular component disassembly

Tissue: Muscle_Skeletal=>Adipose_Subcutaneous	Type:	cluster
SourceGene:	ARGLU1(ENSG00000134884.8)
TargetGeneSet:	RP11	CEP104	RPL22	VPS13D	RP1	UBR4	HSPG2	RPL11
ARID1A	EPB41	PUM1	KPNA7	ZBTB8OS	7SK	MFI2	MACF1	SZT2	PTPRF

KDM4A DPH5 HIPK1 MRPS21 ADAR ASH1L ARHGEF11 DCAF8 EDEM3
LPGAT1 URB2 ADAM18 TTC32 ASXL2 SOS1-IT1 CCDC104 USP34 AAK1
ALMS1 TET3 RETSAT TMEM131 HOXD-AS2 NBEAL1 FARP2 BRK1 NR2C2
GOLGA4 ZNF621 USP19 USP4 RNF123 VPRBP RAD54L2 APPL1 PCNP ZBTB11
KPNA1 CNBP H1FX EIF4G1 CTBP1-AS1 NOP14 DANCR WDFY3-AS1
RPL34 MAML3 NIPBL CTD TAF9 BDP1 DMXL1 RAD50 AFF4 HSPA4
CTNNA1 RPS14 SLC2A3P1 HRH2 NSD1 DHX16 DDX39B SRPK1 ZFAND3 CUL9
XPO5 TMEM63B KIAA1919 RP3 C6orf99 TNRC18 FBXL18 PLEKHA8 TRRAP CUX1
COG5 TNPO3 MLL3 POLR3D KIAA1967 KAT6A RPS20 NIPAL2 VPS13B
EIF3H EIF2C2 HEATR7A KCNV2 KIAA2026 SNAPC3 BICD2 SETX BRD3
SEC16A NOTCH1 EHMT1 HK1 SEC24C FUT11 KIAA0913 TNKS2 IDE
DNMBP-AS1 CHUK PPRC1 GBF1 EIF6 PDDC1 CARS-AS1 RPL27A
DNAJC24 TNKS1BP1 KDM2A LRP5 PPP6R3 UBE4A DDX6 SRPR ERC1
SCAF11 CCDC65 MLL4 PFDN5 ANKRD52 PPTC7 ATXN2 C12orf47 MLXIP
SBNO1 EP400 ZDHHC20 DGKH MYCBP2-AS1 TEP1 ARHGEF40 HNRNPC
C14orf21 STRN3 NIN EXOC5 EXD2 CCNK DYNC1H1 TECPR2 CDC42BPB
KIAA0284 CYFIP1 HERC2 TYRO3 MGA GANC CCNDBP1 TP53BP1 USP8
CCPG1 RPS3AP6 MYO9A SIN3A SEC11A ZNF592 POLG NME3 ZNF598 TSC2 PKD1
PDPK1 SLX4 CREBBP RPS15A SMG1 GTF3C1 SRCAP RNF40 RAB43P1 VPS35
LONP2 ADCY7 AARS ZNF778 RP13 ZZEF1 RAI1 TOM1L2 LLGL1 FLII
KIAA0100 RPL23A NUFIP2 TAOK1 RPL19 CDK12 NKIRAS2 KPNB1 CLTC
DCAF7 ERN1 CEP95 UBE2O PGS1 CSNK1D FOXK2 ZNF236 NFIC ZBTB7A
PLIN4 RANBP3 KHSRP CARM1 SMARCA4 C19orf43 BRD4 UPF1 ARHGAP35
RPS11 PRR12 CNOT3 U2AF2 ATRN RNF24 RRBP1 NCOA6 SOGA1 RALGAPB
STAU1 ADRM1 RP4 ZGPAT DNAJC5 SCAF4 SON CBR3-AS1 AGPAT3
UBE2G2 MCM3AP DSTNP1 ZNF70 C22orf45 CTA SYN3 EIF3L SMCR7L
EP300 TCF20 ZBED4 SBF1 CDKL5 UXT KDM5C HUWE1 GNL3L RRAGB
FAM123B RPS4X RBM41
GOBPID Pvalue OddsRatio ExpCount Count Size Term
GO:0000956 6.10642257567849e-10 7.91493652472891 2.52605356030725
17 169 nuclear-transcribed mRNA catabolic process
GO:0006402 1.36881264877844e-09 7.46771122694216 2.66057712269047
17 178 mRNA catabolic process
GO:0000184 2.7035754699696e-09 9.32673267326733 1.77870043595599
14 119 nuclear-transcribed mRNA catabolic process, nonsense-mediated decay
GO:0006614 5.39080792717484e-09 9.84466695223817 1.56944156113764
13 105 SRP-dependent cotranslational protein targeting to membrane
GO:0006613 6.06420928503676e-09 9.73812172254886 1.58438862362466
13 106 cotranslational protein targeting to membrane
GO:0045047 6.06420928503676e-09 9.73812172254886 1.58438862362466
13 106 protein targeting to ER
GO:0072599 6.06420928503676e-09 9.73812172254886 1.58438862362466
13 106 establishment of protein localization to endoplasmic reticulum
GO:0006413 8.40146687823775e-09 7.67948578276501 2.27195349802782
15 152 translational initiation
GO:0006415 1.02439993812341e-08 10.5405807892777 1.36018268631929
12 91 translational termination
GO:0006401 1.1782094541953e-08 6.38295199401262 3.06414780984015
17 205 RNA catabolic process
GO:0016570 1.35055073605818e-08 5.57677140579771 3.90118330911356
19 261 histone modification
GO:0016569 1.95220980776489e-08 5.43951612903226 3.99086568403571
19 267 covalent chromatin modification
GO:0070972 2.26458106688009e-08 8.61787473610134 1.76375337346896
13 118 protein localization to endoplasmic reticulum
GO:0033365 4.24816230312305e-08 4.02808292819746 7.06996055636288
25 473 protein localization to organelle
GO:0006605 4.42651014331273e-08 4.01882018213832 7.08490761884991
25 474 protein targeting
GO:0006612 6.99926641472807e-08 7.02842082769428 2.28690056051484
14 153 protein targeting to membrane
GO:0072594 7.03581564956439e-08 5.97744680851064 3.04920074735312
16 204 establishment of protein localization to organelle

GO:0034623	3.46922599961797e-07	6.09684405940594	2.60078887274237
14	174	cellular macromolecular complex disassembly
GO:0022415	3.76483983363389e-07	4.91891556548173	3.88623624662653
17	260	viral reproductive process
GO:0019058	3.87360169100841e-07	5.21674418604651	3.4527714345028 16
231	viral infectious cycle
GO:0019080	4.22788759172526e-07	6.54176483186978	2.25700643554079
13	151	viral genome expression
GO:0019083	4.22788759172526e-07	6.54176483186978	2.25700643554079
13	151	viral transcription
GO:0006414	4.69793777412849e-07	8.07218474312403	1.56944156113764
11	105	translational elongation
GO:0032984	4.90505725544315e-07	5.90999099909991	2.6755241851775 14
179	macromolecular complex disassembly
GO:0016568	1.29733854344659e-06	3.74406648627754	6.26281918206352
21	419	chromatin modification
GO:0006397	2.34560710933339e-06	3.73054762802218	5.96387793232302
20	399	mRNA processing
GO:0043624	3.18160741122279e-06	5.87984981226533	2.28690056051484
12	153	cellular protein complex disassembly
GO:0043241	4.44707132878579e-06	5.67647058823529	2.36163587294997
12	158	protein complex disassembly
GO:0071845	5.81404164814309e-06	4.16925373134328	4.24496574631513
16	284	cellular component disassembly at cellular level
GO:0022411	6.94154593913884e-06	4.10676470588235	4.30475399626323
16	288	cellular component disassembly
Tissue: Muscle_Skeletal=>Adipose_Subcutaneous	Type:	asymmetric

SourceGene:	CTD-2089O24.1(ENSG00000248966.1)	This is an expressed pseudogene. What does this mean? How high is its expression?

TargetGeneSet:	RP1	RPS8	RPL5	RP11	DDIT3	UBC	CCDC115	EEF1B2
SETD5	RPL15	RPL10AP6		AIMP1	RPS3A	CCNO	NDUFAF2	TAF9	BTF3	CTB
NPM1	RPL15P3	ABT1	RPL10A	DAAM2	TBCC	EEF1A1	HOXA-AS3		SSBP1	CTD
RPL7	PABPC1	EIF3H	RPS6	RPL12	EEF1A1P5		RPL7A	RPS24	TCTN3
MORN4	C11orf46		EIF3M	PHB2	CDK4	NAP1L1	RPL18AP3		DGKH	TPT1
WDFY2	TPPP2	RSL24D1	RPS17L	MEF2A	RSL1D1	RPL26	RPL23A	TAOK1	RPL19
PSMG2	RPL17	AKAP8L	RPL18	RPL13A	RPS9	RPS5	RPL12P4	YDJC	EIF3L
RPSAP9	RPL10

GOBPID	Pvalue	OddsRatio	ExpCount	Count	Size	Term

GO:0006414	2.57E-36	133.8487805	0.385094457	23	105
translational elongation

GO:0006413 3.18E-36 100.3304274 0.557470071 25 152
translational initiation

GO:0006415 1.38E-33 134.325 0.33374853 21 91
translational termination

GO:0000184 6.77E-33 104.6298637 0.436440385 22 119
"nuclear-transcribed mRNA catabolic process, nonsense-mediated decay"

GO:0006614 3.86E-32 111.828125 0.385094457 21 105
SRP-dependent cotranslational protein targeting to membrane

GO:0006613 4.81E-32 110.5047794 0.388762023 21 106
cotranslational protein targeting to membrane

GO:0045047 4.81E-32 110.5047794 0.388762023 21 106
protein targeting to ER

GO:0072599 4.81E-32 110.5047794 0.388762023 21 106
establishment of protein localization to endoplasmic reticulum

GO:0070972	5.61E-31	96.75289948	0.432772818	21	118	protein localization to endoplasmic reticulum
GO:0000956	2.60E-29	68.80008778	0.619818698	22	169	nuclear-transcribed mRNA catabolic process
GO:0006402	8.62E-29	64.78990902	0.652826794	22	178	mRNA catabolic process
GO:0019080	1.43E-28	72.02596154	0.553802505	21	151	viral genome expression
GO:0019083	1.43E-28	72.02596154	0.553802505	21	151	viral transcription
GO:0006612	1.92E-28	70.92471591	0.561137638	21	153	protein targeting to membrane
GO:0043624	1.92E-28	70.92471591	0.561137638	21	153	cellular protein complex disassembly
GO:0043241	3.91E-28	68.3122719	0.579475469	21	158	protein complex disassembly

GO:0006401	2.19E-27	55.12603561	0.751851083	22	205	RNA catabolic process
GO:0034623	3.27E-27	61.09987745	0.638156529	21	174	cellular macromolecular complex disassembly
GO:0032984	6.09E-27	59.14556962	0.65649436	21	179	macromolecular complex disassembly
GO:0072594	1.05E-25	50.97592213	0.748183517	21	204	establishment of protein localization to organelle
GO:0019058	1.53E-24	44.3375	0.847207806	21	231	viral infectious cycle
GO:0022415	1.92E-23	38.87800732	0.953567227	21	260	viral reproductive process
GO:0071845	1.25E-22	35.27031844	1.041588817	21	284	cellular component disassembly at cellular level

GO:0022411	1.68E-22	34.7320927	1.056259082	21	288	
cellular component disassembly						
GO:0016032	8.54E-20	23.10835401	1.654072383	22	451	
viral reproduction						
GO:0033365	5.07E-18	20.24792588	1.73475884	21	473	
protein localization to organelle						
GO:0006605	5.30E-18	20.2017798	1.738426406	21	474	
protein targeting						
GO:0042254	1.34E-10	23.85644972	0.546467373	10	149	
ribosome biogenesis						
GO:0022613	4.38E-10	17.35914553	0.825202408	11	225	
ribonucleoprotein complex biogenesis						
GO:0071843	8.32E-10	16.27715121	0.876548336	11	239	
cellular component biogenesis at cellular level						
GO:0006364	9.58E-08	22.43545495	0.381426891	7	104	rRNA

processing

GO:0016072 1.41E-07 21.11967075 0.403432288 7 110 rRNA metabolic process

GO:0042274 4.75E-07 83.87172012 0.066016193 4 18 ribosomal small subunit biogenesis

GO:0034470 7.87E-06 11.20015769 0.733513252 7 200 ncRNA processing

GO:0034660 8.51E-06 9.062815884 1.045256384 8 285 ncRNA metabolic process

GO:0042273 1.00E-05 95.92666667 0.044010795 3 12 ribosomal large subunit biogenesis

Tissue: Muscle_Skeletal=>Adipose_Subcutaneous Type: cluster
SourceGene: CTD-2089O24.1(ENSG00000248966.1)
TargetGeneSet: RP1 RPS8 RPL5 RP11 DDIT3 UBC CCDC115 EEF1B2 SETD5 RPL15 RPL10AP6 AIMP1 RPS3A CCNO NDUFAF2 TAF9 BTF3 CTB NPM1 RPL15P3 ABT1 RPL10A DAAM2 TBCC EEF1A1 HOXA-AS3 SSBP1 CTD RPL7 PABPC1 EIF3H RPS6 RPL12 EEF1A1P5 RPL7A RPS24 TCTN3 MORN4 C11orf46 EIF3M PHB2 CDK4 NAP1L1 RPL18AP3 DGKH TPT1 WDFY2 TPPP2 RSL24D1 RPS17L MEF2A RSL1D1 RPL26 RPL23A TAOK1 RPL19 PSMG2 RPL17 AKAP8L RPL18 RPL13A RPS9 RPS5 RPL12P4 YDJC EIF3L RPSAP9 RPL10
GOBPID Pvalue OddsRatio ExpCount Count Size Term
GO:0006414 2.57316855367557e-36 133.848780487805 0.385094457130994 23 105 translational elongation
GO:0006413 3.18438255427382e-36 100.330427446569 0.557470071275344

25	152	translational initiation
GO:0006415	1.37683091113982e-33	134.325 0.333748529513528	21	91
translational termination
GO:0000184	6.76824231087336e-33	104.62986365148 0.43644038474846	22
119	nuclear-transcribed mRNA catabolic process, nonsense-mediated decay
GO:0006614	3.86335117243005e-32	111.828125	0.385094457130994	21
105	SRP-dependent cotranslational protein targeting to membrane
GO:0006613	4.80757453431886e-32	110.504779411765	0.388762023389385
21	106	cotranslational protein targeting to membrane
GO:0045047	4.80757453431886e-32	110.504779411765	0.388762023389385
21	106	protein targeting to ER
GO:0072599	4.80757453431886e-32	110.504779411765	0.388762023389385
21	106	establishment of protein localization to endoplasmic reticulum
GO:0070972	5.60861412618874e-31	96.7528994845361	0.43277281849007
21	118	protein localization to endoplasmic reticulum
GO:0000956	2.60238859680657e-29	68.8000877770463	0.619818697667981
22	169	nuclear-transcribed mRNA catabolic process
GO:0006402	8.6246094938347e-29	64.7899090157155	0.652826793993495
22	178	mRNA catabolic process
GO:0019080	1.43194412988401e-28	72.0259615384615	0.553802505016954
21	151	viral genome expression
GO:0019083	1.43194412988401e-28	72.0259615384615	0.553802505016954
21	151	viral transcription
GO:0006612	1.91761007854905e-28	70.9247159090909	0.561137637533735
21	153	protein targeting to membrane
GO:0043624	1.91761007854905e-28	70.9247159090909	0.561137637533735
21	153	cellular protein complex disassembly
GO:0043241	3.90934916237724e-28	68.3122718978102	0.579475468825687
21	158	protein complex disassembly
GO:0006401	2.19300724539094e-27	55.1260356072625	0.751851082970037
22	205	RNA catabolic process
GO:0034623	3.27259657214294e-27	61.0998774509804	0.638156528959934
21	174	cellular macromolecular complex disassembly
GO:0032984	6.08849197704669e-27	59.1455696202532	0.656494360251886
21	179	macromolecular complex disassembly
GO:0072594	1.04927189093189e-25	50.9759221311475	0.748183516711646
21	204	establishment of protein localization to organelle
GO:0019058	1.53164327185711e-24	44.3375 0.847207805688188	21	231
viral infectious cycle
GO:0022415	1.920198072866e-23	38.8780073221757	0.95356722718151
21	260	viral reproductive process
GO:0071845	1.25135930369499e-22	35.2703184410646	1.04158881738288
21	284	cellular component disassembly at cellular level
GO:0022411	1.68221806922201e-22	34.7320926966292	1.05625908241644
21	288	cellular component disassembly
GO:0016032	8.54399763582425e-20	23.1083540115798	1.65407238253408
22	451	viral reproduction
GO:0033365	5.07474222486416e-18	20.2479258849558	1.73475884021867
21	473	protein localization to organelle
GO:0006605	5.2984331346356e-18	20.2017798013245	1.73842640647706
21	474	protein targeting
GO:0042254	1.33696473540869e-10	23.8564497239418	0.546467372500173
10	149	ribosome biogenesis
GO:0022613	4.38238687051113e-10	17.3591455273698	0.825202408137845
11	225	ribonucleoprotein complex biogenesis
GO:0071843	8.31956408197553e-10	16.2771512113617	0.876548335755311
11	239	cellular component biogenesis at cellular level
GO:0006364	9.57804202948499e-08	22.4354549529359	0.381426890872604
7	104	rRNA processing
GO:0016072	1.41089845216968e-07	21.1196707471507	0.403432288422946
7	110	rRNA metabolic process
GO:0042274	4.74914639992733e-07	83.8717201166181	0.0660161926510276
4	18	ribosomal small subunit biogenesis

GO:0034470	7.87291764517478e-06	11.2001576931741	0.733513251678085
7	200	ncRNA processing
GO:0034660	8.51423230299042e-06	9.06281588447653	1.04525638364127
8	285	ncRNA metabolic process
GO:0042273	1.00118700227926e-05	95.9266666666667	0.0440107951006851
3	12	ribosomal large subunit biogenesis
Tissue: Muscle_Skeletal=>Adipose_Subcutaneous	Type:	asymmetric

SourceGene:	ENSG00000229344.1	Also an expressed pseudogene?

TargetGeneSet: MTND2P28 RPL22 ICMT CLSTN1 NMNAT1 DFFA MFN2
VPS13D DDI2 SPEN UBR4 KIAA0090 LDLRAD2 HSPG2 RPL11 ARID1A
WASF2 KPNA7 ZMYM4 THRAP3 MEAF6 MFI2 MACF1 RP1 SZT2 RPS8
RPS15AP10 TM2D1 RP4 FAM73A GNG5 RPL5 RP11 SORT1 ATP5F1
HIPK1 NBPF16 HIST2H2BD RPRD2 JTB RPS27 ASH1L MIR555 SSR2
MEF2D ARHGEF11 COPA POU2F1 PRRC2C CEP350 ARPC5 RPS7 ADAM18 NBAS
ASXL2 PPP1CB DHX57 RHOQ UBC USP34 C1D AAK1 TET3 DCTN1
TMEM131 EIF5B RANBP2 UGGT1 MZT2B MLK7-AS1 SENP2 PLEKHM3 GIGYF2
SETD5 RPL32 NR2C2 RPL15 MAP4 CELSR3 LAMB2 RAD54L2 RPL29 DNAH1
APPL1 KCTD6 PCNP RPL24 CNBP NME9 PDCD10 PHC3 DNAJC19 PSMD2
EIF4G1 CPN2 HTT BOD1L RELL1 COPS4 UBE2D3 AIMP1 RPL34 RPS3A
NIPBL CTD MRPS36 TAF9 TBCA ARSB ERAP1 MAN2A1 APC HINT1 AFF4
SKP1 CTB MTND5P11 KDM3B PAIP2 PCDHGB3 PCDHGB6 RPS14 RPL15P3 NOL7
DEK GPLD1 ABT1 PPP1R10 TAPBP RPS10 RPL10A C6orf89 MTRNR2L9
WDR11 ASCC3 MRPS12 PHACTR2 IGF2R FOXK1 PHF14 MPLKIP CHCHD2 LMTK2
RPS3AP26 TRRAP GNB2 CUX1 SLC26A4 TNPO3 HIPK2 MLL3 KAT6A
HOOK3 RPS20 ASPH RBM12B RPL30 POLR2K TRAPPC9 HEATR7A VPS28 RPL8 RPS6
TLN1 CLTA CNN2P4 HSD17B3 XPA NIPSNAP3A TMEM245 FKBP15 ATP6V1G1
RPL35 RPL12 NUP188 SETX RPL7A COMMD3 PRINS FUT11 KIAA0913
RPS24 GBF1 ARL3 EIF6 RPL27A MTRNR2L8 TSG101 CKAP5 C1QTNF4
C11orf31 DDB1 GANAB FAU PACS1 PPP6R3 TMEM126B TMEM126A
SDHD BACE1 UBE4A ATP5L DDX6 RPL23AP64 VPS11 SIAE KDM5A ERC1
USP5 C12orf57 FGFR1OP2 SCAF11 MLL4 PFDN5 BAZ2A LRP1
TMBIM4 RBMS1P1 EEA1 APAF1 RPL18AP3 VPS29 ACAD10 SBNO1 ZC3H13 ESD
RPL13AP25 COMMD6 DNAJC3 HMGB3P4 CCNB1IP1 CHD8 C14orf119
C14orf21 STRN3 FNTB MED6 YLPM1 SEL1L C14orf102 CCNK
DYNC1H1 CYFIP1 HERC2 HERC2P10 RPAP1 VPS39 CCNDBP1 DUT USP8
CCPG1 RPS3AP6 LRRC49 SIN3A C15orf40 SEC11A IQGAP1 MEF2A JMJD8 NME3
SPSB3 NDUFB10 ECI1 MGRN1 NOMO1 RPS15A ZNF771 SRCAP ZNF629 CES1P2
NFATC3 AARS GABARAPL2 CDYL2 HSBP1 RPL13 VPS53 RP13 METTL16
ZZEF1 SLC35G6 RPL26 TOM1L2 RPS7P1 PIGS SUPT6H RPL23A TAOK1 CPD
TAF15 RPL19 CDK12 WIPF2 NKIRAS2 STAT5A AOC3 RPL27 NMT1 CLTC
HEATR6 DCAF7 COG1 NT5C TNRC6C RPTOR ROCK1P1 C18orf21 C18orf32
RPL17 ZNF407 TPGS1 ATP5D RPS15 RPL36 TRAPPC5 ZNF121 LPHN1 BRD4
YJEFN3 RPS19 POU2F2 MEGF8 SNRPD2 ARHGAP35 RPL18 NUCB1 RPL13A
MYH7B RPS9 ZNF587 RPS5 ATRN KIF3B NCOA6 SCAND1 RALGAPB NCOA3 ADNP
PSMD10P1 TSHZ2 RPL12P4 SON PCBP3 PI4KA YDJC FAM211B CTA MPST
APOBEC3C RPL3 TNRC6B EP300 TCF20 VCX3B TMSB4X RPL9P7 DYNLT3
USP9X CDK16 KDM5C HUWE1 GNL3L CBX1P1 RPS4X APOOL RPL39 THOC2
HCFC1 RPL10

GOBPID Pvalue OddsRatio ExpCount Count Size Term

GO:0006415	1.18E-50	52.83744681	1.851359767	44	91
translational termination					

GO:0006614	2.09E-50	44.32121378	2.136184347	46	105	SRP-dependent cotranslational protein targeting to membrane
GO:0006613	3.63E-50	43.57943548	2.15652896	46	106	cotranslational protein targeting to membrane

GO:0045047	3.63E-50	43.57943548	2.15652896	46	106

protein targeting to ER

GO:0072599	3.63E-50	43.57943548	2.15652896	46	106

establishment of protein localization to endoplasmic reticulum

GO:0070972	1.64E-47	36.28528226	2.400664314	46	118

protein localization to endoplasmic reticulum

GO:0006414	3.78E-47	40.67042623	2.136184347	44	105

translational elongation

GO:0000184	9.27E-46	34.39352003	2.421008927	45	119	"nuclear-transcribed mRNA catabolic process, nonsense-mediated decay"

GO:0006612	3.01E-44	26.11289199	3.112725763	48	153
protein targeting to membrane					
GO:0006413	5.47E-43	25.4653557	3.09238115	47	152
translational initiation					

GO:0019080	9.76E-42	24.82304147	3.072036537	46	151
viral genome expression					
GO:0019083	9.76E-42	24.82304147	3.072036537	46	151
viral transcription					

GO:0043624	4.65E-40	23.50903614	3.112725763	45	153
cellular protein complex disassembly					
GO:0043241	2.34E-39	22.46081672	3.214448827	45	158
protein complex disassembly					

GO:0000956	3.16E-39	21.16325728	3.438239568	46	169

nuclear-transcribed mRNA catabolic process

GO:0072594	6.75E-39	18.06709677	4.150301017	49	204

establishment of protein localization to organelle

GO:0034623	1.37E-38	20.32932208	3.539962632	46	174

cellular macromolecular complex disassembly

GO:0006402	4.30E-38	19.70766129	3.621341084	46	178	mRNA catabolic process
GO:0032984	5.68E-38	19.55808877	3.641685696	46	179	macromolecular complex disassembly

GO:0022415	5.99E-37	14.41012397	5.289599336	52	260
viral reproductive process					
GO:0019058	4.17E-36	15.35714286	4.699605564	49	231
viral infectious cycle					

GO:0006401	4.47E-35	16.32958004	4.17064563	46	205	RNA catabolic process

GO:0016032	3.33E-32	9.027477212	9.175420386	60	451

viral reproduction

GO:0071845	1.69E-29	11.17613898	5.777870044	47	284

cellular component disassembly at cellular level

GO:0022411	3.23E-29	10.98748467	5.859248495	47	288

cellular component disassembly

GO:0033365	2.82E-28	7.944224684	9.623001868	57	473

protein localization to organelle

GO:0006605	3.15E-28	7.924597031	9.643346481	57	474
protein targeting					
GO:0042274	3.53E-08	31.36585366	0.366203031	7	18
ribosomal small subunit biogenesis					

GO:0022613	1.73E-07	4.679064431	4.577537887	19	225	ribonucleoprotein complex biogenesis

GO:0071843	4.42E-07	4.376909091	4.862362466	19	239

cellular component biogenesis at cellular level

GO:0042254	2.12E-06	5.193333333	3.031347312	14	149

ribosome biogenesis

GO:0006364	8.53E-06	5.87803488	2.115839734	11	104	rRNA processing
GO:0016072	1.46E-05	5.519434629	2.237907411	11	110	rRNA metabolic process

Tissue: Muscle_Skeletal=>Adipose_Subcutaneous Type: cluster
SourceGene: ENSG00000229344.1
TargetGeneSet: MTND2P28 RPL22 ICMT CLSTN1 NMNAT1 DFFA MFN2
VPS13D DDI2 SPEN UBR4 KIAA0090 LDLRAD2 HSPG2 RPL11 ARID1A
WASF2 KPNA7 ZMYM4 THRAP3 MEAF6 MFI2 MACF1 RP1 SZT2 RPS8
RPS15AP10 TM2D1 RP4 FAM73A GNG5 RPL5 RP11 SORT1 ATP5F1
HIPK1 NBPF16 HIST2H2BD RPRD2 JTB RPS27 ASH1L MIR555 SSR2
MEF2D ARHGEF11 COPA POU2F1 PRRC2C CEP350 ARPC5 RPS7 ADAM18 NBAS
ASXL2 PPP1CB DHX57 RHOQ UBC USP34 C1D AAK1 TET3 DCTN1
TMEM131 EIF5B RANBP2 UGGT1 MZT2B MLK7-AS1 SENP2 PLEKHM3 GIGYF2
SETD5 RPL32 NR2C2 RPL15 MAP4 CELSR3 LAMB2 RAD54L2 RPL29 DNAH1
APPL1 KCTD6 PCNP RPL24 CNBP NME9 PDCD10 PHC3 DNAJC19 PSMD2
EIF4G1 CPN2 HTT BOD1L RELL1 COPS4 UBE2D3 AIMP1 RPL34 RPS3A
NIPBL CTD MRPS36 TAF9 TBCA ARSB ERAP1 MAN2A1 APC HINT1 AFF4
SKP1 CTB MTND5P11 KDM3B PAIP2 PCDHGB3 PCDHGB6 RPS14 RPL15P3 NOL7
DEK GPLD1 ABT1 PPP1R10 TAPBP RPS10 RPL10A C6orf89 MTRNR2L9
WDR11 ASCC3 MRPS12 PHACTR2 IGF2R FOXK1 PHF14 MPLKIP CHCHD2 LMTK2
RPS3AP26 TRRAP GNB2 CUX1 SLC26A4 TNPO3 HIPK2 MLL3 KAT6A
HOOK3 RPS20 ASPH RBM12B RPL30 POLR2K TRAPPC9 HEATR7A VPS28 RPL8 RPS6
TLN1 CLTA CNN2P4 HSD17B3 XPA NIPSNAP3A TMEM245 FKBP15 ATP6V1G1
RPL35 RPL12 NUP188 SETX RPL7A COMMD3 PRINS FUT11 KIAA0913
RPS24 GBF1 ARL3 EIF6 RPL27A MTRNR2L8 TSG101 CKAP5 C1QTNF4
C11orf31 DDB1 GANAB FAU PACS1 PPP6R3 TMEM126B TMEM126A
SDHD BACE1 UBE4A ATP5L DDX6 RPL23AP64 VPS11 SIAE KDM5A ERC1
USP5 C12orf57 FGFR1OP2 SCAF11 MLL4 PFDN5 BAZ2A LRP1
TMBIM4 RBMS1P1 EEA1 APAF1 RPL18AP3 VPS29 ACAD10 SBNO1 ZC3H13 ESD
RPL13AP25 COMMD6 DNAJC3 HMGB3P4 CCNB1IP1 CHD8 C14orf119
C14orf21 STRN3 FNTB MED6 YLPM1 SEL1L C14orf102 CCNK
DYNC1H1 CYFIP1 HERC2 HERC2P10 RPAP1 VPS39 CCNDBP1 DUT USP8
CCPG1 RPS3AP6 LRRC49 SIN3A C15orf40 SEC11A IQGAP1 MEF2A JMJD8 NME3
SPSB3 NDUFB10 ECI1 MGRN1 NOMO1 RPS15A ZNF771 SRCAP ZNF629 CES1P2
NFATC3 AARS GABARAPL2 CDYL2 HSBP1 RPL13 VPS53 RP13 METTL16
ZZEF1 SLC35G6 RPL26 TOM1L2 RPS7P1 PIGS SUPT6H RPL23A TAOK1 CPD
TAF15 RPL19 CDK12 WIPF2 NKIRAS2 STAT5A AOC3 RPL27 NMT1 CLTC
HEATR6 DCAF7 COG1 NT5C TNRC6C RPTOR ROCK1P1 C18orf21 C18orf32
RPL17 ZNF407 TPGS1 ATP5D RPS15 RPL36 TRAPPC5 ZNF121 LPHN1 BRD4
YJEFN3 RPS19 POU2F2 MEGF8 SNRPD2 ARHGAP35 RPL18 NUCB1 RPL13A
MYH7B RPS9 ZNF587 RPS5 ATRN KIF3B NCOA6 SCAND1 RALGAPB NCOA3 ADNP
PSMD10P1 TSHZ2 RPL12P4 SON PCBP3 PI4KA YDJC FAM211B CTA MPST
APOBEC3C RPL3 TNRC6B EP300 TCF20 VCX3B TMSB4X RPL9P7 DYNLT3
USP9X CDK16 KDM5C HUWE1 GNL3L CBX1P1 RPS4X APOOL RPL39 THOC2
HCFC1 RPL10

GOBPID	Pvalue	OddsRatio	ExpCount	Count	Size	Term
GO:0006415	1.18091173108757e-50	52.8374468085106	1.85135976749014			

44 91 translational termination
GO:0006614 2.09172658551235e-50 44.3212137780208 2.13618434710401
46 105 SRP-dependent cotranslational protein targeting to membrane
GO:0006613 3.63289544301091e-50 43.579435483871 2.15652895993357 46
106 cotranslational protein targeting to membrane
GO:0045047 3.63289544301091e-50 43.579435483871 2.15652895993357 46
106 protein targeting to ER
GO:0072599 3.63289544301091e-50 43.579435483871 2.15652895993357 46
106 establishment of protein localization to endoplasmic reticulum
GO:0070972 1.64396294360088e-47 36.2852822580645 2.40066431388831
46 118 protein localization to endoplasmic reticulum
GO:0006414 3.78449564522234e-47 40.6704262295082 2.13618434710401
44 105 translational elongation
GO:0000184 9.27350257945361e-46 34.3935200260501 2.42100892671787
45 119 nuclear-transcribed mRNA catabolic process, nonsense-mediated decay
GO:0006612 3.00677485738495e-44 26.1128919860627 3.11272576292298
48 153 protein targeting to membrane
GO:0006413 5.46614409583205e-43 25.4653556969346 3.09238115009342
47 152 translational initiation
GO:0019080 9.75572766221803e-42 24.8230414746544 3.07203653726386
46 151 viral genome expression
GO:0019083 9.75572766221803e-42 24.8230414746544 3.07203653726386
46 151 viral transcription
GO:0043624 4.65270176117719e-40 23.5090361445783 3.11272576292298
45 153 cellular protein complex disassembly
GO:0043241 2.34166071234943e-39 22.4608167182002 3.21444882707079
45 158 protein complex disassembly
GO:0000956 3.15866900736828e-39 21.1632572777341 3.43823956819597
46 169 nuclear-transcribed mRNA catabolic process
GO:0072594 6.74610504771765e-39 18.0670967741935 4.15030101723064
49 204 establishment of protein localization to organelle
GO:0034623 1.37459865525761e-38 20.3293220766129 3.53996263234378
46 174 cellular macromolecular complex disassembly
GO:0006402 4.29525212359941e-38 19.7076612903226 3.62134108366203
46 178 mRNA catabolic process
GO:0032984 5.68264718768384e-38 19.5580887703129 3.64168569649159
46 179 macromolecular complex disassembly
GO:0022415 5.99138943130968e-37 14.4101239669421 5.28959933568611
52 260 viral reproductive process
GO:0019058 4.17095704564696e-36 15.3571428571429 4.69960556362881
49 231 viral infectious cycle
GO:0006401 4.47343411260268e-35 16.3295800365186 4.1706456300602 46
205 RNA catabolic process
GO:0016032 3.32587370046433e-32 9.02747721162043 9.17542038613245
60 451 viral reproduction
GO:0071845 1.68874570139949e-29 11.1761389842669 5.7778700435956 47
284 cellular component disassembly at cellular level
GO:0022411 3.22850201111655e-29 10.9874846708216 5.85924849491385
47 288 cellular component disassembly
GO:0033365 2.81601968032666e-28 7.9442246835443 9.62300186838281 57
473 protein localization to organelle
GO:0006605 3.14776323708927e-28 7.92459703123577 9.64334648121237
57 474 protein targeting
GO:0042274 3.52846334098024e-08 31.3658536585366 0.366203030932115
7 18 ribosomal small subunit biogenesis
GO:0022613 1.72939544098139e-07 4.67906443071492 4.57753788665144
19 225 ribonucleoprotein complex biogenesis
GO:0071843 4.41698210520289e-07 4.37690909090909 4.86236246626531
19 239 cellular component biogenesis at cellular level
GO:0042254 2.11823520481386e-06 5.19333333333333 3.03134731160473
14 149 ribosome biogenesis
GO:0006364 8.53332664867264e-06 5.87803487974467 2.11583973427444
11 104 rRNA processing

GO:0016072 1.46426333350594e-05 5.51943462897527 2.23790741125182 11 110 rRNA metabolic process
Tissue: Muscle_Skeletal=>Adipose_Subcutaneous Type: asymmetric

SourceGene: EPCAM(ENSG00000119888.6) epithelial cell adhesion molecule (disease associations)

TargetGeneSet: C1orf200 TRAPPC3 ATRX NUF2 ASPM RRM2 HJURP DNAJC19 CENPE ANLN ESCO2 PAG1 PAPPA CEP55 MKI67 FAM111B CDCA5 ATP5L MPP2 CDCA3 RP11 TROAP HMGA2 CPM SKA3 DIAPH3 BUB1B CASC5 CCNB2 SNAPC5 POLR3K IFT140 PKMYT1 GABARAPL2 SDR42E1 CDT1 FANCA FAM64A SNORA48 TOP2A KIF18B TK1 BIRC5 CTD LINGO3 ASF1B ZNF576 TRPM4 SHANK1 UBE2C

GOBPID	Pvalue	OddsRatio	ExpCount	Count	Size	Term
GO:0000280	1.46E-15	24.97050344	1.041450419	16	350	nuclear division
GO:0007067	1.46E-15	24.97050344	1.041450419	16	350	mitosis
GO:0000087	2.27E-15	24.22739018	1.071206145	16	360	M phase of mitotic cell cycle
GO:0048285	3.49E-15	23.52626072	1.100961871	16	370	organelle fission
GO:0051301	7.88E-14	18.99014611	1.344958826	16	452	cell division

GO:0010564	1.79E-09	13.48675975	1.231887067	12	414	regulation of cell cycle process
GO:0007059	6.91E-09	24.72034632	0.416580167	8	140	chromosome segregation
GO:0071156	6.48E-08	14.6916955	0.785551173	9	264	regulation of cell cycle arrest
GO:0000086	2.01E-07	20.25486618	0.428482458	7	144	G2/M transition of mitotic cell cycle
GO:0000075	4.82E-07	13.84517705	0.720088575	8	242	cell cycle checkpoint
GO:0007050	1.10E-06	10.32941176	1.097986299	9	369	cell cycle arrest
GO:0071103	1.27E-06	15.19871795	0.562383226	7	189	DNA conformation change
GO:0051329	1.37E-06	10.04308426	1.127742025	9	379	interphase of mitotic cell cycle
GO:0051325	1.63E-06	9.824929972	1.151546606	9	387	interphase

GO:0007346	2.61E-06	10.93501211	0.901598505	8	303
regulation of mitotic cell cycle

GO:0045786	4.40E-06	8.646234195	1.300325237	9	437
negative regulation of cell cycle

GO:0000236	5.05E-06	23.56578947	0.252923673	5	85
mitotic prometaphase

GO:0007096	5.30E-06	119.9916667	0.035706871	3	12
regulation of exit from mitosis

GO:0006323	5.63E-06	15.73193602	0.455262612	6	153	DNA
packaging

GO:0031577	7.01E-06	38.7854251	0.12497405	4	42
spindle checkpoint

GO:0010458	1.34E-05	83.04807692	0.047609162	3	16	exit
from mitosis

Tissue: Muscle_Skeletal=>Adipose_Subcutaneous	Type:	asymmetric

SourceGene:	GEN1(ENSG00000178295.9) GEN1 Holliday junction 5' flap endonuclease

TargetGeneSet: RPL22 FBXO6 RPL5 RP11 PPM1J RP1 GAS5 GEN1 SLC40A1 FAM126B NBEAL1 RPL32 RPL15 RPL14 APPL1 RAB6B GRK7 RPS3A TAF9 SMN1 BTF3 CAMLG CTNNA1 CTB SRPK1 IGFBP3 C7orf57 RPS3AP26 PTCD1 RPL30 RPS3AP5 RPP30 RPL27A PFDN5 CDK2 ARPC3 DGKH ATP7B RAB15 EXD2 PSMC1 TYRO3 TP53BP1 ATP8B4 RPS3AP6 FURIN SGSM2 RPL26 MPRIP RPL23A RPL19 AOC3 PGS1 CTD RPL17 RPL36 PRKACA RPL13A RPS11 ASMT UXT RPS4X RBM41 RPL10

GOBPID	Pvalue	OddsRatio	ExpCount	Count	Size	Term
GO:0006415	6.62E-26	99.73751024	0.314857103	17	91	translational termination
GO:0006414	9.16E-25	83.78822314	0.363296658	17	105	translational elongation
GO:0006614	9.16E-25	83.78822314	0.363296658	17	105	SRP-dependent cotranslational protein targeting to membrane
GO:0006613	1.09E-24	82.84099421	0.366756626	17	106	cotranslational protein targeting to membrane
GO:0045047	1.09E-24	82.84099421	0.366756626	17	106	protein targeting to ER
GO:0072599	1.09E-24	82.84099421	0.366756626	17	106	establishment of protein localization to endoplasmic reticulum

GO:0070972	7.59E-24	72.93729373	0.408276244	17	118
protein localization to endoplasmic reticulum					
GO:0000184	8.84E-24	72.21717172	0.411736212	17	119
"nuclear-transcribed mRNA catabolic process, nonsense-mediated decay"					
GO:0019080	6.16E-22	54.84825871	0.522455193	17	151
viral genome expression					
GO:0019083	6.16E-22	54.84825871	0.522455193	17	151
viral transcription					
GO:0019058	6.90E-22	41.02115033	0.799252647	19	231
viral infectious cycle					
GO:0006413	6.92E-22	54.43815937	0.525915162	17	152
translational initiation					
GO:0006612	7.76E-22	54.03409091	0.52937513	17	153
protein targeting to membrane					
GO:0043624	7.76E-22	54.03409091	0.52937513	17	153
cellular protein complex disassembly					

GO:0043241	1.37E-21	52.09972061	0.546674971	17	158	
protein complex disassembly						
GO:0000956	4.45E-21	48.29206539	0.58473462	17	169	
nuclear-transcribed mRNA catabolic process						
GO:0022415	6.71E-21	36.01124347	0.899591724	19	260	
viral reproductive process						
GO:0034623	7.41E-21	46.73769543	0.602034461	17	174	
cellular macromolecular complex disassembly						
GO:0006402	1.10E-20	45.56371165	0.615874334	17	178	mRNA
catabolic process						
GO:0032984	1.21E-20	45.27927422	0.619334302	17	179	
macromolecular complex disassembly						
GO:0072594	1.17E-19	39.15702479	0.705833506	17	204	
establishment of protein localization to organelle						
GO:0006401	1.27E-19	38.94600258	0.709293475	17	205	RNA

catabolic process

GO:0016032	4.42E-19	23.52774659	1.560445644	21	451

viral reproduction

GO:0071845	1.15E-18	29.89074248	0.98263096	18	284

cellular component disassembly at cellular level

GO:0022411	1.47E-18	29.43958333	0.996470832	18	288

cellular component disassembly

GO:0006605	9.61E-15	17.20189145	1.640024912	18	474

protein targeting

GO:0033365	1.55E-13	15.75392079	1.636564944	17	473

protein localization to organelle

GO:0042273	8.39E-06	102.070922	0.041519618	3	12

ribosomal large subunit biogenesis

Tissue: Muscle_Skeletal=>Adipose_Subcutaneous   Type: asymmetric

SourceGene: IGKJ5(ENSG00000211593.2)   immunoglobulin kappa joining 5

TargetGeneSet: RP1 RP11 UBC RPL31 SENP2 C2orf54 RPL32 RPL35A RPL34 NSA2 RPL10A RPL39P3 C8orf59 RPL30 TPT1 COMMD6 RPS3AP6 RPL36

GOBPID	Pvalue	OddsRatio	ExpCount	Count	Size	Term
GO:0006415	5.26E-15	230.5863454	0.088159989	8	91	translational termination
GO:0006414	1.72E-14	197.1134021	0.101723064	8	105	translational elongation
GO:0006614	1.72E-14	197.1134021	0.101723064	8	105	SRP-dependent cotranslational protein targeting to membrane
GO:0006613	1.86E-14	195.0884354	0.102691855	8	106	cotranslational protein targeting to membrane
GO:0045047	1.86E-14	195.0884354	0.102691855	8	106	protein targeting to ER
GO:0072599	1.86E-14	195.0884354	0.102691855	8	106	establishment of protein localization to endoplasmic reticulum
GO:0070972	4.48E-14	173.6606061	0.114317348	8	118	protein localization to endoplasmic reticulum
GO:0000184	4.80E-14	172.0840841	0.115286139	8	119	"nuclear-transcribed mRNA catabolic process, nonsense-mediated decay"
GO:0019080	3.36E-13	133.2773893	0.146287454	8	151	viral genome expression
GO:0019083	3.36E-13	133.2773893	0.146287454	8	151	viral transcription
GO:0006413	3.54E-13	132.3425926	0.147256245	8	152	translational initiation
GO:0006612	3.74E-13	131.4206897	0.148225036	8	153	protein targeting to membrane
GO:0043624	3.74E-13	131.4206897	0.148225036	8	153	cellular protein complex disassembly
GO:0043241	4.85E-13	126.9955556	0.153068992	8	158	protein complex disassembly
GO:0000956	8.38E-13	118.2277433	0.163725694	8	169	

nuclear-transcribed mRNA catabolic process

GO:0034623 1.06E-12 114.626506 0.168569649 8 174
cellular macromolecular complex disassembly

GO:0006402 1.28E-12 111.8980392 0.172444814 8 178 mRNA
catabolic process

GO:0032984 1.34E-12 111.2358674 0.173413605 8 179
macromolecular complex disassembly

GO:0072594 3.84E-12 96.87755102 0.197633382 8 204
establishment of protein localization to organelle

GO:0006401 3.99E-12 96.37901861 0.198602173 8 205 RNA
catabolic process

GO:0019058 1.04E-11 84.98654709 0.223790741 8 231
viral infectious cycle

GO:0022415 2.70E-11 75.05291005 0.251885683 8 260
viral reproductive process

GO:0071845 5.47E-11 68.41062802 0.275136669 8 284
cellular component disassembly at cellular level

GO:0022411 6.12E-11 67.41428571 0.279011833 8 288
cellular component disassembly

GO:0016032 2.16E-09 42.11888638 0.43692478 8 451
viral reproduction

GO:0033365 3.14E-09 40.06308244 0.458238184 8 473
protein localization to organelle

GO:0006605 3.19E-09 39.97424893 0.459206975 8 474
protein targeting

Tissue: Muscle_Skeletal=>Adipose_Subcutaneous Type: cluster
SourceGene: MTND1P23(ENSG00000225972.1)
TargetGeneSet: MTND1P23 MTND2P28 UBR4 KPNA7 RP4 THRAP3 RP1
ATP5F1 HIPK1 CSDE1 ADAR ASH1L MIR555 TKT PRRC2C RP11 CEP350 LAD1
LPGAT1 ASXL2 RHOQ UBC COMMD1 AAK1 TET3 EIF5B UGGT1 MZT2B
RHOQP3 RHOQP2 MZT2A GULP1 HDLBP IL17RC MAP4 RAD54L2 PCNP RPL24
PDCD10 DNAJC19 IQCG RELL1 CISD2 RPL34 RPS3A CTD MRPS36 TBCA ARSB
ERAP1 MAN2A1 HINT1 AFF4 CTB MTND5P11 RPS14 NOL7 GPLD1
HLA-H TAPBP C6orf89 ZFAND3 MTRNR2L9 BCKDHB SNHG5 MRPS12 PHACTR2 RP3
IGF2R FOXK1 CHCHD2 CUX1 TRBV6-5 HOOK3 RBM12B TG CLTA CBWD3 XPA
ATP6V1G1 SETX RPL7A SSNA1 COMMD3 FUT11 RPL27A MTRNR2L8
CCDC85B PACS1 SDHD UBE4A ATP5L DDX6 RPL23AP64 ERC1 C12orf57

MLL4 PFDN5 HMGB1 NKX2-1 FKBP3 C14orf1 C14orf102 DUT RAB11A
KIAA1199 SEC11A NME3 ECI1 ZNF771 SRCAP FBXL19-AS1 RPL13
VPS53 RPL26 TOM1L2 RPS2P48 NT5C C17orf99 ANKRD12 GNAL C18orf32
GCDH BRD4 ARHGAP35 RPL18 ZNF551 ZNF587 RPS5 KIF3B ADNP
ATP9A TSHZ2 RPL12P4 YDJC HMGB1P10 MPST RPL3 RP6 USP9X
HUWE1 GNL3L COX7B RPL39 HCFC1
GOBPID Pvalue OddsRatio ExpCount Count Size Term
GO:0006614 2.85187993110751e-14 23.8361204013378 0.770188914261989
14 105 SRP-dependent cotranslational protein targeting to membrane
GO:0006613 3.2662520390078e-14 23.5753780718336 0.77752404677877
14 106 cotranslational protein targeting to membrane
GO:0045047 3.2662520390078e-14 23.5753780718336 0.77752404677877
14 106 protein targeting to ER
GO:0072599 3.2662520390078e-14 23.5753780718336 0.77752404677877
14 106 establishment of protein localization to endoplasmic reticulum
GO:0006415 9.97513105924338e-14 25.5681003584229 0.667497059027057
13 91 translational termination
GO:0070972 1.50034564504427e-13 20.8375836120401 0.86554563698014
14 118 protein localization to endoplasmic reticulum
GO:0006612 3.3319676772627e-13 16.9696607740086 1.12227527506747
15 153 protein targeting to membrane
GO:0006414 6.67721192852802e-13 21.65603085554 0.770188914261989 13
105 translational elongation
GO:0072594 1.66474874630968e-12 13.3872340425532 1.49636703342329
16 204 establishment of protein localization to organelle
GO:0000184 3.43323659244544e-12 18.7773382024751 0.872880769496921
13 119 nuclear-transcribed mRNA catabolic process, nonsense-mediated decay
GO:0006413 5.1002164221504e-12 15.6661940768746 1.11494014255069
14 152 translational initiation
GO:0000956 2.16589459825813e-11 13.9312762973352 1.23963739533596
14 169 nuclear-transcribed mRNA catabolic process
GO:0006402 4.3664296916785e-11 13.1584040296925 1.30565358798699
14 178 mRNA catabolic process
GO:0019080 7.26080401728964e-11 14.3907589216145 1.10760501003391
13 151 viral genome expression
GO:0019083 7.26080401728964e-11 14.3907589216145 1.10760501003391
13 151 viral transcription
GO:0043624 8.57319919396345e-11 14.1831797235023 1.12227527506747
13 153 cellular protein complex disassembly
GO:0043241 1.28533505959621e-10 13.689284390063 1.15895093765137 13
158 protein complex disassembly
GO:0006401 2.87805973677657e-10 11.2768040063738 1.50370216594007
14 205 RNA catabolic process
GO:0034623 4.29033958791117e-10 12.3149669404929 1.27631305791987
13 174 cellular macromolecular complex disassembly
GO:0032984 6.09615116983799e-10 11.9398238113745 1.31298872050377
13 179 macromolecular complex disassembly
GO:0071845 2.27349123483779e-09 8.62535234282446 2.08317763476576
15 284 cellular component disassembly at cellular level
GO:0022411 2.75236896680963e-09 8.49655838666828 2.11251816483288
15 288 cellular component disassembly
GO:0022415 6.35358966199288e-09 8.72154471544715 1.90713445436302
14 260 viral reproductive process
GO:0033365 9.09854180531894e-09 6.24425574425574 3.46951768043734
18 473 protein localization to organelle
GO:0019058 1.3585501099947e-08 9.05844924533886 1.69441561137638
13 231 viral infectious cycle
GO:0006605 5.84999051009004e-08 5.80473532810464 3.47685281295412
17 474 protein targeting
GO:0016032 9.88474098314292e-07 5.25846859562456 3.30814476506816
15 451 viral reproduction
Tissue: Muscle_Skeletal=>Adipose_Subcutaneous Type: asymmetric

SourceGene: MTND2P28(ENSG00000225630.1) pseudogene

TargetGeneSet: MTND1P23 MTND2P28 RPL22 DFFA VPS13D DDI2
C1orf134 UBR4 ARID1A KPNA7 THRAP3 MFI2 RP1 RPS8 RPS15AP10
RPL5 RP11 SORT1 HIPK1 NBPF16 JTB RPS27 ASH1L POU2F1 PRRC2C
CEP350 LPGAT1 KIDINS220 NBAS ASXL2 PPP1CB RHOQ UBC USP34 AAK1
TET3 DCTN1 COA5 EIF5B RANBP2 UGGT1 MZT2B RHOQP3 RHOQP2 PLEKHM3
HDLBP NR2C2 RPL15 MAP4 LAMB2 RAD54L2 RN5S132 RPL29 SEMA3G CTD PCNP
RPL24 DNAJC13 PDCD10 PHC3 DNAJC19 EIF4G1 HTT RP13 RELL1 OCIAD1
COPS4 AIMP1 RPL34 RPS3A NIPBL MRPS36 TBCA ARSB ERAP1 MAN2A1 APC
HINT1 AFF4 SKP1 CTB MTND5P11 KDM3B PCDHGA6 PCDHGC5 RPS14 NOL7
GPLD1 HLA-H TAPBP RPS10 RPL10A ZFAND3 DST MTRNR2L9 WDR11
SNHG5 ASCC3 MRPS12 PHACTR2 RP3 IGF2R FOXK1 PHF14 THSD7A MALSU1
CHCHD2 LMTK2 RPS3AP26 BUD31 SPDYE2 TNPO3 HIPK2 MLL3 KAT6A
HOOK3 RPS20 ASPH RPL30 TG TRAPPC9 NFIB TLN1 CLTA XPA
RPL12 SETX RPL7A FAM208B COMMD3 PRINS FUT11 RPS24 EIF6 CD151
RPL27A MTRNR2L8 TSG101 C11orf31 GANAB PACS1 PPP6R3 TMEM126B

TMEM126A		UBE4A	DDX6	RPL23AP64		APPL2	KDM5A	ERC1	C12orf57	
PHB2	SCAF11	MLL4	PFDN5	TMBIM4	RBMS1P1	C12orf73		ACAD10	RPL6	
SBNO1	GOLGA3	ESD	COMMD6	HMGB3P4	CHD8	NKX2-1	NIN	C14orf102		
CYFIP1	HERC2	HERC2P10		C15orf40		SEC11A	LRRK1	JMJD8	NME3	
SPSB3	ECI1	MGRN1	EMP2	RPS15A	ZNF771	SRCAP	ATXN1L	CDYL2	BANP	
VPS53	METTL16	ZZEF1	ALOX12	SLC35G6	RPL26	SUPT6H	RPL23A	TAOK1	TAF15	
ZNHIT3	RPL19	CDK12	WIPF2	RPL27	ANKRD40	CLTC	MED13	TNRC6C	ROCK1P1	
ANKRD12	C18orf21		C18orf32		RPL17	ZNF407	ZBTB7A	ZNF121	HNRNPA1P10	
BRD4	RPSAP58	ARHGAP35		RPL18	RPL13A	MYH7B	ZNF587	ZNF8	RPS5	HM13
KIF3B	NCOA6	RALGAPB	NCOA3	ADNP	TSHZ2	RPL12P4	PCMTD2	SON	PEX26	
PI4KA	YDJC	CTA	HMGB1P10		MPST	RPL3	TNRC6B	EP300	VCX3B	
USP9X	HUWE1	GNL3L	RPS4X	APOOL	RPL39	THOC2	XIAP	HCFC1	RPL10	

GOBPID	Pvalue	OddsRatio	ExpCount	Count	Size	Term
GO:0006415	3.38E-39	50.17851788	1.221645561	33	91	translational termination

GO:0006614	2.14E-38	42.45809859	1.409591032	34	105

SRP-dependent cotranslational protein targeting to membrane

GO:0006613	3.11E-38	41.86545139	1.423015708	34	106

cotranslational protein targeting to membrane

GO:0045047	3.11E-38	41.86545139	1.423015708	34	106

protein targeting to ER

GO:0072599	3.11E-38	41.86545139	1.423015708	34	106

establishment of protein localization to endoplasmic reticulum

GO:0006414	8.90E-37	40.38172878	1.409591032	33	105

translational elongation

GO:0070972	2.06E-36	35.85431548	1.584111826	34	118
protein localization to endoplasmic reticulum

GO:0000184	2.85E-36	35.43	1.597536503	34	119
"nuclear-transcribed mRNA catabolic process, nonsense-mediated decay"

GO:0006612	5.07E-35	27.53651412	2.053975503	36	153
protein targeting to membrane

GO:0006413	1.07E-33	26.60323604	2.040550827	35	152
translational initiation					
GO:0019080	2.21E-32	25.68162393	2.02712615	34	151
viral genome expression					

GO:0019083	2.21E-32	25.68162393	2.02712615	34	151

viral transcription

GO:0043624	3.58E-32	25.24642857	2.053975503	34	153

cellular protein complex disassembly

GO:0000956	5.79E-32	23.20027222	2.268770327	35	169

nuclear-transcribed mRNA catabolic process

GO:0043241	1.16E-31	24.21985887	2.121098886	34	158
protein complex disassembly					
GO:0072594	1.54E-31	19.88367215	2.738634005	37	204
establishment of protein localization to organelle					

GO:0034623	1.71E-31	22.35781186	2.33589371	35	174	cellular macromolecular complex disassembly
GO:0006402	3.96E-31	21.72626116	2.389592416	35	178	mRNA catabolic process
GO:0032984	4.87E-31	21.5738557	2.403017092	35	179	macromolecular complex disassembly

GO:0006401	6.84E-29	18.24065853	2.752058681	35	205	RNA catabolic process
GO:0022415	9.58E-29	15.39991915	3.490415888	38	260	viral reproductive process

GO:0019058	4.85E-27	15.79177898	3.10110027	35	231

viral infectious cycle

GO:0033365	6.50E-26	9.758279496	6.349871981	45	473

protein localization to organelle

GO:0071845	4.98E-25	12.87066149	3.812608124	36	284

cellular component disassembly at cellular level

GO:0022411	8.16E-25	12.66274864	3.86630683	36	288
cellular component disassembly					
GO:0006605	6.46E-24	9.135047095	6.363296658	43	474
protein targeting					

GO:0016032 7.82E-23 9.050326797 6.054529098 41 451
viral reproduction

Tissue: Muscle_Skeletal=>Adipose_Subcutaneous Type: cluster
SourceGene: MTND2P28(ENSG00000225630.1)
TargetGeneSet: MTND1P23 MTND2P28 RPL22 DFFA VPS13D DDI2
C1orf134 UBR4 ARID1A KPNA7 THRAP3 MFI2 RP1 RPS8 RPS15AP10
RPL5 RP11 SORT1 HIPK1 NBPF16 JTB RPS27 ASH1L POU2F1 PRRC2C
CEP350 LPGAT1 KIDINS220 NBAS ASXL2 PPP1CB RHOQ UBC USP34 AAK1
TET3 DCTN1 COA5 EIF5B RANBP2 UGGT1 MZT2B RHOQP3 RHOQP2 PLEKHM3
HDLBP NR2C2 RPL15 MAP4 LAMB2 RAD54L2 RN5S132 RPL29 SEMA3G CTD PCNP
RPL24 DNAJC13 PDCD10 PHC3 DNAJC19 EIF4G1 HTT RP13 RELL1 OCIAD1
COPS4 AIMP1 RPL34 RPS3A NIPBL MRPS36 TBCA ARSB ERAP1 MAN2A1 APC
HINT1 AFF4 SKP1 CTB MTND5P11 KDM3B PCDHGA6 PCDHGC5 RPS14 NOL7
GPLD1 HLA-H TAPBP RPS10 RPL10A ZFAND3 DST MTRNR2L9 WDR11
SNHG5 ASCC3 MRPS12 PHACTR2 RP3 IGF2R FOXK1 PHF14 THSD7A MALSU1
CHCHD2 LMTK2 RPS3AP26 BUD31 SPDYE2 TNPO3 HIPK2 MLL3 KAT6A
HOOK3 RPS20 ASPH RPL30 TG TRAPPC9 NFIB TLN1 CLTA XPA
RPL12 SETX RPL7A FAM208B COMMD3 PRINS FUT11 RPS24 EIF6 CD151
RPL27A MTRNR2L8 TSG101 C11orf31 GANAB PACS1 PPP6R3 TMEM126B
TMEM126A UBE4A DDX6 RPL23AP64 APPL2 KDM5A ERC1 C12orf57
PHB2 SCAF11 MLL4 PFDN5 TMBIM4 RBMS1P1 C12orf73 ACAD10 RPL6
SBNO1 GOLGA3 ESD COMMD6 HMGB3P4 CHD8 NKX2-1 NIN C14orf102
CYFIP1 HERC2 HERC2P10 C15orf40 SEC11A LRRK1 JMJD8 NME3
SPSB3 ECI1 MGRN1 EMP2 RPS15A ZNF771 SRCAP ATXN1L CDYL2 BANP
VPS53 METTL16 ZZEF1 ALOX12 SLC35G6 RPL26 SUPT6H RPL23A TAOK1 TAF15
ZNHIT3 RPL19 CDK12 WIPF2 RPL27 ANKRD40 CLTC MED13 TNRC6C ROCK1P1
ANKRD12 C18orf21 C18orf32 RPL17 ZNF407 ZBTB7A ZNF121 HNRNPA1P10
BRD4 RPSAP58 ARHGAP35 RPL18 RPL13A MYH7B ZNF587 ZNF8 RPS5 HM13
KIF3B NCOA6 RALGAPB NCOA3 ADNP TSHZ2 RPL12P4 PCMTD2 SON PEX26
PI4KA YDJC CTA HMGB1P10 MPST RPL3 TNRC6B EP300 VCX3B
USP9X HUWE1 GNL3L RPS4X APOOL RPL39 THOC2 XIAP HCFC1 RPL10
GOBPID Pvalue OddsRatio ExpCount Count Size Term
GO:0006415 3.37848132117428e-39 50.1785178839152 1.22164556086084
33 91 translational termination
GO:0006614 2.13651690835942e-38 42.4580985915493 1.40959103176251
34 105 SRP-dependent cotranslational protein targeting to membrane

GO:0006613 3.11137614749354e-38 41.8654513888889 1.42301570825548
34 106 cotranslational protein targeting to membrane
GO:0045047 3.11137614749354e-38 41.8654513888889 1.42301570825548
34 106 protein targeting to ER
GO:0072599 3.11137614749354e-38 41.8654513888889 1.42301570825548
34 106 establishment of protein localization to endoplasmic reticulum
GO:0006414 8.9001924638916e-37 40.3817287784679 1.40959103176251
33 105 translational elongation
GO:0070972 2.06153783064841e-36 35.8543154761905 1.5841118261712 34
118 protein localization to endoplasmic reticulum
GO:0000184 2.85488460075027e-36 35.43 1.59753650266418 34 119
nuclear-transcribed mRNA catabolic process, nonsense-mediated decay
GO:0006612 5.06588451374648e-35 27.5365141187926 2.05397550342537
36 153 protein targeting to membrane
GO:0006413 1.07329332825106e-33 26.6032360371983 2.04055082693239
35 152 translational initiation
GO:0019080 2.21174037966493e-32 25.6816239316239 2.02712615043942
34 151 viral genome expression
GO:0019083 2.21174037966493e-32 25.6816239316239 2.02712615043942
34 151 viral transcription
GO:0043624 3.5839345031581e-32 25.2464285714286 2.05397550342537
34 153 cellular protein complex disassembly
GO:0000956 5.78680390021384e-32 23.2002722237867 2.26877032731299
35 169 nuclear-transcribed mRNA catabolic process
GO:0043241 1.16020677849e-31 24.2198588709677 2.12109888589025
34 158 protein complex disassembly
GO:0072594 1.54032264360333e-31 19.8836721461536 2.73863400456716
37 204 establishment of protein localization to organelle
GO:0034623 1.70970250788406e-31 22.3578118637166 2.33589370977787
35 174 cellular macromolecular complex disassembly
GO:0006402 3.96285383659401e-31 21.7262611602234 2.38959241574977
35 178 mRNA catabolic process
GO:0032984 4.87277837579616e-31 21.573855695318 2.40301709224275 35
179 macromolecular complex disassembly
GO:0006401 6.84005738847217e-29 18.240658527562 2.75205868106013 35
205 RNA catabolic process
GO:0022415 9.58139378065215e-29 15.3999191499191 3.49041588817383
38 260 viral reproductive process
GO:0019058 4.85276860766041e-27 15.7917789757412 3.10110026987752
35 231 viral infectious cycle
GO:0033365 6.50112578404979e-26 9.75827949570344 6.34987198117777
45 473 protein localization to organelle
GO:0071845 4.98394237888988e-25 12.8706614944875 3.81260812400526
36 284 cellular component disassembly at cellular level
GO:0022411 8.16468621454925e-25 12.6627486437613 3.86630682997716
36 288 cellular component disassembly
GO:0006605 6.45691417132307e-24 9.13504709515834 6.36329665767075
43 474 protein targeting
GO:0016032 7.82496057998823e-23 9.05032679738562 6.0545290983323 41
451 viral reproduction
Tissue: Muscle_Skeletal=>Adipose_Subcutaneous Type: cluster

SourceGene: MTRNR2L8(ENSG00000255823.1) humanin-like protein 8

TargetGeneSet: MTND2P28 NMNAT1 RP4 THRAP3 RPS8 RPS15AP10 RPL5
RP11 ASH1L PRRC2C CEP350 RPS7 ADAM18 EIF5B MAP4K4 UGGT1 MZT2B
RHOQP3 GIGYF2 RPL15 MAP4 RAD54L2 RPL29 APPL1 RPL10AP6 TMF1 PCNP
RPL24 C3orf15 PHC3 DNAJC19 LMLN-AS1 HTT BOD1L RELL1 ZBTB12B
RPL34 CTD ERAP1 PTMAP2 AFF4 CTB MTND5P11 RPL15P3 TAPBP
ZFAND3 MTRNR2L9 WDR11 IYD IGF2R STARD3NL RPS3AP26
TNPO3 TRPV6 MLL3 DRP2 HMBOX1 HOOK3 ARFGEF1 RBM12B TG NIPSNAP3A
SETX EEF1A1P5 RPL7A RPS24 RPS3AP5 EMX2 RPL27A MTRNR2L8
CKAP5 TMEM126B UBE4A DDX6 SIAE C12orf57 SCAF11 MLL4
PFDN5 TMBIM4 EEA1 RP3 RPL6 RP1 SBNO1 RPSAP54 LATS2 MRP63
GTF3A DIS3 HMGB3P4 CCNB1IP1 C14orf21 YLPM1 CCNK HERC2P10
CALML4 MYO9A SEC11A ASB9P1 LRRK1 MPG NME3 NDUFB10 RPS15A LONP2
NFATC3 RPL13 VPS53 WDR81 RPL26 TOM1L2 RPS7P1 SUPT6H RPL23A TAOK1
NKIRAS2 STAT5A RPL27 RPS7P11 NFE2L1 CLTC MED13 GPR142 TNRC6C FN3KRP
ROCK1P1 ANKRD12 FAM108A1 ZNF121 BRD4 RPSAP58 C19orf54 RPL18
NUCB1 ZNF587 RPS5 REM1 KIF3B NCOA3 TSHZ2 RPL12P4 UBE2G2 FAM108A5P
HMGB1P10 MPST UQCRFS1P1 TNRC6B DYNLT3 USP9X GNL3L COX7B
PGAM4 RPL39

GOBPID	Pvalue	OddsRatio	ExpCount	Count	Size	Term
GO:0006614	3.61E-23	38.56389452	0.777454847	20	105	SRP-dependent cotranslational protein targeting to membrane

GO:0006613	4.43E-23	38.11280406	0.784859179	20	106
cotranslational protein targeting to membrane					
GO:0045047	4.43E-23	38.11280406	0.784859179	20	106
protein targeting to ER					
GO:0072599	4.43E-23	38.11280406	0.784859179	20	106
establishment of protein localization to endoplasmic reticulum					
GO:0006415	7.32E-23	42.7979798	0.673794201	19	91
translational termination					

GO:0070972	4.34E-22	33.41778091	0.873711162	20	118
protein localization to endoplasmic reticulum					
GO:0006414	1.36E-21	35.79571882	0.777454847	19	105
translational elongation					
GO:0000184	1.68E-20	30.75409091	0.881115494	19	119
"nuclear-transcribed mRNA catabolic process, nonsense-mediated decay"					
GO:0000956	3.16E-20	23.4220616	1.251332088	21	169
nuclear-transcribed mRNA catabolic process					

GO:0006413	8.40E-20	24.75095785	1.125458446	20	152	translational initiation
GO:0072594	8.52E-20	20.13988365	1.510483704	22	204	establishment of protein localization to organelle
GO:0006402	9.54E-20	22.06539772	1.317971075	21	178	mRNA catabolic process
GO:0006612	9.61E-20	24.56313197	1.132862778	20	153	protein targeting to membrane

GO:0019080	1.80E-18	23.24621212	1.118054114	19	151	
viral genome expression						
GO:0019083	1.80E-18	23.24621212	1.118054114	19	151	
viral transcription						
GO:0006401	1.87E-18	18.7917088	1.517888035	21	205	RNA
catabolic process						
GO:0043624	2.32E-18	22.89603121	1.132862778	19	153	

cellular protein complex disassembly

GO:0043241	4.32E-18	22.06466645	1.169884437	19	158

protein complex disassembly

GO:0034623	2.74E-17	19.76473607	1.288353747	19	174

cellular macromolecular complex disassembly

GO:0032984	4.69E-17	19.14034091	1.325375407	19	179

macromolecular complex disassembly

GO:0033365	5.60E-16	9.979341011	3.502248979	26	473

protein localization to organelle

GO:0019058	5.53E-15	14.39258148	1.710400664	19	231

viral infectious cycle

GO:0006605	5.81E-15	9.434923135	3.509653311	25	474

protein targeting

GO:0022415	4.81E-14	12.63471332	1.925126289	19	260

viral reproductive process

GO:0071845	2.37E-13	11.47088336	2.102830254	19	284
cellular component disassembly at cellular level					
GO:0022411	3.05E-13	11.29710206	2.132447581	19	288
cellular component disassembly					
GO:0016032	7.29E-10	6.953072391	3.339353678	19	451
viral reproduction					
GO:0022613	8.53E-07	7.565712617	1.665974673	11	225
ribonucleoprotein complex biogenesis					

GO:0071843 1.54E-06 7.094115497 1.769635319 11 239
cellular component biogenesis at cellular level

GO:0042254 1.46E-05 8.139838097 1.10324545 8 149
ribosome biogenesis

Tissue: Muscle_Skeletal=>Adipose_Subcutaneous Type: asymmetric
SourceGene: RP1-199J3.5(ENSG00000219755.1)
TargetGeneSet: EIF3I NFYC GAS5 EIF3FP3 HAT1 RPL24 EIF2S1 RP11
RIOK2 GNB2L1 RPL15P3 CYC1 STOML2 FXN SNORA65 RPL7A MMS19 BCCIP PHB2
PPP1CC RP13 RPS15A KARS RPS7P1 RPL19 RPS2P48 MRPL38 TIMM21 RPL18 CA11
RPL13A PCMTD2 C21orf59 EIF3L RPL3 RP3 PQBP1
GOBPID Pvalue OddsRatio ExpCount Count Size Term
GO:0006413 3.51044963663819e-14 67.0610328638498 0.262957580790257
10 152 translational initiation
GO:0006415 1.36125432420966e-10 66.3981481481482 0.157428551657325
7 91 translational termination
GO:0006414 3.76966412411771e-10 56.8571428571429 0.181648328835375
7 105 translational elongation
GO:0006614 3.76966412411771e-10 56.8571428571429 0.181648328835375
7 105 SRP-dependent cotranslational protein targeting to membrane
GO:0006613 4.03178584212367e-10 56.2789001122334 0.183378312919521
7 106 cotranslational protein targeting to membrane
GO:0045047 4.03178584212367e-10 56.2789001122334 0.183378312919521
7 106 protein targeting to ER
GO:0072599 4.03178584212367e-10 56.2789001122334 0.183378312919521
7 106 establishment of protein localization to endoplasmic reticulum
GO:0070972 8.60841328508279e-10 50.1526526526527 0.204138121929278
7 118 protein localization to endoplasmic reticulum
GO:0000184 9.13641681991106e-10 49.7013888888889 0.205868106013425
7 119 nuclear-transcribed mRNA catabolic process, nonsense-mediated decay
GO:0072594 1.21023242562198e-09 34.1656662665066 0.352916753165871
8 204 establishment of protein localization to organelle
GO:0019080 4.85655971717182e-09 38.5702160493827 0.26122759670611

7	151	viral genome expression
GO:0019083	4.85655971717182e-09	38.5702160493827	0.26122759670611
7	151	viral transcription
GO:0006612	5.32340134246467e-09	38.0365296803653	0.264687564874403
7	153	protein targeting to membrane
GO:0043624	5.32340134246467e-09	38.0365296803653	0.264687564874403
7	153	cellular protein complex disassembly
GO:0043241	6.66046984147184e-09	36.7641648270787	0.273337485295135
7	158	protein complex disassembly
GO:0000956	1.0634672804538e-08	34.241426611797 0.292367310220746	7
169	nuclear-transcribed mRNA catabolic process
GO:0034623	1.30186743611952e-08	33.2045908183633	0.301017230641478
7	174	cellular macromolecular complex disassembly
GO:0006402	1.52391365969805e-08	32.4187784275504	0.307937166978064
7	178	mRNA catabolic process
GO:0032984	1.58419103619109e-08	32.2280361757106	0.30966715106221
7	179	macromolecular complex disassembly
GO:0006401	4.03930072703358e-08	27.9450056116723	0.354646737250017
7	205	RNA catabolic process
GO:0019058	9.16197193198717e-08	24.65625	0.399626323437824	7
231	viral infectious cycle
GO:0022415	2.05171463523108e-07	21.7854633289416	0.449795861878071
7	260	viral reproductive process
GO:0071845	3.73284780332888e-07	19.8642198154834	0.491315479897585
7	284	cellular component disassembly at cellular level
GO:0022411	4.10292613740075e-07	19.5759193357058	0.498235416234171
7	288	cellular component disassembly
GO:0033365	8.22122464461807e-07	14.1287792536369	0.818282471801259
8	473	protein localization to organelle
GO:0006605	8.35341126315392e-07	14.0974501388538	0.820012455885406
8	474	protein targeting
GO:0016032	8.12287569685107e-06	12.2464964964965	0.780222821950038
7	451	viral reproduction
Tissue: Muscle_Skeletal=>Adipose_Subcutaneous	Type:	asymmetric

SourceGene:	RP11-10B2.1(ENSG00000230916.1)	agan a processed expressed pseudogene

TargetGeneSet: MTND1P23 MTND2P28 NMNAT1 DFFA UBR4 LDLRAD2
HSPG2 KPNA7 THRAP3 RPS8 RPS15AP10 CCBL2 RPL5 RP11 ATP5F1
RPS27 ASH1L MEF2D COPA PRRC2C CEP350 NBAS PPP1CB UBC C1D AAK1
DCTN1 EIF5B MAP4K4 IL1RL2 RANBP2 UGGT1 MZT2B HAT1 HDLBP CRELD1
RPL15 MAP4 USP4 RAD54L2 RPL29 TMF1 PCNP RPL24 CNBP PDCD10
DNAJC19 AFAP1 BOD1L RELL1 CISD2 RPL34 RPS3A NIPBL CTD MRPS36
ERAP1 AFF4 SKP1 CTB MTND5P11 CTNNA1 PCDHGC5 TRIM41 GNB2L1
TMEM14C RPL15P3 DEK GPLD1 RPS10 RPL10A ZFAND3 MTRNR2L9 WDR11
BCKDHB PHACTR2 IGF2R FOXK1 PHF14 YWHAG CACNA2D1 RPS3AP26
TNPO3 MLL3 DRP2 HOOK3 RPS20 PAG1 RBM12B POLR2K TG TLN1 CLTA
XPA ATP6V1G1 RPL35 SETX RPL7A SEC16A PRINS FUT11 KIAA0913
RPS24 RPS3AP5 MRPL43 ARL3 SFR1 EIF6 CD151 RPL27A MTRNR2L8
CKAP5 C1QTNF4 GANAB PACS1 TMEM126B UBE4A ATP5L DDX6 ERC1
C12orf57 PHB2 SCAF11 PFDN5 LRP1 APAF1 RPL18AP3 VPS29
SBNO1 GOLGA3 HMGB1 ZC3H13 ESD ITM2B CCNB1IP1 TEP1 DNAL1
SEL1L DYNC1H1 MYO9A SEC11A MPG SPSB3 NDUFB10 PGPEP1 MGRN1 ABCC1
RPS15A NFATC3 CDYL2 RPL13 VPS53 RP13 TLCD2 WDR81 RPL26 TOM1L2
SUPT6H RPL23A TAOK1 TAF15 RPL19 CDK12 NKIRAS2 RPL27 NMT1 RPS7P11 CLTC
SUMO2 ANKRD12 C18orf32 RPL17 TPGS1 RPS15 NFIC LONP1 HNRNPA1P10
BRD4 RPL18 NUCB1 RPL13A ZNF587 RPS5 RRBP1 REM1 HM13 KIF3B
SCAND1 RALGAPB NCOA3 ADNP TSHZ2 RPL12P4 UBE2G2 HMGB1P10 RP3 MPST
TNRC6B TCF20 VCX3B USP9X GNL3L RPS4X COX7B APOOL RPL39 THOC2 XIAP
HCFC1

GOBPID	Pvalue	OddsRatio	ExpCount	Count	Size	Term
GO:0006415	1.69E-34	47.85437456	1.057919867	29	91	translational termination

GO:0006614	4.91E-34	41.1826087	1.22067677	30	105

SRP-dependent cotranslational protein targeting to membrane

GO:0006613	6.78E-34	40.63787185	1.232302263	30	106

cotranslational protein targeting to membrane

GO:0045047	6.78E-34	40.63787185	1.232302263	30	106

protein targeting to ER

GO:0072599	6.78E-34	40.63787185	1.232302263	30	106

establishment of protein localization to endoplasmic reticulum

GO:0006414	1.98E-32	39.00066263	1.22067677	29	105

translational elongation

GO:0070972	2.52E-32	35.0666996	1.371808179	30	118

protein localization to endoplasmic reticulum

GO:0000184	1.14E-30	32.90143885	1.383433672	29	119
"nuclear-transcribed mRNA catabolic process, nonsense-mediated decay"

GO:0006612	4.58E-30	26.26486778	1.778700436	31	153
protein targeting to membrane

GO:0019080	7.80E-29	25.44376572	1.75544945	30	151
viral genome expression

GO:0019083	7.80E-29	25.44376572	1.75544945	30	151
viral transcription					
GO:0006413	9.63E-29	25.23342837	1.767074943	30	152
translational initiation					
GO:0072594	2.63E-27	19.30369357	2.371600581	32	204
establishment of protein localization to organelle					

GO:0000956	2.74E-27	22.12073819	1.964708325	30	169

nuclear-transcribed mRNA catabolic process

GO:0043624	2.93E-27	23.82287074	1.778700436	29	153

cellular protein complex disassembly

GO:0043241	7.82E-27	22.8914171	1.836827901	29	158

protein complex disassembly

GO:0006402	1.39E-26	20.76233843	2.069337762	30	178	mRNA catabolic process
GO:0034623	1.44E-25	20.34244604	2.02283579	29	174	cellular macromolecular complex disassembly
GO:0032984	3.36E-25	19.65741007	2.080963255	29	179	macromolecular complex disassembly

GO:0022415	4.37E-25	15.1361723	3.022628192	33	260	
viral reproductive process						
GO:0006401	1.07E-24	17.52546584	2.383226074	30	205	RNA
catabolic process						
GO:0019058	2.47E-24	15.93332117	2.685488894	31	231	
viral infectious cycle						

GO:0016032	2.38E-22	9.816725647	5.243097364	38	451

viral reproduction

GO:0071845	1.37E-21	12.54810883	3.301640025	31	284

cellular component disassembly at cellular level

GO:0022411	2.08E-21	12.34928569	3.348141997	31	288

cellular component disassembly

GO:0033365	1.19E-20	8.97014847	5.498858211	37	473

protein localization to organelle

GO:0006605	1.28E-20	8.948975492	5.510483704	37	474

protein targeting

GO:0022613	2.26E-06	5.566737675	2.615735935	13	225

ribonucleoprotein complex biogenesis

GO:0071843 4.39E-06 5.216699971 2.778492838 13 239
cellular component biogenesis at cellular level

Tissue: Muscle_Skeletal=>Adipose_Subcutaneous Type: cluster
SourceGene: RP11-10B2.1(ENSG00000230916.1)
TargetGeneSet: MTND1P23 MTND2P28 NMNAT1 DFFA UBR4 LDLRAD2
HSPG2 KPNA7 THRAP3 RPS8 RPS15AP10 CCBL2 RPL5 RP11 ATP5F1
RPS27 ASH1L MEF2D COPA PRRC2C CEP350 NBAS PPP1CB UBC C1D AAK1
DCTN1 EIF5B MAP4K4 IL1RL2 RANBP2 UGGT1 MZT2B HAT1 HDLBP CRELD1
RPL15 MAP4 USP4 RAD54L2 RPL29 TMF1 PCNP RPL24 CNBP PDCD10
DNAJC19 AFAP1 BOD1L RELL1 CISD2 RPL34 RPS3A NIPBL CTD MRPS36
ERAP1 AFF4 SKP1 CTB MTND5P11 CTNNA1 PCDHGC5 TRIM41 GNB2L1
TMEM14C RPL15P3 DEK GPLD1 RPS10 RPL10A ZFAND3 MTRNR2L9 WDR11
BCKDHB PHACTR2 IGF2R FOXK1 PHF14 YWHAG CACNA2D1 RPS3AP26
TNPO3 MLL3 DRP2 HOOK3 RPS20 PAG1 RBM12B POLR2K TG TLN1 CLTA
XPA ATP6V1G1 RPL35 SETX RPL7A SEC16A PRINS FUT11 KIAA0913
RPS24 RPS3AP5 MRPL43 ARL3 SFR1 EIF6 CD151 RPL27A MTRNR2L8
CKAP5 C1QTNF4 GANAB PACS1 TMEM126B UBE4A ATP5L DDX6 ERC1
C12orf57 PHB2 SCAF11 PFDN5 LRP1 APAF1 RPL18AP3 VPS29
SBNO1 GOLGA3 HMGB1 ZC3H13 ESD ITM2B CCNB1IP1 TEP1 DNAL1
SEL1L DYNC1H1 MYO9A SEC11A MPG SPSB3 NDUFB10 PGPEP1 MGRN1 ABCC1
RPS15A NFATC3 CDYL2 RPL13 VPS53 RP13 TLCD2 WDR81 RPL26 TOM1L2
SUPT6H RPL23A TAOK1 TAF15 RPL19 CDK12 NKIRAS2 RPL27 NMT1 RPS7P11 CLTC
SUMO2 ANKRD12 C18orf32 RPL17 TPGS1 RPS15 NFIC LONP1 HNRNPA1P10
BRD4 RPL18 NUCB1 RPL13A ZNF587 RPS5 RRBP1 REM1 HM13 KIF3B

SCAND1 RALGAPB NCOA3 ADNP TSHZ2 RPL12P4 UBE2G2 HMGB1P10 RP3 MPST TNRC6B TCF20 VCX3B USP9X GNL3L RPS4X COX7B APOOL RPL39 THOC2 XIAP HCFC1

GOBPID	Pvalue	OddsRatio	ExpCount	Count	Size	Term
GO:0006415	1.69315822596365e-34	47.8543745648642	1.05791986713722	29	91	translational termination
GO:0006614	4.90507034226677e-34	41.1826086956522	1.22067676977372	30	105	SRP-dependent cotranslational protein targeting to membrane
GO:0006613	6.77765453932191e-34	40.6378718535469	1.23230226281918	30	106	cotranslational protein targeting to membrane
GO:0045047	6.77765453932191e-34	40.6378718535469	1.23230226281918	30	106	protein targeting to ER
GO:0072599	6.77765453932191e-34	40.6378718535469	1.23230226281918	30	106	establishment of protein localization to endoplasmic reticulum
GO:0006414	1.98171925672855e-32	39.0006626277925	1.22067676977372	29	105	translational elongation
GO:0070972	2.51687126500944e-32	35.0666996047431	1.37180817936475	30	118	protein localization to endoplasmic reticulum
GO:0000184	1.13652656807989e-30	32.9014388489209	1.38343367241021	29	119	nuclear-transcribed mRNA catabolic process, nonsense-mediated decay
GO:0006612	4.57834943264373e-30	26.2648677755175	1.77870043595599	31	153	protein targeting to membrane
GO:0019080	7.79920054782776e-29	25.4437657204456	1.75544944986506	30	151	viral genome expression
GO:0019083	7.79920054782776e-29	25.4437657204456	1.75544944986506	30	151	viral transcription
GO:0006413	9.62642535139002e-29	25.2334283677833	1.76707494291053	30	152	translational initiation
GO:0072594	2.63344945567504e-27	19.3036935704514	2.37160058127465	32	204	establishment of protein localization to organelle
GO:0000956	2.74432847515856e-27	22.1207381920551	1.96470832468341	30	169	nuclear-transcribed mRNA catabolic process
GO:0043624	2.92947177317936e-27	23.8228707356695	1.77870043595599	29	153	cellular protein complex disassembly
GO:0043241	7.81569614070746e-27	22.891417098879	1.83682790118331	29	158	protein complex disassembly
GO:0006402	1.38563776211798e-26	20.7623384253819	2.06933776209259	30	178	mRNA catabolic process
GO:0034623	1.44028660172029e-25	20.3424460431655	2.02283578991073	29	174	cellular macromolecular complex disassembly
GO:0032984	3.3615600053829e-25	19.6574100719424	2.08096325513805	29	179	macromolecular complex disassembly
GO:0022415	4.36523592449143e-25	15.1361722956437	3.02262819182064	33	260	viral reproductive process
GO:0006401	1.06582774776324e-24	17.5254658385093	2.38322607432012	30	205	RNA catabolic process
GO:0019058	2.47279703702292e-24	15.9333211678832	2.68548889350218	31	231	viral infectious cycle
GO:0016032	2.38073510689295e-22	9.81672564723412	5.24309736350426	38	451	viral reproduction
GO:0071845	1.36745321225318e-21	12.5481088254811	3.30164002491177	31	284	cellular component disassembly at cellular level
GO:0022411	2.08232852202989e-21	12.3492856939987	3.34814199709363	31	288	cellular component disassembly
GO:0033365	1.19041863769823e-20	8.9701484697808	5.49885821050446	37	473	protein localization to organelle
GO:0006605	1.27947337289121e-20	8.94897549216553	5.51048370354993	37	474	protein targeting
GO:0022613	2.25613810346652e-06	5.56673767498478	2.6157359352294	13	225	ribonucleoprotein complex biogenesis
GO:0071843	4.3887913331541e-06	5.21669997145304	2.77849283786589	13	239	cellular component biogenesis at cellular level

Tissue: Muscle_Skeletal=>Adipose_Subcutaneous Type: asymmetric

SourceGene: RP11-809N8.5(ENSG00000256148.1)
TargetGeneSet: MTND1P23 MTND2P28 DFFA HSPG2 WASF2 THRAP3 MFI2
SZT2 RPS8 RPL5 RP11 ATP5F1 RPS27 ASH1L PRRC2C CEP350 CICP13
CDC42BPA ADAM18 NBAS ASXL2 AAK1 DCTN1 EIF5B RANBP2 UGGT1
MZT2B ANKRD44 FAM126B NBEAL1 COL4A4 NR2C2 RPL15 MAP4 USP4 RAD54L2
RPL29 PCNP PHC3 DNAJC19 HTT BOD1L RELL1 DANCR CISD2 RPL34
RPS3A NIPBL CTD ERAP1 MAN2A1 AFF4 CTB MTND5P11 RPS14
TRIM41 RPL15P3 DDX39B ZFAND3 MTRNR2L9 WDR11 PHACTR2 IGF2R FOXK1
HNRNPA1P8 RPS3AP26 TNPO3 MLL3 DRP2 HOOK3 RPS20 ARFGEF1 PAG1
RBM12B SETX RPL7A SEC16A PRINS FUT11 KIAA0913 GBF1 CD151
RPL27A MTRNR2L8 CKAP5 C1QTNF4 DDB1 PPP6R3 UBE4A ATP5L DDX6
KDM5A ERC1 C12orf57 RECQL SCAF11 MLL4 PFDN5 TMBIM4 SBNO1
GOLGA3 ZC3H13 COL4A2 COL4A2-AS1 CCNB1IP1 TEP1 C14orf21
STRN3 NIN YLPM1 SEL1L CCNK CDC42BPB BAHD1 MYO9A SEC11A NME3
NDUFB10 TSC2 RPS15A CDYL2 RPL13 GAS8 VPS53 WDR81 ZZEF1 RPL26
TOM1L2 SUPT6H RPL23A TAOK1 CDK12 NKIRAS2 RPS7P11 CLTC SUMO2 FN3KRP
ROCK1P1 ANKRD12 HNRNPA1P10 BRD4 RPSAP58 RPL18 RPL13A ZNF587 REM1
KIF3B RALGAPB NCOA3 RPL12P4 UBE2G2 HMGB1P10 MPST EIF3L USP9X
KDM5C HUWE1 GNL3L RPS4X COX7B RPL39 HCFC1
GOBPID Pvalue OddsRatio ExpCount Count Size Term
GO:0006614 4.92329849393982e-22 33.185789167152 0.879177911563214 20
105 SRP-dependent cotranslational protein targeting to membrane
GO:0006613 6.02762935647183e-22 32.7976053419295 0.887551034530482
20 106 cotranslational protein targeting to membrane
GO:0045047 6.02762935647183e-22 32.7976053419295 0.887551034530482
20 106 protein targeting to ER
GO:0072599 6.02762935647183e-22 32.7976053419295 0.887551034530482
20 106 establishment of protein localization to endoplasmic reticulum
GO:0006415 8.71361094645675e-22 36.8875272331155 0.761954190021452
19 91 translational termination
GO:0070972 5.84034084046466e-21 28.757324712063 0.988028510137707 20
118 protein localization to endoplasmic reticulum
GO:0006414 1.60244186277121e-20 30.8522571819425 0.879177911563214
19 105 translational elongation
GO:0006413 4.88828125182033e-20 22.7617557251908 1.27271469102484
21 152 translational initiation
GO:0000184 1.95056941221635e-19 26.506862745098 0.996401633104975 19
119 nuclear-transcribed mRNA catabolic process, nonsense-mediated decay
GO:0006612 1.25287033764925e-18 21.1374972083675 1.28108781399211
20 153 protein targeting to membrane
GO:0000956 9.3458581385066e-18 18.8464349790684 1.41505778146841
20 169 nuclear-transcribed mRNA catabolic process
GO:0019080 2.02829989637344e-17 20.0357991681521 1.26434156805757
19 151 viral genome expression
GO:0019083 2.02829989637344e-17 20.0357991681521 1.26434156805757
19 151 viral transcription
GO:0072594 2.44165470363414e-17 16.234262295082 1.70811708532281 21
204 establishment of protein localization to organelle
GO:0043624 2.61108758813079e-17 19.7339771729587 1.28108781399211
19 153 cellular protein complex disassembly
GO:0006402 2.64029367412081e-17 17.7616242636922 1.49041588817383
20 178 mRNA catabolic process
GO:0043241 4.83217148446209e-17 19.017421357032 1.32295342882845 19
158 protein complex disassembly
GO:0034623 3.01775072241743e-16 17.0351043643264 1.45692339630475
19 174 cellular macromolecular complex disassembly
GO:0006401 4.3132110232671e-16 15.1404870216751 1.71649020829008
20 205 RNA catabolic process
GO:0032984 5.14684970725644e-16 16.4969362745098 1.4987890111411 19
179 macromolecular complex disassembly
GO:0033365 1.52662954937172e-15 8.94160862513119 3.9604871635181 27
473 protein localization to organelle
GO:0022415 3.46075199164941e-15 12.3812133891213 2.17701197148986

21	260	viral reproductive process
GO:0006605	1.42515940146057e-14	8.48054511278196	3.96886028648536
26	474	protein targeting
GO:0019058	5.78076611768289e-14	12.4048279689234	1.93419140543907
19	231	viral infectious cycle
GO:0071845	2.25182775650332e-13	10.5505550555056	2.37796692270431
20	284	cellular component disassembly at cellular level
GO:0022411	2.9273467923705e-13	10.3901285650953	2.41145941457339
20	288	cellular component disassembly
GO:0016032	2.00252277318165e-11	7.2007252007252 3.77627845823818	22
451	viral reproduction
GO:0022613	1.92112304952327e-05	5.91451917033312	1.88395266763546
10	225	ribonucleoprotein complex biogenesis
Tissue: Muscle_Skeletal=>Adipose_Subcutaneous	Type:	cluster
SourceGene:	RP11-809N8.5(ENSG00000256148.1)
TargetGeneSet:	MTND1P23	MTND2P28	DFFA	HSPG2	WASF2	THRAP3	MFI2
SZT2	RPS8	RPL5	RP11	ATP5F1	RPS27	ASH1L	PRRC2C	CEP350	CICP13
CDC42BPA	ADAM18	NBAS	ASXL2	AAK1	DCTN1	EIF5B	RANBP2	UGGT1
MZT2B	ANKRD44	FAM126B	NBEAL1	COL4A4	NR2C2	RPL15	MAP4	USP4	RAD54L2
RPL29	PCNP	PHC3	DNAJC19	HTT	BOD1L	RELL1	DANCR	CISD2	RPL34
RPS3A	NIPBL	CTD	ERAP1	MAN2A1	AFF4	CTB	MTND5P11	RPS14
TRIM41	RPL15P3	DDX39B	ZFAND3	MTRNR2L9	WDR11	PHACTR2	IGF2R	FOXK1
HNRNPA1P8	RPS3AP26	TNPO3	MLL3	DRP2	HOOK3	RPS20	ARFGEF1	PAG1
RBM12B	SETX	RPL7A	SEC16A	PRINS	FUT11	KIAA0913	GBF1	CD151
RPL27A	MTRNR2L8	CKAP5	C1QTNF4	DDB1	PPP6R3	UBE4A	ATP5L	DDX6
KDM5A	ERC1	C12orf57	RECQL	SCAF11	MLL4	PFDN5	TMBIM4	SBNO1
GOLGA3	ZC3H13	COL4A2	COL4A2-AS1	CCNB1IP1	TEP1	C14orf21
STRN3	NIN	YLPM1	SEL1L	CCNK	CDC42BPB	BAHD1	MYO9A	SEC11A	NME3
NDUFB10	TSC2	RPS15A	CDYL2	RPL13	GAS8	VPS53	WDR81	ZZEF1	RPL26
TOM1L2	SUPT6H	RPL23A	TAOK1	CDK12	NKIRAS2	RPS7P11	CLTC	SUMO2	FN3KRP
ROCK1P1	ANKRD12	HNRNPA1P10	BRD4	RPSAP58	RPL18	RPL13A	ZNF587	REM1
KIF3B	RALGAPB	NCOA3	RPL12P4	UBE2G2	HMGB1P10	MPST	EIF3L	USP9X
KDM5C	HUWE1	GNL3L	RPS4X	COX7B	RPL39	HCFC1
GOBPID	Pvalue	OddsRatio	ExpCount	Count	Size	Term
GO:0006614	4.92329849393982e-22	33.185789167152 0.879177911563214	20
105	SRP-dependent cotranslational protein targeting to membrane
GO:0006613	6.02762935647183e-22	32.7976053419295	0.887551034530482
20	106	cotranslational protein targeting to membrane
GO:0045047	6.02762935647183e-22	32.7976053419295	0.887551034530482
20	106	protein targeting to ER
GO:0072599	6.02762935647183e-22	32.7976053419295	0.887551034530482
20	106	establishment of protein localization to endoplasmic reticulum
GO:0006415	8.71361094645675e-22	36.8875272331155	0.761954190021452
19	91	translational termination
GO:0070972	5.84034084046466e-21	28.757324712063 0.988028510137707	20
118	protein localization to endoplasmic reticulum
GO:0006414	1.60244186277121e-20	30.8522571819425	0.879177911563214
19	105	translational elongation
GO:0006413	4.88828125182033e-20	22.7617557251908	1.27271469102484
21	152	translational initiation
GO:0000184	1.95056941221635e-19	26.506862745098 0.996401633104975	19
119	nuclear-transcribed mRNA catabolic process, nonsense-mediated decay
GO:0006612	1.25287033764925e-18	21.1374972083675	1.28108781399211
20	153	protein targeting to membrane
GO:0000956	9.3458581385066e-18	18.8464349790684	1.41505778146841
20	169	nuclear-transcribed mRNA catabolic process
GO:0019080	2.02829989637344e-17	20.0357991681521	1.26434156805757
19	151	viral genome expression
GO:0019083	2.02829989637344e-17	20.0357991681521	1.26434156805757
19	151	viral transcription
GO:0072594	2.44165470363414e-17	16.234262295082 1.70811708532281	21
204	establishment of protein localization to organelle
GO:0043624	2.61108758813079e-17	19.7339771729587	1.28108781399211

19	153	cellular protein complex disassembly
GO:0006402	2.64029367412081e-17	17.7616242636922	1.49041588817383
20	178	mRNA catabolic process
GO:0043241	4.83217148446209e-17	19.017421357032 1.32295342882845	19
158	protein complex disassembly
GO:0034623	3.01775072241743e-16	17.0351043643264	1.45692339630475
19	174	cellular macromolecular complex disassembly
GO:0006401	4.3132110232671e-16	15.1404870216751	1.7164902082900 8
20	205	RNA catabolic process
GO:0032984	5.14684970725644e-16	16.4969362745098	1.4987890111411 19
179	macromolecular complex disassembly
GO:0033365	1.52662954937172e-15	8.94160862513119	3.9604871635181 27
473	protein localization to organelle
GO:0022415	3.46075199164941e-15	12.3812133891213	2.17701197148986
21	260	viral reproductive process
GO:0006605	1.42515940146057e-14	8.48054511278196	3.96886028648536
26	474	protein targeting
GO:0019058	5.78076611768289e-14	12.4048279689234	1.93419140543907
19	231	viral infectious cycle
GO:0071845	2.25182775650332e-13	10.5505550555056	2.37796692270431
20	284	cellular component disassembly at cellular level
GO:0022411	2.9273467923705e-13	10.3901285650953	2.41145941457339
20	288	cellular component disassembly
GO:0016032	2.00252277318165e-11	7.2007252007252 3.77627845823818	22
451	viral reproduction
GO:0022613	1.92112304952327e-05	5.91451917033312	1.88395266763546
10	225	ribonucleoprotein complex biogenesis
Tissue: Adipose_Subcutaneous=>Nerve_Tibial	Type:	asymmetric
SourceGene:	ALDH1B1(ENSG00000137124.5)
TargetGeneSet:	RPL11	RPS27	PKLR	GAS5	RPL14	CADM2-AS1	RP11
SCRG1	RPS10	RPS20	RPL30	RPL12	RPL7A	PCBD1	RPL6	RPL7AP6 SNRPN
GOLGA6L4	RPL26	ACACA	RPL19	NETO1	HPN	NKG7	RPL28	LSS
EIF3L	GPC4	RPL10
GOBPID	Pvalue	OddsRatio	ExpCount	Count	Size	Term
GO:0006415	2.36525599733211e-23	217.409090909091	0.151131409591032
13	91	translational termination
GO:0006414	1.70532207841082e-22	184.145256916996	0.17438239568196
13	105	translational elongation
GO:0006614	1.70532207841082e-22	184.145256916996	0.17438239568196
13	105	SRP-dependent cotranslational protein targeting to membrane
GO:0019080	1.79407676843101e-22	146.029197080292	0.250778492837866
14	151	viral genome expression
GO:0019083	1.79407676843101e-22	146.029197080292	0.250778492837866
14	151	viral transcription
GO:0006613	1.94231758806374e-22	182.152492668622	0.17604318040274
13	106	cotranslational protein targeting to membrane
GO:0045047	1.94231758806374e-22	182.152492668622	0.17604318040274
13	106	protein targeting to ER
GO:0072599	1.94231758806374e-22	182.152492668622	0.17604318040274
13	106	establishment of protein localization to endoplasmic reticulum
GO:0006413	1.97479609705954e-22	144.960869565217	0.252439277558646
14	152	translational initiation
GO:0070972	8.41874403674507e-22	161.2	0.195972597052107	13	118
protein localization to endoplasmic reticulum
GO:0000184	9.44450207397793e-22	159.668096054889	0.197633381772888
13	119	nuclear-transcribed mRNA catabolic process, nonsense-mediated decay
GO:0006612	2.82503658286128e-20	120.604545454545	0.254100062279427
13	153	protein targeting to membrane
GO:0043624	2.82503658286128e-20	120.604545454545	0.254100062279427
13	153	cellular protein complex disassembly
GO:0043241	4.34944305560692e-20	116.405015673981	0.26240398588333
13	158	protein complex disassembly
GO:0019058	8.15435952605171e-20	91.6774193548387	0.383641270500311

14	231	viral infectious cycle
GO:0000956	1.07082145951018e-19	108.113636363636	0.280672617811916
13	169	nuclear-transcribed mRNA catabolic process
GO:0034623	1.58064540448204e-19	104.719367588933	0.288976541415819
13	174	cellular macromolecular complex disassembly
GO:0006402	2.14045600967934e-19	102.152066115702	0.295619680298941
13	178	mRNA catabolic process
GO:0032984	2.30643262079744e-19	101.529572836802	0.297280465019722
13	179	macromolecular complex disassembly
GO:0022415	4.38531556145992e-19	80.7048780487805	0.431804027402948
14	260	viral reproductive process
GO:0072594	1.3107907802754e-18	88.0856734888148	0.338800083039236
13	204	establishment of protein localization to organelle
GO:0006401	1.39854045986023e-18	87.6207386363636	0.340460867760017
13	205	RNA catabolic process
GO:0071845	1.53203446987607e-18	73.4066666666667	0.471662860701682
14	284	cellular component disassembly at cellular level
GO:0022411	1.86706634090778e-18	72.314598540146 0.478305999584804	14
288	cellular component disassembly
GO:0016032	1.00514907255067e-15	44.8192219679634	0.749013909072037
14	451	viral reproduction
GO:0033365	7.55931581953024e-14	35.8835968379447	0.785551172929209
13	473	protein localization to organelle
GO:0006605	7.76682818723057e-14	35.8031946361664	0.78721195764999
13	474	protein targeting
GO:0042273	8.76835229964862e-07	228.857142857143	0.0199294166493668
3	12	ribosomal large subunit biogenesis
Tissue: Adipose_Subcutaneous=>Nerve_Tibial	Type:	asymmetric
SourceGene:	CNN1(ENSG00000130176.2)
TargetGeneSet:	RPL11	RP11	RPS27	BCL11A	RPL14	EPB41L4A	FAM135A
MRPS12	RPS20	OTUD6B	RPL30	TATDN1	KLC2	VWA5A	CTD	ALDH3A1 ACACA
RPL17	RPL19	RSL24D1P11	UBA52	RPL28	PCBP3
GOBPID	Pvalue	OddsRatio	ExpCount	Count	Size	Term
GO:0006415	2.42067264909835e-16	196.90243902439 0.107051415126981	9
91	translational termination
GO:0006414	9.216208669879e-16	168.0234375	0.123520863608055	9
105	translational elongation
GO:0006614	9.216208669879e-16	168.0234375	0.123520863608055	9
105	SRP-dependent cotranslational protein targeting to membrane
GO:0006613	1.00662717500152e-15	166.279639175258	0.124697252785274
9	106	cotranslational protein targeting to membrane
GO:0045047	1.00662717500152e-15	166.279639175258	0.124697252785274
9	106	protein targeting to ER
GO:0072599	1.00662717500152e-15	166.279639175258	0.124697252785274
9	106	establishment of protein localization to endoplasmic reticulum
GO:0070972	2.72424673952409e-15	147.849770642202	0.138813922911909
9	118	protein localization to endoplasmic reticulum
GO:0000184	2.94566104471822e-15	146.495454545455	0.139990312089129
9	119	nuclear-transcribed mRNA catabolic process, nonsense-mediated decay
GO:0019080	2.64338118996e-14	113.228873239437	0.177634765760155
9	151	viral genome expression
GO:0019083	2.64338118996e-14	113.228873239437	0.177634765760155
9	151	viral transcription
GO:0006413	2.80833544815286e-14	112.429195804196	0.178811154937375
9	152	translational initiation
GO:0006612	2.98235641610268e-14	111.640625	0.179987544114594	9
153	protein targeting to membrane
GO:0043624	2.98235641610268e-14	111.640625	0.179987544114594	9
153	cellular protein complex disassembly
GO:0043241	4.00419344287656e-14	107.856543624161	0.185869490000692
9	158	protein complex disassembly
GO:0000956	7.41056323208651e-14	100.3640625	0.198809770950107	9
169	nuclear-transcribed mRNA catabolic process

GO:0034623 9.67098404549803e-14 97.2886363636364 0.204691716836205
9 174 cellular macromolecular complex disassembly
GO:0006402 1.1899244880822e-13 94.9593195266272 0.209397273545083
9 178 mRNA catabolic process
GO:0032984 1.25228968636938e-13 94.3941176470588 0.210573662722303
9 179 macromolecular complex disassembly
GO:0072594 4.11396147908514e-13 82.1480769230769 0.239983392152792
9 204 establishment of protein localization to organelle
GO:0006401 4.3006982319074e-13 81.7232142857143 0.241159781330012
9 205 RNA catabolic process
GO:0019058 1.26875360582069e-12 72.0202702702703 0.271745899937721
9 231 viral infectious cycle
GO:0022415 3.68969869097354e-12 63.5692231075697 0.305861186077088
9 260 viral reproductive process
GO:0071845 8.16598085479159e-12 57.9231818181818 0.334094526330358
9 284 cellular component disassembly at cellular level
GO:0022411 9.25924200927783e-12 57.0766129032258 0.338800083039236
9 288 cellular component disassembly
GO:0016032 5.05270111811315e-10 35.6131221719457 0.530551518926026
9 451 viral reproduction
GO:0033365 7.69883716810493e-10 33.8712284482759 0.556432080824856
9 473 protein localization to organelle
GO:0006605 7.84382676642097e-10 33.7959677419355 0.557608470002076
9 474 protein targeting
Tissue: Nerve_Tibial=>Adipose_Subcutaneous Type: asymmetric
SourceGene: ENSG00000231312.2
TargetGeneSet: AGRN RPL11 MED8 PRPF38A STXBP3 RP11 PFDN2 TOMM20
NRBP1 UBC RPL37A DNPEP RPL32 RPL14 SS18L2 RTP3 RPL24 RPL35A
MRPS30 BTF3 TBCA HDAC3 MRPL22 TDP2 COX11P1 LY6G6C LYPLA2P1
MRPS12 PSMB1 DGKB RP1 TMEM68 RPS20 RPL30 LURAP1L RPS6 CCDC107
PSMB7 RPL35 RPL12 WDR5 EIF3M POLR2G LTBP2 OVOL1 RPS25 KCNA5
RPL41 NUP37 C12orf73 RFXAP MED6 DEGS2 NOP10 CDAN1 PARP16
RPL35AP32 AAGAB RPS17L NTRK3 PDIA2 CCP110 LCMT1 ZFHX3 AATF
RPL17 RPL19 KRT23 DNAJC7 UTP18 PTPN2 CASP14 SBSN KLK7 LINC00493
U2AF1 COL18A1 MTMR3 PLA2G3 DRG1 NHP2L1 RPS4X IGSF1 MOSPD1
GOBPID Pvalue OddsRatio ExpCount Count Size Term
GO:0006415 7.30962476000486e-25 67.8461538461538 0.440799944640509
18 91 translational termination
GO:0070972 2.11551730701453e-24 53.7448999801941 0.571586741401979
19 118 protein localization to endoplasmic reticulum
GO:0006414 1.18173310704828e-23 56.8726790450928 0.508615320739049
18 105 translational elongation
GO:0006614 1.18173310704828e-23 56.8726790450928 0.508615320739049
18 105 SRP-dependent cotranslational protein targeting to membrane
GO:0006613 1.4185688397062e-23 56.222465034965 0.513459276174659 18
106 cotranslational protein targeting to membrane
GO:0045047 1.4185688397062e-23 56.222465034965 0.513459276174659 18
106 protein targeting to ER
GO:0072599 1.4185688397062e-23 56.222465034965 0.513459276174659 18
106 establishment of protein localization to endoplasmic reticulum
GO:0000184 1.29820820228028e-22 48.9413556740289 0.576430696837589
18 119 nuclear-transcribed mRNA catabolic process, nonsense-mediated decay
GO:0019080 2.90784919987631e-22 40.2155377302436 0.731437270777109
19 151 viral genome expression
GO:0019083 2.90784919987631e-22 40.2155377302436 0.731437270777109
19 151 viral transcription
GO:0006413 3.31201462109142e-22 39.9103641456583 0.736281226212719
19 152 translational initiation
GO:0006612 3.76883575150336e-22 39.6097453906936 0.741125181648329
19 153 protein targeting to membrane
GO:0043624 1.45617067757464e-20 36.5282051282051 0.741125181648329
18 153 cellular protein complex disassembly
GO:0043241 2.64230363399189e-20 35.2112637362637 0.765344958826379

18	158	protein complex disassembly
GO:0000956	9.14426211627071e-20	32.6209882832399	0.818628468618089
18	169	nuclear-transcribed mRNA catabolic process
GO:0072594	1.01281337619908e-19	28.58759936407	0.988166908864438	19
204	establishment of protein localization to organelle
GO:0034623	1.56212869138555e-19	31.564349112426	0.842848245796139	18
174	cellular macromolecular complex disassembly
GO:0006402	2.36916065429315e-19	30.7665865384615	0.862224067538579
18	178	mRNA catabolic process
GO:0032984	2.6249639709827e-19	30.5733397037745	0.867068022974189
18	179	macromolecular complex disassembly
GO:0022415	4.40429713301696e-19	23.5683333333333	1.2594284132586	20
260	viral reproductive process
GO:0019058	1.08752417944434e-18	24.8992785793563	1.11895370562591
19	231	viral infectious cycle
GO:0006401	3.09399941062075e-18	26.2743726861374	0.993010864300048
18	205	RNA catabolic process
GO:0016032	6.20298111973668e-18	15.9534201630543	2.18462390146011
23	451	viral reproduction
GO:0071845	1.03446605192964e-15	18.3682764603817	1.37568334371324
18	284	cellular component disassembly at cellular level
GO:0022411	1.32254325251912e-15	18.0910256410256	1.39505916545568
18	288	cellular component disassembly
GO:0006605	3.8290047218012e-15	13.1769157994324	2.29603487647914
21	474	protein targeting
GO:0033365	4.76779668040605e-14	12.2984547461369	2.29119092104353
20	473	protein localization to organelle
GO:0042254	2.34275810998796e-09	17.0767386091127	0.721749359905889
10	149	ribosome biogenesis
GO:0022613	9.86397295395444e-09	12.3425471249802	1.08988997301225
11	225	ribonucleoprotein complex biogenesis
GO:0071843	1.84432686540542e-08	11.5732233125186	1.15770534911079
11	239	cellular component biogenesis at cellular level
GO:0006364	6.74004146267171e-07	16.3619702176403	0.503771365303439
7	104	rRNA processing
GO:0016072	9.86739548870654e-07	15.4023732470334	0.532835097917099
7	110	rRNA metabolic process
GO:0000028	2.15448756952043e-06	214.597014925373	0.02906373261366
3	6	ribosomal small subunit assembly
Tissue: Nerve_Tibial=>Adipose_Subcutaneous	Type:	asymmetric
SourceGene:	ENSG00000233702.1
TargetGeneSet:	RPL22	DLEU2L	DPYD	RTCD1	GNRHR2	RP11	RPL37A	RPL32
PTPN23	SLC52A1	PVRL3-AS1	RPL35A	NKX3-2	EEF1A1P19	RPS14	EEF1A1
HOXA13	PEG10	AHCYL2	RPS20	RPL30	TATDN1	NELL1	RPS13	WNK1	ATN1
PRPF40B	RPL41	RPL14P1	CAMTA2	CEP95	SMARCA4	ARHGEF1	SNRPD2	RPS11	PI4KA
TMEM184B	UBA1
GOBPID	Pvalue	OddsRatio	ExpCount	Count	Size	Term
GO:0006414	8.48006399289468e-17	88.2402015677492	0.21797799460245
11	105	translational elongation
GO:0006415	1.59771081355511e-15	88.5185185185185	0.18891426198879
10	91	translational termination
GO:0019080	5.16753494456863e-15	59.0567669172932	0.313473116047332
11	151	viral genome expression
GO:0019083	5.16753494456863e-15	59.0567669172932	0.313473116047332
11	151	viral transcription
GO:0006614	7.04569488407382e-15	75.4	0.21797799460245	10	105
SRP-dependent cotranslational protein targeting to membrane
GO:0006613	7.76977716994345e-15	74.609375	0.220053975503425	10
106	cotranslational protein targeting to membrane
GO:0045047	7.76977716994345e-15	74.609375	0.220053975503425	10
106	protein targeting to ER
GO:0072599	7.76977716994345e-15	74.609375	0.220053975503425	10
106	establishment of protein localization to endoplasmic reticulum

GO:0070972	2.34128555621209e-14	66.2638888888889	0.244965746315134
10	118	protein localization to endoplasmic reticulum
GO:0034623	2.51352674312269e-14	50.6419115272845	0.361220676769774
11	174	cellular macromolecular complex disassembly
GO:0000184	2.55284572541155e-14	65.651376146789	0.24704172721611	10
119	nuclear-transcribed mRNA catabolic process, nonsense-mediated decay
GO:0032984	3.44329372861052e-14	49.1174812030075	0.371600581274652
11	179	macromolecular complex disassembly
GO:0006413	3.08501908715246e-13	50.2781690140845	0.315549096948308
10	152	translational initiation
GO:0006612	3.29656753823955e-13	49.9230769230769	0.317625077849284
10	153	protein targeting to membrane
GO:0043624	3.29656753823955e-13	49.9230769230769	0.317625077849284
10	153	cellular protein complex disassembly
GO:0043241	4.56222333399732e-13	48.2195945945946	0.328004982354162
10	158	protein complex disassembly
GO:0019058	5.73980054119701e-13	37.3710526315789	0.479551588125389
11	231	viral infectious cycle
GO:0000956	8.99047406816903e-13	44.8490566037736	0.350840772264895
10	169	nuclear-transcribed mRNA catabolic process
GO:0006402	1.51432237195242e-12	42.4196428571429	0.369524600373677
10	178	mRNA catabolic process
GO:0022415	2.09096768632073e-12	32.9511731135067	0.539755034253685
11	260	viral reproductive process
GO:0071845	5.46196938724066e-12	30.0034702139965	0.589578575877102
11	284	cellular component disassembly at cellular level
GO:0072594	5.92154903042295e-12	36.6675257731959	0.423500103799045
10	204	establishment of protein localization to organelle
GO:0006401	6.21729949789748e-12	36.4769230769231	0.425576084700021
10	205	RNA catabolic process
GO:0022411	6.35672814117802e-12	29.5618468554057	0.597882499481005
11	288	cellular component disassembly
GO:0016032	7.74981439820864e-10	18.3960526315789	0.936267386340046
11	451	viral reproduction
GO:0033365	2.14146811280286e-08	15.07343412527	0.981938966161511	10
473	protein localization to organelle
GO:0006605	2.18479789659218e-08	15.0398706896552	0.984014947062487
10	474	protein targeting
Tissue: Nerve_Tibial=>Adipose_Subcutaneous	Type:	asymmetric
SourceGene:	FAM179A(ENSG00000189350.7)
TargetGeneSet:	RPL22	RPL11	WDR65	RPS8	RPL5	RP11	SSR2	NENF	MYCN
FAM179A	RPLP0P6	UBC	RPL31	CLASP1	LIMS2	MAP2	SPAG16	RPL37A	RPL32
RPL15	RPL14	ZNF90P1	RPL24	PVRL3-AS1	RPL35A	MSANTD1	RPL34	EEF1A1P19
CTD	BTF3	RPS14	CCNG1	TMEM14C	RPS10	RPL10A	EEF1A1	MRPS12	TNRC18
HOXA13	RABL5	PPAPDC1B	RPS20	RPL30	EIF3H	CPSF1	RABGAP1	RPL7A
RPS24	C10orf32	PNLIPRP3	OR51C1P	RPS13	RIMKLB	PFDN5	RPL41	NACA
RPL6	TPT1	SLC25A21	CNIH	TIMM9	RSL24D1	RPL4	SKOR1	RPS15A
RPL23A	RPL17	RPL19	ROCK1	ELP2	SMARCA4	NOTCH3	RPL18A	ARHGEF1	SNRPD2
RPL18	RPS11	C20orf96	EBF4	LINC00493	RP4	DRG1	EIF3L
RPS4X	RPSAP9	RPL10
GOBPID	Pvalue	OddsRatio	ExpCount	Count	Size	Term
GO:0006414	1.6459967607858e-57	188.263157894737	0.530413120199294
35	105	translational elongation
GO:0006415	1.70215030519587e-55	203.689655172414	0.459691370839388
33	91	translational termination
GO:0006614	2.97791640187156e-55	175.672806067172	0.530413120199294
34	105	SRP-dependent cotranslational protein targeting to membrane
GO:0006613	4.37258017281276e-55	173.220797720798	0.535464673725002
34	106	cotranslational protein targeting to membrane
GO:0045047	4.37258017281276e-55	173.220797720798	0.535464673725002
34	106	protein targeting to ER
GO:0072599	4.37258017281276e-55	173.220797720798	0.535464673725002
34	106	establishment of protein localization to endoplasmic reticulum

GO:0070972	3.19846385812202e-53	148.350427350427	0.596083316033493
34	118	protein localization to endoplasmic reticulum
GO:0006413	5.33433800978301e-51	112.266081871345	0.76783613590755
35	152	translational initiation
GO:0000184	6.31192773072493e-51	137.103488372093	0.6011348695592 33
119	nuclear-transcribed mRNA catabolic process, nonsense-mediated decay
GO:0006612	6.8986947491144e-51	111.306868867083	0.772887689433257
35	153	protein targeting to membrane
GO:0019080	4.50595972262906e-49	106.262108262108	0.762784582381842
34	151	viral genome expression
GO:0019083	4.50595972262906e-49	106.262108262108	0.762784582381842
34	151	viral transcription
GO:0043624	7.42318269568848e-49	104.461538461538	0.772887689433257
34	153	cellular protein complex disassembly
GO:0034623	1.01940428041778e-48	94.3515713744794	0.878970313473116
35	174	cellular macromolecular complex disassembly
GO:0043241	2.50444397395419e-48	100.214226633581	0.798145457061795
34	158	protein complex disassembly
GO:0032984	3.0282059262799e-48	91.0434941520468	0.904228081101654
35	179	macromolecular complex disassembly
GO:0072594	4.38413104884357e-46	77.4392712550607	1.03051691924434
35	204	establishment of protein localization to organelle
GO:0000956	2.7940988881495e-45	86.3944852941176	0.853712545844578
33	169	nuclear-transcribed mRNA catabolic process
GO:0006402	1.81051505713781e-44	80.9808620689655	0.899176527575946
33	178	mRNA catabolic process
GO:0071845	1.75932632861188e-42	55.4359197907585	1.43464120130095
36	284	cellular component disassembly at cellular level
GO:0006401	2.76505748599724e-42	68.1392441860465	1.03556847277005
33	205	RNA catabolic process
GO:0022411	2.97990060333806e-42	54.5405405405405	1.45484741540378
36	288	cellular component disassembly
GO:0019058	3.00185284970202e-42	62.7559547051933	1.16690886443845
34	231	viral infectious cycle
GO:0022415	2.08782623158343e-40	54.5913319718629	1.31340391668397
34	260	viral reproductive process
GO:0033365	8.01380570191847e-33	29.3138668589281	2.38938481765968
35	473	protein localization to organelle
GO:0006605	8.62989115072256e-33	29.2449946049634	2.39443637118538
35	474	protein targeting
GO:0016032	4.52561273743294e-32	29.1873578060628	2.27825064009411
34	451	viral reproduction
GO:0022613	1.2057844796455e-09	13.0824290002309	1.1365995432842 12
225	ribonucleoprotein complex biogenesis
GO:0071843	2.39667406935383e-09	12.2634505669098	1.20732129264411
12	239	cellular component biogenesis at cellular level
GO:0042254	3.57386137050076e-09	16.2601347493434	0.752681475330427
10	149	ribosome biogenesis
GO:0042273	2.87680213437773e-07	104.130434782609	0.0606186423084908
4	12	ribosomal large subunit biogenesis
GO:0006364	9.00074231039749e-07	15.614964073727 0.525361566673587	7
104	rRNA processing
GO:0016072	1.31627386519054e-06	14.6991762283024	0.555670887827832
7	110	rRNA metabolic process
GO:0030490	4.26634606660256e-06	154.007142857143	0.0353608746799529
3	7	maturation of SSU-rRNA
Tissue: Adipose_Subcutaneous=>Nerve_Tibial	Type:	asymmetric
SourceGene:	GGPS1(ENSG00000152904.7)
TargetGeneSet:	PRDM16	RPL11	PABPC4	RPS8	RPL5	CCDC18	RP11	RP4
FAM20B	ARL8A	SCN9A	C2orf88	CFLAR-AS1	EEF1B2	CTDSPL	RPL14	CEP97
IGSF11	KPNA1	SNX4	EIF4G1	EIF4A2	TIGD2	ARFIP1	TBC1D9B	TRIM38	RPS10
RPL10A	FOXP4	PAQR8	L3MBTL3	MRPS12	SERAC1	BCAP29	RPS20	EEF1D	SCRIB	RPS6
GRHPR	NANS	TLR4	RPL7A	RPL13AP5	ACTR1A	RPLP2	RPL27A	CTD

PTPRJ EHD1 PPP2R5B RAB6A RPS3 LETMD1 ERBB3 RPL41 MARS RPL6 EFS
U6 TBC1D2B MIR1225 ROGDI RPS15A RPL26 RPL23A CCL18 RPL17 FAM171A2
KPNA2 FOXK2 TWSG1 TCF3 RPL36 ELAVL1 UBA52 RPS16 HIPK4 TOMM40
PPM1N RPL18 TTYH1 RPL28 ARFGAP3 FAM19A5 MOV10L1 GPC4

GOBPID	Pvalue	OddsRatio	ExpCount	Count	Size	Term
GO:0006414	5.08858156509752e-37	101.576704545455	0.501349387585634	25	105	translational elongation
GO:0006415	1.13805039329915e-34	105.25	0.434502802574216	23	91	translational termination
GO:0000184	1.38536212841634e-33	80.2077192982456	0.568195972597052	24	119	nuclear-transcribed mRNA catabolic process, nonsense-mediated decay
GO:0006614	4.54893549397025e-33	87.1951219512195	0.501349387585634	23	105	SRP-dependent cotranslational protein targeting to membrane
GO:0006613	5.79164416485982e-33	86.1385542168675	0.506124143657878	23	106	cotranslational protein targeting to membrane
GO:0045047	5.79164416485982e-33	86.1385542168675	0.506124143657878	23	106	protein targeting to ER
GO:0072599	5.79164416485982e-33	86.1385542168675	0.506124143657878	23	106	establishment of protein localization to endoplasmic reticulum
GO:0006413	1.27574770764564e-32	63.775053686471	0.725762922981109	25	152	translational initiation
GO:0070972	8.71259019175334e-32	75.1947368421053	0.563421216524808	23	118	protein localization to endoplasmic reticulum
GO:0000956	2.1436292027501e-31	56.1789772727273	0.806933776209259	25	169	nuclear-transcribed mRNA catabolic process
GO:0006401	7.258606590239e-31	47.9768741067949	0.978824994810048	26	205	RNA catabolic process
GO:0006402	8.43308013765557e-31	52.8409090909091	0.849906580859456	25	178	mRNA catabolic process
GO:0072594	2.98468906509292e-29	45.0831640426613	0.974050238737804	25	204	establishment of protein localization to organelle
GO:0019080	3.8827124741542e-29	55.6796875	0.720988166908864	23	151	viral genome expression
GO:0019083	3.8827124741542e-29	55.6796875	0.720988166908864	23	151	viral transcription
GO:0006612	5.35128619543073e-29	54.8153846153846	0.730537679053353	23	153	protein targeting to membrane
GO:0043624	5.35128619543073e-29	54.8153846153846	0.730537679053353	23	153	cellular protein complex disassembly
GO:0043241	1.16992369186893e-28	52.7666666666667	0.754411459414573	23	158	protein complex disassembly
GO:0034623	1.20400417720169e-27	47.1225165562914	0.83080755657048	23	174	cellular macromolecular complex disassembly
GO:0032984	2.37796437734916e-27	45.5961538461538	0.8546813369317	23	179	macromolecular complex disassembly
GO:0019058	2.82782745493207e-26	36.5217391304348	1.1029686526884	24	231	viral infectious cycle
GO:0022415	5.10160442095071e-25	31.9683615819209	1.24143657878348	24	260	viral reproductive process
GO:0071845	4.34119211381525e-24	28.9682051282051	1.35603072451733	24	284	cellular component disassembly at cellular level
GO:0033365	5.78442278102426e-24	21.3886544258701	2.25845962217148	28	473	protein localization to organelle
GO:0022411	6.08552141405383e-24	28.5212121212121	1.37512974880631	24	288	cellular component disassembly
GO:0006605	6.13020669156525e-24	21.3391665755223	2.26323437824372	28	474	protein targeting
GO:0016032	2.51014901109774e-19	17.4301327088212	2.15341498858211	24	451	viral reproduction
GO:0042273	2.2890049584565e-07	110.569230769231	0.0572970728669296	4	12	ribosomal large subunit biogenesis
GO:0042254	5.19842752494549e-07	13.2459016393443	0.711438654764376	8	149	ribosome biogenesis

GO:0006364	6.10181228854743e-07	16.6270369138676	0.49657463151339
7	104	rRNA processing
GO:0016072	8.93626842774286e-07	15.651894769809 0.525223167946855	7
110	rRNA metabolic process
GO:0022613	1.15438184485564e-06	9.8375	1.07432011625493	9	225
ribonucleoprotein complex biogenesis
GO:0071843	1.90358788834925e-06	9.2295652173913 1.14116670126635	9
239	cellular component biogenesis at cellular level
Tissue: Nerve_Tibial=>Adipose_Subcutaneous	Type:	asymmetric
SourceGene:	HIVEP1(ENSG00000095951.11)
TargetGeneSet:	RP3	RP1	RPS8	BTF3L4	WDR78	RP11	RP4	GNG5	RPL5
RWDD3	STXBP3	HSD3BP4 ATF6	PRRC2C	MRPL55	RPS7	CALM2	UBC	COA5
TXNDC9	MMADHC	MTX2	SENP2	HIGD1A	SEMA3G	PCNP	RPL24	YWHAQP6 RNF7
PDCD10	DNAJC19 RELL1	COPS4	LAMTOR3 AIMP1	OSTC	ANAPC10 CMBL	SUB1	CDK7
ANKRA2	CETN3	ERAP1	CTD	MATR3	CTB	NPM1	RPL26L1 U1	NOL7
GPLD1	EYS	IGF2R	MALSU1	CBX3	BET1	BUD31	SPDYE2	HIPK2	ASPH
HEATR7A CHMP5	ATP6V1G1	RPL12	SSNA1	COMMD3	RPS24	MARK2P9 SBF2
RPS13	TSG101	POLR2G	PPP6R3	UCP2	UBE4A	NTM	PFDN5	ACTR6	C12orf45
TRIAP1	STARD13 RFXAP	MRPS31	DGKH	EBPL	PPP2R3C FKBP3	NEK9	C14orf80
CYFIP1	SNAP23	TTBK2	RPS3AP6 PPIB	RAB11A	RPS17L	CHTF8	HSBP1	DERL2
RPL26	NCOR1	PRPSAP2 RPS7P1	ERN1	C17orf90	MYL12A	MYL12B	PSMG2
C18orf32	RPS19	MEGF8	RPL18	RPS5	UBE2V1	PFDN4	RPL12P4 PI4KA
GSTT2	RPS19BP1	GNL3L	RPS4X	PSMD10	DOCK11	SSR4
GOBPID	Pvalue	OddsRatio	ExpCount	Count	Size	Term
GO:0006614	5.31853245541676e-18	32.8850475367329	0.682997716421009
16	105	SRP-dependent cotranslational protein targeting to membrane
GO:0006613	6.23205818299822e-18	32.5173789173789	0.6895024565774 16
106	cotranslational protein targeting to membrane
GO:0045047	6.23205818299822e-18	32.5173789173789	0.6895024565774 16
106	protein targeting to ER
GO:0072599	6.23205818299822e-18	32.5173789173789	0.6895024565774 16
106	establishment of protein localization to endoplasmic reticulum
GO:0006415	1.81309748591631e-17	35.6787141905396	0.591931354231541
15	91	translational termination
GO:0070972	3.70104721246605e-17	28.667672197084 0.767559338454086	16
118	protein localization to endoplasmic reticulum
GO:0019080	8.92631084441153e-17	23.4339019189765	0.982215763614975
17	151	viral genome expression
GO:0019083	8.92631084441153e-17	23.4339019189765	0.982215763614975
17	151	viral transcription
GO:0006414	1.71210179137508e-16	30.0991561181435	0.682997716421009
15	105	translational elongation
GO:0072594	8.18501634781108e-16	18.0445670628183	1.32696699190367
18	204	establishment of protein localization to organelle
GO:0000184	1.18437393455805e-15	26.0217867575463	0.774064078610477
15	119	nuclear-transcribed mRNA catabolic process, nonsense-mediated decay
GO:0006612	2.54076105251855e-15	21.2914093206064	0.995225243927756
16	153	protein targeting to membrane
GO:0022415	3.92873589992143e-15	14.8383955739972	1.69123244066155
19	260	viral reproductive process
GO:0019058	7.34008832451422e-15	15.7272053372869	1.50259497612622
18	231	viral infectious cycle
GO:0006413	4.82600731905916e-14	19.7080291970803	0.988720503771365
15	152	translational initiation
GO:0043624	5.32295636403822e-14	19.563841496973 0.995225243927756	15
153	cellular protein complex disassembly
GO:0043241	8.59927424657248e-14	18.8731521642914	1.02774894470971
15	158	protein complex disassembly
GO:0000956	2.33426310187263e-13	17.5115074798619	1.09930108643	15
169	nuclear-transcribed mRNA catabolic process
GO:0034623	3.58973326371703e-13	16.9548602818247	1.13182478721196
15	174	cellular macromolecular complex disassembly
GO:0006402	5.01601595084808e-13	16.5341306204861	1.15784374783752

15	178	mRNA catabolic process
GO:0032984	5.4465081225471e-13	16.4321549861068	1.16434848799391
15	179	macromolecular complex disassembly
GO:0006401	3.9367873386225e-12	14.1575616255829	1.33347173206007
15	205	RNA catabolic process
GO:0016032	8.11184977974904e-12	8.73267699253778	2.93363781053214
20	451	viral reproduction
GO:0033365	1.58270085419441e-10	7.75791483113069	3.07674209397274
19	473	protein localization to organelle
GO:0006605	1.64045588855248e-10	7.74030769230769	3.08324683412913
19	474	protein targeting
GO:0071845	4.02602609314716e-10	9.94400263517011	1.84734620441492
15	284	cellular component disassembly at cellular level
GO:0022411	4.88941229822078e-10	9.79552093476144	1.87336516504048
15	288	cellular component disassembly
GO:0042254	4.32489332404076e-08	12.1771154504967	0.969206283302194
10	149	ribosome biogenesis
GO:0042274	8.38161833336861e-08	61.9878997407087	0.11708532281503
5	18	ribosomal small subunit biogenesis
GO:0022613	2.26870176916479e-07	8.75875464474721	1.46356653518788
11	225	ribonucleoprotein complex biogenesis
GO:0071843	4.15216692928138e-07	8.21279856267174	1.55463289737734
11	239	cellular component biogenesis at cellular level
GO:0006364	4.99793810148488e-06	11.8284156890627	0.676492976264618
7	104	rRNA processing
GO:0016072	7.25372287208501e-06	11.1346947885281	0.715521417202962
7	110	rRNA metabolic process
Tissue: Adipose_Subcutaneous=>Nerve_Tibial	Type:	asymmetric
SourceGene:	HR(ENSG00000168453.10)
TargetGeneSet:	IGSF21	C1QA	C1QC	C1QB	RPS6KA1	LAPTM5	CSF3R	BRD8
MRPS21	CTSS	CD84	ARHGAP30	PTPRC	TLR5	SP140L	GPX1	UBA7
STAB1	PDE6B	SH3TC1	DTHD1	TLR1	TLR6	NSUN7	DAPP1	FYB	GZMA
SEPT8	PCDHA1	CSF1R	CD74	DOCK2	RP11	F13A1	LY86	AIF1	HLA-DRA
HLA-DMB	HLA-DMA	HLA-DOA	DEFA6	MKRNP2	FGD2	VNN1	VNN3	IPCEF1	AOAH	TFEC
TBXAS1	MKRN1	CTSB	MSR1	ADAM28	DOCK8	SYK	FGD3	COL27A1	GGTA1P	MRC1
ASCL2	MS4A6A	MS4A7	CARNS1	FOLR2	KCNE3	SLCO2B1	IL10RA	AMICA1	CD3G
SORL1	SLC37A2	KCNJ5	C11orf45	CD4	PTPN6	CLEC7A	BIN2	NCKAP1L	LYZ
YEATS4	SELPLG	P2RX4	LCP1	ITGAM	DPEP2	IRF8	ADAP2	EVI2B	EVI2A	MPO
AANAT	TMC8	HMHA1	VAV1	EMR1	STXBP2	MYO1F	LRRC25	GMIP	NUDT19
CEACAM21	CD37	SIGLEC9	CD33	FPR3	ZNF766	LILRB5	LILRA4	LAIR1
LILRB4	RPL28	ITGB2	NFAM1	KLHDC7B	CSF2RA	TLR7	TLR8	CXorf21	CYBB
GPR34	WAS	BTK
GOBPID	Pvalue	OddsRatio	ExpCount	Count	Size	Term
GO:0050778	5.83421819973224e-17	12.5164556962025	2.59442253131271
24	364	positive regulation of immune response
GO:0002253	1.37762253364924e-16	13.7969425502518	2.1311327935783	22
299	activation of immune response
GO:0002757	3.63045077197809e-15	14.798721340388	1.67497059027057	19
235	immune response-activating signal transduction
GO:0002764	9.77356183820755e-15	13.9457787481805	1.76762853781745
19	248	immune response-regulating signaling pathway
GO:0002429	5.35930412653596e-13	18.8089887640449	0.947962078748876
14	133	immune response-activating cell surface receptor signaling pathway
GO:0002768	1.61754876053476e-12	17.2041486603284	1.02636495744239
14	144	immune response-regulating cell surface receptor signaling pathway
GO:0050851	3.62165101025428e-12	18.6963636363636	0.876686734482043
13	123	antigen receptor-mediated signaling pathway
GO:0042110	9.66559309237516e-12	9.88258745358609	2.27368348211196
18	319	T cell activation
GO:0050867	1.26412827960321e-11	11.5960591133005	1.71060826240399
16	240	positive regulation of cell activation
GO:0050870	1.70490997099201e-11	14.2184212409647	1.21880838696284
14	171	positive regulation of T cell activation

GO:0046649	2.29891985491694e-11	8.1303421920126	3.08622240675386	20	433	lymphocyte activation
GO:0051251	3.05709522713005e-11	12.0579545454545	1.5324199017369	15	215	positive regulation of lymphocyte activation
GO:0050865	3.22243936891259e-11	9.13715837104072	2.44474430835236	18	343	regulation of cell activation
GO:0002696	9.57221723963338e-11	11.0482693911593	1.6607155214172	15	233	positive regulation of leukocyte activation
GO:0002694	1.04518405122587e-10	9.13203794369645	2.28793855096533	17	321	regulation of leukocyte activation
GO:0051249	1.49271123631121e-10	9.69891084420337	2.01709224275137	16	283	regulation of lymphocyte activation
GO:0002252	2.22586293386065e-10	8.04475703324808	2.75122828869974	18	386	immune effector process
GO:0050863	5.2475924152608e-10	10.7460239917494	1.575185108297	14	221	regulation of T cell activation
GO:0006954	2.59299777239302e-08	6.17588189182127	3.29292090512767	17	462	inflammatory response
GO:0002521	2.9071042411463e-08	7.6794631710362	2.15251539685835	14	302	leukocyte differentiation
GO:0050852	3.38874650811354e-08	15.168085106383	0.705625908241644	9	99	T cell receptor signaling pathway
GO:0001817	3.55661817144971e-08	6.93908562367865	2.5587848591793	15	359	regulation of cytokine production
GO:0031294	5.75323042297869e-08	17.9494108405342	0.534565082001246	8	75	lymphocyte costimulation
GO:0031295	5.75323042297869e-08	17.9494108405342	0.534565082001246	8	75	T cell costimulation
GO:0001816	1.24326396961947e-07	6.26555023923445	2.81537609853989	15	395	cytokine production
GO:0002250	1.60882790896458e-07	9.05434782608696	1.41125181648329	11	198	adaptive immune response
GO:0030098	2.17512041569067e-07	8.76914845685965	1.45401702304339	11	204	lymphocyte differentiation
GO:0002443	3.0535618324637e-07	8.45804347826087	1.50390976403017	11	211	leukocyte mediated immunity
GO:0002504	3.98933557411334e-07	43.0102040816327	0.156805757387032	5	22	antigen processing and presentation of peptide or polysaccharide antigen via MHC class II
GO:0045619	5.07255268071156e-07	13.1932909196067	0.705625908241644	8	99	regulation of lymphocyte differentiation
GO:0045058	6.36646490171908e-07	38.4774436090226	0.171060826240399	5	24	T cell selection
GO:0042742	7.15487089420626e-07	10.3114119922631	1.00498235416234	9	141	defense response to bacterium
GO:0030097	9.43330894915396e-07	5.28865665584416	3.30004843955436	15	463	hemopoiesis
GO:0030217	1.66945045570303e-06	9.24945722970039	1.11189537056259	9	156	T cell differentiation
GO:0050853	1.72916770905549e-06	30.4506802721088	0.206698498373815	5	29	B cell receptor signaling pathway
GO:0048534	2.16492874544693e-06	4.92471590909091	3.52812954120822	15	495	hemopoietic or lymphoid organ development
GO:0042108	3.75664466245746e-06	16.6835246061078	0.420524531174313	6	59	positive regulation of cytokine biosynthetic process
GO:0071346	4.68617126705722e-06	11.9524664750958	0.669988236108228	7	94	cellular response to interferon-gamma
GO:0045621	5.03771779945367e-06	15.7864506627393	0.441907134454363	6	62	positive regulation of lymphocyte differentiation
GO:0045577	5.39412032474818e-06	44.5532245532246	0.121168085253616	4	17	regulation of B cell differentiation
GO:0002460	5.67703879311226e-06	7.89114299851559	1.29008373122967	9	181	adaptive immune response based on somatic recombination of immune

receptors built from immunoglobulin superfamily domains
GO:0019886	6.92478501404327e-06	143.45	0.0427652065600996	3	6
antigen processing and presentation of exogenous peptide antigen via MHC class II
GO:0042116	6.94617424957758e-06	22.1320346320346	0.270846308213964
5	38	macrophage activation
GO:0050900	8.38207304992738e-06	6.57124237769399	1.71773579683067
10	241	leukocyte migration
GO:0002274	1.10692443349267e-05	10.3891666666667	0.76264618365511
7	107	myeloid leukocyte activation
GO:0034341	1.40863437765481e-05	9.98677884615385	0.791156321361843
7	111	response to interferon-gamma
GO:0030593	1.45280962225576e-05	18.7192569335426	0.313611514774064
5	44	neutrophil chemotaxis
GO:0019724	1.67707201985632e-05	9.70473130841122	0.812538924641893
7	114	B cell mediated immunity
GO:0002275	1.81314245547875e-05	17.8036336485814	0.327866583627431
5	46	myeloid cell activation involved in immune response
GO:0002495	1.91888241560716e-05	86.058	0.0570202754134662	3	8
antigen processing and presentation of peptide antigen via MHC class II
GO:0045059	1.91888241560716e-05	86.058	0.0570202754134662	3	8
positive thymic T cell selection
Tissue: Nerve_Tibial=>Adipose_Subcutaneous	Type:	asymmetric
SourceGene:	JAZF1-AS1(ENSG00000234336.1)
TargetGeneSet:	RPL11	TMEM50A	KHDRBS1	RP4	RP11	NRBP1	UBC	RPL31	MCM6
RPL37A	AAMP	RPL32	RPL14	RPL24	BTLA	MBNL1	RPL35A	PPP2R2C	RPL34
ADAMTS16		TBCA	MAT2B	STC2	CSNK2B	RPS10	RPL10A	RPL39P3	MRPS12	ECD
ARF5	LEP	BIN3	RPL30	RPS6	PSMB7	RPL35	RPL12	RPL7A	MRVI1-AS1
RNF121	RPS25	RPL41	RPL14P1	POLR1D	C14orf119		ANKDD1A	RPL35AP32
RPS17L	RPL17	RPL19	CTIF	TLE6	TLE2	UBA52	ZNF14	XRCC1	PTGIR
SLC1A5	RPS9	RPL28	U2AF1	NHP2L1	SAMM50	RPL10
GOBPID	Pvalue	OddsRatio	ExpCount	Count	Size	Term
GO:0006415	2.19924758994353e-41	169.602272727273	0.3589370977787 25
91	translational termination
GO:0000184	3.45602585800618e-40	128.971904266389	0.469379281710608
26	119	nuclear-transcribed mRNA catabolic process, nonsense-mediated decay
GO:0006414	1.29833321434107e-39	139.78515625	0.414158189744654	25
105	translational elongation
GO:0006614	1.29833321434107e-39	139.78515625	0.414158189744654	25
105	SRP-dependent cotranslational protein targeting to membrane
GO:0006613	1.69541091254078e-39	138.049768518519	0.418102553456508
25	106	cotranslational protein targeting to membrane
GO:0045047	1.69541091254078e-39	138.049768518519	0.418102553456508
25	106	protein targeting to ER
GO:0072599	1.69541091254078e-39	138.049768518519	0.418102553456508
25	106	establishment of protein localization to endoplasmic reticulum
GO:0070972	3.38396096066161e-38	120.136088709677	0.465434917998754
25	118	protein localization to endoplasmic reticulum
GO:0006413	3.70068139013524e-37	94.973886328725 0.599543284201785	26
152	translational initiation
GO:0006612	4.44900001617343e-37	94.2194564389129	0.603487647913639
26	153	protein targeting to membrane
GO:0000956	7.15594190037722e-36	83.58357771261	0.666597467303301	26
169	nuclear-transcribed mRNA catabolic process
GO:0019080	2.8308522503373e-35	88.4672619047619	0.595598920489931
25	151	viral genome expression
GO:0019083	2.8308522503373e-35	88.4672619047619	0.595598920489931
25	151	viral transcription
GO:0006402	3.01470694724752e-35	78.5848896434635	0.702096740709986
26	178	mRNA catabolic process
GO:0043624	4.03244913386787e-35	87.07275390625	0.603487647913639	25
153	cellular protein complex disassembly
GO:0043241	9.55427181679984e-35	83.7699718045113	0.623209466472908
25	158	protein complex disassembly

GO:0034623	1.24961935582655e-33	74.6906459731544	0.68631928586257
25	174	cellular macromolecular complex disassembly
GO:0072594	1.27699410546344e-33	66.9836897426604	0.804650197218186
26	204	establishment of protein localization to organelle
GO:0006401	1.45941791563992e-33	66.6047936565147	0.808594560930039
26	205	RNA catabolic process
GO:0032984	2.64709413072781e-33	72.2402597402597	0.706041104421839
25	179	macromolecular complex disassembly
GO:0019058	2.09619562885012e-30	53.807645631068 0.91114801743824	25
231	viral infectious cycle
GO:0022415	4.42228106329537e-29	47.0711436170213	1.025534565082	25
260	viral reproductive process
GO:0071845	4.23392337754159e-28	42.636944980695 1.12019929416649	25
284	cellular component disassembly at cellular level
GO:0022411	6.04763774659291e-28	41.9765922053232	1.13597674901391
25	288	cellular component disassembly
GO:0033365	2.18099658442602e-25	28.1461883408072	1.86568403570687
27	473	protein localization to organelle
GO:0006605	2.30795948470955e-25	28.0812080536913	1.86962839941873
27	474	protein targeting
GO:0016032	1.74358332784865e-24	27.5669070208729	1.77890803404609
26	451	viral reproduction
GO:0022613	1.56171695073804e-08	14.0316674913409	0.887481835167116
10	225	ribonucleoprotein complex biogenesis
GO:0071843	2.77218191947728e-08	13.1608287652142	0.94270292713307
10	239	cellular component biogenesis at cellular level
GO:0042254	1.14025121156735e-07	16.5036908380373	0.587710193066224
8	149	ribosome biogenesis
GO:0000028	1.153971806579e-06	266.5	0.0236661822711231	3	6
ribosomal small subunit assembly
GO:0042273	1.248183947148e-05	88.7962962962963	0.0473323645422462
3	12	ribosomal large subunit biogenesis
Tissue: Nerve_Tibial=>Adipose_Subcutaneous	Type:	asymmetric
SourceGene:	NANOS3(ENSG00000187556.5)
TargetGeneSet:	RPL22	SPEN	CDC42	RP11	RPS8	RPS15AP10	BTF3L4	RP4
RPL5	EEF1A1P11	SNX7	RPS27	PRRC2C	ARPC5	RPS7	UBC	DUSP11
ANKRD36BP2	TXNDC9	MMADHC	SENP2	EEF1B2	ASIC4	TRNT1	RPL32	UBE2E1
RPL15	RPL29	DNAH1	RPL10AP6	RP13	PCNP	RPL24	C3orf17	TPRG1-AS2
RPL35A	COPS4	EEF1A1P9	RPL34	OSTC	CCDC109B	RPS3A	CTD
EEF1A1P19	BTF3	RPS23	SKP1	CTB	RPS14	RPL15P3	GPLD1	MCFD2P1
RPS10	RPL10A	GLO1	EEF1A1	MRPS12	IGF2R	ANKRD61	EEF1A1P6	MALSU1
VPS41	RPS3AP26	BUD31	FAM3C	ARF5	RPL7	EIF3H	RPL8	RPS6
ELAVL2	UBAP2	CLTA	HNRNPK	ATP6V1G1	RPL12	EEF1A1P5	RPL7A
COMMD3	CCNY	VN1R53P	CDH23	RPS24	RPS3AP5	RPL13AP5	SFR1	LHPP
RPL27A	RPS13	TSG101	RPS3	SETP17	PHB2	CMAS	H3F3C	MYF6	RPL6
RPLP0	TPT1	EBPL	EAPP	FKBP3	RPL32P29	CGRRF1	JKAMP	C14orf159
TTBK2	RSL24D1	RPS3AP6	RAB11A	RPL4	MORF4L1	SEC11A	RPS15A	RRN3P2	HSBP1
RPL26	RPL17P43	RPS7P1	SUPT6H	RPL23A	RPL19	STAT5A	RPL27	RPS7P11
RPS2P48	USP36	MYL12A	MYL12B	VAPA	PSMG2	RPL17	RPS15	PIP5K1C	RPSAP58
RPS19	MEGF8	CEACAMP2	RPL18	RPL13A	ACPT	RPS9	RPS5	SNRPB2	RP1
RPL41P1	PFDN4	RPL12P4	PI4KA	EIF3L	RPL3	GS1	RP6	PDZD11	RPS23P8
RPS4X	RPSAP14	RPL10	VBP1
GOBPID	Pvalue	OddsRatio	ExpCount	Count	Size	Term
GO:0006414	3.84591570766492e-71	154.567796610169	0.857380112102969
46	105	translational elongation
GO:0006415	2.16196924424638e-70	180.731454859114	0.743062763822573
44	91	translational termination
GO:0006614	5.76736347202654e-69	146.640410958904	0.857380112102969
45	105	SRP-dependent cotranslational protein targeting to membrane
GO:0006613	9.9720990216942e-69	144.226364248821	0.86554563698014
45	106	cotranslational protein targeting to membrane
GO:0045047	9.9720990216942e-69	144.226364248821	0.86554563698014
45	106	protein targeting to ER

GO:0072599 9.9720990216942e-69 144.226364248821 0.86554563698014
45 106 establishment of protein localization to endoplasmic reticulum
GO:0070972 3.53917697518047e-68 126.544367283951 0.963531935506193
46 118 protein localization to endoplasmic reticulum
GO:0000184 8.06624369212832e-64 113.036396396396 0.971697460383364
44 119 nuclear-transcribed mRNA catabolic process, nonsense-mediated decay
GO:0006413 4.47527293437489e-62 85.7497379454927 1.24115978133001
46 152 translational initiation
GO:0006612 5.28819604002475e-60 81.1929223744292 1.24932530620718
45 153 protein targeting to membrane
GO:0019080 2.14721967205047e-58 79.0532962869411 1.23299425645284
44 151 viral genome expression
GO:0019083 2.14721967205047e-58 79.0532962869411 1.23299425645284
44 151 viral transcription
GO:0043624 4.19910718122264e-58 77.5918670964543 1.24932530620718
44 153 cellular protein complex disassembly
GO:0043241 2.14664627476667e-57 74.1626363205311 1.29015293059304
44 158 protein complex disassembly
GO:0000956 6.31798476616127e-56 67.584 1.37997370424192 44 169
nuclear-transcribed mRNA catabolic process
GO:0072594 2.16423941995547e-55 57.3180379746835 1.66576707494291
46 204 establishment of protein localization to organelle
GO:0034623 2.69901455226841e-55 64.9617463617464 1.42080132862778
44 174 cellular macromolecular complex disassembly
GO:0006402 8.3276990259618e-55 63.0048406615571 1.45346342813646
44 178 mRNA catabolic process
GO:0032984 1.09859669494889e-54 62.5337337337337 1.46162895301363
44 179 macromolecular complex disassembly
GO:0006401 8.30988327641433e-52 52.339096860836 1.67393259982008 44
205 RNA catabolic process
GO:0019058 5.51750453719069e-51 46.8857711003093 1.88623624662653
45 231 viral infectious cycle
GO:0022415 1.68418515003962e-48 40.4784963364129 2.12303646806449
45 260 viral reproductive process
GO:0071845 4.07200278512508e-45 34.9150900900901 2.3190090651166 44
284 cellular component disassembly at cellular level
GO:0022411 7.77280592205043e-45 34.3329641116526 2.35167116462529
44 288 cellular component disassembly
GO:0033365 4.26057834754705e-39 21.610427825167 3.86229326690194 47
473 protein localization to organelle
GO:0016032 1.01124457558609e-38 21.9714677640604 3.68265171960418
46 451 viral reproduction
GO:0006605 9.99924074929472e-38 20.7564252336449 3.87045879177912
46 474 protein targeting
GO:0042254 1.12936693832616e-12 15.4314592088103 1.2166632066985 15
149 ribosome biogenesis
GO:0022613 3.99845339492456e-11 10.6006191950464 1.8372430973635 16
225 ribonucleoprotein complex biogenesis
GO:0042274 5.97449731042717e-11 82.1081081081081 0.14697944778908
7 18 ribosomal small subunit biogenesis
GO:0071843 9.87685386521456e-11 9.92526158445441 1.9515604456439 16
239 cellular component biogenesis at cellular level
GO:0006364 7.90125225689655e-10 15.7411315445684 0.849214587225798
11 104 rRNA processing
GO:0016072 1.44970325921689e-09 14.7808930425753 0.898207736488824
11 110 rRNA metabolic process
GO:0042273 2.52226602828915e-08 90.5562579013907 0.0979862985260536
5 12 ribosomal large subunit biogenesis
GO:0034470 8.3984688957256e-08 8.51766358892011 1.63310497543423
12 200 ncRNA processing
GO:0034660 3.64835798864627e-06 5.83039601907526 2.32717458999377
12 285 ncRNA metabolic process
GO:0030490 1.81382042849368e-05 93.45 0.0571586741401979 3 7

maturation of SSU-rRNA
Tissue: Nerve_Tibial=>Adipose_Subcutaneous Type: cluster
SourceGene: NANOS3(ENSG00000187556.5)
TargetGeneSet: RPL22 SPEN CDC42 RP11 RPS8 RPS15AP10 BTF3L4 RP4
RPL5 EEF1A1P11 SNX7 RPS27 PRRC2C ARPC5 RPS7 UBC DUSP11
ANKRD36BP2 TXNDC9 MMADHC SENP2 EEF1B2 ASIC4 TRNT1 RPL32 UBE2E1
RPL15 RPL29 DNAH1 RPL10AP6 RP13 PCNP RPL24 C3orf17 TPRG1-AS2
RPL35A COPS4 EEF1A1P9 RPL34 OSTC CCDC109B RPS3A CTD
EEF1A1P19 BTF3 RPS23 SKP1 CTB RPS14 RPL15P3 GPLD1 MCFD2P1
RPS10 RPL10A GLO1 EEF1A1 MRPS12 IGF2R ANKRD61 EEF1A1P6 MALSU1
VPS41 RPS3AP26 BUD31 FAM3C ARF5 RPL7 EIF3H RPL8 RPS6
ELAVL2 UBAP2 CLTA HNRNPK ATP6V1G1 RPL12 EEF1A1P5 RPL7A
COMMD3 CCNY VN1R53P CDH23 RPS24 RPS3AP5 RPL13AP5 SFR1 LHPP
RPL27A RPS13 TSG101 RPS3 SETP17 PHB2 CMAS H3F3C MYF6 RPL6
RPLP0 TPT1 EBPL EAPP FKBP3 RPL32P29 CGRRF1 JKAMP C14orf159
TTBK2 RSL24D1 RPS3AP6 RAB11A RPL4 MORF4L1 SEC11A RPS15A RRN3P2 HSBP1
RPL26 RPL17P43 RPS7P1 SUPT6H RPL23A RPL19 STAT5A RPL27 RPS7P11
RPS2P48 USP36 MYL12A MYL12B VAPA PSMG2 RPL17 RPS15 PIP5K1C RPSAP58
RPS19 MEGF8 CEACAMP2 RPL18 RPL13A ACPT RPS9 RPS5 SNRPB2 RP1
RPL41P1 PFDN4 RPL12P4 PI4KA EIF3L RPL3 GS1 RP6 PDZD11 RPS23P8
RPS4X RPSAP14 RPL10 VBP1

GOBPID	Pvalue	OddsRatio	ExpCount	Count	Size	Term
GO:0006414	3.84591570766492e-71	154.567796610169	0.857380112102969	46	105	translational elongation
GO:0006415	2.16196924424638e-70	180.731454859114	0.743062763822573	44	91	translational termination
GO:0006614	5.76736347202654e-69	146.640410958904	0.857380112102969	45	105	SRP-dependent cotranslational protein targeting to membrane
GO:0006613	9.9720990216942e-69	144.226364248821	0.86554563698014	45	106	cotranslational protein targeting to membrane
GO:0045047	9.9720990216942e-69	144.226364248821	0.86554563698014	45	106	protein targeting to ER
GO:0072599	9.9720990216942e-69	144.226364248821	0.86554563698014	45	106	establishment of protein localization to endoplasmic reticulum
GO:0070972	3.53917697518047e-68	126.544367283951	0.963531935506193	46	118	protein localization to endoplasmic reticulum
GO:0000184	8.06624369212832e-64	113.036396396396	0.971697460383364	44	119	nuclear-transcribed mRNA catabolic process, nonsense-mediated decay
GO:0006413	4.47527293437489e-62	85.7497379454927	1.24115978133001	46	152	translational initiation
GO:0006612	5.28819604002475e-60	81.1929223744292	1.24932530620718	45	153	protein targeting to membrane
GO:0019080	2.14721967205047e-58	79.0532962869411	1.23299425645284	44	151	viral genome expression
GO:0019083	2.14721967205047e-58	79.0532962869411	1.23299425645284	44	151	viral transcription
GO:0043624	4.19910718122264e-58	77.5918670964543	1.24932530620718	44	153	cellular protein complex disassembly
GO:0043241	2.14664627476667e-57	74.1626363205311	1.29015293059304	44	158	protein complex disassembly
GO:0000956	6.31798476616127e-56	67.584	1.37997370424192	44	169	nuclear-transcribed mRNA catabolic process
GO:0072594	2.16423941995547e-55	57.3180379746835	1.66576707494291	46	204	establishment of protein localization to organelle
GO:0034623	2.69901455226841e-55	64.9617463617464	1.42080132862778	44	174	cellular macromolecular complex disassembly
GO:0006402	8.3276990259618e-55	63.0048406615571	1.45346342813646	44	178	mRNA catabolic process
GO:0032984	1.09859669494889e-54	62.5337337337337	1.46162895301363	44	179	macromolecular complex disassembly
GO:0006401	8.30988327641433e-52	52.339096860836	1.67393259982008	44	205	RNA catabolic process
GO:0019058	5.51750453719069e-51	46.8857711003093	1.88623624662653			

45	231	viral infectious cycle
GO:0022415	1.68418515003962e-48	40.4784963364129	2.12303646806449
45	260	viral reproductive process
GO:0071845	4.07200278512508e-45	34.9150900900901	2.3190090651166 44
284	cellular component disassembly at cellular level
GO:0022411	7.77280592205043e-45	34.3329641116526	2.35167116462529
44	288	cellular component disassembly
GO:0033365	4.26057834754705e-39	21.610427825167 3.86229326690194	47
473	protein localization to organelle
GO:0016032	1.01124457558609e-38	21.9714677640604	3.68265171960418
46	451	viral reproduction
GO:0006605	9.99924074929472e-38	20.7564252336449	3.87045879177912
46	474	protein targeting
GO:0042254	1.12936693832616e-12	15.4314592088103	1.2166632066985 15
149	ribosome biogenesis
GO:0022613	3.99845339492456e-11	10.6006191950464	1.8372430973635 16
225	ribonucleoprotein complex biogenesis
GO:0042274	5.97449731042717e-11	82.1081081081081	0.14697944778908
7	18	ribosomal small subunit biogenesis
GO:0071843	9.87685386521456e-11	9.92526158445441	1.9515604456439 16
239	cellular component biogenesis at cellular level
GO:0006364	7.90125225689655e-10	15.7411315445684	0.849214587225798
11	104	rRNA processing
GO:0016072	1.44970325921689e-09	14.7808930425753	0.898207736488824
11	110	rRNA metabolic process
GO:0042273	2.52226602828915e-08	90.5562579013907	0.0979862985260536
5	12	ribosomal large subunit biogenesis
GO:0034470	8.3984688957256e-08	8.51766358892011	1.63310497543423
12	200	ncRNA processing
GO:0034660	3.64835798864627e-06	5.83039601907526	2.32717458999377
12	285	ncRNA metabolic process
GO:0030490	1.81382042849368e-05	93.45	0.0571586741401979	3	7
maturation of SSU-rRNA
Tissue: Nerve_Tibial=>Adipose_Subcutaneous	Type:	cluster
SourceGene:	NHSL2(ENSG00000204131.6)
TargetGeneSet:	AGRN	MRPL20	RPL22	SPEN	HSPG2	RPL11	SYF2	ARID1A	RP4
RPS8	BTF3L4	TM2D1	GNG5	CCBL2	RPL5	RP11	STXBP3	HBXIP	ATP5F1
RAP1A	SRGAP2P1	MRPS21	RPS27	C1orf43	CKS1B	DAP3	SSR2	PMF1
PRRC2C	GAS5	ZBTB37	CR2	C1orf132	NENF	TOMM20	ACP1	RPS7
C2orf28	COX7A2L	PREPL	SPTBN1	UBC	CCDC104	C1D	GMCL1	SNRPG	TPRKB
DGUOK	DGUOK-AS1	DCTN1	VAMP5	ANKRD36	COA5	MITD1	RPL31	RANBP2
GPR17	MMADHC	ARL6IP6	SNORA70F	HAT1	UBE2E3	SENP2	NBEAL1	EEF1B2
RPL37A	USP37	MFF	GIGYF2	RPL32	TBC1D5	RPL15	EIF1B	RPL14	SS18L2	MAP4
LAMB2	NICN1	RAD54L2	RPL29	SPCS1	KCTD6	RPL10AP6	CTD	RP13
C3orf26	NIT2	PCNP	RPL24	ZBTB20	CNBP	RNF7	RNF13	PHC3	RPL22L1
DNAJC19	EIF4G1	PPP1R2	RPL35A	MRFAP1L1	CNO	C4orf52	OCIAD1	MTHFD2L
ENOPH1	COPS4	LAMTOR3	UBE2D3	CISD2	RPL34	RPS3A	ERCC4	NIPBL	EEF1A1P19
MRPS36	TAF9	BTF3	TBCA	COX7C	HSPD1P11	EPB41L4A-AS1	HINT1	SKP1
PCDHGB3	HDAC3	CTB	RPS14	CCNG1	NUDCD2	MAT2B	NPM1	MSX2	GNB2L1
LYRM4	TMEM14C	RPL15P3	CSNK2B	C6orf48	CUTA	RPS10	RPL10A	GLO1	C6orf130
MRPL32	RPL39P3	EEF1A1	WDR11	CCNC	MRPS12	SF3B5	C6orf72	RP3	IGF2R
SFT2D1	PSMB1	RP1	LSM5	OGDH	STAG3L4	C7orf23	RPS3AP26	CUX1
DUS4L	FAM3C	NDUFA5	ARF5	MTPN	ZC3HAV1L	BRAF	TRPV6	RHEB	MLL3
HMBOX1	PPAPDC1B	GOLGA7	KAT6A	C8orf40	HOOK3	TCEA1	RPS20	SNHG6	RPL7
ZFAND1	RPL30	POLR2K	EIF3E	EIF3H	TATDN1	EEF1D	CPSF1	RPL8	SNAPC3	RPS6
CHMP5	TLN1	RGP1	CLTA	SEC61B	INVS	ATP6V1G1	PSMB7	RPL35
RPL12	EEF1A1P5	RPL7A	C10orf31	RAB18	ZFAND4	VPS26A	RPS24
RPS3AP5	RPL13AP5	DNMBP-AS1	NDUFB8	GBF1	SFR1	NHLRC2	IGF2
RPL27A	RPS13	TSG101	EIF3M	CKAP5	AHNAK	FAU	PPP6R3	LAMTOR1	SPCS2	RPS3
CCDC90B	TMEM126B	SDHD	ZBTB16	UBE4A	ATP5L	RPS25	THYN1	WNK1	ERC1
C12orf57	PHB2	SCAF11	MLL4	PFDN5	PRR13	ITGA7	RPL41	CNPY2	NACA
LRP1	NAP1L1	EEA1	USP44	RPL18AP3	ISCU	ARPC3	VPS29	C12orf51
RPL6	RPLP0	ZNF891	RPSAP54	MRP63	POLR1D	RFXAP	DGKH	TPT1	ZC3H13	ESD

MED4 MZT1 CCNB1IP1 OXA1L PPP2R3C RPS29 RPL36A DNAAF2 KLHDC2 CNIH
CGRRF1 JKAMP C14orf142 DYNC1H1 SIVA1 RPS20P33 PGBD4 CDAN1 B2M
RSL24D1 GTF2A2 RPS3AP6 RPS27L USP3 RPL4 MYO9A RCN2 MORF4L1 RPS17L
C15orf40 SEC11A MEF2A MPG RGS11 ARHGDIG TSC2 SRRM2 TCEB2
RPS15A METTL9 SRCAP NAE1 ZFHX3 CDYL2 MLYCD ANKRD11 DERL2 GABARAP
RPL26 RPL17P43 RPS7P1 SDF2 SUPT6H RPL23A TAOK1 ZNHIT3 RPL17
RPL19 SMARCE1 RPL27 RPS7P11 MRPL10 HOXB-AS5 SUPT4H1 SUMO2 MYL12B
PSMG2 C18orf21 C18orf32 RPS15 OAZ1 NFIC RPL36 NDUFA11
EIF3G WDR83OS ASNA1 C19orf53 FAM32A RPL18A UBA52 RPSAP58 SDHAF1
EIF3K RPS16 EID2B FBL RPS19 ZNF222 SNRPD2 RPL18 RPL13A RPS11 RPS9
CCDC106 RPS5 SNRPB2 CHMP4B EIF2S2 PHF20 IFT52 NCOA3 DPM1 PFDN4
RPL12P4 RPS21 U2AF1 THAP7 ASLP1 MYH9 MPST EIF3L RPL3 ST13
PACSIN2 ATXN10 UXT WASF4P WDR45 CACNA1F HUWE1 RPS23P8 RPS4X APOOL
RPL39 RAP2C SSR4 RPL10 MXRA5P1

GOBPID	Pvalue	OddsRatio	ExpCount	Count	Size	Term
GO:0006614	6.45106691204202e-89	105.339485827291	2.28150301017231	68	105	SRP-dependent cotranslational protein targeting to membrane
GO:0006613	1.76910328103097e-88	102.560119811724	2.30323161026919	68	106	cotranslational protein targeting to membrane
GO:0045047	1.76910328103097e-88	102.560119811724	2.30323161026919	68	106	protein targeting to ER
GO:0072599	1.76910328103097e-88	102.560119811724	2.30323161026919	68	106	establishment of protein localization to endoplasmic reticulum
GO:0070972	1.01327684636074e-83	77.8793495934959	2.56397481143173	68	118	protein localization to endoplasmic reticulum
GO:0006415	4.12166115428914e-83	119.690202517789	1.977302608816	62	91	translational termination
GO:0006414	6.02087294137304e-83	91.9984939759036	2.28150301017231	65	105	translational elongation
GO:0006413	1.28380404644126e-78	50.7024335721181	3.30274721472562	71	152	translational initiation
GO:0000184	5.7399879383646e-76	65.5453090909091	2.58570341152861	64	119	nuclear-transcribed mRNA catabolic process, nonsense-mediated decay
GO:0006612	5.55466429894088e-75	47.116472303207	3.3244758148225	69	153	protein targeting to membrane
GO:0019080	1.6869994287557e-70	43.995825426945	3.28101861462875	66	151	viral genome expression
GO:0019083	1.6869994287557e-70	43.995825426945	3.28101861462875	66	151	viral transcription
GO:0072594	2.09893799234257e-67	30.7646895015316	4.43263441976334	71	204	establishment of protein localization to organelle
GO:0043624	3.50991874841927e-65	39.1749003984064	3.3244758148225	63	153	cellular protein complex disassembly
GO:0000956	6.23368157818651e-65	35.2233935742972	3.67213341637257	65	169	nuclear-transcribed mRNA catabolic process
GO:0043241	4.27394447510369e-64	37.0998532187041	3.4331188153069	63	158	protein complex disassembly
GO:0034623	6.13749421481952e-64	33.5956670719576	3.78077641685696	65	174	cellular macromolecular complex disassembly
GO:0006402	3.59616494836834e-63	32.3971994171376	3.86769081724448	65	178	mRNA catabolic process
GO:0032984	5.5496516129275e-63	32.1107235961389	3.88941941734136	65	179	macromolecular complex disassembly
GO:0019058	8.50117794730148e-59	23.6977405356876	5.01930662237907	68	231	viral infectious cycle
GO:0006401	1.62730505587054e-58	26.0988238668962	4.45436301986022	65	205	RNA catabolic process
GO:0022415	6.22967086396807e-55	20.0766429539295	5.64943602518857	68	260	viral reproductive process
GO:0071845	2.0982633911219e-48	16.590013020117	6.17092242751367	65	284	cellular component disassembly at cellular level
GO:0022411	5.47737384812519e-48	16.2877519044789	6.2578368279011 8	65	288	cellular component disassembly

GO:0016032	1.89508869020437e-45	11.4848548918366	9.79959864369248
75	451	viral reproduction
GO:0033365	5.82016907928885e-45	11.0517960332748	10.2776278458238
76	473	protein localization to organelle
GO:0006605	6.81526150444609e-45	11.0232253705502	10.2993564459207
76	474	protein targeting
GO:0071843	9.69179643666664e-16	7.33553615112802	5.19313542315411
31	239	cellular component biogenesis at cellular level
GO:0022613	1.01318606176089e-14	7.2375402792696 4.8889350217978 29	225
ribonucleoprotein complex biogenesis
GO:0042274	1.19067414683568e-14	73.2814710042433	0.391114801743824
11	18	ribosomal small subunit biogenesis
GO:0042254	1.44127100552919e-14	9.27691034482759	3.23756141443499
24	149	ribosome biogenesis
GO:0006364	8.80285104973583e-12	9.93549654305468	2.25977441007543
18	104	rRNA processing
GO:0016072	2.35033860474211e-11	9.28356345475911	2.3901460106567 18
110	rRNA metabolic process
GO:0034470	7.51183818045965e-08	4.9660829665699 4.34572001937582	19
200	ncRNA processing
GO:0042273	8.30654399015847e-08	45.8798701298701	0.260743201162549
6	12	ribosomal large subunit biogenesis
GO:0034660	1.12999254286839e-06	3.76632795532113	6.19265102761055
21	285	ncRNA metabolic process
GO:0000028	3.16981716572228e-06	91.1935483870968	0.130371600581275
4	6	ribosomal small subunit assembly
GO:0030490	7.26944534829214e-06	60.7913978494624	0.152100200678154
4	7	maturation of SSU-rRNA
GO:0042255	1.68315364685945e-05	20.7796410709032	0.347657601550066
5	16	ribosome assembly
Tissue: Adipose_Subcutaneous=>Nerve_Tibial	Type:	asymmetric
SourceGene:	PLN(ENSG00000198523.5)
TargetGeneSet:	RPL22	RPL11	RPS8	RPL5	GAS5	RPL31	RPL37A	RPL32
RPL14	RP11	RPS10	RPL10A	MRPS12	RPS20	RPL30	RPS6	RPL12	RPL7A
PCBD1	OR52V1P RPL27A	RPS3	RPS25	RPL41	RPL6	RPL7AP6 RPS15A	RPL26
RPL23A	ACACA	RPL17	RPL19	RSL24D1P11	RPL36	PTGER1	UBA52	HPN
RPL28	SNORD17 PTTG1IP RPL10
GOBPID	Pvalue	OddsRatio	ExpCount	Count	Size	Term
GO:0006415	8.26332302859534e-60	959.071428571429	0.226697114386548
29	91	translational termination
GO:0006414	1.09589056363541e-57	781.637218045113	0.26157359352294
29	105	translational elongation
GO:0006614	1.09589056363541e-57	781.637218045113	0.26157359352294
29	105	SRP-dependent cotranslational protein targeting to membrane
GO:0006613	1.5079171340912e-57	771.432282003711	0.26406477060411
29	106	cotranslational protein targeting to membrane
GO:0045047	1.5079171340912e-57	771.432282003711	0.26406477060411
29	106	protein targeting to ER
GO:0072599	1.5079171340912e-57	771.432282003711	0.26406477060411
29	106	establishment of protein localization to endoplasmic reticulum
GO:0070972	5.38909278232418e-56	666.860353130016	0.293958895578161
29	118	protein localization to endoplasmic reticulum
GO:0000184	7.12221505251468e-56	659.404761904762	0.296450072659332
29	119	nuclear-transcribed mRNA catabolic process, nonsense-mediated decay
GO:0019080	3.25185068166945e-55	590.661157024793	0.376167739256799
30	151	viral genome expression
GO:0019083	3.25185068166945e-55	590.661157024793	0.376167739256799
30	151	viral transcription
GO:0006413	2.0175966932126e-52	481.379790940767	0.37865891633797
29	152	translational initiation
GO:0006612	2.4882763124305e-52	477.464285714286	0.381150093419141
29	153	protein targeting to membrane
GO:0043624	2.4882763124305e-52	477.464285714286	0.381150093419141

29 153 cellular protein complex disassembly
GO:0043241 6.94257702394279e-52 458.797342192691 0.393605978824995
29 158 protein complex disassembly
GO:0000956 5.87884912504229e-51 422.423469387755 0.421008926717874
29 169 nuclear-transcribed mRNA catabolic process
GO:0034623 1.47658466677695e-50 407.714285714286 0.433464812123728
29 174 cellular macromolecular complex disassembly
GO:0006402 3.02176630964737e-50 396.657718120805 0.443429520448412
29 178 mRNA catabolic process
GO:0032984 3.60426562279457e-50 393.985714285714 0.445920697529583
29 179 macromolecular complex disassembly
GO:0019058 3.33891897263147e-49 353.582089552239 0.575461905750467
30 231 viral infectious cycle
GO:0072594 2.15021886130407e-48 337.110204081633 0.508200124558854
29 204 establishment of protein localization to organelle
GO:0006401 2.50332734036146e-48 335.171266233766 0.510691301640025
29 205 RNA catabolic process
GO:0022415 1.44074904466958e-47 308.369565217391 0.647706041104422
30 260 viral reproductive process
GO:0071845 2.34920545731904e-46 278.759842519685 0.707494291052522
30 284 cellular component disassembly at cellular level
GO:0022411 3.6505774531439e-46 274.360465116279 0.717458999377206
30 288 cellular component disassembly
GO:0016032 4.24588308651024e-40 166.199524940618 1.12352086360805
30 451 viral reproduction
GO:0033365 1.84034379124319e-39 157.697516930023 1.17832675939381
30 473 protein localization to organelle
GO:0006605 1.96387618168055e-39 157.331081081081 1.18081793647498
30 474 protein targeting
GO:0042254 2.5066330389749e-09 28.9240121580547 0.371185385094457
8 149 ribosome biogenesis
GO:0022613 2.99435719193124e-09 21.912037037037 0.560514843263442 9
225 ribonucleoprotein complex biogenesis
GO:0071843 5.0824334643201e-09 20.5579710144928 0.595391322399834
9 239 cellular component biogenesis at cellular level
GO:0042273 1.5826893613868e-08 225.109375 0.0298941249740502 4
12 ribosomal large subunit biogenesis
GO:0006364 1.96388879581855e-07 29.2183673469388 0.259082416441769
6 104 rRNA processing
GO:0016072 2.74257268012292e-07 27.5211538461538 0.274029478928794
6 110 rRNA metabolic process
GO:0034470 8.98402702968043e-06 14.660824742268 0.498235416234171 6
200 ncRNA processing
GO:0042274 1.12920216089308e-05 87.2727272727273 0.0448411874610754
3 18 ribosomal small subunit biogenesis
Tissue: Adipose_Subcutaneous=>Nerve_Tibial Type: asymmetric
SourceGene: RABGAP1(ENSG00000011454.11)
TargetGeneSet: FUCA1 KPNA7 TMEM234 RPS8 RPL5 RP11 RPS27 ADCK3
FAM179A RPLP0P6 IGKV4-1 HNRNPA3 SLC40A1 ZNF197 IMPDH2 RPL29 PRICKLE2-AS2 CNBP
KLF3 CWC27 COL4A3BP EPB41L4A-AS1 CAMLG RPL15P3 NKAPL MICB
RPS10 RPL10A TRIM73 CALU EPHA1 EIF3E RPL12 RPL7A FUT7 FBXL15
EIF3M C1QTNF4 MMP8 IL10RA RPL18AP3 C12orf47 RPL6 RPLP0
ATP8A2 ABHD12B BMF STRC STRCP1 CHD2 NME3 IGFALS RPS2 RPL13
RPL17P43 RPL19 GPX4 RPL36 FUT3 ZNF562 CTD SLC1A6 HPN
RPL18 RPL13A RPS5 DBNDD2 RPL12P4 NPEPL1 LINC00316 PIK3IP1 CELSR1
TRABD IGBP1 TTC3P1 RPL10
GOBPID Pvalue OddsRatio ExpCount Count Size Term
GO:0006414 3.1405522127058e-27 81.0474060822898 0.421424122898069
19 105 translational elongation
GO:0006415 1.50229988544227e-26 88.2739726027397 0.365234239844993
18 91 translational termination
GO:0000184 4.05336502099694e-26 69.6325641025641 0.477614005951145
19 119 nuclear-transcribed mRNA catabolic process, nonsense-mediated decay

GO:0006413 9.97556369848819e-26 56.8620414673046 0.610061587433396
20 152 translational initiation
GO:0006614 2.45585809260828e-25 73.9965517241379 0.421424122898069
18 105 SRP-dependent cotranslational protein targeting to membrane
GO:0006613 2.95038613061024e-25 73.1505681818182 0.425437685973289
18 106 cotranslational protein targeting to membrane
GO:0045047 2.95038613061024e-25 73.1505681818182 0.425437685973289
18 106 protein targeting to ER
GO:0072599 2.95038613061024e-25 73.1505681818182 0.425437685973289
18 106 establishment of protein localization to endoplasmic reticulum
GO:0070972 2.3215428004227e-24 64.3185 0.473600442875926 18 118
protein localization to endoplasmic reticulum
GO:0019080 4.8191242550693e-24 52.6338383838384 0.606048024358176
19 151 viral genome expression
GO:0019083 4.8191242550693e-24 52.6338383838384 0.606048024358176
19 151 viral transcription
GO:0000956 4.45463458394984e-23 46.2593162393162 0.678292159712131
19 169 nuclear-transcribed mRNA catabolic process
GO:0006402 1.2323725263997e-22 43.6132881793259 0.714414227389108
19 178 mRNA catabolic process
GO:0006612 3.14379795856105e-22 47.5266666666667 0.614075150508615
18 153 protein targeting to membrane
GO:0043624 3.14379795856105e-22 47.5266666666667 0.614075150508615
18 153 cellular protein complex disassembly
GO:0043241 5.72732878705262e-22 45.8132142857143 0.634142965884714
18 158 protein complex disassembly
GO:0006401 1.92495522665908e-21 37.2116073890267 0.82278043042004
19 205 RNA catabolic process
GO:0034623 3.42937945510513e-21 41.0682692307692 0.698359975088229
18 174 cellular macromolecular complex disassembly
GO:0032984 5.78563282670552e-21 39.7788819875776 0.718427790464328
18 179 macromolecular complex disassembly
GO:0019058 1.92132847039728e-20 32.5881712626996 0.927133070375753
19 231 viral infectious cycle
GO:0071845 3.57280565206865e-20 28.1678628389155 1.1398519133624 20
284 cellular component disassembly at cellular level
GO:0022411 4.72376399290811e-20 27.7395915161037 1.15590616566328
20 288 cellular component disassembly
GO:0072594 6.36775219951183e-20 34.3717741935484 0.81876686734482
18 204 establishment of protein localization to organelle
GO:0022415 1.83835167582575e-19 28.6081498031706 1.04352639955712
19 260 viral reproductive process
GO:0016032 3.13296268580322e-16 17.0497008181707 1.81011694692409
20 451 viral reproduction
GO:0033365 1.23904310938058e-14 14.9576979554953 1.89841533457892
19 473 protein localization to organelle
GO:0006605 1.28737847597981e-14 14.923753169907 1.90242889765414 19
474 protein targeting
Tissue: Nerve_Tibial=>Adipose_Subcutaneous Type: asymmetric
SourceGene: RP11-340F14.5(ENSG00000261827.1)
TargetGeneSet: C1orf174 MFAP2 RPL11 SYF2 TMEM222 RP4 TRAPPC3
C1orf109 RRAGC CCDC24 USP1 GTF2B RP11 GOLPH3L CTSO UFC1 NENF
DDIT3 MPHOSPH10 RPL31 LIMS2 UBE2E3 RPL32 EIF1B RPL14 SS18L2
RPL24 CDV3 ACPL2 RNF7 COMMD2 RPL22L1 SENP2 RPL35A SLBP CNO
LAMTOR3 SLC9B2 RPL34 CNGB1 DCTD BTF3 EPB41L4A-AS1 CTD HINT1
UBE2D2 CCNG1 NUDCD2 NPM1 BOD1 RNF130 GABBR1 LYPLA2P1 RPL39P3
RAB32 TNRC18 TMEM60 DMTF1 C7orf23 TRIM4 RB1CC1 RPS20 NSMAF SNHG6
RPL30 RNF139 TOMM5 ALG2 SURF2 FAM107B VPS26A RPL13AP5 FAM204A
RPS13 EIF3M C11orf55 RASGRP2 BRMS1 ANKRD49 CWC15 RPS25 RPUSD4
PCBP2 GTSF1 PA2G4 NACA CDK4 CCDC59 METAP2 ALKBH2 PPP1CC SLC15A4
POLR1D WBP4 TPT1 MED4 NGDN RPS29 CGRRF1 TIMM9 MED6 PSEN1
IGHV3-49 RSL24D1 PYGO1 USP3 SEC11A RGS11 ARHGDIG PDIA2 METTL9
LCMT1 AQP8 DNAJA2 CRNDE CMTM3 NOB1 ZDHHC7 CTNS TMEM220 C17orf79

TMEM98 ZNHIT3 RPL17 RPL19 DNAJC7 PTRH2 MYO15B SPIRE1 SLC39A6 KDSR
TMED1 URI1 RPS16 ZNF230 DMWD TRPM4 RPS11 CCDC106 CHMP4B EIF2S2
RNF114 RPS21 MIS18A DSCR3 PSMG1 U2AF1 CCDC117 TOM1 ST13 PHF5A
GOBPID Pvalue OddsRatio ExpCount Count Size Term
GO:0070972 1.40030546437951e-19 27.043504350435 0.979862985260536 19
118 protein localization to endoplasmic reticulum
GO:0006614 4.15334989408457e-19 28.8924949290061 0.871911978409799
18 105 SRP-dependent cotranslational protein targeting to membrane
GO:0006613 4.96926750983946e-19 28.5621657754011 0.880215902013701
18 106 cotranslational protein targeting to membrane
GO:0045047 4.96926750983946e-19 28.5621657754011 0.880215902013701
18 106 protein targeting to ER
GO:0072599 4.96926750983946e-19 28.5621657754011 0.880215902013701
18 106 establishment of protein localization to endoplasmic reticulum
GO:0006415 9.08105050190388e-19 31.7986092889006 0.755657047955159
17 91 translational termination
GO:0006413 9.23301399557737e-19 21.5136363636364 1.26219638779323
20 152 translational initiation
GO:0006414 1.17795622861272e-17 26.7134819064431 0.871911978409799
17 105 translational elongation
GO:0006612 2.22075526967663e-17 19.9307669572927 1.27050031139714
19 153 protein targeting to membrane
GO:0000184 1.06524052959813e-16 23.0242718446602 0.988166908864438
17 119 nuclear-transcribed mRNA catabolic process, nonsense-mediated decay
GO:0072594 3.31139938655963e-16 15.3771739130435 1.69400041519618
20 204 establishment of protein localization to organelle
GO:0019080 6.39840647836301e-15 17.4865236922185 1.25389246418933
17 151 viral genome expression
GO:0019083 6.39840647836301e-15 17.4865236922185 1.25389246418933
17 151 viral transcription
GO:0043624 7.99513633158207e-15 17.2269417475728 1.27050031139714
17 153 cellular protein complex disassembly
GO:0043241 1.37602109238743e-14 16.6102045032018 1.31201992941665
17 158 protein complex disassembly
GO:0000956 4.25718658347756e-14 15.39620592744 1.40336308905958 17
169 nuclear-transcribed mRNA catabolic process
GO:0034623 6.92373527797761e-14 14.9006245748562 1.44488270707909
17 174 cellular macromolecular complex disassembly
GO:0006402 1.01031256913254e-13 14.5263221371284 1.47809840149471
17 178 mRNA catabolic process
GO:0032984 1.10875682814447e-13 14.43563466379 1.48640232509861 17
179 macromolecular complex disassembly
GO:0006401 1.03190226806756e-12 12.416391241479 1.70230433880008 17
205 RNA catabolic process
GO:0019058 7.11829907585459e-12 10.8878050993558 1.91820635250156
17 231 viral infectious cycle
GO:0033365 4.25565290545712e-11 6.90890990542558 3.92775586464605
22 473 protein localization to organelle
GO:0022415 4.67072579848617e-11 9.5687402612969 2.15902013701474 17
260 viral reproductive process
GO:0071845 1.86212395100869e-10 8.69379295298353 2.35831430350841
17 284 cellular component disassembly at cellular level
GO:0022411 2.31415006818054e-10 8.56303514491456 2.39152999792402
17 288 cellular component disassembly
GO:0006605 1.97555401668506e-09 6.11321585903084 3.93605978824995
20 474 protein targeting
GO:0016032 3.25677854497197e-08 5.66417606303491 3.74506954536018
18 451 viral reproduction
GO:0042254 4.41946260253503e-07 9.28188358404186 1.23728461698152
10 149 ribosome biogenesis
GO:0022613 2.67646245926574e-06 6.65724942124668 1.86838281087814
11 225 ribonucleoprotein complex biogenesis
GO:0071843 4.78680650115741e-06 6.24227426364075 1.98463774133278

11	239	cellular component biogenesis at cellular level
Tissue: Nerve_Tibial=>Adipose_Subcutaneous	Type:	asymmetric
SourceGene:	RP11-516A11.1(ENSG00000228328.2)
TargetGeneSet:	ARID1A	RPS8	C1orf68	RPL29	RPL34	RPS3A	AFF4	RP11
LY6G6E	FAM135A	RPS6	TPT1	RPS3AP6	RPL4	IRF8	RPL13	RPL27	GLTSCR2
STK35	RPL39
GOBPID	Pvalue	OddsRatio	ExpCount	Count	Size	Term
GO:0006415	1.14499153212428e-16	225.047038327526	0.100754273060688
9	91	translational termination
GO:0034623	3.71475798518804e-16	145.030487804878	0.192651027610546
10	174	cellular macromolecular complex disassembly
GO:0006414	4.36314244552865e-16	192.040178571429	0.11625493045464
9	105	translational elongation
GO:0006614	4.36314244552865e-16	192.040178571429	0.11625493045464
9	105	SRP-dependent cotranslational protein targeting to membrane
GO:0006613	4.76587842681298e-16	190.047128129602	0.117362120268494
9	106	cotranslational protein targeting to membrane
GO:0045047	4.76587842681298e-16	190.047128129602	0.117362120268494
9	106	protein targeting to ER
GO:0072599	4.76587842681298e-16	190.047128129602	0.117362120268494
9	106	establishment of protein localization to endoplasmic reticulum
GO:0032984	4.95900343977751e-16	140.69033530572	0.198186976679815	10
179	macromolecular complex disassembly
GO:0070972	1.29076584023977e-15	168.982961992136	0.130648398034738
9	118	protein localization to endoplasmic reticulum
GO:0000184	1.39576090835934e-15	167.435064935065	0.131755587848592
9	119	nuclear-transcribed mRNA catabolic process, nonsense-mediated decay
GO:0019080	1.25504809296364e-14	129.413480885312	0.167185661891911
9	151	viral genome expression
GO:0019083	1.25504809296364e-14	129.413480885312	0.167185661891911
9	151	viral transcription
GO:0006413	1.33345031607535e-14	128.4995004995	0.168292851705764	9
152	translational initiation
GO:0006612	1.4161677045545e-14	127.598214285714	0.169400041519618
9	153	protein targeting to membrane
GO:0043624	1.4161677045545e-14	127.598214285714	0.169400041519618
9	153	cellular protein complex disassembly
GO:0043241	1.9019830452476e-14	123.273250239693	0.174935990588887
9	158	protein complex disassembly
GO:0000956	3.52243611240938e-14	114.709821428571	0.187115078541277
9	169	nuclear-transcribed mRNA catabolic process
GO:0071845	5.29855399750741e-14	86.1374695863747	0.314441907134454
10	284	cellular component disassembly at cellular level
GO:0006402	5.65922752959147e-14	108.532544378698	0.197079786865961
9	178	mRNA catabolic process
GO:0022411	6.09830219220966e-14	84.8741007194245	0.318870666389869
10	288	cellular component disassembly
GO:0072594	1.95978694839734e-13	93.8901098901099	0.225866722026157
9	204	establishment of protein localization to organelle
GO:0006401	2.04887268316851e-13	93.4045189504373	0.226973911840011
9	205	RNA catabolic process
GO:0019058	6.05431774037828e-13	82.3146718146718	0.255760847000208
9	231	viral infectious cycle
GO:0022415	1.76390039952359e-12	72.6556630620376	0.287869351601965
9	260	viral reproductive process
GO:0016032	2.44498073565706e-10	40.7036199095023	0.499342606048024
9	451	viral reproduction
GO:0033365	3.73067412356996e-10	38.7127463054187	0.523700781952806
9	473	protein localization to organelle
GO:0006605	3.80117561337389e-10	38.6267281105991	0.52480797176666
9	474	protein targeting
Tissue: Nerve_Tibial=>Adipose_Subcutaneous	Type:	asymmetric
SourceGene:	RP4-792G4.2(ENSG00000230798.1)

TargetGeneSet: IGSF21 RPL11 RP4 TRAPPC3 RPS8 UQCRH RPL5 RP11
HBXIP MRPS21 CTSO UFC1 CYB5R1 DDIT3 UBC IGKV1D-39 RPL31
C2orf49 MMADHC C2orf47 RPL37A RPL32 UBE2E1 EIF1B RPL24 CNBP RNF7 CPB1
C3orf55 DNAJC19 RPL35A C4orf52 ENOPH1 RPL34 CICP16 SGTB TAF9 BTF3
COX7C CTD SNX2 HINT1 SKP1 CTB RPS14 ATP6V0E1 CUTA
RPS10 RPL10A RPL39P3 MRPS12 ABCB5 TMEM60 DMTF1 RPL19P12 SRPK2
RPS20 SNHG6 RPL30 EIF3H EEF1D RPL8 RPS6 NDUFB6 CHCHD2P9
SEC61B PSMB7 RPL35 BAMBI ZFAND4 RPS24 ART5 RPL27A RPS13 EIF3M
MRPL16 LAMTOR1 CLNS1A TMEM126A C11orf93 ATP5L RPS25 C12orf57
CSAD PFDN5 CNPY2 NACA C12orf51 MRP63 EIF4A1P7 TPT1 ESD
COMMD6 EAPP RPS29 RPL36A RPS3AP6 RPS27L RPLP1 MORF4L1 C15orf40
RPS15A METTL9 RPL26 RPS7P1 RPL23A RPL17 RPL19 KRT10 RPL27 RPS15
RPL36 TMED1 UQCRFS1 RPS19 LYPD3 SNRPD2 RPL18 RPS11 KLK5 EIF2S2
RNF114 DPM1 RPS21 ST13 XRCC6 FUNDC1 UXT RPS4X RPL39
GOBPID Pvalue OddsRatio ExpCount Count Size Term
GO:0006415 4.58020427852195e-59 146.366576819407 0.680091343159643
38 91 translational termination
GO:0006414 3.38213362304763e-58 122.266798418972 0.784720780568819
39 105 translational elongation
GO:0006614 3.38213362304763e-58 122.266798418972 0.784720780568819
39 105 SRP-dependent cotranslational protein targeting to membrane
GO:0006613 5.32575348629716e-58 120.433484750162 0.792194311812331
39 106 cotranslational protein targeting to membrane
GO:0045047 5.32575348629716e-58 120.433484750162 0.792194311812331
39 106 protein targeting to ER
GO:0072599 5.32575348629716e-58 120.433484750162 0.792194311812331
39 106 establishment of protein localization to endoplasmic reticulum
GO:0006413 1.7805400085611e-56 82.3396694214876 1.13597674901391
42 152 translational initiation
GO:0070972 8.3862438871158e-56 102.053935057788 0.881876686734482
39 118 protein localization to endoplasmic reticulum
GO:0000184 1.21871333762456e-53 95.5830687830688 0.889350217977995
38 119 nuclear-transcribed mRNA catabolic process, nonsense-mediated decay
GO:0006612 1.07136259207137e-50 70.5480549199085 1.14345028025742
39 153 protein targeting to membrane
GO:0019080 4.20629708041496e-49 68.3615676359039 1.1285032177704 38
151 viral genome expression
GO:0019083 4.20629708041496e-49 68.3615676359039 1.1285032177704 38
151 viral transcription
GO:0043624 7.39063490526272e-49 67.1632298136646 1.14345028025742
38 153 cellular protein complex disassembly
GO:0043241 2.91462560720417e-48 64.3421428571429 1.18081793647498
38 158 protein complex disassembly
GO:0000956 5.02992104891155e-47 58.8937840785169 1.26302678015362
38 169 nuclear-transcribed mRNA catabolic process
GO:0072594 5.23455908171374e-47 50.8572453371593 1.52460037367656
40 204 establishment of protein localization to organelle
GO:0034623 1.7113446454692e-46 56.7086134453781 1.30039443637119
38 174 cellular macromolecular complex disassembly
GO:0019058 2.47949418495197e-46 45.5831107619796 1.7263857172514 41
231 viral infectious cycle
GO:0006402 4.42817693186929e-46 55.0728571428571 1.33028856134524
38 178 mRNA catabolic process
GO:0032984 5.59474172456195e-46 54.6784194528875 1.33776209258875
38 179 macromolecular complex disassembly
GO:0022415 4.32279565024158e-44 39.4659578818238 1.94311812331327
41 260 viral reproductive process
GO:0006401 1.51876948980354e-43 46.0810949529512 1.53207390492007
38 205 RNA catabolic process
GO:0016032 4.45820144890594e-38 23.5405405405405 3.37056259082416
44 451 viral reproduction
GO:0071845 7.23985373208038e-38 31.108362369338 2.12248287315757 38
284 cellular component disassembly at cellular level

GO:0022411 1.25473837791022e-37 30.6019428571429 2.15237699813162
38 288 cellular component disassembly
GO:0033365 3.4103874100032e-33 19.7053275290216 3.53498027818144
41 473 protein localization to organelle
GO:0006605 3.71587653115593e-33 19.6584054324222 3.54245380942495
41 474 protein targeting
GO:0042274 3.07610727149457e-13 114.664 0.134523562383226 8 18
ribosomal small subunit biogenesis
GO:0022613 1.00265578829395e-11 11.7611816101519 1.68154452979033
16 225 ribonucleoprotein complex biogenesis
GO:0071843 2.49966109415737e-11 11.0118931565607 1.7861739671995 16
239 cellular component biogenesis at cellular level
GO:0006364 3.02294246046408e-10 17.3761223811107 0.777247249325306
11 104 rRNA processing
GO:0016072 5.56765771310408e-10 16.3161512027491 0.822088436786382
11 110 rRNA metabolic process
GO:0042254 1.10251701938669e-09 12.9616788321168 1.11355615528337
12 149 ribosome biogenesis
GO:0030490 1.01492952635634e-07 183.846153846154 0.0523147187045879
4 7 maturation of SSU-rRNA
GO:0034660 2.00803015829786e-07 7.07906346749226 2.12995640440108
13 285 ncRNA metabolic process
GO:0034470 2.90059233058808e-07 8.49255441008018 1.49470624870251
11 200 ncRNA processing
GO:0042273 1.39457871039722e-06 68.9182692307692 0.0896823749221507
4 12 ribosomal large subunit biogenesis
GO:0022618 6.89193924097439e-06 11.2269351935194 0.709985468133693
7 95 ribonucleoprotein complex assembly
GO:0000028 7.98768948879993e-06 136.571428571429 0.0448411874610754
3 6 ribosomal small subunit assembly
GO:0000462 7.98768948879993e-06 136.571428571429 0.0448411874610754
3 6 maturation of SSU-rRNA from tricistronic rRNA transcript (SSU-rRNA, 5.8S rRNA, LSU-rRNA)
GO:0071826 9.68333273999527e-06 10.6196103481316 0.747353124351256
7 100 ribonucleoprotein complex subunit organization
Tissue: Skin_Sun_Exposed_Lower_leg=>Adipose_Subcutaneous Type: asymmetric
SourceGene: BACH1-IT1(ENSG00000248476.1)
TargetGeneSet: ATAD3A MRTO4 GMEB1 U6 ATXN7L2 WDR3 GPATCH4 COG2
C1orf124 PPM1G PNO1 POLR1A SNRNP200 POLR1B IWS1 SACM1L
IFRD2 RRP9 STAG1 GFM1 LSG1 FAM193A NOP14 HNRNPD NAA15 NSUN2
UTP15 HARS TCOF1 MAPK9 RIOK1 BAG6 WDR46 XPO5 SYNCRIP PUS7 RP11
KAT6A KIAA2026 BAG1 NOL6 TESK1 CIZ1 ZMYND19 RRP12 CHUK
PPRC1 NOLC1 EIF6 SEC23IP CARS NUP98 SF1 KLC2 DDX10 NOP2
DDX23 MARS UTP20 ANAPC7 DDX55 PUS1 EP400 TSC22D1 KTN1-AS1
ZNF598 TRAP1 USP7 FBRS SRCAP SETD1A E2F4 ZC3H18 MYBBP1A DHX33 PEMT
UTP6 TADA2A SEH1L DNMT1 PDE4A ILF3 TNPO2 CC2D1A ZNF333 CCDC97
TOMM40 CD3EAP DHX34 GRWD1 ZNF335 DDX27 SCAF4 RRP1B RRP1 PWP2
DEPDC5 PPP6R2 TIMM8A
GOBPID Pvalue OddsRatio ExpCount Count Size Term
GO:0071843 5.95310092169323e-15 18.0618618618619 1.27347588402187
17 239 cellular component biogenesis at cellular level
GO:0022613 4.12246425166107e-14 17.7770805553377 1.19887897031347
16 225 ribonucleoprotein complex biogenesis
GO:0042254 9.14114553339902e-13 21.2653952205882 0.793924295896478
13 149 ribosome biogenesis
GO:0034660 1.59772681679339e-12 13.753427996831 1.51858002906373 16
285 ncRNA metabolic process
GO:0034470 3.9079936678959e-11 15.4103442513369 1.0656701958342 13
200 ncRNA processing
GO:0006364 1.76290035351077e-10 22.6738647189584 0.554148501833783
10 104 rRNA processing
GO:0016072 3.08801441221038e-10 21.3044776119403 0.586118607708809
10 110 rRNA metabolic process

GO:0006473	5.21450453179506e-06	11.7793388429752	0.682028925333887
7	128	protein acetylation
GO:0043543	1.1323242897632e-05	10.3919708029197	0.767282541000623
7	144	protein acylation
Tissue: Skin_Sun_Exposed_Lower_leg=>Adipose_Subcutaneous	Type:	cluster
SourceGene:	BACH1-IT1(ENSG00000248476.1)
TargetGeneSet:	ATAD3A	MRTO4	GMEB1	U6	ATXN7L2	WDR3	GPATCH4	COG2
C1orf124	PPM1G	PNO1	POLR1A	SNRNP200	POLR1B	IWS1	SACM1L
IFRD2	RRP9	STAG1	GFM1	LSG1	FAM193A	NOP14	HNRNPD	NAA15	NSUN2
UTP15	HARS	TCOF1	MAPK9	RIOK1	BAG6	WDR46	XPO5	SYNCRIP	PUS7	RP11
KAT6A	KIAA2026	BAG1	NOL6	TESK1	CIZ1	ZMYND19	RRP12	CHUK
PPRC1	NOLC1	EIF6	SEC23IP	CARS	NUP98	SF1	KLC2	DDX10	NOP2
DDX23	MARS	UTP20	ANAPC7	DDX55	PUS1	EP400	TSC22D1	KTN1-AS1
ZNF598	TRAP1	USP7	FBRS	SRCAP	SETD1A	E2F4	ZC3H18	MYBBP1A	DHX33	PEMT
UTP6	TADA2A	SEH1L	DNMT1	PDE4A	ILF3	TNPO2	CC2D1A	ZNF333	CCDC97
TOMM40	CD3EAP	DHX34	GRWD1	ZNF335	DDX27	SCAF4	RRP1B	RRP1	PWP2
DEPDC5	PPP6R2	TIMM8A
GOBPID	Pvalue	OddsRatio	ExpCount	Count	Size	Term
GO:0071843	5.95310092169323e-15	18.0618618618619	1.27347588402187
17	239	cellular component biogenesis at cellular level
GO:0022613	4.12246425166107e-14	17.7770805553377	1.19887897031347
16	225	ribonucleoprotein complex biogenesis
GO:0042254	9.14114553339902e-13	21.2653952205882	0.793924295896478
13	149	ribosome biogenesis
GO:0034660	1.59772681679339e-12	13.753427996831	1.51858002906373	16
285	ncRNA metabolic process
GO:0034470	3.9079936678959e-11	15.4103442513369	1.0656701958342	13
200	ncRNA processing
GO:0006364	1.76290035351077e-10	22.6738647189584	0.554148501833783
10	104	rRNA processing
GO:0016072	3.08801441221038e-10	21.3044776119403	0.586118607708809
10	110	rRNA metabolic process
GO:0006473	5.21450453179506e-06	11.7793388429752	0.682028925333887
7	128	protein acetylation
GO:0043543	1.1323242897632e-05	10.3919708029197	0.767282541000623
7	144	protein acylation
Tissue: Skin_Sun_Exposed_Lower_leg=>Adipose_Subcutaneous	Type:	asymmetric
SourceGene:	CDSN(ENSG00000204539.3)
TargetGeneSet:	SLC35E2B	RP11	PLA2G2F	RP4	RPL5	TSHB	RAB25
SPATA17	RHOQ	UBC	ASPRV1	TPRKB	PMS1	EEF1B2	RPL29	SPCS1	RPL24
ZBTB38	RPL35A	NUP54	RPL34	OSTC	FRG1	CTB	RPS14	GPLD1	HIST1H1B
RPL10A	DST	SENP6	MRPS12	AIG1	HOXA5	NT5C3	GRB10	PRKAR2B	SSBP1	RPS6
HRCT1	DAPK1	RPL12	RPL7A	C9orf104	ITGB1	RPL13AP5	BLOC1S2
ZBED5	PEX16	POLR2G	RPS25	WNT5B	HNRNPA1	SNRPF	RPL18AP3	C12orf45
RPL6	RPLP0	ALG5	EXOC5	ZNF280D	MYO1E	RPS3AP6	TIPIN	RPS2	LITAF
RPL13	CTD	RPL19	DUSP3	ANKRD40	NT5C	TMEM241	HNRNPA1P7	PIP5K1C
ZNF823	ZNF44	RNASEH2A	RPS19	PLA2G4C	RPL18	TEAD2	RPS5	RPL12P4
MRPL39	RPL3	ATXN10	FOXO4
GOBPID	Pvalue	OddsRatio	ExpCount	Count	Size	Term
GO:0070972	4.01447120634584e-31	69.1597894736842	0.596083316033493
23	118	protein localization to endoplasmic reticulum
GO:0006414	1.63532109211547e-30	74.2948263642807	0.530413120199294
22	105	translational elongation
GO:0006614	1.63532109211547e-30	74.2948263642807	0.530413120199294
22	105	SRP-dependent cotranslational protein targeting to membrane
GO:0006613	2.0566077911272e-30	73.4052287581699	0.535464673725002
22	106	cotranslational protein targeting to membrane
GO:0045047	2.0566077911272e-30	73.4052287581699	0.535464673725002
22	106	protein targeting to ER
GO:0072599	2.0566077911272e-30	73.4052287581699	0.535464673725002
22	106	establishment of protein localization to endoplasmic reticulum
GO:0006415	4.27946954834511e-30	82.5461538461538	0.459691370839388
21	91	translational termination

GO:0006612 2.44265744126145e-28 50.416 0.772887689433257 23 153
protein targeting to membrane
GO:0000184 2.03494710616023e-27 58.8461538461538 0.6011348695592 21
119 nuclear-transcribed mRNA catabolic process, nonsense-mediated decay
GO:0019080 8.8461963931469e-27 47.6482748138015 0.762784582381842
22 151 viral genome expression
GO:0019083 8.8461963931469e-27 47.6482748138015 0.762784582381842
22 151 viral transcription
GO:0072594 2.41925650647136e-25 36.080773480663 1.03051691924434 23
204 establishment of protein localization to organelle
GO:0006413 4.75051194318713e-25 43.9205813270699 0.76783613590755
21 152 translational initiation
GO:0043624 5.48718732480764e-25 43.5847902097902 0.772887689433257
21 153 cellular protein complex disassembly
GO:0043241 1.11119910296306e-24 41.979365524986 0.798145457061795 21
158 protein complex disassembly
GO:0000956 4.83123073510223e-24 38.829261954262 0.853712545844578 21
169 nuclear-transcribed mRNA catabolic process
GO:0034623 9.1051683141697e-24 37.5471342383107 0.878970313473116
21 174 cellular macromolecular complex disassembly
GO:0006401 9.51942795376862e-24 33.4608378870674 1.03556847277005
22 205 RNA catabolic process
GO:0006402 1.49043956798558e-23 36.5802302792749 0.899176527575946
21 178 mRNA catabolic process
GO:0032984 1.68267352320826e-23 36.3461538461538 0.904228081101654
21 179 macromolecular complex disassembly
GO:0019058 1.3797664014186e-22 29.2445820433437 1.16690886443845
22 231 viral infectious cycle
GO:0022415 1.8967345325648e-21 25.6286043829296 1.31340391668397
22 260 viral reproductive process
GO:0071845 3.00383735976349e-19 21.6741006142147 1.43464120130095
21 284 cellular component disassembly at cellular level
GO:0022411 4.01641446014241e-19 21.3433448573898 1.45484741540378
21 288 cellular component disassembly
GO:0006605 3.61892328803731e-18 15.1597278911565 2.39443637118538
24 474 protein targeting
GO:0016032 1.80512837404464e-17 14.9929906542056 2.27825064009411
23 451 viral reproduction
GO:0033365 5.1442244587579e-17 14.2375111111111 2.38938481765968
23 473 protein localization to organelle
GO:0071843 2.39667406935383e-09 12.2634505669098 1.20732129264411
12 239 cellular component biogenesis at cellular level
GO:0022613 1.55708901662507e-08 11.7428399155864 1.1365995432842 11
225 ribonucleoprotein complex biogenesis
GO:0042254 5.72128400092112e-08 14.3015625 0.752681475330427 9
149 ribosome biogenesis
GO:0042274 1.73804767524059e-06 59.4782608695652 0.0909279634627361
4 18 ribosomal small subunit biogenesis
GO:0000028 2.44679800247836e-06 205.357142857143 0.0303093211542454
3 6 ribosomal small subunit assembly
GO:0006364 1.38417502241937e-05 13.0490405117271 0.525361566673587
6 104 rRNA processing
GO:0016072 1.90780185477206e-05 12.2910447761194 0.555670887827832
6 110 rRNA metabolic process
Tissue: Skin_Sun_Exposed_Lower_leg=>Adipose_Subcutaneous Type: asymmetric
SourceGene: FLG(ENSG00000143631.9)
TargetGeneSet: TNFRSF25 VPS13D UBR4 SH3BGRL3 KHDRBS1 KPNA7
TMEM234 EIF2C3 MACF1 PPCS RPS8 TXNDC12 EFCAB7 TCTEX1D1 RP4 RPL5
RP11 DPH5 ATP5F1 HIPK1 POGZ RPS27 MTX1 PRRC2C PIK3C2B LPGAT1
ZC3H11B HIST3H2A YWHAQ SOS1-IT1 CCDC104 AAK1 ANKRD36BP2
PDE11A EEF1B2 ITPR1 SETD5 RPL15 GOLGA4 RPSA RPL14 ZNF445 NICN1 RBM6
RPL29 SPCS1 ATXN7 PCNP ALDH1L1-AS1 H1FX IGSF10 RPL22L1 AFAP1
RELL1 OCIAD1 PPA2 CCDC109B RPS3A CTSO WWC2 TAF9 BTF3

ZFYVE16 CTD AFF4 SKP1 CTB SYNPO UBLCP1 RPL15P3 RPL10A GLO1
YIPF3 EEF1A1 VTA1 HIVEP2 ARID1B MLLT4 NPM1P13 EEF1A1P6 NT5C3
RPS3AP26 BUD31 MUC3A SH2B2 ARMC10 MLL3 CHMP7 HMBOX1 KAT6A
HOOK3 CPNE3 EIF3E EBAG9 EIF3H TATDN1 LY6E RPL8 RPS6 TESK1 TLN1
CLTA LINC00092 KLF4 EEF1A1P5 RPL7A U6 RPS3AP5 TCTN3
C10orf12 SFR1 NHLRC2 RPL27A ZBED5 EIF3M PEX16 CELF1 TMEM179B
NAA40 PPP2R5B C11orf2 C11orf30 GUCY1A2 WNT5B PHB2 H3F3C ADCY6
SMARCC2 NACA RPL18AP3 ATXN2 RPL6 RPLP0 MLXIP RPSAP54 ALG5 DGKH
THSD1P1 EAPP LGALS3 SIPA1L1 TTBK2 RPS3AP47 CCNDBP1 COPS2 RSL24D1
RAB11A LRRC49 ZNF592 RPS2 LITAF NDE1 ARL6IP1 EIF3C DDX19B RPL13
SGSM2 RPL26 STX8 NCOR1 RPL17P43 TAOK1 RPL19 SMARCE1 RAMP2
VPS25 AOC3 ANKRD40 CEP95 PRKCA NT5C FOXK2 MPPE1 PSMG2 C18orf21
RPL17 RPS15 PIP5K1C RPL36 ALKBH7 HNRNPA1P10 LPHN1 RPL18A ZNF585A
MEGF8 ZNF221 PVR OPA3 RPL18 RPL13A ZNF551 RPS5 ZNF446 UBE2M DTD1
RPL41P1 RPL12P4 CBR3-AS1 TFF3 UBE2G2 YDJC ZNF70 LIMK2 EIF3L RPL3
TCF20 IGBP1 RPS23P8 RPS4X HK2P1 RP1-177G6.2 RPL10 SLC10A3
GOBPID Pvalue OddsRatio ExpCount Count Size Term
GO:0006414 4.19883651788368e-36 45.1099508599509 1.18434710400664
31 105 translational elongation
GO:0006413 5.16994216446099e-35 31.6502430692419 1.71448342675247
34 152 translational initiation
GO:0006415 6.61977574294832e-35 49.6574386133847 1.02643415680576
29 91 translational termination
GO:0006614 1.85699031974156e-34 42.7458646616541 1.18434710400664
30 105 SRP-dependent cotranslational protein targeting to membrane
GO:0070972 2.54104864908073e-34 38.3342911877395 1.33098055497889
31 118 protein localization to endoplasmic reticulum
GO:0006613 2.56679635719011e-34 42.1804511278196 1.19562660023528
30 106 cotranslational protein targeting to membrane
GO:0045047 2.56679635719011e-34 42.1804511278196 1.19562660023528
30 106 protein targeting to ER
GO:0072599 2.56679635719011e-34 42.1804511278196 1.19562660023528
30 106 establishment of protein localization to endoplasmic reticulum
GO:0000184 1.26820094811398e-32 35.9863140998564 1.34226005120753
30 119 nuclear-transcribed mRNA catabolic process, nonsense-mediated decay
GO:0006612 1.69750253516887e-30 27.2693740685544 1.72576292298111
31 153 protein targeting to membrane
GO:0072594 2.13473847346771e-30 21.8883720930233 2.30101723064148
34 204 establishment of protein localization to organelle
GO:0034623 1.15351800509854e-28 23.2302924348379 1.96263234378244
31 174 cellular macromolecular complex disassembly
GO:0006402 2.40969659025114e-28 22.5917851989281 2.00775032869698
31 178 mRNA catabolic process
GO:0032984 2.88836305185148e-28 22.4375511875512 2.01902982492561
31 179 macromolecular complex disassembly
GO:0019080 7.81204076880227e-28 25.1293124541228 1.70320393052384
29 151 viral genome expression
GO:0019083 7.81204076880227e-28 25.1293124541228 1.70320393052384
29 151 viral transcription
GO:0000956 1.06184197692109e-27 22.9604587007086 1.90623486263926
30 169 nuclear-transcribed mRNA catabolic process
GO:0043624 1.1697180491529e-27 24.7205103514685 1.72576292298111
29 153 cellular protein complex disassembly
GO:0043241 3.12606670420876e-27 23.7539627444174 1.78216040412428
29 158 protein complex disassembly
GO:0006401 2.21540800047131e-26 19.0497213514455 2.31229672687011
31 205 RNA catabolic process
GO:0019058 2.41564116429031e-22 15.0913994384513 2.60556362881462
29 231 viral infectious cycle
GO:0071845 5.30241404916631e-22 13.0280572523656 3.20337692893225
31 284 cellular component disassembly at cellular level
GO:0022411 8.08549203688693e-22 12.8216307039264 3.24849491384679
31 288 cellular component disassembly

GO:0022415	7.07728650055852e-21	13.1696388188926	2.93266901944502
29	260	viral reproductive process
GO:0033365	3.6363156943002e-20	8.98459431701472	5.33520171614421
36	473	protein localization to organelle
GO:0006605	3.90054463529613e-20	8.9634343652249 5.34648121237285	36
474	protein targeting
GO:0016032	5.5316886595421e-18	8.42307692307692	5.08705279911425
33	451	viral reproduction
GO:0071843	8.35096820201633e-08	6.36341698841699	2.69579959864369
15	239	cellular component biogenesis at cellular level
GO:0022613	1.60694009045159e-06	5.75433962264151	2.53788665144281
13	225	ribonucleoprotein complex biogenesis
GO:0042254	7.1899920759704e-06	6.65303051676306	1.68064493806657
10	149	ribosome biogenesis
Tissue: Skin_Sun_Exposed_Lower_leg=>Adipose_Subcutaneous	Type:	cluster
SourceGene:	FLG(ENSG00000143631.9)
TargetGeneSet:	TNFRSF25	VPS13D	UBR4	SH3BGRL3	KHDRBS1 KPNA7
TMEM234 EIF2C3	MACF1	PPCS	RPS8	TXNDC12 EFCAB7	TCTEX1D1	RP4	RPL5
RP11	DPH5	ATP5F1	HIPK1	POGZ	RPS27	MTX1	PRRC2C	PIK3C2B LPGAT1
ZC3H11B HIST3H2A	YWHAQ	SOS1-IT1	CCDC104 AAK1	ANKRD36BP2
PDE11A	EEF1B2	ITPR1	SETD5	RPL15	GOLGA4	RPSA	RPL14	ZNF445	NICN1	RBM6
RPL29	SPCS1	ATXN7	PCNP	ALDH1L1-AS1	H1FX	IGSF10	RPL22L1 AFAP1
RELL1	OCIAD1	PPA2	CCDC109B	RPS3A	CTSO	WWC2	TAF9	BTF3
ZFYVE16 CTD	AFF4	SKP1	CTB	SYNPO	UBLCP1	RPL15P3 RPL10A	GLO1
YIPF3	EEF1A1	VTA1	HIVEP2	ARID1B	MLLT4	NPM1P13 EEF1A1P6	NT5C3
RPS3AP26	BUD31	MUC3A	SH2B2	ARMC10	MLL3	CHMP7	HMBOX1	KAT6A
HOOK3	CPNE3	EIF3E	EBAG9	EIF3H	TATDN1	LY6E	RPL8	RPS6	TESK1	TLN1
CLTA	LINC00092	KLF4	EEF1A1P5	RPL7A	U6	RPS3AP5 TCTN3
C10orf12	SFR1	NHLRC2	RPL27A	ZBED5	EIF3M	PEX16	CELF1	TMEM179B
NAA40	PPP2R5B C11orf2 C11orf30	GUCY1A2 WNT5B	PHB2	H3F3C	ADCY6
SMARCC2 NACA	RPL18AP3	ATXN2	RPL6	RPLP0	MLXIP	RPSAP54 ALG5	DGKH
THSD1P1 EAPP	LGALS3	SIPA1L1 TTBK2	RPS3AP47	CCNDBP1 COPS2	RSL24D1
RAB11A	LRRC49	ZNF592	RPS2	LITAF	NDE1	ARL6IP1 EIF3C	DDX19B	RPL13
SGSM2	RPL26	STX8	NCOR1	RPL17P43	TAOK1	RPL19	SMARCE1 RAMP2
VPS25	AOC3	ANKRD40 CEP95	PRKCA	NT5C	FOXK2	MPPE1	PSMG2	C18orf21
RPL17	RPS15	PIP5K1C RPL36	ALKBH7	HNRNPA1P10	LPHN1	RPL18A	ZNF585A
MEGF8	ZNF221	PVR	OPA3	RPL18	RPL13A	ZNF551	RPS5	ZNF446	UBE2M	DTD1
RPL41P1 RPL12P4 CBR3-AS1	TFF3	UBE2G2	YDJC	ZNF70	LIMK2	EIF3L	RPL3
TCF20	IGBP1	RPS23P8 RPS4X	HK2P1	RP1-177G6.2	RPL10	SLC10A3
GOBPID	Pvalue	OddsRatio	ExpCount	Count	Size	Term
GO:0006414	4.19883651788368e-36	45.1099508599509	1.18434710400664
31	105	translational elongation
GO:0006413	5.16994216446099e-35	31.6502430692419	1.71448342675247
34	152	translational initiation
GO:0006415	6.61977574294832e-35	49.6574386133847	1.02643415680576
29	91	translational termination
GO:0006614	1.85699031974156e-34	42.7458646616541	1.18434710400664
30	105	SRP-dependent cotranslational protein targeting to membrane
GO:0070972	2.54104864908073e-34	38.3342911877395	1.33098055497889
31	118	protein localization to endoplasmic reticulum
GO:0006613	2.56679635719011e-34	42.1804511278196	1.19562660023528
30	106	cotranslational protein targeting to membrane
GO:0045047	2.56679635719011e-34	42.1804511278196	1.19562660023528
30	106	protein targeting to ER
GO:0072599	2.56679635719011e-34	42.1804511278196	1.19562660023528
30	106	establishment of protein localization to endoplasmic reticulum
GO:0000184	1.26820094811398e-32	35.9863140998564	1.34226005120753
30	119	nuclear-transcribed mRNA catabolic process, nonsense-mediated decay
GO:0006612	1.69750253516887e-30	27.2693740685544	1.72576292298111
31	153	protein targeting to membrane
GO:0072594	2.13473847346771e-30	21.8883720930233	2.30101723064148
34	204	establishment of protein localization to organelle
GO:0034623	1.15351800509854e-28	23.2302924348379	1.96263234378244

31	174	cellular macromolecular complex disassembly
GO:0006402	2.40969659025114e-28	22.5917851989281	2.00775032869698
31	178	mRNA catabolic process
GO:0032984	2.88836305185148e-28	22.4375511875512	2.01902982492561
31	179	macromolecular complex disassembly
GO:0019080	7.81204076880227e-28	25.1293124541228	1.70320393052384
29	151	viral genome expression
GO:0019083	7.81204076880227e-28	25.1293124541228	1.70320393052384
29	151	viral transcription
GO:0000956	1.06184197692109e-27	22.9604587007086	1.90623486263926
30	169	nuclear-transcribed mRNA catabolic process
GO:0043624	1.1697180491529e-27	24.7205103514685	1.72576292298111
29	153	cellular protein complex disassembly
GO:0043241	3.12606670420876e-27	23.7539627444174	1.78216040412428
29	158	protein complex disassembly
GO:0006401	2.21540800047131e-26	19.0497213514455	2.31229672687011
31	205	RNA catabolic process
GO:0019058	2.41564116429031e-22	15.0913994384513	2.60556362881462
29	231	viral infectious cycle
GO:0071845	5.30241404916631e-22	13.0280572523656	3.20337692893225
31	284	cellular component disassembly at cellular level
GO:0022411	8.08549203688693e-22	12.8216307039264	3.24849491384679
31	288	cellular component disassembly
GO:0022415	7.07728650055852e-21	13.1696388188926	2.93266901944502
29	260	viral reproductive process
GO:0033365	3.6363156943002e-20	8.98459431701472	5.33520171614421
36	473	protein localization to organelle
GO:0006605	3.90054463529613e-20	8.9634343652249 5.34648121237285	36
474	protein targeting
GO:0016032	5.5316886595421e-18	8.42307692307692	5.08705279911425
33	451	viral reproduction
GO:0071843	8.35096820201633e-08	6.36341698841699	2.69579959864369
15	239	cellular component biogenesis at cellular level
GO:0022613	1.60694009045159e-06	5.75433962264151	2.53788665144281
13	225	ribonucleoprotein complex biogenesis
GO:0042254	7.1899920759704e-06	6.65303051676306	1.68064493806657
10	149	ribosome biogenesis
Tissue: Adipose_Subcutaneous=>Skin_Sun_Exposed_Lower_leg	Type:	asymmetric
SourceGene:	HIST1H2BPS3(ENSG00000226908.1)
TargetGeneSet:	ARHGEF19-AS1	RPL11	ZCCHC17	RP11	PPIE	CCDC23	RPS8
UQCRH	BTF3L4	ZYG11A	HBXIP	MRPS21	PAQR6	DCAF6	OCLM	SNRPE	NFASC	SRP9
TOMM20	RPS7	UBC	COA5	TXNDC9	RPL31	ERCC3	BBS5	NIF3L1	NBEAL1
EEF1B2	CCDC51	RYK	CLDN18	EIF2S1	PARL	NCBP2	TMEM128	C4orf52	RPL34	OSTC
RPS3A	CDKN2AIP	CTD	TAF9	ZFYVE16	CAMLG	PDF	TMEM14C	TMEM14B
EEF1A1	IL6	RPS3AP26	GIGYF1	MRPS33	PRAGMIN	C8orf40	RPS20	PAF1	RPS6
CLTA	C10orf31	RPS24	C10orf95	FAM204A	FAM45A	PRKRIR	CLNS1A
RPS25	KANSL2	PRKAG1	PFDN5	C12orf47	RPL6	EBPL	CCNB1IP1	SNX6
QRSL1P3	PSEN1	MOAP1	TMEM87A	PLDN	RSL24D1	RPS3AP6	SEC11A	RSL1D1	RPS15A
LYRM1	HSBP1	MED11	RPAIN	MIR497HG	RPL26	RPS7P1	ZNHIT3	RPL19
RPL27	RPS7P11	MRPS23	CEP95	NUP85	C18orf21	RPL17	CTB	KXD1
ZNF91	MED29	ZNF350	ZNF530	SNHG11	LINC00266-1	C22orf39	SNRPD3
TFIP11
GOBPID	Pvalue	OddsRatio	ExpCount	Count	Size	Term
GO:0006414	4.68879241829736e-26	56.9770687936191	0.574008719119784
20	105	translational elongation
GO:0006614	2.60000832159518e-24	52.603488372093 0.574008719119784	19
105	SRP-dependent cotranslational protein targeting to membrane
GO:0006613	3.15525435272982e-24	51.9952107279694	0.579475468825687
19	106	cotranslational protein targeting to membrane
GO:0045047	3.15525435272982e-24	51.9952107279694	0.579475468825687
19	106	protein targeting to ER
GO:0072599	3.15525435272982e-24	51.9952107279694	0.579475468825687
19	106	establishment of protein localization to endoplasmic reticulum

GO:0070972	2.77648144031352e-23	45.6543771043771	0.645076465296519
19	118	protein localization to endoplasmic reticulum
GO:0072594	6.07173632209886e-23	30.0925390399075	1.11521694000415
22	204	establishment of protein localization to organelle
GO:0006415	4.66691310185766e-22	52.9786399302528	0.497474223237146
17	91	translational termination
GO:0000184	1.45095272374048e-21	41.6942054861224	0.650543215002422
18	119	nuclear-transcribed mRNA catabolic process, nonsense-mediated decay
GO:0019080	3.74181197590497e-21	34.1616161616162	0.825479205591309
19	151	viral genome expression
GO:0019083	3.74181197590497e-21	34.1616161616162	0.825479205591309
19	151	viral transcription
GO:0006413	4.25934168104889e-21	33.902380952381 0.830945955297211	19
152	translational initiation
GO:0006612	4.84392698848335e-21	33.6470149253731	0.836412705003114
19	153	protein targeting to membrane
GO:0019058	5.97187770952481e-19	22.7504217206201	1.26281918206353
20	231	viral infectious cycle
GO:0000956	9.91995462944465e-19	27.7904679187927	0.923880700297557
18	169	nuclear-transcribed mRNA catabolic process
GO:0006402	2.55635940821192e-18	26.2106557377049	0.973081447650682
18	178	mRNA catabolic process
GO:0043624	4.85934692713925e-18	28.7016129032258	0.836412705003114
17	153	cellular protein complex disassembly
GO:0022415	6.24684969268092e-18	19.9604519774011	1.4213549235347 20
260	viral reproductive process
GO:0043241	8.4773155886639e-18	27.6741020361473	0.863746453532627
17	158	protein complex disassembly
GO:0033365	2.7549704860241e-17	13.5311601538773	2.58577261089198
24	473	protein localization to organelle
GO:0006605	2.89098685052357e-17	13.5001212121212	2.59123936059788
24	474	protein targeting
GO:0006401	3.28504505635192e-17	22.3836240904708	1.12068368971005
18	205	RNA catabolic process
GO:0034623	4.45450046257981e-17	24.8258680912266	0.951214448827071
17	174	cellular macromolecular complex disassembly
GO:0032984	7.23068465679501e-17	24.0511748307447	0.978548197356584
17	179	macromolecular complex disassembly
GO:0016032	1.71301873968885e-15	12.5443094916779	2.46550411736212
22	451	viral reproduction
GO:0071845	1.04758933789708e-14	15.6482189079256	1.55255691647637
18	284	cellular component disassembly at cellular level
GO:0022411	1.33611111028828e-14	15.4120218579235	1.57442391529998
18	288	cellular component disassembly
GO:0022613	2.38077437408928e-10	13.156089193825 1.23001868382811	13
225	ribonucleoprotein complex biogenesis
GO:0071843	5.01844496662537e-10	12.3289085545723	1.30655317971075
13	239	cellular component biogenesis at cellular level
GO:0042274	3.48044442213014e-08	74.6309771309771	0.0984014947062487
5	18	ribosomal small subunit biogenesis
GO:0000387	6.52911111474096e-07	37.2817047817048	0.169469240882984
5	31	spliceosomal snRNP assembly
GO:0022618	8.36310802637277e-07	15.7809343434343	0.519341222060757
7	95	ribonucleoprotein complex assembly
GO:0071826	1.18534517842131e-06	14.9272700119474	0.546674970590271
7	100	ribonucleoprotein complex subunit organization
GO:0042254	1.48704832286028e-06	11.3722904804715	0.814545706179503
8	149	ribosome biogenesis
GO:0034660	3.29909815018209e-06	7.42924901185771	1.55802366618227
10	285	ncRNA metabolic process
Tissue: Adipose_Subcutaneous=>Skin_Sun_Exposed_Lower_leg	Type:	cluster
SourceGene:	HIST1H2BPS3(ENSG00000226908.1)
TargetGeneSet:	ARHGEF19-AS1	RPL11	ZCCHC17 RP11	PPIE	CCDC23	RPS8

UQCRH BTF3L4 ZYG11A HBXIP MRPS21 PAQR6 DCAF6 OCLM SNRPE NFASC SRP9
TOMM20 RPS7 UBC COA5 TXNDC9 RPL31 ERCC3 BBS5 NIF3L1 NBEAL1
EEF1B2 CCDC51 RYK CLDN18 EIF2S1 PARL NCBP2 TMEM128 C4orf52 RPL34 OSTC
RPS3A CDKN2AIP CTD TAF9 ZFYVE16 CAMLG PDF TMEM14C TMEM14B
EEF1A1 IL6 RPS3AP26 GIGYF1 MRPS33 PRAGMIN C8orf40 RPS20 PAF1 RPS6
CLTA C10orf31 RPS24 C10orf95 FAM204A FAM45A PRKRIR CLNS1A
RPS25 KANSL2 PRKAG1 PFDN5 C12orf47 RPL6 EBPL CCNB1IP1 SNX6
QRSL1P3 PSEN1 MOAP1 TMEM87A PLDN RSL24D1 RPS3AP6 SEC11A RSL1D1 RPS15A
LYRM1 HSBP1 MED11 RPAIN MIR497HG RPL26 RPS7P1 ZNHIT3 RPL19
RPL27 RPS7P11 MRPS23 CEP95 NUP85 C18orf21 RPL17 CTB KXD1
ZNF91 MED29 ZNF350 ZNF530 SNHG11 LINC00266-1 C22orf39 SNRPD3
TFIP11

GOBPID	Pvalue	OddsRatio	ExpCount	Count	Size	Term
GO:0006414	4.68879241829736e-26	56.9770687936191	0.574008719119784	20	105	translational elongation
GO:0006614	2.60000832159518e-24	52.603488372093	0.574008719119784	19	105	SRP-dependent cotranslational protein targeting to membrane
GO:0006613	3.15525435272982e-24	51.9952107279694	0.579475468825687	19	106	cotranslational protein targeting to membrane
GO:0045047	3.15525435272982e-24	51.9952107279694	0.579475468825687	19	106	protein targeting to ER
GO:0072599	3.15525435272982e-24	51.9952107279694	0.579475468825687	19	106	establishment of protein localization to endoplasmic reticulum
GO:0070972	2.77648144031352e-23	45.6543771043771	0.645076465296519	19	118	protein localization to endoplasmic reticulum
GO:0072594	6.07173632209886e-23	30.0925390399075	1.11521694000415	22	204	establishment of protein localization to organelle
GO:0006415	4.66691310185766e-22	52.9786399302528	0.497474223237146	17	91	translational termination
GO:0000184	1.45095272374048e-21	41.6942054861224	0.650543215002422	18	119	nuclear-transcribed mRNA catabolic process, nonsense-mediated decay
GO:0019080	3.74181197590497e-21	34.1616161616162	0.825479205591309	19	151	viral genome expression
GO:0019083	3.74181197590497e-21	34.1616161616162	0.825479205591309	19	151	viral transcription
GO:0006413	4.25934168104889e-21	33.902380952381	0.830945955297211	19	152	translational initiation
GO:0006612	4.84392698848335e-21	33.6470149253731	0.836412705003114	19	153	protein targeting to membrane
GO:0019058	5.97187770952481e-19	22.7504217206201	1.26281918206353	20	231	viral infectious cycle
GO:0000956	9.91995462944465e-19	27.7904679187927	0.923880700297557	18	169	nuclear-transcribed mRNA catabolic process
GO:0006402	2.55635940821192e-18	26.2106557377049	0.973081447650682	18	178	mRNA catabolic process
GO:0043624	4.85934692713925e-18	28.7016129032258	0.836412705003114	17	153	cellular protein complex disassembly
GO:0022415	6.24684969268092e-18	19.9604519774011	1.4213549235347	20	260	viral reproductive process
GO:0043241	8.4773155886639e-18	27.6741020361473	0.863746453532627	17	158	protein complex disassembly
GO:0033365	2.7549704860241e-17	13.5311601538773	2.58577261089198	24	473	protein localization to organelle
GO:0006605	2.89098685052357e-17	13.5001212121212	2.59123936059788	24	474	protein targeting
GO:0006401	3.28504505635192e-17	22.3836240904708	1.12068368971005	18	205	RNA catabolic process
GO:0034623	4.45450046257981e-17	24.8258680912266	0.951214448827071	17	174	cellular macromolecular complex disassembly
GO:0032984	7.23068465679501e-17	24.0511748307447	0.978548197356584	17	179	macromolecular complex disassembly
GO:0016032	1.71301873968885e-15	12.5443094916779	2.46550411736212	22	451	viral reproduction

GO:0071845 1.04758933789708e-14 15.6482189079256 1.55255691647637
18 284 cellular component disassembly at cellular level
GO:0022411 1.33611111028828e-14 15.4120218579235 1.57442391529998
18 288 cellular component disassembly
GO:0022613 2.38077437408928e-10 13.156089193825 1.23001868382811 13
225 ribonucleoprotein complex biogenesis
GO:0071843 5.01844496662537e-10 12.3289085545723 1.30655317971075
13 239 cellular component biogenesis at cellular level
GO:0042274 3.48044442213014e-08 74.6309771309771 0.0984014947062487
5 18 ribosomal small subunit biogenesis
GO:0000387 6.52911111474096e-07 37.2817047817048 0.169469240882984
5 31 spliceosomal snRNP assembly
GO:0022618 8.36310802637277e-07 15.7809343434343 0.519341222060757
7 95 ribonucleoprotein complex assembly
GO:0071826 1.18534517842131e-06 14.9272700119474 0.546674970590271
7 100 ribonucleoprotein complex subunit organization
GO:0042254 1.48704832286028e-06 11.3722904804715 0.814545706179503
8 149 ribosome biogenesis
GO:0034660 3.29909815018209e-06 7.42924901185771 1.55802366618227
10 285 ncRNA metabolic process
Tissue: Skin_Sun_Exposed_Lower_leg=>Adipose_Subcutaneous Type: asymmetric
SourceGene: ITIH2(ENSG00000151655.12)
TargetGeneSet: C1QA C1QC C1QB VAV3 CD53 PTPN22 FCGR2A HSPA6
PTPRC KLHL12 NLRP3 REL PLEK HK2 VAMP8 CYTIP PFKFB4 STAB1 CD86
TDGF1P6 KLHL7 YEATS2-AS1 FGF12-AS3 TLR2 MARCH1 SLC1A3 CTD
SERF1B CD14 CSF1R HAVCR2 DOCK2 F13A1 TRIM31 AIF1 FGD2 RNASET2
IKZF1 IRF5 TBXAS1 TAS2R4 RP11 SLA SYK FGD3 FKBP15 MRC1
ALOX5 PIK3AP1 GPAM KCNQ1 TPP1 LYVE1 MS4A6A MS4A7 CCDC88B FOLR2
SLCO2B1 MYO7A IL10RA GRAMD1B SLC37A2 PTPN6 CD163 C3AR1 GPR19 BIN2
ARHGAP9 CHST11 SELPLG DENR ALOX5AP GPR183 RNASE6 RIN3 PLCB2 PSTPIP1 MEFV
ITGAM NOD2 CYBA ARRB2 TMC8 FASN FAM59A GNA15 DENND1C VAV1
CD209 MYO1F LRRC25 QPCTL CD37 CD33 LILRB2 LAIR1 LILRA2 LILRA1
LILRB4 SIGLEC1 RP4 HCK TRPM2 NFAM1 TLR7 TLR8 CYBB WAS
ATP7A BTK SASH3 ARHGAP4
GOBPID Pvalue OddsRatio ExpCount Count Size Term
GO:0002252 4.64929932941652e-15 11.5845250639771 2.53754065462598
22 386 immune effector process
GO:0050778 2.06860823077317e-12 10.1528985507246 2.39291398519134
19 364 positive regulation of immune response
GO:0051249 3.6928613151707e-12 11.5447272026219 1.86042488409107
17 283 regulation of lymphocyte activation
GO:0046649 4.75697541221251e-12 9.00274414850686 2.84651581205453
20 433 lymphocyte activation
GO:0050865 7.75040933824717e-12 10.0922277722278 2.25486125527645
18 343 regulation of cell activation
GO:0002253 8.89163514691197e-12 10.8773413347881 1.96560791640717
17 299 activation of immune response
GO:0006954 1.5444731655871e-11 8.39457013574661 3.03716005812747
20 462 inflammatory response
GO:0001817 1.6552165497323e-11 9.60772365464448 2.36004428759255
18 359 regulation of cytokine production
GO:0002694 2.73910289637392e-11 10.0743927125506 2.11023458584181
17 321 regulation of leukocyte activation
GO:0050867 4.4185339385878e-11 11.7758333333333 1.57774548474154
15 240 positive regulation of cell activation
GO:0001816 7.99175910680171e-11 8.66795273691825 2.59670611030379
18 395 cytokine production
GO:0051251 1.20452972452744e-10 12.1718567655549 1.41339699674763
14 215 positive regulation of lymphocyte activation
GO:0042110 2.4962930731786e-10 9.39332414254084 2.0970867068023 16
319 T cell activation
GO:0002696 3.4920026195469e-10 11.1572241952759 1.53172790810325
14 233 positive regulation of leukocyte activation

GO:0002764	7.91708438270233e-10	10.4309380605677	1.63033700089959
14	248	immune response-regulating signaling pathway
GO:0002521	1.09870989918801e-09	9.19141986062718	1.98532973496644
15	302	leukocyte differentiation
GO:0050863	2.03171116026336e-09	10.7835365853659	1.45284063386617
13	221	regulation of T cell activation
GO:0002757	4.28282767285198e-09	10.0934959349593	1.54487578714276
13	235	immune response-activating signal transduction
GO:0051240	5.83317267710938e-09	6.92559419271748	2.99114248148917
17	455	positive regulation of multicellular organismal process
GO:0030098	8.87693882294628e-09	10.6656626506024	1.34108366203031
12	204	lymphocyte differentiation
GO:0002443	1.29617309746004e-08	10.2854029182055	1.3871012386686 12
211	leukocyte mediated immunity
GO:0050870	1.52546464459416e-08	11.61875	1.12414365787835	11
171	positive regulation of T cell activation
GO:0046651	1.62096576720511e-08	11.5457704821059	1.1307175973981 11
172	lymphocyte proliferation
GO:0032943	1.82811992378325e-08	11.4024978089395	1.14386547643762
11	174	mononuclear cell proliferation
GO:0050853	2.892807956625e-08	42.0117244748412	0.190644246072936
6	29	B cell receptor signaling pathway
GO:0070661	2.91327375525076e-08	10.8629211918686	1.19645699259567
11	182	leukocyte proliferation
GO:0050670	3.460543752458e-08	12.486391571554 0.946647290844924	10
144	regulation of lymphocyte proliferation
GO:0032944	3.69694969388881e-08	12.3930283224401	0.953221230364681
10	145	regulation of mononuclear cell proliferation
GO:0070663	5.1061780141714e-08	11.946218487395 0.986090927963463	10
150	regulation of leukocyte proliferation
GO:0030097	5.1249018514987e-08	6.3020417410019 3.04373399764722	16
463	hemopoiesis
GO:0050864	5.61233553369622e-08	17.9914974019839	0.53248910110027
8	81	regulation of B cell activation
GO:0050871	5.83874944861847e-08	24.2173597678917	0.354992734066847
7	54	positive regulation of B cell activation
GO:0042113	6.162525095668e-08	11.6931303990128	1.00581274652273
10	153	B cell activation
GO:0002250	6.92817607085643e-08	9.92226890756302	1.30164002491177
11	198	adaptive immune response
GO:0002697	8.07845295290836e-08	9.76353383458647	1.32136184347104
11	201	regulation of immune effector process
GO:0048534	1.28303207528184e-07	5.8674982162205 3.25410006227943	16
495	hemopoietic or lymphoid organ development
GO:0002449	1.40755423977504e-07	10.6399400524541	1.09784789979932
10	167	lymphocyte mediated immunity
GO:0050671	2.13483859705324e-07	14.9090909090909	0.631098193896616
8	96	positive regulation of lymphocyte proliferation
GO:0032946	2.31428507863939e-07	14.7405398424383	0.637672133416373
8	97	positive regulation of mononuclear cell proliferation
GO:0070665	2.93235676301156e-07	14.2568715642179	0.657393951975642
8	100	positive regulation of leukocyte proliferation
GO:0002460	2.98387086649269e-07	9.75920192638459	1.18988305307591
10	181	adaptive immune response based on somatic recombination of immune receptors built from immunoglobulin superfamily domains
GO:0006897	3.90705675177078e-07	6.696738172201	2.26800913431596	13
345	endocytosis
GO:0001819	4.03628815686415e-07	9.42439348620804	1.22932669019445
10	187	positive regulation of cytokine production
GO:0002768	4.2789146125138e-07	11.0240310077519	0.946647290844924
9	144	immune response-regulating cell surface receptor signaling pathway
GO:0032695	5.58308299541622e-07	90.1036106750392	0.0723133347173206
4	11	negative regulation of interleukin-12 production

GO:0050707	5.84989016993478e-07	16.7139037433155	0.493045463981731
7	75	regulation of cytokine secretion
GO:0051607	7.52046966491052e-07	12.48035030104	0.742855165732475	8
113	defense response to virus
GO:0030217	8.4110703672493e-07	10.1155671570954	1.025534565082	9
156	T cell differentiation
GO:0042098	1.04663228106465e-06	11.908881922675	0.775724863331257	8
118	T cell proliferation
GO:0050715	1.09788502461903e-06	20.9721543722521	0.341844855027334
6	52	positive regulation of cytokine secretion
GO:0045088	1.44739083782945e-06	8.12109038737446	1.41339699674763
10	215	regulation of innate immune response
GO:0050663	1.74569161787216e-06	14.0186588103255	0.578506677738565
7	88	cytokine secretion
GO:0031347	2.23262646552198e-06	5.67723577235772	2.64929762646184
13	403	regulation of defense response
GO:0042108	2.341651746052e-06	18.1933432266271	0.387862431665629
6	59	positive regulation of cytokine biosynthetic process
GO:0002429	2.58657289106293e-06	10.4687816091954	0.874333956127604
8	133	immune response-activating cell surface receptor signaling pathway
GO:0032621	2.72629797643103e-06	234.032608695652	0.0328696975987821
3	5	interleukin-18 production
GO:0032655	3.06646316422408e-06	26.5296296296296	0.230087883191475
5	35	regulation of interleukin-12 production
GO:0045621	3.14464618440672e-06	17.215088282504	0.407584250224898	6
62	positive regulation of lymphocyte differentiation
GO:0045619	3.86048517389463e-06	12.3330039525692	0.650820012455885
7	99	regulation of lymphocyte differentiation
GO:0032615	4.07494624917772e-06	24.8680555555556	0.243235762230987
5	37	interleukin-12 production
GO:0042129	4.41287739692783e-06	12.0689071566731	0.663967891495398
7	101	regulation of T cell proliferation
GO:0042102	4.55065181314951e-06	16.0629213483146	0.433880008303924
6	66	positive regulation of T cell proliferation
GO:0009615	5.01757228999279e-06	7.00868701911144	1.62376306137984
10	247	response to virus
GO:0007229	5.42439944753266e-06	15.5425878941646	0.447027887343436
6	68	integrin-mediated signaling pathway
GO:0009306	8.06844089082708e-06	8.88826335131754	1.01896062556224
8	155	protein secretion
GO:0071706	8.21678493874274e-06	14.377662250545	0.479897584942219	6
73	tumor necrosis factor superfamily cytokine production
GO:0019724	9.85028159520951e-06	10.5929269328802	0.749429105252232
7	114	B cell mediated immunity
GO:0050708	1.10439899371224e-05	10.3971017514595	0.762576984291744
7	116	regulation of protein secretion
GO:0050714	1.12052339363367e-05	13.5638550403545	0.506193343021244
6	77	positive regulation of protein secretion
GO:0032602	1.36149834757131e-05	18.9338624338624	0.308975157428552
5	47	chemokine production
GO:0006959	1.53663161154454e-05	9.85049407114625	0.802020621410283
7	122	humoral immune response
GO:0046631	1.6118246502793e-05	12.6670609107037	0.539063040620026
6	82	alpha-beta T cell activation
GO:0050851	1.62070283714701e-05	9.76489028213166	0.808594560930039
7	123	antigen receptor-mediated signaling pathway
Tissue: Skin_Sun_Exposed_Lower_leg=>Adipose_Subcutaneous	Type:	cluster
SourceGene:	ITIH2(ENSG00000151655.12)
TargetGeneSet:	C1QA	C1QC	C1QB	VAV3	CD53	PTPN22	FCGR2A	HSPA6
PTPRC	KLHL12	NLRP3	REL	PLEK	HK2	VAMP8	CYTIP	PFKFB4	STAB1	CD86
TDGF1P6	KLHL7	YEATS2-AS1	FGF12-AS3	TLR2	MARCH1	SLC1A3	CTD
SERF1B	CD14	CSF1R	HAVCR2	DOCK2	F13A1	TRIM31	AIF1	FGD2	RNASET2
IKZF1	IRF5	TBXAS1	TAS2R4	RP11	SLA	SYK	FGD3	FKBP15	MRC1

ALOX5 PIK3AP1 GPAM KCNQ1 TPP1 LYVE1 MS4A6A MS4A7 CCDC88B FOLR2
SLCO2B1 MYO7A IL10RA GRAMD1B SLC37A2 PTPN6 CD163 C3AR1 GPR19 BIN2
ARHGAP9 CHST11 SELPLG DENR ALOX5AP GPR183 RNASE6 RIN3 PLCB2 PSTPIP1 MEFV
ITGAM NOD2 CYBA ARRB2 TMC8 FASN FAM59A GNA15 DENND1C VAV1
CD209 MYO1F LRRC25 QPCTL CD37 CD33 LILRB2 LAIR1 LILRA2 LILRA1
LILRB4 SIGLEC1 RP4 HCK TRPM2 NFAM1 TLR7 TLR8 CYBB WAS
ATP7A BTK SASH3 ARHGAP4
GOBPID Pvalue OddsRatio ExpCount Count Size Term
GO:0002252 4.64929932941652e-15 11.5845250639771 2.53754065462598
22 386 immune effector process
GO:0050778 2.06860823077317e-12 10.1528985507246 2.39291398519134
19 364 positive regulation of immune response
GO:0051249 3.6928613151707e-12 11.5447272026219 1.86042488409107
17 283 regulation of lymphocyte activation
GO:0046649 4.75697541221251e-12 9.00274414850686 2.84651581205453
20 433 lymphocyte activation
GO:0050865 7.75040933824717e-12 10.0922277722278 2.25486125527645
18 343 regulation of cell activation
GO:0002253 8.89163514691197e-12 10.8773413347881 1.96560791640717
17 299 activation of immune response
GO:0006954 1.5444731655871e-11 8.39457013574661 3.03716005812747
20 462 inflammatory response
GO:0001817 1.6552165497323e-11 9.60772365464448 2.36004428759255
18 359 regulation of cytokine production
GO:0002694 2.73910289637392e-11 10.0743927125506 2.11023458584181
17 321 regulation of leukocyte activation
GO:0050867 4.4185339385878e-11 11.7758333333333 1.57774548474154
15 240 positive regulation of cell activation
GO:0001816 7.99175910680171e-11 8.66795273691825 2.59670611030379
18 395 cytokine production
GO:0051251 1.20452972452744e-10 12.1718567655549 1.41339699674763
14 215 positive regulation of lymphocyte activation
GO:0042110 2.4962930731786e-10 9.39332414254084 2.0970867068023 16
319 T cell activation
GO:0002696 3.4920026195469e-10 11.1572241952759 1.53172790810325
14 233 positive regulation of leukocyte activation
GO:0002764 7.91708438270233e-10 10.4309380605677 1.63033700089959
14 248 immune response-regulating signaling pathway
GO:0002521 1.09870989918801e-09 9.19141986062718 1.98532973496644
15 302 leukocyte differentiation
GO:0050863 2.03171116026336e-09 10.7835365853659 1.45284063386617
13 221 regulation of T cell activation
GO:0002757 4.28282767285198e-09 10.0934959349593 1.54487578714276
13 235 immune response-activating signal transduction
GO:0051240 5.83317267710938e-09 6.92559419271748 2.99114248148917
17 455 positive regulation of multicellular organismal process
GO:0030098 8.87693882294628e-09 10.6656626506024 1.34108366203031
12 204 lymphocyte differentiation
GO:0002443 1.29617309746004e-08 10.2854029182055 1.3871012386686 12
211 leukocyte mediated immunity
GO:0050870 1.52546464459416e-08 11.61875 1.12414365787835 11
171 positive regulation of T cell activation
GO:0046651 1.62096576720511e-08 11.5457704821059 1.1307175973981 11
172 lymphocyte proliferation
GO:0032943 1.82811992378325e-08 11.4024978089395 1.14386547643762
11 174 mononuclear cell proliferation
GO:0050853 2.892807956625e-08 42.0117244748412 0.190644246072936
6 29 B cell receptor signaling pathway
GO:0070661 2.91327375525076e-08 10.8629211918686 1.19645699259567
11 182 leukocyte proliferation
GO:0050670 3.460543752458e-08 12.486391571554 0.946647290844924 10
144 regulation of lymphocyte proliferation
GO:0032944 3.69694969388881e-08 12.3930283224401 0.953221230364681

10 145 regulation of mononuclear cell proliferation
GO:0070663 5.1061780141714e-08 11.946218487395 0.986090927963463 10
150 regulation of leukocyte proliferation
GO:0030097 5.1249018514987e-08 6.3020417410019 3.04373399764722 16
463 hemopoiesis
GO:0050864 5.61233553369622e-08 17.9914974019839 0.53248910110027
8 81 regulation of B cell activation
GO:0050871 5.83874944861847e-08 24.2173597678917 0.354992734066847
7 54 positive regulation of B cell activation
GO:0042113 6.162525095668e-08 11.6931303990128 1.00581274652273
10 153 B cell activation
GO:0002250 6.92817607085643e-08 9.92226890756302 1.30164002491177
11 198 adaptive immune response
GO:0002697 8.07845295290836e-08 9.76353383458647 1.32136184347104
11 201 regulation of immune effector process
GO:0048534 1.28303207528184e-07 5.8674982162205 3.25410006227943 16
495 hemopoietic or lymphoid organ development
GO:0002449 1.40755423977504e-07 10.6399400524541 1.09784789979932
10 167 lymphocyte mediated immunity
GO:0050671 2.13483859705324e-07 14.9090909090909 0.631098193896616
8 96 positive regulation of lymphocyte proliferation
GO:0032946 2.31428507863939e-07 14.7405398424383 0.637672133416373
8 97 positive regulation of mononuclear cell proliferation
GO:0070665 2.93235676301156e-07 14.2568715642179 0.657393951975642
8 100 positive regulation of leukocyte proliferation
GO:0002460 2.98387086649269e-07 9.75920192638459 1.18988305307591
10 181 adaptive immune response based on somatic recombination of immune receptors built from immunoglobulin superfamily domains
GO:0006897 3.90705675177078e-07 6.696738172201 2.26800913431596 13
345 endocytosis
GO:0001819 4.03628815686415e-07 9.42439348620804 1.22932669019445
10 187 positive regulation of cytokine production
GO:0002768 4.2789146125138e-07 11.0240310077519 0.946647290844924
9 144 immune response-regulating cell surface receptor signaling pathway
GO:0032695 5.58308299541622e-07 90.1036106750392 0.0723133347173206
4 11 negative regulation of interleukin-12 production
GO:0050707 5.84989016993478e-07 16.7139037433155 0.493045463981731
7 75 regulation of cytokine secretion
GO:0051607 7.52046966491052e-07 12.48035030104 0.742855165732475 8
113 defense response to virus
GO:0030217 8.4110703672493e-07 10.1155671570954 1.025534565082 9
156 T cell differentiation
GO:0042098 1.04663228106465e-06 11.908881922675 0.775724863331257 8
118 T cell proliferation
GO:0050715 1.09788502461903e-06 20.9721543722521 0.341844855027334
6 52 positive regulation of cytokine secretion
GO:0045088 1.44739083782945e-06 8.12109038737446 1.41339699674763
10 215 regulation of innate immune response
GO:0050663 1.74569161787216e-06 14.0186588103255 0.578506677738565
7 88 cytokine secretion
GO:0031347 2.23262646552198e-06 5.67723577235772 2.64929762646184
13 403 regulation of defense response
GO:0042108 2.341651746052e-06 18.1933432266271 0.387862431665629
6 59 positive regulation of cytokine biosynthetic process
GO:0002429 2.58657289106293e-06 10.4687816091954 0.874333956127604
8 133 immune response-activating cell surface receptor signaling pathway
GO:0032621 2.72629797643103e-06 234.032608695652 0.0328696975987821
3 5 interleukin-18 production
GO:0032655 3.06646316422408e-06 26.5296296296296 0.230087883191475
5 35 regulation of interleukin-12 production
GO:0045621 3.14464618440672e-06 17.215088282504 0.407584250224898 6
62 positive regulation of lymphocyte differentiation
GO:0045619 3.86048517389463e-06 12.3330039525692 0.650820012455885

7	99	regulation of lymphocyte differentiation
GO:0032615	4.07494624917772e-06	24.8680555555556	0.243235762230987
5	37	interleukin-12 production
GO:0042129	4.41287739692783e-06	12.0689071566731	0.663967891495398
7	101	regulation of T cell proliferation
GO:0042102	4.55065181314951e-06	16.0629213483146	0.433880008303924
6	66	positive regulation of T cell proliferation
GO:0009615	5.01757228999279e-06	7.00868701911144	1.62376306137984
10	247	response to virus
GO:0007229	5.42439944753266e-06	15.5425878941646	0.447027887343436
6	68	integrin-mediated signaling pathway
GO:0009306	8.06844089082708e-06	8.88826335131754	1.01896062556224
8	155	protein secretion
GO:0071706	8.21678493874274e-06	14.377662250545 0.479897584942219	6
73	tumor necrosis factor superfamily cytokine production
GO:0019724	9.85028159520951e-06	10.5929269328802	0.749429105252232
7	114	B cell mediated immunity
GO:0050708	1.10439899371224e-05	10.3971017514595	0.762576984291744
7	116	regulation of protein secretion
GO:0050714	1.12052339363367e-05	13.5638550403545	0.506193343021244
6	77	positive regulation of protein secretion
GO:0032602	1.36149834757131e-05	18.9338624338624	0.308975157428552
5	47	chemokine production
GO:0006959	1.53663161154454e-05	9.85049407114625	0.802020621410283
7	122	humoral immune response
GO:0046631	1.6118246502793e-05	12.6670609107037	0.539063040620026
6	82	alpha-beta T cell activation
GO:0050851	1.62070283714701e-05	9.76489028213166	0.808594560930039
7	123	antigen receptor-mediated signaling pathway
Tissue: Skin_Sun_Exposed_Lower_leg=>Adipose_Subcutaneous	Type:	asymmetric
SourceGene:	ITIH5(ENSG00000123243.10)
TargetGeneSet:	RPL5	RP11	ACBD6	FAM129A SNRPE	RPS7P3	RP4	LINC00184
UBC	ASPRV1	TPRKB	DGUOK	RPIA	CCDC115 EEF1B2	IMPDH2	GRM2	RPL24
ZBTB38	MTHFD2L RPL34	EPB41L4A-AS1	CTB	NPM1	ERGIC1	TDP2	C6orf130
DST	MRPS12	AASS	CCDC136 SSBP1	ACTR3B	ESYT2	PHYHIP	RPS20	SNHG6
RPL30	COL14A1 RPL8	RPS6	RPL12	SSNA1	SLC25A16	ZDHHC6	SFXN4
EIF3M	LAMTOR1 TAF1D	RPS25	JAM3	PA2G4	PMCH	RPS2P5	RPLP0	TPT1	TMX1
GATM	RPS3AP6 PARP16	RPS2	SETD6	NQO1	EIF4A1	RPL27	DUSP3	STRA13	FBL
RPS19	RPL18	CTD	SNRPB	MYL9	RPL3	AMELX	RBM3	RPS4X	FAM122B
GOBPID	Pvalue	OddsRatio	ExpCount	Count	Size	Term
GO:0006414	1.37290287199807e-24	65.751724137931 0.457753788665144	18
105	translational elongation
GO:0006415	6.39401949771585e-24	71.4858989424207	0.396719950176458
17	91	translational termination
GO:0006413	3.40217872490138e-23	46.2824675324675	0.662653103591447
19	152	translational initiation
GO:0006614	8.74213990076292e-23	60.054347826087 0.457753788665144	17
105	SRP-dependent cotranslational protein targeting to membrane
GO:0006613	1.0380492635078e-22	59.3754274548119	0.462113348557193
17	106	cotranslational protein targeting to membrane
GO:0045047	1.0380492635078e-22	59.3754274548119	0.462113348557193
17	106	protein targeting to ER
GO:0072599	1.0380492635078e-22	59.3754274548119	0.462113348557193
17	106	establishment of protein localization to endoplasmic reticulum
GO:0070972	7.16419495487119e-22	52.2770124838571	0.514428067261781
17	118	protein localization to endoplasmic reticulum
GO:0000184	8.33292697399427e-22	51.7608695652174	0.51878762715383
17	119	nuclear-transcribed mRNA catabolic process, nonsense-mediated decay
GO:0000956	1.09434307592245e-20	37.7139072847682	0.73676562175628
18	169	nuclear-transcribed mRNA catabolic process
GO:0006402	2.84717607678322e-20	35.57	0.776001660784721	18	178
mRNA catabolic process
GO:0019080	5.64566457535249e-20	39.3118105126541	0.658293543699398

17	151	viral genome expression
GO:0019083	5.64566457535249e-20	39.3118105126541	0.658293543699398
17	151	viral transcription
GO:0006612	7.10786592667512e-20	38.7282608695652	0.667012663483496
17	153	protein targeting to membrane
GO:0043624	7.10786592667512e-20	38.7282608695652	0.667012663483496
17	153	cellular protein complex disassembly
GO:0043241	1.24656410413481e-19	37.3418131359852	0.68881046294374l
17	158	protein complex disassembly
GO:0006401	3.76521331109698e-19	30.3764705882353	0.893709777870044
18	205	RNA catabolic process
GO:0034623	6.66196464841807e-19	33.4986153420105	0.758563421216525
17	174	cellular macromolecular complex disassembly
GO:0032984	1.08712553227867e-18	32.4533011272142	0.78036122067677
17	179	macromolecular complex disassembly
GO:0072594	1.02653306617973e-17	28.0652173913043	0.889350217977995
17	204	establishment of protein localization to organelle
GO:0019058	8.49142697558317e-17	24.4776513612353	1.00705833506332
17	231	viral infectious cycle
GO:0022415	6.21639667524559e-16	21.5123456790123	1.13348557193274
17	260	viral reproductive process
GO:0071845	2.71358709098729e-15	19.5454323400098	1.23811500934191
17	284	cellular component disassembly at cellular level
GO:0022411	3.42367614275736e-15	19.2514840365795	1.25555324891011
17	288	cellular component disassembly
GO:0016032	5.06635616100943e-12	11.8822881186135	1.9661615113141 17
451	viral reproduction
GO:0033365	1.07906029551034e-11	11.2911899313501	2.06207182893917
17	473	protein localization to organelle
GO:0006605	1.11574457936033e-11	11.2656740557511	2.06643138883122
17	474	protein targeting
GO:0022613	3.08854168778118e-09	14.0109633357297	0.980900975711023
11	225	ribonucleoprotein complex biogenesis
GO:0071843	5.81107353804976e-09	13.1376518218623	1.04193481419971
11	239	cellular component biogenesis at cellular level
GO:0042254	2.53669368384116e-07	14.6970986460348	0.6495744239153 8
149	ribosome biogenesis
GO:0022618	3.45736164571389e-06	16.9118864577173	0.414158189744654
6	95	ribonucleoprotein complex assembly
GO:0071826	4.66249303664084e-06	16.006718924972 0.435955989204899	6
100	ribonucleoprotein complex subunit organization
Tissue: Skin_Sun_Exposed_Lower_leg=>Adipose_Subcutaneous	Type:	cluster
SourceGene:	MAP4(ENSG00000047849.17)
TargetGeneSet:	RP4	SYF2	KHDRBS1 KPNA7	RP11	GAPDHP33	C1orf43
LPIN1	UBC	EIF5B	SNORD89 GLI2	UGGT1	MZT2B	ZRANB3	MMADHC	SLC40A1
SETD5	CRELD1	RPL15	GORASP1 MAP4	ATXN7	NDUFB4	PLXND1	COPB2	EIF2S1
EIF4G1	THPO	NOA1	FAM175A HSD17B11	EPB41L4A-AS1	AFF4	CAMLG
PCDHGC4 RPS14	MAT2B	TRIM41	RPL15P3 DEK	VARS	HLA-C	CSNK2B	AGPAT1
RPS18	YIPF3	C6orf72 ZNF277	ING3	MLL3	GOLGA7	HOOK3	EIF3E	EIF3H
TATDN1	RPL8	SMARCA2 TLN1	CLTA	MCART1	XPA	SPTAN1	EEF1A1P5
RPL7A	EIF2S2P3	PYROXD2 MRPL43	GBF1	SFR1	RPL27A	CKAP5	CD6
DNAJC4	C11orf2 PHB2	ATF7IP	ZCRB1	ARID2	PFDN5	NACA	RPL18AP3	RPL6
TRAC	GOLGA3	POLR1D	TPT1	ITM2B	HNRNPA3P5	RPS19P3 TIMM9	NEK9
KIAA1737	FAN1	UBR1	CCNDBP1 COPS2	C15orf39	SEC11A	MPG	CTD
NTAN1	GTF3C1	FTO	C16orf48	VPS53	RPL26	OMG	RPL19	CNTNAP1 CLTC
SUMO2	RPL17	TPGS1	NFIC	ALKBH7	PGLS	GPI	FAM98C	B3GNT8	GLTSCR2
RPL13A	ZNF524	CCDC106 RPS5	RRBP1	SCAND1	UBE2G2	DSTNP1	THAP7	TTC28
EIF3L	LINC00106	CHST7	IGBP1	SLC16A2 GPR174	AIFM1	RPL10	MT
GOBPID	Pvalue	OddsRatio	ExpCount	Count	Size	Term
GO:0006413	1.50348157356512e-19	23.9208754208754	1.15701335547713
20	152	translational initiation
GO:0000184	5.80761101464227e-16	23.5290229291469	0.905819666459069
16	119	nuclear-transcribed mRNA catabolic process, nonsense-mediated decay

GO:0006414	1.97119866493078e-15	25.0017543859649	0.799252646875649
15	105	translational elongation
GO:0006614	1.97119866493078e-15	25.0017543859649	0.799252646875649
15	105	SRP-dependent cotranslational protein targeting to membrane
GO:0006613	2.28190457074742e-15	24.7252747252747	0.806864576845893
15	106	cotranslational protein targeting to membrane
GO:0045047	2.28190457074742e-15	24.7252747252747	0.806864576845893
15	106	protein targeting to ER
GO:0072599	2.28190457074742e-15	24.7252747252747	0.806864576845893
15	106	establishment of protein localization to endoplasmic reticulum
GO:0006415	6.15016037404275e-15	27.0151515151515	0.692685627292229
14	91	translational termination
GO:0070972	1.18170755331578e-14	21.8262646908533	0.898207736488824
15	118	protein localization to endoplasmic reticulum
GO:0006612	3.34669986240972e-14	17.6474607858363	1.16462528544737
16	153	protein targeting to membrane
GO:0000956	1.61154252642039e-13	15.7841746627729	1.28641616497128
16	169	nuclear-transcribed mRNA catabolic process
GO:0034623	2.54588053133933e-13	15.2792889846485	1.3244758148225 16
174	cellular macromolecular complex disassembly
GO:0006402	3.6322098745073e-13	14.8978198056212	1.35492353470348
16	178	mRNA catabolic process
GO:0032984	3.9641293195755e-13	14.8053778880042	1.36253546467373
16	179	macromolecular complex disassembly
GO:0043624	5.82808726800872e-13	16.2505720823799	1.16462528544737
15	153	cellular protein complex disassembly
GO:0043241	9.36613399522253e-13	15.6768494663231	1.2026849352986 15
158	protein complex disassembly
GO:0072594	2.99965710984588e-12	12.8139429606157	1.55283371392983
16	204	establishment of protein localization to organelle
GO:0006401	3.23349530185963e-12	12.7452437239671	1.56044564390008
16	205	RNA catabolic process
GO:0019080	7.81198589296222e-12	15.1198296836983	1.14940142550689
14	151	viral genome expression
GO:0019083	7.81198589296222e-12	15.1198296836983	1.14940142550689
14	151	viral transcription
GO:0071845	4.51469032940714e-11	9.6354556803995 2.16178811154937	17
284	cellular component disassembly at cellular level
GO:0022411	5.62549538531179e-11	9.49053684085228	2.19223583143035
17	288	cellular component disassembly
GO:0019058	2.2105555614356e-10	10.3252923976608	1.75835582312643
15	231	viral infectious cycle
GO:0022415	1.14607266483423e-09	9.084425349087	1.97910179226351	15
260	viral reproductive process
GO:0033365	1.66579391655435e-08	5.97104634495939	3.60044287592554
18	473	protein localization to organelle
GO:0006605	1.02496465035478e-07	5.55346933013341	3.60805480589579
17	474	protein targeting
GO:0016032	2.93149084197562e-07	5.44133039863047	3.43298041658017
16	451	viral reproduction
Tissue: Skin_Sun_Exposed_Lower_leg=>Adipose_Subcutaneous	Type:	asymmetric
SourceGene:	RAB11FIP5(ENSG00000135631.11)
TargetGeneSet:	NBPF1	MAN1C1	KHDRBS1	EIF2C3	SLFNL1	PPCS	TMEM53	RP4	RP11
AGL	HIPK1	MRPS21	PPP1R12B	SLC8A1	ST3GAL5	PDE11A	EEF1B2	ERBB4
ITM2C	ITPR1	LAMB2	ADAMTS9-AS1	LRIG1	RPL24	RPL22L1	NAT8L	AFAP1
RPS3A	FRG1	CTB	RPL15P3	C6orf130	YIPF3	RP1	DST	RARS2	BVES
HIVEP2	C6orf72	EEF1A1P6	NT5C3	RPS3AP26	CCDC136	SSBP1	RPL7
TATDN1	SLC45A4	RPS6	TLN1	CLTA	RPL12	FNBP1	EEF1A1P5	RPL7A
NACC2	SVIL	PRKG1	RPS24	RPS3AP5	EXOSC1	SFR1	ZDHHC6	RP13	ZBED5
C11orf9	GUCY1A2	JAM3	ADCY6	NACA	SLC35E3	RPL18AP3	RPL6	TPT1
DHRS4L2	EAPP	LGALS3	CCNDBP1	LITAF	PAPD5	RPL13	KIF1C	RPL26	RPL17P43
ANKRD40	PRKCA	NT5C	CTD	CTIF	RPL17	RPS15	PIP5K1C	HNRNPA1P10
ZNF823	LPHN1	RPL18A	UBA52	RPL13A	ZNF551	RPS5	SLC24A3	RPL41P1	RPL12P4

EIF3L RPL3 UXT RPS4X RPL39 RPL10
GOBPID Pvalue OddsRatio ExpCount Count Size Term
GO:0006414 3.34308530451034e-27 56.6785714285714 0.610338384886859
21 105 translational elongation
GO:0006415 8.11219692278849e-27 62.9225352112676 0.528959933568611
20 91 translational termination
GO:0006402 1.03365059022287e-26 36.9168831168831 1.03466888104629
24 178 mRNA catabolic process
GO:0000184 5.71302332623627e-26 48.5340136054422 0.691716836205107
21 119 nuclear-transcribed mRNA catabolic process, nonsense-mediated decay
GO:0000956 1.08166172969118e-25 36.7261396811139 0.982354162341706
23 169 nuclear-transcribed mRNA catabolic process
GO:0006614 1.84568990510762e-25 52.5073529411765 0.610338384886859
20 105 SRP-dependent cotranslational protein targeting to membrane
GO:0006613 2.26526531193945e-25 51.8931686046512 0.616151131409591
20 106 cotranslational protein targeting to membrane
GO:0045047 2.26526531193945e-25 51.8931686046512 0.616151131409591
20 106 protein targeting to ER
GO:0072599 2.26526531193945e-25 51.8931686046512 0.616151131409591
20 106 establishment of protein localization to endoplasmic reticulum
GO:0006413 3.42392842556969e-25 38.8602977667494 0.883537471455263
22 152 translational initiation
GO:0006401 3.43348159778401e-25 31.3502762430939 1.19161303716006
24 205 RNA catabolic process
GO:0070972 2.26075563861961e-24 45.500637755102 0.685904089682375 20
118 protein localization to endoplasmic reticulum
GO:0019080 4.02539928104472e-22 33.9599236641221 0.877724724932531
20 151 viral genome expression
GO:0019083 4.02539928104472e-22 33.9599236641221 0.877724724932531
20 151 viral transcription
GO:0006612 5.28706381099446e-22 33.4445488721804 0.889350217977995
20 153 protein targeting to membrane
GO:0043624 5.28706381099446e-22 33.4445488721804 0.889350217977995
20 153 cellular protein complex disassembly
GO:0043241 1.02780258194451e-21 32.2214673913044 0.918413950591655
20 158 protein complex disassembly
GO:0034623 7.45176687104006e-21 28.8413149350649 1.01141789495537
20 174 cellular macromolecular complex disassembly
GO:0032984 1.32853705363753e-20 27.9245283018868 1.04048162756903
20 179 macromolecular complex disassembly
GO:0072594 1.87577859497437e-19 24.0879755434783 1.18580029063733
20 204 establishment of protein localization to organelle
GO:0019058 2.25399144564704e-18 20.9656398104265 1.34274444675109
20 231 viral infectious cycle
GO:0022415 2.33499315247846e-17 18.39453125 1.51131409591032 20
260 viral reproductive process
GO:0071845 1.31406163340573e-16 16.6938920454545 1.65082001245589
20 284 cellular component disassembly at cellular level
GO:0022411 1.72529628947621e-16 16.4400652985075 1.67407099854681
20 288 cellular component disassembly
GO:0033365 2.03779779665013e-13 10.2617994100295 2.74942910525223
21 473 protein localization to organelle
GO:0006605 2.12314580931642e-13 10.2384105960265 2.75524185177496
21 474 protein targeting
GO:0016032 8.60047376795442e-13 10.1044083526682 2.62154868175213
20 451 viral reproduction
GO:0071843 1.19950273911573e-06 8.34297179275345 1.38924641893295
10 239 cellular component biogenesis at cellular level
GO:0006364 2.34541989196471e-06 13.3739456419869 0.604525638364127
7 104 rRNA processing
GO:0042254 2.37935104935418e-06 10.6203807390817 0.866099231887067
8 149 ribosome biogenesis
GO:0016072 3.41632890821383e-06 12.5895851721094 0.639402117500519

7	110	rRNA metabolic process
GO:0022613	6.12658158286129e-06	7.86166666666667	1.3078679676147 9
225	ribonucleoprotein complex biogenesis
Tissue: Skin_Sun_Exposed_Lower_leg=>Adipose_Subcutaneous	Type:	cluster
SourceGene:	RP11-275F13.1(ENSG00000261664.1)
TargetGeneSet:	MRPL20	LRRC47	CNKSR1	SNHG12	SNRNP40	SFPQ	GNL2	EBNA1BP2
DPH2	MAGOH	TMEM48	ZZZ3	WDR3	SF3B4	GPATCH4	PRCC	METTL13	DHX9
RBBP5	RRP15	COG2	HEATR1	TRMT61B	WDR43	TTC27	GEMIN6	PNPT1	MPHOSPH10
POLR1A	PTCD3	ANAPC1	POLR1B	DDX18	MKI67IP	SPPL2B	FASTKD2	RNF25	ACSL3	CLN5
TATDN2	HHATL-AS1	SACM1L	VPRBP	RRP9	PDE12	SHQ1	ZBTB11	QTRTD1
STAG1	ATR	EIF2B5	ECE2	PSMD2	LSG1	NCBP2-AS1	CTBP1-AS1
NOP14	GRPEL1	METTL19	BST1	HRH2	PDS5A	UTP3	SDAD1	NAA15	PLRG1
NSUN2	MTRR	SLC30A5	CTD	HSPA4	HSPA9	HARS	TCOF1	G3BP1	RIOK1
PAK1IP1	BRD2	WDR46	FTSJD2	RPL7L1	POLR1C	XPO5	MTO1	SYNCRIP	AMD1
HDDC2	HBS1L	TCP1	EIF3B	DDX56	CCT6A	TBL2	EIF4H	SRRT	NAPEPLD	PUS7
ZC3HC1	ABCF2	UBE3C	KIAA1967	ELP3	MAK16	TGS1	NSMAF	C8orf38	POP1
BOP1	MTAP	NOL6	IARS	C9orf102	TEX10	PRPF4	ZNF79	TRUB2
FUBP3	EXOSC2	SURF6	SURF2	C9orf69	SDCCAG3	FAM208B	SEPHS1	PDSS1	BMS1
ZNF239	CSTF2T	SEC24C	NDST2	RRP12	WNT8B	C10orf2	NOLC1	PDCD11	EIF6
SEC23IP	C10orf137	PHRF1	CARS	NUP98	CTR9	IMMP1L	API5	BTBD18
PRPF19	WDR74	SF1	NARS2	DDX10	ZNF202	NOP2	PPHLN1	DDX23	BCDIN3D	RP3
PA2G4	RP11	CYP27B1	CAND1	CCT2	UTP20	POLR3B	SART3	ANAPC7	DDX55
DHX37	PUS1	DDX51	NOC4L	POLE	SACS	COG3	ZC3H13	IPO5	CARKD
SUPT16H	PRMT5	SGPP1	DCAF4	TRIP11	NIPA2	UBE3A	SPATA5L1	CORO2B
IDH3A	AEN	TBL3	RNPS1	USP7	GSPT1	CD2BP2	RNF40	DNAJA2	CTCF
DDX19A	ADAT1	ZC3H18	CTU2	ZNF778	METTL16	MYBBP1A	PELP1	DHX33	NEURL4	PFAS
UTP6	AATF	MED1	PSME3	KPNB1	MRPL27	SRSF1	FTSJ3	NOL11	TMEM104
CCDC137	AFG3L2	TIMM21	SH2D3A	DNMT1	ILF3	TNPO2	GATAD2A	FBL	PSMC4
ZNF576	CD3EAP	DHX34	GRWD1	PPP6R1	U2AF2	TRIM28	PSMF1	TRMT6	SNX5
ACTR5	ZNF335	NCOA5	TP53RK	DDX27	WDR4	RRP1	PWP2	PES1	ANKRD54
POLDIP3	PQBP1	SMC1A	FAM123B	UTP14A	DKC1
GOBPID	Pvalue	OddsRatio	ExpCount	Count	Size	Term
GO:0034660	6.64716275994768e-35	16.1339413223762	4.20074735312435
46	285	ncRNA metabolic process
GO:0071843	3.44265036636425e-32	16.9027484143763	3.52273199086568
41	239	cellular component biogenesis at cellular level
GO:0022613	4.84449512488626e-32	17.563505702234 3.3163794893087 40	225
ribonucleoprotein complex biogenesis
GO:0034470	2.60824130366708e-30	18.1530605131065	2.94789287938551
37	200	ncRNA processing
GO:0042254	1.89943549524489e-28	21.3378665533362	2.1961801951422 32
149	ribosome biogenesis
GO:0016072	4.76998324008044e-23	22.1417396745932	1.62134108366203
25	110	rRNA metabolic process
GO:0006364	2.46624558935442e-22	22.4730158730159	1.53290429728046
24	104	rRNA processing
GO:0006399	3.79284321596101e-12	11.3477891156463	1.84243304961594
17	125	tRNA metabolic process
GO:0000375	3.38084290262527e-11	7.70293609671848	3.08054805895786
20	209	RNA splicing, via transesterification reactions
GO:0008033	8.60588814778553e-11	14.39546875	1.13493875856342	13
77	tRNA processing
GO:0000377	1.71320003984959e-10	7.43959320144887	3.00685073697322
19	204	RNA splicing, via transesterification reactions with bulged adenosine as nucleophile
GO:0000398	1.71320003984959e-10	7.43959320144887	3.00685073697322
19	204	nuclear mRNA splicing, via spliceosome
GO:0006397	2.0163848756993e-10	5.16824131553669	5.88104629437409
26	399	mRNA processing
GO:0009451	2.44197917782365e-10	18.4059405940594	0.78119161303716
11	53	RNA modification
GO:0008380	2.42218096615917e-09	5.33325216120784	4.7608470002076 22
323	RNA splicing

GO:0022618 1.45642237428395e-07 9.17574257425743 1.40024911770812
11 95 ribonucleoprotein complex assembly
GO:0043414 1.67088938757195e-07 7.95944804280484 1.73925679883745
12 118 macromolecule methylation
GO:0071826 2.47709169935886e-07 8.65719212370675 1.47394643969275
11 100 ribonucleoprotein complex subunit organization
GO:0032259 5.25396601047119e-07 7.08340649692713 1.93086983599751
12 131 methylation
GO:0001510 7.75989745425052e-07 42.7584134615385 0.191613037160058
5 13 RNA methylation
GO:0006400 4.86431283957643e-06 26.303624260355 0.265310359144696 5
18 tRNA modification
GO:0000966 1.24937860070488e-05 203.385714285714 0.0589578575877102
3 4 RNA 5'-end processing
Tissue: Skin_Sun_Exposed_Lower_leg=>Adipose_Subcutaneous Type: asymmetric
SourceGene: RP11-485G7.4(ENSG00000263179.1)
TargetGeneSet: SLC2A5 NMNAT1 ARID1A MEAF6 RPS8 RPL5 RP11 KCNA2 RPS7
ASXL2 PPP1CB RGPD5 RPL15 NICN1 RPL29 ATXN7 PCNP RPL24 RYK
EIF2B5-IT1 RELL1 LRBA ACSL1 AFF4 CTB RPL15P3 NKAPL WDR11
ABRACL ARID1B SLC29A4 EEF1A1P6 RPS3AP26 TRBV6-5 CTD RP1 ASPH
EIF3E EIF3H VPS28 RPS6 NIPSNAP3A RPL7A RPS24 FAS RBP4 SCD
CARS-AS1 C11orf2 RSF1 GUCY1A2 MCRS1 NACA NAP1L1 RPL18AP3 RPL6
RPSAP54 STARD13 DGKH SNX6 PYGL CCNDBP1 RAB11A PIAS1 FANCA MIR762
RPL26 SUPT6H RPL19 NMT1 MED13 BPTF AANAT FASN MYL12B C18orf32
C19orf80 HNRNPA1P10 UBA52 RPS5 ID1 PHF20 CHD6 RPL12P4 CTA
EIF3L LMF2 KDM5C-IT1 BCORL1 RPL10
GOBPID Pvalue OddsRatio ExpCount Count Size Term
GO:0006413 9.66275195319599e-21 37.5276558384548 0.725762922981109
18 152 translational initiation
GO:0006415 1.25185366783809e-19 52.2880116959064 0.434502802574216
15 91 translational termination
GO:0000184 2.09983507836892e-19 41.8508884411064 0.568195972597052
16 119 nuclear-transcribed mRNA catabolic process, nonsense-mediated decay
GO:0019080 3.15294642759193e-19 34.7611940298507 0.720988166908864
17 151 viral genome expression
GO:0019083 3.15294642759193e-19 34.7611940298507 0.720988166908864
17 151 viral transcription
GO:0006414 1.2094858532895e-18 44.1111111111111 0.501349387585634
15 105 translational elongation
GO:0006614 1.2094858532895e-18 44.1111111111111 0.501349387585634
15 105 SRP-dependent cotranslational protein targeting to membrane
GO:0006613 1.40388819777656e-18 43.6233211233211 0.506124143657878
15 106 cotranslational protein targeting to membrane
GO:0045047 1.40388819777656e-18 43.6233211233211 0.506124143657878
15 106 protein targeting to ER
GO:0072599 1.40388819777656e-18 43.6233211233211 0.506124143657878
15 106 establishment of protein localization to endoplasmic reticulum
GO:0070972 7.50797766173008e-18 38.5086299892125 0.563421216524808
15 118 protein localization to endoplasmic reticulum
GO:0019058 1.99765200688039e-17 23.4780447390224 1.1029686526884 18
231 viral infectious cycle
GO:0000956 6.66778953631612e-17 28.0754716981132 0.806933776209259
16 169 nuclear-transcribed mRNA catabolic process
GO:0034623 1.06767194586507e-16 27.1774540243611 0.83080755657048
16 174 cellular macromolecular complex disassembly
GO:0006402 1.53978141557745e-16 26.4989517819706 0.849906580859456
16 178 mRNA catabolic process
GO:0022415 1.64417781157647e-16 20.6222654350997 1.24143657878348
18 260 viral reproductive process
GO:0032984 1.68503184580419e-16 26.3345294594282 0.8546813369317 16
179 macromolecular complex disassembly
GO:0006612 4.06752141945652e-16 28.6714975845411 0.730537679053353
15 153 protein targeting to membrane

GO:0043624 4.06752141945652e-16 28.6714975845411 0.730537679053353
15 153 cellular protein complex disassembly
GO:0043241 6.62512402719812e-16 27.6592851592852 0.754411459414573
15 158 protein complex disassembly
GO:0006401 1.47441449051459e-15 22.670260557053 0.978824994810048 16
205 RNA catabolic process
GO:0072594 3.04689059812065e-14 20.8597883597884 0.974050238737804
15 204 establishment of protein localization to organelle
GO:0016032 1.85494133300682e-13 12.2708333333333 2.15341498858211
19 451 viral reproduction
GO:0071845 2.43996182693504e-13 15.8986201070121 1.35603072451733
16 284 cellular component disassembly at cellular level
GO:0022411 3.02770833846076e-13 15.6603773584906 1.37512974880631
16 288 cellular component disassembly
GO:0033365 5.22368641413717e-10 9.19862928863383 2.25845962217148
16 473 protein localization to organelle
GO:0006605 5.38703733188493e-10 9.17788580374063 2.26323437824372
16 474 protein targeting
GO:0042274 1.38476537020871e-06 63.156043956044 0.0859456093003944 4
18 ribosomal small subunit biogenesis
GO:0016568 4.28761626116254e-06 6.49568965517241 2.00062279427029
11 419 chromatin modification
GO:0006338 4.90339324014159e-06 15.8316722037652 0.43927755864646
6 92 chromatin remodeling
GO:0042254 6.79928077522267e-06 11.3221263062244 0.711438654764376
7 149 ribosome biogenesis
GO:0006364 9.97651998625834e-06 13.8814382896016 0.49657463151339
6 104 rRNA processing
GO:0022613 1.12667152753571e-05 8.56085215683312 1.07432011625493
8 225 ribonucleoprotein complex biogenesis
GO:0016072 1.37700397185243e-05 13.0750915750916 0.52522316794685
6 110 rRNA metabolic process
GO:0071843 1.74514667624435e-05 8.03406429635938 1.14116670126635
8 239 cellular component biogenesis at cellular level
Tissue: Skin_Sun_Exposed_Lower_leg=>Adipose_Subcutaneous Type: asymmetric
SourceGene: RP11-930O11.1(ENSG00000259719.1)
TargetGeneSet: VPS13D SPEN UBR4 KPNA7 HIPK1 ASH1L PRRC2C LPGAT1 PXDN
RPS7 ASXL2 MZT2B USP37 USP40 RPL15 RPL29 DNAH1 CD38 TLR2 TBCA
AFF4 RPS14 TNIP1 RP11 AGXT2L2 RPL15P3 GPLD1 TAPBP BRD7P4 MRPS12
MYO1G GS1 CACNA2D1 VPS13B EIF3H TATDN1 CTA RPL8 VPS13A
RPS24 RPL27A RPS13 PACS1 ARID2 NACA SUGT1 VPS13C SEC11A ZNF205 SMG1
RPL13 VPS53 RPL26 RPS7P1 RPL17 STAT5B RPL27 HOXB7 RPS15 PTPRS RYR1
PLAUR RPL18 ZNF587 RALGAPB RPL12P4 CRYBB2 EIF3L EGFL6 HUWE1
GOBPID Pvalue OddsRatio ExpCount Count Size Term
GO:0006614 1.57744928897971e-19 51.8115942028985 0.443221922358314
15 105 SRP-dependent cotranslational protein targeting to membrane
GO:0006613 1.83195288190393e-19 51.2386526516961 0.447443083523632
15 106 cotranslational protein targeting to membrane
GO:0045047 1.83195288190393e-19 51.2386526516961 0.447443083523632
15 106 protein targeting to ER
GO:0072599 1.83195288190393e-19 51.2386526516961 0.447443083523632
15 106 establishment of protein localization to endoplasmic reticulum
GO:0006415 9.63146860465796e-19 55.3694390715667 0.384125666043872
14 91 translational termination
GO:0070972 9.8589662000356e-19 45.2311101730688 0.498097017507439
15 118 protein localization to endoplasmic reticulum
GO:0000184 1.12470471296061e-18 44.7930602006689 0.502318178672756
15 119 nuclear-transcribed mRNA catabolic process, nonsense-mediated decay
GO:0006413 1.35662886591628e-18 37.2653594771242 0.641616497128226
16 152 translational initiation
GO:0006612 1.51058855663393e-18 36.9907542579075 0.645837658293544
16 153 protein targeting to membrane
GO:0006414 7.92606098015508e-18 46.8052373158756 0.443221922358314

14 105 translational elongation
GO:0072594 1.59606545339533e-16 26.8595744680851 0.861116877724725
16 204 establishment of protein localization to organelle
GO:0000956 2.46953791183705e-16 30.1439864483343 0.71337623693862
15 169 nuclear-transcribed mRNA catabolic process
GO:0034623 3.83904766757613e-16 29.1858080393765 0.734482042765207
15 174 cellular macromolecular complex disassembly
GO:0006402 5.41099421018376e-16 28.4615897572686 0.751366687426476
15 178 mRNA catabolic process
GO:0032984 5.8881056306774e-16 28.2860551431601 0.755587848591793
15 179 macromolecular complex disassembly
GO:0019080 1.48143871610431e-15 30.9895946575555 0.637395335962909
14 151 viral genome expression
GO:0019083 1.48143871610431e-15 30.9895946575555 0.637395335962909
14 151 viral transcription
GO:0043624 1.78510167785488e-15 30.5394152762896 0.645837658293544
14 153 cellular protein complex disassembly
GO:0043241 2.81324041445762e-15 29.4686761229314 0.66694346412013
14 158 protein complex disassembly
GO:0006401 4.50225390318053e-15 24.370709382151 0.865338038890042 15
205 RNA catabolic process
GO:0019058 2.65071596581026e-14 21.3979468599034 0.975088229188292
15 231 viral infectious cycle
GO:0022415 1.50858042305001e-13 18.8265306122449 1.09750190298249
15 260 viral reproductive process
GO:0033365 4.8279469174266e-13 12.8203424482494 1.99660923119507
18 473 protein localization to organelle
GO:0006605 5.00398053106689e-13 12.7913096695226 2.00083039236039
18 474 protein targeting
GO:0071845 5.46390082962498e-13 17.1177468886375 1.19880977095011
15 284 cellular component disassembly at cellular level
GO:0022411 6.69346400676617e-13 16.8621595795509 1.21569441561138
15 288 cellular component disassembly
GO:0016032 3.47090805340659e-11 11.4063856960409 1.90374368555809
16 451 viral reproduction
GO:0042274 8.41122338662158e-07 72.0601503759399 0.075980900975711
4 18 ribosomal small subunit biogenesis
Tissue: Adipose_Subcutaneous=>Skin_Sun_Exposed_Lower_leg Type: cluster
SourceGene: RPL7P57(ENSG00000224401.2)
TargetGeneSet: RP11 EIF1AX RPL11 RP4 RPS8 UQCRH PRPF38A BCAS2
PAQR6 SNRPE SH3YL1 RPS7 UBC SNRPG COA5 RPL31 MRPS9 SSB
WDR75 EEF1B2 RPL37A CRBN THUMPD3 RPL32 CMC1 CCDC72 PRKAR2A-AS1
THOC7 CCDC58 RPL35A RPL34 EEF1A1P19 RAD17 NSA2 RPS23 COX7C
LYRM7 SLU7 NPM1 TDP2 BRD2 RPL10A RPL39P3 EEF1A1 C6orf203
MRPS12 ZDHHC14 LSM5 YAE1D1 SEC61G RPS20 UQCRB TATDN1 C9orf123 RPS6
NDUFB6 CHMP5 RBM18 TSC1 RPS24 RPL13AP5 C11orf58 EIF3M FAU
CLNS1A TMEM123 RPS25 NACA RPL14P1 C12orf45 RPL6 CCNA1 EBPL
COMMD6 PLDN RSL24D1 RPS3AP6 NEIL1 BTBD1 UNKL RPAIN RPL26 RPS7P1
RPL17 RPL19 RPL27 KPNA2 RPL38 RIOK3 C18orf8 LSM7 ALKBH6 ZNF829
RPS16 RPS19 SNRPD2 RPS21 EEF1B2P3 VBP1
GOBPID Pvalue OddsRatio ExpCount Count Size Term
GO:0006414 2.76353115943587e-40 107.608695652174 0.530413120199294
27 105 translational elongation
GO:0006614 2.87558700282201e-38 100.127659574468 0.530413120199294
26 105 SRP-dependent cotranslational protein targeting to membrane
GO:0006613 3.79813892675077e-38 98.8691489361702 0.535464673725002
26 106 cotranslational protein targeting to membrane
GO:0045047 3.79813892675077e-38 98.8691489361702 0.535464673725002
26 106 protein targeting to ER
GO:0072599 3.79813892675077e-38 98.8691489361702 0.535464673725002
26 106 establishment of protein localization to endoplasmic reticulum
GO:0006415 4.79075044474379e-38 112.941919191919 0.459691370839388
25 91 translational termination

GO:0070972	8.59890237110418e-37	85.9010175763182	0.596083316033493
26	118	protein localization to endoplasmic reticulum
GO:0006413	1.74243825363436e-35	66.9271304347826	0.76783613590755
27	152	translational initiation
GO:0000184	9.03601568062273e-35	79.1445035460993	0.6011348695592 25
119	nuclear-transcribed mRNA catabolic process, nonsense-mediated decay
GO:0000956	3.75840299789116e-34	58.8444580526638	0.853712545844578
27	169	nuclear-transcribed mRNA catabolic process
GO:0006612	1.36114060192244e-33	62.0750544479812	0.772887689433257
26	153	protein targeting to membrane
GO:0006402	1.66768097955149e-33	55.3021883098186	0.899176527575946
27	178	mRNA catabolic process
GO:0019080	5.78130277058681e-32	58.912037037037 0.762784582381842	25
151	viral genome expression
GO:0019083	5.78130277058681e-32	58.912037037037 0.762784582381842	25
151	viral transcription
GO:0043624	8.21753702828212e-32	57.9833984375	0.772887689433257	25
153	cellular protein complex disassembly
GO:0006401	9.24943435355489e-32	46.8246213971666	1.03556847277005
27	205	RNA catabolic process
GO:0043241	1.93655810887696e-31	55.7839912280702	0.798145457061795
25	158	protein complex disassembly
GO:0034623	2.48953699439478e-30	49.7378355704698	0.878970313473116
25	174	cellular macromolecular complex disassembly
GO:0072594	3.69737420890115e-30	44.1310064546976	1.03051691924434
26	204	establishment of protein localization to organelle
GO:0032984	5.245265723562e-30	48.1060606060606	0.904228081101654
25	179	macromolecular complex disassembly
GO:0022415	2.4576661809878e-27	33.4373522458629	1.31340391668397
26	260	viral reproductive process
GO:0019058	3.92667099875934e-27	35.8313106796116	1.16690886443845
25	231	viral infectious cycle
GO:0071845	7.4883102216333e-25	28.392454954955 1.43464120130095	25
284	cellular component disassembly at cellular level
GO:0022411	1.06497711733202e-24	27.9527091254753	1.45484741540378
25	288	cellular component disassembly
GO:0033365	3.74358203733785e-23	19.481847690387 2.38938481765968	28
473	protein localization to organelle
GO:0006605	3.96627331353422e-23	19.4367713004484	2.39443637118538
28	474	protein targeting
GO:0022613	7.67232272328643e-23	30.1217038539554	1.1365995432842 22
225	ribonucleoprotein complex biogenesis
GO:0071843	2.94216983843665e-22	28.1505376344086	1.20732129264411
22	239	cellular component biogenesis at cellular level
GO:0016032	3.85638659378562e-21	18.1616020025031	2.27825064009411
26	451	viral reproduction
GO:0042254	2.28979588109197e-17	30.0646352723915	0.752681475330427
16	149	ribosome biogenesis
GO:0042274	1.20031454780352e-14	176.836923076923	0.0909279634627361
8	18	ribosomal small subunit biogenesis
GO:0034660	4.20576973391884e-14	15.9828091684435	1.43969275482666
17	285	ncRNA metabolic process
GO:0006364	1.28620598802652e-13	30.5473984319316	0.525361566673587
12	104	rRNA processing
GO:0016072	2.55627992648516e-13	28.6651053864169	0.555670887827832
12	110	rRNA metabolic process
GO:0034470	1.92582378338584e-11	16.4423351158645	1.01031070514151
13	200	ncRNA processing
GO:0022618	1.07038452366635e-09	23.3699127906977	0.479897584942219
9	95	ribonucleoprotein complex assembly
GO:0071826	1.69853349330779e-09	22.078125	0.505155352570756	9
100	ribonucleoprotein complex subunit organization
GO:0000387	4.39079463156449e-07	40.5882352941176	0.156598159296934

5	31	spliceosomal snRNP assembly
GO:0008380	6.07126667275544e-07	7.99865591397849	1.63165178880354
11	323	RNA splicing
GO:0000462	2.44679800247836e-06	205.357142857143	0.0303093211542454
3	6	maturation of SSU-rRNA from tricistronic rRNA transcript (SSU-rRNA, 5.8S rRNA, LSU-rRNA)
GO:0030490	4.26634606660256e-06	154.007142857143	0.0353608746799529
3	7	maturation of SSU-rRNA
GO:0006397	4.73643415142512e-06	6.39715663451945	2.01556985675732
11	399	mRNA processing
GO:0000375	1.0050210757565e-05	8.68090317642556	1.05577468687288
8	209	RNA splicing, via transesterification reactions
Tissue: Skin_Sun_Exposed_Lower_leg=>Adipose_Subcutaneous	Type:	asymmetric
SourceGene:	RPP30(ENSG00000148688.6)
TargetGeneSet:	ZBTB17	KDM1A	KHDRBS1	RP4	EIF2C3	LEPRE1	HECTD3	HSPB11
MRPL37	LEPROT	LRRC40	GNG5	COL24A1	RPL5	CCDC18	RP11	KCNJ9	SNRPE
B3GALNT2	PRORSD1P	ETAA1	FAM136A	UBTFL6	MMADHC	MLK7-AS1
NIF3L1	EEF1B2	TNS1	KCNE4	RPS12P5	RPSA	RPL14	IMPDH2	QARS	NICN1
SPCS1	SNX4	COMMD2	EGFEM1P	EXOC7	OSTC	CCDC109B	DCTD	WWC2	FRG1
AGGF1P1	CTD	CDK7	RASA1	CTB	NPM1	GPLD1	RPS18	YIPF3	EEF1A1
RARS2	MRPS12	EPM2A	CTA	NT5C3	SSBP1	GIMAP7	GIMAP6	CSMD1	CCDC25
C8orf34	DPY19L4	FAM84B	RPS6	DCTN3	HRCT1	SLC35D2	GGTA1P	PSMB7	GDI2
ITGB1	RPL13AP5	GSTO1	RNU4-5P	LHPP	SNORA23	ZBED5	PEX16	NDUFS3
PPP2R5B	CCDC82	MCAM	NTM	HDAC7	TUBA1C	HNRNPA1	GRIP1	SLC35E3	METAP2
SLC25A3	IKBIP	NUP37	ALKBH2	FBXO21	RPLP0	RFXAP	MRPS31	DGKH	EBPL	NEK3
LGALS3	MOK	CCNDBP1	PARP16	RAB11A	RPS17L	SEC11A	RPS2	LITAF	MYH10
MPRIP-AS1	RPS7P1	ALDOC	TMEM101	MAPT	ANKRD40	CEP95	MYOM1	TTC39C
HNRNPA1P7	HAUS1	PIP5K1C	SLC25A23	RNASEH2A	AP1M1	PDCD5
ZNF585A	RPS19	MEGF8	CACNG7	RPL28	RPS5	SLC24A3	EPB41L1	WFDC13	CSTF1	RPL3
EEF1B2P3	RGN	PORCN	YIPF6	RBM41	RP1-177G6.2	SLC10A3
GOBPID	Pvalue	OddsRatio	ExpCount	Count	Size	Term
GO:0070972	7.73350920734173e-15	22.5427395525538	0.873711161857311
15	118	protein localization to endoplasmic reticulum
GO:0006414	3.26182500051036e-14	23.5781637717122	0.777454847415404
14	105	translational elongation
GO:0006614	3.26182500051036e-14	23.5781637717122	0.777454847415404
14	105	SRP-dependent cotranslational protein targeting to membrane
GO:0006613	3.73551811974013e-14	23.3202431042543	0.78485917929555
14	106	cotranslational protein targeting to membrane
GO:0045047	3.73551811974013e-14	23.3202431042543	0.78485917929555
14	106	protein targeting to ER
GO:0072599	3.73551811974013e-14	23.3202431042543	0.78485917929555
14	106	establishment of protein localization to endoplasmic reticulum
GO:0006612	3.84040546543063e-13	16.7840264650284	1.13286277766245
15	153	protein targeting to membrane
GO:0006415	2.80259329975766e-12	22.8087941372418	0.67379420109335
12	91	translational termination
GO:0072594	2.53373654071825e-11	12.2110075914424	1.51048370354993
15	204	establishment of protein localization to organelle
GO:0000184	7.10394135326821e-11	16.8070831283817	0.881115493737458
12	119	nuclear-transcribed mRNA catabolic process, nonsense-mediated decay
GO:0019080	8.19149411134448e-11	14.2366635831021	1.11805411390215
13	151	viral genome expression
GO:0019083	8.19149411134448e-11	14.2366635831021	1.11805411390215
13	151	viral transcription
GO:0006401	3.27041027786169e-10	11.154759894162	1.51788803543007	14
205	RNA catabolic process
GO:0006402	6.40545495569124e-10	11.8843971631206	1.31797107466611
13	178	mRNA catabolic process
GO:0006413	1.24446052195603e-09	12.8156390977444	1.1254584457823	12
152	translational initiation
GO:0043624	1.34214153559714e-09	12.7238521836506	1.13286277766245
12	153	cellular protein complex disassembly

GO:0043241	1.94244085203402e-09	12.2837779379957	1.16988443706318
12	158	protein complex disassembly
GO:0000956	4.1906640972259e-09	11.414280925243 1.25133208774479	12
169	nuclear-transcribed mRNA catabolic process
GO:0034623	5.83519367848991e-09	11.058089668616 1.28835374714553	12
174	cellular macromolecular complex disassembly
GO:0032984	8.03950940262332e-09	10.723227229751 1.32537540654626	12
179	macromolecular complex disassembly
GO:0019058	1.52478347774591e-08	8.96144837009565	1.71040066431389
13	231	viral infectious cycle
GO:0071843	2.28720374097545e-08	8.63933345885897	1.76963531935506
13	239	cellular component biogenesis at cellular level
GO:0022415	6.17921402246615e-08	7.89305711086226	1.92512628883814
13	260	viral reproductive process
GO:0022613	1.020867276516e-07	8.38013343217198	1.66597467303301
12	225	ribonucleoprotein complex biogenesis
GO:0042254	1.49955979284037e-07	10.5354891344656	1.10324545014186
10	149	ribosome biogenesis
GO:0071845	1.72617995788958e-07	7.18179320091073	2.10283025396166
13	284	cellular component disassembly at cellular level
GO:0016032	1.98402473283091e-07	5.62192749778957	3.33935367794616
16	451	viral reproduction
GO:0022411	2.02832419521597e-07	7.07531914893617	2.13244758148225
13	288	cellular component disassembly
GO:0033365	2.0184801718855e-06	4.94327890639833	3.50224897930939
15	473	protein localization to organelle
GO:0006605	2.07177738734815e-06	4.9321540210287 3.50965331118954	15
474	protein targeting
GO:0042255	4.82828383184175e-06	46.3818770226537	0.118469310082347
4	16	ribosome assembly
GO:0000028	7.76702447065936e-06	137.894230769231	0.0444259912808802
3	6	ribosomal small subunit assembly
GO:0042274	8.02575663301537e-06	39.750346740638 0.133277973842641	4
18	ribosomal small subunit biogenesis
GO:0006364	1.17964469341278e-05	10.2813402061856	0.770050515535257
7	104	rRNA processing
GO:0016072	1.70406276135602e-05	9.67834951456311	0.814476506816137
7	110	rRNA metabolic process
Tissue: Skin_Sun_Exposed_Lower_leg=>Adipose_Subcutaneous	Type:	asymmetric
SourceGene:	RSPH9(ENSG00000172426.9)
TargetGeneSet:	TNFRSF25	RP1	RP4	COL24A1 PKN2	RPL5	RP11
ATP5F1	U2	ADAMTSL4	PIGM	KIAA1383	GPR113	UBC	TPRKB
MRPS9	RBM43	EEF1B2	QARS	GRM2	SPCS1	RPL10AP6	ATG3	GPR171
RPL22L1 C3orf43 RPL35A	NAAA	RPL37	NDUFAF2 PPWD1	BTF3	RPS23	NPM1
RPL10A	C6orf130	SENP6	RARS2	UFL1	GOPC	MRPS12	PKD1L1	ECD	POT1
SSBP1	ZNF596	CHCHD7	RPL7	C8orf59 RPL30	U6	ZNF250	RPS6	PSMB7	DPM2
RPS24	BLOC1S2 ZBED5	IMMP1L	EIF3M	PEX16	PPP2R5B RPS25	CBX3P4	UHRF1BP1L
NUP37	RPLP0	ALG5	TPT1	TMTC4	LGALS3	RSL24D1 UNC45A	UBE2I	LITAF
RPL23A	RPL19	VPS25	FECH	TEAD2	RPS9	DTD1	IFT52	CSTF1	MRPL39
REPS2	UXT	RPS4X
GOBPID	Pvalue	OddsRatio	ExpCount	Count	Size	Term
GO:0070972	3.37340022439387e-22	45.8903571428571	0.604248840910664
18	118	protein localization to endoplasmic reticulum
GO:0006612	1.22713074263775e-21	36.7187245590231	0.783475192028233
19	153	protein targeting to membrane
GO:0006414	1.85824725656262e-21	48.4276315789474	0.537679053352709
17	105	translational elongation
GO:0006614	1.85824725656262e-21	48.4276315789474	0.537679053352709
17	105	SRP-dependent cotranslational protein targeting to membrane
GO:0006613	2.20489973980139e-21	47.8801498127341	0.542799806241783
17	106	cotranslational protein targeting to membrane
GO:0045047	2.20489973980139e-21	47.8801498127341	0.542799806241783
17	106	protein targeting to ER

GO:0072599	2.20489973980139e-21	47.8801498127341	0.542799806241783
17	106	establishment of protein localization to endoplasmic reticulum
GO:0006415	7.68629683613963e-21	52.6050574712644	0.465988512905681
16	91	translational termination
GO:0000184	7.13699961349572e-19	38.2296618680951	0.609369593799737
16	119	nuclear-transcribed mRNA catabolic process, nonsense-mediated decay
GO:0006413	1.30190114620767e-18	31.4638076673164	0.77835443913916
17	152	translational initiation
GO:0072594	8.44615921303806e-18	24.5236175115207	1.04463358937098
18	204	establishment of protein localization to organelle
GO:0019080	3.59854005924456e-17	29.1024265644955	0.773233686250086
16	151	viral genome expression
GO:0019083	3.59854005924456e-17	29.1024265644955	0.773233686250086
16	151	viral transcription
GO:0043624	4.45746211556904e-17	28.6735464384596	0.783475192028233
16	153	cellular protein complex disassembly
GO:0043241	7.51285448947764e-17	27.6542010684798	0.8090789564736 16
158	protein complex disassembly
GO:0000956	2.22931742046762e-16	25.6461573135001	0.865407238253408
16	169	nuclear-transcribed mRNA catabolic process
GO:0034623	3.56379789069896e-16	24.8258402444347	0.891011002698775
16	174	cellular macromolecular complex disassembly
GO:0006402	5.13289498287159e-16	24.2060451255854	0.911494014255069
16	178	mRNA catabolic process
GO:0032984	5.61524128513149e-16	24.0558493759255	0.916614767144142
16	179	macromolecular complex disassembly
GO:0006401	4.87147113841289e-15	20.7086298120781	1.04975434226005
16	205	RNA catabolic process
GO:0019058	3.1930834512106e-14	18.1709703287891	1.18289391737596
16	231	viral infectious cycle
GO:0022415	2.01364976172497e-13	15.9785189372527	1.33139575115909
16	260	viral reproductive process
GO:0071845	7.85415494261642e-13	14.5229027277406	1.45429382049685
16	284	cellular component disassembly at cellular level
GO:0022411	9.73321335290367e-13	14.3052738336714	1.47477683205315
16	288	cellular component disassembly
GO:0006605	1.7900267005612e-12	10.5701498501498	2.4272368694208 19
474	protein targeting
GO:0016032	8.18141692971715e-12	10.35103926097	2.30945955297211	18
451	viral reproduction
GO:0033365	1.8035401165751e-11	9.83500784929356	2.42211611653173
18	473	protein localization to organelle
GO:0071843	3.36134997539575e-08	10.8353522695628	1.22385994048855
11	239	cellular component biogenesis at cellular level
GO:0042254	6.45674587116319e-08	14.0805494505494	0.76299218047194
9	149	ribosome biogenesis
GO:0022613	2.06240591122515e-07	10.2921511627907	1.15216940004152
10	225	ribonucleoprotein complex biogenesis
Tissue: Whole_Blood=>Adipose_Subcutaneous	Type:	asymmetric

SourceGene:	A2ML1(ENSG00000166535.14)	alpha-2-macroglobulin-like 1

TargetGeneSet:	RPL22	RP11	RP1	RPL11	RP4	RPL5	UBC	FABP1	COA5

LYG2	RPL31	MTX2	EEF1B2	MYEOV2	PA2G4P4	VWA5B2	RPL35A	RPL34	CTD	
UQCRQ	RPS14	COX7A2	RWDD1	MRPS12	RPS20	SNHG6	COPS5	RPS6	CBWD6	
RPS24	RBM4	RPS25	KRT5	ARL6IP4	EXOSC8	TPT1	RPS3AP6	RPLP1	C15orf38	
KRT10	KRT14	DNAJC7	RPL27	DSC1	C19orf80		CASP14	LGALS7B	ROMO1	TH1L
RPS19BP1		RP6	ARSF	RPL39	PSMA6P1					

GOBPID	Pvalue	OddsRatio	ExpCount	Count	Size	Term
GO:0006414	3.57E-25	117.0500511	0.27610546	16	105	translational elongation
GO:0006413	2.25E-24	85.61763668	0.399695523	17	152	translational initiation
GO:0006415	3.98E-24	123.0291762	0.239291399	15	91	translational termination
GO:0006614	3.96E-23	103.7898551	0.27610546	15	105	SRP-dependent cotranslational protein targeting to membrane
GO:0006613	4.61E-23	102.6421405	0.278735036	15	106	cotranslational protein targeting to membrane
GO:0045047	4.61E-23	102.6421405	0.278735036	15	106	protein targeting to ER
GO:0072599	4.61E-23	102.6421405	0.278735036	15	106	establishment of protein localization to endoplasmic reticulum

GO:0019080	1.64E-22	76.91851852	0.397065947	16	151	
viral genome expression						
GO:0019083	1.64E-22	76.91851852	0.397065947	16	151	
viral transcription						
GO:0070972	2.52E-22	90.60785141	0.310289945	15	118	
protein localization to endoplasmic reticulum						
GO:0000184	2.88E-22	89.73035117	0.312919521	15	119	
"nuclear-transcribed mRNA catabolic process, nonsense-mediated decay"						
GO:0000956	1.06E-21	67.78371955	0.444398312	16	169	
nuclear-transcribed mRNA catabolic process						
GO:0006402	2.49E-21	63.97755331	0.468064494	16	178	mRNA
catabolic process						
GO:0006612	1.47E-20	67.46219282	0.402325099	15	153	
protein targeting to membrane						
GO:0043624	1.47E-20	67.46219282	0.402325099	15	153	
cellular protein complex disassembly						
GO:0072594	2.32E-20	55.02901354	0.536433465	16	204	
establishment of protein localization to organelle						

GO:0043241	2.42E-20	65.0805716	0.415472978	15	158	protein complex disassembly
GO:0006401	2.52E-20	54.73400673	0.539063041	16	205	RNA catabolic process
GO:0034623	1.07E-19	58.4659557	0.457546191	15	174	cellular macromolecular complex disassembly
GO:0032984	1.65E-19	56.6635737	0.47069407	15	179	macromolecular complex disassembly
GO:0019058	1.76E-19	48.02706131	0.607432012	16	231	viral infectious cycle
GO:0022415	1.19E-18	42.23248882	0.68368971	16	260	viral reproductive process
GO:0071845	1.80E-16	34.29125586	0.746799529	15	284	cellular component disassembly at cellular level
GO:0022411	2.21E-16	33.77926421	0.757317833	15	288	cellular component disassembly
GO:0016032	7.37E-15	23.36969697	1.185938689	16	451	viral reproduction

GO:0033365	1.55E-14	22.20966779	1.243789357	16	473
protein localization to organelle

GO:0006605	1.60E-14	22.15958714	1.246418933	16	474
protein targeting

GO:0042254	3.98E-09	26.99196217	0.391806795	8	149
ribosome biogenesis

GO:0022613	5.05E-09	20.39798851	0.591654557	9	225
ribonucleoprotein complex biogenesis

GO:0071843	8.55E-09	19.13748126	0.628468618	9	239
cellular component biogenesis at cellular level

GO:0006364	8.57E-09	33.32623878	0.273475884	7	104	rRNA
processing

GO:0016072	1.27E-08	31.3717507	0.289253339	7	110	rRNA
metabolic process

GO:0042274	1.21E-07	121	0.047332365	4	18	ribosomal
small subunit biogenesis

GO:0000028	3.34E-07	411.7142857	0.015777455	3	6
ribosomal small subunit assembly

GO:0034470 7.68E-07 16.63713856 0.525915162 7 200
ncRNA processing

GO:0042273 3.63E-06 137.1809524 0.03155491 3 12
ribosomal large subunit biogenesis

GO:0034660 8.06E-06 11.48120214 0.749429105 7 285
ncRNA metabolic process

GO:0042255 9.18E-06 94.94505495 0.042073213 3 16
ribosome assembly

Tissue: Whole_Blood=>Adipose_Subcutaneous Type: asymmetric

SourceGene: ACA64(ENSG00000239005.1) "small nucleolar RNA, H/ACA box 78"

TargetGeneSet: RPL11 TAF12 RP11 C2orf28 CCDC115 RPL24 RPL34 RPS3A NDUFS4 MIP BTF3 CTB NIPAL4 CSNK2B RPS20 RPL30 RPS6 RPL35 NUP188 RPS24 SFR1 FAM53B FAU RPS25 DNAL1 RPS3AP6 RPS15A GABARAP CTD RPL26 RPL17 C19orf53 UBA52 CHMP4B LAMA5 HCFC1 AMELY

GOBPID Pvalue OddsRatio ExpCount Count Size Term

GO:0006415 1.50E-25 166.5208978 0.201508546 15 91
translational termination

GO:0006414 1.50E-24 140.4803922 0.232509861 15 105
translational elongation

GO:0006614	1.50E-24	140.4803922	0.232509861	15	105
SRP-dependent cotranslational protein targeting to membrane					
GO:0006613	1.75E-24	138.9269554	0.234724241	15	106
cotranslational protein targeting to membrane					
GO:0045047	1.75E-24	138.9269554	0.234724241	15	106
protein targeting to ER					
GO:0072599	1.75E-24	138.9269554	0.234724241	15	106
establishment of protein localization to endoplasmic reticulum					
GO:0070972	9.61E-24	122.6384923	0.261296796	15	118
protein localization to endoplasmic reticulum					
GO:0000184	1.10E-23	121.4507919	0.263511176	15	119
"nuclear-transcribed mRNA catabolic process, nonsense-mediated decay"					
GO:0019080	4.62E-22	92.66652249	0.334371324	15	151
viral genome expression					
GO:0019083	4.62E-22	92.66652249	0.334371324	15	151
viral transcription					
GO:0006413	5.12E-22	91.98368398	0.336585703	15	152
translational initiation					
GO:0006612	5.67E-22	91.31074169	0.338800083	15	153
protein targeting to membrane					
GO:0043624	5.67E-22	91.31074169	0.338800083	15	153
cellular protein complex disassembly					

GO:0043241 9.34E-22 88.08720691 0.349871981 15 158
protein complex disassembly

GO:0000956 2.65E-21 81.73223835 0.374230157 15 169
nuclear-transcribed mRNA catabolic process

GO:0034623 4.16E-21 79.13429523 0.385302055 15 174
cellular macromolecular complex disassembly

GO:0006402 5.91E-21 77.1706965 0.394159574 15 178 mRNA
catabolic process

GO:0032984 6.44E-21 76.69476327 0.396373953 15 179
macromolecular complex disassembly

GO:0022415 3.53E-20 58.0942623 0.575738703 16 260
viral reproductive process

GO:0072594 4.80E-20 66.43323996 0.451733444 15 204
establishment of protein localization to organelle

GO:0006401 5.18E-20 66.07894737 0.453947824 15 205 RNA
catabolic process

GO:0016032 1.78E-19 41.52886836 0.998685212 18 451
viral reproduction

GO:0019058 3.20E-19 58.01879085 0.511521694 15 231
viral infectious cycle

GO:0071845	7.30E-18	46.41373278	0.628883814	15	284
cellular component disassembly at cellular level					
GO:0022411	9.02E-18	45.72074984	0.637741333	15	288
cellular component disassembly					
GO:0006605	1.60E-17	34.6249453	1.049615944	17	474
protein targeting					
GO:0033365	5.02E-16	30.55142232	1.047401564	16	473
protein localization to organelle					
GO:0042274	7.87E-06	99.33793103	0.039858833	3	18
ribosomal small subunit biogenesis					
GO:0022613	8.61E-06	14.96311907	0.498235416	6	225
ribonucleoprotein complex biogenesis					
GO:0071843	1.21E-05	14.05018158	0.529236731	6	239
cellular component biogenesis at cellular level					
GO:0042254	1.75E-05	18.35776749	0.329942565	5	149
ribosome biogenesis					

Tissue: Whole_Blood=>Adipose_Subcutaneous Type: asymmetric

SourceGene: ANKRD18EP(ENSG00000217165.1) "ankyrin repeat domain 18E, pseudogene"

TargetGeneSet: HSPG2 RPL11 SYNC RPS8 RPL5 RP11 PRX CENPO PPP1CB UBC ANKAR EEF1B2 RPL15 MAP4 RPL29 MIR135A1 RELL1 AIMP1 RPL34 NDUFAF2 BTF3 NSA2 TBCA CTB SYNPO NPM1 JARID2-AS1 GPLD1 RPL10A FILIP1 MRPS12 SFT2D1 RPS20 RPL30 EIF3H RPS6 CLTA ANP32B RPL35 RPL12 RPL7A RPS24 RPL27A EIF3M ZNF705E SNORD15B RPS25 PS5 NAP1L1 CCDC59 RPL18AP3 RPL31P52 RPL21 RSL24D1 RPS3AP6 RPS15A RPL13 RPL26 RPL17 RPL19 NT5C C18orf1 RPS15 RPL36 RPL18 RPS9 ZNF154 ZNF671 SNRPB2 MORC2

GOBPID	Pvalue	OddsRatio	ExpCount	Count	Size	Term
GO:0006414	1.69E-46	185.9480519	0.406892257	28	105	translational elongation
GO:0006415	3.43E-46	208.4789871	0.352639956	27	91	translational termination
GO:0006614	2.99E-44	170.8925729	0.406892257	27	105	SRP-dependent cotranslational protein targeting to membrane
GO:0006613	4.00E-44	168.7175906	0.410767421	27	106	cotranslational protein targeting to membrane
GO:0045047	4.00E-44	168.7175906	0.410767421	27	106	protein targeting to ER

GO:0072599	4.00E-44	168.7175906	0.410767421	27	106
establishment of protein localization to endoplasmic reticulum					
GO:0006413	1.51E-43	124.6275218	0.589024981	29	152
translational initiation					
GO:0070972	1.06E-42	146.3463433	0.457269393	27	118
protein localization to endoplasmic reticulum					
GO:0000184	1.37E-42	144.7455022	0.461144557	27	119
"nuclear-transcribed mRNA catabolic process, nonsense-mediated decay"					
GO:0019080	1.64E-39	107.1515573	0.585149817	27	151
viral genome expression					
GO:0019083	1.64E-39	107.1515573	0.585149817	27	151
viral transcription					
GO:0006612	2.41E-39	105.4359606	0.592900145	27	153
protein targeting to membrane					

GO:0043624	2.41E-39	105.4359606	0.592900145	27	153	cellular protein complex disassembly
GO:0043241	6.18E-39	101.3761516	0.612275967	27	158	protein complex disassembly
GO:0000956	4.38E-38	93.45094706	0.654902775	27	169	nuclear-transcribed mRNA catabolic process
GO:0034623	1.02E-37	90.24067558	0.674278597	27	174	cellular macromolecular complex disassembly
GO:0006402	1.96E-37	87.82553094	0.689779254	27	178	mRNA catabolic process
GO:0032984	2.31E-37	87.24160617	0.693654418	27	179	macromolecular complex disassembly
GO:0072594	9.77E-36	74.78784337	0.790533527	27	204	establishment of protein localization to organelle

GO:0006401	1.12E-35	74.36245641	0.794408691	27	205	RNA catabolic process
GO:0019058	3.31E-34	64.76622718	0.895162965	27	231	viral infectious cycle
GO:0022415	9.14E-33	56.58931478	1.007542731	27	260	viral reproductive process
GO:0071845	1.07E-31	51.21776466	1.100546675	27	284	cellular component disassembly at cellular level
GO:0022411	1.57E-31	50.41854935	1.116047332	27	288	cellular component disassembly
GO:0016032	3.29E-26	30.67802537	1.747699121	27	451	viral reproduction
GO:0033365	1.18E-25	29.11883408	1.832952737	27	473	protein localization to organelle

GO:0006605	1.25E-25	29.05160842	1.836827901	27	474	protein targeting
GO:0042254	3.49E-13	28.38354346	0.577399488	12	149	ribosome biogenesis
GO:0022613	2.34E-12	20.22586661	0.871911978	13	225	ribonucleoprotein complex biogenesis
GO:0071843	5.03E-12	18.95420868	0.926164279	13	239	cellular component biogenesis at cellular level
GO:0006364	5.85E-09	24.82465278	0.403017092	8	104	rRNA processing
GO:0042274	6.00E-09	108.4615385	0.069752958	5	18	ribosomal small subunit biogenesis
GO:0016072	9.14E-09	23.35457516	0.426268078	8	110	rRNA metabolic process

GO:0034470 9.41E-07 12.32899306 0.77503287 8 200
ncRNA processing

GO:0034660 1.34E-06 9.795790934 1.104421839 9 285
ncRNA metabolic process

GO:0042273 1.18E-05 90.47798742 0.046501972 3 12
ribosomal large subunit biogenesis

Tissue: Whole_Blood=>Adipose_Subcutaneous Type: cluster
SourceGene: ENSG00000229344.1
TargetGeneSet: LDLRAD2 HSPG2 RPL11 ARID1A KPNA7 ZMYM4 THRAP3 RPS8
RPS15AP10 RPL5 RP11 SNX7 RPRD2 SPRR2D JTB RPS27 UBAP2L SSR2
RAB25 SHISA4 NENF CICP13 NBAS PPP1CB UBC TMEM131 EIF5B RANBP2
UGGT1 PLEKHA3 UBE2E3 TRIP12 BRK1 UBE2E1 RPL15 RAD54L2 RPL29 KCTD6 PCNP
RPL24 RYK GPR87 EIF4G1 PPP1R2 HTT BOD1L DANCR CISD2 RPL34
NDUFC1 RPS3A NIPBL CTD TAF9 BTF3 AP3S1 CTB MTND5P11 SIL1
HDAC3 RPS14 GNB2L1 RP3 TMEM14C RPL15P3 DEK RSL24D1P1 PPP1R10
AGPAT1 RPS18 GLO1 MRPL32 MTRNR2L9 WDR11 RWDD1 MRPS12 RAB32
IGF2R PSMB1 STARD3NL CLDN3 SPDYE2 ATXN7L1 FAM3C DRP2 HMBOX1
HOOK3 RPS20 ARFGEF1 RPL30 TG NFIB RPS6 CLTA PCSK5 NIPSNAP3A
ZNF462 ATP6V1G1 RPL12 PTGES RPL7A SEC16A CALML5 PRINS KIAA0913
RPS24 MRPL43 GBF1 RPL27A MTRNR2L8 CKAP5 BAD ZNHIT2 CCDC90B
C11orf73 FDX1 UBE4A DDX6 ERC1 A2M SCAF11 MLL4 PFDN5 CBX5
MYF6 USP44 GTF3A ESD NUDT15 MED4 DNAJC3 CCNB1IP1 CHD8
CGRRF1 DHRS7 YLPM1 C14orf142 CCNK HERC2P10 USP8 RPS27L
CALML4 C15orf40 SEC11A MPG SPSB3 TSC2 NTAN1 RPS15A GTF3C1
LONP2 FBXL8 PIEZO1 RPL13 VPS53 WDR81 POLR2A RPL26 RPL23A TAF15
RPL19 CDK12 KRT14 RPL27 XYLT2 SUPT4H1 CLTC TNRC6C ROCK1P1 DSG1
RPS15 AP3D1 NFIC ARRDC5 PTPRS NDUFA11 ZNF121 EIF3G ZNF791 C19orf43
BRD4 UPF1 KRTDAP SBSN ZNF526 ERF ZNF428 RPL18 NUCB1 NUCB1-AS1
RPL13A KLK7 CCDC106 ZNF587 RPS5 KIF3B CHMP4B SCAND1 TOP1 NCOA3
RPL12P4 FAM211B TTC28 EIF3L RPL3 TNRC6B EP300 TMSB4X DYNLT3 UXT
WDR45 HUWE1 RPS4X COX7B RPL39 HCFC1 MT

GOBPID	Pvalue	OddsRatio	ExpCount	Count	Size	Term
GO:0006415	1.06365468144771e-40	56.5663129973475	1.10829700366757	33	91	translational termination
GO:0006614	6.07105534805468e-40	47.9008133306884	1.27880423500104	34	105	SRP-dependent cotranslational protein targeting to membrane
GO:0006613	8.85206189936669e-40	47.2321987480438	1.29098332295343	34	106	cotranslational protein targeting to membrane
GO:0045047	8.85206189936669e-40	47.2321987480438	1.29098332295343	34	106	protein targeting to ER
GO:0072599	8.85206189936669e-40	47.2321987480438	1.29098332295343	34	106	establishment of protein localization to endoplasmic reticulum

GO:0070972	5.95242744048001e-38	40.4505365526492	1.43713237838212
34	118	protein localization to endoplasmic reticulum
GO:0000184	8.25326467032465e-38	39.9718309859155	1.44931146633451
34	119	nuclear-transcribed mRNA catabolic process, nonsense-mediated decay
GO:0006413	9.25877719238335e-37	31.3869458128079	1.85122136876341
36	152	translational initiation
GO:0006414	1.27246393757952e-36	43.2328767123288	1.27880423500104
32	105	translational elongation
GO:0019080	2.22646549820877e-35	30.2986671557838	1.83904228081102
35	151	viral genome expression
GO:0019083	2.22646549820877e-35	30.2986671557838	1.83904228081102
35	151	viral transcription
GO:0006612	1.08039642679469e-33	28.4828973843058	1.8634004567158 34
153	protein targeting to membrane
GO:0000956	1.57734892540332e-33	26.1953530221234	2.05826586395405
35	169	nuclear-transcribed mRNA catabolic process
GO:0006402	1.09225596040921e-32	24.5310717651143	2.16787765552557
35	178	mRNA catabolic process
GO:0043624	3.03514988855692e-32	27.2211538461538	1.8634004567158 33
153	cellular protein complex disassembly
GO:0043241	9.48800926251924e-32	26.1230769230769	1.92429589647775
33	158	protein complex disassembly
GO:0022415	1.13073206866945e-31	18.1030485186775	3.16656286762162
39	260	viral reproductive process
GO:0034623	1.16901936490966e-31	24.1745472837022	2.11916130371601
34	174	cellular macromolecular complex disassembly
GO:0032984	3.23586360878366e-31	23.3326857697912	2.18005674347796
34	179	macromolecular complex disassembly
GO:0019058	4.06249468988904e-31	19.3205147222428	2.81336931700228
37	231	viral infectious cycle
GO:0072594	1.63214336505591e-30	20.7188719627345	2.48453394228773
35	204	establishment of protein localization to organelle
GO:0006401	1.949266726902e-30	20.5955360867751	2.49671303024012
35	205	RNA catabolic process
GO:0033365	7.42989514764804e-28	11.1135585360634	5.76070860148087
45	473	protein localization to organelle
GO:0016032	1.25639378342209e-26	10.9885190918473	5.49276866652827
43	451	viral reproduction
GO:0006605	9.55009675508157e-26	10.384871691991 5.77288768943326	43
474	protein targeting
GO:0071845	2.00161424269755e-25	13.9824546412601	3.458860978479	35
284	cellular component disassembly at cellular level
GO:0022411	3.24250576211197e-25	13.7574636279539	3.50757733028856
35	288	cellular component disassembly
GO:0042274	1.90753772942983e-06	32.0782726045884	0.219223583143035
5	18	ribosomal small subunit biogenesis
GO:0022613	3.78516319362095e-06	5.29051394837365	2.74029478928794
13	225	ribonucleoprotein complex biogenesis
GO:0071843	7.31058455181605e-06	4.95784244530105	2.91080202062141
13	239	cellular component biogenesis at cellular level
GO:0006417	1.27264762000865e-05	5.56449704142012	2.19223583143035
11	180	regulation of translation
GO:0042254	1.41161258095143e-05	6.12637600762763	1.81468410490623
10	149	ribosome biogenesis
Tissue: Whole_Blood=>Adipose_Subcutaneous	Type:	asymmetric
SourceGene:	ENSG00000252229.1
TargetGeneSet:	MTND1P23	DNAJC11 CLSTN1	DFFA	DDI2	PAQR7	ARID1A
KPNA7	ZMYM4-AS1	THRAP3	RPS8	JAK1	WLS	RPL5	RP11	RPRD2
RPS27	UBAP2L	FCGR2C	TKT	POU2F1	RPS7	KIDINS220	CALM2	UBC	AAK1
GMCL1	TET3	EIF5B	UGGT1	RHOQP2	MYO1C	MLK7-AS1	TRIP12	GIGYF2	BRK1
LAMB2	RAD54L2 RPL29	APPL1	KCTD6	CTD	RPL24	ZBTB20	ACTG1P1 PDCD10
DNAJC19 PSMD2	BOD1L	PDGFRA	RPL34	RPS3A	SREK1IP1	SERF1B	FNIP1	SKP1
UBE2B	CTB	MTND5P11	CSF1R	RPS14	DOCK2	TBC1D9B RPL15P3 MTRNR2L9

WDR11 RP1 TBPL1 PHACTR2 SCAF8 IGF2R TRA2A RPS3AP26 ARPC1A MTPN
CLN8 KIF13B HOOK3 ASPH ZFHX4 FABP5 EEF1D VPS28 RPL8 RPS6
RNU2-5P NIPSNAP3A FKBP15 RPL12 GLE1 RPL7A MRC1 PRINS ZNF37BP
RPS24 FAM53B VENTX RPL27A MTRNR2L8 CKAP5 C11orf31 FABP5P7 DDB1
SLCO2B1 TMEM126B BACE1 UBE4A DDX6 APPL2 CD163 GUCY2C PFDN5 CBX5
LRP1 OS9 GNS RBMS1P1 EEA1 PLXNC1 RPL18AP3 PARP4-AS1
PHF11 RPL13AP25 HNRNPA3P5 MYCBP2-AS1 DNAJC3 RNASE6 CHD8
C14orf28 C14orf43 SIVA1 C14orf80 GOLGA8IP HERC2P10
FMN1 USP8 PIAS1 MEF2A LRRK1 MPG NME3 ABCC1 RPS15A SRCAP
ZNF629 NFATC3 VPS4A TERF2 ATXN1L GLG1 VPS53 METTL16 RPL26 SSH2
TAF15 CDK12 DHX8 NMT1 MED13 TNRC6C TBC1D16 ROCK1P1 ANKRD12 RPL17
RPL36 NDUFA11 MYO1F ZNF121 BRD4 CYP4F12 SUPT5H POU2F2 CNFN RPL18
RPL13A RPS5 SIRPG TSHZ2 RPL12P4 TRPM2 CTA TTC28 APOBEC3C RPL3
TNRC6B EP300 TMSB4X DYNLT3 WDR45 TTC3P1 PGAM4 XIAP MT

GOBPID	Pvalue	OddsRatio	ExpCount	Count	Size	Term
GO:0006415	2.00188708910009e-27	39.5168344324887	0.963462736142828	24	91	translational termination
GO:0006414	2.61881020490939e-27	34.7119140625	1.11168777247249	25	105	translational elongation
GO:0070972	6.27573902089993e-26	29.8324092741935	1.24932530620718	25	118	protein localization to endoplasmic reticulum
GO:0006614	8.92408816331945e-26	32.6546080964686	1.11168777247249	24	105	SRP-dependent cotranslational protein targeting to membrane
GO:0006613	1.14365709279469e-25	32.2541123085649	1.12227527506747	24	106	cotranslational protein targeting to membrane
GO:0045047	1.14365709279469e-25	32.2541123085649	1.12227527506747	24	106	protein targeting to ER
GO:0072599	1.14365709279469e-25	32.2541123085649	1.12227527506747	24	106	establishment of protein localization to endoplasmic reticulum
GO:0019080	1.85168014270543e-24	23.2124724409449	1.5987128918414	26	151	viral genome expression
GO:0019083	1.85168014270543e-24	23.2124724409449	1.5987128918414	26	151	viral transcription
GO:0000184	2.28689541570279e-24	27.8149326805386	1.25991280880216	24	119	nuclear-transcribed mRNA catabolic process, nonsense-mediated decay
GO:0006413	5.03204695328668e-23	21.7934916338583	1.60930039443637	25	152	translational initiation
GO:0000956	7.67853154865473e-22	19.1975911458333	1.78928793855097	25	169	nuclear-transcribed mRNA catabolic process
GO:0006612	1.27275227658944e-21	20.434829637642	1.61988789703135	24	153	protein targeting to membrane
GO:0043624	1.27275227658944e-21	20.434829637642	1.61988789703135	24	153	cellular protein complex disassembly
GO:0034623	1.61290482923462e-21	18.5468225671141	1.84222545152585	25	174	cellular macromolecular complex disassembly
GO:0043241	2.80985180226919e-21	19.6653939604304	1.67282541000623	24	158	protein complex disassembly
GO:0006402	2.87041207150359e-21	18.0568321078431	1.88457546190575	25	178	mRNA catabolic process
GO:0032984	3.30768958865989e-21	17.9383116883117	1.89516296450073	25	179	macromolecular complex disassembly
GO:0006401	6.19080285127836e-21	16.1480666871948	2.17043803197011	26	205	RNA catabolic process
GO:0019058	9.13479201410615e-21	14.8046218487395	2.44571309943949	27	231	viral infectious cycle
GO:0072594	8.76506838445195e-20	15.4056826117318	2.15985052937513	25	204	establishment of protein localization to organelle
GO:0022415	2.08452272859195e-19	12.9353157572042	2.75275067469379	27	260	viral reproductive process
GO:0071845	2.0795496701577e-18	11.707337409672	3.00685073697322	27	284	cellular component disassembly at cellular level
GO:0022411	2.98547399506604e-18	11.5246305418719	3.04920074735312	27	288	cellular component disassembly

GO:0016032	5.64076217151945e-17	8.39613583138173	4.77496367033423
31	451	viral reproduction
GO:0033365	2.15625534068943e-16	7.96558118833914	5.00788872742371
31	473	protein localization to organelle
GO:0006605	1.37190566205482e-14	7.28048205871693	5.01847623001868
29	474	protein targeting
GO:0042274	9.55270984107701e-07	37.1231808731809	0.19057504670957
5	18	ribosomal small subunit biogenesis
Tissue: Whole_Blood=>Adipose_Subcutaneous	Type:	cluster
SourceGene:	ENSG00000252229.1
TargetGeneSet:	MTND1P23	DNAJC11	CLSTN1	DFFA	DDI2	PAQR7	ARID1A
KPNA7	ZMYM4-AS1	THRAP3	RPS8	JAK1	WLS	RPL5	RP11	RPRD2
RPS27	UBAP2L	FCGR2C	TKT	POU2F1	RPS7	KIDINS220	CALM2	UBC	AAK1
GMCL1	TET3	EIF5B	UGGT1	RHOQP2	MYO1C	MLK7-AS1	TRIP12	GIGYF2	BRK1
LAMB2	RAD54L2	RPL29	APPL1	KCTD6	CTD	RPL24	ZBTB20	ACTG1P1	PDCD10
DNAJC19	PSMD2	BOD1L	PDGFRA	RPL34	RPS3A	SREK1IP1	SERF1B	FNIP1	SKP1
UBE2B	CTB	MTND5P11	CSF1R	RPS14	DOCK2	TBC1D9B	RPL15P3	MTRNR2L9
WDR11	RP1	TBPL1	PHACTR2	SCAF8	IGF2R	TRA2A	RPS3AP26	ARPC1A	MTPN
CLN8	KIF13B	HOOK3	ASPH	ZFHX4	FABP5	EEF1D	VPS28	RPL8	RPS6
RNU2-5P	NIPSNAP3A	FKBP15	RPL12	GLE1	RPL7A	MRC1	PRINS	ZNF37BP
RPS24	FAM53B	VENTX	RPL27A	MTRNR2L8	CKAP5	C11orf31	FABP5P7	DDB1
SLCO2B1	TMEM126B	BACE1	UBE4A	DDX6	APPL2	CD163	GUCY2C	PFDN5	CBX5
LRP1	OS9	GNS	RBMS1P1	EEA1	PLXNC1	RPL18AP3	PARP4-AS1
PHF11	RPL13AP25	HNRNPA3P5	MYCBP2-AS1	DNAJC3	RNASE6	CHD8
C14orf28	C14orf43	SIVA1	C14orf80	GOLGA8IP	HERC2P10
FMN1	USP8	PIAS1	MEF2A	LRRK1	MPG	NME3	ABCC1	RPS15A	SRCAP
ZNF629	NFATC3	VPS4A	TERF2	ATXN1L	GLG1	VPS53	METTL16	RPL26	SSH2
TAF15	CDK12	DHX8	NMT1	MED13	TNRC6C	TBC1D16	ROCK1P1	ANKRD12	RPL17
RPL36	NDUFA11	MYO1F	ZNF121	BRD4	CYP4F12	SUPT5H	POU2F2	CNFN	RPL18
RPL13A	RPS5	SIRPG	TSHZ2	RPL12P4	TRPM2	CTA	TTC28	APOBEC3C	RPL3
TNRC6B	EP300	TMSB4X	DYNLT3	WDR45	TTC3P1	PGAM4	XIAP	MT
GOBPID	Pvalue	OddsRatio	ExpCount	Count	Size	Term
GO:0006415	2.00188708910009e-27	39.5168344324887	0.963462736142828
24	91	translational termination
GO:0006414	2.61881020490939e-27	34.7119140625	1.11168777247249	25
105	translational elongation
GO:0070972	6.27573902089993e-26	29.8324092741935	1.24932530620718
25	118	protein localization to endoplasmic reticulum
GO:0006614	8.92408816331945e-26	32.6546080964686	1.11168777247249
24	105	SRP-dependent cotranslational protein targeting to membrane
GO:0006613	1.14365709279469e-25	32.2541123085649	1.12227527506747
24	106	cotranslational protein targeting to membrane
GO:0045047	1.14365709279469e-25	32.2541123085649	1.12227527506747
24	106	protein targeting to ER
GO:0072599	1.14365709279469e-25	32.2541123085649	1.12227527506747
24	106	establishment of protein localization to endoplasmic reticulum
GO:0019080	1.85168014270543e-24	23.2124724409449	1.5987128918414	26
151	viral genome expression
GO:0019083	1.85168014270543e-24	23.2124724409449	1.5987128918414	26
151	viral transcription
GO:0000184	2.28689541570279e-24	27.8149326805386	1.25991280880216
24	119	nuclear-transcribed mRNA catabolic process, nonsense-mediated decay
GO:0006413	5.03204695328668e-23	21.7934916338583	1.60930039443637
25	152	translational initiation
GO:0000956	7.67853154865473e-22	19.1975911458333	1.78928793855097
25	169	nuclear-transcribed mRNA catabolic process
GO:0006612	1.27275227658944e-21	20.434829637642	1.61988789703135	24
153	protein targeting to membrane
GO:0043624	1.27275227658944e-21	20.434829637642	1.61988789703135	24
153	cellular protein complex disassembly
GO:0034623	1.61290482923462e-21	18.5468225671141	1.84222545152585
25	174	cellular macromolecular complex disassembly
GO:0043241	2.80985180226919e-21	19.6653939604304	1.67282541000623

24	158	protein complex disassembly
GO:0006402	2.87041207150359e-21	18.0568321078431	1.88457546190575
25	178	mRNA catabolic process
GO:0032984	3.30768958865989e-21	17.9383116883117	1.89516296450073
25	179	macromolecular complex disassembly
GO:0006401	6.19080285127836e-21	16.1480666871948	2.17043803197011
26	205	RNA catabolic process
GO:0019058	9.13479201410615e-21	14.8046218487395	2.44571309943949
27	231	viral infectious cycle
GO:0072594	8.76506838445195e-20	15.4056826117318	2.15985052937513
25	204	establishment of protein localization to organelle
GO:0022415	2.08452272859195e-19	12.9353157572042	2.75275067469379
27	260	viral reproductive process
GO:0071845	2.0795496701577e-18	11.707337409672 3.00685073697322	27
284	cellular component disassembly at cellular level
GO:0022411	2.98547399506604e-18	11.5246305418719	3.04920074735312
27	288	cellular component disassembly
GO:0016032	5.64076217151945e-17	8.39613583138173	4.77496367033423
31	451	viral reproduction
GO:0033365	2.15625534068943e-16	7.96558118833914	5.00788872742371
31	473	protein localization to organelle
GO:0006605	1.37190566205482e-14	7.28048205871693	5.01847623001868
29	474	protein targeting
GO:0042274	9.55270984107701e-07	37.1231808731809	0.19057504670957
5	18	ribosomal small subunit biogenesis
Tissue: Whole_Blood=>Adipose_Subcutaneous	Type:	asymmetric
SourceGene:	KIR3DL2(ENSG00000240403.1)
TargetGeneSet:	RPS8	RP11	RPL32	RPL29	RPL35A	C4orf52 PAIP2	CTB
RPS14	GUSBP2	SNORA20 RPL7	EIF3H	EEF1D	RPS6	CLTA	BLOC1S2 CKAP5
ARPC3	TPT1	CGRRF1	RPS3AP6 RPS27L	RPLP1	RPS17L	C16orf13		RPL13
SUPT6H	RPL23A	C18orf32		SCARNA18		RPS15	RPL18	DHDH	GYG2-AS1
RPS4X	RPL10
GOBPID	Pvalue	OddsRatio	ExpCount	Count	Size	Term
GO:0006414	2.44776479587428e-27	184.038523274478	0.21797799460245
16	105	translational elongation
GO:0006413	1.0044230277057e-26	138.382905982906	0.315549096948308
17	152	translational initiation
GO:0006415	4.15583656027707e-26	188.75	0.18891426198879	15	91
translational termination
GO:0006614	4.16102202144838e-25	159.233333333333	0.21797799460245
15	105	SRP-dependent cotranslational protein targeting to membrane
GO:0006613	4.84215706330037e-25	157.472527472527	0.220053975503425
15	106	cotranslational protein targeting to membrane
GO:0045047	4.84215706330037e-25	157.472527472527	0.220053975503425
15	106	protein targeting to ER
GO:0072599	4.84215706330037e-25	157.472527472527	0.220053975503425
15	106	establishment of protein localization to endoplasmic reticulum
GO:0070972	2.67004705690014e-24	139.009708737864	0.244965746315134
15	118	protein localization to endoplasmic reticulum
GO:0000184	3.05215497429842e-24	137.663461538462	0.24704172721611
15	119	nuclear-transcribed mRNA catabolic process, nonsense-mediated decay
GO:0019080	1.28839226808516e-22	105.036764705882	0.313473116047332
15	151	viral genome expression
GO:0019083	1.28839226808516e-22	105.036764705882	0.313473116047332
15	151	viral transcription
GO:0006612	1.58172070029641e-22	103.5	0.317625077849284	15	153
protein targeting to membrane
GO:0043624	1.58172070029641e-22	103.5	0.317625077849284	15	153
cellular protein complex disassembly
GO:0043241	2.60941149443229e-22	99.8461538461538	0.328004982354162
15	158	protein complex disassembly
GO:0000956	7.41763287803991e-22	92.6428571428571	0.350840772264895
15	169	nuclear-transcribed mRNA catabolic process

GO:0034623	1.16489622571921e-21	89.6981132075472	0.361220676769774
15	174	cellular macromolecular complex disassembly
GO:0006402	1.65528529282346e-21	87.4723926380368	0.369524600373677
15	178	mRNA catabolic process
GO:0032984	1.80490563505889e-21	86.9329268292683	0.371600581274652
15	179	macromolecular complex disassembly
GO:0072594	1.34993586502559e-20	75.3015873015873	0.423500103799045
15	204	establishment of protein localization to organelle
GO:0006401	1.45507429475967e-20	74.9	0.425576084700021	15	205
RNA catabolic process
GO:0019058	9.03282815920188e-20	65.7638888888889	0.479551588125389
15	231	viral infectious cycle
GO:0022415	5.45410010289999e-19	57.8612244897959	0.539755034253685
15	260	viral reproductive process
GO:0071845	2.07466875589035e-18	52.6096654275093	0.589578575877102
15	284	cellular component disassembly at cellular level
GO:0022411	2.56240539447568e-18	51.8241758241758	0.597882499481005
15	288	cellular component disassembly
GO:0016032	2.08323179173028e-15	32.0756880733945	0.936267386340046
15	451	viral reproduction
GO:0033365	4.20956060763729e-15	30.4868995633188	0.981938966161511
15	473	protein localization to organelle
GO:0006605	4.34277122235995e-15	30.4183006535948	0.984014947062487
15	474	protein targeting
GO:0042274	2.28326335864882e-10	221.661538461538	0.0373676562175628
5	18	ribosomal small subunit biogenesis
GO:0006364	1.44839609929631e-09	44.9430748543254	0.215902013701474
7	104	rRNA processing
GO:0016072	2.15150248907704e-09	42.3073026593499	0.228357899107328
7	110	rRNA metabolic process
GO:0022613	1.32899350087514e-08	23.8022622538752	0.467095702719535
8	225	ribonucleoprotein complex biogenesis
GO:0042254	1.79491152814987e-08	30.6041028781384	0.309321154245381
7	149	ribosome biogenesis
GO:0071843	2.12892620731648e-08	22.3376623376623	0.496159435333195
8	239	cellular component biogenesis at cellular level
GO:0034470	1.36107562520756e-07	22.4365848164001	0.415196180195142
7	200	ncRNA processing
GO:0000028	1.60796442683759e-07	534	0.0124558854058543	3	6
ribosomal small subunit assembly
GO:0034660	1.48766010544459e-06	15.4834219580857	0.591654556778078
7	285	ncRNA metabolic process
GO:0042255	4.43961160337844e-06	123.145299145299	0.0332156944156114
3	16	ribosome assembly
Tissue: Whole_Blood=>Adipose_Subcutaneous	Type:	asymmetric

SourceGene: MRPL53P1(ENSG00000235299.1) MRPL53P1 mitochondrial ribosomal protein L53 pseudogene 1

TargetGeneSet: C1orf86 RPL22 ICMT RP11 LDLRAD2 HSPG2 RPL11 ARID1A KPNA7 RP1 KDM4A RPS8 RP4 PTGES3P1 RPL5 JTB RPS27 UBAP2L MTX1 MIR555 MEF2D RPS7 PPP1CB CALM2 UBC AAK1 GMCL1 DGUOK-AS1 EIF5B RANBP2 UGGT1 MYO1C SENP2 EEF1B2 TRIP12 GIGYF2 TRAF3IP1 BRK1 UBE2E1 GORASP1 DALRD3 RAD54L2 RPL29 RPL24 ABTB1 RYK ACTG1P1 PDCD10 DNAJC19 PSMD2 7SK PPP1R2 NOP14 MAN2B2 RPL34 NDUFC1 RPS3A FBXO8 NPR3 CTD NDUFAF2 CHCHD2P2 COX7C HINT1 UBE2B CTB MTND5P11 PAIP2 RPS14 RPL15P3 NOL7 DEK GPLD1 ABT1 RPS10 MTRNR2L9 WDR11 MRPS12 MALSU1 MPLKIP CHCHD2 RPS3AP26 CUX1 SLC26A4 RPL7 MED30 EEF1D RPL8 RPS6 NDUFB6 CLTA NIPSNAP3A RPL35 ZNF79 RPL12 NUP188 RPL7A NACC2 PRINS RPS24 RPS3AP5 MRPL43 SUFU FAM53B NUP98 PRKCDBP RPL27A MTRNR2L8 PIGCP1 CKAP5 C11orf31 DDB1 BAD C11orf2 RNASEH2C CCDC85B TMEM126B TMEM126A SDHD BACE1 UBE4A DDX6 RPS25 USP5 PFDN5 HOXC8 CBX5 OS9 GNS EEA1 RPL18AP3 CUX2 ACADS PARP4-AS1 TPT1 MYCBP2-AS1 DNAJC3 HMGB3P4 CHD8 C14orf21 C14orf28 CGRRF1 YLPM1 CCNK SIVA1 GOLGA8IP MGA SNAP23 DUT RPS3AP6 RPS27L PIAS1 RPLP1 MORF4L1 SEC11A MRPL46 MPG MRPL28 FAM173A SPSB3 MPV17L ABCC1 RPS15A SRCAP NFATC3 HSBP1 RPL13 VPS53 METTL16 RNASEK POLR2A TP53 LSMD1 VAMP2 RPL26 TRIM16 NCOR1 SUPT6H RPL23A NUFIP2 RPL19 WIPF2 RPL27 DHX8 MED13 BPTF NT5C TNRC6C C18orf32 RPL17 ATP5D RPS15 UQCR11 FAM108A1 NFIC RPL36 NDUFA11 TRAPPC5 SLC35E1 PGLS C19orf60 RPSAP58 HSPB6 RPS19 POU2F2 ZNF428 SLC1A5 RPL18 RPL13A ZNF587 ZSCAN22 RPS5 GAPDHP2 HM13 KIF3B SCAND1 SOGA1 TOP1 NCOA3 TSHZ2 DGCR2 DGCR6L FAM211B CTA HMGB1P10 NF2 PRR14L MPST APOBEC3C RPL3 EP300 DYNLT3 WASF4P WDR45 APOOL RPL39 BCORL1 MT

GOBPID	Pvalue	OddsRatio	ExpCount	Count	Size	Term

GO:0006414	3.73E-49	59.03534304	1.365995433	40	105
translational elongation					
GO:0006415	1.46E-48	67.92201258	1.183862708	38	91
translational termination					

GO:0006614	2.16E-47	56.30292862	1.365995433	39	105

SRP-dependent cotranslational protein targeting to membrane

GO:0006613	3.38E-47	55.45867976	1.379004913	39	106

cotranslational protein targeting to membrane

GO:0045047	3.38E-47	55.45867976	1.379004913	39	106

protein targeting to ER

GO:0072599	3.38E-47	55.45867976	1.379004913	39	106

establishment of protein localization to endoplasmic reticulum

GO:0070972	1.03E-46	49.15107415	1.535118677	40	118

protein localization to endoplasmic reticulum

GO:0000184	3.34E-43	44.35522634	1.548128157	38	119

"nuclear-transcribed mRNA catabolic process, nonsense-mediated decay"

GO:0019080	8.74E-42	34.45824203	1.964431527	40	151

viral genome expression

GO:0019083	8.74E-42	34.45824203	1.964431527	40	151

viral transcription

GO:0006413	1.17E-41	34.14816602	1.977441008	40	152

translational initiation

GO:0006612	1.57E-41	33.84357809	1.990450488	40	153

protein targeting to membrane

GO:0043624	1.68E-38	31.1666087	1.990450488	38	153

cellular protein complex disassembly

GO:0000956	3.73E-38	28.4557047	2.198602173	39	169

nuclear-transcribed mRNA catabolic process

GO:0043241	6.45E-38	29.85744444	2.055497889	38	158

protein complex disassembly

GO:0034623	1.28E-37	27.39209545	2.263649574	39	174

cellular macromolecular complex disassembly

GO:0072594	1.76E-37	24.1267059	2.653933984	41	204	establishment of protein localization to organelle
GO:0006402	3.34E-37	26.59630148	2.315687496	39	178	mRNA catabolic process

GO:0032984	4.23E-37	26.40445829	2.328696976	39	179	macromolecular complex disassembly
GO:0006401	5.17E-36	23.09254709	2.666943464	40	205	RNA catabolic process
GO:0019058	3.75E-35	20.65853921	3.005189952	41	231	viral infectious cycle

GO:0022415	3.04E-34	18.53368104	3.382464881	42	260

viral reproductive process

GO:0033365	1.09E-30	11.50588774	6.153484188	49	473

protein localization to organelle

GO:0006605	1.21E-30	11.47798561	6.166493668	49	474
protein targeting					
GO:0016032	1.39E-30	11.79156328	5.867275621	48	451
viral reproduction					
GO:0071845	5.39E-29	14.97608547	3.694692409	39	284
cellular component disassembly at cellular level					

GO:0022411	9.29E-29	14.73130104	3.74673033	39	288

cellular component disassembly

GO:0042274	3.87E-13	79.63128492	0.234170646	9	18

ribosomal small subunit biogenesis

GO:0042254	9.43E-10	9.142222414	1.938412567	15	149

ribosome biogenesis

GO:0022613	5.89E-09	6.717695682	2.92713307	17	225	ribonucleoprotein complex biogenesis
GO:0006364	1.01E-08	10.50222332	1.352985953	12	104	rRNA processing

GO:0071843 1.46E-08 6.287787788 3.109265795 17 239
cellular component biogenesis at cellular level

GO:0016072 1.92E-08 9.855055659 1.431042834 12 110 rRNA
metabolic process

GO:0042273 2.60E-07 55.6440281 0.156113764 5 12
ribosomal large subunit biogenesis

GO:0030490	9.42E-07	103.3333333	0.091066362	4	7

maturation of SSU-rRNA

GO:0034470	1.20E-05	5.104569632	2.601896063	12	200

ncRNA processing

Tissue: Whole_Blood=>Adipose_Subcutaneous Type: cluster
SourceGene: MRPL53P1(ENSG00000235299.1)

TargetGeneSet: C1orf86 RPL22 ICMT RP11 LDLRAD2 HSPG2 RPL11 ARID1A
KPNA7 RP1 KDM4A RPS8 RP4 PTGES3P1 RPL5 JTB RPS27
UBAP2L MTX1 MIR555 MEF2D RPS7 PPP1CB CALM2 UBC AAK1 GMCL1
DGUOK-AS1 EIF5B RANBP2 UGGT1 MYO1C SENP2 EEF1B2 TRIP12 GIGYF2
TRAF3IP1 BRK1 UBE2E1 GORASP1 DALRD3 RAD54L2 RPL29 RPL24 ABTB1 RYK
ACTG1P1 PDCD10 DNAJC19 PSMD2 7SK PPP1R2 NOP14 MAN2B2 RPL34 NDUFC1
RPS3A FBXO8 NPR3 CTD NDUFAF2 CHCHD2P2 COX7C HINT1 UBE2B CTB
MTND5P11 PAIP2 RPS14 RPL15P3 NOL7 DEK GPLD1 ABT1 RPS10
MTRNR2L9 WDR11 MRPS12 MALSU1 MPLKIP CHCHD2 RPS3AP26 CUX1
SLC26A4 RPL7 MED30 EEF1D RPL8 RPS6 NDUFB6 CLTA NIPSNAP3A
RPL35 ZNF79 RPL12 NUP188 RPL7A NACC2 PRINS RPS24 RPS3AP5 MRPL43 SUFU
FAM53B NUP98 PRKCDBP RPL27A MTRNR2L8 PIGCP1 CKAP5 C11orf31 DDB1
BAD C11orf2 RNASEH2C CCDC85B TMEM126B TMEM126A SDHD
BACE1 UBE4A DDX6 RPS25 USP5 PFDN5 HOXC8 CBX5 OS9 GNS EEA1
RPL18AP3 CUX2 ACADS PARP4-AS1 TPT1 MYCBP2-AS1 DNAJC3
HMGB3P4 CHD8 C14orf21 C14orf28 CGRRF1 YLPM1 CCNK SIVA1
GOLGA8IP MGA SNAP23 DUT RPS3AP6 RPS27L PIAS1 RPLP1 MORF4L1
SEC11A MRPL46 MPG MRPL28 FAM173A SPSB3 MPV17L ABCC1 RPS15A SRCAP
NFATC3 HSBP1 RPL13 VPS53 METTL16 RNASEK POLR2A TP53 LSMD1 VAMP2
RPL26 TRIM16 NCOR1 SUPT6H RPL23A NUFIP2 RPL19 WIPF2 RPL27 DHX8
MED13 BPTF NT5C TNRC6C C18orf32 RPL17 ATP5D RPS15 UQCR11
FAM108A1 NFIC RPL36 NDUFA11 TRAPPC5 SLC35E1 PGLS C19orf60
RPSAP58 HSPB6 RPS19 POU2F2 ZNF428 SLC1A5 RPL18 RPL13A ZNF587 ZSCAN22 RPS5
GAPDHP2 HM13 KIF3B SCAND1 SOGA1 TOP1 NCOA3 TSHZ2 DGCR2 DGCR6L
FAM211B CTA HMGB1P10 NF2 PRR14L MPST APOBEC3C RPL3
EP300 DYNLT3 WASF4P WDR45 APOOL RPL39 BCORL1 MT

GOBPID	Pvalue	OddsRatio	ExpCount	Count	Size	Term
GO:0006414	3.73063837383387e-49	59.035343035343	1.36599543284202	40	105	translational elongation
GO:0006415	1.46340793209755e-48	67.9220125786163	1.18386270846308	38	91	translational termination
GO:0006614	2.15619985871634e-47	56.3029286150092	1.36599543284202	39	105	SRP-dependent cotranslational protein targeting to membrane
GO:0006613	3.37677545824514e-47	55.4586797555845	1.3790049131548	39	106	cotranslational protein targeting to membrane
GO:0045047	3.37677545824514e-47	55.4586797555845	1.3790049131548	39	106	protein targeting to ER
GO:0072599	3.37677545824514e-47	55.4586797555845	1.3790049131548	39	106	establishment of protein localization to endoplasmic reticulum
GO:0070972	1.03199852307688e-46	49.1510741510741	1.53511867690817	40	118	protein localization to endoplasmic reticulum
GO:0000184	3.34093162568094e-43	44.3552263374486	1.54812815722095	38	119	nuclear-transcribed mRNA catabolic process, nonsense-mediated decay
GO:0019080	8.7395666550836e-42	34.4582420258096	1.96443152722995	40	151	viral genome expression
GO:0019083	8.7395666550836e-42	34.4582420258096	1.96443152722995	40	151	viral transcription
GO:0006413	1.17411846562303e-41	34.148166023166	1.97744100754273	40	152	translational initiation
GO:0006612	1.57369689042792e-41	33.8435780913657	1.99045048785551	40	153	protein targeting to membrane
GO:0043624	1.68164805971791e-38	31.1666086956522	1.99045048785551	38	153	cellular protein complex disassembly
GO:0000956	3.73184480804594e-38	28.4557046979866	2.19860217286001	39	169	nuclear-transcribed mRNA catabolic process
GO:0043241	6.45243751909214e-38	29.8574444444444	2.05549788941942	38	158	protein complex disassembly
GO:0034623	1.282854874419e-37	27.3920954511559	2.26364957442392	39	174	cellular macromolecular complex disassembly
GO:0072594	1.76295983175462e-37	24.1267058970828	2.65393398380735	41	204	establishment of protein localization to organelle
GO:0006402	3.34364130480759e-37	26.5963014823041	2.31568749567504	39	178	mRNA catabolic process

GO:0032984 4.23162209693341e-37 26.4044582933845 2.32869697598782
39 179 macromolecular complex disassembly
GO:0006401 5.1722689110407e-36 23.0925470925471 2.66694346412013
40 205 RNA catabolic process
GO:0019058 3.75419660603351e-35 20.6585392051557 3.00518995225244
41 231 viral infectious cycle
GO:0022415 3.03706919772999e-34 18.5336810355662 3.38246488132309
42 260 viral reproductive process
GO:0033365 1.09468083270523e-30 11.5058877426361 6.15348418794547
49 473 protein localization to organelle
GO:0006605 1.20902238516489e-30 11.4779856115108 6.16649366825825
49 474 protein targeting
GO:0016032 1.39328519956244e-30 11.7915632754342 5.86727562106429
48 451 viral reproduction
GO:0071845 5.39156390596749e-29 14.9760854677441 3.69469240882984
39 284 cellular component disassembly at cellular level
GO:0022411 9.29068973029194e-29 14.7313010430986 3.74673033008096
39 288 cellular component disassembly
GO:0042274 3.8721725988671e-13 79.6312849162011 0.23417064563006
9 18 ribosomal small subunit biogenesis
GO:0042254 9.43006407331837e-10 9.14222241394185 1.93841256660439
15 149 ribosome biogenesis
GO:0022613 5.89204997575501e-09 6.71769568151147 2.92713307037575
17 225 ribonucleoprotein complex biogenesis
GO:0006364 1.00730880064885e-08 10.5022233201581 1.35298595252924
12 104 rRNA processing
GO:0071843 1.45642476614937e-08 6.28778778778779 3.10926579475469
17 239 cellular component biogenesis at cellular level
GO:0016072 1.91523072560317e-08 9.85505565862709 1.43104283440592
12 110 rRNA metabolic process
GO:0042273 2.5989566538941e-07 55.6440281030445 0.156113763753373
5 12 ribosomal large subunit biogenesis
GO:0030490 9.41895577688388e-07 103.333333333333 0.0910663621894679
4 7 maturation of SSU-rRNA
GO:0034470 1.2033717020449e-05 5.10456963249516 2.60189606255622
12 200 ncRNA processing
Tissue: Whole_Blood=>Adipose_Subcutaneous Type: cluster
SourceGene: MTND1P23(ENSG00000225972.1)
TargetGeneSet: MTND1P23 RPL22 DDI2 RP1 RPL11 ARID1A PRDX3P2
KPNA7 ZMYM4 THRAP3 RPS8 RP11 RPL5 RPRD2 RPS27 UBAP2L RAB25 TKT
RPS7 CALM2 UBC COMMD1 AAK1 TMEM131 EIF5B UGGT1 UBR3 TRIP12
EFHD1 GIGYF2 SNED1 BRK1 RPL15 RAD54L2 RPL29 KCTD6 CTD TMF1 PCNP
RPL24 RYK GPR87 PDCD10 HTT BOD1L ZBTB12B PDGFRA CISD2 RPL34
RPS3A RHOBTB3 SKP1 CTB MTND5P11 CSF1R RPS14 LYRM4 RP3
RPL15P3 AGPAT1 MTRNR2L9 WDR11 PHACTR2 PPP1R14C IGF2R STARD3NL
RPS3AP26 POLR2J3 SPDYE2 TRBV6-5 CLN8 KIAA1967 DRP2 HMBOX1
HOOK3 ASPH ARFGEF1 UBR5 NFIB RPS6 HMGB3P24 CBWD6 NIPSNAP3A
ATP6V1G1 GAPVD1 RPL12 SETX EEF1A1P5 RPL7A SEC16A KIAA0913
RPS3AP5 KLLN MRPL43 DUSP5 RPL27A MTRNR2L8 CKAP5 BTBD18 CCDC90B ATM
FDX1 UBE4A MLL DDX6 RPL23AP64 CD163L1 SCAF11 MLL4 PFDN5 CBX5
LRP1 OS9 GNS EEA1 USP44 RPL18AP3 RPSAP54 GTF3A TNFSF13B
CCNB1IP1 CHD8 C14orf28 CGRRF1 YLPM1 C14orf142 SIVA1
C15orf33 USP8 RPS27L UBL7 SIN3A SEC11A MPG RPS15A
SLX1B-SULT1A4 LONP2 FBXL8 NFATC3 TERF2 RPL13 VPS53 WDR81 RPL26
RPL23A NUFIP2 TAF15 RPL19 CDK12 RPL27 MED13 TNRC6C ROCK1P1 ANKRD12 DSG1
RPL17 FAM108A1 NFIC C3 ZNF121 BRD4 RPSAP58 POU2F2 ZNF526
IGFL2 RPL18 NUCB1-AS1 RPL13A RPS5 RRBP1 NCOA3 TSHZ2 RPL12P4
ICOSLG PI4KAP1 YDJC FAM211B CTA HMGB1P10 TTC28 EIF3L RPL3
TNRC6B EP300 TMSB4X DYNLT3 USP9X RPS4X RLIM TTC3P1 PGAM4 APOOL
DOCK11 RP13 RPL10 MT
GOBPID Pvalue OddsRatio ExpCount Count Size Term
GO:0006415 4.43951750615861e-38 59.8696931483817 0.92567988374507
30 91 translational termination

GO:0006614	1.29222701416958e-37	51.3897949673812	1.068092173552	31	105	SRP-dependent cotranslational protein targeting to membrane
GO:0070972	1.75738623577027e-37	46.0036400404449	1.20033215694416	32	118	protein localization to endoplasmic reticulum
GO:0006613	1.81205197266399e-37	50.7010344827586	1.07826447996678	31	106	cotranslational protein targeting to membrane
GO:0045047	1.81205197266399e-37	50.7010344827586	1.07826447996678	31	106	protein targeting to ER
GO:0072599	1.81205197266399e-37	50.7010344827586	1.07826447996678	31	106	establishment of protein localization to endoplasmic reticulum
GO:0006414	6.50307642326397e-36	48.6461538461538	1.068092173552	30	105	translational elongation
GO:0000184	4.50928103369268e-34	40.9536156727168	1.21050446335894	30	119	nuclear-transcribed mRNA catabolic process, nonsense-mediated decay
GO:0006413	1.36605743496636e-33	32.8904347826087	1.54619057504671	32	152	translational initiation
GO:0019080	3.54962424906352e-32	31.5879310344828	1.53601826863193	31	151	viral genome expression
GO:0019083	3.54962424906352e-32	31.5879310344828	1.53601826863193	31	151	viral transcription
GO:0000956	5.19550891365289e-32	28.7746112345287	1.71911978409799	32	169	nuclear-transcribed mRNA catabolic process
GO:0006612	5.50462448728236e-32	31.065715093273	1.55636288146149	31	153	protein targeting to membrane
GO:0006402	3.01707628337748e-31	26.9836807623585	1.81067054183102	32	178	mRNA catabolic process
GO:0043624	1.6843388982562e-30	29.5622263914947	1.55636288146149	30	153	cellular protein complex disassembly
GO:0034623	3.82730617094672e-30	26.4643718350615	1.76998131617189	31	174	cellular macromolecular complex disassembly
GO:0043241	4.70981669600893e-30	28.3974358974359	1.6072244135354	30	158	protein complex disassembly
GO:0032984	9.63588371523563e-30	25.5612767940354	1.8208428482458	31	179	macromolecular complex disassembly
GO:0072594	2.88086400958539e-29	22.8626895854398	2.07515050861532	32	204	establishment of protein localization to organelle
GO:0006401	3.38706482064813e-29	22.7289268660467	2.0853228150301	32	205	RNA catabolic process
GO:0019058	3.32502076082512e-26	18.8458620689655	2.34980278181441	31	231	viral infectious cycle
GO:0033365	5.44317706017976e-26	11.9755671148907	4.81150093419141	40	473	protein localization to organelle
GO:0071845	8.04758984305396e-26	16.2070664709583	2.8889350217978	33	284	cellular component disassembly at cellular level
GO:0022411	1.2725239793414e-25	15.9482972136223	2.92962424745692	33	288	cellular component disassembly
GO:0022415	1.3202462778126e-24	16.4254253877428	2.64479966784306	31	260	viral reproductive process
GO:0006605	8.78506809219884e-23	10.6735801955482	4.82167324060619	37	474	protein targeting
GO:0016032	1.83222706237215e-18	9.22112690671371	4.58771019306622	32	451	viral reproduction
GO:0044257	1.48355496018936e-05	4.08650458069063	4.08926717874196	15	402	cellular protein catabolic process
GO:0022613	1.88883383604748e-05	5.32538482682793	2.28876894332572	11	225	ribonucleoprotein complex biogenesis

Tissue: Whole_Blood=>Adipose_Subcutaneous Type: cluster

SourceGene: ROCK1P1(ENSG00000263006.1) "Rho-associated, coiled-coil containing protein kinase 1 pseudogene 1"

TargetGeneSet: MTND1P23 RPL22 DNAJC11 DFFA HSPG2 RPL11 PAFAH2 ARID1A PRDX3P2 KPNA7 THRAP3 RP1 RPS8 RPS15AP10 RPL5 RP11 RNU1-5 RPRD2 RPS27 UBAP2L ARHGEF11 TMEM63A UBC COMMD1 GMCL1 DGUOK RETSAT EIF5B UGGT1 MYO1C EEF1B2 RPL15 RAD54L2 RPL29 PCNP RPL24 BOD1L ZBTB12B RPL34 RPS3A MND1 SNORA47 HINT1 SKP1 CTB MTND5P11 PCDHGC5 RPS14 SLC2A3P1 RPL15P3 RPS10 RPL10A USP49 MTRNR2L9 WDR11 KIAA1919 TATDN2P2 IGF2R CYP51A1 RPS3AP26 MLL3 HOOK3 RBM12B EIF3H EEF1D RPL8 RPS6 NIPSNAP3A GAPVD1 RPL7A RPS24 RPS3AP5 SFR1 FAM53B RPL27A MTRNR2L8 CKAP5 TMEM126B BACE1 UBE4A DDX6 SCAF11 CCDC65 MLL4 PFDN5 PSG1 CBX5 LRP1 ARPC3 VPS29 RP3 SBNO1 TPT1 HMGB3P4 CHD8 ZNF410 KIAA1737 SIVA1 GOLGA8IP HERC2P10 CCNDBP1 MORF4L1 SEC11A CTD NTAN1 RPS15A NFATC3 RPL13 VPS53 RPL26 SUPT6H RPL23A RPL19 WIPF2 RAMP2 RPL27 DHX8 ROCK1P1 RPL17 RPS15 FAM108A1 NFIC ZBTB7A ZNF121 MAST1 BRD4 GLT25D1 SUPT5H MIR642A RPL18 RPL13A RPS5 SCAND1 EPB41L1 SOGA1 TSHZ2 SCAF4 DGCR6L CTA RPL3 EP300 RP4 TMSB4X DYNLT3 HUWE1 RPS4X APOOL XIAP HCFC1 RPL10 AMELY MT

GOBPID	Pvalue	OddsRatio	ExpCount	Count	Size	Term
GO:0006414	3.66E-48	83.88823529	0.871911978	35	105	translational elongation

GO:0006415	8.20E-47	93.343044	0.755657048	33	91

translational termination

GO:0006614	2.99E-46	79.40386505	0.871911978	34	105

SRP-dependent cotranslational protein targeting to membrane

GO:0006613	4.38E-46	78.29554264	0.880215902	34	106

cotranslational protein targeting to membrane

GO:0045047	4.38E-46	78.29554264	0.880215902	34	106

protein targeting to ER

GO:0072599	4.38E-46	78.29554264	0.880215902	34	106

establishment of protein localization to endoplasmic reticulum

GO:0070972	3.08E-44	67.05398671	0.979862985	34	118

protein localization to endoplasmic reticulum

GO:0000184	2.78E-42	62.82878909	0.988166909	33	119

"nuclear-transcribed mRNA catabolic process, nonsense-mediated decay"

GO:0019080	7.90E-42	50.45892495	1.253892464	35	151
viral genome expression					
GO:0019083	7.90E-42	50.45892495	1.253892464	35	151
viral transcription					
GO:0006413	1.02E-41	50.02413273	1.262196388	35	152
translational initiation					
GO:0006612	6.40E-40	47.21594684	1.270500311	34	153
protein targeting to membrane					

GO:0000956	2.57E-38	41.57312661	1.403363089	34	169

nuclear-transcribed mRNA catabolic process

GO:0043624	2.96E-38	44.91982759	1.270500311	33	153

cellular protein complex disassembly

GO:0034623	7.50E-38	40.07425249	1.444882707	34	174

cellular macromolecular complex disassembly

GO:0043241	9.44E-38	43.10786207	1.312019929	33	158

protein complex disassembly

GO:0006402	1.72E-37	38.9500969	1.478098401	34	178	mRNA catabolic process
GO:0032984	2.12E-37	38.678749	1.486402325	34	179	macromolecular complex disassembly
GO:0072594	2.41E-35	32.93255814	1.694000415	34	204	establishment of protein localization to organelle
GO:0006401	2.87E-35	32.7376581	1.702304339	34	205	RNA catabolic process

GO:0019058	6.89E-35	29.69537815	1.918206353	35	231
viral infectious cycle					
GO:0022415	1.88E-34	26.99043367	2.159020137	36	260
viral reproductive process					
GO:0071845	1.18E-31	23.28703047	2.358314304	35	284
cellular component disassembly at cellular level					
GO:0022411	1.95E-31	22.91234597	2.391529998	35	288
cellular component disassembly					

GO:0016032	3.36E-28	15.61696096	3.745069545	38	451
viral reproduction					
GO:0033365	1.96E-27	14.80370059	3.927755865	38	473
protein localization to organelle					
GO:0006605	3.09E-26	14.17325136	3.936059788	37	474
protein targeting					
GO:0042274	2.85E-07	47.88628763	0.149470625	5	18
ribosomal small subunit biogenesis					

GO:0022613	3.63E-07	7.364632238	1.868382811	12	225	ribonucleoprotein complex biogenesis
GO:0071843	6.93E-07	6.903573177	1.984637741	12	239	cellular component biogenesis at cellular level
GO:0006364	2.37E-06	10.59151786	0.863608055	8	104	rRNA processing
GO:0016072	3.61E-06	9.964285714	0.913431596	8	110	rRNA metabolic process

GO:0042254 4.09E-06 8.218725869 1.237284617 9 149
ribosome biogenesis

GO:0030490 1.91E-05 91.83974359 0.058127465 3 7
maturation of SSU-rRNA

Tissue: Whole_Blood=>Adipose_Subcutaneous Type: cluster
SourceGene: RP11-156P1.1(ENSG00000263142.1)
TargetGeneSet: MTND1P23 RPL22 CLSTN1 DFFA HSPG2 RPL11 ARID1A
WASF2 PHACTR4 TAF12 PUM1 KPNA7 THRAP3 MFI2 RPS8 RP4 CCBL2 RPL5
RP11 ATP5F1 RPRD2 ZNF687 SPRR1B RPS27 ASH1L MIR555 SSR2 TKT GAS5
ELK4 RPS7 PPP1CB UBC COMMD1 GMCL1 DGUOK COA5 EIF5B RANBP2
UGGT1 SENP2 EEF1B2 TRIP12 GIGYF2 SNED1 BRK1 RPL15 DALRD3 LAMB2
RAD54L2 RPL29 KCTD6 RPL24 PHC3 DNAJC19 EIF4G1 RPL35A ZFYVE28 AFAP1
BOD1L RPL34 NDUFC1 ANAPC10 RPS3A RPL6P12 NDUFS4 CTD NDUFAF2 MRPS36 BTF3
TBCA COX7C APC HINT1 SKP1 CTB MTND5P11 RPS14 SLC2A3P1
GNB2L1 RPL15P3 GFOD1 NOL7 PRRC2A AGPAT1 RPS18 TAPBP SYNGAP1 RPS10
RPL10A MRPL32 MTRNR2L9 COX7A2 WDR11 MRPS12 PHACTR2 IGF2R PSMB1
FOXK1 ADCY1 CHCHD2 RPS3AP26 TRRAP MRPS33 MLL3 KIAA1967 DRP2
KAT6A HOOK3 RPS20 PAG1 KIAA1429 RPL30 EIF3H EEF1D RPL8 NFIB
RPS6 TLN1 CLTA NIPSNAP3A TMEM245 RC3H2 RPL35 RPL12 LRRC8A SETX
GTF3C4 RPL7A SEC16A COMMD3 KIAA0913 RPS24 MRPL43 FAM53B RPL27A
MTRNR2L8 CKAP5 C11orf31 PPP6R3 CCDC90B C11orf73 UBE4A
ATP5L DDX6 RPS25 H2AFJ MLL4 PFDN5 CBX5 LRP1 EEA1 RPL18AP3
SBNO1 GOLGA3 TPT1 ESD MED4-AS1 DNAJC3 CCNB1IP1 CHD8
YLPM1 GOLGA8IP HERC2 USP8 RPS3AP6 RPS27L PIAS1 CALML4 SIN3A
C15orf40 SEC11A MRPL46 CIB1 MPG HBQ1 NME3 SPSB3 NDUFB10 ECI1
RPS15A SRCAP ZNF629 FBXL8 NFATC3 ATXN1L ZNF778 RPL13 VPS53 WDR81
POLR2A RPL26 KIAA0100 RPL23A TAF15 RPL17 RPL19 MED1 CDK12
WIPF2 RPL27 CLTC MED13 NT5C TNRC6C NPLOC4 ANAPC11 ROCK1P1 ANKRD12 DSG4
ZNF407 ATP5D RPS15 NFIC PTPRS RPL36 TRAPPC5 ZNF121 PIN1 C19orf43

BRD4 SLC35E1 UBA52 GATAD2A RPSAP58 POU2F2 ZNF428 IGFL2 ARHGAP35
RPL18 RPL13A ZNF264 RPS5 KIF3B SCAND1 NCOA3 TSHZ2 RPL12P4 SON
C22orf29 MIR1306 DGCR6L HMGB1P10 TTC28 UQCR10 RP3 MPST
EIF3L RPL3 TNRC6B EP300 ZC3H7B ZBED4 TMSB4X DYNLT3 UXT HUWE1
UBQLN2 NHSL2 RPS4X COX7B RPL39 MCTS1 XIAP HCFC1 RPL10 MT
GOBPID Pvalue OddsRatio ExpCount Count Size Term
GO:0006414 5.89933309633214e-57 64.6297948260482 1.57670749429105
46 105 translational elongation
GO:0006614 5.89933309633214e-57 64.6297948260482 1.57670749429105
46 105 SRP-dependent cotranslational protein targeting to membrane
GO:0006415 6.53477919546686e-57 76.7713688353216 1.36647982838558
44 91 translational termination
GO:0006613 1.03006189095429e-56 63.5481481481482 1.59172375614144
46 106 cotranslational protein targeting to membrane
GO:0045047 1.03006189095429e-56 63.5481481481482 1.59172375614144
46 106 protein targeting to ER
GO:0072599 1.03006189095429e-56 63.5481481481482 1.59172375614144
46 106 establishment of protein localization to endoplasmic reticulum
GO:0070972 4.96891286863683e-54 52.9119558154646 1.77191889834614
46 118 protein localization to endoplasmic reticulum
GO:0000184 4.10039540896099e-52 50.0628535512256 1.78693516019653
45 119 nuclear-transcribed mRNA catabolic process, nonsense-mediated decay
GO:0006413 3.57260818519646e-51 38.5889849795175 2.28247180125943
48 152 translational initiation
GO:0019080 3.5160917247145e-48 36.1979392926761 2.26745553940904
46 151 viral genome expression
GO:0019083 3.5160917247145e-48 36.1979392926761 2.26745553940904
46 151 viral transcription
GO:0006612 7.04185746667432e-48 35.5163141498606 2.29748806310982
46 153 protein targeting to membrane
GO:0043624 2.46605133196567e-46 34.2199612403101 2.29748806310982
45 153 cellular protein complex disassembly
GO:0000956 1.25329217738777e-45 30.8613131745353 2.53774825271607
46 169 nuclear-transcribed mRNA catabolic process
GO:0043241 1.27470216854243e-45 32.6942272072443 2.37256937236177
45 158 protein complex disassembly
GO:0034623 5.60180436364997e-45 29.6452850877193 2.61282956196803
46 174 cellular macromolecular complex disassembly
GO:0006402 1.78824078307116e-44 28.7387914230019 2.67289460936959
46 178 mRNA catabolic process
GO:0032984 2.37853567125793e-44 28.5206876841226 2.68791087121998
46 179 macromolecular complex disassembly
GO:0072594 6.88679885481271e-43 24.7890221056576 3.06331741747976
47 204 establishment of protein localization to organelle
GO:0006401 2.15182360495151e-41 23.812939056236 3.07833367933015 46
205 RNA catabolic process
GO:0019058 7.45876356362686e-39 20.4284495021337 3.46875648744032
46 231 viral infectious cycle
GO:0022415 1.1977323386528e-37 18.1990334161834 3.90422808110165
47 260 viral reproductive process
GO:0071845 5.09744630689954e-37 16.8464547186842 4.26461836551104
48 284 cellular component disassembly at cellular level
GO:0022411 1.01277834122005e-36 16.5609467455621 4.3246834129126 48
288 cellular component disassembly
GO:0033365 1.24346992248694e-32 10.9230273657701 7.10269185523493
54 473 protein localization to organelle
GO:0016032 1.33541202097787e-31 10.9276220855168 6.77233409452633
52 451 viral reproduction
GO:0006605 1.50289697290595e-31 10.6032240310527 7.11770811708532
53 474 protein targeting
GO:0042274 4.32260026440763e-09 43.1 0.270292713307038 7 18
ribosomal small subunit biogenesis
GO:0042254 3.81992070276261e-07 6.60589677047289 2.23742301570826

13	149	ribosome biogenesis
GO:0022613	1.55064279758008e-06	4.95898161244696	3.37865891633797
15	225	ribonucleoprotein complex biogenesis
GO:0071843	3.27945978912267e-06	4.64440417256011	3.58888658224344
15	239	cellular component biogenesis at cellular level
GO:0006364	3.61499274462288e-06	7.26693390893206	1.56169123244066
10	104	rRNA processing
GO:0016072	6.00643764925281e-06	6.8280193236715 1.65178880354301	10
110	rRNA metabolic process
GO:0022904	1.37605177398398e-05	7.03597480106101	1.44156113763753
9	96	respiratory electron transport chain
GO:0006417	1.76133241900498e-05	4.90104529616725	2.70292713307038
12	180	regulation of translation
Tissue: Whole_Blood=>Adipose_Subcutaneous	Type:	asymmetric
SourceGene:	RP11-160E2.11(ENSG00000262292.1)
TargetGeneSet:	MTND1P23	DNAJC11 DFFA	MFN2	C1orf144	HSPG2
RPL11	ARID1A	PHACTR4 PRDX3P2 KPNA7	TXLNA	ZBTB8OS MACF1	RPS8	RPS15AP10
RP4	RPL5	RP11	RPRD2	RPS27	ASH1L	SSR2	ARHGEF11	TMEM63A NBAS
PPP1CB	DHX57	UBC	GMCL1	DGUOK	DCTN1	SUCLG1	TMEM131 COA5	EIF5B
RANBP2	UGGT1	USP37	TRIP12	GIGYF2	HDLBP	BRK1	RPL15	RAD54L2 RPL29
FAM208A PCNP	RPL24	DNAJC19 PSMD2	EIF4G1	HTT	AFAP1	BOD1L	RPL34
NDUFC1	RPS3A	NIPBL	CTD	MRPS36	ERAP1	HINT1	SKP1	UBE2B	CTB
MTND5P11	KDM3B	PAIP2	RPS14	SLC2A3P1	RPL15P3 PRRC2A	AGPAT1
SYNGAP1 RPL10A	USP49	MTRNR2L9	WDR11	KIAA1919	RWDD1	MRPS12
TATDN2P2	IGF2R	ANKRD61 MPLKIP	CHCHD2	RPS3AP26	TRRAP	HIPK2	MLL3
DRP2	HMBOX1	KIF13B	KAT6A	HOOK3	PRKDC	RPS20	ARFGEF1 RPL30	EIF3H
MED30	EEF1D	RPL8	RPS6	CLTA	NIPSNAP3A	NUP188	SETX	RPL7A
NACC2	SEC16A	BMS1	RPS24	SFR1	EIF6	NSMCE4A FAM53B	PHRF1	RPL27A
MTRNR2L8	CKAP5	INCENP	ZBTB3	UBE4A	DDX6	USP5	H2AFJ	SCAF11	MLL4
PFDN5	CBX5	USP44	SART3	RP3	SBNO1	EP400	GOLGA3	TPT1	ESD	MED4
MYCBP2-AS1	DNAJC3	CHD8	C14orf21	FNTB	YLPM1	KIAA1737
GOLGA8IP	HERC2	HERC2P10	MGA	CCNDBP1 DUT	RPS27L	MYO9A
C15orf40	SEC11A	LRRK1	C16orf13	FAM173A NME3	SPSB3	TCEB2
RPS15A	GTF3C1	SRCAP	CNOT1	ZNF778	RPL13	VPS53	WDR81	METTL16 ZZEF1
POLR2A	RPL26	TOM1L2	KIAA0100	SUPT6H	RPL23A	TAOK1	RPL17	RPL19
CDK12	RPL27	DHX8	KPNB1	CLTC	DCAF7	NT5C	TNRC6C	TBC1D16 NPLOC4
ANAPC11 RPS15	FAM108A1	AP3D1	NFIC	PTPRS	RPL36	TUBB4A	TRAPPC5
RAB11B	ZNF121	PIN1	TNPO2	C19orf43	NACC1	BRD4	SLC35E1 UPF1
GATAD2A SUPT5H	POU2F2	MEGF8	ZNF428	ARHGAP35	RPL18	RPS5	RRBP1
KIF3B	SCAND1	SOGA1	TOP1	NCOA3	PRPF6	BTG3	SCAF4	URB1	MCM3AP
PI4KA	ZNF70	TTC28	NF2	PRR14L	DEPDC5	MPST	RPL3	EP300	DYNLT3
HUWE1	RPS4X	TTC3P1	RPL39	XIAP	HCFC1	RPL10	MT
GOBPID	Pvalue	OddsRatio	ExpCount	Count	Size	Term
GO:0006415	7.93120786946066e-42	52.4815088757396	1.28461698152377
35	91	translational termination
GO:0006614	7.42072388663324e-41	44.0310559006211	1.48225036329666
36	105	SRP-dependent cotranslational protein targeting to membrane
GO:0006613	1.1109152407343e-40	43.3989795918367	1.49636703342329
36	106	cotranslational protein targeting to membrane
GO:0045047	1.1109152407343e-40	43.3989795918367	1.49636703342329
36	106	protein targeting to ER
GO:0072599	1.1109152407343e-40	43.3989795918367	1.49636703342329
36	106	establishment of protein localization to endoplasmic reticulum
GO:0006414	3.20522472751164e-39	41.9437869822485	1.48225036329666
35	105	translational elongation
GO:0070972	9.97086882210217e-39	37.0165505226481	1.66576707494291
36	118	protein localization to endoplasmic reticulum
GO:0000184	1.41326910528304e-38	36.5679862306368	1.67988374506955
36	119	nuclear-transcribed mRNA catabolic process, nonsense-mediated decay
GO:0019080	2.66846485142243e-37	28.6326900522444	2.13161718912186
38	151	viral genome expression
GO:0019083	2.66846485142243e-37	28.6326900522444	2.13161718912186
38	151	viral transcription

GO:0006413	3.51775361986536e-37	28.3795180722892	2.14573385924849
38	152	translational initiation
GO:0000956	2.83416296036044e-35	24.6669732364573	2.38571725140129
38	169	nuclear-transcribed mRNA catabolic process
GO:0006402	2.35136427778691e-34	23.0665232358003	2.512767282541	38
178	mRNA catabolic process
GO:0006612	3.42251206224134e-34	25.8791208791209	2.15985052937513
36	153	protein targeting to membrane
GO:0043624	8.74469948793941e-33	24.7976632233477	2.15985052937513
35	153	cellular protein complex disassembly
GO:0043241	2.94057867862393e-32	23.7812094097272	2.2304338800083 35
158	protein complex disassembly
GO:0034623	4.941537903453e-32	21.9083850931677	2.45630060203446
36	174	cellular macromolecular complex disassembly
GO:0072594	5.56051456808098e-32	19.4178400348381	2.87980070583351
38	204	establishment of protein localization to organelle
GO:0006401	6.74841506781684e-32	19.3001947911406	2.89391737596014
38	205	RNA catabolic process
GO:0032984	1.4544615601827e-31	21.1348651348651	2.52688395266764
36	179	macromolecular complex disassembly
GO:0022415	1.85677402419733e-31	16.1119421800151	3.67033423292506
41	260	viral reproductive process
GO:0019058	4.23274920995736e-31	17.3025568181818	3.26095079925265
39	231	viral infectious cycle
GO:0033365	6.87570972132975e-27	9.712433838701	6.67718496989828	47
473	protein localization to organelle
GO:0016032	8.45429329251521e-27	9.95049226441632	6.36661822711231
46	451	viral reproduction
GO:0071845	1.90694595434763e-26	13.0286511901264	4.00913431596429
38	284	cellular component disassembly at cellular level
GO:0022411	3.2183712458171e-26	12.8165301204819	4.06560099647083
38	288	cellular component disassembly
GO:0006605	7.27531125689954e-26	9.40012421625458	6.69130164002491
46	474	protein targeting
GO:0016568	5.05915899457672e-07	3.9930389653184 5.91488478306	21	419
chromatin modification
GO:0022613	7.10503497627726e-07	5.30498866213152	3.17625077849284
15	225	ribonucleoprotein complex biogenesis
GO:0071843	1.52047873261881e-06	4.96846655328798	3.37388416026573
15	239	cellular component biogenesis at cellular level
GO:0042274	3.93836789527174e-06	27.5106300734441	0.254100062279427
5	18	ribosomal small subunit biogenesis
GO:0042254	8.55041496082584e-06	5.82710069835549	2.10338384886859
11	149	ribosome biogenesis
Tissue: Whole_Blood=>Adipose_Subcutaneous	Type:	cluster
SourceGene:	RP11-160E2.11(ENSG00000262292.1)
TargetGeneSet:	MTND1P23	DNAJC11 DFFA	MFN2	C1orf144	HSPG2
RPL11	ARID1A	PHACTR4 PRDX3P2 KPNA7	TXLNA	ZBTB8OS MACF1	RPS8	RPS15AP10
RP4	RPL5	RP11	RPRD2	RPS27	ASH1L	SSR2	ARHGEF11	TMEM63A NBAS
PPP1CB	DHX57	UBC	GMCL1	DGUOK	DCTN1	SUCLG1	TMEM131 COA5	EIF5B
RANBP2	UGGT1	USP37	TRIP12	GIGYF2	HDLBP	BRK1	RPL15	RAD54L2 RPL29
FAM208A PCNP	RPL24	DNAJC19 PSMD2	EIF4G1	HTT	AFAP1	BOD1L	RPL34
NDUFC1	RPS3A	NIPBL	CTD	MRPS36	ERAP1	HINT1	SKP1	UBE2B	CTB
MTND5P11	KDM3B	PAIP2	RPS14	SLC2A3P1	RPL15P3 PRRC2A	AGPAT1
SYNGAP1 RPL10A	USP49	MTRNR2L9	WDR11	KIAA1919	RWDD1	MRPS12
TATDN2P2	IGF2R	ANKRD61 MPLKIP	CHCHD2	RPS3AP26	TRRAP	HIPK2	MLL3
DRP2	HMBOX1	KIF13B	KAT6A	HOOK3	PRKDC	RPS20	ARFGEF1 RPL30	EIF3H
MED30	EEF1D	RPL8	RPS6	CLTA	NIPSNAP3A	NUP188	SETX	RPL7A
NACC2	SEC16A	BMS1	RPS24	SFR1	EIF6	NSMCE4A FAM53B	PHRF1	RPL27A
MTRNR2L8	CKAP5	INCENP	ZBTB3	UBE4A	DDX6	USP5	H2AFJ	SCAF11	MLL4
PFDN5	CBX5	USP44	SART3	RP3	SBNO1	EP400	GOLGA3	TPT1	ESD	MED4
MYCBP2-AS1	DNAJC3	CHD8	C14orf21	FNTB	YLPM1	KIAA1737
GOLGA8IP	HERC2	HERC2P10	MGA	CCNDBP1 DUT	RPS27L	MYO9A

C15orf40 SEC11A LRRK1 C16orf13 FAM173A NME3 SPSB3 TCEB2
RPS15A GTF3C1 SRCAP CNOT1 ZNF778 RPL13 VPS53 WDR81 METTL16 ZZEF1
POLR2A RPL26 TOM1L2 KIAA0100 SUPT6H RPL23A TAOK1 RPL17 RPL19
CDK12 RPL27 DHX8 KPNB1 CLTC DCAF7 NT5C TNRC6C TBC1D16 NPLOC4
ANAPC11 RPS15 FAM108A1 AP3D1 NFIC PTPRS RPL36 TUBB4A TRAPPC5
RAB11B ZNF121 PIN1 TNPO2 C19orf43 NACC1 BRD4 SLC35E1 UPF1
GATAD2A SUPT5H POU2F2 MEGF8 ZNF428 ARHGAP35 RPL18 RPS5 RRBP1
KIF3B SCAND1 SOGA1 TOP1 NCOA3 PRPF6 BTG3 SCAF4 URB1 MCM3AP
PI4KA ZNF70 TTC28 NF2 PRR14L DEPDC5 MPST RPL3 EP300 DYNLT3
HUWE1 RPS4X TTC3P1 RPL39 XIAP HCFC1 RPL10 MT
GOBPID Pvalue OddsRatio ExpCount Count Size Term
GO:0006415 7.93120786946066e-42 52.4815088757396 1.28461698152377
35 91 translational termination
GO:0006614 7.42072388663324e-41 44.0310559006211 1.48225036329666
36 105 SRP-dependent cotranslational protein targeting to membrane
GO:0006613 1.1109152407343e-40 43.3989795918367 1.49636703342329
36 106 cotranslational protein targeting to membrane
GO:0045047 1.1109152407343e-40 43.3989795918367 1.49636703342329
36 106 protein targeting to ER
GO:0072599 1.1109152407343e-40 43.3989795918367 1.49636703342329
36 106 establishment of protein localization to endoplasmic reticulum
GO:0006414 3.20522472751164e-39 41.9437869822485 1.48225036329666
35 105 translational elongation
GO:0070972 9.97086882210217e-39 37.0165505226481 1.66576707494291
36 118 protein localization to endoplasmic reticulum
GO:0000184 1.41326910528304e-38 36.5679862306368 1.67988374506955
36 119 nuclear-transcribed mRNA catabolic process, nonsense-mediated decay
GO:0019080 2.66846485142243e-37 28.6326900522444 2.13161718912186
38 151 viral genome expression
GO:0019083 2.66846485142243e-37 28.6326900522444 2.13161718912186
38 151 viral transcription
GO:0006413 3.51775361986536e-37 28.3795180722892 2.14573385924849
38 152 translational initiation
GO:0000956 2.83416296036044e-35 24.6669732364573 2.38571725140129
38 169 nuclear-transcribed mRNA catabolic process
GO:0006402 2.35136427778691e-34 23.0665232358003 2.512767282541 38
178 mRNA catabolic process
GO:0006612 3.42251206224134e-34 25.8791208791209 2.15985052937513
36 153 protein targeting to membrane
GO:0043624 8.74469948793941e-33 24.7976632233477 2.15985052937513
35 153 cellular protein complex disassembly
GO:0043241 2.94057867862393e-32 23.7812094097272 2.2304338800083 35
158 protein complex disassembly
GO:0034623 4.941537903453e-32 21.9083850931677 2.45630060203446
36 174 cellular macromolecular complex disassembly
GO:0072594 5.56051456808098e-32 19.4178400348381 2.87980070583351
38 204 establishment of protein localization to organelle
GO:0006401 6.74841506781684e-32 19.3001947911406 2.89391737596014
38 205 RNA catabolic process
GO:0032984 1.4544615601827e-31 21.1348651348651 2.52688395266764
36 179 macromolecular complex disassembly
GO:0022415 1.85677402419733e-31 16.1119421800151 3.67033423292506
41 260 viral reproductive process
GO:0019058 4.23274920995736e-31 17.3025568181818 3.26095079925265
39 231 viral infectious cycle
GO:0033365 6.87570972132975e-27 9.712433838701 6.67718496989828 47
473 protein localization to organelle
GO:0016032 8.45429329251521e-27 9.95049226441632 6.36661822711231
46 451 viral reproduction
GO:0071845 1.90694595434763e-26 13.0286511901264 4.00913431596429
38 284 cellular component disassembly at cellular level
GO:0022411 3.2183712458171e-26 12.8165301204819 4.06560099647083
38 288 cellular component disassembly

GO:0006605	7.27531125689954e-26	9.40012421625458	6.69130164002491
46	474	protein targeting
GO:0016568	5.05915899457672e-07	3.9930389653184 5.91488478306	21	419
chromatin modification
GO:0022613	7.10503497627726e-07	5.30498866213152	3.17625077849284
15	225	ribonucleoprotein complex biogenesis
GO:0071843	1.52047873261881e-06	4.96846655328798	3.37388416026573
15	239	cellular component biogenesis at cellular level
GO:0042274	3.93836789527174e-06	27.5106300734441	0.254100062279427
5	18	ribosomal small subunit biogenesis
GO:0042254	8.55041496082584e-06	5.82710069835549	2.10338384886859
11	149	ribosome biogenesis
Tissue: Whole_Blood=>Adipose_Subcutaneous	Type:	asymmetric
SourceGene:	RP11-676J12.7(ENSG00000262003.1)
TargetGeneSet:	RPL22	MST1P9	KHDRBS1 RP4	RP11	GAS5	RPS7	UBC
RPL31	EEF1B2	RPL32	RPL15	CCDC12	RPL24	RPL34	BTF3	CTD	UBE2D2
RPS14	RPS18	SLC26A4 RPL30	EIF3H	RPS6	ANP32B	RPL35	RPL7A	RPS24
KRT17	NAP1L1	TPT1	HMGN1P24	MED6	RPS3AP6 SEC11A	CHMP1A	RPL26
RPS7P1	RPL17	RPL27	FASN	FAM32A	RPS11	CCDC106 RPL3	PGAM1P7 RPS4X
GOBPID	Pvalue	OddsRatio	ExpCount	Count	Size	Term
GO:0006414	7.28762727938273e-39	253.238554216867	0.268839526676355
22	105	translational elongation
GO:0006614	7.28762727938273e-39	253.238554216867	0.268839526676355
22	105	SRP-dependent cotranslational protein targeting to membrane
GO:0006613	9.18709778112143e-39	250.206349206349	0.271399903120891
22	106	cotranslational protein targeting to membrane
GO:0045047	9.18709778112143e-39	250.206349206349	0.271399903120891
22	106	protein targeting to ER
GO:0072599	9.18709778112143e-39	250.206349206349	0.271399903120891
22	106	establishment of protein localization to endoplasmic reticulum
GO:0006415	6.00536191284438e-38	268.95	0.232994256452841	21	91
translational termination
GO:0070972	1.23979948816468e-37	218.747222222222	0.302124420455332
22	118	protein localization to endoplasmic reticulum
GO:0000184	3.05396912720757e-35	191.732142857143	0.304684796899869
21	119	nuclear-transcribed mRNA catabolic process, nonsense-mediated decay
GO:0006413	5.14374647620722e-35	161.152820512821	0.38917721956958
22	152	translational initiation
GO:0006612	6.00155745904302e-35	159.911450381679	0.391737596014117
22	153	protein targeting to membrane
GO:0019080	6.65844791257209e-33	144.213461538462	0.386616843125043
21	151	viral genome expression
GO:0019083	6.65844791257209e-33	144.213461538462	0.386616843125043
21	151	viral transcription
GO:0043624	8.93583699021507e-33	142.008522727273	0.391737596014117
21	153	cellular protein complex disassembly
GO:0043241	1.83147031627765e-32	136.777828467153	0.4045394782368 21
158	protein complex disassembly
GO:0072594	4.82396830690036e-32	114.69010989011 0.522316794685489	22
204	establishment of protein localization to organelle
GO:0000956	8.17637173766711e-32	126.514358108108	0.432703619126704
21	169	nuclear-transcribed mRNA catabolic process
GO:0034623	1.55961763645306e-31	122.337009803922	0.445505501349388
21	174	cellular macromolecular complex disassembly
GO:0006402	2.57767079133985e-31	119.186703821656	0.455747007127534
21	178	mRNA catabolic process
GO:0032984	2.91715038613628e-31	118.424050632911	0.458307383572071
21	179	macromolecular complex disassembly
GO:0006401	5.74692238821335e-30	101.504755434783	0.524877171130026
21	205	RNA catabolic process
GO:0019058	7.75910438986615e-29	88.775	0.59144695868798	21	231
viral infectious cycle
GO:0022415	1.00353320410872e-27	77.8438807531381	0.665697875579545

21	260	viral reproductive process
GO:0071845	6.71095433016046e-27	70.6204847908745	0.727146910248426
21	284	cellular component disassembly at cellular level
GO:0022411	9.06057056968201e-27	69.5428370786517	0.737388416026573
21	288	cellular component disassembly
GO:0033365	7.84288111884257e-24	45.4081300813008	1.21105805826586
22	473	protein localization to organelle
GO:0006605	8.2161911069685e-24	45.3044247787611	1.2136184347104 22
474	protein targeting
GO:0016032	1.23042816851143e-22	42.6837209302326	1.15472977648606
21	451	viral reproduction
GO:0022613	7.98120775456516e-08	18.0479898299698	0.57608470002076
8	225	ribonucleoprotein complex biogenesis
GO:0042254	8.54563461789605e-08	23.4516431924883	0.38149609023597
7	149	ribosome biogenesis
GO:0042274	1.08453084725298e-07	124.675324675325	0.0460867760016608
4	18	ribosomal small subunit biogenesis
GO:0071843	1.27078117795642e-07	16.9374533512465	0.611929970244274
8	239	cellular component biogenesis at cellular level
GO:0006364	6.3609947487871e-06	22.5931186868687	0.266279150231818
5	104	rRNA processing
GO:0016072	8.37237154329821e-06	21.2931547619048	0.281641408899038
5	110	rRNA metabolic process
Tissue: Whole_Blood=>Adipose_Subcutaneous	Type:	asymmetric

SourceGene:	SKA2(ENSG00000182628.7) spindle and kinetochore associated complex subunit 2

TargetGeneSet:	VPS13D	UBR4	KIAA0090	HSPG2	RPL11	PRDX3P2	KPNA7	SZT2
RPS8	RPL5	RP11	ASH1L	MIR555	GON4L	LPGAT1	SOS1	COX7A2L	USP34	TET3
VAMP5	RANBP2	UGGT1	TRIP12	GPC1	RPL32	RPL15	RPL29	PCNP	SNORA7B
DNAJC19	HTT	BOD1L	COPS4	RPL34	NDUFC1	MTRR	NIPBL	BDP1	SKP1
RPS14	RPL15P3	LINC00243	CUL9	XPO5	MLL3	NOM1	KAT6A	PRKDC
TTC35	EIF3H	CTA	RANBP6	RPS6	CLTA	TNC	NUP188	SETX	RPL7A
QSOX2	PHYH	KIAA0913	PDZD8	EIF6	LRRC56	RPL27A	PIGCP1	KDM2A	GAL
CCDC90B	ATM	UBE4A	MLL4	PFDN5	XPOT	RP1	MLXIP	SBNO1	TPT1
ZC3H13	COMMD6	COL4A2-AS1	TEP1	CHD8	NIN	HERC2	MGA	RPS27L
CALML4	CIB1	NDUFB10	CIITA	RPS15A	SMG1	SRCAP	CNOT1	AARS	ZNF23
CHST4	RPL13	ZZEF1	RPL26	C17orf48	SLC47A1	SUPT6H	RPL23A	RPL17
RPL19	AOC2	CTD	ZNF236	RPS15	BRD4	ZNF587	LBP	RALGAPB	SYNJ1	SON

UBE2G2 MCM3AP MPST EP300 ZBED4 ZNF81 KDM5C HUWE1 RPS4X RPL39 RP4 RPL10

GOBPID	Pvalue	OddsRatio	ExpCount	Count	Size	Term
GO:0006415	5.08E-26	48.65454545	0.686388485	21	91	translational termination
GO:0000184	6.21E-25	37.13591658	0.897584942	22	119	"nuclear-transcribed mRNA catabolic process, nonsense-mediated decay"
GO:0006414	1.35E-24	40.50568182	0.791986714	21	105	translational elongation
GO:0006614	1.35E-24	40.50568182	0.791986714	21	105	SRP-dependent cotranslational protein targeting to membrane

GO:0006613	1.68E-24	40.0263369	0.799529444	21	106
cotranslational protein targeting to membrane					
GO:0045047	1.68E-24	40.0263369	0.799529444	21	106
protein targeting to ER					
GO:0072599	1.68E-24	40.0263369	0.799529444	21	106
establishment of protein localization to endoplasmic reticulum					
GO:0000956	2.84E-24	27.64527383	1.274721473	24	169
nuclear-transcribed mRNA catabolic process					

GO:0006402	1.03E-23	26.0131398	1.342606048	24	178	mRNA catabolic process
GO:0070972	1.87E-23	35.04510309	0.890042212	21	118	protein localization to endoplasmic reticulum
GO:0019080	1.54E-22	27.86117794	1.138952322	22	151	viral genome expression
GO:0019083	1.54E-22	27.86117794	1.138952322	22	151	viral transcription
GO:0006413	1.79E-22	27.644916	1.146495052	22	152	translational initiation

GO:0043624	2.08E-22	27.43195578	1.154037783	22	153
cellular protein complex disassembly

GO:0006401	3.26E-22	22.09060773	1.546259774	24	205	RNA
catabolic process

GO:0043241	4.32E-22	26.41413117	1.191751436	22	158
protein complex disassembly

GO:0034623	3.81E-21	23.60707804	1.312435126	22	174
cellular macromolecular complex disassembly

GO:0006612	5.61E-21	25.68956612	1.154037783	21	153
protein targeting to membrane					
GO:0032984	7.19E-21	22.84720697	1.350148779	22	179
macromolecular complex disassembly					
GO:0072594	1.31E-19	19.67411898	1.538717044	22	204
establishment of protein localization to organelle					
GO:0019058	1.98E-18	17.09981851	1.74237077	22	231
viral infectious cycle					
GO:0006605	8.86E-18	10.77030394	3.575254308	28	474
protein targeting					

GO:0022415	2.54E-17	14.98541486	1.961109958	22	260
viral reproductive process					
GO:0033365	8.96E-17	10.25899595	3.567711577	27	473
protein localization to organelle					
GO:0071845	1.67E-16	13.58954111	2.142135492	22	284
cellular component disassembly at cellular level					
GO:0022411	2.24E-16	13.3813845	2.172306415	22	288
cellular component disassembly					

GO:0016032	2.94E-15	9.722222222	3.401771504	25	451
viral reproduction					
GO:0042254	1.83E-06	9.129857143	1.12386686	9	149
ribosome biogenesis					
GO:0016570	4.35E-06	6.327020408	1.968652688	11	261
histone modification					
GO:0016569	5.40E-06	6.176100128	2.013909072	11	267
covalent chromatin modification					
GO:0022613	7.61E-06	6.637068358	1.697114387	10	225
ribonucleoprotein complex biogenesis					

GO:0042274	8.64E-06	38.9877551	0.135769151	4	18	ribosomal small subunit biogenesis
GO:0071843	1.29E-05	6.22513343	1.802712615	10	239	cellular component biogenesis at cellular level
GO:0006364	1.33E-05	10.0783303	0.784443983	7	104	rRNA processing
GO:0016568	1.59E-05	4.64819376	3.160404124	13	419	chromatin modification

GO:0016072 1.92E-05 9.487245384 0.829700367 7 110 rRNA metabolic process

Tissue: Whole_Blood=>Adipose_Subcutaneous Type: cluster
SourceGene: SKA2(ENSG00000182628.7)
TargetGeneSet: VPS13D UBR4 KIAA0090 HSPG2 RPL11 PRDX3P2 KPNA7 SZT2 RPS8 RPL5 RP11 ASH1L MIR555 GON4L LPGAT1 SOS1 COX7A2L USP34 TET3 VAMP5 RANBP2 UGGT1 TRIP12 GPC1 RPL32 RPL15 RPL29 PCNP SNORA7B DNAJC19 HTT BOD1L COPS4 RPL34 NDUFC1 MTRR NIPBL BDP1 SKP1 RPS14 RPL15P3 LINC00243 CUL9 XPO5 MLL3 NOM1 KAT6A PRKDC TTC35 EIF3H CTA RANBP6 RPS6 CLTA TNC NUP188 SETX RPL7A QSOX2 PHYH KIAA0913 PDZD8 EIF6 LRRC56 RPL27A PIGCP1 KDM2A GAL CCDC90B ATM UBE4A MLL4 PFDN5 XPOT RP1 MLXIP SBNO1 TPT1 ZC3H13 COMMD6 COL4A2-AS1 TEP1 CHD8 NIN HERC2 MGA RPS27L CALML4 CIB1 NDUFB10 CIITA RPS15A SMG1 SRCAP CNOT1 AARS ZNF23 CHST4 RPL13 ZZEF1 RPL26 C17orf48 SLC47A1 SUPT6H RPL23A RPL17 RPL19 AOC2 CTD ZNF236 RPS15 BRD4 ZNF587 LBP RALGAPB SYNJ1 SON UBE2G2 MCM3AP MPST EP300 ZBED4 ZNF81 KDM5C HUWE1 RPS4X RPL39 RP4 RPL10

GOBPID	Pvalue	OddsRatio	ExpCount	Count	Size	Term
GO:0006415	5.08265285222481e-26	48.6545454545455	0.686388485225936	21	91	translational termination
GO:0000184	6.20707647381988e-25	37.1359165777936	0.897584942218532	22	119	nuclear-transcribed mRNA catabolic process, nonsense-mediated decay
GO:0006414	1.35355223956287e-24	40.5056818181818	0.791986713722234	21	105	translational elongation
GO:0006614	1.35355223956287e-24	40.5056818181818	0.791986713722234	21	105	SRP-dependent cotranslational protein targeting to membrane
GO:0006613	1.67808866830512e-24	40.0263368983957	0.799529444329112	21	106	cotranslational protein targeting to membrane
GO:0045047	1.67808866830512e-24	40.0263368983957	0.799529444329112	21	106	protein targeting to ER
GO:0072599	1.67808866830512e-24	40.0263368983957	0.799529444329112	21	106	establishment of protein localization to endoplasmic reticulum
GO:0000956	2.84276513215265e-24	27.6452738336714	1.27472147256245	24	169	nuclear-transcribed mRNA catabolic process
GO:0006402	1.02718916831435e-23	26.0131398013751	1.34260604802436	24	178	mRNA catabolic process
GO:0070972	1.8718659352694e-23	35.0451030927835	0.890042211611653	21	118	protein localization to endoplasmic reticulum
GO:0019080	1.54188313293787e-22	27.8611779381627	1.13895232163864	22	151	viral genome expression
GO:0019083	1.54188313293787e-22	27.8611779381627	1.13895232163864	22	151	viral transcription
GO:0006413	1.79234540152662e-22	27.6449160035367	1.14649505224552	22	152	translational initiation
GO:0043624	2.08118992313669e-22	27.4319557778363	1.1540377828524	22	153	cellular protein complex disassembly
GO:0006401	3.26019612331964e-22	22.0906077348066	1.54625977441008	24	205	RNA catabolic process

GO:0043241	4.32304443474305e-22	26.4141311697093	1.19175143588679
22	158	protein complex disassembly
GO:0034623	3.80972730832128e-21	23.6070780399274	1.31243512559684
22	174	cellular macromolecular complex disassembly
GO:0006612	5.61471104620423e-21	25.6895661157025	1.1540377828524 21
153	protein targeting to membrane
GO:0032984	7.18547446031269e-21	22.8472069697635	1.35014877863124
22	179	macromolecular complex disassembly
GO:0072594	1.30764677196797e-19	19.6741189844638	1.5387170438032 22
204	establishment of protein localization to organelle
GO:0019058	1.982088327887e-18	17.0998185117967	1.74237077018891
22	231	viral infectious cycle
GO:0006605	8.86029571348654e-18	10.7703039362232	3.57525430766037
28	474	protein targeting
GO:0022415	2.5398257890994e-17	14.9854148555974	1.96110995778839
22	260	viral reproductive process
GO:0033365	8.96421478629371e-17	10.2589959531882	3.56771157705349
27	473	protein localization to organelle
GO:0071845	1.66613343238302e-16	13.5895411073089	2.14213549235347
22	284	cellular component disassembly at cellular level
GO:0022411	2.24033549592335e-16	13.3813844957221	2.17230641478098
22	288	cellular component disassembly
GO:0016032	2.94083958466102e-15	9.72222222222222	3.40177150370217
25	451	viral reproduction
GO:0042254	1.83429122788905e-06	9.12985714285714	1.12386686042488
9	149	ribosome biogenesis
GO:0016570	4.34628421000627e-06	6.32702040816327	1.96865268839527
11	261	histone modification
GO:0016569	5.40119841554895e-06	6.17610012755102	2.01390907203654
11	267	covalent chromatin modification
GO:0022613	7.6149980667054e-06	6.63706835799859	1.69711438654764
10	225	ribonucleoprotein complex biogenesis
GO:0042274	8.6384978716643e-06	38.9877551020408	0.135769150923812
4	18	ribosomal small subunit biogenesis
GO:0071843	1.2912402130024e-05	6.2251334303736 1.80271261504394	10
239	cellular component biogenesis at cellular level
GO:0006364	1.33223485277612e-05	10.0783303011926	0.784443983115355
7	104	rRNA processing
GO:0016568	1.58957143921517e-05	4.64819376026273	3.16040412428206
13	419	chromatin modification
GO:0016072	1.92310225544999e-05	9.48724538359033	0.829700366756626
7	110	rRNA metabolic process
Tissue: Whole_Blood=>Adipose_Subcutaneous	Type:	asymmetric

SourceGene:	SPRR2E(ENSG00000203785.4)	small proline-rich protein 2E

TargetGeneSet: RPL22 RP11 RPL11 RP4 RPS8 RPL5 LCE2C S100A13
CICP13 B3GALNT2 PREPL UBC LONRF2 RPL31 R3HDM1 EEF1B2 SP100
CHL1-AS2 RPL32 UBE2E1 GORASP1 RHOA RNF123 FAM208A RPL24 EIF4G1 LPP
RPL35A UBE2D3 RPL34 FRG1 CTD BTF3 DTWD2 HINT1 UBE2B PAIP2
RPS14 SLU7 GRM4 RPL39P3 ASCC3 TNRC18 DAGLB RP9 CUX1 GTF2E2
RPS20 RPL30 PLEC RPS6 FOXD4L5 CALML5 RPS24 KLLN GBF1 NSMCE4A FAU
CCND1 RPS25 ING4 USP5 CLEC2B KRT5 PFDN5 RPL41 RPS7P10 TPT1
COMMD6 NGDN KLHDC2 CGRRF1 SNW1 HERC2 CCNDBP1 RPS3AP6 RPS17L SEC11A TSC2
GTF3C1 YPEL3 IST1 TERF2IP MYBBP1A RPL26 RPL23A ZNHIT3 RPL17 RPL19
KRT10 RPL27 RPS26P8 PNPO RPTOR DSG1 WDR7 PIP5K1C C19orf43
CASP14 DMWD CCDC106 CHMP4B TH1L PPIL2 TTC28 RPL3 PHF16 RPS4X
RPL39 RP1 FLNA

GOBPID	Pvalue	OddsRatio	ExpCount	Count	Size	Term
GO:0006415	7.73E-39	91.36789773	0.585634212	27	91	translational termination
GO:0006414	8.37E-39	79.89370629	0.675731783	28	105	translational elongation
GO:0006614	8.37E-39	79.89370629	0.675731783	28	105	SRP-dependent cotranslational protein targeting to membrane

GO:0006613	1.13E-38	78.86390533	0.682167324	28	106
cotranslational protein targeting to membrane					
GO:0045047	1.13E-38	78.86390533	0.682167324	28	106
protein targeting to ER					
GO:0072599	1.13E-38	78.86390533	0.682167324	28	106
establishment of protein localization to endoplasmic reticulum					
GO:0070972	3.35E-37	68.29128205	0.759393814	28	118
protein localization to endoplasmic reticulum					
GO:0000184	4.36E-37	67.53609467	0.765829354	28	119
"nuclear-transcribed mRNA catabolic process, nonsense-mediated decay"					

GO:0019080	1.26E-35	52.8744877	0.97176666	29	151
viral genome expression					
GO:0019083	1.26E-35	52.8744877	0.97176666	29	151
viral transcription					
GO:0006413	1.55E-35	52.44092988	0.978202201	29	152
translational initiation					
GO:0006612	9.70E-34	49.04910769	0.984637741	28	153
protein targeting to membrane					
GO:0000956	1.91E-32	43.43436989	1.087606394	28	169
nuclear-transcribed mRNA catabolic process					

GO:0043624	4.66E-32	46.20779221	0.984637741	27	153	cellular protein complex disassembly
GO:0006402	8.90E-32	40.80246154	1.145526261	28	178	mRNA catabolic process
GO:0043241	1.18E-31	44.42852186	1.016815445	27	158	protein complex disassembly
GO:0034623	1.86E-30	39.54823748	1.119784098	27	174	cellular macromolecular complex disassembly
GO:0032984	4.17E-30	38.23385167	1.151961802	27	179	macromolecular complex disassembly
GO:0072594	4.88E-30	34.71118881	1.312850322	28	204	

establishment of protein localization to organelle

GO:0006401 5.62E-30 34.51264668 1.319285863 28 205 RNA
catabolic process

GO:0019058 5.71E-30 31.75464109 1.486609923 29 231
viral infectious cycle

GO:0022415 1.92E-28 27.71124188 1.673240606 29 260
viral reproductive process

GO:0071845 2.58E-27 25.06047794 1.827693585 29 284
cellular component disassembly at cellular level

GO:0022411 3.89E-27 24.66644546 1.853435748 29 288
cellular component disassembly

GO:0033365	1.47E-24	16.55492361	3.044010795	32	473	protein localization to organelle
GO:0006605	2.55E-23	15.70541761	3.050446336	31	474	protein targeting
GO:0016032	9.34E-23	15.76405384	2.902428898	30	451	viral reproduction
GO:0042274	1.05E-09	82.44827586	0.115839734	6	18	ribosomal small subunit biogenesis
GO:0006364	1.18E-09	18.28249167	0.669296242	10	104	rRNA processing

GO:0022613	1.93E-09	10.84304245	1.447996678	13	225	ribonucleoprotein complex biogenesis
GO:0016072	2.06E-09	17.17831325	0.707909487	10	110	rRNA metabolic process
GO:0042254	2.85E-09	13.82290562	0.958895578	11	149	ribosome biogenesis
GO:0071843	4.02E-09	10.16128319	1.53809425	13	239	cellular component biogenesis at cellular level
GO:0000028	2.39E-08	322.6067416	0.038613245	4	6	ribosomal small subunit assembly

GO:0034470	6.13E-07	8.984147115	1.287108159	10	200
ncRNA processing					
GO:0042273	7.65E-07	80.61797753	0.07722649	4	12
ribosomal large subunit biogenesis					
GO:0042255	2.76E-06	53.73033708	0.102968653	4	16
ribosome assembly					
GO:0030490	8.86E-06	119.6166667	0.045048786	3	7
maturation of SSU-rRNA					
GO:0034660	1.45E-05	6.169989047	1.834129126	10	285
ncRNA metabolic process					

Tissue: Whole_Blood=>Adipose_Subcutaneous Type: cluster
SourceGene: SPRR2E(ENSG00000203785.4)
TargetGeneSet: RPL22 RP11 RPL11 RP4 RPS8 RPL5 LCE2C S100A13
CICP13 B3GALNT2 PREPL UBC LONRF2 RPL31 R3HDM1 EEF1B2 SP100
CHL1-AS2 RPL32 UBE2E1 GORASP1 RHOA RNF123 FAM208A RPL24 EIF4G1 LPP
RPL35A UBE2D3 RPL34 FRG1 CTD BTF3 DTWD2 HINT1 UBE2B PAIP2
RPS14 SLU7 GRM4 RPL39P3 ASCC3 TNRC18 DAGLB RP9 CUX1 GTF2E2
RPS20 RPL30 PLEC RPS6 FOXD4L5 CALML5 RPS24 KLLN GBF1 NSMCE4A FAU
CCND1 RPS25 ING4 USP5 CLEC2B KRT5 PFDN5 RPL41 RPS7P10 TPT1
COMMD6 NGDN KLHDC2 CGRRF1 SNW1 HERC2 CCNDBP1 RPS3AP6 RPS17L SEC11A TSC2
GTF3C1 YPEL3 IST1 TERF2IP MYBBP1A RPL26 RPL23A ZNHIT3 RPL17 RPL19
KRT10 RPL27 RPS26P8 PNPO RPTOR DSG1 WDR7 PIP5K1C C19orf43
CASP14 DMWD CCDC106 CHMP4B TH1L PPIL2 TTC28 RPL3 PHF16 RPS4X
RPL39 RP1 FLNA
GOBPID Pvalue OddsRatio ExpCount Count Size Term
GO:0006415 7.72884393684555e-39 91.3678977272727 0.585634212165248
27 91 translational termination
GO:0006414 8.37337098585874e-39 79.8937062937063 0.675731783267594
28 105 translational elongation
GO:0006614 8.37337098585874e-39 79.8937062937063 0.675731783267594
28 105 SRP-dependent cotranslational protein targeting to membrane
GO:0006613 1.13294458388643e-38 78.8639053254438 0.682167324060619
28 106 cotranslational protein targeting to membrane
GO:0045047 1.13294458388643e-38 78.8639053254438 0.682167324060619
28 106 protein targeting to ER
GO:0072599 1.13294458388643e-38 78.8639053254438 0.682167324060619
28 106 establishment of protein localization to endoplasmic reticulum
GO:0070972 3.34821915675842e-37 68.2912820512821 0.759393813576915
28 118 protein localization to endoplasmic reticulum
GO:0000184 4.35928217000196e-37 67.5360946745562 0.76582935436994
28 119 nuclear-transcribed mRNA catabolic process, nonsense-mediated decay
GO:0019080 1.26227486656478e-35 52.874487704918 0.97176665974673 29
151 viral genome expression
GO:0019083 1.26227486656478e-35 52.874487704918 0.97176665974673 29
151 viral transcription
GO:0006413 1.55314173234673e-35 52.4409298780488 0.978202200539755
29 152 translational initiation
GO:0006612 9.70480562031126e-34 49.0491076923077 0.98463774133278
28 153 protein targeting to membrane
GO:0000956 1.90901476025287e-32 43.4343698854337 1.08760639402118
28 169 nuclear-transcribed mRNA catabolic process
GO:0043624 4.65639857893906e-32 46.2077922077922 0.98463774133278
27 153 cellular protein complex disassembly
GO:0006402 8.90346234957301e-32 40.8024615384615 1.1455262611584 28
178 mRNA catabolic process
GO:0043241 1.17784356769807e-31 44.4285218598196 1.0168154452979 27
158 protein complex disassembly
GO:0034623 1.86496318324784e-30 39.5482374768089 1.1197840979863 27
174 cellular macromolecular complex disassembly
GO:0032984 4.17269589186328e-30 38.2338516746412 1.15196180195142
27 179 macromolecular complex disassembly
GO:0072594 4.87501965816886e-30 34.7111888111888 1.31285032177704
28 204 establishment of protein localization to organelle
GO:0006401 5.62137155944716e-30 34.5126466753585 1.31928586257006
28 205 RNA catabolic process
GO:0019058 5.71349547817322e-30 31.7546410891089 1.48660992318871
29 231 viral infectious cycle
GO:0022415 1.92259800752942e-28 27.7112418831169 1.67324060618642
29 260 viral reproductive process
GO:0071845 2.58264470548763e-27 25.0604779411765 1.82769358521902
29 284 cellular component disassembly at cellular level

GO:0022411	3.88927150912513e-27	24.6664454633205	1.85343574839111
29	288	cellular component disassembly
GO:0033365	1.46869717982695e-24	16.5549236087878	3.04401079510068
32	473	protein localization to organelle
GO:0006605	2.55084263704195e-23	15.7054176072235	3.05044633589371
31	474	protein targeting
GO:0016032	9.34258344922867e-23	15.7640538400633	2.90242889765414
30	451	viral reproduction
GO:0042274	1.05243575360753e-09	82.448275862069 0.115839734274445	6
18	ribosomal small subunit biogenesis
GO:0006364	1.18066354692682e-09	18.2824916688029	0.669296242474569
10	104	rRNA processing
GO:0022613	1.93229251667979e-09	10.8430424528302	1.44799667843056
13	225	ribonucleoprotein complex biogenesis
GO:0016072	2.05563449216074e-09	17.178313253012 0.707909487232717	10
110	rRNA metabolic process
GO:0042254	2.85318349956482e-09	13.8229056203606	0.958895578160681
11	149	ribosome biogenesis
GO:0071843	4.02171194587049e-09	10.1612831858407	1.5380942495329 13
239	cellular component biogenesis at cellular level
GO:0000028	2.38750166526337e-08	322.606741573034	0.0386132447581482
4	6	ribosomal small subunit assembly
GO:0034470	6.13445314774594e-07	8.98414711477489	1.28710815860494
10	200	ncRNA processing
GO:0042273	7.64878389605521e-07	80.6179775280899	0.0772264895162964
4	12	ribosomal large subunit biogenesis
GO:0042255	2.75732673726494e-06	53.7303370786517	0.102968652688395
4	16	ribosome assembly
GO:0030490	8.86430808154284e-06	119.616666666667	0.0450487855511729
3	7	maturation of SSU-rRNA
GO:0034660	1.45155741712303e-05	6.16998904709748	1.83412912601204
10	285	ncRNA metabolic process
Tissue: Artery_Tibial=>Adipose_Subcutaneous	Type:	asymmetric
SourceGene:	C11orf31(ENSG00000211450.4)
TargetGeneSet:	SDHB	RPL11	USP1	RPL5	RP11	SARS	ATP5F1	PSMB4	DAP3
MRPL55	TOMM20	FOSL2	UBC	FAM136A	MMADHC	RPL15	NICN1	NICN1-AS1
RPL29	EIF2S1	PDCD10	PARL	OCIAD1	COPS4	RPS3A	NDUFS4	MRPS36	BTF3	TBCA
SNX2	HINT1	CTB	RPS14	TTC1	NUDCD2	NPM1	NOL7	C6orf136
RPS10	RP1	RWDD1	C6orf72	BTF3P10	PMPCB	MLL5	HMBOX1	DCTN6	RPS20P22
RPS20	SNHG6	RPL30	POLR2K	EIF3H	NDUFB9	CLTA	ARHGAP12	VDAC2
RPS24	RPP30	HBG2	RPL27A	C11orf2	RPS25	WNK1	C12orf57	PHB2
PFDN5	RBMS2	SLC25A3	ARL6IP4	POLR1D	ESD	JKAMP	ATPBD4	TGM6	PSMA4
SEC11A	RPL23A	RPL17	RPL19	RPL27	MSI2	NT5C	PSMG2	ABCA7	EEF2
RPL36	EIF3K	EID2B	RPL18	RPL13A	A1BG	RPS5	MANBAL	PFDN4	POLR2F
SLC25A6	RBM3
GOBPID	Pvalue	OddsRatio	ExpCount	Count	Size	Term
GO:0006414	3.08424583132542e-29	63.110843373494 0.595806518580029	22
105	translational elongation
GO:0006614	3.08424583132542e-29	63.110843373494 0.595806518580029	22
105	SRP-dependent cotranslational protein targeting to membrane
GO:0006613	3.87646818659544e-29	62.3551587301587	0.601480866376029
22	106	cotranslational protein targeting to membrane
GO:0045047	3.87646818659544e-29	62.3551587301587	0.601480866376029
22	106	protein targeting to ER
GO:0072599	3.87646818659544e-29	62.3551587301587	0.601480866376029
22	106	establishment of protein localization to endoplasmic reticulum
GO:0006415	6.93494022351022e-29	70.3229508196721	0.516365649436025
21	91	translational termination
GO:0006413	9.992261528195e-29	46.0377155172414	0.862500864992042
24	152	translational initiation
GO:0070972	5.04596411503333e-28	54.5149305555556	0.669573039928033
22	118	protein localization to endoplasmic reticulum
GO:0019080	3.82882451442477e-27	43.3716896186441	0.856826517196042

23	151	viral genome expression
GO:0019083	3.82882451442477e-27	43.3716896186441	0.85682651719604 2
23	151	viral transcription
GO:0072594	4.65097648153165e-27	34.7691855336666	1.15756695038406
25	204	establishment of protein localization to organelle
GO:0000184	3.24271177524783e-26	50.132318501171 0.675247387724033	21
119	nuclear-transcribed mRNA catabolic process, nonsense-mediated decay
GO:0006612	2.19839305005828e-25	39.8519083969466	0.868175212788042
22	153	protein targeting to membrane
GO:0019058	3.33679887462828e-24	28.3098450774613	1.31077434087606
24	231	viral infectious cycle
GO:0043624	8.56702202828602e-24	37.1307749627422	0.868175212788042
21	153	cellular protein complex disassembly
GO:0043241	1.72967910578967e-23	35.7630728730406	0.896546951768044
21	158	protein complex disassembly
GO:0022415	5.86835954597837e-23	24.7802454704851	1.47533042696007
24	260	viral reproductive process
GO:0000956	7.47061096541792e-23	33.0794195835179	0.958964777524047
21	169	nuclear-transcribed mRNA catabolic process
GO:0034623	1.40371466395077e-22	31.9871423979428	0.987336516504048
21	174	cellular macromolecular complex disassembly
GO:0006402	2.29223431219755e-22	31.1634123420695	1.01003390768805
21	178	mRNA catabolic process
GO:0032984	2.58632388693747e-22	30.9639966798091	1.01570825548405
21	179	macromolecular complex disassembly
GO:0006401	4.71128993918159e-21	26.5400035637919	1.16324129818006
21	205	RNA catabolic process
GO:0016032	1.15092522315007e-19	15.2329411764706	2.55913085599612
26	451	viral reproduction
GO:0071845	4.33331843302748e-18	18.4645016518108	1.61151477406408
21	284	cellular component disassembly at cellular level
GO:0033365	5.3770560060619e-18	13.6287985588972	2.68396650750813
25	473	protein localization to organelle
GO:0006605	5.65427916447554e-18	13.597468057672 2.68964085530413	25
474	protein targeting
GO:0022411	5.7800394069971e-18	18.1827224166513	1.63421216524808
21	288	cellular component disassembly
Tissue: Artery_Tibial=>Adipose_Subcutaneous	Type:	asymmetric
SourceGene:	CHCHD2(ENSG00000106153.12)
TargetGeneSet:	PARK7	RPL11	RP4	NDUFS5	SNRPE	ACP1	TPRKB	RPL31	MTX2
RPL32	RNF7	RPL35A	NDUFS4	MRPS36	TAF9	BTF3	TBCA	RPS23	COX7C
CETN3	CTD	HINT1	RPS14	CTB	C6orf130		RHAG	RWDD1	RP11
SEC61G	ECD	POP7	NDUFA5	RPS20	SNHG6	UQCRB	RPL30	NDUFB9	DCTN3
SEC61B	RPL35	COMMD3	VDAC2	C11orf10		ATP5L	RPS25	PHB2	PFDN5
DIABLO	ARL6IP4	MZT1	CGRRF1	RPS17L	SEC11A	NDUFAB1	SPAG7	RPS7P1	RPL17
FBXO47	RPL19	RPL27	ATP5H	ARL16	RPL36	CHST8	COX6B1	EIF3K	SNRPD2
ATP5J	RPL18AP2		POLR2F	RBM3
GOBPID	Pvalue	OddsRatio	ExpCount	Count	Size	Term
GO:0006614	7.03976132543265e-25	68.8195669607057	0.443221922358314
18	105	SRP-dependent cotranslational protein targeting to membrane
GO:0006613	8.45565769191813e-25	68.0327695560254	0.447443083523632
18	106	cotranslational protein targeting to membrane
GO:0045047	8.45565769191813e-25	68.0327695560254	0.447443083523632
18	106	protein targeting to ER
GO:0072599	8.45565769191813e-25	68.0327695560254	0.447443083523632
18	106	establishment of protein localization to endoplasmic reticulum
GO:0070972	6.63760362954319e-24	59.8186046511628	0.498097017507439
18	118	protein localization to endoplasmic reticulum
GO:0006612	8.92627169749687e-22	44.2015503875969	0.645837658293544
18	153	protein targeting to membrane
GO:0072594	5.29130411215114e-21	34.7355212355212	0.861116877724725
19	204	establishment of protein localization to organelle
GO:0006415	1.62079772068301e-20	61.4159038901602	0.384125666043872

15	91	translational termination
GO:0006414	1.57744928897971e-19	51.8115942028985	0.443221922358314
15	105	translational elongation
GO:0000184	1.12470471296061e-18	44.7930602006689	0.502318178672756
15	119	nuclear-transcribed mRNA catabolic process, nonsense-mediated decay
GO:0019080	1.21742447933262e-18	37.5440329218107	0.637395335962909
16	151	viral genome expression
GO:0019083	1.21742447933262e-18	37.5440329218107	0.637395335962909
16	151	viral transcription
GO:0006413	1.35662886591628e-18	37.2653594771242	0.641616497128226
16	152	translational initiation
GO:0043624	5.43998896255014e-17	33.6767485822306	0.645837658293544
15	153	cellular protein complex disassembly
GO:0043241	8.88382813665698e-17	32.4878382487078	0.66694346412013
15	158	protein complex disassembly
GO:0000956	2.46953791183705e-16	30.1439864483343	0.71337623693862
15	169	nuclear-transcribed mRNA catabolic process
GO:0034623	3.83904766757613e-16	29.1858080393765	0.734482042765207
15	174	cellular macromolecular complex disassembly
GO:0006402	5.41099421018376e-16	28.4615897572686	0.751366687426476
15	178	mRNA catabolic process
GO:0032984	5.8881056306774e-16	28.2860551431601	0.755587848591793
15	179	macromolecular complex disassembly
GO:0019058	1.15687302256038e-15	23.4418604651163	0.975088229188292
16	231	viral infectious cycle
GO:0006401	4.50225390318053e-15	24.370709382151 0.865338038890042	15
205	RNA catabolic process
GO:0022415	7.47946083592459e-15	20.6134790528233	1.09750190298249
16	260	viral reproductive process
GO:0033365	3.54667723281708e-14	13.8863016572268	1.99660923119507
19	473	protein localization to organelle
GO:0006605	3.6842870520675e-14	13.8547880690738	2.00083039236039
19	474	protein targeting
GO:0022904	1.96361923406354e-13	37.0247058823529	0.405231471870459
11	96	respiratory electron transport chain
GO:0071845	5.46390082962498e-13	17.1177468886375	1.19880977095011
15	284	cellular component disassembly at cellular level
GO:0022411	6.69346400676617e-13	16.8621595795509	1.21569441561138
15	288	cellular component disassembly
GO:0016032	2.84394503268349e-12	12.424172601592 1.90374368555809	17
451	viral reproduction
GO:0022900	8.10682903244319e-12	25.5182113821138	0.565635596152515
11	134	electron transport chain
GO:0045333	1.53232386652478e-11	23.9464122137405	0.599404885475054
11	142	cellular respiration
GO:0015980	4.68549160853122e-09	11.7011414735386	1.2958964777524 12
307	energy derivation by oxidation of organic compounds
GO:0042773	4.12663652303786e-08	38.1791574279379	0.198394574769912
6	47	ATP synthesis coupled electron transport
GO:0042775	4.12663652303786e-08	38.1791574279379	0.198394574769912
6	47	mitochondrial ATP synthesis coupled electron transport
GO:0006119	1.50061983272357e-07	30.0797202797203	0.244827347588402
6	58	oxidative phosphorylation
GO:0006091	1.82777171040905e-07	8.22645211930926	1.8066569787558 12
428	generation of precursor metabolites and energy
GO:0006120	3.8658007803264e-07	41.3565668202765	0.151961801951422
5	36	mitochondrial electron transport, NADH to ubiquinone
GO:0000028	1.4185070319737e-06	248.051724137931	0.0253269669919037
3	6	ribosomal small subunit assembly
GO:0022618	2.85536039820472e-06	17.529315628192 0.401010310705142	6
95	ribonucleoprotein complex assembly
GO:0071826	3.85292758948488e-06	16.5911025145068	0.422116116531728
6	100	ribonucleoprotein complex subunit organization

GO:0022613 4.41542322260067e-06 9.85862098947918 0.949761262196388
8 225 ribonucleoprotein complex biogenesis
GO:0071843 6.88629834733271e-06 9.25198072367884 1.00885751851083
8 239 cellular component biogenesis at cellular level
GO:0042776 1.98613510017866e-05 74.3793103448276 0.0548750951491246
3 13 mitochondrial ATP synthesis coupled proton transport
Tissue: Artery_Tibial=>Adipose_Subcutaneous Type: cluster
SourceGene: ENSG00000233280.1
TargetGeneSet: RP1 RPL22 VPS13D DDI2 HSPG2 SYF2 PPP1R8 RP4 MFI2
PTPRF RPS8 RPS15AP10 BTF3L4 GNG5 CCBL2 RPL5 BCAS2 MRPS21
LCE2C ASH1L SSR2 F11R TKT PRRC2C ABL2 RP11 CEP350 RABIF
C1orf31 ACP1 RPS7 EIF2AK1 PRKD3 COX7A2L RHOQ UBC REL USP34 TET3
RANBP2 MMADHC ACVR1C MLK7-AS1 MTX2 COQ10B NBEAL1 EFHD1 GIGYF2
SETD5 RPL32 NR2C2 UBE2E1 RPL15 HIGD1A RAD54L2 CTD EBLN2 CRYBG3 PCNP
RPL24 RNF7 COMMD2 PDCD10 PHC3 DNAJC19 RPL35A RELL1 OCIAD1 COPS4
HSP90AB3P AIMP1 RPL34 OSMR NDUFAF2 MRPS36 TAF9 BTF3 TBCA
CETN3 ERAP1 HINT1 AFF4 SKP1 UBE2B CAMLG CTB UBE2D2 CD14
PCDHGB3 ADRA1B SLC2A3P1 PHACTR1 NOL7 HLA-H PGBD1 RNF5 RPS10
RPL10A WDR11 ASF1A RP3 IGF2R ADCY1 BAIAP2L1 TRRAP TRIP6 CUX1
BCAP29 MLL3 DCTN6 HOOK3 FNTA POLR2K UBR5 MED30 TRAPPC9 EIF2C2
HEATR7A C9orf82 TLN1 CLTA CBWD3 ATP6V1G1 PSMB7 SETX RPL7A
COMMD3 RPS24 GVINP2 RPL27A SBF2 TSG101 PPP6R3 NUMA1 CWC15 UBE4A
RPS25 C12orf57 H2AFJ SCAF11 MLL4 PFDN5 COPZ1 SMARCC2 BAZ2A
STAT6 LRP1 RBMS1P1 HELB RAP1B KITLG SSH1 MLXIP DGKH ESD MZT1
DCUN1D2-AS2 CCNB1IP1 PPP2R3C QRSL1P3 TRAPPC6B CGRRF1 DNAL1
DYNC1H1 CYFIP1 GANC RPS3AP6 DENND4A RAB11A MORF4L1 KIAA1199 SEC11A SELS
TSC2 SRRM2 RPS15A SRCAP CNOT1 CFDP1 MON1B HSBP1 VPS53 MINK1
CCDC144B RPL23A MYO18A TAOK1 RPL19 CDK12 RPL27 NSFP1 SUPT4H1
PRKCA PGS1 RP13 MYL12A PSMG2 C18orf32 CD209 BRD4 ZNF573
SIPA1L3 EIF3K EID2B CYP2A6 DEDD2 MEGF8 SNRPD2 RPL18 NUCB1 RPS9
ZNF587 RPS5 RPL12P4 SON PI4KA RPL9P7 USP9X UXT KDM5C HUWE1
GNL3L RBM41 RPL39 HCFC1 RPL10
GOBPID Pvalue OddsRatio ExpCount Count Size Term
GO:0006614 3.60452441375796e-28 31.2839294463498 1.33693170022836
27 105 SRP-dependent cotranslational protein targeting to membrane
GO:0006613 4.78547717282206e-28 30.8857534467468 1.34966438308768
27 106 cotranslational protein targeting to membrane
GO:0045047 4.78547717282206e-28 30.8857534467468 1.34966438308768
27 106 protein targeting to ER
GO:0072599 4.78547717282206e-28 30.8857534467468 1.34966438308768
27 106 establishment of protein localization to endoplasmic reticulum
GO:0006415 6.14875651840199e-27 33.8312368972746 1.15867414019791
25 91 translational termination
GO:0070972 1.13952422292122e-26 26.7902288794009 1.50245657739949
27 118 protein localization to endoplasmic reticulum
GO:0006414 3.22049758618174e-25 27.8832547169811 1.33693170022836
25 105 translational elongation
GO:0000184 9.40671726302102e-24 23.707011909541 1.51518926025881 25
119 nuclear-transcribed mRNA catabolic process, nonsense-mediated decay
GO:0019080 1.3272647340486e-23 19.614803780563 1.92263511175697 27
151 viral genome expression
GO:0019083 1.3272647340486e-23 19.614803780563 1.92263511175697 27
151 viral transcription
GO:0006413 1.59690271830262e-23 19.4565095541401 1.93536779461629
27 152 translational initiation
GO:0006612 1.9186088076088e-23 19.3007279344859 1.94810047747561
27 153 protein targeting to membrane
GO:0072594 2.34653730682892e-22 15.0661013824885 2.59746730330081
29 204 establishment of protein localization to organelle
GO:0019058 6.19337934755397e-22 13.632486916069 2.94124974050239 30
231 viral infectious cycle
GO:0022415 1.61670274422966e-21 12.4205268715929 3.3104975434226 31
260 viral reproductive process

GO:0006401 4.01545056268925e-21 14.2879907286687 2.61019998616013
28 205 RNA catabolic process
GO:0000956 5.13191516470086e-21 16.253164556962 2.15182340322469 26
169 nuclear-transcribed mRNA catabolic process
GO:0043624 6.6319908939653e-21 17.3680719339623 1.94810047747561
25 153 cellular protein complex disassembly
GO:0034623 1.10404650952067e-20 15.6985118029422 2.21548681752128
26 174 cellular macromolecular complex disassembly
GO:0043241 1.50542847107781e-20 16.709225894926 2.0117638917722 25 158
protein complex disassembly
GO:0006402 2.00110659828975e-20 15.2810626249167 2.26641754895855
26 178 mRNA catabolic process
GO:0032984 2.31627685820607e-20 15.1801108629106 2.27915023181787
26 179 macromolecular complex disassembly
GO:0016032 5.33743806455075e-19 8.11904916965158 5.74243996955228
36 451 viral reproduction
GO:0071845 2.72366671185017e-18 10.2807843137255 3.61608193204622
29 284 cellular component disassembly at cellular level
GO:0022411 4.00080715567273e-18 10.1191181965376 3.6670126634835 29
288 cellular component disassembly
GO:0033365 1.94130986388419e-17 7.41649045386289 6.02255899245727
35 473 protein localization to organelle
GO:0006605 1.5298532013436e-16 7.123 6.03529167531659 34 474
protein targeting
GO:0016570 1.24741135191901e-06 5.05881079520854 3.32323022628192
15 261 histone modification
GO:0016569 1.65689785694498e-06 4.93624964778811 3.39962632343782
15 267 covalent chromatin modification
GO:0016568 1.68963740738602e-06 3.99201515151515 5.33499411805411
19 419 chromatin modification
GO:0071843 1.18315312369579e-05 4.72320550639135 3.04311120337693
13 239 cellular component biogenesis at cellular level
GO:0006511 1.70610103794359e-05 3.80925013683634 4.63469656079164
16 364 ubiquitin-dependent protein catabolic process
Tissue: Artery_Tibial=>Adipose_Subcutaneous Type: asymmetric
SourceGene: HIST1H2BI(ENSG00000168242.3)
TargetGeneSet: STK40 RP4 RP11 HIPK1 MRPS21 BATF3 OR7E62P RPL31
RPL32 RPL35A RPL34 UBE2QL1 JARID2-AS1 FOXP4 RP1 MRPS12 YWHAZP6
VPS13A RPL12 ATP5L RPL41 MLXIP RILPL1 ANKRD20A19P RPS15A RPL26
C17orf51 RPL17 VPS25 RPL27 ANKRD40 PIP5K1C ZNF823
GOBPID Pvalue OddsRatio ExpCount Count Size Term
GO:0006415 1.78325549682361e-16 118.065843621399 0.157428551657325
10 91 translational termination
GO:0006414 7.89882819762901e-16 100.568421052632 0.181648328835375
10 105 translational elongation
GO:0006614 7.89882819762901e-16 100.568421052632 0.181648328835375
10 105 SRP-dependent cotranslational protein targeting to membrane
GO:0006613 8.7133434798242e-16 99.5138888888889 0.183378312919521
10 106 cotranslational protein targeting to membrane
GO:0045047 8.7133434798242e-16 99.5138888888889 0.183378312919521
10 106 protein targeting to ER
GO:0072599 8.7133434798242e-16 99.5138888888889 0.183378312919521
10 106 establishment of protein localization to endoplasmic reticulum
GO:0070972 2.63560516523313e-15 88.3827160493827 0.204138121929278
10 118 protein localization to endoplasmic reticulum
GO:0000184 2.87467034349544e-15 87.565749235474 0.205868106013425 10
119 nuclear-transcribed mRNA catabolic process, nonsense-mediated decay
GO:0019080 3.28262315323143e-14 67.5413711583924 0.26122759670611
10 151 viral genome expression
GO:0019083 3.28262315323143e-14 67.5413711583924 0.26122759670611
10 151 viral transcription
GO:0006413 3.51044963663819e-14 67.0610328638498 0.262957580790257
10 152 translational initiation

GO:0006612 3.75236081255074e-14 66.5874125874126 0.264687564874403
10 153 protein targeting to membrane
GO:0043624 3.75236081255074e-14 66.5874125874126 0.264687564874403
10 153 cellular protein complex disassembly
GO:0043241 5.20125158025491e-14 64.3153153153153 0.273337485295135
10 158 protein complex disassembly
GO:0000956 1.02855993134244e-13 59.8197064989518 0.292367310220746
10 169 nuclear-transcribed mRNA catabolic process
GO:0034623 1.38113512859077e-13 57.9756097560976 0.301017230641478
10 174 cellular macromolecular complex disassembly
GO:0006402 1.73742476558946e-13 56.5793650793651 0.307937166978064
10 178 mRNA catabolic process
GO:0032984 1.83847693355907e-13 56.2406311637081 0.30966715106221
10 179 macromolecular complex disassembly
GO:0072594 6.85030496520499e-13 48.9072164948454 0.352916753165871
10 204 establishment of protein localization to organelle
GO:0006401 7.19472842217583e-13 48.6529914529915 0.354646737250017
10 205 RNA catabolic process
GO:0019058 2.37606065091673e-12 42.8506787330317 0.399626323437824
10 231 viral infectious cycle
GO:0022415 7.71088005492001e-12 37.8026666666667 0.449795861878071
10 260 viral reproductive process
GO:0071845 1.849551847592e-11 34.4330900243309 0.491315479897585
10 284 cellular component disassembly at cellular level
GO:0022411 2.12382569663949e-11 33.9280575539568 0.498235416234171
10 288 cellular component disassembly
GO:0016032 1.70492184763235e-09 21.1413454270597 0.780222821950038
10 451 viral reproduction
GO:0033365 2.69983390067445e-09 20.1051115910727 0.818282471801259
10 473 protein localization to organelle
GO:0006605 2.75534740067316e-09 20.0603448275862 0.820012455885406
10 474 protein targeting
Tissue: Artery_Tibial=>Adipose_Subcutaneous Type: asymmetric
SourceGene: MRPL13(ENSG00000172172.3)
TargetGeneSet: RP4 NDUFS5 C1orf210 ACP1 RP11 CRYBG3 RPL7AP11
PDCD10 MRPS36 CTD RP1 RPL24P4 COX7A2 NDUFA4 SEC61G ECD UQCRB
COX6C SEC61B RPL13AP5 NDUFS3 C11orf10 OR7E2P ATP5L HYLS1 IL26
VPS29 RPS7P10 CGRRF1 RDH11 ERH SLIRP C14orf2 TMEM85 RPS17L POLR3K
NPRL3 NDUFAB1 TXNDC17 RPS7P1 RPL27 ATP5G1 ATP5H ARL16 NDUFA7 PIN1
COX6B1 POLR2I ZNF569 SNRPD2 TMSB4XP6 ATP5J SNX12 COX7B TCEAL3 RP3
GOBPID Pvalue OddsRatio ExpCount Count Size Term
GO:0022904 4.60628960515308e-19 77.3734939759036 0.279011833091136
13 96 respiratory electron transport chain
GO:0022900 4.20164127634141e-17 52.9335993160445 0.389454017023043
13 134 electron transport chain
GO:0045333 9.1083609869236e-17 49.6230954290297 0.412705003113971
13 142 cellular respiration
GO:0015980 2.04719997843038e-12 21.5218156228008 0.892256591239361
13 307 energy derivation by oxidation of organic compounds
GO:0006091 1.31534330343842e-10 15.1160781055256 1.24392775586465
13 428 generation of precursor metabolites and energy
GO:0042773 4.08341038300971e-09 58.4065040650407 0.136599543284202
6 47 ATP synthesis coupled electron transport
GO:0042775 4.08341038300971e-09 58.4065040650407 0.136599543284202
6 47 mitochondrial ATP synthesis coupled electron transport
GO:0006119 1.50343407811389e-08 46.0160256410256 0.168569649159228
6 58 oxidative phosphorylation
GO:0042776 4.32334705153945e-08 168.421052631579 0.0377828523977579
4 13 mitochondrial ATP synthesis coupled proton transport
GO:0006120 5.71898886355174e-08 62.6765475152572 0.104629437409176
5 36 mitochondrial electron transport, NADH to ubiquinone
GO:0015985 1.83089273168167e-07 108.233082706767 0.0523147187045879
4 18 energy coupled proton transport, down electrochemical gradient

GO:0015986	1.83089273168167e-07	108.233082706767	0.0523147187045879
4	18	ATP synthesis coupled proton transport
GO:0006754	1.18765230172136e-05	33.6	0.142412289806934	4	49
ATP biosynthetic process
Tissue: Artery_Tibial=>Adipose_Subcutaneous	Type:	asymmetric
SourceGene:	NDUFA11(ENSG00000174886.7)
TargetGeneSet:	MINOS1	RP4	MRPL9	MIR555	SNRPE	RP11	ACP1	MORN2
SOS1-IT1	C1D	TPRKB	RPL31	HOXD-AS2	KCTD18	KRBOX1	PCNP
PDCD10	DNAJC19	RPL35A	LAMTOR3	RPL34	NDUFAF2	ISCA1P1	TAF9	COX7C	CTD	RP1
CENPQ	RPL39P3	NDUFA4	LSM5	SEC61G	SRI	ECD	NDUFA5	DCTN6	UQCRB
RPL30	COX6C	POLR2K	SEC61B	RPL35	COMMD3	RAB18	C11orf10	FAU
TMEM126A	ATP5L	RPS25	CCDC59	SNRPF	C12orf45	C14orf28	ERH
SLIRP	RPLP1	MORF4L1	SEC11A	NDUFAB1	RPL27	ICT1	PFDN4	ATP5J	EEF1B2P3
NDUFB11	TBL1Y
GOBPID	Pvalue	OddsRatio	ExpCount	Count	Size	Term
GO:0006614	1.42433965812439e-15	47.3349875930521	0.370562590824164
12	105	SRP-dependent cotranslational protein targeting to membrane
GO:0006613	1.60214729655642e-15	46.8281505728314	0.374091758355823
12	106	cotranslational protein targeting to membrane
GO:0045047	1.60214729655642e-15	46.8281505728314	0.374091758355823
12	106	protein targeting to ER
GO:0072599	1.60214729655642e-15	46.8281505728314	0.374091758355823
12	106	establishment of protein localization to endoplasmic reticulum
GO:0070972	6.02682002649471e-15	41.4920174165457	0.416441768735728
12	118	protein localization to endoplasmic reticulum
GO:0006612	1.42762418440875e-13	31.1162029459902	0.539962632343782
12	153	protein targeting to membrane
GO:0072594	1.79133086949973e-13	25.4501240011022	0.719950176458377
13	204	establishment of protein localization to organelle
GO:0006415	6.10276563851381e-13	43.1165311653117	0.321154245380943
10	91	translational termination
GO:0022904	4.15506443813974e-11	35.2536945812808	0.338800083039236
9	96	respiratory electron transport chain
GO:0006414	9.4051309650409e-11	31.9285714285714	0.370562590824164
9	105	translational elongation
GO:0019080	1.01777498621358e-10	24.665282823041	0.532904297280465	10
151	viral genome expression
GO:0019083	1.01777498621358e-10	24.665282823041	0.532904297280465	10
151	viral transcription
GO:0043624	1.15959009805225e-10	24.3169026095855	0.539962632343782
10	153	cellular protein complex disassembly
GO:0043241	1.59415400058667e-10	23.4871456822676	0.557608470002076
10	158	protein complex disassembly
GO:0000184	2.91777662601654e-10	27.8376623376623	0.419970936267386
9	119	nuclear-transcribed mRNA catabolic process, nonsense-mediated decay
GO:0000956	3.09584731097889e-10	21.8453750575242	0.596429312850322
10	169	nuclear-transcribed mRNA catabolic process
GO:0034623	4.12293794258691e-10	21.1719214753123	0.614075150508615
10	174	cellular macromolecular complex disassembly
GO:0006402	5.15243812730897e-10	20.6620209059233	0.62819182063525
10	178	mRNA catabolic process
GO:0032984	5.44313297866754e-10	20.5383172174917	0.631720988166909
10	179	macromolecular complex disassembly
GO:0022900	8.45924531024241e-10	24.4714285714286	0.472908449242267
9	134	electron transport chain
GO:0045333	1.41805383370691e-09	22.9865735767991	0.501141789495537
9	142	cellular respiration
GO:0006401	2.04070786799352e-09	17.7673545966229	0.723479343990035
10	205	RNA catabolic process
GO:0006413	2.59291329191954e-09	21.3641358641359	0.536433464812124
9	152	translational initiation
GO:0033365	6.42348114808723e-09	10.3672768878719	1.66929624247457
13	473	protein localization to organelle

GO:0019058	6.4563142589381e-09	15.6483831806644	0.815237699813162
10	231	viral infectious cycle
GO:0006605	6.58809317519468e-09	10.3440461239868	1.67282541000623
13	474	protein targeting
GO:0022415	1.99720989599994e-08	13.8048780487805	0.917583558231264
10	260	viral reproductive process
GO:0071845	4.60418442504915e-08	12.5743279330604	1.00228357899107
10	284	cellular component disassembly at cellular level
GO:0022411	5.25208351095055e-08	12.3898929636778	1.01640024911771
10	288	cellular component disassembly
GO:0015980	9.56108317800119e-08	11.5816703621582	1.08345443221922
10	307	energy derivation by oxidation of organic compounds
GO:0016032	3.73676437074146e-07	8.725	1.59165455677808	11	451
viral reproduction
GO:0006091	2.01425804648476e-06	8.15847823550006	1.51048370354993
10	428	generation of precursor metabolites and energy
Tissue: Artery_Tibial=>Adipose_Subcutaneous	Type:	asymmetric
SourceGene:	NDUFB3(ENSG00000119013.4)
TargetGeneSet:	RP11	RP1	NDUFS5	TPI1P1	RP4	PFN1P1	MIR4426	MRPS36P1
RPL7AP11	RBBP4P1	CTD	RPL24P4	COX7A2	NDUFA4	SEC61G	ECD	UQCRB
COX6C	ST13P5	NDUFS3	C11orf10	ATP5L	RPS7P10	MRPL52	C14orf2	POLR3K
NPRL3	NDUFAB1	PFN1	TXNDC17	ATP5G1	SNRPEP4	COX6B1	TMSB4XP6	ATP5J
EEF1B2P3	COX7B	RP3
GOBPID	Pvalue	OddsRatio	ExpCount	Count	Size	Term
GO:0022904	7.80090279040335e-19	154.679411764706	0.152792194311812
11	96	respiratory electron transport chain
GO:0022900	3.5316252424918e-17	106.609078590786	0.213272437893571
11	134	electron transport chain
GO:0045333	6.80627528109078e-17	100.04262086514	0.226005120752889	11
142	cellular respiration
GO:0015980	3.58734576149402e-13	43.7646396396396	0.488616704726317
11	307	energy derivation by oxidation of organic compounds
GO:0006091	1.32945171280711e-11	30.7995603517186	0.681198532973497
11	428	generation of precursor metabolites and energy
GO:0042773	9.41480564335991e-09	95.1455026455026	0.0748045117984915
5	47	ATP synthesis coupled electron transport
GO:0042775	9.41480564335991e-09	95.1455026455026	0.0748045117984915
5	47	mitochondrial ATP synthesis coupled electron transport
GO:0006119	2.78034991876831e-08	75.3406708595388	0.0923119507300533
5	58	oxidative phosphorylation
GO:0006120	2.77656681191249e-07	94.7105263157895	0.0572970728669296
4	36	mitochondrial electron transport, NADH to ubiquinone
GO:0042776	9.96830635216091e-07	216.27	0.0206906096463913	3	13
mitochondrial ATP synthesis coupled proton transport
GO:0015985	2.82937347018751e-06	144.13	0.0286485364334648	3	18
energy coupled proton transport, down electrochemical gradient
GO:0015986	2.82937347018751e-06	144.13	0.0286485364334648	3	18
ATP synthesis coupled proton transport
Tissue: Artery_Tibial=>Adipose_Subcutaneous	Type:	asymmetric
SourceGene:	NDUFB7(ENSG00000099795.1)
TargetGeneSet:	PARK7	RPL11	ZBTB8OS	RP4	NDUFS5	RPL5	ATP5F1	SIKE1
MIR4426	SNRPE	CHML	ACP1	RPS7	CALM2	UBC	C1D	SNRPG	FAM136A
TPRKB	TXNDC9	RPL31	MZT2B	MTX2	MOB4	RPL32	LSM3	UBE2E1	DLEC1	RP11
PCNP	SNX4	COMMD2	PDCD10	DNAJC19	RPL35A	COMMD8	UGT2B17	RPL34	AP1AR
ANAPC10	NDUFS4	MRPS36	TAF9	BTF3	TBCA	COX7C	CETN3	CTD	HINT1
UBE2D2	RPS14	RPL26L1	NOL7	HMGN4	RPL39P3	COX7A2	RWDD1	MRPS12	NDUFA4
GPR141	SEC61G	ECD	ACN9	NDUFA5	SSBP1	DCTN6	RPS20	SNHG6	UQCRB
RPL30	COX6C	POLR2K	NDUFB9	SEC61B	RPL35	COMMD3	ARHGAP12	RPL13AP5
FAU	TMEM126B	ATP5L	RPS25	PHB2	PFDN5	C12orf73	VPS29
ARL6IP4	RPS7P10	EIF4A1P7	HMGB1	MZT1	MRPL52	CGRRF1	ERH	SLIRP
C14orf2	ATPBD4	RPLP1	RPS17L	HOMER2	SEC11A	C16orf13	TCEB2	NDUFAB1
SPAG7	TXNDC17	RPS7P1	RPL17	RPL27	ATP5G1	ATP5H	STRA13	ZNF519	RPS15
RPL36	PIN1	POLR2I	RPS16	MEGF8	SNRPD2	RPL18	PFDN4	ATP5J	SNRPD3

POLR2F EEF1B2P3 RBM3 COX7B TCEAL4 TCEAL3 CXorf69
GOBPID Pvalue OddsRatio ExpCount Count Size Term
GO:0006614 1.28353323524086e-33 60.2025316455696 0.755657047955159
26 105 SRP-dependent cotranslational protein targeting to membrane
GO:0006613 1.69178424414832e-33 59.4458333333333 0.762853781745208
26 106 cotranslational protein targeting to membrane
GO:0045047 1.69178424414832e-33 59.4458333333333 0.762853781745208
26 106 protein targeting to ER
GO:0072599 1.69178424414832e-33 59.4458333333333 0.762853781745208
26 106 establishment of protein localization to endoplasmic reticulum
GO:0070972 3.73538629239976e-32 51.6485507246377 0.849214587225798
26 118 protein localization to endoplasmic reticulum
GO:0019080 9.68825446215926e-31 40.2200460829493 1.08670680229742
27 151 viral genome expression
GO:0019083 9.68825446215926e-31 40.2200460829493 1.08670680229742
27 151 viral transcription
GO:0006415 5.54551082225793e-30 59.6254538852578 0.654902774894471
23 91 translational termination
GO:0006612 5.49561410491831e-29 37.3228346456693 1.10110026987752
26 153 protein targeting to membrane
GO:0072594 1.6657132242262e-28 29.6641746411483 1.46813369317002
28 204 establishment of protein localization to organelle
GO:0006414 2.14506421732264e-28 49.3970189701897 0.755657047955159
23 105 translational elongation
GO:0000184 4.91311555762776e-27 42.1518775720165 0.856411321015847
23 119 nuclear-transcribed mRNA catabolic process, nonsense-mediated decay
GO:0019058 1.5065378015306e-25 24.3099694423224 1.66244550550135
27 231 viral infectious cycle
GO:0000956 8.3796949490573e-25 29.3834482758621 1.2162480105183 24
169 nuclear-transcribed mRNA catabolic process
GO:0006413 1.92604936992911e-24 31.2962005933582 1.09390353608747
23 152 translational initiation
GO:0043624 2.2545261378898e-24 31.0532763532764 1.10110026987752
23 153 cellular protein complex disassembly
GO:0006402 3.03706954557625e-24 27.6487012987013 1.28101861462875
24 178 mRNA catabolic process
GO:0022415 3.78675213310944e-24 21.2406220389053 1.87115078541277
27 260 viral reproductive process
GO:0043241 4.87136289438155e-24 29.8926383173297 1.13708393882776
23 158 protein complex disassembly
GO:0034623 4.82812957457821e-23 26.6951189600196 1.25223167946855
23 174 cellular macromolecular complex disassembly
GO:0032984 9.42424685329472e-23 25.8304051915163 1.28821534841879
23 179 macromolecular complex disassembly
GO:0006401 9.72753108292477e-23 23.4795580110497 1.47533042696007
24 205 RNA catabolic process
GO:0071845 2.36747278556812e-19 16.2542307692308 2.04387239637395
24 284 cellular component disassembly at cellular level
GO:0022411 3.28741623876774e-19 16.0034090909091 2.07265933153415
24 288 cellular component disassembly
GO:0016032 6.07495664728637e-19 12.127410725395 3.24572693931216 28
451 viral reproduction
GO:0033365 2.13944444062467e-18 11.5096392667061 3.40405508269324
28 473 protein localization to organelle
GO:0006605 2.26187252998951e-18 11.4830068444654 3.41125181648329
28 474 protein targeting
GO:0022904 2.05207720126966e-16 29.6837286724927 0.690886443844717
15 96 respiratory electron transport chain
GO:0022900 3.43762193935482e-14 20.1510716646209 0.964362327866584
15 134 electron transport chain
GO:0045333 8.21109615471839e-14 18.8710961691586 1.02193619818698
15 142 cellular respiration
GO:0022613 6.72834175581413e-11 11.3459069020867 1.61926510276105

15	225	ribonucleoprotein complex biogenesis
GO:0071843	1.57751916069337e-10	10.6262540128411	1.72001937582174
15	239	cellular component biogenesis at cellular level
GO:0015980	5.73780055812087e-10	8.78225554514214	2.20939727354508
16	307	energy derivation by oxidation of organic compounds
GO:0042274	2.07863538610071e-09	73.1377551020408	0.129541208220884
6	18	ribosomal small subunit biogenesis
GO:0042776	2.15422177732748e-08	90.5239898989899	0.0935575392706387
5	13	mitochondrial ATP synthesis coupled proton transport
GO:0042773	4.0483575735676e-08	25.8115979381443	0.338246488132309
7	47	ATP synthesis coupled electron transport
GO:0042775	4.0483575735676e-08	25.8115979381443	0.338246488132309
7	47	mitochondrial ATP synthesis coupled electron transport
GO:0006364	5.6838232291922e-08	14.2125207756233	0.74846031416511
9	104	rRNA processing
GO:0006091	6.45780521719966e-08	6.14960282436011	3.08020206214103
16	428	generation of precursor metabolites and energy
GO:0016072	9.26968797246056e-08	13.3625846795206	0.791640716905404
9	110	rRNA metabolic process
GO:0042254	1.1436294305676e-07	10.8740241849074	1.07231333471732
10	149	ribosome biogenesis
GO:0034660	1.2797969306365e-07	7.39233193277311	2.051069130164	13
285	ncRNA metabolic process
GO:0015985	1.39369542087303e-07	55.6876456876457	0.129541208220884
5	18	energy coupled proton transport, down electrochemical gradient
GO:0015986	1.39369542087303e-07	55.6876456876457	0.129541208220884
5	18	ATP synthesis coupled proton transport
GO:0006119	1.81414863204859e-07	20.2288255508389	0.41741055982285
7	58	oxidative phosphorylation
GO:0006120	1.96388879581855e-07	29.2183673469388	0.259082416441769
6	36	mitochondrial electron transport, NADH to ubiquinone
GO:0022618	3.97268581095797e-07	13.6590038314176	0.683689710054667
8	95	ribonucleoprotein complex assembly
GO:0071826	5.90465807038686e-07	12.9121376811594	0.719673379004913
8	100	ribonucleoprotein complex subunit organization
GO:0000387	2.56590481848436e-06	27.8185703185703	0.223098747491523
5	31	spliceosomal snRNP assembly
GO:0000028	7.12935624986512e-06	142.019801980198	0.0431804027402948
3	6	ribosomal small subunit assembly
GO:0015992	7.6944232837172e-06	14.5785714285714	0.474984430143243
6	66	proton transport
GO:0006818	9.16205574648414e-06	14.1063199473338	0.489377897723341
6	68	hydrogen transport
GO:0006839	9.78099672067121e-06	10.6015517058136	0.74846031416511
7	104	mitochondrial transport
GO:0034470	1.37541921510622e-05	7.02143841278589	1.43934675800983
9	200	ncRNA processing
GO:0000377	1.61104282598361e-05	6.87546558704453	1.46813369317002
9	204	RNA splicing, via transesterification reactions with bulged adenosine as nucleophile
GO:0000398	1.61104282598361e-05	6.87546558704453	1.46813369317002
9	204	nuclear mRNA splicing, via spliceosome
GO:0000375	1.95323648034354e-05	6.70121052631579	1.50411736212027
9	209	RNA splicing, via transesterification reactions
Tissue: Adipose_Subcutaneous=>Artery_Tibial	Type:	asymmetric
SourceGene:	NDUFS5(ENSG00000168653.6)
TargetGeneSet:	AURKAIP1	MRPL20	PARK7	MECR	NDUFS5	PPCS	ERI3
UQCRH	HBXIP	ATP5F1	SDHC	TMCO1	SNRPE	C1orf31	SLC4A1AP	COMMD1	MDH1
SNRPG	PRADC1	TPRKB	BOLA3	FAM138B	TSN	ATP5G3	MTX2	NDUFB3	AP1S3
MYEOV2	ARPC4	LSM3	CMC1	CCDC72	SPCS1	ATG3	RNF7	MRPL47	NDUFB5
MRPL1	LAMTOR3	SETP20	LARP7	EXOSC9	NDUFC1	SCOC	LSM6	PDCD6	NDUFS6
C5orf44	ANKRD32	CAST	GIN1	MAN2A1	HINT1	UQCRQ	PDF	ATOX1	CTB
EEF1E1	HLA-F-AS1	PFDN6	HNRNPC	MEA1	COX7A2	COQ3	RTN4IP1	SF3B5	LSM5

MPLKIP SEC61G CHCHD2 STAG3L4 PMS2P1 RP11 LSM1 TCEB1 MRPS35 DECR1
MRPL13 CTA CTD C9orf123 ACER2 NDUFB6 DCTN3 SEC61B ATP6V1G1
COQ4 TMEM141 PRPF18 ECHS1 POLR2L MRPL23 DENND5A SBF2 IMMP1L NDUFS3
TIMM10 C11orf10 FIBP BCRP1 MRPL11 NDUFS8 MRPL21 MRPL48 CLNS1A
TMEM126A SDHD ATP5L MRPL51 COPS7A MAGOHB TFCP2 NDUFA12 SNRPF
NUP37 TRIAP1 POP5 DENR EFHA1 MED4 MRPL52 SCFD1 C14orf126
ARHGAP5-AS1 C14orf166 ACTR10 COX16 SLIRP NDUFB1 CINP C14orf2
ATPBD4 GTF2A2 RAB11A COX5A MRPS11 IGF1R MRPL28 NDUFAB1 COX4NB PSMB6
TXNDC17 SMCR8 STAT5B VPS25 ATP5G1 MRPL27 NACA2 ICT1 RNF213 C17orf89
SNRPD1 IER3IP1 UQCR11 TIMM13 MRPL54 C19orf70 PIN1 MRPL4 NDUFB7
C19orf42 NDUFA13 POP4 COX6B1 TBCB HSPBP1 SLC27A5 ITPA COMMD7
ATP5E ATP5J SNRPD3 TXN2 PDZD11 COX7B NDUFA1 NAA10

GOBPID Pvalue OddsRatio ExpCount Count Size Term
GO:0022904 3.18715826637257e-32 46.8118377785897 0.969898276935852
27 96 respiratory electron transport chain
GO:0022900 2.10603152505072e-29 31.7854173329069 1.35381634488963
28 134 electron transport chain
GO:0045333 1.20422694257494e-28 29.5382099316087 1.43464120130095
28 142 cellular respiration
GO:0015980 2.98979089463873e-20 12.5063948840927 3.10165386478444
29 307 energy derivation by oxidation of organic compounds
GO:0042773 1.57166228753119e-17 45.8696051423324 0.474846031416511
14 47 ATP synthesis coupled electron transport
GO:0042775 1.57166228753119e-17 45.8696051423324 0.474846031416511
14 47 mitochondrial ATP synthesis coupled electron transport
GO:0006091 2.86164959095461e-17 9.03677872119217 4.32412981800567
30 428 generation of precursor metabolites and energy
GO:0006119 4.24587644473892e-16 34.3756887052342 0.585980208982077
14 58 oxidative phosphorylation
GO:0006120 7.27037195699802e-16 53.2873134328358 0.363711853850945
12 36 mitochondrial electron transport, NADH to ubiquinone
GO:0015985 1.61144132962743e-08 51.0464285714286 0.181855926925472
6 18 energy coupled proton transport, down electrochemical gradient
GO:0015986 1.61144132962743e-08 51.0464285714286 0.181855926925472
6 18 ATP synthesis coupled proton transport
GO:0000387 1.99629258587082e-08 29.9661270983213 0.313196318593869
7 31 spliceosomal snRNP assembly
GO:0042776 1.18505858352551e-07 63.3732269503546 0.131340391668397
5 13 mitochondrial ATP synthesis coupled proton transport
GO:0008380 1.68507574076243e-07 5.61182660987221 3.26330357760709
16 323 RNA splicing
GO:0006397 5.73525880711992e-07 4.80317788871302 4.03113971351464
17 399 mRNA processing
GO:0000375 1.37645706149734e-06 6.41321312220623 2.11154937374576
12 209 RNA splicing, via transesterification reactions
GO:0015992 4.40188110241071e-06 12.1597366174857 0.666805065393398
7 66 proton transport
GO:0008334 4.43650775997925e-06 25.3280141843972 0.252577676285378
5 25 histone mRNA metabolic process
GO:0006818 5.38804658805275e-06 11.7594055902819 0.687011279496229
7 68 hydrogen transport
GO:0000291 5.44830224774408e-06 24.1202296521445 0.262680783336793
5 26 nuclear-transcribed mRNA catabolic process, exonucleolytic
GO:0043928 5.44830224774408e-06 24.1202296521445 0.262680783336793
5 26 exonucleolytic nuclear-transcribed mRNA catabolic process involved in deadenylation-dependent decay
GO:0000377 7.03630480951238e-06 5.95785837651123 2.06103383848869
11 204 RNA splicing, via transesterification reactions with bulged adenosine as nucleophile
GO:0000398 7.03630480951238e-06 5.95785837651123 2.06103383848869
11 204 nuclear mRNA splicing, via spliceosome
GO:0006754 9.38036671582839e-06 14.2146179401993 0.495052245519341
6 49 ATP biosynthetic process

GO:0006839	1.01830676064649e-05	8.58031400966184	1.05072313334717
8	104	mitochondrial transport
Tissue: Artery_Tibial=>Adipose_Subcutaneous	Type:	asymmetric
SourceGene:	PIN1(ENSG00000127445.7)
TargetGeneSet:	RPL22	VPS13D	RPL11	SYF2	PRDX3P2	KHDRBS1	RP4	RPS8	RPL5
RP11	SNX7	ATP5F1	SNRPE	ZNF672	ACP1	YWHAQ	TTC32	COX7A2L	UBC
PPP3R1	RPL31	MMADHC	MTX2	KCTD18	RPL15	GORASP1	HIGD1A	GRM2	PCNP
ITGB5-AS1	PDCD10	PARL	RPL35A	OCIAD1	HSD17B11	RPL34	ANAPC10
RPS3A	C4orf46	NDUFS4	MRPS36	TAF9	FCHO2	BTF3	TBCA	RPS23	CTD	SNX2
UBE2D2	NDFIP1	RPS14	TDP2	RPS10	RPL10A	C6orf130	RWDD1	MRPS12
C6orf72	BTF3P10	NDUFA5	SSBP1	DCTN6	RPL30	VPS13B	POLR2K	EIF3H	CLTA
SLC35D2	RPL35	RAB18	VN1R53P	AGAP6	VDAC2	RPS24	SFR1	RPL27A	C11orf58
FAU	RPS25	PFDN5	YEATS4	C12orf45	MLXIP	POLR1D	DGKH	TPT1
RSL24D1	RPS3AP6	RAB11A	MORF4L1	SEC11A	SRRM2	VPS53	MINK1	GABARAP	RPL19
RAPGEFL1	RPL27	RNF213	FOXK2	PSMG2	RNF138	RPL17	ABCA7	RPS15
RPL36	BRD4	RPL18	ZNF667	SLC4A11	PFDN4	PI4KA	MORC2	POLR2F	KDM5C
HUWE1	RPL10	VAMP7
GOBPID	Pvalue	OddsRatio	ExpCount	Count	Size	Term
GO:0006415	1.82968058331037e-37	79.2902960526316	0.648605632828178
27	91	translational termination
GO:0006614	2.2625275620165e-37	69.1927272727273	0.748391114801744
28	105	SRP-dependent cotranslational protein targeting to membrane
GO:0006613	3.05920692959468e-37	68.3008547008547	0.755518649228427
28	106	cotranslational protein targeting to membrane
GO:0045047	3.05920692959468e-37	68.3008547008547	0.755518649228427
28	106	protein targeting to ER
GO:0072599	3.05920692959468e-37	68.3008547008547	0.755518649228427
28	106	establishment of protein localization to endoplasmic reticulum
GO:0070972	8.96793570318084e-36	59.1442962962963	0.841049062348626
28	118	protein localization to endoplasmic reticulum
GO:0006414	1.52646240815652e-35	64.9949392712551	0.748391114801744
27	105	translational elongation
GO:0019080	3.81709586088656e-34	45.6971643774922	1.07625769842917
29	151	viral genome expression
GO:0019083	3.81709586088656e-34	45.6971643774922	1.07625769842917
29	151	viral transcription
GO:0000184	6.66852194647906e-34	55.0503432494279	0.84817659677531
27	119	nuclear-transcribed mRNA catabolic process, nonsense-mediated decay
GO:0006413	2.08450014353447e-32	42.8249462365591	1.08338523285586
28	152	translational initiation
GO:0006612	2.53852081156221e-32	42.47936	1.09051276728254	28
153	protein targeting to membrane
GO:0043624	1.05713437487409e-30	40.0996240601504	1.09051276728254
27	153	cellular protein complex disassembly
GO:0043241	2.665009726269e-30	38.5555443953395	1.12615043941596
27	158	protein complex disassembly
GO:0072594	3.94930482849469e-30	31.7387644787645	1.45401702304339
29	204	establishment of protein localization to organelle
GO:0000956	1.82349348747595e-29	35.5413269088213	1.20455331810947
27	169	nuclear-transcribed mRNA catabolic process
GO:0034623	4.17424947541628e-29	34.3203544575725	1.24019099024289
27	174	cellular macromolecular complex disassembly
GO:0006402	7.94469852801563e-29	33.4017950505403	1.26870112794962
27	178	mRNA catabolic process
GO:0032984	9.30795624462891e-29	33.1797091412742	1.27582866237631
27	179	macromolecular complex disassembly
GO:0019058	1.63632534474771e-28	27.4440727856569	1.64646045256384
29	231	viral infectious cycle
GO:0006401	4.17105803182987e-27	28.2813424009462	1.46114455747007
27	205	RNA catabolic process
GO:0022415	5.39847106719936e-27	23.9495144495144	1.85315895093765
29	260	viral reproductive process
GO:0071845	3.08334812511755e-23	19.4786504198239	2.02421977717805

27	284	cellular component disassembly at cellular level
GO:0022411	4.48730900500021e-23	19.1746823956443	2.05272991488478
27	288	cellular component disassembly
GO:0006605	8.29034726793334e-22	13.5143905191874	3.37845131824787
31	474	protein targeting
GO:0016032	2.66158111989196e-21	13.5948329157583	3.21451802643416
30	451	viral reproduction
GO:0033365	1.32816501122266e-19	12.2722181641101	3.37132378382119
29	473	protein localization to organelle
GO:0042274	1.32769017726668e-07	56.2598116169545	0.128295619680299
5	18	ribosomal small subunit biogenesis
GO:0022613	5.79743579126563e-07	7.89689150751727	1.60369524600374
11	225	ribonucleoprotein complex biogenesis
GO:0071843	1.05259489543742e-06	7.40465293668955	1.7034807279773 11
239	cellular component biogenesis at cellular level
GO:0042254	1.13787450749353e-06	9.71671732522796	1.06200262957581
9	149	ribosome biogenesis
GO:0006364	9.17638429143643e-06	10.7127362542955	0.741263580375061
7	104	rRNA processing
GO:0030490	1.20555222457629e-05	107.58	0.0498927409867829	3	7
maturation of SSU-rRNA
GO:0016072	1.32749181066227e-05	10.0844457928803	0.78402878693516
7	110	rRNA metabolic process
Tissue: Artery_Tibial=>Adipose_Subcutaneous	Type:	asymmetric
SourceGene:	PPP1R35(ENSG00000160813.2)
TargetGeneSet:	IL28RA	RAB42	CTSS	C1orf85	SLAMF9	CD84	NUF2	ASPM	NEK2
ZNF692	GCC2	BUB1	HJURP	FANCD2	SGOL1	HEMK1	RP11	SKIL	NCEH1	PARL
CTBP1	CENPE	MAD2L1	NEK1	HEXB	KCNN2	KIF20A	GM2A	RGL2	TREML4
PLA2G7	IMPG1	NDUFAF4	HSF2	BLVRA	CHMP7	DNAJC5B	SCRIB	MELK	FBP1
IKBKAP	DAB2IP	ZNF248	CEP55	CTBP2	MKI67	DDB2	RBM14	MPP2	TCTN2
SLC7A8	DLGAP5	NPC2	BUB1B	CASC5	NUSAP1	CCNB2	APH1B	PRC1	ADCY9
GLIS2	ASPHD1	ORAI3	ORC6	ELMO3	DPEP2	CDT1	AURKB	EVI2B	CCT6B
TOP2A	KIF18B	QRICH2	BIRC5	RP13	PSTPIP2	SKA1	MUM1	ACP5	ASF1B	TPX2
MYBL2	UBE2C	C21orf59		SOWAHD
GOBPID	Pvalue	OddsRatio	ExpCount	Count	Size	Term
GO:0000087	8.11545192707253e-18	16.610650887574	1.91820635250156	22
360	M phase of mitotic cell cycle
GO:0000280	7.48784246976667e-17	16.0087386018237	1.86492284270985
21	350	nuclear division
GO:0007067	7.48784246976667e-17	16.0087386018237	1.86492284270985
21	350	mitosis
GO:0048285	2.30635355685262e-16	15.0698424068768	1.97148986229327
21	370	organelle fission
GO:0051301	9.84499542895747e-16	12.9711627906977	2.40841464258529
22	452	cell division
GO:0007059	1.65357408919032e-14	25.1287477954145	0.745969137083939
14	140	chromosome segregation
GO:0000236	2.29063332390665e-11	28.4557213930348	0.452909833229534
10	85	mitotic prometaphase
GO:0007017	1.98673818066692e-10	9.7383136930904	2.0940419348142	16	393
microtubule-based process
GO:0010564	4.26290201811313e-10	9.21064338083862	2.20593730537679
16	414	regulation of cell cycle process
GO:0000226	1.53348202076876e-09	11.2020263671875	1.433326413397	13
269	microtubule cytoskeleton organization
GO:0007346	6.32103781543842e-08	8.93449643140365	1.61449034668881
12	303	regulation of mitotic cell cycle
GO:0031577	8.45574181198128e-08	33.6572769953052	0.223790741125182
6	42	spindle checkpoint
GO:0071156	1.41374166330406e-07	9.30237154150198	1.40668465850114
11	264	regulation of cell cycle arrest
GO:0007051	2.14855989955282e-07	19.5904109589041	0.426268078333679
7	80	spindle organization

GO:0000819 3.14610820744488e-07 26.3221065523576 0.277074250916892
6 52 sister chromatid segregation
GO:0007088 4.15025086482453e-07 17.6456790123457 0.468894886167047
7 88 regulation of mitosis
GO:0051783 4.15025086482453e-07 17.6456790123457 0.468894886167047
7 88 regulation of nuclear division
GO:0045786 4.67338759798248e-07 6.68300412735849 2.32848937789772
13 437 negative regulation of cell cycle
GO:0007050 5.30062805352234e-07 7.2486102133161 1.9661615113141 12 369
cell cycle arrest
GO:0000075 5.91210608297621e-07 9.09804426145136 1.28946093695938
10 242 cell cycle checkpoint
GO:0071173 9.28542025236463e-07 34.3510536398467 0.181163933291814
5 34 spindle assembly checkpoint
GO:0034453 1.07880770747736e-06 33.2037037037037 0.186492284270985
5 35 microtubule anchoring
GO:0008608 1.68232414067792e-06 60.5310853530032 0.0905819666459069
4 17 attachment of spindle microtubules to kinetochore
GO:0051313 2.71771295428337e-06 52.4529680365297 0.101238668604249
4 19 attachment of spindle microtubules to chromosome
GO:0030071 3.09433502478164e-06 26.1988304093567 0.229119092104353
5 43 regulation of mitotic metaphase/anaphase transition
GO:0007091 3.89494661238576e-06 24.8854166666667 0.239775794062695
5 45 mitotic metaphase/anaphase transition
GO:0000070 5.9790036781241e-06 22.6167929292929 0.261089197979379
5 49 mitotic sister chromatid segregation
GO:0000910 1.05418242064569e-05 13.7189500640205 0.500864992042073
6 94 cytokinesis
GO:0000086 1.1323242897632e-05 10.3919708029197 0.767282541000623
7 144 G2/M transition of mitotic cell cycle
Tissue: Artery_Tibial=>Adipose_Subcutaneous Type: asymmetric
SourceGene: RP11-122C5.1(ENSG00000249911.1)
TargetGeneSet: RP11 RPL32 RPL15 RPL34 NKD2 BTF3 CTB RPS14
C6orf130 ARF5 RPL30 RPS6 ANP32B HIF1AN UBXN1 RPS25 PFDN5
RPL41 RPL31P52 TPT1 RPL36A SEC11A RPS15A CTD RPL26 RPL17
RPL19 MSI2 NFIC RPL36 CCDC106 RPS4X APOOL RPL10
GOBPID Pvalue OddsRatio ExpCount Count Size Term
GO:0006614 1.14896144109454e-27 198.209161624892 0.210712061449035
16 105 SRP-dependent cotranslational protein targeting to membrane
GO:0006613 1.35206783370638e-27 195.993162393162 0.212718842986645
16 106 cotranslational protein targeting to membrane
GO:0045047 1.35206783370638e-27 195.993162393162 0.212718842986645
16 106 protein targeting to ER
GO:0072599 1.35206783370638e-27 195.993162393162 0.212718842986645
16 106 establishment of protein localization to endoplasmic reticulum
GO:0070972 8.45158216386469e-27 172.790346907994 0.236800221437963
16 118 protein localization to endoplasmic reticulum
GO:0006415 2.08823149905626e-26 202.246240601504 0.182617119922497
15 91 translational termination
GO:0006414 2.09274983470356e-25 170.619047619048 0.210712061449035
15 105 translational elongation
GO:0006612 6.73796736003385e-25 128.332397529478 0.307037575254308
16 153 protein targeting to membrane
GO:0000184 1.53646023528533e-24 147.506868131868 0.238807002975573
15 119 nuclear-transcribed mRNA catabolic process, nonsense-mediated decay
GO:0019080 6.49938859362646e-23 112.547268907563 0.303024012179088
15 151 viral genome expression
GO:0019083 6.49938859362646e-23 112.547268907563 0.303024012179088
15 151 viral transcription
GO:0006413 7.20438898109483e-23 111.717935349322 0.305030793716698
15 152 translational initiation
GO:0072594 7.92730701992663e-23 93.1849427168576 0.40938343367241
16 204 establishment of protein localization to organelle

GO:0043624	7.98015086234435e-23	110.900621118012	0.307037575254308
15	153	cellular protein complex disassembly
GO:0043241	1.31694056865968e-22	106.985514485514	0.317071482942357
15	158	protein complex disassembly
GO:0000956	3.74629653040939e-22	99.2671614100186	0.339146079856065
15	169	nuclear-transcribed mRNA catabolic process
GO:0034623	5.88527129669815e-22	96.1118598382749	0.349179987544115
15	174	cellular macromolecular complex disassembly
GO:0006402	8.36500265154566e-22	93.7269938650307	0.357207113694554
15	178	mRNA catabolic process
GO:0032984	9.12170926387528e-22	93.1489547038328	0.359213895232164
15	179	macromolecular complex disassembly
GO:0006401	7.3662835824345e-21	80.2556390977444	0.41139021521002
15	205	RNA catabolic process
GO:0019058	4.58067706336739e-20	70.4662698412698	0.463566535187876
15	231	viral infectious cycle
GO:0022415	2.77114154915799e-19	61.9985422740525	0.521763199778562
15	260	viral reproductive process
GO:0071845	1.0557766715806e-18	56.3714816781731	0.569925956681198
15	284	cellular component disassembly at cellular level
GO:0022411	1.30432507449168e-18	55.5298273155416	0.577953082831638
15	288	cellular component disassembly
GO:0033365	6.20173941789631e-17	37.6098299949503	0.949207667289461
16	473	protein localization to organelle
GO:0006605	6.41279420733435e-17	37.5250251931475	0.951214448827071
16	474	protein targeting
GO:0016032	1.07194567004352e-15	34.3692660550459	0.905058473462044
15	451	viral reproduction
GO:0000028	1.44739365195045e-07	554.576923076923	0.0120406892256591
3	6	ribosomal small subunit assembly
GO:0042255	3.99834937900122e-06	127.890532544379	0.0321085046017577
3	16	ribosome assembly
GO:0042274	5.81045750171972e-06	110.823076923077	0.0361220676769774
3	18	ribosomal small subunit biogenesis
Tissue: Adipose_Subcutaneous=>Artery_Tibial	Type:	asymmetric
SourceGene:	RP11-40H20.1(ENSG00000214973.3)
TargetGeneSet:	ATPIF1	PPCS	C1orf50	BTBD19	UQCRH	TMEM167B	RP11
TRIB2	TPRKB	MRPS9	NDUFB3	DZIP1L	IGSF10	NDUFB5	FGFRL1	THEGL	NDUFC1
NDUFS6	ISCA1P1	COX7C	HINT1	COX7A2	SLC22A2	STAG3L1	PMS2P1	FAM160B2
RPL10AP2	DECR1	C9orf123	NUDT2	FAM27E1	PSMB7	ATP5C1	NDUFB8
USMG5	NAV2	NDUFS3	EML3	NEAT1	C11orf51	CCDC90B	ATP5L	NDUFA9
MRPL51	FAM90A1	C12orf39	NDUFA12	RPS7P10	ZBTB25	COX16	SLIRP	NDUFB1
C14orf2	CTD	MYEF2	GTF2A2	POLR3K	PKD1P6	PSMB10	C17orf89	NPC1
UQCR11	PIN1	ATP5J	RPL12P9	ATP5O	POLR2F	COX7B	NDUFA1
GOBPID	Pvalue	OddsRatio	ExpCount	Count	Size	Term
GO:0022904	2.86037904656724e-28	118.07967032967	0.305584388623625	18
96	respiratory electron transport chain
GO:0022900	1.78822752289842e-25	79.1878078817734	0.426544875787143
18	134	electron transport chain
GO:0045333	5.36833090300841e-25	74.0374423963134	0.452010241505778
18	142	cellular respiration
GO:0015980	7.78780791892873e-19	31.3999011369254	0.977233409452633
18	307	energy derivation by oxidation of organic compounds
GO:0006091	1.22161031685955e-17	24.0807751516798	1.36239706594699
19	428	generation of precursor metabolites and energy
GO:0006119	1.90989284439664e-17	96.0109422492401	0.184623901460107
11	58	oxidative phosphorylation
GO:0042773	1.77864292754985e-16	107.867867867868	0.149609023596983
10	47	ATP synthesis coupled electron transport
GO:0042775	1.77864292754985e-16	107.867867867868	0.149609023596983
10	47	mitochondrial ATP synthesis coupled electron transport
GO:0006120	1.2898541533863e-15	129.531531531532	0.114594145733859
9	36	mitochondrial electron transport, NADH to ubiquinone

GO:0042776	6.2905206281311e-08	152.338624338624	0.0413812192927825
4	13	mitochondrial ATP synthesis coupled proton transport
GO:0015985	2.66101931278596e-07	97.8979591836735	0.0572970728669296
4	18	energy coupled proton transport, down electrochemical gradient
GO:0015986	2.66101931278596e-07	97.8979591836735	0.0572970728669296
4	18	ATP synthesis coupled proton transport
GO:0006200	3.81956829649728e-06	24.9738675958188	0.238737803612207
5	75	ATP catabolic process
GO:0006754	1.71434037712978e-05	30.3915343915344	0.155975365026642
4	49	ATP biosynthetic process
Tissue: Adipose_Subcutaneous=>Artery_Tibial	Type:	cluster
SourceGene:	RPS7P10(ENSG00000226525.3)
TargetGeneSet:	AURKAIP1	MRPL20	FAM213B	PARK7	CELA2B	PLEKHM2	RP11	UBR4
MINOS1	SDHDP1	RP1	ATPIF1	7SK	PSMB2	RP3	C1orf122	PPCS
UQCRH	HBXIP	ATP5F1	S100A6	CRTC2	LAMTOR2	SDHC	TMCO1	PFN1P1	MIR4426
SNRPE	MORF4L1P1	C1orf31	SCCPDH	ITGB1BP1	OST4	C2orf28	FTH1P3
CALM2	MDH1	SNRPG	TPRKB	BOLA3	SUCLG1	COX5B	TXNDC9	TSPO	SCRN3
ATP5G3	MTX2	NIF3L1	NDUFB3	MYEOV2	PPP1R7	LSM3	SPCS1	PDHB	ATG3
NDUFB4	MRPS22	RNF7	UBE2V1P2	MRPL47	MCCC1	NCBP2	CPZ	COMMD8
MRPL1	ENOPH1	MRPS18C	PPA2	NUDT6	NDUFC1	SCOC	LSM6	C4orf27	PDCD6
NDUFS6	RBBP4P1	MOCS2	C5orf43	ISCA1P1	COX7C	CTD	CETN3	HINT1	FNIP1
UQCRQ	PDF	ARHGAP26	ATOX1	PPP1R2P3	RPL26L1	HIGD2A	NHP2	LSM2
PFDN6	HNRNPC	COX6A1P2	RPL24P4	MEA1	C6orf108	COX7A2	COQ3
FOXO3	MRPS12	SF3B5	MRPL18	NDUFA4	ANKMY2	LSM5	SEPT7	YAE1D1	PPIA
SEC61G	MDH2	TP53TG1	CDK6	ACN9	COPS6	PMS2P1	LRRN4	BPGM	JHDM1D
NDUFB2	SSBP1	DEFA3	CCDC25	LSM1	RPL10AP2	MRPS35	DECR1	COX6C
MRPL13	NDUFB9	C9orf46	SNRPEP2	C9orf123	PLIN2	NDUFB6	YBX1P10	RPS10P3
SEC61B	ATP5C1	CDC123	YWHAZP3	KIAA0913	SRP9P1	NDUFB8	USMG5	TCF7L2	UROS
ECHS1	POLR2L	MRPL23	ST13P5	IMMP1L	NDUFS3	C11orf10	CFL1	MRPL11
PPP1CA	MRPL21	C11orf51	CLNS1A	CCDC90B	TMEM126A	SDHD	ATP5L	MLL
DCPS	DYRK4	COPS7A	COX14	MYL6	NDUFA12	SNRPF	CCDC53	NUP37	C12orf45
MED13L	COX6A1	TRIAP1	POP5	DENR	ULK1	FAM207BP	RPS7P10	N4BP2L2-IT1
DGKH	MED4	COX7CP1	MRPL52	MYH6	RPS29	COX16	SLIRP	NDUFB1	SLC25A29
C14orf2	TMEM85	GTF2A2	RAB11A	COX5A	WDR61	RPS17L	MRPS11	U6	MRPL28
MAPK8IP3	NDUFB10	NPIPP1	PKD1P6	NDUFAB1	ATXN2L	NUTF2	PSMB10	HSBP1
PSMB6	TXNDC17	LSMD1	C17orf75	PSMB3	G6PC3	GPATCH8	PLEKHM1	ATP5G1	PHB
RAD51C	ICT1	C17orf89	ANAPC11	EPG5	ATP5A1	IER3IP1	UQCR11	C19orf70
INSR	C19orf79	NDUFA7	PIN1	ZNF791	WDR83OS	NDUFB7	AKAP8L	NDUFA13	POP4
COX6B1	PSENEN	TBCB	CYP2G1P	NDUFA3	TBC1D20	SNRPB2	NAA20	ROMO1	ATP5E
PPIAP22	ATP5J	ATP5O	SNRPD3	MTMR3	SLC5A1	POLR2F	GTPBP1	NDUFA6	TTLL1	RP6
GS1	EEF1B2P3	GAPDHP1	RPS2P55	RP13	PDZD11	ITGB1BP2	COX7B
PSMD10	NXT2	NDUFA1	FTH1P8	CETN2	PSMA6P1
GOBPID	Pvalue	OddsRatio	ExpCount	Count	Size	Term
GO:0022904	2.28900664353924e-48	57.2154963680387	1.44156113763753
40	96	respiratory electron transport chain
GO:0045333	1.95163034183628e-46	36.6865636427982	2.13230918275552
44	142	cellular respiration
GO:0022900	1.89851180194962e-41	33.9944704892415	2.01217908795239
40	134	electron transport chain
GO:0015980	2.28260667625191e-35	15.3252153252153	4.60999238807003
48	307	energy derivation by oxidation of organic compounds
GO:0006091	1.25140480086024e-30	10.9748756455343	6.42696007196734
50	428	generation of precursor metabolites and energy
GO:0042773	9.56750064780683e-25	53.4198157548411	0.705764306968376
20	47	ATP synthesis coupled electron transport
GO:0042775	9.56750064780683e-25	53.4198157548411	0.705764306968376
20	47	mitochondrial ATP synthesis coupled electron transport
GO:0006119	3.92800276161135e-24	41.1110038610039	0.870943187322677
21	58	oxidative phosphorylation
GO:0006120	5.28408124888484e-24	71.4371859296482	0.540585426614075
18	36	mitochondrial electron transport, NADH to ubiquinone
GO:0015985	1.42221004526642e-12	68.3894230769231	0.270292713307038
9	18	energy coupled proton transport, down electrochemical gradient

GO:0015986 1.42221004526642e-12 68.3894230769231 0.270292713307038
9 18 ATP synthesis coupled proton transport
GO:0042776 2.74228857669388e-12 108.93014354067 0.195211404055083 8
13 mitochondrial ATP synthesis coupled proton transport
GO:0006754 4.01334088685586e-08 15.3540865384615 0.735796830669158
9 49 ATP biosynthetic process
GO:0015992 4.80013218877625e-08 12.2308488612836 0.991073282125804
10 66 proton transport
GO:0006818 6.44429769370182e-08 11.8074296185241 1.02110580582659
10 68 hydrogen transport
GO:0000375 1.04999803973142e-07 5.79114788750548 3.13839872673171
16 209 RNA splicing, via transesterification reactions
GO:0009206 2.50301770650288e-07 12.0330882352941 0.900975711023459
9 60 purine ribonucleoside triphosphate biosynthetic process
GO:0009145 2.89832274965178e-07 11.800850591716 0.91599197287385 9
61 purine nucleoside triphosphate biosynthetic process
GO:0000387 3.02533742730919e-07 19.7361111111111 0.46550411736212
7 31 spliceosomal snRNP assembly
GO:0006839 4.46196502600813e-07 8.11937571771584 1.56169123244066
11 104 mitochondrial transport
GO:0000377 4.47596051955915e-07 5.51823039446802 3.06331741747976
15 204 RNA splicing, via transesterification reactions with bulged adenosine as nucleophile
GO:0000398 4.47596051955915e-07 5.51823039446802 3.06331741747976
15 204 nuclear mRNA splicing, via spliceosome
GO:0009201 5.0720347144576e-07 10.954842032967 0.976057020275413 9
65 ribonucleoside triphosphate biosynthetic process
GO:0009142 8.53672039585274e-07 10.2216346153846 1.03612206767698
9 69 nucleoside triphosphate biosynthetic process
GO:0008334 1.49277132324399e-06 21.2746320778249 0.375406546259774
6 25 histone mRNA metabolic process
GO:0006200 1.75012456482418e-06 9.28846153846154 1.12621963877932
9 75 ATP catabolic process
GO:0008380 1.86170795560085e-06 4.13085097619244 4.85025257767629
18 323 RNA splicing
GO:0000291 1.91640257732766e-06 20.2094786729858 0.390422808110165
6 26 nuclear-transcribed mRNA catabolic process, exonucleolytic
GO:0006099 1.91640257732766e-06 20.2094786729858 0.390422808110165
6 26 tricarboxylic acid cycle
GO:0043928 1.91640257732766e-06 20.2094786729858 0.390422808110165
6 26 exonucleolytic nuclear-transcribed mRNA catabolic process involved in deadenylation-dependent decay
GO:0046034 2.04380086286044e-06 6.85631067961165 1.81696768389731
11 121 ATP metabolic process
GO:0046356 2.43322641419875e-06 19.2457684495599 0.405439069960556
6 27 acetyl-CoA catabolic process
GO:0009152 2.44415243484746e-06 8.88273411371237 1.1712684243305 9
78 purine ribonucleotide biosynthetic process
GO:0009060 2.69779069651442e-06 13.5228571428571 0.630682997716421
7 42 aerobic respiration
GO:0009109 6.99634120062945e-06 15.5391906671528 0.480520379212511
6 32 coenzyme catabolic process
GO:0009260 8.10155114180119e-06 7.56036324786325 1.35146356653519
9 90 ribonucleotide biosynthetic process
GO:0006397 9.4570397907309e-06 3.49848484848485 5.991488478306 19
399 mRNA processing
GO:0051187 1.97713738455066e-05 12.6202606635071 0.570617950314857
6 38 cofactor catabolic process
Tissue: Adipose_Subcutaneous=>Artery_Tibial Type: cluster
SourceGene: SHFM1(ENSG00000127922.5)
TargetGeneSet: AURKAIP1 MRPL20 PARK7 SLC45A1 CASZ1 PLEKHM2 RP11 UBR4
ATPIF1 7SK PSMB2 INSL3 PPCS TDPX2 UQCRH MAGOH MRPL37 HBXIP
ATP5F1 MRPS21 SCNM1 THEM4 S100A6 DENND4B LAMTOR2 PFDN2 UFC1 SDHC

TMCO1 RP4 MIR4426 SNRPE ACP1 PXDN PTRHD1 C2orf28 COMMD1 SNRPG
TPRKB KDM3A COX5B IL1R1 TSN MTX2 NDUFB3 EEF1B2 MYEOV2 PPP1R7 LSM3
TRANK1 CCDC72 SPCS1 C3orf26 ATG3 NDUFB4 UMPS MRPS22 RNF7 UBE2V1P2
MRPL47 MRPL1 MRPS18C RG9MTD2 LAMTOR3 NPNT EXOSC9 NDUFC1 LSM6 C4orf27
PDCD6 NDUFS6 RBBP4P1 COX7C CTD CETN3 HINT1 UQCRQ PDF ATOX1
LSM11 CTB BNIP1 EEF1E1 NOL7 HLA-F-AS1 LSM2 PFDN6 HNRNPC
TEAD3 COX6A1P2 RPL24P4 MEA1 COX7A2 C6orf203 MRPS12 SF3B5
PSMB1 PSMG3 LSM5 YAE1D1 MPLKIP CHCHD2 BUD31 COPS6 PMS2P1 ZNHIT1
MKLN1 JHDM1D MRPS33 SSBP1 RBPMS CHCHD7 TCEB1 MRPS35 DECR1 VPS13B
MRPL13 NDUFB9 CTA HEATR7A PLIN2 ACER2 NDUFB6 DCTN3 SEC61B COQ4
SURF2 EDF1 ATP5C1 CDC123 RPP30 EXOSC1 NDUFB8 LZTS2 USMG5 ADRA2A
TCF7L2 UROS BCCIP BNIP3 ECHS1 POLR2L MRPL23 PRKCDBP DENND5A ST13P5
IMMP1L NDUFS3 TIMM10 C11orf10 TRMT112 MRPL11 GSTP1 MRPL21 CLNS1A
TMEM126A CWC15 SDHD ATP5L ARHGAP32 ITFG2 NDUFA9 MRPL51
COPS7A CLSTN3 NDUFA12 SNRPF NUP37 C12orf45 MED13L POP5 DENR TRAC
SAP18 DGKH MED4 MRPL52 ARHGAP5-AS1 RPS29 C14orf166 ACTR10
COX16 MED6 SLIRP NDUFB1 GLRX5 C14orf2 TMEM85 ATPBD4 SRP14 NDUFAF1
GTF2A2 RPLP1 COMMD4 WDR61 KIF7 MRPL28 PKD1P6 NDUFAB1 LCMT1 PSMB10
TRAPPC2L PSMB6 TXNDC17 FAM18B1 USP32P3 STAT5B VPS25 CCDC56 RPL27
ATP5G1 MRPL27 RAD51C USP32 ICT1 RNF213 C17orf89 ARL16 STRA13
FOXK2 SNRPD1 NPC1 TTC39C IER3IP1 CTIF UQCR11 DOT1L PIP5K1C MRPL54
C19orf70 NDUFA11 C19orf79 NDUFA7 PIN1 ZNF791 WDR83OS NDUFB7
AKAP8L C19orf42 NDUFA13 POP4 COX6B1 TBCB EIF3K TIMM50 NDUFA3
HSPBP1 TBC1D20 EBF4 SNRPB2 NAA20 ROMO1 SRC C20orf43 ATP5E
ATP5J ATP5O SNRPD3 TXN2 POLR2F C22orf32 RP6 EEF1B2P3 UXT
PDZD11 COX7B NDUFA1 RP3 FTH1P8 PSMA6P1
GOBPID Pvalue OddsRatio ExpCount Count Size Term
GO:0022904 4.592840749001e-40 45.4400586134261 1.42163172098817
35 96 respiratory electron transport chain
GO:0022900 1.09760855787478e-35 29.1793166704884 1.98436094387932
36 134 electron transport chain
GO:0045333 2.94674400912896e-33 25.8210202057119 2.10283025396166
35 142 cellular respiration
GO:0015980 2.3719550345587e-23 10.8135383971542 4.54625977441008
37 307 energy derivation by oxidation of organic compounds
GO:0042773 1.78318070340241e-21 44.9936664320901 0.69600719673379
18 47 ATP synthesis coupled electron transport
GO:0042775 1.78318070340241e-21 44.9936664320901 0.69600719673379
18 47 mitochondrial ATP synthesis coupled electron transport
GO:0006119 4.41564138584492e-21 35.4716633793557 0.858902498097017
19 58 oxidative phosphorylation
GO:0006120 1.71055328476584e-20 57.4424242424242 0.533111895370563
16 36 mitochondrial electron transport, NADH to ubiquinone
GO:0006091 3.75656934907675e-20 7.93348512669849 6.33810808940558
39 428 generation of precursor metabolites and energy
GO:0042776 2.25337263936207e-10 80.20692431562 0.192512628883814 7
13 mitochondrial ATP synthesis coupled proton transport
GO:0015985 3.9237361525384e-09 43.733860342556 0.266555947685281 7
18 energy coupled proton transport, down electrochemical gradient
GO:0015986 3.9237361525384e-09 43.733860342556 0.266555947685281 7
18 ATP synthesis coupled proton transport
GO:0000377 6.18089095008291e-08 6.03868471953578 3.02096740709985
16 204 RNA splicing, via transesterification reactions with bulged adenosine as nucleophile
GO:0000398 6.18089095008291e-08 6.03868471953578 3.02096740709985
16 204 nuclear mRNA splicing, via spliceosome
GO:0000375 8.65885874068974e-08 5.88014863662532 3.09501072590132
16 209 RNA splicing, via transesterification reactions
GO:0000387 2.75271548332563e-07 20.0263687600644 0.459068576569096
7 31 spliceosomal snRNP assembly
GO:0008380 3.38592482816071e-07 4.46570512820513 4.78319839457477
19 323 RNA splicing
GO:0006839 3.88301096441851e-07 8.24111446580857 1.54010103107051

11	104	mitochondrial transport
GO:0006397	5.04419637022262e-07	3.98934945308002	5.90865684035707
21	399	mRNA processing
GO:0000291	1.76742644042912e-06	20.5052884615385	0.385025257767629
6	26	nuclear-transcribed mRNA catabolic process, exonucleolytic
GO:0043928	1.76742644042912e-06	20.5052884615385	0.385025257767629
6	26	exonucleolytic nuclear-transcribed mRNA catabolic process involved in deadenylation-dependent decay
GO:0015992	5.57481779288666e-06	9.49380649481085	0.977371808179365
8	66	proton transport
GO:0006818	6.99519943735294e-06	9.17605177993527	1.00698913569995
8	68	hydrogen transport
GO:0006754	7.1730428209445e-06	11.4291465378422	0.725624524254377
7	49	ATP biosynthetic process
GO:0006200	1.46104216620105e-05	8.21330241993914	1.11064978202201
8	75	ATP catabolic process
Tissue: Artery_Tibial=>Heart_Left_Ventricle	Type:	asymmetric
SourceGene:	ALG10B(ENSG00000175548.4)
TargetGeneSet:	CELA2B	CELA3B	CELA3A	AMY2A	CPB1	SPINK1	CLPS	SNHG5	CPA2	CPA1	PRSS1	PRSS3P1	GSDMD	PIGO	PNLIP	PNLIPRP1	PNLIPRP2	CFL1	GP2	CTRB2	CTRB1	DOHH	SYCN	RBPJL
GOBPID	Pvalue	OddsRatio	ExpCount	Count	Size	Term
GO:0007586	1.51426135092439e-12	87.3054074638233	0.158397342744447
8	109	digestion
GO:0044241	1.18997840725084e-06	200.25	0.021797799460245	3	15
lipid digestion
GO:0022617	1.83105550247234e-05	72.7121212121212	0.0523147187045879
3	36	extracellular matrix disassembly
Tissue: Artery_Tibial=>Heart_Left_Ventricle	Type:	asymmetric
SourceGene:	ATCAY(ENSG00000167654.11)
TargetGeneSet:	SLC35E2	RPL11	DCDC2B	KIAA1522	RPL5	ANXA9	ASH1L	USP34	TGOLN2	ENO1P4	NGLY1	RPL14	RNF123	MGLL	RP11	FRYL	WWC2	CTD	SERF1B	GNB2L1	COQ3	TNRC18	MRPS24	RPS3AP26	RPS20	DCAF13	RPS24	TUB	PPP6R3	ERC1	MLL4	SMARCC2	COX17P1	ISCA2	C14orf1	CDC42BPB	VPS39	HERC1	RPL4	SEC11A	HDDC3	NDUFB10	TSC2	RPS15A	POLR2C	ZZEF1	RPL26	RPS7P1	TAOK1	RPL27	RPS7P11	MRPL27	SNRPD1	C18orf21	TNPO2	CIC	IQSEC2	COX7B	FUNDC2
GOBPID	Pvalue	OddsRatio	ExpCount	Count	Size	Term
GO:0006614	5.3498284809783e-13	44.3095975232198	0.31970105875026
10	105	SRP-dependent cotranslational protein targeting to membrane
GO:0006613	5.89440433861055e-13	43.8449754901961	0.322745830738357
10	106	cotranslational protein targeting to membrane
GO:0045047	5.89440433861055e-13	43.8449754901961	0.322745830738357
10	106	protein targeting to ER
GO:0072599	5.89440433861055e-13	43.8449754901961	0.322745830738357
10	106	establishment of protein localization to endoplasmic reticulum
GO:0070972	1.75739236583815e-12	38.9406318082789	0.35928309459553
10	118	protein localization to endoplasmic reticulum
GO:0006415	6.14705621497614e-12	44.9216027874564	0.277074250916892
9	91	translational termination
GO:0019080	2.10359359521579e-11	29.7580308719232	0.459760570202754
10	151	viral genome expression
GO:0019083	2.10359359521579e-11	29.7580308719232	0.459760570202754
10	151	viral transcription
GO:0006414	2.28563692869326e-11	38.3330357142857	0.31970105875026
9	105	translational elongation
GO:0006612	2.39882978429815e-11	29.337721102427	0.46585011417895	10
153	protein targeting to membrane
GO:0000184	7.13438509531244e-11	33.4215584415584	0.362327866583627
9	119	nuclear-transcribed mRNA catabolic process, nonsense-mediated decay
GO:0034623	8.60882279304478e-11	25.543400286944	0.529790325929001	10
174	cellular macromolecular complex disassembly
GO:0032984	1.13906511263041e-10	24.7789766794292	0.54501418586949

10	179	macromolecular complex disassembly
GO:0072594	4.11819688568161e-10	21.547907822923	0.621133485571933	10
204	establishment of protein localization to organelle
GO:0006413	6.43233951888559e-10	25.6495504495504	0.462805342190852
9	152	translational initiation
GO:0043624	6.81936805266407e-10	25.4696428571429	0.46585011417895
9	153	cellular protein complex disassembly
GO:0043241	9.07867156857262e-10	24.6063279002876	0.481073974119438
9	158	protein complex disassembly
GO:0019058	1.38261437436767e-09	18.8794250731967	0.703342329250571
10	231	viral infectious cycle
GO:0000956	1.64917272782406e-09	22.8969642857143	0.514566465988513
9	169	nuclear-transcribed mRNA catabolic process
GO:0006402	2.60802104409707e-09	21.6639053254438	0.541969413881392
9	178	mRNA catabolic process
GO:0022415	4.33241926972594e-09	16.6552941176471	0.791640716905404
10	260	viral reproductive process
GO:0006401	9.00427829797012e-09	18.6441690962099	0.62417825756003
9	205	RNA catabolic process
GO:0071845	1.00945754043179e-08	15.1706741090597	0.864715244619749
10	284	cellular component disassembly at cellular level
GO:0022411	1.15355570861207e-08	14.948159119763	0.87689433257214	10
288	cellular component disassembly
GO:0042273	3.63450224529092e-08	179.9875	0.0365372638571725	4
12	ribosomal large subunit biogenesis
GO:0033365	1.18742772274158e-07	10.0613275613276	1.44017715037022
11	473	protein localization to organelle
GO:0006605	1.21303468067644e-07	10.0388768898488	1.44322192235831
11	474	protein targeting
GO:0022613	3.33587853221997e-07	14.531490015361	0.685073697321985	8
225	ribonucleoprotein complex biogenesis
GO:0071843	5.27937993328423e-07	13.6373256373256	0.727700505155353
8	239	cellular component biogenesis at cellular level
GO:0006364	6.7936221870074e-07	23.0542427497315	0.316656286762162
6	104	rRNA processing
GO:0016032	7.598600501804e-07	9.31439242363612	1.37319216663207
10	451	viral reproduction
GO:0016072	9.46033587040975e-07	21.7150809716599	0.334924918690748
6	110	rRNA metabolic process
GO:0042254	5.55064422131899e-06	15.7497239602503	0.453671026226559
6	149	ribosome biogenesis
Tissue: Artery_Tibial=>Heart_Left_Ventricle	Type:	asymmetric
SourceGene:	ENSG00000230807.1
TargetGeneSet:	MRPL20	UBE4B	RP4	SPEN	EIF1AX	RP1	PUM1	AK2
AKR1A1	TAL1	ZCCHC11	RP11	ITGA10	MRPS21	PMF1	TMCO1	FAM5B	COG2
PQLC3	SOCS5	COX5B	UXS1	RANBP2	SCTR	CLASP1	ATP5G3	MYO1B	NDUFB3
KANSL1L	RPL37A	CCDC72	RNF123	C3orf78	ABTB1	RPL35A	GAK	MXD4	LAMTOR3
PAPSS1	SMARCA5	CTSO	TMA16	TXNDC15	SOX9	SLC35A4	FBXO38	ARMC12	MRPL2
TDRD6	HMGN3	GTF3C6	C7orf26	NDUFA4	MRPS24	SEC61G	ECD	BRI3	ATP5J2	POP7
ZNF800	SLC13A4	NDUFB2	ZNF746	MCPH1	KAT6A	UBE2V2	MRPS35	C8orf59	NDUFB9	IL33
C9orf123		COL15A1	SLC31A1	RC3H2	RSU1	KIAA0913		C10orf11
EIF5AL1	USMG5	PPIAP19	AP2A2	C11orf10		FADS2	COX8A	VEGFB	PRDX5	FAU
PCNXL3	RELA	PPP6R3	PKNOX2	COX14	TENC1	NDUFA12	C12orf73		ULK1	DAD1
MRPL52	ADCY4	RPS29	FRMD6	PPM1A	ERH	ZFYVE1	NEK9	SLIRP	NDUFB1
VPS39	RPL4	IMP3	NPRL3	NDUFB10	FLYWCH2	SETD1A	COX4I1	ZC3H18	PSMB6
TXNDC17	CTC1	RPS7P1	GIT1	RASL10B	RPL27	HDAC5	ITGA2B	PLEKHM1	NFE2L1
CALCOCO2		PPM1D	RPL38	ATP5H	LLGL2	C17orf89		MYL12B	GALNT1
CTDP1	C19orf35		SAFB	XAB2	MRPL4	CC2D1A	MAST3	MAU2	LPAR2
ATP13A1	COX6B1	PSENEN	BLVRB	TMEM91	PPP6R1	PLCG1	RPS21	CABIN1	TPST2
SNORD125		PLA2G6	TUBGCP6	SMS	EEF1B2P3		COX7B	NDUFA1	APLN
GOBPID	Pvalue	OddsRatio	ExpCount	Count	Size	Term
GO:0022904	7.98769694576923e-15	22.5356125356125	0.87689433257214
15	96	respiratory electron transport chain

GO:0022900 7.61119557949721e-14 16.5996493278784 1.22399833921528
16 134 electron transport chain
GO:0045333 2.9378183147757e-12 14.3266707046235 1.29707286692962
15 142 cellular respiration
GO:0015980 1.9929231767191e-08 6.64912904372556 2.80423500103799
16 307 energy derivation by oxidation of organic compounds
GO:0006120 3.02707805738153e-08 27.5944827586207 0.328835374714553
7 36 mitochondrial electron transport, NADH to ubiquinone
GO:0006415 1.40107317860821e-07 12.7040452111838 0.831222752750675
9 91 translational termination
GO:0042773 2.09804652824833e-07 19.9906 0.429312850321777 7 47
ATP synthesis coupled electron transport
GO:0042775 2.09804652824833e-07 19.9906 0.429312850321777 7 47
mitochondrial ATP synthesis coupled electron transport
GO:0006091 3.57086772064615e-07 5.00234846080609 3.90948723271746
17 428 generation of precursor metabolites and energy
GO:0006414 4.82418233945863e-07 10.8407012195122 0.959103176250778
9 105 translational elongation
GO:0006614 4.82418233945863e-07 10.8407012195122 0.959103176250778
9 105 SRP-dependent cotranslational protein targeting to membrane
GO:0006613 5.23145762962558e-07 10.7281870756852 0.968237492215072
9 106 cotranslational protein targeting to membrane
GO:0045047 5.23145762962558e-07 10.7281870756852 0.968237492215072
9 106 protein targeting to ER
GO:0072599 5.23145762962558e-07 10.7281870756852 0.968237492215072
9 106 establishment of protein localization to endoplasmic reticulum
GO:0006119 9.22808339435138e-07 15.6668235294118 0.529790325929001
7 58 oxidative phosphorylation
GO:0070972 1.29897347402138e-06 9.53904676661445 1.07784928378659
9 118 protein localization to endoplasmic reticulum
GO:0043624 1.3627953712964e-06 8.12564484695632 1.39755034253685
10 153 cellular protein complex disassembly
GO:0043241 1.82632862887981e-06 7.8483606557377 1.44322192235831 10
158 protein complex disassembly
GO:0034623 4.35335253573461e-06 7.07467013194722 1.589370977787 10
174 cellular macromolecular complex disassembly
GO:0032984 5.60271956255934e-06 6.86293529925308 1.63504255760847
10 179 macromolecular complex disassembly
GO:0019080 9.94819710749006e-06 7.30522157334249 1.37928171060826
9 151 viral genome expression
GO:0019083 9.94819710749006e-06 7.30522157334249 1.37928171060826
9 151 viral transcription
GO:0006413 1.0493523203216e-05 7.25362442435613 1.38841602657256
9 152 translational initiation
GO:0006612 1.1064193692711e-05 7.20274390243902 1.39755034253685
9 153 protein targeting to membrane
GO:0000184 1.3130513094014e-05 8.25806451612903 1.08698359975088
8 119 nuclear-transcribed mRNA catabolic process, nonsense-mediated decay
Tissue: Artery_Tibial=>Heart_Left_Ventricle Type: cluster
SourceGene: ENSG00000230807.1
TargetGeneSet: MRPL20 UBE4B RP4 SPEN EIF1AX RP1 PUM1 AK2
AKR1A1 TAL1 ZCCHC11 RP11 ITGA10 MRPS21 PMF1 TMCO1 FAM5B COG2
PQLC3 SOCS5 COX5B UXS1 RANBP2 SCTR CLASP1 ATP5G3 MYO1B NDUFB3
KANSL1L RPL37A CCDC72 RNF123 C3orf78 ABTB1 RPL35A GAK MXD4 LAMTOR3
PAPSS1 SMARCA5 CTSO TMA16 TXNDC15 SOX9 SLC35A4 FBXO38 ARMC12 MRPL2
TDRD6 HMGN3 GTF3C6 C7orf26 NDUFA4 MRPS24 SEC61G ECD BRI3 ATP5J2 POP7
ZNF800 SLC13A4 NDUFB2 ZNF746 MCPH1 KAT6A UBE2V2 MRPS35 C8orf59 NDUFB9 IL33
C9orf123 COL15A1 SLC31A1 RC3H2 RSU1 KIAA0913 C10orf11
EIF5AL1 USMG5 PPIAP19 AP2A2 C11orf10 FADS2 COX8A VEGFB PRDX5 FAU
PCNXL3 RELA PPP6R3 PKNOX2 COX14 TENC1 NDUFA12 C12orf73 ULK1 DAD1
MRPL52 ADCY4 RPS29 FRMD6 PPM1A ERH ZFYVE1 NEK9 SLIRP NDUFB1
VPS39 RPL4 IMP3 NPRL3 NDUFB10 FLYWCH2 SETD1A COX4I1 ZC3H18 PSMB6
TXNDC17 CTC1 RPS7P1 GIT1 RASL10B RPL27 HDAC5 ITGA2B PLEKHM1 NFE2L1

CALCOCO2 PPM1D RPL38 ATP5H LLGL2 C17orf89 MYL12B GALNT1
CTDP1 C19orf35 SAFB XAB2 MRPL4 CC2D1A MAST3 MAU2 LPAR2
ATP13A1 COX6B1 PSENEN BLVRB TMEM91 PPP6R1 PLCG1 RPS21 CABIN1 TPST2
SNORD125 PLA2G6 TUBGCP6 SMS EEF1B2P3 COX7B NDUFA1 APLN
GOBPID Pvalue OddsRatio ExpCount Count Size Term
GO:0022904 7.98769694576923e-15 22.5356125356125 0.87689433257214
15 96 respiratory electron transport chain
GO:0022900 7.61119557949721e-14 16.5996493278784 1.22399833921528
16 134 electron transport chain
GO:0045333 2.9378183147757e-12 14.3266707046235 1.29707286692962
15 142 cellular respiration
GO:0015980 1.9929231767191e-08 6.64912904372556 2.80423500103799
16 307 energy derivation by oxidation of organic compounds
GO:0006120 3.02707805738153e-08 27.5944827586207 0.328835374714553
7 36 mitochondrial electron transport, NADH to ubiquinone
GO:0006415 1.40107317860821e-07 12.7040452111838 0.831222752750675
9 91 translational termination
GO:0042773 2.09804652824833e-07 19.9906 0.429312850321777 7 47
ATP synthesis coupled electron transport
GO:0042775 2.09804652824833e-07 19.9906 0.429312850321777 7 47
mitochondrial ATP synthesis coupled electron transport
GO:0006091 3.57086772064615e-07 5.00234846080609 3.90948723271746
17 428 generation of precursor metabolites and energy
GO:0006414 4.82418233945863e-07 10.8407012195122 0.959103176250778
9 105 translational elongation
GO:0006614 4.82418233945863e-07 10.8407012195122 0.959103176250778
9 105 SRP-dependent cotranslational protein targeting to membrane
GO:0006613 5.23145762962558e-07 10.7281870756852 0.968237492215072
9 106 cotranslational protein targeting to membrane
GO:0045047 5.23145762962558e-07 10.7281870756852 0.968237492215072
9 106 protein targeting to ER
GO:0072599 5.23145762962558e-07 10.7281870756852 0.968237492215072
9 106 establishment of protein localization to endoplasmic reticulum
GO:0006119 9.22808339435138e-07 15.6668235294118 0.529790325929001
7 58 oxidative phosphorylation
GO:0070972 1.29897347402138e-06 9.53904676661445 1.07784928378659
9 118 protein localization to endoplasmic reticulum
GO:0043624 1.3627953712964e-06 8.12564484695632 1.39755034253685
10 153 cellular protein complex disassembly
GO:0043241 1.82632862887981e-06 7.8483606557377 1.44322192235831 10
158 protein complex disassembly
GO:0034623 4.35335253573461e-06 7.07467013194722 1.589370977787 10
174 cellular macromolecular complex disassembly
GO:0032984 5.60271956255934e-06 6.86293529925308 1.63504255760847
10 179 macromolecular complex disassembly
GO:0019080 9.94819710749006e-06 7.30522157334249 1.37928171060826
9 151 viral genome expression
GO:0019083 9.94819710749006e-06 7.30522157334249 1.37928171060826
9 151 viral transcription
GO:0006413 1.0493523203216e-05 7.25362442435613 1.38841602657256
9 152 translational initiation
GO:0006612 1.1064193692711e-05 7.20274390243902 1.39755034253685
9 153 protein targeting to membrane
GO:0000184 1.3130513094014e-05 8.25806451612903 1.08698359975088
8 119 nuclear-transcribed mRNA catabolic process, nonsense-mediated decay
Tissue: Heart_Left_Ventricle=>Artery_Tibial Type: asymmetric
SourceGene: ENSG00000239899.1
TargetGeneSet: RPL22 SDHB RP11 PPIH MAGOH PFN1P3 C1orf54 SNRPE ARV1
RPS7 UBC CCDC115 BTF3L4P2 HDLBP SPCS1 CHMP2B ATG3 MRPS18C BDH2
RPL34 LARP7 TBCA MRPL22 UBLCP1 RPL26L1 SBDS C7orf59 DCTN6 SEC61B
SH3PXD2A POLR2G SNRPF TCP11L2 ARPC3 COMMD6 SRP54 C14orf166 ERH
COX16 SNURF RPS7P1 ARL16 SNRPD1 MIR1181 SNRPB2 RP4 PSMD10
GOBPID Pvalue OddsRatio ExpCount Count Size Term

GO:0006612	1.27453364898476e-10	31.8526785714286	0.391737596014117
9	153	protein targeting to membrane
GO:0006614	1.92211993322925e-10	40.7166725915393	0.268839526676355
8	105	SRP-dependent cotranslational protein targeting to membrane
GO:0006613	2.07530277581746e-10	40.2983814215341	0.271399903120891
8	106	cotranslational protein targeting to membrane
GO:0045047	2.07530277581746e-10	40.2983814215341	0.271399903120891
8	106	protein targeting to ER
GO:0072599	2.07530277581746e-10	40.2983814215341	0.271399903120891
8	106	establishment of protein localization to endoplasmic reticulum
GO:0070972	4.92673214021216e-10	35.8721003134796	0.302124420455332
8	118	protein localization to endoplasmic reticulum
GO:0000377	3.73574243788099e-08	20.0112596762843	0.522316794685489
8	204	RNA splicing, via transesterification reactions with bulged adenosine as nucleophile
GO:0000398	3.73574243788099e-08	20.0112596762843	0.522316794685489
8	204	nuclear mRNA splicing, via spliceosome
GO:0072594	3.73574243788099e-08	20.0112596762843	0.522316794685489
8	204	establishment of protein localization to organelle
GO:0000375	4.50877038966395e-08	19.506604906502 0.535118676908172	8
209	RNA splicing, via transesterification reactions
GO:0006605	2.06029408872816e-07	11.1350574712644	1.2136184347104 10
474	protein targeting
GO:0000184	5.1838945958636e-07	24.495004282044 0.304684796899869	6
119	nuclear-transcribed mRNA catabolic process, nonsense-mediated decay
GO:0000387	1.08899456367171e-06	64.5881032547699	0.079371669780638
4	31	spliceosomal snRNP assembly
GO:0008380	1.25349641021633e-06	12.3472359058566	0.827001591585357
8	323	RNA splicing
GO:0019080	2.09657457101439e-06	19.0464961067853	0.386616843125043
6	151	viral genome expression
GO:0019083	2.09657457101439e-06	19.0464961067853	0.386616843125043
6	151	viral transcription
GO:0033365	2.19659825432831e-06	9.66363916256158	1.21105805826586
9	473	protein localization to organelle
GO:0043624	2.26345082545556e-06	18.7847267939434	0.391737596014117
6	153	cellular protein complex disassembly
GO:0043241	2.72856670166277e-06	18.160441426146 0.4045394782368 6	158
protein complex disassembly
GO:0006415	3.29492299643312e-06	26.031976744186 0.232994256452841	5
91	translational termination
GO:0000956	4.02933468661639e-06	16.9218286166634	0.432703619126704
6	169	nuclear-transcribed mRNA catabolic process
GO:0022618	4.07494624917772e-06	24.8680555555556	0.243235762230987
5	95	ribonucleoprotein complex assembly
GO:0034623	4.76791874302665e-06	16.4124423963134	0.445505501349388
6	174	cellular macromolecular complex disassembly
GO:0071826	5.24624360037402e-06	23.5509868421053	0.256037644453671
5	100	ribonucleoprotein complex subunit organization
GO:0006402	5.43504343328772e-06	16.026256564141 0.455747007127534	6
178	mRNA catabolic process
GO:0032984	5.61316103833135e-06	15.932500466157 0.458307383572071	6
179	macromolecular complex disassembly
GO:0006397	6.02270326970651e-06	9.89364141458682	1.02159020137015
8	399	mRNA processing
GO:0006414	6.66670912870521e-06	22.365625	0.268839526676355	5
105	translational elongation
GO:0006401	1.22025309393652e-05	13.8255795104555	0.524877171130026
6	205	RNA catabolic process
Tissue: Heart_Left_Ventricle=>Artery_Tibial	Type:	asymmetric
SourceGene:	LINC00342(ENSG00000232931.1)
TargetGeneSet:	TTLL10	RPL11	ATPIF1	BEND5	HSD17B7 NTSR2	RPL31	RPL37A
RPL32	RPL10AP6	RP11	ATP5I	RPL37	RPS23	RPS18	RPL10A	RPL39P3

TSPYL4 C7orf50 FTSJ2 RPL30 RPS24 PIK3AP1 RPL13AP5 RPS10P18
RPLP2 FAU C11orf51 SNORD15A RPS25 SAP18 RPS26P47
RNASE3 RPS29 BATF HMGN2P5 IGSF6 SNORA70D C17orf108 RPL27 SMG8
RPL38 ZNF77 B3GNT3 RPS11 CHMP2A EEF1B2P3 RPS23P8
GOBPID Pvalue OddsRatio ExpCount Count Size Term
GO:0000184 7.42719078799523e-30 170.10297029703 0.271745899937721 18
119 nuclear-transcribed mRNA catabolic process, nonsense-mediated decay
GO:0006415 8.52135771726807e-30 205.952702702703 0.207805688187669
17 91 translational termination
GO:0006414 1.19774132786802e-28 173.018465909091 0.239775794062695
17 105 translational elongation
GO:0006614 1.19774132786802e-28 173.018465909091 0.239775794062695
17 105 SRP-dependent cotranslational protein targeting to membrane
GO:0006613 1.42502169615293e-28 171.0625 0.242059373053768 17
106 cotranslational protein targeting to membrane
GO:0045047 1.42502169615293e-28 171.0625 0.242059373053768 17
106 protein targeting to ER
GO:0072599 1.42502169615293e-28 171.0625 0.242059373053768 17
106 establishment of protein localization to endoplasmic reticulum
GO:0070972 1.00710139228052e-27 150.612004950495 0.269462320946647
17 118 protein localization to endoplasmic reticulum
GO:0006401 2.26457001719109e-27 103.84331797235 0.468133693170023 19
205 RNA catabolic process
GO:0000956 5.91341668537133e-27 113.380132450331 0.385924849491385
18 169 nuclear-transcribed mRNA catabolic process
GO:0006402 1.56630778630558e-26 106.935 0.406477060411044 18 178
mRNA catabolic process
GO:0019080 8.4719982604753e-26 113.259328358209 0.344820427652066
17 151 viral genome expression
GO:0019083 8.4719982604753e-26 113.259328358209 0.344820427652066
17 151 viral transcription
GO:0006413 9.52877204336302e-26 112.4125 0.347104006643139 17
152 translational initiation
GO:0006612 1.07085505506166e-25 111.578125 0.349387585634212 17
153 protein targeting to membrane
GO:0043624 1.07085505506166e-25 111.578125 0.349387585634212 17
153 cellular protein complex disassembly
GO:0043241 1.89674334653211e-25 107.583776595745 0.360805480589579
17 158 protein complex disassembly
GO:0034623 1.04633766693221e-24 96.5115445859873 0.397342744446751
17 174 cellular macromolecular complex disassembly
GO:0032984 1.72447333136711e-24 93.5 0.408760639402117 17 179
macromolecular complex disassembly
GO:0072594 1.71120873279172e-23 80.8579545454545 0.46585011417895
17 204 establishment of protein localization to organelle
GO:0019058 1.4935510238952e-22 70.5221962616822 0.527506746937928
17 231 viral infectious cycle
GO:0022415 1.15837947159721e-21 61.9791666666667 0.593730537679053
17 260 viral reproductive process
GO:0071845 5.30437914201888e-21 56.3125 0.648536433464812 17 284
cellular component disassembly at cellular level
GO:0022411 6.74604316332267e-21 55.4656365313653 0.657670749429105
17 288 cellular component disassembly
GO:0016032 1.38385996314761e-17 34.2350230414747 1.02989412497405
17 451 viral reproduction
GO:0033365 3.0809023528745e-17 32.5320723684211 1.08013286277766
17 473 protein localization to organelle
GO:0006605 3.19206611551764e-17 32.4585612691466 1.08241644176874
17 474 protein targeting
GO:0022613 1.03848533290597e-05 14.4079147640791 0.513805272991488
6 225 ribonucleoprotein complex biogenesis
GO:0071843 1.46423493164358e-05 13.5288507391512 0.545775378866514
6 239 cellular component biogenesis at cellular level

Tissue: Heart_Left_Ventricle=>Artery_Tibial Type: cluster
SourceGene: MCM9(ENSG00000111877.12)
TargetGeneSet: TPRG1L RPL22 RP11 PSMB4 RP4 MIR4426 MRPS9 EEF1B2
XRCC5 RPL37A RPL32 LSM3 SPCS1 SLC25A26 PLSCR4 KLHL7 EREG
RPL37 RPS23 COX7C CTB RPS14 MRPL22 PFDN6 C6orf203 ECD
UQCRB RPL30 EIF3H NDUFB9 RPS24 RPL13AP5 CUTC SH3PXD2A
RPLP2 RPS13 ST13P5 FAU MRPL11 ATP5L RPS25 CLECL1 SLC25A3 RPS29
RPL36A C14orf166 GCH1 SLIRP RPL4 SEC11A NAE1 TRAPPC2L
RPL26 GSDMA PSMD3 TOP2A RPL38 ICT1 CTD POLR2I EIF3K RPS16 RPS5
CHMP2A ATP5O POLR2F TOMM22 XRCC6 EEF1B2P3 PIN4 NDUFA1 MT
GOBPID Pvalue OddsRatio ExpCount Count Size Term
GO:0006614 3.77581317469522e-28 76.4812834224599 0.465019721818559
20 105 SRP-dependent cotranslational protein targeting to membrane
GO:0006613 4.6403266368115e-28 75.5866807610994 0.469448481073974
20 106 cotranslational protein targeting to membrane
GO:0045047 4.6403266368115e-28 75.5866807610994 0.469448481073974
20 106 protein targeting to ER
GO:0072599 4.6403266368115e-28 75.5866807610994 0.469448481073974
20 106 establishment of protein localization to endoplasmic reticulum
GO:0006415 1.4408454599776e-27 83.945987654321 0.403017092242751 19
91 translational termination
GO:0070972 4.70563162418768e-27 66.2755102040816 0.522593592138952
20 118 protein localization to endoplasmic reticulum
GO:0006612 2.2506420447113e-26 52.7404862579281 0.677600166078472
21 153 protein targeting to membrane
GO:0006414 2.79382704897336e-26 70.2116279069767 0.465019721818559
19 105 translational elongation
GO:0019058 1.31734390049684e-25 38.2407363977486 1.02304338800083
23 231 viral infectious cycle
GO:0019080 8.75518207734649e-25 49.4656488549618 0.668742647567642
20 151 viral genome expression
GO:0019083 8.75518207734649e-25 49.4656488549618 0.668742647567642
20 151 viral transcription
GO:0006413 1.0052200665236e-24 49.0874655647383 0.673171406823057
20 152 translational initiation
GO:0022415 2.10250796483243e-24 33.4928475867037 1.15147740640786
23 260 viral reproductive process
GO:0072594 1.18613646303371e-23 37.9062142584827 0.903466888104629
21 204 establishment of protein localization to organelle
GO:0000184 2.09800801047902e-23 55.3482565647869 0.527022351394367
18 119 nuclear-transcribed mRNA catabolic process, nonsense-mediated decay
GO:0043624 5.45992282597877e-23 44.9099502487562 0.677600166078472
19 153 cellular protein complex disassembly
GO:0043241 1.02976878829302e-22 43.2792965627498 0.699743962355546
19 158 protein complex disassembly
GO:0000956 3.86304711295922e-22 40.0745185185185 0.74846031416511
19 169 nuclear-transcribed mRNA catabolic process
GO:0034623 6.83337010906345e-22 38.7681720430108 0.770604110442184
19 174 cellular macromolecular complex disassembly
GO:0006402 1.0648771015588e-21 37.782250174703 0.788319147463843 19
178 mRNA catabolic process
GO:0032984 1.18776931445319e-21 37.5434722222222 0.792747906719258
19 179 macromolecular complex disassembly
GO:0016032 1.53748871462114e-21 21.0078849163356 1.9973704241921 25
451 viral reproduction
GO:0006401 1.64547942256295e-20 32.2364396654719 0.907895647360044
19 205 RNA catabolic process
GO:0071845 3.41452377745716e-19 24.3164600550964 1.25776762853782
20 284 cellular component disassembly at cellular level
GO:0022411 4.50722596817026e-19 23.9467435549525 1.27548266555948
20 288 cellular component disassembly
GO:0033365 4.69806337960388e-16 15.0563387528298 2.09480312781122
21 473 protein localization to organelle

GO:0006605 4.9014825936731e-16 15.02202371785 2.09923188706664 21
474 protein targeting
GO:0042274 1.02171131577435e-06 68.4428571428571 0.0797176665974673
4 18 ribosomal small subunit biogenesis
GO:0022904 4.03401597916559e-06 16.4333333333333 0.425160888519826
6 96 respiratory electron transport chain
Tissue: Artery_Tibial=>Heart_Left_Ventricle Type: asymmetric
SourceGene: RP11-330O11.2(ENSG00000237135.1)
TargetGeneSet: LRRC47 RP11 KPNA7 MTMR9LP PKP1 NDUFB3 GRIP2 QRICH1
C3orf18 PSMC1P1 ZDHHC23 FGF12 NDUFS6 PAK1IP1 NUDT3 COX7A2 CYCS MRPS24
PKD1L1 ATP5J2 HEMGN GLE1 USMG5 NUMA1 SCARNA11 TMBIM6 PYGL NPC2
TECPR2 CHTF18 NDUFB10 PARN ELAC2 EFTUD2 ATP5G1 RNMT SNRPD1 CYP2T2P
TRAPPC6A ERCC1 NDUFA3 MKKS CSTF1 ATP5J C22orf32 LMF2
ZMYM3 NDUFA1
GOBPID Pvalue OddsRatio ExpCount Count Size Term
GO:0022904 1.39504059708167e-12 54.8965517241379 0.239152999792402
9 96 respiratory electron transport chain
GO:0022900 2.9432682968323e-11 38.1066666666667 0.333817728876894
9 134 electron transport chain
GO:0045333 4.97110600141933e-11 35.7944862155388 0.353747145526261
9 142 cellular respiration
GO:0015980 2.5219447117279e-09 18.2828282828283 0.764791363919452
10 307 energy derivation by oxidation of organic compounds
GO:0006120 2.56186638236061e-08 74.8387096774194 0.0896823749221507
5 36 mitochondrial electron transport, NADH to ubiquinone
GO:0006091 5.96347794793088e-08 12.8790945896209 1.06622379074113
10 428 generation of precursor metabolites and energy
GO:0042773 1.02206716530266e-07 55.1958525345622 0.11708532281503
5 47 ATP synthesis coupled electron transport
GO:0042775 1.02206716530266e-07 55.1958525345622 0.11708532281503
5 47 mitochondrial ATP synthesis coupled electron transport
GO:0006119 2.993531095236e-07 43.7066342057212 0.144488270707909
5 58 oxidative phosphorylation
GO:0042776 3.9917922038184e-06 130.954545454545 0.0323853020552211
3 13 mitochondrial ATP synthesis coupled proton transport
GO:0015985 1.12920216089308e-05 87.2727272727273 0.0448411874610754
3 18 energy coupled proton transport, down electrochemical gradient
GO:0015986 1.12920216089308e-05 87.2727272727273 0.0448411874610754
3 18 ATP synthesis coupled proton transport
GO:0090305 1.73993115539579e-05 30.4152542372881 0.156944156113764
4 63 nucleic acid phosphodiester bond hydrolysis
Tissue: Heart_Left_Ventricle=>Artery_Tibial Type: asymmetric
SourceGene: RP11-385D13.1(ENSG00000251537.2)
TargetGeneSet: RPL11 RP11 RP4 VPS72 PSMB4 IAH1 C2orf28 COX7A2L
BTF3L4P2 RPL32 CMC1 SPCS1 RPL24 NDUFB4 RPL35A WDFY3-AS1
RPL34 CHCHD2P2 BTF3 RPS23 COX7C CTB RPS14 RPS10 MRPL2
PSMB1 NUDT1 DCTN6 RPS20 C8orf59 UQCRB RPL30 VPS13B TTC35 NSMCE2
RPS24 RPL27A RPS13 TSPAN18 MRPL11 ATP5L RPS25 PFDN5 ATP5G2 SLC25A3
MAPKBP1 C16orf13 RPS15A TMEM219 RPL26 RPL17 CTD RPS15 RPL36
C19orf53 EIF3K SNRPD2 ATP5O CXorf31 UXT RPS4X
GOBPID Pvalue OddsRatio ExpCount Count Size Term
GO:0006614 2.78776767256928e-33 132.583333333333 0.348764791363919
21 105 SRP-dependent cotranslational protein targeting to membrane
GO:0006613 3.47026825666604e-33 131.014379084967 0.352086360805481
21 106 cotranslational protein targeting to membrane
GO:0045047 3.47026825666604e-33 131.014379084967 0.352086360805481
21 106 protein targeting to ER
GO:0072599 3.47026825666604e-33 131.014379084967 0.352086360805481
21 106 establishment of protein localization to endoplasmic reticulum
GO:0006415 1.4981523225137e-32 144.185110663984 0.302262819182064
20 91 translational termination
GO:0070972 4.06470290988334e-32 114.710194730813 0.391945194104214
21 118 protein localization to endoplasmic reticulum

GO:0006414	3.52471816938722e-31	120.319327731092	0.348764791363919	20	105	translational elongation
GO:0000184	5.34369164096264e-30	103.203463203463	0.395266763545775	20	119	nuclear-transcribed mRNA catabolic process, nonsense-mediated decay
GO:0006413	1.21526158742964e-29	84.7362171331637	0.504878555117293	21	152	translational initiation
GO:0006612	1.40606205506072e-29	84.0883838383838	0.508200124558854	21	153	protein targeting to membrane
GO:0019080	8.58322204781122e-28	77.8189749182116	0.501556985675732	20	151	viral genome expression
GO:0019083	8.58322204781122e-28	77.8189749182116	0.501556985675732	20	151	viral transcription
GO:0043624	1.13276567279339e-27	76.6380236305048	0.508200124558854	20	153	cellular protein complex disassembly
GO:0043241	2.22867395160935e-27	73.8354037267081	0.52480797176666	20	158	protein complex disassembly
GO:0072594	7.82600038625716e-27	60.4371584699454	0.677600166078472	21	204	establishment of protein localization to organelle
GO:0000956	9.1346235840105e-27	68.3317353787153	0.561345235623832	20	169	nuclear-transcribed mRNA catabolic process
GO:0034623	1.6791184182238e-26	66.0899814471243	0.577953082831638	20	174	cellular macromolecular complex disassembly
GO:0006402	2.69629863716682e-26	64.3987341772152	0.591239360597883	20	178	mRNA catabolic process
GO:0032984	3.0297912127465e-26	63.989218328841	0.594560930039444	20	179	macromolecular complex disassembly
GO:0006401	5.02452753202794e-25	54.8957528957529	0.680921735520033	20	205	RNA catabolic process
GO:0019058	5.82542280477427e-24	48.0433310765064	0.767282541000623	20	231	viral infectious cycle
GO:0022415	6.47182437736391e-23	42.1517857142857	0.863608054805896	20	260	viral reproductive process
GO:0071845	3.8594136907233e-22	38.2548701298701	0.943325721403363	20	284	cellular component disassembly at cellular level
GO:0022411	5.11642205134461e-22	37.6732409381663	0.956611999169608	20	288	cellular component disassembly
GO:0016032	5.84405441841655e-21	27.5621301775148	1.49802781814407	22	451	viral reproduction
GO:0033365	4.14480729927265e-19	24.006145526057	1.57110234585842	21	473	protein localization to organelle
GO:0006605	4.3289800511741e-19	23.9514348785872	1.57442391529998	21	474	protein targeting
GO:0022613	4.53557893831128e-08	15.1570512820513	0.747353124351256	9	225	ribonucleoprotein complex biogenesis
GO:0071843	7.61773941984215e-08	14.2204013377926	0.793855096533112	9	239	cellular component biogenesis at cellular level
GO:0042274	3.168047146843e-07	93.4350649350649	0.0597882499481005	4	18	ribosomal small subunit biogenesis
GO:0042254	5.55893644049581e-07	17.1465132256956	0.49491384679261	7	149	ribosome biogenesis
GO:0022904	7.19167981058667e-07	22.7190476190476	0.318870666389869	6	96	respiratory electron transport chain
GO:0006364	1.15384517465125e-06	20.8527696793003	0.345443221922358	6	104	rRNA processing
GO:0016072	1.60448419455537e-06	19.6414835164835	0.365372638571725	6	110	rRNA metabolic process
GO:0022900	5.06430411526244e-06	15.9319196428571	0.445090305169192	6	134	electron transport chain
GO:0045333	7.07551797530415e-06	14.9863445378151	0.471662860701682	6	142	cellular respiration
GO:0042273	7.40932461213239e-06	106.622222222222	0.0398588332987337	3	12	ribosomal large subunit biogenesis

Tissue: Heart_Left_Ventricle=>Artery_Tibial Type: cluster

SourceGene: RP11-385D13.1(ENSG00000251537.2)
TargetGeneSet: RPL11 RP11 RP4 VPS72 PSMB4 IAH1 C2orf28 COX7A2L
BTF3L4P2 RPL32 CMC1 SPCS1 RPL24 NDUFB4 RPL35A WDFY3-AS1
RPL34 CHCHD2P2 BTF3 RPS23 COX7C CTB RPS14 RPS10 MRPL2
PSMB1 NUDT1 DCTN6 RPS20 C8orf59 UQCRB RPL30 VPS13B TTC35 NSMCE2
RPS24 RPL27A RPS13 TSPAN18 MRPL11 ATP5L RPS25 PFDN5 ATP5G2 SLC25A3
MAPKBP1 C16orf13 RPS15A TMEM219 RPL26 RPL17 CTD RPS15 RPL36
C19orf53 EIF3K SNRPD2 ATP5O CXorf31 UXT RPS4X
GOBPID Pvalue OddsRatio ExpCount Count Size Term
GO:0006614 2.78776767256928e-33 132.583333333333 0.348764791363919
21 105 SRP-dependent cotranslational protein targeting to membrane
GO:0006613 3.47026825666604e-33 131.014379084967 0.352086360805481
21 106 cotranslational protein targeting to membrane
GO:0045047 3.47026825666604e-33 131.014379084967 0.352086360805481
21 106 protein targeting to ER
GO:0072599 3.47026825666604e-33 131.014379084967 0.352086360805481
21 106 establishment of protein localization to endoplasmic reticulum
GO:0006415 1.4981523225137e-32 144.185110663984 0.302262819182064
20 91 translational termination
GO:0070972 4.06470290988334e-32 114.710194730813 0.391945194104214
21 118 protein localization to endoplasmic reticulum
GO:0006414 3.52471816938722e-31 120.319327731092 0.348764791363919
20 105 translational elongation
GO:0000184 5.34369164096264e-30 103.203463203463 0.395266763545775
20 119 nuclear-transcribed mRNA catabolic process, nonsense-mediated decay
GO:0006413 1.21526158742964e-29 84.7362171331637 0.504878555117293
21 152 translational initiation
GO:0006612 1.40606205506072e-29 84.0883838383838 0.508200124558854
21 153 protein targeting to membrane
GO:0019080 8.58322204781122e-28 77.8189749182116 0.501556985675732
20 151 viral genome expression
GO:0019083 8.58322204781122e-28 77.8189749182116 0.501556985675732
20 151 viral transcription
GO:0043624 1.13276567279339e-27 76.6380236305048 0.508200124558854
20 153 cellular protein complex disassembly
GO:0043241 2.22867395160935e-27 73.8354037267081 0.52480797176666
20 158 protein complex disassembly
GO:0072594 7.82600038625716e-27 60.4371584699454 0.677600166078472
21 204 establishment of protein localization to organelle
GO:0000956 9.1346235840105e-27 68.3317353787153 0.561345235623832
20 169 nuclear-transcribed mRNA catabolic process
GO:0034623 1.6791184182238e-26 66.0899814471243 0.577953082831638
20 174 cellular macromolecular complex disassembly
GO:0006402 2.69629863716682e-26 64.3987341772152 0.591239360597883
20 178 mRNA catabolic process
GO:0032984 3.0297912127465e-26 63.989218328841 0.594560930039444 20
179 macromolecular complex disassembly
GO:0006401 5.02452753202794e-25 54.8957528957529 0.680921735520033
20 205 RNA catabolic process
GO:0019058 5.82542280477427e-24 48.0433310765064 0.767282541000623
20 231 viral infectious cycle
GO:0022415 6.47182437736391e-23 42.1517857142857 0.863608054805896
20 260 viral reproductive process
GO:0071845 3.8594136907233e-22 38.2548701298701 0.943325721403363
20 284 cellular component disassembly at cellular level
GO:0022411 5.11642205134461e-22 37.6732409381663 0.956611999169608
20 288 cellular component disassembly
GO:0016032 5.84405441841655e-21 27.5621301775148 1.49802781814407
22 451 viral reproduction
GO:0033365 4.14480729927265e-19 24.006145526057 1.57110234585842 21
473 protein localization to organelle
GO:0006605 4.3289800511741e-19 23.9514348785872 1.57442391529998
21 474 protein targeting

GO:0022613	4.53557893831128e-08	15.1570512820513	0.747353124351256
9	225	ribonucleoprotein complex biogenesis
GO:0071843	7.61773941984215e-08	14.2204013377926	0.793855096533112
9	239	cellular component biogenesis at cellular level
GO:0042274	3.168047146843e-07	93.4350649350649	0.0597882499481005
4	18	ribosomal small subunit biogenesis
GO:0042254	5.55893644049581e-07	17.1465132256956	0.49491384679261
7	149	ribosome biogenesis
GO:0022904	7.19167981058667e-07	22.7190476190476	0.318870666389869
6	96	respiratory electron transport chain
GO:0006364	1.15384517465125e-06	20.8527696793003	0.345443221922358
6	104	rRNA processing
GO:0016072	1.60448419455537e-06	19.6414835164835	0.365372638571725
6	110	rRNA metabolic process
GO:0022900	5.06430411526244e-06	15.9319196428571	0.445090305169192
6	134	electron transport chain
GO:0045333	7.07551797530415e-06	14.9863445378151	0.471662860701682
6	142	cellular respiration
GO:0042273	7.40932461213239e-06	106.622222222222	0.0398588332987337
3	12	ribosomal large subunit biogenesis
Tissue: Heart_Left_Ventricle=>Artery_Tibial	Type:	cluster
SourceGene:	RP11-6O2.3(ENSG00000261616.1)
TargetGeneSet:	MEGF6	ATPIF1	SNORA16A	SNRNP40	TCHH	EIF5B	PAX8
FMNL2	EEF1B2	RPL37A	RPL32	LSM3	KIF9	RP11	CCDC72	MRPS18C	LARP7
RPL37	RPS23	CTB	MRPL2	C6orf203	C7orf50	TOMM7	CPVL	TRGV10	ECD
ATP5J2	PMS2P1	SSBP1	BRF2	NDUFB9	LINC00032	FXN	RPL35	MRC1
RPL13AP5	IMMP1L	MS4A7	CTD	FAU	BARX2	PSMC1P9	SAP18	RPL13AP25
STK24-AS1	RPS29	SLIRP	NDUFB1	IGHV3-7	HMGN2P5	MEFV	UBE2MP1	MTHFSD
TRAPPC2L	RPL38	PPP4R1	SERPINB7	C19orf70	COX6B1	NDUFA3
PSMF1	RPS21	SNRPD3	POLR2F	TOMM22	RRP7A	PARVG	RP6	EEF1B2P3
RGAG1	NDUFA1	RP13
GOBPID	Pvalue	OddsRatio	ExpCount	Count	Size	Term
GO:0006414	3.98314419680397e-12	35.0134638922889	0.385094457130994
10	105	translational elongation
GO:0000956	2.01102768541235e-11	23.6045810729355	0.619818697667981
11	169	nuclear-transcribed mRNA catabolic process
GO:0006402	3.53363687110955e-11	22.3183632734531	0.652826793993495
11	178	mRNA catabolic process
GO:0006415	3.66938032653629e-11	35.7106430155211	0.333748529513528
9	91	translational termination
GO:0006614	1.35366372521252e-10	30.4730113636364	0.385094457130994
9	105	SRP-dependent cotranslational protein targeting to membrane
GO:0006613	1.4751918093616e-10	30.156747891284	0.388762023389385	9
106	cotranslational protein targeting to membrane
GO:0045047	1.4751918093616e-10	30.156747891284	0.388762023389385	9
106	protein targeting to ER
GO:0072599	1.4751918093616e-10	30.156747891284	0.388762023389385	9
106	establishment of protein localization to endoplasmic reticulum
GO:0019080	1.52571484891548e-10	23.5147616691407	0.553802505016954
10	151	viral genome expression
GO:0019083	1.52571484891548e-10	23.5147616691407	0.553802505016954
10	151	viral transcription
GO:0072594	1.53781885538094e-10	19.2764618800888	0.748183516711646
11	204	establishment of protein localization to organelle
GO:0006401	1.62064477386812e-10	19.1757486499755	0.751851082970037
11	205	RNA catabolic process
GO:0006413	1.62871361935406e-10	23.3475270226007	0.557470071275344
10	152	translational initiation
GO:0006612	1.73786559763246e-10	23.1826313221662	0.561137637533735
10	153	protein targeting to membrane
GO:0070972	3.8857989319948e-10	26.8142201834862	0.43277281849007
9	118	protein localization to endoplasmic reticulum
GO:0000184	4.19215804296945e-10	26.5685950413223	0.43644038474846

9	119	nuclear-transcribed mRNA catabolic process, nonsense-mediated decay
GO:0043624	3.93107983477693e-09	20.2471590909091	0.561137637533735
9	153	cellular protein complex disassembly
GO:0043241	5.21875967628323e-09	19.5608602806589	0.579475468825687
9	158	protein complex disassembly
GO:0019058	9.58092578263479e-09	14.9184468062717	0.847207805688188
10	231	viral infectious cycle
GO:0034623	1.21573298519984e-08	17.6442148760331	0.638156528959934
9	174	cellular macromolecular complex disassembly
GO:0032984	1.5565729221242e-08	17.1192513368984	0.656494360251886
9	179	macromolecular complex disassembly
GO:0022415	2.95291480224285e-08	13.1609302325581	0.95356722718151
10	260	viral reproductive process
GO:0016032	5.65316012502317e-07	8.30833333333333	1.65407238253408
11	451	viral reproduction
GO:0071845	8.00505984618337e-07	10.5047107438017	1.04158881738288
9	284	cellular component disassembly at cellular level
GO:0045333	8.05893126774758e-07	16.0774557165862	0.52079440869144
7	142	cellular respiration
GO:0022411	8.99494055057545e-07	10.3511730205279	1.05625908241644
9	288	cellular component disassembly
GO:0033365	9.04296479496618e-07	7.90022675736961	1.73475884021867
11	473	protein localization to organelle
GO:0006605	9.23262607950261e-07	7.88259796359148	1.73842640647706
11	474	protein targeting
GO:0022904	1.30995586247285e-06	20.2950354609929	0.352086360805481
6	96	respiratory electron transport chain
GO:0006119	2.16324166921229e-06	28.1937893081761	0.212718842986645
5	58	oxidative phosphorylation
GO:0006120	8.7062961231538e-06	36.6479591836735	0.132032385302055
4	36	mitochondrial electron transport, NADH to ubiquinone
GO:0022900	9.12150819894347e-06	14.2320478723404	0.491453878624317
6	134	electron transport chain
Tissue: Heart_Left_Ventricle=>Artery_Tibial	Type:	asymmetric
SourceGene:	RP11-881M11.4(ENSG00000256196.1)
TargetGeneSet:	RP1	PPIH	RP11	RP4	HSD3BP5	MRPS21	PSMB4	S100A6	PMF1
ACBD6	DHX9	NENF	RPS7	ROCK2	GTF3C2	MCEE	EXOC6B	DGUOK	RNF181
RPL31	MAP4K4	SMARCAL1		HDLBP	THUMPD3	RPL32	TBC1D5	THRB-IT1
TMEM89	VPRBP	SPCS1	NDUFB4	ZBTB38	UBQLN4P1		UBE2V1P2		RPL35A
TMEM128	RPL34	EXOSC9	NDUFC1	FRG1	CDK7	COL4A3BP		CTD	MAN2A1	PDF
ATOX1	RPL26L1	HIVEP1	APOM	CSNK2B	TAF11	PEX6	MRPL2	C6orf72	ARID1B
C6orf70	PSMB1	CHCHD2	SBDS	NSUN5	TRRAP	C7orf59	PMS2P1	FIS1	SH2B2
ST7-AS1	ARF5	KAT6A	C8orf40	RPL10AP2		CHCHD7	C8orf59	RBM12B	DCTN3
SEC61B	STX17	AKAP2	KIF20B	FAM204A	METTL10	TMEM80	RPL27A	RPS13	POLR2G
OTUB1	KLC2	GAL	ATP5L	DAZAP2	WIBG	SSH1	RPS7P10	MED4-AS1
FANCM	RPS29	TCF12	COMMD4	CPEB1	KIF22	DDX19B	ZNF18	C17orf108
PGAP3	RPL27	MRPS23	BRIP1	C17orf90		C18orf21		C19orf79		BRD4
ZNF226	SNRPD2	RPS11	C19orf48		ZNF611	NLRP9	TRAPPC2	ZNF530	ZNF552
ZNF256	MT1P3	MMP24	SNHG11	IFT52	SLC35C2	USP16	IGLV1-41		EBP	RBM3
GOBPID	Pvalue	OddsRatio	ExpCount	Count	Size	Term
GO:0006614	1.01827296410455e-13	25.518849752339	0.668465850114179	13
105	SRP-dependent cotranslational protein targeting to membrane
GO:0006613	1.15469488928884e-13	25.2426840887437	0.674832191543838
13	106	cotranslational protein targeting to membrane
GO:0045047	1.15469488928884e-13	25.2426840887437	0.674832191543838
13	106	protein targeting to ER
GO:0072599	1.15469488928884e-13	25.2426840887437	0.674832191543838
13	106	establishment of protein localization to endoplasmic reticulum
GO:0070972	4.74744411074256e-13	22.3389993972272	0.751228288699744
13	118	protein localization to endoplasmic reticulum
GO:0019080	6.29003231242625e-13	18.632603406326	0.961317555878486	14
151	viral genome expression
GO:0019083	6.29003231242625e-13	18.632603406326	0.961317555878486	14

151 viral transcription
GO:0000184 1.15281512984895e-11 19.9794392523364 0.757594630129403
12 119 nuclear-transcribed mRNA catabolic process, nonsense-mediated decay
GO:0006415 1.175221973048e-11 24.2390432098765 0.579337070098955
11 91 translational termination
GO:0006612 1.36550618939728e-11 16.7131103074141 0.974050238737804
13 153 protein targeting to membrane
GO:0072594 3.82294072612714e-11 13.385020242915 1.2987336516504 14 204
establishment of protein localization to organelle
GO:0000956 4.82346741374753e-11 14.9820675105485 1.07591170161235
13 169 nuclear-transcribed mRNA catabolic process
GO:0006414 5.7552382341731e-11 20.6087470449173 0.668465850114179
11 105 translational elongation
GO:0006402 9.26646282702262e-11 14.1558879938627 1.13320877447927
13 178 mRNA catabolic process
GO:0019058 2.00447753500612e-10 11.697270471464 1.47062487025119 14
231 viral infectious cycle
GO:0043624 2.25052217805629e-10 15.1255319148936 0.974050238737804
12 153 cellular protein complex disassembly
GO:0043241 3.27283440030383e-10 14.602397260274 1.0058819458861 12 158
protein complex disassembly
GO:0006401 5.37465029970302e-10 12.1420754219409 1.30509999308006
13 205 RNA catabolic process
GO:0022415 9.47557506538276e-10 10.2971648947259 1.6552487717113 14
260 viral reproductive process
GO:0034623 9.98440746345093e-10 13.1453703703704 1.10774340876064
12 174 cellular macromolecular complex disassembly
GO:0032984 1.38225142147504e-09 12.7473053892216 1.13957511590893
12 179 macromolecular complex disassembly
GO:0016032 3.06268020579448e-09 7.2726574500768 2.87121998477614 17
451 viral reproduction
GO:0006413 3.13538362690941e-09 13.6938972068996 0.967683897308145
11 152 translational initiation
GO:0071845 2.3556966040621e-07 7.76856617647059 1.80804096602311
12 284 cellular component disassembly at cellular level
GO:0022411 2.73821998260101e-07 7.65380434782609 1.83350633174175
12 288 cellular component disassembly
GO:0033365 1.62631130964055e-06 5.43545053348975 3.01127949622863
14 473 protein localization to organelle
GO:0006605 1.66720723715982e-06 5.42324414715719 3.01764583765829
14 474 protein targeting
Tissue: Heart_Left_Ventricle=>Artery_Tibial Type: cluster
SourceGene: RP4-631H13.6(ENSG00000230953.1)
TargetGeneSet: RPL22 RPL11 TRAPPC3 MED8 RP11 RP4 CTSS RPS27 DPM3
RPS7 UBC MITD1 RAMP1 RPL29 KCTD6 RPL35A TACC3 GRPEL1 RPL34
RPS3A ZCCHC9 UBE2D2 CTB HMMR PRELID1 PRL C2 RPS10 RPL10A RPF2
RPS3AP26 C7orf59 FNTA FBXL6 RPS6 TUSC1 SWI5 RPS3AP5 KAZALD1
FBXL15 C10orf137 RPL27A RPS13 FAU CCDC85B TMEM126A EMG1 RPL6
LINC00417 POMP ERH FMN1 RPS3AP6 NUBP2 RPS15A NSMCE1 BCL7C CTU2
RPL26 RPL23A RPL17 KRT13 GOSR2 CTD RPL36 RYR1 RPS16 RPL18
ZNF133 RANBP1 UXT
GOBPID Pvalue OddsRatio ExpCount Count Size Term
GO:0006415 3.68277265270592e-36 127.479102167183 0.384125666043872
23 91 translational termination
GO:0006414 1.48312179992786e-34 105.611039794608 0.443221922358314
23 105 translational elongation
GO:0006614 1.48312179992786e-34 105.611039794608 0.443221922358314
23 105 SRP-dependent cotranslational protein targeting to membrane
GO:0006613 1.88930212116187e-34 104.331325301205 0.447443083523632
23 106 cotranslational protein targeting to membrane
GO:0045047 1.88930212116187e-34 104.331325301205 0.447443083523632
23 106 protein targeting to ER
GO:0072599 1.88930212116187e-34 104.331325301205 0.447443083523632

23	106	establishment of protein localization to endoplasmic reticulum
GO:0070972	2.86047310536738e-33	91.0761772853186	0.498097017507439
23	118	protein localization to endoplasmic reticulum
GO:0000184	3.53679202202736e-33	90.1211622807018	0.502318178672756
23	119	nuclear-transcribed mRNA catabolic process, nonsense-mediated decay
GO:0019080	1.2975108437028e-30	67.4395559210526	0.637395335962909
23	151	viral genome expression
GO:0019083	1.2975108437028e-30	67.4395559210526	0.637395335962909
23	151	viral transcription
GO:0006413	1.52496023742732e-30	66.9120767033864	0.641616497128226
23	152	translational initiation
GO:0006612	1.79019432167935e-30	66.3927125506073	0.645837658293544
23	153	protein targeting to membrane
GO:0043624	1.79019432167935e-30	66.3927125506073	0.645837658293544
23	153	cellular protein complex disassembly
GO:0043241	3.92432840569913e-30	63.911306042885 0.66694346412013	23
158	protein complex disassembly
GO:0000956	2.01266739516958e-29	59.0504686373468	0.71337623693862
23	169	nuclear-transcribed mRNA catabolic process
GO:0072594	3.72057301998404e-29	51.2072072072072	0.861116877724725
24	204	establishment of protein localization to organelle
GO:0034623	4.07350845599307e-29	57.0751132798885	0.734482042765207
23	174	cellular macromolecular complex disassembly
GO:0006402	7.04843933636908e-29	55.5865874363328	0.751366687426476
23	178	mRNA catabolic process
GO:0032984	8.06702076175517e-29	55.226383265857 0.755587848591793	23
179	macromolecular complex disassembly
GO:0006401	2.07564556529311e-27	47.2504337767496	0.865338038890042
23	205	RNA catabolic process
GO:0019058	3.51439709011963e-26	41.268471659919 0.975088229188292	23
231	viral infectious cycle
GO:0071845	1.2870944248384e-25	35.2515592515593	1.19880977095011
24	284	cellular component disassembly at cellular level
GO:0022411	1.80817577907636e-25	34.7076167076167	1.21569441561138
24	288	cellular component disassembly
GO:0022415	5.64206211747876e-25	36.1446813235621	1.09750190298249
23	260	viral reproductive process
GO:0033365	5.24664523475711e-23	23.1714924896133	1.99660923119507
26	473	protein localization to organelle
GO:0006605	5.53778955384153e-23	23.118112244898 2.00083039236039	26
474	protein targeting
GO:0016032	8.15064581497228e-21	21.2109627191594	1.90374368555809
24	451	viral reproduction
GO:0006364	2.58623905985937e-07	19.1009927453226	0.439000761192997
7	104	rRNA processing
GO:0016072	3.79862200443738e-07	17.9807623157138	0.464327728184901
7	110	rRNA metabolic process
GO:0042274	8.41122338662158e-07	72.0601503759399	0.075980900975711
4	18	ribosomal small subunit biogenesis
GO:0034470	1.83839607117553e-06	11.1619496855346	0.844232233063456
8	200	ncRNA processing
GO:0042254	2.94517320801079e-06	13.0067814293166	0.628953013632275
7	149	ribosome biogenesis
GO:0022613	4.41542322260067e-06	9.85862098947918	0.949761262196388
8	225	ribonucleoprotein complex biogenesis
GO:0071843	6.88629834733271e-06	9.25198072367884	1.00885751851083
8	239	cellular component biogenesis at cellular level
GO:0042273	1.53240389507124e-05	82.6494252873563	0.0506539339838073
3	12	ribosomal large subunit biogenesis
Tissue: Heart_Left_Ventricle=>Artery_Tibial	Type:	cluster
SourceGene:	SGK3(ENSG00000104205.7)
TargetGeneSet:	SSU72	RPL22	RPL11	TRNAU1AP	SERINC2 RP1	RP11
MAGOH	RP4	MRPS21	RPS27	SNRPE	ARF1	RPL35P1 FTH1P3	UBC	DGUOK

RPL31 MZT2B ARPC4 RPL32 RPL14 RPL10AP6 SENP7 RPL24 RPL35A
RPL34 BTF3 TBCA RPS23 ATG12 CTD RPS14 RDBP RPS18 RPS10
TAF11 RPL10A MRPS12 RPA3 TOMM7 C7orf59 RPS20 C8orf59 RPL30 SEC61B
RPL13AP5 BUB3 RPL27A C11orf58 RPS13 C11orf10 FAU
MRPL11 RPS25 C12orf57 PFDN5 CCDC59 RPL18AP3 GPN3 SRSF9
ARL6IP4 KIAA1704 RPL13AP25 COMMD6 PPP2R3C RPS29 RPL36A ERH
RPL7AP6 DYNC1H1 RSL24D1 RPLP1 IST1 RPS7P1 RPL17 RPL19 RPL27 ACBD4
RPL38 RPS15 RPL36 FBXL12 WDR83OS RPL18A UBA52 SNRPA RPS19 SNRPD2
RPL18 RPS11 RPS9 RPS5 SLC27A5 MT1P3 RPS21 UXT RRAGB RPS23P8 PIN4
XIST FAM127B

GOBPID	Pvalue	OddsRatio	ExpCount	Count	Size	Term
GO:0006415	1.57455419803483e-63	239.29674796748	0.491177081170853	37	91	translational termination
GO:0006414	5.78022036667412e-63	202.846268656716	0.566742785966369	38	105	translational elongation
GO:0006614	5.78022036667412e-63	202.846268656716	0.566742785966369	38	105	SRP-dependent cotranslational protein targeting to membrane
GO:0006613	8.98587057611901e-63	199.849264705882	0.572140336308906	38	106	cotranslational protein targeting to membrane
GO:0045047	8.98587057611901e-63	199.849264705882	0.572140336308906	38	106	protein targeting to ER
GO:0072599	8.98587057611901e-63	199.849264705882	0.572140336308906	38	106	establishment of protein localization to endoplasmic reticulum
GO:0070972	1.23164253147627e-60	169.729375	0.636910940419348	38	118	protein localization to endoplasmic reticulum
GO:0000184	1.80453127497669e-60	167.622222222222	0.642308490761885	38	119	nuclear-transcribed mRNA catabolic process, nonsense-mediated decay
GO:0019080	5.40186848028664e-58	127.330357142857	0.815030101723064	39	151	viral genome expression
GO:0019083	5.40186848028664e-58	127.330357142857	0.815030101723064	39	151	viral transcription
GO:0006612	1.17324426745036e-55	117.78347826087	0.825825202408138	38	153	protein targeting to membrane
GO:0000956	8.25130701679587e-54	103.281679389313	0.912186007888727	38	169	nuclear-transcribed mRNA catabolic process
GO:0006413	1.0166946493539e-53	111.886744432662	0.820427652065601	37	152	translational initiation
GO:0043624	1.33724293941541e-53	110.914423885618	0.825825202408138	37	153	cellular protein complex disassembly
GO:0043241	5.10614434635478e-53	106.293892360411	0.852812954120822	37	158	protein complex disassembly
GO:0006402	7.39965128898052e-53	96.5810714285714	0.960763960971559	38	178	mRNA catabolic process
GO:0072594	2.62428925183606e-52	86.1090909090909	1.10110026987752	39	204	establishment of protein localization to organelle
GO:0034623	2.73344903640097e-51	93.7746127826242	0.939173759601412	37	174	cellular macromolecular complex disassembly
GO:0032984	8.70764332879594e-51	90.4409137753349	0.966161511314096	37	179	macromolecular complex disassembly
GO:0006401	2.68274690096029e-50	80.8125748502994	1.10649782022005	38	205	RNA catabolic process
GO:0019058	5.04178657435553e-50	73.859375	1.24683412912601	39	231	viral infectious cycle
GO:0022415	7.01197806148395e-48	64.0361990950226	1.40336308905958	39	260	viral reproductive process
GO:0071845	7.98676431043288e-43	51.6107435568283	1.53290429728046	37	284	cellular component disassembly at cellular level
GO:0022411	1.37307247233716e-42	50.7738800893985	1.55449449865061	37	288	cellular component disassembly
GO:0016032	9.51183752138724e-40	35.7587399154821	2.43429520448412	40	451	viral reproduction
GO:0033365	2.16702394685727e-37	32.1175115207373	2.55304131201993	39	473	protein localization to organelle

GO:0006605	2.35489516309937e-37	32.0413793103448	2.55843886236247
39	474	protein targeting
GO:0022613	5.11558695623592e-14	17.489118691156 1.21444882707079	16
225	ribonucleoprotein complex biogenesis
GO:0071843	1.31113260928741e-13	16.3749457543758	1.29001453186631
16	239	cellular component biogenesis at cellular level
GO:0042254	4.15417956967026e-10	16.9354315379624	0.804235001037991
11	149	ribosome biogenesis
GO:0042274	3.26261657760861e-08	75.6585879873551	0.0971559061656633
5	18	ribosomal small subunit biogenesis
GO:0006364	8.47438320281285e-08	16.9964285714286	0.561345235623832
8	104	rRNA processing
GO:0016072	1.31346588354507e-07	15.9899159663866	0.593730537679053
8	110	rRNA metabolic process
GO:0022618	7.66284453746082e-07	16.0043213828425	0.512767282541001
7	95	ribonucleoprotein complex assembly
GO:0071826	1.08642122355679e-06	15.1385733757383	0.539755034253685
7	100	ribonucleoprotein complex subunit organization
GO:0034660	2.93190961612754e-06	7.53903743315508	1.538301847623	10
285	ncRNA metabolic process
GO:0034470	1.19737078681023e-05	8.44107142857143	1.07951006850737
8	200	ncRNA processing
Tissue: Heart_Left_Ventricle=>Artery_Tibial	Type:	asymmetric
SourceGene:	SRPX2(ENSG00000102359.5)
TargetGeneSet:	CHCHD2P6	SPEN	SDHDP1	SRSF4	MECR	SLC2A1	CRTC2	SDHC
RP4	SLC41A1	RP11	ODC1	ITSN2	OST4	MORN2	COX5B	NDUFB3	LSM3
SETD2	LAMB2	DZIP3	NDUFB4	UBA5	OCIAD1	NDUFC1	C4orf27	CCDC111	PDCD6
NIPBL	UQCRQ	SAP30L	FBXW11	NHP2	HNRNPC	FOXP4	HMGN3	GTF2H5	RPA3
NDUFA4	GNG11	ATP5J2	PMS2P1	UBE2V2	DECR1	COX6C	PLIN2	NDUFB6	YBX1P10 DLG5
NDUFB8	PDZD8	RPLP2	C11orf84	MARK2	MRPL11	C11orf51	INPPL1
CLNS1A	DCUN1D5 MLL	PRDM10	SNRPF	MRPS31	SNRPGP14	C14orf2 MNS1
ZNF609	LARP6	AXIN1	CREBBP	NDUFAB1 C1QBP	ARHGAP44	ATP5G1	PPP1R9B ICT1
SPIRE1	SNRPD1	UQCR11	GNA11	NDUFA11 NDUFA7	DNM2	NDUFB7	NDUFA13 COX6B1
CAPNS1	COX7A1	SAMD4B	ARHGAP35	NDUFA3	CDS2	ITCH	SYS1	PCIF1
ATP5E	MRPL40	TCF20	SYAP1	RPS2P55 PDZD11	COX7B	RPL39	NDUFA1	FAM127A
CETN2	MECP2
GOBPID	Pvalue	OddsRatio	ExpCount	Count	Size	Term
GO:0022904	5.39257369326029e-31	67.1895317930893	0.597882499481005
23	96	respiratory electron transport chain
GO:0022900	2.31735939610556e-27	44.0701895925777	0.834544322192236
23	134	electron transport chain
GO:0045333	9.56187231160494e-27	41.0844098833563	0.884367863815653
23	142	cellular respiration
GO:0015980	1.91663543718354e-21	19.2021276595745	1.91197840979863
25	307	energy derivation by oxidation of organic compounds
GO:0006091	4.36092091650197e-19	14.1065764925373	2.66555947685281
26	428	generation of precursor metabolites and energy
GO:0006120	1.53887312940392e-16	79.8460759493671	0.224205937305377
11	36	mitochondrial electron transport, NADH to ubiquinone
GO:0042773	4.2209206802955e-15	55.4061181434599	0.292713307037575
11	47	ATP synthesis coupled electron transport
GO:0042775	4.2209206802955e-15	55.4061181434599	0.292713307037575
11	47	mitochondrial ATP synthesis coupled electron transport
GO:0006119	5.22075051791067e-14	42.4061405871263	0.361220676769774
11	58	oxidative phosphorylation
Tissue: Heart_Left_Ventricle=>Artery_Tibial	Type:	asymmetric
SourceGene:	TMEM209(ENSG00000146842.11)
TargetGeneSet:	MTND1P23	DVL1	MRPL20	PANK4	CTNNBIP1	SNORA59A
ZBTB17	KHDRBS1 RP11	PSMB2	STK40	RP1	PPIH	PRPF38A RP4	LRRC39
MAGI3	PEX11B	MRPS21	SMG5	EDEM3	IVNS1ABP	MIR4426 NUCKS1	INTS7	NENF
TATDN3	NUP133	TOMM20	PXDN	EIF3FP3 STAMBP	DGUOK	RPL31	MAP4K4	CCDC115
PLEKHB2 FMNL2	UBE2V1	WIPF1	SMARCAL1	RPL37A	RNF25	TTLL4	INHA
HDLBP	THUMPD3 RPL32	GOLGA4	ZNF662	KRBOX1	KIF9	PHF7	SPCS1	RPL10AP6

RPL24 CD200R1 RPL7AP11 FAM86HP ZBTB38 UBQLN4P1 RPL35A ZNF718
ABCA11P MYL5 TMEM128 DCAF16 SLC30A9 MTHFD2L LARP7 PLK4 OTUD4 WWC2 FRG1
FAM105B RPL37 FBXO4 CTD RPS23 PPIP5K2 P4HA2 SEC24A CTB PDF
RPS14 UBLCP1 BRD2 TAF11 RPL10A FTSJD2 MRPL2 GSTA4 RPL39P3 HMGN3
RARS2 RSPH4A TAB2 PHF10 C6orf70 FAM120B TOMM7 PMS2P4 STAG3L4 EIF4H
GTF2IP1 ECD PMS2P1 ST7-AS1 IMPDH1 TMEM209 SSBP1 SLC4A2 KIAA1967
C8orf40 RPL10AP2 CHCHD7 C8orf44 HNRNPA1P4 C8orf59 RPL30 UTP23
NDUFB9 ERMP1 DCTN3 FAM205A C9orf103 RPS10P3 BICD2 INVS CDC26
RPL35 SCAI STXBP1 PPP1R26 CDNF C1DP1 FGFBP3 RPL13AP5 C10orf12
ACADSB METTL10 SIRT3 RPLP2 AP2A2 SWAP70 PLEKHA7 PEX16 C11orf10 FAU
PITPNM1 C11orf51 C2CD3 C11orf1 USP28 DPAGT1 PRKAG1 DAZAP2 PRR13
ATP5G2 WIBG RPL41 RPL14P1 XPOT C12orf34 DIABLO RFC3 CLN5
STK24-AS1 A2LD1 FANCM RPS29 EXD2 ERH GSTZ1 RPL3P4 HMGN2P5
TMEM85 ATPBD4 TYRO3 TP53BP1 GALK2 TCF12 COMMD4 PEAK1 UBE2I ADCY9 PARN
MPV17L KIF22 FUS NUDT21 NAE1 TMEM170A CYB5D2 UPF3AP1 DRG2
LLGL1 FOXO3B C17orf108 RPS7P1 CCT6B RPL17 RPL19 PGAP3 SMARCE1
DHX58 VPS25 RPL27 MRPL10 ANKRD40 BRIP1 RPL38 SLC25A19 ELAC1
CIRBP PNPLA6 NDUFA7 HOOK2 PIK3R2 UBA52 GAPDHS COX6B1 ALKBH6 EIF3K
HNRNPL RPS16 SNRPA RPS19 ZNF155 ZNF226 TRAPPC6A SNRPD2 RPS11
AKT1S1 C19orf48 PPP2R1A NLRP9 ZSCAN22 ZNF446 CHMP2A DTD1 CDK5RAP1
MT1P3 MMP24 C20orf4 SNHG11 IFT52 SLC35C2 RPS21 C21orf119 GCFC1-AS1
U2AF1 CRKL EWSR1 PICK1 CTA RANGAP1 TCF20 EBP RBM3 SUV39H1
RRAGB RPS23P8 CXorf56 FAM122C IDH3G
GOBPID Pvalue OddsRatio ExpCount Count Size Term
GO:0000184 4.4475426789295e-27 25.6325791855204 1.63047539962632
28 119 nuclear-transcribed mRNA catabolic process, nonsense-mediated decay
GO:0006415 1.24960699750726e-24 29.2043232115286 1.24683412912601
24 91 translational termination
GO:0006614 2.1188291150343e-24 25.6015173410405 1.43865476437617
25 105 SRP-dependent cotranslational protein targeting to membrane
GO:0070972 2.12773192742727e-24 23.2675682507583 1.61677392567988
26 118 protein localization to endoplasmic reticulum
GO:0006613 2.74069048104523e-24 25.2836651680582 1.45235623832261
25 106 cotranslational protein targeting to membrane
GO:0045047 2.74069048104523e-24 25.2836651680582 1.45235623832261
25 106 protein targeting to ER
GO:0072599 2.74069048104523e-24 25.2836651680582 1.45235623832261
25 106 establishment of protein localization to endoplasmic reticulum
GO:0006414 5.33902265678678e-23 24.1328224776501 1.43865476437617
24 105 translational elongation
GO:0000956 1.36403264097387e-22 16.4846057571965 2.31554909694831
28 169 nuclear-transcribed mRNA catabolic process
GO:0006402 5.96612376275673e-22 15.4856470588235 2.43886236246627
28 178 mRNA catabolic process
GO:0072594 1.98155304673631e-21 13.8042941673711 2.7951006850737 29
204 establishment of protein localization to organelle
GO:0006413 2.10436781491553e-21 16.9482281284607 2.08262403985883
26 152 translational initiation
GO:0006612 2.5058898393247e-21 16.8135872550815 2.09632551380527
26 153 protein targeting to membrane
GO:0019080 2.9833039674986e-20 16.2021745114231 2.06892256591239
25 151 viral genome expression
GO:0019083 2.9833039674986e-20 16.2021745114231 2.06892256591239
25 151 viral transcription
GO:0006401 3.10642150925915e-20 13.0983050847458 2.80880215902014
28 205 RNA catabolic process
GO:0043624 6.58223766990509e-19 15.1018444266239 2.09632551380527
24 153 cellular protein complex disassembly
GO:0034623 1.05790892337272e-18 13.6788610001164 2.38405646668051
25 174 cellular macromolecular complex disassembly
GO:0043241 1.43125680767102e-18 14.5331960885229 2.16483288353747
24 158 protein complex disassembly
GO:0032984 2.13673782115282e-18 13.2300502965243 2.4525638364127 25

179	macromolecular complex disassembly
GO:0019058	1.03035553385597e-15	9.85394803299848	3.16504048162757
25	231	viral infectious cycle
GO:0022415	1.64760724398278e-14	8.62009592916	3.56238322607432	25
260	viral reproductive process
GO:0006605	6.2895206258191e-14	6.02344218502971	6.49449865061241
32	474	protein targeting
GO:0071845	1.25384141502437e-13	7.8079317963711	3.89121860078887	25
284	cellular component disassembly at cellular level
GO:0022411	1.72368501580996e-13	7.68698213147542	3.94602449657463
25	288	cellular component disassembly
GO:0016032	6.12524478137023e-13	5.86698337292161	6.1793647498443	30
451	viral reproduction
GO:0033365	2.07562715114111e-12	5.56675266043212	6.48079717666598
30	473	protein localization to organelle
Tissue: Heart_Left_Ventricle=>Artery_Tibial	Type:	cluster
SourceGene:	TMEM209(ENSG00000146842.11)
TargetGeneSet:	MTND1P23	DVL1	MRPL20	PANK4	CTNNBIP1	SNORA59A
ZBTB17	KHDRBS1	RP11	PSMB2	STK40	RP1	PPIH	PRPF38A	RP4	LRRC39
MAGI3	PEX11B	MRPS21	SMG5	EDEM3	IVNS1ABP	MIR4426	NUCKS1	INTS7	NENF
TATDN3	NUP133	TOMM20	PXDN	EIF3FP3	STAMBP	DGUOK	RPL31	MAP4K4	CCDC115
PLEKHB2	FMNL2	UBE2V1	WIPF1	SMARCAL1	RPL37A	RNF25	TTLL4	INHA
HDLBP	THUMPD3	RPL32	GOLGA4	ZNF662	KRBOX1	KIF9	PHF7	SPCS1	RPL10AP6
RPL24	CD200R1	RPL7AP11	FAM86HP	ZBTB38	UBQLN4P1	RPL35A	ZNF718
ABCA11P	MYL5	TMEM128	DCAF16	SLC30A9	MTHFD2L	LARP7	PLK4	OTUD4	WWC2	FRG1
FAM105B	RPL37	FBXO4	CTD	RPS23	PPIP5K2	P4HA2	SEC24A	CTB	PDF
RPS14	UBLCP1	BRD2	TAF11	RPL10A	FTSJD2	MRPL2	GSTA4	RPL39P3	HMGN3
RARS2	RSPH4A	TAB2	PHF10	C6orf70	FAM120B	TOMM7	PMS2P4	STAG3L4	EIF4H
GTF2IP1	ECD	PMS2P1	ST7-AS1	IMPDH1	TMEM209	SSBP1	SLC4A2	KIAA1967
C8orf40	RPL10AP2	CHCHD7	C8orf44	HNRNPA1P4	C8orf59	RPL30	UTP23
NDUFB9	ERMP1	DCTN3	FAM205A	C9orf103	RPS10P3	BICD2	INVS	CDC26
RPL35	SCAI	STXBP1	PPP1R26	CDNF	C1DP1	FGFBP3	RPL13AP5	C10orf12
ACADSB	METTL10	SIRT3	RPLP2	AP2A2	SWAP70	PLEKHA7	PEX16	C11orf10	FAU
PITPNM1	C11orf51	C2CD3	C11orf1	USP28	DPAGT1	PRKAG1	DAZAP2	PRR13
ATP5G2	WIBG	RPL41	RPL14P1	XPOT	C12orf34	DIABLO	RFC3	CLN5
STK24-AS1	A2LD1	FANCM	RPS29	EXD2	ERH	GSTZ1	RPL3P4	HMGN2P5
TMEM85	ATPBD4	TYRO3	TP53BP1	GALK2	TCF12	COMMD4	PEAK1	UBE2I	ADCY9	PARN
MPV17L	KIF22	FUS	NUDT21	NAE1	TMEM170A	CYB5D2	UPF3AP1	DRG2
LLGL1	FOXO3B	C17orf108	RPS7P1	CCT6B	RPL17	RPL19	PGAP3	SMARCE1
DHX58	VPS25	RPL27	MRPL10	ANKRD40	BRIP1	RPL38	SLC25A19	ELAC1
CIRBP	PNPLA6	NDUFA7	HOOK2	PIK3R2	UBA52	GAPDHS	COX6B1	ALKBH6	EIF3K
HNRNPL	RPS16	SNRPA	RPS19	ZNF155	ZNF226	TRAPPC6A	SNRPD2	RPS11
AKT1S1	C19orf48	PPP2R1A	NLRP9	ZSCAN22	ZNF446	CHMP2A	DTD1	CDK5RAP1
MT1P3	MMP24	C20orf4	SNHG11	IFT52	SLC35C2	RPS21	C21orf119	GCFC1-AS1
U2AF1	CRKL	EWSR1	PICK1	CTA	RANGAP1	TCF20	EBP	RBM3	SUV39H1
RRAGB	RPS23P8	CXorf56	FAM122C	IDH3G
GOBPID	Pvalue	OddsRatio	ExpCount	Count	Size	Term
GO:0000184	4.4475426789295e-27	25.6325791855204	1.63047539962632
28	119	nuclear-transcribed mRNA catabolic process, nonsense-mediated decay
GO:0006415	1.24960699750726e-24	29.2043232115286	1.24683412912601
24	91	translational termination
GO:0006614	2.1188291150343e-24	25.6015173410405	1.43865476437617
25	105	SRP-dependent cotranslational protein targeting to membrane
GO:0070972	2.12773192742727e-24	23.2675682507583	1.61677392567988
26	118	protein localization to endoplasmic reticulum
GO:0006613	2.74069048104523e-24	25.2836651680582	1.45235623832261
25	106	cotranslational protein targeting to membrane
GO:0045047	2.74069048104523e-24	25.2836651680582	1.45235623832261
25	106	protein targeting to ER
GO:0072599	2.74069048104523e-24	25.2836651680582	1.45235623832261
25	106	establishment of protein localization to endoplasmic reticulum
GO:0006414	5.33902265678678e-23	24.1328224776501	1.43865476437617
24	105	translational elongation

GO:0000956 1.36403264097387e-22 16.4846057571965 2.31554909694831
28 169 nuclear-transcribed mRNA catabolic process
GO:0006402 5.96612376275673e-22 15.4856470588235 2.43886236246627
28 178 mRNA catabolic process
GO:0072594 1.98155304673631e-21 13.8042941673711 2.7951006850737 29
204 establishment of protein localization to organelle
GO:0006413 2.10436781491553e-21 16.9482281284607 2.08262403985883
26 152 translational initiation
GO:0006612 2.5058898393247e-21 16.8135872550815 2.09632551380527
26 153 protein targeting to membrane
GO:0019080 2.9833039674986e-20 16.2021745114231 2.06892256591239
25 151 viral genome expression
GO:0019083 2.9833039674986e-20 16.2021745114231 2.06892256591239
25 151 viral transcription
GO:0006401 3.10642150925915e-20 13.0983050847458 2.80880215902014
28 205 RNA catabolic process
GO:0043624 6.58223766990509e-19 15.1018444266239 2.09632551380527
24 153 cellular protein complex disassembly
GO:0034623 1.05790892337272e-18 13.6788610001164 2.38405646668051
25 174 cellular macromolecular complex disassembly
GO:0043241 1.43125680767102e-18 14.5331960885229 2.16483288353747
24 158 protein complex disassembly
GO:0032984 2.13673782115282e-18 13.2300502965243 2.4525638364127 25
179 macromolecular complex disassembly
GO:0019058 1.03035553385597e-15 9.85394803299848 3.16504048162757
25 231 viral infectious cycle
GO:0022415 1.64760724398278e-14 8.62009592916 3.56238322607432 25
260 viral reproductive process
GO:0006605 6.2895206258191e-14 6.02344218502971 6.49449865061241
32 474 protein targeting
GO:0071845 1.25384141502437e-13 7.8079317963711 3.89121860078887 25
284 cellular component disassembly at cellular level
GO:0022411 1.72368501580996e-13 7.68698213147542 3.94602449657463
25 288 cellular component disassembly
GO:0016032 6.12524478137023e-13 5.86698337292161 6.1793647498443 30
451 viral reproduction
GO:0033365 2.07562715114111e-12 5.56675266043212 6.48079717666598
30 473 protein localization to organelle
Tissue: Heart_Left_Ventricle=>Artery_Tibial Type: asymmetric
SourceGene: TNFSF8(ENSG00000106952.3)
TargetGeneSet: SSR2 DNM3 EIF3FP3 IGKV2-28 GRIP2 BAP1 ACTR8 PARL
LGALS13 MED10 RPS14 C6orf47 TOX EXOSC2 RPL7A RPL13AP5 SF3B2
NFRKB KRT80 ATP5G2 RPLP0 LINC00402 CTD RP11 RPL4 ARNT2
CMTM4 MRPL10 MBD1 GTF2F1 ELOF1 TNPO2 KXD1 CD22 PAK4 RPS16 FBL
SNRPA TOMM40 RPS9 RPS5 U2AF1 PES1 TSR2 CD24P4
GOBPID Pvalue OddsRatio ExpCount Count Size Term
GO:0042254 5.80270567036366e-11 35.2978021978022 0.360874679952944
9 149 ribosome biogenesis
GO:0022613 8.95478012879855e-11 26.4204651162791 0.544944986506124
10 225 ribonucleoprotein complex biogenesis
GO:0006614 1.18585378692479e-10 43.7388316151203 0.254307660369525
8 105 SRP-dependent cotranslational protein targeting to membrane
GO:0006613 1.28051824785142e-10 43.2894935752079 0.25672963808733
8 106 cotranslational protein targeting to membrane
GO:0045047 1.28051824785142e-10 43.2894935752079 0.25672963808733
8 106 protein targeting to ER
GO:0072599 1.28051824785142e-10 43.2894935752079 0.25672963808733
8 106 establishment of protein localization to endoplasmic reticulum
GO:0071843 1.62090198429492e-10 24.7807860262009 0.578852674555394
10 239 cellular component biogenesis at cellular level
GO:0070972 3.04443730394314e-10 38.5346801346801 0.28579337070099
8 118 protein localization to endoplasmic reticulum
GO:0072594 9.59066382228669e-10 25.2443786982249 0.494083454432219

9	204	establishment of protein localization to organelle
GO:0006415	1.80959242668293e-09	42.6547619047619	0.220399972320255
7	91	translational termination
GO:0019080	2.18633765244899e-09	29.5736855736856	0.365718635388554
8	151	viral genome expression
GO:0019083	2.18633765244899e-09	29.5736855736856	0.365718635388554
8	151	viral transcription
GO:0006612	2.42699389846001e-09	29.1616858237548	0.370562590824164
8	153	protein targeting to membrane
GO:0006414	4.96878665174025e-09	36.5255102040816	0.254307660369525
7	105	translational elongation
GO:0000956	5.33051794798591e-09	26.2341844950541	0.409314234309044
8	169	nuclear-transcribed mRNA catabolic process
GO:0006402	8.02117185783308e-09	24.8296296296296	0.431112033769289
8	178	mRNA catabolic process
GO:0000184	1.1940679007949e-08	31.9285714285714	0.288215348418795
7	119	nuclear-transcribed mRNA catabolic process, nonsense-mediated decay
GO:0006401	2.42382829928545e-08	21.3859748072946	0.496505432150024
8	205	RNA catabolic process
GO:0019058	6.12718217428007e-08	18.8579970104634	0.559476852812954
8	231	viral infectious cycle
GO:0006413	6.51431909511458e-08	24.6051724137931	0.368140613106359
7	152	translational initiation
GO:0043624	6.81501319057261e-08	24.4349315068493	0.370562590824164
7	153	cellular protein complex disassembly
GO:0043241	8.50084415069431e-08	23.6175496688742	0.382672479413189
7	158	protein complex disassembly
GO:0033365	1.12931857761601e-07	12.0544276457883	1.14559546052176
10	473	protein localization to organelle
GO:0022415	1.52404299006734e-07	16.6537330981775	0.629714206629299
8	260	viral reproductive process
GO:0034623	1.64550189728571e-07	21.3308383233533	0.421424122898069
7	174	cellular macromolecular complex disassembly
GO:0006364	1.64612882806908e-07	30.2280084447572	0.25188568265172
6	104	rRNA processing
GO:0032984	1.99626642814782e-07	20.703488372093 0.433534011487094	7
179	macromolecular complex disassembly
GO:0016072	2.29963934403525e-07	28.4721485411141	0.26641754895855
6	110	rRNA metabolic process
GO:0006605	1.3448823802972e-06	10.3853598014888	1.14801743823957
9	474	protein targeting
GO:0071845	4.41264252702976e-06	12.7608303249097	0.687841671856619
7	284	cellular component disassembly at cellular level
GO:0022411	4.83831668235229e-06	12.5756227758007	0.697529582727839
7	288	cellular component disassembly
GO:0034470	7.57338935750658e-06	15.1674369001066	0.484395543560999
6	200	ncRNA processing
GO:0016032	9.48070968541251e-06	9.34570688069559	1.09231195073005
8	451	viral reproduction
Tissue: Artery_Tibial=>Muscle_Skeletal	Type:	asymmetric

SourceGene:	ANGPTL1(ENSG00000116194.7)	angiopoietin-like 1

TargetGeneSet: GPR153 PHF13 TMEM201 NMNAT1 RP11 PTAFR ZMYM1 GPX6
FAM159A MYSM1 SH3GLB1 SELE DNM3OS SYT14 SDCCAG8 KCNK3 SUPT7L MAP4K4
NFE2L2 SLC11A1 COL6A3 TIMP4 TMEM43 ABHD14A-ACY1 PTPRG DBR1 TM4SF1
SNORA63 ATP13A4 IL8 G3BP2 NFKB1 GAB1 ZNF827 CLCN3 NLN PIK3R1
ZNF225 HBEGF FGF18 COL23A1 JARID2 FKBP5 PIM1 ENPP4 COQ3 GJA1
PDGFA MAFK KBTBD2 POR LAMB1 PODXL EPHB6 GIMAP6 CTD RNF122
C8orf4 GEM NIPAL2 U6 HAS2 DENND3 GADD45G AIF1L LCN6 GATA3 RIN2
CSTF2T EGR2 KIAA1274 PANK1 CHST15 DAGLA NAA40 RELT FZD4
SLC2A3 MFAP5 TSPAN31 IGF1 TDG GPR133 MRPS31 FOS POMT2 CSPG4
NARFL TMEM204 NPIP EEF2K PALB2 CX3CL1 ZNRF1 TAF1C RP4 RAP1GAP2
CCL2 C1QTNF1 CCDC57 SLC16A3 RNF125 IZUMO4 CYP4F22 HIF3A PPP1R15A
UBOX5 RBM38 JAM2 DGCR2 GGT5 DNAL4 RPA4 CSTF2

GOBPID	Pvalue	OddsRatio	ExpCount	Count	Size	Term
GO:2000147	5.93E-08	10.11642857	1.284893779	11	211	positive regulation of cell motility
GO:0040017	7.90E-08	9.817614424	1.321431043	11	217	positive regulation of locomotion
GO:0051272	8.67E-08	9.721840659	1.333610131	11	219	positive regulation of cellular component movement

GO:0030335	5.00E-07	9.219055057	1.260535603	10	207
positive regulation of cell migration					
GO:0070555	3.47E-06	16.87765539	0.41408899	6	68
response to interleukin-1					
GO:0071347	6.72E-06	22.12542478	0.267939935	5	44
cellular response to interleukin-1					
GO:0050900	1.56E-05	6.939054998	1.467580098	9	241
leukocyte migration					

GO:0043491	1.66E-05	12.58889215	0.541969414	6	89
protein kinase B signaling cascade

GO:0051897	1.87E-05	17.59773789	0.328835375	5	54
positive regulation of protein kinase B signaling cascade

Tissue: Muscle_Skeletal=>Artery_Tibial	Type:	asymmetric

SourceGene:	DNAH11(ENSG00000105877.12)

TargetGeneSet:	MECR	NFASC	MDH1	MXD1	SLC25A12	NDUFB3	EPHA6	RP11
TIMMDC1 HCLS1	TM4SF1-AS1	ZAR1	CCNI	PRDM8	WDFY3-AS2	PCDH10	CTD
SKP2	F2R	FNIP1	UQCRQ	GRIA1	RANBP17 SH3PXD2B	HLA-F-AS1
LAMA4	NDUFA4	C9orf123	TLR4	H2AFY2	KIAA1274	POU6F1	MTIF3
LACC1	TRAV9-2 KIF7	LSMD1	MMP28	SLC14A1 NDUFA7	GMIP	C20orf118
ARFGEF2 ATP5J	LANCL3	PDZD11	COX7B	PCDH11X

GOBPID	Pvalue	OddsRatio	ExpCount	Count	Size	Term

GO:0045333	1.34E-09	31.57987839	0.343920836	8	142
cellular respiration

GO:0022904	2.64E-09	40.24438202	0.232509861	7	96
respiratory electron transport chain

GO:0022900 2.73E-08 28.12795276 0.324545014 7 134
electron transport chain

GO:0015980 5.41E-07 13.98934721 0.743547159 8 307
energy derivation by oxidation of organic compounds

GO:0006091 6.46E-06 9.87372134 1.036606463 8 428
generation of precursor metabolites and energy

Tissue: Artery_Tibial=>Muscle_Skeletal Type: asymmetric

SourceGene: ENSG00000260884.1 AC009120.5 (novel lincRNA)

TargetGeneSet: MTND1P23 RPL22 CELA2A WDTC1 MFI2 KDM4A RP4
RPS27 C1orf192 PRRC2C OBSCN UBC IGKV1-5 RPL31 ORMDL1 EEF1B2
GIGYF2 RAD54L2 RPL29 RPL24 COPG1 RP11 RPL35A BOD1L LGALS13 RPL34
H3F3AP6 RPS3A RPL37 CHCHD2P2 BTF3 NSA2 MAN2A1 CTD CTB
MTND5P11 GFOD1-AS1 RPS18 RPL10A UBR2 MTRNR2L9 MRPS12
FOXK1 FBXL18 HIP1 RPS3AP26 TRIM56 PRSS3P2 RPS20 RPL30 EIF3E
FAM91A1 NFIB PCSK5 FAM35B POLR3A HIF1AN FBXL15 OR52E8 RPL27A MTRNR2L8
RPS13 MS4A1 RPL37P2 C11orf73 MMP1 RPS25 SNX19 MLL4 SPRYD3
PFDN5 VPS29 C12orf51 GCN1L1 RPLP0 RPSAP54 PARP4-AS1 KCTD4 TPT1
ZC3H13 LPAR6 CHD8 U6 C14orf166 IGHD GOLGA8IP HERC2
RPS3AP47 SPG11 POLR2M RPS3AP6 HERC1 RPL4 RPL13 TOM1L2 MTRNR2L1
RPS7P1 RPL23A TBC1D3H RPL27 DHX8 RPS2P48 TNRC6C ROCK1P1 IER3IP1 ZNF737
KCNK6 CEACAM5 SULT2B1 KLK1 RPS5 BFSP1 CEP250 ZNFX1 PEX26 NF2
LDOC1L GNL3L ZC3H12B MED12 RPL36A RPL39 FLNA MT

GOBPID	Pvalue	OddsRatio	ExpCount	Count	Size	Term
GO:0006414	4.81E-34	71.99596774	0.632136184	25	105	translational elongation
GO:0006415	7.10E-34	81.29068941	0.54785136	24	91	translational termination
GO:0000184	1.54E-32	61.21310913	0.716421009	25	119	"nuclear-transcribed mRNA catabolic process, nonsense-mediated decay"
GO:0006614	3.37E-32	67.17460317	0.632136184	24	105	SRP-dependent cotranslational protein targeting to membrane

GO:0006613	4.33E-32	66.35075494	0.638156529	24	106

cotranslational protein targeting to membrane

GO:0045047	4.33E-32	66.35075494	0.638156529	24	106

protein targeting to ER

GO:0072599	4.33E-32	66.35075494	0.638156529	24	106

establishment of protein localization to endoplasmic reticulum

GO:0070972	7.36E-31	57.83181358	0.710400664	24	118

protein localization to endoplasmic reticulum

GO:0006413	1.14E-29	45.20256541	0.915092381	25	152

translational initiation

GO:0006612	1.36E-29	44.84627016	0.921112726	25	153

protein targeting to membrane

GO:0000956	1.87E-28	39.81854839	1.01743824	25	169

nuclear-transcribed mRNA catabolic process

GO:0019080	4.26E-28	42.70566179	0.909072037	24	151

viral genome expression

GO:0019083	4.26E-28	42.70566179	0.909072037	24	151

viral transcription

GO:0043624 5.94E-28 42.03765227 0.921112726 24 153
cellular protein complex disassembly

GO:0006402 7.29E-28 37.45256167 1.071621341 25 178 mRNA
catabolic process

GO:0043241 1.34E-27 40.45486851 0.951214449 24 158
protein complex disassembly

GO:0034623 1.52E-26 36.09904762 1.047539963 24 174
cellular macromolecular complex disassembly

GO:0072594 2.50E-26 31.95395567 1.228150301 25 204
establishment of protein localization to organelle

GO:0006401	2.84E-26	31.77419355	1.234170646	25	205	RNA catabolic process
GO:0032984	3.08E-26	34.92227343	1.077641686	24	179	macromolecular complex disassembly
GO:0019058	6.02E-25	27.71296586	1.390699606	25	231	viral infectious cycle
GO:0022415	1.20E-23	24.24330817	1.565289599	25	260	viral reproductive process

GO:0071845	1.09E-22	21.95945946	1.70977787	25	284

cellular component disassembly at cellular level

GO:0022411	1.55E-22	21.61934257	1.733859248	25	288

cellular component disassembly

GO:0033365	1.46E-19	14.04282511	2.847623002	27	473

protein localization to organelle

GO:0006605	1.54E-19	14.01040268	2.853643346	27	474

protein targeting

GO:0016032	8.53E-18	13.19286688	2.71517542	25	451

viral reproduction

Tissue: Artery_Tibial=>Muscle_Skeletal Type: asymmetric

SourceGene: GAS8(ENSG00000141013.10) growth arrest-specific 8

TargetGeneSet: FAM41C H6PD SNRNP40 IPP ALG6 SGIP1 RP11 GAPDHP32
EFNA4 MSTO1 B4GALT3 HSPA6 POU2F1 TOR1AIP1 ZNF678 JMJD4 RNASEH1
IGKJ4 ARL6IP6 ICA1L CRBN GHRL KIAA1143 MON1A PROS1 TMEM45A
ZDHHC23 CCNL1 H1FX PLD1 DCAF16 SLC30A9 POLR2B ENAM EXOSC9 GARS
FHDC1 LPCAT1 CTD ZFR SKP2 YTHDC2 HNRNPA0 IK GALNT10 NHLRC1
TOB2P1 TRIM39 VPS52 MAPK13 RP1 SCAF8 TATDN2P2 WDR27 AP5Z1
WIPI2 CCDC126 GPC2 SPDYE3 CTA C8orf42 PPP2R2A BRF2 RNF170 KIAA0196
ZNF7 RCL1 SLC44A1 C9orf148 STXBP1 C9orf106 USP20 NUP214
SEC16A FAM21C STOX1 RRP12 C10orf32 PLEKHA1 BET1L ART1 CHRNA10 RIC3
PPP2R5B SART1 ANKRD13D ATG16L2 SRSF8 TTC12 RPL23AP64 NPFF
THAP2 ANO4 PLBD2 FBXO21 TPTE2 CDK8 MTRF1 LINC00452 C14orf21
ZBTB1 ATPBD4 GCHFR ZFYVE19 CLPX U6 FAM103A1 ZNF75A DOC2A FUS
MMP15 SETD6 CMC2 GAS8 PIGS SUPT6H TP53I13 NSRP1 PGAP3 GSDMB
H3F3B ZNF236 C19orf6 CIRBP VMAC TNFSF14 MEF2BNB ZNF681 ZNF30 HNRNPL
MED29 SUPT5H HNRNPUL1 TSKS C19orf48 PPP2R1A LENG8 ZNF324B
C20orf96 FAM110A UBOX5 RP4 CDH4 PRPF6 IGLL5 IGLC1 PES1 H1F0
7SK GSPT2 TSR2 ACRC

GOBPID	Pvalue	OddsRatio	ExpCount	Count	Size	Term

GO:0008380	6.72E-07	6.328650711	2.369247803	13	323	RNA splicing
GO:0006397	1.25E-06	5.517786561	2.926717874	14	399	mRNA processing
GO:0000377	2.47E-06	7.598260309	1.496367033	10	204	"RNA splicing, via transesterification reactions with bulged adenosine as nucleophile"
GO:0000398	2.47E-06	7.598260309	1.496367033	10	204	"nuclear mRNA splicing, via spliceosome"

GO:0000375	3.07E-06	7.404731993	1.533042696	10	209	"RNA splicing, via transesterification reactions"

Tissue: Artery_Tibial=>Muscle_Skeletal	Type:	asymmetric
SourceGene:	KRT8P46(ENSG00000248971.1)
TargetGeneSet:	FAM41C	IPP	RP11	JTB	RABIF	RNASEH1	EMX1	DCAF17	MREG
CRBN	KIAA1143		CTD	UBA3	C3orf58	METTL15P1		PHC3	AFAP1
RG9MTD2	RPS3A	SKP2	TTC37	RAPGEF6	IK	CDKAL1	ZNF192	WDR11	SNX14
USP45	DCBLD1	SCAF8	TATDN2P2		FOXK1	GLCCI1	RALA	TMEM60	C8orf42
PPP2R2A	BRF2	RALGAPA1P		USP20	NUP214	RPL7A	SEC16A	DUPD1	GBF1
SART1	NPFF	THAP2	NOC4L	CDK8	EBPL	ALG11	C14orf21		HERC2
HERC1	FAM108C1		NMB	CCDC113	TOM1L2	SUPT6H	TAOK1	DHX8	SLC16A5	SGSH
METTL4	GNA11	HSH2D	DDA1	RYR1	MED29	SUPT5H	DYRK1B	HNRNPUL1		RP4
TGM2	PRPF6	SON	MCM5	RAB9A	RBM10	SUV39H1	TSR2	APOOL	RPL39
IDH3G
GOBPID	Pvalue	OddsRatio	ExpCount	Count	Size	Term
Tissue: Muscle_Skeletal=>Artery_Tibial	Type:	asymmetric

SourceGene:	MTRNR2L1(ENSG00000256618.1)	MT-RNR2-like 1

TargetGeneSet: MTND2P28 ISG15 DFFA CELA2A CHCHD2P6 EIF1AX RP1 CELA3B CELA3A PHACTR4 YRDC MFI2 CLDN19 AMY2A CLCC1 TARS2 JTB RPS27 VAMP4 ZBTB37 RP11 PIGR PTPN14 PREPL EPCAM CEP68 AAK1 REG1B REG1A REG3A POLR1A EIF5B H3F3AP4 ORMDL1 TRIP12 HDLBP NR2C2 CTD ZBTB20 STAG1 CPB1 MIR943 ABLIM2 FRG1 NUP155 CTB MTND5P11 PCDHGA5 GEMIN5 CLPS FTH1P5 MTRNR2L9 WDR11 PHACTR2 IYD PHF14 STK31 CBX3 NUDCD3 TRIM56 CPA2 CPA1 BRAF PRSS1 PRSS3P1 PRSS3P2 MTMR9 VAT1 ELP3 PRKDC NFIB PRSS3 RGP1 ZNF462 ARPC5L C9orf16 ATOH7 FBXL15 PDCD11 PNLIP SLC22A18AS OR52E8 NAT10 TRIM44 CKAP5 DDB1 DLAT SLC38A1 RP3 DAZAP2 HIGD1AP1 PLA2G1B STRN3 KLHL28 L2HGDH YLPM1 GOLGA8IP USP8 MYO9A IQGAP1 RPL21P119 GP2 LONP2 SLC12A4 NFATC3 CHTF8 CTRB2 CTRB1 VPS53 TSR1 RNASEK MYH1 TAOK1 LIG3 CDC27 KPNB1 DYNLL2 RNF126 ALKBH7 TRAPPC5 ADAMTS10 SYCN LINC00085 RBPJL URB1 CBR3 NBEAP3 FAM108A5P CTA GS1 HUWE1 PAGE2B RPL39 MT

GOBPID	Pvalue	OddsRatio	ExpCount	Count	Size	Term
GO:0007586	7.23E-08	13.78935484	0.769358522	9	109	digestion

Tissue: Muscle_Skeletal=>Artery_Tibial Type: asymmetric

SourceGene: PABPC3(ENSG00000151846.6) "poly(A) binding protein, cytoplasmic 3"

TargetGeneSet: CELA2B ZMYM1 TXNIP GLUL SUCLG1 SLC25A12 NIF3L1 ING5 PDHB GTF2E1 KLF15 C3orf70 RASL11B ARL9 AREG USP53 ARHGAP26 ADRA1B LY6G6C MED20 GAPDHP15 LYRM2 FAXC QRSL1 LAMA4 RP1 RP11 UST FAM103A2P NDUFA4 MEOX2 ACN9 PMS2P1 NEIL2 VDAC3 LAPTM4B DENND3 RANBP6 ALDH1B1 KLF9 TLR4 CISD1 ADO NODAL SLC16A12 FAM118B COPS7A COX14 TSFM IRAK3 TPCN1 ZCCHC8 SFSWAP QRSL1P3 NID2 SLC25A29 ATP10A ARID3B CTD FAM108C1 IL16 IDH2 PER1 NDEL1 COX10 PSMB3 C17orf89 ATP5A1 NDUFA7 NXNL1 ITPKC RBM39 ARFGAP3 KLHL13 NDUFA1

GOBPID	Pvalue	OddsRatio	ExpCount	Count	Size	Term
GO:0045333	1.22E-10	24.01346801	0.540447028	10	142	cellular respiration
GO:0015980	1.73E-08	11.90878378	1.16843125	11	307	energy derivation by oxidation of organic compounds
GO:0006091	5.00E-07	8.380695444	1.628953014	11	428	generation of precursor metabolites and energy
GO:0022904	1.64E-06	19.46394558	0.365372639	6	96	respiratory electron transport chain
GO:0022900	1.13E-05	13.64923469	0.509999308	6	134	electron transport chain

GO:0009060 1.89E-05 29.63467492 0.159850529 4 42
aerobic respiration

Tissue: Artery_Tibial=>Muscle_Skeletal Type: asymmetric

SourceGene: PPIG(ENSG00000138398.10) peptidylprolyl isomerase G (cyclophilin G)

TargetGeneSet: MINOS1 TMSB4XP1 APOA1BP RP4 LBR GNPAT RP11 MRPL33 MDH1 SLC1A4 TPRKB SUCLG1 NDUFB3 COL4A3 GIGYF2 ABHD14A CCNL1 CKMT2 IK MNF1 PHACTR2 WDR27 EEF1A1P6 RPS2P32 CYCS GATS NDUFA5 WASL MRPS33 GSTK1 KIAA0196 ST13P6 ERP44 MRPL41 FAM21C NDUFB8 GBF1 NRAP PLEKHA1 SART1 NDUFS8 C11orf73 SDHD LIN7A NDUFA12 SLC25A3 LINC00441 ARHGEF7-IT1 C14orf21 FOXN3 GLRX5 COX5A NDUFB10 GOT2 CA7 SUPT6H TP53I13 ATP5G1 NDUFV2 CCDC68 ATP5D WDR88 SUPT5H ESF1 PRPF6 ATP5J C21orf33 RAB9A

GOBPID	Pvalue	OddsRatio	ExpCount	Count	Size	Term
GO:0045333	1.19E-18	45.5586295	0.510968099	15	142	cellular respiration
GO:0022904	1.09E-17	57.4939759	0.345443222	13	96	respiratory electron transport chain

GO:0022900 9.67E-16 39.33333333 0.482181164 13 134
electron transport chain

GO:0015980 1.27E-13 19.58580155 1.104698637 15 307
energy derivation by oxidation of organic compounds

GO:0006091 1.04E-12 15.08845739 1.540101031 16 428
generation of precursor metabolites and energy

GO:0006119 2.68E-11 52.17818182 0.20870528 8 58
oxidative phosphorylation

GO:0042773 2.89E-10 55.84055556 0.169123244 7 47 ATP
synthesis coupled electron transport

GO:0042775 2.89E-10 55.84055556 0.169123244 7 47
mitochondrial ATP synthesis coupled electron transport

GO:0006120 2.89E-09 62.47391304 0.129541208 6 36
"mitochondrial electron transport, NADH to ubiquinone"

GO:0042776 1.23E-05 88.09591837 0.04677877 3 13
mitochondrial ATP synthesis coupled proton transport

Tissue: Artery_Tibial=>Muscle_Skeletal Type: asymmetric

SourceGene: PVRL3-AS1(ENSG00000242242.1) PVRL3 antisense RNA 1 (MiscRNA)

TargetGeneSet: PPIH MRPL9 RP11 LRRN2 DYNC2LI1 UBC MPHOSPH10
RPL31 METTL5 RPL37A FAM208A RPL24 RAB6B PARL RPL35A FRYL RPL34
BRIX1 RPL37 MAN2A1 NOL7 PRRC2A NOTCH4 RPL10A C6orf57 L3MBTL3 MRPS12
PNLDC1 PINX1 HOOK3 RPS20 C8orf59 RPL30 POLR2K RPS13 C11orf10
RCOR2 FAU RPS25 ERC1 RPL41 RAP2A ARHGEF7-AS2 HEATR5A ERH
ADAM20 BTBD7 UNC45A FBXL19 GIT1 TMEM106A PPP1R9B SOGA2 C18orf21
SNRPD2 MID1 NKAP RPL7P56 PLXNA3

GOBPID	Pvalue	OddsRatio	ExpCount	Count	Size	Term
GO:0006415	5.35E-21	86.84848485	0.277074251	14	91	translational termination
GO:0006414	4.47E-20	73.41538462	0.319701059	14	105	translational elongation
GO:0006614	4.47E-20	73.41538462	0.319701059	14	105	SRP-dependent cotranslational protein targeting to membrane

GO:0006613	5.15E-20	72.61231884	0.322745831	14	106
cotranslational protein targeting to membrane					
GO:0045047	5.15E-20	72.61231884	0.322745831	14	106
protein targeting to ER					
GO:0072599	5.15E-20	72.61231884	0.322745831	14	106
establishment of protein localization to endoplasmic reticulum					
GO:0019080	1.69E-19	54.27611562	0.45976057	15	151
viral genome expression					
GO:0019083	1.69E-19	54.27611562	0.45976057	15	151
viral transcription					
GO:0043624	2.07E-19	53.482009	0.465850114	15	153
cellular protein complex disassembly					
GO:0070972	2.48E-19	64.18012821	0.359283095	14	118
protein localization to endoplasmic reticulum					
GO:0000184	2.81E-19	63.56444444	0.362327867	14	119
"nuclear-transcribed mRNA catabolic process, nonsense-mediated decay"					

GO:0043241 3.39E-19 51.59392332 0.481073974 15 158
protein complex disassembly

GO:0034623 1.49E-18 46.35003253 0.529790326 15 174
cellular macromolecular complex disassembly

GO:0032984 2.30E-18 44.92115223 0.545014186 15 179
macromolecular complex disassembly

GO:0006413 9.67E-18 48.252657 0.462805342 14 152
translational initiation

GO:0006612 1.06E-17 47.90215827 0.465850114 14 153
protein targeting to membrane

GO:0000956 4.40E-17 42.90924731 0.514566466 14 169
nuclear-transcribed mRNA catabolic process

GO:0006402 9.20E-17 40.52886179 0.541969414 14 178 mRNA
catabolic process

GO:0019058 1.10E-16 33.98227969 0.703342329 15 231
viral infectious cycle

GO:0072594	6.31E-16	34.91894737	0.621133486	14	204	establishment of protein localization to organelle
GO:0022415	6.46E-16	29.89866291	0.791640717	15	260	viral reproductive process
GO:0006401	6.76E-16	34.73368237	0.624178258	14	205	RNA catabolic process
GO:0071845	2.40E-15	27.18497629	0.864715245	15	284	cellular component disassembly at cellular level
GO:0022411	2.96E-15	26.77908299	0.876894333	15	288	cellular component disassembly
GO:0016032	2.07E-12	16.57426447	1.373192167	15	451	viral reproduction
GO:0033365	4.09E-12	15.75327511	1.44017715	15	473	protein localization to organelle
GO:0006605	6.43E-11	14.14913043	1.443221922	14	474	protein targeting

Tissue: Artery_Tibial=>Muscle_Skeletal Type: asymmetric

SourceGene: RAB32(ENSG00000118508.4) "RAB32, member RAS oncogene family"

TargetGeneSet: RP1 UBC RPL31 EEF1B2 RPL37A RP11 RPL35A RPL34 LSM6 CTD MRPS12 RPS20 U6 RPS13 RPS25 PFDN5 RPL41 HERC2 NDNL2 RPS3AP6 MED31 RPL23A RPL27 RPS5 EEF1B2P3

GOBPID	Pvalue	OddsRatio	ExpCount	Count	Size	Term
GO:0006414	1.42E-23	253.2527174	0.152584596	13	105	translational elongation
GO:0006415	5.14E-22	242.2109705	0.132239983	12	91	translational termination
GO:0006614	3.15E-21	205.5483871	0.152584596	12	105	SRP-dependent cotranslational protein targeting to membrane
GO:0006613	3.55E-21	203.3475177	0.154037783	12	106	cotranslational protein targeting to membrane
GO:0045047	3.55E-21	203.3475177	0.154037783	12	106	protein targeting to ER
GO:0072599	3.55E-21	203.3475177	0.154037783	12	106	establishment of protein localization to endoplasmic reticulum
GO:0000956	9.00E-21	148.6875	0.245588541	13	169	nuclear-transcribed mRNA catabolic process
GO:0070972	1.37E-20	180.1761006	0.171476022	12	118	protein localization to endoplasmic reticulum
GO:0000184	1.52E-20	178.4797508	0.172929209	12	119	"nuclear-transcribed mRNA catabolic process, nonsense-mediated decay"

GO:0006402	1.80E-20	140.4886364	0.25866722	13	178	mRNA catabolic process
GO:0006401	1.18E-19	120.5039063	0.297903259	13	205	RNA catabolic process
GO:0019080	2.95E-19	137.0839329	0.219431181	12	151	viral genome expression
GO:0019083	2.95E-19	137.0839329	0.219431181	12	151	viral transcription
GO:0006413	3.20E-19	136.0952381	0.220884368	12	152	translational initiation
GO:0006612	3.47E-19	135.1205674	0.222337554	12	153	protein targeting to membrane
GO:0043624	3.47E-19	135.1205674	0.222337554	12	153	cellular protein complex disassembly
GO:0043241	5.16E-19	130.4474886	0.229603488	12	158	protein complex disassembly
GO:0034623	1.69E-18	117.4320988	0.252854474	12	174	cellular macromolecular complex disassembly
GO:0032984	2.40E-18	113.8762475	0.260120407	12	179	macromolecular complex disassembly
GO:0072594	1.19E-17	98.875	0.296450073	12	204	establishment of protein localization to organelle
GO:0019058	5.41E-17	86.52054795	0.335686112	12	231	viral infectious cycle
GO:0022415	2.27E-16	76.24731183	0.377828524	12	260	viral reproductive process
GO:0071845	6.61E-16	69.40196078	0.412705003	12	284	cellular component disassembly at cellular level

GO:0022411 7.83E-16 68.37681159 0.41851775 12 288
cellular component disassembly

GO:0016032 1.68E-13 42.49354594 0.65538717 12 451
viral reproduction

GO:0033365 2.96E-13 40.40202458 0.687357276 12 473
protein localization to organelle

GO:0006605 3.04E-13 40.31168831 0.688810463 12 474
protein targeting

Tissue: Muscle_Skeletal=>Artery_Tibial Type: asymmetric
SourceGene: RP1-159M24.1(ENSG00000225580.2)
TargetGeneSet: PIK3CD CELA2B RP11 MECR CRTC2 MIR4426 ODC1 KLHL29 RBKS RPLP0P6 MAP4K3 ASB3 TET3 BTF3L4P2 FARSB LSM3 ZNF662 KRBOX1 RPL10AP6 LMOD3 DZIP3 DDX50P2 AASDH NDUFC1 ANXA2P1 CTD GPR150 RNF145 CREBRF BNIP1 SUMO2P1 RPL24P4 RP3 LYRM2 UFL1 PMS2P1 C7orf61 C7orf60 MRPS33 AGAP3 PPP3CC KIF13B TRIM55 NDUFB9 C9orf123 FKTN SYNPO2L RP13 COX15 UROS CHST1 CLNS1A C11orf70 ATP5L SPA17 GPD1 NDUFA12 RPS7P10 C14orf39 NDUFB1 C14orf2 HMGN2P5 BMF FURIN MYLPF CCDC113 PSMB10 PPM1D WDR83 SNRPB2 C20orf43 OGFR COMT PNPLA4 SMPX EEF1B2P3 RPS2P55 ITGB1BP2 COX7B DOCK11 NDUFA1
GOBPID Pvalue OddsRatio ExpCount Count Size Term
GO:0022904 3.5669491973475e-09 26.5417439703154 0.37865891633797 8 96 respiratory electron transport chain
GO:0022900 4.99586841258498e-08 18.4878522837706 0.528544737388416 8 134 electron transport chain
GO:0045333 7.84983626824484e-08 17.3743527261651 0.560099647083247 8 142 cellular respiration
GO:0042773 1.07881748832632e-06 32.8571428571429 0.185385094457131 5 47 ATP synthesis coupled electron transport
GO:0042775 1.07881748832632e-06 32.8571428571429 0.185385094457131 5 47 mitochondrial ATP synthesis coupled electron transport
GO:0006119 3.11798050635669e-06 26.0177793904209 0.228773095287523 5 58 oxidative phosphorylation
GO:0006120 1.16615178243753e-05 33.872641509434 0.141997093626739 4 36 mitochondrial electron transport, NADH to ubiquinone
Tissue: Muscle_Skeletal=>Artery_Tibial Type: asymmetric
SourceGene: RP11-350G8.5(ENSG00000228013.1)
TargetGeneSet: HMGN2 SERINC2 SFPQ PPIE RP3 MUTYH CMPK1 OSBPL9 PRPF38A GNG12 RP11 NSRP1P1 FAM69A AGL NBPF6 BCAS2 MAN1A2 RAB7L1 RD3 SMYD2 ANKS1B CAPN2 DEGS1 ZRANB3 COBLL1 BBS5 NCKAP1 RPL7P14 FARSB TMEM43 BTD OSBPL10 ZDHHC3 RBM5 EIF4E3 SCHIP1 AP2M1 CHRD CCDC50 STK32B CC2D2A MED28 PARM1 SH3D19 HADHAP1 FBXL7 OXCT1 ARL15 MAP1B HEXB FBXL17 ERGIC1 MAPK9 MRPS18B SCUBE3 NFYA MRPL2 HSP90AB1 SLC35B2 FKBP9 DPY19L1 ANKIB1 MEPCE RBM28 OPN1SW KCNH2 NEIL2 ZMAT4 PTDSS1 APTX SMU1 VPS13A C9orf156 EXOSC2 DDX31 SURF6 EDF1 CHAF1B FAM35A ZFYVE27 CWF19L1 PDCD11 C10orf137 BET1L OR51A9P FAM160A2 NAT10 PPME1 GDPD5 FAT3 GUCY1A2 DIXDC1 SIDT2 FEZ1 WNT5B NECAP1 ART4 TMEM117 CACNB3 DCTN2 GNS CHPT1 SART3 PLBD2 CDK2AP1 SETD8 MTUS2 RBM26 PRMT5-AS1 MED6 EML1 ULK4P3 SLC30A4 NEO1 UBE2Q2 HMG20A AEN FANCI C16orf91 TELO2 NTAN1 GDE1 KIF22 INO80E CCDC102A CTU2 METTL16 SPAG5 UTP6 SRSF1 NAT9 THOC1 TTC39C MBD1 FZR1 HNRNPM

OLFM2 DDX49 U2AF1L4 C19orf55 SUPT5H ZNF546 RTN2 SYMPK CCDC9
DHX34 ZNF324B FAM83D DDX27 COL20A1 USP25 ITSN1 PKNOX1 U2AF1 GAS2L1 DRG1
RASD2 TRIOBP XPNPEP3 TRMU SCML1 RAI2 RBM10 LAS1L TAF1 ENOX2 EMD
GOBPID Pvalue OddsRatio ExpCount Count Size Term
GO:0006397 4.68594899917893e-07 5.19955240581872 3.53414988582105
16 399 mRNA processing
Tissue: Artery_Tibial=>Muscle_Skeletal Type: asymmetric
SourceGene: RP11-370B11.1(ENSG00000236739.2)
TargetGeneSet: CELA2A CELA3B CELA3A TMSB4XP1 RP4 AMYP1 RPS27
MIR4426 PTPN14 PPP1CB RHOQ MCM6 RPL29 CTBP1-AS1 RPL7AP31 ZFR
RP11 CLPS MTRNR2L9 EEF1A1P6 TRIM56 PRSS1 YWHAZP3 PNLIP
MTRNR2L8 CARD16 LINC00441 IGHA2 GP2 CTRB2 CTRB1 ADAMTS18
CYP2F1P SON RP1 WASF4P RP6
GOBPID Pvalue OddsRatio ExpCount Count Size Term
GO:0007586 1.93179639713465e-08 46.3559870550162 0.181025534565082
6 109 digestion
Tissue: Artery_Tibial=>Muscle_Skeletal Type: asymmetric
SourceGene: RP11-452F19.3(ENSG00000228106.1)
TargetGeneSet: RPL22 RP4 SRGAP2P1 SNX18P15 RPS27 FAM189B
ACBD6 RP11 UBC RPL31 RPL37A RPL14 CCDC72 FAM208A RPL24 RPL35A
RPL34 RPL37 NNT BTF3 NSA2 TBCA RPS23 CTD RELL2 HCP5
CSNK2B NOTCH4 RPS18 SYNGAP1 RPL10A MRPS12 RPS20 C8orf59 RPL30 THNSL1
RPL27A RPS13 LPXN C11orf10 FAU RPS25 PFDN5 WIBG RPL41
SLC24A6 TPT1 MED4-AS1 BIVM RPL4 TCEB2 ZCCHC14 RPS7P1 SPAG5-AS1
RPL23A RPL27 IER3IP1 HMHA1 MAP3K10 SNRPD2 RPS11 CPT1C EEF1B2P3
TCEAL3
GOBPID Pvalue OddsRatio ExpCount Count Size Term
GO:0006415 2.06633390345245e-40 183.351812366738 0.327451387447236
24 91 translational termination
GO:0000184 2.12166164030355e-39 140.908195429472 0.428205660507923
25 119 nuclear-transcribed mRNA catabolic process, nonsense-mediated decay
GO:0006414 1.01286699559936e-38 151.513227513228 0.377828523977579
24 105 translational elongation
GO:0006614 1.01286699559936e-38 151.513227513228 0.377828523977579
24 105 SRP-dependent cotranslational protein targeting to membrane
GO:0006613 1.30686375341027e-38 149.655052264808 0.381426890872604
24 106 cotranslational protein targeting to membrane
GO:0045047 1.30686375341027e-38 149.655052264808 0.381426890872604
24 106 protein targeting to ER
GO:0072599 1.30686375341027e-38 149.655052264808 0.381426890872604
24 106 establishment of protein localization to endoplasmic reticulum
GO:0070972 2.28287588990214e-37 130.440729483283 0.424607293612899
24 118 protein localization to endoplasmic reticulum
GO:0019080 1.42021310224845e-36 104.88683127572 0.543353401148709 25
151 viral genome expression
GO:0019083 1.42021310224845e-36 104.88683127572 0.543353401148709 25
151 viral transcription
GO:0000956 2.90698812152579e-35 91.6602366255144 0.608124005259152
25 169 nuclear-transcribed mRNA catabolic process
GO:0006402 1.15547026151555e-34 86.2139917695473 0.640509307314373
25 178 mRNA catabolic process
GO:0006413 1.69049505340889e-34 95.5647321428571 0.546951768043734
24 152 translational initiation
GO:0006612 2.0012394252553e-34 94.8172757475083 0.550550134938759
24 153 protein targeting to membrane
GO:0043624 2.0012394252553e-34 94.8172757475083 0.550550134938759
24 153 cellular protein complex disassembly
GO:0043241 4.57092535173956e-34 91.2473347547974 0.568541969413881
24 158 protein complex disassembly
GO:0006401 4.78976746455963e-33 73.1430041152263 0.737665213480036
25 205 RNA catabolic process
GO:0034623 5.364037335902e-33 81.4228571428571 0.626115839734274
24 174 cellular macromolecular complex disassembly

GO:0032984	1.10112153428467e-32	78.76866359447	0.644107674209397	24
179	macromolecular complex disassembly
GO:0019058	1.08040811381849e-31	63.794498381877	0.831222752750675	25
231	viral infectious cycle
GO:0072594	2.97522236261082e-31	67.7095238095238	0.734066846585011
24	204	establishment of protein localization to organelle
GO:0022415	2.30178622896268e-30	55.8077226162333	0.935575392706387
25	260	viral reproductive process
GO:0071845	1.08055864628249e-27	46.6120879120879	1.02193619818698
24	284	cellular component disassembly at cellular level
GO:0022411	1.52173021425648e-27	45.8928571428571	1.03632966576707
24	288	cellular component disassembly
GO:0016032	2.58745487499351e-24	30.3708050773778	1.62286346965608
25	451	viral reproduction
GO:0033365	2.34727705743791e-22	26.63060769965	1.70202754134662	24
473	protein localization to organelle
GO:0006605	2.46772339950581e-22	26.5695238095238	1.70562590824164
24	474	protein targeting
GO:0022613	1.49006029914864e-05	10.1189602446483	0.809632551380527
7	225	ribonucleoprotein complex biogenesis
Tissue: Muscle_Skeletal=>Artery_Tibial	Type:	asymmetric
SourceGene:	RP11-56B16.2(ENSG00000259556.1)
TargetGeneSet:	KHDRBS1 CYP4A11 FPGT-TNNI3K	PSMB4	C1orf61 RPL15	C3orf37 RP11
EXOSC9	RXFP1	MRPS18B MRPL2	RSPH9	AGPAT4	TRGV5	EIF4H	KRT8P3	PYCRL
TMEM38B OPALIN	SH3PXD2A	ARL2	SF3B2	RPL18AP3	ARHGAP5-AS1	RPS2
MIR4519 LDHD	SPIRE2	WNK4	NT5C	HNRNPM	CTD	RPS5	PSMF1	MT1P3
U2AF1	XRCC6	RP13	RPS23P8
GOBPID	Pvalue	OddsRatio	ExpCount	Count	Size	Term
Tissue: Artery_Tibial=>Muscle_Skeletal	Type:	asymmetric

SourceGene:	S100A6(ENSG00000197956.4)	S100 calcium binding protein A6

TargetGeneSet:	WDTC1	CCDC23	KDM4A	INADL	IL6R	MTX1	PRRC2C	PPP2R5A PUM2
UBC	ORMDL1	NIF3L1	FAM117B PAX3	D2HGDH	NR1D2	OR7E94P RP11	CTD
MAN2A1	FAM53C	FOXP4	PEX6	PHF3	MDN1	STXBP5	ARID1B	TNRC18	C7orf41
BAZ1B	CUX1	OPN1SW	MLL3	SEPT7P6 RPS20	C8orf59 FAM91A1 FAM83H	MPDZ
FAM13C	LDB1	HBE1	OR52E8	RPL27A	RPS13	PIGCP1	UCP2	MLL	RPS25
DSTNP2	ITPR2	RP3	NR2C1	ACAD10	LRRC43	PARP4-AS1	RFXAP	MED4-AS1
HERC1	TNFRSF12A	TXNDC11 RPL7P47 GDPD3	SULT1A3 FBXL19	ANKFY1	TOM1L2
TMEM11	RPS7P1	RPL27	FAM171A2	TNRC6C	IER3IP1 MAP3K10 SNRPD2	SYMPK	BAX
RP1	SLC16A8 XPNPEP3 WWC3	MED12	RPL39	BCORL1

GOBPID	Pvalue	OddsRatio	ExpCount	Count	Size	Term

GO:0006415	7.20E-08	23.38071895	0.36523424	7	91
translational termination					
GO:0072594	1.09E-07	13.3733124	0.818766867	9	204
establishment of protein localization to organelle					
GO:0006414	1.94E-07	20.0210084	0.421424123	7	105
translational elongation					
GO:0006614	1.94E-07	20.0210084	0.421424123	7	105
SRP-dependent cotranslational protein targeting to membrane					
GO:0006613	2.07E-07	19.81738958	0.425437686	7	106
cotranslational protein targeting to membrane					
GO:0045047	2.07E-07	19.81738958	0.425437686	7	106
protein targeting to ER					

GO:0072599	2.07E-07	19.81738958	0.425437686	7	106
establishment of protein localization to endoplasmic reticulum					
GO:0070972	4.31E-07	17.66013072	0.473600443	7	118
protein localization to endoplasmic reticulum					
GO:0034623	4.32E-07	13.71277108	0.698359975	8	174
cellular macromolecular complex disassembly					
GO:0000184	4.57E-07	17.50122549	0.477614006	7	119
"nuclear-transcribed mRNA catabolic process, nonsense-mediated decay"					
GO:0032984	5.36E-07	13.3071345	0.71842779	8	179
macromolecular complex disassembly					
GO:0071845	1.77E-06	9.429461967	1.139851913	9	284
cellular component disassembly at cellular level					
GO:0022411	1.98E-06	9.291639236	1.155906166	9	288
cellular component disassembly					

GO:0019080	2.28E-06	13.58156318	0.606048024	7	151
viral genome expression					
GO:0019083	2.28E-06	13.58156318	0.606048024	7	151
viral transcription					
GO:0006413	2.38E-06	13.48695064	0.610061587	7	152
translational initiation					
GO:0006612	2.49E-06	13.39363417	0.614075151	7	153
protein targeting to membrane					
GO:0043624	2.49E-06	13.39363417	0.614075151	7	153
cellular protein complex disassembly					
GO:0043241	3.08E-06	12.94559148	0.634142966	7	158
protein complex disassembly					

GO:0000956	4.81E-06	12.05725006	0.67829216	7	169
nuclear-transcribed mRNA catabolic process

GO:0006402	6.77E-06	11.41543401	0.714414227	7	178	mRNA
catabolic process

GO:0006401	1.70E-05	9.84006734	0.82278043	7	205	RNA
catabolic process

Tissue: Muscle_Skeletal=>Artery_Tibial	Type:	asymmetric
SourceGene:	TMED4(ENSG00000158604.9)
TargetGeneSet:	FAM132A GNB1	PANK4	GPR157	SLC25A34		RP4	FAM43B
EIF4G3	RP1	SYF2	DLGAP3	STK40	KCNQ4	IPO13	SLC6A9	ZFYVE9	RP11
FPGT-TNNI3K	LRRC39	SLC16A1 GDAP2	NOTCH2	PMF1	SMG5	PEA15	FAM78B
TATDN3	LIN9	NUP133	TOMM20	LYST-IT1		PXDN	TRAPPC12-AS1	KHK	PREB
EXOC6B	SPR	FAHD2A	STARD7	ACVR1	BTF3L4P2		FASTKD1 UBE2V1	NCKAP1	KLF7
INHA	AGAP1	MTMR14	UBP1	GOLGA4	TRAK1	PHF7	PBRM1	SPCS1	TMEM110
RPL10AP6		PTPLB	LRRC34	ABCC5	VPS8	TM4SF19-AS1		TMEM128 SHROOM3
NUDT10	EXOSC9	SPATA5	NAA15	OTUD4	WWC2	CTD	C7	FBXO4	RNF138P1
PPIP5K2 SEC24A	SLU7	ARL10	C6orf228		LST1	AGER	RPL10A	SRPK1
ASCC3	BVES	C6orf72 MTRF1L	PSMB1	C7orf50 TMEM196 TMED4	GTF2I	GTF2IRD2B
TAF6	FBXO24	PMPCB	COG5	WDR91	SLC4A2	LMBR1	ARHGEF10		CHMP7
SGK196	CHD7	C8orf46 MYBL1	FBXO43	C8orf33 SNAPC3	ACO1	DCTN3	VPS13A
BICD2	FSD1L	STXBP1	AK8	CAMSAP1 C9orf69 RNF224	NOXA1	PFKP	FAM208B CDNF
FAM190B C10orf12		MGEA5	SFR1	ACADSB	PWWP2B	CARS	TRIM44	PEX16	DDB2
PLCB3	KDM2A	PANX1	FAM76B	TIMM8B	TTC12	CCDC84	DPAGT1	ERC1	C12orf33
LRP6	C12orf69		LMO3	KIF21A	H1FNT	LETMD1	PRR13	HOXC13	HOXC5
B4GALNT1		XPOT	RIC8B	SSH1	TAOK3	MLEC	ZNF605	TPTE2	SACS	DGKH
LINC00441		DIAPH3-AS1		OXA1L	EAPP	FANCM	EXOC5	CHURC1	EXD2
SRSF5	GSTZ1	PTPN21	TTC7B	MEG8	TYRO3	TP53BP1 UBAP1L	HEXA	NEO1
C15orf27		ZNF592	RHOT2	ADCY9	GLIS2	CLEC16A NDE1	KIF22	TBX6
GNAO1	MMP15	DDX19B	GAN	MPRIP	ALKBH5	LLGL1	CCDC144C		C17orf51
ANKRD13B		CCT6B	PGAP3	SMARCE1 DUSP3	CDK5RAP3		BPTF	NT5C
SOGA2	GNAL	WDR7	APC2	REEP6	LMNB2	PIP5K1C CATSPERD		CARM1
SYCE2	SAMD1	U2AF1L4 ALKBH6	ZNF345	SIPA1L3 ZNF226	EHD2	CYTH2	C19orf48
NLRP9	ZNF460	ZNF552	ZSCAN22 ZNF446	SNPH	TASP1	POLR3F	RP3	SLC35C2
USP25	CHAF1B	MICAL3	ZNF74	CRKL	MAPK1	ZNF70	GAS2L1	PICK1	CTA

RANGAP1 C22orf32 TCF20 ZBED4 POLA1 RBM3 SUV39H1 RRAGB THOC2
RBMX2 RBMX RP13
GOBPID Pvalue OddsRatio ExpCount Count Size Term
Tissue: Artery_Tibial=>Muscle_Skeletal Type: asymmetric

SourceGene: TMEM106C(ENSG00000134291.7) ?

TargetGeneSet: C1orf174 RPL22 H6PD CTNNBIP1 IPP CPT2 RP4
GBP3 SLC35A3 RP11 S100A12 CHTOP POU2F1 JMJD4 IBA57 UBC POLE4 RPIA
RPL31 ARL6IP6 BBS5 EEF1B2 D2HGDH CHCHD4 RPL29 PTPRG RPL24 RAB41 H1FX
VPS8 RPL34 PHF17 RTN3P1 GARS RPS3A LPCAT1 LEAP2 ZFP2 CCDC90A
TOB2P1 RPS18 MRPS12 IYD TATDN2P2 FBXL18 EEF1A1P6 LINC00265
RPS3AP26 IFRD1 PPP2R2A ASPH SLC26A7 ZNF707 C9orf106 USP20
NUP214 NKX2-1 RPL7A SEC16A VDAC2 ART1 CHRNA10 RNF141 NAALADL1
ATG16L2 MMP8 TTC12 RPS25 TBCEL PFDN5 ARHGAP9 DDIT3 THAP2 TRAFD1 CDK8
TPT1 C14orf21 SFTA3 DDX24 WDR25 RPS3AP47 TGM7 RPS3AP6
NAGPA ITGAX CKLF DYNC1LI2 TMEM170A CMC2 MTHFSD RPS7P1 PIGS
RPL23A RPL27 GNG8 COG1 CTD ADNP2 PRR22 EMR1 PEX11G C19orf59
KLHL26 ZNF30 FXYD7 PRR19 VASP PYGB CDH4 RAC2 LDOC1L GPR64
SUV39H1 PQBP1 RPL39

GOBPID	Pvalue	OddsRatio	ExpCount	Count	Size	Term
GO:0006414	9.75E-16	31.37582418	0.610338385	14	105	translational elongation

GO:0006415	4.38E-15	33.54225352	0.528959934	13	91	translational termination
GO:0000184	5.90E-15	27.16571429	0.691716836	14	119	"nuclear-transcribed mRNA catabolic process, nonsense-mediated decay"
GO:0006614	2.99E-14	28.41013472	0.610338385	13	105	SRP-dependent cotranslational protein targeting to membrane
GO:0006613	3.40E-14	28.1026806	0.616151131	13	106	cotranslational protein targeting to membrane
GO:0045047	3.40E-14	28.1026806	0.616151131	13	106	protein targeting to ER

GO:0072599	3.40E-14	28.1026806	0.616151131	13	106
establishment of protein localization to endoplasmic reticulum

GO:0070972	1.40E-13	24.87002012	0.68590409	13	118
protein localization to endoplasmic reticulum

GO:0072594	6.61E-13	16.30779848	1.185800291	15	204
establishment of protein localization to organelle

GO:0000956	8.12E-13	18.33806452	0.982354162	14	169
nuclear-transcribed mRNA catabolic process

GO:0006402	1.66E-12	17.32073171	1.034668881	14	178	mRNA
catabolic process

GO:0019080	3.47E-12	18.87905695	0.877724725	13	151
viral genome expression					
GO:0019083	3.47E-12	18.87905695	0.877724725	13	151
viral transcription					
GO:0006413	3.78E-12	18.74191914	0.883537471	13	152
translational initiation					
GO:0006612	4.11E-12	18.60674044	0.889350218	13	153
protein targeting to membrane					
GO:0043624	4.11E-12	18.60674044	0.889350218	13	153
cellular protein complex disassembly					

GO:0043241	6.21E-12	17.95881496	0.918413951	13	158	protein complex disassembly
GO:0006401	1.14E-11	14.84397906	1.191613037	14	205	RNA catabolic process
GO:0034623	2.12E-11	16.15589187	1.011417895	13	174	cellular macromolecular complex disassembly
GO:0032984	3.04E-11	15.66375361	1.040481628	13	179	macromolecular complex disassembly
GO:0019058	7.29E-10	11.88377051	1.342744447	13	231	viral infectious cycle

GO:0071845	8.63E-10	10.44222222	1.650820012	14	284
cellular component disassembly at cellular level					
GO:0022411	1.03E-09	10.28686131	1.674070999	14	288
cellular component disassembly					
GO:0022415	3.08E-09	10.4670126	1.511314096	13	260
viral reproductive process					
GO:0033365	1.11E-08	7.161796885	2.749429105	16	473
protein localization to organelle					

GO:0006605 1.14E-08 7.145646031 2.755241852 16 474
protein targeting

GO:0016032 2.93E-07 6.375286041 2.621548682 14 451
viral reproduction

Tissue: Muscle_Skeletal=>Artery_Tibial Type: asymmetric
SourceGene: all_source
TargetGeneSet: MIR133A2 RPL36P4 MT1P2 APLP1 SLC7A11 CALCRL MAGI2
PMP22 ELOVL3 RBM38 TPH1 SNAI1 C6orf25 C10orf99 PRMT8 GK5
FHDC1 CTB HSD11B2 MAP3K1 DGCR2 OXSM LINC00452 CCL21 ITIH4-AS1
ICOS DUTP7 ZBTB34 LRRC6 APAF1 TSPYL6 LSR B3GALTL C10orf32
TRIB1 NHSL1 LCE2A PQBP1 RPS12P26 ART1 C1orf229 CD247 CSF3
GALNT14 EEF1A1P12 NUAK1 PBK TELO2 ATP6V1A NF2 RP13 HBEGF
FAM174A WDR25 TBC1D2B RP1 DOPEY1 MPO CLVS2 SORBS1 ZC3HC1 RNF122
SAMD15 PROM1 TM4SF1 TMED4 PRICKLE2 PHEX-AS1 WISP3 FAM46A
MCRS1 USP20 RNF141 MAPK13 MAFK HOMEZ PLCXD2 GPRC5C C1orf174 KLF4
PIGL BUD13 NFE2L3 NACA2 GABRB2 NPPB EEPD1 U6 LSM14B MTERFD2 CINP
IGHV3-74 ZSWIM3 RASD1 NPFF RBAK C15orf57 PPP1R10 C15orf23
MYO18B ADAM33 PDZK1IP1-AS1 PITX3 PITX2 KLLN FAM173A DNAJC12 GIT1
HIBADH PDF MTHFD1P1 FAM181A-AS1 ABCA9 RALGAPA2 DNAH11 FOS
VEZF1P1 LYL1 SNORA7A SRGAP2-IT1 CDH4 PCBP2P1 RALGPS1 PDK1 NOG
ARPC3P1 MTRNR2L1 USP54 GPR124 C2CD2 GAPDHP44 CHAD MMP28
SLC35A3 C8orf42 BACE2 DNM1P33 CTD JAKMIP1 UBOX5 SVIP HIPK4 C1orf9
CCDC57 SH2B1 RP4 U1 TRAF3IP1 MYSM1 C6orf162 ARFGAP3
AP5Z1 SOCS1 AVIL CHRNA10 PABPC3 C16orf45 PDCD6 FAM53C MTA2
TRAPPC9 C15orf44 CYB5R1 IQSEC2 ADPRHL1 POLH JMJD4 SULT1A3 MRPS2
PAFAH1B2 IRF2BPL POLR2B TTLL7-IT1 AIF1L APOBEC2 CACNG1 PCP2
MTHFSD RP11 SYT12 TMEM140 TMEM171 HCG20 CEBPE NR1I3 PTGDR2 COX7A2P1
RNASEH1 IL13 SLC22A13 ZNF678 EBF2 GAPDHP62 VGLL4 LIPJ
EIF4A1P7 HNRNPH2 PITPNM3 FAM181A KLHL26 TAF1C GLTPD1 TEAD3 ZNF689 PBX1
C3orf37 SPHK2 WIPI2 TYMP IFNG TNFAIP8L1 PHLDA1 ZNF329 TCEB1P2 IL6R
PEX10 PUF60 EBF1 BMP4 E2F3 BMP5 TMEM50B HMOX1 DFFB FTLP3
MTND4P24 MT
GOBPID Pvalue OddsRatio ExpCount Count Size Term
GO:0048660 2.77023798858757e-08 15.7433660933661 0.695315203100132
9 64 regulation of smooth muscle cell proliferation
GO:0048659 4.18321374063363e-08 14.925908667288 0.72790810324545 9
67 smooth muscle cell proliferation
GO:0033002 7.86852492477653e-08 11.3717486994798 1.03210850460176
10 95 muscle cell proliferation
GO:0048661 3.62512358780466e-06 17.1715833835039 0.423707701889143
6 39 positive regulation of smooth muscle cell proliferation

Tissue: Artery_Tibial=>Nerve_Tibial Type: asymmetric
SourceGene: C10orf35(ENSG00000171224.7)
TargetGeneSet: RPL22 ZMYND12 PPCS LRRC41 ATPAF1 BTF3L4 RP11 RPL5 RP4
ATP5F1 RPS27 CAMSAP2 KLHL12 SNRPE SOX13 PLXNA2 BROX ARV1 CNST
PPM1B MTHFD2 COA5 UNC50 CCDC115 NIF3L1 RPL37A SETD5 RPL15 KIF9
TRAIP RPL29 SPCS1 RNF7 MBNL1 RSRC1 FNDC3B METTL19 C4orf52 THAP6
COPS4 MRPS18C RPL34 KIAA0922 TAF9 BTF3 XRCC4 HINT1 AFF4 SKP1
SH3PXD2B NOL7 MRPS18B RPL10A GAPDHP15 RPL39P3 MRPS12 RP1
ARID1B TMEM181 BUD31 FAM200A FBXO24 KAT6A C8orf40 C8orf22 XKR4 RPS20
RPL30 POLR2K ZHX1 C9orf123 DCTN3 RPL12 MRPL41 COX15 SFR1
RPS13 EIF3M DDB2 POLR2G FCHSD2 C11orf1 ATP5L RPS25 C12orf57
C12orf47 ELF1 COMMD6 STK24 LTB4R KTN1 ISCA2 PSMC1 C14orf142
RPL3P4 DTWD1 CARHSP1 CTA LCMT1 NAE1 DERL2 RPL26 MAPK7 RPL17
RPL38 DUS1L FOXK2 CTD RPL36 BRD4 JPH2 ATP5J CSDC2 C22orf40
RPS2P55 ATRX

GOBPID	Pvalue	OddsRatio	ExpCount	Count	Size	Term
GO:0006614	1.38481720267066e-21	41.5978630403108	0.646668050653934	18	105	SRP-dependent cotranslational protein targeting to membrane
GO:0006613	1.6602657582738e-21	41.1222791293214	0.652826793993495	18	106	cotranslational protein targeting to membrane
GO:0045047	1.6602657582738e-21	41.1222791293214	0.652826793993495	18	106	protein targeting to ER
GO:0072599	1.6602657582738e-21	41.1222791293214	0.652826793993495	18	106	establishment of protein localization to endoplasmic reticulum
GO:0006415	4.21536756381393e-21	45.5885885885886	0.560445643900076	17	91	translational termination
GO:0070972	1.27463902762592e-20	36.1571830985916	0.726731714068231	18	118	protein localization to endoplasmic reticulum
GO:0019080	4.3944017071538e-20	29.2608225108225	0.929970244273753	19	151	viral genome expression
GO:0019083	4.3944017071538e-20	29.2608225108225	0.929970244273753	19	151	viral transcription
GO:0006414	5.62679419065913e-20	38.2982954545455	0.646668050653934	17	105	translational elongation
GO:0000184	5.23620634255959e-19	33.0092592592593	0.732890457407792	17	119	nuclear-transcribed mRNA catabolic process, nonsense-mediated decay
GO:0006413	1.42411153623148e-18	26.9186462055918	0.936128987613314	18	152	translational initiation
GO:0006612	1.60641818017974e-18	26.7173708920188	0.942287730952875	18	153	protein targeting to membrane
GO:0019058	7.75988172814626e-18	19.4395219451885	1.42266971143865	20	231	viral infectious cycle
GO:0043624	4.213261542162e-17	24.6979166666667	0.942287730952875	17	153	cellular protein complex disassembly
GO:0043241	7.32595854230213e-17	23.813731284476	0.973081447650682	17	158	protein complex disassembly
GO:0022415	7.96107871597996e-17	17.0555555555556	1.60127326828593	20	260	viral reproductive process
GO:0000956	2.31817905642901e-16	22.0732821637427	1.04082762438586	17	169	nuclear-transcribed mRNA catabolic process
GO:0072594	2.92975829806545e-16	19.3221263062244	1.2563836412705	18	204	establishment of protein localization to organelle
GO:0034623	3.80902046211827e-16	21.3627919320594	1.07162134108366	17	174	cellular macromolecular complex disassembly
GO:0006402	5.60386723225121e-16	20.8261732229124	1.09625631444191	17	178	mRNA catabolic process
GO:0032984	6.16251868877569e-16	20.696159122085	1.10241505778147	17	179	macromolecular complex disassembly
GO:0016032	2.34714639752683e-15	11.3452987822147	2.77759324614214	23	451	viral reproduction
GO:0006401	6.04928694818813e-15	17.8012706855792	1.26254238461006	17	205	RNA catabolic process
GO:0071845	6.78592096436273e-15	14.4389757412399	1.7490831084354	19		

284	cellular component disassembly at cellular level
GO:0022411	8.76344183537776e-15	14.220233669676 1.77371808179365	19
288	cellular component disassembly
GO:0033365	5.79041800110511e-11	8.31504090623033	2.91308559961248
19	473	protein localization to organelle
GO:0006605	6.00373783552746e-11	8.29616954474097	2.91924434295204
19	474	protein targeting
Tissue: Nerve_Tibial=>Artery_Tibial	Type:	asymmetric
SourceGene:	CROCCP3(ENSG00000080947.9)
TargetGeneSet:	RP13	RP11	KHDRBS1 IPP	BEND5	LYPLAL1 TOMM20	ABHD1
DDIT3	EIF3FP3 FAHD2B	MCM6	FMNL2	PPIG	LRRN4	RPL15	SPCS1	CPOX
RPL24	NDUFB4	ITGB5-AS1	DHX36	ATP5G1P4	ABCC5	WDFY3-AS1
OTUD4	EPB41L4A-AS1	CCT5P1	CTB	RPL26L1 ARL10	TBC1D9B RPS10	RPL10A
C6orf226	MRPL2	RP1	DDO	SOGA3	SPDYE1	RP4	RHBDD2	STAG3
HMBOX1	C8orf40 PRKDC	RPS20	CHCHD7	SNHG6	TATDN1	EEF1D	TONSL	RPL8
RPL35	RPL12	NOXA1	CDNF	ZNF37BP DDIT4	CTD	EIF3F	RPL27A	NDUFV1	PHB2
ZCRB1	TIMELESS	NACA	YEATS4	ISCU	C12orf51	SMAD9-AS1
C13orf44-IT1	DHRS12	SYNE2	CEP152	GS1	SMG1	GTF3C1	NUDT21	DDX19B
COX4I1	RPL13	C17orf108	ACACA	RPL19	CCR10	ZADH2	RPL36	PRR24
RPL18	ZNF341	MT1P3	SCAND1	SLC2A4RG	USP16	URB1	CHCHD10 CTA
PLCXD1	RP3	FAM122C RPL10
GOBPID	Pvalue	OddsRatio	ExpCount	Count	Size	Term
GO:0006414	6.90619840747159e-20	45.0668243642815	0.530413120199294
16	105	translational elongation
GO:0006614	6.90619840747159e-20	45.0668243642815	0.530413120199294
16	105	SRP-dependent cotranslational protein targeting to membrane
GO:0006613	8.10359646248825e-20	44.562962962963 0.535464673725002	16
106	cotranslational protein targeting to membrane
GO:0045047	8.10359646248825e-20	44.562962962963 0.535464673725002	16
106	protein targeting to ER
GO:0072599	8.10359646248825e-20	44.562962962963 0.535464673725002	16
106	establishment of protein localization to endoplasmic reticulum
GO:0006415	3.14843541559965e-19	48.6683303085299	0.459691370839388
15	91	translational termination
GO:0070972	4.89284304868317e-19	39.2872377020984	0.596083316033493
16	118	protein localization to endoplasmic reticulum
GO:0000184	5.63177529675806e-19	38.9030829500937	0.6011348695592 16
119	nuclear-transcribed mRNA catabolic process, nonsense-mediated decay
GO:0006413	3.16844260983172e-17	29.3952528379773	0.76783613590755
16	152	translational initiation
GO:0006612	3.52523541807684e-17	29.1786400307338	0.772887689433257
16	153	protein targeting to membrane
GO:0043241	5.94358273078551e-17	28.1413392636521	0.798145457061795
16	158	protein complex disassembly
GO:0072594	1.583500418993e-16	23.0373376623377	1.03051691924434
17	204	establishment of protein localization to organelle
GO:0000956	1.76493880694697e-16	26.0979245499369	0.853712545844578
16	169	nuclear-transcribed mRNA catabolic process
GO:0006402	4.06609441071111e-16	24.6324453108079	0.899176527575946
16	178	mRNA catabolic process
GO:0032984	4.44848468912443e-16	24.4796039177699	0.904228081101654
16	179	macromolecular complex disassembly
GO:0019080	8.244973911826e-16	27.0829107505071	0.762784582381842
15	151	viral genome expression
GO:0019083	8.244973911826e-16	27.0829107505071	0.762784582381842
15	151	viral transcription
GO:0043624	1.00652448871285e-15	26.6866566716642	0.772887689433257
15	153	cellular protein complex disassembly
GO:0006401	3.86587428900499e-15	21.0734243014945	1.03556847277005
16	205	RNA catabolic process
GO:0034623	6.98680755445834e-15	23.1278464541314	0.878970313473116
15	174	cellular macromolecular complex disassembly
GO:0019058	4.61240226551949e-13	16.9564176245211	1.16690886443845

15	231	viral infectious cycle
GO:0022411	7.76646211009645e-13	14.5572755417957	1.45484741540378
16	288	cellular component disassembly
GO:0022415	2.56567103853682e-12	14.9187192118227	1.31340391668397
15	260	viral reproductive process
GO:0071845	9.11819223887337e-12	13.5646071016536	1.43464120130095
15	284	cellular component disassembly at cellular level
GO:0033365	1.40679287257283e-11	10.0145454545455	2.38938481765968
18	473	protein localization to organelle
GO:0006605	1.4569105049423e-11	9.99186602870813	2.39443637118538
18	474	protein targeting
GO:0016032	6.3932193816875e-10	8.99729784230692	2.27825064009411
16	451	viral reproduction
Tissue: Nerve_Tibial=>Artery_Tibial	Type:	asymmetric
SourceGene:	CTA-520D8.2(ENSG00000237445.1)
TargetGeneSet:	RP11	ARNT	EEF1B2	GPR55	TMEM43	RPL29	RPL24	GPRIN3
ITGA1	CTB	RPS10	RPL7	EIF3H	EEF1D	NAA35	EDF1	PIK3AP1	RPS3
BUD13	RPL18AP3	RPL6	RPL13	RPL23A	RPL17	RPL19	RPL18A	RPS16
RPL18	CD33	UBE2M	CHMP4B	RALY	RPS21	SH3KBP1	NUP62CL
GOBPID	Pvalue	OddsRatio	ExpCount	Count	Size	Term
GO:0006414	5.02934023179459e-27	171.757303370787	0.225243927755865
16	105	translational elongation
GO:0006415	1.31799727206099e-23	153.401069518717	0.195211404055083
14	91	translational termination
GO:0006614	1.11465811618562e-22	129.674208144796	0.225243927755865
14	105	SRP-dependent cotranslational protein targeting to membrane
GO:0006613	1.28286079883624e-22	128.255754475703	0.227389108020206
14	106	cotranslational protein targeting to membrane
GO:0045047	1.28286079883624e-22	128.255754475703	0.227389108020206
14	106	protein targeting to ER
GO:0072599	1.28286079883624e-22	128.255754475703	0.227389108020206
14	106	establishment of protein localization to endoplasmic reticulum
GO:0006413	2.74222482694532e-22	97.7395072992701	0.326067400179918
15	152	translational initiation
GO:0070972	6.24825863287656e-22	113.361990950226	0.253131271192305
14	118	protein localization to endoplasmic reticulum
GO:0000184	7.07352833397243e-22	112.274509803922	0.255276451456647
14	119	nuclear-transcribed mRNA catabolic process, nonsense-mediated decay
GO:0019080	2.27927918353134e-20	85.8574495491627	0.323922219915577
14	151	viral genome expression
GO:0019083	2.27927918353134e-20	85.8574495491627	0.323922219915577
14	151	viral transcription
GO:0006612	2.75724644608103e-20	84.6102412187897	0.32821258044426
14	153	protein targeting to membrane
GO:0043624	2.75724644608103e-20	84.6102412187897	0.32821258044426
14	153	cellular protein complex disassembly
GO:0043241	4.3880052088887e-20	81.6437908496732	0.338938481765968
14	158	protein complex disassembly
GO:0000956	1.15722733570687e-19	75.7912713472486	0.362535464673725
14	169	nuclear-transcribed mRNA catabolic process
GO:0034623	1.75945658274205e-19	73.3970588235294	0.373261365995433
14	174	cellular macromolecular complex disassembly
GO:0006402	2.43793015979867e-19	71.5868005738881	0.381842087052799
14	178	mRNA catabolic process
GO:0032984	2.6418492968021e-19	71.1479500891266	0.383987267317141
14	179	macromolecular complex disassembly
GO:0072594	1.71051515428738e-18	61.6780185758514	0.43761677392568
14	204	establishment of protein localization to organelle
GO:0006401	1.83385229797468e-18	61.3507853403141	0.439761954190021
14	205	RNA catabolic process
GO:0019058	9.98733941194616e-18	53.9013282732448	0.495536641062902
14	231	viral infectious cycle
GO:0022415	5.30005349293814e-17	47.4500239120038	0.557746868728808

14	260	viral reproductive process
GO:0071845	1.83128841043921e-16	43.1590413943355	0.609231195073005
14	284	cellular component disassembly at cellular level
GO:0022411	2.22765937577193e-16	42.516960068699 0.617811916130372	14
288	cellular component disassembly
GO:0016032	1.11223409867631e-13	26.3510566698075	0.967476299218047
14	451	viral reproduction
GO:0033365	2.13374419548453e-13	25.0485710624119	1.01467026503356
14	473	protein localization to organelle
GO:0006605	2.19619968406647e-13	24.9923273657289	1.0168154452979 14
474	protein targeting
Tissue: Artery_Tibial=>Nerve_Tibial	Type:	asymmetric
SourceGene:	DCTN3(ENSG00000137100.10)
TargetGeneSet:	RP11	RPL22	ZBTB17	KPNA7	EIF3I	RP4	MAP7D1	UQCRH
DIRAS3	PIGK	COL24A1 ATP5F1	ANKRD35 APH1A	MRPS21	SMG5	MNDA	TSTD1
TMCO1	DENND1B CAMSAP2 SNRPE	TMEM81	IKBKE	BROX	LYST-IT1	ZNF692
YWHAQ	TTC32	SLC5A6	RPLP0P6 DHX57	MORN2	NFU1	TPRKB	CAPG	GGCX
RPL31	SMPD4	CCDC115 FMNL2	SPC25	SCRN3	CHRNA1	HOXD-AS2	RBM45
KCTD18	NDUFB3	IMPDH1P10	LANCL1	GPR55	OR6B3	SETD5	ZNF197	PTPN23	RBM6
VPRBP	SPCS1	ARL6IP5 GXYLT2	CLDN18	ARID4A	MRPL47	NDUFB5	EIF4G1	KIAA0226
RPL35A	MYL5	NOP14	TLR1	UTP3	MRPS18C RPL34	PLK4	MGST2	OTUD4	BTF3
XRCC4	CTD	UQCRQ	UBE2D2	CTB	TCOF1	CD74	RPS14	ARL10	TMEM14C
TMEM14B PRSS16	PPP1R10 LST1	C6orf48 HLA-DMB HLA-DMA RPS10	HNRNPC	RP3
KIAA0240	C6orf226	MAD2L1BP	VEGFA	RPL39P3 COX7A2	ASF1A
SLC18B1 MRPS12	ARID1B	C6orf70 SNX8	NDUFA4	LSM5	SEC61G	GS1	ACN9	PUS7
NDUFA5	KAT6A	MRPL15	RPS20	PENK	C8orf59 FAM82B	TP53INP1	UQCRB
COX6C	POLR2K	KIAA0196	RP13	FAM83H	C9orf123	DCTN3	GBA2
LINC00475	SEC61B	STXBP1	ODF2	ZDHHC12 C9orf114	FAM78A	FAM208B
FBXO18	ATP5C1	COMMD3	PARD3	TMEM26	PBLD	IFIT2	RPP30	RPL13AP5
PPRC1	MRPL23	NUP98	ZNF143	RPS13	NDUFS3	FNBP4	MRPL16	CPSF7	SDHAF2
INCENP	MTA2	SF1	KLC2	LAMTOR1 CLPB	C11orf30	INTS4	USP28
PCSK7	ATP5L	RPS25	DPAGT1	TBCEL	KCNJ5	RAD52	C12orf57	DDX23
SPRYD3	PHC1P1	SLC25A3 SART3	TMEM119 COX6A1	RPS7P10 SACS	LPAR6	COMMD6
ACIN1	RPS29	BCRP2	ERH	NEK9	POMT2	NDUFB1	GLRX5	RPL3P4	BTBD6	NDN
SLC24A5 RSL24D1 PIGB	GTF2A2	FAM96A	NDUFB10 NPIPL3	POLR3E	NDUFAB1 KIF22
BCL7C	MIR4519 PYCARD	C16orf57	NAE1	RANBP10 AARS	DDX19A	SF3B3
USP10	ZNF469	NDEL1	CDRT4	FAM106B LIG3	MLLT6	RPL17	PGAP3	VPS25
RPL27	SAMD14	HILS1	SUPT4H1 SKA2	H3F3B	CBX4	C17orf90	ZFP161
IMPACT	UQCR11	C19orf10	C19orf70	RPL36	TRIP10	PEX11G	CARM1	BRD4
PGLS	ZNF85	UQCRFS1 ANKRD27 CLASRP	TRAPPC6A	RTN2	SNRPD2	ZNF541
TBC1D17 CLEC11A CNOT3	RPS9	NLRP9	ZIK1	ZNF552	TBC1D20 ZNF343	FTLP3
SNRPB2	TM9SF4	ROMO1	SNHG11	PFDN4	STMN3	ATP5J	MIS18A	C21orf67
MRPL40	DGCR6L	PI4KA	BCR	CRYBB3	UQCR10	PIK3IP1 APOBEC3H	RANGAP1
GTPBP6	SLC25A6 EEF1B2P3	ATP6AP2 RPS2P55 TIMP1	RPL7P57 PHF8	PDZD11
RPS23P8 MED12	RGAG4	TREX2	ARHGAP4
GOBPID	Pvalue	OddsRatio	ExpCount	Count	Size	Term
GO:0022904	1.88458437076499e-18	18.9515789473684	1.52792194311812
21	96	respiratory electron transport chain
GO:0022900	2.47680787658444e-15	12.5446923826057	2.13272437893571
21	134	electron transport chain
GO:0045333	8.25666345961817e-15	11.7086480287872	2.26005120752889
21	142	cellular respiration
GO:0006614	7.07593781295201e-13	12.8180217669654	1.67116462528545
17	105	SRP-dependent cotranslational protein targeting to membrane
GO:0006613	8.30987319424839e-13	12.6731022841167	1.68708047885959
17	106	cotranslational protein targeting to membrane
GO:0045047	8.30987319424839e-13	12.6731022841167	1.68708047885959
17	106	protein targeting to ER
GO:0072599	8.30987319424839e-13	12.6731022841167	1.68708047885959
17	106	establishment of protein localization to endoplasmic reticulum
GO:0006119	5.01854264201393e-12	18.8722990271377	0.923119507300533
13	58	oxidative phosphorylation
GO:0070972	5.0241719492898e-12	11.1579045228467	1.87807072174936

17	118	protein localization to endoplasmic reticulum
GO:0042773	6.33921836763452e-12	22.310878112713 0.748045117984915	12
47	ATP synthesis coupled electron transport
GO:0042775	6.33921836763452e-12	22.310878112713 0.748045117984915	12
47	mitochondrial ATP synthesis coupled electron transport
GO:0006612	3.84007161473665e-11	8.85911949685535	2.43512559684451
18	153	protein targeting to membrane
GO:0000184	6.33694622180209e-11	10.2480718628074	1.89398657532351
16	119	nuclear-transcribed mRNA catabolic process, nonsense-mediated decay
GO:0006414	1.05640758098325e-10	10.9542635658915	1.67116462528545
15	105	translational elongation
GO:0015980	1.26958528003733e-10	5.73796699715256	4.88616704726317
24	307	energy derivation by oxidation of organic compounds
GO:0006415	1.62733343099736e-10	11.9057239057239	1.44834267524739
14	91	translational termination
GO:0000956	2.03489396210353e-10	7.91140822191678	2.68977925403086
18	169	nuclear-transcribed mRNA catabolic process
GO:0006413	3.05447468900562e-10	8.32766475395583	2.41920974327036
17	152	translational initiation
GO:0006402	4.79192681795886e-10	7.46161556603774	2.83302193619819
18	178	mRNA catabolic process
GO:0019080	2.30109816367139e-09	7.80117687781239	2.40329388969621
16	151	viral genome expression
GO:0019083	2.30109816367139e-09	7.80117687781239	2.40329388969621
16	151	viral transcription
GO:0072594	4.3470597684874e-09	6.40672550213025	3.24683412912601
18	204	establishment of protein localization to organelle
GO:0006401	4.69892492085097e-09	6.37201089698315	3.26274998270016
18	205	RNA catabolic process
GO:0019058	5.12462939122301e-09	5.95034874362872	3.67656217562798
19	231	viral infectious cycle
GO:0006091	2.10454133579994e-08	4.1814440476911 6.81198532973497	25
428	generation of precursor metabolites and energy
GO:0043624	2.14279131117529e-08	7.11981799797775	2.43512559684451
15	153	cellular protein complex disassembly
GO:0043241	3.3111925186411e-08	6.86843389168971	2.51470486471524
15	158	protein complex disassembly
GO:0022415	3.51702201328691e-08	5.22349609643861	4.13812192927825
19	260	viral reproductive process
GO:0006120	7.51400194916751e-08	18.2664092664093	0.572970728669296
8	36	mitochondrial electron transport, NADH to ubiquinone
GO:0034623	1.19904119548825e-07	6.17025010969724	2.7693585219016 15
174	cellular macromolecular complex disassembly
GO:0006605	1.52243093578566e-07	3.74056168178608	7.54411459414573
25	474	protein targeting
GO:0032984	1.74020569964455e-07	5.98000567214974	2.84893778977233
15	179	macromolecular complex disassembly
GO:0071845	6.79424322897455e-07	4.45435522769187	4.52010241505778
18	284	cellular component disassembly at cellular level
GO:0022411	8.32543055384426e-07	4.38710691823899	4.58376582935437
18	288	cellular component disassembly
GO:0033365	1.9965500502194e-06	3.40024691358025	7.52819874057159
23	473	protein localization to organelle
GO:0016032	3.2040933158498e-06	3.40039447731755	7.17804996194035
22	451	viral reproduction
GO:0006122	1.57328230397114e-05	187.929515418502	0.0636634142965885
3	4	mitochondrial electron transport, ubiquinol to cytochrome c
Tissue: Nerve_Tibial=>Artery_Tibial	Type:	asymmetric
SourceGene:	ENSG00000258604.1
TargetGeneSet:	MTND1P23	RPL22	KHDRBS1 RP11	ADORA3	KCNT2	UBC
PLEKHB2 SATB2	EEF1B2	AGFG1	RPL15	RPL14	STAB1	RPL10AP6	RPL24
SEC62	RAC1P2	CTD	RPL10A	MRPL2	EIF4H	FAM71F1 CLEC5A	RPL7	RPS6
RPL35	RPS13	SAA2-SAA4	SAA1	RPL41	RPL14P1 CHPT1	RPL7AP6 NETO2

ZNF29P RPS7P1 RPL23A RPL17 RPL19 RAB31 CRLF1 FXYD1 HNRNPL PRR24
RPL18 SYP RPL10
GOBPID Pvalue OddsRatio ExpCount Count Size Term
GO:0006414 4.60435519401376e-28 153.772727272727 0.254307660369525
17 105 translational elongation
GO:0006613 5.47734464382063e-28 152.034332084894 0.25672963808733
17 106 cotranslational protein targeting to membrane
GO:0006415 5.61898119021901e-27 161.021754385965 0.220399972320255
16 91 translational termination
GO:0006614 6.63757336611401e-26 135.560023654642 0.254307660369525
16 105 SRP-dependent cotranslational protein targeting to membrane
GO:0045047 7.80784890738954e-26 134.044444444444 0.25672963808733
16 106 protein targeting to ER
GO:0072599 7.80784890738954e-26 134.044444444444 0.25672963808733
16 106 establishment of protein localization to endoplasmic reticulum
GO:0006413 3.64041620113799e-25 99.9082304526749 0.368140613106359
17 152 translational initiation
GO:0006612 4.09060402366082e-25 99.1666666666667 0.370562590824164
17 153 protein targeting to membrane
GO:0070972 4.85756512067118e-25 118.175438596491 0.28579337070099
16 118 protein localization to endoplasmic reticulum
GO:0000184 5.60514234428549e-25 117.019928461932 0.288215348418795
16 119 nuclear-transcribed mRNA catabolic process, nonsense-mediated decay
GO:0019080 3.06778938257395e-23 89.082261208577 0.365718635388554 16
151 viral genome expression
GO:0019083 3.06778938257395e-23 89.082261208577 0.365718635388554 16
151 viral transcription
GO:0043624 3.81957819605949e-23 87.7694967345371 0.370562590824164
16 153 cellular protein complex disassembly
GO:0043241 6.52086191731666e-23 84.6493699036323 0.382672479413189
16 158 protein complex disassembly
GO:0000956 1.99037797474661e-22 78.5029239766082 0.409314234309044
16 169 nuclear-transcribed mRNA catabolic process
GO:0034623 3.22294709479738e-22 75.9920053297801 0.421424122898069
16 174 cellular macromolecular complex disassembly
GO:0006402 4.68991096999289e-22 74.0948667966212 0.431112033769289
16 178 mRNA catabolic process
GO:0032984 5.14382636216027e-22 73.6351307717146 0.433534011487094
16 179 macromolecular complex disassembly
GO:0072594 4.40408747444263e-21 63.7312430011198 0.494083454432219
16 204 establishment of protein localization to organelle
GO:0006401 4.77093386266361e-21 63.3895850737956 0.496505432150024
16 205 RNA catabolic process
GO:0019058 3.3435978412586e-20 55.6220318237454 0.559476852812954
16 231 viral infectious cycle
GO:0022415 2.27217933231035e-19 48.9111302847282 0.629714206629299
16 260 viral reproductive process
GO:0071845 9.42866345048754e-19 44.4556166535742 0.687841671856619
16 284 cellular component disassembly at cellular level
GO:0022411 1.18057012549749e-18 43.7894736842105 0.697529582727839
16 288 cellular component disassembly
GO:0006605 1.16826820766969e-16 28.8479212253829 1.14801743823957
17 474 protein targeting
GO:0016032 1.46671598495031e-15 27.0654567453116 1.09231195073005
16 451 viral reproduction
GO:0033365 3.09473638919039e-15 25.7219854888863 1.14559546052176
16 473 protein localization to organelle
Tissue: Nerve_Tibial=>Artery_Tibial Type: asymmetric
SourceGene: GBA2(ENSG00000070610.10)
TargetGeneSet: RPL22 ZBTB40 PPIE RPS8 RP11 WDR77 BCAS2 HIST2H2AC
SMG5 HDGF KCNJ9 SMYD2 LYPLAL1 PSEN2 DDIT3 HAAO RPL31 ANKRD44
SATB2 TATDN2 RPL15 CTDSPL KRBOX1 ZDHHC3 RNF123 RPL29 ZNF717 DZIP3
NDUFB4 ALG1L EIF4G1 TRA2B OCIAD1 CLOCK MAPK10 NDUFC1 TAF9 PCDHB16

ARL10 RPS10 RPL10A XPO5 MICAL1 C7orf50 IQCE GPNMB CDK13 IMPDH1 RP4
DCTN6 C8orf40 PAF1 GS1 ZNF706 DCTN3 FAM27D1 VPS13A RABGAP1 RPL35
RPL12 SLC27A4 CAMSAP1 CXCL12 RP13 ACTR1A RPL27A MRVI1-AS1 MRVI1
RPS13 RARRES3 CPT1A CTD CBL LTBR RARG STAT2 TIMELESS
PIP4K2C YEATS4 RP1 CHPT1 EID3 C12orf51 MLEC DIABLO TPTE2P6
KATNAL1 DGKZP1 MED4 DHRS12 POMT2 AKT1 KIAA0284 AVEN RPLP1
ZNF592 NUDT21 DOK4 FHOD1 WWOX COX4I1 RPL13 KIF1C KCTD11 FLII
C17orf108 SPAG5-AS1 KIAA0100 RPL23A PIP4K2B RPL19 MSL1 ACLY
ATXN7L3 TRIM65 RPL36 DNMT1 KCTD15 RPL18 TBC1D20 DUSP15 SOGA1 RALGAPB
DHX35 ELMO2 COL18A1 CECR5 PI4KA RANGAP1 TCF20 SYP MAGED2 KLF8
NAP1L2 TCEAL7
GOBPID Pvalue OddsRatio ExpCount Count Size Term
GO:0000184 4.19812441977784e-20 29.0904301075269 0.922289114940143
19 119 nuclear-transcribed mRNA catabolic process, nonsense-mediated decay
GO:0006415 2.67184523221966e-19 34.4957325746799 0.705279911424815
17 91 translational termination
GO:0006414 3.49151455770065e-18 28.9793062200957 0.813784513182479
17 105 translational elongation
GO:0006614 3.49151455770065e-18 28.9793062200957 0.813784513182479
17 105 SRP-dependent cotranslational protein targeting to membrane
GO:0006613 4.13247995207642e-18 28.6516853932584 0.821534841879455
17 106 cotranslational protein targeting to membrane
GO:0045047 4.13247995207642e-18 28.6516853932584 0.821534841879455
17 106 protein targeting to ER
GO:0072599 4.13247995207642e-18 28.6516853932584 0.821534841879455
17 106 establishment of protein localization to endoplasmic reticulum
GO:0070972 2.74357353697432e-17 25.2262636789995 0.914538786243167
17 118 protein localization to endoplasmic reticulum
GO:0000956 3.85574698609524e-17 19.3255197132616 1.30980554978894
19 169 nuclear-transcribed mRNA catabolic process
GO:0006402 1.03274059701408e-16 18.2200581591939 1.37955850806173
19 178 mRNA catabolic process
GO:0006413 1.08657065817354e-16 20.2993013655129 1.17804996194035
18 152 translational initiation
GO:0043624 1.22379974471189e-16 20.1475177304965 1.18580029063733
18 153 cellular protein complex disassembly
GO:0043241 2.1899510816277e-16 19.4211246200608 1.22455193412221
18 158 protein complex disassembly
GO:0034623 1.23826262812777e-15 17.4095744680851 1.34855719327382
18 174 cellular macromolecular complex disassembly
GO:0006401 1.4638504110895e-15 15.5455543993525 1.58881738288008
19 205 RNA catabolic process
GO:0019080 1.94316001343214e-15 18.9697564807541 1.17029963324337
17 151 viral genome expression
GO:0019083 1.94316001343214e-15 18.9697564807541 1.17029963324337
17 151 viral transcription
GO:0032984 2.05195752714321e-15 16.8629575789613 1.3873088367587 18
179 macromolecular complex disassembly
GO:0006612 2.43064543588102e-15 18.6881578947368 1.18580029063733
17 153 protein targeting to membrane
GO:0072594 2.0648227822419e-14 14.5706932052162 1.5810670541831 18
204 establishment of protein localization to organelle
GO:0019058 2.25520135315448e-12 11.8113625184456 1.79032592900145
17 231 viral infectious cycle
GO:0071845 6.0318344171858e-12 10.130939049752 2.20109334994118 18
284 cellular component disassembly at cellular level
GO:0022411 7.62577873374238e-12 9.97801418439716 2.23209466472908
18 288 cellular component disassembly
GO:0006605 1.05901152812948e-11 7.51017699115044 3.67365580236662
22 474 protein targeting
GO:0022415 1.50265383879605e-11 10.3804201862681 2.01508546121376
17 260 viral reproductive process
GO:0033365 3.57356105773459e-09 6.2482828856994 3.66590547366964 19

473	protein localization to organelle
GO:0016032	1.06978778669909e-08	6.14977150999951	3.49539824233617
18	451	viral reproduction
Tissue: Nerve_Tibial=>Artery_Tibial	Type:	asymmetric
SourceGene:	HDAC6(ENSG00000094631.13)
TargetGeneSet:	RPL22	HMGN2	KHDRBS1 RPS8	IPP	RP11	CD53	DPM3
KCNJ9	FCER1G	FCGR3A	SFT2D2	KCNT2	SNRPE	SRGAP3	CAPN8	LRPPRC	CAMKMT	UBC
RPL31	TTL	MZT2A	ANKRD44 SATB2	STK36	SLC23A3 RPL15	RPL14	RPL24
ALCAM	ALG1L	FAM86HP PCOLCE2 PARL	CLOCK	NAP1L5	RXFP1	CTD	THOC3	RP1
RPS10	RPL10A	MRPL2	EFHC1	MRAP2	SNHG5	MICAL1	MRPS12	C7orf50 ABCB5
TOMM7	OSBPL3	EIF4H	GTF2IP1 SSBP1	RARRES2 RPL7	EIF3H	EEF1D	RPS6
POLR1E	C9orf103		SLC44A1 C9orf43 RPL35	RPL12	AKR1C2	DDIT4	EIF3F
RPS13	MS4A7	RARRES3 RPS3	PHB2	AEBP2	CYP27B1 STAT2	RPL18AP3		RPL6
MLEC	MED4	TMCO3	RPS29	GS1	RPS3AP6 RPLP1	BCL7C	NETO2	NUDT21
FHOD1	EXOSC6	COX4I1	RPL13	C17orf108		KIAA0100		RPL23A	RPL17
RPL19	CCR10	HEXIM2	PPP4R1	RPS15	SNRPEP4 RPL36	RPL18A	FXYD1	OVOL3
HNRNPL	RPS16	PRR24	RPL18	SIGLEC9 RPL28	ZNF530	RPL24P2 NDRG3	ELMO2
PPDPF	SLC2A4RG		CLIC6	GPKOW	SYP	SRPX2	RBMX	RPL10
GOBPID	Pvalue	OddsRatio	ExpCount	Count	Size	Term
GO:0006415	1.82808473763696e-41	91.5428634555899	0.642308490761885
29	91	translational termination
GO:0006414	2.94892989231113e-41	79.3	0.741125181648329	30	105
translational elongation
GO:0006614	2.27785307734369e-39	74.6065248738284	0.741125181648329
29	105	SRP-dependent cotranslational protein targeting to membrane
GO:0006413	2.67922162204485e-39	54.2057142857143	1.07286692962425
32	152	translational initiation
GO:0006613	3.12033054069613e-39	73.6324497420388	0.748183516711646
29	106	cotranslational protein targeting to membrane
GO:0045047	3.12033054069613e-39	73.6324497420388	0.748183516711646
29	106	protein targeting to ER
GO:0072599	3.12033054069613e-39	73.6324497420388	0.748183516711646
29	106	establishment of protein localization to endoplasmic reticulum
GO:0070972	1.05707251201559e-37	63.6509158072957	0.832883537471455
29	118	protein localization to endoplasmic reticulum
GO:0000184	1.39080826296839e-37	62.9392694063927	0.839941872534773
29	119	nuclear-transcribed mRNA catabolic process, nonsense-mediated decay
GO:0043241	5.24348696189237e-37	48.8945325496285	1.11521694000415
31	158	protein complex disassembly
GO:0043624	8.84432528147779e-36	48.1910569105691	1.07992526468756
30	153	cellular protein complex disassembly
GO:0032984	3.3630335073767e-35	41.8948420251237	1.26344197633382
31	179	macromolecular complex disassembly
GO:0000956	2.19848923185318e-34	42.5959232613909	1.19285862570064
30	169	nuclear-transcribed mRNA catabolic process
GO:0019080	2.76508618823873e-34	46.3264091623625	1.06580859456093
29	151	viral genome expression
GO:0019083	2.76508618823873e-34	46.3264091623625	1.06580859456093
29	151	viral transcription
GO:0006612	4.17468835703725e-34	45.5728015908087	1.07992526468756
29	153	protein targeting to membrane
GO:0034623	5.59434162136755e-34	41.1024305555556	1.22815030101723
30	174	cellular macromolecular complex disassembly
GO:0006402	1.15585282491115e-33	39.9802927927928	1.2563836412705 30
178	mRNA catabolic process
GO:0072594	8.61177604755942e-32	33.9439655172414	1.43990035291675
30	204	establishment of protein localization to organelle
GO:0006401	1.00388074289027e-31	33.747619047619 1.44695868798007	30
205	RNA catabolic process
GO:0019058	1.19180948039549e-28	27.8219856232199	1.63047539962632
29	231	viral infectious cycle
GO:0022411	1.48329247308381e-28	23.9410314024223	2.03280049823542
31	288	cellular component disassembly

GO:0022415	1.56525903941967e-28	25.5778985507246	1.83516711646253
30	260	viral reproductive process
GO:0071845	2.29052558913642e-27	23.121719160105 2.00456715798215	30
284	cellular component disassembly at cellular level
GO:0016032	1.9413471071617e-21	13.784639746635 3.18330911355616	30
451	viral reproduction
GO:0033365	7.61119892224637e-21	13.0793829947329	3.33859248494914
30	473	protein localization to organelle
GO:0006605	8.08510821876735e-21	13.0489864864865	3.34565082001246
30	474	protein targeting
GO:0042274	6.63466085483646e-06	41.7930029154519	0.127050031139714
4	18	ribosomal small subunit biogenesis
GO:0006364	8.60311641238993e-06	10.8262615301139	0.734066846585011
7	104	rRNA processing
GO:0016072	1.24500930613032e-05	10.1913132345427	0.776416856964916
7	110	rRNA metabolic process
Tissue: Nerve_Tibial=>Artery_Tibial	Type:	asymmetric
SourceGene:	HLA-DMA(ENSG00000204257.8)
TargetGeneSet:	RP11	HMGN2	KHDRBS1 STK40	RPS8	IPP	POMGNT1 SMG5	HDGF
LYPLAL1 EIF3FP3 RPL31	MAP3K2	SATB2	PPP1R7	TATDN2	CHDH	RPL10AP6
DZIP3	LIAS	OTUD4	COX7C	CTD	ARL10	RPS10	RPL10A	XPO5	MRPS12
GTF2H5	C7orf50 TOMM7	SLC4A2	AGPAT6	CHCHD7	RPL7	COX6C	EIF3H	RPS6
DCTN3	C9orf103	RPL35	RPL12	VAV2	RP13	CARS	RPS13	DAK	NXF1
PRDM10	TULP3	TNFRSF1A	STAT2	XPOT	NDUFA12 CHPT1	EID3	C12orf51
DHRS12	CMTM5	RPS29	RPL7AP6 AKT1	KIAA0284	HERC2P3 HMGN2P5 RPLP1
MAN2A2	C16orf79	FHOD1	HSDL1	COX4I1	CYB5D2	MINK1	FLII	C17orf108
RPL17	RPL19	HEXIM2	MRPL45P2	NPEPPS	BAHCC1	PPP4R1	DNMT1	PRKCSH
SUGP2	COX6B1	NDUFA3	RPS9	RPL28	ZNF667	ZSCAN1	TBC1D20 ELMO2	RPS21
APOBEC3G	RANGAP1 EFCAB6	SYP	KLF8	RP3	RPL10
GOBPID	Pvalue	OddsRatio	ExpCount	Count	Size	Term
GO:0006415	1.81110837456362e-21	48.2837837837838	0.535257075634904
17	91	translational termination
GO:0000184	6.10927534779988e-21	37.9444362346682	0.699951560445644
18	119	nuclear-transcribed mRNA catabolic process, nonsense-mediated decay
GO:0006414	2.42646128577078e-20	40.5625 0.617604318040274	17	105
translational elongation
GO:0006614	2.42646128577078e-20	40.5625 0.617604318040274	17	105
SRP-dependent cotranslational protein targeting to membrane
GO:0006613	2.87702575868489e-20	40.1039325842697	0.623486263926372
17	106	cotranslational protein targeting to membrane
GO:0045047	2.87702575868489e-20	40.1039325842697	0.623486263926372
17	106	protein targeting to ER
GO:0072599	2.87702575868489e-20	40.1039325842697	0.623486263926372
17	106	establishment of protein localization to endoplasmic reticulum
GO:0070972	1.95134119188602e-19	35.3094059405941	0.694069614559546
17	118	protein localization to endoplasmic reticulum
GO:0006413	5.87161916046592e-19	28.5337491646246	0.894055774686873
18	152	translational initiation
GO:0000956	4.09461599280805e-18	25.2910941978847	0.994048854750536
18	169	nuclear-transcribed mRNA catabolic process
GO:0006402	1.05140400979372e-17	23.8533582089552	1.04698636772542
18	178	mRNA catabolic process
GO:0019080	1.46579017978226e-17	26.5522388059701	0.888173828800775
17	151	viral genome expression
GO:0019083	1.46579017978226e-17	26.5522388059701	0.888173828800775
17	151	viral transcription
GO:0006612	1.84006815045489e-17	26.1580882352941	0.899937720572971
17	153	protein targeting to membrane
GO:0043624	1.84006815045489e-17	26.1580882352941	0.899937720572971
17	153	cellular protein complex disassembly
GO:0043241	3.20371381281421e-17	25.2216312056738	0.92934745000346
17	158	protein complex disassembly
GO:0072594	1.22472715482202e-16	20.4814636494945	1.19991696076396

18	204	establishment of protein localization to organelle
GO:0006401	1.3366520779621e-16	20.3705004389816	1.20579890665006
18	205	RNA catabolic process
GO:0034623	1.67278171419204e-16	22.625796178344 1.02345858418103	17
174	cellular macromolecular complex disassembly
GO:0032984	2.70993198091825e-16	21.9197530864198	1.05286831361151
17	179	macromolecular complex disassembly
GO:0019058	1.11617743754793e-15	17.8511667017028	1.3587294996886 18
231	viral infectious cycle
GO:0022415	8.90746454878533e-15	15.6797829036635	1.52930593038544
18	260	viral reproductive process
GO:0006605	2.57460605906515e-14	10.7496839443742	2.78804235001038
22	474	protein targeting
GO:0071845	5.80436860630791e-13	13.2013108614232	1.67047263165179
17	284	cellular component disassembly at cellular level
GO:0022411	7.28058789392234e-13	13.0027675276753	1.69400041519618
17	288	cellular component disassembly
GO:0033365	2.6233042470814e-12	9.45016131771099	2.78216040412428
20	473	protein localization to organelle
GO:0016032	1.07578100071674e-11	9.28542368125702	2.65275759463013
19	451	viral reproduction
Tissue: Artery_Tibial=>Nerve_Tibial	Type:	asymmetric
SourceGene:	LAMC3(ENSG00000050555.11)
TargetGeneSet:	THBS3	BZW1	RPL37A	C3orf39 SEC61A1 EEF1A1P19	RPS23
HLA-DOA RPS10	FOXP4	EEF1A1	AHCYL2	MTFR1	EEF1D	RPS6	RPL12	LRP10	FAU
TM7SF3	RPS3AP6 OSTBETA UBA52	RPS16	NUMBL	RPS9	RPS21	CTA	SLC16A2
RPL10
GOBPID	Pvalue	OddsRatio	ExpCount	Count	Size	Term
GO:0006414	1.70532207841082e-22	184.145256916996	0.17438239568196
13	105	translational elongation
GO:0045047	3.21172747861733e-20	152.478723404255	0.17604318040274
12	106	protein targeting to ER
GO:0072599	3.21172747861733e-20	152.478723404255	0.17604318040274
12	106	establishment of protein localization to endoplasmic reticulum
GO:0070972	1.23360123967505e-19	135.103773584906	0.195972597052107
12	118	protein localization to endoplasmic reticulum
GO:0006415	7.71872065757033e-19	151.747115384615	0.151131409591032
11	91	translational termination
GO:0006614	4.01661343482609e-18	129.020458265139	0.17438239568196
11	105	SRP-dependent cotranslational protein targeting to membrane
GO:0006613	4.47797614809388e-18	127.653441295547	0.17604318040274
11	106	cotranslational protein targeting to membrane
GO:0000184	1.68002490885081e-17	112.185897435897	0.197633381772888
11	119	nuclear-transcribed mRNA catabolic process, nonsense-mediated decay
GO:0072594	1.05491597867372e-16	74.140625	0.338800083039236	12
204	establishment of protein localization to organelle
GO:0019080	2.49118165200418e-16	86.35	0.250778492837866	11	151
viral genome expression
GO:0019083	2.49118165200418e-16	86.35	0.250778492837866	11	151
viral transcription
GO:0006413	2.68329475132038e-16	85.7315875613748	0.252439277558646
11	152	translational initiation
GO:0006612	2.88874998161738e-16	85.1218851570964	0.254100062279427
11	153	protein targeting to membrane
GO:0043624	2.88874998161738e-16	85.1218851570964	0.254100062279427
11	153	cellular protein complex disassembly
GO:0043241	4.14666655261344e-16	82.1978021978022	0.26240398588333
11	158	protein complex disassembly
GO:0000956	8.82122496932847e-16	76.4162609542356	0.280672617811916
11	169	nuclear-transcribed mRNA catabolic process
GO:0034623	1.22248223566935e-15	74.0462482302973	0.288976541415819
11	174	cellular macromolecular complex disassembly
GO:0006402	1.57610865522108e-15	72.2524182404422	0.295619680298941

11	178	mRNA catabolic process
GO:0032984	1.67790682697873e-15	71.8173076923077	0.297280465019722
11	179	macromolecular complex disassembly
GO:0006401	7.60023279136492e-15	62.078905630452 0.340460867760017	11
205	RNA catabolic process
GO:0019058	2.85353956380093e-14	54.6423076923077	0.383641270500311
11	231	viral infectious cycle
GO:0022415	1.05121680674353e-13	48.179796107507 0.431804027402948	11
260	viral reproductive process
GO:0071845	2.77153128770952e-13	43.8698224852071	0.471662860701682
11	284	cellular component disassembly at cellular level
GO:0022411	3.23054316537605e-13	43.2241044154402	0.478305999584804
11	288	cellular component disassembly
GO:0033365	2.49276638795817e-12	30.295010845987 0.785551172929209	12
473	protein localization to organelle
GO:0006605	2.55549620889547e-12	30.2272727272727	0.78721195764999
12	474	protein targeting
GO:0016032	4.19569745135523e-11	26.8980769230769	0.749013909072037
11	451	viral reproduction
GO:0042274	3.23105168313163e-06	137.257142857143	0.0298941249740502
3	18	ribosomal small subunit biogenesis
Tissue: Artery_Tibial=>Nerve_Tibial	Type:	asymmetric
SourceGene:	NGFRAP1(ENSG00000166681.9)
TargetGeneSet:	ZCCHC17 PSMB2	NDUFS5	UQCRH	ST6GALNAC5	ALG14	CAPZA1
MRPS21	TMCO1	RP4	PFN1P1	SNRPE	LINC00467	RP11	TARBP1	PTRHD1	OST4
MRPL33	MORN2	MDH1	NFU1	TPRKB	TMSB10	NDUFB3	BRK1	UBE2E1	CCDC72
VPRBP	SPCS1	NEK4	MRPL47	NDUFB5	ATP5I	STX18	EVC2	MAN2B2	OCIAD1
NDUFC1	MGST2	FSTL5	NEIL3	SUB1	CTD	UQCRQ	PHF15	PDF	DIAPH1
TCOF1	ATOX1	PTTG1	AIF1	C6orf48 KIFC1	COX6A1P2	FOXP4	KIAA0240
RPL39P3 COX7A2	HMGN3	PRSS35	PEX7	NUDT1	NDUFA4	TOMM7	MALSU1	LSM5
SEC61G	TP53TG1 ECD	PMS2P1	AP1S1	GSTK1	MRPL15	PENK	C8orf59 DECR1
UQCRB	COX6C	MRPL13	C9orf123	SEC61B	TXN	ATP5C1	TAF3	POLR2L
NUP98	IMMP1L	SLC43A3 C11orf10	SNX15	RELA	ARAP1-AS1	CLNS1A
ATP5L	ZC3H10	C12orf45	GCN1L1	CCDC92	POMP	COMMD6	ACIN1	RPS29	ERH
RP3	EFCAB11 NDUFB1	IFI27L1 C14orf2 HMGN2P5 SRP14	GTF2A2	COX5A	WASH3P
NDUFAB1 NSMCE1	MIR4519 PSMB10	TRAPPC1 ATP5H	H3F3B	SEC11C	UQCR11	C19orf70
C19orf79	MAP2K7	PIN1	RAVER1	ZNF653	ZNF625	ZNF726	ANKRD27 COX6B1
POLR2I	MIA	CCDC97	SNRPD2	SLC17A7 TBC1D17 NDUFA3	C20orf7 SNRPB2	ROMO1
PFDN4	ATP5J	SNRPD3	UQCR10	LGALS1	CBX7	RP6	EEF1B2P3	NDUFB11
TIMP1	PRICKLE3	PDZD11	BCORL1	SSR4	DKC1
GOBPID	Pvalue	OddsRatio	ExpCount	Count	Size	Term
GO:0022904	3.60072593790556e-26	43.2506894649752	0.797176665974673
22	96	respiratory electron transport chain
GO:0022900	3.16534702822751e-24	30.3761493452215	1.11272576292298
23	134	electron transport chain
GO:0045333	1.28520448731778e-23	28.3181148748159	1.1791571517542 23
142	cellular respiration
GO:0015980	3.892942073092e-18	13.1103023516237	2.54930454639817
25	307	energy derivation by oxidation of organic compounds
GO:0006091	1.01142087492698e-15	9.58383613845665	3.55407930247042
26	428	generation of precursor metabolites and energy
GO:0006119	3.96541852571518e-14	34.5048309178744	0.481627569026365
12	58	oxidative phosphorylation
GO:0042773	1.09909586770632e-13	40.072629969419 0.390284409383434	11
47	ATP synthesis coupled electron transport
GO:0042775	1.09909586770632e-13	40.072629969419 0.390284409383434	11
47	mitochondrial ATP synthesis coupled electron transport
GO:0006120	4.45026373362782e-10	36.4872448979592	0.298941249740502
8	36	mitochondrial electron transport, NADH to ubiquinone
GO:0042776	4.43127881906548e-08	77.8423913043478	0.107951006850737
5	13	mitochondrial ATP synthesis coupled proton transport
GO:0015985	2.85361870179688e-07	47.8862876254181	0.149470624870251
5	18	energy coupled proton transport, down electrochemical gradient

GO:0015986 2.85361870179688e-07 47.8862876254181 0.149470624870251
5 18 ATP synthesis coupled proton transport
GO:0006122 2.22034742164306e-06 367.435897435897 0.0332156944156114
3 4 mitochondrial electron transport, ubiquinol to cytochrome c
GO:0006200 2.84068659625795e-06 12.9933628318584 0.622794270292713
7 75 ATP catabolic process
GO:0000387 5.19105516446692e-06 23.9214046822742 0.257421631720988
5 31 spliceosomal snRNP assembly
Tissue: Nerve_Tibial=>Artery_Tibial Type: cluster
SourceGene: NUP98(ENSG00000110713.11)
TargetGeneSet: CDK11A PARK7 UBR4 WBP1 CNKSR1 SRSF4 PPT1 PPCS
UQCRH ATPAF1 SCP2 RP4 LRRC40 CRYZ PIGK RWDD3 ACTG1P4 TAF13
HBXIP RP11 ATP5F1 SIKE1 C1orf54 CRTC2 PMVK C1orf85 HDGF DCAF8 SDHC
MGST3 CREG1 BRP44 DNM3OS TIMM17A IKBKE NSL1 LYPLAL1 ARV1 RRM2 OST4
CGREF1 MPV17 RBKS USP34 COMMD1 MDH1 NFU1 TPRKB BOLA3 SNRNP200
TXNDC9 CCDC74A PSMD14 H3F3AP4 ATP5G3 DUSP19 MOB4 C2orf47 NDUFB3 IMPDH1P10
SENP2 MYEOV2 BRK1 LSM3 MLH1 ENTPD3-AS1 LZTFL1 ARIH2 ABHD14A
SPCS1 ACOX2 TFG ABHD10 SLC35A5 NDUFB4 RPL32P3 MRPL3 ANAPC13 HLTF
LRRC34 MRPL47 NDUFB5 DNAJC19 RNF168 KIAA0226 FAM193A GNPDA2 OCIAD1
MRPL1 MRPS18C SLC10A6 LAMTOR3 PPA2 TRAM1L1 NUDT6 NDUFC1 SCOC ANAPC10
PET112 SCRG1 FBXO8 C5orf55 FASTKD3 SUB1 C5orf43 MAST4-AS1 COX7C
CETN3 COMMD10 UQCRQ LEAP2 SKP1 SIL1 PDF PCDHB9 PCDHB10 DIAPH1 GM2A
ATOX1 UBLCP1 NUDCD2 HMMR RPL26L1 HIGD2A UIMC1 FGFR4 LY86 TMEM14B NOL7
HMGN4 BAG6 HNRNPC BRPF3 PNPLA1 MRPS10 MRPS18A TMEM14A PHF3 COX7A2
IRAK1BP1 HMGN3 CYB5R4 GTF3C6 ASF1A PKIB MRPS12 AIG1 PEX3
SF3B5 RPA3 NDUFA4 RPS2P32 LSM5 SEPT7 EPDR1 PPIA SEC61G CHCHD2
SKP1P1 STAG3L4 TRIM74 UPK3B TP53TG1 SRI SMURF1 ZNHIT1 RABL5 CAPZA2
NDUFA5 C7orf73 NDUFB2 MRPS33 KIAA1147 MLL3 RBM33 NAT1 SORBS3
KIF13B DCTN6 LSM1 ATP6V1H RAB2A C8orf45 COPS5 TCEB1 TMEM70 PAF1
MRPS35 DECR1 HRSP12 COX6C MRPL13 ASAP1 CTD RLN2 DENND4C NDUFB6
DCTN3 CCL19 GBA2 SHC3 KIAA0368 ATP6V1G1 CDK9 CIZ1
RALGDS LINC00094 NOTCH1 TMEM141 ATP5C1 CDC123 MSRB2 C10orf68
PARD3 MRPS16 GHITM ZRANB1 ECHS1 NUP98 SMPD1 C11orf58 IMMP1L ELP4
C11orf74 ETFA FNBP4 PATL1 MTA2 BAD SF1 FRMD8 RNASEH2C
MRPL11 NDUFS8 MYEOV PPFIA1 CLNS1A CCDC90B TMEM126B TMEM126A
C11orf70 DCUN1D5 SIK3 ATP5L MLL TBCEL FEZ1 NFRKB DYRK4 ATN1
ALG1L2 MAGOHB CCDC91 COX14 POU6F1 PCBP2 SMARCC2 BAZ2A R3HDM2 C12orf29
TMTC3 UBE2N MRPL42 CCDC53 ARPC3 C12orf51 MED13L COX6A1 TRIAP1 POP5
TRAC RAN MPHOSPH8 MRP63 EFHA1 TPTE2P1 MTMR6 POMP MED4
COMMD6 A2LD1 C14orf126 BAZ1A MBIP MGAT2 AP5M1 ACTR10 PSMA3
ATP6V1D BCRP2 SLIRP TTC8 NDUFB1 C14orf129 C14orf2 NDN SRP14
SERF2 DYX1C1-CCPG1 GTF2A2 FAM96A PPIB ZNF609 PARP6 WDR61 TMED3
C15orf40 MRPS11 MAN2A2 NDUFB10 SRRM2 SMG1 NDUFAB1 ATXN2L ITFG1
CNOT1 FAM65A SLC12A4 DHX38 COX4NB PITPNA PFN1 C17orf61-PLSCR3 FLII GIT1
MLLT6 STARD3 CASC3 STAT5A VPS25 GPATCH8 EFTUD2 SCRN2 SPAG9 ICT1
ATP5H ANAPC11 IER3IP1 C18orf32 RBFA BSG UQCR11 MRPL54 SAFB
C19orf70 NDUFA11 PIN1 DNM2 ASNA1 PRDX2 NDUFB7 ZNF333 AKAP8L
EPS15L1 NDUFA13 RPSAP58 POP4 CYP2G1P GEMIN7 AP2S1 C19orf63 JOSD2
TMEM230 SNRPB2 COMMD7 MAPRE1 CTSA ZNF334 ATP5E PPIAP22 ATP5J SCAF4 CBR1
ERVH48-1 CSTB MCM3AP DIP2A MRPL40 DGCR6L PI4KAP2 UQCR10 MTMR3
EIF4ENIF1 RP1 TXN2 LGALS1 GTPBP1 COX5BP7 RBX1 NDUFA6 PRDX4
ATP6AP2 FUNDC1 OTUD5 PHF8 PDZD11 MED12 ITGB1BP2 COX7B TAF9B
NAP1L3 TCEAL8 NGFRAP1 RPL39 C1GALT1C1 THOC2 FAM127C CETN2 SSR4
MECP2 FUNDC2 VAMP7
GOBPID Pvalue OddsRatio ExpCount Count Size Term
GO:0022904 4.81829348728712e-29 23.6666666666667 2.18559269254723
32 96 respiratory electron transport chain
GO:0045333 1.95904953081311e-25 14.9553044569994 3.23285585772611
34 142 cellular respiration
GO:0022900 3.86376520601551e-25 15.4767527428419 3.05072313334717
33 134 electron transport chain
GO:0042773 2.87665841927614e-15 23.2357002988766 1.07002975572625
16 47 ATP synthesis coupled electron transport

GO:0042775	2.87665841927614e-15	23.2357002988766	1.07002975572625
16	47	mitochondrial ATP synthesis coupled electron transport
GO:0015980	3.07145615883108e-15	6.06179971988796	6.98934329804166
35	307	energy derivation by oxidation of organic compounds
GO:0006119	6.66748543546713e-15	18.713023764853 1.32046225174728	17
58	oxidative phosphorylation
GO:0006120	1.84479784875503e-14	28.4848484848485	0.819597259705211
14	36	mitochondrial electron transport, NADH to ubiquinone
GO:0006091	2.74007973506067e-12	4.44983883964545	9.74410075427306
37	428	generation of precursor metabolites and energy
GO:0015985	7.63100566924428e-08	27.8873517786561	0.409798629852605
7	18	energy coupled proton transport, down electrochemical gradient
GO:0015986	7.63100566924428e-08	27.8873517786561	0.409798629852605
7	18	ATP synthesis coupled proton transport
GO:0042776	1.99560289510032e-07	37.4568774878372	0.295965677115771
6	13	mitochondrial ATP synthesis coupled proton transport
GO:0015992	2.22247353014058e-06	7.87393640841917	1.50259497612622
10	66	proton transport
GO:0006818	2.94185482576975e-06	7.6013403956329 1.54812815722095	10
68	hydrogen transport
Tissue: Nerve_Tibial=>Artery_Tibial	Type:	asymmetric
SourceGene:	PIK3IP1(ENSG00000100100.7)
TargetGeneSet:	HMGN2	RPS8	CCDC17	RP11	C1orf54 CREB3L4 RPS27	SSR2
NCOA1	EIF3FP3 SATB2	RPL15	CTDSPL	RPL14	ZBTB47	RPL29	RPL10AP6
PCOLCE2 EIF4G1	GPR125	LIAS	C5orf34 SIL1	CTB	ARL10	LYRM4	RPL15P3
RPS10	RPL10A	SNHG5	BEND3	MRPS12	C7orf50 FBXO24	RPS20	RPL7	C8orf73
DCTN3	VPS13A	RPL35	RPL12	RPL7A	FAM208B STIM1	RPL27A	RPS13	PTPRCAP
CHPT1	RPL18AP3		EID3	RPL6	RPLP0	DYNC1H1 AKT1	RPLP1	C16orf57
RPL13	KIF1C	KIAA0100		RPL19	MRPL45P2		BAHCC1	FASN	RPL17
RPS15	PRKCSH	CTD	RPL18A	C19orf12		RPL18	RPS9	RPL28	A1BG	RPS5
RPL12P4 PPDPF	EIF3FP1 MICALL1 CTA	GLUD2	RPL10
GOBPID	Pvalue	OddsRatio	ExpCount	Count	Size	Term
GO:0006614	4.11941428480046e-45	157.719008264463	0.443221922358314
28	105	SRP-dependent cotranslational protein targeting to membrane
GO:0006613	5.58574977734701e-45	155.686091686092	0.447443083523632
28	106	cotranslational protein targeting to membrane
GO:0045047	5.58574977734701e-45	155.686091686092	0.447443083523632
28	106	protein targeting to ER
GO:0072599	5.58574977734701e-45	155.686091686092	0.447443083523632
28	106	establishment of protein localization to endoplasmic reticulum
GO:0006415	7.17549268866088e-45	177.758272058824	0.384125666043872
27	91	translational termination
GO:0070972	1.69411579208698e-43	134.814814814815	0.498097017507439
28	118	protein localization to endoplasmic reticulum
GO:0000184	2.21045964572133e-43	133.324009324009	0.502318178672756
28	119	nuclear-transcribed mRNA catabolic process, nonsense-mediated decay
GO:0006414	6.22826737110555e-43	145.710407239819	0.443221922358314
27	105	translational elongation
GO:0006413	4.33690436240561e-40	97.6168132942326	0.641616497128226
28	152	translational initiation
GO:0006612	5.29654982714096e-40	96.8290909090909	0.645837658293544
28	153	protein targeting to membrane
GO:0000956	1.07861822522739e-38	85.7451106812809	0.71337623693862
28	169	nuclear-transcribed mRNA catabolic process
GO:0019080	3.36234410657138e-38	91.361954459203 0.637395335962909	27
151	viral genome expression
GO:0019083	3.36234410657138e-38	91.361954459203 0.637395335962909	27
151	viral transcription
GO:0043624	4.94201641057437e-38	89.8991596638655	0.645837658293544
27	153	cellular protein complex disassembly
GO:0006402	5.12968344097234e-38	80.549494949495 0.751366687426476	28
178	mRNA catabolic process
GO:0043241	1.26368164806305e-37	86.4375841939829	0.66694346412013

27	158	protein complex disassembly
GO:0034623	2.07136599701717e-36	76.9429771908763	0.734482042765207
27	174	cellular macromolecular complex disassembly
GO:0072594	2.97174463481531e-36	68.5247933884298	0.861116877724725
28	204	establishment of protein localization to organelle
GO:0006401	3.43416062535662e-36	68.1328539633624	0.865338038890042
28	205	RNA catabolic process
GO:0032984	4.68495160608245e-36	74.3858359133127	0.755587848591793
27	179	macromolecular complex disassembly
GO:0019058	6.60041849918665e-33	55.2223183391003	0.975088229188292
27	231	viral infectious cycle
GO:0022415	1.80574338752716e-31	48.2503155768745	1.09750190298249
27	260	viral reproductive process
GO:0071845	2.09364027324473e-30	43.6702906843671	1.19880977095011
27	284	cellular component disassembly at cellular level
GO:0022411	3.08201273765649e-30	42.9888438133874	1.21569441561138
27	288	cellular component disassembly
GO:0033365	3.05041458481956e-27	28.4652308558559	1.99660923119507
29	473	protein localization to organelle
GO:0006605	3.2420308424832e-27	28.3992275280899	2.00083039236039
29	474	protein targeting
GO:0016032	6.09273795948175e-25	26.1571864594895	1.90374368555809
27	451	viral reproduction
GO:0022613	4.41542322260067e-06	9.85862098947918	0.949761262196388
8	225	ribonucleoprotein complex biogenesis
GO:0071843	6.88629834733271e-06	9.25198072367884	1.00885751851083
8	239	cellular component biogenesis at cellular level
Tissue: Nerve_Tibial=>Artery_Tibial	Type:	asymmetric
SourceGene:	PLXNA2(ENSG00000076356.6)
TargetGeneSet:	RPL22	RP11	RHCE	HMGN2	KHDRBS1	RPS8	IPP	RP4
CHI3L2	ANP32E	CREB3L4	SSR2	TOMM20	NCAPH	RPL31	FMNL2	EEF1B2	RPL15
CTDSPL	RPL14	RNF123	RPL10AP6		PSMC1P1	RPL24	PLXNA1	PARL	TFRC
YTHDC1	AIMP1	METTL14	COX7C	RIOK2	CTD	NPM1	MRPS18B	RPL10A	MRPL2
MRPS12	C7orf50	TOMM7	EIF4H	SSBP1	CHCHD7	EIF3H	RPL8	RPS6	RPL35
RPL12	RPL7A	C10orf35		C10orf116		AGAP11	CCDC147	WDR11	C11orf58
RPS13	PLA2G16	U4	RPS3	C11orf1	NRGN	RPL41	RPL14P1	RPL18AP3		TCHP
DIABLO	IFT88	SAP18	DIAPH3-AS1		RPL7AP6	SLC30A4	RSL24D1	RPLP1	PEAK1	RPS2
NUDT21	RPL13	ALDOC	RPL23A	ACACA	RPL17	RPL19	SMARCE1	ZNF385C	HEXIM2
PTPRM	PPP4R1	RPL36	DNMT1	RPS16	RPL18	RPL28	RPS5	SNPH	DTD1
CHMP4B	DHX35	RPS21	PPDPF	GRIK1	PIGP	USP51	BEX4	RPL10
GOBPID	Pvalue	OddsRatio		ExpCount		Count	Size	Term
GO:0006415	6.1703430167461e-42	109.593869731801	0.541554217701197
28	91	translational termination
GO:0006414	6.87236085561065e-42	95.6558171745152	0.624870251193689
29	105	translational elongation
GO:0006614	6.87236085561065e-42	95.6558171745152	0.624870251193689
29	105	SRP-dependent cotranslational protein targeting to membrane
GO:0006613	9.42433443774958e-42	94.4069264069264	0.630821396443153
29	106	cotranslational protein targeting to membrane
GO:0045047	9.42433443774958e-42	94.4069264069264	0.630821396443153
29	106	protein targeting to ER
GO:0072599	9.42433443774958e-42	94.4069264069264	0.630821396443153
29	106	establishment of protein localization to endoplasmic reticulum
GO:0070972	3.23440990448545e-40	81.6093041592746	0.702235139436717
29	118	protein localization to endoplasmic reticulum
GO:0006413	1.76944804945208e-38	62.5424473067916	0.904574077918483
30	152	translational initiation
GO:0000184	3.344037340499e-38	75.7241379310345	0.708186284686181
28	119	nuclear-transcribed mRNA catabolic process, nonsense-mediated decay
GO:0006612	1.3267439040668e-36	58.4308149405772	0.910525223167947
29	153	protein targeting to membrane
GO:0072594	4.99225871832265e-36	46.2377299001576	1.2140336308906 31
204	establishment of protein localization to organelle

GO:0019080	5.08201334118564e-35	55.8979534622932	0.898622932669019
28	151	viral genome expression
GO:0019083	5.08201334118564e-35	55.8979534622932	0.898622932669019
28	151	viral transcription
GO:0034623	7.33260248617606e-35	49.8947368421053	1.03549927340668
29	174	cellular macromolecular complex disassembly
GO:0043624	7.56543424743669e-35	54.9958620689655	0.910525223167947
28	153	cellular protein complex disassembly
GO:0032984	1.75977086521413e-34	48.2146198830409	1.065254999654	29
179	macromolecular complex disassembly
GO:0043241	1.99496508092307e-34	52.8620689655172	0.940280949415265
28	158	protein complex disassembly
GO:0000956	1.49951507432058e-33	48.700415749572	1.00574354715937	28
169	nuclear-transcribed mRNA catabolic process
GO:0006402	7.02352240549742e-33	45.7494252873563	1.05930385440454
28	178	mRNA catabolic process
GO:0006401	4.49166690403213e-31	38.6970582505357	1.21998477614006
28	205	RNA catabolic process
GO:0019058	1.44054718261466e-29	33.678953626635	1.37471455262612	28
231	viral infectious cycle
GO:0071845	1.91139572050037e-28	28.1520467836257	1.69012525084769
29	284	cellular component disassembly at cellular level
GO:0022411	2.88393105145004e-28	27.7094086567771	1.71392983184555
29	288	cellular component disassembly
GO:0022415	4.2927540672964e-28	29.4087395957194	1.54729776486056
28	260	viral reproductive process
GO:0033365	8.35406024658428e-26	18.7103384563702	2.81489170299633
32	473	protein localization to organelle
GO:0006605	8.92540399046233e-26	18.6666666666667	2.8208428482458	32
474	protein targeting
GO:0016032	1.9244691752017e-21	15.9116328360642	2.68396650750813
28	451	viral reproduction
GO:0042254	2.84611120448856e-06	10.346608474268	0.886720642170092	8
149	ribosome biogenesis
GO:0042274	3.35689577045844e-06	50.0034843205575	0.107120614490347
4	18	ribosomal small subunit biogenesis
GO:0022613	7.44969396446811e-06	7.65638528138528	1.33900768112933
9	225	ribonucleoprotein complex biogenesis
GO:0071843	1.21044720135613e-05	7.1832298136646	1.42232371462183	9
239	cellular component biogenesis at cellular level
Tissue: Artery_Tibial=>Nerve_Tibial	Type:	asymmetric
SourceGene:	RPL35(ENSG00000136942.9)
TargetGeneSet:	RPL22	MTOR	CROCCP3	PHACTR4	ZMYND12	RNU5E-6P	NASP	MSH4
GTF2B	FAM40A	RP4	NOTCH2NL	GPR89A	NBPF16	CREB3L4	ARHGEF11	LHX4
RP11	SOX13	PLXNA2	NENF	NVL	NTPCR	SLC5A6	THADA	CAMKMT	PNPT1	TIA1
LINC00152	RPL31	RFX8	CCDC115	R3HDM1	KIAA1715	RPL37A	ABCB6
OR6B3	RPL32	UBE2E1	APEH	RBM6	IFRD2	MYH15	WDR52	RNF7	U2SURP
MCCC1	EIF4G1	RPL35A	MYL5	LETM1	SOD3	CLOCK	IL8	CXCL1	RPL34
NDUFC1	MED10	CCDC152	NDUFS4	MCCC2	BTF3	RPS23	CTD	PHF15	CAMLG	HARS
ARHGAP26	TCERG1	TCOF1	LYRM4	PAK1IP1	GABBR1	PPT2	HLA-DMA	VPS52
RPS10	TCP11	C6orf226	RPL39P3	BCKDHB	MICAL1	SERINC1	HINT3	MRPS12
ALDH8A1	AIG1	TOMM7	HNRNPA2B1	CDK13	GS1	TMEM120A	C7orf59
TRIP6	ABCF2	INTS10	C8orf40	RPS20	RPL30	POP1	COX6C	EIF3E	KIFC2
MFSD3	GBA2	FAM120A	ZNF169	IKBKAP	IKBKAP-IT1	C5	RPL35	PRPS1P2
SLC27A4	ODF2	WDR34	C9orf114	NUP188	FAM208B	FBXO18	CDNF	AGAP6
TSPAN14	MMS19	SFXN2	TUBGCP2	ZNF195	RPS13	FAM111A	CPSF7	MTA2	GANAB	FAU
CCL13	RPS6KB2	CHKA	C2CD3	PIWIL4	RNF214	DPAGT1	KLHDC5	PHC1P1	TIMELESS
MARS	POLR3B	ANAPC7	RBM19	ANAPC5	PXMP2	MRPS31P2	POMP	KLF12
COMMD6	RALGAPA1	RPS29	DHRS7	NEK9	POMT2	RPL3P4	TMEM85	SRP14
RSL24D1	SLTM	VPS13C	PIAS1	C16orf79	GSPT1	NPIPL3	MIR4519	C16orf57
CTCF	RANBP10	HAS3	DDX19A	SF3B3	GALNS	AFG3L1P	CTNS	C17orf85
MYBBP1A	ZMYND15	FAM106B	RPL19	PNMT	RPL27	GPATCH8	ZNF652	MTMR4	SLC39A11
RPL38	ASPSCR1	HEXDC	RAB12	RPL36	ZNF558	DNMT1	RAVER1	SMARCA4	ZNF69

SAMD1 MAST3 ARMC6 U2AF1L4 ZNF781 SMG9 TOMM40 QPCTL PPP5C BCAT2
SNRNP70 RPS9 TRAPPC2 LINC00493 TM9SF4 NCOA6 TAF4 RPS21 ATP5J
IFNGR2 TMEM50B PI4KA CCDC116 BCR CABIN1 PIK3IP1 NOL12 RP3 L3MBTL2
TTLL12 PPARA GTPBP6 ZNF81 HDAC6 FAM156A GPR173 MAMLD1 G6PD
GOBPID Pvalue OddsRatio ExpCount Count Size Term
GO:0000184 1.1190553214695e-22 23.1068251273345 1.47401563905612
24 119 nuclear-transcribed mRNA catabolic process, nonsense-mediated decay
GO:0006414 7.07099984389583e-20 22.4493670886076 1.30060203446128
21 105 translational elongation
GO:0006614 7.07099984389583e-20 22.4493670886076 1.30060203446128
21 105 SRP-dependent cotranslational protein targeting to membrane
GO:0006415 7.50866062168435e-20 25.1590043405085 1.12718842986645
20 91 translational termination
GO:0006613 8.72545248415072e-20 22.1836932241251 1.31298872050377
21 106 cotranslational protein targeting to membrane
GO:0045047 8.72545248415072e-20 22.1836932241251 1.31298872050377
21 106 protein targeting to ER
GO:0072599 8.72545248415072e-20 22.1836932241251 1.31298872050377
21 106 establishment of protein localization to endoplasmic reticulum
GO:0006402 1.52369410364955e-19 14.9806892453951 2.20483011556294
25 178 mRNA catabolic process
GO:0006401 3.77488203989601e-19 13.3792675356921 2.53927063871012
26 205 RNA catabolic process
GO:0000956 6.53020082084425e-19 15.0855617352614 2.09334994118054
24 169 nuclear-transcribed mRNA catabolic process
GO:0006413 8.46080250430692e-19 16.164231763069 1.88277627845824 23
152 translational initiation
GO:0070972 9.20132139045525e-19 19.4228761581626 1.46162895301363
21 118 protein localization to endoplasmic reticulum
GO:0043624 1.56413266464346e-17 15.1262702387319 1.89516296450073
22 153 cellular protein complex disassembly
GO:0034623 1.89801598067534e-17 13.7876974019358 2.15528337139298
23 174 cellular macromolecular complex disassembly
GO:0043241 3.17356756115267e-17 14.5650056200824 1.95709639471317
22 158 protein complex disassembly
GO:0032984 3.61245688578415e-17 13.3410585141354 2.21721680160543
23 179 macromolecular complex disassembly
GO:0019080 1.77903389701337e-16 14.4587147030185 1.87038959241575
21 151 viral genome expression
GO:0019083 1.77903389701337e-16 14.4587147030185 1.87038959241575
21 151 viral transcription
GO:0006612 2.34259044725607e-16 14.2376294591484 1.89516296450073
21 153 protein targeting to membrane
GO:0072594 6.78280275089811e-16 11.47800679983 2.52688395266764 23
204 establishment of protein localization to organelle
GO:0019058 1.03212754697973e-13 9.42876299027824 2.86132447581482
22 231 viral infectious cycle
GO:0071845 8.56361057481229e-13 7.91465271637685 3.51781883606671
23 284 cellular component disassembly at cellular level
GO:0022411 1.14741289271562e-12 7.79296081277213 3.56736558023666
23 288 cellular component disassembly
GO:0022415 1.153664293109e-12 8.26280575924637 3.22053837104699
22 260 viral reproductive process
GO:0016032 3.23326928406376e-10 5.27635510029876 5.58639540516227
25 451 viral reproduction
GO:0033365 8.7095212843007e-10 5.00927643784787 5.85890249809702
25 473 protein localization to organelle
GO:0006605 9.09802069589796e-10 4.99775836635525 5.87128918413951
25 474 protein targeting
Tissue: Nerve_Tibial=>Artery_Tibial Type: asymmetric
SourceGene: SNORD14E(ENSG00000200879.1)
TargetGeneSet: RPS8 GAS5 EIF3FP3 IWS1 RPL15 RPL14 RPL24 EPB41L4A-AS1
MIR4454 RPS10 RPL10A RPS20 RPL7A RPS13 RP11 U6 MARS DHRS12

RPL23A ACACA RPL19 RPS10P2 PHEX-AS1
GOBPID Pvalue OddsRatio ExpCount Count Size Term
GO:0006415 4.41889237162837e-22 493.4875 0.0944571309943949 11
91 translational termination
GO:0006414 2.31803923774973e-21 419.579787234043 0.108988997301225
11 105 translational elongation
GO:0006614 2.31803923774973e-21 419.579787234043 0.108988997301225
11 105 SRP-dependent cotranslational protein targeting to membrane
GO:0006613 2.58578316147057e-21 415.134210526316 0.110026987751713
11 106 cotranslational protein targeting to membrane
GO:0045047 2.58578316147057e-21 415.134210526316 0.110026987751713
11 106 protein targeting to ER
GO:0072599 2.58578316147057e-21 415.134210526316 0.110026987751713
11 106 establishment of protein localization to endoplasmic reticulum
GO:0070972 8.87277980524406e-21 368.268691588785 0.122482873157567
11 118 protein localization to endoplasmic reticulum
GO:0000184 9.77398975571312e-21 364.833333333333 0.123520863608055
11 119 nuclear-transcribed mRNA catabolic process, nonsense-mediated decay
GO:0019080 1.47625530308852e-19 280.814285714286 0.156736558023666
11 151 viral genome expression
GO:0019083 1.47625530308852e-19 280.814285714286 0.156736558023666
11 151 viral transcription
GO:0006413 1.59101650924014e-19 278.803191489362 0.157774548474154
11 152 translational initiation
GO:0006612 1.71382512639667e-19 276.820422535211 0.158812538924642
11 153 protein targeting to membrane
GO:0043624 1.71382512639667e-19 276.820422535211 0.158812538924642
11 153 cellular protein complex disassembly
GO:0043241 2.46721559375237e-19 267.311224489796 0.164002491177081
11 158 protein complex disassembly
GO:0000956 5.28191363405507e-19 248.509493670886 0.175420386132448
11 169 nuclear-transcribed mRNA catabolic process
GO:0034623 7.34103891523919e-19 240.802147239264 0.180610338384887
11 174 cellular macromolecular complex disassembly
GO:0006402 9.48644375554893e-19 234.968562874251 0.184762300186838
11 178 mRNA catabolic process
GO:0032984 1.01049864840513e-18 233.553571428571 0.185800290637326
11 179 macromolecular complex disassembly
GO:0072594 4.39820261133927e-18 202.944300518135 0.211750051899523
11 204 establishment of protein localization to organelle
GO:0006401 4.64639077626831e-18 201.884020618557 0.21278804235001
11 205 RNA catabolic process
GO:0019058 1.77093774552353e-17 177.7 0.239775794062695 11 231
viral infectious cycle
GO:0022415 6.63448392011275e-17 156.683734939759 0.269877517126842
11 260 viral reproductive process
GO:0071845 1.77370479174115e-16 142.667582417582 0.294789287938551
11 284 cellular component disassembly at cellular level
GO:0022411 2.07226688558269e-16 140.567689530686 0.298941249740502
11 288 cellular component disassembly
GO:0016032 2.95985130748601e-14 87.475 0.468133693170023 11 451
viral reproduction
GO:0033365 4.99810743614271e-14 83.1785714285714 0.490969483080756
11 473 protein localization to organelle
GO:0006605 5.11551529694795e-14 82.9929805615551 0.492007473531244
11 474 protein targeting
Tissue: Nerve_Tibial=>Artery_Tibial Type: asymmetric
SourceGene: TCERG1(ENSG00000113649.7)
TargetGeneSet: MTND1P23 RBBP4 BTBD19 PRPF38A DDAH1 RP11 RP4
WDR77 CD58 GAPDHP32 SF3B4 RPRD2 KCNJ9 TRAF5 CAPN2 TOMM20
COX20 PXDN DDIT3 LRPPRC EIF3FP3 TIA1 TTL KIF5C FMNL2 ANKRD44
SATB2 CLN5 RPL15 RPL14 KIAA1143 MFSD1 PARL YTHDC1 METTL14 INTU
RXFP1 OXCT1 ITGA1 HNRNPA0 RBM27 ATXN1 RPL10A EFHC1 SNHG5 MICAL1 DDO

RNF217 SCAF8 OSBPL3 EIF4H MEPCE CBLL1 EEF1D POLR1E VPS13A RPL35
RPL12 NUP214 UCK1 ARHGAP12 HECTD2 WDR11 RPS3 SIK2 BCAT1
RPL14P1 CRADD RPL18AP3 BRAP KATNAL1 VPS36 CARS2 OXA1L PRKD1
L2HGDH ATP6V1D STRCP1 GS1 NUDT21 FHOD1 TERF2 ATXN1L COX4I1 METTL16
PELP1 POLR2A ATP1B2 RPL23A RPL19 HEXIM2 ROCK1P1 SF3A2 KHSRP ZNF426
RPL18A FXYD7 ZNF383 RPS16 FBL HNRNPUL1 BCAT2 RPL18 ATF5
RPL28 PSMF1 NSFL1C DHX35 STX16 SF3A1 ARSA SYP USP51 GPRASP2

GOBPID	Pvalue	OddsRatio	ExpCount	Count	Size	Term
GO:0006414	1.56335372159536e-13	24.5800636267232	0.690263649574424	13	105	translational elongation
GO:0006415	6.56816657422186e-13	26.1284123837121	0.598228496297834	12	91	translational termination
GO:0006614	3.77056663558359e-12	22.1733385153517	0.690263649574424	12	105	SRP-dependent cotranslational protein targeting to membrane
GO:0006613	4.22928844262792e-12	21.9359138682389	0.69683758909418	12	106	cotranslational protein targeting to membrane
GO:0045047	4.22928844262792e-12	21.9359138682389	0.69683758909418	12	106	protein targeting to ER
GO:0072599	4.22928844262792e-12	21.9359138682389	0.69683758909418	12	106	establishment of protein localization to endoplasmic reticulum
GO:0070972	1.53784848908999e-11	19.43623550807	0.775724863331257	12	118	protein localization to endoplasmic reticulum
GO:0000184	1.70121226068845e-11	19.2532372480577	0.782298802851014	12	119	nuclear-transcribed mRNA catabolic process, nonsense-mediated decay
GO:0019080	1.75656333887671e-11	16.3338635560269	0.992664867483219	13	151	viral genome expression
GO:0019083	1.75656333887671e-11	16.3338635560269	0.992664867483219	13	151	viral transcription
GO:0006413	1.91066564467994e-11	16.2152131952974	0.999238807002976	13	152	translational initiation
GO:0043624	2.0770172462924e-11	16.0982578397213	1.00581274652273	13	153	cellular protein complex disassembly
GO:0043241	3.12506272799019e-11	15.5376787216148	1.03868244412151	13	158	protein complex disassembly
GO:0034623	1.05506307305378e-10	13.9778063929708	1.14386547643762	13	174	cellular macromolecular complex disassembly
GO:0032984	1.50448375706582e-10	13.5520129297679	1.1767351740364	13	179	macromolecular complex disassembly
GO:0006612	3.29941084523034e-10	14.5757498077416	1.00581274652273	12	153	protein targeting to membrane
GO:0072594	7.62029550948366e-10	11.7574383859022	1.34108366203031	13	204	establishment of protein localization to organelle
GO:0000375	1.02626805736932e-09	11.4534594325535	1.37395335962909	13	209	RNA splicing, via transesterification reactions
GO:0000956	1.04297389651935e-09	13.0755889801243	1.11099577883883	12	169	nuclear-transcribed mRNA catabolic process
GO:0006402	1.89125595617685e-09	12.3588329220496	1.17016123451664	12	178	mRNA catabolic process
GO:0008380	2.75953277936573e-09	8.55194805194805	2.12338246488132	15	323	RNA splicing
GO:0019058	3.47914056166851e-09	10.2816066234057	1.51858002906373	13	231	viral infectious cycle
GO:0006397	6.36362819839496e-09	7.38896784215223	2.62300186838281	16	399	mRNA processing
GO:0000377	8.87693882294628e-09	10.6656626506024	1.34108366203031	12	204	RNA splicing, via transesterification reactions with bulged adenosine as nucleophile
GO:0000398	8.87693882294628e-09	10.6656626506024	1.34108366203031	12	204	nuclear mRNA splicing, via spliceosome
GO:0006401	9.37855334306275e-09	10.6096510393907	1.34765760155007	12	205	RNA catabolic process
GO:0022415	1.44200116716393e-08	9.05584082156611	1.70922427513667	13	260	viral reproductive process

GO:0071845	4.10392679977806e-08	8.23980739807398	1.86699882361082
13	284	cellular component disassembly at cellular level
GO:0022411	4.83723793618626e-08	8.11764966740576	1.89329458168985
13	288	cellular component disassembly
GO:0033365	6.88199245180791e-08	6.1597097194139	3.10947339284479	16
473	protein localization to organelle
GO:0016032	2.30380449189281e-07	5.98623853211009	2.96484672341014
15	451	viral reproduction
GO:0006605	4.34989579975314e-07	5.67687908496732	3.11604733236454
15	474	protein targeting
Tissue: Nerve_Tibial=>Artery_Tibial	Type:	asymmetric
SourceGene:	UBE2E1(ENSG00000170142.7)
TargetGeneSet:	SDHB	ATPIF1	TAF12	ZCCHC17	MACF1	RP1	PPCS	RPS8
UQCRH	RWDD3	RP11	HBXIP	NOTCH2	C1orf54	SCNM1	PSMB4	RPS27	DPM3
PFDN2	NAV1	SNRPE	LYST-IT2	RPS7	UBC	NFU1	SNRNP27	SNRPG
TPRKB	DGUOK	MITD1	RPL31	MZT2B	MZT2A	BTF3L4P2	EEF1B2	MYEOV2
CCDC72	SPCS1	RPL24	NDUFB4	SEC22A	RNF7	PDCD10	DNAJC19	TMEM128	OCIAD1
CLOCK	BDH2	NDUFC1	ANAPC10	LSM6	NDUFS4	NDUFAF2	CHCHD2P2	TAF9
MAP1B	TBCA	COX7C	CTD	MRPL22	RPL26L1	RAB24	TMEM14B	NOL7	C6orf48
RPS10	RPL10A	MEA1	MRPL2	C6orf162	MRPS12	PSMB1	YAE1D1	MPLKIP
CHCHD2	BUD31	C7orf59	PMS2P1	MOSPD3	POP7	TRIP6	ZNHIT1	FIS1	NAA38	ARF5
SSBP1	DCTN6	C8orf40	FNTA	RPS20	SNHG6	TCEB1	PAF1	TTC35	MRPL13
TATDN1	C9orf133	NDUFB6	VPS13A	SEC61B	ATP6V1G1	OR1N1	RPL35
RPL12	SWI5	NUP188	VPS26A	GSTO1	MRPL23	RPL27A	DENND5A	RPS13	IMMP1L
C11orf31	C11orf10	GANAB	POLR2G	COX8A	GAL	CWC15	AASDHPPT
UBE4A	ATP5L	COX14	RPL41	RPL18AP3	ISCU	GPN3	C12orf47
MED13L	DIABLO	ARL6IP4	POMP	MED4	RPS29	CGRRF1	ERH	COX16	SIPA1L1
NDUFB1	DYNC1H1	C14orf2	SRP14	RSL24D1	GTF2A2	RPS3AP6	VPS13C	RPLP1	BBS4
RPS17L	IQGAP1	RPS15A	TERF2IP	RPL13	RPAIN	KIAA0100	ACACA	RPL17
RPL19	RPS7P11	BPTF	TRIM65	ANAPC11	C18orf32	UQCR11	RPL36	TRAPPC5
NDUFA7	PIN1	NDUFA13	ALKBH6	EID2	SEPW1	RPL18	LIN7B	RPS9	TRAPPC2	A1BG
RPS5	LINC00493	ROMO1	UQCR10	RPS19BP1	SLC25A17	USP9X	UXT
TIMM17B	HUWE1	TCEAL8	NGFRAP1	PSMD10	NDUFA1	SSR4
GOBPID	Pvalue	OddsRatio	ExpCount	Count	Size	Term
GO:0006614	1.93501935783393e-35	46.6360655737705	1.10442183931908
30	105	SRP-dependent cotranslational protein targeting to membrane
GO:0006613	2.67665026944318e-35	46.0191975841242	1.11494014255069
30	106	cotranslational protein targeting to membrane
GO:0045047	2.67665026944318e-35	46.0191975841242	1.11494014255069
30	106	protein targeting to ER
GO:0072599	2.67665026944318e-35	46.0191975841242	1.11494014255069
30	106	establishment of protein localization to endoplasmic reticulum
GO:0070972	1.0070255646683e-33	39.7103204172876	1.24115978133001
30	118	protein localization to endoplasmic reticulum
GO:0006414	3.82515260888377e-32	41.7067448680352	1.10442183931908
28	105	translational elongation
GO:0006415	9.22894034660175e-31	45.1873015873016	0.957165594076534
26	91	translational termination
GO:0072594	3.98518260107929e-30	22.9113961374023	2.14573385924849
33	204	establishment of protein localization to organelle
GO:0006612	4.93071587865502e-30	28.3406637345062	1.60930039443637
30	153	protein targeting to membrane
GO:0000184	2.13947470155618e-27	31.5203959720089	1.25167808456162
26	119	nuclear-transcribed mRNA catabolic process, nonsense-mediated decay
GO:0019080	2.48046465937381e-27	26.0246525045896	1.58826378797315
28	151	viral genome expression
GO:0019083	2.48046465937381e-27	26.0246525045896	1.58826378797315
28	151	viral transcription
GO:0043624	9.2406972904508e-26	24.2965714285714	1.60930039443637
27	153	cellular protein complex disassembly
GO:0043241	2.29121967989197e-25	23.3609770992366	1.66189191059442
27	158	protein complex disassembly
GO:0000956	1.51151156239037e-24	21.5345915492958	1.77759324614214

27 169 nuclear-transcribed mRNA catabolic process
GO:0006413 1.85405285265539e-24 23.2110103300579 1.59878209120476
26 152 translational initiation
GO:0033365 2.50837908810917e-24 11.0259573426859 4.97515742855166
39 473 protein localization to organelle
GO:0034623 3.40309038164766e-24 20.7947755102041 1.83018476230019
27 174 cellular macromolecular complex disassembly
GO:0006402 6.39148121649979e-24 20.2381986754967 1.87225797522663
27 178 mRNA catabolic process
GO:0032984 7.46337920381415e-24 20.1036315789474 1.88277627845824
27 179 macromolecular complex disassembly
GO:0019058 3.01553722160842e-23 16.4538758753924 2.42972804650197
29 231 viral infectious cycle
GO:0006401 3.06751891538302e-22 17.135595505618 2.15625216248011 27
205 RNA catabolic process
GO:0006605 3.11520177880595e-22 10.2058302656452 4.98567573178327
37 474 protein targeting
GO:0016032 5.90534725579859e-22 10.3827170751973 4.74375475745623
36 451 viral reproduction
GO:0022415 9.02941465379931e-22 14.3586386513216 2.73475884021867
29 260 viral reproductive process
GO:0071845 1.10110053202151e-20 12.9850470269409 2.98719811777732
29 284 cellular component disassembly at cellular level
GO:0022411 1.63163906717007e-20 12.7808644881816 3.02927133070376
29 288 cellular component disassembly
GO:0022904 6.67156536268756e-14 19.2187077588537 1.00975711023459
15 96 respiratory electron transport chain
GO:0022900 9.91866284412494e-12 13.0466785254248 1.40945263303578
15 134 electron transport chain
GO:0045333 2.31037940751085e-11 12.2179435599747 1.49359905888866
15 142 cellular respiration
GO:0042773 1.20364804133444e-09 23.6196172248804 0.494360251885683
9 47 ATP synthesis coupled electron transport
GO:0042775 1.20364804133444e-09 23.6196172248804 0.494360251885683
9 47 mitochondrial ATP synthesis coupled electron transport
GO:0015980 3.72440504716403e-09 6.51190414708465 3.22911909210435
18 307 energy derivation by oxidation of organic compounds
GO:0006119 8.52537130747957e-09 18.3031254459826 0.610061587433396
9 58 oxidative phosphorylation
GO:0006120 8.01587204193473e-08 23.7550535077289 0.37865891633797
7 36 mitochondrial electron transport, NADH to ubiquinone
GO:0006091 1.1846145623813e-07 4.851554313657 4.50183378312919 19
428 generation of precursor metabolites and energy
Tissue: Artery_Tibial=>Nerve_Tibial Type: asymmetric
SourceGene: YAE1D1(ENSG00000241127.3)
TargetGeneSet: MRPL20 MINOS1 CDC42 ZCCHC17 RP11 PSMB2 PPCS CCDC30
MAGOH SLC35D1 COL24A1 ACTG1P4 BCAS2 U1 FAM72C MRPS21 RP1 ALDH9A1
TMCO1 XPR1 SNRPE TARBP1 YWHAQ SLC4A1AP MORN2 PPM1B CCDC115 AGPS
UBE2E3 C2orf47 NDUFB3 MRPL44 SEPT2 CNTN4-AS1 TPRXL UBE2E1 CCDC72 RBM6
SPCS1 PSMC1P1 RNF7 MRPL47 NDUFB5 MYL5 EVC2 UTP3 MRPS18C TMSB4XP8
NDUFC1 NDUFS6 RBBP4P1 ZNF622 SUB1 TAF9 CTD PRRC1 UQCRQ TCOF1 CTB
FGFR4 TMEM14B NHLRC1 HIST1H1E PPP1R10 HNRNPC COX6A1P2 FRS3
KIAA0240 C6orf226 MAD2L1BP RPL39P3 COX7A2 FAM54A PSMB1
FTSJ2 NUDT1 NDUFA4 TOMM7 MALSU1 POM121C GNG11 BUD31 PMS2P1 PSMC2
MRPS33 LSM1 KAT6A MRPL15 TCEB1 C8orf59 COX6C POLR2K C9orf123
C9orf133 CTSL1 SEC61B MSANTD3 CTNNAL1 TXN DFNB31 FBXW2 ODF2
C9orf104 FBXO18 ATP5C1 CDC123 SAR1A POLR2L ARNTL IMMP1L FNBP4
MRPL16 FEN1 C11orf30 SNORA32 ATP5L PANX3 PHC1P1 MYL6 MED13L PXN
RPL31P52 DIABLO RPS7P10 POMP COMMD6 RPS29 PSMA3 ERH NDUFB1
C14orf2 HMGN2P5 TMEM85 SRP14 GTF2A2 ANXA2 COX5A FAM100A NDUFAB1 HMGN2P3
KIF22 TBX6 MIR4519 RANBP10 MLLT6 MSI2 ATP5H MRPS7 H3F3B USP36
MYL12B IER3IP1 DYM C19orf70 PIN1 ZNF653 C19orf44 UQCRFS1
COX6B1 POLR2I TBC1D17 NDUFA3 MCM8 SNRPB2 ROMO1 RP3 PFDN4 ATP5J PIGP

BCR BCRP8 SNRPD3 UQCR10 MORC2 LGALS1 NDUFA6 EEF1B2P3 FATE1
HMGN1P26
GOBPID Pvalue OddsRatio ExpCount Count Size Term
GO:0022904 1.08446173673158e-20 31.6644436644437 0.863608054805896
19 96 respiratory electron transport chain
GO:0022900 3.66206446297357e-19 22.658692185008 1.20545290983323 20
134 electron transport chain
GO:0045333 1.20307042226636e-18 21.1609538002981 1.27742024773372
20 142 cellular respiration
GO:0015980 4.10583548678421e-14 10.032228719948 2.76174659193135 22
307 energy derivation by oxidation of organic compounds
GO:0006119 3.23436620881736e-12 28.0733059181119 0.521763199778562
11 58 oxidative phosphorylation
GO:0006091 4.297180398149e-12 7.38590054228684 3.85025257767629
23 428 generation of precursor metabolites and energy
GO:0042773 9.54978224417832e-12 32.1711711711712 0.422808110165386
10 47 ATP synthesis coupled electron transport
GO:0042775 9.54978224417832e-12 32.1711711711712 0.422808110165386
10 47 mitochondrial ATP synthesis coupled electron transport
GO:0006120 2.23648849422177e-11 39.3774104683196 0.323853020552211
9 36 mitochondrial electron transport, NADH to ubiquinone
GO:0000375 2.87742340673222e-06 6.59337068160598 1.88014670265034
11 209 RNA splicing, via transesterification reactions
GO:0042776 4.19856001155518e-06 50.4832451499118 0.116946924088298
4 13 mitochondrial ATP synthesis coupled proton transport
GO:0015985 1.73520865905422e-05 32.4421768707483 0.161926510276105
4 18 energy coupled proton transport, down electrochemical gradient
GO:0015986 1.73520865905422e-05 32.4421768707483 0.161926510276105
4 18 ATP synthesis coupled proton transport
Tissue: Artery_Tibial=>Nerve_Tibial Type: asymmetric
SourceGene: YEATS4(ENSG00000127337.2)
TargetGeneSet: RP11 RP13 SLC45A1 TMEM201 FAM131C CROCCP3 RPL11 MAP7D1
MFSD2A RP3 SNORD46 TMEM69 CSRP3 CELSR2 GDAP2 CCT3 ATP1A2 RCSD1
KDM5B-AS1 TMEM81 ST13P19 ZNF692 LPIN1 SRSF7 EIF3FP3 NFU1 CAPG
RPL31 RFX8 SLC6A6 PLCL2 RPL14 QARS PHF7 SHQ1 P2RY13 EIF4G1
RPL35A MYL5 RPL7AP31 PAICS RPL34 INTU NPY1R EXOC3 MTRR CCT5
BTF3 F2RL1 FBXL17 CAMLG CD74 RPS14 GAPDHP71 BTN2A3P RPS10
C6orf226 SLC18B1 MRPS12 PERP RPL23P8 GS1 TMEM120A MKRN1
ABCF2 C8orf40 RPS20 RPL30 POP1 COX6C CTHRC1 SCRIB GBA2 ZNF169
IKBKAP GGTA1P PRPS1P2 C9orf114 ABCA2 PARD3 ZNF37A TSPAN14 RPL13AP5
SFRP5 PLEKHA1 ZNF195 EIF3F RPS13 MTA2 DNAJC4 FAU CDK2AP2 UNC93B1
CEP57 RPS25 HYOU1 KCNJ5 FKBP4 RP4 KLRF1 FMNL3 ATP5G2 MARS
RPL14P1 KRT8P22 YEATS4 SLC25A3 GLT8D2 ISCU PUS1 RPS7P10 WBP4 OXA1L
NUBPL RPS6KL1 NEK9 TTLL5 GLRX5 MEG8 BTBD6 IGHV3-7 OIP5-AS1
PLA2G4B GRAMD2 HEXA C16orf79 ERVK13-1 HCFC1R1 CLEC16A CTD
PYCARD C16orf46 ZNF469 RP1 CTNS MYBBP1A ALDOC GPR179 RPL17
RPL19 KRT223P GHDC RPL27 HILS1 RPL38 FBF1 ZFP161 RAB12 DSC2
ZCCHC2 RPL36 SMARCA4 UBA52 ZNF708 RPS16 ZNF541 BCAT2 ZNF808 RPS9
ZNF470 NOP56 ELMO2 RPS21 RTN4R ZNF280B RANGAP1 CRELD2 STS ZNF81
RPL7P57 DIAPH2 CXorf1
GOBPID Pvalue OddsRatio ExpCount Count Size Term
GO:0006415 3.1428413168975e-24 38.8636363636364 0.824925610684382
21 91 translational termination
GO:0000184 4.60956574832672e-23 29.5948169866641 1.07874887551035
22 119 nuclear-transcribed mRNA catabolic process, nonsense-mediated decay
GO:0006414 8.19925129889536e-23 32.3545454545455 0.951837243097364
21 105 translational elongation
GO:0006614 8.19925129889536e-23 32.3545454545455 0.951837243097364
21 105 SRP-dependent cotranslational protein targeting to membrane
GO:0006613 1.01502290635405e-22 31.9716577540107 0.960902359698291
21 106 cotranslational protein targeting to membrane
GO:0045047 1.01502290635405e-22 31.9716577540107 0.960902359698291
21 106 protein targeting to ER

GO:0072599	1.01502290635405e-22	31.9716577540107	0.960902359698291
21	106	establishment of protein localization to endoplasmic reticulum
GO:0006413	5.48981075299118e-22	23.4275768016078	1.37789772334095
23	152	translational initiation
GO:0034623	6.32326500172459e-22	21.188785046729 1.57733028856135	24
174	cellular macromolecular complex disassembly
GO:0043624	6.41441462649512e-22	23.2457264957265	1.38696283994187
23	153	cellular protein complex disassembly
GO:0070972	1.11243590681068e-21	27.9927835051546	1.06968375890942
21	118	protein localization to endoplasmic reticulum
GO:0032984	1.26394354349116e-21	20.49804039795	1.62265587156598	24
179	macromolecular complex disassembly
GO:0043241	1.37344324543283e-21	22.3768861454047	1.43228842294651
23	158	protein complex disassembly
GO:0019080	1.09227039286321e-20	22.2033994737216	1.36883260674002
22	151	viral genome expression
GO:0019083	1.09227039286321e-20	22.2033994737216	1.36883260674002
22	151	viral transcription
GO:0006612	1.46997069689745e-20	21.8613348273689	1.38696283994187
22	153	protein targeting to membrane
GO:0000956	1.3648602001262e-19	19.4599013917494	1.53200470555671
22	169	nuclear-transcribed mRNA catabolic process
GO:0006402	4.31521714479814e-19	18.3255704540108	1.61359075496505
22	178	mRNA catabolic process
GO:0006401	5.82224420843747e-19	16.5432437932438	1.85834890319009
23	205	RNA catabolic process
GO:0071845	7.0820758368039e-17	12.1294033069734	2.57449311466334
24	284	cellular component disassembly at cellular level
GO:0022411	9.76231353416179e-17	11.9422259983008	2.61075358106705
24	288	cellular component disassembly
GO:0019058	1.24754327370916e-16	13.6272332206663	2.0940419348142 22
231	viral infectious cycle
GO:0072594	1.32874576152819e-16	14.7479880774963	1.84928378658916
21	204	establishment of protein localization to organelle
GO:0022415	1.5313899745717e-15	11.9421787063449	2.35693031624109
22	260	viral reproductive process
GO:0006605	8.02267033340791e-13	7.28610749254108	4.29686526883953
25	474	protein targeting
GO:0033365	3.84720495659742e-11	6.56399176954733	4.2878001522386 23
473	protein localization to organelle
GO:0016032	1.01508531448093e-10	6.535403434486	4.0883675870182 22	451
viral reproduction
GO:0006364	4.56921504894388e-06	9.63685636856369	0.942772126496436
8	104	rRNA processing
GO:0016072	6.94523902936424e-06	9.06615654391838	0.997162826102	8
110	rRNA metabolic process
GO:0042254	8.39393584807817e-06	7.47189695550351	1.35070237353816
9	149	ribosome biogenesis
GO:0034660	1.08073032336964e-05	5.18866007941638	2.58355823126427
12	285	ncRNA metabolic process
GO:0071843	1.11563267010338e-05	5.6656432748538 2.16656286762162	11
239	cellular component biogenesis at cellular level
GO:0034470	1.38935912753098e-05	6.14615050021749	1.81302332018545
10	200	ncRNA processing
GO:0042274	1.7884689744967e-05	32.1844769403825	0.163172098816691
4	18	ribosomal small subunit biogenesis
Tissue: Skin_Sun_Exposed_Lower_leg=>Artery_Tibial	Type:	cluster
SourceGene:	ACTG1P3(ENSG00000215388.3)
TargetGeneSet:	RP11	NBPF5	SNAPIN	FLAD1	LAMTOR2 MRPL24	TIMM17A PYCR2
TFB2M	OST4	TRIM54	GEMIN6	COMMD1	SNRPG	TANC1	ATP5G3	IQSEC1	UMPS
ETV5-AS1		NDUFC1	NDUFS6	SKP2	EGFLAM	POC5	CETN3	ASS1P10 EEF1E1
TBC1D7	MED20	COX7A2	YAE1D1	MTERF	PMS2P1	POP7	NDUFB2	TMEM74	PCBD1
MRPS16	SRP9P1	PTPRE	POLR2L	HARBI1	COX8A	EI24	SMUG1	MMAB	POP5	DENR

MRPL52 ARG2 COX16 SLIRP C14orf2 MRPS11 POLR3K C16orf91 NDUFAB1
PSMB6 TXNDC17 ATP5G1 PHB C17orf89 UQCR11 NDUFA7 PIN1 LSM4
DDX49 NDUFA13 COX6B1 GEMIN7 AP2S1 PLCB1 RPS27AP2 ZNFX1 ATP5E
TUBA8 UQCR10 HDHD1 NDUFA1
GOBPID Pvalue OddsRatio ExpCount Count Size Term
GO:0022904 3.24556440118388e-16 42.2655148897477 0.43844716628607
13 96 respiratory electron transport chain
GO:0022900 2.79075475944671e-14 28.9150163729924 0.61199916960764
13 134 electron transport chain
GO:0045333 5.97504778100445e-14 27.106625713032 0.648536433464812 13
142 cellular respiration
GO:0015980 1.03829670147942e-09 11.756064690027 1.402117500519 13 307
energy derivation by oxidation of organic compounds
GO:0042773 1.62773256372155e-09 42.5487288135593 0.214656425160889
7 47 ATP synthesis coupled electron transport
GO:0042775 1.62773256372155e-09 42.5487288135593 0.214656425160889
7 47 mitochondrial ATP synthesis coupled electron transport
GO:0006119 7.48133722625455e-09 33.345962113659 0.264895162964501 7
58 oxidative phosphorylation
GO:0006120 1.25847848280961e-08 47.85 0.164417687357276 6 36
mitochondrial electron transport, NADH to ubiquinone
GO:0006091 5.5191971543385e-08 8.25687656285519 1.95474361635873
13 428 generation of precursor metabolites and energy
GO:0000387 1.13792270258561e-05 34.3082437275986 0.141581897446543
4 31 spliceosomal snRNP assembly
Tissue: Skin_Sun_Exposed_Lower_leg=>Artery_Tibial Type: cluster
SourceGene: CNOT6(ENSG00000113300.7)
TargetGeneSet: AURKAIP1 XKR8 GPBP1L1 MAGOH RWDD3 RP11 PYCR2
MRPL33 SNRPG MYEOV2 GTPBP8 RNF7 PDCD6 SKP2 FST ATOX1 HNRNPC
KLHDC3 COX7A2 GPR126 RP1 TMEM242 SEC61G SKP1P1 BRI3 POP7 ADCK2
C7orf29 BAG4 MRPS35 ZBTB34 CACFD1 C9orf69 CHCHD1 BUB3 C11orf10
COX8A PPP1R14B PHLDB1 MRPL51 FAM86FP COX14 ESRRAP1 MRPL52 C14orf167
COX16 GTF2A2 CORO2B COX5A POLR3K NDUFAB1 CNEP1R1 TMEM208 C17orf59
SNX11 MRPL27 ATP5H UQCR11 SIRT6 CLPP C19orf79 PIN1 LSM4
NDUFA13 COX6B1 CTU1 ATP5E UQCR10 RPS19BP1
GOBPID Pvalue OddsRatio ExpCount Count Size Term
GO:0022904 2.83657356242712e-11 37.0215517241379 0.325513805272992
9 96 respiratory electron transport chain
GO:0022900 5.80245845438919e-10 25.6986 0.454363019860217 9 134
electron transport chain
GO:0045333 9.73660960242165e-10 24.1392857142857 0.481489170299633
9 142 cellular respiration
GO:0015980 7.63431626646694e-07 10.6489932885906 1.04096602311259
9 307 energy derivation by oxidation of organic compounds
GO:0006091 1.15696279473892e-05 7.50877088305489 1.45124904850875
9 428 generation of precursor metabolites and energy
Tissue: Artery_Tibial=>Skin_Sun_Exposed_Lower_leg Type: asymmetric
SourceGene: CTD-2547L16.3(ENSG00000253384.1)
TargetGeneSet: C1QC C1QB LAPTM5 BMP8A CD53 FCER1G PTPRC CR1
HS1BP3-IT1 BRE U6 CHST13 CP SPOCK1 DOCK2 CTB SDCBP
HRCT1 SVEP1 SUSD1 APBB1IP HPS1 SERPING1 MS4A7 SLC15A3 ALDH3B1 NNMT
C1S C3AR1 LRP1 SELPLG CHST14 TEPP IRF8 CYBA CD68 NSF
ANKFN1 C19orf59 LRRC25 TYROBP ZSCAN18 CTSA ATP6AP2 FAM120C GLA
SASH3 TMLHE
GOBPID Pvalue OddsRatio ExpCount Count Size Term
GO:0002460 7.41189648208857e-10 25.6954691259022 0.475953221230365
9 181 adaptive immune response based on somatic recombination of immune
receptors built from immunoglobulin superfamily domains
GO:0002250 1.64056603770991e-09 23.3563218390805 0.520656009964708
9 198 adaptive immune response
GO:0002449 9.79027258973292e-09 23.9060796645702 0.439139159919729
8 167 lymphocyte mediated immunity
GO:0002252 3.99113913874788e-08 13.3330167173252 1.01501626185039

10	386	immune effector process
GO:0002443	6.07249161939955e-08	18.6666666666667	0.554840495467442
8	211	leukocyte mediated immunity
GO:0006958	1.85988125384329e-07	48.3771043771044	0.131478790395128
5	50	complement activation, classical pathway
GO:0016064	4.05110284594765e-07	25.55	0.291882914677185	6	111
immunoglobulin mediated immune response
GO:0019724	4.74438212221084e-07	24.8350694444444	0.299771642100893
6	114	B cell mediated immunity
GO:0006956	6.0190430144779e-07	37.5	0.165663275897862	5	63
complement activation
GO:0002455	7.04631537401598e-07	36.2449494949495	0.170922427513667
5	65	humoral immune response mediated by circulating immunoglobulin
GO:0072376	2.25343803708071e-06	28.2093663911846	0.215625216248011
5	82	protein activation cascade
GO:0050778	3.78880573433601e-06	10.5295880149813	0.957165594076534
8	364	positive regulation of immune response
GO:0002253	1.10142986865853e-05	10.9199072028281	0.786243166562868
7	299	activation of immune response
GO:0002697	1.28020566668088e-05	13.6711538461538	0.528544737388416
6	201	regulation of immune effector process
GO:0006959	1.58627604246756e-05	18.5133385133385	0.320808248564113
5	122	humoral immune response
Tissue: Artery_Tibial=>Skin_Sun_Exposed_Lower_leg	Type:	asymmetric
SourceGene:	ENSG00000225059.1
TargetGeneSet:	DENND2D FLG2	LCE1F	LCE1E	LCE1C	LCE1B	LCE1A	HMCN1	GDF7
ADAMTS9-AS1	BCL6	BTC	ANKRD32 GLIS3	CCDC77	PLEKHG6 NFE2	LRCH1	RP11
G2E3	CES4A	VN2R10P GSDMA	CALR	SYS1-DBNDD2	MIS18A	ETS2	GRPR
GOBPID	Pvalue	OddsRatio	ExpCount	Count	Size	Term
GO:0031424	1.82922189146044e-09	138.947876447876	0.0552210919659539
5	42	keratinization
GO:0030216	1.13167210273534e-07	57.5561797752809	0.123590062971421
5	94	keratinocyte differentiation
GO:0009913	2.49078956408045e-07	48.7312925170068	0.144626669434641
5	110	epidermal cell differentiation
GO:0030855	1.14741895604588e-06	23.6722408026756	0.370770188914262
6	282	epithelial cell differentiation
GO:0008544	1.62241952436155e-05	20.0963718820862	0.33790049131548
5	257	epidermis development
Tissue: Skin_Sun_Exposed_Lower_leg=>Artery_Tibial	Type:	asymmetric
SourceGene:	IGFBP5(ENSG00000115461.4)
TargetGeneSet:	FOXO6	RPL5	RPS7	COX7A2L NCAPH	RPL15	MAP1B	RP11	NPM1
RPL15P3 MICA	RPL10A	SPDYE1	SEMA3C	EIF3E	RPL7A	VTI1A	RPL27A	FAT3
PLBD2	RSL24D1 SEC11A	NLRP12
GOBPID	Pvalue	OddsRatio	ExpCount	Count	Size	Term
GO:0006614	9.33838843882724e-11	73.1224489795918	0.152584596221715
7	105	SRP-dependent cotranslational protein targeting to membrane
GO:0006613	9.99015863106583e-11	72.3787878787879	0.154037782852398
7	106	cotranslational protein targeting to membrane
GO:0045047	9.99015863106583e-11	72.3787878787879	0.154037782852398
7	106	protein targeting to ER
GO:0072599	9.99015863106583e-11	72.3787878787879	0.154037782852398
7	106	establishment of protein localization to endoplasmic reticulum
GO:0070972	2.13927239416294e-10	64.5	0.171476022420594	7	118
protein localization to endoplasmic reticulum
GO:0000184	2.27103890190469e-10	63.9196428571429	0.172929209051277
7	119	nuclear-transcribed mRNA catabolic process, nonsense-mediated decay
GO:0006413	1.27428244380048e-09	49.2586206896552	0.220884367863816
7	152	translational initiation
GO:0006612	1.33423773257923e-09	48.9178082191781	0.222337554494499
7	153	protein targeting to membrane
GO:0043624	1.33423773257923e-09	48.9178082191781	0.222337554494499
7	153	cellular protein complex disassembly

GO:0043241 1.67139039472262e-09 47.2814569536424 0.229603487647914
7 158 protein complex disassembly
GO:0006415 2.65350413836654e-09 67.5058823529412 0.132239983392153
6 91 translational termination
GO:0000956 2.67584712446114e-09 44.037037037037 0.245588540585427 7
169 nuclear-transcribed mRNA catabolic process
GO:0034623 3.27969306330938e-09 42.7035928143713 0.252854473738842
7 174 cellular macromolecular complex disassembly
GO:0006402 3.84282202852439e-09 41.6929824561403 0.258667220261574
7 178 mRNA catabolic process
GO:0032984 3.99579626860457e-09 41.4476744186046 0.260120406892257
7 179 macromolecular complex disassembly
GO:0006414 6.32691400295807e-09 57.9030303030303 0.152584596221715
6 105 translational elongation
GO:0072594 9.91149384902272e-09 36.1243654822335 0.296450072659332
7 204 establishment of protein localization to organelle
GO:0006401 1.02531144534909e-08 35.9393939393939 0.297903259290015
7 205 RNA catabolic process
GO:0019080 5.61716063210416e-08 39.4068965517241 0.219431181233133
6 151 viral genome expression
GO:0019083 5.61716063210416e-08 39.4068965517241 0.219431181233133
6 151 viral transcription
GO:0071845 9.65983470197068e-08 25.5469314079422 0.412705003113971
7 284 cellular component disassembly at cellular level
GO:0022411 1.06279105736804e-07 25.1761565836299 0.418517749636703
7 288 cellular component disassembly
GO:0033365 1.73630693240662e-07 18.4813895781638 0.687357276313058
8 473 protein localization to organelle
GO:0006605 1.76466691210442e-07 18.4404093760317 0.688810462943741
8 474 protein targeting
GO:0022613 5.9483776486576e-07 25.9561643835616 0.326966991903675
6 225 ribonucleoprotein complex biogenesis
GO:0019058 6.9406123036348e-07 25.2533333333333 0.335686111687772
6 231 viral infectious cycle
GO:0071843 8.47127712974072e-07 24.3725321888412 0.347311604733236
6 239 cellular component biogenesis at cellular level
GO:0022415 1.38494421022697e-06 22.3244094488189 0.377828523977579
6 260 viral reproductive process
GO:0042254 1.94069486231348e-06 31.0026041666667 0.216524807971767
5 149 ribosome biogenesis
Tissue: Artery_Tibial=>Skin_Sun_Exposed_Lower_leg Type: asymmetric
SourceGene: MIR590(ENSG00000207741.1)
TargetGeneSet: MXRA8 DNAJC16 EYA3 CCDC23 RP4 CTSS UFC1 DNM3OS
GPR37L1 RAB4A IAH1 SPDYA UBC SP5 NIF3L1 EEF1B2 RPL15 CP
TTC14 PPP1R2 RP11 FAM190A RPS3A SPCS3 BDP1 ADAMTS19 F13A1
KLHDC3 EEF1A1 UBE2J1 BRD7P3 HEBP2 PSMB1 EEF1A1P6 SBDS NCF1B
CCL26 RPS3AP26 LY96 CHMP4C DAPK1 RPS3AP5 SLC35G1 TSSC2 EIF3F
RPS13 HPS5 LMO2 RP1 FTH1 RBM14 JRKL GAPDH DAZAP2 PFDN5
APPL2 RPL6 TPT1 CCNB1IP1 TRDC EXD2 PAPLN TRPM7 PKM2
NTAN1 RPS15A GOT2 CHTF8 CTD RNF167 CD68 RPL26 COX10 ZNHIT3
RPL17 FTL RP3
GOBPID Pvalue OddsRatio ExpCount Count Size Term
GO:0006414 1.47673546624249e-11 30.0968421052632 0.435955989204899
10 105 translational elongation
GO:0006614 4.32985424798658e-10 26.2775735294118 0.435955989204899
9 105 SRP-dependent cotranslational protein targeting to membrane
GO:0006613 4.71651216055801e-10 26.0048514251061 0.440107951006851
9 106 cotranslational protein targeting to membrane
GO:0045047 4.71651216055801e-10 26.0048514251061 0.440107951006851
9 106 protein targeting to ER
GO:0072599 4.71651216055801e-10 26.0048514251061 0.440107951006851
9 106 establishment of protein localization to endoplasmic reticulum
GO:0070972 1.23586942312019e-09 23.1225040474906 0.489931492630268

9	118	protein localization to endoplasmic reticulum
GO:0006415	3.53907690704808e-09	26.5208526413346	0.377828523977579
8	91	translational termination
GO:0006413	1.16255483641681e-08	17.5828877005348	0.631098193896616
9	152	translational initiation
GO:0006612	1.23127224219466e-08	17.4595588235294	0.635250155698568
9	153	protein targeting to membrane
GO:0043624	1.23127224219466e-08	17.4595588235294	0.635250155698568
9	153	cellular protein complex disassembly
GO:0043241	1.63102119620009e-08	16.8677457560205	0.656009964708325
9	158	protein complex disassembly
GO:0000184	2.98491410807608e-08	19.7920997920998	0.494083454432219
8	119	nuclear-transcribed mRNA catabolic process, nonsense-mediated decay
GO:0034623	3.7730360310148e-08	15.2149732620321	0.722441353539547
9	174	cellular macromolecular complex disassembly
GO:0032984	4.8202817690404e-08	14.7622837370242	0.743201162549305
9	179	macromolecular complex disassembly
GO:0072594	1.48031863913011e-07	12.8470588235294	0.84700020759809
9	204	establishment of protein localization to organelle
GO:0019080	1.90639954577891e-07	15.3286713286713	0.626946232094665
8	151	viral genome expression
GO:0019083	1.90639954577891e-07	15.3286713286713	0.626946232094665
8	151	viral transcription
GO:0000956	4.52078350387159e-07	13.5977066411849	0.70168154452979
8	169	nuclear-transcribed mRNA catabolic process
GO:0006402	6.70898009617185e-07	12.8696832579186	0.739049200747353
8	178	mRNA catabolic process
GO:0006401	1.9446719025694e-06	11.0847325263569	0.851152169400042
8	205	RNA catabolic process
GO:0071845	2.36772714650336e-06	9.05839572192513	1.1791571517542 9
284	cellular component disassembly at cellular level
GO:0022411	2.65586737324971e-06	8.92599620493359	1.19576499896201
9	288	cellular component disassembly
GO:0019058	4.72285749714866e-06	9.77440496723008	0.959103176250778
8	231	viral infectious cycle
GO:0022415	1.12341186163975e-05	8.63186813186813	1.07951006850737
8	260	viral reproductive process
Tissue: Artery_Tibial=>Skin_Sun_Exposed_Lower_leg	Type:	cluster
SourceGene:	MIR590(ENSG00000207741.1)
TargetGeneSet:	MXRA8	DNAJC16	EYA3	CCDC23	RP4	CTSS	UFC1	DNM3OS
GPR37L1	RAB4A	IAH1	SPDYA	UBC	SP5	NIF3L1	EEF1B2	RPL15	CP
TTC14	PPP1R2	RP11	FAM190A	RPS3A	SPCS3	BDP1	ADAMTS19		F13A1
KLHDC3	EEF1A1	UBE2J1	BRD7P3	HEBP2	PSMB1	EEF1A1P6		SBDS	NCF1B
CCL26	RPS3AP26		LY96	CHMP4C	DAPK1	RPS3AP5	SLC35G1	TSSC2	EIF3F
RPS13	HPS5	LMO2	RP1	FTH1	RBM14	JRKL	GAPDH	DAZAP2	PFDN5
APPL2	RPL6	TPT1	CCNB1IP1		TRDC	EXD2	PAPLN	TRPM7	PKM2
NTAN1	RPS15A	GOT2	CHTF8	CTD	RNF167	CD68	RPL26	COX10	ZNHIT3
RPL17	FTL	RP3
GOBPID	Pvalue	OddsRatio	ExpCount	Count	Size	Term
GO:0006414	1.47673546624249e-11	30.0968421052632	0.435955989204899
10	105	translational elongation
GO:0006614	4.32985424798658e-10	26.2775735294118	0.435955989204899
9	105	SRP-dependent cotranslational protein targeting to membrane
GO:0006613	4.71651216055801e-10	26.0048514251061	0.440107951006851
9	106	cotranslational protein targeting to membrane
GO:0045047	4.71651216055801e-10	26.0048514251061	0.440107951006851
9	106	protein targeting to ER
GO:0072599	4.71651216055801e-10	26.0048514251061	0.440107951006851
9	106	establishment of protein localization to endoplasmic reticulum
GO:0070972	1.23586942312019e-09	23.1225040474906	0.489931492630268
9	118	protein localization to endoplasmic reticulum
GO:0006415	3.53907690704808e-09	26.5208526413346	0.377828523977579
8	91	translational termination

GO:0006413	1.16255483641681e-08	17.5828877005348	0.631098193896616
9	152	translational initiation
GO:0006612	1.23127224219466e-08	17.4595588235294	0.635250155698568
9	153	protein targeting to membrane
GO:0043624	1.23127224219466e-08	17.4595588235294	0.635250155698568
9	153	cellular protein complex disassembly
GO:0043241	1.63102119620009e-08	16.8677457560205	0.656009964708325
9	158	protein complex disassembly
GO:0000184	2.98491410807608e-08	19.7920997920998	0.494083454432219
8	119	nuclear-transcribed mRNA catabolic process, nonsense-mediated decay
GO:0034623	3.7730360310148e-08	15.2149732620321	0.722441353539547
9	174	cellular macromolecular complex disassembly
GO:0032984	4.8202817690404e-08	14.7622837370242	0.743201162549305
9	179	macromolecular complex disassembly
GO:0072594	1.48031863913011e-07	12.8470588235294	0.84700020759809
9	204	establishment of protein localization to organelle
GO:0019080	1.90639954577891e-07	15.3286713286713	0.626946232094665
8	151	viral genome expression
GO:0019083	1.90639954577891e-07	15.3286713286713	0.626946232094665
8	151	viral transcription
GO:0000956	4.52078350387159e-07	13.5977066411849	0.70168154452979
8	169	nuclear-transcribed mRNA catabolic process
GO:0006402	6.70898009617185e-07	12.8696832579186	0.739049200747353
8	178	mRNA catabolic process
GO:0006401	1.9446719025694e-06	11.0847325263569	0.851152169400042
8	205	RNA catabolic process
GO:0071845	2.36772714650336e-06	9.05839572192513	1.1791571517542 9
284	cellular component disassembly at cellular level
GO:0022411	2.65586737324971e-06	8.92599620493359	1.19576499896201
9	288	cellular component disassembly
GO:0019058	4.72285749714866e-06	9.77440496723008	0.959103176250778
8	231	viral infectious cycle
GO:0022415	1.12341186163975e-05	8.63186813186813	1.07951006850737
8	260	viral reproductive process
Tissue: Artery_Tibial=>Skin_Sun_Exposed_Lower_leg	Type:	asymmetric
SourceGene:	MYL6P3(ENSG00000227173.1)
TargetGeneSet:	AURKAIP1	ARHGEF16	FBXO44	SIKE1	C2CD4D	SLC19A2
SNRPG	ARL5A	FASTKD1	MTX2	ZC3H15	DNAJC19	NDUFS6	HMGCR	RPL26L1	7SK	FUT9
RN5S216	ZFAND2A	PSMG3	ACTB	NDUFA4	POP7	RABL5	NDUFB2	COX6C	C8orf55
EXOSC4	ARHGAP39	AK1	FAM73B	ZMYND19	CHCHD1	HECTD2	USMG5	TSSC4
CTNND1	ESRRA	EFHA1	RP11	CTD	SNRPA1	FAM195A	NARFL	MRPS34	TCEB2
SPIRE2	ITGAE	MIS12	COASY	LLGL2	SCARNA17	UQCR11	CSNK1G2	TJP3
DDX39A	NDUFB7	UQCRFS1	U2AF1L4	GNG8	EHD2	ATP5J	MAPK1	CRYBB3	RPS19BP1
GOBPID	Pvalue	OddsRatio	ExpCount	Count	Size	Term
GO:0045333	5.35321403002086e-10	26.1020117862223	0.452010241505778
9	142	cellular respiration
GO:0022904	5.97919403603125e-10	34.2511961722488	0.305584388623625
8	96	respiratory electron transport chain
GO:0022900	8.59309419206233e-09	23.8579782790309	0.426544875787143
8	134	electron transport chain
GO:0015980	4.32947608406704e-07	11.514873934337 0.977233409452633	9
307	energy derivation by oxidation of organic compounds
GO:0006120	4.91239267601197e-06	42.7767857142857	0.114594145733859
4	36	mitochondrial electron transport, NADH to ubiquinone
GO:0006091	6.71209554667473e-06	8.11933174224344	1.36239706594699
9	428	generation of precursor metabolites and energy
GO:0042773	1.44991914134624e-05	31.8095238095238	0.149609023596983
4	47	ATP synthesis coupled electron transport
GO:0042775	1.44991914134624e-05	31.8095238095238	0.149609023596983
4	47	mitochondrial ATP synthesis coupled electron transport
Tissue: Skin_Sun_Exposed_Lower_leg=>Artery_Tibial	Type:	cluster
SourceGene:	PTPRU(ENSG00000060656.14)
TargetGeneSet:	MRPL20	RPL22	ATPIF1	RPS8	UQCRH	PRPF38A	MAGOH	RP4	RP11

BCAS2 C1orf54 MRPS21 GAS5 SNRPE DNAJC27-AS1 HAAO TTC7A MPHOSPH10
DGUOK DGUOK-AS1 RPL31 PPIG FKBP7 EEF1B2 LSM3 RPL15 LZTFL1
RPL10AP6 C3orf15 METTL14 CEP44 BRIX1 MAP1B COX7C RFESD RPL15P3 NOL7
RPS10 RPL10A ENPP5 C6orf225 MRPS12 C7orf50 TOMM7 RABL5 JHDM1D
SSBP1 R3HCC1 RPS6 RPL7A EDF1 AGAP11 DPCD MRPL23 RPL27A C11orf58
SVIP CELF1 TRMT112 EHD1 C11orf70 RPS25 DYRK4 C12orf57 EID3
DIABLO MRPS31 ERH SLIRP LRRC49 PPCDC ANPEP SNHG9 HSDL1 ALDOC SDF2
EIF1 RPL27 COIL RPL38 H3F3B TRIM47 CTD PTPRM RPL17 RPS15
ZNF559 ZNF226 RPL18 ZNF548 ZNF274 RPS5 SLC27A5 RPL12P4 PPDPF PIGP
SNRPD3 RP6 NAP1L2 RPL10
GOBPID Pvalue OddsRatio ExpCount Count Size Term
GO:0006414 3.61800527309792e-23 52.7955665024631 0.537679053352709
18 105 translational elongation
GO:0006415 1.37298687599072e-22 57.6460407776197 0.465988512905681
17 91 translational termination
GO:0000184 3.95990920916677e-22 45.4328147100424 0.609369593799737
18 119 nuclear-transcribed mRNA catabolic process, nonsense-mediated decay
GO:0006614 1.85824725656262e-21 48.4276315789474 0.537679053352709
17 105 SRP-dependent cotranslational protein targeting to membrane
GO:0006613 2.20489973980139e-21 47.8801498127341 0.542799806241783
17 106 cotranslational protein targeting to membrane
GO:0045047 2.20489973980139e-21 47.8801498127341 0.542799806241783
17 106 protein targeting to ER
GO:0072599 2.20489973980139e-21 47.8801498127341 0.542799806241783
17 106 establishment of protein localization to endoplasmic reticulum
GO:0000956 8.59039655703612e-21 32.7652121212121 0.865407238253408
19 169 nuclear-transcribed mRNA catabolic process
GO:0070972 1.50854685816494e-20 42.1559840194546 0.604248840910664
17 118 protein localization to endoplasmic reticulum
GO:0006402 2.35386601763777e-20 30.8910234419668 0.911494014255069
19 178 mRNA catabolic process
GO:0006413 3.89847881365398e-20 34.1649786780384 0.77835443913916
18 152 translational initiation
GO:0043624 4.4019142455727e-20 33.9095238095238 0.783475192028233
18 153 cellular protein complex disassembly
GO:0043241 7.97693921172502e-20 32.6869897959184 0.8090789564736 18
158 protein complex disassembly
GO:0006401 3.57244086218085e-19 26.3566959921799 1.04975434226005
19 205 RNA catabolic process
GO:0034623 4.6959898820806e-19 29.301510989011 0.891011002698775 18
174 cellular macromolecular complex disassembly
GO:0032984 7.88056609561584e-19 28.3815439219166 0.916614767144142
18 179 macromolecular complex disassembly
GO:0019080 1.16065392194971e-18 31.7008379156847 0.773233686250086
17 151 viral genome expression
GO:0019083 1.16065392194971e-18 31.7008379156847 0.773233686250086
17 151 viral transcription
GO:0006612 1.45913639667665e-18 31.2302631578947 0.783475192028233
17 153 protein targeting to membrane
GO:0072594 8.44615921303806e-18 24.5236175115207 1.04463358937098
18 204 establishment of protein localization to organelle
GO:0019058 1.64697046410397e-15 19.738563698967 1.18289391737596 17
231 viral infectious cycle
GO:0071845 3.0200856157906e-15 17.0514232008593 1.45429382049685
18 284 cellular component disassembly at cellular level
GO:0022411 3.85700023655921e-15 16.7940476190476 1.47477683205315
18 288 cellular component disassembly
GO:0022415 1.18047761501009e-14 17.3473395422713 1.33139575115909
17 260 viral reproductive process
GO:0016032 8.18141692971715e-12 10.35103926097 2.30945955297211 18
451 viral reproduction
GO:0033365 1.8035401165751e-11 9.83500784929356 2.42211611653173
18 473 protein localization to organelle

GO:0006605 1.86766659472713e-11 9.81273496240602 2.4272368694208 18
474 protein targeting
GO:0022613 1.80417516525998e-08 11.5556297285269 1.15216940004152
11 225 ribonucleoprotein complex biogenesis
GO:0071843 3.36134997539575e-08 10.8353522695628 1.22385994048855
11 239 cellular component biogenesis at cellular level
GO:0042274 1.83593780917654e-06 58.6244897959184 0.0921735520033216
4 18 ribosomal small subunit biogenesis
GO:0008380 5.47716940716468e-06 7.02076677316294 1.65400318317071
10 323 RNA splicing
GO:0022618 8.89474588802644e-06 14.1652346331791 0.486471524461975
6 95 ribonucleoprotein complex assembly
GO:0042254 1.08641351200467e-05 10.4735127180996 0.76299218047194
7 149 ribosome biogenesis
GO:0000375 1.11299866289987e-05 8.54877129503995 1.07023735381634
8 209 RNA splicing, via transesterification reactions
GO:0071826 1.19563278429271e-05 13.407071339174 0.512075288907342 6
100 ribonucleoprotein complex subunit organization
Tissue: Artery_Tibial=>Skin_Sun_Exposed_Lower_leg Type: asymmetric
SourceGene: RP11-54D18.2(ENSG00000215237.5)
TargetGeneSet: KCNAB2 RP11 CDCA8 IQGAP3 CKAP2L HJURP CAND2 POC1A
NCAPG PALLD TTK CALD1 JPH1 DNAJB5 TPP1 VWA5A CDCA3 ESPL1
KNTC1 LINC00427 CASC4 AURKB SPAG5 NDC80 KANK2 UBE2C C21orf58
GOBPID Pvalue OddsRatio ExpCount Count Size Term
GO:0000280 1.70149855514261e-11 37.6737967914438 0.508615320739049
10 350 nuclear division
GO:0007067 1.70149855514261e-11 37.6737967914438 0.508615320739049
10 350 mitosis
GO:0000087 2.2474302087212e-11 36.5714285714286 0.523147187045879
10 360 M phase of mitotic cell cycle
GO:0048285 2.94510377886324e-11 35.530303030303 0.537679053352709 10
370 organelle fission
GO:0007051 1.20806651538886e-09 77.6 0.11625493045464 6 80
spindle organization
GO:0051301 5.5714723193295e-09 23.6800225733634 0.656840357068715
9 452 cell division
GO:0007059 3.57457495212115e-08 42.6746268656716 0.20344612829562
6 140 chromosome segregation
GO:0000070 6.69163696986665e-07 75.2156862745098 0.0712061449034669
4 49 mitotic sister chromatid segregation
GO:0000819 8.52610879416797e-07 70.5 0.0755657047955159 4 52
sister chromatid segregation
GO:0000226 1.68825441609451e-06 21.5467680608365 0.390907203653726
6 269 microtubule cytoskeleton organization
GO:0008608 1.7751076400133e-06 171.619047619048 0.024704172721611
3 17 attachment of spindle microtubules to kinetochore
GO:0051313 2.52480397672365e-06 150.145833333333 0.027610545982977
3 19 attachment of spindle microtubules to chromosome
GO:0051983 5.2490984608998e-06 114.357142857143 0.0348764791363919
3 24 regulation of chromosome segregation
GO:0000236 6.1814169372142e-06 41.681917211329 0.123520863608055 4
85 mitotic prometaphase
GO:0010948 1.00368476133952e-05 36.6700767263427 0.139505916545568
4 96 negative regulation of cell cycle process
GO:0007052 1.40312171277173e-05 80 0.0479551588125389 3 33
mitotic spindle organization
GO:0007017 1.49419123300948e-05 14.5147286821705 0.571102345858418
6 393 microtubule-based process
GO:0034453 1.68003697599912e-05 74.9895833333333 0.0508615320739049
3 35 microtubule anchoring
Tissue: Artery_Tibial=>Skin_Sun_Exposed_Lower_leg Type: asymmetric
SourceGene: RP11-800K23.2(ENSG00000237350.1)
TargetGeneSet: CTRC CELA2A CELA3B CELA3A AKIRIN1 TPI1P1 AMY2A RP11

APOA2 SSFA2 DPH3 RN5S155 FGB FGA GPBP1 SFTA2 CLPS RP1 PLG
PSMG3 CPA2 CPA1 PRSS1 PRSS3P2 TMUB1 FGL1 DMRT2 BAG1 ARPC5L
PNLIP PNLIPRP1 PNLIPRP2 INS APOC3 APOA1 PLA2G1B NMB
C15orf42 GP2 CTRB2 CTRB1 BTG3
GOBPID Pvalue OddsRatio ExpCount Count Size Term
GO:0007586 6.63892895159599e-16 69.879769299024 0.256452840633866 11
109 digestion
GO:0044241 5.24796719084791e-13 343.047619047619 0.0352916753165871
6 15 lipid digestion
GO:0010903 1.1899789965411e-08 Inf 0.00705833506331742 3 3
negative regulation of very-low-density lipoprotein particle remodeling
GO:0010901 1.18615230345785e-07 697.5 0.0117638917721957 3 5
regulation of very-low-density lipoprotein particle remodeling
GO:0035376 2.36848744672549e-07 464.967741935484 0.0141166701266348
3 6 sterol import
GO:0035382 2.36848744672549e-07 464.967741935484 0.0141166701266348
3 6 sterol transmembrane transport
GO:0070508 2.36848744672549e-07 464.967741935484 0.0141166701266348
3 6 cholesterol import
GO:0034384 9.89971067513112e-07 232.435483870968 0.0211750051899523
3 9 high-density lipoprotein particle clearance
GO:0016042 1.23887328377334e-06 15.6460204885737 0.569372361774272
7 242 lipid catabolic process
GO:0033700 1.41196926651202e-06 199.216589861751 0.0235277835443914
3 10 phospholipid efflux
GO:0022617 1.42607879604727e-06 59.9375 0.084700020759809 4 36
extracellular matrix disassembly
GO:0050709 1.42607879604727e-06 59.9375 0.084700020759809 4 36
negative regulation of protein secretion
GO:0034370 1.93833465391372e-06 174.302419354839 0.0258805618988305
3 11 triglyceride-rich lipoprotein particle remodeling
GO:0034372 1.93833465391372e-06 174.302419354839 0.0258805618988305
3 11 very-low-density lipoprotein particle remodeling
GO:0030299 2.5802890055433e-06 154.924731182796 0.0282333402532697
3 12 intestinal cholesterol absorption
GO:0008202 2.83207296481381e-06 13.7397697322791 0.644661269116324
7 274 steroid metabolic process
GO:0034377 3.34898032474373e-06 139.422580645161 0.0305861186077088
3 13 plasma lipoprotein particle assembly
GO:0065005 3.34898032474373e-06 139.422580645161 0.0305861186077088
3 13 protein-lipid complex assembly
GO:0034375 4.25548315102518e-06 126.739002932551 0.0329388969621479
3 14 high-density lipoprotein particle remodeling
GO:0008203 5.94554982932334e-06 23.0583306477602 0.263511175697184
5 112 cholesterol metabolic process
GO:0050708 7.06079297055722e-06 22.2211867039453 0.27292228911494
5 116 regulation of protein secretion
GO:0043691 7.91151056259763e-06 99.5599078341014 0.0399972320254654
3 17 reverse cholesterol transport
GO:0050995 7.91151056259763e-06 99.5599078341014 0.0399972320254654
3 17 negative regulation of lipid catabolic process
GO:0016125 7.99987331388902e-06 21.6318814277072 0.279980624178258
5 119 sterol metabolic process
GO:0015918 1.21199112232295e-05 33.5906432748538 0.143519479620787
4 61 sterol transport
GO:0030301 1.21199112232295e-05 33.5906432748538 0.143519479620787
4 61 cholesterol transport
GO:0050892 1.31995368287061e-05 81.9734345351044 0.0470555670887828
3 20 intestinal absorption
GO:0018158 1.60955815335276e-05 901 0.00705833506331742 2 3
protein oxidation
GO:0060620 1.60955815335276e-05 901 0.00705833506331742 2 3
regulation of cholesterol import

GO:2000909 1.60955815335276e-05 901 0.00705833506331742 2 3
regulation of sterol import
Tissue: Skin_Sun_Exposed_Lower_leg=>Artery_Tibial Type: asymmetric
SourceGene: RP11-874J12.2(ENSG00000177337.2)
TargetGeneSet: PARK7 PPCS MRPL9 RP11 COX5B WDSUB1 BRK1 LSM3 RP4
ZMYND10 THOC7 NDUFB4 MRPS18C NDUFC1 C5orf42 CDK7 COX7C TTC1 PNPLA1
MRPS18A OGFRL1 C6orf203 MRPS12 ZDHHC4 SKP1P1 COPS5 C10orf31
USMG5 MRPL11 ACER3 SRGAP1 MRP63 MRPS31 DGKH HEATR4 GLRX5 TMEM121
TMEM85 RPL9P25 MT1H COIL SUPT4H1 ATP5H COX6B1 FAM71E1 SNRPB2 FAM182A
ATP5J PIGP SNRPD3 CBY1 NDUFA6
GOBPID Pvalue OddsRatio ExpCount Count Size Term
GO:0022904 9.28994304269143e-11 44.9090909090909 0.245796138675524
8 96 respiratory electron transport chain
GO:0022900 1.36360471479738e-09 31.2818828680898 0.343090443567919
8 134 electron transport chain
GO:0045333 2.16383598522514e-09 29.3978383942357 0.363573455124213
8 142 cellular respiration
GO:0015980 8.55415930448744e-07 13.022719409526 0.78603556847277 8
307 energy derivation by oxidation of organic compounds
GO:0006091 1.0064653866618e-05 9.19146141215107 1.09584111826171
8 428 generation of precursor metabolites and energy
Tissue: Skin_Sun_Exposed_Lower_leg=>Artery_Tibial Type: cluster
SourceGene: RP3-393E18.2(ENSG00000237927.1)
TargetGeneSet: C1QA CD53 RP4 FCGR1A CTSS FCER1G FCGR2A PTPRC PLEK
ITGA4 CCR1 EEF1A1P25 RP11 SFRP2 SLC1A3 GAPT YTHDC2 CD14 DOK3
AIF1 CLEC5A REPIN1 SYK PIK3AP1 SLK SPI1 MS4A7 EFEMP2 KCNE3 BIN2
LCP1 GPR65 ITGAM GFOD2 SLC16A11 PIK3R6 TEX2 CD300A IMPA2
GNA15 VAV1 IFI30 LRRC25 TYROBP FPR3 LAIR1 HCK FAM207CP
TRPM2 CTA CRYBB1 RAC2 CYBB GPR82
GOBPID Pvalue OddsRatio ExpCount Count Size Term
GO:0007229 2.00025953351907e-11 54.6590476190476 0.202338938481766
8 68 integrin-mediated signaling pathway
GO:0006909 5.58738652465578e-09 35.2682841068917 0.255899245726939
7 86 phagocytosis
GO:0030168 1.19105452995402e-08 18.0712669683258 0.645699259566812
9 217 platelet activation
GO:0007596 1.42457603637426e-08 11.0888092393469 1.48183516711646
12 498 blood coagulation
GO:0050900 4.66788626629229e-07 13.9055793991416 0.717113002560376
8 241 leukocyte migration
GO:0002252 1.59135061919244e-06 9.85169293181464 1.1485710331465 9
386 immune effector process
GO:0042590 3.5142206581175e-06 25.4871977240398 0.235070237353816
5 79 antigen processing and presentation of exogenous peptide antigen via
MHC class I
GO:0002283 3.98256092190817e-06 135 0.0327312988720504 3 11
neutrophil activation involved in immune response
GO:0002478 4.48771252450626e-06 24.1734143049933 0.246972527852744
5 83 antigen processing and presentation of exogenous peptide antigen
GO:0019884 5.04778463581109e-06 23.5657894736842 0.252923673102207
5 85 antigen processing and presentation of exogenous antigen
GO:0050867 6.18133996269474e-06 11.8293991416309 0.714137429935645
7 240 positive regulation of cell activation
GO:0002474 1.067374454247e-05 20.0363941769317 0.294581689848453
5 99 antigen processing and presentation of peptide antigen via MHC class
I
GO:0006911 1.08913549185673e-05 89.975 0.0446335893709778 3 15
phagocytosis, engulfment
GO:0010324 1.62097344171137e-05 77.1107142857143 0.0505847346204415
3 17 membrane invagination
GO:0048002 1.6309931321023e-05 18.2741440981093 0.32136184347104
5 108 antigen processing and presentation of peptide antigen
Tissue: Skin_Sun_Exposed_Lower_leg=>Artery_Tibial Type: asymmetric

SourceGene: SLC26A11(ENSG00000181045.10)
TargetGeneSet: RP3 ZNF362 INSL3 TNNI1 KDM3A SMYD1 NEB KBTBD10 PDK1
OXSM SLC22A14 WNT5A LMOD3 SPINK1 HIST1H2BH ADCYAP1R1 AVL9
FZD9 RELN LRRN3 RP11 DLGAP2 ZNF395 C9orf95 IPMK SLC35G1 NRAP
BNIP3 ADM TULP3 CD27 MYF6 MYL2 ZMYM5 MYH7 GRIN2A FA2H
SMTNL2 JMJD6 TGIF1 ANGPTL4 MYBPC2 TNNC2 PVALB MAFF FAM155B
GOBPID Pvalue OddsRatio ExpCount Count Size Term
GO:0030049 9.271688710528e-10 76.8877005347594 0.107951006850737
6 39 muscle filament sliding
GO:0033275 9.271688710528e-10 76.8877005347594 0.107951006850737
6 39 actin-myosin filament sliding
GO:0070252 1.48173203631027e-09 70.4656862745098 0.11625493045464
6 42 actin-mediated cell contraction
GO:0030048 1.22971324712985e-08 47.8068812430633 0.163310497543423
6 59 actin filament-based movement
GO:0006936 2.42527363012568e-07 15.3463203463203 0.661545913777593
8 239 muscle contraction
GO:0060415 2.42552850360207e-07 45.6063492063492 0.138398726731714
5 50 muscle tissue morphogenesis
GO:0048644 4.32016652732376e-07 40.2240896358543 0.15500657393952
5 56 muscle organ morphogenesis
GO:0003012 5.4774389387088e-07 13.7141472868217 0.736281226212719
8 266 muscle system process
GO:0006941 1.87847787915929e-06 29.2673469387755 0.207598090097571
5 75 striated muscle contraction
GO:0007507 3.59997407179999e-06 10.5366766467066 0.946647290844924
8 342 heart development
GO:0055008 6.90807921637003e-06 38.9430894308943 0.124558854058543
4 45 cardiac muscle tissue morphogenesis
GO:0048729 1.22150628670457e-05 8.84785353535353 1.11826171199225
8 404 tissue morphogenesis
Tissue: Artery_Tibial=>Skin_Sun_Exposed_Lower_leg Type: asymmetric
SourceGene: TBL2(ENSG00000106638.9)
TargetGeneSet: CPSF3L CAMK2N1 HTR1D RPL11 ATPIF1 EIF2C4 RPS8 RP4 RP11
RBM8A MRPL55 GUK1 RPS7 UBC WBP1 TXNDC9 RPL31 UGGT1 EEF1B2
CXCR2 CRBN TTLL3 EXOSC7 THOC7 C3orf70 RPL34 UBE2B CAMLG FAM193B
RWDD1 MAP3K4 BRAT1 RALA WBSCR17 HIPK2 OR9A3P COPS5 CDC37L1 RPS6
UBAC1 ANAPC2 TET1 RPS24 LDB1 HRAS C11orf35 FBXO3 CCS
RPS25 CLEC2B HMGB1 N4BP2L1 COMMD6 NDFIP2 ABCD4 IFT43 PLDN SHC4
SPSB3 NUPR1 FUK ZFHX3 NEURL4 RPL26 UTP18 FN3KRP C18orf21
HAUS1 RPL17 LSM7 MLL4 ARHGAP33 ZNF224 ARFGAP3 SLC2A4RG
UCKL1 BTG3 PISD AKAP17A RP3 CXorf56 GPC4
GOBPID Pvalue OddsRatio ExpCount Count Size Term
GO:0006402 2.48818502035394e-15 25.1669419537518 0.825271607501211
15 178 mRNA catabolic process
GO:0006401 2.04842705634507e-14 21.5495951417004 0.950453255830046
15 205 RNA catabolic process
GO:0000956 2.97119394913301e-14 24.2490566037736 0.783544391391599
14 169 nuclear-transcribed mRNA catabolic process
GO:0006414 4.89025394882655e-14 33.5272727272727 0.486817521278804
12 105 translational elongation
GO:0006614 4.89025394882655e-14 33.5272727272727 0.486817521278804
12 105 SRP-dependent cotranslational protein targeting to membrane
GO:0006613 5.49507456161732e-14 33.1682785299807 0.491453878624317
12 106 cotranslational protein targeting to membrane
GO:0045047 5.49507456161732e-14 33.1682785299807 0.491453878624317
12 106 protein targeting to ER
GO:0072599 5.49507456161732e-14 33.1682785299807 0.491453878624317
12 106 establishment of protein localization to endoplasmic reticulum
GO:0070972 2.0417290081453e-13 29.388679245283 0.547090166770466 12
118 protein localization to endoplasmic reticulum
GO:0000184 2.26268763505438e-13 29.1119796091759 0.551726524115978
12 119 nuclear-transcribed mRNA catabolic process, nonsense-mediated decay

GO:0006415 3.19175769529315e-13 35.1214285714286 0.42190851844163
11 91 translational termination
GO:0006413 4.31463379196058e-12 22.1984415584416 0.704726316517888
12 152 translational initiation
GO:0006612 4.66527642874167e-12 22.0394584139265 0.7093626738634 12
153 protein targeting to membrane
GO:0019080 8.73681239153119e-11 19.9852040816327 0.700089959172376
11 151 viral genome expression
GO:0019083 8.73681239153119e-11 19.9852040816327 0.700089959172376
11 151 viral transcription
GO:0043624 1.00755954318332e-10 19.7009557344064 0.7093626738634 11
153 cellular protein complex disassembly
GO:0072594 1.37585044045365e-10 16.1272727272727 0.945816898484534
12 204 establishment of protein localization to organelle
GO:0043241 1.42656683439314e-10 19.0241739552964 0.732544460590963
11 158 protein complex disassembly
GO:0022415 1.64029906771953e-10 13.7787524366472 1.20545290983323
13 260 viral reproductive process
GO:0034623 4.02469233199967e-10 17.1374890446976 0.806726178119161
11 174 cellular macromolecular complex disassembly
GO:0032984 5.44861896223371e-10 16.6215986394558 0.829907964846723
11 179 macromolecular complex disassembly
GO:0071845 6.02781333295069e-09 11.3197860962567 1.31672548612553
12 284 cellular component disassembly at cellular level
GO:0022411 7.0518130646347e-09 11.1525691699605 1.33527091550758
12 288 cellular component disassembly
GO:0019058 8.03056910643846e-09 12.6464285714286 1.07099854681337
11 231 viral infectious cycle
GO:0042274 1.50494185231174e-08 89.1501240694789 0.0834544322192236
5 18 ribosomal small subunit biogenesis
GO:0006364 2.51599543897936e-08 20.180790960452 0.482181163933292 8
104 rRNA processing
GO:0042254 2.65558944957181e-08 15.787684729064 0.690817244481351 9
149 ribosome biogenesis
GO:0016072 3.91547118036927e-08 18.9857095380525 0.509999308006366
8 110 rRNA metabolic process
GO:0022613 7.8293624186879e-08 11.561811505508 1.04318040274029 10
225 ribonucleoprotein complex biogenesis
GO:0016032 1.22484734410462e-07 7.66522915609674 2.0909971628261 13
451 viral reproduction
GO:0071843 1.37753688339582e-07 10.844250363901 1.10808940557747 10
239 cellular component biogenesis at cellular level
GO:0033365 1.53598385943145e-06 6.58946953263656 2.19299702442738
12 473 protein localization to organelle
GO:0006605 1.57023409742074e-06 6.57473435655254 2.19763338177289
12 474 protein targeting
GO:0034470 3.79294432966074e-06 10.0225988700565 0.927271469102484
8 200 ncRNA processing
Tissue: Artery_Tibial=>Skin_Sun_Exposed_Lower_leg Type: asymmetric
SourceGene: TYRP1(ENSG00000107165.7)
TargetGeneSet: CTNNBIP1 RPL11 MEAF6 YBX1 RPS8 PSMD4 B3GALT2
RAB4A TOMM20 RPS7 UBC KCMF1 RPL31 MZT2A BBS5 EIF2S2P4
EEF1B2 RPL15 RPL24 GRK7 EIF2S1 NCBP2 RPL35A RP11 CTD EEF1A1
MRPS12 ZFAND2A POP7 DNAJC2 CHMP4C MTDH RPS6 SURF2 CCNY RPS13 FAU
KRT8P26 RPS25 METAP2 RPL6 MRP63 GTF3A TPT1 C14orf166 MOAP1
HAUS2 RPS3AP6 BTBD1 AXIN1 GFER PALB2 EXOSC6 TAF1C IFT20 RPS7P1
RPL17 RPL19 RPL27 HOXB4 RP13 NAA20 EIF2S2 C20orf24 EEF1B2P3
GOBPID Pvalue OddsRatio ExpCount Count Size Term
GO:0006414 8.00011629816487e-26 80.0130475302889 0.399626323437824
18 105 translational elongation
GO:0006413 1.6705875399692e-24 56.5992063492063 0.578506677738565
19 152 translational initiation
GO:0006415 3.73230934449678e-23 78.337094017094 0.346342813646114 16

91 translational termination
GO:0000184 5.92378821595437e-23 62.6929824561403 0.452909833229534
17 119 nuclear-transcribed mRNA catabolic process, nonsense-mediated decay
GO:0006614 4.32864406170196e-22 65.9498703543647 0.399626323437824
16 105 SRP-dependent cotranslational protein targeting to membrane
GO:0006613 5.0851515085895e-22 65.2125356125356 0.403432288422946
16 106 cotranslational protein targeting to membrane
GO:0045047 5.0851515085895e-22 65.2125356125356 0.403432288422946
16 106 protein targeting to ER
GO:0072599 5.0851515085895e-22 65.2125356125356 0.403432288422946
16 106 establishment of protein localization to endoplasmic reticulum
GO:0000956 6.59679309573166e-22 45.8940397350993 0.643208082485641
18 169 nuclear-transcribed mRNA catabolic process
GO:0006402 1.72451866100566e-21 43.2851351351351 0.67746176735174
18 178 mRNA catabolic process
GO:0070972 3.11421270420208e-21 57.492207139266 0.449103868244412 16
118 protein localization to endoplasmic reticulum
GO:0019080 4.08181428053345e-21 47.614689709348 0.574700712753443 17
151 viral genome expression
GO:0019083 4.08181428053345e-21 47.614689709348 0.574700712753443 17
151 viral transcription
GO:0072594 2.1155673178974e-20 37.1665213600697 0.776416856964916
18 204 establishment of protein localization to organelle
GO:0006401 2.31350997401875e-20 36.9651683769331 0.780222821950038
18 205 RNA catabolic process
GO:0006612 2.33863422759867e-19 42.6996069623807 0.582312642723687
16 153 protein targeting to membrane
GO:0043624 2.33863422759867e-19 42.6996069623807 0.582312642723687
16 153 cellular protein complex disassembly
GO:0043241 3.96637940556713e-19 41.1816540267245 0.601342467649298
16 158 protein complex disassembly
GO:0034623 1.91952148295049e-18 36.9698149951315 0.662237907411252
16 174 cellular macromolecular complex disassembly
GO:0032984 3.0434441731485e-18 35.823187037911 0.681267732336863 16
179 macromolecular complex disassembly
GO:0019058 6.40484091892523e-18 29.6475651746188 0.879177911563214
17 231 viral infectious cycle
GO:0022415 4.76156532692629e-17 26.0559887372753 0.989550896131756
17 260 viral reproductive process
GO:0071845 2.10519686334829e-16 23.6736644983245 1.08089405577469
17 284 cellular component disassembly at cellular level
GO:0022411 2.66173745686471e-16 23.3176344921344 1.09611791571518
17 288 cellular component disassembly
GO:0033365 4.01932061762941e-15 16.2076603034753 1.80022143796277
19 473 protein localization to organelle
GO:0006605 4.17696218883589e-15 16.1708791208791 1.80402740294789
19 474 protein targeting
GO:0016032 2.80398946136548e-14 15.687784782473 1.71649020829008 18
451 viral reproduction
GO:0022613 1.56445492953574e-07 12.8442028985507 0.856342121652481
9 225 ribonucleoprotein complex biogenesis
GO:0071843 2.61152742793885e-07 12.0504725897921 0.909625631444191
9 239 cellular component biogenesis at cellular level
GO:0042274 5.52277506078789e-07 80.5714285714286 0.0685073697321985
4 18 ribosomal small subunit biogenesis
GO:0042254 1.44249463700459e-06 14.6387910798122 0.567088782783198
7 149 ribosome biogenesis
GO:0006364 2.61706669591879e-06 17.8650562265723 0.395820358452702
6 104 rRNA processing
GO:0016072 3.63014331363447e-06 16.8273155416013 0.418656148363435
6 110 rRNA metabolic process
GO:0034470 1.01251906931225e-05 10.7319732297064 0.761192997024427
7 200 ncRNA processing

GO:0034660 1.13038814308804e-05 8.67593517167217 1.08470002075981 8 285 ncRNA metabolic process
Tissue: Skin_Sun_Exposed_Lower_leg=>Artery_Tibial Type: asymmetric
SourceGene: ZNF826P(ENSG00000231205.6)
TargetGeneSet: RPL22 CAMTA1-IT1 RPS8 RP11 RPL31 MAP4K4 MZT2A MRPS18B LY6G5C MRPS12 PMS2P4 RPS6 RPS3AP5 RPS25 KLHDC5 HOXC4 UBC MCF2L ARHGEF40 RPS5 RPL12P4 ASB9
GOBPID Pvalue OddsRatio ExpCount Count Size Term
GO:0006415 1.91762679497595e-12 149.5 0.0944571309943949 7 91 translational termination
GO:0006414 5.35581710072394e-12 128.017857142857 0.108988997301225 7 105 translational elongation
GO:0006614 5.35581710072394e-12 128.017857142857 0.108988997301225 7 105 SRP-dependent cotranslational protein targeting to membrane
GO:0006613 5.73171739753588e-12 126.715909090909 0.110026987751713 7 106 cotranslational protein targeting to membrane
GO:0045047 5.73171739753588e-12 126.715909090909 0.110026987751713 7 106 protein targeting to ER
GO:0072599 5.73171739753588e-12 126.715909090909 0.110026987751713 7 106 establishment of protein localization to endoplasmic reticulum
GO:0070972 1.23276970424851e-11 112.922297297297 0.122482873157567 7 118 protein localization to endoplasmic reticulum
GO:0000184 1.30917922215508e-11 111.90625 0.123520863608055 7 119 nuclear-transcribed mRNA catabolic process, nonsense-mediated decay
GO:0019080 7.09604372233481e-11 86.84375 0.156736558023666 7 151 viral genome expression
GO:0019083 7.09604372233481e-11 86.84375 0.156736558023666 7 151 viral transcription
GO:0006413 7.43497825634392e-11 86.2387931034483 0.157774548474154 7 152 translational initiation
GO:0006612 7.78764418694332e-11 85.6421232876712 0.158812538924642 7 153 protein targeting to membrane
GO:0043624 7.78764418694332e-11 85.6421232876712 0.158812538924642 7 153 cellular protein complex disassembly
GO:0043241 9.77339101656688e-11 82.7773178807947 0.164002491177081 7 158 protein complex disassembly
GO:0000956 1.57100332169022e-10 77.0972222222222 0.175420386132448 7 169 nuclear-transcribed mRNA catabolic process
GO:0034623 1.92905229329879e-10 74.7627245508982 0.180610338384887 7 174 cellular macromolecular complex disassembly
GO:0006402 2.26358693985382e-10 72.9934210526316 0.184762300186838 7 178 mRNA catabolic process
GO:0032984 2.35455743001986e-10 72.5639534883721 0.185800290637326 7 179 macromolecular complex disassembly
GO:0072594 5.89417708112577e-10 63.2442893401015 0.211750051899523 7 204 establishment of protein localization to organelle
GO:0006401 6.09956796824973e-10 62.9204545454545 0.21278804235001 7 205 RNA catabolic process
GO:0019058 1.40565670508218e-09 55.515625 0.239775794062695 7 231 viral infectious cycle
GO:0022415 3.20411362178548e-09 49.0518774703557 0.269877517126842 7 260 viral reproductive process
GO:0071845 5.91575276257488e-09 44.726083032491 0.29478928793855 1 7 284 cellular component disassembly at cellular level
GO:0022411 6.51819085454377e-09 44.0769572953737 0.298941249740502 7 288 cellular component disassembly
GO:0016032 1.42629435581143e-07 27.5743243243243 0.468133693170023 7 451 viral reproduction
GO:0033365 1.97334860926187e-07 26.2312231759657 0.490969483080756 7 473 protein localization to organelle
GO:0006605 2.0019322032261e-07 26.1731798715203 0.492007473531244 7 474 protein targeting
GO:0042274 7.31458391870453e-07 240.35 0.0186838281087814 3 18

ribosomal small subunit biogenesis
GO:0000028 1.50488235864716e-05 555.076923076923 0.00622794270292713
2 6 ribosomal small subunit assembly
Tissue: Whole_Blood=>Artery_Tibial Type: cluster
SourceGene: C6orf226(ENSG00000221821.2)
TargetGeneSet: CTNNBIP1 PPCS HBXIP TMCO1 RP11 SNRPE SLC41A1
TATDN3 LYST-IT1 C2orf28 MORN2 MGAT5 BTF3L4P2 NDUFB3 ARMC9
MYEOV2 GLYCTK SPCS1 NIT2 TCTEX1D2 EXOSC9 NDUFC1 FRG1 PDCD6
RBBP4P1 POC5 COX7C CTD HINT1 SYNPO ATOX1 TMEM14B AGER PPP1R2P1
COX7A2 RP1 PEX7 RP4 PSMB1 NDUFA4 YAE1D1 CLIP2 GTPBP10 C7orf59
PMS2P1 ARF5 CHCHD7 HNRNPA1P4 DECR1 VPS28 NDUFB6 NUDT2 DCTN3
BICD2 SEC61B ATP6V1G1 FAM208B KCNMA1 USMG5 POLR2L IMMP1L CFL1
LAMTOR1 ATP5L SPA17 FOXRED1 FAM60A PRR13 NUP37 PEBP1 RPS7P10 MED4
AKAP6 ERH SLIRP TTC7B NDUFB1 C14orf2 SRP14 FAM96A WDR61 C15orf40
GNAO1 GAN TXNDC17 ARHGAP44 MPRIP C17orf75 PSMB3 MRPS23 BPTF
NT5C ELAC1 UQCR11 NDUFA11 TRAPPC5 NDUFA7 PIN1 NDUFB7 RASAL3 EPS15L1 LSM4
NDUFA13 ALKBH6 SNRPD2 ZNF552 PRNP ROMO1 EPB41L1 ATP5E ABHD16B ATP5J DDT
ADORA2A RP3 RP6 PSMA6P2 EEF1B2P3 RPS2P55 PDZD11 NDUFA1 SSR4
GOBPID Pvalue OddsRatio ExpCount Count Size Term
GO:0022904 2.52654969692243e-17 34.7928849902534 0.604525638364127
15 96 respiratory electron transport chain
GO:0022900 4.37148497605088e-15 23.6195267580717 0.843817036883261
15 134 electron transport chain
GO:0045333 1.05130405045952e-14 22.1192498963945 0.894194173413605
15 142 cellular respiration
GO:0015980 6.54425517789902e-12 11.145852749301 1.93322261435195 17
307 energy derivation by oxidation of organic compounds
GO:0006120 4.73293577437247e-11 49.3356282271945 0.226697114386548
8 36 mitochondrial electron transport, NADH to ubiquinone
GO:0042773 4.64895699389261e-10 35.393265369169 0.295965677115771 8
47 ATP synthesis coupled electron transport
GO:0042775 4.64895699389261e-10 35.393265369169 0.295965677115771 8
47 mitochondrial ATP synthesis coupled electron transport
GO:0006091 1.16324885284373e-09 7.79683698296837 2.6951768043734 17
428 generation of precursor metabolites and energy
GO:0006119 2.67859930206568e-09 27.5855421686747 0.365234239844993
8 58 oxidative phosphorylation
Tissue: Artery_Tibial=>Whole_Blood Type: cluster
SourceGene: CPLX2(ENSG00000145920.9)
TargetGeneSet: CACNA1A HAPLN2 KCNJ9 MYOC ASTN1 C1orf95 ARID4B-IT1 GNG4
MYT1L VSNL1 DPYSL5 CTNNA2 SCN2A NEUROD1 CDK5R2 MEN1 DNER KIF1A
ATP2B2 SLC6A1 ARPP21 CAMKV CADPS SYNPR TAGLN3 GAP43 STXBP5L ZIC1
SOX2-OT PPP2R2C SYNPO2 GRIA2 CTNND2 SOWAHA CAMK2A GABRB2 GABRA6 GABRA1 SNCB
NRSN1 MOG GRM4 RUNX2 RIMS1 SNAP91 FABP7 DGKB VSTM2A WBSCR17
CALN1 SRRM3 TMEM130 DPP6 RP11 NEFM STMN4 STMN2 SH3GL2 DIRAS2
GABBR2 DBC1 NCS1 GRIN1 GPR158 GAD2 INA CALY PTPN5 ANO3
LRRC10B CHRM1 PIH1D2 KCNA1 LMO3 FAIM2 KCNC2 SYT1 N4BP2L2 DCLK1 ZIC2
PPP4R4 CYP46A1 SCG5 CKMT1B RBFOX1 CBLN1 JPH3 PPP1R1B CA10 NPTX1
SOGA2 SYT4 ST8SIA3 CDH20 CELF5 ELAVL3 UNC13A NCAN MAG APLP1
SLC8A2 PRKCG CHGB SNAP25 SLC32A1 PCP4 RASD2 RP1 SEPT3 SULT4A1
TMEM35 TCEAL2 TCEAL5 TCEAL7 PLP1 GRIA3
GOBPID Pvalue OddsRatio ExpCount Count Size Term
GO:0007269 1.12636120430139e-11 24.3517829457364 0.577261089197979
11 86 neurotransmitter secretion
GO:0006836 4.0501787538111e-10 16.8718776916451 0.798768251332088
11 119 neurotransmitter transport
GO:0001505 5.79176591583528e-10 16.2647425249169 0.82561760431804
11 123 regulation of neurotransmitter levels
GO:0003001 2.34256424736022e-08 7.8484002234102 2.12109888589025 14
316 generation of a signal involved in cell-cell signaling
GO:0023061 2.34256424736022e-08 7.8484002234102 2.12109888589025 14
316 signal release
GO:0007214 9.8184283972011e-08 59.9540133779264 0.120822088436786

5	18	gamma-aminobutyric acid signaling pathway
GO:0048489	7.64673956903262e-07	22.4678178963893	0.32219223583143
6	48	synaptic vesicle transport
GO:0016079	1.53219011289151e-06	31.15	0.201370147394644	5	30
synaptic vesicle exocytosis
GO:0007270	2.00961671328466e-06	13.7052126200274	0.590685765690956
7	88	neuron-neuron synaptic transmission
GO:0007611	4.68254573777622e-06	9.61121905888316	0.946439692754827
8	141	learning or memory
GO:0050890	8.98410609145878e-06	8.74742188702478	1.03370008995917
8	154	cognition
GO:0050808	1.13040743836982e-05	10.3560747663551	0.765206560099647
7	114	synapse organization
Tissue: Whole_Blood=>Artery_Tibial	Type:	asymmetric
SourceGene:	CPSF1(ENSG00000071894.9)
TargetGeneSet:	RPL22	NDUFS5	C1orf50	RPS8	RP4	RPL5	RP11	C1orf54
PSMB4	ACBD6	CYB5R1	RPS7	RNF181	CHCHD5	BRK1	RPL15	EIF1B	SPCS1
RPL10AP6		C3orf33	COX7C	CTD	BOD1	TMEM14C	CSNK2B	C6orf48	RPS10
RPL10A	MRPS12	SF3B5	RPA3	CHCHD2	TMEM60	C7orf59	ZNHIT1	TERF1	EIF3H
CPSF1	RPS6	CLTA	RPL7A	ST13P13	MRPL23	RNASEH2C		MRPL11	FTH1P16
ATP5L	U6	WNT1	YEATS4	SLC25A3	RPL18AP3		RPL6	PEBP1	ARL6IP4
NUFIP1	TRAV13-2		AP4S1	COX16	SRP14	RSL24D1	RPS3AP6	COMMD4	SEC11A
TM2D3	ECI1	BCL7C	LDHD	COX4I1	RPAIN	SAT2	RPS7P1	CCDC56	RPL27
SUMO2	ALKBH7	WDR83OS	NDUFB7	MYO9B	RPL18A	SNRPD2	SEPW1	RPS5	FTLP3
MT1P3	YBEY	TXN2	XRCC6	C22orf32		UXT	PQBP1
GOBPID	Pvalue	OddsRatio	ExpCount	Count	Size	Term
GO:0006614	3.81818462543385e-21	55.8866634098681	0.450487855511729
16	105	SRP-dependent cotranslational protein targeting to membrane
GO:0006613	4.48341940480774e-21	55.2618357487923	0.454778216040412
16	106	cotranslational protein targeting to membrane
GO:0045047	4.48341940480774e-21	55.2618357487923	0.454778216040412
16	106	protein targeting to ER
GO:0072599	4.48341940480774e-21	55.2618357487923	0.454778216040412
16	106	establishment of protein localization to endoplasmic reticulum
GO:0070972	2.7306000495633e-20	48.7195225916454	0.50626254238461
16	118	protein localization to endoplasmic reticulum
GO:0006612	2.01779658634231e-18	36.1840685496668	0.65642516088852
16	153	protein targeting to membrane
GO:0006414	1.01774915410727e-17	45.8269230769231	0.450487855511729
14	105	translational elongation
GO:0006413	6.43430904318494e-17	33.2008075788166	0.652134800359837
15	152	translational initiation
GO:0006415	6.50171905539358e-17	48.6768707482993	0.390422808110165
13	91	translational termination
GO:0072594	2.12482464322994e-16	26.2738205365402	0.87523354785136
16	204	establishment of protein localization to organelle
GO:0000184	2.42343410352065e-15	35.7487485560262	0.510552902913293
13	119	nuclear-transcribed mRNA catabolic process, nonsense-mediated decay
GO:0019080	5.61422610328355e-14	27.3976634131914	0.647844439831154
13	151	viral genome expression
GO:0019083	5.61422610328355e-14	27.3976634131914	0.647844439831154
13	151	viral transcription
GO:0043624	6.66687276281697e-14	27.0024781341108	0.65642516088852
13	153	cellular protein complex disassembly
GO:0043241	1.01386702052561e-13	26.0622097114708	0.677876963531935
13	158	protein complex disassembly
GO:0006401	1.36798618509885e-13	21.6810645724258	0.879523908380043
14	205	RNA catabolic process
GO:0000956	2.42934209262384e-13	24.2057823129252	0.72507092934745
13	169	nuclear-transcribed mRNA catabolic process
GO:0034623	3.54202475228955e-13	23.4458106223856	0.746522731990866
13	174	cellular macromolecular complex disassembly
GO:0006402	4.74940310853451e-13	22.8709956709957	0.763684174105598

13	178	mRNA catabolic process
GO:0032984	5.10507677451707e-13	22.731620358987 0.767974534634281	13
179	macromolecular complex disassembly
GO:0019058	7.06772061132108e-13	19.0483870967742	0.991073282125804
14	231	viral infectious cycle
GO:0022415	3.53630696857751e-12	16.7684620596206	1.11549373745762
14	260	viral reproductive process
GO:0033365	8.12075047112856e-12	11.5429337231969	2.02934053006712
17	473	protein localization to organelle
GO:0006605	8.39739015155602e-12	11.5168490153173	2.03363089059581
17	474	protein targeting
GO:0071845	1.74322364236693e-10	13.8213720912719	1.21846239014601
13	284	cellular component disassembly at cellular level
GO:0022411	2.0723769572223e-10	13.6164749536178	1.23562383226074
13	288	cellular component disassembly
GO:0016032	4.99245580574251e-10	10.2134979504197	1.93495259843609
15	451	viral reproduction
GO:0022613	4.54143754582648e-07	11.1422955974843	0.965331118953706
9	225	ribonucleoprotein complex biogenesis
GO:0071843	7.53473849246801e-07	10.4537325676784	1.02539616635527
9	239	cellular component biogenesis at cellular level
Tissue: Whole_Blood=>Artery_Tibial	Type:	cluster
SourceGene:	CPSF1(ENSG00000071894.9)
TargetGeneSet:	RPL22	NDUFS5	C1orf50	RPS8	RP4	RPL5	RP11	C1orf54
PSMB4	ACBD6	CYB5R1	RPS7	RNF181	CHCHD5	BRK1	RPL15	EIF1B	SPCS1
RPL10AP6		C3orf33	COX7C	CTD	BOD1	TMEM14C	CSNK2B	C6orf48	RPS10
RPL10A	MRPS12	SF3B5	RPA3	CHCHD2	TMEM60	C7orf59	ZNHIT1	TERF1	EIF3H
CPSF1	RPS6	CLTA	RPL7A	ST13P13	MRPL23	RNASEH2C		MRPL11	FTH1P16
ATP5L	U6	WNT1	YEATS4	SLC25A3	RPL18AP3		RPL6	PEBP1	ARL6IP4
NUFIP1	TRAV13-2		AP4S1	COX16	SRP14	RSL24D1	RPS3AP6	COMMD4	SEC11A
TM2D3	ECI1	BCL7C	LDHD	COX4I1	RPAIN	SAT2	RPS7P1	CCDC56	RPL27
SUMO2	ALKBH7	WDR83OS	NDUFB7	MYO9B	RPL18A	SNRPD2	SEPW1	RPS5	FTLP3
MT1P3	YBEY	TXN2	XRCC6	C22orf32		UXT	PQBP1
GOBPID	Pvalue	OddsRatio	ExpCount	Count	Size	Term
GO:0006614	3.81818462543385e-21	55.8866634098681	0.450487855511729
16	105	SRP-dependent cotranslational protein targeting to membrane
GO:0006613	4.48341940480774e-21	55.2618357487923	0.454778216040412
16	106	cotranslational protein targeting to membrane
GO:0045047	4.48341940480774e-21	55.2618357487923	0.454778216040412
16	106	protein targeting to ER
GO:0072599	4.48341940480774e-21	55.2618357487923	0.454778216040412
16	106	establishment of protein localization to endoplasmic reticulum
GO:0070972	2.7306000495633e-20	48.7195225916454	0.50626254238461
16	118	protein localization to endoplasmic reticulum
GO:0006612	2.01779658634231e-18	36.1840685496668	0.65642516088852
16	153	protein targeting to membrane
GO:0006414	1.01774915410727e-17	45.8269230769231	0.450487855511729
14	105	translational elongation
GO:0006413	6.43430904318494e-17	33.2008075788166	0.652134800359837
15	152	translational initiation
GO:0006415	6.50171905539358e-17	48.6768707482993	0.390422808110165
13	91	translational termination
GO:0072594	2.12482464322994e-16	26.2738205365402	0.87523354785136
16	204	establishment of protein localization to organelle
GO:0000184	2.42343410352065e-15	35.7487485560262	0.510552902913293
13	119	nuclear-transcribed mRNA catabolic process, nonsense-mediated decay
GO:0019080	5.61422610328355e-14	27.3976634131914	0.647844439831154
13	151	viral genome expression
GO:0019083	5.61422610328355e-14	27.3976634131914	0.647844439831154
13	151	viral transcription
GO:0043624	6.66687276281697e-14	27.0024781341108	0.65642516088852
13	153	cellular protein complex disassembly
GO:0043241	1.01386702052561e-13	26.0622097114708	0.677876963531935

13	158	protein complex disassembly
GO:0006401	1.36798618509885e-13	21.6810645724258	0.879523908380043
14	205	RNA catabolic process
GO:0000956	2.42934209262384e-13	24.2057823129252	0.72507092934745
13	169	nuclear-transcribed mRNA catabolic process
GO:0034623	3.54202475228955e-13	23.4458106223856	0.746522731990866
13	174	cellular macromolecular complex disassembly
GO:0006402	4.74940310853451e-13	22.8709956709957	0.763684174105598
13	178	mRNA catabolic process
GO:0032984	5.10507677451707e-13	22.731620358987 0.767974534634281	13
179	macromolecular complex disassembly
GO:0019058	7.06772061132108e-13	19.0483870967742	0.991073282125804
14	231	viral infectious cycle
GO:0022415	3.53630696857751e-12	16.7684620596206	1.11549373745762
14	260	viral reproductive process
GO:0033365	8.12075047112856e-12	11.5429337231969	2.02934053006712
17	473	protein localization to organelle
GO:0006605	8.39739015155602e-12	11.5168490153173	2.03363089059581
17	474	protein targeting
GO:0071845	1.74322364236693e-10	13.8213720912719	1.21846239014601
13	284	cellular component disassembly at cellular level
GO:0022411	2.0723769572223e-10	13.6164749536178	1.23562383226074
13	288	cellular component disassembly
GO:0016032	4.99245580574251e-10	10.2134979504197	1.93495259843609
15	451	viral reproduction
GO:0022613	4.54143754582648e-07	11.1422955974843	0.965331118953706
9	225	ribonucleoprotein complex biogenesis
GO:0071843	7.53473849246801e-07	10.4537325676784	1.02539616635527
9	239	cellular component biogenesis at cellular level
Tissue: Artery_Tibial=>Whole_Blood	Type:	asymmetric
SourceGene:	EIF1AP1(ENSG00000236698.1)
TargetGeneSet:	CELA2A	CELA3B	CELA3A	COL9A2	TCTEX1D4	AMY2A	ARHGAP30
RP11	REG1A	RAB3GAP1	TMEM45A	CPB1	BASP1	SREK1IP1	CTB
ADAMTS2	TAPBP	ARMC12	CLPS	PKIB	EEPD1	TPST1	CPA2	CPA1	PRSS1
ST13P6	KLF9	OLAH	YWHAZP3	PNLIP	PNLIPRP1	PNLIPRP2	HTRA1
PTGDR2	BIRC3	IL18	CD163	ERGIC2	CPM	PLA2G1B	HDC	GP2	CTD
CTRB2	CTRB1	WRAP53	NF1	ZNF385C	CACNA1A	CYP4F22	RAB3A	SYCN	CEACAM3
CALM3	SIRPG	PRAF2	F8A1
GOBPID	Pvalue	OddsRatio	ExpCount	Count	Size	Term
GO:0007586	4.93335756690257e-11	34.7959459459459	0.346965607916407
9	109	digestion
GO:0044241	1.19534012668532e-07	124.623376623377	0.0477475607224414
4	15	lipid digestion
Tissue: Whole_Blood=>Artery_Tibial	Type:	asymmetric
SourceGene:	ENSG00000205233.4
TargetGeneSet:	RPL22	EIF4G3	RP11	AGL	TOMM20	DGUOK-AS1	HDLBP
RPL15	EIF1B	RPL15P3	DDAH2	RPL10A	SRPK1	ASCC3	FAM120B	GTF2I	COG5
LUC7L2	EIF3H	NFIB	ALAD	PFKP	DAZAP2	TMEM19	NME2P1	OXA1L	GMPR2
RSL24D1	SEC11A	CEP95	TSEN34	BACH1	EIF3D	RRAGB	TTC3P1	RBMX2
GOBPID	Pvalue	OddsRatio	ExpCount	Count	Size	Term
GO:0006413	1.21995167857677e-08	32.8229885057471	0.294512490485088
7	152	translational initiation
Tissue: Whole_Blood=>Artery_Tibial	Type:	asymmetric
SourceGene:	HERC2P2(ENSG00000140181.10)
TargetGeneSet:	KLHL17	RPL22	HMGN2	PPCS	SLC6A9	RPS8	RP11	FGGY
SGIP1	RPL5	BCAS2	PRKAB2	MRPL9	GBAP1	PEX19	PPFIA4	ANKS1B	NUP133
TMEM18	RPS7	C2orf28	MEMO1	GFPT1	SFXN5	CHCHD5	PTPN4	MAP3K2	NCKAP1
SATB2	RAB17	XPC	ACAA1	SPCS1	RPL10AP6	CNTN3	PVRL3-AS1
SPICE1	ZMAT3	CHRD	EXOSC9	NDUFC1	NSA2	COX7C	CTD	P4HA2	SEPT8	IK
CPEB4	ARL10	TMEM14B	BTN2A3P	GPANK1	DDAH2	RPS18	RPS10	RPL10A	RPL24P4
MRPL2	VEGFA	MRPS12	PSMB1	C7orf50	MICALL2	TOMM7	DPY19L1	GTF2I	C7orf59
PMS2P1	MUC3A	ARF5	MRPS33	SLC4A2	CHCHD7	DECR1	C9orf123	RPS6
CHMP5	NUDT2	FAM219A	VPS13A	FSD1L	VAV2	EDF1	RP13	USMG5	OCRL

EIF3M ALKBH3 PLCB3 UCP2 USP35 C11orf1 ATP5L CACNA1C-AS1 LEPREL2
ADCY6 CACNB3 LETMD1 DGKA SLC25A3 CKAP4 PEBP1 SETD8 SAP18 RPS7P10
COMMD6 OXA1L GMPR2 RPS29 DDHD1 SNW1 C14orf2 HERC2P2 HMGN2P5 SRP14
MAPKBP1 DUOX1 RPS3AP6 MAN2A2 GLIS2 UBFD1 NSMCE1 FBXL8 KIAA0513
COX4I1 CYB5D2 LLGL1 RPS7P1 SMARCE1 RPL27 TBKBP1 SLC16A3 CIRBP REEP6
MRPL54 NDUFA7 MYO9B USE1 DPY19L3 FXYD1 ZNF226 SNRPD2 RPL28 RPS5 SNPH
EBF4 VPS16 LINC00493 DTD1 C20orf43 ATP5J CRYZL1 C21orf33
FAM118A ARSD EEF1B2P3 UXT FGD1 PDZD11 RPS23P8 PIN4 RPS6KA6
CXorf56 RP3

GOBPID	Pvalue	OddsRatio	ExpCount	Count	Size	Term
GO:0006614	2.35395294561711e-13	20.0945659844742	0.893709777870044	14	105	SRP-dependent cotranslational protein targeting to membrane
GO:0006613	2.69299584376163e-13	19.8747506980455	0.902221299564044	14	106	cotranslational protein targeting to membrane
GO:0045047	2.69299584376163e-13	19.8747506980455	0.902221299564044	14	106	protein targeting to ER
GO:0072599	2.69299584376163e-13	19.8747506980455	0.902221299564044	14	106	establishment of protein localization to endoplasmic reticulum
GO:0070972	1.22070507063295e-12	17.5666901905434	1.00435955989205	14	118	protein localization to endoplasmic reticulum
GO:0006612	3.0908997356395e-12	14.2814009661836	1.30226281918206	15	153	protein targeting to membrane
GO:0006414	4.65713112799928e-12	18.2873517786561	0.893709777870044	13	105	translational elongation
GO:0006415	1.49929257841184e-11	19.4991447143346	0.774548474154038	12	91	translational termination
GO:0072594	1.73193862839245e-11	11.2467687413005	1.73635042557608	16	204	establishment of protein localization to organelle
GO:0000184	3.69256863969297e-10	14.3682748168729	1.01287108158605	12	119	nuclear-transcribed mRNA catabolic process, nonsense-mediated decay
GO:0006413	5.22731058408295e-10	12.0638979725311	1.29375129748806	13	152	translational initiation
GO:0000956	1.94036717768034e-09	10.7363636363636	1.43844716628607	13	169	nuclear-transcribed mRNA catabolic process
GO:0034623	2.77379799910712e-09	10.3992659514399	1.48100477475607	13	174	cellular macromolecular complex disassembly
GO:0006402	3.66104137082604e-09	10.1442975206612	1.51505086153207	13	178	mRNA catabolic process
GO:0032984	3.91970579103365e-09	10.0824753559693	1.52356238322607	13	179	macromolecular complex disassembly
GO:0019080	5.80069085687583e-09	11.0355823449349	1.28523977579406	12	151	viral genome expression
GO:0019083	5.80069085687583e-09	11.0355823449349	1.28523977579406	12	151	viral transcription
GO:0043624	6.73699148366134e-09	10.877515813686	1.30226281918206	12	153	cellular protein complex disassembly
GO:0043241	9.70034631340651e-09	10.5012958163643	1.34482042765207	12	158	protein complex disassembly
GO:0006401	2.01161612809203e-08	8.70113636363636	1.74486194727009	13	205	RNA catabolic process
GO:0071843	1.22741358088792e-07	7.37433628318584	2.0342536848661	13	239	cellular component biogenesis at cellular level
GO:0019058	6.30297826240347e-07	6.96482784154017	1.9661615113141	12	231	viral infectious cycle
GO:0071845	8.84326593732462e-07	6.13019121100302	2.41727216109612	13	284	cellular component disassembly at cellular level
GO:0022411	1.03484073380331e-06	6.03930578512397	2.45131824787212	13	288	cellular component disassembly
GO:0022415	2.18850039024828e-06	6.13775065387969	2.21299564044011	12	260	viral reproductive process
GO:0033365	2.54245048213041e-06	4.53866132231743	4.0259497612622	16	473	protein localization to organelle
GO:0006605	2.61261196657949e-06	4.52842509080521	4.0344612829562	16		

474	protein targeting
GO:0022613	3.41149192836991e-06	6.47755340453939	1.91509238115009
11	225	ribonucleoprotein complex biogenesis
GO:0015980	1.19544966232335e-05	5.14264773247824	2.61303716005813
12	307	energy derivation by oxidation of organic compounds
GO:0022904	1.73595233003197e-05	9.65449438202247	0.81710608262404
7	96	respiratory electron transport chain
Tissue: Whole_Blood=>Artery_Tibial	Type:	cluster
SourceGene:	HERC2P2(ENSG00000140181.10)
TargetGeneSet:	KLHL17	RPL22	HMGN2	PPCS	SLC6A9	RPS8	RP11	FGGY
SGIP1	RPL5	BCAS2	PRKAB2	MRPL9	GBAP1	PEX19	PPFIA4	ANKS1B	NUP133
TMEM18	RPS7	C2orf28	MEMO1	GFPT1	SFXN5	CHCHD5	PTPN4	MAP3K2	NCKAP1
SATB2	RAB17	XPC	ACAA1	SPCS1	RPL10AP6		CNTN3	PVRL3-AS1
SPICE1	ZMAT3	CHRD	EXOSC9	NDUFC1	NSA2	COX7C	CTD	P4HA2	SEPT8	IK
CPEB4	ARL10	TMEM14B	BTN2A3P	GPANK1	DDAH2	RPS18	RPS10	RPL10A	RPL24P4
MRPL2	VEGFA	MRPS12	PSMB1	C7orf50	MICALL2	TOMM7	DPY19L1	GTF2I	C7orf59
PMS2P1	MUC3A	ARF5	MRPS33	SLC4A2	CHCHD7	DECR1	C9orf123		RPS6
CHMP5	NUDT2	FAM219A	VPS13A	FSD1L	VAV2	EDF1	RP13	USMG5	OCRL
EIF3M	ALKBH3	PLCB3	UCP2	USP35	C11orf1	ATP5L	CACNA1C-AS1		LEPREL2
ADCY6	CACNB3	LETMD1	DGKA	SLC25A3	CKAP4	PEBP1	SETD8	SAP18	RPS7P10
COMMD6	OXA1L	GMPR2	RPS29	DDHD1	SNW1	C14orf2	HERC2P2	HMGN2P5	SRP14
MAPKBP1	DUOX1	RPS3AP6	MAN2A2	GLIS2	UBFD1	NSMCE1	FBXL8	KIAA0513
COX4I1	CYB5D2	LLGL1	RPS7P1	SMARCE1	RPL27	TBKBP1	SLC16A3	CIRBP	REEP6
MRPL54	NDUFA7	MYO9B	USE1	DPY19L3	FXYD1	ZNF226	SNRPD2	RPL28	RPS5	SNPH
EBF4	VPS16	LINC00493		DTD1	C20orf43		ATP5J	CRYZL1	C21orf33
FAM118A	ARSD	EEF1B2P3		UXT	FGD1	PDZD11	RPS23P8	PIN4	RPS6KA6
CXorf56	RP3
GOBPID	Pvalue	OddsRatio	ExpCount	Count	Size	Term
GO:0006614	2.35395294561711e-13	20.0945659844742	0.893709777870044
14	105	SRP-dependent cotranslational protein targeting to membrane
GO:0006613	2.69299584376163e-13	19.8747506980455	0.902221299564044
14	106	cotranslational protein targeting to membrane
GO:0045047	2.69299584376163e-13	19.8747506980455	0.902221299564044
14	106	protein targeting to ER
GO:0072599	2.69299584376163e-13	19.8747506980455	0.902221299564044
14	106	establishment of protein localization to endoplasmic reticulum
GO:0070972	1.22070507063295e-12	17.5666901905434	1.00435955989205
14	118	protein localization to endoplasmic reticulum
GO:0006612	3.09089973563950e-12	14.2814009661836	1.30226281918206
15	153	protein targeting to membrane
GO:0006414	4.65713112799928e-12	18.2873517786561	0.893709777870044
13	105	translational elongation
GO:0006415	1.49929257841184e-11	19.4991447143346	0.774548474154038
12	91	translational termination
GO:0072594	1.73193862839245e-11	11.2467687413005	1.73635042557608
16	204	establishment of protein localization to organelle
GO:0000184	3.69256863969297e-10	14.3682748168729	1.01287108158605
12	119	nuclear-transcribed mRNA catabolic process, nonsense-mediated decay
GO:0006413	5.22731058408295e-10	12.0638979725311	1.29375129748806
13	152	translational initiation
GO:0000956	1.94036717768034e-09	10.7363636363636	1.43844716628607
13	169	nuclear-transcribed mRNA catabolic process
GO:0034623	2.77379799910712e-09	10.3992659514399	1.48100477475607
13	174	cellular macromolecular complex disassembly
GO:0006402	3.66104137082604e-09	10.1442975206612	1.51505086153207
13	178	mRNA catabolic process
GO:0032984	3.91970579103365e-09	10.0824753559693	1.52356238322607
13	179	macromolecular complex disassembly
GO:0019080	5.80069085687583e-09	11.0355823449349	1.28523977579406
12	151	viral genome expression
GO:0019083	5.80069085687583e-09	11.0355823449349	1.28523977579406
12	151	viral transcription
GO:0043624	6.73699148366134e-09	10.877515813686	1.30226281918206	12

153	cellular protein complex disassembly
GO:0043241	9.70034631340651e-09	10.5012958163643	1.34482042765207
12	158	protein complex disassembly
GO:0006401	2.01161612809203e-08	8.70113636363636	1.74486194727009
13	205	RNA catabolic process
GO:0071843	1.22741358088792e-07	7.37433628318584	2.0342536848661 13
239	cellular component biogenesis at cellular level
GO:0019058	6.30297826240347e-07	6.96482784154017	1.9661615113141 12
231	viral infectious cycle
GO:0071845	8.84326593732462e-07	6.13019121100302	2.41727216109612
13	284	cellular component disassembly at cellular level
GO:0022411	1.03484073380331e-06	6.03930578512397	2.45131824787212
13	288	cellular component disassembly
GO:0022415	2.18850039024828e-06	6.13775065387969	2.21299564044011
12	260	viral reproductive process
GO:0033365	2.54245048213041e-06	4.53866132231743	4.0259497612622 16
473	protein localization to organelle
GO:0006605	2.61261196657949e-06	4.52842509080521	4.0344612829562 16
474	protein targeting
GO:0022613	3.41149192836991e-06	6.47755340453939	1.91509238115009
11	225	ribonucleoprotein complex biogenesis
GO:0015980	1.19544966232335e-05	5.14264773247824	2.61303716005813
12	307	energy derivation by oxidation of organic compounds
GO:0022904	1.73595233003197e-05	9.65449438202247	0.81710608262404
7	96	respiratory electron transport chain
Tissue: Whole_Blood=>Artery_Tibial	Type:	cluster
SourceGene:	MED24(ENSG00000008838.11)
TargetGeneSet:	RPL22	ATPIF1	NDUFS5	C1orf50	MAGOH	RP11	PSMB4	RPS27	DPM3
SNRPE	CAPN2	RPL35P1	ACP1	MPHOSPH10	RNF181	CHCHD5	BRK1	C3orf37
GRPEL1	MED10	BRIX1	COX7C	RIOK2	CTD	RP1	ZNRD1	GPANK1	RPL10A
MRPL2	COX7A2	C6orf203	MRPS12	FTSJ2	PMS2CL	TOMM7	YAE1D1	SEC61G
CHCHD2	BUD31	C7orf59	R3HCC1	BRF2	RPL7	VPS13B	EBAG9	EIF3H	EDF1
AGAP11	TSSC4	EIF3F	GSTP1	C11orf1	ATP5L	CACNB3	SLC9A7P1	RPL18AP3
ARL6IP4	SAP18	CHMP4B	RPS29	SLIRP	NDUFB1	GLRX5	C14orf2	SIVA1	SRP14
RSL24D1	SNRNP25	C16orf91	ECI1	TRAPPC2L	RPL27	UTP18	COIL
UQCR11	ZNF554	MRPL54	TIMM44	NDUFA7	PIN1	C19orf42	FXYD1	ZNF568
SNRPD2	SEPW1	A1BG	RPS5	MRPS26	LINC00493	ATP5J	PIGP	THAP7
MAPK1	C22orf32	RAB40A
GOBPID	Pvalue	OddsRatio	ExpCount	Count	Size	Term
GO:0072594	3.31379968836637e-10	14.778125	1.01640024911771	12
204	establishment of protein localization to organelle
GO:0006614	2.31969129398839e-09	21.2544642857143	0.523147187045879
9	105	SRP-dependent cotranslational protein targeting to membrane
GO:0006613	2.52494744947732e-09	21.0338733431517	0.528129541208221
9	106	cotranslational protein targeting to membrane
GO:0045047	2.52494744947732e-09	21.0338733431517	0.528129541208221
9	106	protein targeting to ER
GO:0072599	2.52494744947732e-09	21.0338733431517	0.528129541208221
9	106	establishment of protein localization to endoplasmic reticulum
GO:0070972	6.55689224613394e-09	18.7024901703801	0.587917791156321
9	118	protein localization to endoplasmic reticulum
GO:0006414	4.83291413978044e-08	18.4046391752577	0.523147187045879
8	105	translational elongation
GO:0006413	6.01280058429219e-08	14.2217782217782	0.757317832675939
9	152	translational initiation
GO:0006612	6.3634435923876e-08	14.1220238095238	0.762300186838281
9	153	protein targeting to membrane
GO:0000184	1.28622128383345e-07	16.0675675675676	0.592900145318663
8	119	nuclear-transcribed mRNA catabolic process, nonsense-mediated decay
GO:0000956	1.49643787481541e-07	12.6955357142857	0.842017853435748
9	169	nuclear-transcribed mRNA catabolic process
GO:0006402	2.32952248574824e-07	12.0118343195266	0.886859040896824
9	178	mRNA catabolic process

GO:0006415	3.28291819940666e-07	18.3269230769231	0.453394228773095
7	91	translational termination
GO:0022904	4.74001401796263e-07	17.2912705272256	0.478305999584804
7	96	respiratory electron transport chain
GO:0033365	5.10693945824097e-07	6.6671702284451 2.35665351878763	13
473	protein localization to organelle
GO:0006605	5.23063420198314e-07	6.65222986139196	2.36163587294997
13	474	protein targeting
GO:0006401	7.6718164493642e-07	10.3374635568513	1.02138260328005
9	205	RNA catabolic process
GO:0022613	1.66498998425485e-06	9.36706349206349	1.12102968652688
9	225	ribonucleoprotein complex biogenesis
GO:0071843	2.73842229720722e-06	8.78819875776398	1.19078264479967
9	239	cellular component biogenesis at cellular level
GO:0022900	4.49330549730544e-06	12.0852816474864	0.667635457753789
7	134	electron transport chain
GO:0045333	6.59343012241467e-06	11.362735042735 0.707494291052522	7
142	cellular respiration
GO:0019080	9.8734089327833e-06	10.6458333333333	0.752335478513598
7	151	viral genome expression
GO:0019083	9.8734089327833e-06	10.6458333333333	0.752335478513598
7	151	viral transcription
GO:0043624	1.0761109893349e-05	10.4985247629083	0.762300186838281
7	153	cellular protein complex disassembly
GO:0043241	1.32738544957744e-05	10.14732552216	0.78721195764999	7
158	protein complex disassembly
Tissue: Whole_Blood=>Artery_Tibial	Type:	asymmetric
SourceGene:	NCCRP1(ENSG00000188505.3)
TargetGeneSet:	SPEN	FBXO42	WASF2	MECR	FCRLA	TSEN15	RP11	TET3
BOLA3	UXS1	MTX2	U6	NDUFB3	PTH1R	SETD2	GOLGB1	BCL6	FAM193A
WDR19	ANKRD20A17P	CICP16	NDUFC1	MGST2	NIPBL	C5orf43 RPS23P5 MAN2A1
EEF1E1	LRRC16A HCG23	UBR2	FKBP1C	ARID1B	CYCS	EPDR1	CHCHD2	ACN9
PMS2P1	TRIM56	NAT2	COX6C	COL14A1 C9orf123	NDUFB6	KLF9	SEC61B	RSU1
KIAA0913	UROS	METTL15 NUMA1	MLL	CRABP1	GS1	CPM	UBE2N
MED13L	DENR	ULK1	POLE	GTF2F2	GLRX5	DUOX1	WDR61	IFT140	SRCAP
NUTF2	PSMB10	SLC12A4 GLG1	ANKRD11 GLOD4	CCL13	C17orf96	IER3IP1
NDUFA13 CYP2A7	FKBP1A	MAVS	TMEM230 ARFGEF2 ATP5J	PSMG1	LSS	SLC25A18
SBF1	SYAP1	ACTG1P10	COX7B	C1GALT1C1
GOBPID	Pvalue	OddsRatio	ExpCount	Count	Size	Term
GO:0022904	1.18070954806934e-08	22.4090909090909	0.43844716628607
8	96	respiratory electron transport chain
GO:0022900	1.61923684969259e-07	15.6091954022988	0.61199916960764
8	134	electron transport chain
GO:0045333	2.53300312874695e-07	14.6690684508492	0.648536433464812
8	142	cellular respiration
GO:0018023	3.14349433431358e-06	171.202380952381	0.031970105875026
3	7	peptidyl-lysine trimethylation
Tissue: Whole_Blood=>Artery_Tibial	Type:	cluster
SourceGene:	NCCRP1(ENSG00000188505.3)
TargetGeneSet:	SPEN	FBXO42	WASF2	MECR	FCRLA	TSEN15	RP11	TET3
BOLA3	UXS1	MTX2	U6	NDUFB3	PTH1R	SETD2	GOLGB1	BCL6	FAM193A
WDR19	ANKRD20A17P	CICP16	NDUFC1	MGST2	NIPBL	C5orf43 RPS23P5 MAN2A1
EEF1E1	LRRC16A HCG23	UBR2	FKBP1C	ARID1B	CYCS	EPDR1	CHCHD2	ACN9
PMS2P1	TRIM56	NAT2	COX6C	COL14A1 C9orf123	NDUFB6	KLF9	SEC61B	RSU1
KIAA0913	UROS	METTL15 NUMA1	MLL	CRABP1	GS1	CPM	UBE2N
MED13L	DENR	ULK1	POLE	GTF2F2	GLRX5	DUOX1	WDR61	IFT140	SRCAP
NUTF2	PSMB10	SLC12A4 GLG1	ANKRD11 GLOD4	CCL13	C17orf96	IER3IP1
NDUFA13 CYP2A7	FKBP1A	MAVS	TMEM230 ARFGEF2 ATP5J	PSMG1	LSS	SLC25A18
SBF1	SYAP1	ACTG1P10	COX7B	C1GALT1C1
GOBPID	Pvalue	OddsRatio	ExpCount	Count	Size	Term
GO:0022904	1.18070954806934e-08	22.4090909090909	0.43844716628607
8	96	respiratory electron transport chain
GO:0022900	1.61923684969259e-07	15.6091954022988	0.61199916960764

8	134	electron transport chain
GO:0045333	2.53300312874695e-07	14.6690684508492	0.648536433464812
8	142	cellular respiration
GO:0018023	3.14349433431358e-06	171.202380952381	0.031970105875026
3	7	peptidyl-lysine trimethylation
Tissue: Whole_Blood=>Artery_Tibial	Type:	asymmetric
SourceGene:	PCBP1(ENSG00000169564.5)
TargetGeneSet:	ATPIF1	SLC16A1	DPM3	NAV1	RP11	MAPKAPK2	RPL35P1
CHRM3-AS2	STEAP3	PDE11A	ARMC9	LSM3	C3orf78	CNTN3	PDGFC	GLRB	CTD
ANKH	DAB2	MAN2A1	CPEB4	ECI2	COX6A1P2	SLC29A1	COX7A2	TRAF3IP2
GJA1	HDAC7	AEBP1	AUTS2	CHCHD7	DECR1	COX6C	C9orf123	C9orf133
PLXDC2	HK1	USMG5	POLR2L	NAV2	ODZ4	ATP5L	CLMP	ADCY6	SAP18
RPS29	ERH	NDUFB1	C14orf2	THBS1	SRP14	MYEF2	ALPK3	EIF3C	BCAR1
MMP28	TOB1	UQCR11	NDUFA7	PIN1	NDUFA13	EPS8L1	ROMO1	ATP5E	ATP5J
RBFOX2	RP6	PIN4	NDUFA1	GRIA3
GOBPID	Pvalue	OddsRatio	ExpCount	Count	Size	Term
GO:0022904	7.14548993627377e-11	32.8965517241379	0.358729499688603
9	96	respiratory electron transport chain
GO:0022900	1.44440940593033e-09	22.8352	0.500726593315342	9	134
electron transport chain
GO:0045333	2.41768474624567e-09	21.4496240601504	0.530620718289392
9	142	cellular respiration
GO:0006091	4.34195864528954e-08	9.60233516483516	1.59933568611169
12	428	generation of precursor metabolites and energy
GO:0015980	1.69250226508262e-07	10.7897153351699	1.14718704587918
10	307	energy derivation by oxidation of organic compounds
GO:0042776	1.37381713630155e-05	84.6294117647059	0.0485779530828316
3	13	mitochondrial ATP synthesis coupled proton transport
Tissue: Whole_Blood=>Artery_Tibial	Type:	cluster
SourceGene:	PISD(ENSG00000241878.4)
TargetGeneSet:	NPHP4	SPEN	RP11	UBR4	ATPIF1	SRSF4	ZCCHC17	INPP5B
SCMH1	PPCS	RPS8	NSUN4	SCP2	ACOT11	JAK1	PIGK	RWDD3	HBXIP
LRIG2	S100A6	UBQLN4	SMG5	ARHGEF11	BRP44	CEP350	TSEN15	C1orf132
NSL1	ARV1	AHCTF1	PQLC3	PTRHD1	SLC5A6	C2orf28	RPLP0P6	MORN2	CALM2
COMMD1	MDH1	SUCLG1	RNF181	SNRNP200	SMPD4	DAPL1	BTF3L4P2
NDUFB3	TTLL4	SETD5	TATDN2	LSM3	SCAP	SPCS1	PDHB	LRRC34	LETM1
SLC30A9	TMSB4XP8	LAMTOR3	PPA2	HIGD1AP14	NDUFC1	MGST2	SUB1
NIPBL	COX7C	CTD	RFESD	HINT1	PDF	ATOX1	TTC1	ARL10	TMEM14C
TMEM14B	ABCF1	BAG6	RPS18	RP3	BRPF3	XPO5	SLC29A1	COX7A2	WDR11
ECHDC1	MRPS12	RP1	HIVEP2	ZDHHC14	RP4	PSMB1	FBXL18	RPA3	NDUFA4
SEPT7	EPDR1	SPDYE1	CHCHD2	POM121C	PMS2P1	TRIM56	RABL5	ARF5	C7orf73
MRPS33	VDAC3	CHCHD7	DECR1	COX6C	C9orf123	MPDZ	LINC00032
NDUFB6	CHMP5	DCTN3	SNX18P3	FAM27D1	KIF27	TSTD2	TXN	ATP6V1G1
ZNF79	FAM102A	CIZ1	HMGA1P4	SETX	FAM208B	SEC24C	KIAA0913	RP13	LGI1
DPCD	USMG5	PDCD11	PHRF1	CYB5R2	AKIP1	NAV2-AS2	IMMP1L	ELP4
NAT10	INCENP	NXF1	MARK2	KLC2	BBS1	KDM2A	LAMTOR1	ARHGEF17
CLNS1A	TAGLN	ATP5L	IGSF9B	NCAPD3	CACNA1C-AS2	ADCY6	POU6F1	SRGAP1
NDUFA12	CCDC53	NUP37	RBM19	RPLP0	POP5	KDM2B	POLE	POMP	RFXAP	DGKH
MED4	COMMD6	A2LD1	RASA3	ACIN1	C14orf166	HEATR4	MLH3	NDUFB1
C14orf2	KIAA0284	SRP14	MAPKBP1	DYX1C1-CCPG1	GTF2A2	KIAA0101
RAB11A	RPLP1	MYO9A	WDR61	C15orf40	ZNF592	KIF7	RPL36AP43
MAN2A2	ZNF598	CREBBP	CLEC16A	PDXDC1	SRCAP	RNF40	ITFG1	EDC4	SLC12A4	AARS
FBXO31	ZC3H18	VPS53	MINK1	LSMD1	AURKB	STX8	FLCN	SUPT6H	MMP28
MLLT6	TOP2A	SMARCE1	STAT5B	STAT3	HSD17B1P1	ICT1	UNK	UBE2O
TNRC6C	USP36	MYL12B	ZNF397	EPG5	CYB5A	UQCR11	DOT1L	NDUFA11	TRAPPC5
NDUFA7	ZNF317	PIN1	KRI1	TNPO2	NDUFB7	MYO9B	NDUFA13	RPSAP58	MLL4
SAMD4B	SYMPK	ZNF841	ZNF417	RPS5	EBF4	MAVS	SNRPB2	DTD1	ZNF334
ZNFX1	PFDN4	RPL12P4	C20orf43	ATP5E	PPIAP22	LINC00515	ATP5J
SCAF4	URB1	LRRC3-AS1	MCM3AP	DIP2A	MTMR3	CTA	GTPBP1	TTLL12	SBF1
TAB3-AS1	ATP6AP2	RPS2P55	FUNDC1	UBA1	PAGE2B	PDZD11	PIN4	COX7B
RPSAP9	NGFRAP1	NDUFA1	VAMP7	MT
GOBPID	Pvalue	OddsRatio	ExpCount	Count	Size	Term
GO:0045333	3.07183877498828e-15	12.3687703481092	2.15196180195142

21	142	cellular respiration
GO:0022904	4.24854673243702e-15	16.2502870264064	1.45484741540378
18	96	respiratory electron transport chain
GO:0022900	1.20300542110449e-14	12.4464427400159	2.03072451733444
20	134	electron transport chain
GO:0015980	2.74983792662288e-10	5.76322219028456	4.65248079717667
23	307	energy derivation by oxidation of organic compounds
GO:0006091	1.67109622141055e-09	4.6345990255974 6.48619472700851	26
428	generation of precursor metabolites and energy
GO:0006120	5.14275840883601e-08	19.2335815842925	0.545567780776417
8	36	mitochondrial electron transport, NADH to ubiquinone
GO:0042773	4.63091380783589e-07	13.7980313525337	0.712269047124766
8	47	ATP synthesis coupled electron transport
GO:0042775	4.63091380783589e-07	13.7980313525337	0.712269047124766
8	47	mitochondrial ATP synthesis coupled electron transport
GO:0006119	2.44649806854381e-06	10.7541232227488	0.878970313473116
8	58	oxidative phosphorylation
Tissue: Whole_Blood=>Artery_Tibial	Type:	asymmetric
SourceGene:	RP11-403P17.2(ENSG00000260650.1)
TargetGeneSet:	C1orf122	PPCS	MAGOH	USP24	HBXIP	ARV1	OST4
MORN2	MDH1	BOLA3	NIF3L1	NDUFB3	MYEOV2	BRK1	CMC1	SPTSSB	MRPL47
NDUFC1	PDCD6	SLC9A3	C5orf43 COMMD10 ATOX1	FAM114A2	COX6A1P2
COX7A2	WDR11	RPA3	NDUFA4	MPLKIP	SEC61G	PMS2P1	ZNHIT1	C7orf73 TCEB1
DECR1	COX6C	HAS2	C9orf123	NDUFB6	SEC61B	RP11	UROS	IMMP1L
TMEM126B	TMEM126A	ATP5L	HIGD1AP1	NDUFA12 CCDC53	POP5	DENR
ULK1	ERH	SLIRP	NDUFB1	ATG2B	C14orf2 DYX1C1-CCPG1	GTF2A2	ANKRD11
TXNDC17 ICT1	NDUFA7	PIN1	NDUFB7	NDUFA13 COX7A1	SNRPD2	ATP5E	PPIAP22
ATP5J	FAM104B PDZD11	COX7B	NXT2	NDUFA1	C1GALT1C1
GOBPID	Pvalue	OddsRatio	ExpCount	Count	Size	Term
GO:0022904	2.1771608472011e-20	60.2314814814815	0.391945194104214
15	96	respiratory electron transport chain
GO:0045333	2.43668636871758e-19	42.1291989664083	0.57975226627915
16	142	cellular respiration
GO:0022900	4.07883718536453e-18	40.8890374331551	0.547090166770466
15	134	electron transport chain
GO:0015980	5.61468734236273e-14	18.0305282506194	1.25340806864577
16	307	energy derivation by oxidation of organic compounds
GO:0006091	6.71170885717984e-13	13.7687985169737	1.74742232371462
17	428	generation of precursor metabolites and energy
GO:0006120	1.30518094614004e-12	80.4705882352941	0.14697944778908
8	36	mitochondrial electron transport, NADH to ubiquinone
GO:0042773	1.31017536476418e-11	57.7295123177476	0.191889834613522
8	47	ATP synthesis coupled electron transport
GO:0042775	1.31017536476418e-11	57.7295123177476	0.191889834613522
8	47	mitochondrial ATP synthesis coupled electron transport
GO:0006119	7.71454992997175e-11	44.9945098039216	0.236800221437963
8	58	oxidative phosphorylation
GO:0042776	1.79589970093787e-05	77.0464285714286	0.0530759117016123
3	13	mitochondrial ATP synthesis coupled proton transport
Tissue: Whole_Blood=>Artery_Tibial	Type:	cluster
SourceGene:	RP11-403P17.2(ENSG00000260650.1)
TargetGeneSet:	C1orf122	PPCS	MAGOH	USP24	HBXIP	ARV1	OST4
MORN2	MDH1	BOLA3	NIF3L1	NDUFB3	MYEOV2	BRK1	CMC1	SPTSSB	MRPL47
NDUFC1	PDCD6	SLC9A3	C5orf43 COMMD10 ATOX1	FAM114A2	COX6A1P2
COX7A2	WDR11	RPA3	NDUFA4	MPLKIP	SEC61G	PMS2P1	ZNHIT1	C7orf73 TCEB1
DECR1	COX6C	HAS2	C9orf123	NDUFB6	SEC61B	RP11	UROS	IMMP1L
TMEM126B	TMEM126A	ATP5L	HIGD1AP1	NDUFA12 CCDC53	POP5	DENR
ULK1	ERH	SLIRP	NDUFB1	ATG2B	C14orf2 DYX1C1-CCPG1	GTF2A2	ANKRD11
TXNDC17 ICT1	NDUFA7	PIN1	NDUFB7	NDUFA13 COX7A1	SNRPD2	ATP5E	PPIAP22
ATP5J	FAM104B PDZD11	COX7B	NXT2	NDUFA1	C1GALT1C1
GOBPID	Pvalue	OddsRatio	ExpCount	Count	Size	Term
GO:0022904	2.1771608472011e-20	60.2314814814815	0.391945194104214
15	96	respiratory electron transport chain

GO:0045333	2.43668636871758e-19	42.1291989664083	0.57975226627915
16	142	cellular respiration
GO:0022900	4.07883718536453e-18	40.8890374331551	0.547090166770466
15	134	electron transport chain
GO:0015980	5.61468734236273e-14	18.0305282506194	1.25340806864577
16	307	energy derivation by oxidation of organic compounds
GO:0006091	6.71170885717984e-13	13.7687985169737	1.74742232371462
17	428	generation of precursor metabolites and energy
GO:0006120	1.30518094614004e-12	80.4705882352941	0.14697944778908
8	36	mitochondrial electron transport, NADH to ubiquinone
GO:0042773	1.31017536476418e-11	57.7295123177476	0.191889834613522
8	47	ATP synthesis coupled electron transport
GO:0042775	1.31017536476418e-11	57.7295123177476	0.191889834613522
8	47	mitochondrial ATP synthesis coupled electron transport
GO:0006119	7.71454992997175e-11	44.9945098039216	0.236800221437963
8	58	oxidative phosphorylation
GO:0042776	1.79589970093787e-05	77.0464285714286	0.0530759117016123
3	13	mitochondrial ATP synthesis coupled proton transport
Tissue: Whole_Blood=>Artery_Tibial	Type:	cluster
SourceGene:	RP11-84A14.5(ENSG00000223989.1)
TargetGeneSet:	TPRG1L	RP11	NDUFS5	RP4	GTF2B	BCAS2	PSMB4	DPM3	STX6
MIR4426 RPS7	FAHD2A	MGAT5	DNPEP	ZNF385D SPCS1	RPL10AP6	ZMAT3
NDUFC1	SEMA5A	COX7C	CTD	HINT1	P4HA2	TMEM14B RPS18	RPS10	RPL10A
RPL24P4 MRPL2	ASCC3	RP1	MRPS12	TOMM7	PMS2P1	ARF5	NDUFB9	PUF60
C9orf133	NUDT2	IER5L	USMG5	PEX16	LAMTOR1 ATP5L	FAM60A	PRKAG1
PRR13	PEBP1	RPS7P10 EAPP	RPS29	ERH	COQ6	SLIRP	NDUFB1	C14orf2
SRP14	SEC11A	FAM192A MIR22HG SLC52A1 ZSWIM7	MPRIP-AS1	RPS7P1	SMARCE1
RPL27	SLC16A3 MADCAM1 UQCR11	NDUFA7	NDUFB7	EID2	SNRPD2	NLRP9	RPS5
LINC00493	ROMO1	C20orf43	ATP5J	U2AF1	EEF1B2P3	PIN4
CXorf56 NDUFA1	RP3	PLAC1	MTCP1
GOBPID	Pvalue	OddsRatio	ExpCount	Count	Size	Term
GO:0022904	5.84239041621181e-13	33.0439075630252	0.445090305169192
11	96	respiratory electron transport chain
GO:0070972	5.85953004275699e-12	26.2094459279039	0.547090166770466
11	118	protein localization to endoplasmic reticulum
GO:0022900	2.37721101989593e-11	22.7745354239257	0.621271884298664
11	134	electron transport chain
GO:0045333	4.47957813120369e-11	21.3717284623773	0.658362743062764
11	142	cellular respiration
GO:0006614	4.65758776247962e-11	26.387811634349 0.486817521278804	10
105	SRP-dependent cotranslational protein targeting to membrane
GO:0006613	5.12424242892513e-11	26.1111111111111	0.491453878624317
10	106	cotranslational protein targeting to membrane
GO:0045047	5.12424242892513e-11	26.1111111111111	0.491453878624317
10	106	protein targeting to ER
GO:0072599	5.12424242892513e-11	26.1111111111111	0.491453878624317
10	106	establishment of protein localization to endoplasmic reticulum
GO:0006612	1.00755954318332e-10	19.7009557344064	0.7093626738634 11
153	protein targeting to membrane
GO:0072594	1.37585044045365e-10	16.1272727272727	0.945816898484534
12	204	establishment of protein localization to organelle
GO:0006120	2.50508621655278e-10	57.75	0.166908864438447	7	36
mitochondrial electron transport, NADH to ubiquinone
GO:0015980	1.26395112380204e-09	11.5375409423028	1.42336170507231
13	307	energy derivation by oxidation of organic compounds
GO:0042773	1.81322448644403e-09	41.8366666666667	0.217908795239084
7	47	ATP synthesis coupled electron transport
GO:0042775	1.81322448644403e-09	41.8366666666667	0.217908795239084
7	47	mitochondrial ATP synthesis coupled electron transport
GO:0006091	7.55600629287101e-09	8.91349922523015	1.98436094387932
14	428	generation of precursor metabolites and energy
GO:0006119	8.3283323725346e-09	32.787908496732 0.26890872603972	7
58	oxidative phosphorylation

GO:0006414 2.71359653037751e-08 19.9713437008562 0.486817521278804
8 105 translational elongation
GO:0006415 1.98806726427512e-07 19.8611111111111 0.42190851844163
7 91 translational termination
GO:0000184 1.24253009417208e-06 14.8666666666667 0.551726524115978
7 119 nuclear-transcribed mRNA catabolic process, nonsense-mediated decay
GO:0034623 1.33958056585863e-06 11.6136410046968 0.806726178119161
8 174 cellular macromolecular complex disassembly
GO:0033365 1.53598385943145e-06 6.58946953263656 2.19299702442738
12 473 protein localization to organelle
GO:0006605 1.57023409742074e-06 6.57473435655254 2.19763338177289
12 474 protein targeting
GO:0032984 1.65764254102156e-06 11.2700961443156 0.829907964846723
8 179 macromolecular complex disassembly
GO:0019080 6.08834441710184e-06 11.537037037037 0.700089959172376 7
151 viral genome expression
GO:0019083 6.08834441710184e-06 11.537037037037 0.700089959172376 7
151 viral transcription
GO:0006413 6.35906666054627e-06 11.4566666666667 0.704726316517888
7 152 translational initiation
GO:0043624 6.63973181469041e-06 11.377397260274 0.7093626738634 7 153
cellular protein complex disassembly
GO:0043241 8.20245376989087e-06 10.9967991169978 0.732544460590963
7 158 protein complex disassembly
GO:0000956 1.27355058579536e-05 10.2421810699588 0.783544391391599
7 169 nuclear-transcribed mRNA catabolic process
GO:0006402 1.78367876385405e-05 9.69697855750487 0.825271607501211
7 178 mRNA catabolic process
Tissue: Nerve_Tibial=>Heart_Left_Ventricle Type: cluster
SourceGene: AIFM3(ENSG00000183773.10)
TargetGeneSet: NUDC NR0B2 CDC20 NEDD4 RP4 HSD3BP3 FAM72B SNX18P15
IQGAP3 USP21 ASPM NEK2 CEP170 STARD7 NCAPH HJURP JAGN1 KCTD6
IFT122 CTA GLRB KIF20A CDC25C LSM11 HMMR GPANK1 LY6G6E KIFC1
MTCH1 FKBP9 ANLN NSUN5 POR ASB15 FZD3 RP11 MELK GKAP1
COBRA1 CEP55 MKI67 TSSC4 CDCA5 CARNS1 MPP2 FANCB CGRRF1 DLGAP5
BUB1B CASC5 NUSAP1 KIAA0101 PKM2 FANCI C15orf42 ZNF48 DEF8
GGT6 AURKB TOP2A KIF18B MRPL45P2 BIRC5 NDC80 ASXL3 SKA1 CTD
MAN2B1 UNC13A LGALS4 SNPH SIRPG TPX2 UBE2C PHEX RBM10 SPIN4
GOBPID Pvalue OddsRatio ExpCount Count Size Term
GO:0000087 5.77840233219617e-20 22.3048058882956 1.56944156113764
22 360 M phase of mitotic cell cycle
GO:0051301 4.18480609018208e-19 18.7096153846154 1.97052107120614
23 452 cell division
GO:0000280 7.0432102634161e-19 21.3662613981763 1.52584596221715
21 350 nuclear division
GO:0007067 7.0432102634161e-19 21.3662613981763 1.52584596221715
21 350 mitosis
GO:0048285 2.21082121499435e-18 20.1131805157593 1.61303716005813
21 370 organelle fission
GO:0007059 8.25432922759039e-16 32.3401360544218 0.610338384886859
14 140 chromosome segregation
GO:0010564 1.28904743512527e-12 13.0241485050925 1.80485779530828
17 414 regulation of cell cycle process
GO:0000819 5.33240829785879e-11 47.4181818181818 0.226697114386548
8 52 sister chromatid segregation
GO:0007346 4.7916273970545e-10 12.6395862068966 1.32094664729084
13 303 regulation of mitotic cell cycle
GO:0007017 1.07010218486297e-09 10.5608744817188 1.71330703757525
14 393 microtubule-based process
GO:0000070 1.5802192543395e-09 42.6964285714286 0.213618434710401
7 49 mitotic sister chromatid segregation
GO:0000236 3.04867722569876e-09 27.0337662337662 0.370562590824164
8 85 mitotic prometaphase

GO:0000910 6.83952572361645e-09 24.1894291754757 0.409798629852605
8 94 cytokinesis
GO:0051313 1.48596772101876e-08 88.5098522167488 0.0828316379489309
5 19 attachment of spindle microtubules to chromosome
GO:0000226 1.98210574086758e-08 11.5854203935599 1.17272161096118
11 269 microtubule cytoskeleton organization
GO:0007088 1.02282884249281e-07 22.0787037037037 0.383641270500311
7 88 regulation of mitosis
GO:0051783 1.02282884249281e-07 22.0787037037037 0.383641270500311
7 88 regulation of nuclear division
GO:0051656 2.65134781667083e-07 19.0079787234043 0.440315549096948
7 101 establishment of organelle localization
GO:0051640 2.67051307331495e-07 14.5925736235595 0.653933983807349
8 150 organelle localization
GO:0034453 3.93211835776371e-07 41.2586206896552 0.152584596221715
5 35 microtubule anchoring
GO:0051329 6.32935923564417e-07 8.05915551839465 1.65227319908657
11 379 interphase of mitotic cell cycle
GO:0008608 7.48018024905164e-07 74.9674054758801 0.0741125181648329
4 17 attachment of spindle microtubules to kinetochore
GO:0051325 7.7772033780125e-07 7.88318330605565 1.68714967822296
11 387 interphase
GO:0031577 1.00651369015586e-06 33.4366262814539 0.183101515466058
5 42 spindle checkpoint
GO:0007051 1.25766306931074e-06 20.3613086770982 0.348764791363919
6 80 spindle organization
GO:0050000 1.85661134868087e-06 57.3120638085743 0.0915507577330289
4 21 chromosome localization
GO:0051303 1.85661134868087e-06 57.3120638085743 0.0915507577330289
4 21 establishment of chromosome localization
GO:0071156 1.97760347735792e-06 9.23725490196078 1.15092381150093
9 264 regulation of cell cycle arrest
GO:0000086 2.92583069556219e-06 13.0027372262774 0.627776624455055
7 144 G2/M transition of mitotic cell cycle
GO:0007050 3.99539455496947e-06 7.37320649603195 1.60867760016608
10 369 cell cycle arrest
GO:0051651 6.90324640129683e-06 14.8900468994268 0.466472908449242
6 107 maintenance of location in cell
GO:0030261 8.25500485226602e-06 37.4498044328553 0.13078679676147
4 30 chromosome condensation
GO:0000075 9.64188258652613e-06 8.7981351981352 1.05501349387586 8
242 cell cycle checkpoint
GO:0071173 1.37882324114023e-05 32.4474576271186 0.148225036329666
4 34 spindle assembly checkpoint
GO:0007096 1.68925712656398e-05 79.8833333333333 0.0523147187045879
3 12 regulation of exit from mitosis
GO:0045786 1.75605492402451e-05 6.16897176439397 1.90512767282541
10 437 negative regulation of cell cycle
GO:0033043 1.89948618539111e-05 6.10996804272644 1.92256591239361
10 441 regulation of organelle organization
Tissue: Nerve_Tibial=>Heart_Left_Ventricle Type: cluster
SourceGene: CCDC90B(ENSG00000137500.5)
TargetGeneSet: RP11 GPX6 MTMR11 PRELP ETNK2 KIAA1383 RPL31 TSPO
RPL37A SNED1 RBMS3 ZBTB47 RPL10AP6 BBX ITGB5 ABTB1 FGF12
GMPSP1 RPL34 USP53 MSNP1 RPL37 JMY CREBRF RPS18 ADCYAP1R1
YAE1D1 SRI CTB ATP5J2 GSTK1 GS1 GRIP1 TMEM55A RPL30 YWHAZ
CYHR1 C9orf156 SLC44A1 NTNG2 ARRDC1 RSU1 PRTFDC1 ZNF22 ERCC6
RPL13AP5 FXC1 NR1H3 ZBTB16 DYRK4 TMTC1 FGD4 RPL14P1 C12orf26
GNPTAB ULK1 STXBP6 RPL36A NEK9 JDP2 POLR2M FAM81A SEC11A CTD
JMJD5 SLC7A6OS MON1B FXR2 PER1 RPL26 HS3ST3A1 RPL23A GIT1
RPL17 PLEKHM1 SLC38A10 OSBPL1A TRAPPC8 SBNO2 MKNK2 C19orf43
UBA52 SLC25A42 CCDC9 MYH7B ATP9A URB1 BCL2L13 PLA2G6 EEF1B2P3
RP3 FAM127B

GOBPID Pvalue OddsRatio ExpCount Count Size Term
GO:0072594 2.58412319246211e-13 20.5102167182663 0.917583558231264
14 204 establishment of protein localization to organelle
GO:0006614 1.12668141048972e-12 30.9716312056738 0.472285654971974
11 105 SRP-dependent cotranslational protein targeting to membrane
GO:0006613 1.25281388558025e-12 30.643469785575 0.476783613590755 11
106 cotranslational protein targeting to membrane
GO:0045047 1.25281388558025e-12 30.643469785575 0.476783613590755 11
106 protein targeting to ER
GO:0072599 1.25281388558025e-12 30.643469785575 0.476783613590755 11
106 establishment of protein localization to endoplasmic reticulum
GO:0006612 3.19068225975015e-12 22.8743476515456 0.688187668673448
12 153 protein targeting to membrane
GO:0070972 4.13732373446474e-12 27.1839736933195 0.530759117016123
11 118 protein localization to endoplasmic reticulum
GO:0006415 7.95881032947131e-12 32.1099887766554 0.409314234309044
10 91 translational termination
GO:0006414 3.40266175495566e-11 27.3511961722488 0.472285654971974
10 105 translational elongation
GO:0000184 1.19525470078418e-10 23.814845704754 0.535257075634904 10
119 nuclear-transcribed mRNA catabolic process, nonsense-mediated decay
GO:0019080 1.25866887322011e-09 18.3687943262411 0.679191751435887
10 151 viral genome expression
GO:0019083 1.25866887322011e-09 18.3687943262411 0.679191751435887
10 151 viral transcription
GO:0006413 1.34262074072221e-09 18.2381562099872 0.683689710054667
10 152 translational initiation
GO:0043624 1.43151305453085e-09 18.1093452002543 0.688187668673448
10 153 cellular protein complex disassembly
GO:0043241 1.95929027695537e-09 17.4914004914005 0.710677461767352
10 158 protein complex disassembly
GO:0000956 3.76806827694243e-09 16.2687249857061 0.76015500657394
10 169 nuclear-transcribed mRNA catabolic process
GO:0034623 4.99601276650178e-09 15.7671840354767 0.782644799667843
10 174 cellular macromolecular complex disassembly
GO:0006402 6.2214483141093e-09 15.3874458874459 0.800636634142966
10 178 mRNA catabolic process
GO:0032984 6.56663903908814e-09 15.2953200645508 0.805134592761747
10 179 macromolecular complex disassembly
GO:0033365 1.76727736295494e-08 8.32918962792088 2.12753442668327
14 473 protein localization to organelle
GO:0006605 1.81492378685088e-08 8.31048593350384 2.13203238530206
14 474 protein targeting
GO:0006401 2.40589720690824e-08 13.2317016317016 0.922081516850045
10 205 RNA catabolic process
GO:0019058 7.43844078156544e-08 11.6536404771699 1.03902844093834
10 231 viral infectious cycle
GO:0022415 2.24268094993652e-07 10.2807272727273 1.16946924088298
10 260 viral reproductive process
GO:0071845 5.06134324788978e-07 9.364299933643 1.27742024773372 10
284 cellular component disassembly at cellular level
GO:0022411 5.75315735175906e-07 9.22694571615435 1.29541208220884
10 288 cellular component disassembly
Tissue: Nerve_Tibial=>Heart_Left_Ventricle Type: asymmetric
SourceGene: CNTD2(ENSG00000105219.3)
TargetGeneSet: RP11 MRPL20 RPL22 DNAJC11 RPL11 EIF2C3 SZT2 BEND5
ANXA9 INSRR SRP9 UBC EIF3FP3 RPL15 HIGD1A CHMP2B RPL24 C3orf27
C3orf37 RPL35A ANAPC10 RPS3A C5orf22 BRIX1 BTF3 SKP1 SPOCK1 TMEM14B
RPS10 RPL10A RPL39P3 MRPS12 SNHG6 RPL30 EEF1D KANK1 DCTN3 FAM22G ASB6
POLR3A RPS24 RPL27A RPS13 HTATIP2 FAU RPS25 KDM5A PFDN5 OR10P1
SYCP3 RILPL1 STARD13 RNASEH2B RPS3AP6 RPLP1 ZZEF1 KIAA0100
RPL19 EIF3G RPL18A RPS16 EID2B EXOSC3P1 RGN RPS23P8 RPS4X
RAB11FIP1P1 EMD

GOBPID	Pvalue	OddsRatio	ExpCount	Count	Size	Term
GO:0006414	1.12426377239108e-34	130.857498961363	0.370562590824164	22	105	translational elongation
GO:0006614	1.40619194885409e-32	119.3	0.370562590824164	21	105	SRP-dependent cotranslational protein targeting to membrane
GO:0006613	1.75010579333706e-32	117.888235294118	0.374091758355823	21	106	cotranslational protein targeting to membrane
GO:0045047	1.75010579333706e-32	117.888235294118	0.374091758355823	21	106	protein targeting to ER
GO:0072599	1.75010579333706e-32	117.888235294118	0.374091758355823	21	106	establishment of protein localization to endoplasmic reticulum
GO:0006415	6.84356161348383e-32	130.204452521581	0.321154245380943	20	91	translational termination
GO:0070972	2.04497673012893e-31	103.217525773196	0.416441768735728	21	118	protein localization to endoplasmic reticulum
GO:0000184	2.42741266830333e-29	93.1964809384164	0.419970936267386	20	119	nuclear-transcribed mRNA catabolic process, nonsense-mediated decay
GO:0006413	6.07258898491126e-29	76.2465648854962	0.536433464812124	21	152	translational initiation
GO:0006612	7.02459986295605e-29	75.6636363636364	0.539962632343782	21	153	protein targeting to membrane
GO:0006401	7.5833112102058e-28	58.936122102883	0.723479343990035	22	205	RNA catabolic process
GO:0006402	1.97950146336038e-27	63.503821656051	0.62819182063525	21	178	mRNA catabolic process
GO:0019080	3.87415982015813e-27	70.2733316917016	0.532904297280465	20	151	viral genome expression
GO:0019083	3.87415982015813e-27	70.2733316917016	0.532904297280465	20	151	viral transcription
GO:0043624	5.11085612430377e-27	69.2068881882125	0.539962632343782	20	153	cellular protein complex disassembly
GO:0043241	1.00453714998166e-26	66.6760168302945	0.557608470002076	20	158	protein complex disassembly
GO:0072594	3.87001346254351e-26	54.3819672131148	0.719950176458377	21	204	establishment of protein localization to organelle
GO:0000956	4.10822896760333e-26	61.7059969690409	0.596429312850322	20	169	nuclear-transcribed mRNA catabolic process
GO:0034623	7.54415845132792e-26	59.6816087138668	0.614075150508615	20	174	cellular macromolecular complex disassembly
GO:0032984	1.35990178644407e-25	57.7845404747413	0.631720988166909	20	179	macromolecular complex disassembly
GO:0019058	2.58758551892479e-23	43.3848035468583	0.815237699813162	20	231	viral infectious cycle
GO:0022415	2.85800587275904e-22	38.0645161290323	0.917583558231264	20	260	viral reproductive process
GO:0071845	1.6961335005656e-21	34.5454545454545	1.00228357899107	20	284	cellular component disassembly at cellular level
GO:0022411	2.24675214330027e-21	34.0202214732788	1.01640024911771	20	288	cellular component disassembly
GO:0033365	3.47241803156095e-21	25.4642857142857	1.66929624247457	23	473	protein localization to organelle
GO:0006605	3.64251793132596e-21	25.4060025340513	1.67282541000623	23	474	protein targeting
GO:0016032	7.30222353574565e-19	22.7418604651163	1.59165455677808	21	451	viral reproduction
GO:0022613	1.30660656937396e-05	10.3496663886572	0.794062694623209	7	225	ribonucleoprotein complex biogenesis
GO:0042254	1.33451810417935e-05	13.2932400932401	0.525845962217148	6	149	ribosome biogenesis
GO:0071843	1.93101608071394e-05	9.71551724137931	0.843471040066431	7	239	cellular component biogenesis at cellular level

Tissue: Nerve_Tibial=>Heart_Left_Ventricle Type: asymmetric
SourceGene: ENSG00000215346.3

TargetGeneSet: CCNL2 NPHP4 SRRM1 KHDRBS1 PTGFR CNN3 RNPC3 PIAS3
TIPRL RAB10 NRXN1 AHSA2 PPP3R1 ANTXR1 RABL2A GALNT13 NDUFB3 STK36
COX17 ZXDC RP11 NPHP3 TMEM14E MAP3K13 METTL19 C4orf3 CPE NDUFS6
EGFLAM COX7C PPIC WDR55 HMGXB3 ATF6B NUDT3 TBCC EFHC1 MARCKS
TOMM7 MRPS24 UBE2D4 CCT6P1 SGCE FAM71F2 NDUFB2 RBM33 GFRA2 KIAA1875
IL33 C9orf123 CDK9 ANAPC2 MLLT10 RET C10orf102 USMG5
MRPL23 TMEM138 COX8A VEGFB CCL13 PCNXL3 NDUFS8 C11orf67 KIAA1731
MAML2 VPS11 FEZ1 COX14 CAPS2 GLTP ARL6IP4 LTB4R RDH11 SLIRP
NDUFB1 RAB8B LUC7L TCEB2 POLR3E CTD NDUFAB1 SETD6 SLC7A6 YWHAE
MYBBP1A VMO1 KIAA0753 NXPH3 SUMO2 C17orf89 CTDP1 CFD
UQCR11 C19orf70 CAPS C3 C19orf79 TRAPPC5 ADAMTS10
GPATCH1 USF2 COX6B1 POLR2I COX7A1 QPCTL LENG8 XRN2 ROMO1 ZMYND8
RPS21 SLC2A4RG PCP4 NDUFV3 CHCHD10 DDX17 RP1 TOMM22 TUBGCP6
PRPS2 NDUFA1
GOBPID Pvalue OddsRatio ExpCount Count Size Term
GO:0022904 2.67348502771974e-12 22.8988764044944 0.67095702719535
12 96 respiratory electron transport chain
GO:0006120 1.10616062802446e-10 44 0.251608885198256 8 36
mitochondrial electron transport, NADH to ubiquinone
GO:0022900 1.450782056823e-10 15.7244428071468 0.936544183793509
12 134 electron transport chain
GO:0006119 2.22073209059671e-10 28.5512422360248 0.40536987059719
9 58 oxidative phosphorylation
GO:0045333 2.86203743916216e-10 14.7484874675886 0.992457269393122
12 142 cellular respiration
GO:0042773 1.07918168525966e-09 31.5654811138682 0.328489377897723
8 47 ATP synthesis coupled electron transport
GO:0042775 1.07918168525966e-09 31.5654811138682 0.328489377897723
8 47 mitochondrial ATP synthesis coupled electron transport
GO:0006091 4.22139006051641e-08 6.36801827527127 2.99135007957927
16 428 generation of precursor metabolites and energy
GO:0015980 2.12874277364463e-07 7.06277056277056 2.14566465988513
13 307 energy derivation by oxidation of organic compounds
Tissue: Nerve_Tibial=>Heart_Left_Ventricle Type: asymmetric
SourceGene: ENSG00000223442.1
TargetGeneSet: ISG15 AGRN MXRA8 ZBTB48 MT1XP1 MFAP2 ATPIF1 RP11
FAM176B FTH1P1 CYP4B1 SPATA6 ALG6 HHLA3 IFI44L IFI44 TGFBR3 AMIGO1
AMPD2 INTS3 CRABP2 KIRREL GLUL CNST TRAPPC12-AS1 C2orf18 EMILIN1 XPO1
ATOH8 IL1B INHBB METAP1D ITGAV COL3A1 FN1 TMBIM1 SERPINE2
MTMR14 TIMP4 GALNTL2 TRANK1 ABHD6 SLC9C1 CCDC80 KLF15 GOLIM4 PHF17
ZDHHC11 CDH6 NREP CDX1 SPARC PDLIM7 RMND5B SCAND3 RXRB FKBP5
MAPK14 BYSL CCND3 MTO1 COL12A1 SH3BGRL2 PGM3 KIAA1009
SESN1 FAN1 SEPT7 HGF SAMD9L COL1A2 MKLN1 ADCK2 LOXL2 DRP2
PROSC MSC CRISPLD1 CTHRC1 SYBU SNTB1 OGN ASPN ANKS6
COL15A1 ZFP37 IER5L COL5A1 LCN6 ECHDC3 ZNF438 ITGB1 ARHGAP22
MICU1 IFIT1 PANK1 POLL LSP1 PRKCDBP DCHS1 ZNF214 FJX1 PRR5L
NR1H3 LRRC55 SLC43A1 TMEM223 NRXN2 DPF2 KAT5 OAF MGST1 LIMA1
TENC1 PAN2 IRAK3 LRRC10 LUM BRAP LPAR6 LINC00346 ARHGEF40
PRMT5-AS1 ZFHX2 C14orf101 ARID4A DACT1 MTA1 NIPA2 GPR176 CILP
LOXL1 FAM174B CCDC101 MT1A FOXC2 DEF8 SERPINF2 RP1 ZMYND15
ZNF594 CCR10 EZH1 ARL17B COL1A1 TMEM100 MRC2 CDC42EP4 BAIAP2
ABHD3 C19orf6 TCF3 CSNK1G2 MOB3A PLIN4 XAB2 ZNF844 ZNF564 ARRDC2
ISYNA1 ZNF233 HIF3A RCN3 VRK3 CLEC11A SSC5D ZNF274 C20orf202
CSRP2BP BCL2L1 TTI1 KIAA1755 SLCO4A1 ADAMTS5 FAM176C RUNX1 CBS
SCARF2 DRG1 C1QTNF6 NOL12 TRIOBP GTPBP1 MXRA5 RP3 TSC22D3 KIAA1210
SEPT6 NLGN4Y
GOBPID Pvalue OddsRatio ExpCount Count Size Term
GO:0030199 2.52257794560002e-11 39.3805147058824 0.331118953705626
9 33 collagen fibril organization
GO:0030198 6.52673092890722e-07 7.74706467661692 1.61545913777593
11 161 extracellular matrix organization
GO:0043062 6.94264786395083e-07 7.69521597311456 1.62549304546398
11 162 extracellular structure organization

Tissue: Heart_Left_Ventricle=>Nerve_Tibial Type: asymmetric
SourceGene: ENSG00000228404.1
TargetGeneSet: C1QA C1QC C1QB RPS6KA1 LAPTM5 BRD8 CD53 C1orf162
ADORA3 DENND4B ARHGAP30 FCER1G NPL CHIT1 FAM177B ENAH CCDC75
RAB11FIP5 VAMP8 ARID5A BUB1 SLC11A1 IRS1 ATRIP STAB1 LRIG1
GAP43 HCLS1 TM4SF1 TLR6 RBM47 CXCL13 TLR2 SAP30 KCNN2 P4HA2-AS1
ERGIC1 ATP6V0E1 MUTED GPSM3 CYB5R4 RP3 C6orf123 TWISTNB
IKZF1 MTERF GAL3ST4 TFEC TES IRF5 TBXAS1 RP11 PREX2 SLA
CDKN2A CDKN2B-AS1 GNA14 SYK FGD3 ARRDC1 ITIH5 ASCC1 SAA2-SAA4
SAA1 MS4A6A MS4A7 SLC15A3 FERMT3 PGM2L1 SLCO2B1 MYO7A GPR162 PTPN6
CD163 C3AR1 ERP27 IRAK4 BIN2 MSRB3 HSPB8 ALOX5AP RNASE6 RNASE2
SLC7A7 FMN1 ANP32A APOBR CORO1A RPH3AL CD68 ALOX15B PIK3R5 EVI2A
FMNL1 YES1 ABHD3 STXBP2 CD97 RASAL3 ZNF738 RASGRP4 SHKBP1 SIGLEC9
SIGLEC7 NLRP12 SIRPG NNAT PKIG ATP9A SLC17A9 SAMSN1 TLR8 WAS
WDR44 SASH3 MTM1 ARHGAP4
GOBPID Pvalue OddsRatio ExpCount Count Size Term
GO:0002250 1.14803998294445e-08 10.3848973607038 1.37014739464397
12 198 adaptive immune response
GO:0050778 2.00383920420658e-07 6.51209302325581 2.5188568265172 14
364 positive regulation of immune response
GO:0002460 4.84306528829036e-07 9.21377517868746 1.25250847692201
10 181 adaptive immune response based on somatic recombination of immune receptors built from immunoglobulin superfamily domains
GO:0006954 6.21981120977108e-07 5.48914330833004 3.1970105875026 15
462 inflammatory response
GO:0050900 8.47628373614584e-07 7.58822667318026 1.66770465711715
11 241 leukocyte migration
GO:0050865 4.25529148021367e-06 5.77588574567427 2.3735381634489 12
343 regulation of cell activation
GO:0002253 6.83481194313715e-06 6.0351513732834 2.06906096463913 11
299 activation of immune response
GO:0002694 1.33374702695963e-05 5.59807901413556 2.22129956404401
11 321 regulation of leukocyte activation
GO:0002443 1.53366305529808e-05 6.92748340768143 1.46010656701958
9 211 leukocyte mediated immunity
Tissue: Heart_Left_Ventricle=>Nerve_Tibial Type: cluster
SourceGene: ENSG00000232821.1
TargetGeneSet: HSPB7 PTGER3 PXDN RETSAT MOGAT1 CIDEC TIMP4 PPARG
ADIPOQ ADIPOQ-AS1 HSP90AB2P KLB RP11 ACSL1 PPARGC1B
KLHL31 SLC29A4 MLXIPL CD36 LEP PRRT4 DUSP4 FABP4 AQP9 AGPAT2
O3FAR1 SCD KCNIP2 GPAM PNPLA2 LGALS12 DGAT2 THRSP GPD1 PPP1R1A
PLIN1 ABCC6 SPIRE2 TUSC5 SCN4A FASN PLIN4 PLIN5 OLFM2 RFXANK
CEBPA LIPE RPL12L3 BLCAP PCK1 RP1 GYG2 TMEM164
GOBPID Pvalue OddsRatio ExpCount Count Size Term
GO:0006641 2.38359895737063e-18 66.367201426025 0.311950730053283 13
98 triglyceride metabolic process
GO:0006639 4.69741644196249e-18 62.6582491582492 0.327866583627431
13 103 acylglycerol metabolic process
GO:0006638 5.3570268371576e-18 61.965367965368 0.33104975434226 13
104 neutral lipid metabolic process
GO:0019432 5.83776216399773e-14 79.3912776412776 0.168708047885959
9 53 triglyceride biosynthetic process
GO:0046460 8.33727060110283e-14 75.9289071680376 0.175074389315618
9 55 neutral lipid biosynthetic process
GO:0046463 8.33727060110283e-14 75.9289071680376 0.175074389315618
9 55 acylglycerol biosynthetic process
GO:0046486 8.76471418799091e-13 22.6739344666174 0.824441215140821
13 259 glycerolipid metabolic process
GO:0045017 1.23681112604198e-09 23.5929398786542 0.49657463151339
9 156 glycerolipid biosynthetic process
GO:0044262 1.47954503401583e-07 16.1820768136558 0.614351947962079
8 193 cellular carbohydrate metabolic process
GO:0019216 1.5395511703538e-07 16.0939445387663 0.617535118676908

8	194	regulation of lipid metabolic process
GO:0006071	6.30229179542734e-07	76.1216931216931	0.0700297557262473
4	22	glycerol metabolic process
GO:0019433	7.61135957108558e-07	72.1102756892231	0.0732129264410767
4	23	triglyceride catabolic process
GO:0019400	9.11240520275404e-07	68.5	0.0763960971559062	4	24
alditol metabolic process
GO:0046461	9.11240520275404e-07	68.5	0.0763960971559062	4	24
neutral lipid catabolic process
GO:0046464	9.11240520275404e-07	68.5	0.0763960971559062	4	24
acylglycerol catabolic process
GO:0050873	9.11240520275404e-07	68.5	0.0763960971559062	4	24
brown fat cell differentiation
GO:0055089	2.50194174192626e-06	167.43023255814 0.0286485364334648	3
9	fatty acid homeostasis
GO:0046503	3.02691826860864e-06	48.9013605442177	0.101861462874542
4	32	glycerolipid catabolic process
GO:0006631	3.05314827031352e-06	10.6203007518797	0.916753165870874
8	288	fatty acid metabolic process
GO:0019751	3.88556489186654e-06	45.6349206349206	0.1082278043042 4
34	polyol metabolic process
GO:0032787	3.95504925652452e-06	8.69532542746829	1.2764514566466 9
401	monocarboxylic acid metabolic process
GO:0044242	4.46166843388477e-06	16.3442748091603	0.436094387931631
6	137	cellular lipid catabolic process
GO:0010876	4.78571076242548e-06	12.2508629191321	0.684381703688326
7	215	lipid localization
GO:0046339	4.89262644019665e-06	125.55523255814 0.0350148778631237	3
11	diacylglycerol metabolic process
GO:0050872	4.89262644019665e-06	125.55523255814 0.0350148778631237	3
11	white fat cell differentiation
GO:0055088	5.59022469380387e-06	22.9926187419769	0.257836827901183
5	81	lipid homeostasis
GO:0006637	7.55092524771267e-06	38.0132275132275	0.127326828593177
4	40	acyl-CoA metabolic process
GO:0035383	7.55092524771267e-06	38.0132275132275	0.127326828593177
4	40	thioester metabolic process
GO:0045834	1.04540248717122e-05	20.0700869077656	0.292851705764307
5	92	positive regulation of lipid metabolic process
GO:0010035	1.53444045941357e-05	8.42944969260759	1.14275828662376
8	359	response to inorganic substance
GO:0034383	1.64209495918165e-05	77.2379248658318	0.0509307314372708
3	16	low-density lipoprotein particle clearance
GO:0071285	1.64209495918165e-05	77.2379248658318	0.0509307314372708
3	16	cellular response to lithium ion
GO:0019915	1.71434037712978e-05	30.3915343915344	0.155975365026642
4	49	lipid storage
Tissue: Nerve_Tibial=>Heart_Left_Ventricle	Type:	asymmetric
SourceGene:	ENSG00000239935.1
TargetGeneSet:	DFFB	RBP7	UBE4B	HNRNPR	RP1	KHDRBS1 NDUFS5	TAL1	NRD1
RP4	FAM69A	WNT2B	BCAS2	ITGA10	C1orf54 SPRR1A	S100A4	INTS3	RP11
CRTC2	RGS5	TIPRL	CEP350	MIR4426 HHAT	LYST	SOS1	KDM3A	COX5B
RABL2A	INHBB	MAP3K2	SLC40A1 STK36	STK11IP ASB1	ING5	MRPS36P1
MTMR14	CCDC72	SNORD19B	PDHB	ROBO1	CD200R1 CCDC58	ITGB5	ABTB1
UBQLN4P1	RTP4	BCL6	ATP5I	METTL19 QDPR	FAM184B WDR19	RPL7AP31
FAM13A-AS1	TMSB4XP8	ENPEP	PDE5A	LSM6	CTSO	PTGER4	C5orf43
PIK3R1	COX7C	CTD	KIF3A	SOX4	ANKS1A	FKBP5	ETV7	PEX6	PPP2R5D NT5E
MARCKS	GTF2H5	AGPAT4	FAM120B IQCE	AGR2	DNAH11	CDK14	SGCE	ECD
ATP5J2	GFRA2	BIN3	EBF2	UBE2V2	ENY2	DENND3	IL33	GLIPR2	ZNF169
COL15A1 GRIN3A	C9orf114	USP20	CAMSAP1 RET	CXCL12	ZNF22	RHOBTB1
CALM2P2 BLNK	TM9SF3	C10orf82	POLR2L	LSP1	PHLDA2	OLFML1	ATG13
LRRN4CL COX8A	FAU	SYVN1	PRCP	FEZ1	TSPAN11 C12orf35	PKP2	TWF1
TENC1	ITGA5	NDUFA12 RPS11P5 RPS7P10 MED4	MED4-AS1	MRPL52	AP1G2

FITM1 ARF6 GNG2 MAX ERH MLH3 SLIRP NDUFB1 SLC25A29 PLDN
FBN1 SCAND2 IFT140 SEPHS2 MT1JP PSMB10 SLC7A6 DHX38 ZC3H18 INPP5K
WDR81 DPH1 MNT SPAG7 FXR2 PER1 LRRC48 EZH1 MTMR4 SLC16A6
RPL38 RECQL5 SMCHD1 ZNF397 MBD1 DPP9 SNRPEP4 CAPS GPR108 C19orf60
GPATCH1 COX6B1 POLR2I COX7A1 ZNF428 HIF3A NDUFA3 ZNF274 ZNF341 ROMO1
PTPRT BCAS4 RPS21 ATP5J URB1 NDUFV3 CHCHD10 TPST2 EIF4ENIF1
PLA2G6 TOMM22 EEF1B2P3 MAGEH1 SH3BGRL PGRMC1 NDUFA1 RP3
GOBPID Pvalue OddsRatio ExpCount Count Size Term
GO:0022904 2.38067494063953e-11 15.9031841652324 1.01640024911771
13 96 respiratory electron transport chain
GO:0045333 3.05580499993376e-10 11.1499550359712 1.50342536848661
14 142 cellular respiration
GO:0022900 1.65293176557921e-09 10.8796340023613 1.4187253477268 13
134 electron transport chain
GO:0015980 2.67741731030971e-08 6.03793103448276 3.25036329665767
17 307 energy derivation by oxidation of organic compounds
GO:0006091 1.31625266919999e-07 4.81500200707952 4.53145111064978
19 428 generation of precursor metabolites and energy
Tissue: Nerve_Tibial=>Heart_Left_Ventricle Type: asymmetric
SourceGene: ENSG00000243172.1
TargetGeneSet: RBP7 UBE4B HNRNPR RP1 KHDRBS1 NDUFS5 TAL1 BCAS2
SPRR1A S100A4 MIR4426 HHAT SOS1 KDM3A SH3RF3 RABL2A INHBB NDUFB3 ING5
MTMR14 PDHB ROBO1 CCDC58 UBQLN4P1 RP11 BCL6 ATP5I METTL19 QDPR
FAM184B COX7C CTD ADAMTS19 RP3 ETV7 USP49 PPP2R5D MARCKS IQCE
AGR2 DNAH11 GFRA2 EBF2 GLIPR2 COL15A1 C9orf114 USP20 CAMSAP1 RET
CXCL12 CALM2P2 BLNK PHLDA2 ATG13 OR4C3 COX8A SYVN1 UCP2 PRCP
TSPAN11 PKP2 ITGA5 RIC8B RPS11P5 RPS7P10 MRPL52 ARF6 U3 MAX
SLIRP NDUFB1 FBN1 SCAND2 IFT140 POLR3E MT1JP PSMB10 DHX38 ZC3H18
INPP5K MNT SPAG7 LRRC48 EZH1 MTMR4 SLC16A6 SMCHD1 ZNF397 DPP9
SNRPEP4 CAPS C19orf60 GPATCH1 COX6B1 NDUFA3 ZNF274 ROMO1 BCAS4
RPS21 ATP5J CHCHD10 PLA2G6 TOMM22 MAGEH1 HDAC8 RP13 PGRMC1 NDUFA1
PNMA6C
GOBPID Pvalue OddsRatio ExpCount Count Size Term
GO:0022904 6.72920021658114e-12 25.6720588235294 0.551380527299149
11 96 respiratory electron transport chain
GO:0045333 2.70262888587389e-11 18.5109425785482 0.815583696629991
12 142 cellular respiration
GO:0022900 2.63392577042806e-10 17.6936540198735 0.769635319355062
11 134 electron transport chain
GO:0015980 2.01036838139178e-09 9.7467477865163 1.7632689779254 14 307
energy derivation by oxidation of organic compounds
GO:0006091 1.7873964604507e-08 7.45353226036177 2.45823818420871
15 428 generation of precursor metabolites and energy
GO:0006119 9.53058109375238e-07 21.4525474525475 0.333125735243236
6 58 oxidative phosphorylation
GO:0006120 1.81379446212975e-06 29.6464019851117 0.206767697737181
5 36 mitochondrial electron transport, NADH to ubiquinone
GO:0042773 7.02387280262989e-06 21.8650793650794 0.269946716490208
5 47 ATP synthesis coupled electron transport
GO:0042775 7.02387280262989e-06 21.8650793650794 0.269946716490208
5 47 mitochondrial ATP synthesis coupled electron transport
Tissue: Heart_Left_Ventricle=>Nerve_Tibial Type: asymmetric
SourceGene: ENSG00000258953.2
TargetGeneSet: C1QC RPS6KA1 ADORA3 RP11 SLAMF8 ARHGAP30 FCER1G
TRMT1L TLR5 VAMP8 SLC4A3 CCR2 HCLS1 KLHL7 PDE6B SLC2A9 RBM47 ARSJ
RP13 SLC1A3 GNPDA1 LCP2 HLA-DRB5 FGD2 ZC3H12D TFEC IRF5
TBXAS1 SLA SYK ENG SFMBT2 ASCC1 VENTX MS4A6A MS4A4E MS4A7
CCDC88B SLCO2B1 LRRC32 SLC37A2 C11orf45 PTPN6 CD163 C3AR1 IRAK4
RNASE6 SLC7A7 FMN1 PMFBP1 BCAR1 HIC1 ZFP3 ALOX15B PIK3R5 AKAP10
STXBP2 ZNF812 NUDT19 NR1H2 SIGLEC9 SIGLEC7 CD33 SIGLEC10 CTD
LILRB2 HCK TLR8 WAS BTK SASH3 ARHGAP4
GOBPID Pvalue OddsRatio ExpCount Count Size Term
GO:0050778 2.48504833504871e-11 12.8391147960739 1.56169123244066

15	364	positive regulation of immune response
GO:0002764	5.05636428672103e-10	14.3928813559322	1.06400941111342
12	248	immune response-regulating signaling pathway
GO:0002757	4.07884376650866e-09	13.6392682072829	1.00823472424054
11	235	immune response-activating signal transduction
GO:0002253	4.23671184581676e-09	11.7926132404181	1.28281779807626
12	299	activation of immune response
GO:0001816	9.6346617077943e-09	9.72812266267764	1.69469240882984
13	395	cytokine production
GO:0002250	1.53746943109868e-07	12.7583108715184	0.849491384679261
9	198	adaptive immune response
GO:0002768	1.71706727782569e-07	15.5261437908497	0.617811916130372
8	144	immune response-regulating cell surface receptor signaling pathway
GO:0050865	1.9773336970043e-07	9.1322348216395 1.47159366133832	11
343	regulation of cell activation
GO:0002443	2.63990345932064e-07	11.9263030076593	0.905266071552142
9	211	leukocyte mediated immunity
GO:0001817	3.12044145533633e-07	8.70244534595447	1.54023942979725
11	359	regulation of cytokine production
GO:0043299	5.55191241907973e-07	38.160552897395 0.163033700089959	5
38	leukocyte degranulation
GO:0050867	7.80228360848356e-07	10.407743200196 1.02968652688395	9
240	positive regulation of cell activation
GO:0002694	9.82156060142484e-07	8.7051694286421 1.37720572970729	10
321	regulation of leukocyte activation
GO:0002460	9.85128122379462e-07	12.1738385784629	0.776555255691648
8	181	adaptive immune response based on somatic recombination of immune receptors built from immunoglobulin superfamily domains
GO:0045055	1.17774441339446e-06	32.2762033288349	0.188775863262058
5	44	regulated secretory pathway
GO:0002429	1.54053548353115e-06	14.4070707070707	0.570617950314857
7	133	immune response-activating cell surface receptor signaling pathway
GO:0043300	1.74037131440556e-06	58.3042596348884	0.0900975711023459
4	21	regulation of leukocyte degranulation
GO:0002886	2.55844426015257e-06	52.1597096188748	0.0986782921597121
4	23	regulation of myeloid leukocyte mediated immunity
GO:0002703	2.60592313300984e-06	17.8193521594684	0.394713168638849
6	92	regulation of leukocyte mediated immunity
GO:0042129	4.49497462692755e-06	16.1210526315789	0.433326413396997
6	101	regulation of T cell proliferation
GO:0002252	5.12654125636125e-06	7.16704173486088	1.65607916407169
10	386	immune effector process
GO:0002696	6.46907816332389e-06	9.32609053497942	0.999654003183171
8	233	positive regulation of leukocyte activation
GO:0042098	1.10449759516756e-05	13.6578443877551	0.50626254238461
6	118	T cell proliferation
GO:0002283	1.21088113765642e-05	91.4046610169491	0.0471939658155145
3	11	neutrophil activation involved in immune response
GO:0050851	1.40087942779846e-05	13.0695970695971	0.527714345028026
6	123	antigen receptor-mediated signaling pathway
GO:0045576	1.45564056343773e-05	31.9421579532814	0.15016261850391
4	35	mast cell activation
Tissue: Nerve_Tibial=>Heart_Left_Ventricle	Type:	asymmetric
SourceGene:	FAM132A(ENSG00000184163.3)
TargetGeneSet:	DNAJC11 RP11	SLC39A1 LRRC71	TNFAIP6 BTF3L4P2	RPL15	LNP1
OSBPL11 PPP1R2	LETM1	SLC2A9	RPS3A	NDUFAF2 SKP1	MATR3	RPS14	TMEM14C RDBP
RXRB	RPS10	RPL10A	RPL39P3 MTO1	ELMO1	ZNF706	SYBU	RPL8	CHMP5
FRMPD1	RPL35	TRMT112 SNX32	CCDC59	RILPL1	ZNF605	ESR2	RPS3AP6 BCL7C
MIR4519 SLC12A4 RPL26	RPL19	KCNH4	BAIAP2	LAMA3	CABYR	GPX4	RPS15
MOB3A	RPL18A	ZNF790	ZNF585B OPA3	SNRPD2	SIGLEC10	UBE2S	CTD
SLC5A1	XRCC6	RAB11FIP1P1
GOBPID	Pvalue	OddsRatio	ExpCount	Count	Size	Term
GO:0006415	3.68790031645846e-15	56.2767857142857	0.289668535049478

11	91	translational termination
GO:0006414	1.88173460707377e-14	47.8483282674772	0.334232925057089
11	105	translational elongation
GO:0006614	1.88173460707377e-14	47.8483282674772	0.334232925057089
11	105	SRP-dependent cotranslational protein targeting to membrane
GO:0006613	2.09493349418219e-14	47.3413533834586	0.337416095771919
11	106	cotranslational protein targeting to membrane
GO:0045047	2.09493349418219e-14	47.3413533834586	0.337416095771919
11	106	protein targeting to ER
GO:0072599	2.09493349418219e-14	47.3413533834586	0.337416095771919
11	106	establishment of protein localization to endoplasmic reticulum
GO:0019080	3.12135750018348e-14	36.2234447735929	0.480658777939243
12	151	viral genome expression
GO:0019083	3.12135750018348e-14	36.2234447735929	0.480658777939243
12	151	viral transcription
GO:0070972	7.01974491092106e-14	41.9967957276369	0.375614144349872
11	118	protein localization to endoplasmic reticulum
GO:0000184	7.71744494046882e-14	41.6050264550265	0.378797315064701
11	119	nuclear-transcribed mRNA catabolic process, nonsense-mediated decay
GO:0019058	2.02553255535598e-13	25.6367806505421	0.735312435125597
13	231	viral infectious cycle
GO:0022415	9.20617414433382e-13	22.5805422647528	0.82762438585565
13	260	viral reproductive process
GO:0006413	1.17672574858471e-12	31.7941236068896	0.483841948654072
11	152	translational initiation
GO:0006612	1.26504439078283e-12	31.5680080482897	0.487025119368902
11	153	protein targeting to membrane
GO:0043624	1.26504439078283e-12	31.5680080482897	0.487025119368902
11	153	cellular protein complex disassembly
GO:0043241	1.8031805503896e-12	30.4835762876579	0.502940972943049
11	158	protein complex disassembly
GO:0000956	3.77697590408088e-12	28.3394213381555	0.537955850806173
11	169	nuclear-transcribed mRNA catabolic process
GO:0016032	4.34289999368037e-12	15.5027374963007	1.43560999238807
15	451	viral reproduction
GO:0034623	5.19756755499635e-12	27.4604732690622	0.55387170438032
11	174	cellular macromolecular complex disassembly
GO:0006402	6.66341867427501e-12	26.7952095808383	0.566604387239637
11	178	mRNA catabolic process
GO:0032984	7.08381264271001e-12	26.633843537415 0.569787557954467	11
179	macromolecular complex disassembly
GO:0072594	2.9338328188775e-11	23.1431532198372	0.649366825825202
11	204	establishment of protein localization to organelle
GO:0006401	3.09323206622306e-11	23.0222385861561	0.652549996540032
11	205	RNA catabolic process
GO:0071845	1.00890170914966e-09	16.269178440607 0.904020483011556	11
284	cellular component disassembly at cellular level
GO:0022411	1.16935669744987e-09	16.0297060340382	0.916753165870874
11	288	cellular component disassembly
GO:0033365	1.94678745871024e-07	9.48503401360544	1.50563974811432
11	473	protein localization to organelle
GO:0006605	1.98851357911984e-07	9.46386917618019	1.50882291882915
11	474	protein targeting
Tissue: Nerve_Tibial=>Heart_Left_Ventricle	Type:	asymmetric
SourceGene:	NDP(ENSG00000124479.8)
TargetGeneSet:	LCE1B	SPRR1B	LOR	RASGRP3 ATP13A5-AS1	RP11	C8orf40
RPL35	TTC40	ALDH3B2 TRIM29	KRT2	KRT1	BRF1	KRT15	KRT14	CD300E	SBSN
NCOA3	MT
GOBPID	Pvalue	OddsRatio	ExpCount	Count	Size	Term
GO:0008544	5.18751359582003e-11	65.1153184165232	0.266763545775379
8	257	epidermis development
GO:0030216	2.98334499526174e-08	80.6011235955056	0.0975711023458584
5	94	keratinocyte differentiation

GO:0009913 6.59063634319035e-08 68.2428571428571 0.114178949553664
5 110 epidermal cell differentiation
GO:0031424 8.21897370340797e-08 137.77990430622 0.0435955989204899 4
42 keratinization
GO:0030855 2.26069284363087e-07 34.2028985507246 0.292713307037575
6 282 epithelial cell differentiation
Tissue: Heart_Left_Ventricle=>Nerve_Tibial Type: asymmetric
SourceGene: NLGN4Y(ENSG00000165246.7)
TargetGeneSet: ZNF684 RPS8 ITGB3BP RP11 RPL5 TLR5 C2orf43 LTBP1 FEZ2
C2orf40 CXCR2P1 BCS1L RPL14 CTNNB1 NME6 RPL29 CPOX RPS3A BRD9 ANKH
CTD LYRM7 NDST1 RPL10A LRRC1 MALSU1 SGCE RP4 DYNLL1 RPS6
MICU1 OAT TUT1 KLRD1 LETMD1 RPL18AP3 LINC00327 CARS2
OSTBETA RPS2 RPS15A LYRM1 WDR81 ALOX15B MYO19 HAP1 TMEM101 CDK5RAP3
TMC6 RPL17 ZNF44 RLN3 LPAR2 ZC3H4 RPL18 RPL13A RPS9 RPS5 ESF1
CXADR TUBGCP6
GOBPID Pvalue OddsRatio ExpCount Count Size Term
GO:0006415 1.10615651250552e-20 81.4090909090909 0.289668535049478
14 91 translational termination
GO:0006414 9.22812056959534e-20 68.8173076923077 0.334232925057089
14 105 translational elongation
GO:0006614 9.22812056959534e-20 68.8173076923077 0.334232925057089
14 105 SRP-dependent cotranslational protein targeting to membrane
GO:0006613 1.06102922106085e-19 68.0645380434783 0.337416095771919
14 106 cotranslational protein targeting to membrane
GO:0045047 1.06102922106085e-19 68.0645380434783 0.337416095771919
14 106 protein targeting to ER
GO:0072599 1.06102922106085e-19 68.0645380434783 0.337416095771919
14 106 establishment of protein localization to endoplasmic reticulum
GO:0070972 5.10763590601569e-19 60.1604567307692 0.375614144349872
14 118 protein localization to endoplasmic reticulum
GO:0000184 5.77660916999985e-19 59.5833333333333 0.378797315064701
14 119 nuclear-transcribed mRNA catabolic process, nonsense-mediated decay
GO:0019080 1.80407109799649e-17 45.5638686131387 0.480658777939243
14 151 viral genome expression
GO:0019083 1.80407109799649e-17 45.5638686131387 0.480658777939243
14 151 viral transcription
GO:0006413 1.98295052106258e-17 45.2305253623188 0.483841948654072
14 152 translational initiation
GO:0006612 2.17812191695451e-17 44.9019784172662 0.487025119368902
14 153 protein targeting to membrane
GO:0043624 2.17812191695451e-17 44.9019784172662 0.487025119368902
14 153 cellular protein complex disassembly
GO:0043241 3.44944755146313e-17 43.3276909722222 0.502940972943049
14 158 protein complex disassembly
GO:0000956 8.99965785951009e-17 40.2217741935484 0.537955850806173
14 169 nuclear-transcribed mRNA catabolic process
GO:0034623 1.36163269298018e-16 38.951171875 0.55387170438032 14
174 cellular macromolecular complex disassembly
GO:0006402 1.87932361720925e-16 37.9904725609756 0.566604387239637
14 178 mRNA catabolic process
GO:0032984 2.03452495739994e-16 37.7575757575758 0.569787557954467
14 179 macromolecular complex disassembly
GO:0072594 1.28541013487958e-15 32.7319078947368 0.649366825825202
14 204 establishment of protein localization to organelle
GO:0006401 1.37674422062526e-15 32.5582460732984 0.652549996540032
14 205 RNA catabolic process
GO:0071845 5.1628724778575e-15 25.4275092936803 0.904020483011556
15 284 cellular component disassembly at cellular level
GO:0022411 6.34974776454939e-15 25.047855370436 0.916753165870874 15
288 cellular component disassembly
GO:0019058 7.30900396665305e-15 28.6048387096774 0.735312435125597
14 231 viral infectious cycle
GO:0022415 3.76975017475305e-14 25.1811483739837 0.82762438585565

14	260	viral reproductive process
GO:0016032	6.55042999470477e-11	13.9839816933638	1.43560999238807
14	451	viral reproduction
GO:0033365	1.22949274315806e-10	13.2927559912854	1.50563974811432
14	473	protein localization to organelle
GO:0006605	1.26422343196093e-10	13.2629076086957	1.50882291882915
14	474	protein targeting
GO:0042273	6.50895150443359e-06	111.596899224806	0.0381980485779531
3	12	ribosomal large subunit biogenesis
Tissue: Heart_Left_Ventricle=>Nerve_Tibial	Type:	asymmetric
SourceGene:	RP1-64K7.4(ENSG00000229472.1)
TargetGeneSet:	C1QA	C1QC	C1QB	RUNX3	C1orf38	LAPTM5	BRD8	CD2
TBX15	LYSMD1	RP11	SLAMF6	CD84	CD48	RGS1	PTPRC	TLR5	PPM1B
IGKV2-28		DUSP2	ZAP70	RNF149	LAP3	CD28	TRAT1	HCLS1	FNDC3B
KLHL7	SH3TC1	DTHD1	DAPP1	BBS12	TLR2	IL7R	FYB	EMB	GZMK	GZMA
C5orf56	HAVCR2	RPS15AP18		STK38	FGD2	PRDM1	WASF1	MAN1A1	CTGF
CARD11	AOAH	IKZF1	TFEC	IRF5	TBXAS1	C8orf80	SLA	DOCK8	AQP7P4	FGD3
SLC31A2	AKNA	MRC1	PRF1	FIBIN	MS4A6A	MS4A4E	MS4A7	CCDC88B	TBC1D10C
MYO7A	IL10RA	CD3E	LPAR5	CD4	CD163	CD69	SAT2	ARHGAP9	SELPLG
ALOX5AP	GPR183	RNASE6	U6	RIN3	FMN1	PSTPIP1	SPN	RNF166	ARRB2
ACAP1	GUCY2D	TMEM106A		HMHA1	LINGO3	EBI3	STXBP2	RASAL3	IL12RB1	HCST
TGFB1	C5AR1	CD37	SIGLEC11		CD33	HCK	STK4	CASS4	ZNF831
RGS19	ITGB2	CECR1	NCF4	MKL1	WAS	IL2RG	BTK	TSC22D3	MTCP1
GOBPID	Pvalue	OddsRatio	ExpCount	Count	Size	Term
GO:0050867	6.16703954911104e-13	12.9761737533092	1.66078472078057
17	240	positive regulation of cell activation
GO:0002696	5.04670518126635e-12	12.4064077243801	1.61234516642447
16	233	positive regulation of leukocyte activation
GO:0050778	5.41880613303461e-12	9.52277688316336	2.5188568265172	19
364	positive regulation of immune response
GO:0030098	9.28197829187571e-12	13.2231559290383	1.41166701266348
15	204	lymphocyte differentiation
GO:0002252	1.50708337585715e-11	8.93786793151008	2.67109542592208
19	386	immune effector process
GO:0050865	1.91997384763899e-11	9.47347091932458	2.3735381634489	18
343	regulation of cell activation
GO:0051251	1.97286924531344e-11	12.4861764705882	1.48778631236593
15	215	positive regulation of lymphocyte activation
GO:0002694	6.42392528192572e-11	9.46413284717819	2.22129956404401
17	321	regulation of leukocyte activation
GO:0051249	9.43043132679043e-11	10.0474406991261	1.95834198325375
16	283	regulation of lymphocyte activation
GO:0002697	9.96774978837845e-11	12.3303071757244	1.39090720365373
14	201	regulation of immune effector process
GO:0002253	2.1294807690829e-10	9.46861854282349	2.06906096463913
16	299	activation of immune response
GO:0002521	2.4671329272346e-10	9.36729936729937	2.08982077364888
16	302	leukocyte differentiation
GO:0002703	7.17720097508706e-10	19.3346883468835	0.636634142965885
10	92	regulation of leukocyte mediated immunity
GO:0030217	7.58713217794386e-10	13.4535984848485	1.07951006850737
12	156	T cell differentiation
GO:0046649	8.54058368578435e-10	7.37137819570967	2.99633243374161
18	433	lymphocyte activation
GO:0002449	1.66273990593222e-09	12.4891495601173	1.15562936820981
12	167	lymphocyte mediated immunity
GO:0002706	1.69853349330778e-09	22.078125	0.505155352570756	9
73	regulation of lymphocyte mediated immunity
GO:0002443	2.20446041873009e-09	10.6808893533031	1.46010656701958
13	211	leukocyte mediated immunity
GO:0046651	2.3307192597608e-09	12.0946022727273	1.19022904989274
12	172	lymphocyte proliferation
GO:0030097	2.47490834918128e-09	6.85963277610304	3.20393052383918

18	463	hemopoiesis
GO:0032943	2.65963372781222e-09	11.9436026936027	1.20406892256591
12	174	mononuclear cell proliferation
GO:0050863	3.87037939776661e-09	10.1602011494253	1.52930593038544
13	221	regulation of T cell activation
GO:0050670	4.34613577562348e-09	13.2126383374166	0.996470832468341
11	144	regulation of lymphocyte proliferation
GO:0070661	4.43571420482504e-09	11.3751336898396	1.2594284132586 12
182	leukocyte proliferation
GO:0032944	4.67626092337837e-09	13.1131142042596	1.00339076880493
11	145	regulation of mononuclear cell proliferation
GO:0042110	4.83737255784548e-09	8.15421826625387	2.20745969137084
15	319	T cell activation
GO:0070663	6.68777336065435e-09	12.6369735672136	1.03799045048786
11	150	regulation of leukocyte proliferation
GO:0048534	7.05979434556716e-09	6.38472158306489	3.42536848660992
18	495	hemopoietic or lymphoid organ development
GO:0002757	8.1218685742489e-09	9.51004452728591	1.62618503909764
13	235	immune response-activating signal transduction
GO:0002250	1.14803998294445e-08	10.3848973607038	1.37014739464397
12	198	adaptive immune response
GO:0045058	1.14820459955949e-08	50.8262411347518	0.166078472078057
6	24	T cell selection
GO:0002764	1.54722792979759e-08	8.97569087796527	1.71614421147325
13	248	immune response-regulating signaling pathway
GO:0042102	1.63340291382369e-08	21.4287856071964	0.456715798214656
8	66	positive regulation of T cell proliferation
GO:0050671	1.99022653682482e-08	16.2152330428192	0.664313888312227
9	96	positive regulation of lymphocyte proliferation
GO:0032946	2.18132883609966e-08	16.0298451548452	0.671233824648813
9	97	positive regulation of mononuclear cell proliferation
GO:0050870	2.62938137882742e-08	10.9621488764045	1.18330911355616
11	171	positive regulation of T cell activation
GO:0002429	2.65982143388681e-08	12.8527551942186	0.920351532765899
10	133	immune response-activating cell surface receptor signaling pathway
GO:0070665	2.85428414469622e-08	15.498128245381 0.69199363365857	9
100	positive regulation of leukocyte proliferation
GO:0042129	3.11573362566166e-08	15.3285953177258	0.698913569995156
9	101	regulation of T cell proliferation
GO:0002460	4.72833113101083e-08	10.3100462656973	1.25250847692201
11	181	adaptive immune response based on somatic recombination of immune receptors built from immunoglobulin superfamily domains
GO:0002768	5.68250300970559e-08	11.7885572139303	0.996470832468341
10	144	immune response-regulating cell surface receptor signaling pathway
GO:0045061	8.31079430657567e-08	62.890350877193 0.117638917721957	5
17	thymic T cell selection
GO:0006917	8.31237658421176e-08	6.48808049535604	2.73337485295135
15	395	induction of apoptosis
GO:0050871	8.33834134099843e-08	22.9073438572409	0.373676562175628
7	54	positive regulation of B cell activation
GO:0012502	9.17451499795423e-08	6.43587774535402	2.75413466196111
15	398	induction of programmed cell death
GO:0042113	1.00907522818003e-07	11.039627039627 1.05875025949761	10
153	B cell activation
GO:0042098	1.2109996796369e-07	12.9224720233895	0.816552487717113
9	118	T cell proliferation
GO:0042108	1.56154634860335e-07	20.6974772539289	0.408276243858556
7	59	positive regulation of cytokine biosynthetic process
GO:0050851	1.73242213071227e-07	12.3513591671486	0.851152169400042
9	123	antigen receptor-mediated signaling pathway
GO:0045619	4.03748991580462e-07	13.6263736263736	0.685073697321985
8	99	regulation of lymphocyte differentiation
GO:0050852	4.03748991580462e-07	13.6263736263736	0.685073697321985

8	99	T cell receptor signaling pathway
GO:0045060	6.86340650750316e-07	85.3809523809524	0.0761192997024427
4	11	negative thymic T cell selection
GO:0001909	8.05595548383086e-07	22.2781525687597	0.325237007819528
6	47	leukocyte mediated cytotoxicity
GO:0050900	8.47628373614584e-07	7.58822667318026	1.66770465711715
11	241	leukocyte migration
GO:0043383	1.02404447386817e-06	74.703125	0.0830392360390284	4
12	negative T cell selection
GO:0002822	1.29170712742541e-06	14.7217557814111	0.553594906926856
7	80	regulation of adaptive immune response based on somatic recombination of immune receptors built from immunoglobulin superfamily domains
GO:0050864	1.40594890246263e-06	14.5217959895379	0.560514843263442
7	81	regulation of B cell activation
GO:0002704	1.4713225342121e-06	66.3981481481482	0.0899591723756141
4	13	negative regulation of leukocyte mediated immunity
GO:0002707	1.4713225342121e-06	66.3981481481482	0.0899591723756141
4	13	negative regulation of lymphocyte mediated immunity
GO:0045086	1.4713225342121e-06	66.3981481481482	0.0899591723756141
4	13	positive regulation of interleukin-2 biosynthetic process
GO:0001910	1.78219897861988e-06	30.16	0.207598090097571	5	30
regulation of leukocyte mediated cytotoxicity
GO:0006959	1.99315232100243e-06	10.859649122807	0.844232233063456	8
122	humoral immune response
GO:0046641	2.04891780529454e-06	59.7541666666667	0.0968791087121998
4	14	positive regulation of alpha-beta T cell proliferation
GO:0046634	2.08004396836927e-06	18.6304819800261	0.380596498512214
6	55	regulation of alpha-beta T cell activation
GO:0002819	2.28410014652837e-06	13.4270161290323	0.602034461282956
7	87	regulation of adaptive immune response
GO:0042035	3.0933376075761e-06	12.7840501792115	0.629714206629299
7	91	regulation of cytokine biosynthetic process
GO:0001911	3.18327162209952e-06	221.89175257732	0.0345996816829285	3
5	negative regulation of leukocyte mediated cytotoxicity
GO:0033077	3.85292758948487e-06	16.5911025145068	0.422116116531728
6	61	T cell differentiation in thymus
GO:0045621	4.2420269848367e-06	16.2936930091185	0.429036052868314
6	62	positive regulation of lymphocyte differentiation
GO:0002699	4.42872181967054e-06	12.0616165277274	0.664313888312227
7	96	positive regulation of immune effector process
GO:0031341	4.5622148997105e-06	24.3123938879457	0.249117708117085
5	36	regulation of cell killing
GO:0042089	5.81529802683322e-06	11.5395999537519	0.69199363365857
7	100	cytokine biosynthetic process
GO:0046640	6.1315496243756e-06	42.6696428571429	0.124558854058543
4	18	regulation of alpha-beta T cell proliferation
GO:0043900	6.21326030865359e-06	11.4160375200183	0.698913569995156
7	101	regulation of multi-organism process
GO:0031342	6.33450389734255e-06	147.917525773196	0.0415196180195142
3	6	negative regulation of cell killing
GO:0042107	6.63361502968287e-06	11.2950764006791	0.705833506331742
7	102	cytokine metabolic process
GO:0001817	6.77334646325785e-06	5.50327482315955	2.48425714483427
12	359	regulation of cytokine production
GO:0045076	7.72543885785311e-06	39.8222222222222	0.131478790395128
4	19	regulation of interleukin-2 biosynthetic process
GO:0001906	7.95559633014956e-06	14.4761904761905	0.477475607224414
6	69	cell killing
GO:0046635	1.12109039081268e-05	19.8240997229917	0.297557262473185
5	43	positive regulation of alpha-beta T cell activation
GO:0042094	1.18028222758029e-05	35.1323529411765	0.1453186630683 4
21	interleukin-2 biosynthetic process
GO:0046633	1.18028222758029e-05	35.1323529411765	0.1453186630683 4

21	alpha-beta T cell proliferation
GO:0051607	1.30398546158758e-05	10.1151349158044	0.781952806034184
7	113	defense response to virus
GO:0002831	1.39536464786824e-05	13.022188449848	0.525915161580513	6
76	regulation of response to biotic stimulus
GO:0002698	1.57045022338153e-05	18.3697047496791	0.318317071482942
5	46	negative regulation of immune effector process
GO:0050777	1.74781816011287e-05	17.9310776942356	0.325237007819528
5	47	negative regulation of immune response
GO:0001816	1.7671017577208e-05	4.97317825777356	2.73337485295135
12	395	cytokine production
Tissue: Heart_Left_Ventricle=>Nerve_Tibial	Type:	asymmetric
SourceGene:	RP11-598F7.6(ENSG00000256540.1)
TargetGeneSet:	DFFB	SNHG3	RPS8	RPL5	RTCD1	NBPF12	RPS27	LHX4
ANGEL2	TP53BP2	LTBP1	PRKD3	PMS1	RPE	CXCR2P1	STRA13	RPL29	ARHGAP31
IFT122	DNAJC19	RP11	CRYGS	EEF1A1P9	RPS3A	BRD9	ADAMTS16	CTD
SERINC5	NDST1	RPL15P3	RPS10	RPL10A	RP3	CREB5	CTB	FASTK	FDFT1
SULF1	COL14A1	SHARPIN	DNAJB5	NR4A3	DPP7	SSNA1	FAM35B	ASAH2B	MICU1
RPS3AP5	MGEA5	OAT	FAM160A2	ZBED5	TUT1	ARF3	LETMD1	RPL18AP3
PPTC7	RPLP0	RPSAP54	PARP4-AS1	HNRNPA1L2	C14orf28	POLR2M
RASL12	NUBP2	RPS2	TNRC6A	ZNF23	RPL13	TMUB2	CDK5RAP3	EPX	HGS
RP13	MAPRE2	RPL17	ZNF44	LPAR2	RPSAP58	HAMP	HAUS5	U2AF1L4	PHLDB3
RPL18	RPL13A	NAPSA	ZNF761	RPS5	RPL41P1	RPL12P4	LINC00478	YDJC
FAM3C2
GOBPID	Pvalue	OddsRatio	ExpCount	Count	Size	Term
GO:0006415	4.06302085636613e-18	49.0806174957118	0.42190851844163
14	91	translational termination
GO:0006414	3.32537700549594e-17	41.489114658926	0.486817521278804	14
105	translational elongation
GO:0006614	3.32537700549594e-17	41.489114658926	0.486817521278804	14
105	SRP-dependent cotranslational protein targeting to membrane
GO:0006613	3.81822569968961e-17	41.0352748154225	0.491453878624317
14	106	cotranslational protein targeting to membrane
GO:0045047	3.81822569968961e-17	41.0352748154225	0.491453878624317
14	106	protein targeting to ER
GO:0072599	3.81822569968961e-17	41.0352748154225	0.491453878624317
14	106	establishment of protein localization to endoplasmic reticulum
GO:0070972	1.80813913102758e-16	36.2699564586357	0.547090166770466
14	118	protein localization to endoplasmic reticulum
GO:0000184	2.04216684217347e-16	35.9220125786164	0.551726524115978
14	119	nuclear-transcribed mRNA catabolic process, nonsense-mediated decay
GO:0006612	2.52622870824264e-16	29.7784280936455	0.7093626738634	15
153	protein targeting to membrane
GO:0043241	4.11738341002713e-16	28.727138246369	0.732544460590963	15
158	protein complex disassembly
GO:0032984	2.70651444135387e-15	25.0117260787992	0.829907964846723
15	179	macromolecular complex disassembly
GO:0019080	6.10466740217526e-15	27.4697700041317	0.700089959172376
14	151	viral genome expression
GO:0019083	6.10466740217526e-15	27.4697700041317	0.700089959172376
14	151	viral transcription
GO:0006413	6.70078678434336e-15	27.2687995624829	0.704726316517888
14	152	translational initiation
GO:0043624	7.35024214253945e-15	27.0707207818651	0.7093626738634	14
153	cellular protein complex disassembly
GO:0022411	1.06073992363403e-14	17.7063468634686	1.33527091550758
17	288	cellular component disassembly
GO:0072594	1.90503835490424e-14	21.6651404151404	0.945816898484534
15	204	establishment of protein localization to organelle
GO:0000956	2.97119394913301e-14	24.2490566037736	0.783544391391599
14	169	nuclear-transcribed mRNA catabolic process
GO:0034623	4.46468179576022e-14	23.4830188679245	0.806726178119161
14	174	cellular macromolecular complex disassembly

GO:0006402	6.12847100379525e-14	22.9038196042338	0.825271607501211
14	178	mRNA catabolic process
GO:0071845	1.48462342162118e-13	16.5244366403278	1.31672548612553
16	284	cellular component disassembly at cellular level
GO:0006401	4.32642861684128e-13	19.6287661760348	0.950453255830046
14	205	RNA catabolic process
GO:0019058	2.21635904397534e-12	17.2452830188679	1.07099854681337
14	231	viral infectious cycle
GO:0022415	1.09848634987544e-11	15.1811627550238	1.20545290983323
14	260	viral reproductive process
GO:0006605	3.36150423800675e-10	9.53917287438993	2.19763338177289
16	474	protein targeting
GO:0033365	3.07755665728302e-09	8.77099428955324	2.19299702442738
15	473	protein localization to organelle
GO:0016032	1.46677606973516e-08	8.43046500582876	2.0909971628261 14
451	viral reproduction
Tissue: Nerve_Tibial=>Heart_Left_Ventricle	Type:	asymmetric
SourceGene:	U2AF1L4(ENSG00000161265.6)
TargetGeneSet:	MRPL20	UBR4	USP24	WARS2	RPS7	UBC	TXNDC9	SEC13
HIGD1A	RPL24	FAM162A	BCL6	HMGB2	RP11	ROPN1L	SUB1	TBCA	APC
HINT1	NDFIP1	IP6K3	RPL10A	MRPL32	HMGN3	C6orf225	RP1	SEC61G
DNAJB9	RPS20	RPL30	C9orf131	PHF2	RC3H2	ASB6	POLR3A	RPL13AP5
PRDM11	CELF1	C11orf10	AHNAK	MTA2	OTUB1	IGSF9B	RPL18AP3
C12orf45	ARPC3	RPL6	MED13L	MED4	DAD1	SCFD1	CDC42BPB
RPS3AP6	RPL4	SEC11A	RPS15A	CYB5B	RPS7P1	KIAA0100	COPZ2	RPL17
RPL18A	COPE	COX6B1	PAK4	AP2S1	ATP5E	MRPL40	TCF20	PRDX4	UXT
MORF4L2	MCTS1
GOBPID	Pvalue	OddsRatio	ExpCount	Count	Size	Term
GO:0006614	3.60079167193237e-16	42.0910326086956	0.443221922358314
13	105	SRP-dependent cotranslational protein targeting to membrane
GO:0006613	4.09140166898113e-16	41.6355286738351	0.447443083523632
13	106	cotranslational protein targeting to membrane
GO:0045047	4.09140166898113e-16	41.6355286738351	0.447443083523632
13	106	protein targeting to ER
GO:0072599	4.09140166898113e-16	41.6355286738351	0.447443083523632
13	106	establishment of protein localization to endoplasmic reticulum
GO:0070972	1.72313424537865e-15	36.8462301587302	0.498097017507439
13	118	protein localization to endoplasmic reticulum
GO:0006612	5.31689166446401e-14	27.5669642857143	0.645837658293544
13	153	protein targeting to membrane
GO:0006415	1.07069485258902e-13	39.3525 0.384125666043872	11	91
translational termination
GO:0006414	5.39047585870216e-13	33.4587234042553	0.443221922358314
11	105	translational elongation
GO:0043624	1.43091558368718e-12	24.748588797221 0.645837658293544	12
153	cellular protein complex disassembly
GO:0043241	2.10175972426795e-12	23.8926474699469	0.66694346412013
12	158	protein complex disassembly
GO:0000184	2.18133588763371e-12	29.092962962963 0.502318178672756	11
119	nuclear-transcribed mRNA catabolic process, nonsense-mediated decay
GO:0072594	2.18258509004625e-12	20.133835078534 0.861116877724725	13
204	establishment of protein localization to organelle
GO:0034623	6.61961889197239e-12	21.5086923658352	0.734482042765207
12	174	cellular macromolecular complex disassembly
GO:0032984	9.2560710604677e-12	20.8573872662838	0.755587848591793
12	179	macromolecular complex disassembly
GO:0019080	2.99890731462083e-11	22.3928571428571	0.637395335962909
11	151	viral genome expression
GO:0019083	2.99890731462083e-11	22.3928571428571	0.637395335962909
11	151	viral transcription
GO:0006413	3.2225444616925e-11	22.2324822695035	0.641616497128226
11	152	translational initiation
GO:0000956	1.01763794507333e-10	19.8167088607595	0.71337623693862

11	169	nuclear-transcribed mRNA catabolic process
GO:0071845	1.40210960078767e-10	14.1103167281673	1.19880977095011
13	284	cellular component disassembly at cellular level
GO:0022411	1.66728785202561e-10	13.9011363636364	1.21569441561138
13	288	cellular component disassembly
GO:0006402	1.77991700667082e-10	18.7368862275449	0.751366687426476
11	178	mRNA catabolic process
GO:0006401	8.0514033024519e-10	16.0985567010309	0.865338038890042
11	205	RNA catabolic process
GO:0019058	2.84226767064228e-09	14.17	0.975088229188292	11	231
viral infectious cycle
GO:0033365	7.35475100774916e-09	9.04065266768646	1.99660923119507
14	473	protein localization to organelle
GO:0006605	7.5550260948262e-09	9.02035152636448	2.00083039236039
14	474	protein targeting
GO:0022415	9.77451514386162e-09	12.4940562248996	1.09750190298249
11	260	viral reproductive process
GO:0016032	3.20819743699977e-07	7.78262284412626	1.90374368555809
12	451	viral reproduction
Tissue: Nerve_Tibial=>Heart_Left_Ventricle	Type:	asymmetric
SourceGene:	U6(ENSG00000206712.1)
TargetGeneSet:	MRPL20	CAMTA1	SZT2	CMPK1	RP4	BRP44	RPS7	UBC
BOLA3	UBE2E3	HIGD1A	CHMP2B	NDUFB4	RP11	RPL35A	RPL34	NDUFC1	NDUFS6
NDUFAF2	GEMIN5	RN5S201	HCP5	C4B	ABCC10	RAB23	HMGN3	MMS22L	MRPS12
ZC2HC1B	CYCS	MRPS24	INTS4L2	BRI3	RPS20	EEF1D	RPL8	NDUFB6	RPL35
RPL27A	MYBPC3	FAU	NDUFS8	RPL18AP3		MRP63	ABCD4	NPC2	GTF2A2
RPLP1	CTD	IFT140	CTA	CLN3	MINK1	FAM106A	ANAPC11	RAB12	GPX4
RPS15	MAP2K2	RPL36	GLTSCR2	SEPW1	RPL18	DGCR6L	ARFGAP3	PNPLA4	SYAP1
DYNLT3	MED12
GOBPID	Pvalue	OddsRatio	ExpCount	Count	Size	Term
GO:0006414	3.63490671144248e-18	50.006993006993	0.421424122898069	14
105	translational elongation
GO:0006415	2.51969853306545e-17	53.0185185185185	0.365234239844993
13	91	translational termination
GO:0006614	1.76159372811462e-16	44.9065217391304	0.421424122898069
13	105	SRP-dependent cotranslational protein targeting to membrane
GO:0006613	2.00200048842737e-16	44.4205495818399	0.425437685973289
13	106	cotranslational protein targeting to membrane
GO:0045047	2.00200048842737e-16	44.4205495818399	0.425437685973289
13	106	protein targeting to ER
GO:0072599	2.00200048842737e-16	44.4205495818399	0.425437685973289
13	106	establishment of protein localization to endoplasmic reticulum
GO:0070972	8.4512898968713e-16	39.3108994708995	0.473600442875926
13	118	protein localization to endoplasmic reticulum
GO:0000184	9.46016673155329e-16	38.9373165618449	0.477614005951145
13	119	nuclear-transcribed mRNA catabolic process, nonsense-mediated decay
GO:0019080	2.20982379957687e-14	29.841384863124	0.606048024358176	13
151	viral genome expression
GO:0019083	2.20982379957687e-14	29.841384863124	0.606048024358176	13
151	viral transcription
GO:0006413	2.40945640094878e-14	29.624620303757	0.610061587433396	13
152	translational initiation
GO:0006612	2.62551825901736e-14	29.4109523809524	0.614075150508615
13	153	protein targeting to membrane
GO:0043624	2.62551825901736e-14	29.4109523809524	0.614075150508615
13	153	cellular protein complex disassembly
GO:0043241	3.99794444238319e-14	28.3868199233716	0.634142965884714
13	158	protein complex disassembly
GO:0000956	9.60689568646353e-14	26.3648148148148	0.678292159712131
13	169	nuclear-transcribed mRNA catabolic process
GO:0034623	1.40252027061988e-13	25.5370600414079	0.698359975088229
13	174	cellular macromolecular complex disassembly
GO:0006402	1.88255282837985e-13	24.9109764309764	0.714414227389108

13	178	mRNA catabolic process
GO:0032984	2.0240585564025e-13	24.7591700133869	0.718427790464328
13	179	macromolecular complex disassembly
GO:0072594	1.0885229075839e-12	21.4806282722513	0.81876686734482
13	204	establishment of protein localization to organelle
GO:0006401	1.15882951268592e-12	21.3672453703704	0.82278043042004
13	205	RNA catabolic process
GO:0071845	4.35509721910966e-12	16.643265993266 1.1398519133624 14	284
cellular component disassembly at cellular level
GO:0022411	5.26033126274226e-12	16.3956536164565	1.15590616566328
14	288	cellular component disassembly
GO:0019058	5.30225772852097e-12	18.7844036697248	0.927133070375753
13	231	viral infectious cycle
GO:0022415	2.35598091127924e-11	16.5450292397661	1.04352639955712
13	260	viral reproductive process
GO:0016032	1.96566610969025e-09	10.1614312460994	1.81011694692409
14	451	viral reproduction
GO:0033365	3.45228616168807e-08	8.75019323671498	1.89841533457892
13	473	protein localization to organelle
GO:0006605	3.53913412529147e-08	8.73058568329718	1.90242889765414
13	474	protein targeting
GO:0006120	2.993531095236e-07	43.7066342057212	0.144488270707909
5	36	mitochondrial electron transport, NADH to ubiquinone
GO:0042773	1.17773404871539e-06	32.2349505840072	0.188637464535326
5	47	ATP synthesis coupled electron transport
GO:0042775	1.17773404871539e-06	32.2349505840072	0.188637464535326
5	47	mitochondrial ATP synthesis coupled electron transport
GO:0022904	2.24867507836758e-06	18.3371794871795	0.385302055221092
6	96	respiratory electron transport chain
GO:0006119	3.40171389065233e-06	25.5250978996084	0.232786658362743
5	58	oxidative phosphorylation
GO:0022900	1.54832871168784e-05	12.8590745192308	0.537817452079441
6	134	electron transport chain
Tissue: Nerve_Tibial=>Heart_Left_Ventricle	Type:	asymmetric
SourceGene:	ZNF681(ENSG00000196172.8)
TargetGeneSet:	KLHL17	ZBTB48	HNRNPR	RPL11	RP4	RP1	CCDC23	TAL1	JUN
COG2	TFB2M	PFN4	EMILIN1 LBX2	COX5B	RPL31	RPL37A	GMPPA	GORASP1 RP11
KBTBD5	SCAP	TNNC1	OSBPL11 ACPL2	EIF2B5	MB21D2	RPL35A	ATP5I	ALPK1
BBS12	NDUFS6	RPL37	ANKRD32 PGGT1B	BRD8	RPS14	TMEM14B CCND3	UBR2
RPL39P3 MICAL1	MRPS12	TCF21	FNDC1	AGPAT4	SGCE	CLDN15	ATP6V1F TNPO3	PTN
C8orf59 RPL30	COX6C	AZIN1	EEF1D	RPL8	LINC00475	CDK5RAP2
ITIH2	RPLP2	TAF10	RPL27A	CTD	VEGFB	MEN1	FAU	ALDH3B2 C11orf65
RPS25	PKNOX2	C12orf23	RPS2P5	ADPRHL1 AP1G2	RPS29	ZFYVE26 SLIRP
NDUFB1	PPP1R13B	FAN1	ATPBD4	PLDN	RPS3AP6 RPLP1	RBL2	CDK10
RPL23A	LRRC37B RPL19	RPL27	NMT1	TRIM37	RPL38	ALYREF	CABYR	TRAPPC8
RPS15	RPL36	DNM2	JUND	ATP13A1 USF2	POLR2I	COX7A1	TMEM91	GLTSCR2
KCNJ14	ZNF137P SAMHD1	NCOA3	NRIP1	CHCHD10 PNPLA4	GAGE10	COX7B	KIAA1210
MTM1
GOBPID	Pvalue	OddsRatio	ExpCount	Count	Size	Term
GO:0006414	1.51549713154803e-25	45.7307692307692	0.719327382188084
21	105	translational elongation
GO:0006415	3.01870293711272e-25	50.9217329292209	0.623417064563006
20	91	translational termination
GO:0019080	4.77951986072147e-25	33.6299342105263	1.0344612829562 23
151	viral genome expression
GO:0019083	4.77951986072147e-25	33.6299342105263	1.0344612829562 23
151	viral transcription
GO:0006614	6.77286508437162e-24	42.4929262844378	0.719327382188084
20	105	SRP-dependent cotranslational protein targeting to membrane
GO:0006613	8.30422633248145e-24	41.9958787165146	0.726178119161304
20	106	cotranslational protein targeting to membrane
GO:0045047	8.30422633248145e-24	41.9958787165146	0.726178119161304
20	106	protein targeting to ER

GO:0072599	8.30422633248145e-24	41.9958787165146	0.726178119161304
20	106	establishment of protein localization to endoplasmic reticulum
GO:0070972	8.18896918089433e-23	36.822526478946	0.808386962839942	20
118	protein localization to endoplasmic reticulum
GO:0000184	9.79171447939428e-23	36.4480245492904	0.815237699813162
20	119	nuclear-transcribed mRNA catabolic process, nonsense-mediated decay
GO:0006413	5.62966617062338e-22	29.2269524368761	1.04131201992942
21	152	translational initiation
GO:0043624	6.49138132258503e-22	29.0034965034965	1.04816275690264
21	153	cellular protein complex disassembly
GO:0043241	1.30317942730842e-21	27.935148792813	1.08241644176874	21
158	protein complex disassembly
GO:0022415	8.33452138219346e-21	19.1403389830508	1.78119161303716
24	260	viral reproductive process
GO:0034623	1.03851872134011e-20	24.9856711915535	1.19202823334025
21	174	cellular macromolecular complex disassembly
GO:0019058	1.05514302782179e-20	20.5789473684211	1.58252024081378
23	231	viral infectious cycle
GO:0006612	1.84924729330284e-20	27.0657656800228	1.04816275690264
20	153	protein targeting to membrane
GO:0032984	1.90259994217449e-20	24.1864654333009	1.22628191820635
21	179	macromolecular complex disassembly
GO:0000956	1.41224709153608e-19	24.1321892787359	1.15777454847415
20	169	nuclear-transcribed mRNA catabolic process
GO:0006402	4.04282799305268e-19	22.7431501361961	1.21943118123313
20	178	mRNA catabolic process
GO:0072594	6.23418712364457e-18	19.493670886076	1.39755034253685	20
204	establishment of protein localization to organelle
GO:0006401	6.87177814556804e-18	19.3869312350325	1.40440107951007
20	205	RNA catabolic process
GO:0016032	2.20165744349223e-17	11.6712973408541	3.08968237492215
26	451	viral reproduction
GO:0071845	2.82792175904935e-16	14.4227844398947	1.94560930039444
21	284	cellular component disassembly at cellular level
GO:0022411	3.75483763168943e-16	14.2026793431288	1.97301224828732
21	288	cellular component disassembly
GO:0033365	7.29247973035502e-13	8.80646183085207	3.24039858833299
22	473	protein localization to organelle
GO:0006605	7.60738743476219e-13	8.78634639696587	3.24724932530621
22	474	protein targeting
Tissue: Skin_Sun_Exposed_Lower_leg=>Heart_Left_Ventricle	Type:	asymmetric
SourceGene:	7SK(ENSG00000201600.1)
TargetGeneSet:	WASH7P	ATPAF1	SCP2	ACADM	GCLM	ACTG1P4	ATP5F1	KDM5B	RP11
TOMM20	FH	CNST	MDH1	C1D	NFU1	SUCLG1	SNRNP200		ATP5G3	PASK
VPRBP	CHMP2B	NDUFB4	RNF7	PIK3CA	HTT	ISOC1	SKP1	TMEM14C	TMEM14B	AIG1
BRP44L	GBAS	JHDM1D	AGK	C7orf34	VDAC3	MSC	ATAD2	FAM49B	NDUFB6
FAM108B1		HSDL2	NUP214	PHYH	RAB18	NDUFB8	PRDM11	TMEM126B		PTS
SLC25A3	C12orf23		ARL6IP4	FKBP3	PLEKHH1	ZFYVE26	PPP1R13B		DUT
COX5A	ETFA	UQCRC2	RNF40	RBL2	ANKFY1	TNRC6C	CTD	NDUFV2	VAPA
CDH20	NDUFS7	NDUFA13	UQCRFS1	RPL13A	CRLS1	C20orf94		TTLL9	ZHX3
TNRC6B	TCF20	FAM19A5	CDK16
GOBPID	Pvalue	OddsRatio	ExpCount	Count	Size	Term
GO:0045333	2.04011661884743e-15	29.9876802884615	0.648536433464812
14	142	cellular respiration
GO:0022904	4.90972084106769e-13	33.6470588235294	0.43844716628607
11	96	respiratory electron transport chain
GO:0006091	5.02898241236289e-12	11.7959183673469	1.95474361635873
17	428	generation of precursor metabolites and energy
GO:0015980	5.82989536677808e-12	14.1952054794521	1.402117500519	15
307	energy derivation by oxidation of organic compounds
GO:0022900	2.00256500788561e-11	23.190243902439	0.61199916960764	11
134	electron transport chain
GO:0006119	7.48133722625455e-09	33.345962113659	0.264895162964501	7

58	oxidative phosphorylation
GO:0006120	5.75552198565753e-07	37.95346377578	0.164417687357276	5
36	mitochondrial electron transport, NADH to ubiquinone
GO:0042773	2.25292599187876e-06	27.9918032786885	0.214656425160889
5	47	ATP synthesis coupled electron transport
GO:0042775	2.25292599187876e-06	27.9918032786885	0.214656425160889
5	47	mitochondrial ATP synthesis coupled electron transport
Tissue: Skin_Sun_Exposed_Lower_leg=>Heart_Left_Ventricle	Type:	cluster
SourceGene:	7SK(ENSG00000201600.1)
TargetGeneSet:	WASH7P	ATPAF1	SCP2	ACADM	GCLM	ACTG1P4	ATP5F1	KDM5B	RP11
TOMM20	FH	CNST	MDH1	C1D	NFU1	SUCLG1	SNRNP200	ATP5G3	PASK
VPRBP	CHMP2B	NDUFB4	RNF7	PIK3CA	HTT	ISOC1	SKP1	TMEM14C	TMEM14B	AIG1
BRP44L	GBAS	JHDM1D	AGK	C7orf34	VDAC3	MSC	ATAD2	FAM49B	NDUFB6
FAM108B1	HSDL2	NUP214	PHYH	RAB18	NDUFB8	PRDM11	TMEM126B	PTS
SLC25A3	C12orf23	ARL6IP4	FKBP3	PLEKHH1	ZFYVE26	PPP1R13B	DUT
COX5A	ETFA	UQCRC2	RNF40	RBL2	ANKFY1	TNRC6C	CTD	NDUFV2	VAPA
CDH20	NDUFS7	NDUFA13	UQCRFS1	RPL13A	CRLS1	C20orf94	TTLL9	ZHX3
TNRC6B	TCF20	FAM19A5	CDK16
GOBPID	Pvalue	OddsRatio	ExpCount	Count	Size	Term
GO:0045333	2.04011661884743e-15	29.9876802884615	0.648536433464812
14	142	cellular respiration
GO:0022904	4.90972084106769e-13	33.6470588235294	0.43844716628607
11	96	respiratory electron transport chain
GO:0006091	5.02898241236289e-12	11.7959183673469	1.95474361635873
17	428	generation of precursor metabolites and energy
GO:0015980	5.82989536677808e-12	14.1952054794521	1.402117500519	15
307	energy derivation by oxidation of organic compounds
GO:0022900	2.00256500788561e-11	23.190243902439	0.61199916960764	11
134	electron transport chain
GO:0006119	7.48133722625455e-09	33.345962113659	0.264895162964501	7
58	oxidative phosphorylation
GO:0006120	5.75552198565753e-07	37.95346377578	0.164417687357276	5
36	mitochondrial electron transport, NADH to ubiquinone
GO:0042773	2.25292599187876e-06	27.9918032786885	0.214656425160889
5	47	ATP synthesis coupled electron transport
GO:0042775	2.25292599187876e-06	27.9918032786885	0.214656425160889
5	47	mitochondrial ATP synthesis coupled electron transport
Tissue: Heart_Left_Ventricle=>Skin_Sun_Exposed_Lower_leg	Type:	asymmetric
SourceGene:	C1DP1(ENSG00000231245.2)
TargetGeneSet:	RPL11	RPS8	RP11	RPL5	MAEL	FAM89A	RPS7	UBC
RPL31	GLS	EEF1B2	C3orf19	RPL24	CSTA	CNBP	RPL34	RPL37	RIOK2
RPL26L1	CDC40	BUD31	NSMCE2	ZDHHC21	RPS6	ZNF658	C9orf78	ADK	RPS24
CCDC90B	RPS25	SETD8	SFSWAP	ARF4P4	EAPP	COX16	RPS27L	LRRK1	MAP1LC3B
RPS4XP17	LINC00324	RPL26	TMEM11	RPL19	SMARCE1	BECN1	RPL27
RPS7P11	CEP112	ICT1	NFKBIB	MED29	ZNF230	PIH1D1	ZNF350	SNRPB2	ZNRF3
PHF5A
GOBPID	Pvalue	OddsRatio	ExpCount	Count	Size	Term
GO:0006415	1.87027438692148e-26	109.734234234234	0.295965677115771
17	91	translational termination
GO:0006414	2.5951120880528e-25	92.1863636363636	0.341498858210504
17	105	translational elongation
GO:0006614	2.29167751868088e-23	83.0155853570134	0.341498858210504
16	105	SRP-dependent cotranslational protein targeting to membrane
GO:0006613	2.69360292890147e-23	82.0874551971326	0.3447512282887	16
106	cotranslational protein targeting to membrane
GO:0045047	2.69360292890147e-23	82.0874551971326	0.3447512282887	16
106	protein targeting to ER
GO:0072599	2.69360292890147e-23	82.0874551971326	0.3447512282887	16
106	establishment of protein localization to endoplasmic reticulum
GO:0034623	3.89985821497553e-23	56.6896551724138	0.565912393605979
18	174	cellular macromolecular complex disassembly
GO:0032984	6.60341824934804e-23	54.9098307988863	0.582174243996955
18	179	macromolecular complex disassembly

GO:0070972	1.66002805142341e-22	72.3693864642631	0.383779669227043
16	118	protein localization to endoplasmic reticulum
GO:0000184	1.91399679581293e-22	71.6617601002192	0.387032039305238
16	119	nuclear-transcribed mRNA catabolic process, nonsense-mediated decay
GO:0043624	2.21961866314831e-22	59.45	0.497612621963878	17	153
cellular protein complex disassembly
GO:0043241	3.91334845523995e-22	57.3217494089835	0.513874472354854
17	158	protein complex disassembly
GO:0019080	1.02145400016613e-20	54.5529271206691	0.491107881807487
16	151	viral genome expression
GO:0019083	1.02145400016613e-20	54.5529271206691	0.491107881807487
16	151	viral transcription
GO:0006413	1.13929928685966e-20	54.1480075901328	0.494360251885683
16	152	translational initiation
GO:0006612	1.26976388250673e-20	53.748999293619	0.497612621963878	16
153	protein targeting to membrane
GO:0000956	6.53363370348099e-20	48.0742146320894	0.549650543215002
16	169	nuclear-transcribed mRNA catabolic process
GO:0006402	1.52860245164895e-19	45.3747510951812	0.57892187391876
16	178	mRNA catabolic process
GO:0071845	3.05587798645251e-19	32.9898885143894	0.92367310220746
18	284	cellular component disassembly at cellular level
GO:0022411	3.93092665671937e-19	32.4919540229885	0.936682582520241
18	288	cellular component disassembly
GO:0072594	1.40621880723957e-18	39.0281400137268	0.663483495951837
16	204	establishment of protein localization to organelle
GO:0006401	1.52214678734768e-18	38.8189110769756	0.666735866030033
16	205	RNA catabolic process
GO:0019058	1.04501178139908e-17	34.0621155288822	0.75129748806311
16	231	viral infectious cycle
GO:0022415	6.93995669532707e-17	29.9524061343205	0.845616220330773
16	260	viral reproductive process
GO:0033365	4.73789041113529e-14	17.3330409356725	1.53837104698637
17	473	protein localization to organelle
GO:0006605	4.90422245348897e-14	17.2938730853392	1.54162341706456
17	474	protein targeting
GO:0016032	3.84640051771802e-13	16.5742677048572	1.46681890526607
16	451	viral reproduction
GO:0022613	1.08689794804515e-10	20.2609034267913	0.731783267593938
11	225	ribonucleoprotein complex biogenesis
GO:0071843	2.07450223779525e-10	18.9980506822612	0.777316448688672
11	239	cellular component biogenesis at cellular level
GO:0042274	2.42652791495935e-09	131.785714285714	0.0585426614075151
5	18	ribosomal small subunit biogenesis
GO:0042254	2.36707126530468e-08	20.7499545371886	0.484603141651097
8	149	ribosome biogenesis
GO:0006364	4.0483575735676e-08	25.8115979381443	0.338246488132309
7	104	rRNA processing
GO:0016072	5.97647474353613e-08	24.2978155339806	0.357760708601481
7	110	rRNA metabolic process
GO:0034660	3.34660909614529e-06	10.4615384615385	0.926925472285655
8	285	ncRNA metabolic process
GO:0034470	3.44565047883556e-06	12.885621761658	0.650474015639056	7
200	ncRNA processing
GO:0042273	6.94949201671952e-06	109.05303030303	0.0390284409383434	3
12	ribosomal large subunit biogenesis
GO:0022618	1.36149834757131e-05	18.9338624338624	0.308975157428552
5	95	ribonucleoprotein complex assembly
GO:0071826	1.74781816011287e-05	17.9310776942356	0.325237007819528
5	100	ribonucleoprotein complex subunit organization
Tissue: Heart_Left_Ventricle=>Skin_Sun_Exposed_Lower_leg	Type:	asymmetric
SourceGene:	ENSG00000214754.3
TargetGeneSet:	RP4	KCNAB2	CDCA8	IQGAP3	NCAPH	CKAP2L	SPC25	POC1A

NCAPG RP11 NUDT6 NEIL3 CCNB1 NR2F2 CDC25C HMMR USP49 PMS2P9
IMMP2L-IT1 BAAT ZWINT KIF11 MKI67 MPP2 E2F7 DLGAP5 PAPOLA
BUB1B C15orf42 PRC1 PKMYT1 SNX29P2 FAM64A SPAG5 TOP2A KIF18B
PALM3 UBE2C CA5BP1 HTATSF1
GOBPID Pvalue OddsRatio ExpCount Count Size Term
GO:0000280 5.86836681853956e-15 32.599537037037 0.775032869697599 14
350 nuclear division
GO:0007067 5.86836681853956e-15 32.599537037037 0.775032869697599 14
350 mitosis
GO:0000087 8.66675876103026e-15 31.6348747591522 0.797176665974673
14 360 M phase of mitotic cell cycle
GO:0048285 1.2656791333764e-14 30.7244069912609 0.819320462251747
14 370 organelle fission
GO:0051301 1.98055752977858e-13 24.8267376966007 1.00089959172376
14 452 cell division
GO:0007051 5.82160862710856e-12 66.4212962962963 0.177150370216594
8 80 spindle organization
GO:0000819 1.61299677968602e-11 89.4382222222222 0.115147740640786
7 52 sister chromatid segregation
GO:0007059 5.48522525331172e-10 36.0782828282828 0.310013147879039
8 140 chromosome segregation
GO:0000070 9.38407128529748e-10 77.1520572450805 0.108504601757664
6 49 mitotic sister chromatid segregation
GO:0007017 7.46425377494542e-09 16.6579634464752 0.870251193689018
10 393 microtubule-based process
GO:0010564 1.22667010602256e-08 15.7684518451845 0.916753165870874
10 414 regulation of cell cycle process
GO:0007346 1.2775245293167e-08 18.7999112688554 0.67095702719535
9 303 regulation of mitotic cell cycle
GO:0071156 8.02928498929883e-08 18.44140625 0.58459622171476 8
264 regulation of cell cycle arrest
GO:0000226 9.27877212936575e-08 18.0817369093231 0.595668119853297
8 269 microtubule cytoskeleton organization
GO:0030261 5.21110494056672e-07 79.0824175824176 0.0664313888312228
4 30 chromosome condensation
GO:0007093 5.24238690105645e-07 24.7877385772123 0.307798768251332
6 139 mitotic cell cycle checkpoint
GO:0000086 6.45536442742863e-07 23.88127090301 0.318870666389869 6
144 G2/M transition of mitotic cell cycle
GO:0007052 7.74487414122206e-07 70.8866995073892 0.073074527714345
4 33 mitotic spindle organization
GO:0000075 7.97570394176808e-07 16.900085106383 0.535879869905197 7
242 cell cycle checkpoint
GO:0007050 1.030563893515e-06 12.9806094182825 0.81710608262404
8 369 cell cycle arrest
GO:0000236 1.11098295158155e-06 33.1921296296296 0.188222268355131
5 85 mitotic prometaphase
GO:0007088 1.32068018549993e-06 31.9857206604195 0.194865407238253
5 88 regulation of mitosis
GO:0051783 1.32068018549993e-06 31.9857206604195 0.194865407238253
5 88 regulation of nuclear division
GO:0045786 3.64344177840547e-06 10.8702408702409 0.967683897308145
8 437 negative regulation of cell cycle
GO:0048015 7.75901084799842e-06 21.8824609733701 0.279011833091136
5 126 phosphatidylinositol-mediated signaling
GO:0048017 7.75901084799842e-06 21.8824609733701 0.279011833091136
5 126 inositol lipid-mediated signaling
GO:0051329 1.54238193478613e-05 10.5730107526882 0.839249878901114
7 379 interphase of mitotic cell cycle
GO:0051325 1.76546950891511e-05 10.3445263157895 0.856964915922773
7 387 interphase
Tissue: Skin_Sun_Exposed_Lower_leg=>Heart_Left_Ventricle Type: cluster
SourceGene: ENSG00000214897.3

TargetGeneSet: SLC35E2 CAPZB ASAP3 GNAI3 RP11 PMF1 PTRHD1 FAM161A RABL2A MYL3 RHOA MAP3K13 FGF12 NOP14 FAM184B SLAIN2 ENPEP NDUFC1 CTD ISOC1 PCDHB9 DHX16 IBTK UBE2J1 TRIM73 ANKIB1 ECD ATP5J2 UQCRB COX6C NOL8 CAMSAP1 DLG5 EIF5AL1 RPL13AP5 NDUFB8 RPLP2 C11orf10 PRDX5 FAU EFEMP2 RPS25 AVIL C12orf51 TESC ARL6IP4 RFC3 DHRS12 PSME2 LGALS3 NDUFB1 SRP14 ZFP106 POLR2M PSMA4 NDUFB10 HCFC1R1 TMEM219 RANBP10 CLEC18B PSMB6 KIAA0753 LRRC48 RPL27 EFTUD2 RPL38 C17orf89 SIRT7 IER3IP1 PIN1 GADD45GIP1 C19orf53 TPM4 COMP ZNF461 SEPW1 KCNJ14 RPS11 NDUFA3 ZNF471 RPS21 URB1 PI4KA NDUFB11 HUWE1 TAF1 UPRT COX7B CXorf57 NUP62CL NDUFA1

GOBPID	Pvalue	OddsRatio	ExpCount	Count	Size	Term
GO:0022904	1.33732100616129e-12	30.3247830279653	0.478305999584804	11	96	respiratory electron transport chain
GO:0022900	5.37588670989758e-11	20.9004398240704	0.667635457753789	11	134	electron transport chain
GO:0045333	1.01044201491657e-10	19.613064697785	0.707494291052522	11	142	cellular respiration
GO:0006414	2.31969129398839e-09	21.2544642857143	0.523147187045879	9	105	translational elongation
GO:0042773	3.03436326636512e-09	38.605	0.23417064563006	7	47	ATP synthesis coupled electron transport
GO:0042775	3.03436326636512e-09	38.605	0.23417064563006	7	47	mitochondrial ATP synthesis coupled electron transport
GO:0043624	4.02512454765841e-09	16.056846379427	0.762300186838281	10	153	cellular protein complex disassembly
GO:0043241	5.49696234036314e-09	15.5089363557105	0.78721195764999	10	158	protein complex disassembly
GO:0006119	1.38907487019764e-08	30.2552036199095	0.288976541415819	7	58	oxidative phosphorylation
GO:0034623	1.39179631874333e-08	13.9801337529504	0.866929624247457	10	174	cellular macromolecular complex disassembly
GO:0006415	1.55689248791091e-08	21.5301204819277	0.453394228773095	8	91	translational termination
GO:0032984	1.82530494398229e-08	13.561748425272	0.891841395059165	10	179	macromolecular complex disassembly
GO:0006120	2.14101005549352e-08	43.4818181818182	0.179364749844301	6	36	mitochondrial electron transport, NADH to ubiquinone
GO:0006614	4.83291413978044e-08	18.4046391752577	0.523147187045879	8	105	SRP-dependent cotranslational protein targeting to membrane
GO:0006613	5.20682592711374e-08	18.2155612244898	0.528129541208221	8	106	cotranslational protein targeting to membrane
GO:0045047	5.20682592711374e-08	18.2155612244898	0.528129541208221	8	106	protein targeting to ER
GO:0072599	5.20682592711374e-08	18.2155612244898	0.528129541208221	8	106	establishment of protein localization to endoplasmic reticulum
GO:0006612	6.3634435923876e-08	14.1220238095238	0.762300186838281	9	153	protein targeting to membrane
GO:0070972	1.20450250923153e-07	16.2147727272727	0.587917791156321	8	118	protein localization to endoplasmic reticulum
GO:0015980	3.17379971457975e-07	8.57958573327426	1.5295827278389	11	307	energy derivation by oxidation of organic compounds
GO:0071845	1.34311920586872e-06	8.3029197080292	1.41498858210504	10	284	cellular component disassembly at cellular level
GO:0022411	1.52401164617413e-06	8.18113251334416	1.43491799875441	10	288	cellular component disassembly
GO:0000184	2.03452396418553e-06	13.7182692307692	0.592900145318663	7	119	nuclear-transcribed mRNA catabolic process, nonsense-mediated decay
GO:0072594	7.58802498178715e-06	9.0452806122449	1.01640024911771	8	204	establishment of protein localization to organelle
GO:0006091	8.03621405590083e-06	6.03774029956363	2.13244758148225	11	428	generation of precursor metabolites and energy
GO:0019080	9.8734089327833e-06	10.6458333333333	0.752335478513598	7	151	viral genome expression

GO:0019083 9.8734089327833e-06 10.6458333333333 0.752335478513598
7 151 viral transcription
GO:0006413 1.0309333582479e-05 10.5716710875332 0.757317832675939
7 152 translational initiation
GO:0016032 1.31432848272601e-05 5.71270491803279 2.24704172721611
11 451 viral reproduction
Tissue: Heart_Left_Ventricle=>Skin_Sun_Exposed_Lower_leg Type: cluster
SourceGene: ENSG00000224505.1
TargetGeneSet: RPL22 RPL11 RP1 TMEM222 SNRNP40 MFSD2A ST7L RBM8A MCL1
CHTOP CLK2 DDX59 ARL8A ADCK3 OXER1 PPP3R1 RPIA IL1RL2 CCDC115
WDR75 FAM117B FAM134A 7SK CIDECP SLC6A11 RPL32 C3orf19 ZNF501 FAM212A
C3orf14 RYBP IFT57 QTRTD1 RAB41 MSL2 YTHDC1 DCK RCHY1 CDKN2AIP
RP11 CARD6 ZNF131 RIOK2 RPS14 BOD1 BTN2A1 SRPK1 FRS3 TJAP1 RPF2
PPIL4 LATS1 AP5Z1 PMS2CL TRA2A RP9 RALA URGCP NUDCD3 COBL
CCT6P1 SBDSP1 MTERF PON1 ZNF3 EPO THAP5 ZC3HC1 TMEM209 TBXAS1
SLC37A3 C7orf34 ZNF212 NEIL2 R3HCC1 SGK3 TERF1 RPL30 EBAG9 UTP23 RPL8
KLHL9 C9orf156 EPB41L4B ZFAND4 SIRT1 SLC25A16 SUPV3L1
TYSND1 DNAJB12 SMC3 RPLP2 RRP8 ARFGAP2 FAM111B MS4A7 VPS37C ZBTB3
TRMT112 FAU C11orf68 SF3B2 PITPNM1 ZNF259 RPS25 VPS11 NECAP1
MIR614 RPL14P1 PARPBP C12orf73 SART3 RSRC2 USP12 KBTBD7 ARL11 TTC5
RBM23 NGDN IFT43 NDNL2 FAM82A2 RPL4 WHAMM VPS33B TBC1D24 ZNF205
TFAP4 CCDC101 HIRIP3 VPS4A GLOD4 DVL2 SNORA48 PLD6 FLCN ZNF286B
TLK2P1 ZNF830 RPL19 MED1 PGAP3 SNX11 FAM117A SMG8 RPS6KB1 APPBP2
SNORA76 NOL11 KPNA2 SAP30BP L3MBTL4 GREB1L SERPINB13 TMX3 SAFB XAB2
NR2C2AP SUGP1 TSSK6 ZNF430 ZNF461 ZNF569 ZNF571 RPS16 CCDC97 ZNF574
ZNF180 SNRPD2 GLTSCR2 RPL18 RPS11 ZNF649 ZNF766 CHMP2A ZCCHC3 RSPO4
PSMF1 MRPS26 FLRT3 DTD1 CRNKL1 PDRG1 CDK5RAP1 PCIF1 NCOA5
ZFP64 LSM14B PRPF6 ICOSLG C22orf39 SF3A1 LIMK2 IL2RB RPL3 TEF
ARAF TIMM17B GPKOW DKC1
GOBPID Pvalue OddsRatio ExpCount Count Size Term
GO:0000184 1.47353697992736e-14 16.537296037296 1.31755587848592 17
119 nuclear-transcribed mRNA catabolic process, nonsense-mediated decay
GO:0006415 6.29387212451571e-14 19.3489110707804 1.00754273060688
15 91 translational termination
GO:0006414 5.59455896737114e-13 16.3229885057471 1.1625493045464 15
105 translational elongation
GO:0006614 5.59455896737114e-13 16.3229885057471 1.1625493045464 15
105 SRP-dependent cotranslational protein targeting to membrane
GO:0006613 6.45523066346816e-13 16.1424782114437 1.17362120268494
15 106 cotranslational protein targeting to membrane
GO:0045047 6.45523066346816e-13 16.1424782114437 1.17362120268494
15 106 protein targeting to ER
GO:0072599 6.45523066346816e-13 16.1424782114437 1.17362120268494
15 106 establishment of protein localization to endoplasmic reticulum
GO:0006402 1.07553284885558e-12 11.1953345070423 1.97079786865961
18 178 mRNA catabolic process
GO:0070972 3.21418506412267e-12 14.2497489119518 1.30648398034738
15 118 protein localization to endoplasmic reticulum
GO:0000956 5.16016841499698e-12 11.0582903938167 1.87115078541277
17 169 nuclear-transcribed mRNA catabolic process
GO:0019080 1.01293782346996e-11 11.6510288065844 1.67185661891911
16 151 viral genome expression
GO:0019083 1.01293782346996e-11 11.6510288065844 1.67185661891911
16 151 viral transcription
GO:0006413 1.12143467704931e-11 11.5645424836601 1.68292851705764
16 152 translational initiation
GO:0006401 1.195397214553e-11 9.56059350756948 2.26973911840011
18 205 RNA catabolic process
GO:0019058 8.7700419798809e-11 8.37809958341599 2.55760847000208
18 231 viral infectious cycle
GO:0072594 1.05287618598566e-10 8.96630642085188 2.25866722026157
17 204 establishment of protein localization to organelle
GO:0006612 1.4136980983579e-10 10.6094452773613 1.69400041519618

15	153	protein targeting to membrane
GO:0043624	1.4136980983579e-10	10.6094452773613	1.69400041519618
15	153	cellular protein complex disassembly
GO:0043241	2.23505409197828e-10	10.2348685796962	1.74935990588887
15	158	protein complex disassembly
GO:0022415	6.05733383335712e-10	7.35892212780817	2.87869351601965
18	260	viral reproductive process
GO:0034623	8.70727292601652e-10	9.19453480806766	1.92651027610546
15	174	cellular macromolecular complex disassembly
GO:0032984	1.29289078788587e-09	8.91105971404542	1.98186976679815
15	179	macromolecular complex disassembly
GO:0033365	2.7716193002126e-07	4.36392305266477	5.23700781952806
20	473	protein localization to organelle
GO:0000377	4.28867801478446e-07	6.52847526445133	2.25866722026157
13	204	RNA splicing, via transesterification reactions with bulged adenosine as nucleophile
GO:0000398	4.28867801478446e-07	6.52847526445133	2.25866722026157
13	204	nuclear mRNA splicing, via spliceosome
GO:0000375	5.65602294868465e-07	6.35967652367069	2.31402671095426
13	209	RNA splicing, via transesterification reactions
GO:0016032	5.95962511640301e-07	4.32297412660888	4.99342606048024
19	451	viral reproduction
GO:0071845	6.07494944339125e-07	5.39238559159082	3.14441907134454
15	284	cellular component disassembly at cellular level
GO:0022411	7.25158637153665e-07	5.31186055323986	3.18870666389869
15	288	cellular component disassembly
GO:0006605	1.25658739599681e-06	4.09763853168108	5.2480797176666 19
474	protein targeting
GO:0022613	1.30272075961984e-06	5.87302656911821	2.49117708117085
13	225	ribonucleoprotein complex biogenesis
GO:0071843	2.55238701816115e-06	5.5037324664379 2.64618365511037	13
239	cellular component biogenesis at cellular level
GO:0008380	3.02433911209088e-06	4.69648454993283	3.57622309874749
15	323	RNA splicing
Tissue: Skin_Sun_Exposed_Lower_leg=>Heart_Left_Ventricle	Type:	asymmetric
SourceGene:	ENSG00000250699.1
TargetGeneSet:	RPL11	C1orf212	UBC	R3HDM1	RPL32	RPL14	ANTXR2
RPL15P3 RPS10	WDR11	CASP8AP2	PNPLA8	TAS2R5	MLL3	RPS20	RP11
DEPTOR	PRPF4	UCK1	RPL27A	RPS13	MRGPRX2 RPL14P1 RPL18AP3	HMGB3P4
MAPK1IP1L	FAN1	RPS3AP6 RPL4	RPS2	CTA	CLN3	SPAG7	RPL23A
RPL19	RPL27	UBA52	GLTSCR2 RPS5	RPS4X
GOBPID	Pvalue	OddsRatio	ExpCount	Count	Size	Term
GO:0006415	4.24193765571411e-28	204.017777777778	0.195211404055083
16	91	translational termination
GO:0006414	5.02934023179459e-27	171.757303370787	0.225243927755865
16	105	translational elongation
GO:0006614	5.02934023179459e-27	171.757303370787	0.225243927755865
16	105	SRP-dependent cotranslational protein targeting to membrane
GO:0006613	5.91761980235816e-27	169.837037037037	0.227389108020206
16	106	cotranslational protein targeting to membrane
GO:0045047	5.91761980235816e-27	169.837037037037	0.227389108020206
16	106	protein targeting to ER
GO:0072599	5.91761980235816e-27	169.837037037037	0.227389108020206
16	106	establishment of protein localization to endoplasmic reticulum
GO:0070972	3.69319741403028e-26	149.730718954248	0.253131271192305
16	118	protein localization to endoplasmic reticulum
GO:0000184	4.26269833892774e-26	148.266666666667	0.255276451456647
16	119	nuclear-transcribed mRNA catabolic process, nonsense-mediated decay
GO:0019080	2.35275647218531e-24	112.869135802469	0.323922219915577
16	151	viral genome expression
GO:0019083	2.35275647218531e-24	112.869135802469	0.323922219915577
16	151	viral transcription
GO:0006413	2.62695791137595e-24	112.03137254902 0.326067400179918	16

152	translational initiation
GO:0006612	2.93086226808131e-24	111.205839416058	0.32821258044426
16	153	protein targeting to membrane
GO:0043624	2.93086226808131e-24	111.205839416058	0.32821258044426
16	153	cellular protein complex disassembly
GO:0043241	5.01021707818843e-24	107.252582159624	0.338938481765968
16	158	protein complex disassembly
GO:0000956	1.53371637953331e-23	99.4649237472767	0.362535464673725
16	169	nuclear-transcribed mRNA catabolic process
GO:0034623	2.48676496348234e-23	96.2835443037975	0.373261365995433
16	174	cellular macromolecular complex disassembly
GO:0006402	3.62246278899431e-23	93.8798353909465	0.381842087052799
16	178	mRNA catabolic process
GO:0032984	3.97411192101242e-23	93.2973415132924	0.383987267317141
16	179	macromolecular complex disassembly
GO:0072594	3.42510091437734e-22	80.7489361702128	0.43761677392568
16	204	establishment of protein localization to organelle
GO:0006401	3.71138034536168e-22	80.316049382716 0.439761954190021	16
205	RNA catabolic process
GO:0019058	2.61896002440318e-21	70.4744186046512	0.495536641062902
16	231	viral infectious cycle
GO:0022415	1.79345168034597e-20	61.9715846994536	0.557746868728808
16	260	viral reproductive process
GO:0071845	7.48960941953688e-20	56.326368159204 0.609231195073005	16
284	cellular component disassembly at cellular level
GO:0022411	9.38775022166801e-20	55.4823529411765	0.617811916130372
16	288	cellular component disassembly
GO:0033365	7.43915310463247e-18	37.1848370927318	1.01467026503356
17	473	protein localization to organelle
GO:0006605	7.70860707812217e-18	37.1008127539856	1.0168154452979 17
474	protein targeting
GO:0016032	1.21809364465966e-16	34.2927203065134	0.967476299218047
16	451	viral reproduction
Tissue: Skin_Sun_Exposed_Lower_leg=>Heart_Left_Ventricle	Type:	asymmetric
SourceGene:	ENSG00000258513.1
TargetGeneSet:	RP4	RPS7	RPL24	RP11	MECOM	CTD	RPL39P3 SNHG6
RPL35	RPLP2	RPL27A	U6	TRAC	RPS29	RPL7AP6 RPS7P1	RPS15	RPL36
GOBPID	Pvalue	OddsRatio	ExpCount	Count	Size	Term
GO:0006415	8.75466515106586e-16	345.927710843374	0.0755657047955159
8	91	translational termination
GO:0006414	2.86223155543203e-15	295.711340206186	0.0871911978409799
8	105	translational elongation
GO:0006614	2.86223155543203e-15	295.711340206186	0.0871911978409799
8	105	SRP-dependent cotranslational protein targeting to membrane
GO:0006613	3.09511656806613e-15	292.673469387755	0.0880215902013702
8	106	cotranslational protein targeting to membrane
GO:0045047	3.09511656806613e-15	292.673469387755	0.0880215902013702
8	106	protein targeting to ER
GO:0072599	3.09511656806613e-15	292.673469387755	0.0880215902013702
8	106	establishment of protein localization to endoplasmic reticulum
GO:0070972	7.48533940788438e-15	260.527272727273	0.0979862985260536
8	118	protein localization to endoplasmic reticulum
GO:0000184	8.02283483790861e-15	258.162162162162	0.0988166908864438
8	119	nuclear-transcribed mRNA catabolic process, nonsense-mediated decay
GO:0019080	5.63410912080144e-14	199.944055944056	0.125389246418933
8	151	viral genome expression
GO:0019083	5.63410912080144e-14	199.944055944056	0.125389246418933
8	151	viral transcription
GO:0006413	5.94563862927435e-14	198.541666666667	0.126219638779323
8	152	translational initiation
GO:0006612	6.27211613900626e-14	197.158620689655	0.127050031139714
8	153	protein targeting to membrane
GO:0043624	6.27211613900626e-14	197.158620689655	0.127050031139714

8	153	cellular protein complex disassembly
GO:0043241	8.15077837252388e-14	190.52	0.131201992941665	8	158
protein complex disassembly
GO:0000956	1.40934469304936e-13	177.366459627329	0.140336308905958
8	169	nuclear-transcribed mRNA catabolic process
GO:0034623	1.78610797332018e-13	171.963855421687	0.144488270707909
8	174	cellular macromolecular complex disassembly
GO:0006402	2.14811893079371e-13	167.870588235294	0.14780984014947l
8	178	mRNA catabolic process
GO:0032984	2.2480565338914e-13	166.877192982456	0.148640232509861
8	179	macromolecular complex disassembly
GO:0072594	6.48443399499177e-13	145.336734693878	0.169400041519618
8	204	establishment of protein localization to organelle
GO:0006401	6.74608060895868e-13	144.58883248731	0.170230433880008	8
205	RNA catabolic process
GO:0019058	1.76984701078215e-12	127.497757847534	0.191820635250156
8	231	viral infectious cycle
GO:0022415	4.58855624840892e-12	112.595238095238	0.215902013701474
8	260	viral reproductive process
GO:0071845	9.32944532399793e-12	102.630434782609	0.235831430350841
8	284	cellular component disassembly at cellular level
GO:0022411	1.04380518630009e-11	101.135714285714	0.239152999792402
8	288	cellular component disassembly
GO:0016032	3.75459422710082e-10	63.1873589164786	0.374506954536018
8	451	viral reproduction
GO:0033365	5.48138591881141e-10	60.1032258064516	0.392775586464605
8	473	protein localization to organelle
GO:0006605	5.57407557789227e-10	59.9699570815451	0.393605978824995
8	474	protein targeting
Tissue: Heart_Left_Ventricle=>Skin_Sun_Exposed_Lower_leg	Type:	asymmetric
SourceGene:	KCTD9P1(ENSG00000225014.1)
TargetGeneSet:	FAM132A	CTNNBIP1	CROCC	RPL11	C1orf135	SH3BGRL3
PPP1R8	YRDC	RPS8	UQCRH	TTC39A	KTI12	RP4	RPL5	DNTTIP2	HIAT1	RP11
HBXIP	ATP5F1	B3GALT2	TIMM17A	TMEM183A	TFB2M	SH3YL1	RPS7	SPDYA
SNORA67	UBC	TPRKB	ANKRD36C	RPL31	GLI2	DAPL1	SENP2	NBEAL1
EEF1B2	MRPL44	MYEOV2	RPL15	CBX5P1	ZNF90P1	RPL24	MRPL47	SDHAP2	RPL35A	HOPX
FAM190A	RPL34	OSTC	RPS3A	FBXO8	CDKN2AIP	SLC38A9	NDUFAF2	ADAMTS6
ZFYVE16	CAMLG	RPL26L1	TMEM14C	TMEM14B	TBCC	RP13	EEF1A1	RP1	TOMM7	GBAS
MIR1255B1	RPS3AP26	HBP1	TRIM35	EFCAB1	RDH10	TCEB1	PAF1
POLR2K	EIF3E	TTC35	EIF3H	RPS6	LINC00032	NDUFB6	ZNF658	KLF9
NCBP1	ATP6V1G1	ASB6	ANAPC16	CHCHD1	RPS24	MARK2P9	C10orf12
C11orf58	RPS13	EIF3M	VWCE	MALAT1	PAK1	CCDC90B	RPS25	U6
TAS2R18	PFDN5	ANKS1B	PSMD9	TPT1	EBPL	THSD1	COMMD6	CLYBL-AS2	NGDN
EAPP	PSMA6	KLHDC2	PSMA3	MOAP1	BAG5	RSL24D1	RPS3AP6	RPS27L	WDR61
MORF4L1	MRPL46	SPSB3	RPS15A	LYRM1	TMED6	MAP1LC3B	RPL26	ZNF286B
RPS7P1	RPL17	RPL19	RPL27	HIGD1B	CTD	ICT1	ZFP161	WDR7	PQLC1
HSBP1L1	CAPS	WDR83	ZNF350	ZNF460	GCNT1P1	PSMA7	SNRPD3	PHF5A	EEF1B2P3
COX7B
GOBPID	Pvalue	OddsRatio	ExpCount	Count	Size	Term
GO:0006414	7.00108156243048e-28	46.5087918321044	0.791986713722234
23	105	translational elongation
GO:0006415	9.9573812581339e-28	52.3081792437115	0.686388485225936
22	91	translational termination
GO:0006413	6.13779952928328e-27	33.3122422197225	1.14649505224552
25	152	translational initiation
GO:0000184	1.59603668113705e-26	39.687257751938	0.897584942218532	23
119	nuclear-transcribed mRNA catabolic process, nonsense-mediated decay
GO:0019080	1.70098294364476e-25	31.6035201482168	1.13895232163864
24	151	viral genome expression
GO:0019083	1.70098294364476e-25	31.6035201482168	1.13895232163864
24	151	viral transcription
GO:0006614	1.35355223956287e-24	40.5056818181818	0.791986713722234
21	105	SRP-dependent cotranslational protein targeting to membrane

GO:0006613 1.67808866830512e-24 40.0263368983957 0.799529444329112
21 106 cotranslational protein targeting to membrane
GO:0045047 1.67808866830512e-24 40.0263368983957 0.799529444329112
21 106 protein targeting to ER
GO:0072599 1.67808866830512e-24 40.0263368983957 0.799529444329112
21 106 establishment of protein localization to endoplasmic reticulum
GO:0072594 1.2479011519117e-23 23.5484836392658 1.5387170438032 25
204 establishment of protein localization to organelle
GO:0019058 1.35404133555629e-23 21.6022333235381 1.74237077018891
26 231 viral infectious cycle
GO:0070972 1.8718659352694e-23 35.0451030927835 0.890042211611653
21 118 protein localization to endoplasmic reticulum
GO:0000956 7.73126774786728e-23 26.0041414463205 1.27472147256245
23 169 nuclear-transcribed mRNA catabolic process
GO:0043624 2.08118992313669e-22 27.4319557778363 1.1540377828524 22
153 cellular protein complex disassembly
GO:0006402 2.63003009516724e-22 24.4786946736684 1.34260604802436
23 178 mRNA catabolic process
GO:0022415 2.94732327295664e-22 18.8862115127175 1.96110995778839
26 260 viral reproductive process
GO:0006401 3.26019612331964e-22 22.0906077348066 1.54625977441008
24 205 RNA catabolic process
GO:0043241 4.32304443474305e-22 26.4141311697093 1.19175143588679
22 158 protein complex disassembly
GO:0034623 3.80972730832128e-21 23.6070780399274 1.31243512559684
22 174 cellular macromolecular complex disassembly
GO:0006612 5.61471104620423e-21 25.6895661157025 1.1540377828524 21
153 protein targeting to membrane
GO:0032984 7.18547446031269e-21 22.8472069697635 1.35014877863124
22 179 macromolecular complex disassembly
GO:0016032 1.63195272019299e-20 12.5569018912174 3.40177150370217
30 451 viral reproduction
GO:0071845 1.16772050414388e-17 14.4285396061659 2.14213549235347
23 284 cellular component disassembly at cellular level
GO:0022411 1.59447401062236e-17 14.2067134708205 2.17230641478098
23 288 cellular component disassembly
GO:0033365 8.80019870315439e-15 9.23017644557823 3.56771157705349
25 473 protein localization to organelle
GO:0006605 9.23680250813398e-15 9.20895641107222 3.57525430766037
25 474 protein targeting
GO:0022613 1.39770626050915e-08 9.02564858490566 1.69711438654764
13 225 ribonucleoprotein complex biogenesis
GO:0071843 2.8672468897145e-08 8.45814896755162 1.80271261504394
13 239 cellular component biogenesis at cellular level
GO:0042274 1.76388272266686e-07 52.9918639053254 0.135769150923812
5 18 ribosomal small subunit biogenesis
GO:0042254 1.7878479343382e-07 10.3211975873846 1.12386686042488
10 149 ribosome biogenesis
GO:0006364 1.14295904297322e-06 11.7541254125413 0.784443983115355
8 104 rRNA processing
GO:0042273 1.44706157301348e-06 68.2571428571429 0.090512767282541
4 12 ribosomal large subunit biogenesis
GO:0016072 1.75139242922031e-06 11.0580469811687 0.829700366756626
8 110 rRNA metabolic process
GO:0051352 1.67224777408421e-05 12.6001765225066 0.543076603695246
6 72 negative regulation of ligase activity
GO:0051444 1.67224777408421e-05 12.6001765225066 0.543076603695246
6 72 negative regulation of ubiquitin-protein ligase activity
GO:0031571 1.81091961104918e-05 12.4112447471381 0.550619334302124
6 73 mitotic cell cycle G1/S transition DNA damage checkpoint
Tissue: Heart_Left_Ventricle=>Skin_Sun_Exposed_Lower_leg Type: cluster
SourceGene: KCTD9P1(ENSG00000225014.1)
TargetGeneSet: FAM132A CTNNBIP1 CROCC RPL11 C1orf135 SH3BGRL3

PPP1R8 YRDC RPS8 UQCRH TTC39A KTI12 RP4 RPL5 DNTTIP2 HIAT1 RP11
HBXIP ATP5F1 B3GALT2 TIMM17A TMEM183A TFB2M SH3YL1 RPS7 SPDYA
SNORA67 UBC TPRKB ANKRD36C RPL31 GLI2 DAPL1 SENP2 NBEAL1
EEF1B2 MRPL44 MYEOV2 RPL15 CBX5P1 ZNF90P1 RPL24 MRPL47 SDHAP2 RPL35A HOPX
FAM190A RPL34 OSTC RPS3A FBXO8 CDKN2AIP SLC38A9 NDUFAF2 ADAMTS6
ZFYVE16 CAMLG RPL26L1 TMEM14C TMEM14B TBCC RP13 EEF1A1 RP1 TOMM7 GBAS
MIR1255B1 RPS3AP26 HBP1 TRIM35 EFCAB1 RDH10 TCEB1 PAF1
POLR2K EIF3E TTC35 EIF3H RPS6 LINC00032 NDUFB6 ZNF658 KLF9
NCBP1 ATP6V1G1 ASB6 ANAPC16 CHCHD1 RPS24 MARK2P9 C10orf12
C11orf58 RPS13 EIF3M VWCE MALAT1 PAK1 CCDC90B RPS25 U6
TAS2R18 PFDN5 ANKS1B PSMD9 TPT1 EBPL THSD1 COMMD6 CLYBL-AS2 NGDN
EAPP PSMA6 KLHDC2 PSMA3 MOAP1 BAG5 RSL24D1 RPS3AP6 RPS27L WDR61
MORF4L1 MRPL46 SPSB3 RPS15A LYRM1 TMED6 MAP1LC3B RPL26 ZNF286B
RPS7P1 RPL17 RPL19 RPL27 HIGD1B CTD ICT1 ZFP161 WDR7 PQLC1
HSBP1L1 CAPS WDR83 ZNF350 ZNF460 GCNT1P1 PSMA7 SNRPD3 PHF5A EEF1B2P3
COX7B
GOBPID Pvalue OddsRatio ExpCount Count Size Term
GO:0006414 7.00108156243048e-28 46.5087918321044 0.791986713722234
23 105 translational elongation
GO:0006415 9.9573812581339e-28 52.3081792437115 0.686388485225936
22 91 translational termination
GO:0006413 6.13779952928328e-27 33.3122422197225 1.14649505224552
25 152 translational initiation
GO:0000184 1.59603668113705e-26 39.687257751938 0.897584942218532 23
119 nuclear-transcribed mRNA catabolic process, nonsense-mediated decay
GO:0019080 1.70098294364476e-25 31.6035201482168 1.13895232163864
24 151 viral genome expression
GO:0019083 1.70098294364476e-25 31.6035201482168 1.13895232163864
24 151 viral transcription
GO:0006614 1.35355223956287e-24 40.5056818181818 0.791986713722234
21 105 SRP-dependent cotranslational protein targeting to membrane
GO:0006613 1.67808866830512e-24 40.0263368983957 0.799529444329112
21 106 cotranslational protein targeting to membrane
GO:0045047 1.67808866830512e-24 40.0263368983957 0.799529444329112
21 106 protein targeting to ER
GO:0072599 1.67808866830512e-24 40.0263368983957 0.799529444329112
21 106 establishment of protein localization to endoplasmic reticulum
GO:0072594 1.2479011519117e-23 23.5484836392658 1.5387170438032 25
204 establishment of protein localization to organelle
GO:0019058 1.35404133555629e-23 21.6022333235381 1.74237077018891
26 231 viral infectious cycle
GO:0070972 1.8718659352694e-23 35.0451030927835 0.890042211611653
21 118 protein localization to endoplasmic reticulum
GO:0000956 7.73126774786728e-23 26.0041414463205 1.27472147256245
23 169 nuclear-transcribed mRNA catabolic process
GO:0043624 2.08118992313669e-22 27.4319557778363 1.1540377828524 22
153 cellular protein complex disassembly
GO:0006402 2.63003009516724e-22 24.4786946736684 1.34260604802436
23 178 mRNA catabolic process
GO:0022415 2.94732327295664e-22 18.8862115127175 1.96110995778839
26 260 viral reproductive process
GO:0006401 3.26019612331964e-22 22.0906077348066 1.54625977441008
24 205 RNA catabolic process
GO:0043241 4.32304443474305e-22 26.4141311697093 1.19175143588679
22 158 protein complex disassembly
GO:0034623 3.80972730832128e-21 23.6070780399274 1.31243512559684
22 174 cellular macromolecular complex disassembly
GO:0006612 5.61471104620423e-21 25.6895661157025 1.1540377828524 21
153 protein targeting to membrane
GO:0032984 7.18547446031269e-21 22.8472069697635 1.35014877863124
22 179 macromolecular complex disassembly
GO:0016032 1.63195272019299e-20 12.5569018912174 3.40177150370217
30 451 viral reproduction

GO:0071845	1.16772050414388e-17	14.4285396061659	2.14213549235347
23	284	cellular component disassembly at cellular level
GO:0022411	1.59447401062236e-17	14.2067134708205	2.17230641478098
23	288	cellular component disassembly
GO:0033365	8.80019870315439e-15	9.23017644557823	3.56771157705349
25	473	protein localization to organelle
GO:0006605	9.23680250813398e-15	9.20895641107222	3.57525430766037
25	474	protein targeting
GO:0022613	1.39770626050915e-08	9.02564858490566	1.69711438654764
13	225	ribonucleoprotein complex biogenesis
GO:0071843	2.8672468897145e-08	8.45814896755162	1.80271261504394
13	239	cellular component biogenesis at cellular level
GO:0042274	1.76388272266686e-07	52.9918639053254	0.135769150923812
5	18	ribosomal small subunit biogenesis
GO:0042254	1.7878479343382e-07	10.3211975873846	1.12386686042488
10	149	ribosome biogenesis
GO:0006364	1.14295904297322e-06	11.7541254125413	0.784443983115355
8	104	rRNA processing
GO:0042273	1.44706157301348e-06	68.2571428571429	0.090512767282541
4	12	ribosomal large subunit biogenesis
GO:0016072	1.75139242922031e-06	11.0580469811687	0.829700366756626
8	110	rRNA metabolic process
GO:0051352	1.67224777408421e-05	12.6001765225066	0.543076603695246
6	72	negative regulation of ligase activity
GO:0051444	1.67224777408421e-05	12.6001765225066	0.543076603695246
6	72	negative regulation of ubiquitin-protein ligase activity
GO:0031571	1.81091961104918e-05	12.4112447471381	0.550619334302124
6	73	mitotic cell cycle G1/S transition DNA damage checkpoint
Tissue: Heart_Left_Ventricle=>Skin_Sun_Exposed_Lower_leg	Type:	asymmetric
SourceGene:	MYADM(ENSG00000179820.10)
TargetGeneSet:	PRPF38A	PFDN2	TFB2M	RPS7	PEX13	SNRPG	RPL31	PSMD14
EEF1B2	MYEOV2	RP11	LARS2	CCDC72	RPL35A	ATP5I	BRIP1	C5orf44	RPL26L1
HIST1H2BL	EEF1A1	LYRM2	RP1	GET4	SEC61G	CTB	HBP1	ARMC1
C9orf123	RPS6	CHMP5	YME1L1	NRBF2	CCDC90B	U6	TRIAP1	MRP63
MTMR6	MOAP1	RPS3AP6	FAM96B	ZNHIT3	RPL27	MBD1	LONP1	PSMA7	RPS21
GOBPID	Pvalue	OddsRatio	ExpCount	Count	Size	Term
GO:0006414	5.44827067695416e-12	46.2834051724138	0.27610545982977
9	105	translational elongation
GO:0006614	2.42015652695186e-10	39.3567010309278	0.27610545982977
8	105	SRP-dependent cotranslational protein targeting to membrane
GO:0006613	2.61286891729163e-10	38.9523809523809	0.278735035637672
8	106	cotranslational protein targeting to membrane
GO:0045047	2.61286891729163e-10	38.9523809523809	0.278735035637672
8	106	protein targeting to ER
GO:0072599	2.61286891729163e-10	38.9523809523809	0.278735035637672
8	106	establishment of protein localization to endoplasmic reticulum
GO:0070972	6.19830965847345e-10	34.6739393939394	0.310289945332503
8	118	protein localization to endoplasmic reticulum
GO:0072594	2.13459292520077e-09	22.6281167108753	0.536433464812124
9	204	establishment of protein localization to organelle
GO:0006415	3.3446054642188e-09	38.5188172043011	0.239291398519134
7	91	translational termination
GO:0006612	4.90927691750099e-09	26.24	0.402325098609093	8	153
protein targeting to membrane
GO:0000184	2.19572843448776e-08	28.8326612903226	0.312919521140405
7	119	nuclear-transcribed mRNA catabolic process, nonsense-mediated decay
GO:0019080	1.13802818805091e-07	22.3752240143369	0.397065946993288
7	151	viral genome expression
GO:0019083	1.13802818805091e-07	22.3752240143369	0.397065946993288
7	151	viral transcription
GO:0006413	1.1907172276201e-07	22.2193548387097	0.39969552280119
7	152	translational initiation
GO:0043624	1.24545264686063e-07	22.0656208572691	0.402325098609093

7	153	cellular protein complex disassembly
GO:0043241	1.55212654087642e-07	21.3274941251869	0.415472977648606
7	158	protein complex disassembly
GO:0000956	2.45680923715139e-07	19.8639984070092	0.444398311535534
7	169	nuclear-transcribed mRNA catabolic process
GO:0033365	2.6624087110527e-07	10.7605677260105	1.24378935713791
10	473	protein localization to organelle
GO:0034623	2.99569810090014e-07	19.2625072435774	0.457546190575047
7	174	cellular macromolecular complex disassembly
GO:0006402	3.49557611814805e-07	18.8066402565554	0.468064493806657
7	178	mRNA catabolic process
GO:0032984	3.63097054203649e-07	18.6959864966242	0.47069406961456
7	179	macromolecular complex disassembly
GO:0071845	5.91091697188702e-07	13.6589371980676	0.746799529444329
8	284	cellular component disassembly at cellular level
GO:0022411	6.57065928879444e-07	13.46	0.757317832675939	8	288
cellular component disassembly
GO:0006401	9.06977118532895e-07	16.2113066145324	0.539063040620026
7	205	RNA catabolic process
GO:0016032	1.88937321413605e-06	9.80956467467624	1.18593868936406
9	451	viral reproduction
GO:0019058	2.01535733254347e-06	14.3034274193548	0.607432011625493
7	231	viral infectious cycle
GO:0006605	2.84452989698194e-06	9.30901001112347	1.24641893294582
9	474	protein targeting
GO:0022415	4.41083526082151e-06	12.6380211653704	0.683689710054667
7	260	viral reproductive process
GO:0042274	1.33208946821051e-05	82.2742857142857	0.0473323645422462
3	18	ribosomal small subunit biogenesis
Tissue: Heart_Left_Ventricle=>Skin_Sun_Exposed_Lower_leg	Type:	cluster
SourceGene:	NUDT12(ENSG00000112874.5)
TargetGeneSet:	MTND2P28	RPL22	PLOD1	DMAP1	RPS8	FGGY	RPL5	GAS5
DPYSL5	UBC	MYO3B	NGEF	HDLBP	RPL15	ZBTB20	RYK	GOLIM4	EXOSC9
RPS3A	NPY1R	C4orf27	CDKN2AIP	VDAC1	EEF1A1	RP11	MESTP1	STX7
CCT6P1	SBDSP1	RPS3AP26	DDHD2	C8orf40	HOOK3	VPS28	RPS6	EEF1A1P5
NRG3	PRG2	C11orf31	FAM55D	TAS2R18	RPL6	RIMBP2	KIAA1704
PCDH9	SNX6	AP5M1	IFT43	SPSB3	GPT2	SPG7	RPL26	RPL19	FAM195B	CTD
RPL17	TNFRSF11A	CCDC130	KXD1	ZNF208	RPL13A	ZNF350	OLIG1	EIF3D
TMPRSS6	TBC1D22A	RBM3	RP3	TBC1D8B	MT
GOBPID	Pvalue	OddsRatio	ExpCount	Count	Size	Term
GO:0006414	4.83871524356167e-17	50.5375	0.385094457130994	13	105
translational elongation
GO:0006415	3.96725263306637e-16	53.0497066995986	0.333748529513528
12	91	translational termination
GO:0006614	2.36517683263292e-15	45.0196695515342	0.385094457130994
12	105	SRP-dependent cotranslational protein targeting to membrane
GO:0006613	2.66009195205438e-15	44.5376232485729	0.388762023389385
12	106	cotranslational protein targeting to membrane
GO:0045047	2.66009195205438e-15	44.5376232485729	0.388762023389385
12	106	protein targeting to ER
GO:0072599	2.66009195205438e-15	44.5376232485729	0.388762023389385
12	106	establishment of protein localization to endoplasmic reticulum
GO:0006413	6.71973677287656e-15	33.3393884892086	0.557470071275344
13	152	translational initiation
GO:0070972	9.99107432151821e-15	39.462494247584	0.43277281849007	12
118	protein localization to endoplasmic reticulum
GO:0000184	1.10822942350723e-14	39.0909505356736	0.43644038474846
12	119	nuclear-transcribed mRNA catabolic process, nonsense-mediated decay
GO:0000956	2.69407286782552e-14	29.6708333333333	0.619818697667981
13	169	nuclear-transcribed mRNA catabolic process
GO:0006402	5.29469460127444e-14	28.034696969697	0.652826793993495	13
178	mRNA catabolic process
GO:0019080	2.01042907346556e-13	30.0242147745218	0.553802505016954

12	151	viral genome expression
GO:0019083	2.01042907346556e-13	30.0242147745218	0.553802505016954
12	151	viral transcription
GO:0006612	2.35603391360195e-13	29.5941878567722	0.561137637533735
12	153	protein targeting to membrane
GO:0043624	2.35603391360195e-13	29.5941878567722	0.561137637533735
12	153	cellular protein complex disassembly
GO:0006401	3.28789564667675e-13	24.0466145833333	0.751851082970037
13	205	RNA catabolic process
GO:0043241	3.46951584994278e-13	28.570664884731 0.579475468825687	12
158	protein complex disassembly
GO:0034623	1.1017886676168e-12	25.7199638663053	0.638156528959934
12	174	cellular macromolecular complex disassembly
GO:0019058	1.51714240944642e-12	21.1399082568807	0.847207805688188
13	231	viral infectious cycle
GO:0032984	1.54458173719597e-12	24.9411421060318	0.656494360251886
12	179	macromolecular complex disassembly
GO:0022415	6.80504659985568e-12	18.6197368421053	0.95356722718151
13	260	viral reproductive process
GO:0072594	7.27518368965008e-12	21.655487804878 0.748183516711646	12
204	establishment of protein localization to organelle
GO:0071845	3.42610342688023e-10	15.200143472023 1.04158881738288	12
284	cellular component disassembly at cellular level
GO:0022411	4.02264026677077e-10	14.9756097560976	1.05625908241644
12	288	cellular component disassembly
GO:0033365	9.9851388680155e-10	10.9014021563041	1.73475884021867
14	473	protein localization to organelle
GO:0016032	6.04874592179812e-09	10.3584474885845	1.65407238253408
13	451	viral reproduction
GO:0006605	1.09617153304727e-08	9.82543383947939	1.73842640647706
13	474	protein targeting
GO:0016197	9.12150819894347e-06	14.2320478723404	0.491453878624317
6	134	endosomal transport
GO:0022613	1.69430301796759e-05	9.89828480255285	0.825202408137845
7	225	ribonucleoprotein complex biogenesis
Tissue: Skin_Sun_Exposed_Lower_leg=>Heart_Left_Ventricle	Type:	asymmetric
SourceGene:	PGRMC1(ENSG00000101856.8)
TargetGeneSet:	TRAPPC12	TCEB1P21	RPL37A	KLHL30	RPL32	RPL29	ERC2
RP11	LYSMD3	RPS14	RPS10	MRPS12	RP3	ELP3	TMEM65	RPL8	DNLZ	CST6
SETP17	C11orf75	MRPS36P4	CACNA1C	RILPL1	RPL13	OR3A3	CCL2
RPS15	RPL36	RPL18	RPS11	RPS5	RPS21
GOBPID	Pvalue	OddsRatio	ExpCount	Count	Size	Term
GO:0006415	2.36525599733211e-23	217.409090909091	0.151131409591032
13	91	translational termination
GO:0006414	1.70532207841082e-22	184.145256916996	0.17438239568196
13	105	translational elongation
GO:0006614	1.70532207841082e-22	184.145256916996	0.17438239568196
13	105	SRP-dependent cotranslational protein targeting to membrane
GO:0006613	1.94231758806374e-22	182.152492668622	0.17604318040274
13	106	cotranslational protein targeting to membrane
GO:0045047	1.94231758806374e-22	182.152492668622	0.17604318040274
13	106	protein targeting to ER
GO:0072599	1.94231758806374e-22	182.152492668622	0.17604318040274
13	106	establishment of protein localization to endoplasmic reticulum
GO:0070972	8.41874403674507e-22	161.2	0.195972597052107	13	118
protein localization to endoplasmic reticulum
GO:0000184	9.44450207397793e-22	159.668096054889	0.197633381772888
13	119	nuclear-transcribed mRNA catabolic process, nonsense-mediated decay
GO:0019080	2.36729203985515e-20	122.369565217391	0.250778492837866
13	151	viral genome expression
GO:0019083	2.36729203985515e-20	122.369565217391	0.250778492837866
13	151	viral transcription
GO:0006413	2.58684652205297e-20	121.480706344016	0.252439277558646

13	152	translational initiation
GO:0006612	2.82503658286128e-20	120.604545454545	0.254100062279427
13	153	protein targeting to membrane
GO:0043624	2.82503658286128e-20	120.604545454545	0.254100062279427
13	153	cellular protein complex disassembly
GO:0043241	4.34944305560692e-20	116.405015673981	0.26240398588333
13	158	protein complex disassembly
GO:0019058	8.15435952605171e-20	91.6774193548387	0.383641270500311
14	231	viral infectious cycle
GO:0000956	1.07082145951018e-19	108.113636363636	0.280672617811916
13	169	nuclear-transcribed mRNA catabolic process
GO:0034623	1.58064540448204e-19	104.719367588933	0.288976541415819
13	174	cellular macromolecular complex disassembly
GO:0006402	2.14045600967934e-19	102.152066115702	0.295619680298941
13	178	mRNA catabolic process
GO:0032984	2.30643262079744e-19	101.529572836802	0.297280465019722
13	179	macromolecular complex disassembly
GO:0022415	4.38531556145992e-19	80.7048780487805	0.431804027402948
14	260	viral reproductive process
GO:0072594	1.3107907802754e-18	88.0856734888148	0.338800083039236
13	204	establishment of protein localization to organelle
GO:0006401	1.39854045986023e-18	87.6207386363636	0.340460867760017
13	205	RNA catabolic process
GO:0071845	1.02100164815464e-16	61.7336464273734	0.471662860701682
13	284	cellular component disassembly at cellular level
GO:0022411	1.22587654717886e-16	60.8185123966942	0.478305999584804
13	288	cellular component disassembly
GO:0016032	1.00514907255067e-15	44.8192219679634	0.749013909072037
14	451	viral reproduction
GO:0033365	7.55931581953024e-14	35.8835968379447	0.785551172929209
13	473	protein localization to organelle
GO:0006605	7.76682818723057e-14	35.8031946361664	0.78721195764999
13	474	protein targeting
GO:0042274	3.23105168313163e-06	137.257142857143	0.0298941249740502
3	18	ribosomal small subunit biogenesis
Tissue: Skin_Sun_Exposed_Lower_leg=>Heart_Left_Ventricle	Type:	cluster
SourceGene:	RP11-16C18.3(ENSG00000237027.1)
TargetGeneSet:	CEP104	RP13	RPL11	RPL5	ACBD6	BCL11A	LRRTM4	COX5B
RPL31	RPL15	RPL24	RP11	BCL6	RPL35A	BRIX1	CTD	SOX9	ARHGEF37
SSR1	C6orf106		MRPL2	MRPS12	SEC61B	CTSL1P7	LCOR	FAM160B1
RPLP2	EIF3F	RPS13	FAU	RPS25	ZBTB44	RAPGEF3	RPL14P1	SNRPF	RPS2P5
LINC00427		UCHL3	MRPL52	AP1G2	SCFD1	RPS29	SLIRP	LINC00521		RPL4
AKAP13	HN1L	U6	WWOX	RPL26	NT5M	SLC47A1	RPL23A	RPL17	RPL19
ALYREF	RPL36	UBA52	PDCD2L	RPL18	HSPA13	EP300	SMS	EEF1B2P3
PHF16	RPS4X	UTP14A
GOBPID	Pvalue	OddsRatio	ExpCount	Count	Size	Term
GO:0006614	3.29506663625902e-34	122.397979012826	0.385094457130994
22	105	SRP-dependent cotranslational protein targeting to membrane
GO:0006613	4.14947076419811e-34	120.932411674347	0.388762023389385
22	106	cotranslational protein targeting to membrane
GO:0045047	4.14947076419811e-34	120.932411674347	0.388762023389385
22	106	protein targeting to ER
GO:0072599	4.14947076419811e-34	120.932411674347	0.388762023389385
22	106	establishment of protein localization to endoplasmic reticulum
GO:0070972	5.52840024039884e-33	105.727150537634	0.43277281849007
22	118	protein localization to endoplasmic reticulum
GO:0006415	1.76933814408103e-31	122.296201451131	0.333748529513528
20	91	translational termination
GO:0006612	2.57782576196683e-30	77.2898300911106	0.561137637533735
22	153	protein targeting to membrane
GO:0006414	4.14342998131729e-30	102.053475935829	0.385094457130994
20	105	translational elongation
GO:0000184	6.25253485109073e-29	87.5359657177839	0.43644038474846

20	119	nuclear-transcribed mRNA catabolic process, nonsense-mediated decay
GO:0019080	1.43194412988401e-28	72.0259615384615	0.553802505016954
21	151	viral genome expression
GO:0019083	1.43194412988401e-28	72.0259615384615	0.553802505016954
21	151	viral transcription
GO:0006413	1.65794688744108e-28	71.4711354961832	0.557470071275344
21	152	translational initiation
GO:0072594	1.9617378947663e-27	55.4328252392769	0.748183516711646
22	204	establishment of protein localization to organelle
GO:0043624	1.31050972554044e-26	65.0034176349966	0.561137637533735
20	153	cellular protein complex disassembly
GO:0043241	2.57408670569871e-26	62.6262626262626	0.579475468825687
20	158	protein complex disassembly
GO:0000956	1.05117443232579e-25	57.9581045352857	0.619818697667981
20	169	nuclear-transcribed mRNA catabolic process
GO:0034623	1.92904013762762e-25	56.0566706021251	0.638156528959934
20	174	cellular macromolecular complex disassembly
GO:0006402	3.09348793557785e-25	54.6221710778673	0.652826793993495
20	178	mRNA catabolic process
GO:0032984	3.47494743442156e-25	54.274823708786 0.656494360251886	20
179	macromolecular complex disassembly
GO:0022415	4.70508112989575e-25	42.2228246137165	0.95356722718151
22	260	viral reproductive process
GO:0019058	1.53164327185711e-24	44.3375 0.847207805688188	21	231
viral infectious cycle
GO:0006401	5.71295170166455e-24	46.5618345618346	0.751851082970037
20	205	RNA catabolic process
GO:0033365	4.16162608129544e-22	25.7104677060134	1.73475884021867
24	473	protein localization to organelle
GO:0006605	4.37487273719778e-22	25.6514942528736	1.73842640647706
24	474	protein targeting
GO:0071845	4.27362753063403e-21	32.4471992653811	1.04158881738288
20	284	cellular component disassembly at cellular level
GO:0022411	5.65794276077651e-21	31.9538670284939	1.05625908241644
20	288	cellular component disassembly
GO:0016032	8.54399763582425e-20	23.1083540115798	1.65407238253408
22	451	viral reproduction
GO:0042273	7.80863562374655e-08	146.836734693878	0.0440107951006851
4	12	ribosomal large subunit biogenesis
GO:0022613	1.12022401523491e-07	13.4299242424242	0.825202408137845
9	225	ribonucleoprotein complex biogenesis
GO:0071843	1.87325294137031e-07	12.6	0.876548335755311	9	239
cellular component biogenesis at cellular level
GO:0042254	1.11473650659671e-06	15.2774035517453	0.546467372500173
7	149	ribosome biogenesis
Tissue: Heart_Left_Ventricle=>Skin_Sun_Exposed_Lower_leg	Type:	cluster
SourceGene:	RP11-332L8.1(ENSG00000237861.1)
TargetGeneSet:	RP11	RP4	RBM8A	MIR4426	ST13P19	ZC3H11B	YPEL5	DPY30
RPL31	PSMD14	RPL37A	CRBN	RPL32	ATG3	RPL35A	PRSS12	CTD	RPL37	NSA2
PHAX	DCTN4	TTC1	RPL24P4	ZBTB2	BCL7B	NYAP1	WASL	SORBS3	RPL30
UTP23	C9orf78	UCK1	SRP9P1	RPL13AP5		NAV2-AS1		TAS2R20	RPL14P1
RILPL2	RPS7P10	RNF6	UPF3A	NDNL2	SELS	EIF3C	CD2BP2	AMFR	RPS2P46
RPL19	ORMDL3	DHX40	RPS16	EID2	RPS11	EEF1B2P3		GPKOW	ARMCX1	RP3
RP13	DAZ3
GOBPID	Pvalue	OddsRatio	ExpCount	Count	Size	Term
GO:0000184	1.89169548238353e-14	48.5561728395062	0.337623693862016
11	119	nuclear-transcribed mRNA catabolic process, nonsense-mediated decay
GO:0000956	9.40718587582297e-13	33.0742616033755	0.479482388762023
11	169	nuclear-transcribed mRNA catabolic process
GO:0006402	1.66441886117584e-12	31.2720558882236	0.505016953844025
11	178	mRNA catabolic process
GO:0006415	3.0848556803244e-12	49.1432926829268	0.258182824718013
9	91	translational termination

GO:0006401	7.79345903785994e-12	26.8687285223368	0.581620649090028
11	205	RNA catabolic process
GO:0006413	1.0429104271136e-11	32.412539754657 0.431250432496021	10
152	translational initiation
GO:0006612	1.11364910838922e-11	32.1836228287841	0.434087606394021
10	153	protein targeting to membrane
GO:0006414	1.1500479901178e-11	41.935546875	0.297903259290015	9
105	translational elongation
GO:0006614	1.1500479901178e-11	41.935546875	0.297903259290015	9
105	SRP-dependent cotranslational protein targeting to membrane
GO:0006613	1.25423704941304e-11	41.5003221649485	0.300740433188015
9	106	cotranslational protein targeting to membrane
GO:0045047	1.25423704941304e-11	41.5003221649485	0.300740433188015
9	106	protein targeting to ER
GO:0072599	1.25423704941304e-11	41.5003221649485	0.300740433188015
9	106	establishment of protein localization to endoplasmic reticulum
GO:0070972	3.33367514143575e-11	36.9005160550459	0.334786519964016
9	118	protein localization to endoplasmic reticulum
GO:0019080	3.0780454364018e-10	28.2596830985915	0.428413258598021
9	151	viral genome expression
GO:0019083	3.0780454364018e-10	28.2596830985915	0.428413258598021
9	151	viral transcription
GO:0043624	3.46231979714923e-10	27.86328125	0.434087606394021	9
153	cellular protein complex disassembly
GO:0043241	4.61374173750134e-10	26.9188338926174	0.448273475884022
9	158	protein complex disassembly
GO:0034623	1.08778441201842e-09	24.28125	0.493668258252024	9
174	cellular macromolecular complex disassembly
GO:0032984	1.39799328206266e-09	23.5588235294118	0.507854127742025
9	179	macromolecular complex disassembly
GO:0072594	4.4227488584892e-09	20.5024038461538	0.578783475192028
9	204	establishment of protein localization to organelle
GO:0019058	1.30952947058241e-08	17.9746621621622	0.655387170438032
9	231	viral infectious cycle
GO:0022415	3.6450655186889e-08	15.8654133466135	0.737665213480036
9	260	viral reproductive process
GO:0071845	7.7797305744964e-08	14.45625	0.805757387032039	9
284	cellular component disassembly at cellular level
GO:0022411	8.76806949627709e-08	14.2449596774194	0.81710608262404
9	288	cellular component disassembly
GO:0016032	3.73388083111285e-07	10.2179796649843	1.27956542799806
10	451	viral reproduction
GO:0006605	5.89368934217687e-07	9.69549499443827	1.34482042765207
10	474	protein targeting
GO:0022613	2.89559201436361e-06	13.4031300593632	0.638364127050031
7	225	ribonucleoprotein complex biogenesis
GO:0071843	4.31571459631471e-06	12.5818965517241	0.678084561622033
7	239	cellular component biogenesis at cellular level
GO:0033365	5.50598763044479e-06	8.45325969827586	1.34198325375407
9	473	protein localization to organelle
Tissue: Skin_Sun_Exposed_Lower_leg=>Heart_Left_Ventricle	Type:	asymmetric
SourceGene:	RP11-697K23.1(ENSG00000230530.1)
TargetGeneSet:	RPL11	RP4	HSPB11	RP11	ISG20L2	SNRPE	LINC00467
RPL31	UBE2V1	RPL37A	MRPS36P1		CCDC72	RPL35A	FGFBP1	RPL37	CTD
ZMAT2	CTB	HMGN3	HOXA10	PPIA	ECD	SNHG6	RPL7	C8orf59	RPL30
EIF5AL1	LGI1	RPL13AP5		RPS13	RPS25	PFDN5	NDUFA12	RPS7P10	RPL7AP6
SLIRP	SLC9A5	RPL26	RPL17	RPL38	ZNF446	ZNF788	C19orf43		CHMP4B
ZFP64	ZNF280B	EEF1B2P3
GOBPID	Pvalue	OddsRatio	ExpCount	Count	Size	Term
GO:0006415	1.3235913401609e-20	108.621212121212	0.220399972320255
13	91	translational termination
GO:0006414	9.4483628916079e-20	92.001976284585 0.254307660369525	13
105	translational elongation

GO:0006614	8.23690392874817e-18	80.3534361851332	0.254307660369525	12	105	SRP-dependent cotranslational protein targeting to membrane
GO:0006613	9.2746953447363e-18	79.4930619796485	0.25672963808733	12	106	cotranslational protein targeting to membrane
GO:0045047	9.2746953447363e-18	79.4930619796485	0.25672963808733	12	106	protein targeting to ER
GO:0072599	9.2746953447363e-18	79.4930619796485	0.25672963808733	12	106	establishment of protein localization to endoplasmic reticulum
GO:0043624	1.51258162845701e-17	60.2558441558442	0.370562590824164	13	153	cellular protein complex disassembly
GO:0043241	2.32048493093861e-17	58.1576802507837	0.382672479413189	13	158	protein complex disassembly
GO:0070972	3.53222326000842e-17	70.4347826086957	0.28579337070099	12	118	protein localization to endoplasmic reticulum
GO:0000184	3.92255015796649e-17	69.7716375457131	0.288215348418795	12	119	nuclear-transcribed mRNA catabolic process, nonsense-mediated decay
GO:0034623	8.33715556476071e-17	52.3193111236589	0.421424122898069	13	174	cellular macromolecular complex disassembly
GO:0032984	1.21219596404493e-16	50.7256297918949	0.433534011487094	13	179	macromolecular complex disassembly
GO:0019080	7.38464718823052e-16	53.588989677823	0.365718635388554	12	151	viral genome expression
GO:0019083	7.38464718823052e-16	53.588989677823	0.365718635388554	12	151	viral transcription
GO:0006413	8.00573661742737e-16	53.2024844720497	0.368140613106359	12	152	translational initiation
GO:0006612	8.67420286888113e-16	52.8214616096207	0.370562590824164	12	153	protein targeting to membrane
GO:0000956	2.9167203549835e-15	47.3852118526724	0.409314234309044	12	169	nuclear-transcribed mRNA catabolic process
GO:0019058	3.43206911950644e-15	38.4849874895746	0.559476852812954	13	231	viral infectious cycle
GO:0006402	5.47603091337228e-15	44.7878470403353	0.431112033769289	12	178	mRNA catabolic process
GO:0022415	1.59263847671525e-14	33.8971291866029	0.629714206629299	13	260	viral reproductive process
GO:0072594	2.84182596419508e-14	38.6521739130435	0.494083454432219	12	204	establishment of protein localization to organelle
GO:0006401	3.01403382904676e-14	38.4492002703312	0.496505432150024	12	205	RNA catabolic process
GO:0071845	4.97717584360643e-14	30.8428379738343	0.687841671856619	13	284	cellular component disassembly at cellular level
GO:0022411	5.95874574839357e-14	30.3856198347107	0.697529582727839	13	288	cellular component disassembly
GO:0016032	8.67784210839148e-13	21.3257055682685	1.09231195073005	14	451	viral reproduction
GO:0033365	5.53917792190179e-10	15.7936433084976	1.14559546052176	12	473	protein localization to organelle
GO:0006605	5.67447946559664e-10	15.7583286278938	1.14801743823957	12	474	protein targeting
GO:0042273	1.40745924811973e-08	232.387096774194	0.02906373261366	4	12	ribosomal large subunit biogenesis
GO:0022613	4.99760970587808e-08	19.3876088069636	0.544944986506124	8	225	ribonucleoprotein complex biogenesis
GO:0071843	7.97107515811943e-08	18.1946448613115	0.578852674555394	8	239	cellular component biogenesis at cellular level
GO:0042254	1.37762246487303e-06	20.6505907885218	0.360874679952944	6	149	ribosome biogenesis

Tissue: Whole_Blood=>Heart_Left_Ventricle	Type:	asymmetric

SourceGene: CAMK1D(ENSG00000183049.8) calcium/calmodulin-dependent protein kinase ID

TargetGeneSet: ATP6V0B RP11 HFE2 RNF115 NES CCL15 ASPM CENPF EIF2AK3 BUB1 EXOG ZNF595 CD38 NADKD1 RHOBTB3 HSPA9 ARHGAP26 SH3PXD2B LRRC16A TNFRSF21 UBE3D EGFR GATSL2 GNRH1 PBK COBRA1 IL2RA CDK1 PBLD PDLIM1 NT5C2 C11orf92 C11orf93 VPS26B TROAP MIS18BP1 DLGAP5 VRK1 ARHGAP11A BUB1B CASC5 PRC1 VPS35 CRISPLD2 MIR22HG TOP2A CCDC40 TPX2

GOBPID	Pvalue	OddsRatio	ExpCount	Count	Size	Term
GO:0000280	4.14E-09	14.26613201	1.041450419	11	350	nuclear division
GO:0007067	4.14E-09	14.26613201	1.041450419	11	350	mitosis
GO:0000087	5.55E-09	13.84751074	1.071206145	11	360	M phase of mitotic cell cycle
GO:0048285	7.38E-09	13.452211	1.100961871	11	370	organelle fission
GO:0051301	5.78E-08	10.88697562	1.344958826	11	452	cell division
GO:0071173	2.95E-06	49.15555556	0.101169469	4	34	spindle assembly checkpoint
GO:0031577	7.01E-06	38.7854251	0.12497405	4	42	spindle checkpoint

GO:0030071	7.71E-06	37.78829717	0.127949623	4	43
regulation of mitotic metaphase/anaphase transition

GO:0007091	9.27E-06	35.93996248	0.133900768	4	45
mitotic metaphase/anaphase transition

Tissue: Whole_Blood=>Heart_Left_Ventricle	Type:	cluster

SourceGene:	CCDC144A(ENSG00000170160.10)	coiled-coil domain containing 144A

TargetGeneSet:	KHDRBS1 GNG5	HBXIP	MDM4	MOGS	SUCLG1	TSPO	NDUFB3
RPL32	KIAA2018		UBXN7	NDUFC1	NDUFS6	JMY	RPL39P3 COQ3	REV3L	RP11
RNASET2 RBAK	SRI	RBM48	GIGYF1	DOCK5	RPL30	CYC1	NDUFB6	RPL35	U8
ZNF22	POLR3A	RPL13AP5		RPL27A	NDUFS3	TRMT112 FAU	NUMA1	C11orf67
USP35	C11orf1 FOXRED1 DHX37	RPS29	PROX2	SLIRP	C15orf57		ANP32A
GOLGA6L4		SLC9A5	C1QBP	LSMD1	CCDC144A		FAM106A SRP68P2 FBXL20	GJC1
SNRPD1	RPS15	RPL36	MRPL4	C19orf43		MAU2	PDCD2L	MYH7B	RPL28
RPS21	IFNGR2	ATP5O	COX7B	IDH3G

GOBPID	Pvalue	OddsRatio	ExpCount	Count	Size	Term

GO:0006415	7.52E-13	42.08700764	0.327451387	10	91
translational termination

GO:0006414	3.25E-12	35.84962406	0.377828524	10	105

translational elongation

GO:0006614	3.25E-12	35.84962406	0.377828524	10	105

SRP-dependent cotranslational protein targeting to membrane

GO:0006613	3.58E-12	35.47371032	0.381426891	10	106

cotranslational protein targeting to membrane

GO:0045047	3.58E-12	35.47371032	0.381426891	10	106

protein targeting to ER

GO:0072599	3.58E-12	35.47371032	0.381426891	10	106

establishment of protein localization to endoplasmic reticulum

GO:0070972	1.06E-11	31.50573192	0.424607294	10	118

protein localization to endoplasmic reticulum

GO:0000184	1.16E-11	31.21450415	0.428205661	10	119

"nuclear-transcribed mRNA catabolic process, nonsense-mediated decay"

GO:0045333	6.78E-11	25.73412698	0.510968099	10	142

cellular respiration

GO:0072594	1.23E-10	19.7480096	0.734066847	11	204	

establishment of protein localization to organelle

GO:0019080	1.25E-10	24.07632557	0.543353401	10	151	

viral genome expression

GO:0019083	1.25E-10	24.07632557	0.543353401	10	151	

viral transcription

GO:0006401	1.30E-10	19.64483279	0.737665213	11	205	RNA

catabolic process

GO:0006413	1.33E-10	23.90509725	0.546951768	10	152	

translational initiation

GO:0006612	1.42E-10	23.73626374	0.550550135	10	153	

protein targeting to membrane

GO:0043624	1.42E-10	23.73626374	0.550550135	10	153	

cellular protein complex disassembly

GO:0043241	1.96E-10	22.92631918	0.568541969	10	158	protein complex disassembly
GO:0000956	3.79E-10	21.32374963	0.608124005	10	169	nuclear-transcribed mRNA catabolic process
GO:0019058	4.66E-10	17.29146341	0.831222753	11	231	viral infectious cycle
GO:0034623	5.05E-10	20.66637631	0.62611584	10	174	cellular macromolecular complex disassembly
GO:0006402	6.31E-10	20.16865079	0.640509307	10	178	mRNA catabolic process
GO:0032984	6.67E-10	20.04790082	0.644107674	10	179	macromolecular complex disassembly
GO:0022415	1.63E-09	15.24635126	0.935575393	11	260	

viral reproductive process

GO:0022904	1.67E-09	29.56818182	0.345443222	8	96

respiratory electron transport chain

GO:0015980	9.26E-09	12.78287739	1.104698637	11	307

energy derivation by oxidation of organic compounds

GO:0022900	2.37E-08	20.5959596	0.482181164	8	134

electron transport chain

GO:0071845	5.60E-08	12.27407021	1.021936198	10	284

cellular component disassembly at cellular level

GO:0022411	6.39E-08	12.09403905	1.036329666	10	288

cellular component disassembly

GO:0006120	1.72E-07	49.30679478	0.129541208	5	36

"mitochondrial electron transport, NADH to ubiquinone"

GO:0006091	2.74E-07	8.995847225	1.540101031	11	428

generation of precursor metabolites and energy

GO:0016032 4.61E-07 8.511585366 1.62286347 11 451
viral reproduction

GO:0042773 6.78E-07 36.36524823 0.169123244 5 47 ATP
synthesis coupled electron transport

GO:0042775 6.78E-07 36.36524823 0.169123244 5 47
mitochondrial ATP synthesis coupled electron transport

GO:0033365 7.38E-07 8.093495935 1.702027541 11 473
protein localization to organelle

GO:0006605 7.54E-07 8.075435916 1.705625908 11 474
protein targeting

GO:0006119 1.97E-06 28.79566439 0.20870528 5 58
oxidative phosphorylation

Tissue: Whole_Blood=>Heart_Left_Ventricle Type: asymmetric

SourceGene: CLEC5A(ENSG00000258227.2) "C-type lectin domain family 5, member A"

TargetGeneSet: WRAP73 THAP3 H6PD ZBTB17 KIF2C NOTCH2NL JTB IQGAP3 CCDC19 ASPM KIF14 GALNT3 ILKAP FANCD2 CD38 GLRB KIF20A CDC25C ARHGAP26 ZNF193 KIFC1 RP11 NDUFAF4 VIP CCT6A SND1 PBK GINS4 GKAP1 CDK1 PBLD MKI67 U6 CDCA5 SIDT2 ZNF202 TROAP PLBD2 DLGAP5 GSTZ1 ALKBH1 ARHGAP11A FMN1 BUB1B RAD51 NUSAP1 FANCI PRC1 SULT1A2 WWOX ATP6V0CP1 AURKB C17orf108 TOP2A KIF18B PRR11 BRIP1 QRICH2 BIRC5 THOC1 ATP9B PIAS4 ZGLP1 IRF2BP1 TPX2 RP3 GTSE1 RBM3 KIF4A

GOBPID	Pvalue	OddsRatio	ExpCount	Count	Size	Term
GO:0000087	1.16E-12	14.51782946	1.51961802	16	360	M phase of mitotic cell cycle
GO:0000280	1.10E-11	13.68105127	1.477406408	15	350	nuclear division
GO:0007067	1.10E-11	13.68105127	1.477406408	15	350	mitosis
GO:0048285	2.43E-11	12.89191672	1.561829631	15	370	organelle fission

GO:0051301	3.59E-11	11.37940877	1.907964847	16	452	cell division
GO:0007017	6.77E-10	11.01184528	1.658916338	14	393	microtubule-based process
GO:0007059	1.21E-07	16.30417381	0.590962563	8	140	chromosome segregation
GO:0010564	1.28E-07	8.521474261	1.747560722	12	414	regulation of cell cycle process
GO:0007346	4.96E-07	9.433848625	1.279011833	10	303	regulation of mitotic cell cycle
GO:0034501	1.42E-06	248.0517241	0.025326967	3	6	protein localization to kinetochore
GO:0007018	2.14E-06	13.68792867	0.599404885	7	142	microtubule-based movement

GO:0034502	2.40E-06	53.07848569	0.097086707	4	23
protein localization to chromosome

GO:0000819	2.53E-06	27.24734043	0.219500381	5	52
sister chromatid segregation

GO:0000910	2.68E-06	17.72975207	0.39678915	6	94
cytokinesis

GO:0030261	7.25E-06	38.76923077	0.126634835	4	30
chromosome condensation

GO:0000226	1.63E-05	8.171184848	1.135492353	8	269
microtubule cytoskeleton organization

Tissue: Heart_Left_Ventricle=>Whole_Blood	Type:	cluster

SourceGene:	DPP4(ENSG00000197635.5) dipeptidyl-peptidase 4

TargetGeneSet:	RP11	CDCA8	KIF2C	TNFSF13B	CENPF	NCAPH	SMPD4
SPC25	FANCD2	CXCL3	CCNA2	CDC25C	GFPT2	MCM7	EZH2	CDCA2	ESCO2	MCM4
DSCC1	ATAD2	TONSL	ANP32B	SMC2	ATP6V1G1	WDR34	MCM10	ZWINT	CDK1

KIF11 HELLS SMC3 FEN1 CDCA5 H2AFX CHEK1 NCAPD3 MPP2 RACGAP1 RFC3 WDHD1 DLGAP5 CASC5 WDR76 KIF23 FANCI C15orf42 RCCD1 KIF22 HIRIP3 CDT1 HSPB9 PSMC3IP TK1 BIRC5 TYMS SKA1 UHRF1 SPC24 RNASEH2A FKBP1A-SDCBP2 TPX2 AURKA C21orf128 CDC45

GOBPID	Pvalue	OddsRatio	ExpCount	Count	Size	Term
GO:0000280	1.54E-30	42.19490255	1.380527299	28	350	nuclear division
GO:0007067	1.54E-30	42.19490255	1.380527299	28	350	mitosis
GO:0000087	3.44E-30	40.8948899	1.419970936	28	360	M phase of mitotic cell cycle
GO:0048285	7.48E-30	39.67090139	1.459414573	28	370	organelle fission
GO:0010564	1.52E-22	26.11468531	1.632966577	24	414	regulation of cell cycle process
GO:0051301	1.22E-21	23.73152082	1.782852398	24	452	cell division
GO:0006260	2.23E-19	28.40361446	1.057089475	19	268	DNA replication

GO:0007059	4.51E-18	40.76857143	0.55221092	15	140	
chromosome segregation						
GO:0071156	1.22E-16	24.34200405	1.04131202	17	264	
regulation of cell cycle arrest						
GO:0000075	7.32E-16	24.46449385	0.954536018	16	242	cell
cycle checkpoint						
GO:0007050	3.18E-14	16.95411932	1.45547021	17	369	cell
cycle arrest						
GO:0051329	4.93E-14	16.47403315	1.494913847	17	379	
interphase of mitotic cell cycle						
GO:0051325	6.94E-14	16.10864865	1.526468756	17	387	
interphase						
GO:0045786	4.99E-13	14.14035714	1.723686942	17	437	
negative regulation of cell cycle						
GO:0006281	9.33E-13	14.91539842	1.510691302	16	383	DNA

repair

GO:0000236	9.91E-13	40.6212766	0.335270916	10	85	mitotic prometaphase
GO:0007017	1.97E-11	13.24263039	1.550134939	15	393	microtubule-based process
GO:0000226	2.85E-11	16.31693892	1.061033838	13	269	microtubule cytoskeleton organization
GO:0006261	8.90E-11	31.94196429	0.366825825	9	93	DNA-dependent DNA replication
GO:0007346	1.25E-10	14.369279	1.195142205	13	303	regulation of mitotic cell cycle
GO:0000086	2.04E-10	22.64210861	0.567988375	10	144	G2/M transition of mitotic cell cycle
GO:0000070	7.67E-10	47.84	0.193273822	7	49	mitotic sister chromatid segregation

GO:0007051	8.18E-10	32.47619048	0.315549097	8	80	
spindle organization						
GO:0000819	1.18E-09	44.64133333	0.205106913	7	52	
sister chromatid segregation						
GO:0010389	1.59E-09	70.44117647	0.118330911	6	30	
regulation of G2/M transition of mitotic cell cycle						
GO:0007052	2.93E-09	62.60130719	0.130164002	6	33	
mitotic spindle organization						
GO:0051052	4.22E-08	15.06038136	0.73365165	9	186	
regulation of DNA metabolic process						
GO:0071103	4.85E-08	14.80625	0.745484742	9	189	DNA
conformation change						
GO:0006270	7.32E-08	60.07943144	0.110442184	5	28	
DNA-dependent DNA replication initiation						
GO:0030261	1.05E-07	55.26538462	0.118330911	5	30	
chromosome condensation						

GO:0006323	1.40E-07	16.04391274	0.603487648	8	153	DNA packaging
GO:0007076	1.51E-07	120.6289308	0.051276728	4	13	mitotic chromosome condensation
GO:0006271	2.03E-07	47.62931034	0.134108366	5	34	DNA strand elongation involved in DNA replication
GO:0022616	3.16E-07	43.15504808	0.145941457	5	37	DNA strand elongation
GO:0000082	6.25E-07	13.03921119	0.73365165	8	186	G1/S transition of mitotic cell cycle
GO:0031570	1.00E-06	15.48139535	0.536433465	7	136	DNA integrity checkpoint
GO:0050000	1.24E-06	63.82685905	0.082831638	4	21	chromosome localization
GO:0051303	1.24E-06	63.82685905	0.082831638	4	21	establishment of chromosome localization

GO:0051320	1.40E-06	14.67735294	0.564044011	7	143	S phase
GO:0000083	1.51E-06	60.27672956	0.086776002	4	22	regulation of transcription involved in G1/S phase of mitotic cell cycle
GO:0006275	1.58E-06	19.57318741	0.362881461	6	92	regulation of DNA replication
GO:0010948	2.03E-06	18.69803922	0.378658916	6	96	negative regulation of cell cycle process
GO:0006302	2.89E-06	17.52205882	0.402325099	6	102	double-strand break repair
GO:0000076	6.85E-06	114.1825397	0.039443637	3	10	DNA replication checkpoint
GO:0007062	7.20E-06	38.72237197	0.126219639	4	32	sister chromatid cohesion
GO:0070507	1.19E-05	19.39734561	0.299771642	5	76	regulation of microtubule cytoskeleton organization

GO:0010972	1.25E-05	88.7962963	0.047332365	3	12	
negative regulation of G2/M transition of mitotic cell cycle						
GO:0000084	1.40E-05	13.11213235	0.528544737	6	134	S
phase of mitotic cell cycle						
GO:0000216	1.44E-05	18.60706861	0.311604733	5	79	M/G1
transition of mitotic cell cycle						
GO:0090307	1.62E-05	79.91111111	0.051276728	3	13	
spindle assembly involved in mitosis						
GO:0006310	1.71E-05	9.836039604	0.824372016	7	209	DNA
recombination						
GO:0031576	1.79E-05	30.10062893	0.157774548	4	40	G2/M
transition checkpoint						
GO:0007018	1.95E-05	12.33391003	0.560099647	6	142	
microtubule-based movement						
GO:0007098	1.98E-05	29.28505864	0.161718912	4	41	
centrosome cycle						

Tissue: Heart_Left_Ventricle=>Whole_Blood Type: cluster

SourceGene: ENSG00000237298.1

TargetGeneSet: CDCA8 CDC20 KIF2C RWDD3 IQGAP3 TNFSF13B RRM2 IGKJ5 IGKC IGKJ4 IGKJ1 IGKV1-5 IGKV1-9 IGKV1-17 IGKV2-28 NCAPH BUB1 ITM2C HJURP KIF15 CDC25A CCNB1 RP11 MCM7 EZH2 MYOM2 MCM4 WDR34 MCM10 CDK1 ART5 CDCA5 POU2AF1 ABCB9 RFC3 DLGAP5 IFI27L2 IGHA2 IGHG2 IGHGP IGHA1 IGHV6-1 BUB1B CASC5 NUSAP1 FANCI PKMYT1 PLK1 GINS2 AURKB SPAG5 CDC6 TYMS SKA1 PCNA TPX2 MYBL2 CDC45 IGLV4-69 IGLV1-51 IGLV7-46 IGLV7-43 IGLV2-23 IGLV3-19 IGLV2-14 IGLV2-8 IGLL5 IGLJ1 IGLC1 DERL3

GOBPID	Pvalue	OddsRatio	ExpCount	Count	Size	Term
GO:0000280	7.03E-23	33.7114547	1.210988859	22	350	nuclear division
GO:0007067	7.03E-23	33.7114547	1.210988859	22	350	mitosis
GO:0000087	1.31E-22	32.6908284	1.245588541	22	360	M phase of mitotic cell cycle

GO:0048285	2.38E-22	31.72885878	1.280188222	22	370	
organelle fission						
GO:0010564	1.88E-18	23.70050761	1.432426822	20	414	
regulation of cell cycle process						
GO:0007059	9.82E-16	39.48967866	0.484395544	13	140	
chromosome segregation						
GO:0000082	1.31E-15	32.17151163	0.643554079	14	186	G1/S
transition of mitotic cell cycle						
GO:0051329	4.12E-15	19.97848652	1.311327936	17	379	
interphase of mitotic cell cycle						
GO:0051301	4.22E-15	18.10239055	1.563905612	18	452	cell
division						
GO:0051325	5.81E-15	19.53538084	1.339007681	17	387	
interphase						

GO:0000075	5.14E-14	24.17422027	0.837312297	14	242	cell cycle checkpoint
GO:0071156	1.71E-13	22.01266667	0.913431596	14	264	regulation of cell cycle arrest
GO:0000236	2.44E-13	47.75333333	0.294097294	10	85	mitotic prometaphase
GO:0051983	1.27E-12	137.7400821	0.083039236	7	24	regulation of chromosome segregation
GO:0007050	1.60E-11	15.38685446	1.276728254	14	369	cell cycle arrest
GO:0007017	3.70E-11	14.3878628	1.35976749	14	393	microtubule-based process
GO:0006260	8.40E-11	17.44860197	0.927271469	12	268	DNA replication
GO:0000226	8.77E-11	17.37947983	0.930731437	12	269	microtubule cytoskeleton organization

GO:0000083	9.00E-11	122.5994318	0.0761193	6	22	
regulation of transcription involved in G1/S phase of mitotic cell cycle						
GO:0045786	1.51E-10	12.85080116	1.51200609	14	437	
negative regulation of cell cycle						
GO:0007346	3.46E-10	15.31199132	1.048370355	12	303	
regulation of mitotic cell cycle						
GO:0007088	5.96E-10	34.09761905	0.304477199	8	88	
regulation of mitosis						
GO:0051783	5.96E-10	34.09761905	0.304477199	8	88	
regulation of nuclear division						
GO:0006271	1.57E-09	69.99837662	0.117638918	6	34	DNA
strand elongation involved in DNA replication						
GO:0033043	2.12E-09	11.47063652	1.525845962	13	441	
regulation of organelle organization						

GO:0022616	2.70E-09	63.2111437	0.128018822	6	37	DNA strand elongation
GO:0034502	1.30E-08	88.78395062	0.079579268	5	23	protein localization to chromosome
GO:0000070	1.57E-08	45.53276956	0.16953844	6	49	mitotic sister chromatid segregation
GO:0000084	1.71E-08	21.57974301	0.463635735	8	134	S phase of mitotic cell cycle
GO:0000819	2.27E-08	42.55434783	0.179918345	6	52	sister chromatid segregation
GO:0051320	2.86E-08	20.12839506	0.494775448	8	143	S phase
GO:0030261	5.39E-08	63.89333333	0.103799045	5	30	chromosome condensation

GO:0000090	6.15E-08	156.4456522	0.041519618	4	12	
mitotic anaphase						
GO:0051322	1.24E-07	125.1391304	0.048439554	4	14	
anaphase						
GO:0071103	2.48E-07	14.96448303	0.653933984	8	189	DNA
conformation change						
GO:0008608	2.92E-07	96.24080268	0.058819459	4	17	
attachment of spindle microtubules to kinetochore						
GO:0007051	3.11E-07	26.40110565	0.276797453	6	80	
spindle organization						
GO:0031577	3.12E-07	43.13513514	0.145318663	5	42	
spindle checkpoint						
GO:0030071	3.52E-07	41.99707602	0.148778631	5	43	
regulation of mitotic metaphase/anaphase transition						

GO:0031145	3.88E-07	25.36717828	0.287177358	6	83	
anaphase-promoting complex-dependent proteasomal ubiquitin-dependent protein catabolic process						
GO:0007091	4.45E-07	39.89166667	0.155698568	5	45	
mitotic metaphase/anaphase transition						
GO:0051313	4.73E-07	83.39710145	0.065739395	4	19	
attachment of spindle microtubules to chromosome						
GO:0000086	5.88E-07	16.94924461	0.498235416	7	144	G2/M
transition of mitotic cell cycle						
GO:0006958	7.61E-07	35.44691358	0.172998408	5	50	
"complement activation, classical pathway"						
GO:0006261	7.64E-07	22.43573668	0.32177704	6	93	
DNA-dependent DNA replication						
GO:0034501	7.74E-07	306.3404255	0.020759809	3	6	
protein localization to kinetochore						

GO:0006323	8.86E-07	15.89439312	0.52937513	7	153	DNA packaging
GO:0006956	2.44E-06	27.47701149	0.217977995	5	63	complement activation
GO:0002460	2.73E-06	13.31047848	0.626254238	7	181	adaptive immune response based on somatic recombination of immune receptors built from immunoglobulin superfamily domains
GO:0033205	2.83E-06	50.00347826	0.100339077	4	29	cell cycle cytokinesis
GO:0002455	2.85E-06	26.55740741	0.224897931	5	65	humoral immune response mediated by circulating immunoglobulin
GO:0006959	3.75E-06	16.7927116	0.422116117	6	122	humoral immune response
GO:0007052	4.82E-06	43.09445277	0.11417895	4	33	mitotic spindle organization

GO:0002250	4.95E-06	12.11128698	0.685073697	7	198	
adaptive immune response						
GO:0071173	5.45E-06	41.65507246	0.117638918	4	34	
spindle assembly checkpoint						
GO:0034453	6.13E-06	40.3085554	0.121098886	4	35	
microtubule anchoring						
GO:0071174	6.88E-06	39.04619565	0.124558854	4	36	
mitotic cell cycle spindle checkpoint						
GO:0051439	7.05E-06	21.80821918	0.269877517	5	78	
regulation of ubiquitin-protein ligase activity involved in mitotic cell cycle						
GO:0043161	7.06E-06	11.44289662	0.723133347	7	209	
proteasomal ubiquitin-dependent protein catabolic process						
GO:0000216	7.51E-06	21.51201201	0.273337485	5	79	M/G1
transition of mitotic cell cycle						

GO:0010498	8.75E-06	11.05418938	0.747353124	7	216
proteasomal protein catabolic process					
GO:0072376	9.02E-06	20.66955267	0.28371739	5	82
protein activation cascade					
GO:0000079	9.57E-06	20.4031339	0.287177358	5	83
regulation of cyclin-dependent protein kinase activity					
GO:0007076	1.09E-05	91.85744681	0.044979586	3	13
mitotic chromosome condensation					
GO:0010948	1.95E-05	17.47252747	0.332156944	5	96
negative regulation of cell cycle process					
GO:0051438	1.95E-05	17.47252747	0.332156944	5	96
regulation of ubiquitin-protein ligase activity					

Tissue: Heart_Left_Ventricle=>Whole_Blood	Type:	cluster
SourceGene:	ENSG00000260964.1
TargetGeneSet:	CDC20	KIF2C	RP11	DEPDC1	IQGAP3	APOA1BP	CENPF	NCAPH

CKAP2L DNAJC10 PID1 CAMK1 KIF15 CD86 PLK4 MARCH1 CLCN3 CCNB1
PTTG1 HLA-DPA1 KIFC1 EZH2 CDCA2 FBXO43 ENG ESPL1 IKBIP
GPR183 CEBPE DLGAP5 BUB1B TLN2 RCCD1 NME4 HN1L ABI3 BRIP1 SKA1
CTD SUGP1 WDR62 ARHGAP35 GINS1 KCTD17 ATXN10 RP13
GOBPID Pvalue OddsRatio ExpCount Count Size Term
GO:0000280 1.07074260135991e-11 18.7185101811112 1.0172306414781 13
350 nuclear division
GO:0007067 1.07074260135991e-11 18.7185101811112 1.0172306414781 13
350 mitosis
GO:0000087 1.5248419935658e-11 18.1661532346219 1.04629437409176
13 360 M phase of mitotic cell cycle
GO:0048285 2.14907455434004e-11 17.6447406548826 1.07535810670542
13 370 organelle fission
GO:0007059 1.97213566160475e-10 29.7251908396947 0.406892256591239
9 140 chromosome segregation
GO:0051301 4.42700214233742e-08 11.2389730085583 1.31368071413743
11 452 cell division
GO:0030071 1.4385899176527e-07 51.1059743954481 0.124974050238738
5 43 regulation of mitotic metaphase/anaphase transition
GO:0000236 1.53329632772209e-07 30.2320675105485 0.24704172721611
6 85 mitotic prometaphase
GO:0007091 1.81811730010386e-07 48.5439189189189 0.13078679676147
5 45 mitotic metaphase/anaphase transition
GO:0000070 2.81351512788933e-07 44.1185503685504 0.142412289806934
5 49 mitotic sister chromatid segregation
GO:0000819 3.81014127946705e-07 41.2938470385279 0.151131409591032
5 52 sister chromatid segregation
GO:0007017 1.49505621429259e-06 9.9609375 1.14220469171684 9
393 microtubule-based process
GO:0071174 3.39342499763601e-06 47.2927631578947 0.104629437409176
4 36 mitotic cell cycle spindle checkpoint
GO:0033043 3.85331021273676e-06 8.82386363636364 1.2817106082624 9
441 regulation of organelle organization
GO:0007088 5.31813666563279e-06 23.324649951156 0.255760847000208 5
88 regulation of mitosis
GO:0051783 5.31813666563279e-06 23.324649951156 0.255760847000208 5
88 regulation of nuclear division
GO:0031577 6.36721579592055e-06 39.808864265928 0.122067676977372 4
42 spindle checkpoint
Tissue: Whole_Blood=>Heart_Left_Ventricle Type: asymmetric

SourceGene: FTH1P25(ENSG00000232792.1) "ferritin, heavy polypeptide 1
pseudogene 25"

TargetGeneSet: LAPTM5 CD53 CTSS LCP2 DOK3 DEFA6 CARD9 SPI1
MS4A7 FERMT3 PTPN6 RP11 SELPLG KIAA1737 PLCB2 CORO1A SCIMP
ACAP1 HMHA1 VAV1 STXBP2 IFI30 TYROBP CD37 LILRB1 HCK CYTH4
SASH3

GOBPID Pvalue OddsRatio ExpCount Count Size Term

GO:0002252 3.90E-06 13.65393695 0.694484811 7 386
immune effector process

GO:0050870	1.18E-05	20.45180723	0.30766037	5	171
positive regulation of T cell activation

GO:0002694	1.81E-05	13.43809524	0.577537887	6	321
regulation of leukocyte activation

Tissue: Whole_Blood=>Heart_Left_Ventricle	Type:	asymmetric

SourceGene:	NF1(ENSG00000196712.10) neurofibromin 1

TargetGeneSet:	RP4	FLG2	CRCT1	LCE1A	IVL	NPR1	SHE	RAB25	MALL
SPC25	RNU5B-3P	DKK2	RP11	BAG2	INTS10	SLURP1	ZCCHC7	SH2D3C
EMILIN3	TRIM29	ESAM	KRT5	KRT2	KRT1	KRT77	DCD	MUCL1	SCEL
CLEC14A	PRKCH	IFI27	CHST14	FES	TNS4	KRT10	KRT15	TK1	DSC2
SERPINB5	SBSN	RASIP1	KLK5	SMOX	FAM83C

GOBPID	Pvalue	OddsRatio	ExpCount	Count	Size	Term

GO:0008544	3.30E-10	22.94574899	0.622448273	10	257
epidermis development

GO:0030216	8.99E-08	33.68652038	0.227665905	6	94
keratinocyte differentiation

GO:0009913	2.30E-07	28.47214854	0.266417549	6	110
epidermal cell differentiation

Tissue: Whole_Blood=>Heart_Left_Ventricle	Type:	asymmetric

SourceGene:	OSBP2(ENSG00000184792.11)	oxysterol binding protein 2

TargetGeneSet: THAP3 ATP6V0B IQGAP3 RP11 AUP1 ILKAP FANCD2 ARHGAP31-AS1 ZCCHC9 GTF2H4 HCG22 ZNHIT1 FAM115B TM2D2 ARMC1 WDR67 CD72 TLN1 MELK MRPS2 C9orf142 C10orf71 MKI67 IGF2 ACER3 TROAP DDIT3 SLC7A7 BRMS1L SAMD4A BUB1B RAD51 KIF23 STARD5 FANCI CCDC78 NARFL E4F1 COX4NB SPATA2L DERL2 SCO1 TOP2A KIF18B BRIP1 ABHD8 LGALS4 BCAT2 CTB FKBP1A-SDCBP2 SLMO2 APOBEC3B SGSM3 GTSE1

GOBPID	Pvalue	OddsRatio	ExpCount	Count	Size	Term

Tissue: Heart_Left_Ventricle=>Whole_Blood Type: asymmetric

SourceGene: PIK3R2(ENSG00000105647.8) "phosphoinositide-3-kinase, regulatory subunit 2"

TargetGeneSet: CDCA8 KIF2C PKLR LZTFL1 PHF7 BMP2K TRIP13 CTD CDC25C HLA-DRB5 HLA-DRB1 HLA-DQB1 KIFC1 NDUFAF4 CD164 PKIB PCMT1 WIPI2 RP11 MRPL50 ZNF32-AS2 SIGIRR CDCA3 ESPL1 CHST11 RFC3 SLAIN1 CLYBL PCID2 GSTZ1 HMGN2P5 KIAA0101 CLN6 BLM CCDC101 ITGAM PITPNM3 BRIP1 TK1 TYMS CIRBP SPC24 TNPO2 RASAL3 JAK3 PRR24 FUZ ZNF446 CDC45 RP4 RIBC1 CETN2

GOBPID	Pvalue	OddsRatio	ExpCount	Count	Size	Term
GO:0051301	3.80E-06	8.867310948	1.282402602	9	452	cell division
GO:0000280	5.20E-06	9.972000709	0.993010864	8	350	nuclear division
GO:0007067	5.20E-06	9.972000709	0.993010864	8	350	mitosis

GO:0000087	6.39E-06	9.681818182	1.021382603	8	360	M
phase of mitotic cell cycle

GO:0048285	7.81E-06	9.407667839	1.049754342	8	370
organelle fission

Tissue: Heart_Left_Ventricle=>Whole_Blood	Type:	asymmetric

SourceGene:	RCAN3(ENSG00000117602.6)	RCAN family member 3

TargetGeneSet:	FCRL5	RP11	SH2D6	SULT1C2	BANK1	GALNTL6	RSPH9	BLK	PAX5
ACTR1A	GYLTL1B	MS4A1	CD27	DTX1	TCL1B	KIAA0125		IGHV1-2	IGHV1-24
PLA2G4B	CD19	SPDYE4	HDAC5	CD79B	FCER2	RHPN2	CD79A	SPIB	IGLC5
TNFRSF13C		TCEAL6

GOBPID	Pvalue	OddsRatio	ExpCount	Count	Size	Term

GO:0042113	8.28E-08	36.43367347	0.232925057	6	153	B
cell activation

GO:0046649	2.39E-06	15.33974961	0.659193135	7	433
lymphocyte activation

Tissue: Whole_Blood=>Heart_Left_Ventricle	Type:	asymmetric
SourceGene:	RP11-1033H12.3(ENSG00000258440.1)
TargetGeneSet:	RPL22	RPL11	AK2	RPL5	RP11	PSMA5	ACP1	SLC4A1AP
UBC	RNF181	RPL15	C3orf26	RPL24	RPL35A	BTF3	PCDHGA1	CTB	RPS14
MRPS12	ATP6V1F	UBE3C	PPP2CB	RPS20	RPL7	ENY2	GLRX3	RPS13	CRYAB
STRAP	PSMC6	PSMC1	AHNAK2	RPL26	KCNJ12	PSMB3	RPL17	DNAJC7	SLMO1
C18orf21		RPL18	SNRPB	EEF1B2P3		RPS23P8
GOBPID	Pvalue	OddsRatio	ExpCount	Count	Size	Term
GO:0006415	1.13024861698362e-22	124.147186147186	0.220399972320255
14	91	translational termination
GO:0006414	9.52400953644563e-22	104.945054945055	0.254307660369525
14	105	translational elongation
GO:0006614	9.52400953644563e-22	104.945054945055	0.254307660369525
14	105	SRP-dependent cotranslational protein targeting to membrane
GO:0006613	1.0958337582712e-21	103.797101449275	0.25672963808733
14	106	cotranslational protein targeting to membrane
GO:0045047	1.0958337582712e-21	103.797101449275	0.25672963808733
14	106	protein targeting to ER

GO:0072599	1.0958337582712e-21	103.797101449275	0.25672963808733
14	106	establishment of protein localization to endoplasmic reticulum
GO:0070972	5.32068668405306e-21	91.7435897435897	0.28579337070099
14	118	protein localization to endoplasmic reticulum
GO:0000184	6.02187460500713e-21	90.8634920634921	0.288215348418795
14	119	nuclear-transcribed mRNA catabolic process, nonsense-mediated decay
GO:0019080	1.92429404839666e-19	69.4841849148418	0.365718635388554
14	151	viral genome expression
GO:0019083	1.92429404839666e-19	69.4841849148418	0.365718635388554
14	151	viral transcription
GO:0006413	2.11661143776567e-19	68.975845410628 0.368140613106359	14
152	translational initiation
GO:0006612	2.32660648605977e-19	68.4748201438849	0.370562590824164
14	153	protein targeting to membrane
GO:0043624	2.32660648605977e-19	68.4748201438849	0.370562590824164
14	153	cellular protein complex disassembly
GO:0043241	3.6978387346578e-19	66.0740740740741	0.382672479413189
14	158	protein complex disassembly
GO:0000956	9.72417227373162e-19	61.3376344086021	0.409314234309044
14	169	nuclear-transcribed mRNA catabolic process
GO:0034623	1.47654099777653e-18	59.4	0.421424122898069	14	174
cellular macromolecular complex disassembly
GO:0016032	1.57149853098814e-18	34.1929085722049	1.09231195073005
18	451	viral reproduction
GO:0006402	2.04378168080614e-18	57.9349593495935	0.431112033769289
14	178	mRNA catabolic process
GO:0032984	2.21415407274473e-18	57.579797979798 0.433534011487094	14
179	macromolecular complex disassembly
GO:0072594	1.42425792613121e-17	49.9157894736842	0.494083454432219
14	204	establishment of protein localization to organelle
GO:0006401	1.52655504908356e-17	49.6509598603839	0.496505432150024
14	205	RNA catabolic process
GO:0019058	8.25737690205258e-17	43.6221198156682	0.559476852812954
14	231	viral infectious cycle
GO:0022415	4.34881362463966e-16	38.4010840108401	0.629714206629299
14	260	viral reproductive process
GO:0071845	1.49317566910718e-15	34.9283950617284	0.687841671856619
14	284	cellular component disassembly at cellular level
GO:0022411	1.81445639266429e-15	34.4087591240876	0.697529582727839
14	288	cellular component disassembly
GO:0033365	1.65509849938895e-12	20.2716049382716	1.14559546052176
14	473	protein localization to organelle
GO:0006605	1.70309181669195e-12	20.2260869565217	1.14801743823957
14	474	protein targeting
GO:0042273	4.83997390650977e-11	343.071428571429	0.02906373261366
5	12	ribosomal large subunit biogenesis
GO:0022613	4.99760970587808e-08	19.3876088069636	0.544944986506124
8	225	ribonucleoprotein complex biogenesis
GO:0071843	7.97107515811943e-08	18.1946448613115	0.578852674555394
8	239	cellular component biogenesis at cellular level
GO:0006364	1.64612882806908e-07	30.2280084447572	0.25188568265172
6	104	rRNA processing
GO:0016072	2.29963934403525e-07	28.4721485411141	0.26641754895855
6	110	rRNA metabolic process
GO:0006977	4.97362279991076e-07	39.2213114754098	0.15985052937513
5	66	DNA damage response, signal transduction by p53 class mediator
resulting in cell cycle arrest
GO:0072413	4.97362279991076e-07	39.2213114754098	0.15985052937513
5	66	signal transduction involved in mitotic cell cycle checkpoint
GO:0072431	4.97362279991076e-07	39.2213114754098	0.15985052937513
5	66	signal transduction involved in mitotic cell cycle G1/S transition
DNA damage checkpoint
GO:0072474	4.97362279991076e-07	39.2213114754098	0.15985052937513

5	66	signal transduction involved in mitotic cell cycle G1/S checkpoint
GO:0051436	5.36541489798949e-07	38.5860215053763	0.162272507092935
5	67	negative regulation of ubiquitin-protein ligase activity involved in mitotic cell cycle
GO:0072401	5.36541489798949e-07	38.5860215053763	0.162272507092935
5	67	signal transduction involved in DNA integrity checkpoint
GO:0072404	5.36541489798949e-07	38.5860215053763	0.162272507092935
5	67	signal transduction involved in G1/S transition checkpoint
GO:0072422	5.36541489798949e-07	38.5860215053763	0.162272507092935
5	67	signal transduction involved in DNA damage checkpoint
GO:0072395	5.78121366136425e-07	37.9708994708995	0.16469448481074
5	68	signal transduction involved in cell cycle checkpoint
GO:0051352	7.70609308533534e-07	35.6940298507463	0.17438239568196
5	72	negative regulation of ligase activity
GO:0051437	7.70609308533534e-07	35.6940298507463	0.17438239568196
5	72	positive regulation of ubiquitin-protein ligase activity involved in mitotic cell cycle
GO:0051444	7.70609308533534e-07	35.6940298507463	0.17438239568196
5	72	negative regulation of ubiquitin-protein ligase activity
GO:0031571	8.25839238324015e-07	35.1666666666667	0.176804373399765
5	73	mitotic cell cycle G1/S transition DNA damage checkpoint
GO:0002479	9.45662089990647e-07	34.1571428571429	0.181648328835375
5	75	antigen processing and presentation of exogenous peptide antigen via MHC class I, TAP-dependent
GO:0051439	1.15070253195326e-06	32.7465753424658	0.18891426198879
5	78	regulation of ubiquitin-protein ligase activity involved in mitotic cell cycle
GO:0000216	1.22632538943352e-06	32.3018018018018	0.191336239706595
5	79	M/G1 transition of mitotic cell cycle
GO:0042590	1.22632538943352e-06	32.3018018018018	0.191336239706595
5	79	antigen processing and presentation of exogenous peptide antigen via MHC class I
GO:0071158	1.3058151925104e-06	31.8688888888889	0.1937582174244 5
80	positive regulation of cell cycle arrest
GO:0042254	1.37762246487303e-06	20.6505907885218	0.360874679952944
6	149	ribosome biogenesis
GO:0031575	1.47696702907015e-06	31.0367965367965	0.19860217286001
5	82	mitotic cell cycle G1/S transition checkpoint
GO:0051443	1.47696702907015e-06	31.0367965367965	0.19860217286001
5	82	positive regulation of ubiquitin-protein ligase activity
GO:0002478	1.56892269816487e-06	30.6367521367521	0.201024150577815
5	83	antigen processing and presentation of exogenous peptide antigen
GO:0031145	1.56892269816487e-06	30.6367521367521	0.201024150577815
5	83	anaphase-promoting complex-dependent proteasomal ubiquitin-dependent protein catabolic process
GO:0019884	1.76635167310192e-06	29.8666666666667	0.205868106013425
5	85	antigen processing and presentation of exogenous antigen
GO:0051351	1.87213794113529e-06	29.4958847736626	0.20829008373123
5	86	positive regulation of ligase activity
GO:0071779	1.87213794113529e-06	29.4958847736626	0.20829008373123
5	86	G1/S transition checkpoint
GO:0000209	2.16420693584304e-06	19.0358175750834	0.389938412566604
6	161	protein polyubiquitination
GO:0031397	2.61623622572555e-06	27.4501915708812	0.22282195003806
5	92	negative regulation of protein ubiquitination
GO:0051438	3.22934745288586e-06	26.2362637362637	0.23250986090928
5	96	regulation of ubiquitin-protein ligase activity
GO:0002474	3.7591586958813e-06	25.3936170212766	0.239775794062695
5	99	antigen processing and presentation of peptide antigen via MHC class I
GO:2000045	3.7591586958813e-06	25.3936170212766	0.239775794062695
5	99	regulation of G1/S transition of mitotic cell cycle
GO:0051340	3.95015569920992e-06	25.1245614035088	0.2421977717805 5

100	regulation of ligase activity
GO:0034660	4.51600848980766e-06	12.7140287769784	0.690263649574424
7	285	ncRNA metabolic process
GO:0048002	5.76797129387682e-06	23.1601941747573	0.26157359352294
5	108	antigen processing and presentation of peptide antigen
GO:0034470	7.57338935750658e-06	15.1674369001066	0.484395543560999
6	200	ncRNA processing
GO:0030330	8.53563514448506e-06	21.2857142857143	0.283371392983185
5	117	DNA damage response, signal transduction by p53 class mediator
GO:0043161	9.74212639797436e-06	14.4858162051979	0.506193343021244
6	209	proteasomal ubiquitin-dependent protein catabolic process
GO:0006521	1.03962421825924e-05	34.9677419354839	0.138052729914885
4	57	regulation of cellular amino acid metabolic process
GO:0072331	1.08936063853011e-05	20.1949152542373	0.297903259290015
5	123	signal transduction by p53 class mediator
GO:0010498	1.17561679581293e-05	13.9960591133005	0.523147187045879
6	216	proteasomal protein catabolic process
GO:0031398	1.27289428155871e-05	19.5273224043716	0.307591170161234
5	127	positive regulation of protein ubiquitination
GO:0000077	1.42573752894829e-05	19.0546666666667	0.31485710331465
5	130	DNA damage checkpoint
GO:0000084	1.65141134739317e-05	18.4586563307494	0.32454501418587
5	134	S phase of mitotic cell cycle
GO:0031570	1.77421908200658e-05	18.1743002544529	0.329388969621479
5	136	DNA integrity checkpoint
GO:0007093	1.97166089152038e-05	17.7636815920398	0.336654902774894
5	139	mitotic cell cycle checkpoint
Tissue: Whole_Blood=>Heart_Left_Ventricle	Type:	asymmetric
SourceGene:	RP11-166P13.3(ENSG00000263004.1)
TargetGeneSet:	CTNNBIP1	RSG1	WBP1	FUCA1	PPIE	UROD	RP11
ZFYVE9	ZRANB2	MTF2	PSMA5	CERS2	PSMB4	C1orf61	SMG7	GS1	RBBP5
KIF3C	GCFC2	RNF181	POU3F3	PSMD14	PSMD6	PSMC1P1	C3orf17	GK5	NMD3
YEATS2	GRIA2	GPM6A	BRD9	WDR70	PAPD4	YTHDC2	CTB	PDLIM7	UFL1
PSMB1	AMPH	CDK13	UBE3C	PYCRL	KIAA2026		NFIB	XPA	FPGS	COQ4
NOXA1	TAF3	DLG5	BTAF1	PSMD13	OR52E8	ZNF143	YIF1A	C12orf5	AKAP3
STRAP	PSG1	ATF7	MVK	IFT81	VPS29	PABPC3	EXOSC8	UCHL3	FBXL3
PSMC6	PSMC1	VRK1	RPL4	COMMD4	SNX33	WDR61	FBXL16	CREBBP	MAZ
SULT1A3	SALL1	TMEM208	CTCF	ZFHX3	ZBTB4	C17orf39		RPL17P43
KCNJ12	WIPF2	G6PC3	GFAP	ANKRD40	TUBD1	MAP3K3	CHMP6	MIR133A1
C19orf6	ZBTB7A	NCAN	ZNF461	CTU1	ZNF580	SNRPB	ITPA	PYGB	SLPI
ZNF512B	CBS	TCF20	RBM3	MAGED1	PABPC1P3		BCORL1
GOBPID	Pvalue	OddsRatio	ExpCount	Count	Size	Term
GO:0006521	2.53435722653352e-09	27.8134110787172	0.362881461490554
8	57	regulation of cellular amino acid metabolic process
GO:0031397	6.49469187792333e-09	18.6506024096386	0.585703411528614
9	92	negative regulation of protein ubiquitination
GO:0006977	8.40770360980823e-09	23.4827586206897	0.420178534357484
8	66	DNA damage response, signal transduction by p53 class mediator resulting in cell cycle arrest
GO:0072413	8.40770360980823e-09	23.4827586206897	0.420178534357484
8	66	signal transduction involved in mitotic cell cycle checkpoint
GO:0072431	8.40770360980823e-09	23.4827586206897	0.420178534357484
8	66	signal transduction involved in mitotic cell cycle G1/S transition DNA damage checkpoint
GO:0072474	8.40770360980823e-09	23.4827586206897	0.420178534357484
8	66	signal transduction involved in mitotic cell cycle G1/S checkpoint
GO:0051436	9.49838521246666e-09	23.0831315577078	0.426544875787143
8	67	negative regulation of ubiquitin-protein ligase activity involved in mitotic cell cycle
GO:0072401	9.49838521246666e-09	23.0831315577078	0.426544875787143
8	67	signal transduction involved in DNA integrity checkpoint
GO:0072404	9.49838521246666e-09	23.0831315577078	0.426544875787143
8	67	signal transduction involved in G1/S transition checkpoint

GO:0072422	9.49838521246666e-09	23.0831315577078	0.426544875787143
8	67	signal transduction involved in DNA damage checkpoint
GO:0072395	1.07092005205718e-08	22.6968253968254	0.432911217216802
8	68	signal transduction involved in cell cycle checkpoint
GO:0033238	1.69844533038307e-08	21.2723214285714	0.458376582935437
8	72	regulation of cellular amine metabolic process
GO:0051352	1.69844533038307e-08	21.2723214285714	0.458376582935437
8	72	negative regulation of ligase activity
GO:0051437	1.69844533038307e-08	21.2723214285714	0.458376582935437
8	72	positive regulation of ubiquitin-protein ligase activity involved in mitotic cell cycle
GO:0051444	1.69844533038307e-08	21.2723214285714	0.458376582935437
8	72	negative regulation of ubiquitin-protein ligase activity
GO:0031571	1.8976266206716e-08	20.9435897435897	0.464742924365096
8	73	mitotic cell cycle G1/S transition DNA damage checkpoint
GO:0002479	2.35713488196993e-08	20.3155650319829	0.477475607224414
8	75	antigen processing and presentation of exogenous peptide antigen via MHC class I, TAP-dependent
GO:0051439	3.22534796497062e-08	19.4408163265306	0.49657463151339
8	78	regulation of ubiquitin-protein ligase activity involved in mitotic cell cycle
GO:0000216	3.57022105002875e-08	19.1656606304494	0.502940972943049
8	79	M/G1 transition of mitotic cell cycle
GO:0042590	3.57022105002875e-08	19.1656606304494	0.502940972943049
8	79	antigen processing and presentation of exogenous peptide antigen via MHC class I
GO:0071158	3.94641181496668e-08	18.8981481481481	0.509307314372708
8	80	positive regulation of cell cycle arrest
GO:0031575	4.80226761571519e-08	18.3848133848134	0.522039997232025
8	82	mitotic cell cycle G1/S transition checkpoint
GO:0051443	4.80226761571519e-08	18.3848133848134	0.522039997232025
8	82	positive regulation of ubiquitin-protein ligase activity
GO:0002478	5.2870454764339e-08	18.1384126984127	0.528406338661684
8	83	antigen processing and presentation of exogenous peptide antigen
GO:0031145	5.2870454764339e-08	18.1384126984127	0.528406338661684
8	83	anaphase-promoting complex-dependent proteasomal ubiquitin-dependent protein catabolic process
GO:0019884	6.38420335084693e-08	17.6648113790971	0.541139021521002
8	85	antigen processing and presentation of exogenous antigen
GO:0051351	7.00261860721298e-08	17.4371184371184	0.547505362950661
8	86	positive regulation of ligase activity
GO:0071779	7.00261860721298e-08	17.4371184371184	0.547505362950661
8	86	G1/S transition checkpoint
GO:0051438	1.66164145842762e-07	15.4448051948052	0.611168777247249
8	96	regulation of ubiquitin-protein ligase activity
GO:0002474	2.11225746466807e-07	14.9324960753532	0.630267801536226
8	99	antigen processing and presentation of peptide antigen via MHC class I
GO:2000045	2.11225746466807e-07	14.9324960753532	0.630267801536226
8	99	regulation of G1/S transition of mitotic cell cycle
GO:0051340	2.28406896786785e-07	14.7691511387164	0.636634142965885
8	100	regulation of ligase activity
GO:0042770	2.55183165295285e-07	11.7770624482663	0.891287800152239
9	140	signal transduction in response to DNA damage
GO:0051320	3.05775335256105e-07	11.5109692501349	0.910386824441215
9	143	S phase
GO:0048002	4.14541140637089e-07	13.58	0.687564874403156	8	108
antigen processing and presentation of peptide antigen
GO:0030330	7.66453401281928e-07	12.4508519003932	0.744861947270085
8	117	DNA damage response, signal transduction by p53 class mediator
GO:0000209	8.3355571864438e-07	10.1349873176918	1.02498097017507
9	161	protein polyubiquitination
GO:0072331	1.12210981268672e-06	11.7962732919255	0.783059995848038

8	123	signal transduction by p53 class mediator
GO:0031396	1.19040865829112e-06	9.68401909524892	1.06954536018269
9	168	regulation of protein ubiquitination
GO:0031398	1.43044643452998e-06	11.3965586234494	0.808525361566674
8	127	positive regulation of protein ubiquitination
GO:0000077	1.7066354953522e-06	11.1139734582358	0.82762438585565
8	130	DNA damage checkpoint
GO:0000084	2.1445634729058e-06	10.7581254724112	0.853089751574286
8	134	S phase of mitotic cell cycle
GO:0031570	2.39717622022303e-06	10.5885416666667	0.865822434433603
8	136	DNA integrity checkpoint
GO:0007093	2.82334120209182e-06	10.343874954562 0.88492145872258	8
139	mitotic cell cycle checkpoint
GO:2000602	4.28674983908019e-06	9.74306269270298	0.935852190159851
8	147	regulation of interphase of mitotic cell cycle
GO:0090068	5.23212999626783e-06	9.46786546786547	0.961317555878486
8	151	positive regulation of cell cycle process
GO:0019882	5.49428429262543e-06	9.40145502645503	0.967683897308145
8	152	antigen processing and presentation
GO:0010565	6.05209203002869e-06	9.27136333985649	0.980416580167462
8	154	regulation of cellular ketone metabolic process
GO:0043161	7.17183441957019e-06	7.67656626506024	1.3305653587987 9
209	proteasomal ubiquitin-dependent protein catabolic process
GO:0010498	9.35637758101783e-06	7.41330539549502	1.37512974880631
9	216	proteasomal protein catabolic process
GO:0006511	1.92912918139558e-05	5.38824187738258	2.31734828039582
11	364	ubiquitin-dependent protein catabolic process
Tissue: Whole_Blood=>Heart_Left_Ventricle	Type:	cluster
SourceGene:	RP11-166P13.3(ENSG00000263004.1)
TargetGeneSet:	CTNNBIP1	RSG1	WBP1	FUCA1	PPIE	UROD	RP11
ZFYVE9	ZRANB2	MTF2	PSMA5	CERS2	PSMB4	C1orf61	SMG7	GS1	RBBP5
KIF3C	GCFC2	RNF181	POU3F3	PSMD14	PSMD6	PSMC1P1	C3orf17	GK5	NMD3
YEATS2	GRIA2	GPM6A	BRD9	WDR70	PAPD4	YTHDC2	CTB	PDLIM7	UFL1
PSMB1	AMPH	CDK13	UBE3C	PYCRL	KIAA2026	NFIB	XPA	FPGS	COQ4
NOXA1	TAF3	DLG5	BTAF1	PSMD13	OR52E8	ZNF143	YIF1A	C12orf5	AKAP3
STRAP	PSG1	ATF7	MVK	IFT81	VPS29	PABPC3	EXOSC8	UCHL3	FBXL3
PSMC6	PSMC1	VRK1	RPL4	COMMD4	SNX33	WDR61	FBXL16	CREBBP	MAZ
SULT1A3	SALL1	TMEM208	CTCF	ZFHX3	ZBTB4	C17orf39	RPL17P43
KCNJ12	WIPF2	G6PC3	GFAP	ANKRD40	TUBD1	MAP3K3	CHMP6	MIR133A1
C19orf6	ZBTB7A	NCAN	ZNF461	CTU1	ZNF580	SNRPB	ITPA	PYGB	SLPI
ZNF512B	CBS	TCF20	RBM3	MAGED1	PABPC1P3	BCORL1
GOBPID	Pvalue	OddsRatio	ExpCount	Count	Size	Term
GO:0006521	2.53435722653352e-09	27.8134110787172	0.362881461490554
8	57	regulation of cellular amino acid metabolic process
GO:0031397	6.49469187792333e-09	18.6506024096386	0.585703411528614
9	92	negative regulation of protein ubiquitination
GO:0006977	8.40770360980823e-09	23.4827586206897	0.420178534357484
8	66	DNA damage response, signal transduction by p53 class mediator resulting in cell cycle arrest
GO:0072413	8.40770360980823e-09	23.4827586206897	0.420178534357484
8	66	signal transduction involved in mitotic cell cycle checkpoint
GO:0072431	8.40770360980823e-09	23.4827586206897	0.420178534357484
8	66	signal transduction involved in mitotic cell cycle G1/S transition DNA damage checkpoint
GO:0072474	8.40770360980823e-09	23.4827586206897	0.420178534357484
8	66	signal transduction involved in mitotic cell cycle G1/S checkpoint
GO:0051436	9.49838521246666e-09	23.0831315577078	0.426544875787143
8	67	negative regulation of ubiquitin-protein ligase activity involved in mitotic cell cycle
GO:0072401	9.49838521246666e-09	23.0831315577078	0.426544875787143
8	67	signal transduction involved in DNA integrity checkpoint
GO:0072404	9.49838521246666e-09	23.0831315577078	0.426544875787143
8	67	signal transduction involved in G1/S transition checkpoint

GO:0072422 9.49838521246666e-09 23.0831315577078 0.426544875787143
8 67 signal transduction involved in DNA damage checkpoint
GO:0072395 1.07092005205718e-08 22.6968253968254 0.432911217216802
8 68 signal transduction involved in cell cycle checkpoint
GO:0033238 1.69844533038307e-08 21.2723214285714 0.458376582935437
8 72 regulation of cellular amine metabolic process
GO:0051352 1.69844533038307e-08 21.2723214285714 0.458376582935437
8 72 negative regulation of ligase activity
GO:0051437 1.69844533038307e-08 21.2723214285714 0.458376582935437
8 72 positive regulation of ubiquitin-protein ligase activity involved in mitotic cell cycle
GO:0051444 1.69844533038307e-08 21.2723214285714 0.458376582935437
8 72 negative regulation of ubiquitin-protein ligase activity
GO:0031571 1.8976266206716e-08 20.9435897435897 0.464742924365096
8 73 mitotic cell cycle G1/S transition DNA damage checkpoint
GO:0002479 2.35713488196993e-08 20.3155650319829 0.477475607224414
8 75 antigen processing and presentation of exogenous peptide antigen via MHC class I, TAP-dependent
GO:0051439 3.22534796497062e-08 19.4408163265306 0.49657463151339
8 78 regulation of ubiquitin-protein ligase activity involved in mitotic cell cycle
GO:0000216 3.57022105002875e-08 19.1656606304494 0.502940972943049
8 79 M/G1 transition of mitotic cell cycle
GO:0042590 3.57022105002875e-08 19.1656606304494 0.502940972943049
8 79 antigen processing and presentation of exogenous peptide antigen via MHC class I
GO:0071158 3.94641181496668e-08 18.8981481481481 0.509307314372708
8 80 positive regulation of cell cycle arrest
GO:0031575 4.80226761571519e-08 18.3848133848134 0.522039997232025
8 82 mitotic cell cycle G1/S transition checkpoint
GO:0051443 4.80226761571519e-08 18.3848133848134 0.522039997232025
8 82 positive regulation of ubiquitin-protein ligase activity
GO:0002478 5.2870454764339e-08 18.1384126984127 0.528406338661684
8 83 antigen processing and presentation of exogenous peptide antigen
GO:0031145 5.2870454764339e-08 18.1384126984127 0.528406338661684
8 83 anaphase-promoting complex-dependent proteasomal ubiquitin-dependent protein catabolic process
GO:0019884 6.38420335084693e-08 17.6648113790971 0.541139021521002
8 85 antigen processing and presentation of exogenous antigen
GO:0051351 7.00261860721298e-08 17.4371184371184 0.547505362950661
8 86 positive regulation of ligase activity
GO:0071779 7.00261860721298e-08 17.4371184371184 0.547505362950661
8 86 G1/S transition checkpoint
GO:0051438 1.66164145842762e-07 15.4448051948052 0.611168777247249
8 96 regulation of ubiquitin-protein ligase activity
GO:0002474 2.11225746466807e-07 14.9324960753532 0.630267801536226
8 99 antigen processing and presentation of peptide antigen via MHC class I
GO:2000045 2.11225746466807e-07 14.9324960753532 0.630267801536226
8 99 regulation of G1/S transition of mitotic cell cycle
GO:0051340 2.28406896786785e-07 14.7691511387164 0.636634142965885
8 100 regulation of ligase activity
GO:0042770 2.55183165295285e-07 11.7770624482663 0.891287800152239
9 140 signal transduction in response to DNA damage
GO:0051320 3.05775335256105e-07 11.5109692501349 0.910386824441215
9 143 S phase
GO:0048002 4.14541140637089e-07 13.58 0.687564874403156 8 108
antigen processing and presentation of peptide antigen
GO:0030330 7.66453401281928e-07 12.4508519003932 0.744861947270085
8 117 DNA damage response, signal transduction by p53 class mediator
GO:0000209 8.3355571864438e-07 10.1349873176918 1.02498097017507
9 161 protein polyubiquitination
GO:0072331 1.12210981268672e-06 11.7962732919255 0.783059995848038

8	123	signal transduction by p53 class mediator
GO:0031396	1.19040865829112e-06	9.68401909524892	1.06954536018269
9	168	regulation of protein ubiquitination
GO:0031398	1.43044643452998e-06	11.3965586234494	0.808525361566674
8	127	positive regulation of protein ubiquitination
GO:0000077	1.7066354953522e-06	11.1139734582358	0.82762438585565
8	130	DNA damage checkpoint
GO:0000084	2.1445634729058e-06	10.7581254724112	0.853089751574286
8	134	S phase of mitotic cell cycle
GO:0031570	2.39717622022303e-06	10.5885416666667	0.865822434433603
8	136	DNA integrity checkpoint
GO:0007093	2.82334120209182e-06	10.343874954562 0.88492145872258	8
139	mitotic cell cycle checkpoint
GO:2000602	4.28674983908019e-06	9.74306269270298	0.935852190159851
8	147	regulation of interphase of mitotic cell cycle
GO:0090068	5.23212999626783e-06	9.46786546786547	0.961317555878486
8	151	positive regulation of cell cycle process
GO:0019882	5.49428429262543e-06	9.40145502645503	0.967683897308145
8	152	antigen processing and presentation
GO:0010565	6.05209203002869e-06	9.27136333985649	0.980416580167462
8	154	regulation of cellular ketone metabolic process
GO:0043161	7.17183441957019e-06	7.67656626506024	1.3305653587987 9
209	proteasomal ubiquitin-dependent protein catabolic process
GO:0010498	9.35637758101783e-06	7.41330539549502	1.37512974880631
9	216	proteasomal protein catabolic process
GO:0006511	1.92912918139558e-05	5.38824187738258	2.31734828039582
11	364	ubiquitin-dependent protein catabolic process
Tissue: Heart_Left_Ventricle=>Whole_Blood	Type:	asymmetric
SourceGene:	RP11-29H23.6(ENSG00000223503.1)
TargetGeneSet:	RP11	TNFSF13B	AKT3	STK25	STEAP2	XRCC2	SMC3	FEN1
KNTC1	RFC3	CACNA1H PKMYT1	CDH11	CDT1	CDK5R1	CDC6	EME1	TYMS
RNASEH2A	CDC45
GOBPID	Pvalue	OddsRatio	ExpCount	Count	Size	Term
GO:0006260	1.30978487834235e-09	36.3358974358974	0.370908587640994
8	268	DNA replication
GO:0000083	1.92033805358757e-08	200.180555555556	0.0304477198809771
4	22	regulation of transcription involved in G1/S phase of mitotic cell cycle
GO:0006261	1.42254481950831e-07	54.3295454545455	0.128710815860494
5	93	DNA-dependent DNA replication
GO:0000076	2.70364629542563e-07	363.63025210084 0.0138398726731714	3
10	DNA replication checkpoint
GO:0051329	4.66216865145218e-07	20.3500827129859	0.524531174313196
7	379	interphase of mitotic cell cycle
GO:0010972	4.94794066892285e-07	282.78431372549 0.0166078472078057	3
12	negative regulation of G2/M transition of mitotic cell cycle
GO:0051325	5.36791290777933e-07	19.9103238866397	0.535603072451733
7	387	interphase
GO:0010564	8.45215652260883e-07	18.5537705537706	0.572970728669296
7	414	regulation of cell cycle process
GO:0000084	8.81850629341559e-07	36.9560723514212	0.185454293820497
5	134	S phase of mitotic cell cycle
GO:0051320	1.21685080455504e-06	34.524154589372 0.197910179226351	5
143	S phase
GO:0000082	4.43631168046153e-06	26.2430939226519	0.257421631720988
5	186	G1/S transition of mitotic cell cycle
GO:0010948	8.1665664208113e-06	38.9646739130435	0.132862777662446
4	96	negative regulation of cell cycle process
GO:0010389	8.98724677238467e-06	94.1437908496732	0.0415196180195142
3	30	regulation of G2/M transition of mitotic cell cycle
GO:0006281	9.42699119265486e-06	15.9765062523683	0.530067123382465
6	383	DNA repair
GO:0046683	1.0900525832417e-05	801.611111111111	0.00553594906926856

2 4 response to organophosphorus
GO:0006271 1.31995368287061e-05 81.9734345351044 0.0470555670887828
3 34 DNA strand elongation involved in DNA replication
GO:0000075 1.5951907560306e-05 19.9634317862166 0.334924918690748
5 242 cell cycle checkpoint
GO:0022616 1.7093775598175e-05 74.7249134948097 0.0512075288907342
3 37 DNA strand elongation
Tissue: Heart_Left_Ventricle=>Whole_Blood Type: asymmetric
SourceGene: RP3-434O14.8(ENSG00000232222.1)
TargetGeneSet: MPL RP11 PEAR1 SELP C1orf198 C2orf88 VIL1
KLF15 PPBP ARHGAP21 CTD SH3TC2 F13A1 LY6G6F C6orf25 TREML1 DAO
EDF1 TSPAN15 CTTN PDE2A NRGN ESAM ARG2 C14orf133 LGMN
PCSK6 ITGB3 C17orf109 KXD1 SYTL4
GOBPID Pvalue OddsRatio ExpCount Count Size Term
GO:0007596 2.57611964243697e-07 17.5070643642072 0.723686942080133
8 498 blood coagulation
GO:0030168 4.80947826801868e-07 26.9554502369668 0.315341498858211
6 217 platelet activation
GO:0002576 5.62061770647382e-06 42.7431124348474 0.120614490346689
4 83 platelet degranulation
Tissue: Whole_Blood=>Heart_Left_Ventricle Type: asymmetric

SourceGene: ZUFSP(ENSG00000153975.5) zinc finger with UFM1-specific peptidase domain

TargetGeneSet: MRPL20 WBP1 ZBTB40 MRPL37 HBXIP ATP5F1 HAX1 MRPL24
LINC00467 MSH6 EIF3FP3 COMMD1 SNRNP200 CHCHD5 ATP5G3 UBE2E3
WDR48 FYCO1 MYL3 PBRM1 SPCS1 THOC7 GTPBP8 FBXO40 SRPRB TRA2B
OCIAD1 NDUFC1 SMARCA5 KIAA0922 KIAA0947 SUB1 NDUFS4 PCBD2
SLC35A4 TBC1D9B TMEM14B CUL9 GABRR2 GTF3C6 HDDC2 BAZ1B FIS1 TNPO3 RP4
DCTN6 MRPL15 RPS20 ARFGEF1 KLHL38 NDUFB9 CTD CYC1 C9orf46 ERP44
NIPSNAP3B RABGAP1 PSMB7 AK1 RP11 METTL11A FCN2 KIAA0913
SMC3 NUDT22 RELA YIF1A PPP6R3 NUMA1 LAMTOR1 SPCS2 ERC1 MRPL51
SMARCC2 ANKRD52 GLIPR1L1 NDUFA12 SLC25A3 ALDH1L2 TCP11L2 ISCU KDM2B MED4
MED4-AS1 SALL2 CEBPE C14orf182 CDC42BPB HERC2 TPM1
DENND4A UBL7 COX5A SIN3A MRPS11 ITFG3 MRPL28 ZNF598 CREBBP SETD1A RBL2
COX4I1 PSMB6 DVL2 CTC1 POLDIP2 SYNRG SOCS7 C17orf53 NPEPPS
TBKBP1 SNF8 SLC35B1 ICT1 RNF213 C18orf32 HAUS8 POP4 COX6B1
PPP6R1 CHMP2A CBFA2T2 CEP250 ATP5O MRPL40 LDOC1L PRDX4 SAT1 HSD17B10

COX7B FAM127A MT

GOBPID	Pvalue	OddsRatio	ExpCount	Count	Size	Term
GO:0022904	7.46E-11	20.06304348	0.684243305	11	96	respiratory electron transport chain
GO:0022900	2.78E-09	13.827766	0.955089613	11	134	electron transport chain
GO:0045333	5.15E-09	12.97602058	1.012109889	11	142	cellular respiration
GO:0015980	2.69E-07	6.904837491	2.188153069	13	307	energy derivation by oxidation of organic compounds

GO:0006091 2.01E-06 5.29436031 3.050584735 14 428
generation of precursor metabolites and energy

Tissue: Whole_Blood=>Heart_Left_Ventricle Type: cluster
SourceGene: ZUFSP(ENSG00000153975.5)
TargetGeneSet: MRPL20 WBP1 ZBTB40 MRPL37 HBXIP ATP5F1 HAX1 MRPL24
LINC00467 MSH6 EIF3FP3 COMMD1 SNRNP200 CHCHD5 ATP5G3 UBE2E3
WDR48 FYCO1 MYL3 PBRM1 SPCS1 THOC7 GTPBP8 FBXO40 SRPRB TRA2B
OCIAD1 NDUFC1 SMARCA5 KIAA0922 KIAA0947 SUB1 NDUFS4 PCBD2
SLC35A4 TBC1D9B TMEM14B CUL9 GABRR2 GTF3C6 HDDC2 BAZ1B FIS1 TNPO3 RP4
DCTN6 MRPL15 RPS20 ARFGEF1 KLHL38 NDUFB9 CTD CYC1 C9orf46 ERP44
NIPSNAP3B RABGAP1 PSMB7 AK1 RP11 METTL11A FCN2 KIAA0913
SMC3 NUDT22 RELA YIF1A PPP6R3 NUMA1 LAMTOR1 SPCS2 ERC1 MRPL51
SMARCC2 ANKRD52 GLIPR1L1 NDUFA12 SLC25A3 ALDH1L2 TCP11L2 ISCU KDM2B MED4
MED4-AS1 SALL2 CEBPE C14orf182 CDC42BPB HERC2 TPM1
DENND4A UBL7 COX5A SIN3A MRPS11 ITFG3 MRPL28 ZNF598 CREBBP SETD1A RBL2
COX4I1 PSMB6 DVL2 CTC1 POLDIP2 SYNRG SOCS7 C17orf53 NPEPPS
TBKBP1 SNF8 SLC35B1 ICT1 RNF213 C18orf32 HAUS8 POP4 COX6B1
PPP6R1 CHMP2A CBFA2T2 CEP250 ATP5O MRPL40 LDOC1L PRDX4 SAT1 HSD17B10
COX7B FAM127A MT

GOBPID	Pvalue	OddsRatio	ExpCount	Count	Size	Term
GO:0022904	7.46040687237286e-11	20.0630434782609	0.684243304961594	11	96	respiratory electron transport chain
GO:0022900	2.78209342845419e-09	13.8277659950513	0.955089613175559	11	134	electron transport chain
GO:0045333	5.14719971426576e-09	12.9760205774975	1.01210988858902	11	142	cellular respiration
GO:0015980	2.68791510830652e-07	6.90483749055178	2.18815306899177	13	307	energy derivation by oxidation of organic compounds
GO:0006091	2.0110456640133e-06	5.29436031048146	3.05058473462044	14	428	generation of precursor metabolites and energy

Tissue: Lung=>Adipose_Subcutaneous Type: asymmetric
SourceGene: CHPF(ENSG00000123989.9)
TargetGeneSet: RP4 C1QC RPS6KA1 MTMR9LP PPP1R8P1 VAV3 ADORA3 RHOC
SLAMF8 PEA15 CD84 ARHGAP30 MR1 PTPRC PTPN7 PLEK VAMP8
HCLS1 CD86 RBM47 TXK RP13 RP11 DOCK2 AIF1 DEFA6 AOAH
PKD1L1 IRF5 TSPAN33 TBXAS1 SYK FGD3 CARD9 C1DP3 PIK3AP1 MS4A6A
MS4A7 FOLR2 SLCO2B1 PTPN6 BIN2 NCKAP1L ARHGAP9 SELPLG STX2 GPR65
PLCB2 MCTP2 PRKCB CORO1A CD68 CD209 MYO1F IFI30 LRRC25 HCST CD37
SIGLEC7 CD33 LILRB4 HCK PLTP CYTH4 WAS BTK

GOBPID	Pvalue	OddsRatio	ExpCount	Count	Size	Term
GO:0002757	6.11223291950693e-11	17.7365731567421	0.894401771503702			

12 235 immune response-activating signal transduction
GO:0002253 6.54365716990887e-11 15.2705627705628 1.13798353055152
13 299 activation of immune response
GO:0002764 1.14208734396507e-10 16.7441860465116 0.94387931631029
12 248 immune response-regulating signaling pathway
GO:0046649 5.41088583227107e-10 11.3905349554689 1.64798283855789
14 433 lymphocyte activation
GO:0050778 7.40609348814129e-10 12.3853615520282 1.38537125458446
13 364 positive regulation of immune response
GO:0050853 1.01921517828083e-09 76.5199645075421 0.110372984568542
6 29 B cell receptor signaling pathway
GO:0050867 1.32569903754072e-09 15.4661572052402 0.913431596429313
11 240 positive regulation of cell activation
GO:0050865 4.63022740892164e-09 11.8583573385794 1.30544598989689
12 343 regulation of cell activation
GO:0050851 1.90796505601299e-08 21.1374653098982 0.468133693170023
8 123 antigen receptor-mediated signaling pathway
GO:0042110 2.57057938060402e-08 11.4350649350649 1.21410283025396
11 319 T cell activation
GO:0002429 3.52583829389788e-08 19.4328510638298 0.506193343021244
8 133 immune response-activating cell surface receptor signaling pathway
GO:0002768 6.55712897074683e-08 17.8473091364205 0.548058957857588
8 144 immune response-regulating cell surface receptor signaling pathway
GO:0051249 9.51426925376304e-08 11.4961334961335 1.07708809078956
10 283 regulation of lymphocyte activation
GO:0051251 1.06145383596951e-07 13.4772055719713 0.818282471801259
9 215 positive regulation of lymphocyte activation
GO:0008154 1.7226534053312e-07 20.4366830065359 0.414850183378313
7 109 actin polymerization or depolymerization
GO:0030036 1.92858143964797e-07 9.27116402116402 1.48052037921251
11 389 actin cytoskeleton organization
GO:0002696 2.10535661092062e-07 12.3784937888199 0.886789841533458
9 233 positive regulation of leukocyte activation
GO:0046651 2.58755171194995e-07 14.7711468604048 0.654625977441008
8 172 lymphocyte proliferation
GO:0032943 2.8272629926242e-07 14.5911304793643 0.662237907411252
8 174 mononuclear cell proliferation
GO:0042098 2.96909936061453e-07 18.7678303303303 0.449103868244412
7 118 T cell proliferation
GO:0002694 3.06600623033077e-07 10.064308681672 1.22171476022421 10
321 regulation of leukocyte activation
GO:0070661 3.98656356977441e-07 13.9124480313035 0.692685627292229
8 182 leukocyte proliferation
GO:0030029 5.48532531646207e-07 8.29869358669834 1.64417687357276
11 432 actin filament-based process
GO:0007015 6.73682319805254e-07 12.9334395266811 0.742163172098817
8 195 actin filament organization
GO:0030041 7.97214287029456e-07 22.1911650736244 0.323507023735382
6 85 actin filament polymerization
GO:0051495 9.79249704460455e-07 21.3748133399701 0.334924918690748
6 88 positive regulation of cytoskeleton organization
GO:0030097 1.08560817286059e-06 7.71238938053097 1.76216178811155
11 463 hemopoiesis
GO:0050670 1.14681197787023e-06 15.1783759124088 0.548058957857588
7 144 regulation of lymphocyte proliferation
GO:0032944 1.20147351317198e-06 15.0673309178744 0.55186492284271
7 145 regulation of mononuclear cell proliferation
GO:0071346 1.44578315745954e-06 19.9090909090909 0.357760708601481
6 94 cellular response to interferon-gamma
GO:0070663 1.50869838822367e-06 14.5354020979021 0.57089474776832
7 150 regulation of leukocyte proliferation
GO:0050671 1.6365783844003e-06 19.4639455782313 0.365372638571725
6 96 positive regulation of lymphocyte proliferation

GO:0002252	1.64707071475623e-06	8.28605200945626	1.46910248425714
10	386	immune effector process
GO:0042113	1.72278053118714e-06	14.2337328767123	0.582312642723687
7	153	B cell activation
GO:0050863	1.72969697843863e-06	11.3339326740585	0.841118261711992
8	221	regulation of T cell activation
GO:0032946	1.73941345316669e-06	19.2487104732003	0.369178603556847
6	97	positive regulation of mononuclear cell proliferation
GO:0050871	1.81977169383397e-06	29.2795918367347	0.205522109196595
5	54	positive regulation of B cell activation
GO:0002521	1.84552895574968e-06	9.41734678735718	1.14940142550689
9	302	leukocyte differentiation
GO:0070665	2.08004396836927e-06	18.6304819800261	0.380596498512214
6	100	positive regulation of leukocyte proliferation
GO:0048534	2.08294024346492e-06	7.18595041322314	1.88395266763546
11	495	hemopoietic or lymphoid organ development
GO:0042129	2.20498672155191e-06	18.4330827067669	0.384402463497336
6	101	regulation of T cell proliferation
GO:0032956	2.52346204665754e-06	13.3987903225806	0.616566327589786
7	162	regulation of actin cytoskeleton organization
GO:0050900	3.29870176098336e-06	10.3464523787782	0.917237561414435
8	241	leukocyte migration
GO:0050870	3.61411491160361e-06	12.655487804878 0.650820012455885	7
171	positive regulation of T cell activation
GO:0034341	3.82642501010807e-06	16.665889212828 0.422462113348557	6
111	response to interferon-gamma
GO:0032970	3.90346141969277e-06	12.5012550200803	0.65843194242613
7	173	regulation of actin filament-based process
GO:0045579	4.31188258041909e-06	138.365384615385	0.0342536848660992
3	9	positive regulation of B cell differentiation
GO:0042102	4.96753059948155e-06	23.5	0.251193689018061	5	66
positive regulation of T cell proliferation
GO:0030838	6.23852637015201e-06	40.2464985994398	0.121790879523908
4	32	positive regulation of actin filament polymerization
GO:0002697	1.04599573534299e-05	10.6759020618557	0.76499896200955
7	201	regulation of immune effector process
GO:0030098	1.15198716201983e-05	10.5111040609137	0.776416856964916
7	204	lymphocyte differentiation
GO:0060333	1.20664215297118e-05	19.3540540540541	0.300671233824649
5	79	interferon-gamma-mediated signaling pathway
GO:0050864	1.36387504586515e-05	18.8421052631579	0.308283163794893
5	81	regulation of B cell activation
GO:0046631	1.44825732697815e-05	18.5961038961039	0.312089128780015
5	82	alpha-beta T cell activation
GO:0051258	1.45711156481009e-05	13.0325921413341	0.532835097917099
6	140	protein polymerization
GO:0045088	1.61993107460472e-05	9.94751602564103	0.818282471801259
7	215	regulation of innate immune response
GO:0008064	1.72605505608459e-05	17.895	0.323507023735382	5	85
regulation of actin polymerization or depolymerization
GO:0006909	1.82732688707784e-05	17.6728395061728	0.327312988720504
5	86	phagocytosis
GO:0030832	1.82732688707784e-05	17.6728395061728	0.327312988720504
5	86	regulation of actin filament length
GO:0031347	1.9042756689007e-05	6.95310086073714	1.53380388900422
9	403	regulation of defense response
GO:0031334	1.93316911744593e-05	17.4560975609756	0.331118953705626
5	87	positive regulation of protein complex assembly
Tissue: Lung=>Adipose_Subcutaneous	Type:	asymmetric
SourceGene:	DSC1(ENSG00000134765.5)
TargetGeneSet:	FBXO2	RPL11	KHDRBS1 ANKRD13C	RP4	RPL5	RP11
SNRPE	RCOR3	TTC32	UBC	C1D	TPRKB	DGUOK	PTCD3	RPL31	MMADHC
PPIL3	EEF1B2	CNOT10	TRANK1	RPL29	H1FX	COMMD2	RPL22L1 RPL35A	NUP54

ENOPH1 RPL34 TAF9 BTF3 RPS10 RPL10A RP1 TRMT11 BZW2 C7orf44
ABHD11 SYPL1 NUDT18 SNHG6 RPL30 RPS6 DCTN3 PSMB7 RPL35 RPL7A
NSMCE4A C10orf137 ZBED5 EIF3M RPS25 PHB2 EMG1 COPZ1 RPL18AP3
RPL6 OXA1L GOLGA8J TMEM85 TMOD2 RPS15A RPL26 RPL23A RPL17 RPL19
VPS25 RPL27 RPS15 RPL36 NDUFA11 RPS16 RPL18 C19orf48 NLRP9
ZNF805 RPS5 SOGA1 DPM1 H1F0 RPL9P7 UXT IDH3G

GOBPID	Pvalue	OddsRatio	ExpCount	Count	Size	Term
GO:0006414	6.8517814376163e-41	115.140429338104	0.508615320739049	27	105	translational elongation
GO:0006415	1.07660031965565e-40	130.145454545455	0.440799944640509	26	91	translational termination
GO:0070972	2.39429347011336e-39	98.6020955788398	0.571586741401979	27	118	protein localization to endoplasmic reticulum
GO:0006614	7.61708551753886e-39	106.976985040276	0.508615320739049	26	105	SRP-dependent cotranslational protein targeting to membrane
GO:0006613	1.00628456576097e-38	105.632386363636	0.513459276174659	26	106	cotranslational protein targeting to membrane
GO:0045047	1.00628456576097e-38	105.632386363636	0.513459276174659	26	106	protein targeting to ER
GO:0072599	1.00628456576097e-38	105.632386363636	0.513459276174659	26	106	establishment of protein localization to endoplasmic reticulum
GO:0000184	2.91356155036349e-37	90.7839687194526	0.576430696837589	26	119	nuclear-transcribed mRNA catabolic process, nonsense-mediated decay
GO:0006413	4.36141351998054e-36	71.6115348837209	0.736281226212719	27	152	translational initiation
GO:0000956	9.43996005117733e-35	62.9631509990174	0.818628468618089	27	169	nuclear-transcribed mRNA catabolic process
GO:0019080	2.5199545712287e-34	67.392	0.731437270777109	26	151	viral genome expression
GO:0019083	2.5199545712287e-34	67.392	0.731437270777109	26	151	viral transcription
GO:0006612	3.64064317998685e-34	66.3214030064424	0.741125181648329	26	153	protein targeting to membrane
GO:0043624	3.64064317998685e-34	66.3214030064424	0.741125181648329	26	153	cellular protein complex disassembly
GO:0006402	4.19636154062652e-34	59.1729554905283	0.862224067538579	27	178	mRNA catabolic process
GO:0043241	8.92655360355916e-34	63.7868457300276	0.765344958826379	26	158	protein complex disassembly
GO:0034623	1.29106482638928e-32	56.8270884520885	0.842848245796139	26	174	cellular macromolecular complex disassembly
GO:0006401	2.34022159727951e-32	50.1020381499869	0.993010864300048	27	205	RNA catabolic process
GO:0032984	2.8151867905633e-32	54.9506833036245	0.867068022974189	26	179	macromolecular complex disassembly
GO:0072594	9.99212004379702e-31	47.149897854954	0.988166908864438	26	204	establishment of protein localization to organelle
GO:0019058	2.85830184933422e-29	40.8620842572062	1.11895370562591	26	231	viral infectious cycle
GO:0071845	2.06522322414906e-28	34.5080083250385	1.37568334371324	27	284	cellular component disassembly at cellular level
GO:0022411	3.03273804619751e-28	33.9695268644747	1.39505916545568	27	288	cellular component disassembly
GO:0022415	6.71787336382133e-28	35.7247474747475	1.2594284132586	26	260	viral reproductive process
GO:0016032	1.10406752191663e-25	23.3967749393134	2.18462390146011	29	451	viral reproduction
GO:0033365	1.90883405523007e-22	19.6185733653144	2.29119092104353	27	473	protein localization to organelle
GO:0006605	2.01814587967951e-22	19.5732792258467	2.29603487647914	27	474	protein targeting
GO:0071843	6.65226882536092e-12	15.7288888888889	1.15770534911079	14	239	cellular component biogenesis at cellular level

GO:0022613 4.84441706346201e-11 15.2430486593843 1.08988997301225
13 225 ribonucleoprotein complex biogenesis
GO:0042254 1.23986568456352e-10 19.2425693932695 0.721749359905889
11 149 ribosome biogenesis
GO:0006364 1.64635398508183e-09 22.1870578084556 0.503771365303439
9 104 rRNA processing
GO:0016072 2.71939754178963e-09 20.8602499594222 0.532835097917099
9 110 rRNA metabolic process
GO:0042274 1.88175089364032e-08 85.0177514792899 0.0871911978409799
5 18 ribosomal small subunit biogenesis
GO:0034470 3.96892570148744e-08 12.4482456140351 0.968791087121998
10 200 ncRNA processing
GO:0034660 1.11608774414704e-07 9.59897315353211 1.38052729914885
11 285 ncRNA metabolic process
GO:0042273 2.42665430939766e-07 108.886363636364 0.0581274652273199
4 12 ribosomal large subunit biogenesis
Tissue: Lung=>Adipose_Subcutaneous Type: cluster
SourceGene: DSC1(ENSG00000134765.5)
TargetGeneSet: FBXO2 RPL11 KHDRBS1 ANKRD13C RP4 RPL5 RP11
SNRPE RCOR3 TTC32 UBC C1D TPRKB DGUOK PTCD3 RPL31 MMADHC
PPIL3 EEF1B2 CNOT10 TRANK1 RPL29 H1FX COMMD2 RPL22L1 RPL35A NUP54
ENOPH1 RPL34 TAF9 BTF3 RPS10 RPL10A RP1 TRMT11 BZW2 C7orf44
ABHD11 SYPL1 NUDT18 SNHG6 RPL30 RPS6 DCTN3 PSMB7 RPL35 RPL7A
NSMCE4A C10orf137 ZBED5 EIF3M RPS25 PHB2 EMG1 COPZ1 RPL18AP3
RPL6 OXA1L GOLGA8J TMEM85 TMOD2 RPS15A RPL26 RPL23A RPL17 RPL19
VPS25 RPL27 RPS15 RPL36 NDUFA11 RPS16 RPL18 C19orf48 NLRP9
ZNF805 RPS5 SOGA1 DPM1 H1F0 RPL9P7 UXT IDH3G
GOBPID Pvalue OddsRatio ExpCount Count Size Term
GO:0006414 6.8517814376163e-41 115.140429338104 0.508615320739049
27 105 translational elongation
GO:0006415 1.07660031965565e-40 130.145454545455 0.440799944640509
26 91 translational termination
GO:0070972 2.39429347011336e-39 98.6020955788398 0.571586741401979
27 118 protein localization to endoplasmic reticulum
GO:0006614 7.61708551753886e-39 106.976985040276 0.508615320739049
26 105 SRP-dependent cotranslational protein targeting to membrane
GO:0006613 1.00628456576097e-38 105.632386363636 0.513459276174659
26 106 cotranslational protein targeting to membrane
GO:0045047 1.00628456576097e-38 105.632386363636 0.513459276174659
26 106 protein targeting to ER
GO:0072599 1.00628456576097e-38 105.632386363636 0.513459276174659
26 106 establishment of protein localization to endoplasmic reticulum
GO:0000184 2.91356155036349e-37 90.7839687194526 0.576430696837589
26 119 nuclear-transcribed mRNA catabolic process, nonsense-mediated decay
GO:0006413 4.36141351998054e-36 71.6115348837209 0.736281226212719
27 152 translational initiation
GO:0000956 9.43996005117733e-35 62.9631509990174 0.818628468618089
27 169 nuclear-transcribed mRNA catabolic process
GO:0019080 2.5199545712287e-34 67.392 0.731437270777109 26 151
viral genome expression
GO:0019083 2.5199545712287e-34 67.392 0.731437270777109 26 151
viral transcription
GO:0006612 3.64064317998685e-34 66.3214030064424 0.741125181648329
26 153 protein targeting to membrane
GO:0043624 3.64064317998685e-34 66.3214030064424 0.741125181648329
26 153 cellular protein complex disassembly
GO:0006402 4.19636154062652e-34 59.1729554905283 0.862224067538579
27 178 mRNA catabolic process
GO:0043241 8.92655360355916e-34 63.7868457300276 0.765344958826379
26 158 protein complex disassembly
GO:0034623 1.29106482638928e-32 56.8270884520885 0.842848245796139
26 174 cellular macromolecular complex disassembly
GO:0006401 2.34022159727951e-32 50.1020381499869 0.993010864300048

27	205	RNA catabolic process
GO:0032984	2.8151867905633e-32	54.9506833036245	0.867068022974189
26	179	macromolecular complex disassembly
GO:0072594	9.99212004379702e-31	47.149897854954 0.988166908864438	26
204	establishment of protein localization to organelle
GO:0019058	2.85830184933422e-29	40.8620842572062	1.11895370562591
26	231	viral infectious cycle
GO:0071845	2.06522322414906e-28	34.5080083250385	1.37568334371324
27	284	cellular component disassembly at cellular level
GO:0022411	3.03273804619751e-28	33.9695268644747	1.39505916545568
27	288	cellular component disassembly
GO:0022415	6.71787336382133e-28	35.7247474747475	1.2594284132586 26
260	viral reproductive process
GO:0016032	1.10406752191663e-25	23.3967749393134	2.18462390146011
29	451	viral reproduction
GO:0033365	1.90883405523007e-22	19.6185733653144	2.29119092104353
27	473	protein localization to organelle
GO:0006605	2.01814587967951e-22	19.5732792258467	2.29603487647914
27	474	protein targeting
GO:0071843	6.65226882536092e-12	15.7288888888889	1.15770534911079
14	239	cellular component biogenesis at cellular level
GO:0022613	4.84441706346201e-11	15.2430486593843	1.08988997301225
13	225	ribonucleoprotein complex biogenesis
GO:0042254	1.23986568456352e-10	19.2425693932695	0.721749359905889
11	149	ribosome biogenesis
GO:0006364	1.64635398508183e-09	22.1870578084556	0.503771365303439
9	104	rRNA processing
GO:0016072	2.71939754178963e-09	20.8602499594222	0.532835097917099
9	110	rRNA metabolic process
GO:0042274	1.88175089364032e-08	85.0177514792899	0.0871911978409799
5	18	ribosomal small subunit biogenesis
GO:0034470	3.96892570148744e-08	12.4482456140351	0.968791087121998
10	200	ncRNA processing
GO:0034660	1.11608774414704e-07	9.59897315353211	1.38052729914885
11	285	ncRNA metabolic process
GO:0042273	2.42665430939766e-07	108.886363636364	0.0581274652273199
4	12	ribosomal large subunit biogenesis
Tissue: Lung=>Adipose_Subcutaneous	Type:	cluster
SourceGene:	ENSG00000229344.1
TargetGeneSet:	RP11	DDI2	SDHB	HSPG2	RPL11	RP1	MEAF6	PPCS	RPS8
RPS15AP10	RNF11	JAK1	RPL5	HBXIP	ATP5F1	RAP1A	SETDB1	LCE1C
RPS27	ELL2P1	COPA	CEP350	ARPC5	RPS7	NBAS	BIRC6	SOS1-IT1
CALM2	UBC	GMCL1	DGUOK-AS1	EIF5B	MZT2B	H3F3AP4	COL4A4	MFF
EFHD1	GIGYF2	SNED1	HDLBP	BRK1	RAD54L2	TMF1	PCNP	RPL24	NDUFB4	RYK
PDCD10	DNAJC19	PPP1R2	HTT	BOD1L	OCIAD2	PPM1K	RPL34	NDUFC1	NIPBL	CTD
MRPS36	TAF9	COX7C	ERAP1	SKP1	MTND5P11	SIL1	NDFIP1	TMEM14C	RP3
WDR11	RWDD1	IGF2R	ANKRD61	STARD3NL	TRGV5	ZNF117	TRIM56	RPL19P12
NDUFA5	DCTN6	HOOK3	ARFGEF1	POLR2K	EIF3H	TG	RPL8	CLTA	RFK
ATP6V1G1	RPL35	UBAC1	DNLZ	SEC16A	C10orf31	PRINS	ADRA2A
RPS24	RPL27A	RPS13	CKAP5	BTBD18	DDB1	RNASEH2C	SPCS2	TMEM126B
C11orf73	ATM	SDHD	BACE1	UBE4A	ATP5L	DDX6	SCAF11	MLL4
PFDN5	NACA	LRP1	GNS	RBMS1P1	EEA1	APAF1	VPS29	KNTC1	N6AMT2
MRP63	ESD	MED4	RPL13AP25	COMMD6	CCNB1IP1	C14orf21
CGRRF1	YLPM1	CCNK	VPS39	DUT	SEC11A	MPG	NME3	NDUFB10	RPS15A
VPS53	TLCD2	WDR81	RPL26	ZSWIM7	TOM1L2	RPS7P1	SUPT6H	RFFL	RPL19
RPL27	TNRC6C	ROCK1P1	C18orf32	RPS15	PTPRS	NDUFA11	ZNF121	NDUFB7
SLC35E1	ZNF429	KRTDAP	EIF3K	ZNF587	TMEM230	MKKS	CHD6	RP4	TSHZ2
ATP5J	LINC00310	DDT	TTC28	SLC5A1	APOBEC3C	DYNLT3	USP9X
WDR45	GNL3L	COX7B	G6PD	FUNDC2
GOBPID	Pvalue	OddsRatio	ExpCount	Count	Size	Term
GO:0006614	3.26207169581812e-20	26.1360294117647	1.07535810670542
20	105	SRP-dependent cotranslational protein targeting to membrane
GO:0006613	3.98659510008024e-20	25.8303052325581	1.08559961248357

20 106 cotranslational protein targeting to membrane
GO:0045047 3.98659510008024e-20 25.8303052325581 1.08559961248357
20 106 protein targeting to ER
GO:0072599 3.98659510008024e-20 25.8303052325581 1.08559961248357
20 106 establishment of protein localization to endoplasmic reticulum
GO:0070972 3.78016822177201e-19 22.6482780612245 1.20849768182133
20 118 protein localization to endoplasmic reticulum
GO:0006415 1.33530892782906e-18 26.9905163329821 0.931977025811363
18 91 translational termination
GO:0006413 3.77607043734472e-18 17.8885616397187 1.55670887827832
21 152 translational initiation
GO:0006414 2.00822316971168e-17 22.6249336870027 1.07535810670542
18 105 translational elongation
GO:0006612 7.61369365212001e-17 16.6470864661654 1.56695038405647
20 153 protein targeting to membrane
GO:0072594 1.2786141947727e-16 13.5470957613815 2.08926717874196
22 204 establishment of protein localization to organelle
GO:0000184 2.05224819323133e-16 19.4696115765423 1.21873918759947
18 119 nuclear-transcribed mRNA catabolic process, nonsense-mediated decay
GO:0000956 5.51800123988594e-16 14.8427013422819 1.73081447650682
20 169 nuclear-transcribed mRNA catabolic process
GO:0019080 9.77020477086083e-16 15.8121329574818 1.54646737250017
19 151 viral genome expression
GO:0019083 9.77020477086083e-16 15.8121329574818 1.54646737250017
19 151 viral transcription
GO:0006402 1.53379161530991e-15 13.9883306962025 1.82298802851014
20 178 mRNA catabolic process
GO:0006401 1.89653849367975e-15 12.6882488873673 2.0995086845201 21
205 RNA catabolic process
GO:0043624 1.93095066362258e-14 14.5312820512821 1.56695038405647
18 153 cellular protein complex disassembly
GO:0043241 3.41437332424465e-14 14.0073626373626 1.6181579129472 18
158 protein complex disassembly
GO:0034623 1.85821896107544e-13 12.5565088757396 1.78202200539755
18 174 cellular macromolecular complex disassembly
GO:0019058 2.32531915226996e-13 10.4354265402844 2.36578783475192
20 231 viral infectious cycle
GO:0032984 3.04275127705642e-13 12.1622551361682 1.83322953428828
18 179 macromolecular complex disassembly
GO:0033365 1.97347455481443e-12 6.60608061026149 4.84423223306346
26 473 protein localization to organelle
GO:0022415 2.12750949238825e-12 9.15559895833333 2.66279150231818
20 260 viral reproductive process
GO:0006605 1.36183104177285e-11 6.27138899451355 4.8544737388416 25
474 protein targeting
GO:0022904 1.55909165751853e-11 16.4979919678715 0.983184554702097
13 96 respiratory electron transport chain
GO:0071845 6.95920595890273e-10 7.30670908039329 2.9085876409937 18
284 cellular component disassembly at cellular level
GO:0022411 8.7144524615395e-10 7.19641025641026 2.94955366410629
18 288 cellular component disassembly
GO:0022900 1.09587499561989e-09 11.2865625956535 1.37236177427168
13 134 electron transport chain
GO:0016032 1.14150660593146e-09 5.64672364672365 4.61891910594422
22 451 viral reproduction
GO:0045333 2.24881783961179e-09 10.5806488659202 1.45429382049685
13 142 cellular respiration
GO:0015980 2.41573730619789e-09 6.71418685121107 3.14414227389108
18 307 energy derivation by oxidation of organic compounds
GO:0006091 3.86827183404706e-07 4.69181988742964 4.38336447304685
18 428 generation of precursor metabolites and energy
GO:0042773 7.91887131465248e-06 14.6980419099966 0.481350771572902
6 47 ATP synthesis coupled electron transport

GO:0042775	7.91887131465248e-06	14.6980419099966	0.481350771572902
6	47	mitochondrial ATP synthesis coupled electron transport
Tissue: Adipose_Subcutaneous=>Lung	Type:	asymmetric
SourceGene:	ENSG00000232098.1
TargetGeneSet:	RPL22	SPEN	KIAA0090	RPL11	CSF3R	RPS8	UQCRH	RP4
CSRP3	RPL5	CDC14A	RPS27	RAB4A	RPS7	TCEB1P21	RPL31	MIR1302-3
ILKAP	RPL32	RPL15	RPL29	CNBP	ANAPC13	RP11	RPL35A	RPL34	ACSL1	BTF3
TBCA	HINT1	CTB	ZNF300	HIGD2A	TMEM14C	MDC1	CUTA	USP49	RWDD1
TNFAIP3	HAUS6P1	NAMPT	DOCK5	TCEA1	KIAA1429	RPL30	MRPL41	POLR3A
RPS24	RPS3AP5	IFITM2	EIF3F	RPL27A	RPS13	C11orf31	UBXN1	PRDX5	FAU
AIP	ATP5L	RPS25	C12orf57	IRAK4	NACA	IRAK3	ISCU	C12orf47
USP12	MTIF3	TPT1	CHD8	TRAV41	RPL36A	RPL7AP6	C14orf142	FAN1
RPAP1	LYSMD2	RPL4	SEC11A	NDUFB10	ECI1	RPS15A	PRSS53	GPR97	SPAG7
LSMD1	RPL26	C17orf48	RPS7P1	RPL17	RPL19	MYL12B	GPX4	RPS15	AES
C19orf43	C19orf55	RPL18	RPS11	FPR2	C22orf32	TBC1D22A
IGBP1	RPS4X
GOBPID	Pvalue	OddsRatio	ExpCount	Count	Size	Term
GO:0006614	1.30157353101289e-40	96.2424242424242	0.595806518580029
28	105	SRP-dependent cotranslational protein targeting to membrane
GO:0006415	1.42773035295904e-40	109.725852272727	0.516365649436025
27	91	translational termination
GO:0006613	1.76237998532743e-40	95.0018993352327	0.601480866376029
28	106	cotranslational protein targeting to membrane
GO:0045047	1.76237998532743e-40	95.0018993352327	0.601480866376029
28	106	protein targeting to ER
GO:0072599	1.76237998532743e-40	95.0018993352327	0.601480866376029
28	106	establishment of protein localization to endoplasmic reticulum
GO:0070972	5.25504361418136e-39	82.2658436213992	0.669573039928033
28	118	protein localization to endoplasmic reticulum
GO:0006414	1.21496653414584e-38	89.9433566433566	0.595806518580029
27	105	translational elongation
GO:0006413	2.07569771424529e-37	63.373830342077	0.862500864992042	29
152	translational initiation
GO:0000184	5.41404405242449e-37	76.1816205533597	0.675247387724033
27	119	nuclear-transcribed mRNA catabolic process, nonsense-mediated decay
GO:0019080	1.04959904748575e-35	60.0554049984944	0.856826517196042
28	151	viral genome expression
GO:0019083	1.04959904748575e-35	60.0554049984944	0.856826517196042
28	151	viral transcription
GO:0006612	1.56335148583073e-35	59.0862222222222	0.868175212788042
28	153	protein targeting to membrane
GO:0032984	3.40885401343939e-35	51.8680503144654	1.01570825548405
29	179	macromolecular complex disassembly
GO:0043241	4.12806040857741e-35	56.7937321937322	0.896546951768044
28	158	protein complex disassembly
GO:0034623	7.42987953103547e-34	50.5129375951294	0.987336516504048
28	174	cellular macromolecular complex disassembly
GO:0043624	9.00678986623874e-34	55.4922077922078	0.868175212788042
27	153	cellular protein complex disassembly
GO:0072594	1.88099283904718e-33	44.3801617250674	1.15756695038406
29	204	establishment of protein localization to organelle
GO:0000956	1.58933499772773e-32	49.1842509603073	0.958964777524047
27	169	nuclear-transcribed mRNA catabolic process
GO:0006402	7.01366727036605e-32	46.2234798314269	1.01003390768805
27	178	mRNA catabolic process
GO:0019058	3.04048001079587e-30	36.183908045977	1.31077434087606	28
231	viral infectious cycle
GO:0006401	3.82648578122869e-30	39.1375893769152	1.16324129818006
27	205	RNA catabolic process
GO:0022411	5.75479917681415e-29	29.8091352808334	1.63421216524808
29	288	cellular component disassembly
GO:0022415	9.13227108067185e-29	31.5961047254151	1.47533042696007
28	260	viral reproductive process

GO:0071845 1.13022894149101e-27 28.5853587962963 1.61151477406408
28 284 cellular component disassembly at cellular level
GO:0033365 9.31581315988051e-23 17.1606748257692 2.68396650750813
29 473 protein localization to organelle
GO:0006605 9.88664938193443e-23 17.1208819164723 2.68964085530413
29 474 protein targeting
GO:0016032 4.31396126097778e-22 17.0951755538044 2.55913085599612
28 451 viral reproduction
GO:0042274 4.20414596204079e-08 71.7082917082917 0.102138260328005
5 18 ribosomal small subunit biogenesis
GO:0006364 1.25804355440942e-07 16.0731981981982 0.590132170784029
8 104 rRNA processing
GO:0042254 1.59632331877313e-07 12.5304305283757 0.845477821604041
9 149 ribosome biogenesis
GO:0016072 1.94699862244784e-07 15.1213566507684 0.62417825756003
8 110 rRNA metabolic process
GO:0042273 4.6044454228744e-07 92.0576923076923 0.0680921735520033
4 12 ribosomal large subunit biogenesis
GO:0022613 5.50957393660927e-07 9.14341085271318 1.27672825410006
10 225 ribonucleoprotein complex biogenesis
GO:0071843 9.56637484526541e-07 8.57593401261524 1.35616912324407
10 239 cellular component biogenesis at cellular level
GO:0034470 1.73618969894452e-05 7.98254504504505 1.13486955920006
8 200 ncRNA processing
Tissue: Adipose_Subcutaneous=>Lung Type: cluster
SourceGene: HBE1(ENSG00000213931.1)
TargetGeneSet: DDX11L1 RP11 RPL22 PIK3CD RPL11 RP4 UQCRH LRP8 RPL5
NENF MFSD2B UBC CCDC104 RPL31 DDX11L2 CXCR2P1 RPL24 KLHL6-AS1
RPL35A HTT RPL34 CDC20B BTF3 RFESD EPB41L4A-AS1 U6 TRIM40
LY6G6F C6orf25 RPL10A RPL24P4 TOMM7 CTD RPL30 TLN1 EDF1 PTGDS
RPL27A RPS13 PTPRJ UBXN1 SYT12 ARAP1-AS2 RPS25 C12orf57
KCTD4 TPT1 HS6ST3 HERC1 RPS15A C16orf74 GP1BA SLC46A1 CCR10
ITGA2B PRKCA GLTSCR2 RPS11 SNORD88B LINC00493 WFDC12 PPPDE2 UXT
IGBP1 RPL39
GOBPID Pvalue OddsRatio ExpCount Count Size Term
GO:0006415 5.51818513167869e-25 109.19619047619 0.277074250916892 16
91 translational termination
GO:0006414 6.46485337105153e-24 91.9293739967897 0.31970105875026
16 105 translational elongation
GO:0006614 6.46485337105153e-24 91.9293739967897 0.31970105875026
16 105 SRP-dependent cotranslational protein targeting to membrane
GO:0006613 7.60018665220828e-24 90.9015873015873 0.322745830738357
16 106 cotranslational protein targeting to membrane
GO:0045047 7.60018665220828e-24 90.9015873015873 0.322745830738357
16 106 protein targeting to ER
GO:0072599 7.60018665220828e-24 90.9015873015873 0.322745830738357
16 106 establishment of protein localization to endoplasmic reticulum
GO:0070972 4.69496705520481e-23 80.140056022409 0.35928309459553 16
118 protein localization to endoplasmic reticulum
GO:0000184 5.41431967622629e-23 79.3564493758668 0.362327866583627
16 119 nuclear-transcribed mRNA catabolic process, nonsense-mediated decay
GO:0019080 2.90778262073247e-21 60.410582010582 0.459760570202754 16
151 viral genome expression
GO:0019083 2.90778262073247e-21 60.410582010582 0.459760570202754 16
151 viral transcription
GO:0006413 3.24389404359499e-21 59.9621848739496 0.462805342190852
16 152 translational initiation
GO:0006612 3.61607575891177e-21 59.5203336809176 0.46585011417895
16 153 protein targeting to membrane
GO:0043624 3.61607575891177e-21 59.5203336809176 0.46585011417895
16 153 cellular protein complex disassembly
GO:0043241 6.15518583250217e-21 57.4044265593561 0.481073974119438
16 158 protein complex disassembly

GO:0072594	8.79035658425368e-21	47.8787878787879	0.621133485571933
17	204	establishment of protein localization to organelle
GO:0000956	1.86655759439021e-20	53.2362278244631	0.514566465988513
16	169	nuclear-transcribed mRNA catabolic process
GO:0034623	3.01350515566819e-20	51.5334538878843	0.529790325929001
16	174	cellular macromolecular complex disassembly
GO:0006402	4.3747520852651e-20	50.2469135802469	0.541969413881392
16	178	mRNA catabolic process
GO:0032984	4.79532045155869e-20	49.9351446099912	0.54501418586949
16	179	macromolecular complex disassembly
GO:0006401	4.37959984370942e-19	42.9871504157218	0.62417825756003
16	205	RNA catabolic process
GO:0019058	3.02228381570828e-18	37.7196013289037	0.703342329250571
16	231	viral infectious cycle
GO:0022415	2.01868487634857e-17	33.1686182669789	0.791640716905404
16	260	viral reproductive process
GO:0071845	8.2577947910492e-17	30.1471215351812	0.864715244619749
16	284	cellular component disassembly at cellular level
GO:0022411	1.03150092486073e-16	29.6953781512605	0.87689433257214
16	288	cellular component disassembly
GO:0033365	6.33950229792127e-16	21.228740490279 1.44017715037022	18
473	protein localization to organelle
GO:0006605	6.57817992612401e-16	21.1806680161943	1.44322192235831
18	474	protein targeting
GO:0016032	1.16227648846917e-13	18.3540229885057	1.37319216663207
16	451	viral reproduction
GO:0042273	5.68413960043026e-06	117.056910569106	0.0365372638571725
3	12	ribosomal large subunit biogenesis
Tissue: Lung=>Adipose_Subcutaneous	Type:	cluster
SourceGene:	HSPB1P2(ENSG00000230216.1)
TargetGeneSet:	RPS6KA1	SYTL1	C1orf38	CSF3R	VAV3	CD53	RSBN1	CTSS	CD84
ARHGAP30	PTPRC	ADIPOR1	MERTK	CXXC11	NUP210	PP2D1	HCLS1	CD86
P2RY13	RPL7L1P8	TLR1	TXK	EMB	GZMK	MAP3K1	IQGAP2	CD14
DOCK2	GRK6	DOK3	DEFA6	OSBPL3	IKZF1	TFEC	TBXAS1	LYN	KIF24	PAX5
SYK	CARD9	MRC1	FAM21C	TNKS2	TM9SF3	PIK3AP1	FRAT1	SNORA19	KCNQ1
KCNQ1OT1	MS4A6A	MS4A4E	MS4A7	LGALS12	CCDC88B	TBC1D10C	SLCO2B1	CTSC
CRTAM	PTPN6	CD163	C3AR1	SELPLG	ELF1	LCP1	CLCN7	C16orf54
ADCY7	RNF166	CXCL16	PIK3R5	EVI2B	FMNL1	BTBD17	HMHA1	DENND1C	VAV1
MAN2B1	LRRC25	GMIP	HCST	SPINT2	CEACAM4	CD37	CTD	CD33	SIGLEC5
LILRA2	LILRB1	LILRB4	CASS4	ZBP1	SH3BP1	WAS	API5P1
GOBPID	Pvalue	OddsRatio	ExpCount	Count	Size	Term
GO:0002764	2.83025612685634e-10	13.0396453900709	1.25278527437548
13	248	immune response-regulating signaling pathway
GO:0002757	1.9787310374037e-09	12.4869514077777	1.18711507854128
12	235	immune response-activating signal transduction
GO:0050778	2.9229016976017e-08	8.65864197530864	1.83876548335755
13	364	positive regulation of immune response
GO:0002253	2.94324989621189e-08	9.65853658536585	1.51041450418656
12	299	activation of immune response
GO:0042110	5.99918653887682e-08	9.01650024029476	1.61144557470071
12	319	T cell activation
GO:0045088	1.18297775136088e-07	10.9740611691831	1.08608400802713
10	215	regulation of innate immune response
GO:0001817	2.1639261029611e-07	7.95445741011953	1.81350771572902
12	359	regulation of cytokine production
GO:0046649	2.20399140272921e-07	7.20055555555556	2.18732267663137
13	433	lymphocyte activation
GO:0050867	3.28882310531892e-07	9.7639751552795 1.21237284616982	10
240	positive regulation of cell activation
GO:0002252	4.70331852634171e-07	7.36600333128781	1.94989966092312
12	386	immune effector process
GO:0001816	6.00831906475875e-07	7.18828917519154	1.99536364265449
12	395	cytokine production

GO:0002768	6.22527330396889e-07	12.8886877828054	0.727423707701889
8	144	immune response-regulating cell surface receptor signaling pathway
GO:0051251	1.28561864970946e-06	9.67445388349515	1.08608400802713
9	215	positive regulation of lymphocyte activation
GO:0050870	2.28002046053706e-06	10.7333647947145	0.863815652895993
8	171	positive regulation of T cell activation
GO:0002696	2.49899579738861e-06	8.8857421875	1.1770197148986	9
233	positive regulation of leukocyte activation
GO:0050851	2.78889692962869e-06	13.0399686520376	0.62134108366203
7	123	antigen receptor-mediated signaling pathway
GO:0046631	3.48609898204514e-06	16.8523173605656	0.41422738910802
6	82	alpha-beta T cell activation
GO:0002429	4.69291948171638e-06	11.996632996633 0.671856618919106	7
133	immune response-activating cell surface receptor signaling pathway
GO:0031347	5.20921106174128e-06	6.33006912442396	2.03577607086015
11	403	regulation of defense response
GO:0042035	6.4028684164143e-06	15.0584723441615	0.459691370839388
6	91	regulation of cytokine biosynthetic process
GO:0002697	7.55348514979535e-06	9.04583499402152	1.01536225866722
8	201	regulation of immune effector process
GO:0050865	8.19942858096007e-06	6.69479002812336	1.73268285931769
10	343	regulation of cell activation
GO:0002699	8.72980708353583e-06	14.2169154228856	0.484949138467926
6	96	positive regulation of immune effector process
GO:0042089	1.10482670915257e-05	13.6081295649413	0.505155352570756
6	100	cytokine biosynthetic process
GO:0051249	1.20945271920179e-05	7.23859489051095	1.42958964777524
9	283	regulation of lymphocyte activation
GO:0042107	1.23813915098647e-05	13.3227611940299	0.515258459622171
6	102	cytokine metabolic process
GO:0050853	1.29351108289805e-05	33.2823188405797	0.146495052245519
4	29	B cell receptor signaling pathway
GO:0045089	1.3959425733553e-05	10.060202020202 0.793093903536087	7
157	positive regulation of innate immune response
GO:0050863	1.50818786243891e-05	8.18490429758035	1.11639332918137
8	221	regulation of T cell activation
Tissue: Lung=>Adipose_Subcutaneous	Type:	asymmetric
SourceGene:	MARCH10(ENSG00000173838.6)
TargetGeneSet:	TNFRSF4 C1QC	RPS6KA1 C1orf38 LAPTM5	CSF3R	MCOLN2	CD53
ADORA3	ARHGEF11		MNDA	SLAMF8	CD84	ARHGAP30		FCGR2A	FCGR3A
POU2F1	SELL	RP11	PTPRC	CR1	WDR26	GJC2	NLRC4	PLEK	VAMP8
MERTK	TFCP2L1 SLC11A1 CAB39	CDCP1	HCLS1	CD86	P2RY1	PHC3	ATP11B	TFRC
HTT	TLR1	RBM47	WDFY3	SNCA	DAPP1	C4orf21 FAM105A FYB	GM2A
HAVCR2	DOCK2	DOK3	ZNF192	AIF1	GPSM3	DEFA6	TTYH3	AOAH	AKAP9
PILRA	TFEC	IRF5	TBXAS1	MSR1	DOK2	DOCK5	CTD	VPS13B	UBR5	SLA
HEATR7A SYK	FBP1	FKBP15	CARD9	C10orf31		MRC1	WDFY4	PIK3AP1
PTPRE	MUC6	RP13	COMMD9	PTPRJ	MS4A6A	MS4A7	MS4A6E	CCDC88B UNC93B5
SLCO2B1 MYO7A	IL10RA	IFT46	C11orf45		PTPN6	CD163	M6PR	CLEC7A	BIN2
NCKAP1L ARHGAP9 MAPKAPK5		GLT1D1	LCP1	PLCB2	DMXL2	AQP9	DENND4A
FANCI	PRKCB	APOBR	C16orf54		CORO1A	ATP6V0D1		ZZEF1	ARRB2
SNORA48 CD68	PIK3R5	LGALS9	CD300A	HMHA1	C19orf59		CD209	PRAM1
MYO1F	MAN2B1	KLF2	IFI30	LRRC25	GMIP	ZNF714	SPINT2	RASGRP4 PLD3
CEACAM4 CD37	SIGLEC7 FPR2	LILRB2	LILRB1	ZIM2	SIGLEC1 HCK	MMP9
CRYBB1	CYTH4	SH3BP1	TLR8	OFD1	CYBB	WAS	VSIG4	BTK	RP4
ARHGAP4
GOBPID	Pvalue	OddsRatio	ExpCount	Count	Size	Term
GO:0002764	3.17525917561392e-12	10.5078260869565	2.1108573801121 18
248	immune response-regulating signaling pathway
GO:0050778	3.5639430893975e-12	8.39435774309724	3.09819389661615
21	364	positive regulation of immune response
GO:0002253	7.88480122848184e-12	9.16593406593407	2.54494498650612
19	299	activation of immune response
GO:0050865	8.50416276958404e-11	7.89636752136752	2.91945194104214

19	343	regulation of cell activation
GO:0002252	8.5223353516287e-11	7.40728951137991	3.28544737388416
20	386	immune effector process
GO:0002757	1.45183016632472e-10	9.6335936499808 2.0002075980901 16	235
immune response-activating signal transduction
GO:0042098	3.34398676056419e-10	14.5048444671086	1.00435955989205
12	118	T cell proliferation
GO:0046649	6.46625481834432e-10	6.54223183431674	3.68548889350218
20	433	lymphocyte activation
GO:0042110	1.64901707330439e-09	7.44851930526053	2.71517542038613
17	319	T cell activation
GO:0002694	1.8131862504206e-09	7.39846077457795	2.73219846377413
17	321	regulation of leukocyte activation
GO:0046651	2.40778009111816e-09	10.5315608919383	1.46398173136807
13	172	lymphocyte proliferation
GO:0032943	2.77379799910712e-09	10.3992659514399	1.48100477475607
13	174	mononuclear cell proliferation
GO:0002768	3.37344018686564e-09	11.6265356265356	1.22565912393606
12	144	immune response-regulating cell surface receptor signaling pathway
GO:0070661	4.79816032963925e-09	9.9013986013986 1.54909694830808	13
182	leukocyte proliferation
GO:0006897	5.3768325469158e-09	6.84537505752416	2.93647498443014
17	345	endocytosis
GO:0050670	3.89120289605634e-08	10.4823442534909	1.22565912393606
11	144	regulation of lymphocyte proliferation
GO:0032944	4.18065378295491e-08	10.4033848614072	1.23417064563006
11	145	regulation of mononuclear cell proliferation
GO:0070663	5.93542564562744e-08	10.0256294964029	1.27672825410006
11	150	regulation of leukocyte proliferation
GO:0006954	6.94440190076239e-08	5.36061776061776	3.93232302262819
18	462	inflammatory response
GO:0051249	1.26540204035743e-07	6.71279969987381	2.40876063940212
14	283	regulation of lymphocyte activation
GO:0050867	1.28847622001187e-07	7.34132959551462	2.0427652065601 13
240	positive regulation of cell activation
GO:0050853	1.36117103737063e-07	31.8952062430323	0.246834129126012
6	29	B cell receptor signaling pathway
GO:0002697	1.40749861590465e-07	8.08751608751609	1.71081586049408
12	201	regulation of immune effector process
GO:0042129	1.88840355589208e-07	12.2162471395881	0.859663691094042
9	101	regulation of T cell proliferation
GO:0002429	1.92794548484794e-07	10.2201597237211	1.13203238530206
10	133	immune response-activating cell surface receptor signaling pathway
GO:0001816	2.35237047731635e-07	5.50351392005524	3.36205106913016
16	395	cytokine production
GO:0045088	2.92116919480973e-07	7.5223006257489 1.82997716421009	12
215	regulation of innate immune response
GO:0001817	3.89407282254098e-07	5.64599483204134	3.05563628814615
15	359	regulation of cytokine production
GO:0006909	6.674296027887e-07	12.7090301003344	0.731990865684036
8	86	phagocytosis
GO:0050764	7.43106896175798e-07	22.9102564102564	0.323437824372016
6	38	regulation of phagocytosis
GO:0050851	1.01746170274446e-06	9.84349030470914	1.04691716836205
9	123	antigen receptor-mediated signaling pathway
GO:0002443	1.82900287747965e-06	6.93785714285714	1.79593107743409
11	211	leukocyte mediated immunity
GO:0051251	2.19631347999062e-06	6.79989495798319	1.82997716421009
11	215	positive regulation of lymphocyte activation
GO:0050863	2.86867805888058e-06	6.60280612244898	1.88104629437409
11	221	regulation of T cell activation
GO:0002274	3.5334009097762e-06	9.99841897233201	0.910732821258044
8	107	myeloid leukocyte activation

GO:0002696 4.77291829778004e-06 6.2405888030888 1.9831845547021 11 233
positive regulation of leukocyte activation
GO:0001911 5.94319790784005e-06 179.075 0.0425576084700021 3 5
negative regulation of leukocyte mediated cytotoxicity
GO:0030100 7.33596052824878e-06 8.99162055335968 1.00435955989205
8 118 regulation of endocytosis
GO:0031347 8.362296822036e-06 4.60239145303177 3.43014324268217
14 403 regulation of defense response
GO:0030097 9.01559837148166e-06 4.3030753968254 3.94083454432219 15
463 hemopoiesis
GO:0002263 9.96894014034679e-06 8.59765595463138 1.04691716836205
8 123 cell activation involved in immune response
GO:0002366 9.96894014034679e-06 8.59765595463138 1.04691716836205
8 123 leukocyte activation involved in immune response
GO:0031342 1.18124064034452e-05 119.375 0.0510691301640025 3 6
negative regulation of cell killing
GO:0002703 1.31223329447269e-05 10.1116632860041 0.783059995848038
7 92 regulation of leukocyte mediated immunity
GO:0050870 1.52829267162052e-05 6.90350877192982 1.45547020967407
9 171 positive regulation of T cell activation
GO:0032675 1.53764606366223e-05 12.8394062078273 0.536225866722026
6 63 regulation of interleukin-6 production
GO:0042116 1.65034237097815e-05 18.3551617873652 0.323437824372016
5 38 macrophage activation
GO:0050671 1.73595233003197e-05 9.65449438202247 0.81710608262404
7 96 positive regulation of lymphocyte proliferation
GO:0032635 1.84353107544292e-05 12.4024337244676 0.553248910110027
6 65 interleukin-6 production
GO:0032946 1.85789172614029e-05 9.54655172413793 0.82561760431804
7 97 positive regulation of mononuclear cell proliferation
GO:0048534 1.98443236597909e-05 4.00694444444444 4.21320323853021
15 495 hemopoietic or lymphoid organ development
Tissue: Lung=>Adipose_Subcutaneous Type: cluster
SourceGene: MARCH10(ENSG00000173838.6)
TargetGeneSet: TNFRSF4 C1QC RPS6KA1 C1orf38 LAPTM5 CSF3R MCOLN2 CD53
ADORA3 ARHGEF11 MNDA SLAMF8 CD84 ARHGAP30 FCGR2A FCGR3A
POU2F1 SELL RP11 PTPRC CR1 WDR26 GJC2 NLRC4 PLEK VAMP8
MERTK TFCP2L1 SLC11A1 CAB39 CDCP1 HCLS1 CD86 P2RY1 PHC3 ATP11B TFRC
HTT TLR1 RBM47 WDFY3 SNCA DAPP1 C4orf21 FAM105A FYB GM2A
HAVCR2 DOCK2 DOK3 ZNF192 AIF1 GPSM3 DEFA6 TTYH3 AOAH AKAP9
PILRA TFEC IRF5 TBXAS1 MSR1 DOK2 DOCK5 CTD VPS13B UBR5 SLA
HEATR7A SYK FBP1 FKBP15 CARD9 C10orf31 MRC1 WDFY4 PIK3AP1
PTPRE MUC6 RP13 COMMD9 PTPRJ MS4A6A MS4A7 MS4A6E CCDC88B UNC93B5
SLCO2B1 MYO7A IL10RA IFT46 C11orf45 PTPN6 CD163 M6PR CLEC7A BIN2
NCKAP1L ARHGAP9 MAPKAPK5 GLT1D1 LCP1 PLCB2 DMXL2 AQP9 DENND4A
FANCI PRKCB APOBR C16orf54 CORO1A ATP6V0D1 ZZEF1 ARRB2
SNORA48 CD68 PIK3R5 LGALS9 CD300A HMHA1 C19orf59 CD209 PRAM1
MYO1F MAN2B1 KLF2 IFI30 LRRC25 GMIP ZNF714 SPINT2 RASGRP4 PLD3
CEACAM4 CD37 SIGLEC7 FPR2 LILRB2 LILRB1 ZIM2 SIGLEC1 HCK MMP9
CRYBB1 CYTH4 SH3BP1 TLR8 OFD1 CYBB WAS VSIG4 BTK RP4
ARHGAP4
GOBPID Pvalue OddsRatio ExpCount Count Size Term
GO:0002764 3.17525917561392e-12 10.5078260869565 2.1108573801121 18
248 immune response-regulating signaling pathway
GO:0050778 3.5639430893975e-12 8.39435774309724 3.09819389661615
21 364 positive regulation of immune response
GO:0002253 7.88480122848184e-12 9.16593406593407 2.54494498650612
19 299 activation of immune response
GO:0050865 8.50416276958404e-11 7.89636752136752 2.91945194104214
19 343 regulation of cell activation
GO:0002252 8.5223353516287e-11 7.40728951137991 3.28544737388416
20 386 immune effector process
GO:0002757 1.45183016632472e-10 9.6335936499808 2.0002075980901 16 235

immune response-activating signal transduction
GO:0042098	3.34398676056419e-10	14.5048444671086	1.00435955989205
12	118	T cell proliferation
GO:0046649	6.46625481834432e-10	6.54223183431674	3.68548889350218
20	433	lymphocyte activation
GO:0042110	1.64901707330439e-09	7.44851930526053	2.71517542038613
17	319	T cell activation
GO:0002694	1.8131862504206e-09	7.39846077457795	2.73219846377413
17	321	regulation of leukocyte activation
GO:0046651	2.40778009111816e-09	10.5315608919383	1.46398173136807
13	172	lymphocyte proliferation
GO:0032943	2.77379799910712e-09	10.3992659514399	1.48100477475607
13	174	mononuclear cell proliferation
GO:0002768	3.37344018686564e-09	11.6265356265356	1.22565912393606
12	144	immune response-regulating cell surface receptor signaling pathway
GO:0070661	4.79816032963925e-09	9.9013986013986 1.54909694830808	13
182	leukocyte proliferation
GO:0006897	5.3768325469158e-09	6.84537505752416	2.93647498443014
17	345	endocytosis
GO:0050670	3.89120289605634e-08	10.4823442534909	1.22565912393606
11	144	regulation of lymphocyte proliferation
GO:0032944	4.18065378295491e-08	10.4033848614072	1.23417064563006
11	145	regulation of mononuclear cell proliferation
GO:0070663	5.93542564562744e-08	10.0256294964029	1.27672825410006
11	150	regulation of leukocyte proliferation
GO:0006954	6.94440190076239e-08	5.36061776061776	3.93232302262819
18	462	inflammatory response
GO:0051249	1.26540204035743e-07	6.71279969987381	2.40876063940212
14	283	regulation of lymphocyte activation
GO:0050867	1.28847622001187e-07	7.34132959551462	2.0427652065601 13
240	positive regulation of cell activation
GO:0050853	1.36117103737063e-07	31.8952062430323	0.246834129126012
6	29	B cell receptor signaling pathway
GO:0002697	1.40749861590465e-07	8.08751608751609	1.71081586049408
12	201	regulation of immune effector process
GO:0042129	1.88840355589208e-07	12.2162471395881	0.859663691094042
9	101	regulation of T cell proliferation
GO:0002429	1.92794548484794e-07	10.2201597237211	1.13203238530206
10	133	immune response-activating cell surface receptor signaling pathway
GO:0001816	2.35237047731635e-07	5.50351392005524	3.36205106913016
16	395	cytokine production
GO:0045088	2.92116919480973e-07	7.5223006257489 1.82997716421009	12
215	regulation of innate immune response
GO:0001817	3.89407282254098e-07	5.64599483204134	3.05563628814615
15	359	regulation of cytokine production
GO:0006909	6.674296027887e-07	12.7090301003344	0.731990865684036
8	86	phagocytosis
GO:0050764	7.43106896175798e-07	22.9102564102564	0.323437824372016
6	38	regulation of phagocytosis
GO:0050851	1.01746170274446e-06	9.84349030470914	1.04691716836205
9	123	antigen receptor-mediated signaling pathway
GO:0002443	1.82900287747965e-06	6.93785714285714	1.79593107743409
11	211	leukocyte mediated immunity
GO:0051251	2.19631347999062e-06	6.79989495798319	1.82997716421009
11	215	positive regulation of lymphocyte activation
GO:0050863	2.86867805888058e-06	6.60280612244898	1.88104629437409
11	221	regulation of T cell activation
GO:0002274	3.5334009097762e-06	9.99841897233201	0.910732821258044
8	107	myeloid leukocyte activation
GO:0002696	4.77291829778004e-06	6.2405888030888 1.9831845547021 11	233
positive regulation of leukocyte activation
GO:0001911	5.94319790784005e-06	179.075 0.0425576084700021	3	5
negative regulation of leukocyte mediated cytotoxicity

GO:0030100	7.33596052824878e-06	8.99162055335968	1.00435955989205
8	118	regulation of endocytosis
GO:0031347	8.362296822036e-06	4.60239145303177	3.43014324268217
14	403	regulation of defense response
GO:0030097	9.01559837148166e-06	4.3030753968254 3.94083454432219	15
463	hemopoiesis
GO:0002263	9.96894014034679e-06	8.59765595463138	1.04691716836205
8	123	cell activation involved in immune response
GO:0002366	9.96894014034679e-06	8.59765595463138	1.04691716836205
8	123	leukocyte activation involved in immune response
GO:0031342	1.18124064034452e-05	119.375 0.0510691301640025	3	6
negative regulation of cell killing
GO:0002703	1.31223329447269e-05	10.1116632860041	0.783059995848038
7	92	regulation of leukocyte mediated immunity
GO:0050870	1.52829267162052e-05	6.90350877192982	1.45547020967407
9	171	positive regulation of T cell activation
GO:0032675	1.53764606366223e-05	12.8394062078273	0.536225866722026
6	63	regulation of interleukin-6 production
GO:0042116	1.65034237097815e-05	18.3551617873652	0.323437824372016
5	38	macrophage activation
GO:0050671	1.73595233003197e-05	9.65449438202247	0.81710608262404
7	96	positive regulation of lymphocyte proliferation
GO:0032635	1.84353107544292e-05	12.4024337244676	0.553248910110027
6	65	interleukin-6 production
GO:0032946	1.85789172614029e-05	9.54655172413793	0.82561760431804
7	97	positive regulation of mononuclear cell proliferation
GO:0048534	1.98443236597909e-05	4.00694444444444	4.21320323853021
15	495	hemopoietic or lymphoid organ development
Tissue: Lung=>Adipose_Subcutaneous	Type:	asymmetric
SourceGene:	RP11-131H24.4(ENSG00000258987.1)
TargetGeneSet:	GNL2	WDR65	RPS8	RPL5	RP11	FAM46C	S100A6	EEF1B2
C2orf57 ZNF502	GRM2	ACTL6A	SLC30A9 EXOC7	BRD9	CTD	AGGF1	SNX24
ZNF300	TDP2	RARS2	DNAJC2	NAA38	ARF5	RPS20	SNHG6	DCAF13	RPL8	RPS6
NFX1	TXN	RPL12	CDNF	SLC39A13		SLC35E3 RPL18AP3		RPLP0
PRHOXNB CCDC122 GMFB	WDHD1	LGALS3	TRMT5	RPS3AP6 RPLP1	RPS17L	ABCC6
LCMT1	DDX19B	RAC3	RPS15	RPL36	DPY19L3 RPS19	C19orf48		RPS9	DTD1
DMC1	EEF1B2P3		IGBP1-AS1
GOBPID	Pvalue	OddsRatio	ExpCount	Count	Size	Term
GO:0006414	4.47492340187973e-20	73.4153846153846	0.31970105875026
14	105	translational elongation
GO:0006415	4.44824292612228e-19	77.0376344086021	0.277074250916892
13	91	translational termination
GO:0006614	3.1495736053317e-18	65.2507012622721	0.31970105875026
13	105	SRP-dependent cotranslational protein targeting to membrane
GO:0006613	3.58264347410442e-18	64.5445716267777	0.322745830738357
13	106	cotranslational protein targeting to membrane
GO:0045047	3.58264347410442e-18	64.5445716267777	0.322745830738357
13	106	protein targeting to ER
GO:0072599	3.58264347410442e-18	64.5445716267777	0.322745830738357
13	106	establishment of protein localization to endoplasmic reticulum
GO:0070972	1.52891891748631e-17	57.1201228878648	0.35928309459553
13	118	protein localization to endoplasmic reticulum
GO:0000184	1.71298613237649e-17	56.5772976262934	0.362327866583627
13	119	nuclear-transcribed mRNA catabolic process, nonsense-mediated decay
GO:0019080	4.11922810399803e-16	43.3606825619448	0.459760570202754
13	151	viral genome expression
GO:0019083	4.11922810399803e-16	43.3606825619448	0.459760570202754
13	151	viral transcription
GO:0006413	4.49543126155085e-16	43.0457182640984	0.462805342190852
13	152	translational initiation
GO:0006612	4.90299489292293e-16	42.7352534562212	0.46585011417895
13	153	protein targeting to membrane
GO:0043624	4.90299489292293e-16	42.7352534562212	0.46585011417895

13	153	cellular protein complex disassembly
GO:0043241	7.49987316382628e-16	41.2471635150167	0.481073974119438
13	158	protein complex disassembly
GO:0000956	1.82027593177232e-15	38.3091397849462	0.514566465988513
13	169	nuclear-transcribed mRNA catabolic process
GO:0034623	2.66953271065401e-15	37.1063915047085	0.529790325929001
13	174	cellular macromolecular complex disassembly
GO:0006402	3.59626010982814e-15	36.1966764418377	0.541969413881392
13	178	mRNA catabolic process
GO:0032984	3.87009377822261e-15	35.9760979401477	0.54501418586949
13	179	macromolecular complex disassembly
GO:0072594	2.12913434551424e-14	31.212295220402	0.621133485571933	13
204	establishment of protein localization to organelle
GO:0006401	2.26871484396848e-14	31.0475470430108	0.62417825756003
13	205	RNA catabolic process
GO:0019058	1.06290140406222e-13	27.2946137910624	0.703342329250571
13	231	viral infectious cycle
GO:0022415	4.84922544983523e-13	24.0407470288625	0.791640716905404
13	260	viral reproductive process
GO:0071845	1.49424458973798e-12	21.8745387453875	0.864715244619749
13	284	cellular component disassembly at cellular level
GO:0022411	1.78473401619954e-12	21.5502639296188	0.87689433257214
13	288	cellular component disassembly
GO:0033365	6.25701820180238e-11	14.1809731299927	1.44017715037022
14	473	protein localization to organelle
GO:0006605	6.43461749299724e-11	14.1491304347826	1.44322192235831
14	474	protein targeting
GO:0016032	4.82255836927957e-10	13.3743555751952	1.37319216663207
13	451	viral reproduction
GO:0042254	5.38470278183566e-10	26.204693877551	0.453671026226559	9
149	ribosome biogenesis
GO:0022613	1.07083822534352e-09	19.4145006839945	0.685073697321985
10	225	ribonucleoprotein complex biogenesis
GO:0042274	1.72182548076821e-09	141.952662721893	0.0548058957857588
5	18	ribosomal small subunit biogenesis
GO:0071843	1.922874683998e-09	18.2096069868996	0.727700505155353
10	239	cellular component biogenesis at cellular level
GO:0006364	2.51065623580698e-08	27.9102814154361	0.316656286762162
7	104	rRNA processing
GO:0016072	3.71045693642895e-08	26.2734190501181	0.334924918690748
7	110	rRNA metabolic process
GO:0034660	1.98540881113008e-06	11.3357400722022	0.867760016607847
8	285	ncRNA metabolic process
GO:0034470	2.17447811327912e-06	13.933342669094	0.608954397619542	7
200	ncRNA processing
Tissue: Lung=>Adipose_Subcutaneous	Type:	cluster
SourceGene:	RP11-131H24.4(ENSG00000258987.1)
TargetGeneSet:	GNL2	WDR65	RPS8	RPL5	RP11	FAM46C	S100A6	EEF1B2
C2orf57	ZNF502	GRM2	ACTL6A	SLC30A9	EXOC7	BRD9	CTD	AGGF1	SNX24
ZNF300	TDP2	RARS2	DNAJC2	NAA38	ARF5	RPS20	SNHG6	DCAF13	RPL8	RPS6
NFX1	TXN	RPL12	CDNF	SLC39A13		SLC35E3	RPL18AP3		RPLP0
PRHOXNB	CCDC122	GMFB	WDHD1	LGALS3	TRMT5	RPS3AP6	RPLP1	RPS17L	ABCC6
LCMT1	DDX19B	RAC3	RPS15	RPL36	DPY19L3	RPS19	C19orf48		RPS9	DTD1
DMC1	EEF1B2P3		IGBP1-AS1
GOBPID	Pvalue	OddsRatio	ExpCount	Count	Size	Term
GO:0006414	4.47492340187973e-20	73.4153846153846	0.31970105875026
14	105	translational elongation
GO:0006415	4.44824292612228e-19	77.0376344086021	0.277074250916892
13	91	translational termination
GO:0006614	3.1495736053317e-18	65.2507012622721	0.31970105875026
13	105	SRP-dependent cotranslational protein targeting to membrane
GO:0006613	3.58264347410442e-18	64.5445716267777	0.322745830738357
13	106	cotranslational protein targeting to membrane

GO:0045047	3.58264347410442e-18	64.5445716267777	0.322745830738357
13	106	protein targeting to ER
GO:0072599	3.58264347410442e-18	64.5445716267777	0.322745830738357
13	106	establishment of protein localization to endoplasmic reticulum
GO:0070972	1.52891891748631e-17	57.1201228878648	0.35928309459553
13	118	protein localization to endoplasmic reticulum
GO:0000184	1.71298613237649e-17	56.5772976262934	0.362327866583627
13	119	nuclear-transcribed mRNA catabolic process, nonsense-mediated decay
GO:0019080	4.11922810399803e-16	43.3606825619448	0.459760570202754
13	151	viral genome expression
GO:0019083	4.11922810399803e-16	43.3606825619448	0.459760570202754
13	151	viral transcription
GO:0006413	4.49543126155085e-16	43.0457182640984	0.462805342190852
13	152	translational initiation
GO:0006612	4.90299489292293e-16	42.7352534562212	0.46585011417895
13	153	protein targeting to membrane
GO:0043624	4.90299489292293e-16	42.7352534562212	0.46585011417895
13	153	cellular protein complex disassembly
GO:0043241	7.49987316382628e-16	41.2471635150167	0.481073974119438
13	158	protein complex disassembly
GO:0000956	1.82027593177232e-15	38.3091397849462	0.514566465988513
13	169	nuclear-transcribed mRNA catabolic process
GO:0034623	2.66953271065401e-15	37.1063915047085	0.529790325929001
13	174	cellular macromolecular complex disassembly
GO:0006402	3.59626010982814e-15	36.1966764418377	0.541969413881392
13	178	mRNA catabolic process
GO:0032984	3.87009377822261e-15	35.9760979401477	0.54501418586949
13	179	macromolecular complex disassembly
GO:0072594	2.12913434551424e-14	31.212295220402	0.621133485571933	13
204	establishment of protein localization to organelle
GO:0006401	2.26871484396848e-14	31.0475470430108	0.62417825756003
13	205	RNA catabolic process
GO:0019058	1.06290140406222e-13	27.2946137910624	0.703342329250571
13	231	viral infectious cycle
GO:0022415	4.84922544983523e-13	24.0407470288625	0.791640716905404
13	260	viral reproductive process
GO:0071845	1.49424458973798e-12	21.8745387453875	0.864715244619749
13	284	cellular component disassembly at cellular level
GO:0022411	1.78473401619954e-12	21.5502639296188	0.87689433257214
13	288	cellular component disassembly
GO:0033365	6.25701820180238e-11	14.1809731299927	1.44017715037022
14	473	protein localization to organelle
GO:0006605	6.43461749299724e-11	14.1491304347826	1.44322192235831
14	474	protein targeting
GO:0016032	4.82255836927957e-10	13.3743555751952	1.37319216663207
13	451	viral reproduction
GO:0042254	5.38470278183566e-10	26.204693877551	0.453671026226559	9
149	ribosome biogenesis
GO:0022613	1.07083822534352e-09	19.4145006839945	0.685073697321985
10	225	ribonucleoprotein complex biogenesis
GO:0042274	1.72182548076821e-09	141.952662721893	0.0548058957857588
5	18	ribosomal small subunit biogenesis
GO:0071843	1.922874683998e-09	18.2096069868996	0.727700505155353
10	239	cellular component biogenesis at cellular level
GO:0006364	2.51065623580698e-08	27.9102814154361	0.316656286762162
7	104	rRNA processing
GO:0016072	3.71045693642895e-08	26.2734190501181	0.334924918690748
7	110	rRNA metabolic process
GO:0034660	1.98540881113008e-06	11.3357400722022	0.867760016607847
8	285	ncRNA metabolic process
GO:0034470	2.17447811327912e-06	13.933342669094	0.608954397619542	7
200	ncRNA processing
Tissue: Lung=>Adipose_Subcutaneous	Type:	asymmetric

SourceGene: RP11-156P1.1(ENSG00000263142.1)
TargetGeneSet: SDHB DNAJC8 PRDX3P2 ZMYM4 MEAF6 NFYC RPL5 ATP5F1
LCE1C ELL2P1 COPA MGST3 CEP350 CICP13 RPS7 BIRC6 SOS1-IT1
CALM2 COX5B UGGT1 H3F3AP4 FTH1P20 COL4A4 EFHD1 SNED1 HDLBP RP11
RPL24 NDUFB4 CNBP RYK NME9 DNAJC19 PSAPL1 BOD1L CD38 TXK
ANKRD17 PPM1K NDUFC1 CTD TBCA ELL2 ISOC1 SKP1 MTND5P11
TRIM41 RPL15P3 AGPAT1 RP3 COX7A2 RWDD1 CYCS SEPT7 RP4 SYPL1
NDUFA5 FSCN3 CHCHD3 DCTN6 VDAC3 HOOK3 ARFGEF1 OXR1 EIF3E ATP6V1G1
RPL7A SEC16A ATP5C1 C10orf31 VDAC2 C10orf99 NDUFB8 FAM196A
RPL27A CKAP5 NDUFS3 ZBTB3 C11orf73 SDHD UBE4A ATP5L DDX6
NCKAP5L PFDN5 NACA LRP1 GNS CPM CCDC59 RPL6 GOLGA3 MRP63 ESD
CGRRF1 VPS39 SEC11A MPG NDUFB10 ECI1 RPS15A SMG1 MT1X VPS53
TLCD2 WDR81 RPL26 CCDC144B RPS7P1 RFFL RPL19 SUMO2 TNRC6C EPG5
LINGO3 PTPRS CTB ZNF121 ZNF490 BRD4 ZNF429 KRTDAP EIF3K ZNF587
SAMHD1 LBP CHD6 FAM65C ATP5J ATP5O SNRPD3 TNRC6B DYNLT3 USP9X
GNL3L COX7B
GOBPID Pvalue OddsRatio ExpCount Count Size Term
GO:0022904 1.9149620777185e-17 30.0210526315789 0.737388416026573
16 96 respiratory electron transport chain
GO:0022900 4.63841446181159e-15 20.2990187332739 1.02927133070376
16 134 electron transport chain
GO:0045333 1.17844961179359e-14 18.9994987468672 1.09072036537264
16 142 cellular respiration
GO:0015980 1.89261166250888e-11 9.41020203147673 2.35810670541831
18 307 energy derivation by oxidation of organic compounds
GO:0000184 1.74980904851174e-09 14.4955555555556 0.914054390699606
11 119 nuclear-transcribed mRNA catabolic process, nonsense-mediated decay
GO:0006091 4.08077617690187e-09 6.57592446892211 3.28752335478514
18 428 generation of precursor metabolites and energy
GO:0000956 6.40356036300852e-09 10.9500096506466 1.29811085738011
12 169 nuclear-transcribed mRNA catabolic process
GO:0006614 7.43270873231137e-09 14.8462741010943 0.806518580029064
10 105 SRP-dependent cotranslational protein targeting to membrane
GO:0006613 8.15473212166693e-09 14.6905940594059 0.814199709362674
10 106 cotranslational protein targeting to membrane
GO:0045047 8.15473212166693e-09 14.6905940594059 0.814199709362674
10 106 protein targeting to ER
GO:0072599 8.15473212166693e-09 14.6905940594059 0.814199709362674
10 106 establishment of protein localization to endoplasmic reticulum
GO:0006402 1.15049751333791e-08 10.3497626871121 1.3672410213826 12
178 mRNA catabolic process
GO:0006119 1.3082411512096e-08 22.1980582524272 0.445505501349388
8 58 oxidative phosphorylation
GO:0070972 2.31095331448848e-08 13.047304730473 0.906373261365995 10
118 protein localization to endoplasmic reticulum
GO:0006413 2.32705898368316e-08 11.0772340425532 1.16753165870874
11 152 translational initiation
GO:0042776 2.99252715655819e-08 84.5047169811321 0.0998546813369317
5 13 mitochondrial ATP synthesis coupled proton transport
GO:0006415 3.10777257931095e-08 15.3421807747489 0.698982769358522
9 91 translational termination
GO:0006401 5.54930093184093e-08 8.88491128905637 1.57463151339008
12 205 RNA catabolic process
GO:0042773 6.3632460322739e-08 24.0625 0.361013078679676 7 47
ATP synthesis coupled electron transport
GO:0042775 6.3632460322739e-08 24.0625 0.361013078679676 7 47
mitochondrial ATP synthesis coupled electron transport
GO:0006414 1.08983174846975e-07 13.0919117647059 0.806518580029064
9 105 translational elongation
GO:0015985 1.93212855523158e-07 51.9847605224964 0.138260328004982
5 18 energy coupled proton transport, down electrochemical gradient
GO:0015986 1.93212855523158e-07 51.9847605224964 0.138260328004982
5 18 ATP synthesis coupled proton transport

GO:0006612 2.7204775674273e-07 9.82967527521983 1.17521278804235
10 153 protein targeting to membrane
GO:0006120 2.89411785563967e-07 27.2571428571429 0.276520656009965
6 36 mitochondrial electron transport, NADH to ubiquinone
GO:0072594 4.6775763684049e-07 8.06305699481865 1.56695038405647
11 204 establishment of protein localization to organelle
GO:0019080 2.38618692897094e-06 8.82228666114333 1.15985052937513
9 151 viral genome expression
GO:0019083 2.38618692897094e-06 8.82228666114333 1.15985052937513
9 151 viral transcription
GO:0043624 2.66077457554848e-06 8.69852941176471 1.17521278804235
9 153 cellular protein complex disassembly
GO:0043241 3.46915865338344e-06 8.40367153572839 1.2136184347104 9
158 protein complex disassembly
GO:0009206 6.40552059862489e-06 15.1174603174603 0.460867760016608
6 60 purine ribonucleoside triphosphate biosynthetic process
GO:0009145 7.06022074628325e-06 14.8415584415584 0.468548889350218
6 61 purine nucleoside triphosphate biosynthetic process
GO:0034623 7.62820847741854e-06 7.58021390374332 1.33651650404816
9 174 cellular macromolecular complex disassembly
GO:0032984 9.59230485512941e-06 7.35467128027682 1.37492215071621
9 179 macromolecular complex disassembly
GO:0009201 1.0244481206526e-05 13.8314769975787 0.499273406684659
6 65 ribonucleoside triphosphate biosynthetic process
GO:0009142 1.45026023931333e-05 12.9496598639456 0.529997924019099
6 69 nucleoside triphosphate biosynthetic process
Tissue: Lung=>Adipose_Subcutaneous Type: cluster
SourceGene: RP11-160E2.11(ENSG00000262292.1)
TargetGeneSet: RPL22 SDHB KIAA0090 RPL11 RP1 WASF2 DNAJC8 PPCS
RPS8 UQCRH RPL5 RP11 HBXIP RPS27 ELL2P1 GAS5 CEP350 RPS7
BIRC6 UBC TGOLN2 MGAT4A RPL31 CBWD2 H3F3AP4 BRK1 SLC26A6 RPL24
MRPS22 DNAJC19 MRFAP1 MRFAP1L1 CD38 PPM1K GPRIN3 RPL34 RPS3A TAF9
BTF3 TBCA COX7C ISOC1 MTND5P11 CTB RPS14 RPL15P3 AGPAT1
SYNGAP1 WDR11 FOXK1 MPLKIP RPS3AP26 POLR2J MLL3 DRP2 HMBOX1 CTD
DCTN6 HOOK3 RPS20 ARFGEF1 ESRP1 RPL30 EIF3E EIF3H RPS6 RPL35
RPL7A SEC16A RPS24 SFR1 NSMCE4A FAM53B RPL27A RPS13 TSG101 ZBTB3
TMEM151A MED17 CWC15 ATM UBE4A ATP5L DDX6 RPS25 PFDN5 NACA
GNS CPM RPL6 GOLGA3 SLC35E1P1 N6AMT2 ESD COMMD6 TEP1
OXA1L MED6 CCNDBP1 SEC11A NDUFB10 RPS15A VPS53 WDR81 GABARAP RPL26 STX8
RPS7P1 RPL23A ZNHIT3 RPL17 RPL19 RPL27 RPS7P11 RNF213 RPTOR DSG4
RPS15 ZNF121 NACC1 UBA52 KRTDAP EIF3K KLK7 ZNF587 TMEM230 RP4
NCOA3 RPL12P4 DNAJC5 ATP5O DDT EIF3D EIF3L TNRC6B USP9X HCFC1 EMD
FUNDC2
GOBPID Pvalue OddsRatio ExpCount Count Size Term
GO:0006413 2.59532108432318e-42 52.5092328796033 1.22012317486679
35 152 translational initiation
GO:0006415 1.62419912617089e-41 81.6279069767442 0.730468479689987
30 91 translational termination
GO:0006614 3.57187374446435e-41 70.2847376788553 0.842848245796139
31 105 SRP-dependent cotranslational protein targeting to membrane
GO:0006613 5.01928893781292e-41 69.3427450980392 0.850875371946578
31 106 cotranslational protein targeting to membrane
GO:0045047 5.01928893781292e-41 69.3427450980392 0.850875371946578
31 106 protein targeting to ER
GO:0072599 5.01928893781292e-41 69.3427450980392 0.850875371946578
31 106 establishment of protein localization to endoplasmic reticulum
GO:0070972 2.25327351345254e-39 59.7279242731575 0.947200885751851
31 118 protein localization to endoplasmic reticulum
GO:0006414 2.45020924486043e-39 66.3255813953488 0.842848245796139
30 105 translational elongation
GO:0000184 3.02954693644464e-39 59.0450534759358 0.95522801190229
31 119 nuclear-transcribed mRNA catabolic process, nonsense-mediated decay
GO:0000956 2.94901928077747e-37 41.5101878100638 1.35658431942426

33	169	nuclear-transcribed mRNA catabolic process
GO:0006402	1.86114498252538e-36	38.9090153718322	1.42882845477822
33	178	mRNA catabolic process
GO:0019080	1.08105197935443e-35	43.2024509803922	1.21209604871635
31	151	viral genome expression
GO:0019083	1.08105197935443e-35	43.2024509803922	1.21209604871635
31	151	viral transcription
GO:0006612	1.68354596780378e-35	42.4882352941176	1.22815030101723
31	153	protein targeting to membrane
GO:0006401	2.62585967334747e-34	32.7387923788176	1.64556086084008
33	205	RNA catabolic process
GO:0043624	7.0210990329592e-34	40.3062960862167	1.22815030101723
30	153	cellular protein complex disassembly
GO:0043241	1.98407448707917e-33	38.7182049418605	1.26828593176943
30	158	protein complex disassembly
GO:0072594	7.17861863174953e-33	31.3687707641196	1.63753373468964
32	204	establishment of protein localization to organelle
GO:0019058	1.6093317035152e-32	28.3875502008032	1.85426614075151
33	231	viral infectious cycle
GO:0022415	3.50768024999193e-32	25.885279516512 2.08705279911425	34
260	viral reproductive process
GO:0034623	4.35322273150039e-32	34.3774224806202	1.39671995017646
30	174	cellular macromolecular complex disassembly
GO:0032984	1.0701396096537e-31	33.2121117527704	1.43685558092866
30	179	macromolecular complex disassembly
GO:0071845	1.60462062487861e-25	19.3384911188427	2.27970382672479
30	284	cellular component disassembly at cellular level
GO:0022411	2.43955813941746e-25	19.0332612222823	2.31181233132655
30	288	cellular component disassembly
GO:0016032	3.0186688141698e-25	14.4576507597341	3.62023389384818
35	451	viral reproduction
GO:0033365	1.50678394882498e-24	13.7097637972828	3.79683066915784
35	473	protein localization to organelle
GO:0006605	1.617776013179e-24	13.677549987345 3.80485779530828	35
474	protein targeting
GO:0042274	5.29092247522081e-11	83.6263552960801	0.144488270707909
7	18	ribosomal small subunit biogenesis
GO:0071843	7.63415018523914e-10	9.35577263083451	1.91848314995502
15	239	cellular component biogenesis at cellular level
GO:0022613	3.26822337374959e-09	9.18762196821857	1.80610338384887
14	225	ribonucleoprotein complex biogenesis
GO:0042254	3.00501739088863e-08	10.7775707384403	1.19604179641547
11	149	ribosome biogenesis
GO:0006364	1.46867009016005e-07	12.6079685194294	0.834821119645699
9	104	rRNA processing
GO:0016072	2.38452828197917e-07	11.853983529194 0.882983876548336	9
110	rRNA metabolic process
GO:0000028	9.90432079028175e-06	126.83185840708 0.0481627569026365	3
6	ribosomal small subunit assembly
GO:0030490	1.72309942510636e-05	95.1172566371681	0.0561898830530759
3	7	maturation of SSU-rRNA
Tissue: Lung=>Adipose_Subcutaneous	Type:	asymmetric
SourceGene:	RP11-407G23.3(ENSG00000260946.1)
TargetGeneSet:	RPL22	IGSF21	C1QC	RPL11	C1orf38	DNAJC8	THRAP3	MEAF6	RPS8
RPL5	RP11	KCNA2	PTPN22	BOLA1	MRPL9	LCE1C	SPRR2G	FCGR2A	NCF2
TSEN15	PTPRC	RPS7	REL	GMCL1	VAMP5	MMADHC	HOXD8	SLC11A1	RPL32
RPL15	RPL24	NR1I2	HCLS1	CD86	RYK	DNAJC19	HTT	ENOPH1	DAPP1
RPL34	RPS3A	TLR2	MARCH1	FYB	SLC30A5	BTF3	SKP1	CTB	RPS14	TTC1
DOCK2	GNB2L1	RPL15P3	RPS18	WDR11	REV3L	RWDD1	IGF2R	RNASET2	ZNF117
CACNA2D1	GNG11	RPS3AP26	TBXAS1	MLL3	MSR1	CTD	THAP1
HOOK3	RPL30	VPS13B	UBR5	SLA	TRAPPC9	EEF1D	RPL8	RPS6	RPL35
RPL7A	C10orf31	ALOX5	DDX50	RPS24	RPS3AP5	BCCIP	CHID1	KCNQ1
BTBD10	RPS13	TSG101	LRRC55	TRPT1	KRT17	AIP	SPCS2	SLCO2B1	TMEM126B

DDX6 C12orf57 CD163 ITPR2 MCRS1 C12orf44 PFDN5 NACA LRP1
ARHGAP9 U6 TMBIM4 ACTR6 RPL18AP3 SELPLG RPL6 RP1 TPT1 ESD
MED4 COMMD6 TEP1 RIN3 IFI27L1 C15orf41 PLCB2 RPS3AP6 AKAP13 MPG
MEFV RPS15A ADCY7 RPL26 RPS7P1 RPL17 RPL19 RPL27 RPS7P11 SUMO2
ACOX1 RP13 LINGO3 PTPRS MYO1F BRD4 KRTDAP CEACAM4 C5AR1 GPR77
RPL18 RPL13A LENG1 LILRB3 LAIR1 RPS5 PCNA CTSA ATP5O THAP7
EIF3L RPL3 TNRC6B ST13 C22orf32 NCAPH2 TLR8 WAS RPS4X NXF3

GOBPID	Pvalue	OddsRatio	ExpCount	Count	Size	Term
GO:0006415	8.90260066887873e-39	63.7108792846498	0.881599889281019	30	91	translational termination
GO:0006414	2.45146164253578e-38	54.7169600793454	1.0172306414781	31	105	translational elongation
GO:0006614	2.45146164253578e-38	54.7169600793454	1.0172306414781	31	105	SRP-dependent cotranslational protein targeting to membrane
GO:0006613	3.43924825166242e-38	53.9836085626911	1.02691855234932	31	106	cotranslational protein targeting to membrane
GO:0045047	3.43924825166242e-38	53.9836085626911	1.02691855234932	31	106	protein targeting to ER
GO:0072599	3.43924825166242e-38	53.9836085626911	1.02691855234932	31	106	establishment of protein localization to endoplasmic reticulum
GO:0070972	1.51404976931951e-36	46.498365496151	1.14317348280396	31	118	protein localization to endoplasmic reticulum
GO:0000184	9.16360688403386e-35	43.5812053115424	1.15286139367518	30	119	nuclear-transcribed mRNA catabolic process, nonsense-mediated decay
GO:0006413	8.58274082744216e-33	33.3527939949958	1.47256245242544	31	152	translational initiation
GO:0006612	1.06842080691773e-32	33.0770792600391	1.48225036329666	31	153	protein targeting to membrane
GO:0072594	2.1385152007553e-31	25.5025414002295	1.97633381772888	33	204	establishment of protein localization to organelle
GO:0019080	2.27838923299197e-31	31.9834710743802	1.46287454155422	30	151	viral genome expression
GO:0019083	2.27838923299197e-31	31.9834710743802	1.46287454155422	30	151	viral transcription
GO:0000956	2.88251403007626e-31	29.2090812391969	1.63725693723618	31	169	nuclear-transcribed mRNA catabolic process
GO:0043624	3.47869053599559e-31	31.4589800443459	1.48225036329666	30	153	cellular protein complex disassembly
GO:0043241	9.75045185487522e-31	30.2194602272727	1.53068991765276	30	158	protein complex disassembly
GO:0006402	1.57733266169576e-30	27.4033576733446	1.72444813507716	31	178	mRNA catabolic process
GO:0006401	6.33025622993196e-30	24.2140869192892	1.9860217286001	32	205	RNA catabolic process
GO:0034623	2.08415886227826e-29	26.8314393939394	1.68569649159228	30	174	cellular macromolecular complex disassembly
GO:0032984	5.08174098584053e-29	25.921903599756	1.73413604594838	30	179	macromolecular complex disassembly
GO:0019058	6.69876711852795e-27	20.0661009174312	2.23790741125182	31	231	viral infectious cycle
GO:0033365	6.8736100075274e-27	12.8203233256351	4.58238184208705	40	473	protein localization to organelle
GO:0006605	9.44776718180015e-26	12.317377944691	4.59206975295827	39	474	protein targeting
GO:0022415	2.69705410457792e-25	17.4889627819398	2.5188568265172	31	260	viral reproductive process
GO:0071845	6.41542633135444e-23	15.0934144595562	2.75136668742648	30	284	cellular component disassembly at cellular level
GO:0022411	9.68968918339025e-23	14.8551797040169	2.79011833091136	30	288	cellular component disassembly
GO:0016032	3.78840189522482e-20	10.2506148548942	4.36924780292021	33	451	viral reproduction
GO:0042274	6.15001974211389e-07	40.7350427350427	0.17438239568196			

5	18	ribosomal small subunit biogenesis
GO:0042254	1.83285082907639e-06	7.84283342556724	1.44349871981178
10	149	ribosome biogenesis
GO:0022613	1.91185981805711e-06	6.20510563380282	2.1797799460245 12
225	ribonucleoprotein complex biogenesis
GO:0071843	3.57861456959509e-06	5.81662995594714	2.31541069822158
12	239	cellular component biogenesis at cellular level
GO:0006364	7.47797117621961e-06	8.97411616161616	1.00754273060688
8	104	rRNA processing
GO:0016072	1.13291539428615e-05	8.44266191325015	1.0656701958342 8
110	rRNA metabolic process
Tissue: Lung=>Adipose_Subcutaneous	Type:	cluster
SourceGene:	RP11-407G23.3(ENSG00000260946.1)
TargetGeneSet:	RPL22	IGSF21	C1QC	RPL11	C1orf38 DNAJC8	THRAP3	MEAF6	RPS8
RPL5	RP11	KCNA2	PTPN22	BOLA1	MRPL9	LCE1C	SPRR2G	FCGR2A	NCF2
TSEN15	PTPRC	RPS7	REL	GMCL1	VAMP5	MMADHC	HOXD8	SLC11A1 RPL32
RPL15	RPL24	NR1I2	HCLS1	CD86	RYK	DNAJC19 HTT	ENOPH1	DAPP1
RPL34	RPS3A	TLR2	MARCH1	FYB	SLC30A5 BTF3	SKP1	CTB	RPS14	TTC1
DOCK2	GNB2L1	RPL15P3 RPS18	WDR11	REV3L	RWDD1	IGF2R	RNASET2 ZNF117
CACNA2D1	GNG11	RPS3AP26	TBXAS1	MLL3	MSR1	CTD	THAP1
HOOK3	RPL30	VPS13B	UBR5	SLA	TRAPPC9 EEF1D	RPL8	RPS6	RPL35
RPL7A	C10orf31	ALOX5	DDX50	RPS24	RPS3AP5 BCCIP	CHID1	KCNQ1
BTBD10	RPS13	TSG101	LRRC55	TRPT1	KRT17	AIP	SPCS2	SLCO2B1 TMEM126B
DDX6	C12orf57	CD163	ITPR2	MCRS1	C12orf44	PFDN5	NACA	LRP1
ARHGAP9 U6	TMBIM4	ACTR6	RPL18AP3	SELPLG	RPL6	RP1	TPT1	ESD
MED4	COMMD6	TEP1	RIN3	IFI27L1 C15orf41	PLCB2	RPS3AP6 AKAP13	MPG
MEFV	RPS15A	ADCY7	RPL26	RPS7P1	RPL17	RPL19	RPL27	RPS7P11 SUMO2
ACOX1	RP13	LINGO3	PTPRS	MYO1F	BRD4	KRTDAP	CEACAM4 C5AR1	GPR77
RPL18	RPL13A	LENG1	LILRB3	LAIR1	RPS5	PCNA	CTSA	ATP5O	THAP7
EIF3L	RPL3	TNRC6B	ST13	C22orf32	NCAPH2	TLR8	WAS	RPS4X	NXF3
GOBPID	Pvalue	OddsRatio	ExpCount	Count	Size	Term
GO:0006415	8.90260066887873e-39	63.7108792846498	0.881599889281019
30	91	translational termination
GO:0006414	2.45146164253578e-38	54.7169600793454	1.0172306414781 31
105	translational elongation
GO:0006614	2.45146164253578e-38	54.7169600793454	1.0172306414781 31
105	SRP-dependent cotranslational protein targeting to membrane
GO:0006613	3.43924825166242e-38	53.9836085626911	1.02691855234932
31	106	cotranslational protein targeting to membrane
GO:0045047	3.43924825166242e-38	53.9836085626911	1.02691855234932
31	106	protein targeting to ER
GO:0072599	3.43924825166242e-38	53.9836085626911	1.02691855234932
31	106	establishment of protein localization to endoplasmic reticulum
GO:0070972	1.51404976931951e-36	46.498365496151 1.14317348280396	31
118	protein localization to endoplasmic reticulum
GO:0000184	9.16360688403386e-35	43.5812053115424	1.15286139367518
30	119	nuclear-transcribed mRNA catabolic process, nonsense-mediated decay
GO:0006413	8.58274082744216e-33	33.3527939949958	1.47256245242544
31	152	translational initiation
GO:0006612	1.06842080691773e-32	33.0770792600391	1.48225036329666
31	153	protein targeting to membrane
GO:0072594	2.1385152007553e-31	25.5025414002295	1.97633381772888
33	204	establishment of protein localization to organelle
GO:0019080	2.27838923299197e-31	31.9834710743802	1.46287454155422
30	151	viral genome expression
GO:0019083	2.27838923299197e-31	31.9834710743802	1.46287454155422
30	151	viral transcription
GO:0000956	2.88251403007626e-31	29.2090812391969	1.63725693723618
31	169	nuclear-transcribed mRNA catabolic process
GO:0043624	3.47869053599559e-31	31.4589800443459	1.48225036329666
30	153	cellular protein complex disassembly
GO:0043241	9.75045185487522e-31	30.2194602272727	1.53068991765276
30	158	protein complex disassembly

GO:0006402	1.57733266169576e-30	27.4033576733446	1.72444813507716
31	178	mRNA catabolic process
GO:0006401	6.33025622993196e-30	24.2140869192892	1.9860217286001 32
205	RNA catabolic process
GO:0034623	2.08415886227826e-29	26.8314393939394	1.68569649159228
30	174	cellular macromolecular complex disassembly
GO:0032984	5.08174098584053e-29	25.921903599756 1.73413604594838	30
179	macromolecular complex disassembly
GO:0019058	6.69876711852795e-27	20.0661009174312	2.23790741125182
31	231	viral infectious cycle
GO:0033365	6.8736100075274e-27	12.8203233256351	4.58238184208705
40	473	protein localization to organelle
GO:0006605	9.44776718180015e-26	12.317377944691 4.59206975295827	39
474	protein targeting
GO:0022415	2.69705410457792e-25	17.4889627819398	2.5188568265172 31
260	viral reproductive process
GO:0071845	6.41542633135444e-23	15.0934144595562	2.75136668742648
30	284	cellular component disassembly at cellular level
GO:0022411	9.68968918339025e-23	14.8551797040169	2.79011833091136
30	288	cellular component disassembly
GO:0016032	3.78840189522482e-20	10.2506148548942	4.36924780292021
33	451	viral reproduction
GO:0042274	6.15001974211389e-07	40.7350427350427	0.17438239568196
5	18	ribosomal small subunit biogenesis
GO:0042254	1.83285082907639e-06	7.84283342556724	1.44349871981178
10	149	ribosome biogenesis
GO:0022613	1.91185981805711e-06	6.20510563380282	2.1797799460245 12
225	ribonucleoprotein complex biogenesis
GO:0071843	3.57861456959509e-06	5.81662995594714	2.31541069822158
12	239	cellular component biogenesis at cellular level
GO:0006364	7.47797117621961e-06	8.97411616161616	1.00754273060688
8	104	rRNA processing
GO:0016072	1.13291539428615e-05	8.44266191325015	1.0656701958342 8
110	rRNA metabolic process
Tissue: Lung=>Adipose_Subcutaneous	Type:	asymmetric
SourceGene:	SIT1(ENSG00000137078.4)
TargetGeneSet:	MRPL20	RPL22	DNAJC8	TAF12	MAGOH	JTB	RPS27	RP11
RAB4A	ILKAP	RPL24	GRPEL1	RPS3A	GPBP1	RPS10	CHCHD2	RPS3AP26	CUX1
RPL12	RPS3AP5	RPL27A	ARHGAP1	ZNHIT2	MED17	CWC15	RPS25	RPL6	MED6	DLK1
SIVA1	RPS15A	KAT8	RNASEK	ALOX12B	ZNHIT3	RPL17	RNF126	DOHH	C19orf43
AXL	GPR77	RPL18	DDT
GOBPID	Pvalue	OddsRatio	ExpCount	Count	Size	Term
GO:0000184	1.23316123375953e-18	73.1147798742138	0.304684796899869
13	119	nuclear-transcribed mRNA catabolic process, nonsense-mediated decay
GO:0006415	2.98749533634624e-18	87.0987341772152	0.232994256452841
12	91	translational termination
GO:0006414	1.80689935585985e-17	73.9148387096774	0.268839526676355
12	105	translational elongation
GO:0006614	1.80689935585985e-17	73.9148387096774	0.268839526676355
12	105	SRP-dependent cotranslational protein targeting to membrane
GO:0006613	2.03429421514149e-17	73.1234042553191	0.271399903120891
12	106	cotranslational protein targeting to membrane
GO:0045047	2.03429421514149e-17	73.1234042553191	0.271399903120891
12	106	protein targeting to ER
GO:0072599	2.03429421514149e-17	73.1234042553191	0.271399903120891
12	106	establishment of protein localization to endoplasmic reticulum
GO:0070972	7.73556153387231e-17	64.7909433962264	0.302124420455332
12	118	protein localization to endoplasmic reticulum
GO:0000956	1.34046564043452e-16	49.5069444444444	0.432703619126704
13	169	nuclear-transcribed mRNA catabolic process
GO:0006402	2.65916559860613e-16	46.7770202020202	0.455747007127534
13	178	mRNA catabolic process
GO:0072594	1.59305806352616e-15	40.3357329842932	0.522316794685489

13	204	establishment of protein localization to organelle
GO:0019080	1.61037838056575e-15	49.294964028777	0.386616843125043	12
151	viral genome expression
GO:0019083	1.61037838056575e-15	49.294964028777	0.386616843125043	12
151	viral transcription
GO:0006401	1.69826728764919e-15	40.1228298611111	0.524877171130026
13	205	RNA catabolic process
GO:0006413	1.74559508534522e-15	48.9394285714286	0.38917721956958
12	152	translational initiation
GO:0006612	1.89110575840793e-15	48.5889361702128	0.391737596014117
12	153	protein targeting to membrane
GO:0043624	1.89110575840793e-15	48.5889361702128	0.391737596014117
12	153	cellular protein complex disassembly
GO:0043241	2.79924691482103e-15	46.9084931506849	0.4045394782368	12
158	protein complex disassembly
GO:0034623	9.03722727194804e-15	42.2281481481482	0.445505501349388
12	174	cellular macromolecular complex disassembly
GO:0032984	1.27346671987408e-14	40.9494610778443	0.458307383572071
12	179	macromolecular complex disassembly
GO:0019058	2.72312793902935e-13	31.1123287671233	0.59144695868798
12	231	viral infectious cycle
GO:0022415	1.10991614205457e-12	27.418064516129	0.665697875579545	12
260	viral reproductive process
GO:0071845	3.14852810412604e-12	24.9564705882353	0.727146910248426
12	284	cellular component disassembly at cellular level
GO:0022411	3.71207946366908e-12	24.5878260869565	0.737388416026573
12	288	cellular component disassembly
GO:0016032	4.03999074838108e-11	17.2838660578387	1.15472977648606
13	451	viral reproduction
GO:0033365	7.3062961268106e-11	16.4313405797101	1.21105805826586
13	473	protein localization to organelle
GO:0006605	7.50042751609051e-11	16.3945227765727	1.2136184347104	13
474	protein targeting
Tissue: Adipose_Subcutaneous=>Lung	Type:	asymmetric
SourceGene:	WDFY3(ENSG00000163625.11)
TargetGeneSet:	RPL22	RPL11	CSMD2	RPS27	SHE	EIF3FP3	RPL31	KRT18P17
RP11	RPL34	RPL37	EEF1A1P19	EEF1A1	POT1	RPL30	PLIN2	ZFAND5
RPS13	NACA	POP5	RPL4	TMCO7	DNAAF1	FAM108A7P	RPL19	MARCH10
RPS11
GOBPID	Pvalue	OddsRatio	ExpCount	Count	Size	Term
GO:0006414	1.35985670371787e-21	231.258064516129	0.1453186630683	12
105	translational elongation
GO:0006415	5.30064694715127e-20	219.251388888889	0.12594284132586
11	91	translational termination
GO:0006614	2.76818952993206e-19	186.414893617021	0.1453186630683	11
105	SRP-dependent cotranslational protein targeting to membrane
GO:0006613	3.08694207198322e-19	184.439766081871	0.146702650335617
11	106	cotranslational protein targeting to membrane
GO:0045047	3.08694207198322e-19	184.439766081871	0.146702650335617
11	106	protein targeting to ER
GO:0072599	3.08694207198322e-19	184.439766081871	0.146702650335617
11	106	establishment of protein localization to endoplasmic reticulum
GO:0070972	1.05519204053085e-18	163.617860851506	0.163310497543423
11	118	protein localization to endoplasmic reticulum
GO:0000184	1.1619967546663e-18	162.091563786008	0.16469448481074
11	119	nuclear-transcribed mRNA catabolic process, nonsense-mediated decay
GO:0019080	1.73719940600089e-17	124.762698412698	0.208982077364888
11	151	viral genome expression
GO:0019083	1.73719940600089e-17	124.762698412698	0.208982077364888
11	151	viral transcription
GO:0006413	1.87164661224655e-17	123.869188337273	0.210366064632205
11	152	translational initiation
GO:0006612	2.01547134919857e-17	122.988262910798	0.211750051899523

11 153 protein targeting to membrane
GO:0043624 2.01547134919857e-17 122.988262910798 0.211750051899523
11 153 cellular protein complex disassembly
GO:0043241 2.89682250741231e-17 118.763416477702 0.218669988236108
11 158 protein complex disassembly
GO:0000956 6.17981707707572e-17 110.409985935302 0.233893848176597
11 169 nuclear-transcribed mRNA catabolic process
GO:0034623 8.57523165633006e-17 106.985685071575 0.240813784513182
11 174 cellular macromolecular complex disassembly
GO:0006402 1.10671254440759e-16 104.393878908849 0.246349733582451
11 178 mRNA catabolic process
GO:0032984 1.17849536408632e-16 103.765211640212 0.247733720849768
11 179 macromolecular complex disassembly
GO:0072594 5.08842463871518e-16 90.1658031088083 0.282333402532697
11 204 establishment of protein localization to organelle
GO:0006401 5.37383564971277e-16 89.6947308132875 0.283717389800014
11 205 RNA catabolic process
GO:0019058 2.03115432450918e-15 78.95 0.31970105875026 11 231
viral infectious cycle
GO:0022415 7.53863094679851e-15 69.6126729138777 0.359836689502457
11 260 viral reproductive process
GO:0071845 1.99989030693318e-14 63.3854293854294 0.393052383918068
11 284 cellular component disassembly at cellular level
GO:0022411 2.33351167860072e-14 62.4524669073406 0.398588332987337
11 288 cellular component disassembly
GO:0016032 3.16139853576349e-12 38.8638888888889 0.62417825756003
11 451 viral reproduction
GO:0033365 5.30029164637227e-12 36.9550264550265 0.654625977441008
11 473 protein localization to organelle
GO:0006605 5.423028319588e-12 36.8725701943845 0.656009964708325
11 474 protein targeting
Tissue: Lung=>Artery_Tibial Type: asymmetric
SourceGene: AGAP11(ENSG00000151303.10)
TargetGeneSet: ARID1A WASF2 PUM1 TMEM234 HIVEP3 PPCS LRRC39 RP11
S100A6 S100A2 LINC00467 MIA3 ACP1 KIDINS220 KIF3C MTA3
DCTN1 GCFC2 RNF181 COX5B FAM134A INHA GIGYF2 SETD2 CSPG5 IP6K1
WDR82 STAG1 PPP1R2 BOD1L RFC1 WDFY3 WDFY3-AS1 NDUFC1 NIPBL
MAN2A1 YTHDC2 EHMT2 DAXX BRPF3 KIAA0240 PHF3 ARID1B IGF2R
PHF14 SEPT7P2 BAZ1B TMEM168 ARF5 CPA1 AGK WHSC1L1 CHCHD7 UBR5
SLC45A4 MPDZ DENND1A MIR181A2HG STXBP1 CALM2P2 C10orf131 FAM204A UROS
STIM1 PRKCDBP SBF2 ZDHHC5 INCENP RSF1 DYNC2H1 MLL ERC1 GOLT1B
LRRK2 PFKM ATF7 CTDSP2 VSIG10 PDS5B MED4 RP3 HERC2 SMAD3 CIB1
DNM1P46 FAM173A CREBBP KIAA0430 CTCF GLG1 GCSH MBTPS1 RPS4XP17
ZBTB4 LSMD1 C17orf39 THRA WIPF2 STAT5B NMT1 USP32 BPTF DYM
ATP8B1 CTB SAMD4B ARHGAP35 MAVS RP1 NCOA6 CEP250 SOGA1 CHD6
SYS1 STAU1 ATP9A ATP5E CTD MORC2 LGALS1 EP300 TCF20 PPEF1 TFE3
SMC1A EIF4BP7 BCORL1 RAP2C
GOBPID Pvalue OddsRatio ExpCount Count Size Term
GO:0016568 1.99813512363887e-09 7.46901568108823 2.78347519202823
17 419 chromatin modification
GO:0016570 1.22838361857895e-06 7.30141176470588 1.73385924849491
11 261 histone modification
GO:0016569 1.53408747062912e-06 7.12725183823529 1.77371808179365
11 267 covalent chromatin modification
GO:0016571 2.48991183776502e-06 17.9899371069182 0.391945194104214
6 59 histone methylation
GO:0048096 5.60074418648522e-06 154.322580645161 0.0398588332987337
3 6 chromatin-mediated maintenance of transcription
GO:0006479 1.19020364805122e-05 13.41220657277 0.511521694000415 6
77 protein methylation
GO:0008213 1.19020364805122e-05 13.41220657277 0.511521694000415 6
77 protein alkylation
GO:0034968 1.4328298882083e-05 18.7244897959184 0.312227527506747

5	47	histone lysine methylation
Tissue: Artery_Tibial=>Lung	Type:	cluster
SourceGene:	CKS1BP7(ENSG00000254331.1)
TargetGeneSet:	NADK	PIK3CD	ZDHHC18	WDTC1	RP11	HAX1	MNDA	CEP350
RGS18	PRKD3	RMND5A	IL1R2	GCC2	SPOPL	CXCR2	CXCR1	LTF	NICN1-AS1
PRKCD	ACOX2	EIF4E3	ABTB1	TLR1	KLHL8	TLR2	IRF2	FYB	PDF
CYFIP2	DOCK2	E2F3	FAM65B	GPSM3	VNN2	ARID1B	RPS6KA2	SNX13	TRGJP2
RPL13AP17	TSC22D4	HIPK2	TNFRSF10C	KAT6A	SGK196	UBXN2B	NECAB1
RAD21	ZCCHC6	SEMA4D	TRIM14	TNFSF8	TSPAN14	NAALAD2	SORL1	C3AR1	CLEC4D
CLEC4E	TAS2R30	PLXNC1	SLC9A7P1	APAF1	GLT1D1	ULK1	BAZ1A	TECPR2	AQP9
MEFV	UBN1	PRKCB	XPO6	C16orf54	CORO1A	ZNF267	CNOT1	ARRB2
PIK3R5	TRIM16	FMNL1	IL27RA	EMR2	MAST3	KLK5	ZNF765	FCAR	RNF24
OSBPL2	IL17RA	CYTH4	SH3BP1	NFAM1	NUP50	TLR8	CXorf38
GOBPID	Pvalue	OddsRatio	ExpCount	Count	Size	Term
GO:0001816	9.01575364855086e-08	7.80769890996481	2.022697391184	13
395	cytokine production
GO:0001817	1.96600952919472e-06	7.03881590950556	1.83835028717736
11	359	regulation of cytokine production
GO:0050778	2.24798288077245e-06	6.93664283465983	1.86395405162273
11	364	positive regulation of immune response
GO:0002757	3.00931514065176e-06	8.66977535738598	1.20337692893225
9	235	immune response-activating signal transduction
GO:0002764	4.67177874673838e-06	8.19066623752816	1.26994671649021
9	248	immune response-regulating signaling pathway
GO:0060326	5.96165913046696e-06	11.5395117436075	0.696422392913985
7	136	cell chemotaxis
GO:0042113	1.29158075474608e-05	10.183704763852	0.783475192028233	7
153	B cell activation
GO:0050853	1.36553311380717e-05	32.8045714285714	0.148501833783129
4	29	B cell receptor signaling pathway
Tissue: Lung=>Artery_Tibial	Type:	asymmetric
SourceGene:	CTD-2537O9.1(ENSG00000254787.1)
TargetGeneSet:	SDF4	GLTPD1	KLHL21	UBE4B	CASP9	OTUD3	GPATCH3	WDTC1	RP11
WASF2	PTAFR	COL16A1	MTMR9LP	AK2	TMEM53	CYP4B1	CDKN2C	JUN	DBT
SORT1	TSPAN2	PEX11B	FAM63A	C1orf56	S100A10	ZBTB7B	LMNA	RRNAD1	CCDC19	RXRG
PRRX1	FMO2	PTGS2	LMOD1	ADIPOR1	ETNK2	TRIM11	RYR2	VSNL1	PPP1CB
PPM1B	ATOH8	RMND5A	CLASP1	TNFAIP6	CACNB4	GPR155	KANSL1L	ACADL	CNPPD1
ATG9A	IRS1	GPC1	RNPEPL1	JAGN1	SLC25A38	SLC26A6	GPX1	IP6K1
TEX264	SEMA5B	ALDH1L1	ALDH1L1-AS2	KLF15	ZXDC	PPM1L	EGFEM1P	NAALADL2
MCCC1	ABCF3	SNORA81	BDH1	FAM53A	WDR1	CD38	TAPT1	CXCL1	CXCL2	ADH5
ADH4	ADH1A	ADAMTS16	C1QTNF3	PTGER4	JMY	SSBP2	PDLIM4	IRF1
PHF15	KDM3B	PPP2R2B	DPYSL3	GPX3	CTB	FGF18	TUBB	RP3	CDYL
PAK1IP1	GFOD1	LRRC16A	HIST1H1C	DHX16	FKBPL	PRRT1	B3GALT4	CCND3	UBR2
KLHDC3	CDC5L	MUT	PGM3	CNR1	STXBP2	SESN1	CDK19	LAMA2	EPB41L2
KATNA1	SYNJ2	RPS6KA2	WIPI2	BZW2	IL6	HOXA6	CREB5	AQP1	PHKG1	ADSL
WBSCR16	HIP1	FGL2	HGF	ZNF789	C7orf58	HILPDA	FAM180A	ZNF282	ZNF212
DDHD2	PPAPDC1B	FGFR1	SLCO5A1	GRIP1	PVT1	DENND3	ZC3H3	CYHR1
SLC35D2	MSANTD3	ZDHHC12	ZER1	TBC1D13	PHYHD1	ASB13	ITIH5	DHTKD1	CAMK1D	OLAH
ARL5B	PRTFDC1	KIAA1279	EIF4EBP2	ASCC1	DNAJB12	P4HA1	PLAU
FRAT1	ANKRD2	HOGA1	POLL	DUSP5	SMC3	SHOC2	TALDO1	TOLLIP	RRM1	CD44
RP1	PRDM11	PEX16	AMBRA1	ARFGAP2	TNKS1BP1	SLC43A1	ZBTB3	TAF6L
RAB1B	POLDIP2	CDK2AP2	SNORD5	VPS11	C2CD2L	CD9	H2AFJ	FGD4	METTL7A
TENC1	ELK3	TXNRD1	RAB35	PITPNM2	PXMP2	ELF1	FBXL3	ERCC5	IRS2
C14orf93	SLC7A8	PCK2	ARID4A	RHOJ	GALNTL1	ZFYVE1	ALDH6A1	DLST
TGFB3	DIO2	BAHD1	GCHFR	KBTBD13	STRA6	ANKRD34C	PEX11A	CHSY1
MSRB1	FAM100A	CDIPT	RBL2	PDP2	C16orf70	MLYCD	CHMP1A	FAM57A
INPP5K	WDR81	KIF1C	TMEM102	SNORA48	FXR2	ALOX15B	C17orf59	ABHD15	BLMH
CCL2	ERBB2	CSF3	THRA	GHDC	NMT1	KANSL1	CALCOCO2	PDK2	VMP1
PSMC5	SMARCD2	TEX2	SLC39A11	GPRC5C	ACOX1	SPHK1	CBX4	PCYT2
SIRT7	DCXR	C17orf101	HEXDC	APCDD1	C18orf8	VPS4B	PIAS4	UBXN6
RANBP3	ACSBG2	CLPP	INSR	MCOLN1	MAP2K7	SLC44A2	TMED1	ZNF490	WDR83	GCDH
ABHD8	MAP1S	KLHL26	NFKBIB	SHKBP1	RABAC1	PPP1R37	FBXO46	SIX5	HIF3A
SPHK2	BCAT2	SCAF1	AP2A1	MED25	TBC1D17	SIGLEC16	SIGLEC10	EPN1

FKBP1A MAVS MACROD2 CRNKL1 ACSS2 ZHX3 SERINC3 ACOT8 LSM14B STMN3
RCAN1 SLC25A18 PRODH CLTCL1 YPEL1 GGT5 ZNRF3 MCM5 TST MPST
SUN2 CBX6 SNORD83B TAB1 PMM1 TTC38 ZBED1 GPX1P1 REPS2 MAOA
MIR221 ARAF WDR13 VSIG4 TCEAL4 TCEAL3 TMEM164 ABCD1
GOBPID Pvalue OddsRatio ExpCount Count Size Term
GO:0044282 2.06735142438574e-10 6.15278111244498 4.17867275621064
22 218 small molecule catabolic process
GO:0016054 1.85992761282614e-08 5.79398786541644 3.56528959933569
18 186 organic acid catabolic process
GO:0046395 1.85992761282614e-08 5.79398786541644 3.56528959933569
18 186 carboxylic acid catabolic process
GO:0030258 1.45692307301709e-07 6.09038861901457 2.81772887689433
15 147 lipid modification
GO:0072329 2.99830484500555e-07 8.57839451570102 1.51428966853505
11 79 monocarboxylic acid catabolic process
GO:0019395 8.11356084996354e-07 7.67105263157895 1.66763545775379
11 87 fatty acid oxidation
GO:0006635 1.05650632978825e-06 10.0939187043506 1.07342052453117
9 56 fatty acid beta-oxidation
GO:0034440 1.14562991314059e-06 7.37817645379271 1.72514012871082
11 90 lipid oxidation
GO:0032787 2.95377075405425e-06 3.30487855684706 7.6864576845893 23
401 monocarboxylic acid metabolic process
GO:0009062 5.62208561143766e-06 8.03408803440425 1.30343920835928
9 68 fatty acid catabolic process
Tissue: Lung=>Artery_Tibial Type: cluster
SourceGene: CTD-2537O9.1(ENSG00000254787.1)
TargetGeneSet: SDF4 GLTPD1 KLHL21 UBE4B CASP9 OTUD3 GPATCH3 WDTC1 RP11
WASF2 PTAFR COL16A1 MTMR9LP AK2 TMEM53 CYP4B1 CDKN2C JUN DBT
SORT1 TSPAN2 PEX11B FAM63A C1orf56 S100A10 ZBTB7B LMNA RRNAD1 CCDC19 RXRG
PRRX1 FMO2 PTGS2 LMOD1 ADIPOR1 ETNK2 TRIM11 RYR2 VSNL1 PPP1CB
PPM1B ATOH8 RMND5A CLASP1 TNFAIP6 CACNB4 GPR155 KANSL1L ACADL CNPPD1
ATG9A IRS1 GPC1 RNPEPL1 JAGN1 SLC25A38 SLC26A6 GPX1 IP6K1
TEX264 SEMA5B ALDH1L1 ALDH1L1-AS2 KLF15 ZXDC PPM1L EGFEM1P NAALADL2
MCCC1 ABCF3 SNORA81 BDH1 FAM53A WDR1 CD38 TAPT1 CXCL1 CXCL2 ADH5
ADH4 ADH1A ADAMTS16 C1QTNF3 PTGER4 JMY SSBP2 PDLIM4 IRF1
PHF15 KDM3B PPP2R2B DPYSL3 GPX3 CTB FGF18 TUBB RP3 CDYL
PAK1IP1 GFOD1 LRRC16A HIST1H1C DHX16 FKBPL PRRT1 B3GALT4 CCND3 UBR2
KLHDC3 CDC5L MUT PGM3 CNR1 STXBP2 SESN1 CDK19 LAMA2 EPB41L2
KATNA1 SYNJ2 RPS6KA2 WIPI2 BZW2 IL6 HOXA6 CREB5 AQP1 PHKG1 ADSL
WBSCR16 HIP1 FGL2 HGF ZNF789 C7orf58 HILPDA FAM180A ZNF282 ZNF212
DDHD2 PPAPDC1B FGFR1 SLCO5A1 GRIP1 PVT1 DENND3 ZC3H3 CYHR1
SLC35D2 MSANTD3 ZDHHC12 ZER1 TBC1D13 PHYHD1 ASB13 ITIH5 DHTKD1 CAMK1D OLAH
ARL5B PRTFDC1 KIAA1279 EIF4EBP2 ASCC1 DNAJB12 P4HA1 PLAU
FRAT1 ANKRD2 HOGA1 POLL DUSP5 SMC3 SHOC2 TALDO1 TOLLIP RRM1 CD44
RP1 PRDM11 PEX16 AMBRA1 ARFGAP2 TNKS1BP1 SLC43A1 ZBTB3 TAF6L
RAB1B POLDIP2 CDK2AP2 SNORD5 VPS11 C2CD2L CD9 H2AFJ FGD4 METTL7A
TENC1 ELK3 TXNRD1 RAB35 PITPNM2 PXMP2 ELF1 FBXL3 ERCC5 IRS2
C14orf93 SLC7A8 PCK2 ARID4A RHOJ GALNTL1 ZFYVE1 ALDH6A1 DLST
TGFB3 DIO2 BAHD1 GCHFR KBTBD13 STRA6 ANKRD34C PEX11A CHSY1
MSRB1 FAM100A CDIPT RBL2 PDP2 C16orf70 MLYCD CHMP1A FAM57A
INPP5K WDR81 KIF1C TMEM102 SNORA48 FXR2 ALOX15B C17orf59 ABHD15 BLMH
CCL2 ERBB2 CSF3 THRA GHDC NMT1 KANSL1 CALCOCO2 PDK2 VMP1
PSMC5 SMARCD2 TEX2 SLC39A11 GPRC5C ACOX1 SPHK1 CBX4 PCYT2
SIRT7 DCXR C17orf101 HEXDC APCDD1 C18orf8 VPS4B PIAS4 UBXN6
RANBP3 ACSBG2 CLPP INSR MCOLN1 MAP2K7 SLC44A2 TMED1 ZNF490 WDR83 GCDH
ABHD8 MAP1S KLHL26 NFKBIB SHKBP1 RABAC1 PPP1R37 FBXO46 SIX5 HIF3A
SPHK2 BCAT2 SCAF1 AP2A1 MED25 TBC1D17 SIGLEC16 SIGLEC10 EPN1
FKBP1A MAVS MACROD2 CRNKL1 ACSS2 ZHX3 SERINC3 ACOT8 LSM14B STMN3
RCAN1 SLC25A18 PRODH CLTCL1 YPEL1 GGT5 ZNRF3 MCM5 TST MPST
SUN2 CBX6 SNORD83B TAB1 PMM1 TTC38 ZBED1 GPX1P1 REPS2 MAOA
MIR221 ARAF WDR13 VSIG4 TCEAL4 TCEAL3 TMEM164 ABCD1
GOBPID Pvalue OddsRatio ExpCount Count Size Term

GO:0044282	2.06735142438574e-10	6.15278111244498	4.17867275621064
22	218	small molecule catabolic process
GO:0016054	1.85992761282614e-08	5.79398786541644	3.56528959933569
18	186	organic acid catabolic process
GO:0046395	1.85992761282614e-08	5.79398786541644	3.56528959933569
18	186	carboxylic acid catabolic process
GO:0030258	1.45692307301709e-07	6.09038861901457	2.81772887689433
15	147	lipid modification
GO:0072329	2.99830484500555e-07	8.57839451570102	1.51428966853505
11	79	monocarboxylic acid catabolic process
GO:0019395	8.11356084996354e-07	7.67105263157895	1.66763545775379
11	87	fatty acid oxidation
GO:0006635	1.05650632978825e-06	10.0939187043506	1.07342052453117
9	56	fatty acid beta-oxidation
GO:0034440	1.14562991314059e-06	7.37817645379271	1.72514012871082
11	90	lipid oxidation
GO:0032787	2.95377075405425e-06	3.30487855684706	7.6864576845893 23
401	monocarboxylic acid metabolic process
GO:0009062	5.62208561143766e-06	8.03408803440425	1.30343920835928
9	68	fatty acid catabolic process
Tissue: Lung=>Artery_Tibial	Type:	asymmetric
SourceGene:	E2F3(ENSG00000112242.9)
TargetGeneSet:	UQCRH	DNTTIP2	BCAS2	NOTCH2	RP11	ZBTB7B	PLXNA2	MIA3
HS1BP3-IT1	C2orf28	COX5B	NDUFB4	MRPS22	MBNL1	ATP11B	ATP5I	POLN
NDUFC1	WWC2	CTD	COX7C	FER	HINT1	UQCRQ	SPINK5	TBC1D9B	SNORA40	RP3
MRPL2	PSMB1	NDUFA4	MALSU1	ECD	CKS1BP7	COX6C	HEMGN	INVS	LRRC8A
KIAA0913	NAV2-AS2	NDUFA12	MED4	SLIRP	C14orf2	ARHGAP11B
ZNF609	NDE1	SLC12A4	MINK1	ICT1	CTIF	C19orf70	NDUFB7	CYP2G1P
TBC1D20	SNRPB2	LPIN3	ATP5J	MIAT	TCF20	NDUFA1
GOBPID	Pvalue	OddsRatio	ExpCount	Count	Size	Term
GO:0022904	4.56552515661742e-18	62.2981927710843	0.325513805272992
13	96	respiratory electron transport chain
GO:0022900	4.09336789036212e-16	42.6200642791552	0.454363019860217
13	134	electron transport chain
GO:0045333	8.84150635104301e-16	39.9545650301464	0.481489170299633
13	142	cellular respiration
GO:0015980	1.84344044969469e-11	17.3284202569917	1.04096602311259
13	307	energy derivation by oxidation of organic compounds
GO:0006091	1.1205995440234e-09	12.170749665328 1.45124904850875	13
428	generation of precursor metabolites and energy
GO:0042773	1.07011493057609e-08	48.874645490641 0.159366133831569	6
47	ATP synthesis coupled electron transport
GO:0042775	1.07011493057609e-08	48.874645490641 0.159366133831569	6
47	mitochondrial ATP synthesis coupled electron transport
GO:0006119	3.92198943479283e-08	38.5062611806798	0.196664590685766
6	58	oxidative phosphorylation
GO:0006120	1.26601969064591e-07	52.6796187683284	0.122067676977372
5	36	mitochondrial electron transport, NADH to ubiquinone
Tissue: Lung=>Artery_Tibial	Type:	cluster
SourceGene:	FBXO18(ENSG00000134452.12)
TargetGeneSet:	MECR	ZCCHC17	KHDRBS1	PSMB2	OSCP1	RPS8	RP1	RP11
ALG14	RP4	SRGAP2P1	SRGAP2P2	MRPS21	SCNM1	PSMB4	DENND4B	PMF1
MYOC	KLHDC8A	SLC41A1	SRGAP3	LINC00467	TATDN3	KLHL29	C2orf28	RPLP0P6
SFXN5	RNF181	COX5B	CHCHD5	RPL37A	PPP1R7	LSM3	KRBOX1	ZNF852	KIF9	WDR6
SPCS1	CLCP1	BBX	CLDN18	GMPS	SMC4	OCIAD1-AS1	MRPL1	LARP7	WWC2
CTD	ARHGAP26	RPS14	MED7	WRNIP1	TMEM14B	NOL7	HLA-F-AS1	MICD
RP3	COX6A1P2	MRPL2	EYS	C6orf57	RPL39P3	IMPG1	MRPS12	PEX7
SF3B5	PSMB1	ANKMY2	MALSU1	ECD	C7orf61	SSBP1	SLC4A2	AGAP3	KIF13B
CHCHD7	COX6C	UTP23	NDUFB9	SNAPC3	RPS6	DCTN3	FAM205A	RC3H2	FAM129B
FAM208B	FBXO18	CALM2P2	NHP2P1	NDUFB8	TCF7L2	FAM204A	RPLP2	CARS	C11orf10
PLCB3	PPP2R5B	FAU	SIPA1	MRPL11	KDM2A	C11orf1	PCSK7	ATP5L	SPA17
GALNT8	GPD1	ATP5G2	RBMS2	SLC35E3	NDUFA12	CHPT1	RPL18AP3	SSH1
MED13L	RPS11P5	SAP18	PDS5B	WDFY2	OR11H7	RPS29	SLIRP	U6	RCOR1

C14orf2 NDN MAPKBP1 TCF12 RPLP1 KIF7 RAB11FIP3 C16orf79 NDE1
SMG1 ATXN2L DOC2A NUDT21 SLC12A4 GAN KIAA0513 TRAPPC2L
MINK1 ARHGAP44 FOXO3B C17orf51 RPS7P1 USP32 RPL38 CDR2L ICT1
NT5C SLC16A3 FOXK2 ZNF532 PIP5K1C CCDC94 C19orf70 PNPLA6 C19orf53
NDUFA13 COX6B1 POLR2I GGN EIF3K SMG9 SNRPD2 SYNGR4 VSIG10L ZNF8 RPS5
CHMP2A TBC1D20 MCM8 SNRPB2 POLR3F ITCH MT1P3 ROMO1 ATP5J SYNJ1
ZNF280B SAMM50 SBF1 PNPLA4 PSMA6P2 EEF1B2P3 GSPT2 ATRX CXorf56
RPL10 MT
GOBPID Pvalue OddsRatio ExpCount Count Size Term
GO:0006415 3.78849351354665e-12 18.6784776902887 0.881599889281019
13 91 translational termination
GO:0000184 8.24992733445461e-12 15.0328042328042 1.15286139367518
14 119 nuclear-transcribed mRNA catabolic process, nonsense-mediated decay
GO:0000956 8.63920865152007e-11 11.0314285714286 1.63725693723618
15 169 nuclear-transcribed mRNA catabolic process
GO:0006402 1.80682231573148e-10 10.4157055214724 1.72444813507716
15 178 mRNA catabolic process
GO:0043624 2.52635066746848e-10 11.3285371702638 1.48225036329666
14 153 cellular protein complex disassembly
GO:0006414 3.84141034299822e-10 14.3326612903226 1.0172306414781 12
105 translational elongation
GO:0006614 3.84141034299822e-10 14.3326612903226 1.0172306414781 12
105 SRP-dependent cotranslational protein targeting to membrane
GO:0043241 3.88033221363672e-10 10.9313271604938 1.53068991765276
14 158 protein complex disassembly
GO:0006613 4.29631560125202e-10 14.1791888297872 1.02691855234932
12 106 cotranslational protein targeting to membrane
GO:0045047 4.29631560125202e-10 14.1791888297872 1.02691855234932
12 106 protein targeting to ER
GO:0072599 4.29631560125202e-10 14.1791888297872 1.02691855234932
12 106 establishment of protein localization to endoplasmic reticulum
GO:0006401 1.30736137255254e-09 8.91852631578947 1.9860217286001 15
205 RNA catabolic process
GO:0034623 1.38880095640572e-09 9.82708333333333 1.68569649159228
14 174 cellular macromolecular complex disassembly
GO:0070972 1.50897771532649e-09 12.5633844339623 1.14317348280396
12 118 protein localization to endoplasmic reticulum
GO:0032984 2.0125567867469e-09 9.52592592592593 1.73413604594838
14 179 macromolecular complex disassembly
GO:0006612 2.84111606763006e-09 10.3612485939258 1.48225036329666
13 153 protein targeting to membrane
GO:0072594 1.09104705392107e-08 8.25789473684211 1.97633381772888
14 204 establishment of protein localization to organelle
GO:0019080 2.52507886289359e-08 9.55845323741007 1.46287454155422
12 151 viral genome expression
GO:0019083 2.52507886289359e-08 9.55845323741007 1.46287454155422
12 151 viral transcription
GO:0006413 2.71904693879787e-08 9.48950892857143 1.47256245242544
12 152 translational initiation
GO:0071845 1.0540381480096e-07 6.26408921933086 2.75136668742648
15 284 cellular component disassembly at cellular level
GO:0022411 1.26476224341199e-07 6.17054945054945 2.79011833091136
15 288 cellular component disassembly
GO:0022904 3.68580792010241e-07 11.2324295867334 0.930039443637119
9 96 respiratory electron transport chain
GO:0019058 3.79386595355862e-07 6.6173878494546 2.23790741125182 13
231 viral infectious cycle
GO:0022415 1.44555172918941e-06 5.82842934106921 2.5188568265172 13
260 viral reproductive process
GO:0006605 3.34412438836537e-06 4.18989165821636 4.59206975295827
17 474 protein targeting
GO:0022900 6.07869643150417e-06 7.79688549618321 1.29818005674348
9 134 electron transport chain

GO:0016032	7.60754506047406e-06	4.11598071931776	4.36924780292021
16	451	viral reproduction
GO:0045333	9.74864465719486e-06	7.32376743385181	1.37568334371324
9	142	cellular respiration
Tissue: Artery_Tibial=>Lung	Type:	asymmetric
SourceGene:	HOXC-AS5(ENSG00000249641.2)
TargetGeneSet:	RPL22	UBR4	RPL11	SYF2	CEP85	WDTC1	PTAFR	KPNA7
KIAA1522		SLC2A1	ZCCHC11	MAGOH	ALG14	SNX27	TMCO1	UBC	USP34
MYEOV2	VHL	RPL32	LSM3	ZNF852	RPL24	RP11	PSMD2	RPL35A	HTT	UBA6
ANKRD17	RPL34	LARP7	NIPBL	RPL37	NDUFS4	BTF3	TBCA	ARAP3	FOXC1
HNRNPC	C6orf130		RPL24P4	RPL39P3	TOMM7	LSM5	SEC61G	CHCHD2	SBDS
BUD31	PMS2P1	SSBP1	PINX1	DCTN6	C8orf40	RPL30	POLR2K	ATP6V1C1		U6
KIAA1797		OR1K1	NR6A1	NUP188	ETFA	PTPRJ	FAU	KDM2A	C11orf30
RPS25	DPAGT1	ZCRB1	PFDN5	PSG1	DIABLO	SAP18	ESD	HEATR5A	ERH
MAPKBP1	SRRM2	GSPT1	CNOT1	NFAT5	CAMTA2	COPS3	KIAA0100		CRLF3	NF1
WNT3	NPEPPS	MED13	GGA3	SPACA4	SNRPB2	DTD1	RPS21	EEF1B2P3
MIR98	MED12	ZMYM3
GOBPID	Pvalue	OddsRatio	ExpCount	Count	Size	Term
GO:0000956	3.39701043801567e-17	25.2425856307435	0.935575392706387
17	169	nuclear-transcribed mRNA catabolic process
GO:0006401	4.18664409670476e-17	22.0210453682939	1.13486955920006
18	205	RNA catabolic process
GO:0006402	8.2558719796398e-17	23.8164251207729	0.985398934329804
17	178	mRNA catabolic process
GO:0000184	2.87997198583813e-15	28.820202020202	0.658777939242959	14
119	nuclear-transcribed mRNA catabolic process, nonsense-mediated decay
GO:0006614	1.54354116335653e-14	30.114698247891	0.581274652273199	13
105	SRP-dependent cotranslational protein targeting to membrane
GO:0006613	1.75169478408974e-14	29.7887979457551	0.586810601342468
13	106	cotranslational protein targeting to membrane
GO:0045047	1.75169478408974e-14	29.7887979457551	0.586810601342468
13	106	protein targeting to ER
GO:0072599	1.75169478408974e-14	29.7887979457551	0.586810601342468
13	106	establishment of protein localization to endoplasmic reticulum
GO:0070972	7.26938441718237e-14	26.3621890547264	0.65324199017369
13	118	protein localization to endoplasmic reticulum
GO:0006415	7.81242114831192e-14	31.9255398361876	0.503771365303439
12	91	translational termination
GO:0006414	4.54578329727447e-13	27.0929791271347	0.581274652273199
12	105	translational elongation
GO:0019080	1.81349865949945e-12	20.0117888816786	0.835928309459553
13	151	viral genome expression
GO:0019083	1.81349865949945e-12	20.0117888816786	0.835928309459553
13	151	viral transcription
GO:0006612	2.14850737372985e-12	19.7231343283582	0.84700020759809
13	153	protein targeting to membrane
GO:0043624	2.14850737372985e-12	19.7231343283582	0.84700020759809
13	153	cellular protein complex disassembly
GO:0043241	3.24835767185265e-12	19.0363355635615	0.874679952944433
13	158	protein complex disassembly
GO:0072594	5.30806290630558e-12	15.8320574162679	1.12933361013079
14	204	establishment of protein localization to organelle
GO:0034623	1.11386038621489e-11	17.125243348475	0.96325513805273	13
174	cellular macromolecular complex disassembly
GO:0032984	1.59605733806533e-11	16.6035784930768	0.990934883399073
13	179	macromolecular complex disassembly
GO:0006413	3.85621560422897e-11	17.9382352941176	0.841464258528822
12	152	translational initiation
GO:0019058	3.88209261568777e-10	12.59680953033	1.27880423500104	13
231	viral infectious cycle
GO:0033365	6.46696766313897e-10	8.23430103035366	2.61850390976403
17	473	protein localization to organelle
GO:0006605	6.67922392713022e-10	8.21569240387621	2.6240398588333	17

474	protein targeting
GO:0022415	1.65498000540588e-09	11.095051060487 1.43934675800983	13
260	viral reproductive process
GO:0071845	4.82115272069261e-09	10.0952800572782	1.57220953567227
13	284	cellular component disassembly at cellular level
GO:0022411	5.70474276916864e-09	9.94561736770692	1.59435333194935
13	288	cellular component disassembly
GO:0016032	2.12671550358649e-08	7.37561750176429	2.49671303024012
15	451	viral reproduction
GO:0008380	1.11510363562749e-05	6.41624828845276	1.78811154937375
10	323	RNA splicing
GO:0006397	1.17302553785348e-05	5.74529359031824	2.20884367863816
11	399	mRNA processing
GO:0000377	1.66882052411206e-05	8.03571428571429	1.12933361013079
8	204	RNA splicing, via transesterification reactions with bulged adenosine as nucleophile
GO:0000398	1.66882052411206e-05	8.03571428571429	1.12933361013079
8	204	nuclear mRNA splicing, via spliceosome
GO:0000375	1.98771484760446e-05	7.83305693753455	1.15701335547713
8	209	RNA splicing, via transesterification reactions
Tissue: Artery_Tibial=>Lung	Type:	asymmetric
SourceGene:	IGHV3-7(ENSG00000211938.2)
TargetGeneSet:	AMYP1	RP11	IGKJ5	IGKC	IGKJ3	IGKJ2	IGKJ1	IGKV4-1
IGKV1-5 IGKV1-9 IGKV1-12	IGKV3-15	IGKV1-16	IGKV1-17
IGKV3-20	IGKV6-21	IGKV1-27	IGKV2-28	IGKV1-33
IGKV1D-39	IGKV1D-37	IGKV1D-33	IGKV2D-30	IGKV2D-28
IGKV3D-20	HOXD3	TWIST2	CCR10	FYTTD1	LRRC66	IGJ	PGRMC2	CTB
ZNF815	PCP2	MATN2	IGHV6-1 IGHV3-21	IGHV5-51	SPG11	UBE2Q2	TMC5
CTA	SIRT6	TPD52L2 IGLV5-48	IGLV5-45	IGLV1-40	IGLV2-14
IGLV3-9 IGLC6
GOBPID	Pvalue	OddsRatio	ExpCount	Count	Size	Term
GO:0006958	6.56174599161319e-11	130.781818181818	0.0726593315341499
6	50	complement activation, classical pathway
GO:0006956	2.7733150750002e-10	100.863157894737	0.0915507577330289
6	63	complement activation
GO:0002455	3.3654040772284e-10	97.4305084745763	0.0944571309943949
6	65	humoral immune response mediated by circulating immunoglobulin
GO:0072376	1.40520274699429e-09	75.5473684210526	0.119161303716006
6	82	protein activation cascade
GO:0016064	8.85392507192161e-09	54.5714285714286	0.161303716005813
6	111	immunoglobulin mediated immune response
GO:0019724	1.04006006824943e-08	53.0444444444444	0.165663275897862
6	114	B cell mediated immunity
GO:0006959	1.5651210711786e-08	49.3586206896552	0.177288768943326
6	122	humoral immune response
GO:0002449	1.02319466325083e-07	35.4509316770186	0.242682167324061
6	167	lymphocyte mediated immunity
GO:0002460	1.64953366296707e-07	32.5828571428571	0.263026780153623
6	181	adaptive immune response based on somatic recombination of immune receptors built from immunoglobulin superfamily domains
GO:0002250	2.80419069122154e-07	29.6625 0.287730952875234	6	198
adaptive immune response
GO:0002443	4.07855411949053e-07	27.7560975609756	0.306622379074112
6	211	leukocyte mediated immunity
GO:0002253	3.116569335006e-06	19.2996587030717	0.434502802574216
6	299	activation of immune response
GO:0050778	9.65416030917766e-06	15.722905027933 0.528959933568611	6
364	positive regulation of immune response
GO:0002252	1.34902972300728e-05	14.7894736842105	0.560930039443637
6	386	immune effector process
Tissue: Lung=>Artery_Tibial	Type:	cluster
SourceGene:	MALSU1(ENSG00000156928.4)
TargetGeneSet:	RERE	UBE4B	SPEN	ARID1A	WASF2	EYA3	PHACTR4 SRSF4	MECR

PUM1 BSDC1 PPCS ERI3 PCSK9 LRRC40 FPGT-TNNI3K RP4 ARNT
SETDB1 GATAD2B DENND4B CRTC2 ARHGEF11 PCP4L1 PRRC2C SLC41A1 RP11 ACP1
MORN2 MDH1 PRADC1 MTHFD2 SUCLG1 COX5B BCL2L11 AMMECR1L TANC1 MOB4
C2orf69 NELL2 KANSL1L SEC13 WDR48 ARF4 PDHB UBA3 FOXP1 KALRN
PPP1R2 MIR570 SLC30A9 COMMD8 CCNI SEC24B TRAM1L1 NUDT6 NDUFC1 SUB1
NIPBL C5orf28 ERCC8 C5orf44 MAN2A1 LSM11 UIMC1 PRL DAXX FANCE
BRPF3 PHF3 IRAK1BP1 IBTK UBE2J1 UFL1 CDK19 ECHDC1 RP3
ARID1B TCTE3 NDUFA4 TMEM106B ETV1 SEPT7 GRB10 CHCHD2 DTX2
PSMC2 CAPZA2 BPGM PPP1R3B RBPMS LSM1 CTD RAB2A MRPS35 DECR1
RBM12B COX6C SLC45A4 NDUFB6 RUSC2 AKNA CIZ1 IDI1 ERCC6 ADK
TCF7L2 FAM204A AKIP1 IMMP1L MARK2 NDUFS8 C11orf51 FAT3 C11orf70
TAGLN RNF214 MLL SC5DL SLC38A4 ZNF740 RARG PSG1 ATF7 SMARCC2
R3HDM2 CTDSP2 C12orf61 ARL1 NUP37 ATXN2 MRPL52 PSMA3 BCRP2
BAHD1 ZNF609 CLK3 WDR61 CIB1 IGF1R CREBBP BFAR KIAA0430
TMEM159 ATXN2L TBC1D10B GCSH ZC3H18 GLOD4 LSMD1 CTC1 PEMT
FBXL20 HSD17B1P1 ATP5G1 NDC80 AFG3L2 ZNF397 KIAA1328 IER3IP1
ATP8B1 UQCR11 TICAM1 C19orf70 INSR RAVER1 NDUFA13 KDELR1 CDS2
STAU1 SCAF4 HSF2BP ZNF280B TTC28-AS1 MTMR3 MORC2 GTPBP1 STS
TBC1D25 COX7B NDUFA1 BCORL1

GOBPID	Pvalue	OddsRatio	ExpCount	Count	Size	Term
GO:0045333	9.26263010788783e-11	12.3116319444444	1.37568334371324	14	142	cellular respiration
GO:0015980	9.71131285330417e-10	7.15854557830847	2.97418863746454	18	307	energy derivation by oxidation of organic compounds
GO:0022904	2.0746907009595e-09	14.2714090287278	0.930039443637119	11	96	respiratory electron transport chain
GO:0006091	5.4481847749318e-09	5.67933006535948	4.14642585288215	20	428	generation of precursor metabolites and energy
GO:0022900	7.07465700228225e-08	9.83601184849058	1.29818005674348	11	134	electron transport chain

Tissue: Lung=>Artery_Tibial Type: cluster
SourceGene: MED30(ENSG00000164758.3)
TargetGeneSet: MRPL20 RPL22 SDHB MECR ZCCHC17 ZBTB8OS ERI3 UQCRH RP4
PSMB4 DPM3 PMF1 ACBD6 NAV1 SNRPE ARV1 ACP1 FAM150B KIDINS220
RP11 TET3 COX5B UNC50 MZT2B TANC1 MOB4 SLC23A3 FARSB HDLBP LSM3
OXSR1 EXOSC7 USP4 SPCS1 CHMP2B STAG1 MRPS22 ATP11B ST6GAL1 RGS12
MRPL1 MRPS18C GRID2 NDUFC1 WWC2 SUB1 BRIX1 MOCS2 NDUFAF2 COX7C
RIOK2 MAN2A1 HINT1 UQCRQ SKP1 GFRA3 PDF RPS14 MRPL22 UBLCP1 MSX2
TMEM14B NOL7 HNRNPC SRPK1 BRPF3 MEA1 MRPL2 C6orf57 COX7A2 IMPG1
C6orf203 RP1 L3MBTL3 SF3B5 ARID1B PSMB1 NDUFA4 MALSU1 LSM5
YAE1D1 CHCHD2 STAG3L4 PMS2P1 PSMC2 BRAF SSBP1 CCDC25 GS1 VPS13B
COX6C ZNF706 NDUFB9 CPSF1 C8orf33 GLIS3 KIAA2026 PLIN2 NDUFB6
DCTN3 INVS FAM149B1 FAM204A TPP1 RPL27A C11orf31 ARL2
CCDC85B NDUFS8 LAMTOR1 CWC15 MAML2 C11orf1 TMPRSS5 ATP5L ERC1 ATF7
RPS26 RBMS2 R3HDM2 C12orf61 CHST11 ALKBH2 MED13L COX6A1 TRAC MED4
MBIP C14orf101 PSMA3 ERH ENTPD5 SLIRP PSMC1 C14orf2 CDCA4
CYFIP1 NDN SRP14 GALK2 RPLP1 MRPL28 C16orf42 NDE1 NDUFAB1
BCL7C MIR4519 NUDT21 SLC12A4 GCSH TRAPPC2L PSMB6 MINK1 CCL13 RFFL
PSMB3 SMARCE1 KRT223P COPZ2 MRPS23 ICT1 ATP5H NT5C TNRC6C KIAA1328
IER3IP1 ELAC1 ZNF532 CFD UQCR11 MRPL54 DPP9 C19orf70 NDUFA11
SWSAP1 NDUFB7 CPAMD8 NDUFA13 PDCD2L COX6B1 SIPA1L3 SNRPA ZNF428 GEMIN7
SNRPD2 RPS5 SNRPB SNORA51 MRPS26 SNRPB2 DYNLRB1 ROMO1 MYL9 ATP5E
ATP5J RANBP1 ZNF280B DEPDC5 TCF20 RP6 TIMM17B COX7B NDUFA1 VMA21

GOBPID	Pvalue	OddsRatio	ExpCount	Count	Size	Term
GO:0022904	4.80768187824279e-26	33.8452380952381	1.08947477683205	24	96	respiratory electron transport chain
GO:0022900	2.60589388894084e-22	22.094025974026	1.52072520932807	24	134	electron transport chain
GO:0045333	1.10821348634874e-21	20.5845036319613	1.61151477406408	24	142	cellular respiration
GO:0015980	8.88607998440733e-15	8.93221592938415	3.48404954674417	25	307	energy derivation by oxidation of organic compounds
GO:0006091	2.35233740270025e-12	6.50749873819309	4.85724171337624			

26	428	generation of precursor metabolites and energy
GO:0042773	2.35984507743052e-09	21.7726655348048	0.533388692824026
9	47	ATP synthesis coupled electron transport
GO:0042775	2.35984507743052e-09	21.7726655348048	0.533388692824026
9	47	mitochondrial ATP synthesis coupled electron transport
GO:0006120	5.3550209187755e-09	26.1153846153846	0.40855304131202
8	36	mitochondrial electron transport, NADH to ubiquinone
GO:0006119	1.65772557638898e-08	16.8718894009217	0.658224344336032
9	58	oxidative phosphorylation
GO:0000377	8.22136912979041e-08	6.92484210526316	2.31513390076811
14	204	RNA splicing, via transesterification reactions with bulged adenosine as nucleophile
GO:0000398	8.22136912979041e-08	6.92484210526316	2.31513390076811
14	204	nuclear mRNA splicing, via spliceosome
GO:0000375	1.11120510449526e-07	6.74488888888889	2.37187737872812
14	209	RNA splicing, via transesterification reactions
GO:0008380	8.29527736929109e-07	4.92296857117704	3.66562867621618
16	323	RNA splicing
GO:0051437	1.49815446664285e-06	11.3966346153846	0.81710608262404
8	72	positive regulation of ubiquitin-protein ligase activity involved in mitotic cell cycle
GO:0006200	2.05175450682395e-06	10.8840413318025	0.851152169400042
8	75	ATP catabolic process
GO:0051439	2.77048720394003e-06	10.4153846153846	0.885198256176043
8	78	regulation of ubiquitin-protein ligase activity involved in mitotic cell cycle
GO:0006521	3.5003022957087e-06	12.6954140127389	0.646875648744032
7	57	regulation of cellular amino acid metabolic process
GO:0051443	4.05273567851081e-06	9.84961884961885	0.930593038544045
8	82	positive regulation of ubiquitin-protein ligase activity
GO:0031145	4.44218161283476e-06	9.71760683760684	0.941941734136046
8	83	anaphase-promoting complex-dependent proteasomal ubiquitin-dependent protein catabolic process
GO:0051351	5.80616794550596e-06	9.34188034188034	0.975987820912048
8	86	positive regulation of ligase activity
GO:0006977	9.46532592415997e-06	10.7520241822304	0.749013909072037
7	66	DNA damage response, signal transduction by p53 class mediator resulting in cell cycle arrest
GO:0072413	9.46532592415997e-06	10.7520241822304	0.749013909072037
7	66	signal transduction involved in mitotic cell cycle checkpoint
GO:0072431	9.46532592415997e-06	10.7520241822304	0.749013909072037
7	66	signal transduction involved in mitotic cell cycle G1/S transition DNA damage checkpoint
GO:0072474	9.46532592415997e-06	10.7520241822304	0.749013909072037
7	66	signal transduction involved in mitotic cell cycle G1/S checkpoint
GO:0046034	9.56129334288728e-06	7.34879032258065	1.37319216663207
9	121	ATP metabolic process
GO:0031397	9.6156759661805e-06	8.67094017094017	1.04407999446405
8	92	negative regulation of protein ubiquitination
GO:0051436	1.04696797282814e-05	10.5720806794055	0.760362604664037
7	67	negative regulation of ubiquitin-protein ligase activity involved in mitotic cell cycle
GO:0072401	1.04696797282814e-05	10.5720806794055	0.760362604664037
7	67	signal transduction involved in DNA integrity checkpoint
GO:0072404	1.04696797282814e-05	10.5720806794055	0.760362604664037
7	67	signal transduction involved in G1/S transition checkpoint
GO:0072422	1.04696797282814e-05	10.5720806794055	0.760362604664037
7	67	signal transduction involved in DNA damage checkpoint
GO:0042776	1.05566600669119e-05	39.6611111111111	0.147533042696007
4	13	mitochondrial ATP synthesis coupled proton transport
GO:0072395	1.15607817328684e-05	10.3980369635585	0.771711300256038
7	68	signal transduction involved in cell cycle checkpoint
GO:0051438	1.31827508045277e-05	8.27447552447553	1.08947477683205

8	96	regulation of ubiquitin-protein ligase activity
GO:0033238	1.69123168055106e-05	9.75541401273885	0.81710608262404
7	72	regulation of cellular amine metabolic process
GO:0051352	1.69123168055106e-05	9.75541401273885	0.81710608262404
7	72	negative regulation of ligase activity
GO:0051444	1.69123168055106e-05	9.75541401273885	0.81710608262404
7	72	negative regulation of ubiquitin-protein ligase activity
GO:0051340	1.78042045340318e-05	7.91248606465998	1.13486955920006
8	100	regulation of ligase activity
GO:0031571	1.85293019263661e-05	9.60692916425401	0.82845477821604
7	73	mitotic cell cycle G1/S transition DNA damage checkpoint
Tissue: Artery_Tibial=>Lung	Type:	asymmetric
SourceGene:	RFC1(ENSG00000035928.10)
TargetGeneSet:	MTND2P28	RPL22	DNAJC11 RPS8	RP4	SRP9	UBC	COA5
BRK1	RPL15	SCN5A	RP11	ANXA2R	KIAA0825	HINT1	PRDM1	CHCHD2
SYPL1	ATP6V1C1	AGAP11	RPL27A	C11orf31	ANGPTL5 STAT2	HECTD1
DACT1	ZFYVE26 SIVA1	VPS18	C15orf61	HIC1	RPL27	RPL17	ZNF236	MIF
RPL36
GOBPID	Pvalue	OddsRatio	ExpCount	Count	Size	Term
GO:0006414	5.01697336459565e-13	63.9508928571429	0.21797799460245
9	105	translational elongation
GO:0006614	5.01697336459565e-13	63.9508928571429	0.21797799460245
9	105	SRP-dependent cotranslational protein targeting to membrane
GO:0006613	5.47525666702937e-13	63.2871870397644	0.220053975503425
9	106	cotranslational protein targeting to membrane
GO:0045047	5.47525666702937e-13	63.2871870397644	0.220053975503425
9	106	protein targeting to ER
GO:0072599	5.47525666702937e-13	63.2871870397644	0.220053975503425
9	106	establishment of protein localization to endoplasmic reticulum
GO:0070972	1.46736138181706e-12	56.2726081258191	0.244965746315134
9	118	protein localization to endoplasmic reticulum
GO:0006415	9.44003889127307e-12	62.8170865279299	0.18891426198879
8	91	translational termination
GO:0006612	1.56118916760135e-11	42.4910714285714	0.317625077849284
9	153	protein targeting to membrane
GO:0000184	8.38542969311404e-11	46.8796068796069	0.24704172721611
8	119	nuclear-transcribed mRNA catabolic process, nonsense-mediated decay
GO:0072594	2.06567116178528e-10	31.2659340659341	0.423500103799045
9	204	establishment of protein localization to organelle
GO:0019080	5.68241249197198e-10	36.3076923076923	0.313473116047332
8	151	viral genome expression
GO:0019083	5.68241249197198e-10	36.3076923076923	0.313473116047332
8	151	viral transcription
GO:0006413	5.98992822114789e-10	36.0530303030303	0.315549096948308
8	152	translational initiation
GO:0043624	6.31179356207962e-10	35.8018808777429	0.317625077849284
8	153	cellular protein complex disassembly
GO:0043241	8.15671725087671e-10	34.5963636363636	0.328004982354162
8	158	protein complex disassembly
GO:0000956	1.39316502393249e-09	32.2077922077922	0.350840772264895
8	169	nuclear-transcribed mRNA catabolic process
GO:0034623	1.75577584099916e-09	31.2267250821468	0.361220676769774
8	174	cellular macromolecular complex disassembly
GO:0006402	2.102229557835e-09	30.483422459893 0.369524600373677	8
178	mRNA catabolic process
GO:0032984	2.19757703533489e-09	30.3030303030303	0.371600581274652
8	179	macromolecular complex disassembly
GO:0006401	6.40579489682571e-09	26.2556529764652	0.425576084700021
8	205	RNA catabolic process
GO:0019058	1.63240654798781e-08	23.1520587036282	0.479551588125389
8	231	viral infectious cycle
GO:0022415	4.09700665663054e-08	20.4458874458874	0.539755034253685
8	260	viral reproductive process

GO:0071845	8.1089228630068e-08	18.6363636363636	0.589578575877102
8	284	cellular component disassembly at cellular level
GO:0022411	9.03188654516489e-08	18.3649350649351	0.597882499481005
8	288	cellular component disassembly
GO:0033365	3.09825975321922e-07	12.8913177339901	0.981938966161511
9	473	protein localization to organelle
GO:0006605	3.15399742993329e-07	12.8626728110599	0.984014947062487
9	474	protein targeting
GO:0016032	2.70433110559206e-06	11.4738354196593	0.936267386340046
8	451	viral reproduction
Tissue: Lung=>Artery_Tibial	Type:	asymmetric
SourceGene:	RP11-14I17.3(ENSG00000254362.1)
TargetGeneSet:	RPL22	RP11	AMY2A	FAHD2B	UBE2V1	RPL37A	RPL10AP6
COX7C	SEC24A	RPS10	TOMM7	GTF2I	SNHG6	FBXO43	EIF3H	PSCA	RPLP2	FAU
ATP5G2	ANKS1B	RPS29	RPL36A	MGAT2	RPLP1	C16orf55		RPL38	EIF3K
GOBPID	Pvalue	OddsRatio	ExpCount	Count	Size	Term
GO:0006413	3.92646057137708e-15	91.4724711907811	0.220884367863816
10	152	translational initiation
GO:0006415	3.43684867188714e-13	106.372567191844	0.132239983392153
8	91	translational termination
GO:0006414	1.11491359288827e-12	90.9310071371927	0.152584596221715
8	105	translational elongation
GO:0006614	1.11491359288827e-12	90.9310071371927	0.152584596221715
8	105	SRP-dependent cotranslational protein targeting to membrane
GO:0006613	1.2049572002447e-12	89.9968602825746	0.154037782852398
8	106	cotranslational protein targeting to membrane
GO:0045047	1.2049572002447e-12	89.9968602825746	0.154037782852398
8	106	protein targeting to ER
GO:0072599	1.2049572002447e-12	89.9968602825746	0.154037782852398
8	106	establishment of protein localization to endoplasmic reticulum
GO:0070972	2.89469742734692e-12	80.1118881118881	0.171476022420594
8	118	protein localization to endoplasmic reticulum
GO:0000184	3.10082702951292e-12	79.3846153846154	0.17292920905127
8	119	nuclear-transcribed mRNA catabolic process, nonsense-mediated decay
GO:0072594	4.73687877974069e-12	54.75	0.296450072659332	9	204
establishment of protein localization to organelle
GO:0019080	2.13907017560942e-11	61.4825174825175	0.219431181233133
8	151	viral genome expression
GO:0019083	2.13907017560942e-11	61.4825174825175	0.219431181233133
8	151	viral transcription
GO:0006612	2.37864207008614e-11	60.6259946949602	0.222337554494499
8	153	protein targeting to membrane
GO:0043624	2.37864207008614e-11	60.6259946949602	0.222337554494499
8	153	cellular protein complex disassembly
GO:0043241	3.08249190817365e-11	58.5846153846154	0.229603487647914
8	158	protein complex disassembly
GO:0000956	5.2972725883944e-11	54.539894887721	0.245588540585427	8
169	nuclear-transcribed mRNA catabolic process
GO:0034623	6.69467825914751e-11	52.8785912882298	0.252854473738842
8	174	cellular macromolecular complex disassembly
GO:0006402	8.03358941506834e-11	51.6199095022624	0.258667220261574
8	178	mRNA catabolic process
GO:0032984	8.40264151260377e-11	51.3144399460189	0.260120406892257
8	179	macromolecular complex disassembly
GO:0006401	2.48511036922702e-10	44.4607575165951	0.297903259290015
8	205	RNA catabolic process
GO:0019058	6.42550655272773e-10	39.205243187306	0.335686111687772	8
231	viral infectious cycle
GO:0022415	1.63902718783161e-09	34.6227106227106	0.377828523977579
8	260	viral reproductive process
GO:0071845	3.28786737084684e-09	31.5585284280936	0.412705003113971
8	284	cellular component disassembly at cellular level
GO:0022411	3.67030348448347e-09	31.0989010989011	0.418517749636703

8	288	cellular component disassembly
GO:0033365	8.28002994255104e-09	22.5743534482759	0.687357276313058
9	473	protein localization to organelle
GO:0006605	8.43381150091961e-09	22.5241935483871	0.688810462943741
9	474	protein targeting
GO:0016032	1.20424027420166e-07	19.4297621114777	0.655387170438032
8	451	viral reproduction
Tissue: Lung=>Artery_Tibial	Type:	asymmetric
SourceGene:	RP11-469J4.3(ENSG00000242578.1)
TargetGeneSet:	NOTCH2	MRPS21	KIRREL	RP11	NAV1	RPL35P1	KIDINS220
EXOC6B	MZT2A	RPL37A	TBC1D5	ZNF852	PBRM1	STAG1	TM4SF19-AS1	OCIAD1-AS1
CLOCK	WWC2	COX7C	CTD	RPS14	MRPL2	RPL39P3	PSMB1	C7orf50	TOMM7
MALSU1	HIP1	TAF6	SSBP1	SLC4A2	UQCRB	ZNF706	HK1	RPLP2	FAU
ARHGEF17	YAP1	PFDN5	ATF7	RPL18AP3	MED13L	PSME2P2	RPS29
C14orf2	MAPKBP1	NR2F2	COX4I1	RPL13	CCT6B	RPL38	WDR7	RSL24D1P8
C19orf53	RPL18A	NDUFA13	FXYD1	ETFB	IFNGR2	SLC38A5	ATRX	RGAG1	MT
GOBPID	Pvalue	OddsRatio	ExpCount	Count	Size	Term
GO:0006415	3.21955477760918e-10	37.3116248778899	0.283371392983185
8	91	translational termination
GO:0006414	1.02297167185496e-09	31.8952354416272	0.326966991903675
8	105	translational elongation
GO:0006614	1.02297167185496e-09	31.8952354416272	0.326966991903675
8	105	SRP-dependent cotranslational protein targeting to membrane
GO:0006613	1.10395183700959e-09	31.5675675675676	0.330080963255138
8	106	cotranslational protein targeting to membrane
GO:0045047	1.10395183700959e-09	31.5675675675676	0.330080963255138
8	106	protein targeting to ER
GO:0072599	1.10395183700959e-09	31.5675675675676	0.330080963255138
8	106	establishment of protein localization to endoplasmic reticulum
GO:0070972	2.60527694582146e-09	28.1002457002457	0.367448619472701
8	118	protein localization to endoplasmic reticulum
GO:0000184	2.78666132651158e-09	27.845142439737	0.370562590824164	8
119	nuclear-transcribed mRNA catabolic process, nonsense-mediated decay
GO:0072594	1.06541617984882e-08	18.2192307692308	0.635250155698568
9	204	establishment of protein localization to organelle
GO:0019080	1.83321836171405e-08	21.5656775656776	0.470209674070999
8	151	viral genome expression
GO:0019083	1.83321836171405e-08	21.5656775656776	0.470209674070999
8	151	viral transcription
GO:0006413	1.93063668823183e-08	21.4144144144144	0.473323645422462
8	152	translational initiation
GO:0006612	2.03249372007879e-08	21.2652376514445	0.476437616773926
8	153	protein targeting to membrane
GO:0043624	2.03249372007879e-08	21.2652376514445	0.476437616773926
8	153	cellular protein complex disassembly
GO:0043241	2.61444317343914e-08	20.5491891891892	0.492007473531244
8	158	protein complex disassembly
GO:0000956	4.42016157169263e-08	19.1304347826087	0.526261158397343
8	169	nuclear-transcribed mRNA catabolic process
GO:0034623	5.54487334207817e-08	18.5477043308369	0.541831015154661
8	174	cellular macromolecular complex disassembly
GO:0006402	6.6144265321733e-08	18.106200317965	0.554286900560515	8
178	mRNA catabolic process
GO:0032984	6.90801909820277e-08	17.9990516832622	0.557400871911978
8	179	macromolecular complex disassembly
GO:0071845	1.83687142817769e-07	12.8463636363636	0.884367863815653
9	284	cellular component disassembly at cellular level
GO:0006401	1.9656774814511e-07	15.5950061736864	0.638364127050031
8	205	RNA catabolic process
GO:0022411	2.06815135429612e-07	12.6586021505376	0.896823749221507
9	288	cellular component disassembly
GO:0019058	4.88983744313021e-07	13.7515452672403	0.719327382188084
8	231	viral infectious cycle

GO:0016032	9.5001796262223e-07	9.04761904761905	1.40440107951007
10	451	viral reproduction
GO:0022415	1.19467009618048e-06	12.1441441441441	0.809632551380527
8	260	viral reproductive process
GO:0033365	1.46260855257869e-06	8.60413452638075	1.47290844924227
10	473	protein localization to organelle
GO:0006605	1.49077133793387e-06	8.58497536945813	1.47602242059373
10	474	protein targeting
GO:0022904	1.15325666764779e-05	19.6634615384615	0.298941249740502
5	96	respiratory electron transport chain
Tissue: Artery_Tibial=>Lung	Type:	asymmetric
SourceGene:	RP11-96L14.7(ENSG00000236782.1)
TargetGeneSet:	RPL22	RPL11	ZNF593	RP11	RPS8	RPL5	GGCX	RPL31
RPL32	RPL14	RPL24	RPL34	RPL37	RPS14	EEF1A1	MRPS12	TRBV24-1	CTSB
RPS20	RPL30	EIF3H	RPS6	RPL35	RPS24	RPLP2	RPL27A	RPS13	FAU
RPS25	NACA	TPT1	RPS29	RPS3AP6	RPLP1	RPL13	RPL17	RPL19	RPL27
RPL36	RPS16	RPL13A	RPS11	RPS9	RPS21	RPL3	UQCRBP1	RPL10
GOBPID	Pvalue	OddsRatio	ExpCount	Count	Size	Term
GO:0006414	1.97525674743371e-72	935.086956521739	0.31970105875026
36	105	translational elongation
GO:0006415	3.43864902203077e-72	996.597222222222	0.277074250916892
35	91	translational termination
GO:0006614	1.61864780111005e-69	796.5	0.31970105875026	35	105
SRP-dependent cotranslational protein targeting to membrane
GO:0006613	2.41509908595394e-69	785.226917057903	0.322745830738357
35	106	cotranslational protein targeting to membrane
GO:0045047	2.41509908595394e-69	785.226917057903	0.322745830738357
35	106	protein targeting to ER
GO:0072599	2.41509908595394e-69	785.226917057903	0.322745830738357
35	106	establishment of protein localization to endoplasmic reticulum
GO:0070972	2.11197063344298e-67	671.137884872825	0.35928309459553
35	118	protein localization to endoplasmic reticulum
GO:0000184	2.99013222654803e-67	663.101851851852	0.362327866583627
35	119	nuclear-transcribed mRNA catabolic process, nonsense-mediated decay
GO:0006413	1.17751969382613e-65	554.39224137931	0.462805342190852	36
152	translational initiation
GO:0019080	4.43240956748714e-63	479.104406130268	0.459760570202754
35	151	viral genome expression
GO:0019083	4.43240956748714e-63	479.104406130268	0.459760570202754
35	151	viral transcription
GO:0006612	7.45719533960017e-63	470.918079096045	0.46585011417895
35	153	protein targeting to membrane
GO:0043624	7.45719533960017e-63	470.918079096045	0.46585011417895
35	153	cellular protein complex disassembly
GO:0043241	2.64851024598509e-62	451.616982836495	0.481073974119438
35	158	protein complex disassembly
GO:0000956	3.69334038571315e-61	414.224709784411	0.514566465988513
35	169	nuclear-transcribed mRNA catabolic process
GO:0034623	1.14860445592635e-60	399.184652278177	0.529790325929001
35	174	cellular macromolecular complex disassembly
GO:0006402	2.77381950384788e-60	387.909867909868	0.541969413881392
35	178	mRNA catabolic process
GO:0032984	3.44589867297839e-60	385.189043209877	0.54501418586949
35	179	macromolecular complex disassembly
GO:0072594	5.24187975638026e-58	327.633136094675	0.621133485571933
35	204	establishment of protein localization to organelle
GO:0006401	6.31720966569355e-58	325.683006535948	0.62417825756003
35	205	RNA catabolic process
GO:0019058	5.85559460952628e-56	281.964285714286	0.703342329250571
35	231	viral infectious cycle
GO:0022415	4.95867185056708e-54	245.120987654321	0.791640716905404
35	260	viral reproductive process
GO:0071845	1.32699761335688e-52	221.120035698349	0.864715244619749

35	284	cellular component disassembly at cellular level
GO:0022411	2.22919411718453e-52	217.562582345191	0.87689433257214
35	288	cellular component disassembly
GO:0016032	2.94340186076153e-45	130.791933760684	1.37319216663207
35	451	viral reproduction
GO:0033365	1.64019212160537e-44	124.027143581938	1.44017715037022
35	473	protein localization to organelle
GO:0006605	1.76985173622779e-44	123.73576309795 1.44322192235831	35
474	protein targeting
GO:0042274	4.56291058623043e-14	247.597051597052	0.0548058957857588
7	18	ribosomal small subunit biogenesis
GO:0042254	1.84125380903576e-11	30.1904358865849	0.453671026226559
10	149	ribosome biogenesis
GO:0006364	2.09431075921755e-11	38.7392481203008	0.316656286762162
9	104	rRNA processing
GO:0016072	3.49321364128278e-11	36.422630834512 0.334924918690748	9
110	rRNA metabolic process
GO:0022613	4.98619319428979e-11	22.107476635514 0.685073697321985	11
225	ribonucleoprotein complex biogenesis
GO:0071843	9.5425123723057e-11	20.7295321637427	0.727700505155353
11	239	cellular component biogenesis at cellular level
GO:0030490	2.59849612795674e-09	480.133333333333	0.021313403916684
4	7	maturation of SSU-rRNA
GO:0034470	7.25689103555196e-09	19.1389678384443	0.608954397619542
9	200	ncRNA processing
GO:0034660	1.54035450347411e-07	13.1655279503106	0.867760016607847
9	285	ncRNA metabolic process
GO:0000028	5.23384924917998e-07	351.317073170732	0.0182686319285863
3	6	ribosomal small subunit assembly
GO:0000462	5.23384924917998e-07	351.317073170732	0.0182686319285863
3	6	maturation of SSU-rRNA from tricistronic rRNA transcript (SSU-rRNA, 5.8S rRNA, LSU-rRNA)
GO:0042273	5.68413960043026e-06	117.056910569106	0.0365372638571725
3	12	ribosomal large subunit biogenesis
GO:0042255	1.43460475172112e-05	81.0168855534709	0.0487163518095634
3	16	ribosome assembly
Tissue: Artery_Tibial=>Lung	Type:	asymmetric
SourceGene:	SPOCD1(ENSG00000134668.7)
TargetGeneSet:	RPL22	AKR7L	C1orf210	RPS8	RPL5	RP11	RPS7
ITGB1BP1	PEX13	GGCX	FHL2	HOXD8	MFF	RPL15	LIMD1	PRKCD
THOC7	YEATS2	RPL34	FAM160A1	BTF3	AP3S1	THG1L	FNDC1	LANCL2
TRIM50	RUNDC3B	SLC25A13	RPL30	CHRAC1	TUSC1	ASPN	NR6A1	FAM35A
TRAF6	MSANTD4	TEX12	RPS25	SIAE	ZCRB1	RNASEH2B	RP4	RAP1AP
DICER1-AS1	DUOXA1	DUOX1	RPL4	RPS15A	RABEP2	RPL26	CDH2	RAB3D
ZNF737	ZNF428	SSC5D	PTPRA	SMARCB1	SEC14L4	OGT	RP6	KLHL4	PCDH19
BCORL1	IGSF1
GOBPID	Pvalue	OddsRatio	ExpCount	Count	Size	Term
GO:0006415	6.18283030552637e-15	53.2273648648649	0.302262819182064
11	91	translational termination
GO:0006414	3.14913099296321e-14	45.2556066705003	0.348764791363919
11	105	translational elongation
GO:0006614	3.14913099296321e-14	45.2556066705003	0.348764791363919
11	105	SRP-dependent cotranslational protein targeting to membrane
GO:0006613	3.50547736857288e-14	44.7761024182077	0.352086360805481
11	106	cotranslational protein targeting to membrane
GO:0045047	3.50547736857288e-14	44.7761024182077	0.352086360805481
11	106	protein targeting to ER
GO:0072599	3.50547736857288e-14	44.7761024182077	0.352086360805481
11	106	establishment of protein localization to endoplasmic reticulum
GO:0019080	5.48979982789341e-14	34.2062350119904	0.501556985675732
12	151	viral genome expression
GO:0019083	5.48979982789341e-14	34.2062350119904	0.501556985675732
12	151	viral transcription

GO:0072594 7.53061646116167e-14 27.637397157816 0.677600166078472 13
204 establishment of protein localization to organelle
GO:0070972 1.17282443718462e-13 39.7211417024501 0.391945194104214
11 118 protein localization to endoplasmic reticulum
GO:0000184 1.2892281125846e-13 39.3506006006006 0.395266763545775
11 119 nuclear-transcribed mRNA catabolic process, nonsense-mediated decay
GO:0034623 3.03097968682246e-13 29.3024691358025 0.577953082831638
12 174 cellular macromolecular complex disassembly
GO:0032984 4.25593784698778e-13 28.4151696606786 0.594560930039444
12 179 macromolecular complex disassembly
GO:0022415 1.69033341926163e-12 21.2872180451128 0.863608054805896
13 260 viral reproductive process
GO:0006413 1.95749546305293e-12 30.0713053479011 0.504878555117293
11 152 translational initiation
GO:0006612 2.10414550553252e-12 29.8574419489912 0.508200124558854
11 153 protein targeting to membrane
GO:0043624 2.10414550553252e-12 29.8574419489912 0.508200124558854
11 153 cellular protein complex disassembly
GO:0006401 2.14148223372021e-12 24.5423143350604 0.680921735520033
12 205 RNA catabolic process
GO:0043241 2.99731007256948e-12 28.8317705460563 0.52480797176666
11 158 protein complex disassembly
GO:0071845 5.17613432623823e-12 19.3691091196626 0.943325721403363
13 284 cellular component disassembly at cellular level
GO:0022411 6.17595894880333e-12 19.081974025974 0.956611999169608 13
288 cellular component disassembly
GO:0000956 6.2693993305163e-12 26.8037974683544 0.561345235623832
11 169 nuclear-transcribed mRNA catabolic process
GO:0019058 8.77082327890228e-12 21.5890410958904 0.767282541000623
12 231 viral infectious cycle
GO:0006402 1.1047880814047e-11 25.3432594270918 0.591239360597883
11 178 mRNA catabolic process
GO:0016032 1.59915347183911e-09 11.8424657534247 1.49802781814407
13 451 viral reproduction
GO:0033365 2.84643893940064e-09 11.2583229813665 1.57110234585842
13 473 protein localization to organelle
GO:0006605 2.91995796837528e-09 11.2330957545708 1.57442391529998
13 474 protein targeting
GO:0022613 8.64531046197235e-06 11.1093085701499 0.747353124351256
7 225 ribonucleoprotein complex biogenesis
GO:0071843 1.28092044106524e-05 10.4286164844407 0.793855096533112
7 239 cellular component biogenesis at cellular level
Tissue: Lung=>Artery_Tibial Type: cluster
SourceGene: SYCN(ENSG00000179751.5)
TargetGeneSet: HES2 CELA2A CELA3B CELA3A RP1 RP11 CLCA4 AMY2A
SCARNA2 BCL9 CRNN SPRR1B SPRR2D TKT PPP1R12B C4BPA PRKD3
MAP4K3 REG1B REG1A REG3A IGKV2-24 ITGA9 APPL1 CPB1 SCARNA7
C3orf70 CC2D2A NR3C2 LRBA SPINK1 PRL HIST1H1E MUC21 CLPS
SRPK1 PGC 7SK NHSL1 JAZF1 CPA2 CPA1 PRSS1 PRSS3P1 PRSS3P2 RHEB
KBTBD11 EFHA2 FGL1 VPS28 RGP1 ORM1 RP11-88G17 MRPL41 WDR85
MAPK8 PNLIP PNLIPRP2 PARVA CD248 FAM168A CREBZF TAF1D RECQL
KRT6A KRT4 MSRB3 PLA2G1B GJB2 HMGB1 EXOC5 DNAL1 C14orf45
TTLL5 HERC2P10 MAP1A RHCG C15orf38 CIB1 PCSK6 SPSB3 E4F1
GP2 CTRB2 CTRB1 TSR1 KRT33B KRT13 AOC3 GH1 CEP112 SCARNA17
SCARNA18 SERPINB3 FGF22 GPX4 TMIGD2 ALKBH7 ZNF426 SYCN KLK1
RP4 MAP1LC3A CTD C21orf119 PEX26 HIRA RBFOX2 APOBEC3C
TRABD FAM123B ZC4H2 TTC3P1 APOOL MID2 MT
GOBPID Pvalue OddsRatio ExpCount Count Size Term
GO:0007586 7.64920615419947e-10 19.2148148148148 0.641132101584665
10 109 digestion
GO:0044241 1.44827079758011e-06 64.4444444444444 0.0882291882914677
4 15 lipid digestion
Tissue: Lung=>Artery_Tibial Type: cluster

SourceGene: TMEM138(ENSG00000149483.6)
TargetGeneSet: MRPL20 SDHB UBR4 ATPIF1 MECR PSMB2 UQCRH MAGOH
ALG14 RP4 UFC1 SDHC SNRPE MORN2 COX5B CCDC93 PKP4 MOB4
MYEOV2 HDLBP LSM3 SLC6A6 KRBOX1 LZTFL1 SPCS1 CHMP2B RP11 MRPL47
MRPL1 MRPS18C LAMTOR3 ANAPC10 FRG1 PDCD6 FASTKD3 SUB1 MRPS30 COX7C SKP1
PDF DIAPH1 ATOX1 MRPL22 UBLCP1 NUDCD2 TMEM14B HLA-F-AS1 HNRNPC
SRPK1 SLC29A1 ECHDC1 MRPS12 TAB2 RP1 MLLT4 PSMB1 C7orf50 NUDT1 RPA3
NDUFA4 LSM5 EPDR1 YAE1D1 PPIA CHCHD2 SKP1P1 STAG3L4 KIAA1147
SSBP1 TAS2R5 NAT1 ZNF703 MRPS35 COX6C UBR5 UTP23 NDUFB9 TUSC1
NDUFB6 DCTN3 ISCA1 ATP6V1G1 CDC123 OPTN PLAC9 C10orf32
MRPL23 C11orf58 IMMP1L INCENP BAD MRPL11 NDUFS8 LAMTOR1 DCUN1D5
C11orf1 ATP5L CBL ARHGEF12 C12orf69 PRR13 R3HDM2 UBE2N
CCDC53 COX6A1 RFXAP MED4 COMMD6 A2LD1 C14orf126 MBIP PSMA3 ERH
RP3 SLIRP CDCA4 NDN SRP14 PHGR1 CHST14 GTF2A2 WDR61 MAN2A2 CTD
NDUFAB1 TMEM219 PLD2 FAM106A TMEM132E PSMB3 MRPS23 MAP2K6 ICT1
ELAC1 BSG UQCR11 MRPL54 C19orf70 NDUFA11 ALKBH7 OR7D2 PIN1 DNM2
SWSAP1 WDR83OS C19orf53 GIPC1 NDUFB7 NDUFA13 ZNF726 COX6B1 ZNF428
AP2S1 C19orf63 MCM8 SNRPB2 C20orf4 ZNF334 DIP2A MTMR3 PATZ1 TXN2
CXorf38 FUNDC1 GSPT2 PDZD11 PIN4 NGFRAP1 NXT2 NDUFA1 THOC2

GOBPID	Pvalue	OddsRatio	ExpCount	Count	Size	Term
GO:0022904	2.28502963868585e-20	30.288961038961	0.896823749221507	19	96	respiratory electron transport chain
GO:0022900	7.964367203689e-19	21.6659038901602	1.25181648328835	20	134	electron transport chain
GO:0045333	2.60949951688323e-18	20.2337847469708	1.32655179572348	20	142	cellular respiration
GO:0015980	8.78165891987501e-13	9.03662127346338	2.86796761469794	21	307	energy derivation by oxidation of organic compounds
GO:0006091	9.6882034216554e-12	7.05363756613757	3.99833921527922	23	428	generation of precursor metabolites and energy
GO:0042773	1.08965520046757e-08	23.0599636583889	0.439069960556363	8	47	ATP synthesis coupled electron transport
GO:0042775	1.08965520046757e-08	23.0599636583889	0.439069960556363	8	47	mitochondrial ATP synthesis coupled electron transport
GO:0006120	3.53695208346047e-08	26.9420797413793	0.336308905958065	7	36	mitochondrial electron transport, NADH to ubiquinone
GO:0006119	6.09371845813226e-08	17.9729133858268	0.541831015154661	8	58	oxidative phosphorylation
GO:0051443	9.3903136075027e-07	12.1234305171313	0.766036952460037	8	82	positive regulation of ubiquitin-protein ligase activity
GO:0051351	1.35489818955297e-06	11.4984857662023	0.8034046086776	8	86	positive regulation of ligase activity
GO:0051438	3.13130240229175e-06	10.184681460272	0.896823749221507	8	96	regulation of ubiquitin-protein ligase activity
GO:0051340	4.259098915901e-06	9.73913043478261	0.93419140543907	8	100	regulation of ligase activity

Tissue: Lung=>Heart_Left_Ventricle Type: asymmetric

SourceGene: BCL6(ENSG00000113916.13) B-cell lymphoma 6 protein isoform 1

TargetGeneSet: KLHL17 s CAPZB PLA2G2A ASAP3 INPP5B UTP11L KIAA1107
ECM1 BNIPL LYSMD1 SNX27 ILF2 FAM189B LMNA PMF1 CRABP2 RGS5 DPT
CEP350 TGFB2 C2orf44 AFF3 GCC2 GALNT5 FRZB COL3A1 COL5A2 SATB2
KANSL1L FN1 ARPC2 SLC23A3 GALNTL2 TGFBR2 TRANK1 ROBO1 CCDC80 KLF15
ABTB1 TNIK BCL6 11-Sep AFF1 UNC5C ARSJ PDE5A BBS12 PHF17
EDNRA LPCAT1 RAI14 PTGER4 SLC38A9 GUSBP9 KLHL35 ARSK PPP2CA PHF15 BRD8
SOX9 SPARC CNOT8 ADAM19 ADRA1B PDLIM7 DTNBP1 IER3 CCND3 FBXO9
COL12A1 KIAA1009 RP11 LFNG 7-Sep COL1A2 ECD RP13 PCOLCE
NAMPT IMPDH1 CALU TNPO3 DCTN6 UBXN2B MSC SDC2 YWHAZ EXT1
COL14A1 CER1 FREM1 DNAJB5 OGN ASPN ECM2 C9orf3 PTCH1 COL15A1
MSANTD3 CDK5RAP2 PSMB7 RXRA COL5A1 WAC ZNF37BP CSGALNACT2
KIAA0913 FRAT2 AP2A2 HTATIP2 PRG2 CARNS1 DYNC2H1 KBTBD3 ZBTB16
SCN4B HINFP TSPAN9 NTN4 EMP1 C12orf35 TENC1 PSG1 ZC3H10 AVIL
CTDSP2 LUM GLT8D2 CMKLR1 TMEM119 MPHOSPH8 AP1G2 LRFN5 MLH3 DIO2
ACTC1 ANKDD1A CILP ZWILCH FEM1B LRRC49 COMMD4 WDR61 MRPL28 TNFRSF12A
PPL MT1M MT1X HSD11B2 AARS ZNRF1 CENPN KIAA1609 ACSF3
WDR81 SPAG7 CTC1 C17orf39 GHDC STAT3 EZH1 SCRN2 COPZ2
STXBP4 MRC2 PRKAR1A GGA3 LLGL2 RECQL5 RHBDF2 MYL12B CXXC1 MOB3A
DAPK3 CDC37 WDR83 C19orf44 LSM4 ZNF14 PSMC4 ADCK4 TMEM91
ZNF233 SNRPB MAVS PHF20 SAMHD1 PLCG1 PKIG ELMO2 APCDD1L SYNJ1
COL6A2 TFIP11 TPST2 TTLL12 NRK TSC22D3 KIAA1210 PGRMC1 HMGB3
PNMA6C GAB3

GOBPID Pvalue OddsRatio ExpCount Count Size Term

GO:0030199	1.00E-10	33.4171875	0.385924849	9	33
collagen fibril organization					
GO:0030198	5.13E-08	7.958333333	1.882845478	13	161
extracellular matrix organization					
GO:0043062	5.53E-08	7.904362416	1.89454017	13	162
extracellular structure organization					

Tissue: Heart_Left_Ventricle=>Lung	Type:	asymmetric

SourceGene:	C15orf40(ENSG00000169609.8)	"Unknown protein (BLAST reveals a conserved domain of unknown function, DIF167 superfamily)"

TargetGeneSet:	RPL5	RP11	ZEB2	RPL32	CTB	RPL10A	MRPS12	PDGFA	AIMP2	ZDHHC4	RPS6	NFKBIA	EXD1	RPL4	RANBP10	R3HDM4	TIMM44	LILRB4	CTA	RPL3	RPL10

GOBPID	Pvalue	OddsRatio	ExpCount	Count	Size	Term
GO:0006415	9.34E-12	108.7045455	0.113348557	7	91	translational termination
GO:0006414	2.60E-11	93.08441558	0.130786797	7	105	translational elongation
GO:0006614	2.60E-11	93.08441558	0.130786797	7	105	SRP-dependent cotranslational protein targeting to membrane
GO:0006613	2.78E-11	92.13774105	0.132032385	7	106	cotranslational protein targeting to membrane
GO:0045047	2.78E-11	92.13774105	0.132032385	7	106	protein targeting to ER
GO:0072599	2.78E-11	92.13774105	0.132032385	7	106	establishment of protein localization to endoplasmic reticulum
GO:0072594	5.33E-11	58.11020408	0.254100062	8	204	establishment of protein localization to organelle
GO:0070972	5.97E-11	82.10810811	0.146979448	7	118	protein localization to endoplasmic reticulum
GO:0000184	6.34E-11	81.36931818	0.148225036	7	119	"nuclear-transcribed mRNA catabolic process, nonsense-mediated decay"
GO:0019080	3.42E-10	63.14583333	0.18808387	7	151	viral genome expression

GO:0019083	3.42E-10	63.14583333	0.18808387	7	151	
viral transcription						
GO:0006413	3.58E-10	62.70595611	0.189329458	7	152	
translational initiation						
GO:0006612	3.75E-10	62.27210461	0.190575047	7	153	
protein targeting to membrane						
GO:0043624	3.75E-10	62.27210461	0.190575047	7	153	
cellular protein complex disassembly						
GO:0043241	4.70E-10	60.18904275	0.196802989	7	158	
protein complex disassembly						
GO:0000956	7.54E-10	56.05892256	0.210504463	7	169	
nuclear-transcribed mRNA catabolic process						
GO:0034623	9.25E-10	54.3614589	0.216732406	7	174	
cellular macromolecular complex disassembly						
GO:0006402	1.09E-09	53.07496013	0.22171476	7	178	mRNA
catabolic process						
GO:0032984	1.13E-09	52.76268499	0.222960349	7	179	
macromolecular complex disassembly						
GO:0033365	1.50E-09	30.10560345	0.58916338	9	473	
protein localization to organelle						
GO:0006605	1.52E-09	30.03870968	0.590408968	9	474	
protein targeting						
GO:0006401	2.91E-09	45.75068871	0.255345651	7	205	RNA
catabolic process						
GO:0019058	6.67E-09	40.36647727	0.287730953	7	231	
viral infectious cycle						
GO:0022415	1.51E-08	35.66654689	0.323853021	7	260	
viral reproductive process						
GO:0071845	2.78E-08	32.52116836	0.353747146	7	284	
cellular component disassembly at cellular level						
GO:0022411	3.06E-08	32.04917502	0.3587295	7	288	
cellular component disassembly						

GO:0016032 6.50E-07 20.0497543 0.561760432 7 451
viral reproduction

Tissue: Lung=>Heart_Left_Ventricle Type: asymmetric
SourceGene: CTD-2224J9.8(ENSG00000213777.4)
TargetGeneSet: KIF17 AK2 MRPL37 USP24 ATP5F1 FH BOLA3 COX5B
UBE2F HIGD1A VPRBP CHMP2B RP11 TMEM14E RPL39L NDUFS6 NDFIP1 RP1 COQ3
SEC61G STAG3L4 PTK2B CYC1 NDUFB6 ATP5C1 SEMA4G GLRX3 MACROD1 SSSCA1 FIBP
NDUFS8 PTS RAPGEF3 SMARCC2 RPL6 SALL2 COX16 ANGEL1 CDC42BPB
C15orf29 TPM1 RPL4 SIN3A CTD NDUFB10 DCTPP1 TXNDC17 RPL17P43
PCGF2 NDUFV2 NDUFS7 UQCR11 MRPL4 NDUFB7 TIMM50 ZNF765 YWHAB NCOA3
FOXO4

GOBPID	Pvalue	OddsRatio	ExpCount	Count	Size	Term
GO:0022904	7.75203118275651e-16	49.8745644599303	0.352086360805481	12	96	respiratory electron transport chain
GO:0045333	2.74000707798801e-15	35.9490310077519	0.52079440869144	13	142	cellular respiration
GO:0022900	4.72993834892767e-14	34.2487005197921	0.491453878624317	12	134	electron transport chain
GO:0015980	3.24049693201275e-12	17.2810011376564	1.12594284132586	14	307	energy derivation by oxidation of organic compounds
GO:0006119	3.14111757666897e-11	51.0151111111111	0.212718842986645	8	58	oxidative phosphorylation
GO:0006120	4.55106132982168e-11	75.3995502248876	0.132032385302055	7	36	mitochondrial electron transport, NADH to ubiquinone
GO:0006091	2.71045007873877e-10	12.1253561253561	1.5697183585911	14	428	generation of precursor metabolites and energy
GO:0042773	3.32506633580914e-10	54.6228260869565	0.17237561414435	7	47	ATP synthesis coupled electron transport
GO:0042775	3.32506633580914e-10	54.6228260869565	0.17237561414435	7	47	mitochondrial ATP synthesis coupled electron transport

Tissue: Heart_Left_Ventricle=>Lung Type: asymmetric

SourceGene: GCC2(ENSG00000135968.13) GRIP and coiled-coil domain containing 2

TargetGeneSet: 7SK BRD8 FCER1A CEP350 TSEN15 PLB1 EHD3 IL1R2
EAF1-AS1 BCL6 RP11 SULT1B1 HSP90AB3P EDNRA TLR2 RNF175 NAIP
PCDHA3 SH3PXD2B DRD1 C6orf1 FKBP5 ARMC12 RAB23 WASF1 GUCY2C
FBXO32 TLR4 SUPV3L1 CHST3 ABCC2 HRAS TMEM223 ZBTB16 NNMT RP1
IRAK3 UBE3A ST20 MT1M MT1JP MT1A MYO1C CANT1 PSTPIP2 C19orf35
ITPKC GRIK5 FPR2 VN1R1 SEC14L2 RP3 WNK3 PCDH11X TSC22D3

GOBPID	Pvalue	OddsRatio	ExpCount	Count	Size	Term
GO:0061082	9.06E-08	134.2377622	0.044633589	4	15	

myeloid leukocyte cytokine production

GO:0010934	2.90E-06	154.2964286	0.029755726	3	10
macrophage cytokine production

GO:0010935	2.90E-06	154.2964286	0.029755726	3	10
regulation of macrophage cytokine production

GO:0002718	4.66E-06	43.36048265	0.11307176	4	38
regulation of cytokine production involved in immune response

GO:0002367	7.71E-06	37.78829717	0.127949623	4	43
cytokine production involved in immune response

Tissue: Lung=>Heart_Left_Ventricle	Type:	asymmetric

SourceGene:	GCKR(ENSG00000084734.4) "glucokinase (hexokinase 4) regulator (is expressed mainly in liver, but also in lung, funtion unknown)"

TargetGeneSet:		CAPZB	PLA2G2A	CDA	ASAP3	FGR	COL16A1	CYR61	ST7L	
PSMB4	SLC39A1	EFNA4	EFNA1	RP11	FAM189B	LMNA	CRABP2	PTGS2	CALM2	
ANTXR1	STEAP3	TNFAIP6	PSMD14	LIPA	FRZB	COL3A1	COL5A2	KANSL1L	FN1	
PSMD1	COL6A3	MTMR14	TRANK1	PTH1R	COL8A1	ABI3BP	CCDC80	GAP43	KLF15	SKIL
SPON2	KIT	11-Sep	UNC5C	PDE5A	LPCAT1	SUB1	RAI14	PTGER4	CTD	
KLHL35	LHFPL2	PPP2CA	PHF15	MATR3	SPARC	CTB	HK3	DTNBP1	RBM24	
MOCS1	MTO1	USP45	TCF21	LFNG	CYTH3	7-Sep	SFRP4	YKT6	COL1A2	ECD
PDAP1	SLC12A9	ZNHIT1	NAMPT	DYNLL1	TNFRSF10C		EBF2	TRPA1	SDC2	
YWHAZ	COL14A1	FREM1	OGN	OMD	ASPN	ECM2	PTCH1	PSMB7	COL5A1	
ITIH5	FAM107B	KAZALD1	ADAM12	NRXN2	RIN1	TEAD4	NTN4	EMP1	MGP	
STK38L	C12orf35		TENC1	LUM	GLT8D2	CMKLR1	TMEM119	AP1G2	LRFN5	
COX16	DIO2	CILP	FEM1B	ISLR	SCAMP2	WDR61	FAM174B	MRPL28	PPL	CDR2
MMP2	PDP2	SLC9A5	WDR81	RASL10B	CCR7	STAT3	NBR2	COPZ2	COL1A1	
MTMR4	MRC2	RHBDF2	PRCD	C17orf62		GALNT1	DSEL	MOB3A	KRI1	LSM4
U2AF1L4	PSMC4	CLEC11A	ZNF766	ZNF600	ZNF845	ZNF525	CHMP2A	MZF1	ZNF341	
PTPRT	HSPA13	CBS	COL6A1	COL6A2	TPST2	MN1	MFNG	ATP6AP2	TCEAL4	NRK
CAPN6	PLS3	PGRMC1								

GOBPID	Pvalue	OddsRatio	ExpCount	Count	Size	Term
GO:0070208	1.32E-10	163.8549618	0.094803128	6	10	protein heterotrimerization
GO:0030198	3.73E-10	10.96941541	1.526330358	14	161	extracellular matrix organization

GO:0043062	4.05E-10	10.89452868	1.535810671	14	162
extracellular structure organization					
GO:0030199	2.05E-08	29.59053254	0.312850322	7	33
collagen fibril organization					
GO:0071230	3.18E-08	27.47307692	0.331810947	7	35
cellular response to amino acid stimulus					

GO:0051291	5.15E-08	18.43152455	0.530897516	8	56
protein heterooligomerization					
GO:0071229	1.01E-07	22.61538462	0.388692824	7	41
cellular response to acid					
GO:0071418	4.20E-07	17.8706619	0.474015639	7	50
cellular response to amine stimulus					
GO:0031589	4.84E-07	7.136336842	1.915023182	12	202
cell-substrate adhesion					

GO:0071417	5.53E-07	17.07401709	0.492976265	7	52

cellular response to organic nitrogen

GO:0070206	5.84E-07	24.23579304	0.312850322	6	33

protein trimerization

GO:0001101	1.38E-06	11.46642505	0.805826586	8	85

response to acid

GO:0001503	1.46E-06	5.826628841	2.5217632	13	266

ossification

GO:0001501	1.57E-06	4.993089973	3.403432288	15	359
skeletal system development					
GO:0043200	2.60E-06	13.23501326	0.616220331	7	65
response to amino acid stimulus					
GO:0010811	6.51E-06	15.20078111	0.464535326	6	49
positive regulation of cell-substrate adhesion					
GO:0001957	1.63E-05	106.7985075	0.056881877	3	6

intramembranous ossification

GO:0036072 1.63E-05 106.7985075 0.056881877 3 6
direct ossification

Tissue: Lung=>Heart_Left_Ventricle Type: asymmetric

SourceGene: HS3ST6(ENSG00000162040.5) heparan sulfate (glucosamine) 3-O-sulfotransferase 6

TargetGeneSet: CAPZB ZFYVE9 PSMA5 RPRD2 PSMB4 PMF1 SLC30A10 MIA3
FAM110C NBAS COMMD1 PSMD14 ARPC2 SNED1 GALNTL2 SLC12A8 PIK3CB PHF17
PHF15 SPARC DTNBP1 KIAA1009 ECD MEPCE ZNHIT1 ATXN7L1 ADCK2
MIR939 NFX1 ECM2 MSANTD3 ZFP37 CDK5RAP2 PSMB7 ST6GALNAC6
COL5A1 WAC TM9SF3 SLC43A1 DYNC2H1 NPAT SCN4B NTN4 MPHOSPH8
PSMB5 PSME2 C14orf149 CILP MRPL28 PPL HSD11B2 DEF8 EZH1
RPL27 LLGL2 SRSF2 MYL12B CDC37 PDCD5 PSMC4 ZNF615 ZNF766 ZNF845
ZNF525 ZNF329 CSRP2BP PHF20 EIF4ENIF1 RIPPLY1 KIAA1210 HMGB3

GOBPID Pvalue OddsRatio ExpCount Count Size Term

GO:0006521	2.95E-09	38.61307692	0.232717459	7	57
regulation of cellular amino acid metabolic process

GO:0006977	8.46E-09	32.70241199	0.269462321	7	66	"DNA damage response, signal transduction by p53 class mediator resulting in cell cycle arrest"

GO:0072413	8.46E-09	32.70241199	0.269462321	7	66
signal transduction involved in mitotic cell cycle checkpoint

GO:0072431	8.46E-09	32.70241199	0.269462321	7	66
signal transduction involved in mitotic cell cycle G1/S transition DNA damage checkpoint

GO:0072474	8.46E-09	32.70241199	0.269462321	7	66
signal transduction involved in mitotic cell cycle G1/S checkpoint

GO:0051436	9.42E-09	32.15512821	0.273545083	7	67
negative regulation of ubiquitin-protein ligase activity involved in mitotic cell cycle

GO:0072401	9.42E-09	32.15512821	0.273545083	7	67
signal transduction involved in DNA integrity checkpoint

GO:0072404 9.42E-09 32.15512821 0.273545083 7 67
signal transduction involved in G1/S transition checkpoint

GO:0072422 9.42E-09 32.15512821 0.273545083 7 67
signal transduction involved in DNA damage checkpoint

GO:0072395 1.05E-08 31.62578815 0.277627846 7 68
signal transduction involved in cell cycle checkpoint

GO:0033238 1.57E-08 29.67130178 0.293958896 7 72
regulation of cellular amine metabolic process

GO:0051352 1.57E-08 29.67130178 0.293958896 7 72
negative regulation of ligase activity

GO:0051437 1.57E-08 29.67130178 0.293958896 7 72
positive regulation of ubiquitin-protein ligase activity involved in mitotic cell cycle

GO:0051444 1.57E-08 29.67130178 0.293958896 7 72
negative regulation of ubiquitin-protein ligase activity

GO:0031571 1.73E-08 29.21969697 0.298041658 7 73
mitotic cell cycle G1/S transition DNA damage checkpoint

GO:0002479 2.10E-08 28.35633484 0.306207183 7 75
"antigen processing and presentation of exogenous peptide antigen via MHC class I, TAP-dependent"

GO:0051439 2.76E-08 27.15249187 0.31845547 7 78
regulation of ubiquitin-protein ligase activity involved in mitotic cell cycle

GO:0000216 3.02E-08 26.77350427 0.322538233 7 79 M/G1
transition of mitotic cell cycle

GO:0042590 3.02E-08 26.77350427 0.322538233 7 79
antigen processing and presentation of exogenous peptide antigen via MHC class I

GO:0071158 3.30E-08 26.40489989 0.326620995 7 80
positive regulation of cell cycle arrest

GO:0031575 3.93E-08 25.69717949 0.33478652 7 82
mitotic cell cycle G1/S transition checkpoint

GO:0051443 3.93E-08 25.69717949 0.33478652 7 82
positive regulation of ubiquitin-protein ligase activity

GO:0002478 4.27E-08 25.35728745 0.338869282 7 83
antigen processing and presentation of exogenous peptide antigen

GO:0031145 4.27E-08 25.35728745 0.338869282 7 83
anaphase-promoting complex-dependent proteasomal ubiquitin-dependent protein catabolic process

GO:0031398 4.34E-08 18.81430219 0.51851083 8 127
positive regulation of protein ubiquitination

GO:0019884 5.05E-08 24.70364892 0.347034807 7 85
antigen processing and presentation of exogenous antigen

GO:0051351 5.48E-08 24.38924051 0.35111757 7 86
positive regulation of ligase activity

GO:0071779 5.48E-08 24.38924051 0.35111757 7 86 G1/S transition checkpoint

GO:0031397 8.76E-08 22.6581448 0.375614144 7 92
negative regulation of protein ubiquitination

GO:0051438 1.18E-07 21.63375108 0.391945194 7 96
regulation of ubiquitin-protein ligase activity

GO:0002474 1.46E-07 20.92391304 0.404193481 7 99
antigen processing and presentation of peptide antigen via MHC class I

GO:2000045 1.46E-07 20.92391304 0.404193481 7 99
regulation of G1/S transition of mitotic cell cycle

GO:0051340 1.56E-07 20.69747725 0.408276244 7 100
regulation of ligase activity

GO:0043161 1.57E-07 12.7728 0.85329735 9 209 proteasomal
ubiquitin-dependent protein catabolic process

GO:0010498 2.07E-07 12.33478261 0.881876687 9 216
proteasomal protein catabolic process

GO:0048002	2.65E-07	19.04741051	0.440938343	7	108	antigen processing and presentation of peptide antigen
GO:0031396	3.78E-07	13.95294118	0.68590409	8	168	regulation of protein ubiquitination
GO:0030330	4.59E-07	17.47797203	0.477683205	7	117	"DNA damage response, signal transduction by p53 class mediator"
GO:0000075	5.41E-07	10.93828326	0.98802851	9	242	cell cycle checkpoint
GO:0072331	6.44E-07	16.56697613	0.50217978	7	123	signal transduction by p53 class mediator
GO:0000082	8.20E-07	12.52610707	0.759393814	8	186	G1/S transition of mitotic cell cycle
GO:0000077	9.37E-07	15.61647905	0.530759117	7	130	DNA damage checkpoint

GO:0071156	1.12E-06	9.979058824	1.077849284	9	264	
regulation of cell cycle arrest						
GO:0000084	1.15E-06	15.12038159	0.547090167	7	134	S
phase of mitotic cell cycle						
GO:0031570	1.27E-06	14.88387001	0.555255692	7	136	DNA
integrity checkpoint						
GO:0007093	1.47E-06	14.54254079	0.567503979	7	139	
mitotic cell cycle checkpoint						
GO:0042770	1.54E-06	14.43218623	0.571586741	7	140	
signal transduction in response to DNA damage						
GO:0051320	1.78E-06	14.11085973	0.583835029	7	143	S
phase						
GO:2000602	2.14E-06	13.70384615	0.600166078	7	147	
regulation of interphase of mitotic cell cycle						

GO:0090068	2.56E-06	13.31944444	0.616497128	7	151
positive regulation of cell cycle process					
GO:0019882	2.68E-06	13.22665782	0.620579891	7	152
antigen processing and presentation					
GO:0010565	2.92E-06	13.04487179	0.628745416	7	154
regulation of cellular ketone metabolic process					
GO:0000209	3.92E-06	12.4458042	0.657324753	7	161
protein polyubiquitination					
GO:0044106	9.11E-06	10.87325175	0.747145526	7	183
cellular amine metabolic process					
GO:0006511	1.53E-05	7.117352113	1.486125528	9	364
ubiquitin-dependent protein catabolic process					
GO:0009308	1.73E-05	9.800690335	0.824718013	7	202
amine metabolic process					

GO:0019941 1.78E-05 6.976243094 1.514704865 9 371
modification-dependent protein catabolic process

GO:0043632 1.89E-05 6.917424658 1.526953152 9 374
modification-dependent macromolecule catabolic process

Tissue: Heart_Left_Ventricle=>Lung Type: asymmetric

SourceGene: KIAA1797(ENSG00000188352.7) FOCAD focadhesin

TargetGeneSet: RPL11 RP4 RP11 RPL5 U1 UBC RPL31 EEF1A1P12
CYBRD1 TMEFF2 RPL37A RPL32 RPL15 RPL24 RPL35A RPL34 CTD RPL37
EEF1A1P19 RPS14 RPL24P4 MRPS12 TRBV6-1 RPS20 RPL30 RPS6 STRA13 7SK
RPL13AP5 RPS13 FAU C12orf57 AMHR2 NACA RPL14P1 RPL3P4 U4
RPLP1 RPL26 MFAP4 RPL19 RPL27 RPS15 RPL18A RPS16 FBL ZNF574
RPL18 RPL13A RPS9 KIR3DX1 RPL28 RPS5 INSM1 RPS21 H2BFS RPL3
SLC25A6 EEF1B2P3 RPS23P8 RP3 RPL10

GOBPID Pvalue OddsRatio ExpCount Count Size Term

GO:0006415 1.75E-62 555.7772397 0.289668535 32 91
translational termination

GO:0006414	4.28E-60	448.7514677	0.334232925	32	105
translational elongation					
GO:0006614	4.28E-60	448.7514677	0.334232925	32	105
SRP-dependent cotranslational protein targeting to membrane					
GO:0006613	6.13E-60	442.6563707	0.337416096	32	106
cotranslational protein targeting to membrane					
GO:0045047	6.13E-60	442.6563707	0.337416096	32	106
protein targeting to ER					
GO:0072599	6.13E-60	442.6563707	0.337416096	32	106
establishment of protein localization to endoplasmic reticulum					
GO:0070972	3.38E-58	380.5714286	0.375614144	32	118
protein localization to endoplasmic reticulum					
GO:0000184	4.62E-58	376.1707718	0.378797315	32	119
"nuclear-transcribed mRNA catabolic process, nonsense-mediated decay"					
GO:0019080	2.62E-54	274.4009604	0.480658778	32	151
viral genome expression					

GO:0019083	2.62E-54	274.4009604	0.480658778	32	151
viral transcription					
GO:0006413	3.32E-54	272.0952381	0.483841949	32	152
translational initiation					
GO:0006612	4.19E-54	269.8276269	0.487025119	32	153
protein targeting to membrane					
GO:0043624	4.19E-54	269.8276269	0.487025119	32	153
cellular protein complex disassembly					
GO:0043241	1.32E-53	259.0294785	0.502940973	32	158
protein complex disassembly					
GO:0000956	1.42E-52	238.0479666	0.537955851	32	169
nuclear-transcribed mRNA catabolic process					
GO:0034623	3.95E-52	229.5855131	0.553871704	32	174
cellular macromolecular complex disassembly					
GO:0072594	5.13E-52	211.3009447	0.649366826	33	204
establishment of protein localization to organelle					

GO:0006402	8.77E-52	223.2328767	0.566604387	32	178	mRNA catabolic process
GO:0032984	1.07E-51	221.6987366	0.569787558	32	179	macromolecular complex disassembly
GO:0019058	4.24E-50	182.1410256	0.735312435	33	231	viral infectious cycle
GO:0006401	1.19E-49	188.0363336	0.652549997	32	205	RNA catabolic process
GO:0022415	2.71E-48	158.547611	0.827624386	33	260	viral reproductive process
GO:0071845	7.80E-45	128.3718821	0.904020483	32	284	cellular component disassembly at cellular level
GO:0022411	1.25E-44	126.3303571	0.916753166	32	288	cellular component disassembly
GO:0016032	4.49E-40	84.94129555	1.435609992	33	451	viral reproduction

GO:0033365	2.24E-39	80.56730769	1.505639748	33	473	protein localization to organelle
GO:0006605	2.41E-39	80.37885924	1.508822919	33	474	protein targeting
GO:0006364	7.88E-13	42.29018913	0.331049754	10	104	rRNA processing
GO:0016072	1.40E-12	39.73611111	0.350148779	10	110	rRNA metabolic process
GO:0042254	2.97E-11	28.50919265	0.474292437	10	149	ribosome biogenesis
GO:0022613	8.44E-11	20.84125501	0.716213411	11	225	ribonucleoprotein complex biogenesis
GO:0071843	1.61E-10	19.54223058	0.760777801	11	239	cellular component biogenesis at cellular level
GO:0034470	5.44E-10	20.78216374	0.636634143	10	200	ncRNA processing

GO:0042274 2.17E-09 135.0093809 0.057297073 5 18
ribosomal small subunit biogenesis

GO:0034660 1.66E-08 14.27272727 0.907203654 10 285
ncRNA metabolic process

GO:0042273 4.37E-08 171.3928571 0.038198049 4 12
ribosomal large subunit biogenesis

Tissue: Heart_Left_Ventricle=>Lung Type: cluster
SourceGene: RP11-110I1.11(ENSG00000254428.1)
TargetGeneSet: FAM87B TTC34 H6PD PRDX3P2 LCK CDC20 ATG4C LEPROT
LRRC39 NRAS GAPDHP32 JTB PYGO2 IQGAP3 RP11 B4GALT3 POU2F1
C1orf106 SH3YL1 DHX57 STAMBP PCGF1 IMMT TMEM127 CKAP2L TANC1
C2orf77 CCDC150 ALS2CR8 HJURP MSL3P1 CRELD1 ATG7 GLYCTK KCTD6 PDZRN3
KIAA1524 POLQ CCNL1 SERP1 GFM1 CTA ABCA11P PCDH7 HOPX
C4orf21 MAD2L1 OTUD4 TRIP13 NUP155 CENPH ARSB KIF20A CTB PCDHB6 RARS
TRIM27 TBCC KLC4 SLC35B2 FAM184A SCAF8 CLDN20 TRA2A STK17A NPC1L1
LANCL2 STX1A RBM48 SLC25A13 RINT1 MCM4 IMPAD1 MLLT3 PIP5K1B
C9orf85 SMC2 FKTN FBXW2 FPGS C9orf106 MAN1B1 RP1 CDC123
NSUN6 ZNF33A ZWINT CDK1 PBLD PLA2G12B KIF11 CEP55 ANO9 CTD
C11orf16 PARVA PRMT3 ALKBH3 ARHGAP1 F2 DDB1 FEN1 GANAB
ZDHHC24 CDK2AP2 KDM4D CHEK1 DSTNP2 FGFR1OP2 MED21 REP15 IPO8
TROAP ESPL1 AVIL NUP107 NEDD1 C12orf76 DYNLL1 RPL22P19 SKA3
FANCB TUBGCP3 SUPT16H RNF31 FANCM POLE2 BUB1B CASC5 RTF1 CCNB2
KIF23 FANCI C15orf42 PRC1 PLA2G10 FAM57B ZNF629 CIAPIN1 DHX38
CFDP1 TMEM170A SPG7 TMEM220 ELAC2 B9D1 ATAD5 CCT6B GRB7
GSDMB TOP2A RAB5C G6PC3 C17orf53 EFTUD2 KIF18B TUBD1 BRIP1
GRIN2C CHAF1A PCP2 SPC24 TNPO2 DCAF15 ZNF493 WDR62 PPP1R13L IRF3
CTU1 TMEM190 ZNF444 ZNF274 ZSCAN22 GINS1 TPX2 MYH7B DHX35 RNF114
FAM207CP UFD1L DEPDC5 PRR5 CPT1B MBTPS2 MED14 SUV39H1 KIF4A ACRC
PGAM4 TMSB15B CD99L2 CLIC2

GOBPID	Pvalue	OddsRatio	ExpCount	Count	Size	Term
GO:0000087	3.41459242023721e-11	6.88313609467456	3.83641270500311	22	360	M phase of mitotic cell cycle
GO:0051301	7.54876927158181e-11	5.9823148813803	4.81682928517058	24	452	cell division
GO:0000280	1.39624192754594e-10	6.70356742921133	3.72984568541969	21	350	nuclear division
GO:0007067	1.39624192754594e-10	6.70356742921133	3.72984568541969	21	350	mitosis
GO:0048285	3.87675594808876e-10	6.31036042829136	3.94297972458653	21	370	organelle fission
GO:0007017	6.84997114978721e-09	5.57160577808011	4.1880838696284	20		

393	microtubule-based process
GO:0000226	1.8416091459656e-07	5.96626635699315	2.86665282679399
15	269	microtubule cytoskeleton organization
GO:0007059	2.9322238130853e-07	8.44841979725701	1.49193827416788
11	140	chromosome segregation
GO:0000075	3.08459887073844e-07	6.17061403508772	2.57892187391876
14	242	cell cycle checkpoint
GO:0010564	4.3275899288451e-07	4.64605614973262	4.41187461075358
18	414	regulation of cell cycle process
GO:0006261	6.290165268519e-07	10.5022167487685	0.991073282125804
9	93	DNA-dependent DNA replication
GO:0071156	8.81285201837645e-07	5.6188	2.81336931700228	14	264
regulation of cell cycle arrest
GO:0006260	1.054382882607e-06	5.52874015748031	2.85599612483565
14	268	DNA replication
GO:0051329	2.88979446412345e-06	4.45051303549327	4.03889004221161
16	379	interphase of mitotic cell cycle
GO:0006281	3.30705403402202e-06	4.40074240808751	4.08151685004498
16	383	DNA repair
GO:0000236	3.33212173069237e-06	10.1191958726205	0.905819666459069
8	85	mitotic prometaphase
GO:0051325	3.77768379451081e-06	4.35204500175788	4.12414365787835
16	387	interphase
GO:0007050	9.4456641151353e-06	4.25039629313498	3.93232302262819
15	369	cell cycle arrest
Tissue: Heart_Left_Ventricle=>Lung	Type:	asymmetric
SourceGene:	RP11-115C9.2(ENSG00000223582.1)
TargetGeneSet:	ALPL	STMN1	SDC3	CLSPN	RP11	OLFML3	PRCC	RNASEL
CSRP1	TNFSF13B		TLR5	CENPO	HAT1	TRAIP	NIT2	CCNL1	BCHE
SLIT2	CTD	SGCD	KIF13A	TCF19	RDBP	TBC1D22B		SAP25	DENND2A
ADAMDEC1		GGH	ZNF704	SMC2	CERCAM	MCM10	PARVA	FEN1	ME3
H2AFJ	DCTN2	FANCB	CAB39L	TRAV13-2		VRK1	KIF23	CHTF18	MPDU1
DUSP3	BRIP1	CDH2	CHAF1A	ZNF844	TPM4	CCNE1	GINS1	E2F1	LMF2
NCAPH2	ACOT9	SUV39H1	MAGED2	RP3	FHL1	HTATSF1
GOBPID	Pvalue	OddsRatio	ExpCount	Count	Size	Term
GO:0006260	4.92726359204836e-07	11.1662864162864	0.982907757248633
9	268	DNA replication
GO:0051329	9.75469589256103e-07	8.84162097434928	1.39000761192997
10	379	interphase of mitotic cell cycle
GO:0051325	1.17885522139013e-06	8.64906544938622	1.41934814199709
10	387	interphase
GO:0006323	1.33198077694893e-06	14.854675402025	0.561137637533735	7
153	DNA packaging
GO:0071103	5.43013306545825e-06	11.8862876254181	0.69317002283579
7	189	DNA conformation change
Tissue: Heart_Left_Ventricle=>Lung	Type:	asymmetric
SourceGene:	RP11-322F10.2(ENSG00000225782.1)
TargetGeneSet:	RP4	PYGO2	KRTCAP3	EPCAM	UBC	XYLB	RPL24	ALCAM	CD47
RPL35A	MRPS36	MARVELD2		RPL39P3	RP11	PPP1R35	RPS6	C9orf40	FOLR1
CARD16	SIRT4	MSI1	ENTPD5	RPS21	CLIC2
GOBPID	Pvalue	OddsRatio	ExpCount	Count	Size	Term
GO:0006415	1.66530354802416e-07	52.1220930232558	0.132239983392153
5	91	translational termination
GO:0006414	3.41324614487545e-07	44.78125	0.152584596221715	5
105	translational elongation
GO:0006614	3.41324614487545e-07	44.78125	0.152584596221715	5
105	SRP-dependent cotranslational protein targeting to membrane
GO:0006613	3.57890162989507e-07	44.3347772277228	0.154037782852398
5	106	cotranslational protein targeting to membrane
GO:0045047	3.57890162989507e-07	44.3347772277228	0.154037782852398
5	106	protein targeting to ER
GO:0072599	3.57890162989507e-07	44.3347772277228	0.154037782852398
5	106	establishment of protein localization to endoplasmic reticulum

GO:0070972 6.11059274672063e-07 39.5934734513274 0.171476022420594
5 118 protein localization to endoplasmic reticulum
GO:0000184 6.37269221328044e-07 39.2434210526316 0.17292920905127
5 119 nuclear-transcribed mRNA catabolic process, nonsense-mediated decay
GO:0019080 2.07252023160669e-06 30.5736301369863 0.219431181233133
5 151 viral genome expression
GO:0019083 2.07252023160669e-06 30.5736301369863 0.219431181233133
5 151 viral transcription
GO:0006413 2.14102678163662e-06 30.3635204081633 0.220884367863816
5 152 translational initiation
GO:0006612 2.21130613221479e-06 30.15625 0.222337554494499 5
153 protein targeting to membrane
GO:0043624 2.21130613221479e-06 30.15625 0.222337554494499 5
153 cellular protein complex disassembly
GO:0043241 2.59046790562861e-06 29.1605392156863 0.229603487647914
5 158 protein complex disassembly
GO:0000956 3.60508079251103e-06 27.1836890243902 0.245588540585427
5 169 nuclear-transcribed mRNA catabolic process
GO:0034623 4.15875560962841e-06 26.3701923076923 0.252854473738842
5 174 cellular macromolecular complex disassembly
GO:0006402 4.64809310483942e-06 25.7532514450867 0.258667220261574
5 178 mRNA catabolic process
GO:0032984 4.77722089716488e-06 25.6034482758621 0.260120406892257
5 179 macromolecular complex disassembly
GO:0072594 9.03640818071238e-06 22.347675879397 0.296450072659332 5
204 establishment of protein localization to organelle
GO:0006401 9.25371449781464e-06 22.234375 0.297903259290015 5
205 RNA catabolic process
GO:0019058 1.65016098367336e-05 19.6404867256637 0.335686111687772
5 231 viral infectious cycle
Tissue: Heart_Left_Ventricle=>Lung Type: asymmetric
SourceGene: RP11-469M7.1(ENSG00000260006.1)
TargetGeneSet: EXOSC10 DHDDS IPP RP11 TMED5 NRAS GAPDHP32 ECM1
BGLAP POU2F1 TARBP1 DDX1 DHX57 ANKRD36 PSMD14 SPC25 THUMPD3 MAP4
TRAIP FAM116A PSMC1P1 POLQ KALRN CCNL1 ACTG1P1 PSMD2 CTA FAM200B
TRIP13 NUP155 AP3B1 CTB ANXA6 RARS MDC1 SLC35B2 MMS22L RP1
LPAL2 BET1 SAP25 RINT1 U1 ZDHHC2 WRN MAL2 VCP FBXW2
ITGB1 ZNF33A PBLD SFTPA2 SMPD1 PARVA FEN1 C11orf75 GUCY1A2
RPL23AP64 RNF26 CHEK1 RAD52 MED21 NUP107 NEDD1 UBL3 FANCB
FANCM POLE2 PLEKHG3 LRRC57 TPM1 KBTBD13 KIAA1199 FANCI PRC1
GDPD3 ZNF629 DHX38 TMEM170A ELAC2 ATAD5 CCT6B KIF18B TUBD1
BRIP1 ATP5A1 HAUS1 DSEL CHAF1A S1PR2 SPC24 SLC7A9 ZNF615 CDK5RAP1
MYH9 MBTPS2 SUV39H1 LONRF3
GOBPID Pvalue OddsRatio ExpCount Count Size Term
GO:0006281 1.96003099635743e-09 8.91898777173913 2.09376513736074
15 383 DNA repair
GO:0006302 4.15365138271882e-09 19.7405529953917 0.557608470002076
9 102 double-strand break repair
GO:0006310 1.95112202809718e-07 10.3218993518316 1.14255068853367
10 209 DNA recombination
GO:0045005 3.96064953679518e-07 95.76 0.0656009964708325 4 12
maintenance of fidelity involved in DNA-dependent DNA replication
GO:0006261 7.23288120010436e-07 16.1501937984496 0.508407722648952
7 93 DNA-dependent DNA replication
GO:0006260 1.90186696360858e-06 7.92832266037524 1.46508892118193
10 268 DNA replication
GO:0031297 5.4176636865537e-06 141.789473684211 0.0382672479413189
3 7 replication fork processing
GO:0000724 1.00390326144296e-05 20.1632882882883 0.289737734412843
5 53 double-strand break repair via homologous recombination
GO:0000725 1.10163471052765e-05 19.7504136789851 0.295204484118746
5 54 recombinational repair
GO:0051320 1.28279379400116e-05 10.1768790849673 0.781745207944087

7	143	S phase
Tissue: Heart_Left_Ventricle=>Lung	Type:	asymmetric
SourceGene:	RP11-705O3.1(ENSG00000246174.2)
TargetGeneSet:	RP11	TIGD2	GYPA	PCDHA5	TDP2	ABHD11	DEFA4	DEFA3
FAM108B1		MS4A3	TMEM121	MPO	CST2	RHOT1P2
GOBPID	Pvalue	OddsRatio	ExpCount	Count	Size	Term
GO:0050832	1.89422397636428e-07	424.264705882353	0.0124558854058543
3	20	defense response to fungus
GO:0009620	9.02902190532709e-07	240.2	0.0205522109196595	3	33
response to fungus
Tissue: Heart_Left_Ventricle=>Lung	Type:	asymmetric
SourceGene:	RP11-723D22.3(ENSG00000253829.1)
TargetGeneSet:	RPL11	7SK	RP4	RPL5	OCLM	ASXL2	UBC	RPL15	RP11
USP4	SLCO2A1	RPL35A	RPL34	RPS14	RPS10	RPL10A	MRPS12	KRIT1	IMMP2L	CTD
RPL30	RPS6	GKAP1	HSD17B3	SNORD90	LRRC8A	RPS24	RPL13AP5		C10orf12
PTPRE	RPS13	PRDX5	FAU	PFDN5	RBMS1P1	MDM2	CHST11	ESD	RPS3AP6
RPLP1	NFATC2IP		CDYL2	ALOX15B	RPL26	FOXO3B	RFFL	RPL17	RPL27
ZNF813	RPS9	NCOA3	ARFGEF2	MID2
GOBPID	Pvalue	OddsRatio	ExpCount	Count	Size	Term
GO:0006415	2.5603980931057e-31	151.313888888889	0.277074250916892
19	91	translational termination
GO:0006414	5.05762996742098e-30	126.557674418605	0.31970105875026
19	105	translational elongation
GO:0006614	5.05762996742098e-30	126.557674418605	0.31970105875026
19	105	SRP-dependent cotranslational protein targeting to membrane
GO:0006613	6.1519748877624e-30	125.094252873563	0.322745830738357
19	106	cotranslational protein targeting to membrane
GO:0045047	6.1519748877624e-30	125.094252873563	0.322745830738357
19	106	protein targeting to ER
GO:0072599	6.1519748877624e-30	125.094252873563	0.322745830738357
19	106	establishment of protein localization to endoplasmic reticulum
GO:0070972	5.56638703863715e-29	109.839191919192	0.35928309459553
19	118	protein localization to endoplasmic reticulum
GO:0000184	6.61303269373228e-29	108.7332	0.362327866583627	19
119	nuclear-transcribed mRNA catabolic process, nonsense-mediated decay
GO:0019080	8.09971816547013e-27	82.1893939393939	0.459760570202754
19	151	viral genome expression
GO:0019083	8.09971816547013e-27	82.1893939393939	0.459760570202754
19	151	viral transcription
GO:0006413	9.24146203052846e-27	81.5657142857143	0.462805342190852
19	152	translational initiation
GO:0006612	1.0534310133535e-26	80.9513432835821	0.46585011417895
19	153	protein targeting to membrane
GO:0043624	1.0534310133535e-26	80.9513432835821	0.46585011417895
19	153	cellular protein complex disassembly
GO:0043241	2.00007357656436e-26	78.0120863309352	0.481073974119438
19	158	protein complex disassembly
GO:0072594	5.01990939016781e-26	64.4157608695652	0.621133485571933
20	204	establishment of protein localization to organelle
GO:0000956	7.61359271306488e-26	72.2354666666667	0.514566465988513
19	169	nuclear-transcribed mRNA catabolic process
GO:0034623	1.35576286702987e-25	69.8807741935484	0.529790325929001
19	174	cellular macromolecular complex disassembly
GO:0006402	2.12402765554839e-25	68.1036477987421	0.541969413881392
19	178	mRNA catabolic process
GO:0032984	2.37230667119594e-25	67.67325	0.54501418586949	19
179	macromolecular complex disassembly
GO:0006401	3.40229111940558e-24	58.107311827957	0.62417825756003	19
205	RNA catabolic process
GO:0019058	3.47933016120422e-23	50.8877358490566	0.703342329250571
19	231	viral infectious cycle
GO:0022415	3.42058338548897e-22	44.6728630705394	0.791640716905404
19	260	viral reproductive process

GO:0071845	1.86427534219837e-21	40.5581886792453	0.864715244619749
19	284	cellular component disassembly at cellular level
GO:0022411	2.43672401642247e-21	39.9437918215613	0.87689433257214
19	288	cellular component disassembly
GO:0033365	4.22101928156436e-20	28.1892073874567	1.44017715037022
21	473	protein localization to organelle
GO:0006605	1.18992594108223e-18	25.6112334801762	1.44322192235831
20	474	protein targeting
GO:0016032	1.1618245029038e-17	24.5856481481481	1.37319216663207
19	451	viral reproduction
GO:0006364	2.51065623580698e-08	27.9102814154361	0.316656286762162
7	104	rRNA processing
GO:0042273	3.63450224529092e-08	179.9875	0.0365372638571725	4
12	ribosomal large subunit biogenesis
GO:0016072	3.71045693642895e-08	26.2734190501181	0.334924918690748
7	110	rRNA metabolic process
GO:0042254	2.99457733795892e-07	19.0055196041112	0.453671026226559
7	149	ribosome biogenesis
GO:0022613	3.33587853221997e-07	14.531490015361	0.685073697321985	8
225	ribonucleoprotein complex biogenesis
GO:0071843	5.27937993328423e-07	13.6373256373256	0.727700505155353
8	239	cellular component biogenesis at cellular level
GO:0034470	2.17447811327912e-06	13.933342669094	0.608954397619542	7
200	ncRNA processing
Tissue: Heart_Left_Ventricle=>Lung	Type:	asymmetric
SourceGene:	RP3-322G13.7(ENSG00000234832.1)
TargetGeneSet:	RP11	SNORA55	CYP4Z2P	ITGB5-AS1	MSMO1	SAP30	CTD
HMGCS1	PCDHB9	PPP1R16A	RP13	UPF3AP3	IKZF5	NTN4	MAP1LC3B2
ZDHHC20-IT1	C14orf1	MVD	CYCS	RBBP9	RPL36P4	MT
GOBPID	Pvalue	OddsRatio	ExpCount	Count	Size	Term
GO:0016126	1.138892003711e-07	127.928888888889	0.0474707632689779
4	49	sterol biosynthetic process
GO:0016125	4.10560577174981e-06	49.815652173913	0.115286139367518	4
119	sterol metabolic process
GO:0006694	7.8338182759668e-06	42.0617647058824	0.13563075219708
4	140	steroid biosynthetic process
GO:0006695	8.7311967723947e-06	98.1613636363636	0.0416580167462459
3	43	cholesterol biosynthetic process
Tissue: Lung=>Heart_Left_Ventricle	Type:	asymmetric
SourceGene:	RP4-604G5.1(ENSG00000237640.1)
TargetGeneSet:	KLHL17	NPPB	RP11	GDI2P2	DPYD-AS1	ATXN7L2	MYOC
PFN1P1	MIR4426	FTH1P2	ZNF692	DPY30	GALNT3	RPL37A	ILKAP	PDCD6IP	RPL7AP11
EEF1A1P25	RAP2B	CXCL5	TMSB4XP8	TNIP3	CTD	RPS23	FAM193B
GTF2H4	LYPLA2P1	RPL24P4	RP1	RPL23P8	EGFR	PDGFRL	FAM160B2
KIAA1875	ADCK5	ROR2	SKA2L	C9orf114	C10orf62	C11orf10
MIR1304	NOP2	EEF1A1P33	AAAS	TESPA1	RPL41	SPPL3	PUS1	RPS11P5
RPS29	CASC5	C16orf79	IRX6	YWHAE	GLP2R	PRR11	INTS2	RPL38
SLC38A10	CCDC137	NCLN	SNRPEP4	C19orf79	QTRT1	TRMT1	AP1M1
GRIK5	RP4	RPL24P2	C20orf24	RPS21	UCKL1	NCAM2	CTA	SGSM3
MAPK8IP2	PSMA6P2	PRICKLE3	PGAM4	FAM199X	RP3	FTH1P8
GOBPID	Pvalue	OddsRatio	ExpCount	Count	Size	Term
GO:0070972	9.71104964429525e-08	22.534009009009	0.383779669227043	7
118	protein localization to endoplasmic reticulum
GO:0006415	4.60459441584333e-07	24.6525107604017	0.295965677115771
6	91	translational termination
GO:0019080	5.24239948151167e-07	17.3298611111111	0.491107881807487
7	151	viral genome expression
GO:0019083	5.24239948151167e-07	17.3298611111111	0.491107881807487
7	151	viral transcription
GO:0006414	1.07442747741021e-06	21.1456023651146	0.341498858210504
6	105	translational elongation
GO:0006614	1.07442747741021e-06	21.1456023651146	0.341498858210504
6	105	SRP-dependent cotranslational protein targeting to membrane

GO:0006613 1.13612011101643e-06 20.9326829268293 0.3447512282887 6
106 cotranslational protein targeting to membrane
GO:0045047 1.13612011101643e-06 20.9326829268293 0.3447512282887 6
106 protein targeting to ER
GO:0072599 1.13612011101643e-06 20.9326829268293 0.3447512282887 6
106 establishment of protein localization to endoplasmic reticulum
GO:0000184 2.23897097097223e-06 18.507662421757 0.387032039305238 6
119 nuclear-transcribed mRNA catabolic process, nonsense-mediated decay
GO:0019058 8.88767604249368e-06 11.078125 0.75129748806311 7
231 viral infectious cycle
GO:0006413 9.22796613593862e-06 14.2913464751086 0.494360251885683
6 152 translational initiation
GO:0006612 9.58122597442802e-06 14.1931309109009 0.497612621963878
6 153 protein targeting to membrane
GO:0043624 9.58122597442802e-06 14.1931309109009 0.497612621963878
6 153 cellular protein complex disassembly
GO:0043241 1.15159550618145e-05 13.7214377406932 0.513874472354854
6 158 protein complex disassembly
GO:0016032 1.23085283597950e-05 7.48142414860681 1.46681890526607
9 451 viral reproduction
GO:0000956 1.68955941365674e-05 12.785575340416 0.549650543215002 6
169 nuclear-transcribed mRNA catabolic process
GO:0022415 1.91468811180636e-05 9.78824110671937 0.845616220330773
7 260 viral reproductive process
GO:0034623 1.99335748672826e-05 12.4006968641115 0.565912393605979
6 174 cellular macromolecular complex disassembly
Tissue: Lung=>Heart_Left_Ventricle Type: cluster
SourceGene: RP4-604G5.1(ENSG00000237640.1)
TargetGeneSet: KLHL17 NPPB RP11 GDI2P2 DPYD-AS1 ATXN7L2 MYOC
PFN1P1 MIR4426 FTH1P2 ZNF692 DPY30 GALNT3 RPL37A ILKAP PDCD6IP RPL7AP11
EEF1A1P25 RAP2B CXCL5 TMSB4XP8 TNIP3 CTD RPS23 FAM193B
GTF2H4 LYPLA2P1 RPL24P4 RP1 RPL23P8 EGFR PDGFRL FAM160B2
KIAA1875 ADCK5 ROR2 SKA2L C9orf114 C10orf62 C11orf10
MIR1304 NOP2 EEF1A1P33 AAAS TESPA1 RPL41 SPPL3 PUS1 RPS11P5
RPS29 CASC5 C16orf79 IRX6 YWHAE GLP2R PRR11 INTS2 RPL38
SLC38A10 CCDC137 NCLN SNRPEP4 C19orf79 QTRT1 TRMT1 AP1M1
GRIK5 RP4 RPL24P2 C20orf24 RPS21 UCKL1 NCAM2 CTA SGSM3
MAPK8IP2 PSMA6P2 PRICKLE3 PGAM4 FAM199X RP3 FTH1P8
GOBPID Pvalue OddsRatio ExpCount Count Size Term
GO:0070972 9.71104964429525e-08 22.534009009009 0.383779669227043 7
118 protein localization to endoplasmic reticulum
GO:0006415 4.60459441584333e-07 24.6525107604017 0.295965677115771
6 91 translational termination
GO:0019080 5.24239948151167e-07 17.3298611111111 0.491107881807487
7 151 viral genome expression
GO:0019083 5.24239948151167e-07 17.3298611111111 0.491107881807487
7 151 viral transcription
GO:0006414 1.07442747741021e-06 21.1456023651146 0.341498858210504
6 105 translational elongation
GO:0006614 1.07442747741021e-06 21.1456023651146 0.341498858210504
6 105 SRP-dependent cotranslational protein targeting to membrane
GO:0006613 1.13612011101643e-06 20.9326829268293 0.3447512282887 6
106 cotranslational protein targeting to membrane
GO:0045047 1.13612011101643e-06 20.9326829268293 0.3447512282887 6
106 protein targeting to ER
GO:0072599 1.13612011101643e-06 20.9326829268293 0.3447512282887 6
106 establishment of protein localization to endoplasmic reticulum
GO:0000184 2.23897097097223e-06 18.507662421757 0.387032039305238 6
119 nuclear-transcribed mRNA catabolic process, nonsense-mediated decay
GO:0019058 8.88767604249368e-06 11.078125 0.75129748806311 7
231 viral infectious cycle
GO:0006413 9.22796613593862e-06 14.2913464751086 0.494360251885683
6 152 translational initiation

GO:0006612	9.58122597442802e-06	14.1931309109009	0.497612621963878
6	153	protein targeting to membrane
GO:0043624	9.58122597442802e-06	14.1931309109009	0.497612621963878
6	153	cellular protein complex disassembly
GO:0043241	1.15159550618145e-05	13.7214377406932	0.513874472354854
6	158	protein complex disassembly
GO:0016032	1.2308528359795e-05	7.48142414860681	1.46681890526607
9	451	viral reproduction
GO:0000956	1.68955941365674e-05	12.785575340416 0.549650543215002	6
169	nuclear-transcribed mRNA catabolic process
GO:0022415	1.91468811180636e-05	9.78824110671937	0.845616220330773
7	260	viral reproductive process
GO:0034623	1.99335748672826e-05	12.4006968641115	0.565912393605979
6	174	cellular macromolecular complex disassembly
Tissue: Lung=>Heart_Left_Ventricle	Type:	asymmetric

SourceGene:	VENTXP6(ENSG00000254105.1)	VENT homeobox pseudogene 6

TargetGeneSet:	LIMS1	NDUFB3	CNPPD1	RP11	RP1	LSM5	SDHDP2	RPL8
RPS13	EEF1A1P33	RPS29	RPS17L	RPL38	ELMO2	ALG12	MED14-AS1

GOBPID	Pvalue	OddsRatio	ExpCount	Count	Size	Term

GO:0000956	3.75E-09	75.06573181	0.152031001	6	169
nuclear-transcribed mRNA catabolic process

GO:0006402	5.13E-09	71.09302326	0.160127327	6	178	mRNA
catabolic process

GO:0006415	1.10E-08	104.3023256	0.081862847	5	91
translational termination

GO:0006401	1.20E-08	61.33094042	0.184416303	6	205	RNA
catabolic process

GO:0006414	2.26E-08	89.6125 0.094457131	5	105
translational elongation

GO:0006614	2.26E-08	89.6125 0.094457131	5	105
SRP-dependent cotranslational protein targeting to membrane

GO:0006613	2.37E-08	88.71905941	0.095356723	5	106
cotranslational protein targeting to membrane

GO:0045047	2.37E-08	88.71905941	0.095356723	5	106
protein targeting to ER

GO:0072599	2.37E-08	88.71905941	0.095356723	5	106
establishment of protein localization to endoplasmic reticulum

GO:0070972	4.07E-08	79.23119469	0.106151823	5	118
protein localization to endoplasmic reticulum					
GO:0000184	4.25E-08	78.53070175	0.107051415	5	119
"nuclear-transcribed mRNA catabolic process, nonsense-mediated decay"					
GO:0019080	1.40E-07	61.18150685	0.13583835	5	151
viral genome expression					
GO:0019083	1.40E-07	61.18150685	0.13583835	5	151
viral transcription					
GO:0006413	1.45E-07	60.76105442	0.136737942	5	152
translational initiation					
GO:0006612	1.50E-07	60.34628378	0.137637534	5	153
protein targeting to membrane					
GO:0043624	1.50E-07	60.34628378	0.137637534	5	153
cellular protein complex disassembly					
GO:0043241	1.76E-07	58.35375817	0.142135492	5	158
protein complex disassembly					
GO:0034623	2.84E-07	52.76997041	0.15652896	5	174
cellular macromolecular complex disassembly					
GO:0032984	3.27E-07	51.23563218	0.161026919	5	179
macromolecular complex disassembly					
GO:0072594	6.27E-07	44.72047739	0.183516712	5	204
establishment of protein localization to organelle					
GO:0019058	1.16E-06	39.30309735	0.207805688	5	231
viral infectious cycle					
GO:0022415	2.08E-06	34.7622549	0.233893848	5	260
viral reproductive process					
GO:0071845	3.20E-06	31.71818996	0.25548405	5	284
cellular component disassembly at cellular level					
GO:0022411	3.43E-06	31.2610424	0.259082416	5	288
cellular component disassembly					

Tissue: Lung=>Muscle_Skeletal Type: asymmetric
SourceGene: CALR(ENSG00000179218.6)
TargetGeneSet: C1orf192 C2orf44 OLA1 SNORD19B RP11 PCNP

SLC41A3 TBCA DEK CSNK2B NT5C3 ZKSCAN5 EIF3E EIF3F RPL27A RPS13
KDM5A CLEC4A VPS29 RPLP0 TPT1 RNASEH2B C14orf132 RPAP1 MGA
SPG11 RPS15A DNAJC7 SDK2 CTD IER3IP1 RPSAP58 ZNF526 ZNF765 ZSWIM1
PRR14L C22orf32 RPL10 EIF1AY
GOBPID Pvalue OddsRatio ExpCount Count Size Term
GO:0006413 9.23058599239458e-09 34.4665517241379 0.283994187253477
7 152 translational initiation
GO:0000184 7.05889254764862e-08 36.1845764854614 0.222337554494499
6 119 nuclear-transcribed mRNA catabolic process, nonsense-mediated decay
GO:0006401 7.28458370460635e-08 25.1469696969697 0.383018476230019
7 205 RNA catabolic process
GO:0000956 5.65205978561604e-07 24.9973707274321 0.315756695038406
6 169 nuclear-transcribed mRNA catabolic process
GO:0006415 6.41277891250554e-07 37.8911205073996 0.170022835789911
5 91 translational termination
GO:0006402 7.66472280893686e-07 23.6744186046512 0.332572140336309
6 178 mRNA catabolic process
GO:0006414 1.3080209252186e-06 32.5545454545455 0.196180195142205
5 105 translational elongation
GO:0006614 1.3080209252186e-06 32.5545454545455 0.196180195142205
5 105 SRP-dependent cotranslational protein targeting to membrane
GO:0006613 1.3710283642215e-06 32.2299729972997 0.198048577953083
5 106 cotranslational protein targeting to membrane
GO:0045047 1.3710283642215e-06 32.2299729972997 0.198048577953083
5 106 protein targeting to ER
GO:0072599 1.3710283642215e-06 32.2299729972997 0.198048577953083
5 106 establishment of protein localization to endoplasmic reticulum
GO:0070972 2.33117693976938e-06 28.783185840708 0.220469171683621 5
118 protein localization to endoplasmic reticulum
GO:0019058 3.49388704343774e-06 18.0304761904762 0.43159642931285
6 231 viral infectious cycle
GO:0022415 6.89985689854706e-06 15.9392575928009 0.485779530828316
6 260 viral reproductive process
GO:0019080 7.81678864797814e-06 22.2260273972603 0.282125804442599
5 151 viral genome expression
GO:0019083 7.81678864797814e-06 22.2260273972603 0.282125804442599
5 151 viral transcription
GO:0006612 8.33446558546097e-06 21.9226044226044 0.285862570064355
5 153 protein targeting to membrane
GO:0043624 8.33446558546097e-06 21.9226044226044 0.285862570064355
5 153 cellular protein complex disassembly
GO:0043241 9.74664991833363e-06 21.198752228164 0.295204484118746 5
158 protein complex disassembly
GO:0016032 1.42153456865043e-05 11.0202702702703 0.842640647706041
7 451 viral reproduction
GO:0034623 1.55609181597683e-05 19.170252824099 0.325098609092796 5
174 cellular macromolecular complex disassembly
GO:0032984 1.78441369704505e-05 18.6128526645768 0.334440523147187
5 179 macromolecular complex disassembly
Tissue: Muscle_Skeletal=>Lung Type: asymmetric
SourceGene: CTD-3064C13.1(ENSG00000255372.1)
TargetGeneSet: RPL22 RPL5 RP11 RPL15 DNAJC13 KLHL24 HMGCS1 BTF3 CTB
RPS14 RP3 RPL10A EEF1A1 RAC1 TOMM7 UQCRB EIF3H EEF1D IDI2-AS1
PFN1P11 RPS13 CTSF DHCR7 IL18BP B3GAT1 TPT1 GZMH MAPKBP1 RPL4
ANKFY1 RPS11 PNPLA4
GOBPID Pvalue OddsRatio ExpCount Count Size Term
GO:0006414 2.0142792696908e-15 88.7244582043344 0.196180195142205
10 105 translational elongation
GO:0006415 3.63613987228667e-12 72.7507926442613 0.170022835789911
8 91 translational termination
GO:0006413 4.95086588893475e-12 49.9335664335664 0.283994187253477
9 152 translational initiation
GO:0006614 1.17345503194058e-11 62.1899077590884 0.196180195142205

8	105	SRP-dependent cotranslational protein targeting to membrane
GO:0006613	1.26775622401036e-11	61.5510204081633	0.198048577953083
8	106	cotranslational protein targeting to membrane
GO:0045047	1.26775622401036e-11	61.5510204081633	0.198048577953083
8	106	protein targeting to ER
GO:0072599	1.26775622401036e-11	61.5510204081633	0.198048577953083
8	106	establishment of protein localization to endoplasmic reticulum
GO:0070972	3.03203010637571e-11	54.7904306220096	0.220469171683621
8	118	protein localization to endoplasmic reticulum
GO:0000184	3.24673383521069e-11	54.2930298719772	0.222337554494499
8	119	nuclear-transcribed mRNA catabolic process, nonsense-mediated decay
GO:0072594	7.01935279172028e-11	36.4846153846154	0.381150093419141
9	204	establishment of protein localization to organelle
GO:0019080	2.21326302210561e-10	42.0493191019507	0.282125804442599
8	151	viral genome expression
GO:0019083	2.21326302210561e-10	42.0493191019507	0.282125804442599
8	151	viral transcription
GO:0006612	2.45931590628955e-10	41.4635208711434	0.285862570064355
8	153	protein targeting to membrane
GO:0043624	2.45931590628955e-10	41.4635208711434	0.285862570064355
8	153	cellular protein complex disassembly
GO:0043241	3.1811205942258e-10	40.0673684210526	0.295204484118746
8	158	protein complex disassembly
GO:0000956	5.44445628484361e-10	37.3010787839163	0.315756695038406
8	169	nuclear-transcribed mRNA catabolic process
GO:0034623	6.86790688362788e-10	36.1648700063412	0.325098609092796
8	174	cellular macromolecular complex disassembly
GO:0006402	8.22921123361849e-10	35.3040247678019	0.332572140336309
8	178	mRNA catabolic process
GO:0032984	8.6040489338959e-10	35.0951061865189	0.334440523147187
8	179	macromolecular complex disassembly
GO:0006401	2.52017424606074e-09	30.4076943628106	0.383018476230019
8	205	RNA catabolic process
GO:0019058	6.4533641082341e-09	26.8133113051688	0.43159642931285
8	231	viral infectious cycle
GO:0022415	1.62842937280258e-08	23.6791979949875	0.485779530828316
8	260	viral reproductive process
GO:0071845	3.23747950135079e-08	21.58352402746	0.530620718289392	8
284	cellular component disassembly at cellular level
GO:0022411	3.60866072584282e-08	21.2691729323308	0.538094249532904
8	288	cellular component disassembly
GO:0016032	7.37343343458781e-08	15.816742081448	0.842640647706041	9
451	viral reproduction
GO:0033365	1.10786645196307e-07	15.0431034482759	0.88374506954536
9	473	protein localization to organelle
GO:0006605	1.12801165729686e-07	15.0096774193548	0.885613452356238
9	474	protein targeting
Tissue: Lung=>Muscle_Skeletal	Type:	asymmetric
SourceGene:	DCTN1(ENSG00000204843.7)
TargetGeneSet:	GNL2	C1orf109	DPH2	SORT1	LRIF1	PFDN2	PAPPA2
TIMM17A	BROX	SRSF7	PSME4	RAB1A	CNNM3	MKI67IP	NUP35	ANKAR	MARS2
C2orf47	PPIL3	WDR12	STK36	RAMP1	LSM3	ARF4	MITF	CCDC58	ABTB1	UBA5
USP13	GRPEL1	GPR125	ATP8A1	OCIAD1	PAICS	EGF	GAB1	ANAPC10	CLPTM1L
BRIX1	C5orf43	SRFBP1	PPP2CA	HSPA9	MRPL22	PAK1IP1	TPMT	HLA-DMA	PPIL1
MAD2L1BP	PHF3	NDUFAF4	HECA	LTV1	MALSU1	FKBP14	GGCT	GATSL2
ASB15	MKRN1	SORBS3	FAM219A	DNAJB5	SEC61B	MRPL50	DPM2	ZER1	ZNF25
TIMM23	PPIF	PGAM1	MXI1	RP11	HIPK3	FIBP	DNAJB13	TMEM126A
ARHGAP42	NOP2	DTX3	CDK4	CTDSP2	CCT2	COX6A1	TBC1D4	ZBTB25
BCRP2	COX16	CINP	CTD	UNKL	C16orf91	IFT140	NPIPP1	MPHOSPH6
TIMM22	ANKFY1	C1QBP	ATP1B2	ERAL1	MAP3K3	DDX5	MEP1B	MRPL4	ANKRD27
TIMM50	SRSF6	SDC4	ATP5E	FBXW11P1	C21orf33	RP3	PLA2G6
NHP2L1
GOBPID	Pvalue	OddsRatio	ExpCount	Count	Size	Term

GO:0006457 1.34450967259763e-07 9.26133254015467 1.39007681129334
11 216 protein folding
GO:0022613 1.79241599838792e-06 7.92546931913701 1.44799667843056
10 225 ribonucleoprotein complex biogenesis
GO:0071843 3.0822405348495e-06 7.43357710317252 1.5380942495329 10
239 cellular component biogenesis at cellular level
GO:0006839 4.65300458254365e-06 11.966794533685 0.669296242474569 7
104 mitochondrial transport
GO:0042254 5.14077077224417e-06 9.48986232790989 0.958895578160681
8 149 ribosome biogenesis
GO:0006626 1.84154687313195e-05 17.6778656126482 0.32821258044426
5 51 protein targeting to mitochondrion
Tissue: Lung=>Muscle_Skeletal Type: cluster
SourceGene: DCTN1(ENSG00000204843.7)
TargetGeneSet: GNL2 C1orf109 DPH2 SORT1 LRIF1 PFDN2 PAPPA2
TIMM17A BROX SRSF7 PSME4 RAB1A CNNM3 MKI67IP NUP35 ANKAR MARS2
C2orf47 PPIL3 WDR12 STK36 RAMP1 LSM3 ARF4 MITF CCDC58 ABTB1 UBA5
USP13 GRPEL1 GPR125 ATP8A1 OCIAD1 PAICS EGF GAB1 ANAPC10 CLPTM1L
BRIX1 C5orf43 SRFBP1 PPP2CA HSPA9 MRPL22 PAK1IP1 TPMT HLA-DMA PPIL1
MAD2L1BP PHF3 NDUFAF4 HECA LTV1 MALSU1 FKBP14 GGCT GATSL2
ASB15 MKRN1 SORBS3 FAM219A DNAJB5 SEC61B MRPL50 DPM2 ZER1 ZNF25
TIMM23 PPIF PGAM1 MXI1 RP11 HIPK3 FIBP DNAJB13 TMEM126A
ARHGAP42 NOP2 DTX3 CDK4 CTDSP2 CCT2 COX6A1 TBC1D4 ZBTB25
BCRP2 COX16 CINP CTD UNKL C16orf91 IFT140 NPIPP1 MPHOSPH6
TIMM22 ANKFY1 C1QBP ATP1B2 ERAL1 MAP3K3 DDX5 MEP1B MRPL4 ANKRD27
TIMM50 SRSF6 SDC4 ATP5E FBXW11P1 C21orf33 RP3 PLA2G6
NHP2L1
GOBPID Pvalue OddsRatio ExpCount Count Size Term
GO:0006457 1.34450967259763e-07 9.26133254015467 1.39007681129334
11 216 protein folding
GO:0022613 1.79241599838792e-06 7.92546931913701 1.44799667843056
10 225 ribonucleoprotein complex biogenesis
GO:0071843 3.0822405348495e-06 7.43357710317252 1.5380942495329 10
239 cellular component biogenesis at cellular level
GO:0006839 4.65300458254365e-06 11.966794533685 0.669296242474569 7
104 mitochondrial transport
GO:0042254 5.14077077224417e-06 9.48986232790989 0.958895578160681
8 149 ribosome biogenesis
GO:0006626 1.84154687313195e-05 17.6778656126482 0.32821258044426
5 51 protein targeting to mitochondrion
Tissue: Muscle_Skeletal=>Lung Type: asymmetric
SourceGene: EIF1AY(ENSG00000198692.5)
TargetGeneSet: EIF2B4 UBC DYSF IL18RAP LIPA CXCR1 TBC1D5 ALAS1
RPL24 TMED10P2 UTS2D RP11 BTF3 NSA2 TBCA KRT8P32 ETF1
PCDHGB7 RPS14 SRPK1 SKAP2 IMPDH1 EEF1D BAAT EDF1 RPL27A SBF2 CTSF
RPL36A TARSL2 C16orf5 RPL17 CALR FFAR2 SNRPD2 RPL18 RPL13A
GOBPID Pvalue OddsRatio ExpCount Count Size Term
GO:0006415 1.67918420777686e-11 57.574297188755 0.201508546121376 8
91 translational termination
GO:0006414 5.39567704313388e-11 49.2164948453608 0.23250986090928
8 105 translational elongation
GO:0000184 1.48643933171072e-10 42.966966966967 0.263511175697184 8
119 nuclear-transcribed mRNA catabolic process, nonsense-mediated decay
GO:0006413 1.05747743506729e-09 33.0439814814815 0.336585703411529
8 152 translational initiation
GO:0043624 1.11416252329696e-09 32.8137931034483 0.338800083039236
8 153 cellular protein complex disassembly
GO:0043241 1.43893936799346e-09 31.7088888888889 0.349871981177773
8 158 protein complex disassembly
GO:0000956 2.45436106968739e-09 29.51966873706 0.374230157082555 8
169 nuclear-transcribed mRNA catabolic process
GO:0006614 2.53183530314578e-09 40.9171428571429 0.23250986090928
7 105 SRP-dependent cotranslational protein targeting to membrane

GO:0006613	2.70673351802074e-09	40.5010101010101	0.234724240536987
7	106	cotranslational protein targeting to membrane
GO:0045047	2.70673351802074e-09	40.5010101010101	0.234724240536987
7	106	protein targeting to ER
GO:0072599	2.70673351802074e-09	40.5010101010101	0.234724240536987
7	106	establishment of protein localization to endoplasmic reticulum
GO:0034623	3.0912646162229e-09	28.6204819277108	0.385302055221092
8	174	cellular macromolecular complex disassembly
GO:0006402	3.69940779345068e-09	27.9392156862745	0.394159573731922
8	178	mRNA catabolic process
GO:0032984	3.86671722201437e-09	27.7738791423002	0.396373953359629
8	179	macromolecular complex disassembly
GO:0070972	5.74981541988534e-09	36.0922522522523	0.261296796069476
7	118	protein localization to endoplasmic reticulum
GO:0006401	1.1234998341734e-08	24.0642978003384	0.453947823680022
8	205	RNA catabolic process
GO:0019058	2.85383685772976e-08	21.219730941704 0.511521694000415	8
231	viral infectious cycle
GO:0019080	3.19859803578109e-08	27.7569444444444	0.334371323783821
7	151	viral genome expression
GO:0019083	3.19859803578109e-08	27.7569444444444	0.334371323783821
7	151	viral transcription
GO:0006612	3.50308231415754e-08	27.3728767123288	0.338800083039236
7	153	protein targeting to membrane
GO:0022415	7.13683891621245e-08	18.739417989418 0.575738703203931	8
260	viral reproductive process
GO:0071845	1.40834573230078e-07	17.0809178743961	0.628883814268909
8	284	cellular component disassembly at cellular level
GO:0022411	1.56786621481419e-07	16.8321428571429	0.637741332779738
8	288	cellular component disassembly
GO:0072594	2.51491040811721e-07	20.2140101522843	0.451733444052315
7	204	establishment of protein localization to organelle
GO:0016032	4.60034359857129e-06	10.5161775771257	0.998685212096049
8	451	viral reproduction
GO:0033365	6.53335266389927e-06	10.0028673835125	1.04740156390561
8	473	protein localization to organelle
GO:0006605	6.63550110170467e-06	9.98068669527897	1.04961594353332
8	474	protein targeting
Tissue: Lung=>Muscle_Skeletal	Type:	asymmetric
SourceGene:	ENSG00000217643.1
TargetGeneSet:	ACAP3	TNFRSF25	CROCCP3 C1orf63 CPT2	DOCK7	SGIP1	RP4
HFM1	MTMR11	FAM189B PEX19	B4GALT3 KCNF1	MSH6	STON1	GFPT1	POLR1A
ARL6IP6 RFTN2	SLC23A3 TAMM41	KIAA1143	CCDC12	RRP9	PPP4R2	UBA5	RP11
PARL	ZNF718	HGFAC	OCIAD1	EXOSC9	GAB1	TMA16	LPCAT1	YTHDC2	SRFBP1
SLC23A2 IK	NHLRC1	HCG20	RP1	TRMT11	HECA	VIP	NSUN5	CLIP2
AGAP3	GNRH1	PPP2R2A GDAP1	NOL6	FANCC	XPA	STXBP1	FPGS	URM1
C9orf106	USP20	EXOSC2	EDF1	SLC34A3 ARHGAP21	ECD	SEC23IP
KCNQ1-AS1	DNAJC24 PEX16	FNBP4	BEST1	ANKRD13D	TCIRG1	ATG16L2
SRSF8	TECTA	RARG	NPFF	AVIL	THAP2	SNRPF	U4	FAM207BP
MRP63	NUPL1	STK24-AS1	FAM70B	C14orf21	SCFD1	SOCS6	ZBTB1	ERH
ABCD4	ACYP1	C15orf44	COMMD4	FAM103A1	MRPL28	ALG1	PMM2	MAZ
ITGAX	CCDC102A	KATNB1	DYNC1LI2	KLHDC4	TRAPPC2L	SNORD68 PIGS
TBC1D3	KLHL11	VPS25	ALYREF	ENOSF1	RNMT	CABLES1 IMPACT	CXXC1	CIRBP	CAPS
PDE4A	SAMD1	MEF2BNB URI1	WDR88	MED29	TIMM50	RUVBL2	MBOAT7	TMEM238
ZNF667	MZF1	ANKRD5	CBFA2T2 PABPC1L STX16	USP16	C21orf67	RAB9A
FAM3C2	RBM3	HSD17B10	TSR2	HNRNPH2 FERP1
GOBPID	Pvalue	OddsRatio	ExpCount	Count	Size	Term
GO:0071843	1.54298032144831e-06	7.09411549707602	1.76963531935506
11	239	cellular component biogenesis at cellular level
Tissue: Lung=>Muscle_Skeletal	Type:	cluster
SourceGene:	ENSG00000217643.1
TargetGeneSet:	ACAP3	TNFRSF25	CROCCP3 C1orf63 CPT2	DOCK7	SGIP1	RP4
HFM1	MTMR11	FAM189B PEX19	B4GALT3 KCNF1	MSH6	STON1	GFPT1	POLR1A

ARL6IP6 RFTN2 SLC23A3 TAMM41 KIAA1143 CCDC12 RRP9 PPP4R2 UBA5 RP11
PARL ZNF718 HGFAC OCIAD1 EXOSC9 GAB1 TMA16 LPCAT1 YTHDC2 SRFBP1
SLC23A2 IK NHLRC1 HCG20 RP1 TRMT11 HECA VIP NSUN5 CLIP2
AGAP3 GNRH1 PPP2R2A GDAP1 NOL6 FANCC XPA STXBP1 FPGS URM1
C9orf106 USP20 EXOSC2 EDF1 SLC34A3 ARHGAP21 ECD SEC23IP
KCNQ1-AS1 DNAJC24 PEX16 FNBP4 BEST1 ANKRD13D TCIRG1 ATG16L2
SRSF8 TECTA RARG NPFF AVIL THAP2 SNRPF U4 FAM207BP
MRP63 NUPL1 STK24-AS1 FAM70B C14orf21 SCFD1 SOCS6 ZBTB1 ERH
ABCD4 ACYP1 C15orf44 COMMD4 FAM103A1 MRPL28 ALG1 PMM2 MAZ
ITGAX CCDC102A KATNB1 DYNC1LI2 KLHDC4 TRAPPC2L SNORD68 PIGS
TBC1D3 KLHL11 VPS25 ALYREF ENOSF1 RNMT CABLES1 IMPACT CXXC1 CIRBP CAPS
PDE4A SAMD1 MEF2BNB URI1 WDR88 MED29 TIMM50 RUVBL2 MBOAT7 TMEM238
ZNF667 MZF1 ANKRD5 CBFA2T2 PABPC1L STX16 USP16 C21orf67 RAB9A
FAM3C2 RBM3 HSD17B10 TSR2 HNRNPH2 FERP1
GOBPID Pvalue OddsRatio ExpCount Count Size Term
GO:0071843 1.54298032144831e-06 7.09411549707602 1.76963531935506
11 239 cellular component biogenesis at cellular level
Tissue: Lung=>Muscle_Skeletal Type: asymmetric
SourceGene: OCA2(ENSG00000104044.10)
TargetGeneSet: CPT2 THEM5 CHTOP KLHL29 RAB10 MGAT4A MFF RP11 LDB2
RASGRF2 EIF4EBP3 GEMIN5 TSPYL4 RSPO3 FAM220A TSPAN13 TRGC1 DBNL CDK6
ACHE PPP2R2A PDP1 KIAA2026 FRMD3 USP20 FAM78A ARRDC1 BMI1 KLLN
BET1L MAP4K2 SIPA1 ANKRD42 FLI1 EFNB2 RHOJ CTD FMN1 CD2BP2
DYNC1LI2 AKAP1 ZFP161 SOGA2 LONP1 SAMD1 MEF2BNB URI1 ERCC2
RUVBL2 PRPF6 H1F0 GSPT2
GOBPID Pvalue OddsRatio ExpCount Count Size Term
GO:0000244 3.61442722939206e-07 400.25 0.0161926510276105 3 6
assembly of spliceosomal tri-snRNP
GO:0000387 1.35215838565712e-06 60.8888888888889 0.0836620303093212
4 31 spliceosomal snRNP assembly
Tissue: Muscle_Skeletal=>Lung Type: asymmetric
SourceGene: OR7C1(ENSG00000127530.2)
TargetGeneSet: RP11 CD53 SETP9 ARHGAP30 TRAF3IP3 WIPF1
EEF1A1P25 TMED11P IRF2 DOCK2 BTN3A2 IKZF1 FDPSP2 NCKAP1L ARL11
SLC7A7 SNX20 EVI2B PRR22 CD37 SASH3
GOBPID Pvalue OddsRatio ExpCount Count Size Term
GO:0046631 9.23103529146026e-07 73.6358974358974 0.0794408691440039
4 82 alpha-beta T cell activation
GO:0046638 2.89385018698768e-06 145.555555555556 0.02906373261366
3 30 positive regulation of alpha-beta T cell differentiation
GO:0046637 7.00204710918774e-06 106.142506142506 0.0387516434848799
3 40 regulation of alpha-beta T cell differentiation
GO:0046635 8.7311967723947e-06 98.1613636363636 0.0416580167462459
3 43 positive regulation of alpha-beta T cell activation
GO:0030217 1.20235178036476e-05 37.5921052631579 0.151131409591032
4 156 T cell differentiation
GO:0045582 1.64793124999097e-05 78.4745454545454 0.0513459276174659
3 53 positive regulation of T cell differentiation
GO:0002639 1.82525001619232e-05 481.066666666667 0.00678153760985399
2 7 positive regulation of immunoglobulin production
GO:0046634 1.84342500519685e-05 75.4458041958042 0.0532835097917099
3 55 regulation of alpha-beta T cell activation
Tissue: Muscle_Skeletal=>Lung Type: asymmetric
SourceGene: RASA3-IT1(ENSG00000232487.1)
TargetGeneSet: RPL11 RP11 RPL32 LNP1 RPL35A GNPDA2 FAM198B RPL37
ARHGAP26 RPS14 RPL30 RPL35 DNAJC9 PRKRIR COX16 RPS21 CRLF2
GOBPID Pvalue OddsRatio ExpCount Count Size Term
GO:0006415 5.2570415996395e-15 230.586345381526 0.0881599889281019
8 91 translational termination
GO:0006414 1.71575130044712e-14 197.113402061856 0.10172306414781
8 105 translational elongation
GO:0006614 1.71575130044712e-14 197.113402061856 0.10172306414781
8 105 SRP-dependent cotranslational protein targeting to membrane

GO:0006613	1.85512348484428e-14	195.08843537415 0.102691855234932	8
106	cotranslational protein targeting to membrane
GO:0045047	1.85512348484428e-14	195.08843537415 0.102691855234932	8
106	protein targeting to ER
GO:0072599	1.85512348484428e-14	195.08843537415 0.102691855234932	8
106	establishment of protein localization to endoplasmic reticulum
GO:0070972	4.47983465610689e-14	173.660606060606	0.114317348280396
8	118	protein localization to endoplasmic reticulum
GO:0000184	4.80092081827107e-14	172.084084084084	0.115286139367518
8	119	nuclear-transcribed mRNA catabolic process, nonsense-mediated decay
GO:0019080	3.35814083575264e-13	133.277389277389	0.146287454155422
8	151	viral genome expression
GO:0019083	3.35814083575264e-13	133.277389277389	0.146287454155422
8	151	viral transcription
GO:0006413	3.54338439066552e-13	132.342592592593	0.147256245242544
8	152	translational initiation
GO:0006612	3.73748925539744e-13	131.420689655172	0.148225036329666
8	153	protein targeting to membrane
GO:0043624	3.73748925539744e-13	131.420689655172	0.148225036329666
8	153	cellular protein complex disassembly
GO:0043241	4.85395133590566e-13	126.995555555556	0.153068991765276
8	158	protein complex disassembly
GO:0000956	8.38147272952354e-13	118.227743271222	0.163725693723618
8	169	nuclear-transcribed mRNA catabolic process
GO:0034623	1.06155147079757e-12	114.626506024096	0.168569649159228
8	174	cellular macromolecular complex disassembly
GO:0006402	1.2760738371205e-12	111.898039215686	0.172444813507716
8	178	mRNA catabolic process
GO:0032984	1.33527505900153e-12	111.235867446394	0.173413604594838
8	179	macromolecular complex disassembly
GO:0072594	3.83959375516869e-12	96.8775510204082	0.197633381772888
8	204	establishment of protein localization to organelle
GO:0006401	3.99402407328448e-12	96.3790186125212	0.19860217286001
8	205	RNA catabolic process
GO:0019058	1.04445206758584e-11	84.9865470852018	0.223790741125182
8	231	viral infectious cycle
GO:0022415	2.69809871595179e-11	75.0529100529101	0.25188568265172
8	260	viral reproductive process
GO:0071845	5.46934795574173e-11	68.4106280193237	0.275136668742648
8	284	cellular component disassembly at cellular level
GO:0022411	6.11620460408393e-11	67.4142857142857	0.279011833091136
8	288	cellular component disassembly
GO:0016032	2.15543429215612e-09	42.1188863807374	0.436924780292021
8	451	viral reproduction
GO:0033365	3.13802233915605e-09	40.063082437276 0.458238184208705	8
473	protein localization to organelle
GO:0006605	3.19068290243744e-09	39.9742489270386	0.459206975295827
8	474	protein targeting
GO:0022613	1.56277436514447e-06	35.9015151515151	0.21797799460245
5	225	ribonucleoprotein complex biogenesis
GO:0071843	2.10343614093234e-06	33.7203228869896	0.231541069822158
5	239	cellular component biogenesis at cellular level
GO:0006364	2.39727680017796e-06	57.348	0.100754273060688	4	104
rRNA processing
GO:0016072	2.99988324947846e-06	54.0792452830189	0.10656701958342
4	110	rRNA metabolic process
GO:0042254	1.00270629752182e-05	39.4262068965517	0.144349871981178
4	149	ribosome biogenesis
GO:0030490	1.82525001619232e-05	481.066666666667	0.00678153760985399
2	7	maturation of SSU-rRNA
Tissue: Lung=>Muscle_Skeletal	Type:	asymmetric
SourceGene:	RLBP1(ENSG00000140522.7)
TargetGeneSet:	LRRC8D	C1orf68 PTPRC	LINC00467	RP11	PLEK	NGLY1	RP1

TTYH3 ST7-OT4 TRIM14 SUSD1 OR5B2 PTPN6 TWF1 ATF1 LCP1 TSHR
PSTPIP1 CORO1A ITGAX RAB11FIP4 VAV1 IFI30 HCST LGALS7B SIGLEC9 HCK
NCF4 CTA PDZD4
GOBPID Pvalue OddsRatio ExpCount Count Size Term
GO:0007229 5.26466458883855e-06 42.7440476190476 0.117638917721957
4 68 integrin-mediated signaling pathway
GO:0008064 1.28328803890419e-05 33.7330981775426 0.147048647152446
4 85 regulation of actin polymerization or depolymerization
GO:0030832 1.34432130162966e-05 33.3193960511034 0.148778631236593
4 86 regulation of actin filament length
GO:0007015 1.81297937854322e-05 18.7315789473684 0.337346896408553
5 195 actin filament organization
Tissue: Muscle_Skeletal=>Lung Type: asymmetric
SourceGene: RP11-417L19.4(ENSG00000255992.1)
TargetGeneSet: RP4 CDC20 DEPDC1 WDR26 SGOL1 RP1 HIPK2 RPL3P9 SF1
DNAJC12 FZD4 RP11 C15orf41 FAM149B2 PLK1 KIF18B U6
GOBPID Pvalue OddsRatio ExpCount Count Size Term
GO:0060041 7.98517850623222e-07 79.7166666666667 0.0780568818766867
4 94 retina development in camera-type eye
GO:0051488 1.32441885892811e-05 577.36 0.00581274652273199 2 7
activation of anaphase-promoting complex activity
GO:0043010 1.85010637569267e-05 35.064039408867 0.171891218600789 4
207 camera-type eye development
Tissue: Muscle_Skeletal=>Lung Type: cluster
SourceGene: RP11-462G12.2(ENSG00000262888.1)
TargetGeneSet: IGKJ5 IGKC IGKJ4 IGKJ3 IGKJ2 IGKJ1 IGKV1-12
IGKV1-27 IGKV1-33 IGKV1D-27 TM4SF1-AS1 CHRD IGJ
CALML5 RP11 IGHG1 IGHG3 IGHJ6 IGHJ5 IGHJ3 IGHV4-34 IGHV5-51
KRT10 DSC2 IGLV2-14 IGLL5 IGLC1 IGLC3 IGLC4
GOBPID Pvalue OddsRatio ExpCount Count Size Term
GO:0006958 1.83629841921271e-10 266.574074074074 0.0380596498512214
5 50 complement activation, classical pathway
GO:0006956 6.06437703370626e-10 206.637931034483 0.0479551588125389
5 63 complement activation
GO:0002455 7.12155292763391e-10 199.722222222222 0.0494775448065878
5 65 humoral immune response mediated by circulating immunoglobulin
GO:0072376 2.33865100137353e-09 155.443722943723 0.062417825756003
5 82 protein activation cascade
GO:0016064 1.08748917995986e-08 112.688679245283 0.0844924226697114
5 111 immunoglobulin mediated immune response
GO:0019724 1.24433927273075e-08 109.564220183486 0.0867760016607847
5 114 B cell mediated immunity
GO:0006959 1.75213588880413e-08 102.01566951567 0.0928655456369801 5
122 humoral immune response
GO:0002449 8.47891311072625e-08 73.4465020576132 0.127119230503079
5 167 lymphocyte mediated immunity
GO:0002460 1.26787130696983e-07 67.5378787878788 0.137775932461421
5 181 adaptive immune response based on somatic recombination of immune receptors built from immunoglobulin superfamily domains
GO:0002250 1.98391196917433e-07 61.5155440414508 0.150716213410837
5 198 adaptive immune response
GO:0002443 2.72274185450078e-07 57.5809061488673 0.160611722372154
5 211 leukocyte mediated immunity
GO:0002253 1.52998646745843e-06 40.0963718820862 0.227596706110304
5 299 activation of immune response
GO:0050778 4.02276505197398e-06 32.6857010213556 0.277074250916892
5 364 positive regulation of immune response
GO:0002252 5.36131152318692e-06 30.7502187226597 0.293820496851429
5 386 immune effector process
Tissue: Muscle_Skeletal=>Lung Type: asymmetric
SourceGene: RP11-706O15.5(ENSG00000205663.4)
TargetGeneSet: RPL11 RPL5 RPL31 ALDH7A1P2 RPL37A RPL32 RP11
RPL35A RPL34 RPL37 CTB PANK3 RPL10A SLC4A2 CHPF2 FAM150A UQCRB

RPL30 POLR2K SCRIB RPS6 C9orf103 SLC27A4 UNC5B RPS24 RPS25
C12orf39 SNORA49 SAP18 TPT1 EGLN3 POMT2 MAPKBP1 RPL4 FBXO22
PDPK1 CTD PKD1P6 SLC12A4 RPL23A UBA52 RPS11 TMC2 MAFIPL MIS18A-AS1
PNPLA4 GPC3
GOBPID Pvalue OddsRatio ExpCount Count Size Term
GO:0006415 1.13924126257327e-28 164.716216216216 0.232994256452841
17 91 translational termination
GO:0006414 1.59539864162419e-27 138.376136363636 0.268839526676355
17 105 translational elongation
GO:0006614 1.59539864162419e-27 138.376136363636 0.268839526676355
17 105 SRP-dependent cotranslational protein targeting to membrane
GO:0006613 1.89763757700381e-27 136.811797752809 0.271399903120891
17 106 cotranslational protein targeting to membrane
GO:0045047 1.89763757700381e-27 136.811797752809 0.271399903120891
17 106 protein targeting to ER
GO:0072599 1.89763757700381e-27 136.811797752809 0.271399903120891
17 106 establishment of protein localization to endoplasmic reticulum
GO:0019080 1.16101996113734e-26 101.724574594381 0.386616843125043
18 151 viral genome expression
GO:0019083 1.16101996113734e-26 101.724574594381 0.386616843125043
18 151 viral transcription
GO:0070972 1.3368781209105e-26 120.455940594059 0.302124420455332
17 118 protein localization to endoplasmic reticulum
GO:0000184 1.55763730660219e-26 119.266666666667 0.304684796899869
17 119 nuclear-transcribed mRNA catabolic process, nonsense-mediated decay
GO:0006413 1.25360192660306e-24 89.9048148148148 0.38917721956958
17 152 translational initiation
GO:0006612 1.40844113849234e-24 89.2375 0.391737596014117 17 153
protein targeting to membrane
GO:0043624 1.40844113849234e-24 89.2375 0.391737596014117 17 153
cellular protein complex disassembly
GO:0043241 2.49139715694258e-24 86.0429078014184 0.4045394782368 17
158 protein complex disassembly
GO:0000956 8.18727855160578e-24 79.7546052631579 0.432703619126704
17 169 nuclear-transcribed mRNA catabolic process
GO:0034623 1.3685780068508e-23 77.1875796178344 0.445505501349388
17 174 cellular macromolecular complex disassembly
GO:0006402 2.04134419859622e-23 75.2487577639751 0.455747007127534
17 178 mRNA catabolic process
GO:0032984 2.25257810251158e-23 74.779012345679 0.458307383572071 17
179 macromolecular complex disassembly
GO:0019058 3.21621458870359e-23 63.1623424759081 0.59144695868798
18 231 viral infectious cycle
GO:0072594 2.22050743212406e-22 64.6681818181818 0.522316794685489
17 204 establishment of protein localization to organelle
GO:0006401 2.41809254046337e-22 64.3196808510638 0.524877171130026
17 205 RNA catabolic process
GO:0022415 2.81584247458275e-22 55.479773814702 0.665697875579545 18
260 viral reproductive process
GO:0071845 6.73842211348502e-20 45.037265917603 0.727146910248426 17
284 cellular component disassembly at cellular level
GO:0022411 8.56072118352137e-20 44.359963099631 0.737388416026573 17
288 cellular component disassembly
GO:0016032 5.77770676639793e-18 30.5892792026255 1.15472977648606
18 451 viral reproduction
GO:0033365 3.72077288339009e-16 26.018201754386 1.21105805826586 17
473 protein localization to organelle
GO:0006605 3.8539867726553e-16 25.959409190372 1.2136184347104 17 474
protein targeting
GO:0022613 1.39806468068982e-06 15.194495412844 0.57608470002076 7
225 ribonucleoprotein complex biogenesis
GO:0042254 1.9398878296968e-06 19.3155876381683 0.38149609023597
6 149 ribosome biogenesis

GO:0071843	2.09082168711077e-06	14.2635057471264	0.611929970244274
7	239	cellular component biogenesis at cellular level
GO:0042273	3.34571139845119e-06	141.225490196078	0.0307245173344405
3	12	ribosomal large subunit biogenesis
GO:0006364	6.3609947487871e-06	22.5931186868687	0.266279150231818
5	104	rRNA processing
GO:0016072	8.37237154329821e-06	21.2931547619048	0.281641408899038
5	110	rRNA metabolic process
GO:0042274	1.22788070669489e-05	84.7	0.0460867760016608	3	18
ribosomal small subunit biogenesis
Tissue: Muscle_Skeletal=>Lung	Type:	asymmetric
SourceGene:	SDS(ENSG00000135094.4)
TargetGeneSet:	VPS45	RPS7	RPL15	CTB	EEF1A1P6	CTA	TRBV7-8 MYPN
RPS3AP5 FBXL15	RPL27A	RP11	C11orf31	CTD	NME3	RPL13	RPL26
MRPL34	RPL13A	NOSIP
GOBPID	Pvalue	OddsRatio	ExpCount	Count	Size	Term
GO:0006415	4.01611449165716e-10	101.294117647059	0.100754273060688
6	91	translational termination
GO:0006414	9.61586459350121e-10	86.8848484848485	0.11625493045464
6	105	translational elongation
GO:0006614	9.61586459350121e-10	86.8848484848485	0.11625493045464
6	105	SRP-dependent cotranslational protein targeting to membrane
GO:0006613	1.01867392935224e-09	86.01	0.117362120268494	6	106
cotranslational protein targeting to membrane
GO:0045047	1.01867392935224e-09	86.01	0.117362120268494	6	106
protein targeting to ER
GO:0072599	1.01867392935224e-09	86.01	0.117362120268494	6	106
establishment of protein localization to endoplasmic reticulum
GO:0070972	1.95364559544598e-09	76.7303571428571	0.130648398034738
6	118	protein localization to endoplasmic reticulum
GO:0000184	2.05615156802194e-09	76.046017699115 0.131755587848592	6
119	nuclear-transcribed mRNA catabolic process, nonsense-mediated decay
GO:0019080	8.65497447098544e-09	59.1310344827586	0.167185661891911
6	151	viral genome expression
GO:0019083	8.65497447098544e-09	59.1310344827586	0.167185661891911
6	151	viral transcription
GO:0006413	9.00527408901662e-09	58.7219178082192	0.168292851705764
6	152	translational initiation
GO:0006612	9.36723534542493e-09	58.3183673469388	0.169400041519618
6	153	protein targeting to membrane
GO:0043624	9.36723534542493e-09	58.3183673469388	0.169400041519618
6	153	cellular protein complex disassembly
GO:0043241	1.13628486543256e-08	56.3802631578947	0.174935990588887
6	158	protein complex disassembly
GO:0000956	1.70119170987948e-08	52.5349693251534	0.187115078541277
6	169	nuclear-transcribed mRNA catabolic process
GO:0034623	2.0256353412196e-08	50.9535714285714	0.192651027610546
6	174	cellular macromolecular complex disassembly
GO:0006402	2.32062780153623e-08	49.7546511627907	0.197079786865961
6	178	mRNA catabolic process
GO:0032984	2.39967536614885e-08	49.4635838150289	0.198186976679815
6	179	macromolecular complex disassembly
GO:0072594	5.23432551511703e-08	43.1424242424242	0.225866722026157
6	204	establishment of protein localization to organelle
GO:0006401	5.38891409876322e-08	42.9226130653266	0.226973911840011
6	205	RNA catabolic process
GO:0019058	1.09526014563922e-07	37.8933333333333	0.255760847000208
6	231	viral infectious cycle
GO:0022415	2.20453366736355e-07	33.4984251968504	0.287869351601965
6	260	viral reproductive process
GO:0071845	3.70908453898079e-07	30.5546762589928	0.314441907134454
6	284	cellular component disassembly at cellular level
GO:0022411	4.02707066625748e-07	30.1127659574468	0.318870666389869

6	288	cellular component disassembly
GO:0016032	5.48524295696051e-06	18.8629213483146	0.499342606048024
6	451	viral reproduction
GO:0033365	7.21412751227361e-06	17.9460385438972	0.523700781952806
6	473	protein localization to organelle
GO:0006605	7.30218217842615e-06	17.9064102564103	0.52480797176666
6	474	protein targeting
Tissue: Lung=>Muscle_Skeletal	Type:	cluster
SourceGene:	TTC12(ENSG00000149292.11)
TargetGeneSet:	RHD	MACF1	GPBP1L1	DIEXF	ATAD2B	LGALSL	SLC1A4	MAP4K4
SS18L2	FAM198A	ARF4	RP11	TBL1XR1	TNIP2	RELL1	NFKB1	PPP1R14BP3
CLPTM1L	NLN	PIK3R1	ANKRD32	HBEGF	E2F3	FKBP5	APOBEC2	CENPQ	FKBP1C
HDAC2	RP3	HIVEP2	PDGFA	HOXA5	IGFBP3	YWHAG	RBM48	AZIN1	OPLAH
TTLL11	PRRC2B	WDR5	UBAC1	ARHGAP21		ABI1	ARID5B	TSPAN15	FUT11
PANK1	C10orf2	PCGF6	TEAD1	TTC12	FAIM2	PCBP2	KCTD10	SACS	SHISA2
FOXO1	GRTP1	PARP2	STRN3	TJP1	HIC1	PER1	PMP22	AKAP10	UBE2O
NPTX1	TSHZ3	B4GALT5	PPM1F	TMEM184B		CSNK1E	PDGFB	RBBP7
GOBPID	Pvalue	OddsRatio		ExpCount		Count	Size	Term
Tissue: Lung=>Muscle_Skeletal	Type:	cluster
SourceGene:	ZNF673(ENSG00000147121.10)
TargetGeneSet:	ACAP3	ALPL	ZBTB40	HMGN2	SNRNP40	UROD	RP11	HAX1
C1orf112		GS1	LINC00467		INTS7	CCDC85A	TIA1	MKI67IP	WDR75
MRPS36P1		SLC22A14		TMEM42	MON1A	RUVBL1	PLS1	ACAP2	TM4SF19
TMA16	CTD	GEMIN5	NUDCD2	B4GALT7	C6orf62	BYSL	MMS22L	MICAL1	FAM126A
GNAI1	SLC4A2	PABPC1	ZNF706	NOL3	C8orf55	KIAA0020		NOL6	SWI5
USP20	EXOSC2	RBM17	AFAP1L2	KCNC1	NAT10	POLR2G	ESRRA	MAP4K2	MUS81
TCIRG1	NEU3	CLNS1A	SIDT2	PVRL1	CLEC2D	LOH12CR1		DTX3	MDM1
RIC8B	ISCU	MAP1LC3B2		TRMT5	DCAF4	SMEK1	HMGN2P5	CHRNA7	HEXA	NMB
AEN	SBK1	RABEP2	RRAD	SLC43A2	DPH1	CTDNEP1	GPS2	TP53I13	TUBG1
UBE2O	AFMID	HNRNPA1P7		CCDC68	ANKRD24	DCAF15	SAMD1	SNRPA	KCNJ14	CD37
ZNF628	ZNF324B	C20orf72		NAA20	HM13-AS1		RBL1	C20orf132
FAM210B	SYCP2	ZGPAT	SETD4	DGCR8	HMOX1	NCF4	ZC4H2	PIN4	UTP14A
GOBPID	Pvalue	OddsRatio		ExpCount		Count	Size	Term
GO:0008380	7.88259161784815e-06	6.7049735348815	1.72105736627223	10
323	RNA splicing
GO:0000377	1.25598439456655e-05	8.3868677905945	1.08698359975088	8
204	RNA splicing, via transesterification reactions with bulged adenosine as nucleophile
GO:0000398	1.25598439456655e-05	8.3868677905945	1.08698359975088	8
204	nuclear mRNA splicing, via spliceosome
GO:0000375	1.4973605780224e-05	8.17535510851539	1.11362535464674
8	209	RNA splicing, via transesterification reactions
GO:0034660	1.9847296795506e-05	6.76054987212276	1.51858002906373
9	285	ncRNA metabolic process
Tissue: Lung=>Nerve_Tibial	Type:	cluster
SourceGene:	ACSL3(ENSG00000123983.9)
TargetGeneSet:	CEP104	UBR4	HMGCL	GBP7	HIPK1	CRTC2	ATP1A4	TARBP1
ITSN2	SMPD4	PKP4	RAX	RP11	THAP4	SETD5	CHCHD4	C3orf75	DHX30	NEK4
CGGBP1	RPL7AP11		UBQLN4P1		NDUFB5	WHSC2	CCNI	WWC2	CTD
SPATA9	CTB	TCOF1	ZNF300	ATOX1	UBLCP1	RP3	BRD2	C6orf106
FOXP4	FRS3	HMGN3	SMPDL3A	TAB2	FBXO5	LSM5	GSTK1	KAT6A	FAM82B
ZNF572	EIF2C2	FAM83H	CCL19	ATP8B5P	PHF2	ODF2	PRRC2B	UCK1	EHMT1	WAC
PARD3	PPRC1	NOLC1	MXI1	ZRANB1	CTBP2	PPP2R2D	PHRF1	NUP98	ZNF143
SPTY2D1	INCENP	NXF1	SNX15	MRPL49	SLC25A45		FIBP	SART1	KLC2
KDM2A	CLNS1A	7SK	PCSK7	ZNF384	ATN1	CCDC91	DDX23	KCNH3	PHC1P1
PRDM4	GCN1L1	EP400	A2LD1	RBM23	GSTZ1	IFI27L1	DENND4A	SIN3A	AP3B2
AXIN1	MAPK8IP3		RNPS1	CREBBP	NOMO1	NOMO3	SETD1A	ZC3H18	LINC00304
RP13	FOXO3B	SOCS7	NT5C3L	ANKRD40	MAP3K3	ABCA10	SYNGR2	FOXK2	ZNF625
CHERP	FAM187B	SUPT5H	MAP3K10	ZNF574	ZNF526	CLPTM1	CLASRP	TRAPPC6A
SCAF1	ZNF473	ZNF71	NCOA5	ZBP1	ADRM1	SCAF4	C21orf67		EP300	SBF1
GAPDHP1	RPS2P55	DDX3X	RP6	TIMP1	FOXO4	IDS
GOBPID	Pvalue	OddsRatio		ExpCount		Count	Size	Term
GO:0006397	4.88462197370097e-07	5.56202168367347	3.1199916960764	15

399	mRNA processing
Tissue: Lung=>Nerve_Tibial	Type:	asymmetric
SourceGene:	ENSG00000211482.1
TargetGeneSet:	TMEM234 LAMTOR2 RP11	TMSB10	CAPG	CHRNA1	UBE2E3	SPCS1	PDHB
UQCRQ	ATOX1	PTTG1	GCNT2	DEFA6	FAM54A	RP1	NDUFA4	CUL1	TAF5
PHRF1	MIR611	KLC2	REP15	ATP5G2	RPL41	SLC16A7 GCN1L1	EP400	GOLGA3
LPAR6	LRFN5	NPC2	KCNK13	KLHL25	CTD	PKD1P6	MLLT6	FOXK2	RP13
COX6B1	POLR2I	MAP3K10 NDUFA3	SNRPB2	RP4	ROMO1	CTSZ	MIR647	TTLL1	SBF1
DDX3X	CXorf26 NDUFA1
GOBPID	Pvalue	OddsRatio	ExpCount	Count	Size	Term
GO:0045333	2.53312645571106e-07	19.4730994152047	0.442183931907826
7	142	cellular respiration
GO:0015980	4.12017193659578e-06	10.2012112446895	0.955989204899315
8	307	energy derivation by oxidation of organic compounds
GO:0022904	1.15325666764779e-05	19.6634615384615	0.298941249740502
5	96	respiratory electron transport chain
Tissue: Nerve_Tibial=>Lung	Type:	asymmetric
SourceGene:	ENSG00000223461.1
TargetGeneSet:	HS6ST1P1	STMN1	KIF2C	ATRX	IQGAP3	TNFSF13B	RRM2
SLC5A6	HMGN2P21	NCAPH	BUB1	CKAP2L	C2orf27B	FABP5P10
ENO1P4	MYL3	CACNA1D CCDC66	RP11	SPSB4	SIAH2	MSANTD1 NCAPG	EDNRA
NEIL3	ZNF131	CDC25C	RP1	KIFC1	APOBEC2 LAMA4	BTF3L4P3	SAMD9L
DNAJB9	NCAPG2	PBK	PHF19	KIF11	HOGA1	E2F8	FAT3	RAD51AP1
CDCA3	WBP11	ESPL1	DLGAP5	RAD51	KIAA0101	ANP32BP1	HMGN2P3
CES1P1	HERPUD1 CENPN	FANCA	AURKB	C17orf67	RPS10L	GINS1	TPX2
CPT1B
GOBPID	Pvalue	OddsRatio	ExpCount	Count	Size	Term
GO:0000280	5.22653288661906e-14	21.7272259392692	1.0656701958342 15
350	nuclear division
GO:0007067	5.22653288661906e-14	21.7272259392692	1.0656701958342 15
350	mitosis
GO:0000087	7.89044901168891e-14	21.0824587706147	1.09611791571518
15	360	M phase of mitotic cell cycle
GO:0048285	1.17693520690794e-13	20.4740165128703	1.12656563559615
15	370	organelle fission
GO:0051301	6.44458844443446e-09	11.9036931818182	1.37623693862016
12	452	cell division
GO:0007059	8.38176051439041e-09	24.0319865319865	0.426268078333679
8	140	chromosome segregation
GO:0000070	3.57368219700349e-07	41.8502331002331	0.149193827416788
5	49	mitotic sister chromatid segregation
GO:0000819	4.83790765365382e-07	39.1707583196945	0.158328143381081
5	52	sister chromatid segregation
GO:0051983	7.59729081247084e-07	71.935	0.073074527714345	4	24
regulation of chromosome segregation
GO:0007017	2.26535970998001e-06	9.39040178571429	1.1965953913224 9
393	microtubule-based process
GO:0007052	2.86790487866996e-06	49.5793103448276	0.100477475607224
4	33	mitotic spindle organization
GO:0007051	4.20160213903285e-06	24.4991452991453	0.243581759047817
5	80	spindle organization
GO:0000226	1.52310376407441e-05	10.2140499277904	0.819043664798284
7	269	microtubule cytoskeleton organization
Tissue: Nerve_Tibial=>Lung	Type:	asymmetric
SourceGene:	FOLR4(ENSG00000183560.4)
TargetGeneSet:	DDX11L1 PAQR7	DMBX1	CMPK1	GNG5	S100A10 TCEB1P21	GNLY
CCDC72	RP11	LINC00578	HNRPDL	MRPS36	CTB	PPIA	ZNF92	FIS1
TRBV5-6 GTF2E2	COX6C	ATP6V1C1	CHMP5	PCDH15	TMEM9B	ATP5L	KLRB1
HEBP1	GYS2	MYL6	COX6A1	ACIN1	PPP2R5C SRP14	MGA	NDUFAB1 RPL27
SUMO2	MYL12A	MYL12B	C18orf32	NDUFA13 PPIAP22 ATP5J	SEC14L3 COX7B
ZBTB33
GOBPID	Pvalue	OddsRatio	ExpCount	Count	Size	Term
GO:0022904	4.87906245786462e-09	36.3421529539688	0.252439277558646

7	96	respiratory electron transport chain
GO:0022900	5.00125557113411e-08	25.4005588011176	0.352363158258944
7	134	electron transport chain
GO:0015980	7.43733814647176e-08	14.6997222865078	0.807279773026088
9	307	energy derivation by oxidation of organic compounds
GO:0045333	7.46046853932828e-08	23.8819593787336	0.373399764722165
7	142	cellular respiration
GO:0006091	1.22530510802485e-06	10.3650728335116	1.1254584457823 9
428	generation of precursor metabolites and energy
Tissue: Lung=>Nerve_Tibial	Type:	asymmetric
SourceGene:	GFAP(ENSG00000131095.5)
TargetGeneSet:	UTP11L	ANKRD13C	EVI5	PFDN2	MRPL33	C1D	SUGT1P2
SEMA3B	UBA3	ATG3	RP11	IL12A	SUB1	TAF9	FAM174A	UQCRQ	CTB	TUBB
RANP1	COX7A2	PSMB1	RPA3	NDUFA4	MALSU1	HUS1	TRIM56	FIS1	PSMC2
DCTN6	COX6C	TTC35	EXOSC4	PRSS3	DCTN3	PSMB7	ATP5C1	FAM149B1
SNORA19	NDUFS8	CWF19L2	MRP63	POMP	MZT1	L2HGDH	SNORA70	PSMA3	ERH
GTF2A2	COX5A	DNAJA4	PSMA4	MRPL28	NFATC3	SMYD4	PSMB6	TXNDC17	DHRS7C
PSMB3	AATK	IER3IP1	SEC11C	UQCR11	NFIC	PIN1	UBE2S	TGM3	SNRPB2
ZMAT5	CCDC160
GOBPID	Pvalue	OddsRatio	ExpCount	Count	Size	Term
GO:0051443	2.37160466144218e-11	37.5686388807928	0.317763476576016
9	82	positive regulation of ubiquitin-protein ligase activity
GO:0051351	3.67800011272365e-11	35.6070737772865	0.333264133969967
9	86	positive regulation of ligase activity
GO:0051438	1.00693999163763e-10	31.492296404989 0.372015777454847	9
96	regulation of ubiquitin-protein ligase activity
GO:0051340	1.46005668686652e-10	30.0996025251344	0.387516434848799
9	100	regulation of ligase activity
GO:0000216	6.3792202051005e-10	33.6244131455399	0.306137983530551
8	79	M/G1 transition of mitotic cell cycle
GO:0031575	8.63789621157269e-10	32.2545045045045	0.317763476576016
8	82	mitotic cell cycle G1/S transition checkpoint
GO:0031145	9.5309813187546e-10	31.8222222222222	0.321638640924504
8	83	anaphase-promoting complex-dependent proteasomal ubiquitin-dependent protein catabolic process
GO:0031398	1.25636639401995e-09	23.1685899747566	0.492145872257975
9	127	positive regulation of protein ubiquitination
GO:0071779	1.27078945669152e-09	30.5918803418803	0.333264133969967
8	86	G1/S transition checkpoint
GO:0006521	2.02662410128543e-09	40.9857142857143	0.220884367863816
7	57	regulation of cellular amino acid metabolic process
GO:2000045	3.94522660797564e-09	26.1978021978022	0.383641270500311
8	99	regulation of G1/S transition of mitotic cell cycle
GO:0006977	5.8120202579892e-09	34.7118644067797	0.255760847000208
7	66	DNA damage response, signal transduction by p53 class mediator resulting in cell cycle arrest
GO:0072413	5.8120202579892e-09	34.7118644067797	0.255760847000208
7	66	signal transduction involved in mitotic cell cycle checkpoint
GO:0072431	5.8120202579892e-09	34.7118644067797	0.255760847000208
7	66	signal transduction involved in mitotic cell cycle G1/S transition DNA damage checkpoint
GO:0072474	5.8120202579892e-09	34.7118644067797	0.255760847000208
7	66	signal transduction involved in mitotic cell cycle G1/S checkpoint
GO:0051436	6.47080923363157e-09	34.1309523809524	0.259636011348696
7	67	negative regulation of ubiquitin-protein ligase activity involved in mitotic cell cycle
GO:0072401	6.47080923363157e-09	34.1309523809524	0.259636011348696
7	67	signal transduction involved in DNA integrity checkpoint
GO:0072404	6.47080923363157e-09	34.1309523809524	0.259636011348696
7	67	signal transduction involved in G1/S transition checkpoint
GO:0072422	6.47080923363157e-09	34.1309523809524	0.259636011348696
7	67	signal transduction involved in DNA damage checkpoint
GO:0072395	7.19193217728347e-09	33.5690866510539	0.263511175697184

7	68	signal transduction involved in cell cycle checkpoint
GO:0000209	1.02305678494934e-08	17.9433090705487	0.623901460106567
9	161	protein polyubiquitination
GO:0033238	1.07991473500473e-08	31.4945054945055	0.279011833091136
7	72	regulation of cellular amine metabolic process
GO:0051352	1.07991473500473e-08	31.4945054945055	0.279011833091136
7	72	negative regulation of ligase activity
GO:0051437	1.07991473500473e-08	31.4945054945055	0.279011833091136
7	72	positive regulation of ubiquitin-protein ligase activity involved in mitotic cell cycle
GO:0051444	1.07991473500473e-08	31.4945054945055	0.279011833091136
7	72	negative regulation of ubiquitin-protein ligase activity
GO:0031571	1.19090254014592e-08	31.0151515151515	0.282886997439624
7	73	mitotic cell cycle G1/S transition DNA damage checkpoint
GO:0002479	1.44211901233994e-08	30.0987394957983	0.2906373261366 7
75	antigen processing and presentation of exogenous peptide antigen via MHC class I, TAP-dependent
GO:0031396	1.48415781669281e-08	17.1449217181855	0.651027610545983
9	168	regulation of protein ubiquitination
GO:0051439	1.9024701276404e-08	28.8209255533199	0.302262819182064
7	78	regulation of ubiquitin-protein ligase activity involved in mitotic cell cycle
GO:0042590	2.08123048151715e-08	28.4186507936508	0.306137983530551
7	79	antigen processing and presentation of exogenous peptide antigen via MHC class I
GO:0071158	2.2740239072208e-08	28.027397260274 0.310013147879039	7
80	positive regulation of cell cycle arrest
GO:0002478	2.94569981048109e-08	26.9154135338346	0.321638640924504
7	83	antigen processing and presentation of exogenous peptide antigen
GO:0000077	3.41402806989042e-08	19.4986338797814	0.503771365303439
8	130	DNA damage checkpoint
GO:0019884	3.48110141843876e-08	26.2216117216117	0.329388969621479
7	85	antigen processing and presentation of exogenous antigen
GO:0000082	3.59390157517707e-08	15.3818968626037	0.720780568818767
9	186	G1/S transition of mitotic cell cycle
GO:0000084	4.32819461581721e-08	18.8743386243386	0.519272022697391
8	134	S phase of mitotic cell cycle
GO:0031570	4.85946306035468e-08	18.5768229166667	0.527022351394367
8	136	DNA integrity checkpoint
GO:0007093	5.76145194306118e-08	18.147582697201 0.538647844439831	8
139	mitotic cell cycle checkpoint
GO:0031397	6.05117384076545e-08	24.0504201680672	0.356515120060895
7	92	negative regulation of protein ubiquitination
GO:0045333	6.80409505498185e-08	17.7375621890547	0.550273337485295
8	142	cellular respiration
GO:0051320	7.18594164906072e-08	17.6049382716049	0.554148501833783
8	143	S phase
GO:0022904	8.13551220948746e-08	22.9630818619583	0.372015777454847
7	96	respiratory electron transport chain
GO:2000602	8.90378995282056e-08	17.0935251798561	0.569649159227735
8	147	regulation of interphase of mitotic cell cycle
GO:0043161	9.80205424264059e-08	13.5909574468085	0.809909348833991
9	209	proteasomal ubiquitin-dependent protein catabolic process
GO:0002474	1.0070905787803e-07	22.2096273291925	0.383641270500311
7	99	antigen processing and presentation of peptide antigen via MHC class I
GO:0010498	1.29897464201097e-07	13.1248843663275	0.837035499273407
9	216	proteasomal protein catabolic process
GO:0048002	1.83640985175241e-07	20.2178217821782	0.418517749636703
7	108	antigen processing and presentation of peptide antigen
GO:0030330	3.18000147089335e-07	18.5519480519481	0.453394228773095
7	117	DNA damage response, signal transduction by p53 class mediator
GO:0000075	3.40784712969514e-07	11.6389370833714	0.937789772334095

9	242	cell cycle checkpoint
GO:0072331	4.4735319198022e-07	17.5849753694581	0.476645214864023
7	123	signal transduction by p53 class mediator
GO:0071156	7.076942782611e-07	10.6182728410513	1.02304338800083
9	264	regulation of cell cycle arrest
GO:0022900	8.00066537061481e-07	16.0494938132733	0.519272022697391
7	134	electron transport chain
GO:0042770	1.07533269909024e-06	15.3190118152524	0.542523008788319
7	140	signal transduction in response to DNA damage
GO:0007050	1.30476957963186e-06	8.49945500787211	1.42993564459207
10	369	cell cycle arrest
GO:0051329	1.66080666485495e-06	8.26322611052198	1.46868728807695
10	379	interphase of mitotic cell cycle
GO:0090068	1.78711354681157e-06	14.1378968253968	0.585149816621687
7	151	positive regulation of cell cycle process
GO:0019882	1.86781761245699e-06	14.0394088669951	0.589024980970175
7	152	antigen processing and presentation
GO:0051325	2.0040363970074e-06	8.08326605927805	1.49968860286485
10	387	interphase
GO:0010565	2.03840210248302e-06	13.846452866861 0.596775309667151	7
154	regulation of cellular ketone metabolic process
GO:0007346	2.21855020581098e-06	9.18432479374729	1.17417479759186
9	303	regulation of mitotic cell cycle
GO:0015980	2.47100756082141e-06	9.05847493931172	1.18967545498581
9	307	energy derivation by oxidation of organic compounds
GO:0010564	3.66142377567205e-06	7.52851915626345	1.60431804027403
10	414	regulation of cell cycle process
GO:0045786	5.90980336392712e-06	7.11129212911109	1.69344682028925
10	437	negative regulation of cell cycle
GO:0044106	6.39627865973228e-06	11.5413961038961	0.709155075773303
7	183	cellular amine metabolic process
GO:0006511	9.84736623766474e-06	7.57326940365598	1.41055982284963
9	364	ubiquitin-dependent protein catabolic process
GO:0019941	1.14704978264928e-05	7.42312213471259	1.43768597328905
9	371	modification-dependent protein catabolic process
GO:0009308	1.22023885936851e-05	10.4029304029304	0.782783198394575
7	202	amine metabolic process
GO:0043632	1.22332329984429e-05	7.36053628679685	1.44931146633451
9	374	modification-dependent macromolecule catabolic process
GO:0051603	1.67323685365422e-05	7.06243001119821	1.50743893156183
9	389	proteolysis involved in cellular protein catabolic process
Tissue: Nerve_Tibial=>Lung	Type:	asymmetric
SourceGene:	GPRC5A(ENSG00000013588.5)
TargetGeneSet:	YARS	SARS	RP11	HADH	GARS	C9orf9	SRGN	CFL1P1	CARS
KRTAP5-10	KRR1	ITGA11	MPRIP	RAD23A	CYP4F2	SLC1A5	SETD4	TSR2
GOBPID	Pvalue	OddsRatio	ExpCount	Count	Size	Term
GO:0006418	7.49326144892686e-08	145.393939393939	0.0431804027402948
4	48	tRNA aminoacylation for protein translation
GO:0034660	8.52978289875066e-08	43.4992319508449	0.2563836412705 6
285	ncRNA metabolic process
GO:0043038	9.60922540719905e-08	136.085106382979	0.0458791779115632
4	51	amino acid activation
GO:0043039	9.60922540719905e-08	136.085106382979	0.0458791779115632
4	51	tRNA aminoacylation
GO:0006399	3.59118375176098e-06	52.5876951331497	0.112448965469518
4	125	tRNA metabolic process
Tissue: Lung=>Nerve_Tibial	Type:	asymmetric
SourceGene:	GSTA1(ENSG00000243955.1)
TargetGeneSet:	UQCRH	CSRP3	KCNC4	C1orf54 LHX4	LHX9	RP11	ERLEC1
GPAT2	LYAR	CRMP1	CC2D2A	ATP1B1P1	NDUFS6	TMEM14C RHAG	TMEM120A
PTPN12	PUS7	LINC00032	ATP5C1	NCOA4	IFIT2	MYOF	BBIP1	NDUFS3
MRPL16	NDUFA9	KRR1	FRMD6	CHURC1	TDP1	NDUFB1	WASH3P	HCFC1R1 USP31
ZNF629	COX4I1	SMARCE1 KRT223P PSMG2	COX6B1	RP3	ROMO1	DNTTIP1 MRPL39

VDAC1P2 TCEAL8 NDUFA1
GOBPID Pvalue OddsRatio ExpCount Count Size Term
GO:0022904 3.96471358198034e-12 47.7997775305895 0.265725555324891
9 96 respiratory electron transport chain
GO:0022900 8.28557327429333e-11 33.1803870967742 0.370908587640994
9 134 electron transport chain
GO:0045333 1.39661050307714e-10 31.1671113267039 0.393052383918068
9 142 cellular respiration
GO:0006119 1.94109208173366e-10 59.726678550208 0.160542523008788 7
58 oxidative phosphorylation
GO:0042773 3.0024573107284e-09 61.8507890961263 0.130094803127811
6 47 ATP synthesis coupled electron transport
GO:0042775 3.0024573107284e-09 61.8507890961263 0.130094803127811
6 47 mitochondrial ATP synthesis coupled electron transport
GO:0006120 4.43961605146332e-08 66.2672811059908 0.0996470832468341
5 36 mitochondrial electron transport, NADH to ubiquinone
GO:0015980 1.20183139616916e-07 13.7494046330375 0.849768182132724
9 307 energy derivation by oxidation of organic compounds
GO:0006091 1.95011088415191e-06 9.69497266918161 1.18469310082347
9 428 generation of precursor metabolites and energy
Tissue: Nerve_Tibial=>Lung Type: asymmetric
SourceGene: IGKV1OR2-118(ENSG00000219041.3)
TargetGeneSet: DRAM2 KRT8P29 CNIH4 MAT2A IL1R2 STEAP3 TNNC1 WDR52 CNBP
RP11 LINC00578 TMEM128 PCDHA5 GS1 SGCE PENK C8orf34 RPL8
OR1L8 RPL35 MRPL41 ZNF37BP VDAC2 RPL27A FDX1 ATP5L C12orf57
RIC8B HMGB3P4 TCEB2 RPL19 MYO5B RPL36 EIF3K ZNF814 DPH3P1
GOBPID Pvalue OddsRatio ExpCount Count Size Term
GO:0019080 3.67143006109308e-07 26.8551724137931 0.292574908310844
6 151 viral genome expression
GO:0019083 3.67143006109308e-07 26.8551724137931 0.292574908310844
6 151 viral transcription
GO:0006413 3.81729724848633e-07 26.6693648816936 0.294512490485088
6 152 translational initiation
GO:0006415 7.76820389467076e-07 36.2411526794742 0.176319977856204
5 91 translational termination
GO:0006414 1.58320955019598e-06 31.1369565217391 0.20344612829562
5 105 translational elongation
GO:0006614 1.58320955019598e-06 31.1369565217391 0.20344612829562
5 105 SRP-dependent cotranslational protein targeting to membrane
GO:0006613 1.6593771554176e-06 30.8265174343521 0.205383710469864
5 106 cotranslational protein targeting to membrane
GO:0045047 1.6593771554176e-06 30.8265174343521 0.205383710469864
5 106 protein targeting to ER
GO:0072599 1.6593771554176e-06 30.8265174343521 0.205383710469864
5 106 establishment of protein localization to endoplasmic reticulum
GO:0070972 2.81950870580034e-06 27.5298191612159 0.228634696560792
5 118 protein localization to endoplasmic reticulum
GO:0000184 2.93925737315813e-06 27.2864225781846 0.230572278735036
5 119 nuclear-transcribed mRNA catabolic process, nonsense-mediated decay
GO:0019058 4.38768208778296e-06 17.209696969697 0.447581482250363 6
231 viral infectious cycle
GO:0022415 8.65002609782669e-06 15.213672154617 0.503771365303439 6
260 viral reproductive process
GO:0006612 1.00600473682889e-05 20.9679788484136 0.296450072659332
5 153 protein targeting to membrane
GO:0043624 1.00600473682889e-05 20.9679788484136 0.296450072659332
5 153 cellular protein complex disassembly
GO:0043241 1.17612249258428e-05 20.2756464904802 0.306137983530551
5 158 protein complex disassembly
GO:0000956 1.62952306616806e-05 18.9011134676564 0.327451387447236
5 169 nuclear-transcribed mRNA catabolic process
GO:0034623 1.87599746165927e-05 18.3354772317983 0.337139298318455
5 174 cellular macromolecular complex disassembly

Tissue: Lung=>Nerve_Tibial Type: asymmetric
SourceGene: IGLV4-69(ENSG00000211637.2)
TargetGeneSet: PRDM16 CLCN6 RPL11 BMP8B DOCK7 RP11 SORT1 SLC27A3
C1orf9 RPS6KC1 FIGLA PDCL3 FHL2 SCN7A CRTAP ABHD14B GFM1 KIAA0232
C1QTNF7 SPP1 FNIP2 CTD RPL37 EEF1A1P19 HMGCR PAQR8 EEF1A1
NDUFAF4 PERP PLAGL1 RP4 INSIG1 PHYHIP MRPS35 RPL30 PABPC1 EIF3H
SCRIB GPSM1 MAMDC4 IDI1 SEC61A2 TOLLIP UBXN1 FAU BCRP1 NCAM1
RPL18AP3 AHSA1 MAP1A POLG GAS7 MAPK7 HDHD2 ACAA2 BCL2
LMNB2 PIAS4 EMR4P ELAVL1 PRKCSH RPL18A RPS16 TTYH1 RPL28 RPL24P2
EIF3L SAMM50 XG PNMA5 RPL10

GOBPID	Pvalue	OddsRatio	ExpCount	Count	Size	Term
GO:0006414	1.75572187950827e-11	29.5046439628483	0.443221922358314	10	105	translational elongation
GO:0006413	3.2225444616925e-11	22.2324822695035	0.641616497128226	11	152	translational initiation
GO:0000184	1.55267453698717e-09	22.4685314685315	0.502318178672756	9	119	nuclear-transcribed mRNA catabolic process, nonsense-mediated decay
GO:0006415	4.0524739989832e-09	26.0186406001364	0.384125666043872	8	91	translational termination
GO:0006614	1.26995874179205e-08	22.2415872398366	0.443221922358314	8	105	SRP-dependent cotranslational protein targeting to membrane
GO:0006613	1.3691395478107e-08	22.0130920292645	0.447443083523632	8	106	cotranslational protein targeting to membrane
GO:0045047	1.3691395478107e-08	22.0130920292645	0.447443083523632	8	106	protein targeting to ER
GO:0072599	1.3691395478107e-08	22.0130920292645	0.447443083523632	8	106	establishment of protein localization to endoplasmic reticulum
GO:0006612	1.43143685183038e-08	17.1225961538462	0.645837658293544	9	153	protein targeting to membrane
GO:0070972	3.19308852501457e-08	19.5951972555746	0.498097017507439	8	118	protein localization to endoplasmic reticulum
GO:0000956	3.40335834635391e-08	15.3930288461538	0.71337623693862	9	169	nuclear-transcribed mRNA catabolic process
GO:0006402	5.3308649991659e-08	14.5640646335913	0.751366687426476	9	178	mRNA catabolic process
GO:0072594	1.71549864677873e-07	12.5991124260355	0.861116877724725	9	204	establishment of protein localization to organelle
GO:0006401	1.78842527534319e-07	12.5339481946625	0.865338038890042	9	205	RNA catabolic process
GO:0019080	2.17490132258189e-07	15.0383955666975	0.637395335962909	8	151	viral genome expression
GO:0019083	2.17490132258189e-07	15.0383955666975	0.637395335962909	8	151	viral transcription
GO:0043624	2.40656914726628e-07	14.8288874430709	0.645837658293544	8	153	cellular protein complex disassembly
GO:0043241	3.08040556832121e-07	14.3295597484277	0.66694346412013	8	158	protein complex disassembly
GO:0019058	4.91070992933821e-07	11.045738045738	0.975088229188292	9	231	viral infectious cycle
GO:0034623	6.43090629727571e-07	12.933848601955	0.734482042765207	8	174	cellular macromolecular complex disassembly
GO:0032984	7.9724984921292e-07	12.5512523446982	0.755587848591793	8	179	macromolecular complex disassembly
GO:0022415	1.31819270190311e-06	9.74954030033711	1.09750190298249	9	260	viral reproductive process

Tissue: Lung=>Nerve_Tibial Type: asymmetric
SourceGene: MICF(ENSG00000233265.1)
TargetGeneSet: HSPB11 RP4 NAP1L4P1 C1orf54 ZNF672 PTRHD1 RP11
HACE1 COX6C CTBP2 NDUFS3 MRPL16 ATP5L TBCEL RPSAP51 HIGD1C STAT6
ST6GALNAC4P1 PLEKHG3 IFI27L2 NDUFB10 COX4I1 PVR MAVS ERGIC3 DHRSX

GOBPID	Pvalue	OddsRatio	ExpCount	Count	Size	Term
GO:0022904	6.76611078259766e-08	65.6730769230769	0.112933361013079	5	96	respiratory electron transport chain

GO:0022900	3.59926044765517e-07	46.2047803617571	0.157636149747422
5	134	electron transport chain
GO:0045333	4.80373639711739e-07	43.4823600973236	0.167047263165179
5	142	cellular respiration
Tissue: Nerve_Tibial=>Lung	Type:	asymmetric
SourceGene:	PIGK(ENSG00000142892.10)
TargetGeneSet:	UBE4B	SPEN	ARHGEF10L	RNF11	TTC24	RP11	KRT8P29
RAB4A	FEZ2	MDH1	NFU1	COX5B	KLHL18	CHMP2B	ANKRD17	SLC25A4	CCT6P2
NDUFS4	PTCD2	FAM174A	PHACTR1	CNR1	RN5S216	SEPT7	CHCHD2	EIF4H	FZD1
NDUFA5	METTL2A	CTD	RAB2A	IMPDH1P6	NDUFA8	MRPL41	ATP5C1	ANKRD22
ABCC2	FBXL15	PTPMT1	C11orf31	CPSF7	RAB6A	TMEM123	PHC1	STAT6	AVIL
COX6A1	ZNF605	ACIN1	TSSK6	HMGB1P14	VRK1	SIVA1	SRP14	GANC
RAB11A	MRPL46	FAM173A	NME3	LSMD1	KRT31	AANAT	MYL12B	NDUFV2	IER3IP1
C18orf32	ATP5D	DAPK3	TRAPPC5	ZNF414	ZNF558	RBM42	ZNF428	ZNF284
SIGLEC10	NOP56	SCAND1	ATP5J	DGCR6L	CHCHD10	RAB9A	ZFX	COX7B
GOBPID	Pvalue	OddsRatio	ExpCount	Count	Size	Term
GO:0022904	1.37277815089332e-11	30.2325581395349	0.431804027402948
10	96	respiratory electron transport chain
GO:0045333	3.1726615113612e-11	22.1663839411931	0.63871012386686
11	142	cellular respiration
GO:0022900	3.88793784774641e-10	20.9120234604106	0.602726454916615
10	134	electron transport chain
GO:0006119	6.70860201440995e-09	33.9232589587559	0.260881599889281
7	58	oxidative phosphorylation
GO:0015980	1.00411779914637e-08	10.8149664214902	1.38087329596568
12	307	energy derivation by oxidation of organic compounds
GO:0006091	4.55303983869757e-08	8.41626506024096	1.92512628883814
13	428	generation of precursor metabolites and energy
GO:0009206	7.10485190235733e-06	21.7136363636364	0.269877517126842
5	60	purine ribonucleoside triphosphate biosynthetic process
GO:0009145	7.71248956275583e-06	21.3244047619048	0.274375475745623
5	61	purine nucleoside triphosphate biosynthetic process
GO:0009201	1.05610168824303e-05	19.8972222222222	0.292367310220746
5	65	ribonucleoside triphosphate biosynthetic process
GO:0009205	1.14367548275899e-05	5.8510101010101	2.22648951629645	11
495	purine ribonucleoside triphosphate metabolic process
GO:0009144	1.23331147428029e-05	5.80138129933212	2.24448135077157
11	499	purine nucleoside triphosphate metabolic process
GO:0009142	1.41720677300219e-05	18.6484375	0.310359144695869	5
69	nucleoside triphosphate biosynthetic process
GO:0006120	1.97063151171034e-05	29.4139344262295	0.161926510276105
4	36	mitochondrial electron transport, NADH to ubiquinone
Tissue: Nerve_Tibial=>Lung	Type:	asymmetric
SourceGene:	RAD52(ENSG00000002016.11)
TargetGeneSet:	MRPL20	DFFB	UBE4B	ARHGEF19	CDC42	IFI6	SNHG3	RP11
MSH4	RP4	APH1A	INTS3	UBQLN4	CCT3	FAM78B	BRP44	CSRP1	RAB4A
ADCY3	SLC5A6	MORN2	NFU1	AUP1	RNF181	CHMP3	COX5B	CKAP2L	CCDC115
TMEM194B	SATB2	SPAG16	BCS1L	STK36	CYP8B1	TNNC1	SPCS1	SLC25A26
SUCLG2	CCDC48	CNBP	COL6A5	RNF7	GRK7	NAALADL2-AS2	PARL	RTP4
TMEM128	ANKRD17	TMSB4XP8	PPA2	NDUFC1	C4orf27	CCT6P2	SLC1A3	ZNF131
NDUFS4	FAM174A	CTB	RP1	TMEM14C	TMEM14B	ZDHHC20P1	C6orf130
C6orf226	CNR1	LINC00271	PEX7	AIG1	C6orf72	WDR27	LSM5
CHCHD2	EIF4H	FZD1	SGCE	PILRB	DUS4L	CAV2	NDUFA5	METTL2A	UBN2
ASH2L	C8orf40	PKIA	DECR1	COX6C	POLR2K	NDUFB9	NSMCE2	CHRAC1	CHMP5
MED27	ATP5C1	C1DP1	ZNF37BP	C10orf116	AGAP11	ABCC2	AP2A2	TNNI2
ZNF195	TMEM9B	PLEKHA7	PIGCP1	ALKBH3	CPSF7	PLA2G16	DRAP1	KDM2A	CLNS1A
TMEM123	CARD16	ATM	C11orf1	HYOU1	RAD52	DAZAP2	MYL6	PAN2	LUM
SLC41A2	RIC8B	UBE3B	COX6A1	MLEC	GOLGA3	ZNF605	POMP	SPG20	ACIN1
MAP3K9	POMT2	TRIP11	NDUFB1	IFI27	SRP14	PLA2G4E	WDR61	SRRM2	TCEB2
CLDN9	PKD1P1	NDUFAB1	TNRC6A	SULT1A1	MIR4519	PSMB10	SERPINF2	CTD
NLGN2	CTC1	TEKT3	SLC46A1	HMGB1P24	CNTNAP1	SUPT4H1	MTMR4	SKA2
ICAM2	ATP5H	TBC1D16	MYL12A	MYL12B	C18orf32	MUM1	TRAPPC5	ZNF558
HOOK2	DCAF15	ZNF793	FAM98C	ZNF428	NAPA	SNRNP70	VSIG10L	PPP1R12C

ZSCAN22 DSTN DTD1 CHMP4B RALY PKIG PABPC1L RP3 ZMYND8 SLC17A9
ATP5J SNRPD3 PRR14L ZFX TSR2 ACRC CXorf41 AKAP14 APLN EMD
GOBPID Pvalue OddsRatio ExpCount Count Size Term
GO:0022904 2.38067494063953e-11 15.9031841652324 1.01640024911771
13 96 respiratory electron transport chain
GO:0045333 3.05580499993376e-10 11.1499550359712 1.50342536848661
14 142 cellular respiration
GO:0022900 1.65293176557921e-09 10.8796340023613 1.4187253477268 13
134 electron transport chain
GO:0015980 1.61968757953848e-07 5.62149145910151 3.25036329665767
16 307 energy derivation by oxidation of organic compounds
GO:0006120 1.91302751155676e-06 19.4122448979592 0.381150093419141
6 36 mitochondrial electron transport, NADH to ubiquinone
GO:0006119 2.48576023154465e-06 13.3936341659952 0.614075150508615
7 58 oxidative phosphorylation
GO:0042773 9.58122597442801e-06 14.1931309109009 0.497612621963878
6 47 ATP synthesis coupled electron transport
GO:0042775 9.58122597442801e-06 14.1931309109009 0.497612621963878
6 47 mitochondrial ATP synthesis coupled electron transport
GO:0006091 1.23508777015847e-05 3.93621997023599 4.53145111064978
16 428 generation of precursor metabolites and energy
Tissue: Lung=>Nerve_Tibial Type: asymmetric
SourceGene: RP11-645C24.1(ENSG00000185864.12)
TargetGeneSet: ISG15 NADK CEP104 RP11 WDTC1 IFI6 EYA3 ATPIF1
KHDRBS1 PIGK IFI44 LPPR5 NBPF8 NBPF10 NBPF24 INTS3 CRTC2 DCST2
PFN1P1 MDM4 PUM2 MORN2 GALNT5 BTF3L4P2 SPC25 UBE2E3 TRIP12 ING5
UBE2E1 SPCS1 FTH1P23 RPL7AP11 SNORA4 CCNI BMP2K TMSB4XP8
NDUFC1 C5orf55 RBBP4P1 SUB1 CTD FAM174A UQCRQ TCOF1 C6orf226 PEX6
C6orf108 RP3 NDUFA4 CCDC126 CDK13 GS1 GTF2IRD2B SGCE
PILRB PPP1R35 DUS4L ARF5 RBM28 C7orf49 GSTK1 ZNF862 C8orf59 COX6C
KIAA2026 C9orf133 GKAP1 SEC61B TSC1 TUBBP5 ATP5C1 PBLD DNA2
CALM2P2 FGFBP3 CPSF7 NXF1 SNX15 RCE1 RAD52 GYS2 MED13L GCN1L1 PXN
MLXIP MPHOSPH8 IPO5 ACIN1 DHRS2 CTAGE5 PSMA3 ZNF410 PROX2 NEK9
NDUFB1 IFI27L1 IFI27 PPP1R13B HMGN2P5 TUBGCP4 NPRL3 IFT140 PDXDC1
USP31 ITFG1 C17orf85 TXNDC17 NEURL4 CTC1 FLII EPN2 NPEPPS
ATP5H LLGL2 EPG5 IER3IP1 UQCR11 PIP5K1C INSR ANKLE1 ANKRD27 PPP1R13L
SLC17A7 TBC1D17 SNRPB2 LPIN3 GDAP1L1 BCAS4 SS18L1 LINC00478 H1F0 RP6
TIMP1 MORC4 PGRMC1 NDUFA1 LINC00087 VAMP7
GOBPID Pvalue OddsRatio ExpCount Count Size Term
GO:0022904 1.38635549180869e-08 16.9655172413793 0.637741332779738
9 96 respiratory electron transport chain
GO:0022900 2.53676180473649e-07 11.7765517241379 0.890180610338385
9 134 electron transport chain
GO:0045333 4.15805339285461e-07 11.0619652579725 0.943325721403363
9 142 cellular respiration
GO:0015980 8.65198381291436e-07 6.80871670702179 2.03944363711854
12 307 energy derivation by oxidation of organic compounds
GO:0006091 4.86402676188033e-06 5.26114094933953 2.84326344197633
13 428 generation of precursor metabolites and energy
Tissue: Nerve_Tibial=>Lung Type: asymmetric
SourceGene: RP11-74D7.1(ENSG00000259505.1)
TargetGeneSet: RP11 S100A8 MNDA CCDC85A FHL2 IL1RN ZEB2-AS1
MYD88 LMOD3 HTR1F SSR3 LIMCH1 LST1 C7orf31 MYO1G PILRA LYN SYK
PIK3AP1 CATSPER1 LRRC23 STAC3 GLT1D1 LCP1 RNASE2 TRAV20 AQP9
RPS17L GDE1 TOX3 CTU2 CENPBD1 BECN1 MEOX1 CD300E MIR1227 C19orf38
TYROBP FPR2 NLRP12 VSTM1 OSCAR LILRA1 HCK IFNGR2 IGLV3-16 CYBB
FTH1P8
GOBPID Pvalue OddsRatio ExpCount Count Size Term
GO:0006954 6.28500911262047e-07 11.5604304635762 1.05501349387586
9 462 inflammatory response
GO:0031663 1.2605373894478e-06 62.0086206896552 0.0822088436786382
4 36 lipopolysaccharide-mediated signaling pathway
GO:0071216 3.21972438124354e-06 26.3641384388807 0.23292505057089475

5 102 cellular response to biotic stimulus
GO:0002757 1.33033524414724e-05 13.7690441533236 0.536641062902221
6 235 immune response-activating signal transduction
GO:0002764 1.80564638741489e-05 13.0174471992654 0.566327589786174
6 248 immune response-regulating signaling pathway
Tissue: Nerve_Tibial=>Lung Type: asymmetric
SourceGene: RPL23AP80(ENSG00000213035.3)
TargetGeneSet: AGTRAP EFHD2 SH3BGRL3 GBP1 GBP4 FCRL6 RAB7L1
OR14L1P ARHGAP25 SHISA5 ZBTB20-AS4 MYLK-AS2 LRRC33 CD38
CXCL9 CXCL10 HERC3 LRBA ST8SIA4 STC2 GMNN UBD HLA-F HCG4P11 HCP5
PSMB8 PSMB9 TAP1 RSPO3 HIVEP2 TRGC2 RP11 DENND4C SH2D3C C9orf167
MAP4K2 PLXNC1 DGKZP1 LCP1 PSME1 GZMH GZMB RTN1 CCDC88C WARS RYR3
B2M POLR2M CILP IL32 CORO1A NLRC5 CDH5 PSMB10 PFN1 TRPV2 CCL5
ICAM2 BBC3 MED25 KIR2DL3 ITGB2 TYMP NUDT10
GOBPID Pvalue OddsRatio ExpCount Count Size Term
GO:0002479 9.70439617785782e-09 32.0684143222506 0.275067469379282
7 75 antigen processing and presentation of exogenous peptide antigen via MHC class I, TAP-dependent
GO:0042590 1.40153348417612e-08 30.2783816425121 0.289737734412843
7 79 antigen processing and presentation of exogenous peptide antigen via MHC class I
GO:0002478 1.985123929517e-08 28.6767734553776 0.304407999446405
7 83 antigen processing and presentation of exogenous peptide antigen
GO:0019884 2.34678722332923e-08 27.9375696767001 0.311743131963186
7 85 antigen processing and presentation of exogenous antigen
GO:0002474 6.80660493184403e-08 23.6630434782609 0.363089059580652
7 99 antigen processing and presentation of peptide antigen via MHC class I
GO:0048002 1.24320184663247e-07 21.5408953938872 0.396097155906166
7 108 antigen processing and presentation of peptide antigen
GO:0019882 1.27460005119315e-06 14.9581709145427 0.557470071275344
7 152 antigen processing and presentation
GO:0034341 3.068198791441e-06 17.3775075987842 0.407099854681337
6 111 response to interferon-gamma
GO:0000216 1.00390326144296e-05 20.1632882882883 0.289737734412843
5 79 M/G1 transition of mitotic cell cycle
Tissue: Nerve_Tibial=>Lung Type: asymmetric
SourceGene: RPL7AP11(ENSG00000242445.1)
TargetGeneSet: WASH7P TAS1R3 AKR7A2 NBPF8 FCRL5 LPIN1 IGKJ5 IGKC
IGKJ4 IGKJ2 IGKJ1 IGKV4-1 IGKV3-7 IGKV3-15 IGKV2-24 IGKV2-28
IGKV2-30 IGKV1-33 IGKV3D-11 HAT1 ACSL3 CGGBP1 LRRC34 IGJ
VTI1BP2 RP11 PCDHAC1 MUM1 FILIP1 GABRR2 NT5DC1 CLDN15 TPD52 PVT1 GLDC
LCN10 BMI1 ZNF32-AS2 IGHG2 IGHGP IGHJ1 KIAA0125 HERC2P3
MRPL28 RPS12P27 CD79B JSRP1 CD79A FER1L4 CLDN14 IGLV1-36
IGLV3-16 IGLV3-12 IGLC2 DERL3 SAMM50 PIM2
GOBPID Pvalue OddsRatio ExpCount Count Size Term
GO:0006958 5.39127483595445e-08 63.8933333333333 0.103799045048786
5 50 complement activation, classical pathway
GO:0006959 1.60955212150291e-07 30.8297413793103 0.253269669919037
6 122 humoral immune response
GO:0006956 1.7552478587324e-07 49.5275862068966 0.13078679676147
5 63 complement activation
GO:0002455 2.05671299082553e-07 47.87 0.134938758563421 5 65
humoral immune response mediated by circulating immunoglobulin
GO:0072376 6.62919932256574e-07 37.2571428571429 0.170230433880008
5 82 protein activation cascade
GO:0002253 2.0475684111775e-06 14.7264740917213 0.620718289391738
7 299 activation of immune response
GO:0016064 2.98603796326027e-06 27.0094339622642 0.230433880008304
5 111 immunoglobulin mediated immune response
GO:0019724 3.40548408730857e-06 26.2605504587156 0.236661822711231
5 114 B cell mediated immunity
GO:0050778 7.49754686067186e-06 11.9897698209719 0.755657047955159

7	364	positive regulation of immune response
Tissue: Lung=>Nerve_Tibial	Type:	asymmetric
SourceGene:	SLC1A2(ENSG00000110436.6)
TargetGeneSet:	PODN	PRKAA2	RP4	ANKRD13C	MLLT11	MEX3A	UBXN2A	C1D
DGUOK	SSFA2	HSPE1	TIMP4	SUGT1P2	RP11	PRICKLE2	ATG3	ANXA3
ENOPH1	MRPS18C	SNCA	RAP1GDS1	CISD2	UGT8	CTD	RNF130	TUBB	MNF1
MOCS1	COX7A2	RP1	QKI	SFT2D1	RPA3	NDUFA4	FIS1	FAM3C	PUF60
EXOSC4	PSMC3	POLR2G	SPCS2	ANGPTL5	ZBTB16	SIDT2	FEZ1	MRPL51	ACVR1B
UBE2N	C12orf76	COX6A1	MRP63	POMP	GTF2F2	MZT1	DAD1	MRPL52
PSME2	PSMA3	RN5S392	GATM	GTF2A2	FAM96B	IL34	GLOD4	PSMB6	TXNDC17
TRAPPC1	DHRS7C	PSMB3	STAT5A	ICT1	SEC11C	NFIC	PIN1	FDX1L	STX10
UBE2S	ISOC2	SNRPB2	DYNLRB1	MRPS6	ZMAT5	APOO
GOBPID	Pvalue	OddsRatio	ExpCount	Count	Size	Term
GO:0051443	7.56359909178388e-09	23.7904365904366	0.41422738910802
8	82	positive regulation of ubiquitin-protein ligase activity
GO:0051351	1.10808786561741e-08	22.5641025641026	0.43443360321085
8	86	positive regulation of ligase activity
GO:0051438	2.66028170384164e-08	19.986013986014	0.484949138467926	8
96	regulation of ubiquitin-protein ligase activity
GO:0051340	3.67391571978226e-08	19.1117056856187	0.505155352570756
8	100	regulation of ligase activity
GO:0031396	1.60633534296587e-07	12.575766509434	0.848660992318871	9
168	regulation of protein ubiquitination
GO:0031398	2.37452569917641e-07	14.7475113122172	0.641547297764861
8	127	positive regulation of protein ubiquitination
GO:0000216	2.80051382406854e-06	17.5485585769781	0.399072728530898
6	79	M/G1 transition of mitotic cell cycle
GO:0031145	3.74283272588697e-06	16.6322930800543	0.419278942633728
6	83	anaphase-promoting complex-dependent proteasomal ubiquitin-dependent protein catabolic process
GO:0006521	9.77125334533065e-06	20.2573529411765	0.287938550965331
5	57	regulation of cellular amino acid metabolic process
GO:0010565	1.23102059570465e-05	10.2676767676768	0.777939242958965
7	154	regulation of cellular ketone metabolic process
GO:0000209	1.64392722055561e-05	9.79614325068871	0.813300117638918
7	161	protein polyubiquitination
Tissue: Nerve_Tibial=>Lung	Type:	asymmetric
SourceGene:	THNSL1(ENSG00000185875.8)
TargetGeneSet:	ISG15	SSU72	ATP13A2	IFI6	SDC3	YBX1	IFI44L	IFI44	GBP4
GABPB1	ZNF687	RNF187	CMPK2	SRSF7	UBE2E3	FGD5	SHISA5	RP11	RNF7	KLF3
KCNN2	PCYOX1L	NRM	PSMB8	TAP1	C6orf106	FGFR1OP	PSMB1	WIPI2	FIS1
PARP12	PTK2	LY6E	C9orf167	SEPHS1	GHITM	IFIT3	ACTR1A	RAB1B
NDUFS8	FDX1	ARHGEF12	NTN4	TFCP2	PAN2	SLC25A3	OAS3	OAS2
COX6A1	FBRSL1	ZNF219	ANKRD9	PSMA4	CTD	MRPL28	TCEB2	SBK1	ZNF267
CX3CL1	PSMB10	CHMP1A	GIT1	GJD3	SUPT4H1	TBX2	ATP5A1	PTBP1	UQCR11	AES
UBXN6	ZNF358	TRAPPC5	MAP2K7	TTC9B	EPN1	RBM39	SCAND1	STAU1	OGFR
ATP5J	MX1	SUMO3	DGCR2	ZMAT1	RP13
GOBPID	Pvalue	OddsRatio	ExpCount	Count	Size	Term
GO:0060337	1.12793285414446e-07	21.7022751895991	0.388623624662653
7	72	type I interferon-mediated signaling pathway
GO:0071357	1.12793285414446e-07	21.7022751895991	0.388623624662653
7	72	cellular response to type I interferon
GO:0034340	1.24219943078799e-07	21.3719590268886	0.39402117500519
7	73	response to type I interferon
Tissue: Nerve_Tibial=>Lung	Type:	asymmetric
SourceGene:	UBE2S(ENSG00000108106.8)
TargetGeneSet:	MTND1P23	MTND2P28	DVL1	RP11	JTB	BCAN
ATP1A2	MIR4426	ST13P19	ANKS1B	CTNNA2	C2orf77	ATP2B2	ARPP21	MOBP	RPL7AP31
H3F3AP6	CHCHD2P2	CTD	LIX1	RAB24	PPIAP29	MOG	7SK	EEF1A1P6
CDK6	STMN2	TG	AQP7P3	GRIN3A	TRAF2	OPALIN	COX15	INA	GPR123
SLC1A2	C11orf31	PPP2R5B	CXCR5	KIF5A	LPCAT4	IQGAP1	HBA1	NME3
CACNG3	TRADD	TUSC5	TMEM11	GFAP	TCF3	PRKCSH	ZNF491	TSHZ3	TTYH1	PTK6
RP6	PLP1	HTR2C	RP1	FTH1P8	MT

GOBPID	Pvalue	OddsRatio	ExpCount	Count	Size	Term
GO:0060134	3.98256092190817e-06	135	0.0327312988720504	3	11
prepulse inhibition
GO:0010976	1.53398505003001e-05	31.3387888707038	0.151754203861324
4	51	positive regulation of neuron projection development
GO:0001964	1.62097344171137e-05	77.1107142857143	0.0505847346204415
3	17	startle response
Tissue: Lung=>Thyroid	Type:	asymmetric
SourceGene:	ATP9B(ENSG00000166377.13)
TargetGeneSet:	C1orf159	SLC45A1 WDTC1	CCDC28B TMEM234 RP4	HMCN1
COL5A2	FAM134A STK25	NIT2	CP	FGFBP1	RP11	CTD	PDLIM7	ADAMTS2
CD109	ADAT2	AP4M1	GFRA2	CTHRC1	ANXA2P2 DNM1	COL5A1	ZEB1	SLC25A45
CAPN5	KRR1P1	COL4A2-AS1	SCFD1	XRCC3	SLC28A2 COL1A1	PGPEP1	ZNF738
DPY19L3 CHMP2A	TSPY26P ADIG	COL9A3	LINC00266-1	TSSK1A	RGAG1
GOBPID	Pvalue	OddsRatio	ExpCount	Count	Size	Term
GO:0030199	7.36751988167153e-09	98.8461538461538	0.0707909487232717
5	33	collagen fibril organization
GO:0043588	4.03512162778021e-06	45.3049645390071	0.10940419348142
4	51	skin development
Tissue: Thyroid=>Lung	Type:	asymmetric
SourceGene:	C1orf177(ENSG00000162398.7)
TargetGeneSet:	FGR	BTBD8	C1orf61 SELL	IKBKE	LBR	CCT4	IL1R2
SPOPL	SH3BP5	CMTM6	RP11	FAM208A FAM55C	MIR567	CPA3	CTD	TLR1	TLR6
KIT	FYB	CTB	MAP3K5	NCF1C	ATXN7L1 MTPN	PCMTD1	NTNG2	ABCA2	FUT7
ABI1	ACBD5	RASGEF1A	IKZF5	CLEC4D	IRAK3	PLXNC1	APAF1	GIT2
GLT1D1	ZDHHC20 TNFSF13B	C14orf118	CCNK	DYX1C1-CCPG1	CORO1A	TNS4
NKIRAS2 BECN1	FMNL1	UTP18	MBP	C19orf38	RAB8A	CD177	VSTM1
SIRPD	PMEPA1	ITGB2	IL17RA	GTPBP1	RP3	NFAM1	PANX2	CFP	PRAF2
GOBPID	Pvalue	OddsRatio	ExpCount	Count	Size	Term
GO:0002764	2.63465017376357e-06	10.7265151515152	0.892394989966092
8	248	immune response-regulating signaling pathway
GO:0050778	5.22588770360399e-06	8.28011791680314	1.30980554978894
9	364	positive regulation of immune response
GO:0002253	1.04359138787685e-05	8.81474539206498	1.07591170161235
8	299	activation of immune response
Tissue: Lung=>Thyroid	Type:	cluster
SourceGene:	CCDC104(ENSG00000163001.7)
TargetGeneSet:	WASH7P	MTND1P23	MTND2P28	NOL9	DFFA	CLCN6
DNAJC16 NECAP2	RP4	KDM1A	TCEB3	PAQR7	RP1	EYA3	MED18	YTHDF2
TXLNA	ZMYM6NB ZMYM1	EIF2C1	KDM4A	CCDC24	TOE1	IPP	NSUN4	SELRC1
DDX20	BCL9	RP11	RPRD2	GOLPH3L SCNM1	PYGO2	FLAD1	ETV3	HMGN1P4
RBBP5	ZC3H11B C2orf18 MSH6	ERLEC1	GPR75	DCTN1	C2orf68 KDM3A	TMEM127 LYG2
POLR1B	RBP4	AMMECR1L	STAM2	KRT8P15 RQCD1	EIF4E2	HMGB1P5 ACAA1
MYD88	PPM1M	PDE12	FAM3D	QTRTD1	MSL2	P2RY1	RAP2B	ABCF3	CRYGS
LRCH3	TADA2B	ENOPH1	AGPAT9	PLRG1	MARVELD2	PHAX	MTND5P11
RBM22	ABCF1	C6orf47 AGPAT1	ZBTB9	TAF8	RPL7L1	BEND6	FAM120B FTSJ2
FBXL18	INMT	AQP1	URGCP	YKT6	PURB	SNORA5A ZNF713	BAZ1B	FAM200A
ZNF498	MEPCE	GCC1	METTL2A SMO	ZC3HC1	ZNF212	ZNF746	CTD	INTS9	TTI2
SGK196	YTHDF3	PDP1	KIAA1429	TSPYL5	UTP23	TSSK5P1 ZNF517	RRAGA
KLHL9	STOML2	FOXE1	LINC00256B	ZBTB6	TBC1D13 EXOSC2	BRD3	C9orf167
C10orf140	THNSL1	ADO	AP3M1	ADK	SMC3	SIRT3	CHRNA10 SLC35C1
ARHGAP1 HEXIM1	BTBD18	MTA2	HNRNPUL2	ZBTB3	DPF2	C11orf68
EIF1AD	IL18BP	FAM76B	MSANTD4 PAFAH1B2	DDX6	PKNOX2	CERS5	SMUG1
ZC3H10	CAND1	TDG	BRAP	RNF6	KBTBD7	KCNRG	ALG11	CHAMP1	SALL2
RBM23	PROX2	DLST	MLH3	GOLGA8IP	TJP1	RNF111	PLEKHO2 FEM1B	NRG4
WASH3P	POLR3K	MAPK8IP3	THUMPD1 MAZ	TBC1D10B	CIAPIN1 CTRL
NFATC3	CHTF8	CENPBD1 PRDM7	VPS53	SLC16A13	TP53	ERAL1	CRLF3
ZNF830	TADA2A	MED1	WIPF2	IGFBP4	CNP	FAM134C VPS25	RUNDC1	ZNF652
PPP1R9B ANKRD40 TACO1	CANT1	ZBTB7A	FEM1A	ZNF557	ZNF562	FBXL12	ZNF440
ZNF429	C19orf12	ZNF526	BLOC1S3 CPT1C	SIGLEC7 ZNF613	ZNF542	ZNF264
ZNF543	ZNF547	ZNF549	ZNF134	AVP	FASTKD5 NANP	PLAGL2	NFS1	C20orf4
ZSWIM3	ZSWIM1	NCOA5	TP53RK	ZNFX1	PARD6B	ADNP	CSTF1	C20orf11
DNAJC28 SIM2	NDUFV3	NF2	NUP50	GEMIN8	GSPT2	SMC1A	BHLHB9

GOBPID	Pvalue	OddsRatio	ExpCount	Count	Size	Term
Tissue: Lung=>Thyroid	Type:	asymmetric
SourceGene:	CES1(ENSG00000198848.7)
TargetGeneSet:	NOL9	DNAJC16	RP11	WASF2	EYA3	ZSCAN20	EIF2C1	MFI2
SF3A3	RBBP5	DIEXF	HEATR1	FKBP1B	POLR1B	C2orf76	CLN5	ZXDC	UBQLN4P1
MYNN	ABCF3	RNF168	NOP14	SRP72	NUP155	CTB	DAGLB	ZNF713	MEPCE
CBLL1	TNPO3	PRSS37	TRIM35	SGK196	ZNF623	LINC00476	TBC1D13	EXOSC2	ADK
HIF1AN	C10orf2	SMC3	CTR9	SPTY2D1	NAT10	NUP160	ZBTB3	EIF1AD	DDX6
LPCAT3	KIAA1467	DDX23	CAND1	BRAP	SUPT16H	C14orf21	HMGN2P5	TSR1
C17orf85	NLGN2	TMEM199	FLOT2	FAM134C	FTSJ3	NOL11	CANT1	SUGP1
KCNJ14	C19orf76	ZNF264	ZNF543	ZNF304	ZNF547	ZNF749	DSTN	CRNKL1
COL18A1	RP13	UTP14A
GOBPID	Pvalue	OddsRatio	ExpCount	Count	Size	Term
GO:0071843	3.11080879409177e-11	15.9719764011799	1.05847346204415
13	239	cellular component biogenesis at cellular level
GO:0022613	2.4363836226506e-10	15.3564463705309	0.996470832468341
12	225	ribonucleoprotein complex biogenesis
GO:0022618	3.79493421339188e-06	16.6191398682681	0.420732129264411
6	95	ribonucleoprotein complex assembly
GO:0042254	4.08689031830945e-06	12.3196194712132	0.659885129056813
7	149	ribosome biogenesis
GO:0071826	5.11622235357972e-06	15.7296404988995	0.442875925541485
6	100	ribonucleoprotein complex subunit organization
GO:0006364	6.42344639483031e-06	15.0833919774806	0.460590962563144
6	104	rRNA processing
GO:0016072	8.88162284088054e-06	14.2072281167109	0.487163518095634
6	110	rRNA metabolic process
Tissue: Lung=>Thyroid	Type:	cluster
SourceGene:	CHIC2(ENSG00000109220.6)
TargetGeneSet:	TP73-AS1	KLHL21	ERRFI1	FBXO44	RP3	DNAJC16	TCEB3
TXLNA	RP4	YIPF1	CTH	ST7L	BCL2L15	GABPB1	SLC27A3	PYGO2	FLAD1	BAP1
NAV1	PFKFB2	IRF6	VSNL1	BRE	NFU1	AMMECR1L	STAM2	CFLAR	RP11
RQCD1	PSMD1	VGLL4	SATB1	CNOT10	MLH1	USP4	TFG	QTRTD1	TMEM39A
CCDC37	COPG1	KLHL24	CRYGS	UBXN7	TEC	SCFD2	ANKRD17	SOWAHB	MRPL1
SEC31A	SEC24D	TMEM154	DHX29	CTD	GTF2H2C	ANKRA2	TMEM167A	ARSK
SAR1B	CAMK2A	MYOZ3	SAP30L	RARS	BNIP1	SNCB	GRM6	TRIM27	ABCF1
PPP1R10	HSPA1L	AGPAT1	LINC00336	PPIL1	BYSL	COQ3	RTN4IP1	SESN1
LATS1	IGF2R	DLL1	C7orf26	POLR2J4	TBL2	ZNF3	SRRT	CEP41	PPP2R2A
CEBPD	RAD21	PLAA	FAM219A	GBA2	ALDH1B1	NCBP1	TGFBR1	C9orf91	GOLGA2	EDF1
CCNY	TIMM23	STOX1	PPP3CB	SEC24C	AP3M1	TBC1D12	ALDH18A1	ENTPD7	GBF1
RNU4-5P	ZNF195	CHRNA10	C11orf42	MUC15	HIPK3	CAPRIN1	PSMC3	MTCH2
CTNND1	SLC22A20	UCP3	ACER3	ZBTB16	PAFAH1B2	ARCN1	NOP2	ASUN
PRKAG1	AQP6	CERS5	TFCP2	COPZ1	METTL1	METTL21B	ALDH1L2	CKAP4
MYO1H	KCTD10	ANAPC7	ZNF140	N4BP2L1	CYCSP34	TDRD3	GNPNAT1	ARG2	PGF
EIF2B2	DIO2	GTF2A1	PSMC1	CALM1	FAM181A	MARK3	TJP1	TRIP4	C15orf39
NRG4	AKAP13	IGF1R	PMM2	UBFD1	DCTN5	CIAPIN1	GOT2	CTCF	HAS3
AFG3L1P	TP53	KCNAB3	VAMP2	ZSWIM7	DRG2	UTP6	PIGW	PSME3	BRCA1
TMEM106A-AS1	UBTF	RSAD1	MBTD1	FAM100B	ZNF562	ZNF44	MPV17L2	LSM4
ZNF429	ZNF565	ZNF790	PSMC4	ZNF470	ZNF586	ZNF135	ZNF544	FKBP1A	CDK5RAP1
MANBAL	SLC13A3	DOK5	RP1	CTSZ	LINC00315	ARL2BPP10	KREMEN1
L3MBTL2	APOO	BCOR	NDP	WBP5	RP6	LHFPL1
GOBPID	Pvalue	OddsRatio	ExpCount	Count	Size	Term
GO:0048199	3.18112059422577e-10	40.0673684210526	0.295204484118746
8	27	vesicle targeting, to, from or within Golgi
GO:0006901	3.13155329490008e-09	28.1797530864198	0.382672479413189
8	35	vesicle coating
GO:0006900	5.04272049283586e-09	26.2326436781609	0.4045394782368	8
37	membrane budding
GO:0006903	5.04272049283586e-09	26.2326436781609	0.4045394782368	8
37	vesicle targeting
GO:0051650	4.45154798487893e-08	19.004	0.52480797176666	8	48
establishment of vesicle localization
GO:0051648	5.08809713526669e-07	13.3202339181287	0.710677461767352

8	65	vesicle localization
GO:0031400	1.03206276884596e-06	5.15568641884431	3.28004982354162
15	300	negative regulation of protein modification process
GO:0002474	1.32864507079132e-06	9.53221476510067	1.08241644176874
9	99	antigen processing and presentation of peptide antigen via MHC class I
GO:0051656	1.57335645071639e-06	9.32367960315144	1.10428344059235
9	101	establishment of organelle localization
GO:0048002	2.76127071889211e-06	8.660158633313	1.18081793647498	9
108	antigen processing and presentation of peptide antigen
GO:0032269	3.91005541938262e-06	4.33616061006926	4.13286277766245
16	378	negative regulation of cellular protein metabolic process
GO:0016050	6.73855416381985e-06	9.13092369477912	0.994948446474292
8	91	vesicle organization
GO:0035964	9.10906238497008e-06	41.2236652236652	0.14213549235347
4	13	COPI-coated vesicle budding
GO:0048194	9.10906238497008e-06	41.2236652236652	0.14213549235347
4	13	Golgi vesicle budding
GO:0048200	9.10906238497008e-06	41.2236652236652	0.14213549235347
4	13	Golgi transport vesicle coating
GO:0048205	9.10906238497008e-06	41.2236652236652	0.14213549235347
4	13	COPI coating of Golgi vesicle
GO:0051248	1.46951828398415e-05	3.87365778831404	4.59206975295827
16	420	negative regulation of protein metabolic process
GO:0031396	1.57121162212506e-05	6.04473144030106	1.83682790118331
10	168	regulation of protein ubiquitination
GO:0048207	1.70955476155306e-05	33.7237308146399	0.164002491177081
4	15	vesicle targeting, rough ER to cis-Golgi
GO:0048208	1.70955476155306e-05	33.7237308146399	0.164002491177081
4	15	COPII vesicle coating
GO:0090114	1.70955476155306e-05	33.7237308146399	0.164002491177081
4	15	COPII-coated vesicle budding
Tissue: Thyroid=>Lung	Type:	asymmetric
SourceGene:	CLEC17A(ENSG00000187912.7)
TargetGeneSet:	MXRA8	RPL11	PRDX3P2	RNF11	EFNA4	APOA2	SMG7	DIEXF
COX5B	MZT2B	SETD5	RPL15	CRTAP	RPL29	RP11	C4orf48	OCIAD1	WDFY3-AS2
RPL34	NDUFS4	SYNGAP1	C6orf162	LINC00271	C6orf72	SEPT7	GBAS
CHCHD2	NDUFA5	C8orf40	RAB2A	POLR2K	MRPL41	FAM204A	RPLP2	SVIP	FAU
ATP5L	ZCRB1	ATXN2	SIVA1	RPLP1	AKAP13	TTLL13	NDUFB10	BCL7C	RPL13
WDR81	ALOX15B	RPS7P1	CHST9	IER3IP1	ATP5D	RPS15	C19orf70	ZNF414
C19orf60	ZNF428	SEL1L2	ELMO2	MORC2	COX7B	TAF9B	FERP1
GOBPID	Pvalue	OddsRatio	ExpCount	Count	Size	Term
GO:0000184	1.15108487616834e-12	40.9977064220183	0.345858418102553
10	119	nuclear-transcribed mRNA catabolic process, nonsense-mediated decay
GO:0006415	3.90611021708204e-12	47.6507760532151	0.264479966784306
9	91	translational termination
GO:0019080	1.2699145793037e-11	31.6223404255319	0.438862362466265
10	151	viral genome expression
GO:0019083	1.2699145793037e-11	31.6223404255319	0.438862362466265
10	151	viral transcription
GO:0006414	1.45494050106561e-11	40.6619318181818	0.305169192443429
9	105	translational elongation
GO:0006614	1.45494050106561e-11	40.6619318181818	0.305169192443429
9	105	SRP-dependent cotranslational protein targeting to membrane
GO:0006613	1.5866521412126e-11	40.2399250234302	0.308075565704795
9	106	cotranslational protein targeting to membrane
GO:0045047	1.5866521412126e-11	40.2399250234302	0.308075565704795
9	106	protein targeting to ER
GO:0072599	1.5866521412126e-11	40.2399250234302	0.308075565704795
9	106	establishment of protein localization to endoplasmic reticulum
GO:0000956	3.90262604607315e-11	28.0070754716981	0.491177081170853
10	169	nuclear-transcribed mRNA catabolic process
GO:0070972	4.21404579777737e-11	35.7798165137615	0.342952044841187

9	118	protein localization to endoplasmic reticulum
GO:0006402	6.52857140390416e-11	26.4899553571429	0.517334440523147
10	178	mRNA catabolic process
GO:0006401	2.62586613772342e-10	22.7788461538462	0.595806518580029
10	205	RNA catabolic process
GO:0006413	4.11871732601169e-10	27.2078830260648	0.441768735727631
9	152	translational initiation
GO:0006612	4.36708395855424e-10	27.0170454545455	0.444675108988997
9	153	protein targeting to membrane
GO:0043624	4.36708395855424e-10	27.0170454545455	0.444675108988997
9	153	cellular protein complex disassembly
GO:0043241	5.81757041012065e-10	26.1012812690665	0.459206975295827
9	158	protein complex disassembly
GO:0019058	8.43176571987788e-10	20.0622171945701	0.671372223375545
10	231	viral infectious cycle
GO:0034623	1.37023776962067e-09	23.5438016528926	0.505708947477683
9	174	cellular macromolecular complex disassembly
GO:0032984	1.7604437071694e-09	22.8433155080214	0.520240813784513
9	179	macromolecular complex disassembly
GO:0022415	2.65183685286986e-09	17.69875	0.755657047955159	10
260	viral reproductive process
GO:0072594	5.56069320907923e-09	19.8797202797203	0.592900145318663
9	204	establishment of protein localization to organelle
GO:0022904	1.0207041821532e-08	32.1797752808989	0.279011833091136
7	96	respiratory electron transport chain
GO:0071845	9.73244793540003e-08	14.0171900826446	0.825410006227943
9	284	cellular component disassembly at cellular level
GO:0022900	1.03666298067087e-07	22.4913385826772	0.389454017023043
7	134	electron transport chain
GO:0022411	1.09661073000473e-07	13.8123167155425	0.837035499273407
9	288	cellular component disassembly
GO:0045333	1.54341118457122e-07	21.1466666666667	0.412705003113971
7	142	cellular respiration
GO:0016032	4.76546954001427e-07	9.89795918367347	1.31077434087606
10	451	viral reproduction
GO:0006119	6.63194113585121e-07	36.6037735849057	0.168569649159228
5	58	oxidative phosphorylation
GO:0033365	6.80668467421228e-06	8.19651253918495	1.37471455262612
9	473	protein localization to organelle
GO:0006605	6.92388962939753e-06	8.1782991202346	1.37762092588748	9
474	protein targeting
Tissue: Lung=>Thyroid	Type:	cluster
SourceGene:	DHX40(ENSG00000108406.4)
TargetGeneSet:	NOC2L	DNAJC16	ARHGEF19	ARHGEF19-AS1	PAQR7	RP11	NCDN
CCDC24	C1orf123	INSL5	WDR77	ST7L	MOV10	RP4	PIAS3	TARS2
FLAD1	CCT3	METTL13	DHX9	BAP1	NAV1	C1orf131	ODC1	EIF2B4
SRBD1	POLR1A	PSMD14	RQCD1	PSMD1	C3orf62	PBRM1	WNT5A	WNT5A-AS1
GRAMD1C	QTRTD1	RNF13	MRPL47	KLHL24	EIF2B5	AP2M1	ABCF3	ALG3	PSMD2
SCFD2	PPAT	SEC31A	ETFDH	MFAP3L	TRAPPC11	DHX29	ARSB	PAIP2
GALNT10	SAP30L	TRIM27	PRR3	PTMAP1	TUBB	VARS	WDR46	PPIL1	AARS2
GPR63	RP1	LATS1	IQCE	YKT6	TBRG4	COPS6	ORC5	PUS7	METTL2A	SMO
CEP41	SLC35B4	TTC26	ABCB8	SMARCD3	YBX1P4	BNIP3L	TRIM35	CHMP4C	VCP
STOML2	FXN	NCBP1	ANKS6	DNM1	NGB	C10orf114	C10orf140
RRP12	ENTPD7	RRP8	RCOR2	CD248	RIN1	C2CD2L	NOP2	COPS7A	TUBA1C
TFCP2	TARBP2	CYP27B1	METTL1	NT5DC3	ALDH1L2	CKAP4	POLR3B	MYO1H	KCTD10
ANAPC7	ATXN2	GTF2H3	PUS1	GOLGA3	N4BP2L1	PRMT5-AS1	PRMT5	ARG2
TUBGCP4	CTD	GLYR1	TBC1D10B	E2F4	CTCF	C16orf46	COX4NB
FAM57A	TSR1	ANKFY1	ERAL1	UTP6	LIG3	TADA2A	LEPREL4	PSME3	TMEM106A
ATXN7L3	UBTF	PNPO	RSAD1	INTS2	GPS1	NACC1	PRKACA	PIK3R2	RAB3A
C19orf12	ZNF790	PSMC4	GRWD1	ZNF135	NSFL1C	NOP56	ZNF341	ELMO2	ADNP
ZFP64	GTPBP5	PATZ1	L3MBTL2	KIAA0930	FAM123B
GOBPID	Pvalue	OddsRatio	ExpCount	Count	Size	Term
GO:0034660	6.22355165320264e-09	7.26736705996444	2.58355823126427

16	285	ncRNA metabolic process
GO:0022613	1.60833835969351e-08	8.00121521448536	2.03965123520864
14	225	ribonucleoprotein complex biogenesis
GO:0071843	3.44876780339617e-08	7.49591642924976	2.16656286762162
14	239	cellular component biogenesis at cellular level
GO:0032269	1.68553856327261e-06	4.97185807922485	3.42661407515051
15	378	negative regulation of cellular protein metabolic process
GO:0022618	2.30828000140848e-06	10.6405008877675	0.861186077088091
8	95	ribonucleoprotein complex assembly
GO:0071826	3.4025624660899e-06	10.0586779780841	0.906511660092727
8	100	ribonucleoprotein complex subunit organization
GO:0051248	6.10921304185342e-06	4.44284802043423	3.80734897238945
15	420	negative regulation of protein metabolic process
GO:0071158	7.85092709955775e-06	11.0173442333186	0.725209328074182
7	80	positive regulation of cell cycle arrest
GO:0006521	1.22894247264736e-05	13.4296470588235	0.516711646252854
6	57	regulation of cellular amino acid metabolic process
GO:0034470	1.38935912753098e-05	6.14615050021749	1.81302332018545
10	200	ncRNA processing
GO:0031400	1.80428717651274e-05	4.91316526610644	2.71953498027818
12	300	negative regulation of protein modification process
GO:0031397	1.9787170992772e-05	9.45398481973434	0.833990727285309
7	92	negative regulation of protein ubiquitination
Tissue: Lung=>Thyroid	Type:	asymmetric
SourceGene:	ENSG00000203606.3
TargetGeneSet:	ZNF683	KIF21B	LRRN4	RPSA	XCR1	RSRC1	HELQ	RPL34	RP11
IL12B	ANKRD61	AHCYL2	RPS20	RPS6	RPL35	NRG3	RPL13AP5	MED17
RPL18AP3	RPS3AP6	FTLP14	CTD	RPS15	IL12RB1	UBA52	RPL13A	RPL28	RPS5
BACH1	SOX10	GRAP2	MAGEE2
GOBPID	Pvalue	OddsRatio	ExpCount	Count	Size	Term
GO:0006415	1.07543866020772e-16	126.507936507937	0.151131409591032
10	91	translational termination
GO:0006414	4.76781576616223e-16	107.759398496241	0.17438239568196
10	105	translational elongation
GO:0006614	4.76781576616223e-16	107.759398496241	0.17438239568196
10	105	SRP-dependent cotranslational protein targeting to membrane
GO:0006613	5.25979868331653e-16	106.629464285714	0.17604318040274
10	106	cotranslational protein targeting to membrane
GO:0045047	5.25979868331653e-16	106.629464285714	0.17604318040274
10	106	protein targeting to ER
GO:0072599	5.25979868331653e-16	106.629464285714	0.17604318040274
10	106	establishment of protein localization to endoplasmic reticulum
GO:0070972	1.5921890154812e-15	94.7023809523809	0.195972597052107
10	118	protein localization to endoplasmic reticulum
GO:0000184	1.73672009496678e-15	93.826998689384	0.197633381772888	10
119	nuclear-transcribed mRNA catabolic process, nonsense-mediated decay
GO:0019080	1.98720990239267e-14	72.370820668693	0.250778492837866	10
151	viral genome expression
GO:0019083	1.98720990239267e-14	72.370820668693	0.250778492837866	10
151	viral transcription
GO:0006413	2.12526461752503e-14	71.8561368209256	0.252439277558646
10	152	translational initiation
GO:0006612	2.27186441596622e-14	71.3486513486513	0.254100062279427
10	153	protein targeting to membrane
GO:0043624	2.27186441596622e-14	71.3486513486513	0.254100062279427
10	153	cellular protein complex disassembly
GO:0043241	3.15009360763803e-14	68.9140926640927	0.26240398588333
10	158	protein complex disassembly
GO:0000956	6.23373642231738e-14	64.0970350404313	0.280672617811916
10	169	nuclear-transcribed mRNA catabolic process
GO:0034623	8.37322751028232e-14	62.1210801393728	0.288976541415819
10	174	cellular macromolecular complex disassembly
GO:0006402	1.05359344762146e-13	60.625	0.295619680298941	10	178

mRNA catabolic process
GO:0032984	1.11494341089671e-13	60.262045646661 0.297280465019722	10
179	macromolecular complex disassembly
GO:0072594	4.16097052637557e-13	52.4042709867452	0.338800083039236
10	204	establishment of protein localization to organelle
GO:0006401	4.37045607501466e-13	52.1318681318681	0.340460867760017
10	205	RNA catabolic process
GO:0019058	1.44573429335072e-12	45.9146735617324	0.383641270500311
10	231	viral infectious cycle
GO:0022415	4.70042883773067e-12	40.5057142857143	0.431804027402948
10	260	viral reproductive process
GO:0071845	1.12918495204337e-11	36.8952033368092	0.471662860701682
10	284	cellular component disassembly at cellular level
GO:0022411	1.29696529821864e-11	36.3540596094553	0.478305999584804
10	288	cellular component disassembly
GO:0033365	6.99418000080748e-11	25.5769230769231	0.785551172929209
11	473	protein localization to organelle
GO:0006605	7.15427705087035e-11	25.5198537963117	0.78721195764999
11	474	protein targeting
GO:0016032	1.05208683112948e-09	22.6530612244898	0.749013909072037
10	451	viral reproduction
GO:0042274	3.23105168313163e-06	137.257142857143	0.0298941249740502
3	18	ribosomal small subunit biogenesis
GO:0072540	1.58285995353423e-05	655.681818181818	0.00664313888312228
2	4	T-helper 17 cell lineage commitment
GO:2000328	1.58285995353423e-05	655.681818181818	0.00664313888312228
2	4	regulation of T-helper 17 cell lineage commitment
GO:2000330	1.58285995353423e-05	655.681818181818	0.00664313888312228
2	4	positive regulation of T-helper 17 cell lineage commitment
Tissue: Thyroid=>Lung	Type:	asymmetric
SourceGene:	EXOC6B(ENSG00000144036.9)
TargetGeneSet:	RPL22	PIK3CD	CLCNKB	RP1	RPL11	NIPAL3	RPS8	S100A3
ARHGAP30	DUSP12	RGS18	RPS7	COMMD1	COA5	RPL31	RPL37A	GPBAR1
DDX11	RPL32	LSM3	RPL14	CCR2	C3orf27	RPL35A	MYL5	HTT	OCIAD1
RPL34	TLR2	RPL37	CTD	PLK2	ERCC8	CDC23	DOCK2	RUFY1	CSNK2B
HNRNPC	MEA1	IGF2R	LINC00473	AOAH	SBDS	MAGI2-AS3	PON2
IMPDH1	RP11	SPIN1	C9orf3	BAAT	EDF1	PIK3AP1	FAM204A	RPLP2	RP13	CTR9
RPS13	SAA1	DEPDC7	PTPRJ	FAU	SIPA1	ARAP1-AS2	ATP5L	RPS25
C3AR1	B4GALNT1	APAF1	GLT8D2	PSPC1	RPL3P4	AQP9	CSK	C16orf70
GNA15	EMR2	POU2F2	RPL18	SIGLEC5	FPR2	ZNF813	LILRB4	SIRPG	CDS2	HCK
RP4	SLCO4A1	SH3BP1	PPARA	AFF2
GOBPID	Pvalue	OddsRatio	ExpCount	Count	Size	Term
GO:0006415	1.22551557183846e-20	50.8444444444444	0.478582797038267
16	91	translational termination
GO:0006414	1.39415830001593e-19	42.8044943820225	0.552210919659539
16	105	translational elongation
GO:0006614	1.39415830001593e-19	42.8044943820225	0.552210919659539
16	105	SRP-dependent cotranslational protein targeting to membrane
GO:0006613	1.63555547543825e-19	42.3259259259259	0.557470071275344
16	106	cotranslational protein targeting to membrane
GO:0045047	1.63555547543825e-19	42.3259259259259	0.557470071275344
16	106	protein targeting to ER
GO:0072599	1.63555547543825e-19	42.3259259259259	0.557470071275344
16	106	establishment of protein localization to endoplasmic reticulum
GO:0070972	9.85193947891127e-19	37.3150326797386	0.620579890665006
16	118	protein localization to endoplasmic reticulum
GO:0000184	1.13375747411844e-18	36.9501618122977	0.625839042280811
16	119	nuclear-transcribed mRNA catabolic process, nonsense-mediated decay
GO:0043624	2.38731745532775e-18	30.1673728813559	0.804650197218186
17	153	cellular protein complex disassembly
GO:0043241	4.16889475989191e-18	29.0873903113355	0.830945955297211
17	158	protein complex disassembly
GO:0000956	1.33170191905417e-17	26.9615298840321	0.888796623071068

17	169	nuclear-transcribed mRNA catabolic process
GO:0034623	2.19755195044071e-17	26.0937061427183	0.915092381150093
17	174	cellular macromolecular complex disassembly
GO:0006402	3.24419208790125e-17	25.4382566585956	0.936128987613314
17	178	mRNA catabolic process
GO:0032984	3.57067420166205e-17	25.2794517681523	0.941388139229119
17	179	macromolecular complex disassembly
GO:0019080	5.69250548323201e-17	28.1283950617284	0.794131893986575
16	151	viral genome expression
GO:0019083	5.69250548323201e-17	28.1283950617284	0.794131893986575
16	151	viral transcription
GO:0006413	6.33714914455192e-17	27.9196078431373	0.799391045602381
16	152	translational initiation
GO:0006612	7.04937307436429e-17	27.7138686131387	0.804650197218186
16	153	protein targeting to membrane
GO:0006401	3.58430859198859e-16	21.7435088351965	1.07812608124005
17	205	RNA catabolic process
GO:0072594	7.08232069808961e-15	20.1234042553191	1.07286692962425
16	204	establishment of protein localization to organelle
GO:0019058	4.99815591437332e-14	17.5627906976744	1.21486402325099
16	231	viral infectious cycle
GO:0071845	8.14201157117466e-14	15.2247825810957	1.49359905888866
17	284	cellular component disassembly at cellular level
GO:0022411	1.02371903515935e-13	14.9958096191131	1.51463566535188
17	288	cellular component disassembly
GO:0022415	3.13993713003726e-13	15.4437158469945	1.36737942010933
16	260	viral reproductive process
GO:0016032	1.33164525692891e-11	9.9926734092538 2.37187737872812	18
451	viral reproduction
GO:0033365	2.92686818870582e-11	9.49450549450549	2.48757871427583
18	473	protein localization to organelle
GO:0006605	3.03052855217993e-11	9.47300362976407	2.49283786589163
18	474	protein targeting
GO:0042273	3.38648218066051e-07	99.7708333333333	0.0631098193896616
4	12	ribosomal large subunit biogenesis
GO:0050900	4.6320715091572e-06	8.18881883685023	1.26745553940904
9	241	leukocyte migration
GO:0042254	1.29733160687055e-05	10.1685037762809	0.783613590754965
7	149	ribosome biogenesis
GO:0006364	1.74693870538412e-05	12.4871720116618	0.546951768043734
6	104	rRNA processing
Tissue: Lung=>Thyroid	Type:	asymmetric
SourceGene:	MEIS3(ENSG00000105419.10)
TargetGeneSet:	C1QA	C1QC	C1QB	RP4	RP11	CD84	SLAMF1	FCGR2A
TRIM54	C2orf68 IGKV4-1 IGKV1-5 IGKV3-20	IGKV2-28	CLASP1	WIPF1	BTD
TEX264	CD86	CTD	CD180	SMN2	DOCK2	MIR590	NCF1	PIK3CG	TMEM209 DOK2
TNFSF8	ZDHHC12 FAM78A	MS4A7	NCKAP1L RP13	LINC00543	RPL21P110
TRAV12-3	IGHA2	IGHA1	IGHV3-23	IGHV1-69	IGHV3-74
PLCB2	CD68	VAV1	MYO1F	TYROBP	MAP4K1	CEACAMP3	CD37	SIGLEC9 FPR3
LAIR1	C21orf128	ITGB2	IGLV3-1 IGLL5	IGLC1	NCF4	SH3BP1	APOBEC3F
VSIG4	CXCR3	ZDHHC15 SASH3
GOBPID	Pvalue	OddsRatio	ExpCount	Count	Size	Term
GO:0002252	2.87071538339873e-17	25.4311594202899	1.20199294166494
18	386	immune effector process
GO:0006956	2.45869768457851e-13	66.4444444444444	0.196180195142205
9	63	complement activation
GO:0002443	1.2928520344683e-12	25.96071265418	0.657047955158813	12
211	leukocyte mediated immunity
GO:0006958	2.23355898765962e-12	73.945945945946 0.155698567573178	8
50	complement activation, classical pathway
GO:0072376	2.91685239485629e-12	49.0856164383562	0.255345650820012
9	82	protein activation cascade
GO:0006959	3.14566521722145e-12	36.4642857142857	0.379904504878555

10	122	humoral immune response
GO:0002455	2.02913909719507e-11	54.4295874822191	0.202408137845132
8	65	humoral immune response mediated by circulating immunoglobulin
GO:0002250	3.90545676405975e-10	21.6079027355623	0.616566327589786
10	198	adaptive immune response
GO:0050778	7.41040877791082e-10	14.5185950413223	1.13348557193274
12	364	positive regulation of immune response
GO:0002253	1.34484561432163e-09	15.859681372549 0.931077434087606	11
299	activation of immune response
GO:0016064	1.59786108920534e-09	30.0246654421412	0.345650820012456
8	111	immunoglobulin mediated immune response
GO:0002449	1.83702363129305e-09	22.5443037974684	0.520033215694416
9	167	lymphocyte mediated immunity
GO:0019724	1.9780230461189e-09	29.1687914329424	0.354992734066847
8	114	B cell mediated immunity
GO:0002460	3.73693396883311e-09	20.6889534883721	0.563628814614906
9	181	adaptive immune response based on somatic recombination of immune receptors built from immunoglobulin superfamily domains
GO:0046631	5.3171472034183e-06	23.2613636363636	0.255345650820012
5	82	alpha-beta T cell activation
GO:0042110	5.46578104487067e-06	9.79925262883462	0.993356861116878
8	319	T cell activation
GO:0046649	6.08926902884319e-06	8.24410377358491	1.34834959518372
9	433	lymphocyte activation
GO:0043372	1.86112825578915e-05	73.4285714285714	0.0529375129748806
3	17	positive regulation of CD4-positive, alpha-beta T cell differentiation
Tissue: Lung=>Thyroid	Type:	asymmetric
SourceGene:	MORC2(ENSG00000133422.7)
TargetGeneSet:	RP4	C1orf159	UBE2J2	CEP104	DNAJC16	HSPB7	RP11	EYA3
KPNA7	TAL1	RP1	NFIA	SLC35A3	GOLPH3L	UBQLN4	CCT3	POGK	CDC73
TMEM81	RBBP5	LINC00467	SMC6	TTC32	UBXN2A	PREB	SLC5A6	EIF2B4
ERLEC1	PNPT1	CCT4	SPR	CCT7	AUP1	RMND5A	MRPS5	ASTL	AMMECR1L
TFPI	HSPD1	HDLBP	NKIRAS1	ABHD5	GLYCTK	NEK4	PPP4R2	PTPLB	A4GNT
SLC25A36	ABCF3	CEP19	LRCH3	ZNF141	CLOCK	EIF4E	FAM105B	ZFR
DNAJC21	CTD	IPO11	ZFYVE16	SHROOM1	CSNK1A1	TSPAN17	TRIM27	HCG20	HLA-DOB
RING1	TAF8	MTO1	PGM3	PEX7	NHSL1	AQP1	PURB	YWHAG	ZKSCAN1
AGFG2	CTA	C7orf53	CADPS2	SMO	NFE2L1	TRBV7-3	ABCF2	DNAJB6	KBTBD11
NEIL2	POLR3D	NKX3-1	PPP2R2A	LSM12P1	KAT6A	C8orf40	ZHX1	IFNK	DNAJA1
TESK1	DCAF10	C9orf40	SUSD3	NIPSNAP3B	NOXA1	FBXO18	TAF3	BMS1
ZFAND4	P4HA1	AP2A2	RIC3	ETFA	MTA2	C11orf84	MARK2	STIP1	KLC2
RBM14	C11orf30	DDX10	C11orf92	PAFAH1B2	HSPA8	FKBP4
TULP3	C12orf33	KIAA1467	REP15	SLC35E3	CCT2	TDG	C12orf34
ATXN2	C12orf65	RPL22P19	RAN	PUS1	GOLGA3	RPL23AP67
MPHOSPH8	HSPH1	NHLRC3	DHRS12	SUGT1	A2LD1	PRMT5-AS1	PRMT5
ACIN1	SOCS6	DHRS7	AHSA1	HSP90AA1	GOLGA8IP	PPP1R14D
TP53BP1	AP4E1	TNFAIP8L3	PIGB	CSNK1G1	PEAK1	DNAJA4	CARHSP1	MAZ
SULT1A3	CD2BP2	TBC1D10B	DNAJA2	CCL22	GFOD2	MAF	GAN	ZDHHC7
KLHDC4	VPS53	DLG4	GIT1	CCL16	WIPF2	LEPREL4	SRP68	JMJD6	CANT1	RP13
ADAT3	SAFB	ELAVL1	ZNF562	CLEC17A	LIN37	ZNF781	MAP3K10	MIA	IRGQ
MYPOP	KCNJ14	ZNF347	ZNF761	ZNF814	ZNF329	RP3	KRT18P4	KRT18P5	CRKL
TOP3B	SGSM1	MORC2	MID2
GOBPID	Pvalue	OddsRatio	ExpCount	Count	Size	Term
GO:0006457	1.23318444524738e-09	8.17217391304348	2.30184762300187
16	216	protein folding
GO:0006986	9.67775375886308e-06	7.33293103448276	1.37471455262612
9	129	response to unfolded protein
GO:0035966	1.48379429324467e-05	6.92533260928591	1.44931146633451
9	136	response to topologically incorrect protein
GO:0006458	1.61056528419891e-05	12.8396396396396	0.543491799875441
6	51	'de novo' protein folding
Tissue: Lung=>Thyroid	Type:	asymmetric
SourceGene:	MRS2P2(ENSG00000257802.1)

TargetGeneSet: MTND1P23 MTND2P28 NOL9 SPEN RP11 RP1
WASF2 EYA3 MED18 PHACTR4 PFN1P8 RPRD2 POGK RBBP5 CICP5 RN5S78
GPR75 AAK1 POLR1A RQCD1 QTRTD1 ANKRD17 CTD APC PHAX CTB
MTND5P11 PCDH1 GFOD1 ZBTB9 TAF8 MTRNR2L9 URGCP ZNF713
ZKSCAN5 MEPCE CBLL1 ZNF800 TRIM35 KIAA1429 KLHL9 ZFP37 EXOSC2
PRINS LRRC37A6P AK3P5 DUPD1 PFN1P11 CHRNA10 MTRNR2L8 FNBP4
ZBTB3 MSANTD4 ZNF202 CD9 ACVRL1 PRKAB1 RBM22P2 ARGLU1 GOLGA8IP TJP1
MGA RPL35AP32 C15orf58 MAZ FTO-IT1 NFATC3 SDR42E1 RNASEK
WIPF2 IGFBP4 FEM1A ZNF557 ZNF317 ZNF562 ZNF440 ZNF44 ZNF429 LIN37
ZNF565 ZFP82 ZNF480 ZNF765 ZNF264 ZNF543 ZNF17 ZNF544 NCOA5 ADNP
NDUFV3 NF2 PRR14L ZNF41 UTP14A
GOBPID Pvalue OddsRatio ExpCount Count Size Term
Tissue: Lung=>Thyroid Type: asymmetric
SourceGene: OLA1(ENSG00000138430.11)
TargetGeneSet: RP11 NCDN CCDC24 VANGL1 METTL13 C1D USP39 RPL31
CCDC74B BDP1 CAB39 TOP2B ZNF860 XYLB ZNF167 RPL29 POC1A EIF4E3
IFT57 CNBP LRCH3 LAMTOR3 ELOVL6 GTF2F2P1 HINT1 SKP1 PEX7
ATP6V1B2 RPS20 IMPDH1P6 ZNF618 ATP6V1G1 THNSL1 SUFU
C10orf32 C11orf58 FAM76B ZC3H12C COPS7A HOXC5 HMGB1 ITM2B
AKAP5 RPL4 C16orf59 RPL23A RPL19 GPR142 CBX8 RPS15 SIN3B
ZNF536 SIGLEC7 LINC00493 ZFP64 PPM1F CTPS2 RPS4X BEX4 MIR224

GOBPID	Pvalue	OddsRatio	ExpCount	Count	Size	Term
GO:0006415	3.21955477760918e-10	37.3116248778899	0.283371392983185	8	91	translational termination
GO:0006414	1.02297167185496e-09	31.8952354416272	0.326966991903675	8	105	translational elongation
GO:0006614	1.02297167185496e-09	31.8952354416272	0.326966991903675	8	105	SRP-dependent cotranslational protein targeting to membrane
GO:0006613	1.10395183700959e-09	31.5675675675676	0.330080963255138	8	106	cotranslational protein targeting to membrane
GO:0045047	1.10395183700959e-09	31.5675675675676	0.330080963255138	8	106	protein targeting to ER
GO:0072599	1.10395183700959e-09	31.5675675675676	0.330080963255138	8	106	establishment of protein localization to endoplasmic reticulum
GO:0070972	2.60527694582146e-09	28.1002457002457	0.367448619472701	8	118	protein localization to endoplasmic reticulum
GO:0000184	2.78666132651158e-09	27.845142439737	0.370562590824164	8	119	nuclear-transcribed mRNA catabolic process, nonsense-mediated decay
GO:0072594	1.06541617984882e-08	18.2192307692308	0.635250155698568	9	204	establishment of protein localization to organelle
GO:0019080	1.83321836171405e-08	21.5656775656776	0.470209674070999	8	151	viral genome expression
GO:0019083	1.83321836171405e-08	21.5656775656776	0.470209674070999	8	151	viral transcription
GO:0006413	1.93063668823183e-08	21.4144144144144	0.473323645422462	8	152	translational initiation
GO:0006612	2.03249372007879e-08	21.2652376514445	0.476437616773926	8	153	protein targeting to membrane
GO:0043624	2.03249372007879e-08	21.2652376514445	0.476437616773926	8	153	cellular protein complex disassembly
GO:0043241	2.61444317343914e-08	20.5491891891892	0.492007473531244	8	158	protein complex disassembly
GO:0000956	4.42016157169263e-08	19.1304347826087	0.526261158397343	8	169	nuclear-transcribed mRNA catabolic process
GO:0034623	5.54487334207817e-08	18.5477043308369	0.541831015154661	8	174	cellular macromolecular complex disassembly
GO:0006402	6.6144265321733e-08	18.106200317965	0.554286900560515	8	178	mRNA catabolic process
GO:0032984	6.90801909820277e-08	17.9990516832622	0.557400871911978	8	179	macromolecular complex disassembly
GO:0006605	1.55855312098603e-07	9.74291703722526	1.47602242059373	11	474	protein targeting
GO:0071845	1.83687142817769e-07	12.8463636363636	0.884367863815653			

9	284	cellular component disassembly at cellular level
GO:0006401	1.9656774814511e-07	15.5950061736864	0.638364127050031
8	205	RNA catabolic process
GO:0022411	2.06815135429612e-07	12.6586021505376	0.896823749221507
9	288	cellular component disassembly
GO:0019058	4.88983744313021e-07	13.7515452672403	0.719327382188084
8	231	viral infectious cycle
GO:0016032	9.5001796262223e-07	9.04761904761905	1.40440107951007
10	451	viral reproduction
GO:0022415	1.19467009618048e-06	12.1441441441441	0.809632551380527
8	260	viral reproductive process
GO:0033365	1.46260855257869e-06	8.60413452638075	1.47290844924227
10	473	protein localization to organelle
Tissue: Lung=>Thyroid	Type:	asymmetric
SourceGene:	PRKCDBP(ENSG00000170955.9)
TargetGeneSet:	CEP104	RP4	RP1	EYA3	PEF1	TAL1	RP11	INTS3
UBQLN4	CCT3	RBBP5	URB2	MFSD2B	SLC5A6	PCBP1-AS1	HTRA2	LMAN2L
ANKRD39	AMMECR1L	RBM43	CYP8B1	PHF7	NEK4	SLC25A36	KIAA0226
IDUA	FAM105B	GABBR1	C6orf25	DAXX	USP42	METTL2A	DNAJB6	NEIL2	CHMP7
KAT6A	DNAJA1	TBC1D13	FBXO18	CEP57L1P1	P4HA1	AP2A2	SLC35C1	C11orf84
STIP1	KLC2	RCE1	C11orf30	C2CD2L	HSPA8	SCN3B	FKBP4	REP15
GCN1L1	DYNLL1	C12orf65	MPHOSPH8	HSPH1	RFC3	PRMT5	SOCS6
ACOT2	DNAL1	AHSA1	HSP90AA1	BUB1B	PPP1R14D	PIGB	CTD
SH2D7	DNAJA4	PRC1	TELO2	USP31	MAZ	C17orf39	C17orf51	GIT1
PIP4K2B	WIPF2	KAT2A	PSME3	UBE2Z	CDR2L	QRICH2	JMJD6	CSNK1D	ELOF1
DNAJB1	C19orf60	HIPK4	MIA	IRGQ	SMG9	NOVA2	ZNF329	ZSCAN22	RP3
LINC00478	SGSM1	NF2	PRR14L	LARGE	FAM123B	ACRC
GOBPID	Pvalue	OddsRatio	ExpCount	Count	Size	Term
GO:0006457	1.01918576706817e-08	12.2661683713611	1.09113556155283
11	216	protein folding
Tissue: Lung=>Thyroid	Type:	asymmetric
SourceGene:	RP11-114F3.5(ENSG00000255566.1)
TargetGeneSet:	DNAJC16	KIAA0090	TXLNA	SF3A3	EBNA1BP2	RP11	CTH
TMEM56	RLIMP2	PIAS3	BCL9	PSMD4	ADAR	FLAD1	FAM5B	NAV1	AKT3
POU3F3	PSMD14	TYW5	RQCD1	PSMD1	QTRTD1	RUVBL1	ALG3	PSMD2	PPAT	TET2
XBP1P1	GALNT10	PRR3	TUBB	VARS	ESR1	LINC00574	FAM71F1	SLC35B4
TAS2R5	TRIM35	OSGIN2	NCBP1	ANKS6	NGB	C10orf140	C11orf42
MRPL17	CAPRIN1	SLC1A2	GANAB	IL18BP	ASUN	METTL1	CAND1	POLR3B	PSME2
TUBGCP4	SRL	HSD3B7	CIAPIN1	TSR1	PIGW	PSMD3	PSME3	FAM100B	PIK3R2
RAB3A	SOX12	NOP56	TRMT6	NCOA5	SEPT3	TTC3P1	UTP14A
GOBPID	Pvalue	OddsRatio	ExpCount	Count	Size	Term
GO:0006521	4.22002709693428e-09	36.4992727272727	0.244550550134939
7	57	regulation of cellular amino acid metabolic process
GO:0031397	5.0530653684476e-09	25.2292768959436	0.394713168638849
8	92	negative regulation of protein ubiquitination
GO:0006977	1.20627742697857e-08	30.9121725731895	0.283163794893087
7	66	DNA damage response, signal transduction by p53 class mediator resulting in cell cycle arrest
GO:0072413	1.20627742697857e-08	30.9121725731895	0.283163794893087
7	66	signal transduction involved in mitotic cell cycle checkpoint
GO:0072431	1.20627742697857e-08	30.9121725731895	0.283163794893087
7	66	signal transduction involved in mitotic cell cycle G1/S transition DNA damage checkpoint
GO:0072474	1.20627742697857e-08	30.9121725731895	0.283163794893087
7	66	signal transduction involved in mitotic cell cycle G1/S checkpoint
GO:0051436	1.342519859634e-08	30.3948484848485	0.28745415542177
7	67	negative regulation of ubiquitin-protein ligase activity involved in mitotic cell cycle
GO:0072401	1.342519859634e-08	30.3948484848485	0.28745415542177
7	67	signal transduction involved in DNA integrity checkpoint
GO:0072404	1.342519859634e-08	30.3948484848485	0.28745415542177
7	67	signal transduction involved in G1/S transition checkpoint
GO:0072422	1.342519859634e-08	30.3948484848485	0.28745415542177

7	67	signal transduction involved in DNA damage checkpoint
GO:0072395	1.49159099716136e-08	29.8944858420268	0.291744515950453
7	68	signal transduction involved in cell cycle checkpoint
GO:0033238	2.23646394635328e-08	28.046993006993 0.308905958065186	7
72	regulation of cellular amine metabolic process
GO:0051352	2.23646394635328e-08	28.046993006993 0.308905958065186	7
72	negative regulation of ligase activity
GO:0051437	2.23646394635328e-08	28.046993006993 0.308905958065186	7
72	positive regulation of ubiquitin-protein ligase activity involved in mitotic cell cycle
GO:0051444	2.23646394635328e-08	28.046993006993 0.308905958065186	7
72	negative regulation of ubiquitin-protein ligase activity
GO:0031571	2.46541900927055e-08	27.6201101928375	0.313196318593869
7	73	mitotic cell cycle G1/S transition DNA damage checkpoint
GO:0002479	2.98331980719466e-08	26.8040106951872	0.321777039651235
7	75	antigen processing and presentation of exogenous peptide antigen via MHC class I, TAP-dependent
GO:0051439	3.93136073909734e-08	25.6660691421255	0.334648121237285
7	78	regulation of ubiquitin-protein ligase activity involved in mitotic cell cycle
GO:0000216	4.29919710162785e-08	25.3078282828283	0.338938481765968
7	79	M/G1 transition of mitotic cell cycle
GO:0042590	4.29919710162785e-08	25.3078282828283	0.338938481765968
7	79	antigen processing and presentation of exogenous peptide antigen via MHC class I
GO:0071158	4.69574322344922e-08	24.959402241594 0.343228842294651	7
80	positive regulation of cell cycle arrest
GO:0031575	5.58225818904681e-08	24.2904242424242	0.351809563352017
7	82	mitotic cell cycle G1/S transition checkpoint
GO:0051443	5.58225818904681e-08	24.2904242424242	0.351809563352017
7	82	positive regulation of ubiquitin-protein ligase activity
GO:0002478	6.07609077336516e-08	23.9691387559809	0.3560999238807 7
83	antigen processing and presentation of exogenous peptide antigen
GO:0031145	6.07609077336516e-08	23.9691387559809	0.3560999238807 7
83	anaphase-promoting complex-dependent proteasomal ubiquitin-dependent protein catabolic process
GO:0019884	7.17524577732959e-08	23.3512820512821	0.364680644938067
7	85	antigen processing and presentation of exogenous antigen
GO:0051351	7.78498809155745e-08	23.054085155351 0.36897100546675	7
86	positive regulation of ligase activity
GO:0071779	7.78498809155745e-08	23.054085155351 0.36897100546675	7
86	G1/S transition checkpoint
GO:0051438	1.67020359586843e-07	20.4494382022472	0.411874610753581
7	96	regulation of ubiquitin-protein ligase activity
GO:0002474	2.06528439981576e-07	19.7784584980237	0.42474569233963
7	99	antigen processing and presentation of peptide antigen via MHC class I
GO:2000045	2.06528439981576e-07	19.7784584980237	0.42474569233963
7	99	regulation of G1/S transition of mitotic cell cycle
GO:0051340	2.21333232867953e-07	19.5644183773216	0.429036052868314
7	100	regulation of ligase activity
GO:0048002	3.75371955881779e-07	18.0046804680468	0.463358937097779
7	108	antigen processing and presentation of peptide antigen
GO:0031396	5.59805003514572e-07	13.175	0.720780568818767	8	168
regulation of protein ubiquitination
GO:0030330	6.47889667386071e-07	16.5211570247934	0.501972181855927
7	117	DNA damage response, signal transduction by p53 class mediator
GO:0072331	9.0945033689989e-07	15.6600313479624	0.527714345028026
7	123	signal transduction by p53 class mediator
GO:0031398	1.12898228644264e-06	15.1337878787879	0.544875787142758
7	127	positive regulation of protein ubiquitination
GO:0000077	1.32138230143508e-06	14.7615668883962	0.557746868728808
7	130	DNA damage checkpoint

GO:0000084	1.6200271999712e-06	14.2926270579814	0.57490831084354
7	134	S phase of mitotic cell cycle
GO:0031570	1.78934603801533e-06	14.0690627202255	0.583489031900907
7	136	DNA integrity checkpoint
GO:0007093	2.07094577702927e-06	13.7464187327824	0.596360113486956
7	139	mitotic cell cycle checkpoint
GO:0042770	2.17267426545028e-06	13.6421052631579	0.600650474015639
7	140	signal transduction in response to DNA damage
GO:0051320	2.5032781502014e-06	13.3383689839572	0.613521555601688
7	143	S phase
GO:2000602	3.0086052567158e-06	12.9536363636364	0.630682997716421
7	147	regulation of interphase of mitotic cell cycle
GO:0090068	3.59644367545003e-06	12.5902777777778	0.647844439831154
7	151	positive regulation of cell cycle process
GO:0019882	3.75749406192381e-06	12.5025705329154	0.652134800359837
7	152	antigen processing and presentation
GO:0010565	4.09769059308859e-06	12.3307359307359	0.660715521417203
7	154	regulation of cellular ketone metabolic process
GO:0022613	5.00227944008482e-06	9.67537122375832	0.965331118953706
8	225	ribonucleoprotein complex biogenesis
GO:0007346	5.32100917347733e-06	8.14112437427801	1.29997924019099
9	303	regulation of mitotic cell cycle
GO:0000209	5.49755148002312e-06	11.7644628099174	0.690748045117985
7	161	protein polyubiquitination
GO:0071843	7.79486493964174e-06	9.08000641333975	1.02539616635527
8	239	cellular component biogenesis at cellular level
GO:0044106	1.27238935544509e-05	10.2779958677686	0.785135976749014
7	183	cellular amine metabolic process
GO:0000082	1.41424410995654e-05	10.1036058913154	0.798007058335063
7	186	G1/S transition of mitotic cell cycle
GO:0006520	1.51770278059607e-05	6.28805620608899	1.87488755103453
10	437	cellular amino acid metabolic process
Tissue: Lung=>Thyroid	Type:	asymmetric
SourceGene:	RP11-252A24.3(ENSG00000261079.1)
TargetGeneSet:	RPL22	RPL11	RPS8	RP4	GNG5	RPL5	ABCA4	RP11
VANGL1	RPS27	OLFML2B	SLC45A3	PROX1	RPS7	MYCN	DCTN1	COA5	RPL31
STEAP3-AS1	WDR75	TBC1D5	RPL15	RPL14	ZNF445	RPL29	CACNA1D	CCDC66
RPL24	IGSF10	SUCNR1	NCBP2	WFS1	RPL34	GARS	RPS3A	WWC2-AS2	ING2
CDH10	NDUFAF2	TAF9	SKP1	ANXA6	LINC00340	CLIP2	C7orf58	SLC37A3
MRPS33	FNTA	RPL30	EIF3E	EBAG9	EIF3H	RPS6	TLN1	PGM5	ATP6V1G1
RPL12	ZNF488	RPS24	RPS3AP5	BLOC1S2	RPL27A	C11orf58	SVIP	BDNF
CYBASC3	INTS5	ARHGAP42	RPS25	RNF26	PPFIBP1	NAP1L1	RASSF9	GPN3	RPL6
TPT1	CYFIP1	RPS3AP6	RPL4	NEO1	SCAMP2	PEAK1	SEC11A	RPS15A	LYRM1
RPS7P1	RPL19	TANC2	CTD	RPL36	AP5S1	CDH22	HMGN1	SLC25A15P5
SNRPD3	RBFOX2	DMD	RPS4X	BEX5	HPRT1
GOBPID	Pvalue	OddsRatio	ExpCount	Count	Size	Term
GO:0000184	3.65391600310303e-37	77.5978260869565	0.667012663483496
27	119	nuclear-transcribed mRNA catabolic process, nonsense-mediated decay
GO:0006614	7.28082251612448e-37	85.5157652474108	0.588540585426614
26	105	SRP-dependent cotranslational protein targeting to membrane
GO:0006613	9.61149598680075e-37	84.4409090909091	0.594145733859249
26	106	cotranslational protein targeting to membrane
GO:0045047	9.61149598680075e-37	84.4409090909091	0.594145733859249
26	106	protein targeting to ER
GO:0072599	9.61149598680075e-37	84.4409090909091	0.594145733859249
26	106	establishment of protein localization to endoplasmic reticulum
GO:0006415	1.04706135830411e-36	96.7532467532468	0.510068507369732
25	91	translational termination
GO:0006413	8.51277798678427e-36	60.6950699939136	0.851982561760432
28	152	translational initiation
GO:0070972	2.16200471202676e-35	73.3652173913043	0.661407515050861
26	118	protein localization to endoplasmic reticulum
GO:0006414	6.04349422700425e-35	79.7433035714286	0.588540585426614

25	105	translational elongation
GO:0000956	1.07626458665364e-32	50.0985915492958	0.947270085115217
27	169	nuclear-transcribed mRNA catabolic process
GO:0019080	2.32797887638224e-32	53.872	0.846377413327797	26	151
viral genome expression
GO:0019083	2.32797887638224e-32	53.872	0.846377413327797	26	151
viral transcription
GO:0006612	3.3583026947795e-32	53.0161775232641	0.857587710193066
26	153	protein targeting to membrane
GO:0006402	4.75240277667812e-32	47.0827814569536	0.997716421008927
27	178	mRNA catabolic process
GO:0043624	1.73709570067169e-30	49.6721540178571	0.857587710193066
25	153	cellular protein complex disassembly
GO:0006401	2.597541189495e-30	39.8651685393258	1.14905542869006
27	205	RNA catabolic process
GO:0043241	4.08264984439921e-30	47.7879967776584	0.885613452356238
25	158	protein complex disassembly
GO:0034623	5.20334291210057e-29	42.6084611697028	0.975295827278389
25	174	cellular macromolecular complex disassembly
GO:0019058	7.32145164881366e-29	34.7205882352941	1.29478928793855
27	231	viral infectious cycle
GO:0072594	8.87435596441952e-29	37.6907048008172	1.14345028025742
26	204	establishment of protein localization to organelle
GO:0032984	1.09335072581424e-28	41.2105751391466	1.00332156944156
25	179	macromolecular complex disassembly
GO:0022415	1.92562465238357e-27	30.3369098712446	1.45733859248495
27	260	viral reproductive process
GO:0071845	5.81550425045238e-25	25.8570824524313	1.59186215486818
26	284	cellular component disassembly at cellular level
GO:0022411	8.38231358726506e-25	25.4551006245663	1.61428274859871
26	288	cellular component disassembly
GO:0016032	8.44656826549977e-25	19.4900097806343	2.52792194311812
30	451	viral reproduction
GO:0006605	1.83009408600185e-20	15.5738255033557	2.65684035706871
27	474	protein targeting
GO:0033365	2.65914396454758e-19	14.7243441122636	2.65123520863608
26	473	protein localization to organelle
GO:0042274	3.95076686206528e-08	72.6568825910931	0.10089267178742
5	18	ribosomal small subunit biogenesis
GO:0022613	4.75662848291033e-08	10.3949265687583	1.26115839734274
11	225	ribonucleoprotein complex biogenesis
GO:0071843	8.8068938230315e-08	9.74699248120301	1.33963047539963
11	239	cellular component biogenesis at cellular level
GO:0034660	5.16038454072388e-07	8.08425443169969	1.59746730330081
11	285	ncRNA metabolic process
GO:0042254	1.80178798662566e-06	11.0591664237832	0.835167116462529
8	149	ribosome biogenesis
GO:0006364	1.83261892452835e-06	13.9190582334912	0.58293543699398
7	104	rRNA processing
GO:0016072	2.6722829451218e-06	13.1027289425348	0.616566327589786
7	110	rRNA metabolic process
GO:0034470	1.5854029672272e-05	8.09246575342466	1.12102968652688
8	200	ncRNA processing
Tissue: Lung=>Thyroid	Type:	asymmetric
SourceGene:	RP11-321G12.1(ENSG00000259459.1)
TargetGeneSet:	FCRL5	FCRL2	FCRLA	SLAMF7	IGKJ4	IGKV4-1	IGKV1-9	IGKV3-15
IGKV1-17	IGKV3-20	IGKV2-28	IGKV2-30	IGKV1-39
IGKV3D-11	TMEM156	RP11	POU2AF1	TRAV10	IGHG2	IGHG3	IGHM	IGHJ6
IGHJ5	KIAA0125	IGHV6-1	IGHV4-4	IGHV3-7	IGHV3-11	IGHV3-23
IGHV3-30	IGHV4-39	IGHV1-46	IGHV1-69	CTD	UBE2I
TNFRSF17	TNFRSF13B	JSRP1	CD79A	IGLV1-51	IGLV1-44
IGLV3-25	IGLV2-23	IGLV3-19	IL2RG
GOBPID	Pvalue	OddsRatio	ExpCount	Count	Size	Term

GO:0006958 1.512271440273e-11 178.388429752066 0.0588194588609785
6 50 complement activation, classical pathway
GO:0006959 4.65871068408555e-11 87.1591304347826 0.143519479620787
7 122 humoral immune response
GO:0006956 6.41139492144295e-11 137.578947368421 0.0741125181648329
6 63 complement activation
GO:0002455 7.78389893721975e-11 132.896764252696 0.076465296519272
6 65 humoral immune response mediated by circulating immunoglobulin
GO:0072376 3.26329245860814e-10 103.047846889952 0.0964639125320047
6 82 protein activation cascade
GO:0002449 4.28173735817395e-10 62.44875 0.196456992595668 7
167 lymphocyte mediated immunity
GO:0016064 2.07038944420833e-09 74.4363636363636 0.130579198671372
6 111 immunoglobulin mediated immune response
GO:0002443 2.2006586756438e-09 48.828431372549 0.248218116393329 7
211 leukocyte mediated immunity
GO:0019724 2.4338007599768e-09 72.3535353535353 0.134108366203031
6 114 B cell mediated immunity
GO:0002253 2.46469607360309e-08 33.9020547945205 0.351740363988651
7 299 activation of immune response
GO:0002460 3.92217118292969e-08 44.4436363636364 0.212926441076742
6 181 adaptive immune response based on somatic recombination of immune receptors built from immunoglobulin superfamily domains
GO:0002250 6.69476686163987e-08 40.4602272727273 0.232925057089475
6 198 adaptive immune response
GO:0050778 9.50380640091185e-08 27.6019607843137 0.428205660507923
7 364 positive regulation of immune response
GO:0002252 1.41854204788672e-07 25.9591029023747 0.454086222406754
7 386 immune effector process
Tissue: Lung=>Thyroid Type: cluster
SourceGene: RP11-61I13.2(ENSG00000240126.1)
TargetGeneSet: TAS1R3 RPL22 ICMT RPL11 RPS8 RP4 RPL5 RP11
PDIA3P DUSP10 FAM177B RPS7 FNDC4 RPLP0P6 UBC SLC1A4 RPL31 RPL23AP7
PSMD14 WIPF1 ZNF804A RPL32 RPL15 RPL14 STAB1 PBRM1 RPL24 LRRC33
RPL35A CLNK PCDH7 RPL34 RAPGEF2 ST8SIA4 CAMLG CTB GNB2L1 EXOC2
RPL10A RPL39P3 SNHG5 UBE2J1 MRPS12 MBLAC1 RPS20 RPL30 LRRC14 FAM125B
RPL7A ADAMTSL2 CARD9 RPL13AP5 GSTO1 CTBP2 CTD RPS13
SLC37A2 PHB2 PPM1H RPL6 RB1 TM9SF2 GNG2 C14orf166 IRF2BPL
RPL3P4 PLCB2 GALK2 RPS3AP6 RSL1D1 RPS15A ADCY7 SNX20 RPL23A RPL19 HAP1
RPL27 MRPL10 C18orf21 RPL17 WDR7 HMHA1 ANO8 RPL18A UBA52
KCNN4 ALDH16A1 RPS11 RPL28 SIGLEC1 ZNFX1-AS1 EIF3D RPL3
ALG12 EIF2S3 CYBB WAS
GOBPID Pvalue OddsRatio ExpCount Count Size Term
GO:0006415 6.90789313649414e-46 142.44234728895 0.478582797038267 29
91 translational termination
GO:0006414 7.3412890508891e-46 124.347826086957 0.552210919659539
30 105 translational elongation
GO:0006614 8.82178008367986e-44 116.089305711086 0.552210919659539
29 105 SRP-dependent cotranslational protein targeting to membrane
GO:0006613 1.21058252063162e-43 114.573639126831 0.557470071275344
29 106 cotranslational protein targeting to membrane
GO:0045047 1.21058252063162e-43 114.573639126831 0.557470071275344
29 106 protein targeting to ER
GO:0072599 1.21058252063162e-43 114.573639126831 0.557470071275344
29 106 establishment of protein localization to endoplasmic reticulum
GO:0006413 2.45218234314814e-42 81.1522497704316 0.799391045602381
31 152 translational initiation
GO:0070972 4.18852763160563e-42 99.0423141286158 0.620579890665006
29 118 protein localization to endoplasmic reticulum
GO:0000184 5.52061833857549e-42 97.934988179669 0.625839042280811 29
119 nuclear-transcribed mRNA catabolic process, nonsense-mediated decay
GO:0006612 2.39608350147145e-40 75.5673382820785 0.804650197218186
30 153 protein targeting to membrane

GO:0019080 1.16117058049162e-38 72.0852807813045 0.794131893986575
29 151 viral genome expression
GO:0019083 1.16117058049162e-38 72.0852807813045 0.794131893986575
29 151 viral transcription
GO:0043624 1.75930892269342e-38 70.9126630061771 0.804650197218186
29 153 cellular protein complex disassembly
GO:0043241 4.8426714214098e-38 68.1401946231239 0.830945955297211
29 158 protein complex disassembly
GO:0000956 3.98029982689823e-37 62.7378419452888 0.888796623071068
29 169 nuclear-transcribed mRNA catabolic process
GO:0034623 9.86271865464611e-37 60.5531914893617 0.915092381150093
29 174 cellular macromolecular complex disassembly
GO:0006402 1.99659358825539e-36 58.9110381265172 0.936128987613314
29 178 mRNA catabolic process
GO:0032984 2.37502413074469e-36 58.5141843971631 0.941388139229119
29 179 macromolecular complex disassembly
GO:0019058 1.2853668530197e-34 45.9896171317326 1.21486402325099
30 231 viral infectious cycle
GO:0072594 1.32394683462339e-34 50.0668693009119 1.07286692962425
29 204 establishment of protein localization to organelle
GO:0006401 1.53718455382506e-34 49.7788926499033 1.07812608124005
29 205 RNA catabolic process
GO:0022415 5.12477291362589e-33 40.1086956521739 1.36737942010933
30 260 viral reproductive process
GO:0071845 2.77080103453608e-30 34.1660408844389 1.49359905888866
29 284 cellular component disassembly at cellular level
GO:0022411 4.19204585584231e-30 33.6288507352337 1.51463566535188
29 288 cellular component disassembly
GO:0016032 3.99163768454759e-27 22.8891534391534 2.37187737872812
31 451 viral reproduction
GO:0006605 1.82993938591163e-26 21.6650112866817 2.49283786589163
31 474 protein targeting
GO:0033365 3.64606300734513e-25 20.5103543036608 2.48757871427583
30 473 protein localization to organelle
GO:0022613 2.40552140418525e-08 11.198490294752 1.18330911355616 11
225 ribonucleoprotein complex biogenesis
GO:0071843 4.47372398009831e-08 10.5004723346829 1.25693723617743
11 239 cellular component biogenesis at cellular level
GO:0042254 8.1785039138301e-08 13.658315565032 0.783613590754965 9
149 ribosome biogenesis
GO:0042273 3.38648218066051e-07 99.7708333333333 0.0631098193896616
4 12 ribosomal large subunit biogenesis
GO:0006364 1.74693870538412e-05 12.4871720116618 0.546951768043734
6 104 rRNA processing
Tissue: Lung=>Whole_Blood Type: asymmetric
SourceGene: ALB(ENSG00000163631.11)
TargetGeneSet: C8A HHLA3 RP11 APCS CFHR2 CFHR5 APOB NAT8
RPL23AP7 SLC2A2 ALB AFM C9 DMGDH BHMT PLG FGL1
CYP2C18 CYP2C9 CTD APOA1 CMA1 SERPINA4 FOXA3 RP4 F9
GOBPID Pvalue OddsRatio ExpCount Count Size Term
GO:0006957 1.10269643018981e-06 206.956937799043 0.021313403916684
3 14 complement activation, alternative pathway
GO:0072376 6.51653803394752e-06 40.8860398860399 0.124835651512006
4 82 protein activation cascade
GO:0001907 6.63126733129481e-06 1442.8 0.00456715798214656 2 3
killing by symbiont of host cells
GO:0044004 6.63126733129481e-06 1442.8 0.00456715798214656 2 3
disruption by symbiont of host cell
GO:0006579 1.32502969607106e-05 721.35 0.00608954397619542 2 4
amino-acid betaine catabolic process
Tissue: Whole_Blood=>Lung Type: cluster
SourceGene: C9orf114(ENSG00000198917.6)
TargetGeneSet: VPS13D NFYC DENND2D SLC50A1 ASH1L PMF1 FCRL3 SLAMF6

CDC73 RP11 TMEM18 PDIA6 ADCY3 DYNC2LI1 C2orf27B METTL5
ABCB6 HDLBP TMEM43 CCR2 SLC26A6 TSC22D2 TRAM1L1 BRIX1 PHF15 TXNDC15 PDF
LARP1 CANX TRIM52 TXNDC5 E2F3 TOMM6 RP3 KDELR2 LSM5 IKZF1
ZKSCAN1 ORC5 CALU PDIA4 ECD RPP30 SFR1 SMNDC1 NUCB2 SPTBN2
CWF19L2 HYOU1 STT3A KIAA0528 FKBP11 NUP37 HSP90B1 SLC41A2 MLEC
ZNF26 GTF3A ESD POLE2 RDH11 LINC00341 COMMD4 SNHG9 CLEC16A
SERPINF2 ACAP1 SPAG5-AS1 KIAA0100 CTB AOC2 INO80C
LMAN1 CALR JUND PGPEP1 PCNA MIS18A RP4 PPARA CRELD2 NCAPH2
PTCHD1 AR MAGT1 TAF9B
GOBPID Pvalue OddsRatio ExpCount Count Size Term
GO:0006457 8.21351440005903e-07 10.2717391304348 1.03134731160473
9 216 protein folding
GO:0006487 4.90339324014159e-06 15.8316722037652 0.43927755864646
6 92 protein N-linked glycosylation
GO:0032075 1.52772707948791e-05 18.3414446721311 0.315133900768113
5 66 positive regulation of nuclease activity
Tissue: Lung=>Whole_Blood Type: asymmetric
SourceGene: CTB-85P21.2(ENSG00000259787.1)
TargetGeneSet: VAV3-AS1 CD48 CAPN2 ARPC2 ANKMY1 UBA3 COL8A1
PDGFRA TMSB4XP8 GTF2H5 TAS2R4 CTA C9orf103 TMSB4XP4
WDR34 TNKS2 RP11 FEN1 CRABP1 CLSTN3 BIN2 DOCK9 WDHD1 TIPIN
GINS3 SIRT6 PPIAP22 RRM2P3 BEX4
GOBPID Pvalue OddsRatio ExpCount Count Size Term
GO:0006260 1.98599441849477e-05 19.2409016838675 0.352363158258944
5 268 DNA replication
Tissue: Whole_Blood=>Lung Type: asymmetric
SourceGene: ENSG00000261008.2
TargetGeneSet: SMPDL3B CNTN2 NEK2 OR2L2 POU3F3 SYNPR MIR922 MAD2L1
BRCC3 SAPCD1 GRM4 MCM3 RPS17P13 PRRT4 DEFA4 DEFA1 DEFA1B
DEFA3 RP11 IFIT1B CALHM1 SIGIRR C11orf41 TCN1 MS4A3 MMP8
TMTC1 SVOP SKA3 TMX1 HERPUD1 MPO AZU1 ELANE CEACAM8 SHISA7 7SK
RP3
GOBPID Pvalue OddsRatio ExpCount Count Size Term
GO:0050832 1.23515231214828e-10 252.842105263158 0.0332156944156114
5 20 defense response to fungus
GO:0009620 1.86399749503958e-09 135.328947368421 0.0548058957857588
5 33 response to fungus
GO:0031640 8.53536891126382e-08 130.954545454545 0.0431804027402948
4 26 killing of cells of other organism
GO:0044364 8.53536891126382e-08 130.954545454545 0.0431804027402948
4 26 disruption of cells of other organism
GO:0009617 9.56888627265752e-07 17.3211589113257 0.567988374506954
7 342 response to bacterium
GO:0045073 1.13864338687663e-06 205.957142857143 0.0215902013701474
3 13 regulation of chemokine biosynthetic process
GO:0042033 1.44760299206345e-06 187.220779220779 0.023250986090928
3 14 chemokine biosynthetic process
GO:0050755 1.80753155153579e-06 171.607142857143 0.0249117708117085
3 15 chemokine metabolic process
GO:0035821 3.93630713736036e-06 46.3387096774194 0.109611791571518
4 66 modification of morphology or physiology of other organism
GO:0001906 4.70590517975697e-06 44.1907692307692 0.114594145733859
4 69 cell killing
GO:0045079 7.92234085030777e-06 1311.45454545455 0.00498235416234171
2 3 negative regulation of chemokine biosynthetic process
Tissue: Whole_Blood=>Lung Type: asymmetric
SourceGene: FHIT(ENSG00000189283.5)
TargetGeneSet: TAGLN2 FAM150B ADCY3 CTD FGF1 EBF1 CDCA7L CA3
CCNT1 RP11 KL MYL12A ACER1 ZNF121 GATSL3 SEC14L2 GUCY2F GPC3
MIR1184-2
GOBPID Pvalue OddsRatio ExpCount Count Size Term
GO:0045542 8.70129881364286e-06 801.888888888889 0.00484395543560999
2 5 positive regulation of cholesterol biosynthetic process

GO:0090205	1.30447221048509e-05	601.375 0.00581274652273199	2	6
positive regulation of cholesterol metabolic process
Tissue: Lung=>Whole_Blood	Type:	cluster
SourceGene:	GABRA1(ENSG00000022355.10)
TargetGeneSet:	MTND2P28	GABRD	C1orf173	KCNJ9	RP3	ASTN1	RP11
VSNL1	POU3F3	SCN2A	KIF1A	SLC6A1	ARPP21	MOBP	CAMKV	CADPS	SYNPR
CADM2	TAGLN3	GAP43	ZIC1	SOX2-OT	CXXC4	GRIA2	CTNND2	SPINK5	CAMK2A
TIMD4	GABRB2	CPLX2	NRSN1	BEND6	RIMS1	SNAP91	MYL7	VSTM2A	CALN1
FAM86B2	PSD3	PENK	STMN2	LY6H	SH3GL2	SLC25A16	GPR158	LGI1	INA
ANO3	SYT13	TMEM151A	SYT12	ELMOD1	OPCML	KCNA1	CNTN1	FAIM2
KCNC2	SYT1	SVOP	CTD	GABRB3	DDX11L9	CBLN1	MT3	VAT1L	EFNB3
PPP1R1B	GFAP	DSG1	ST8SIA3	CELF5	CASP14	UNC13A	VSTM2B	PNMAL1	SLC8A2
TTYH1	SYT5	PEG3	SNAP25	NCAM2	SULT4A1	FRMPD4	TCEAL5	PLP1	GRIA3
H2AFB2
GOBPID	Pvalue	OddsRatio	ExpCount	Count	Size	Term
GO:0048489	1.89882609211438e-09	41.5080611823067	0.219223583143035
7	48	synaptic vesicle transport
GO:0016079	3.91936183998243e-09	59.8375 0.137014739464397	6	30
synaptic vesicle exocytosis
GO:0006836	6.43090363346774e-08	17.7371854613234	0.543491799875441
8	119	neurotransmitter transport
GO:0001505	8.32494509922324e-08	17.1154422788606	0.561760431804027
8	123	regulation of neurotransmitter levels
GO:0007269	1.20939271320294e-07	21.4850890366874	0.392775586464605
7	86	neurotransmitter secretion
GO:0003001	1.26303098339968e-05	7.24052803017315	1.44322192235831
9	316	generation of a signal involved in cell-cell signaling
GO:0023061	1.26303098339968e-05	7.24052803017315	1.44322192235831
9	316	signal release
Tissue: Lung=>Whole_Blood	Type:	asymmetric
SourceGene:	GAD2(ENSG00000136750.7)
TargetGeneSet:	MTND2P28	GABRD	NPPA	NCDN	PRCC	RP3	CNTN2
TMEM178	POU3F3	KIF1A	ARPP21	MOBP	CAMKV	GAP43	CTNND2	MTND5P11
CAMK2A	GABRA6	GABRA1	CPLX2	GRM4	RIMS1	SNAP91	DGKB	CALN1	GPR22
KCND2	NEFM	RP11	ZER1	GRIN1	PTPN5	ANO3	SYT13	SYT12	ELMOD1
C11orf87	OPCML	PRMT8	FAIM2	KCNC2	SYT1	GABRB3	PPP1R1B	GFAP
ST8SIA3	CASP14	TTYH1	SNAP25	RIMS4	NCAM2	FRMPD4	TMEM35	PLP1	H2AFB2	MT
GOBPID	Pvalue	OddsRatio	ExpCount	Count	Size	Term
GO:0006836	1.19686617063823e-07	21.7850609756098	0.395266763545775
7	119	neurotransmitter transport
GO:0016079	2.75548337057949e-06	50.2692307692308	0.0996470832468341
4	30	synaptic vesicle exocytosis
GO:0001505	3.08159567979656e-06	17.4432234432234	0.40855304131202
6	123	regulation of neurotransmitter levels
GO:0007612	5.74622045437605e-06	22.8257406817458	0.259082416441769
5	78	learning
GO:0007611	6.79349737194285e-06	15.0984126984127	0.46834129126012
6	141	learning or memory
GO:0048167	6.92221761903358e-06	21.9201346389229	0.269047124766452
5	81	regulation of synaptic plasticity
GO:0007269	9.29306926751621e-06	20.5598621877692	0.285654971974258
5	86	neurotransmitter secretion
GO:0050890	1.12668599004808e-05	13.7596525096525	0.511521694000415
6	154	cognition
GO:0048489	1.87251837297196e-05	29.6673553719008	0.159435333194935
4	48	synaptic vesicle transport
Tissue: Lung=>Whole_Blood	Type:	cluster
SourceGene:	HIST1H3G(ENSG00000256018.1)
TargetGeneSet:	DPH5	KIF14	IGKJ5	IGKC	IGKJ4	IGKJ1	IGKV1-5	BUB1
C2orf76	ITM2C	RP11	MUM1	TUBB	KIFC1	TMEM217	BHLHA15	PNOC	GINS4	MCM4
ADRA2A	ZWINT	BLNK	FEN1	CDCA5	POU2AF1	IGHG4	IGHGP	IGHG3	IGHJ3
IGHV3-15	IGHV3-23	IGHV3-48	CCNB2	PLK1	SHCBP1	GINS2
AURKB	CDC6	TYMS	RP4	MYBL2	TOX2	UBE2C	CDC45	IGLV6-57

IGLV1-47 IGLV5-45 IGLV2-14 IGLV2-11 IGLV2-5 DERL3 EMID1

GOBPID	Pvalue	OddsRatio	ExpCount	Count	Size	Term
GO:0000280	3.56820526717671e-10	19.03232546706	0.847692201231749	11	350	nuclear division
GO:0007067	3.56820526717671e-10	19.03232546706	0.847692201231749	11	350	mitosis
GO:0000087	4.8108195251696e-10	18.4738538681948	0.871911978409799	11	360	M phase of mitotic cell cycle
GO:0048285	6.42971331608528e-10	17.9464948932219	0.896131755587849	11	370	organelle fission
GO:0051329	1.39906751290807e-08	15.2271002710027	0.917929555048094	10	379	interphase of mitotic cell cycle
GO:0006959	1.42054170376897e-08	31.0891304347826	0.29548128157221	7	122	humoral immune response
GO:0006271	1.63685597606566e-08	82.683908045977	0.0823472424053699	5	34	DNA strand elongation involved in DNA replication
GO:0051325	1.70633811698216e-08	14.8954907161804	0.937305376790533	10	387	interphase
GO:0022616	2.55089434850277e-08	74.9166666666667	0.0896131755587849	5	37	DNA strand elongation
GO:0010564	3.23201463478949e-08	13.8732673267327	1.00269877517127	10	414	regulation of cell cycle process
GO:0000075	8.77633934063887e-08	17.9575815131371	0.586118607708809	8	242	cell cycle checkpoint
GO:0006958	1.21229372537794e-07	53.2259259259259	0.12109888589025	5	50	complement activation, classical pathway
GO:0071156	1.71334688508739e-07	16.3888888888889	0.639402117500519	8	264	regulation of cell cycle arrest
GO:0051983	2.95970386617748e-07	92.8774193548387	0.0581274652273199	4	24	regulation of chromosome segregation
GO:0006956	3.93211835776371e-07	41.2586206896552	0.152584596221715	5	63	complement activation
GO:0002455	4.60480650898323e-07	39.8777777777778	0.157428551657325	5	65	humoral immune response mediated by circulating immunoglobulin
GO:0000084	7.39386892761105e-07	23.0948275862069	0.32454501418587	6	134	S phase of mitotic cell cycle
GO:0051301	9.05976370007049e-07	10.9183017885049	1.09473392844786	9	452	cell division
GO:0007059	9.56423966186795e-07	22.0514668039115	0.339076880492699	6	140	chromosome segregation
GO:0051320	1.08305274167737e-06	21.5640573873647	0.346342813646114	6	143	S phase
GO:0072376	1.47696702907015e-06	31.0367965367965	0.19860217286001	5	82	protein activation cascade
GO:0007050	2.15655126127038e-06	11.5358571868267	0.893709777870044	8	369	cell cycle arrest
GO:0006261	2.76009753716617e-06	27.1363636363636	0.225243927755865	5	93	DNA-dependent DNA replication
GO:0006260	3.00862339806096e-06	13.5584291187739	0.649090028371739	7	268	DNA replication
GO:0007017	3.44455065565553e-06	10.7982683982684	0.951837243097364	8	393	microtubule-based process
GO:0016064	6.59747971644911e-06	22.5	0.268839526676355	5	111	immunoglobulin mediated immune response
GO:0019724	7.51772815983643e-06	21.8761467889908	0.27610545982977	5	114	B cell mediated immunity
GO:0045786	7.52827705504051e-06	9.66036432703099	1.05840426268078	8	437	negative regulation of cell cycle
GO:0000083	1.94207783226368e-05	71.0378289473684	0.0532835097917099	3	22	regulation of transcription involved in G1/S phase of mitotic cell cycle

Tissue: Whole_Blood=>Lung Type: asymmetric

SourceGene: MMP3(ENSG00000149968.7)
TargetGeneSet: SDHDP6 RP11 PPA2 TMCO6 C6orf72 C8orf82 FBXL15 DDX23 TEP1 MPG SLC9A3R2 EIF3C ATP5D FAM108A1 ZNF358 FAM98C RABAC1 A1BG GATA5
GOBPID Pvalue OddsRatio ExpCount Count Size Term
GO:0014067 1.11832549800475e-05 656.090909090909 0.00539755034253685 2 6 negative regulation of phosphatidylinositol 3-kinase cascade
Tissue: Lung=>Whole_Blood Type: asymmetric
SourceGene: MOG(ENSG00000204655.7)
TargetGeneSet: MTND1P23 MTND2P28 RP11 RGS4 ARID4B-IT1 VSNL1 POU3F3 KIF1A ARPP21 CAMKV GABRB2 SNAP91 KCND2 DIRAS2 OPCML PRMT8 KCNC2 HIGD1B DYNLL2 UNC13A SNAP25 FEM1AP1 CTA FRMPD4 PLP1 GRIA3 H2AFB2
GOBPID Pvalue OddsRatio ExpCount Count Size Term
Tissue: Whole_Blood=>Lung Type: asymmetric
SourceGene: PAMR1(ENSG00000149090.7)
TargetGeneSet: RPS8 RP4 RPS7 UBC DUSP19 RPL32 RP11 EPHB1 SLC30A9 SEPP1 DIMT1 TMEM14B HIST1H2BJ HIST1H2AG DPCR1 SEPT10P1 ZMYND11 RPL27A RPL4 FTLP14 GCSH RPS7P1 RPL19 TIMP2 RPS15 RPS5 DUSP18 CTA RPL10

GOBPID	Pvalue	OddsRatio	ExpCount	Count	Size	Term
GO:0006415	6.30555352061827e-17	136.24881291548	0.144834267524739	10	91	translational termination
GO:0006414	2.79796175290637e-16	116.056680161943	0.167116462528545	10	105	translational elongation
GO:0006614	2.79796175290637e-16	116.056680161943	0.167116462528545	10	105	SRP-dependent cotranslational protein targeting to membrane
GO:0006613	3.08687413255169e-16	114.839743589744	0.168708047885959	10	106	cotranslational protein targeting to membrane
GO:0045047	3.08687413255169e-16	114.839743589744	0.168708047885959	10	106	protein targeting to ER
GO:0072599	3.08687413255169e-16	114.839743589744	0.168708047885959	10	106	establishment of protein localization to endoplasmic reticulum
GO:0070972	9.35135241104897e-16	101.994301994302	0.187807072174936	10	118	protein localization to endoplasmic reticulum
GO:0000184	1.02008682585083e-15	101.051517290049	0.189398657532351	10	119	nuclear-transcribed mRNA catabolic process, nonsense-mediated decay
GO:0019080	1.16958569994575e-14	77.9432624113475	0.240329388969621	10	151	viral genome expression
GO:0019083	1.16958569994575e-14	77.9432624113475	0.240329388969621	10	151	viral transcription
GO:0006413	1.25091808835343e-14	77.3889490790899	0.241920974327036	10	152	translational initiation
GO:0006612	1.33729070090369e-14	76.8423883808499	0.243512559684451	10	153	protein targeting to membrane
GO:0043624	1.33729070090369e-14	76.8423883808499	0.243512559684451	10	153	cellular protein complex disassembly
GO:0043241	1.85483238175808e-14	74.2203742203742	0.251470486471524	10	158	protein complex disassembly
GO:0000956	3.6731013503907e-14	69.0324141267537	0.268977925403086	10	169	nuclear-transcribed mRNA catabolic process
GO:0034623	4.93531947415089e-14	66.9043151969981	0.27693585219016	10	174	cellular macromolecular complex disassembly
GO:0006402	6.21163297175937e-14	65.2930402930403	0.283302193619819	10	178	mRNA catabolic process
GO:0032984	6.57374934802155e-14	64.9021392808375	0.284893778977233	10	179	macromolecular complex disassembly
GO:0071845	1.52770223589797e-13	47.5289987789988	0.452010241505778	11	284	cellular component disassembly at cellular level
GO:0022411	1.78117443726344e-13	46.8294223826715	0.458376582935437	11	288	cellular component disassembly
GO:0072594	2.45722623718009e-13	56.4393338620143	0.324683412912601	10	204	establishment of protein localization to organelle

GO:0006401	2.58110034705589e-13	56.1459566074951	0.326274998270016
10	205	RNA catabolic process
GO:0019058	8.55234752637089e-13	49.4500522102332	0.367656217562798
10	231	viral infectious cycle
GO:0022415	2.78571752229051e-12	43.6246153846154	0.413812192927825
10	260	viral reproductive process
GO:0016032	6.31199390872842e-10	24.397348683063 0.717804996194035	10
451	viral reproduction
GO:0033365	1.00237370241577e-09	23.2015284931052	0.752819874057159
10	473	protein localization to organelle
GO:0006605	1.02311616145228e-09	23.1498673740053	0.754411459414573
10	474	protein targeting
GO:0042274	2.82937347018751e-06	144.13	0.0286485364334648	3	18
ribosomal small subunit biogenesis
Tissue: Whole_Blood=>Lung	Type:	asymmetric
SourceGene:	RAB1C(ENSG00000233111.1)
TargetGeneSet:	TMEM222 RPS8	RP11	TMEM42	LTF	HCLS1	SPARCL1 CCNA2
HMGB2	CTD	HMMR	BOD1	BTNL9	KIFC1	CTSB	CDCA2	ESCO2	IL2RA
RBM17	ITGA8	SRGN	CCDC147 NCAPD2	C12orf57	CMAS	AGAP2	TMCC3
SELPLG	RP1	NUSAP1	BLM	SLC5A2	NKD1	TRAPPC2L	RPL19	BRCA1
RPS7P11 RFNG	SLC16A3 RPL18	RPL28	RPS5	TRIB3	MYLK2	CBLN4	MIOX	BEX2
GOBPID	Pvalue	OddsRatio	ExpCount	Count	Size	Term
GO:0006415	3.77538272921322e-06	25.2413671599718	0.239291398519134
5	91	translational termination
GO:0006414	7.63268820961873e-06	21.6863636363636	0.27610545982977
5	105	translational elongation
GO:0006614	7.63268820961873e-06	21.6863636363636	0.27610545982977
5	105	SRP-dependent cotranslational protein targeting to membrane
GO:0006613	7.99528987057395e-06	21.4701470147015	0.278735035637672
5	106	cotranslational protein targeting to membrane
GO:0045047	7.99528987057395e-06	21.4701470147015	0.278735035637672
5	106	protein targeting to ER
GO:0072599	7.99528987057395e-06	21.4701470147015	0.278735035637672
5	106	establishment of protein localization to endoplasmic reticulum
GO:0070972	1.34915910055372e-05	19.1740412979351	0.310289945332503
5	118	protein localization to endoplasmic reticulum
GO:0000184	1.40565085719009e-05	19.0045188729399	0.312919521140405
5	119	nuclear-transcribed mRNA catabolic process, nonsense-mediated decay
Tissue: Whole_Blood=>Lung	Type:	asymmetric
SourceGene:	RP11-369K16.1(ENSG00000251468.2)
TargetGeneSet:	ANAPC10P1	PMF1	NUF2	TNFSF13B	CCDC150 RP11
CEP19	CDC25C	RPL29P14	FTH1P15 PBK	ESPL1	TIMELESS	RFC3	PRC1
CTD	AURKB	UNK	AP5S1	GSTT1	NFAM1	SCML2	VDAC1P6 RBMY1KP
GOBPID	Pvalue	OddsRatio	ExpCount	Count	Size	Term
GO:0000280	2.50766476846914e-08	35.9515306122449	0.363296657670749
7	350	nuclear division
GO:0007067	2.50766476846914e-08	35.9515306122449	0.363296657670749
7	350	mitosis
GO:0000087	3.04437575889836e-08	34.9082861189802	0.373676562175628
7	360	M phase of mitotic cell cycle
GO:0048285	3.67569432426881e-08	33.922520661157 0.384056466680507	7
370	organelle fission
GO:0051301	1.44801125328211e-07	27.510393258427 0.469171683620511	7
452	cell division
GO:0007059	1.06022401963643e-05	38.2352941176471	0.1453186630683 4
140	chromosome segregation
Tissue: Whole_Blood=>Lung	Type:	cluster
SourceGene:	SORT1(ENSG00000134243.7)
TargetGeneSet:	TMEM51	SPEN	RP11	METTL13 GPR137B ERO1LB	SCN7A	CERS6
GOLGB1	KLF15	C4orf50 SPARCL1 LRRC16A RP3	KHDRBS2 HINT3	ADCY1	UPP1	POP1
AQP7P3	RHOBTB1 MICU1	SLC16A12	TMEM133 ITFG2	METTL7A IRAK3	NT5DC3
RAB35	FLT3	FOXO1	DNAJC3	PCNX	MGA	CHD9	MT1L	MT1M	MT1A	MT1X
GLOD4	TNFSF12 PNMT	MTMR4	NOTCH3	HIF3A	SAMHD1	TTPAL	NCOA3	KCNB1	H1F0

MAGED2 RP6 MAGEC3
GOBPID Pvalue OddsRatio ExpCount Count Size Term
GO:0071294 3.07587567160006e-06 145.515151515152 0.0298941249740502
3 12 cellular response to zinc ion
Tissue: Lung=>Whole_Blood Type: asymmetric
SourceGene: WDR45L(ENSG00000141580.10)
TargetGeneSet: MTND1P23 MTND2P28 SPSB1 ANGPTL7 RP11 BCAN
KCNJ10 HNRNPU HADH CIDEC FOXP1 TAGLN3 AP2M1 ADIPOQ BRD9 GABRA1
GABRP HIST1H4H COL12A1 HRCT1 DPP7 TPP1 MTRNR2L8 C11orf31
PACS1 TSKU CRABP1 RP1 IGFBP6 PCBP2 NME3 CTA NFATC3 SERPINF1
AP2B1 UBTF GFAP ARL16 SMARCA4 NUCB1-AS1 ADAM33 WISP2 MYH9
EIF2S3 NONO USP26
GOBPID Pvalue OddsRatio ExpCount Count Size Term
GO:0050688 1.45278098400454e-05 31.8756936736959 0.149885821050446
4 57 regulation of defense response to virus
Tissue: Whole_Blood=>Lung Type: cluster
SourceGene: all_source
TargetGeneSet: ARL6IP5 SEC31B DYNLL2 CDC42SE1 PSTPIP2 CATSPER1 RP11
OXT SORT1 WDR69 UCN C9orf114
GOBPID Pvalue OddsRatio ExpCount Count Size Term
GO:0042756 1.2041283328032e-05 601.458333333333 0.00553594906926856
2 8 drinking behavior
Tissue: Heart_Left_Ventricle=>Muscle_Skeletal Type: cluster
SourceGene: CTD-2292M16.7(ENSG00000258459.1)
TargetGeneSet: C1orf130 ZCCHC17 FABP3 MRPS15 DPH2 BTBD8 ANKRD20A14P
ATP1A2 TIMM17A SOX13 EML4 FBXO11 MDH1 SLC4A4 RPIA COX5B TXNDC9
C2orf29 RANBP2 NDUFB3 FAM117B THUMPD3 CRTAP RP11 ACVR2B MYL3 NDUFB4
SRPRB MRPL47 MRPS18C CNGB1 NDUFC1 CMBL JMY TMEM167A TTC37
UQCRQ CNOT6 MRPS18B RRP36 CUL7 PHF3 SNAP91 BACH2 RTN4IP1 WISP3
NDUFA4 GGCT CALU MRPS33 MLL3 TNKS VPS37A MRPL15 UQCRB COX6C
C9orf80 C9orf91 MRPL41 SVIL PTPN20C MYPN PPIF MMS19 BCCIP TMX2
LTBP2 NUMA1 SPCS2 TMEM126A C11orf73 ATP5L DNM1L DUSP6
SLC25A3 SRRM4 SLC25A15 RALGAPA1 GLRX5 DYNC1H1 FAM96A UACA
COX5A ETFA TSC2 PKD1 7SK ZNF771 CIAPIN1 COQ9 COX4NB C1QBP
SRP68P3 SUPT6H ETV4 MRPL27 TACO1 ABCA5 ATP5H SLC25A19 WDR45L
NDUFV2 NAPG C18orf32 ACAA2 NEDD4L ABCA7 UQCR11 ITGB1BP3
TIMM44 GADD45GIP1 ILVBL COX6B1 RYR1 EIF3K MRPS12 TBC1D17 SNORA51
TTC28 UQCR10 SLC16A8 RPS19BP1 NDUFA6 CLCN4 DMD SLC9A7 RP1
RAP2C MCF2 FUNDC2
GOBPID Pvalue OddsRatio ExpCount Count Size Term
GO:0022904 5.09756556714145e-19 33.0004083299306 0.73074527714345
17 96 respiratory electron transport chain
GO:0045333 2.24818676451834e-17 22.4321528751753 1.08089405577469
18 142 cellular respiration
GO:0022900 1.82875161660439e-16 22.2229574487639 1.01999861601273
17 134 electron transport chain
GO:0015980 1.59850933047265e-12 10.187996031746 2.33686250086499 19
307 energy derivation by oxidation of organic compounds
GO:0006091 6.56007166244477e-11 7.58877995642702 3.25790602726455
20 428 generation of precursor metabolites and energy
GO:0042773 2.14085965668616e-09 28.7621920563097 0.357760708601481
8 47 ATP synthesis coupled electron transport
GO:0042775 2.14085965668616e-09 28.7621920563097 0.357760708601481
8 47 mitochondrial ATP synthesis coupled electron transport
GO:0006119 1.2177092509027e-08 22.4172549019608 0.441491938274168
8 58 oxidative phosphorylation
GO:0006120 2.74257268012291e-07 27.5211538461538 0.274029478928794
6 36 mitochondrial electron transport, NADH to ubiquinone
Tissue: Heart_Left_Ventricle=>Muscle_Skeletal Type: cluster
SourceGene: DEFB1(ENSG00000164825.3)
TargetGeneSet: RPL22 C1QA C1QB RPS8 RP11 FNBP1L TKT RGL1 SWT1
SRGAP3 SPTBN1 UBR3 FBLN2 RPL29 ABI3BP RPL24 PSMD2 RPL35A RPL34
RPS3A CTD PCDHB10 CSF1R CTB SLIT1 ADAMTS2 GFPT2 F13A1 RPL15P3

RPS10 RPL10A CLPS NT5E MRPS12 FNDC1 GPNMB TRIP6 IRF5 RPL7
MATN2 NTRK2 RPL12 RPL7A MRC1 ALOX5 TACR2 MYOF ZNF195 RPL27A
RPS25 CD4 PFDN5 LRP1 GLIPR1 RPL6 GPC6 U6 RSL24D1 RPS3AP6 CILP
CORO2B ISLR ANPEP LRRK1 USP7 PIEZO1 RPL13 RPL26 PSMD3 CHAD
TIMP2 RPL17 PRTN3 HRC WISP2 ARSD-AS1 GPR64 RPS4X DOCK11 ELF4
GOBPID Pvalue OddsRatio ExpCount Count Size Term
GO:0006415 1.87298754884005e-26 71.2350628930818 0.453394228773095
19 91 translational termination
GO:0006414 3.60489204692822e-25 59.5802983764809 0.523147187045879
19 105 translational elongation
GO:0006614 3.60489204692822e-25 59.5802983764809 0.523147187045879
19 105 SRP-dependent cotranslational protein targeting to membrane
GO:0006613 4.3767684978085e-25 58.8913467794405 0.528129541208221
19 106 cotranslational protein targeting to membrane
GO:0045047 4.3767684978085e-25 58.8913467794405 0.528129541208221
19 106 protein targeting to ER
GO:0072599 4.3767684978085e-25 58.8913467794405 0.528129541208221
19 106 establishment of protein localization to endoplasmic reticulum
GO:0070972 3.87288690757928e-24 51.7095483133219 0.587917791156321
19 118 protein localization to endoplasmic reticulum
GO:0000184 4.59256530536703e-24 51.1888679245283 0.592900145318663
19 119 nuclear-transcribed mRNA catabolic process, nonsense-mediated decay
GO:0006612 1.70868344544392e-23 41.1972238288028 0.762300186838281
20 153 protein targeting to membrane
GO:0043624 1.70868344544392e-23 41.1972238288028 0.762300186838281
20 153 cellular protein complex disassembly
GO:0043241 3.33498848053864e-23 39.690635451505 0.78721195764999 20
158 protein complex disassembly
GO:0034623 2.44910218365854e-22 35.526973026973 0.866929624247457 20
174 cellular macromolecular complex disassembly
GO:0032984 4.38389985809079e-22 34.3976777939042 0.891841395059165
20 179 macromolecular complex disassembly
GO:0019080 5.30012675642439e-22 38.692538593482 0.752335478513598 19
151 viral genome expression
GO:0019083 5.30012675642439e-22 38.692538593482 0.752335478513598 19
151 viral transcription
GO:0006413 6.03599514109242e-22 38.3989218328841 0.757317832675939
19 152 translational initiation
GO:0000956 4.81784431727102e-21 34.0064150943396 0.842017853435748
19 169 nuclear-transcribed mRNA catabolic process
GO:0071845 9.37713762928254e-21 23.7079389312977 1.41498858210504
22 284 cellular component disassembly at cellular level
GO:0022411 1.27352754784725e-20 23.3448120300752 1.43491799875441
22 288 cellular component disassembly
GO:0006402 1.32172620845689e-20 32.061231755073 0.886859040896824 19
178 mRNA catabolic process
GO:0019058 7.75447417905815e-20 25.8257382427999 1.15092381150093
20 231 viral infectious cycle
GO:0072594 1.83307320726844e-19 27.5049464558899 1.01640024911771
19 204 establishment of protein localization to organelle
GO:0006401 2.01319926029769e-19 27.3551430310408 1.02138260328005
19 205 RNA catabolic process
GO:0022415 8.22254859659035e-19 22.6586538461538 1.29541208220884
20 260 viral reproductive process
GO:0016032 1.91219579250783e-16 14.3076923076923 2.24704172721611
22 451 viral reproduction
GO:0033365 6.926931938195e-15 12.6872722540344 2.35665351878763
21 473 protein localization to organelle
GO:0006605 7.22293049273841e-15 12.6583560576548 2.36163587294997
21 474 protein targeting
GO:0071843 2.73842229720722e-06 8.78819875776398 1.19078264479967
9 239 cellular component biogenesis at cellular level
GO:0042254 9.04735715293693e-06 10.7972914409534 0.742370770188914

7	149	ribosome biogenesis
GO:0022613	1.54948743400183e-05	8.15783410138249	1.12102968652688
8	225	ribonucleoprotein complex biogenesis
Tissue: Heart_Left_Ventricle=>Muscle_Skeletal	Type:	asymmetric
SourceGene:	ELMO1-AS1(ENSG00000224101.1)
TargetGeneSet:	SLC2A5	MIR186	MFSD2B	SFTPB	RAX	VILL	PILRA	SFTPC
FAM163B	LRRC18	SFTPA2	SFTPA1	SFTPD	HBB	CTD	HBA1	RP3	ALAS2
GOBPID	Pvalue	OddsRatio	ExpCount	Count	Size	Term
GO:0007585	5.63941214389412e-10	195.54347826087	0.0458791779115632	5
51	respiratory gaseous exchange
GO:0071732	1.56486111333871e-05	524.836363636364	0.00629714206629299
2	7	cellular response to nitric oxide
Tissue: Heart_Left_Ventricle=>Muscle_Skeletal	Type:	asymmetric
SourceGene:	ENSG00000232970.1
TargetGeneSet:	RP11	TTLL7	GBP5	CSDE1	LCE1A	NR1I3	EBF3	DYSF
COX5B	FASTKD1	C2orf88	CCDC150	CCDC58	PLS1	MB21D2	HAUS3	SDHA	SDHAP3
COX7C	C6orf136	UQCRB	PTPDC1	OLFML2A	MRPL41	VDAC2	COMTD1	GOT1
FBXL15	NRAP	ARNTL	ATP5L	USP5	B4GALNT1	SLC25A3	C15orf57
COX5A	IDH3A	PAQR4	C16orf62	UQCRC2	MLYCD	RILP	ASPA	PSMD11
C18orf25	EIF3K	RP4	SLMO2	DDT	KIAA0930	USP11	RPL23AP83
GOBPID	Pvalue	OddsRatio	ExpCount	Count	Size	Term
GO:0045333	3.75947048454087e-11	37.1737998843262	0.343920835928309
9	142	cellular respiration
GO:0022904	2.64221223061685e-09	40.2443820224719	0.23250986090928
7	96	respiratory electron transport chain
GO:0022900	2.7271926015933e-08	28.1279527559055	0.32454501418587
7	134	electron transport chain
GO:0015980	3.40707675026016e-08	16.3993288590604	0.743547159366134
9	307	energy derivation by oxidation of organic compounds
GO:0006091	4.42283451323363e-08	13.3952153110048	1.03660646322054
10	428	generation of precursor metabolites and energy
GO:0009060	3.02273220774707e-06	48.8217317487267	0.10172306414781
4	42	aerobic respiration
Tissue: Muscle_Skeletal=>Heart_Left_Ventricle	Type:	asymmetric
SourceGene:	ENSG00000243454.1
TargetGeneSet:	ZBTB48	COL24A1	BNIPL	TRAPPC12-AS1	COL3A1	COL5A2	FILIP1L	RP11
CCDC80	KIT	PDE5A	FAM198B	LPCAT1	PCDHGC5	MAPK14	EPB41L2	NFE2L3	CTHRC1
COL14A1	OGN	ASPN	ECM2	ANKS6	COL5A1	CDH23	POLL	LRRC55	LUM
DACT1	C17orf62	ZNF600	ZNF525	C20orf202	CDH26	CAPN6
GOBPID	Pvalue	OddsRatio	ExpCount	Count	Size	Term
GO:0030199	4.28224736216947e-09	111.762422360248	0.0639402117500519
5	33	collagen fibril organization
GO:0030198	1.81828795030155e-08	30.8852813852814	0.311950730053283
7	161	extracellular matrix organization
GO:0043062	1.89797129607994e-08	30.6838709677419	0.313888312227527
7	162	extracellular structure organization
Tissue: Muscle_Skeletal=>Heart_Left_Ventricle	Type:	asymmetric
SourceGene:	ENSG00000256240.1
TargetGeneSet:	PRPF38A	MIR4426	NFU1	RPL37A	RP11	RPS23	CTD	TRIM27
ANKRD20A4	NDUFA8	RPS13	RPS29	RPL38	RPS21	GPM6B
GOBPID	Pvalue	OddsRatio	ExpCount	Count	Size	Term
GO:0006415	4.7286271313409e-11	168.870588235294	0.0755657047955159
6	91	translational termination
GO:0006414	1.13596912439202e-10	144.848484848485	0.0871911978409799
6	105	translational elongation
GO:0006614	1.13596912439202e-10	144.848484848485	0.0871911978409799
6	105	SRP-dependent cotranslational protein targeting to membrane
GO:0006613	1.20369630057904e-10	143.39	0.0880215902013702	6	106
cotranslational protein targeting to membrane
GO:0045047	1.20369630057904e-10	143.39	0.0880215902013702	6	106
protein targeting to ER
GO:0072599	1.20369630057904e-10	143.39	0.0880215902013702	6	106
establishment of protein localization to endoplasmic reticulum

GO:0070972 2.31510341395467e-10 127.919642857143 0.0979862985260536
6 118 protein localization to endoplasmic reticulum
GO:0000184 2.43715605442804e-10 126.778761061947 0.0988166908864438
6 119 nuclear-transcribed mRNA catabolic process, nonsense-mediated decay
GO:0019080 1.03374025467577e-09 98.5793103448276 0.125389246418933
6 151 viral genome expression
GO:0019083 1.03374025467577e-09 98.5793103448276 0.125389246418933
6 151 viral transcription
GO:0006413 1.07583659143465e-09 97.8972602739726 0.126219638779323
6 152 translational initiation
GO:0006612 1.11934651153124e-09 97.2244897959184 0.127050031139714
6 153 protein targeting to membrane
GO:0043624 1.11934651153124e-09 97.2244897959184 0.127050031139714
6 153 cellular protein complex disassembly
GO:0043241 1.35943709635315e-09 93.9934210526316 0.131201992941665
6 158 protein complex disassembly
GO:0000956 2.04064203149548e-09 87.5828220858896 0.140336308905958
6 169 nuclear-transcribed mRNA catabolic process
GO:0034623 2.43273019696167e-09 84.9464285714286 0.144488270707909
6 174 cellular macromolecular complex disassembly
GO:0006402 2.78967451431039e-09 82.9476744186046 0.147809840149471
6 178 mRNA catabolic process
GO:0032984 2.885389111653e-09 82.4624277456647 0.148640232509861
6 179 macromolecular complex disassembly
GO:0072594 6.33155322571186e-09 71.9242424242424 0.169400041519618
6 204 establishment of protein localization to organelle
GO:0006401 6.52010748867559e-09 71.5577889447236 0.170230433880008
6 205 RNA catabolic process
GO:0019058 1.33344527646106e-08 63.1733333333333 0.191820635250156
6 231 viral infectious cycle
GO:0022415 2.70267879691018e-08 55.8464566929134 0.215902013701474
6 260 viral reproductive process
GO:0071845 4.57347393629197e-08 50.9388489208633 0.235831430350841
6 284 cellular component disassembly at cellular level
GO:0022411 4.97033799076331e-08 50.2021276595745 0.239152999792402
6 288 cellular component disassembly
GO:0016032 7.04124660609916e-07 31.447191011236 0.374506954536018 6
451 viral reproduction
GO:0033365 9.30996519068124e-07 29.9186295503212 0.392775586464605
6 473 protein localization to organelle
GO:0006605 9.42588157413463e-07 29.8525641025641 0.393605978824995
6 474 protein targeting
Tissue: Muscle_Skeletal=>Heart_Left_Ventricle Type: asymmetric
SourceGene: HTR7(ENSG00000148680.10)
TargetGeneSet: RPL11 RP11 PSMD4 UBC GNLY EEF1B2 RPS3A BTF3 CTB
RPS10 COPS6 RPL30 HAS2 PSMB7 URM1 GSTO1 TALDO1 RPLP2 RPS13
RPL14P1 CYP51P2 RPS3AP6 PSMB6 NLGN2 RPL23A RPL17 PSMC5 TTC39C ZNF121
KANK2 ZNF69 UBA52 RPL13A HSD17B10 CSAG1
GOBPID Pvalue OddsRatio ExpCount Count Size Term
GO:0006414 8.79632144056685e-19 102.709677419355 0.21797799460245
12 105 translational elongation
GO:0006415 1.63837225706366e-17 103.783552631579 0.18891426198879
11 91 translational termination
GO:0006614 8.48006399289468e-17 88.2402015677492 0.21797799460245
11 105 SRP-dependent cotranslational protein targeting to membrane
GO:0006613 9.45049397256069e-17 87.3052631578947 0.220053975503425
11 106 cotranslational protein targeting to membrane
GO:0045047 9.45049397256069e-17 87.3052631578947 0.220053975503425
11 106 protein targeting to ER
GO:0072599 9.45049397256069e-17 87.3052631578947 0.220053975503425
11 106 establishment of protein localization to endoplasmic reticulum
GO:0070972 3.20574582608876e-16 77.4490900147565 0.244965746315134
11 118 protein localization to endoplasmic reticulum

GO:0000184	3.5279711938347e-16	76.7266081871345	0.24704172721611
11	119	nuclear-transcribed mRNA catabolic process, nonsense-mediated decay
GO:0016032	2.08323179173028e-15	32.0756880733945	0.936267386340046
15	451	viral reproduction
GO:0019080	5.16753494456863e-15	59.0567669172932	0.313473116047332
11	151	viral genome expression
GO:0019083	5.16753494456863e-15	59.0567669172932	0.313473116047332
11	151	viral transcription
GO:0006413	5.56390451368096e-15	58.6338185890258	0.315549096948308
11	152	translational initiation
GO:0006612	5.98762366075637e-15	58.2168272794663	0.317625077849284
11	153	protein targeting to membrane
GO:0043624	5.98762366075637e-15	58.2168272794663	0.317625077849284
11	153	cellular protein complex disassembly
GO:0043241	8.57846919587909e-15	56.2169709989259	0.328004982354162
11	158	protein complex disassembly
GO:0000956	1.81720686333755e-14	52.2628247834777	0.350840772264895
11	169	nuclear-transcribed mRNA catabolic process
GO:0034623	2.51352674312269e-14	50.6419115272845	0.361220676769774
11	174	cellular macromolecular complex disassembly
GO:0006402	3.23563366621432e-14	49.4150646076269	0.369524600373677
11	178	mRNA catabolic process
GO:0032984	3.44329372861052e-14	49.1174812030075	0.371600581274652
11	179	macromolecular complex disassembly
GO:0072594	1.46307605867581e-13	42.6801199890919	0.423500103799045
11	204	establishment of protein localization to organelle
GO:0006401	1.54414914668809e-13	42.4571351058058	0.425576084700021
11	205	RNA catabolic process
GO:0019058	5.73980054119701e-13	37.3710526315789	0.479551588125389
11	231	viral infectious cycle
GO:0022415	2.09096768632073e-12	32.9511731135067	0.539755034253685
11	260	viral reproductive process
GO:0071845	5.46196938724066e-12	30.0034702139965	0.589578575877102
11	284	cellular component disassembly at cellular level
GO:0022411	6.35672814117802e-12	29.5618468554057	0.597882499481005
11	288	cellular component disassembly
GO:2000045	1.02214241136263e-09	47.4021739130435	0.205522109196595
7	99	regulation of G1/S transition of mitotic cell cycle
GO:0033365	1.28085356945784e-09	17.4924812030075	0.981938966161511
11	473	protein localization to organelle
GO:0006605	1.30966144978202e-09	17.4534500397863	0.984014947062487
11	474	protein targeting
GO:0006977	3.91936183998244e-09	59.8375 0.137014739464397	6	66
DNA damage response, signal transduction by p53 class mediator resulting in cell cycle arrest
GO:0072413	3.91936183998244e-09	59.8375 0.137014739464397	6	66
signal transduction involved in mitotic cell cycle checkpoint
GO:0072431	3.91936183998244e-09	59.8375 0.137014739464397	6	66
signal transduction involved in mitotic cell cycle G1/S transition DNA damage checkpoint
GO:0072474	3.91936183998244e-09	59.8375 0.137014739464397	6	66
signal transduction involved in mitotic cell cycle G1/S checkpoint
GO:0051436	4.29873094196168e-09	58.8524590163934	0.139090720365373
6	67	negative regulation of ubiquitin-protein ligase activity involved in mitotic cell cycle
GO:0072401	4.29873094196168e-09	58.8524590163934	0.139090720365373
6	67	signal transduction involved in DNA integrity checkpoint
GO:0072404	4.29873094196168e-09	58.8524590163934	0.139090720365373
6	67	signal transduction involved in G1/S transition checkpoint
GO:0072422	4.29873094196168e-09	58.8524590163934	0.139090720365373
6	67	signal transduction involved in DNA damage checkpoint
GO:0072395	4.70801029401529e-09	57.8991935483871	0.141166701266348
6	68	signal transduction involved in cell cycle checkpoint

GO:0051352	6.68216269281093e-09	54.375	0.149470624870251	6	72	negative regulation of ligase activity
GO:0051437	6.68216269281093e-09	54.375	0.149470624870251	6	72	positive regulation of ubiquitin-protein ligase activity involved in mitotic cell cycle
GO:0051444	6.68216269281093e-09	54.375	0.149470624870251	6	72	negative regulation of ubiquitin-protein ligase activity
GO:0031571	7.27017602897557e-09	53.5597014925373	0.151546605771227	6	73	mitotic cell cycle G1/S transition DNA damage checkpoint
GO:0002479	8.57510599745578e-09	52	0.155698567573178	6	75	antigen processing and presentation of exogenous peptide antigen via MHC class I, TAP-dependent
GO:0051439	1.08915271556663e-08	49.8229166666667	0.161926510276105	6	78	regulation of ubiquitin-protein ligase activity involved in mitotic cell cycle
GO:0000216	1.17698986002428e-08	49.1369863013699	0.164002491177081	6	79	M/G1 transition of mitotic cell cycle
GO:0042590	1.17698986002428e-08	49.1369863013699	0.164002491177081	6	79	antigen processing and presentation of exogenous peptide antigen via MHC class I
GO:0071158	1.27060535538087e-08	48.4695945945946	0.166078472078057	6	80	positive regulation of cell cycle arrest
GO:0031575	1.47636597233999e-08	47.1875	0.170230433880008	6	82	mitotic cell cycle G1/S transition checkpoint
GO:0051443	1.47636597233999e-08	47.1875	0.170230433880008	6	82	positive regulation of ubiquitin-protein ligase activity
GO:0002478	1.58913587152108e-08	46.5714285714286	0.172306414780984	6	83	antigen processing and presentation of exogenous peptide antigen
GO:0031145	1.58913587152108e-08	46.5714285714286	0.172306414780984	6	83	anaphase-promoting complex-dependent proteasomal ubiquitin-dependent protein catabolic process
GO:2000602	1.63425650901189e-08	31.045652173913	0.305169192443429	7	147	regulation of interphase of mitotic cell cycle
GO:0019884	1.83610184479699e-08	45.3860759493671	0.176458376582935	6	85	antigen processing and presentation of exogenous antigen
GO:0051351	1.97099177666051e-08	44.815625	0.178534357483911	6	86	positive regulation of ligase activity
GO:0071779	1.97099177666051e-08	44.815625	0.178534357483911	6	86	G1/S transition checkpoint
GO:0031397	2.96382080598767e-08	41.671511627907	0.190990242889765	6	92	negative regulation of protein ubiquitination
GO:0051438	3.83125302602106e-08	39.8083333333333	0.199294166493668	6	96	regulation of ubiquitin-protein ligase activity
GO:0002474	4.61104909350121e-08	38.5161290322581	0.205522109196595	6	99	antigen processing and presentation of peptide antigen via MHC class I
GO:0051340	4.89836579529392e-08	38.1037234042553	0.207598090097571	6	100	regulation of ligase activity
GO:0048002	7.77374940761755e-08	35.0955882352941	0.224205937305377	6	108	antigen processing and presentation of peptide antigen
GO:0000082	8.28495259004838e-08	24.2152052465387	0.386132447581482	7	186	G1/S transition of mitotic cell cycle
GO:0030330	1.25433059224399e-07	32.2297297297297	0.242889765414158	6	117	DNA damage response, signal transduction by p53 class mediator
GO:0072331	1.68967505186815e-07	30.5641025641026	0.255345650820012	6	123	signal transduction by p53 class mediator
GO:0031398	2.04375567662649e-07	29.5454545454545	0.263649574423915	6	127	positive regulation of protein ubiquitination
GO:0000077	2.34758725350849e-07	28.8245967741935	0.269877517126842	6	130	DNA damage checkpoint
GO:0000084	2.80959714360291e-07	27.916015625	0.278181440730745	6	134	S phase of mitotic cell cycle
GO:0031570	3.06719228726957e-07	27.4826923076923	0.282333402532697			

6	136	DNA integrity checkpoint
GO:0007093	3.48975113536308e-07	26.8571428571429	0.288561345235624
6	139	mitotic cell cycle checkpoint
GO:0042770	3.64079545731552e-07	26.6548507462687	0.2906373261366 6
140	signal transduction in response to DNA damage
GO:0051320	4.12650314043875e-07	26.0656934306569	0.296865268839527
6	143	S phase
GO:0090068	5.68762218632599e-07	24.6137931034483	0.313473116047332
6	151	positive regulation of cell cycle process
GO:0019882	5.91289054146557e-07	24.4434931506849	0.315549096948308
6	152	antigen processing and presentation
GO:0000209	8.28994291915284e-07	23.0096774193548	0.334232925057089
6	161	protein polyubiquitination
GO:0031396	1.06371034376703e-06	22.0046296296296	0.348764791363919
6	168	regulation of protein ubiquitination
GO:0007346	2.23664730534032e-06	14.5233548766157	0.62902221299564
7	303	regulation of mitotic cell cycle
GO:0043161	3.78521980910599e-06	17.5098522167488	0.433880008303924
6	209	proteasomal ubiquitin-dependent protein catabolic process
GO:0010498	4.57725075111673e-06	16.9178571428571	0.448411874610754
6	216	proteasomal protein catabolic process
GO:0006521	5.52175345972073e-06	41.7068214804064	0.118330911355616
4	57	regulation of cellular amino acid metabolic process
GO:0000075	8.78757779016714e-06	15.0264830508475	0.502387378036122
6	242	cell cycle checkpoint
GO:0051329	9.76062238866366e-06	11.4940392706872	0.786796761469794
7	379	interphase of mitotic cell cycle
GO:0051325	1.1183273928385e-05	11.245652173913 0.8034046086776 7	387
interphase
GO:0033238	1.40738090422235e-05	32.472850678733 0.149470624870251	4
72	regulation of cellular amine metabolic process
GO:0071156	1.44265095004602e-05	13.7238372093023	0.548058957857588
6	264	regulation of cell cycle arrest
GO:0010564	1.73202083240063e-05	10.4794359576968	0.859456093003944
7	414	regulation of cell cycle process
Tissue: Heart_Left_Ventricle=>Muscle_Skeletal	Type:	asymmetric
SourceGene:	ITPKA(ENSG00000137825.6)
TargetGeneSet:	TMEM52	UBAP2L	HADH	HADHB	RPLP0P6 UGP2	RUVBL1	DCUN1D1
ZDHHC19 ETFDH	NUDT3	EPHA7	RP11	SND1	ZNF703	TOR1B	FAM213A NXF1
HOXC9	ACADVL	DHX40	ECH1	PIGT	RAB9A
GOBPID	Pvalue	OddsRatio	ExpCount	Count	Size	Term
GO:0006635	1.41289059246051e-08	88.1066176470588	0.0813784513182479
5	56	fatty acid beta-oxidation
GO:0009062	3.81315591051784e-08	71.2648809523809	0.0988166908864438
5	68	fatty acid catabolic process
GO:0072329	8.16074322110899e-08	60.625	0.114801743823957	5	79
monocarboxylic acid catabolic process
GO:0019395	1.32806728447555e-07	54.6798780487805	0.126427236869421
5	87	fatty acid oxidation
GO:0034440	1.57526132238458e-07	52.7389705882353	0.13078679676147
5	90	lipid oxidation
GO:0044242	1.28190144385319e-06	33.8494318181818	0.199086568403571
5	137	cellular lipid catabolic process
GO:0030258	1.81557241724748e-06	31.443661971831 0.213618434710401	5
147	lipid modification
GO:0016054	5.76208919702338e-06	24.6011740331492	0.270292713307038
5	186	organic acid catabolic process
GO:0046395	5.76208919702338e-06	24.6011740331492	0.270292713307038
5	186	carboxylic acid catabolic process
GO:0044282	1.24698933640249e-05	20.8582746478873	0.316794685488894
5	218	small molecule catabolic process
GO:0032787	1.67535472839315e-05	14.2126582278481	0.582727838903882
6	401	monocarboxylic acid metabolic process

Tissue: Muscle_Skeletal=>Heart_Left_Ventricle Type: cluster
SourceGene: MYH13(ENSG00000006788.6)
TargetGeneSet: SSU72 PARK7 DDI2 RPL11 RP4 BTBD8 RPL5 CSDE1 HAX1
NOS1AP SNRPE RP11 UBC AAK1 POLR1B EEF1B2 WDR48 RPL14 UBE2K ANK2
RPS3A SERF1B RAD50 GNB2L1 HIVEP1 PPIAP29 SRSF3 COQ3 PNISR ARID1B PPIA
RPS3AP26 PNPLA8 ZNF467 RPL30 ZNF706 DEPTOR GBA2 RNF20 ZNF483 MRRF
ARPC5L APBB1IP RPS24 RPS3AP5 ZDHHC16 PPIAP19 UROS GLRX3 RPL27A DDX6
COX14 ACVR1B PFDN5 C12orf73 RPL6 GTF3A POMP COX17P1 METTL17
VTI1B TMED8 CDC42BPB GANC RPS3AP6 RNU6 CD276 MRPL28 RPS15A
RPL26 RPS7P1 SYNRG RPL19 RPL27 RPS7P11 RPS2P48 SRSF1 SRSF2 MYL12B
NDUFV2 TXNL4A C19orf35 RIN2 ARFGEF2 PPIAP22 ATP5O HMGB1P10
APOL6 APOL2 APOBEC3C XRCC6 C22orf32 PIGA RPL9P7 EEF1B2P3
RPS2P55 RP6 SPIN4 RPS23P8 MED12 COX7B

GOBPID	Pvalue	OddsRatio	ExpCount	Count	Size	Term
GO:0006414	2.6903825884287e-16	34.8791208791209	0.559476852812954	14	105	translational elongation
GO:0006415	1.3332248432203e-15	37.2291666666667	0.48487993910456	13	91	translational termination
GO:0070972	1.45054291603114e-15	30.491452991453	0.628745415542177	14	118	protein localization to endoplasmic reticulum
GO:0006614	9.16193873515715e-15	31.5329483695652	0.559476852812954	13	105	SRP-dependent cotranslational protein targeting to membrane
GO:0006613	1.03994866934022e-14	31.1917002688172	0.564805203792125	13	106	cotranslational protein targeting to membrane
GO:0045047	1.03994866934022e-14	31.1917002688172	0.564805203792125	13	106	protein targeting to ER
GO:0072599	1.03994866934022e-14	31.1917002688172	0.564805203792125	13	106	establishment of protein localization to endoplasmic reticulum
GO:0000184	4.8362029249584e-14	27.341391509434	0.634073766521348	13	119	nuclear-transcribed mRNA catabolic process, nonsense-mediated decay
GO:0000956	2.30569410904471e-13	20.3856630824373	0.900491315479898	14	169	nuclear-transcribed mRNA catabolic process
GO:0034623	3.45338973426966e-13	19.7416666666667	0.927133070375753	14	174	cellular macromolecular complex disassembly
GO:0006402	4.72796873249243e-13	19.2547425474255	0.948446474292437	14	178	mRNA catabolic process
GO:0032984	5.1080815321368e-13	19.1367003367003	0.953774825271608	14	179	macromolecular complex disassembly
GO:0019058	1.05742489747585e-12	15.8579749103943	1.2308490761885	15	231	viral infectious cycle
GO:0019080	1.08608954059873e-12	20.9542572463768	0.80458099785482	13	151	viral genome expression
GO:0019083	1.08608954059873e-12	20.9542572463768	0.80458099785482	13	151	viral transcription
GO:0006413	1.1827488475147e-12	20.8020458633094	0.809909348833991	13	152	translational initiation
GO:0006612	1.28722352384582e-12	20.6520089285714	0.815237699813162	13	153	protein targeting to membrane
GO:0043624	1.28722352384582e-12	20.6520089285714	0.815237699813162	13	153	cellular protein complex disassembly
GO:0043241	1.9480621205499e-12	19.9328663793103	0.841879454709017	13	158	protein complex disassembly
GO:0006401	3.27946995097057e-12	16.5014543339151	1.09231195073005	14	205	RNA catabolic process
GO:0022415	5.83733746363248e-12	13.9522712310731	1.38537125458446	15	260	viral reproductive process
GO:0072594	5.01185158014528e-11	15.0833606020942	1.08698359975088	13	204	establishment of protein localization to organelle
GO:0071845	2.5819617408704e-10	11.6082304526749	1.51325167808456	14	284	cellular component disassembly at cellular level
GO:0022411	3.10317216976828e-10	11.4355231143552	1.53456508200125	14	288	cellular component disassembly
GO:0016032	1.47811966170085e-09	8.40489918974939	2.40308629160612			

16	451	viral reproduction
GO:0071843	5.14402960702649e-08	10.3406432748538	1.27347588402187
11	239	cellular component biogenesis at cellular level
GO:0033365	1.6986105728012e-07	6.73686758654079	2.52031001314788
14	473	protein localization to organelle
GO:0022613	3.02358418324044e-07	9.82922596320722	1.19887897031347
10	225	ribonucleoprotein complex biogenesis
GO:0042273	3.57045597231164e-07	98.3972602739726	0.0639402117500519
4	12	ribosomal large subunit biogenesis
GO:0006605	1.16729083679998e-06	6.13032131236443	2.52563836412705
13	474	protein targeting
GO:0071826	1.50487845944361e-05	12.8378783338328	0.532835097917099
6	100	ribonucleoprotein complex subunit organization
Tissue: Heart_Left_Ventricle=>Muscle_Skeletal	Type:	asymmetric
SourceGene:	NELF(ENSG00000165802.14)
TargetGeneSet:	UBR4	FABP3	MACF1	HECTD3	C1orf52	CCDC18	RPS27	MTX1
HAPLN2	RP11	DUSP12	SLC19A2	LRRN2	PARP1	C2orf28	ETAA1	TPRKB	RETSAT
COX5B	COA5	MAP4K4	NDUFB3	EFHD1	MYEOV2	HDLBP	FAM208A	GXYLT2	GAP43
NDUFB4	C3orf37	HAUS3	DOK7	WDFY3	MRPS36	COX7C	ID4	VARS	TAF11	CUL7
DST	BACH2	COQ3	EIF3B	NDUFA4	PHF14	GGCT	TRIM56	NDUFA5	KIAA1549
DEFB1	UBE2V2	ARMC1	UQCRB	COX6C	MRPL13	TLN1	CLTA	FYTTD1P1
MRPL41	CWF19L1	PCGF6	C10orf88		TRIM44	NDUFS8	C11orf73		FZD4
TMEM135	ATP5L	ERC1	USP5	SLC25A3	GPN3	EXOSC8	FREM2	SAMD4A	SYNE2
SMEK1	NDUFB1	DYNC1H1	SIVA1	C15orf29		SLC12A6	CCPG1	VPS13C	COX5A	ETFA
PEX11A	MPG	TRAF7	PAQR4	NOMO2	ARL6IP1	UQCRC2	MLYCD	TRIM16	SUPT6H
TMEM106A		MRPL27	ACSF2	TACO1	ATP5H	C18orf21		ACAA2	CNDP2
PQLC1	UQCR11	COX6B1	ECH1	CYP2G1P	GEMIN7	RP1	MAP1LC3A		PI4KA	MPST
C22orf46		NDUFB11	UBE2A
GOBPID	Pvalue	OddsRatio	ExpCount	Count	Size	Term
GO:0022904	7.13933868438904e-22	43.3572874493927	0.624455055013494
18	96	respiratory electron transport chain
GO:0022900	3.95593074504116e-19	29.0764519056261	0.871635180956335
18	134	electron transport chain
GO:0045333	1.15776440546362e-18	27.1852716468591	0.92367310220746
18	142	cellular respiration
GO:0015980	9.91986481979411e-13	11.5290475323256	1.9969552280119	18
307	energy derivation by oxidation of organic compounds
GO:0006091	2.86043603514395e-11	8.63934800325998	2.78402878693516
19	428	generation of precursor metabolites and energy
GO:0006119	3.47605471064466e-09	26.6176744186047	0.377274929070653
8	58	oxidative phosphorylation
GO:0042773	1.99865711717415e-08	28.7985632183908	0.305722787350356
7	47	ATP synthesis coupled electron transport
GO:0042775	1.99865711717415e-08	28.7985632183908	0.305722787350356
7	47	mitochondrial ATP synthesis coupled electron transport
GO:0006120	1.07279395697902e-07	32.5613636363636	0.23417064563006
6	36	mitochondrial electron transport, NADH to ubiquinone
Tissue: Heart_Left_Ventricle=>Muscle_Skeletal	Type:	asymmetric
SourceGene:	OR7A5(ENSG00000188269.3)
TargetGeneSet:	C1orf52	RP11	PCNXL2	UBC	RPL24	RPL35A	RPL34	LSM6
COX7C	CTD	MRPS12	NAA38	RPL27A	USP5	SIPA1L1	SEC11A	RPS7P1	RPL27	CHAD
RPL17	WDR7	SNORA68
GOBPID	Pvalue	OddsRatio	ExpCount	Count	Size	Term
GO:0006614	7.26569440883648e-14	147.814432989691	0.11625493045464
8	105	SRP-dependent cotranslational protein targeting to membrane
GO:0006613	7.85492180676036e-14	146.295918367347	0.117362120268494
8	106	cotranslational protein targeting to membrane
GO:0045047	7.85492180676036e-14	146.295918367347	0.117362120268494
8	106	protein targeting to ER
GO:0072599	7.85492180676036e-14	146.295918367347	0.117362120268494
8	106	establishment of protein localization to endoplasmic reticulum
GO:0070972	1.89402554759958e-13	130.227272727273	0.130648398034738
8	118	protein localization to endoplasmic reticulum

GO:0006612 1.5733295426454e-12 98.551724137931 0.169400041519618 8
153 protein targeting to membrane
GO:0006415 3.39176939742113e-12 132.87962962963 0.100754273060688 7
91 translational termination
GO:0000956 3.52125634699605e-12 88.6583850931677 0.187115078541277
8 169 nuclear-transcribed mRNA catabolic process
GO:0006402 5.355101126179e-12 83.9117647058823 0.197079786865961
8 178 mRNA catabolic process
GO:0006414 9.46494166699606e-12 113.785714285714 0.11625493045464
7 105 translational elongation
GO:0072594 1.60610344859003e-11 72.6479591836735 0.225866722026157
8 204 establishment of protein localization to organelle
GO:0006401 1.67049389414366e-11 72.2741116751269 0.226973911840011
8 205 RNA catabolic process
GO:0000184 2.31164452652353e-11 99.4652777777778 0.131755587848592
7 119 nuclear-transcribed mRNA catabolic process, nonsense-mediated decay
GO:0019080 1.2505211822669e-10 77.1890432098765 0.167185661891911
7 151 viral genome expression
GO:0019083 1.2505211822669e-10 77.1890432098765 0.167185661891911
7 151 viral transcription
GO:0006413 1.31017098892955e-10 76.6513409961686 0.168292851705764
7 152 translational initiation
GO:0043624 1.3722330701249e-10 76.12100456621 0.169400041519618 7
153 cellular protein complex disassembly
GO:0043241 1.7216091487359e-10 73.5746872700515 0.174935990588887
7 158 protein complex disassembly
GO:0034623 3.3947602590686e-10 66.4510978043912 0.192651027610546
7 174 cellular macromolecular complex disassembly
GO:0032984 4.14230218725797e-10 64.4967700258398 0.198186976679815
7 179 macromolecular complex disassembly
GO:0019058 2.46507857012528e-09 49.34375 0.255760847000208 7
231 viral infectious cycle
GO:0022415 5.60903100281073e-09 43.5985946420729 0.287869351601965
7 260 viral reproductive process
GO:0071845 1.03407170427426e-08 39.7537103890894 0.314441907134454
7 284 cellular component disassembly at cellular level
GO:0022411 1.1390980188622e-08 39.1767497034401 0.318870666389869
7 288 cellular component disassembly
GO:0033365 1.26914402183488e-08 30.0430107526882 0.523700781952806
8 473 protein localization to organelle
GO:0006605 1.29027933970099e-08 29.9763948497854 0.52480797176666
8 474 protein targeting
GO:0016032 2.46764369267241e-07 24.5087587587588 0.499342606048024
7 451 viral reproduction
Tissue: Muscle_Skeletal=>Heart_Left_Ventricle Type: asymmetric
SourceGene: PTBP2(ENSG00000117569.12)
TargetGeneSet: AURKAIP1 BTBD8 KIAA1107 GNAI3 OTUD7B ZBTB37 SWT1
SUSD4 UBC NFU1 COX5B TSPO NBEAL1 ASB1 WDR48 ZNF501 BCL6
NAP1L5 RP11 NDUFS6 TRIM23 COX7C LARP1 CREBRF HIGD2A RNF44 COX7A2
SLC18B1 MRPS24 PSPH GATSL2 SRI ATP5J2 GATS RPS26P31 GSTK1 CTD
RPL30 COX6C FBXO32 FAM49B ZFP41 CYC1 NDUFB6 C9orf131 NDUFA8
OLFML2A PRRC2B CAMSAP1 ENTPD2 CREM NDUFB8 NDUFS3 COX8A EFEMP2 NDUFS8
PFDN5 ITGA5 WIBG FITM1 PROX2 LINC00521 COX5A WDR24 UNKL
RPL3L NDUFB10 TCEB2 YPEL3 RBL2 PSMB6 NT5M HELZ RECQL5 ALYREF
SNRPD1 ATP5D C19orf70 NDUFB7 COX6B1 POLR2I NDUFA3 MAPRE1 CEP250
ATP9A PPIAP22 ATP5O MRPL40 RP4 HUWE1 COX7B
GOBPID Pvalue OddsRatio ExpCount Count Size Term
GO:0022904 5.88938071504012e-28 73.8080495356037 0.471662860701682
20 96 respiratory electron transport chain
GO:0022900 7.71309560013103e-25 49.0746474028208 0.658362743062764
20 134 electron transport chain
GO:0045333 2.61492795940944e-24 45.8309225329476 0.697667981454571
20 142 cellular respiration

GO:0015980	1.60320971903948e-17	19.2566782810685	1.50833852328559
20	307	energy derivation by oxidation of organic compounds
GO:0006091	9.75352417986163e-15	13.4294502114571	2.10283025396166
20	428	generation of precursor metabolites and energy
GO:0006120	8.3589123933997e-14	77.1666666666667	0.176873572763131
9	36	mitochondrial electron transport, NADH to ubiquinone
GO:0006119	1.83556053078907e-13	48.948087431694 0.284962978340599	10
58	oxidative phosphorylation
GO:0042773	1.15940979052487e-12	54.7869269949066	0.230918275551865
9	47	ATP synthesis coupled electron transport
GO:0042775	1.15940979052487e-12	54.7869269949066	0.230918275551865
9	47	mitochondrial ATP synthesis coupled electron transport
Tissue: Muscle_Skeletal=>Heart_Left_Ventricle	Type:	asymmetric
SourceGene:	QDPR(ENSG00000151552.7)
TargetGeneSet:	THRAP3	GNG5	ADCK3	FH	NFU1	TSPO	UGGT1	TNS1
SLC9C1	ARHGAP31	POU5F1P6	RP11	ANTXR2	BNIP1	PPP2R5D	MDN1
FBXL18	C7orf41	IGFBP3	DPY19L2P4	GSTK1	TCEB1	COX6C	NDUFB9	FAM49B	CYC1
TSSK5P1	SMARCA2	NDUFB6	FAM122A	C9orf174	RNF20	MUSK	NDUFA8	ARPC5L
PCGF5	SUFU	PDCD4	CSRP3	PHF21A	NDUFS3	PDE2A	TMTC1	TMED8	CDC42BPB
HERC2	LRRC49	NDUFB10	ECI1	SNX29	CSDAP1	RBL2	MT1X	KLHL36	KIAA0513
ZNF624	MLX	NFE2L1	ERN1	NDUFV2	FAM210A	C19orf70	DDX39A	ZNF570	RP1
KCNB1	GGA1	SLC16A8	UQCRFS1P1	C22orf46	RP4	PACSIN2	ACOT9
RPL9P7	GAPDHP1	TIMM17B	COX7B	MBNL3
GOBPID	Pvalue	OddsRatio	ExpCount	Count	Size	Term
GO:0022904	1.92258326527859e-10	29.0141987829615	0.398588332987337
9	96	respiratory electron transport chain
GO:0015980	2.94117361601686e-10	13.262483716891 1.27465227319909	13
307	energy derivation by oxidation of organic compounds
GO:0045333	3.02432975593257e-10	21.6045454545455	0.589578575877102
10	142	cellular respiration
GO:0022900	3.83137500848125e-09	20.1402352941176	0.556362881461491
9	134	electron transport chain
GO:0006120	7.00893603321913e-09	53.1888888888889	0.149470624870251
6	36	mitochondrial electron transport, NADH to ubiquinone
GO:0006091	1.6391297759747e-08	9.31494488592669	1.77703965123521
13	428	generation of precursor metabolites and energy
GO:0042773	3.72981637460749e-08	38.8888888888889	0.195142204691717
6	47	ATP synthesis coupled electron transport
GO:0042775	3.72981637460749e-08	38.8888888888889	0.195142204691717
6	47	mitochondrial ATP synthesis coupled electron transport
GO:0006119	1.35720446957882e-07	30.6388888888889	0.240813784513182
6	58	oxidative phosphorylation
Tissue: Muscle_Skeletal=>Heart_Left_Ventricle	Type:	cluster
SourceGene:	RNY4P14(ENSG00000200526.1)
TargetGeneSet:	DFFB	ZBTB48	LRRC38	COL16A1	CCDC28B	COL24A1	GSTM5	BNIPL	RGS5
RP11	PLA2G4A	ANTXR1	LINC00152	XIRP2	FRZB	COL3A1	COL5A2	FN1
DOCK10	FBLN2	ROBO1	FILIP1L	CCDC80	WDR52	TNIK	DCUN1D1	FAM184B	KIT
SEPT11	HPGDS	CXXC4	PDE5A	FAM198B	CPE	LPCAT1	PTGER4	PHF15	TGFBI
PCDHGC5	ARSI	TRIM41	MAPK14	RP1	PM20D2	MARCKS	SLC35F1	EPB41L2	FNDC1	LFNG
NFE2L3	VWC2	COL1A2	SLC12A9	EBF2	PLAT	MSC	CTHRC1	COL14A1	FREM1
GLIPR2	RG9MTD3	OGN	ASPN	ECM2	ANKS6	COL15A1	NCS1	COL5A1	CCDC3
ARHGAP22	CDH23	POLL	OLFML1	LRRC55	NRXN2	ZC3H12C	SCN4B	KCNA5
LRRC10	LUM	ANO4	GPR133	LPAR6	DACT1	SERPINA3	ALDH1A2	FTSJD1
CENPN	CAMTA2	MFAP4	STAT3	EZH1	MRC2	CDC42EP4	TIMP2	C17orf62
ARHGAP28	MOB3A	ZNF558	ANKRD27	WDR62	PSMC4	ZNF600	ZNF525	ZNF671
PTPRT	PARD6B	CDH26	COL6A1	COL6A2	TOB2	CAPN6	LHFPL1	PLS3
GOBPID	Pvalue	OddsRatio	ExpCount	Count	Size	Term
GO:0030198	3.56054065301709e-09	13.5361904761905	0.980416580167462
11	161	extracellular matrix organization
GO:0043062	3.80116888912781e-09	13.445600756859 0.986506124143658	11
162	extracellular structure organization
GO:0030199	4.16435072997243e-08	38.8509485094851	0.200954951214449
6	33	collagen fibril organization

GO:0071230	2.09866096359796e-06	28.781124497992	0.21313403916684	5
35	cellular response to amino acid stimulus
GO:0071229	4.70749855958424e-06	23.9742302543507	0.249671303024012
5	41	cellular response to acid
GO:0071418	1.27485675500892e-05	19.1673360107095	0.304477198809771
5	50	cellular response to amine stimulus
GO:0071417	1.54890956021022e-05	18.3491412458344	0.316656286762162
5	52	cellular response to organic nitrogen
Tissue: Heart_Left_Ventricle=>Muscle_Skeletal	Type:	asymmetric
SourceGene:	RP1-163G9.1(ENSG00000177133.5)
TargetGeneSet:	GDI2P2	RP11	TMEM183A	KRT18P12	FAM179A	MDH1	UGP2
VAMP8	COX5B	SPPL2B	GIGYF2	PLCL2	PLCL2-AS1	ZNF621	ZDHHC3	TEX264	PDHB
ZBTB20	NDUFB4	C3orf55	LIMCH1	EGF	MRPS36	UQCRQ	GUSBP2	XPO5	WDR11
IFNGR1	PHACTR2	NDUFA4	POR	TMEM120A	CTB	ERVW-1	NDUFA5	GSTK1
HMBOX1	CTD	VDAC3	IMPA1	C8orf38	TAF1L	MRPL41	TIMM23	MYPN	MMS19
FADS3	TMEM135	LDHB	DDX23	ASIC1	HOXC9	SLC25A3	CHPT1	FREM2	EDNRB
CMTM5	SAMD4A	LGALS3	DYNC1H1	CHKB	GANC	COX5A	RPUSD1	GRIN2A	UQCRC2
TMCO7	ADAT1	VPS53	RPS4XP17	NF1	KRT32	ATP5H	METRNL	NDUFV2
ACAA2	FBN3	RYR1	LIPE	SBK2	URB1	CECR2	ASB11	TCEAL2	UBE2A
GOBPID	Pvalue	OddsRatio	ExpCount	Count	Size	Term
GO:0045333	6.25226838176364e-11	20.6318768096868	0.678015362258667
11	142	cellular respiration
GO:0022904	7.01828460502807e-10	24.6465517241379	0.458376582935437
9	96	respiratory electron transport chain
GO:0022900	7.1571976333513e-10	19.4887916894478	0.639817313680714
10	134	electron transport chain
GO:0006091	1.21707375563813e-09	9.39534570890503	2.04359559892049
15	428	generation of precursor metabolites and energy
GO:0015980	2.03692193812284e-08	10.0531668153434	1.46585011417895
12	307	energy derivation by oxidation of organic compounds
GO:0006119	8.0674877637956e-06	21.1217570754717	0.27693585219016
5	58	oxidative phosphorylation
Tissue: Heart_Left_Ventricle=>Muscle_Skeletal	Type:	asymmetric
SourceGene:	RP1-228H13.1(ENSG00000227311.1)
TargetGeneSet:	RP11	NUDT4P1	IL6R	TMEM17	HDAC4	ATP13A4-AS1	RPS10
RPL10A	VPS26A	VDAC2	EIF3F	NUDT4	TIMM9	RP3	IGHD	TGM7	MRPS34	CTD
RPL13	EIF3K	RPL18	FUNDC2
GOBPID	Pvalue	OddsRatio	ExpCount	Count	Size	Term
GO:0006413	1.37968195674388e-08	53.3798256537983	0.178811154937375
6	152	translational initiation
GO:0006612	6.95845230174473e-07	40.2195945945946	0.179987544114594
5	153	protein targeting to membrane
GO:0072594	2.87729689222389e-06	29.8052763819095	0.239983392152792
5	204	establishment of protein localization to organelle
GO:0006415	3.28912916443026e-06	50.7409372236958	0.107051415126981
4	91	translational termination
GO:0006414	5.82364233891752e-06	43.6648895658797	0.123520863608055
4	105	translational elongation
GO:0006614	5.82364233891752e-06	43.6648895658797	0.123520863608055
4	105	SRP-dependent cotranslational protein targeting to membrane
GO:0006613	6.04765176555914e-06	43.2337858220211	0.124697252785274
4	106	cotranslational protein targeting to membrane
GO:0045047	6.04765176555914e-06	43.2337858220211	0.124697252785274
4	106	protein targeting to ER
GO:0072599	6.04765176555914e-06	43.2337858220211	0.124697252785274
4	106	establishment of protein localization to endoplasmic reticulum
GO:0070972	9.26148736805911e-06	38.6504723346829	0.138813922911909
4	118	protein localization to endoplasmic reticulum
GO:0000184	9.57670561999659e-06	38.3117056856187	0.139990312089129
4	119	nuclear-transcribed mRNA catabolic process, nonsense-mediated decay
Tissue: Heart_Left_Ventricle=>Muscle_Skeletal	Type:	asymmetric
SourceGene:	SRSF1(ENSG00000136450.6)
TargetGeneSet:	NKAIN1	USP33	KCNC4	BRP44	FAM129A	MDH1	TIA1	SUCLG1

CNNM3	COX5B	FASTKD1	NDUFB3	ICA1L	CRTAP	NDUFB5	MB21D2	RAB28	FAT4
PHF17	NDUFC1	UFSP2	MRPS36	COX7C	CTXN3	CTB	GNL1	UHRF1BP1
MTRNR2L9		ELFN1	GATS	NDUFA5	SP6	SDCBP	HEY1	UQCRB	MTERFD1 AUH
C9orf106		C9orf142		MRPL41	MLLT10	VDAC2	RP11	NDUFS8	CHKA
ATP5L	POU6F1	SLC25A3 BRI3BP	ZNF839	GOLGA8IP		COX5A	UBE2Q2	UQCRC2
MYH13	MYH1	MTRNR2L1		SRSF1	DHX40	ATP5H	ECSIT	EIF3K	MYBPC2
PSMD4P1 MYO18B	TTC28	UQCR10	C22orf46		CPT1B	APOO	SLC9A7	NDUFB11
GAGE2D	AR
GOBPID	Pvalue	OddsRatio	ExpCount	Count	Size	Term
GO:0045333	9.61868886345563e-22	52.4592432432432	0.530620718289392
17	142	cellular respiration
GO:0022904	3.17953538779795e-19	61.1006097560976	0.358729499688603
14	96	respiratory electron transport chain
GO:0022900	4.11300559421583e-17	41.64125	0.500726593315342	14
134	electron transport chain
GO:0015980	5.4562090248746e-16	22.3503261882572	1.14718704587918
17	307	energy derivation by oxidation of organic compounds
GO:0006091	7.85300294206473e-15	17.0573170731707	1.59933568611169
18	428	generation of precursor metabolites and energy
GO:0006119	3.67604008661309e-11	49.9026086956522	0.216732406061864
8	58	oxidative phosphorylation
GO:0042773	3.81102226867521e-10	53.456914893617 0.175627984222545	7
47	ATP synthesis coupled electron transport
GO:0042775	3.81102226867521e-10	53.456914893617 0.175627984222545	7
47	mitochondrial ATP synthesis coupled electron transport
GO:0006120	2.08094257316656e-07	47.2876892692561	0.134523562383226
5	36	mitochondrial electron transport, NADH to ubiquinone
GO:0009060	1.75417182913406e-05	30.2294736842105	0.156944156113764
4	42	aerobic respiration
Tissue: Heart_Left_Ventricle=>Muscle_Skeletal	Type:	asymmetric
SourceGene:	SUSD2(ENSG00000099994.10)
TargetGeneSet:	NDUFS2	FAM129A TRMT61B SUCLG1	ANKRD36 NIF3L1	NDUFB3	LRRC3B	PDHB
MLF1	COX18	RAB33B	RICTOR	MRPS30	IL6ST	COQ3	RTN4IP1 C6orf203		SGK1
STAG3	RP11	MRPL15	PGM5P1	PDSS1	RHOBTB1 PPIF	GHITM	FZD4	CD9
AP5M1	DIO2	SLC25A29		ETFA	UQCRC2	CIAPIN1 GLG1	TEX2	METRNL
ATP5A1	PSMD4P1 C21orf33		ARVCF	HPS4	NDUFA6	APOO	TIMM8A	SLC25A5
GOBPID	Pvalue	OddsRatio	ExpCount	Count	Size	Term
GO:0045333	2.16383598522514e-09	29.3978383942357	0.363573455124213
8	142	cellular respiration
GO:0015980	3.39155268413446e-09	17.6044394562913	0.78603556847277
10	307	energy derivation by oxidation of organic compounds
GO:0006091	7.95810263568277e-08	12.401205032784 1.09584111826171	10
428	generation of precursor metabolites and energy
GO:0022904	1.44492783454821e-07	30.8043010752688	0.245796138675524
6	96	respiratory electron transport chain
GO:0022900	1.04301560891696e-06	21.601814516129 0.343090443567919	6
134	electron transport chain
GO:0006119	1.39768675208686e-05	32.2334455667789	0.148501833783129
4	58	oxidative phosphorylation
Tissue: Muscle_Skeletal=>Heart_Left_Ventricle	Type:	asymmetric
SourceGene:	TRDN(ENSG00000186439.7)
TargetGeneSet:	MTND2P28		AURKAIP1		MRPL20	RPL5	HBXIP	ZNF687
EEF1B2	UBE2F	MAP4	RPL29	THOC7	HTT-AS1 MRAP	RP11	RPS3A	MTND5P11
CTB	NQO2	SRSF3	HDDC2	FBXL18	CYCS	MRPS24	TCEB1	AK1	METTL11A
GLRX3	RPL27A	NDUFS3	PFDN5	RAP1B	RPL6	HERC2P10		PHGR1	ZFAND6
STUB1	ECI1	RPS15A	METTL16 TMEM93	TXNDC17 STX8	RPS7P1	RPL27	ICT1
NDUFV2	RPL17	C19orf70		RPL36	ZNF121	MRPL4	ECSIT	MRPS12	ERCC1
AP2S1	RP13	COX7B	GLA
GOBPID	Pvalue	OddsRatio	ExpCount	Count	Size	Term
GO:0006415	1.57116185129279e-13	50.5291005291005	0.283371392983185
10	91	translational termination
GO:0006414	6.83719788344114e-13	43.0406015037594	0.326966991903675
10	105	translational elongation

GO:0022415	1.51654404867934e-11	20.7595307917889	0.809632551380527
12	260	viral reproductive process
GO:0019080	2.68062413950479e-11	28.9057750759878	0.470209674070999
10	151	viral genome expression
GO:0019083	2.68062413950479e-11	28.9057750759878	0.470209674070999
10	151	viral transcription
GO:0006614	2.839953166158e-11	37.265625	0.326966991903675	9
105	SRP-dependent cotranslational protein targeting to membrane
GO:0043624	3.05645861104189e-11	28.4975024975025	0.476437616773926
10	153	cellular protein complex disassembly
GO:0006613	3.09646487107332e-11	36.8788659793814	0.330080963255138
9	106	cotranslational protein targeting to membrane
GO:0045047	3.09646487107332e-11	36.8788659793814	0.330080963255138
9	106	protein targeting to ER
GO:0072599	3.09646487107332e-11	36.8788659793814	0.330080963255138
9	106	establishment of protein localization to endoplasmic reticulum
GO:0043241	4.2098732973947e-11	27.5250965250965	0.492007473531244
10	158	protein complex disassembly
GO:0070972	8.20550790764881e-11	32.7912844036697	0.367448619472701
9	118	protein localization to endoplasmic reticulum
GO:0019058	8.63871384160125e-11	20.8617647058824	0.719327382188084
11	231	viral infectious cycle
GO:0000184	8.85686637461013e-11	32.4909090909091	0.370562590824164
9	119	nuclear-transcribed mRNA catabolic process, nonsense-mediated decay
GO:0034623	1.09543149198663e-10	24.8118466898955	0.541831015154661
10	174	cellular macromolecular complex disassembly
GO:0032984	1.44894676833757e-10	24.0693153000845	0.557400871911978
10	179	macromolecular complex disassembly
GO:0072594	5.23025518826207e-10	20.9307805596465	0.635250155698568
10	204	establishment of protein localization to organelle
GO:0016032	6.5912322409242e-10	12.9554794520548	1.40440107951007
13	451	viral reproduction
GO:0071845	7.81150313155869e-10	16.7488687782805	0.884367863815653
11	284	cellular component disassembly at cellular level
GO:0006413	7.96884843940991e-10	24.9353146853147	0.473323645422462
9	152	translational initiation
GO:0006612	8.44779860055398e-10	24.7604166666667	0.476437616773926
9	153	protein targeting to membrane
GO:0022411	9.0561627267405e-10	16.5023359524315	0.896823749221507
11	288	cellular component disassembly
GO:0000956	2.04094139108864e-09	22.259375	0.526261158397343	9
169	nuclear-transcribed mRNA catabolic process
GO:0006402	3.22575021615126e-09	21.060650887574	0.554286900560515	9
178	mRNA catabolic process
GO:0006401	1.11181915330089e-08	18.125	0.638364127050031	9	205
RNA catabolic process
GO:0033365	1.46260855257869e-06	8.60413452638075	1.47290844924227
10	473	protein localization to organelle
GO:0006605	1.49077133793387e-06	8.58497536945813	1.47602242059373
10	474	protein targeting
Tissue: Nerve_Tibial=>Muscle_Skeletal	Type:	asymmetric

SourceGene:	AAK1(ENSG00000115977.13)	AP2 associated kinase 1

TargetGeneSet:	MTND2P28	PLEKHM2	RPL11	RPS27	KLHL12	MRPL55	EIF5B
EEF1B2	ENTPD3-AS1	RPL29	RP11	RPL34	MTND5P11	SYNPO	MTRNR2L9

RPS3AP26 MTUS1 PCM1 RPS20 RPL30 7SK RPS24 RPS3AP5 MTRNR2L8 RBMS1P1 COX17P1 DNAJC3 GOLGA8IP ADCY7 MTRNR2L1 RPL23A RPS7P11 PRR15L RPS2P48 LYPD3 CTA ITIH6 RPL39 MT

GOBPID	Pvalue	OddsRatio	ExpCount	Count	Size	Term
GO:0006414	1.59E-16	125.7368421	0.159850529	10	105	translational elongation
GO:0006415	4.83E-15	121.1285178	0.138537125	9	91	translational termination
GO:0006413	7.13E-15	83.84389671	0.231402671	10	152	translational initiation
GO:0006614	1.83E-14	103.3629808	0.159850529	9	105	SRP-dependent cotranslational protein targeting to membrane
GO:0006613	2.00E-14	102.2902458	0.161372915	9	106	cotranslational protein targeting to membrane
GO:0045047	2.00E-14	102.2902458	0.161372915	9	106	protein targeting to ER
GO:0072599	2.00E-14	102.2902458	0.161372915	9	106	establishment of protein localization to endoplasmic reticulum
GO:0070972	5.39E-14	90.95271701	0.179641547	9	118	protein localization to endoplasmic reticulum
GO:0000184	5.82E-14	90.11958042	0.181163933	9	119	"nuclear-transcribed mRNA catabolic process, nonsense-mediated decay"

GO:0019080	5.17E-13	69.65492958	0.229880285	9	151	
viral genome expression						
GO:0019083	5.17E-13	69.65492958	0.229880285	9	151	
viral transcription						
GO:0006612	5.83E-13	68.67788462	0.232925057	9	153	
protein targeting to membrane						
GO:0043624	5.83E-13	68.67788462	0.232925057	9	153	
cellular protein complex disassembly						
GO:0043241	7.82E-13	66.35002581	0.240536987	9	158	
protein complex disassembly						
GO:0000956	1.44E-12	61.74086538	0.257283233	9	169	
nuclear-transcribed mRNA catabolic process						
GO:0034623	1.88E-12	59.84895105	0.264895163	9	174	
cellular macromolecular complex disassembly						
GO:0006402	2.31E-12	58.41602185	0.270984707	9	178	mRNA
catabolic process						
GO:0032984	2.43E-12	58.06832579	0.272507093	9	179	
macromolecular complex disassembly						
GO:0072594	7.92E-12	50.53491124	0.310566743	9	204	
establishment of protein localization to organelle						
GO:0006401	8.28E-12	50.27354788	0.312089129	9	205	RNA
catabolic process						

GO:0019058 2.42E-11 44.3045738 0.351671165 9 231
viral infectious cycle

GO:0022415 6.98E-11 39.10573092 0.395820358 9 260
viral reproductive process

GO:0071845 1.53E-10 35.63244755 0.432357622 9 284
cellular component disassembly at cellular level

GO:0022411 1.74E-10 35.11166253 0.438447166 9 288
cellular component disassembly

GO:0016032 8.99E-09 21.90793596 0.686596083 9 451
viral reproduction

GO:0033365 1.36E-08 20.83637268 0.720088575 9 473
protein localization to organelle

GO:0006605 1.39E-08 20.79007444 0.721610961 9 474
protein targeting

Tissue: Muscle_Skeletal=>Nerve_Tibial Type: asymmetric

SourceGene: C10orf107(ENSG00000183346.6)

TargetGeneSet: CELA3A ZBTB40 EFNA1 CACNA1S ACTA1 TEX261 REG1A HTR5BP NEB
EXOSC7 RP11 PGM2 TPH2 RPL37 SPINK1 DDX39B RP3 C6orf203
PRSS1 PRSS3P2 LOXL2 RPL10AP2 ST13P6 C9orf72 IL15RA IPMK MYPN
CYP2C9 NRAP PNLIPRP1 TSPAN9 TMTC3 MYBPC1 MYL2 MEG8 IQGAP1

RPL3L CTD GP2 ATP2A1 CTRB2 RPS4XP17 MYH2 MYH1 UNC45B KRT10 TMEM99 CCDC40 ZNF254 CKM MYBPC2 TNNC2 PVALB C22orf32 MTHFD1P1

GOBPID	Pvalue	OddsRatio	ExpCount	Count	Size	Term
GO:0030049	1.03E-11	95.31534091	0.107951007	7	39	muscle filament sliding
GO:0033275	1.03E-11	95.31534091	0.107951007	7	39	actin-myosin filament sliding
GO:0070252	1.80E-11	87.12727273	0.11625493	7	42	actin-mediated cell contraction
GO:0030048	2.20E-10	58.57400932	0.163310498	7	59	actin filament-based movement
GO:0006936	6.96E-10	20.643377	0.661545914	10	239	muscle contraction
GO:0003012	1.97E-09	18.43098958	0.736281226	10	266	muscle system process
GO:0006941	1.88E-06	29.26734694	0.20759809	5	75	striated muscle contraction
GO:0003009	1.85E-05	72.94763514	0.052591516	3	19	skeletal muscle contraction

GO:0030029	1.98E-05	8.247051887	1.195764999	8	432
actin filament-based process

Tissue: Nerve_Tibial=>Muscle_Skeletal	Type:	asymmetric

SourceGene:	CD93(ENSG00000125810.8)

TargetGeneSet:	RPL22	RPS27	PEX19	C1orf192	RHOQP2	TFPI	PHACTR2
EEF1A1P6	RP11	HMCN2	RPL7A	RPS3AP5	ATP5G2	NCKAP1L	UPF3A	RPL13
TLCD2	CLDN7	TIMM13	ATP5SL	ZNF630	VDAC1P1	APOOL

GOBPID	Pvalue	OddsRatio	ExpCount	Count	Size	Term
GO:0006612	3.57E-09	72.90816327	0.148225036	6	153	protein targeting to membrane
GO:0072594	2.01E-08	53.93560606	0.197633382	6	204	establishment of protein localization to organelle
GO:0000184	6.57E-08	69.80019493	0.115286139	5	119	"nuclear-transcribed mRNA catabolic process, nonsense-mediated decay"
GO:0000956	3.79E-07	48.350271	0.163725694	5	169	nuclear-transcribed mRNA catabolic process
GO:0006402	4.90E-07	45.80603725	0.172444814	5	178	mRNA catabolic process
GO:0006401	9.87E-07	39.54722222	0.198602173	5	205	RNA catabolic process
GO:0006415	1.40E-06	65.97701149	0.088159989	4	91	translational termination
GO:0006414	2.49E-06	56.77623762	0.101723064	4	105	translational elongation
GO:0006614	2.49E-06	56.77623762	0.101723064	4	105	

SRP-dependent cotranslational protein targeting to membrane

GO:0006613 2.59E-06 56.21568627 0.102691855 4 106
cotranslational protein targeting to membrane

GO:0045047 2.59E-06 56.21568627 0.102691855 4 106
protein targeting to ER

GO:0072599 2.59E-06 56.21568627 0.102691855 4 106
establishment of protein localization to endoplasmic reticulum

GO:0033365 2.86E-06 22.43576017 0.458238184 6 473
protein localization to organelle

GO:0006605 2.90E-06 22.38621795 0.459206975 6 474
protein targeting

GO:0070972 3.97E-06 50.25614035 0.114317348 4 118
protein localization to endoplasmic reticulum

GO:0019080 1.06E-05 38.88435374 0.146287454 4 151
viral genome expression

GO:0019083 1.06E-05 38.88435374 0.146287454 4 151
viral transcription

GO:0006413 1.08E-05 38.61891892 0.147256245 4 152
translational initiation

GO:0043624 1.11E-05 38.35704698 0.148225036 4 153
cellular protein complex disassembly

GO:0043241 1.26E-05 37.0987013 0.153068992 4 158
protein complex disassembly

GO:0072321 1.83E-05 481.0666667 0.006781538 2 7
chaperone-mediated protein transport

GO:0034623 1.85E-05 33.56941176 0.168569649 4 174
cellular macromolecular complex disassembly

Tissue: Nerve_Tibial=>Muscle_Skeletal Type: asymmetric
SourceGene: ENSG00000239291.1
TargetGeneSet: RPL22 CAMTA1-IT1 CLSTN1 RPS27 PPP1R12B CICP5

SH3YL1 RPS7 BIRC6 SMYD5 RANBP2 NEB TTC21B EEF1B2 UBE2F ATG4B
DTYMK RP11 AP2M1 ZAR1 HNRPDL SUB1 C5orf28 CENPH FAM174A TCERG1 TUBB
PPIL1 PPP2R5D RP1 MTRNR2L9 PHF3 ARID1B MALSU1 DDX56 PPIA
BAIAP2L1 RPS3AP26 CUX1 SLC13A4 MLL3 TNKS PCMTD1 TCEB1
OTUD6B ANXA2P2 SPTLC1 DENND1A RPL35 RPL7A SVIL EIF6 BCCIP TSG101
RPL18AP3 MED4-AS1 RNF31 C14orf166 DYNC1H1 HERC2 HERC1
SNRPA1 SOX8 CTD GLOD4 RPL17P43 SUPT6H TNRC6C WDR45L IER3IP1
C18orf32 TXNL4A GADD45GIP1 ZNF486 RPSAP58 TSHZ3 RYR1 RPS19 RPS5
EIF2S2 PPIAP22 C21orf119 ERVH48-1 SF3A1 RBX1 DMD-AS2 ITIH6
CXorf26 RPL39 RPL10

GOBPID	Pvalue	OddsRatio	ExpCount	Count	Size	Term
GO:0006414	7.31754245214919e-11	25.0631578947368	0.508615320739049	10	105	translational elongation
GO:0006415	4.93542301124678e-10	25.7279088364654	0.440799944640509	9	91	translational termination
GO:0019058	9.82518356581008e-10	13.3793103448276	1.11895370562591	12	231	viral infectious cycle
GO:0006614	1.79383947513436e-09	21.9544057377049	0.508615320739049	9	105	SRP-dependent cotranslational protein targeting to membrane
GO:0006613	1.95280987937582e-09	21.7265506168667	0.513459276174659	9	106	cotranslational protein targeting to membrane
GO:0045047	1.95280987937582e-09	21.7265506168667	0.513459276174659	9	106	protein targeting to ER
GO:0072599	1.95280987937582e-09	21.7265506168667	0.513459276174659	9	106	establishment of protein localization to endoplasmic reticulum
GO:0019080	2.66776487282486e-09	16.8321513002364	0.731437270777109	10	151	viral genome expression
GO:0019083	2.66776487282486e-09	16.8321513002364	0.731437270777109	10	151	viral transcription
GO:0006413	2.84480329887681e-09	16.712441314554	0.736281226212719	10	152	translational initiation
GO:0016032	3.07835109852793e-09	8.72289407839867	2.18462390146011	15	451	viral reproduction
GO:0022415	3.76473354170724e-09	11.7906006674082	1.2594284132586	12	260	viral reproductive process
GO:0070972	5.0787447552387e-09	19.3183937434201	0.571586741401979	9	118	protein localization to endoplasmic reticulum
GO:0000184	5.47334037527631e-09	19.1414307004471	0.576430696837589	9	119	nuclear-transcribed mRNA catabolic process, nonsense-mediated decay
GO:0006612	4.95047982223002e-08	14.5870901639344	0.741125181648329	9	153	protein targeting to membrane
GO:0043624	4.95047982223002e-08	14.5870901639344	0.741125181648329	9	153	cellular protein complex disassembly
GO:0043241	6.53728633758801e-08	14.0926394542854	0.765344958826379	9	158	protein complex disassembly
GO:0000956	1.16648791459562e-07	13.1136270491803	0.818628468618089	9	169	nuclear-transcribed mRNA catabolic process
GO:0022613	1.20242275180982e-07	10.9813953488372	1.08988997301225	10	225	ribonucleoprotein complex biogenesis
GO:0034623	1.49724472349781e-07	12.7117734724292	0.842848245796139	9	174	cellular macromolecular complex disassembly
GO:0006402	1.81792370708096e-07	12.407411000097	0.862224067538579	9	178	mRNA catabolic process
GO:0032984	1.90686320250573e-07	12.3335583413693	0.867068022974189	9	179	macromolecular complex disassembly
GO:0071843	2.11000230823148e-07	10.2998544395924	1.15770534911079	10	239	cellular component biogenesis at cellular level
GO:0072594	5.76541966419243e-07	10.7334174022699	0.988166908864438	9	204	establishment of protein localization to organelle
GO:0006401	6.00713892035634e-07	10.6779023084644	0.993010864300048	9	205	RNA catabolic process
GO:0071845	8.77339691245854e-06	7.5680476900149	1.37568334371324	9	284	cellular component disassembly at cellular level

GO:0022411 9.81661683224337e-06 7.4574299312533 1.39505916545568 9
288 cellular component disassembly
GO:0033365 1.55450055012712e-05 5.61702986279257 2.29119092104353
11 473 protein localization to organelle
Tissue: Nerve_Tibial=>Muscle_Skeletal Type: asymmetric
SourceGene: ENSG00000239791.1
TargetGeneSet: SEPN1 RP11 RPLP0P6 ZAP70 RPL37A PPIAP16 RPL35A RPL34
RPS23 CTD RPL39P3 RPL7 GS1 AQP9 RPL35 RPL13AP5 RPLP2
ORAOV1 ST13P11 SLC11A2 RPS29 PLEKHD1 C15orf52 RPS3AP6 ZNF774 PDZD9
RPL27 S1PR4 WIZ REPS2 RP13 RP3 RPL10
GOBPID Pvalue OddsRatio ExpCount Count Size Term
GO:0006414 2.1887296438331e-18 139.781914893617 0.167116462528545
11 105 translational elongation
GO:0006415 6.30555352061827e-17 136.24881291548 0.144834267524739 10
91 translational termination
GO:0006614 2.79796175290637e-16 116.056680161943 0.167116462528545
10 105 SRP-dependent cotranslational protein targeting to membrane
GO:0006613 3.08687413255169e-16 114.839743589744 0.168708047885959
10 106 cotranslational protein targeting to membrane
GO:0045047 3.08687413255169e-16 114.839743589744 0.168708047885959
10 106 protein targeting to ER
GO:0072599 3.08687413255169e-16 114.839743589744 0.168708047885959
10 106 establishment of protein localization to endoplasmic reticulum
GO:0070972 9.35135241104897e-16 101.994301994302 0.187807072174936
10 118 protein localization to endoplasmic reticulum
GO:0000184 1.02008682585083e-15 101.051517290049 0.189398657532351
10 119 nuclear-transcribed mRNA catabolic process, nonsense-mediated decay
GO:0019080 1.16958569994575e-14 77.9432624113475 0.240329388969621
10 151 viral genome expression
GO:0019083 1.16958569994575e-14 77.9432624113475 0.240329388969621
10 151 viral transcription
GO:0006413 1.25091808835343e-14 77.3889490790899 0.241920974327036
10 152 translational initiation
GO:0006612 1.33729070090369e-14 76.8423883808499 0.243512559684451
10 153 protein targeting to membrane
GO:0043624 1.33729070090369e-14 76.8423883808499 0.243512559684451
10 153 cellular protein complex disassembly
GO:0043241 1.85483238175808e-14 74.2203742203742 0.251470486471524
10 158 protein complex disassembly
GO:0000956 3.6731013503907e-14 69.0324141267537 0.268977925403086
10 169 nuclear-transcribed mRNA catabolic process
GO:0034623 4.93531947415089e-14 66.9043151969981 0.27693585219016
10 174 cellular macromolecular complex disassembly
GO:0006402 6.21163297175937e-14 65.2930402930403 0.283302193619819
10 178 mRNA catabolic process
GO:0032984 6.57374934802155e-14 64.9021392808375 0.284893778977233
10 179 macromolecular complex disassembly
GO:0072594 2.45722623718009e-13 56.4393338620143 0.324683412912601
10 204 establishment of protein localization to organelle
GO:0006401 2.58110034705589e-13 56.1459566074951 0.326274998270016
10 205 RNA catabolic process
GO:0019058 8.55234752637089e-13 49.4500522102332 0.367656217562798
10 231 viral infectious cycle
GO:0022415 2.78571752229051e-12 43.6246153846154 0.413812192927825
10 260 viral reproductive process
GO:0071845 6.70239269057421e-12 39.7361033127456 0.452010241505778
10 284 cellular component disassembly at cellular level
GO:0022411 7.70023687149939e-12 39.1532927504151 0.458376582935437
10 288 cellular component disassembly
GO:0016032 6.31199390872842e-10 24.397348683063 0.717804996194035 10
451 viral reproduction
GO:0033365 1.00237370241577e-09 23.2015284931052 0.752819874057159
10 473 protein localization to organelle

GO:0006605	1.02311616145228e-09	23.1498673740053	0.754411459414573
10	474	protein targeting
Tissue: Nerve_Tibial=>Muscle_Skeletal	Type:	asymmetric

SourceGene:	FBXO18(ENSG00000134452.12)	"F-box protein, helicase, 18"

TargetGeneSet:	ACAP3	OTUD3	HP1BP3	C1orf63	TRAPPC3	ERMAP	PPAP2B	ACTG1P4	RP11
RC3H1	UCHL5	WNT9A	RPLP0P6	LINC00152	MCM6	RFTN2	RPL37A	SLC23A3
IL17RC	ACAA1	KIAA1143	PTPLB	CCNL1	MYL5	AADAT	RANBP3L	RPS23	CTD
GCNT2	CDKAL1	STK19	AGER	NOTCH4	RPL10A	SNHG5	ELMO1	GNRH1	NR6A1	BMI1
USP54	FGFBP3	RPL13AP5	PIDD	RPLP2	TNNT3	MEN1	NADSYN1	MSANTD2	ANO2
FGFR1OP2	PUS7L	SLC11A2	RPS29	PIAS1	PRC1	RPS15A	GDPD3	C16orf57
NFAT5	CCL16	RPL27	RPS15	PDE4A	RPS16	CARD8	RPS5P2	ACRC	FERP1
LINC00087

GOBPID	Pvalue	OddsRatio	ExpCount	Count	Size	Term
GO:0006414	3.25E-12	35.84962406	0.377828524	10	105	translational elongation
GO:0006415	3.06E-11	36.54367555	0.327451387	9	91	translational termination
GO:0006614	1.13E-10	31.18386628	0.377828524	9	105	SRP-dependent cotranslational protein targeting to membrane
GO:0006613	1.23E-10	30.86022537	0.381426891	9	106	

cotranslational protein targeting to membrane						
GO:0045047	1.23E-10	30.86022537	0.381426891	9	106	protein targeting to ER
GO:0072599	1.23E-10	30.86022537	0.381426891	9	106	establishment of protein localization to endoplasmic reticulum
GO:0070972	3.25E-10	27.4397269	0.424607294	9	118	protein localization to endoplasmic reticulum
GO:0000184	3.50E-10	27.18837209	0.428205661	9	119	"nuclear-transcribed mRNA catabolic process, nonsense-mediated decay"
GO:0000956	3.79E-10	21.32374963	0.608124005	10	169	nuclear-transcribed mRNA catabolic process
GO:0006402	6.31E-10	20.16865079	0.640509307	10	178	mRNA catabolic process
GO:0006401	2.50E-09	17.34310134	0.737665213	10	205	RNA catabolic process

GO:0019080	2.93E-09	21.01424828	0.543353401	9	151
viral genome expression					
GO:0019083	2.93E-09	21.01424828	0.543353401	9	151
viral transcription					
GO:0006413	3.11E-09	20.86583184	0.546951768	9	152
translational initiation					
GO:0006612	3.29E-09	20.71947674	0.550550135	9	153
protein targeting to membrane					
GO:0043624	3.29E-09	20.71947674	0.550550135	9	153
cellular protein complex disassembly					
GO:0043241	4.37E-09	20.01716872	0.568541969	9	158
protein complex disassembly					
GO:0034623	1.02E-08	18.05581395	0.62611584	9	174
cellular macromolecular complex disassembly					

GO:0032984	1.31E-08	17.51860465	0.644107674	9	179
macromolecular complex disassembly					
GO:0072594	4.06E-08	15.24579606	0.734066847	9	204
establishment of protein localization to organelle					
GO:0019058	1.18E-07	13.36612194	0.831222753	9	231
viral infectious cycle					
GO:0022415	3.22E-07	11.79764662	0.935575393	9	260
viral reproductive process					
GO:0071845	6.76E-07	10.74976744	1.021936198	9	284
cellular component disassembly at cellular level					
GO:0022411	7.60E-07	10.59264816	1.036329666	9	288
cellular component disassembly					
GO:0016032	3.87E-06	7.53590325	1.62286347	10	451
viral reproduction					

Tissue: Nerve_Tibial=>Muscle_Skeletal Type: asymmetric

SourceGene: GORASP1(ENSG00000114745.9) "golgi reassembly stacking protein 1, 65kDa"

TargetGeneSet: RPL22 SFN RP4 MFI2 RPL5 RP11 AP4B1 TMEM79 SUPT7L OLA1 RPL15 PROK2 RPL34 SPOCK3 CENPH CTD CTB HCG27 RPL7 EIF3E EIF3H RPS24 PCNXL3 CCND1 RPS25 DNAJC3 TRAPPC6B RPL4 LINGO1 TMEM104 RDH13 PRDX4

GOBPID	Pvalue	OddsRatio	ExpCount	Count	Size	Term
GO:0006413	8.90E-14	59.16321458	0.283994187	10	152	translational initiation
GO:0000184	5.30E-13	65.06363636	0.222337554	9	119	"nuclear-transcribed mRNA catabolic process, nonsense-mediated decay"
GO:0006415	3.64E-12	72.75079264	0.170022836	8	91	translational termination
GO:0006414	1.17E-11	62.18990776	0.196180195	8	105	translational elongation
GO:0006614	1.17E-11	62.18990776	0.196180195	8	105	SRP-dependent cotranslational protein targeting to membrane
GO:0006613	1.27E-11	61.55102041	0.198048578	8	106	cotranslational protein targeting to membrane
GO:0045047	1.27E-11	61.55102041	0.198048578	8	106	protein targeting to ER
GO:0072599	1.27E-11	61.55102041	0.198048578	8	106	establishment of protein localization to endoplasmic reticulum

GO:0000956	1.29E-11	44.575	0.315756695	9	169	nuclear-transcribed mRNA catabolic process
GO:0006402	2.06E-11	42.17455621	0.33257214	9	178	mRNA catabolic process
GO:0070972	3.03E-11	54.79043062	0.220469172	8	118	protein localization to endoplasmic reticulum
GO:0006401	7.33E-11	36.29591837	0.383018476	9	205	RNA catabolic process
GO:0019080	2.21E-10	42.0493191	0.282125804	8	151	viral genome expression
GO:0019083	2.21E-10	42.0493191	0.282125804	8	151	viral transcription
GO:0006612	2.46E-10	41.46352087	0.28586257	8	153	protein targeting to membrane
GO:0043624	2.46E-10	41.46352087	0.28586257	8	153	cellular protein complex disassembly
GO:0043241	3.18E-10	40.06736842	0.295204484	8	158	protein complex disassembly
GO:0034623	6.87E-10	36.16487001	0.325098609	8	174	cellular macromolecular complex disassembly
GO:0032984	8.60E-10	35.09510619	0.334440523	8	179	macromolecular complex disassembly
GO:0072594	2.42E-09	30.56498389	0.381150093	8	204	establishment of protein localization to organelle
GO:0019058	6.45E-09	26.81331131	0.431596429	8	231	viral infectious cycle

GO:0022415 1.63E-08 23.67919799 0.485779531 8 260
viral reproductive process

GO:0071845 3.24E-08 21.58352403 0.530620718 8 284
cellular component disassembly at cellular level

GO:0022411 3.61E-08 21.26917293 0.53809425 8 288
cellular component disassembly

GO:0033365 1.11E-07 15.04310345 0.88374507 9 473
protein localization to organelle

GO:0006605 1.13E-07 15.00967742 0.885613452 9 474
protein targeting

GO:0016032 1.11E-06 13.28834502 0.842640648 8 451
viral reproduction

Tissue: Nerve_Tibial=>Muscle_Skeletal Type: asymmetric
SourceGene: HMGN5(ENSG00000198157.6)
TargetGeneSet: RPL22 AGTRAP C1orf144 HMGN2 MRPS15 RSPO1 KIAA0494 ZFYVE9 FOXD3 RABGGTB RP11 AGL HBXIP HIPK1 RPS27 ATP8B2 FAM189B HCN3 CCT3 XPR1 LINC00467 SRP9 C1orf95 SRSF7 COX7A2L RHOQ CCT4 MKI67IP HSPD1 HSPE1 CLN5 ITPR1 RPL15 LAMB2 RPL24 MYLK MAP6D1 ADD1 SLIT2 PDE5A RPS3A SORBS2 RBBP4P1 NDUFS4 PTCD2 BTF3 TBCA CTB SLIT1 KCNMB1 NPM1 RPL26L1 LRRC16A RPL10A TBX18 AKD1 RPF2 RP3 TRDN LTV1 TAB2 FNDC1 PSMB1 SDK1 EEF1A1P6 MRPL32 SEC61G RPS3AP26 ZSCAN21 FIS1 MRPS33 KRBA1 SFTPC SCARA5 HOOK3 MRPS35 WWP1 EIF3E TTC35 KIAA0020 NTRK2 RPL35 RPL7A DNLZ EDF1 CAMK1D COMMD3 TIMM23 HK1 RPS24 RPS3AP5 SFXN4 IGF2 STIM1 TMEM109 MRPL21 LAMTOR1 TMEM126A SNORA8 PHLDB1 SOX5 POLR2KP1 IKZF4 RNF41 SDSL CCDC92 PUS1 IFT88 MRP63 PRMT5-AS1 C14orf166 ACTN1 BCRP2 SRP14 JMJD8 LYRM1 HMGN2P3 TGFB1I1 PARD6A GABARAPL2 WWOX GCSH C1QBP STX8 KCNJ12 STAC2 VAT1 ANKRD40 MSI2 SRSF1 SLC16A5 TRAPPC8 C18orf21 CNN1 FAM71E1 TSEN34 PPP6R1 SNRPB2 AHCY MYL9 CHD6 TCP10L MRPL40 FOXRED2 NHP2L1 ATXN10 ZC3H12B HMGN5 TRMT2B BEX2 DOCK11 SLC9A6

GOBPID	Pvalue	OddsRatio	ExpCount	Count	Size	Term
GO:0006614	5.73767343535094e-12	17.9582686335404	0.908241644176874	13	105	SRP-dependent cotranslational protein targeting to membrane
GO:0006613	6.49249560224171e-12	17.763920890937	0.916891564597606	13	106	cotranslational protein targeting to membrane
GO:0045047	6.49249560224171e-12	17.763920890937	0.916891564597606	13	106	protein targeting to ER
GO:0072599	6.49249560224171e-12	17.763920890937	0.916891564597606	13	106	establishment of protein localization to endoplasmic reticulum
GO:0070972	2.60182755677225e-11	15.7204931972789	1.02069060964639	13	118	protein localization to endoplasmic reticulum

GO:0006414	1.01566689419554e-10	16.2523551241793	0.908241644176874
12	105	translational elongation
GO:0006612	6.94494928981492e-10	11.7613520408163	1.32343782437202
13	153	protein targeting to membrane
GO:0006415	5.79974161324413e-09	15.2925389157273	0.787142758286624
10	91	translational termination
GO:0000184	6.22590156196839e-09	12.7028914879792	1.02934053006712
11	119	nuclear-transcribed mRNA catabolic process, nonsense-mediated decay
GO:0043624	8.10272368894881e-09	10.6834871022406	1.32343782437202
12	153	cellular protein complex disassembly
GO:0043241	1.16593388123638e-08	10.3139774518123	1.36668742647568
12	158	protein complex disassembly
GO:0072594	2.3080843883101e-08	8.58989341810022	1.76458376582935
13	204	establishment of protein localization to organelle
GO:0034623	3.43845483812788e-08	9.28482464765651	1.50508615320739
12	174	cellular macromolecular complex disassembly
GO:0032984	4.71013040491334e-08	9.00365640400615	1.54833575531105
12	179	macromolecular complex disassembly
GO:0006413	8.04011240743188e-08	9.70729127783999	1.31478790395128
11	152	translational initiation
GO:0000956	2.36908829095149e-07	8.65245391960915	1.46183655110373
11	169	nuclear-transcribed mRNA catabolic process
GO:0006402	3.9945788384366e-07	8.18095388171026	1.53968583489032
11	178	mRNA catabolic process
GO:0019080	7.30958509555701e-07	8.74807277212458	1.30613798353055
10	151	viral genome expression
GO:0019083	7.30958509555701e-07	8.74807277212458	1.30613798353055
10	151	viral transcription
GO:0019058	7.5054439855748e-07	6.8405867377864 1.99813161718912	12
231	viral infectious cycle
GO:0006401	1.61889016522879e-06	7.02893832519443	1.77323368625009
11	205	RNA catabolic process
GO:0022415	2.59639333784596e-06	6.0282614901513 2.24897930939035	12
260	viral reproductive process
GO:0071845	6.43058633558557e-06	5.48698594482041	2.45657739948792
12	284	cellular component disassembly at cellular level
GO:0022411	7.41205255207756e-06	5.40592535590612	2.49117708117085
12	288	cellular component disassembly
GO:0016032	8.03903518619091e-06	4.34424520433695	3.90111410975019
15	451	viral reproduction
GO:0033365	1.41529228327999e-05	4.12901945216356	4.0914123590063 15
473	protein localization to organelle
Tissue: Nerve_Tibial=>Muscle_Skeletal	Type:	cluster

SourceGene:	HMGN5(ENSG00000198157.6)	high mobility group nucleosome binding domain 5

TargetGeneSet: RPL22 AGTRAP C1orf144 HMGN2 MRPS15 RSPO1 KIAA0494 ZFYVE9 FOXD3 RABGGTB RP11 AGL HBXIP HIPK1 RPS27 ATP8B2 FAM189B HCN3 CCT3 XPR1 LINC00467 SRP9 C1orf95 SRSF7 COX7A2L RHOQ CCT4 MKI67IP HSPD1 HSPE1 CLN5 ITPR1 RPL15 LAMB2 RPL24 MYLK MAP6D1 ADD1 SLIT2 PDE5A RPS3A SORBS2 RBBP4P1 NDUFS4 PTCD2 BTF3 TBCA CTB SLIT1 KCNMB1 NPM1 RPL26L1 LRRC16A RPL10A TBX18 AKD1 RPF2 RP3 TRDN LTV1 TAB2 FNDC1 PSMB1 SDK1 EEF1A1P6 MRPL32 SEC61G RPS3AP26 ZSCAN21 FIS1 MRPS33 KRBA1 SFTPC SCARA5 HOOK3 MRPS35 WWP1 EIF3E TTC35 KIAA0020 NTRK2 RPL35 RPL7A DNLZ EDF1 CAMK1D COMMD3 TIMM23 HK1 RPS24 RPS3AP5 SFXN4 IGF2 STIM1 TMEM109 MRPL21 LAMTOR1 TMEM126A SNORA8 PHLDB1 SOX5 POLR2KP1 IKZF4 RNF41 SDSL CCDC92 PUS1 IFT88 MRP63 PRMT5-AS1 C14orf166 ACTN1 BCRP2 SRP14 JMJD8 LYRM1 HMGN2P3 TGFB1I1 PARD6A GABARAPL2 WWOX GCSH C1QBP STX8 KCNJ12 STAC2 VAT1 ANKRD40 MSI2 SRSF1 SLC16A5 TRAPPC8 C18orf21 CNN1 FAM71E1 TSEN34 PPP6R1 SNRPB2 AHCY MYL9 CHD6 TCP10L MRPL40 FOXRED2 NHP2L1 ATXN10 ZC3H12B HMGN5 TRMT2B BEX2 DOCK11 SLC9A6

GOBPID	Pvalue	OddsRatio	ExpCount	Count	Size	Term
GO:0006614	5.74E-12	17.95826863	0.908241644	13	105	SRP-dependent cotranslational protein targeting to membrane
GO:0006613	6.49E-12	17.76392089	0.916891565	13	106	cotranslational protein targeting to membrane

GO:0045047	6.49E-12	17.76392089	0.916891565	13	106
protein targeting to ER					
GO:0072599	6.49E-12	17.76392089	0.916891565	13	106
establishment of protein localization to endoplasmic reticulum					
GO:0070972	2.60E-11	15.7204932	1.02069061	13	118
protein localization to endoplasmic reticulum					
GO:0006414	1.02E-10	16.25235512	0.908241644	12	105

translational elongation

GO:0006612	6.94E-10	11.76135204	1.323437824	13	153

protein targeting to membrane

GO:0006415	5.80E-09	15.29253892	0.787142758	10	91

translational termination

GO:0000184	6.23E-09	12.70289149	1.02934053	11	119

"nuclear-transcribed mRNA catabolic process, nonsense-mediated decay"

GO:0043624	8.10E-09	10.6834871	1.323437824	12	153	cellular protein complex disassembly
GO:0043241	1.17E-08	10.31397745	1.366687426	12	158	protein complex disassembly
GO:0072594	2.31E-08	8.589893418	1.764583766	13	204	establishment of protein localization to organelle
GO:0034623	3.44E-08	9.284824648	1.505086153	12	174	cellular macromolecular complex disassembly

GO:0032984	4.71E-08	9.003656404	1.548335755	12	179	macromolecular complex disassembly
GO:0006413	8.04E-08	9.707291278	1.314787904	11	152	translational initiation
GO:0000956	2.37E-07	8.65245392	1.461836551	11	169	nuclear-transcribed mRNA catabolic process
GO:0006402	3.99E-07	8.180953882	1.539685835	11	178	mRNA catabolic process

GO:0019080 viral genome expression	7.31E-07	8.748072772	1.306137984	10	151	
GO:0019083 viral transcription	7.31E-07	8.748072772	1.306137984	10	151	
GO:0019058 viral infectious cycle	7.51E-07	6.840586738	1.998131617	12	231	
GO:0006401 catabolic process	1.62E-06	7.028938325	1.773233686	11	205	RNA

GO:0022415	2.60E-06	6.02826149	2.248979309	12	260
viral reproductive process					
GO:0071845	6.43E-06	5.486985945	2.456577399	12	284
cellular component disassembly at cellular level					
GO:0022411	7.41E-06	5.405925356	2.491177081	12	288
cellular component disassembly					
GO:0016032	8.04E-06	4.344245204	3.90111411	15	451
viral reproduction					

GO:0033365 1.42E-05 4.129019452 4.091412359 15 473
protein localization to organelle

Tissue: Nerve_Tibial=>Muscle_Skeletal Type: asymmetric

SourceGene: KIAA1456(ENSG00000250305.3) putative methyltransferase

TargetGeneSet: ZBTB48 ADPRHL2 RP11 RPS7 ROCK2 RPL37A RPL35A LMLN ZNF827 MYLIP DEK ZBTB12 RPS18 RPL39P3 HIP1 DBF4 RPS20 RPL30 EEF1D RPL8 FRMD3 RPL35 RPL13AP5 FAU RPS3 RPS25 ATP5G2 RPL41 TPT1 RPL36A SIX1 RPS3AP6 RPLP1 C16orf13 CASP16 RPS15A RPL13 RPS7P1 RPL23A RPL27 ZFP161 RPS15 DAPK3 C19orf43 GLTSCR2 TMC2 C21orf33 TCN2 GS1 PQBP1

GOBPID	Pvalue	OddsRatio	ExpCount	Count	Size	Term
GO:0006414	4.82E-31	150.7059801	0.290637326	19	105	translational elongation
GO:0006415	4.13E-30	160.6998755	0.251885683	18	91	translational termination
GO:0006614	6.86E-29	134.7084639	0.290637326	18	105	SRP-dependent cotranslational protein targeting to membrane

GO:0006613	8.25E-29	133.1683884	0.293405301	18	106	cotranslational protein targeting to membrane
GO:0045047	8.25E-29	133.1683884	0.293405301	18	106	protein targeting to ER
GO:0072599	8.25E-29	133.1683884	0.293405301	18	106	establishment of protein localization to endoplasmic reticulum
GO:0070972	6.59E-28	117.09	0.326620995	18	118	protein localization to endoplasmic reticulum
GO:0000184	7.75E-28	115.9225923	0.32938897	18	119	"nuclear-transcribed mRNA catabolic process, nonsense-mediated decay"
GO:0019080	7.26E-26	87.83458647	0.417964155	18	151	viral genome expression
GO:0019083	7.26E-26	87.83458647	0.417964155	18	151	viral transcription
GO:0006413	8.22E-26	87.17299864	0.420732129	18	152	translational initiation
GO:0006612	9.30E-26	86.52121212	0.423500104	18	153	protein targeting to membrane

GO:0043624	9.30E-26	86.52121212	0.423500104	18	153	
cellular protein complex disassembly						
GO:0043241	1.70E-25	83.40194805	0.437339976	18	158	
protein complex disassembly						
GO:0000956	6.03E-25	77.2667068	0.467787696	18	169	
nuclear-transcribed mRNA catabolic process						
GO:0034623	1.04E-24	74.76398601	0.481627569	18	174	
cellular macromolecular complex disassembly						
GO:0006402	1.59E-24	72.87443182	0.492699467	18	178	mRNA
catabolic process						
GO:0032984	1.77E-24	72.41671372	0.495467442	18	179	
macromolecular complex disassembly						
GO:0019058	3.43E-24	60.59770889	0.639402118	19	231	
viral infectious cycle						
GO:0072594	2.00E-23	62.57331378	0.564666805	18	204	
establishment of protein localization to organelle						
GO:0006401	2.19E-23	62.23432183	0.56743478	18	205	RNA
catabolic process						

GO:0022415	3.40E-23	53.19699664	0.719673379	19	260

viral reproductive process

GO:0071845	8.58E-21	43.50820232	0.786104768	18	284

cellular component disassembly at cellular level

GO:0022411	1.11E-20	42.85151515	0.797176666	18	288

cellular component disassembly

GO:0016032	1.22E-18	29.27700617	1.248356515	19	451

viral reproduction

GO:0033365	2.96E-18	27.81445353	1.309251955	19	473

protein localization to organelle

GO:0006605	8.18E-17	25.0388756	1.312019929	18	474

protein targeting

GO:0042274	1.56E-05	77.81621622	0.049823542	3	18

ribosomal small subunit biogenesis

Tissue: Nerve_Tibial=>Muscle_Skeletal   Type: asymmetric

SourceGene: LPP(ENSG00000145012.8)

TargetGeneSet: RPL22 RPL11 BRD8 LRIF1 RPS27 RPS7 NEB RPL37A GAL3ST2 SCAP RP11 RPL24 ZBTB38 RPL35A LMLN HNRPDL RPL34 LARP1B LSM6 RPS3A TNFAIP8 NUDCD2 CSNK2B RPS18 RPL10A ENPP5 RPL39P3 LTV1 OGDH ZMIZ2 C7orf63 TECPR1 C8orf40 RPS20 RPL30 RPL35 ADRA2A ZNF32-AS2 VPS26A RPS24 FAU CWC15 RPS25 ATP5G2 RPL41 UFM1 TPT1 COMMD6 TIMM9 KLF13 RPS3AP6 RPLP1 TCEB2 NUBP1 RPS15A MIR4519 VMO1 RPL23A RPL27 SEH1L UBA52 SNRPD2 SNRPB2 LINC00493 RANBP1 TNRC6B UXT RPL10

GOBPID	Pvalue	OddsRatio	ExpCount	Count	Size	Term
GO:0006415	6.05E-39	150.9323968	0.36523424	24	91	translational termination
GO:0006414	2.95E-37	124.7233115	0.421424123	24	105	translational elongation
GO:0006614	2.95E-37	124.7233115	0.421424123	24	105	SRP-dependent cotranslational protein targeting to membrane
GO:0006613	3.81E-37	123.1936872	0.425437686	24	106	cotranslational protein targeting to membrane
GO:0045047	3.81E-37	123.1936872	0.425437686	24	106	protein targeting to ER

GO:0072599	3.81E-37	123.1936872	0.425437686	24	106
establishment of protein localization to endoplasmic reticulum

GO:0070972	6.62E-36	107.3767209	0.473600443	24	118
protein localization to endoplasmic reticulum

GO:0000184	8.27E-36	106.2390093	0.477614006	24	119
"nuclear-transcribed mRNA catabolic process, nonsense-mediated decay"

GO:0019080	4.93E-35	85.78042328	0.606048024	25	151
viral genome expression

GO:0019083	4.93E-35	85.78042328	0.606048024	25	151
viral transcription

GO:0006612	7.03E-35	84.42826705	0.614075151	25	153
protein targeting to membrane

GO:0000956	1.00E-33	74.9631734	0.67829216	25	169
nuclear-transcribed mRNA catabolic process

GO:0006402	3.97E-33	70.50901169	0.714414227	25	178	mRNA catabolic process
GO:0006413	4.83E-33	78.66727941	0.610061587	24	152	translational initiation
GO:0043624	5.72E-33	78.05198358	0.614075151	24	153	cellular protein complex disassembly
GO:0043241	1.30E-32	75.11325724	0.634142966	24	158	protein complex disassembly
GO:0072594	1.43E-31	60.15744033	0.818766867	25	204	establishment of protein localization to organelle
GO:0034623	1.52E-31	67.02588235	0.698359975	24	174	cellular macromolecular complex disassembly
GO:0006401	1.63E-31	59.81902357	0.82278043	25	205	RNA catabolic process
GO:0032984	3.11E-31	64.84098672	0.71842779	24	179	macromolecular complex disassembly

GO:0019058	3.63E-30	52.17343336	0.92713307	25	231
viral infectious cycle					
GO:0022415	7.65E-29	45.6415216	1.0435264	25	260
viral reproductive process					
GO:0071845	2.93E-26	38.37013575	1.139851913	24	284
cellular component disassembly at cellular level					
GO:0022411	4.12E-26	37.77807487	1.155906166	24	288
cellular component disassembly					
GO:0016032	1.10E-25	28.69468959	1.810116947	27	451
viral reproduction					
GO:0033365	2.57E-22	23.58123647	1.898415335	25	473
protein localization to organelle					
GO:0006605	2.71E-22	23.52702976	1.902428898	25	474
protein targeting					

GO:0022613 2.52E-07 12.05527211 0.903051692 9 225
ribonucleoprotein complex biogenesis

GO:0071843 4.19E-07 11.31029281 0.959241575 9 239
cellular component biogenesis at cellular level

GO:0042254 2.08E-06 13.77478597 0.598020898 7 149
ribosome biogenesis

Tissue: Nerve_Tibial=>Muscle_Skeletal Type: asymmetric
SourceGene: MORN2(ENSG00000188010.8)
TargetGeneSet: RP1 CLSTN1 MTOR ATP13A2 MAGOH RP4 LRIF1 PMF1
DUSP12 TIMM17A SNRPE SRP9 OBSCN C1D TPRKB DGUOK TMEM131 COA5
UNC50 MMADHC NEB C2orf47 SLC23A3 HDLBP LSM3 SS18L2 VPRBP RAD54L2 RP11
CHMP2B RG9MTD1 CCDC58 PEX5L MYL5 SLBP 7SK LARP1B LSM6 SUB1 TAF9
CTD MRPL22 UBLCP1 SYCP2L BAG6 CSNK2B HNRNPC MDGA1 C6orf226 CUL7
HMGN3 SNHG5 C6orf162 LSM5 DDX56 GS1 ACN9 TRRAP BUD31 CUX1
SPDYE2 NAA38 SSBP1 PCM1 LSM1 UBE2V2 RPS20 ARMC1 TCEB1 C8orf59
OTUD6B POLR2K PLEC DENND1A PRRC2B C10orf31 HSPA14 SAR1A CHCHD1
KAT6B PCGF6 BCCIP IMMP1L API5 CWC15 DCUN1D5 PPHLN1 WIBG UBE3B POMP
ITM2B C14orf166 PSMA3 ERH SLIRP SNW1 C15orf23 MAP2K5
ZNF592 SNRPA1 MRPL28 TSC2 SRRM2 NOMO1 CAMTA2 MYH10 FLII GPR179
PSMG2 FHOD3 IER3IP1 C18orf32 SDHAF1 SNRPB SNRPB2 LINC00493
CEP250 DPM1 ATP5E WRB SF3A1 GGA1 SLC16A8 TNRC6B PSMD10 VBP1
GOBPID Pvalue OddsRatio ExpCount Count Size Term
GO:0000375 2.71123063330777e-11 12.1580756013746 1.51858002906373
15 209 RNA splicing, via transesterification reactions
GO:0008380 1.57731304071045e-10 8.86363636363636 2.34689640855304
17 323 RNA splicing
GO:0000377 2.37050046335867e-10 11.4623481781377 1.48225036329666
14 204 RNA splicing, via transesterification reactions with bulged adenosine as nucleophile
GO:0000398 2.37050046335867e-10 11.4623481781377 1.48225036329666
14 204 nuclear mRNA splicing, via spliceosome
GO:0006397 5.27576295806882e-10 7.58349171870758 2.89910732821258
18 399 mRNA processing
GO:0043248 1.27733847387696e-05 105.455882352941 0.0508615320739049
3 7 proteasome assembly
Tissue: Nerve_Tibial=>Muscle_Skeletal Type: asymmetric

SourceGene: NCOA6(ENSG00000198646.7) nuclear receptor coactivator 6

TargetGeneSet: TMEM51 PPIH ZYG11B COL24A1 RP11 GJA5 NMNAT2 SRGAP3 ASAP2 PTPN4 RPL15 SERP1 BTF3 CTD PRELID2 HNRNPC RPL10A UFL1 RPA3 CLIP2 IMMP2L EXTL3 DCTN6 IKBKB RPL35 FUT11 MARVELD1 CALHM2 GRK5 RPLP2 ELP4 EIF3M COMMD9 C11orf10 PCNXL3 ARHGEF17 NRIP2 KLHDC5 STAT6 DNAJC3-AS1 PLEKHG3 RPLP1 LINGO1 IL16 ZNF768 CDH11 CHD3 STX8 RPS15 USF2 SPINT2 RPS19 SNRPD2 RPS9 RPS5 CSRP2BP ZNFX1-AS1 SUSD2 ARSD ARSD-AS1 RP3 HPRT1

GOBPID	Pvalue	OddsRatio	ExpCount	Count	Size	Term
GO:0006415	9.46E-12	42.48747528	0.289668535	9	91	translational termination
GO:0019080	3.39E-11	28.10086682	0.480658778	10	151	viral genome expression
GO:0019083	3.39E-11	28.10086682	0.480658778	10	151	viral transcription
GO:0006414	3.51E-11	36.25591216	0.334232925	9	105	translational elongation
GO:0006614	3.51E-11	36.25591216	0.334232925	9	105	SRP-dependent cotranslational protein targeting to membrane

GO:0006413	3.63E-11	27.90101721	0.483841949	10	152
translational initiation					
GO:0006613	3.83E-11	35.87963221	0.337416096	9	106
cotranslational protein targeting to membrane					
GO:0045047	3.83E-11	35.87963221	0.337416096	9	106
protein targeting to ER					
GO:0072599	3.83E-11	35.87963221	0.337416096	9	106
establishment of protein localization to endoplasmic reticulum					
GO:0070972	1.01E-10	31.90280188	0.375614144	9	118
protein localization to endoplasmic reticulum					
GO:0000184	1.09E-10	31.61056511	0.378797315	9	119
"nuclear-transcribed mRNA catabolic process, nonsense-mediated decay"					
GO:0072594	6.60E-10	20.34793814	0.649366826	10	204
establishment of protein localization to organelle					
GO:0006612	1.04E-09	24.08952703	0.487025119	9	153
protein targeting to membrane					

GO:0043624	1.04E-09	24.08952703	0.487025119	9	153	cellular protein complex disassembly
GO:0043241	1.38E-09	23.27299111	0.502940973	9	158	protein complex disassembly
GO:0019058	2.21E-09	17.8280543	0.735312435	10	231	viral infectious cycle
GO:0000956	2.51E-09	21.65625	0.537955851	9	169	nuclear-transcribed mRNA catabolic process
GO:0034623	3.25E-09	20.99262899	0.553871704	9	174	cellular macromolecular complex disassembly
GO:0006402	3.97E-09	20.4900048	0.566604387	9	178	mRNA catabolic process
GO:0032984	4.17E-09	20.36804452	0.569787558	9	179	macromolecular complex disassembly
GO:0022415	6.90E-09	15.72777778	0.827624386	10	260	viral reproductive process

GO:0006401	1.37E-08	17.6338941	0.652549997	9	205	RNA catabolic process
GO:0071845	2.24E-07	12.4982801	0.904020483	9	284	cellular component disassembly at cellular level
GO:0022411	2.53E-07	12.31560593	0.916753166	9	288	cellular component disassembly
GO:0016032	1.18E-06	8.795666415	1.435609992	10	451	viral reproduction
GO:0033365	1.81E-06	8.364530838	1.505639748	10	473	protein localization to organelle
GO:0006605	1.85E-06	8.345905172	1.508822919	10	474	protein targeting

Tissue: Nerve_Tibial=>Muscle_Skeletal Type: asymmetric

SourceGene: PHF8(ENSG00000172943.13) PHD finger protein 8 (histone lysine

demethylase)

TargetGeneSet: WDTC1 RP11 DLGAP3 C1orf168 ILF2 IL6R DUSP12
TBX19 TIMM17A RPLP0P6 TMEM131 WIPF1 RFTN2 CLK1 RPL37A LSM3 EBP
P4HTM NISCH ABTB1 IFT122 NPHP3-AS1 MRPL47 PEX5L PARL EVC2
MRFAP1L1 WDR1 UBE2K NUP54 GAB1 NSUN2 RPS23 HIST1H2AG
SYNGAP1 RPL39P3 SERAC1 CLDN15 CAPZA2 OPN1SW ARMC1 FAM92A1 INTS8 ENY2
PUF60 C8orf33 MIRLET7DHG RPL35 C9orf173 HSPA14 FGFBP3 TM9SF3
BCCIP RPLP2 CHRNA10 FADS3 FAU MRPL21 SNORA25 WBP11 CPM METAP2
SNRPF RPL18AP3 RFC3 RPS29 METTL21D ERH POMT2 SLIRP SNW1
MAGEL2 RPS3AP6 MAP2K5 RPLP1 UNC45A C16orf91 PKD1 ALG1 RPS15A
PYDC1 HSD11B2 MBTPS1 MIR22HG ANKFY1 SREBF1 RPS7P1 RPL27 HOXB4 ABCA6
H3F3B RHBDF2 WDR45L RPS15 ANKRD27 ZFP14 TMEM91 RPS19 ZNF17 SNRPB
SNRPB2 SRC ZNF334 HSPA13 LGALS1 RP1 SYTL4 RPL36A

GOBPID	Pvalue	OddsRatio	ExpCount	Count	Size	Term
GO:0006414	1.28E-12	24.55225806	0.632136184	12	105	translational elongation
GO:0006415	6.28E-12	25.84276316	0.54785136	11	91	translational termination
GO:0006614	3.09E-11	21.97228443	0.632136184	11	105	SRP-dependent cotranslational protein targeting to membrane

GO:0006613	3.43E-11	21.73947368	0.638156529	11	106
cotranslational protein targeting to membrane					
GO:0045047	3.43E-11	21.73947368	0.638156529	11	106
protein targeting to ER					
GO:0072599	3.43E-11	21.73947368	0.638156529	11	106
establishment of protein localization to endoplasmic reticulum					
GO:0070972	1.11E-10	19.2851697	0.710400664	11	118
protein localization to endoplasmic reticulum					
GO:0043624	1.15E-10	16.13957447	0.921112726	12	153
cellular protein complex disassembly					

GO:0000184	1.22E-10	19.10526316	0.716421009	11	119
"nuclear-transcribed mRNA catabolic process, nonsense-mediated decay"					
GO:0043241	1.68E-10	15.58136986	0.951214449	12	158
protein complex disassembly					
GO:0072594	2.47E-10	13.03587095	1.228150301	13	204
establishment of protein localization to organelle					
GO:0000956	3.67E-10	14.47847134	1.01743824	12	169
nuclear-transcribed mRNA catabolic process					
GO:0034623	5.14E-10	14.02666667	1.047539963	12	174
cellular macromolecular complex disassembly					

GO:0006402	6.68E-10	13.68481928	1.071621341	12	178	mRNA catabolic process
GO:0032984	7.13E-10	13.60191617	1.077641686	12	179	macromolecular complex disassembly
GO:0019080	1.59E-09	14.70526316	0.909072037	11	151	viral genome expression
GO:0019083	1.59E-09	14.70526316	0.909072037	11	151	viral transcription
GO:0006413	1.71E-09	14.59994401	0.915092381	11	152	translational initiation

GO:0006612	1.83E-09	14.49610823	0.921112726	11	153	protein targeting to membrane
GO:0006605	2.63E-09	7.390403251	2.853643346	17	474	protein targeting
GO:0006401	3.36E-09	11.74797927	1.234170646	12	205	RNA catabolic process
GO:0071845	1.26E-07	8.289411765	1.70977787	12	284	cellular component disassembly at cellular level
GO:0033365	1.28E-07	6.325509461	2.847623002	15	473	protein localization to organelle
GO:0019058	1.32E-07	9.305263158	1.390699606	11	231	viral infectious cycle

GO:0022411 1.46E-07 8.166956522 1.733859248 12 288
cellular component disassembly

GO:0022415 4.33E-07 8.204660748 1.565289599 11 260
viral reproductive process

GO:0016032 1.57E-05 5.075170843 2.71517542 12 451
viral reproduction

Tissue: Muscle_Skeletal=>Nerve_Tibial Type: asymmetric

SourceGene: PLXNA1(ENSG00000114554.7) plexin A1

TargetGeneSet: GNB1 RPL22 SLC6A9 RPS8 RP11 RPS27 SYT12 ATP1B1
TP53BP2 TGIF2P1 ASAP2 HPCAL1 TMEM178 UBC CLASP1 BSN-AS2 RPL29 RPS3A
ADAMTS16 CTB RPL10A GLI3 ARPC1B FAM200A XKR4 EIF3E RPS6

S1PR3 RPL12 RPS3AP5 NAV2 AMBRA1 GANAB RDX COMMD6 DICER1-AS1
DTWD1 RPS3AP6 NPTN RPL13 RPL26 KANSL1-AS1 CTD RPL18 ZNF419
SNORD86 FOXRED2 MICALL1 RP2 SMC1A NLGN3 RPL39 RPS4Y1

GOBPID	Pvalue	OddsRatio	ExpCount	Count	Size	Term
GO:0043624	1.19E-22	79.39937435	0.391737596	16	153	cellular protein complex disassembly
GO:0000184	1.76E-22	93.81555944	0.304684797	15	119	"nuclear-transcribed mRNA catabolic process, nonsense-mediated decay"
GO:0043241	2.03E-22	76.5767941	0.404539478	16	158	protein complex disassembly
GO:0006415	2.95E-22	113.3359684	0.232994256	14	91	translational termination
GO:0034623	1.00E-21	68.74502712	0.445505501	16	174	cellular macromolecular complex disassembly
GO:0032984	1.60E-21	66.61291265	0.458307384	16	179	macromolecular complex disassembly
GO:0006414	2.48E-21	95.80602007	0.268839527	14	105	translational elongation
GO:0006614	2.48E-21	95.80602007	0.268839527	14	105	SRP-dependent cotranslational protein targeting to membrane

GO:0006613	2.85E-21	94.75803403	0.271399903	14	106	
cotranslational protein targeting to membrane						
GO:0045047	2.85E-21	94.75803403	0.271399903	14	106	
protein targeting to ER						
GO:0072599	2.85E-21	94.75803403	0.271399903	14	106	
establishment of protein localization to endoplasmic reticulum						
GO:0006413	8.10E-21	71.05341739	0.38917722	15	152	
translational initiation						
GO:0070972	1.38E-20	83.7541806	0.30212442	14	118	
protein localization to endoplasmic reticulum						
GO:0000956	4.17E-20	63.13459268	0.432703619	15	169	
nuclear-transcribed mRNA catabolic process						
GO:0006402	9.28E-20	59.61098717	0.455747007	15	178	mRNA
catabolic process						
GO:0019080	4.98E-19	63.43319581	0.386616843	14	151	
viral genome expression						
GO:0019083	4.98E-19	63.43319581	0.386616843	14	151	
viral transcription						

GO:0006612	6.01E-19	62.51172975	0.391737596	14	153	
protein targeting to membrane						
GO:0006401	8.05E-19	51.0430622	0.524877171	15	205	RNA
catabolic process						
GO:0071845	2.89E-18	40.21606254	0.72714691	16	284	
cellular component disassembly at cellular level						
GO:0022411	3.61E-18	39.61344538	0.737388416	16	288	
cellular component disassembly						
GO:0072594	3.66E-17	45.56887872	0.522316795	14	204	
establishment of protein localization to organelle						
GO:0019058	2.11E-16	39.82328191	0.591446959	14	231	
viral infectious cycle						
GO:0022415	1.11E-15	35.05691057	0.665697876	14	260	
viral reproductive process						
GO:0033365	2.06E-13	20.77610163	1.211058058	15	473	
protein localization to organelle						
GO:0006605	2.12E-13	20.72935235	1.213618435	15	474	
protein targeting						

GO:0016032 2.16E-12 19.4685106 1.154729776 14 451
viral reproduction

Tissue: Muscle_Skeletal=>Nerve_Tibial Type: asymmetric
SourceGene: RP11-241I20.3(ENSG00000234335.1)
TargetGeneSet: RPL22 PQLC2 RPL11 RP4 PDC RP11 GALNT2 PREB PIGF
UBC RPL31 FOXD4L1 BIN1 RPL32 RPP14 RPL35A MSANTD1 HERC5 RPL34 CTD
RPL37 MIP HOMER1 ZFYVE16 RPS23 TCF7 GABRP RPL10A RPL39P3 HDAC2
MRPS12 PERP SNX9 STEAP1B HNRNPA2B1 SLC12A9 SHH RPS20 GRIP1
RPL30 PUF60 RPS6 RPL12 SLC27A4 COBRA1 FBXO18 RPS24 RPL13AP5
RPS13 MTA2 LRRN4CL RNU2-2 FAU CCL13 CDK2AP2 RPS3 RPS25 HYOU1
HEBP1 GXYLT1 PFDN5 RPL41 NACA RPL14P1 DAO RPS29 KIAA0317 SLTM
RPS3AP6 RPS15A TMC7 FBXL19 RPL26 ARHGAP44 RPL23A RPL17 PNMT
ERBB2 RPL27 PHB ARHGDIA LMNB2 UBA52 RPS16 ZNF780A ZNF235 SNRPD2
RPL18 ZNF534 RPL28 ZNFX1-AS1 TAF4 RPS21 ZNF74 BCR GAL3ST1
EIF3L RANGAP1 PPP1R3F RP13 RAB40A GPC4 MTM1 HAUS7 PLXNB3 MT

GOBPID	Pvalue	OddsRatio	ExpCount	Count	Size	Term
GO:0006415	1.2517819831294e-47	127.402873563218	0.560445643900076	31	91	translational termination
GO:0006414	2.38409111527322e-45	103.198508853681	0.646668050653934	31	105	translational elongation
GO:0006614	2.38409111527322e-45	103.198508853681	0.646668050653934	31	105	SRP-dependent cotranslational protein targeting to membrane
GO:0006613	3.35632427922435e-45	101.815402298851	0.652826793993495	31	106	cotranslational protein targeting to membrane
GO:0045047	3.35632427922435e-45	101.815402298851	0.652826793993495	31	106	protein targeting to ER
GO:0072599	3.35632427922435e-45	101.815402298851	0.652826793993495	31	106	establishment of protein localization to endoplasmic reticulum
GO:0070972	1.54020609043224e-43	87.6981767736821	0.726731714068231	31	118	protein localization to endoplasmic reticulum
GO:0000184	2.0746181668463e-43	86.6955329153605	0.732890457407792	31	119	nuclear-transcribed mRNA catabolic process, nonsense-mediated decay
GO:0019080	1.19778126380658e-41	67.1938670204924	0.929970244273753	32	151	viral genome expression
GO:0019083	1.19778126380658e-41	67.1938670204924	0.929970244273753	32	151	viral transcription
GO:0006413	1.51132929845921e-41	66.6292397660819	0.936128987613314	32	152	translational initiation
GO:0006612	1.22704965767786e-39	62.3855285472018	0.942287730952875	31	153	protein targeting to membrane
GO:0043624	1.22704965767786e-39	62.3855285472018	0.942287730952875	31	153	cellular protein complex disassembly
GO:0043241	3.64283852177859e-39	59.90836274776	0.973081447650682	31	158	protein complex disassembly
GO:0000956	3.49951970397202e-38	55.0904547726137	1.04082762438586	31	169	nuclear-transcribed mRNA catabolic process
GO:0034623	9.26909437636963e-38	53.1455268869062	1.07162134108366	31	174	cellular macromolecular complex disassembly
GO:0006402	1.97576698886417e-37	51.684846352334	1.09625631444191	31	178	mRNA catabolic process
GO:0032984	2.38021148919808e-37	51.3320130475303	1.10241505778147	31	179	macromolecular complex disassembly
GO:0072594	3.91513174939744e-37	46.3157894736842	1.2563836412705	32		

204	establishment of protein localization to organelle
GO:0006401	2.08165844613826e-35	43.5818470075307	1.26254238461006
31	205	RNA catabolic process
GO:0019058	2.57901084196969e-35	39.9555673102354	1.42266971143865
32	231	viral infectious cycle
GO:0022415	1.31789591896921e-33	34.8020929516774	1.60127326828593
32	260	viral reproductive process
GO:0071845	7.43165661525524e-31	29.8063922584162	1.7490831084354 31
284	cellular component disassembly at cellular level
GO:0022411	1.15646006109395e-30	29.3341607406414	1.77371808179365
31	288	cellular component disassembly
GO:0006605	4.45999507696312e-29	20.5561883067578	2.91924434295204
35	474	protein targeting
GO:0033365	8.38935600606502e-28	19.6057983019259	2.91308559961248
34	473	protein localization to organelle
GO:0016032	3.45248063126365e-27	19.6578947368421	2.77759324614214
33	451	viral reproduction
GO:0042274	6.36193153430945e-08	65.7005494505494	0.110857380112103
5	18	ribosomal small subunit biogenesis
GO:0022613	1.19098339368093e-06	8.32911392405063	1.38571725140129
10	225	ribonucleoprotein complex biogenesis
GO:0071843	2.0552278343414e-06	7.81217179813167	1.47193965815514
10	239	cellular component biogenesis at cellular level
GO:0006364	3.46465097984344e-06	12.5540608498868	0.640509307314373
7	104	rRNA processing
GO:0042254	3.69092665497524e-06	9.96129936082655	0.91765275759463
8	149	ribosome biogenesis
GO:0016072	5.03748474613913e-06	11.8177835661852	0.67746176735174
7	110	rRNA metabolic process
Tissue: Muscle_Skeletal=>Nerve_Tibial	Type:	asymmetric
SourceGene:	RP11-302M6.4(ENSG00000232236.1)
TargetGeneSet:	RPS8	RP11	RP4	CAMSAP2	SLC35F3	EIF3FP3	FGF5	CTB
HLA-DOA	RPL10A	ESRP1	BAALC	RPS6	RPL12	RPL7A	LINC00173		RPS15A
RPL26	CCDC144C		CDR2L	NFATC1	CTD	FTL	RPL28	FIZ1	ZNF787
RPL10
GOBPID	Pvalue	OddsRatio	ExpCount	Count	Size	Term
GO:0006415	1.64695369752928e-15	143.171840354767	0.12594284132586
9	91	translational termination
GO:0006414	6.25396002161484e-15	122.173295454545	0.1453186630683 9
105	translational elongation
GO:0006614	6.25396002161484e-15	122.173295454545	0.1453186630683 9
105	SRP-dependent cotranslational protein targeting to membrane
GO:0006613	6.8295141899155e-15	120.9053420806	0.146702650335617	9
106	cotranslational protein targeting to membrane
GO:0045047	6.8295141899155e-15	120.9053420806	0.146702650335617	9
106	protein targeting to ER
GO:0072599	6.8295141899155e-15	120.9053420806	0.146702650335617	9
106	establishment of protein localization to endoplasmic reticulum
GO:0070972	1.84411327786449e-14	107.504587155963	0.163310497543423
9	118	protein localization to endoplasmic reticulum
GO:0000184	1.99361931307001e-14	106.519834710744	0.16469448481074
9	119	nuclear-transcribed mRNA catabolic process, nonsense-mediated decay
GO:0019080	1.77829104397303e-13	82.330985915493 0.208982077364888	9
151	viral genome expression
GO:0019083	1.77829104397303e-13	82.330985915493 0.208982077364888	9
151	viral transcription
GO:0006413	1.8889053476204e-13	81.7495232040687	0.210366064632205
9	152	translational initiation
GO:0006612	2.00557506904523e-13	81.1761363636364	0.211750051899523
9	153	protein targeting to membrane
GO:0043624	2.00557506904523e-13	81.1761363636364	0.211750051899523
9	153	cellular protein complex disassembly
GO:0043241	2.69020382903067e-13	78.424649176327 0.218669988236108	9

158	protein complex disassembly
GO:0000956	4.96844691928321e-13	72.9767045454546	0.233893848176597
9	169	nuclear-transcribed mRNA catabolic process
GO:0034623	6.47784464322384e-13	70.7404958677686	0.240813784513182
9	174	cellular macromolecular complex disassembly
GO:0006402	7.96437183648954e-13	69.0467993544917	0.246349733582451
9	178	mRNA catabolic process
GO:0032984	8.38021161068123e-13	68.6358288770054	0.247733720849768
9	179	macromolecular complex disassembly
GO:0072594	2.74006229750891e-12	59.7314685314685	0.282333402532697
9	204	establishment of protein localization to organelle
GO:0006401	2.86389527912518e-12	59.4225417439703	0.283717389800014
9	205	RNA catabolic process
GO:0019058	8.40737870340574e-12	52.3673218673219	0.31970105875026
9	231	viral infectious cycle
GO:0022415	2.43158646465357e-11	46.2223831944947	0.359836689502457
9	260	viral reproductive process
GO:0071845	5.35711693096786e-11	42.1170247933884	0.393052383918068
9	284	cellular component disassembly at cellular level
GO:0022411	6.06971959474303e-11	41.5014662756598	0.398588332987337
9	288	cellular component disassembly
GO:0016032	3.21091968451894e-09	25.8948992184286	0.62417825756003
9	451	viral reproduction
GO:0033365	4.87194104762567e-09	24.6283307210031	0.654625977441008
9	473	protein localization to organelle
GO:0006605	4.96274204060114e-09	24.5736070381232	0.656009964708325
9	474	protein targeting
GO:0022613	1.12128380665978e-05	21.5318181818182	0.311397135146357
5	225	ribonucleoprotein complex biogenesis
GO:0071843	1.50187593401244e-05	20.2236467236467	0.330772956888797
5	239	cellular component biogenesis at cellular level
Tissue: Muscle_Skeletal=>Nerve_Tibial	Type:	asymmetric
SourceGene:	RP11-307I14.2(ENSG00000220105.2)
TargetGeneSet:	RP4	RP11	RPL11	RPS3A	RPL10A	EPHB6	RPS20	RPL7
POLR1E	USP2	GEMIN2	RPS3AP6	RPL17
GOBPID	Pvalue	OddsRatio	ExpCount	Count	Size	Term
GO:0006415	4.7286271313409e-11	168.870588235294	0.0755657047955159
6	91	translational termination
GO:0006414	1.13596912439202e-10	144.848484848485	0.0871911978409799
6	105	translational elongation
GO:0006614	1.13596912439202e-10	144.848484848485	0.0871911978409799
6	105	SRP-dependent cotranslational protein targeting to membrane
GO:0006613	1.20369630057904e-10	143.39	0.0880215902013702	6	106
cotranslational protein targeting to membrane
GO:0045047	1.20369630057904e-10	143.39	0.0880215902013702	6	106
protein targeting to ER
GO:0072599	1.20369630057904e-10	143.39	0.0880215902013702	6	106
establishment of protein localization to endoplasmic reticulum
GO:0070972	2.31510341395467e-10	127.919642857143	0.0979862985260536
6	118	protein localization to endoplasmic reticulum
GO:0000184	2.43715605442804e-10	126.778761061947	0.0988166908864438
6	119	nuclear-transcribed mRNA catabolic process, nonsense-mediated decay
GO:0019080	1.03374025467577e-09	98.5793103448276	0.125389246418933
6	151	viral genome expression
GO:0019083	1.03374025467577e-09	98.5793103448276	0.125389246418933
6	151	viral transcription
GO:0006413	1.07583659143465e-09	97.8972602739726	0.126219638779323
6	152	translational initiation
GO:0006612	1.11934651153124e-09	97.2244897959184	0.127050031139714
6	153	protein targeting to membrane
GO:0043624	1.11934651153124e-09	97.2244897959184	0.127050031139714
6	153	cellular protein complex disassembly
GO:0043241	1.35943709635315e-09	93.9934210526316	0.131201992941665

6	158	protein complex disassembly
GO:0000956	2.04064203149548e-09	87.5828220858896	0.140336308905958
6	169	nuclear-transcribed mRNA catabolic process
GO:0034623	2.43273019696167e-09	84.9464285714286	0.144488270707909
6	174	cellular macromolecular complex disassembly
GO:0006402	2.78967451431039e-09	82.9476744186046	0.147809840149471
6	178	mRNA catabolic process
GO:0032984	2.885389111653e-09	82.4624277456647	0.148640232509861
6	179	macromolecular complex disassembly
GO:0072594	6.33155322571186e-09	71.9242424242424	0.169400041519618
6	204	establishment of protein localization to organelle
GO:0006401	6.52010748867559e-09	71.5577889447236	0.170230433880008
6	205	RNA catabolic process
GO:0019058	1.33344527646106e-08	63.1733333333333	0.191820635250156
6	231	viral infectious cycle
GO:0022415	2.70267879691018e-08	55.8464566929134	0.215902013701474
6	260	viral reproductive process
GO:0071845	4.57347393629197e-08	50.9388489208633	0.235831430350841
6	284	cellular component disassembly at cellular level
GO:0022411	4.97033799076331e-08	50.2021276595745	0.239152999792402
6	288	cellular component disassembly
GO:0016032	7.04124660609916e-07	31.447191011236 0.374506954536018	6
451	viral reproduction
GO:0033365	9.30996519068124e-07	29.9186295503212	0.392775586464605
6	473	protein localization to organelle
GO:0006605	9.42588157413463e-07	29.8525641025641	0.393605978824995
6	474	protein targeting
Tissue: Muscle_Skeletal=>Skin_Sun_Exposed_Lower_leg	Type:	asymmetric
SourceGene:	CDKN2D(ENSG00000129355.5)
TargetGeneSet:	CLCN6	SNHG3	ZNF691	PALMD	FCGR1B	PPIAL4B	CHD1L	PRUNE	SHC1
RHBG	SUPT7L	PLB1	MTHFD2	EN1	TANC1	SLC6A6	RP11	NBEAL2	MRPS18AP1
QRICH1	HNRPDL	CCDC127	NSUN2	LIFR-AS1		LARP1	BEND3	MTHFD1L	MEOX2
TRIM4	YBX1P2	CHPF2	TNFRSF10B		HEATR7A	MIRLET7D		FPGS	OTUD1
LZTS2	PTPRE	CTD	METTL15	TBRG1	DHX37	SPRY2	CBLN3	CDKL1	CRIP2
CYP19A1	SCAPER	CLCN7	EME2	CA4	LINC00338		NPLOC4	ZNF844	LGI4
CAPN12	RTN2	ETFB	FAM113A	ASXL1	TRAPPC10		FTCD	PI4KA	CES5AP1
SREBF2	NOX1	MTMR1
GOBPID	Pvalue	OddsRatio	ExpCount	Count	Size	Term
GO:0006730	4.40453268055253e-08	66.5694444444444	0.0996470832468341
5	32	one-carbon metabolic process
GO:0006760	6.95682568325645e-07	73.8741976893453	0.071621341083662
4	23	folic acid-containing compound metabolic process
GO:0009396	3.33486209636152e-06	146.928571428571	0.0311397135146357
3	10	folic acid-containing compound biosynthetic process
GO:0042558	4.00301924421348e-06	45.2399685287175	0.108988997301225
4	35	pteridine-containing compound metabolic process
GO:0046653	1.5360388194886e-05	79.0824175824176	0.0498235416234171
3	16	tetrahydrofolate metabolic process
Tissue: Muscle_Skeletal=>Skin_Sun_Exposed_Lower_leg	Type:	asymmetric
SourceGene:	CELA2B(ENSG00000215704.4)
TargetGeneSet:	MTND1P23		CELA2A	CELA3B	CELA3A	ODF2L	REG1A	LARS2	RP11
CLPS	RP1	CPA2	CPA1	PRSS1	PNLIP	PNLIPRP1		PNLIPRP2
PLA2G1B	GP2	CTRB1	THAP8	DHDH	KLK3	NXT1	ETS2	CECR9
GOBPID	Pvalue	OddsRatio	ExpCount	Count	Size	Term
GO:0007586	1.21835570087734e-10	70.2352941176471	0.158397342744447
7	109	digestion
GO:0044241	4.45140475278301e-09	308.427807486631	0.021797799460245
4	15	lipid digestion
Tissue: Muscle_Skeletal=>Skin_Sun_Exposed_Lower_leg	Type:	asymmetric
SourceGene:	CHPT1(ENSG00000111666.5)
TargetGeneSet:	RPL11	TAF12	ALG6	PRUNE	PSMB4	BRP44	C1orf35	HIST3H2A
RAB4A	RPS7	UCN	DPY30	UBC	TPRKB	COX5B	OXSM	DALRD3	RASSF1	LIAS
NUDT10	OSTC	PRMT10	UBE2B	CAMLG	MRPL22	CTB	C6orf130		MRPL2

COX7A2 ASF1A C6orf72 GTF2H5 BRP44L LINC00174 TMEM60 FNTA C8orf76
MAPK15 C9orf123 CCL19 RP11 CLTA FAM206A ENTPD2 ATP5C1 FAM188A
COMMD3 CUL2 RPL13AP5 CPXM2 RARRES3 KBTBD3 PLEKHG6 GPN3 MRP63 GPX2
COX16 C15orf24 C16orf48 RPS7P1 CCL13 RPL27 ARHGAP27
C18orf32 SEC11C XAB2 SLC25A42 FAM98C CLDND2 TNNI3 TRAPPC2
SPEF1 CDK5RAP1 PSMA7 ATP5J PMM1 IL3RA COX7B PSMD10
GOBPID Pvalue OddsRatio ExpCount Count Size Term
GO:0031398 1.68165736274848e-05 12.6340023612751 0.544875787142758
6 127 positive regulation of protein ubiquitination
Tissue: Muscle_Skeletal=>Skin_Sun_Exposed_Lower_leg Type: asymmetric
SourceGene: CTD-2576F9.2(ENSG00000260521.1)
TargetGeneSet: RP1 AKIRIN1 RP11 USP21 HNRNPU COQ10B ZNF654 ERAP1
ZCCHC10 DIAPH1 PGBD1 DDX56 RPS3AP34 PTDSS1 PABPC1 FAM91A1 ATP6V1G1
FAM73B ACBD5 FAM35A EIF6 BBOX1 AHNAK STT3A GOLT1B CCDC65 PCBP2 TBK1
ACTR6 ABHD13 SLC22A17 MBIP MED6 YLPM1 WDR76 CTD CDH1
TERF2IP RP13 RPA1 DYNLL2 EEF2 PRKCSH WDR83 ZNF506 SS18L1 OSBPL2
MAFIPL SMC1A MSN
GOBPID Pvalue OddsRatio ExpCount Count Size Term
GO:0000377 7.24601949011397e-07 16.8390862944162 0.522316794685489
7 204 RNA splicing, via transesterification reactions with bulged
adenosine as nucleophile
GO:0000398 7.24601949011397e-07 16.8390862944162 0.522316794685489
7 204 nuclear mRNA splicing, via spliceosome
GO:0000375 8.52766533219009e-07 16.416501650165 0.535118676908172 7
209 RNA splicing, via transesterification reactions
GO:0008380 1.5104647807121e-05 10.4099156118143 0.827001591585357
7 323 RNA splicing
Tissue: Muscle_Skeletal=>Skin_Sun_Exposed_Lower_leg Type: asymmetric
SourceGene: ENSG00000232926.1
TargetGeneSet: PGD CTPS SZT2 RP4 NAP1L4P1 RP11 MLLT11 CCT3
ARHGEF11 GDF7 SLC5A6 FNDC4 RPL36AP15 MREG TRIP12 SLC4A7
NT5DC2 HTR1F LRRC58 KPNA1 SEC61A1 SRPRB UBQLN4P1 LETM1 MT1P2
ACSL1 NSUN2 SV2C HOMER1 VDAC1 LARP1 PRELID1 E2F3 XPO5 RP1
BEND3 LINC00473 FBXL18 SEPT7P3 TRRAP TRIM4 TFR2 ADCK2 ABCF2
UBE3C PRKDC MAPRE1P1 HEATR7A FAM120A DDX21 UNC5B TMEM123 NOLC1
TP53I11 RPS6KA4 PITPNM1 PCSK7 MARS CYP27B1 XPOT MVK EP400 MYCBP2
IFI27L2 TYRO3 COX5A ZNF592 FURIN TRAF7 CTD FAM86A USP7 ABCC1
SLC7A5 ACACA ACLY PSME3 PYCR1 FASN LMNB2 PNPLA6 DNMT1 CILP2
TMEM145 GART TRAPPC10 SEPT5 PPPDE2 SREBF2 PNPLA3 HUWE1 ZMAT1
ZDHHC9 NSDHL
GOBPID Pvalue OddsRatio ExpCount Count Size Term
GO:0035338 1.64408893497176e-06 60.3571428571429 0.0896823749221507
4 18 long-chain fatty-acyl-CoA biosynthetic process
GO:0035336 2.58360213421859e-06 52.8051470588235 0.0996470832468341
4 20 long-chain fatty-acyl-CoA metabolic process
GO:0046949 2.58360213421859e-06 52.8051470588235 0.0996470832468341
4 20 fatty-acyl-CoA biosynthetic process
GO:0035384 3.17951037573131e-06 49.6955017301038 0.104629437409176
4 21 thioester biosynthetic process
GO:0071616 3.17951037573131e-06 49.6955017301038 0.104629437409176
4 21 acyl-CoA biosynthetic process
GO:0035337 5.5816118277697e-06 42.2323529411765 0.119576499896201
4 24 fatty-acyl-CoA metabolic process
GO:0019432 6.35430074420932e-06 22.2807835820896 0.26406477060411
5 53 triglyceride biosynthetic process
GO:0046460 7.64369646350815e-06 21.3865671641791 0.274029478928794
5 55 neutral lipid biosynthetic process
GO:0046463 7.64369646350815e-06 21.3865671641791 0.274029478928794
5 55 acylglycerol biosynthetic process
Tissue: Muscle_Skeletal=>Skin_Sun_Exposed_Lower_leg Type: asymmetric
SourceGene: ENSG00000235978.2
TargetGeneSet: RP11 WDR3 TMOD4 UCK2 ADCY3 SLC5A6 CAD RGPD2
PIKFYVE NT5DC2 C3orf26 PLXNA1 HES1 LETM1 TBC1D14 LARP1 NOP16 PAK1IP1

POU5F1 MDN1 TRRAP TFR2 IMPDH1 MAK16 RDH10 CTA C9orf114
OR52N4 NEU3 DDN DNAJC14 UTP20 DDX55 DHX37 EP400 DDX51 TMTC4
ATP11A SPINT1 CTD PDPR KCNAB3 C17orf67 LINC00338 DOT1L
TRMT1 SNORA68 CYP2G1P ZNF587 ACTG1P3 NOP56 PTPN1 GART RRP1B MCM3AP
NBEAP3 ADSL CLCN5 RHOXF2
GOBPID Pvalue OddsRatio ExpCount Count Size Term
GO:0009156 2.8836915130135e-08 72.9361054766734 0.0917583558231264
5 34 ribonucleoside monophosphate biosynthetic process
GO:0009124 4.49086546209502e-08 66.0845588235294 0.0998546813369317
5 37 nucleoside monophosphate biosynthetic process
GO:0009161 5.90853733529536e-08 62.1885813148789 0.105252231679469
5 39 ribonucleoside monophosphate metabolic process
GO:0009123 1.38749459309604e-07 51.5459110473458 0.124143657878348
5 46 nucleoside monophosphate metabolic process
GO:0009112 6.88112536845422e-07 36.394523326572 0.170022835789911 5
63 nucleobase metabolic process
GO:0009260 4.08040004898645e-06 24.7871972318339 0.242889765414158
5 90 ribonucleotide biosynthetic process
Tissue: Skin_Sun_Exposed_Lower_leg=>Muscle_Skeletal Type: asymmetric
SourceGene: ENSG00000236935.1
TargetGeneSet: SFPQ ACBD6 RP11 RPL10A RPL35 RPS25 RPL41 RPLP1
RPL13 SNORD12 ARSFP1
GOBPID Pvalue OddsRatio ExpCount Count Size Term
GO:0006415 1.46219002870474e-12 506.752941176471 0.0503771365303439
6 91 translational termination
GO:0006414 3.52440227959429e-12 434.666666666667 0.0581274652273199
6 105 translational elongation
GO:0006614 3.52440227959429e-12 434.666666666667 0.0581274652273199
6 105 SRP-dependent cotranslational protein targeting to membrane
GO:0006613 3.73542049121313e-12 430.29 0.0586810601342468 6 106
cotranslational protein targeting to membrane
GO:0045047 3.73542049121313e-12 430.29 0.0586810601342468 6 106
protein targeting to ER
GO:0072599 3.73542049121313e-12 430.29 0.0586810601342468 6 106
establishment of protein localization to endoplasmic reticulum
GO:0070972 7.20504798139751e-12 383.866071428571 0.065324199017369
6 118 protein localization to endoplasmic reticulum
GO:0000184 7.58671052977975e-12 380.442477876106 0.0658777939242959
6 119 nuclear-transcribed mRNA catabolic process, nonsense-mediated decay
GO:0019080 3.24266868858932e-11 295.820689655172 0.0835928309459553
6 151 viral genome expression
GO:0019083 3.24266868858932e-11 295.820689655172 0.0835928309459553
6 151 viral transcription
GO:0006413 3.37552499800433e-11 293.77397260274 0.0841464258528822 6
152 translational initiation
GO:0006612 3.51288101484549e-11 291.755102040816 0.084700020759809
6 153 protein targeting to membrane
GO:0043624 3.51288101484549e-11 291.755102040816 0.084700020759809
6 153 cellular protein complex disassembly
GO:0043241 4.27147087778567e-11 282.059210526316 0.0874679952944433
6 158 protein complex disassembly
GO:0000956 6.42877757668172e-11 262.822085889571 0.0935575392706387
6 169 nuclear-transcribed mRNA catabolic process
GO:0034623 7.67317996887295e-11 254.910714285714 0.096325513805273
6 174 cellular macromolecular complex disassembly
GO:0006402 8.80746571102475e-11 248.912790697674 0.0985398934329804
6 178 mRNA catabolic process
GO:0032984 9.11183454247686e-11 247.456647398844 0.0990934883399073
6 179 macromolecular complex disassembly
GO:0072594 2.01147052447413e-10 215.833333333333 0.112933361013079
6 204 establishment of protein localization to organelle
GO:0006401 2.07186910357011e-10 214.733668341709 0.11486955920006
6 205 RNA catabolic process

GO:0019058	4.26375714687163e-10	189.573333333333	0.127880423500104
6	231	viral infectious cycle
GO:0022415	8.70238502331617e-10	167.586614173228	0.143934675800983
6	260	viral reproductive process
GO:0071845	1.48114719337377e-09	152.859712230216	0.157220953567227
6	284	cellular component disassembly at cellular level
GO:0022411	1.61122524367783e-09	150.648936170213	0.159435333194935
6	288	cellular component disassembly
GO:0016032	2.37432063915819e-08	94.3685393258427	0.249671303024012
6	451	viral reproduction
GO:0033365	3.15616285494933e-08	89.7815845824411	0.261850390976403
6	473	protein localization to organelle
GO:0006605	3.19623654221233e-08	89.5833333333333	0.26240398588333
6	474	protein targeting
Tissue: Muscle_Skeletal=>Skin_Sun_Exposed_Lower_leg	Type:	cluster
SourceGene:	FABP4(ENSG00000170323.4)
TargetGeneSet:	MTND2P28	CCNL2	TMEM240	FBXO44	ZBTB40	SYF2	C1orf63
RBBP4	THRAP3	JAK1	RPS27	NTRK1	TKT	VAMP4	FAM129A	CNTN2	PARP1
C1orf35	MRPL55	KCNK1	C2orf18	YPEL5	DPY30	UBC	FAM136A	DCTN1	ZFAND2B
TRIP12	MAP4	CLDND1	C3orf15	COPG1	CCNL1	ARAP2	H2AFZ	RP11	MYO10
ATG12	HINT1	PCDHB6	LARP1	FAM193B	DEK	LEMD2	DEFA6	PNISR	C7orf23
HIPK2	PAF1	TTC35	LY6D	CD72	FAM206A	UBAC1	COMMD3	ZEB1-AS1
ARHGAP12	CHST3	C10orf118	NSMCE4A	CCDC88B	ANKRD13D	PICALM
CEP57	KBTBD3	TBRG1	GOLGA2B	NFYB	ARL6IP4	MTRF1	TPT1-AS1	RCN1P2
ARGLU1	DCUN1D2	KLHDC2	U6	NEK9	WARS	ANKRD9	LPCAT4	AKAP13	CHD2	CTD
RHBDF1	FOPNL	LONP2	C16orf48	HSDL1	SPG7	TP53	RPL26	GSDMB	CLTC
IER3IP1	MALT1	FAM69C	FGF22	ALKBH7	CCDC114	CYTH2	ZNF772	KIF3B	BCL2L13	RP1
MPST	TRABD	AKAP17A	MAGED1	MSN
GOBPID	Pvalue	OddsRatio	ExpCount	Count	Size	Term
Tissue: Skin_Sun_Exposed_Lower_leg=>Muscle_Skeletal	Type:	asymmetric
SourceGene:	FAM207BP(ENSG00000228797.2)
TargetGeneSet:	AHDC1	TMEM56	ATP5F1	B3GALNT2	C2orf28	COX5B	GULP1	ICOS
THUMPD3	GRPEL1	PPARGC1A	ATP8A1	TIFA	RP11	C5orf63	SH3RF2	MRPS18B
KCNK17	FAXC	STXBP5	NDUFA4	DNAH11	TBRG4	GTF2I	AGBL3	MRPS33	MRPL13
ISCA1	AS3MT	IMMP1L	CYBASC3	IFT46	C14orf142	RPL9P25	TSPAN3	MRPL46
UQCRC2	ZNF821	CHAD	ATP5H	ICAM3	ZNF627	MAST3	COX6B1	MRPS12	GPCPD1	CHD6
C21orf33	DNMT3L	C22orf25	NDUFA6	BTK
GOBPID	Pvalue	OddsRatio	ExpCount	Count	Size	Term
GO:0022900	4.94758116199299e-12	34.9071358748778	0.398726731714068
10	134	electron transport chain
GO:0045333	2.80930273241364e-10	28.4111012826183	0.422531312711923
9	142	cellular respiration
GO:0022904	3.37738933946109e-10	37.1948051948052	0.285654971974258
8	96	respiratory electron transport chain
GO:0006091	3.31242845336531e-08	11.5333483213429	1.27354508338523
11	428	generation of precursor metabolites and energy
GO:0015980	2.34362402309278e-07	12.5335570469799	0.913500795792679
9	307	energy derivation by oxidation of organic compounds
Tissue: Muscle_Skeletal=>Skin_Sun_Exposed_Lower_leg	Type:	cluster
SourceGene:	PLEKHA4(ENSG00000105559.5)
TargetGeneSet:	NADK	DNAJC11	PGD	MTHFR	DDI2	SYF2	CSMD2	SZT2
BCAR3	TSPAN2	FOXE1	SHC1	IER5	CD55	PPP2R5A	CICP13	ADCY3	OTOF
SLC5A6	CAD	CRIM1	MTHFD2	HOXD9	MREG	SGPP2	TRIP12	MYRIP	ZNF621
NICN1	PARP9	ATR	RPL22L1	LETM1	TMEM165	RP11	CYP2U1	SLC25A4	MCCC2
CETN3	SMAD5-AS1	ZNF225	PPP2R2B	HMGXB3	LARP1	ID4	E2F3	SKIV2L	XPO5
SLC29A1	MRAP2	MDN1	AIM1	CCDC28A	MTHFD1L	FBXL18	MEOX2	CLIP2	GNG11
TRRAP	AKR1B10	GIMAP7	CHD7	POP1	RAD21	DPAGT1	IARS	KIAA0368
NUP188	FAM208B	OTUD1	CICP9	SIRT1	SLC25A16	PCBD1	RP13	C10orf116
AGAP11	EML3	C11orf68	AIP	NEU3	DIXDC1	KCNJ1	CD9	CLEC7A
HOXC10	DNAJC14	XPOT	ATP6V0A2	EP400	CRYL1	RNF31	AKAP5	EVL
TRAF3	CTD	PLA2G4E	WDR73	TSC2	USP7	IRX5	SLC7A6	CIRH1A	ADAT1
VPS53	TSR1	RP1	CAMTA2	CDK5R1	GAS2L2	ACACA	PSME3	EFTUD2	PPM1D	TLK2
C17orf28	SGSH	FN3K	BCL2	ILF3	GCDH	TRMT1	DPY19L3	MLL4

C19orf55 MEGF8 SIGLEC10 NLRP12 ZNF587 MAVS PAX1 APCDD1L GART
PEX26 FAM156B TCEAL7
GOBPID Pvalue OddsRatio ExpCount Count Size Term
GO:0006732 6.17803174840816e-07 7.82166918049271 1.61082278043042
11 206 coenzyme metabolic process
GO:0051186 3.37181934434477e-06 6.50008379420144 1.91578437478375
11 245 cofactor metabolic process
Tissue: Muscle_Skeletal=>Skin_Sun_Exposed_Lower_leg Type: asymmetric
SourceGene: PROCR(ENSG00000101000.3)
TargetGeneSet: SKI ICMT ZBTB48 PGD PEX14 FBLIM1 RP11 SEPN1 RP1
EIF2C1 INSL3 CTPS MUTYH RP4 ALX3 CTTNBP2NL MAN1A2 CDC42SE1
CHTOP SNORA58 SHC1 ASH1L ARHGEF11 UCK2 MPZL1 ABL2 ATP2B4
TP53BP2 GALNT2 ATAD2B ADCY3 ASXL2 GPR113 SLC5A6 CAD YPEL5 ASB3
AFTPH TEX261 UGGT1 TMEM163 ACVR2A FMNL2 MTX2 CWC22 PIKFYVE RNF25
SGPP2 COL4A4 HDAC4 ARL8B TBC1D5 HIGD1A CCDC12 PRKAR2A ZNF654 CLDND1
KIAA2018 NCK1 PIK3CB UBQLN4P1 GPR87 BCL6 HES1 LETM1
ZBTB49 MRFAP1L1 TBC1D14 PCDH7 TEC CHIC2 GSX2 CLOCK RASGEF1B
HELQ SGMS2 ANAPC10 LSM6 UBE2B ANKHD1 FBXO38 NDST1 LARP1 MAT2B
SLC2A3P1 NEURL1B CTB DUSP1 ARL10 NSD1 CANX ZKSCAN4 UHRF1BP1
DEFA6 FBXO9 PTP4A1 PHF3 MDN1 MCM9 CCDC28A PPIL4 FOXK1 ARL4A
CCT6P3 ZNF394 FAM200A C7orf59 EPHB4 PRKRIP1 DNAJB9 IMPDH1 KLHDC10 HIPK2 RHEB
GS1 KIAA1456 KCNU1 PRKDC ATP6V1H TMEM68 PTDSS1 POP1 PABPC1
TTC35 ST3GAL1 CTA EPPK1 RGP1 FAM120A GRIN3A SNX33 MIR181A2HG
NUP188 TOR1B WDR5 NACC2 KLF6 OTUD1 RAB18 C10orf25 ZFAND4
P4HA1 C10orf28 TAF5 DUSP5 PPP2R2D HRAS ZBED5 TUT1 CTSC
AMOTL1 C11orf63 SIAE SRPR ERC1 TULP3 CDKN1B ITPR2 FKBP11
TUBA1C TMBIM6 ACVR1B SARNP DDIT3 SLC25A3 EID3 C12orf76 UNC119B
RSRC2 RPL21 AKAP11 MYCBP2 IPO5 CHAMP1 RNF31 TSSK6 BNIP3P1 ARF6
CHMP4BP1 MED6 ANGEL1 ALKBH1 SPATA7 BTBD7 WARS TRAF3 CDC42BPB
HERC2 C15orf24 RNF111 CLK3 CTD UBN1 FAM86A NOMO2 SMG1 COG7
NFATC2IP KCTD13 PRSS8 ARMC5 CNOT1 CMTM4 CBFB PSKH1 CDH1
GABARAPL2 TERF2IP MAP1LC3B ZNF778 GLOD4 C17orf48 TOM1L2
MYO15A USP22 NF1 LRRC37B RHOT1 NLE1 ACACA SOCS7 NR1D1 STAT3
PSME3 DUSP3 SPOP LRRC59 TOB1 MPO PPM1D TLK2 H3F3B EXOC7
JMJD6 SLC26A11 NPLOC4 TGIF1 TUBB6 DSG3 PMAIP1 TMX3 LMNB2 EEF2
XAB2 ZNF426 PIN1 ZNF627 ZNF563 DCAF15 AKAP8L ZFP36 DEDD2 MEGF8
PHLDB3 ZNF225 PPP1R37 PRKD2 ZNF616 ZNF766 CNOT3 ZNF587 CENPB PROCR
DNTTIP1 VAPB C20orf151 C20orf20 TMEM50B GART ATP6V1E1 NF2
SREBF2 CDKL5 SMC1A HUWE1 MSN PHKA1 TTC3P1 EIF4A1P10 PSMD10
GOBPID Pvalue OddsRatio ExpCount Count Size Term
Tissue: Muscle_Skeletal=>Skin_Sun_Exposed_Lower_leg Type: cluster
SourceGene: PROCR(ENSG00000101000.3)
TargetGeneSet: SKI ICMT ZBTB48 PGD PEX14 FBLIM1 RP11 SEPN1 RP1
EIF2C1 INSL3 CTPS MUTYH RP4 ALX3 CTTNBP2NL MAN1A2 CDC42SE1
CHTOP SNORA58 SHC1 ASH1L ARHGEF11 UCK2 MPZL1 ABL2 ATP2B4
TP53BP2 GALNT2 ATAD2B ADCY3 ASXL2 GPR113 SLC5A6 CAD YPEL5 ASB3
AFTPH TEX261 UGGT1 TMEM163 ACVR2A FMNL2 MTX2 CWC22 PIKFYVE RNF25
SGPP2 COL4A4 HDAC4 ARL8B TBC1D5 HIGD1A CCDC12 PRKAR2A ZNF654 CLDND1
KIAA2018 NCK1 PIK3CB UBQLN4P1 GPR87 BCL6 HES1 LETM1
ZBTB49 MRFAP1L1 TBC1D14 PCDH7 TEC CHIC2 GSX2 CLOCK RASGEF1B
HELQ SGMS2 ANAPC10 LSM6 UBE2B ANKHD1 FBXO38 NDST1 LARP1 MAT2B
SLC2A3P1 NEURL1B CTB DUSP1 ARL10 NSD1 CANX ZKSCAN4 UHRF1BP1
DEFA6 FBXO9 PTP4A1 PHF3 MDN1 MCM9 CCDC28A PPIL4 FOXK1 ARL4A
CCT6P3 ZNF394 FAM200A C7orf59 EPHB4 PRKRIP1 DNAJB9 IMPDH1 KLHDC10 HIPK2 RHEB
GS1 KIAA1456 KCNU1 PRKDC ATP6V1H TMEM68 PTDSS1 POP1 PABPC1
TTC35 ST3GAL1 CTA EPPK1 RGP1 FAM120A GRIN3A SNX33 MIR181A2HG
NUP188 TOR1B WDR5 NACC2 KLF6 OTUD1 RAB18 C10orf25 ZFAND4
P4HA1 C10orf28 TAF5 DUSP5 PPP2R2D HRAS ZBED5 TUT1 CTSC
AMOTL1 C11orf63 SIAE SRPR ERC1 TULP3 CDKN1B ITPR2 FKBP11
TUBA1C TMBIM6 ACVR1B SARNP DDIT3 SLC25A3 EID3 C12orf76 UNC119B
RSRC2 RPL21 AKAP11 MYCBP2 IPO5 CHAMP1 RNF31 TSSK6 BNIP3P1 ARF6
CHMP4BP1 MED6 ANGEL1 ALKBH1 SPATA7 BTBD7 WARS TRAF3 CDC42BPB
HERC2 C15orf24 RNF111 CLK3 CTD UBN1 FAM86A NOMO2 SMG1 COG7

NFATC2IP KCTD13 PRSS8 ARMC5 CNOT1 CMTM4 CBFB PSKH1 CDH1
GABARAPL2 TERF2IP MAP1LC3B ZNF778 GLOD4 C17orf48 TOM1L2
MYO15A USP22 NF1 LRRC37B RHOT1 NLE1 ACACA SOCS7 NR1D1 STAT3
PSME3 DUSP3 SPOP LRRC59 TOB1 MPO PPM1D TLK2 H3F3B EXOC7
JMJD6 SLC26A11 NPLOC4 TGIF1 TUBB6 DSG3 PMAIP1 TMX3 LMNB2 EEF2
XAB2 ZNF426 PIN1 ZNF627 ZNF563 DCAF15 AKAP8L ZFP36 DEDD2 MEGF8
PHLDB3 ZNF225 PPP1R37 PRKD2 ZNF616 ZNF766 CNOT3 ZNF587 CENPB PROCR
DNTTIP1 VAPB C20orf151 C20orf20 TMEM50B GART ATP6V1E1 NF2
SREBF2 CDKL5 SMC1A HUWE1 MSN PHKA1 TTC3P1 EIF4A1P10 PSMD10
GOBPID Pvalue OddsRatio ExpCount Count Size Term
Tissue: Muscle_Skeletal=>Skin_Sun_Exposed_Lower_leg Type: asymmetric
SourceGene: RBPJL(ENSG00000124232.6)
TargetGeneSet: CELA2A CELA3B CELA3A REG1B REG1A HIST1H2BL CLPS CGA
CPA2 CPA1 PRSS1 PRSS3P1 PNLIP PNLIPRP1 PNLIPRP2 RPL29P25
PLA2G1B GP2 MT1H CTRB2 CTRB1
GOBPID Pvalue OddsRatio ExpCount Count Size Term
GO:0007586 5.69496585169008e-13 103.193519351935 0.14331188153069
8 109 digestion
GO:0044241 2.88633145415972e-09 349.6 0.0197218185592693 4 15
lipid digestion
GO:0022617 1.33863255514118e-05 81.8125 0.0473323645422462 3 36
extracellular matrix disassembly
Tissue: Muscle_Skeletal=>Skin_Sun_Exposed_Lower_leg Type: cluster
SourceGene: RP11-439E19.1(ENSG00000225300.1)
TargetGeneSet: RP11 FOXE1 C4BPAP2 REL FAM126B CFLAR GPBAR1 POC1A
ITGB5-AS1 STAG1 ECT2 CRYGS RAC1P2 LNX1-AS1 CCNA2 FAM160A1
KIFC1 PRKAR1B ANLN MIR671 XKR6 EXOSC3 TCEB2P4 AGAP7 AFAP1L2 GVINP1
INCENP THSD1P1 MYCBP2-AS2 BUB1B UBE2Q2 C15orf42 PRC1 NPIP
HEXIM2 FAM108A5P GSTT2 FGD1 KIF4A MED12
GOBPID Pvalue OddsRatio ExpCount Count Size Term
GO:0051301 4.07559802103047e-07 15.7466216216216 0.750674693792817
8 452 cell division
GO:0000910 1.61460133934954e-05 31.86 0.156113763753373 4 94
cytokinesis
GO:0000280 1.79929748081699e-05 13.6463178294574 0.581274652273199
6 350 nuclear division
GO:0007067 1.79929748081699e-05 13.6463178294574 0.581274652273199
6 350 mitosis
Tissue: Skin_Sun_Exposed_Lower_leg=>Muscle_Skeletal Type: asymmetric
SourceGene: RPL32P29(ENSG00000244270.1)
TargetGeneSet: SRM PKP1 QARS RP11 WDR43 POLR1A SS18L2 LETM1 GUF1
SCFD2 SNRNP48 RP1 AIMP2 TRGV4 GLIS3 TRUB2 PDCD11 DUSP5 CCDC59
ANAPC5 WARS SAT2 ELAC2 FARSA PSMF1 ATRN RAB22A RRP1
GOBPID Pvalue OddsRatio ExpCount Count Size Term
GO:0034660 1.67741032307719e-08 24.0373752389042 0.493045463981731
8 285 ncRNA metabolic process
GO:0006399 5.74670433869465e-08 37.9663865546218 0.216248010518303
6 125 tRNA metabolic process
GO:0006418 1.2875089575768e-06 62.2597402597403 0.0830392360390284
4 48 tRNA aminoacylation for protein translation
GO:0043038 1.64778958528933e-06 58.273556231003 0.0882291882914677 4
51 amino acid activation
GO:0043039 1.64778958528933e-06 58.273556231003 0.0882291882914677 4
51 tRNA aminoacylation
Tissue: Muscle_Skeletal=>Skin_Sun_Exposed_Lower_leg Type: cluster
SourceGene: SEPT10P1(ENSG00000253541.1)
TargetGeneSet: RPS8 IPP HBXIP BRP44 ACP1 ERLEC1 REL SENP2
CXCR2 THOC7 PCNP RP11 PARL RPS3A ZDHHC11 VTA1 BRP44L RPS3AP26
ZNF277 CTD DCTN6 PAF1 RPS6 RP13 ATP6V1G1 CUL2 ANAPC16
FAM204A MUC5AC TMPRSS5 HELB ARL1 GPN3 GTF3A MEG8 RPS3AP6 RPL26
RPL17 ARX VBP1
GOBPID Pvalue OddsRatio ExpCount Count Size Term
GO:0022415 8.04580785927588e-07 17.0434782608696 0.539755034253685

7	260	viral reproductive process
GO:0006415	1.11526350705577e-06	33.3372093023256	0.18891426198879
5	91	translational termination
GO:0006414	2.26931234975104e-06	28.642	0.21797799460245	5	105
translational elongation
GO:0006614	2.26931234975104e-06	28.642	0.21797799460245	5	105
SRP-dependent cotranslational protein targeting to membrane
GO:0006613	2.37821394029656e-06	28.3564356435644	0.220053975503425
5	106	cotranslational protein targeting to membrane
GO:0045047	2.37821394029656e-06	28.3564356435644	0.220053975503425
5	106	protein targeting to ER
GO:0072599	2.37821394029656e-06	28.3564356435644	0.220053975503425
5	106	establishment of protein localization to endoplasmic reticulum
GO:0072594	3.29101373818799e-06	17.9583333333333	0.423500103799045
6	204	establishment of protein localization to organelle
GO:0070972	4.03532583692599e-06	25.3238938053097	0.244965746315134
5	118	protein localization to endoplasmic reticulum
GO:0000184	4.20622731218036e-06	25.1	0.24704172721611	5	119
nuclear-transcribed mRNA catabolic process, nonsense-mediated decay
GO:0019058	6.73288848653153e-06	15.7733333333333	0.479551588125389
6	231	viral infectious cycle
GO:0019080	1.34541121326142e-05	19.5547945205479	0.313473116047332
5	151	viral genome expression
GO:0019083	1.34541121326142e-05	19.5547945205479	0.313473116047332
5	151	viral transcription
GO:0006413	1.38916231216778e-05	19.4204081632653	0.315549096948308
5	152	translational initiation
GO:0006612	1.43401731253634e-05	19.2878378378378	0.317625077849284
5	153	protein targeting to membrane
GO:0043624	1.43401731253634e-05	19.2878378378378	0.317625077849284
5	153	cellular protein complex disassembly
GO:0043241	1.67554828197996e-05	18.6509803921569	0.328004982354162
5	158	protein complex disassembly
Tissue: Skin_Sun_Exposed_Lower_leg=>Muscle_Skeletal	Type:	asymmetric
SourceGene:	SHROOM1(ENSG00000164403.9)
TargetGeneSet:	C1orf144	CATSPER4	SFN	MACF1	C1orf50	KIAA0494
S100A4	TNFSF13B	RP11	CENPO	DYSF	GPR155	ILKAP	HSPBAP1	TMEM108
SCARNA7	WDR1	MAP1B	TXNDC15	CTNNA1	PCDHB14	NDST1	HIST1H2AG	CYP51A1	BPGM
NUP205	EXTL3	SFRP1	TTC35	MYOF	BNIP3	7SK	SLC43A3	TTC9C	MYEOV
SLC6A13	CDKN1B	BNIP3P1	HSPA2	AHNAK2	YPEL3	CDH11	NRN1L	PFAS	KRT16
TUBB6	SMARCA4	ZNF211	JPH2	COL18A1	NF2	RP3	RNF128	BGN
GOBPID	Pvalue	OddsRatio	ExpCount	Count	Size	Term
Tissue: Whole_Blood=>Muscle_Skeletal	Type:	asymmetric
SourceGene:	BCAN(ENSG00000132692.12)
TargetGeneSet:	GABRD	RP3	ERMN	SCN2A	MOBP	CAMKV	CTB	CPLX2
RPL7L1P3	TMEM130	RP11	VAT1	NEFL	DIRAS2	GRIN1	VPS26A	INA	BUB3
CEND1	SLC1A2	VAT1L	RUNDC3A	GFAP	SYT4	LILRB3	SNAP25	SLC12A5	MYO18B
SEPT3	MLC1
GOBPID	Pvalue	OddsRatio	ExpCount	Count	Size	Term
GO:0006836	1.30506678399032e-06	33.0401662049861	0.197633381772888
5	119	neurotransmitter transport
GO:0001505	1.5371969226498e-06	31.9112399643176	0.20427652065601
5	123	regulation of neurotransmitter levels
GO:0001504	6.97436335720473e-06	102.907142857143	0.0381980485779531
3	23	neurotransmitter uptake
GO:0045104	1.02056064378552e-05	89.4658385093168	0.0431804027402948
3	26	intermediate filament cytoskeleton organization
GO:0045103	1.14687963909998e-05	85.7321428571429	0.0448411874610754
3	27	intermediate filament-based process
Tissue: Whole_Blood=>Muscle_Skeletal	Type:	asymmetric
SourceGene:	C6orf48(ENSG00000204387.7)
TargetGeneSet:	C1orf159	HECTD3	CYP4A22	RP11	INADL	DPYD	ATP5F1
HIST2H2BE	MTX1	FAM189B	ACBD6	TSNAX	CEP170	TRMT61B	C1D	TXNDC9

NIF3L1 NDUFB3 ERBB4 ABCB6 PRRT3-AS1 SATB1 NR1D2 OXSM GRM2
FAM208A UBXN7-AS1 MAN2A1 ARHGEF37 MRPL22 ARL10 HIST1H1E
MRPS18B TAF11 TEAD3 C6orf226 RRP36 DST C6orf162 RTN4IP1
KPNA5 HECA AIMP2 NDUFA4 PMPCB AGAP3 DEFB1 PCM1 GDAP1 ZNF704
SMARCA2 MPDZ FANCG TESK1 STXBP1 FNBP1 ARHGAP21 SGMS1 SUPV3L1
RNF141 TEAD1 MPPED2 PEX16 PHF21A PACS1 RBM4B TEX12 APOA4 CBL RARG
AVIL TMEM19 MRPS31 MED4-AS1 TPP2 PLEK2 WDR25 LPCAT4 C15orf44
ETFA MRPL46 MRPL28 PKD1 NOMO2 MAZ NOD2 CIAPIN1 RP1 ZBTB4
COX10 GIT1 HSD17B1P1 PYCR1 NDUFV2 PQLC1 ABCA7 UQCR11 GIPC3
PGPEP1 RELB PPP2R1A LINC00493 CDK5RAP1 RP3 NFATC2 ATP9A
FAM209B ITSN1 CTA TMEM184B CBX6 SMARCA1
GOBPID Pvalue OddsRatio ExpCount Count Size Term
GO:0022904 1.73404809983961e-06 14.0344101123596 0.577953082831638
7 96 respiratory electron transport chain
GO:0022900 1.58829521297848e-05 9.80895669291339 0.806726178119161
7 134 electron transport chain
Tissue: Whole_Blood=>Muscle_Skeletal Type: cluster
SourceGene: C6orf48(ENSG00000204387.7)
TargetGeneSet: C1orf159 HECTD3 CYP4A22 RP11 INADL DPYD ATP5F1
HIST2H2BE MTX1 FAM189B ACBD6 TSNAX CEP170 TRMT61B C1D TXNDC9
NIF3L1 NDUFB3 ERBB4 ABCB6 PRRT3-AS1 SATB1 NR1D2 OXSM GRM2
FAM208A UBXN7-AS1 MAN2A1 ARHGEF37 MRPL22 ARL10 HIST1H1E
MRPS18B TAF11 TEAD3 C6orf226 RRP36 DST C6orf162 RTN4IP1
KPNA5 HECA AIMP2 NDUFA4 PMPCB AGAP3 DEFB1 PCM1 GDAP1 ZNF704
SMARCA2 MPDZ FANCG TESK1 STXBP1 FNBP1 ARHGAP21 SGMS1 SUPV3L1
RNF141 TEAD1 MPPED2 PEX16 PHF21A PACS1 RBM4B TEX12 APOA4 CBL RARG
AVIL TMEM19 MRPS31 MED4-AS1 TPP2 PLEK2 WDR25 LPCAT4 C15orf44
ETFA MRPL46 MRPL28 PKD1 NOMO2 MAZ NOD2 CIAPIN1 RP1 ZBTB4
COX10 GIT1 HSD17B1P1 PYCR1 NDUFV2 PQLC1 ABCA7 UQCR11 GIPC3
PGPEP1 RELB PPP2R1A LINC00493 CDK5RAP1 RP3 NFATC2 ATP9A
FAM209B ITSN1 CTA TMEM184B CBX6 SMARCA1
GOBPID Pvalue OddsRatio ExpCount Count Size Term
GO:0022904 1.73404809983961e-06 14.0344101123596 0.577953082831638
7 96 respiratory electron transport chain
GO:0022900 1.58829521297848e-05 9.80895669291339 0.806726178119161
7 134 electron transport chain
Tissue: Whole_Blood=>Muscle_Skeletal Type: asymmetric
SourceGene: LINC00422(ENSG00000224429.1)
TargetGeneSet: KLHL21 CAMTA1 RP11 UBE2T TMEM183A JMJD4 RAB4A
ROCK2 EML6 MRPL30 CALCRL TMEM42 SACM1L PABPC1P10 TMED11P C4orf26
SKIV2L2 PTCD2 RAD50 GEMIN5 FARS2 C6orf62 BYSL C6orf108 SNX14
SPDYE2 BPGM RPL23AP53 TNFRSF10B USP20 EXOSC2 DDB1 OVOL1
LOH12CR1 METTL7A B4GALNT1 CCL14 MAP1LC3B2 RCBTB2 NUBPL
SOCS6 ZBTB1 NEK9 DYNC1H1 VPS13C MPI DNAJA3 SBK1 LONP2 DYNC1LI2
HPR ITGAE TP53 TOM1L2 NLE1 MAPT PNPO PHOSPHO1 BCAS3
METTL4 SF3A2 HMG20B BCL2L12 LINC00085 PSMF1 BID EFCAB6 PNPLA4
UTP14A LINC00087 BRCC3
GOBPID Pvalue OddsRatio ExpCount Count Size Term
GO:0022613 1.71308029564519e-06 11.3644560208375 0.840772264895163
8 225 ribonucleoprotein complex biogenesis
GO:0071843 2.68784995566008e-06 10.6651609260305 0.893086983599751
8 239 cellular component biogenesis at cellular level
Tissue: Muscle_Skeletal=>Whole_Blood Type: asymmetric
SourceGene: MTFR1(ENSG00000066855.10)
TargetGeneSet: RP11 PCSK9 CD101 POLR3GL CD1A FAM110C PCYOX1 WDR54
IL18R1 ATRIP MAPKAPK3 DUSP7 B4GALT4 PRLR ARG1 LFNG GATS IRF5
LGI3 DRP2 GPR26 C9orf106 ARID5B MYOF ACP2 ARRB1 C11orf75
SLC37A2 SMARCD1 HVCN1 IRG1 FLVCR2 ZG16B CBFA2T3 RPL7AP64 ASGR1
KCTD15 IL4I1 LILRB1 LILRB4 ADRBK2 PIK3IP1 CYBB
GOBPID Pvalue OddsRatio ExpCount Count Size Term
GO:0031623 9.38615227242075e-06 35.9125 0.134523562383226 4 54
receptor internalization
GO:0006898 1.41258191587134e-05 19.0535857104772 0.313888312227527

5	126	receptor-mediated endocytosis
Tissue: Whole_Blood=>Muscle_Skeletal	Type:	asymmetric
SourceGene:	NCR1(ENSG00000189430.7)
TargetGeneSet:	FAM41C	PLOD1	RP3	FOXO6	RP11	UBE2T	SOCS5	EPHA4
GAL3ST2	LAMB2	DCAF16	MAP1B	ZNF277	GPR85	C8orf12	SPAG6	CSDA	SOCS6
RABEP2	HPR	ARHGAP23		GNA13	HMG20B	RGS19	ABCG1
GOBPID	Pvalue	OddsRatio	ExpCount	Count	Size	Term
GO:0031103	9.78813257602548e-06	90.9726315789474	0.0426268078333679
3	28	axon regeneration
GO:0031102	1.62213937516522e-05	75.7842105263158	0.0502387378036122
3	33	neuron projection regeneration
Tissue: Whole_Blood=>Muscle_Skeletal	Type:	asymmetric
SourceGene:	NEFM(ENSG00000104722.8)
TargetGeneSet:	VPS13D	CACNA1A	KCNJ9	VSNL1	CTNNA2	MOBP	CAMKV	SYNPR
TAGLN3	SPP1	LSM6	GRIA2	CPLX2	CTA	SFT2D1	TMEM130	RP11	NEFL
STMN4	DIRAS2	GRIN1	VPS26A	OPALIN	PSD	INA	GPR123	SLC1A2	PRMT8	SCG5
CKMT1B	FBXL16	GFAP	SYT4	SLC8A2	BCAT2	SNAP25	POLR3F	SLC12A5	MLC1
GOBPID	Pvalue	OddsRatio	ExpCount	Count	Size	Term
GO:0006836	5.86618827844923e-06	23.2374918778428	0.263511175697184
5	119	neurotransmitter transport
GO:0001505	6.89686640537397e-06	22.4435028248588	0.272368694208013
5	123	regulation of neurotransmitter levels
GO:0048791	1.42319897105304e-05	961.2	0.00664313888312228	2	3
calcium ion-dependent exocytosis of neurotransmitter
Tissue: Whole_Blood=>Muscle_Skeletal	Type:	asymmetric
SourceGene:	OLAH(ENSG00000152463.10)
TargetGeneSet:	CORT	PADI2	WNT4	AKR1A1	GADD45A	TMEM206	JMJD4	EBF3	XDH
SOCS5	UGP2	ELMOD3	OSBPL6	ICA1L	ERBB4	EPHA4	EFHD1	ARL8B	ZDHHC3
TEX264	ANKDD1B	CTB	SNX14	AGPAT5	VPS13B	ST3GAL1	SH3GLB2	VPS26A	MICU1
PANK1	SORBS1	SLC18A2	DDB1	IL18	RPUSD4	COPS7A	METTL7A	ABCC4	RP11	SBK1
COX6A2	ABR	TP53	TP53I13	NLE1	MAPT	PNPO	EXOC7	METTL4	SMAD7
CRLF1	RELB	NNAT	RP1	MORC4	MTMR1
GOBPID	Pvalue	OddsRatio	ExpCount	Count	Size	Term
GO:2000379	1.77754256999733e-06	56.8379446640316	0.0896823749221507
4	27	positive regulation of reactive oxygen species metabolic process
Tissue: Muscle_Skeletal=>Whole_Blood	Type:	asymmetric
SourceGene:	PGM5(ENSG00000154330.6)
TargetGeneSet:	RP11	IGKJ5	IGKC	IGKJ1	IGKV4-1	IGKV1-9	IGKV3-20
IGKV2-24		IGKV3D-20		ENOPH1	TRRAP	GML	GLDC	RIN2	IGHG2
IGHV2-5	MRPL38	IGLV5-45		IGLV2-23		IGLV3-16		IGLL5	IGLC3
GOBPID	Pvalue	OddsRatio	ExpCount	Count	Size	Term
GO:0006958	1.14392769649748e-12	327.159090909091	0.0415196180195142
6	50	complement activation, classical pathway
GO:0006956	4.8685606027542e-12	252.315789473684	0.0523147187045879
6	63	complement activation
GO:0002455	5.91430420577553e-12	243.728813559322	0.0539755034253685
6	65	humoral immune response mediated by circulating immunoglobulin
GO:0072376	2.49206776864283e-11	188.986842105263	0.0680921735520033
6	82	protein activation cascade
GO:0016064	1.59480560469431e-10	136.514285714286	0.0921735520033216
6	111	immunoglobulin mediated immune response
GO:0019724	1.87641587000686e-10	132.694444444444	0.0946647290844924
6	114	B cell mediated immunity
GO:0006959	2.8358439605752e-10	123.474137931034	0.101307867967615
6	122	humoral immune response
GO:0002449	1.89926442395287e-09	88.6832298136646	0.138675524185178
6	167	lymphocyte mediated immunity
GO:0002460	3.08500589580792e-09	81.5085714285714	0.150301017230641
6	181	adaptive immune response based on somatic recombination of immune receptors built from immunoglobulin superfamily domains
GO:0002250	5.29263894340719e-09	74.203125	0.164417687357276	6
198	adaptive immune response
GO:0002443	7.75190211419840e-09	69.4341463414634	0.17521278804235

6 211 leukocyte mediated immunity
GO:0002253 6.21116986012747e-08 48.2798634812287 0.248287315756695
6 299 activation of immune response
GO:0050778 1.99278675894729e-07 39.3324022346369 0.302262819182064
6 364 positive regulation of immune response
GO:0002252 2.81797064242895e-07 36.9973684210526 0.32053145111065
6 386 immune effector process
Tissue: Whole_Blood=>Muscle_Skeletal Type: asymmetric
SourceGene: PI3(ENSG00000124102.4)
TargetGeneSet: RP11 RCC2 UBR4 WNT4 LYPLA2 MYOM3 RP3 CSMD2
MACF1 IPO13 TMEM53 AKR1A1 CYP4A22 PDZK1IP1-AS1 MIR761 WDR63 MTX1
HAPLN2 HDGF SLC26A9 TSNAX SIPA1L2 C2orf43 XDH UGP2 CEP68 MYO7B GPD2
OSBPL6 COL4A4 COL4A3 PDE6D ARL8B PRRT3 PRRT3-AS1 NR1D2 OXSM RP13
FAM212A BZW1P2 WHSC1 EGF ANAPC10 PDGFC CKMT2 ANKRD32 SLC12A2 ARHGEF37
MRPL22 TUBB JARID2 HIST1H1E FKBP5 TMEM217 CUL7 MUT BACH2 GJA1
RNF217 PLAGL1 ELN LIMK1 MCM7 AGPAT5 FDFT1 IDO1 ZNF704 IMPA1
ST3GAL1 ZFAT-AS1 OPLAH TESK1 AQP7P2 SEMA4D HSDL2 ASTN2 TRIM32
USP6NL SGMS1 RHOBTB1 TSPAN15 PANK1 TCTN3 GALNTL4 TEAD1 PEX16 PACS1
RPUSD4 CD9 GRASP SUOX TMCC3 GPR133 MRPS31 ABCC4 TEP1 C14orf135
FLVCR2 FOXN3 TTC7B C14orf80 LPCAT4 FAM174B NOMO1 NOMO3 NOMO2 MAZ
PRRT2 COX6A2 LDHD RNF166 CTD RP1 UBE2G1 USP6 SLC2A4 SHBG TP53
ALDH3A1 KSR1 TCAP KRT13 ABCA8 KCTD2 SYNGR2 ARHGAP28 PMAIP1 BCL2
ZNF516 ABCA7 KLF16 FAM108A1 TIMM44 CYP4F22 CRLF1 RELB BBC3
SPHK2 TGM3 VSX1 NNAT B4GALT5 MIR133A2 TIMP3 KAL1 EGFL6
MTMR1
GOBPID Pvalue OddsRatio ExpCount Count Size Term
GO:0046902 1.61334629839148e-06 66.3472222222222 0.0930039443637119
4 12 regulation of mitochondrial membrane permeability
Tissue: Whole_Blood=>Muscle_Skeletal Type: cluster
SourceGene: PI3(ENSG00000124102.4)
TargetGeneSet: RP11 RCC2 UBR4 WNT4 LYPLA2 MYOM3 RP3 CSMD2
MACF1 IPO13 TMEM53 AKR1A1 CYP4A22 PDZK1IP1-AS1 MIR761 WDR63 MTX1
HAPLN2 HDGF SLC26A9 TSNAX SIPA1L2 C2orf43 XDH UGP2 CEP68 MYO7B GPD2
OSBPL6 COL4A4 COL4A3 PDE6D ARL8B PRRT3 PRRT3-AS1 NR1D2 OXSM RP13
FAM212A BZW1P2 WHSC1 EGF ANAPC10 PDGFC CKMT2 ANKRD32 SLC12A2 ARHGEF37
MRPL22 TUBB JARID2 HIST1H1E FKBP5 TMEM217 CUL7 MUT BACH2 GJA1
RNF217 PLAGL1 ELN LIMK1 MCM7 AGPAT5 FDFT1 IDO1 ZNF704 IMPA1
ST3GAL1 ZFAT-AS1 OPLAH TESK1 AQP7P2 SEMA4D HSDL2 ASTN2 TRIM32
USP6NL SGMS1 RHOBTB1 TSPAN15 PANK1 TCTN3 GALNTL4 TEAD1 PEX16 PACS1
RPUSD4 CD9 GRASP SUOX TMCC3 GPR133 MRPS31 ABCC4 TEP1 C14orf135
FLVCR2 FOXN3 TTC7B C14orf80 LPCAT4 FAM174B NOMO1 NOMO3 NOMO2 MAZ
PRRT2 COX6A2 LDHD RNF166 CTD RP1 UBE2G1 USP6 SLC2A4 SHBG TP53
ALDH3A1 KSR1 TCAP KRT13 ABCA8 KCTD2 SYNGR2 ARHGAP28 PMAIP1 BCL2
ZNF516 ABCA7 KLF16 FAM108A1 TIMM44 CYP4F22 CRLF1 RELB BBC3
SPHK2 TGM3 VSX1 NNAT B4GALT5 MIR133A2 TIMP3 KAL1 EGFL6
MTMR1
GOBPID Pvalue OddsRatio ExpCount Count Size Term
GO:0046902 1.61334629839148e-06 66.3472222222222 0.0930039443637119
4 12 regulation of mitochondrial membrane permeability
Tissue: Whole_Blood=>Muscle_Skeletal Type: asymmetric
SourceGene: PTN(ENSG00000105894.7)
TargetGeneSet: CACNA1A RP4 ERMN SLC16A14 SOWAHA CPLX2 TMEM130 RP11
NEFL GRIN1 INA SLC1A2 SYT1 KLHDC1 RPL5P3 RUNDC3A ZNF615 TTYH1
GOBPID Pvalue OddsRatio ExpCount Count Size Term
GO:0060052 1.56486111333871e-05 524.836363636364 0.00629714206629299
2 7 neurofilament cytoskeleton organization
Tissue: Muscle_Skeletal=>Whole_Blood Type: asymmetric
SourceGene: REM1(ENSG00000088320.3)
TargetGeneSet: SYT12 PPP1R12B RP11 PRKCD RPN1 PAICS DAPP1
RTN3P1 CDHR2 CANX OGFRL1 CDK6 PDIA4 RNF32 ANXA1 RPL12P8 COMTD1
TRIM3 GDF3 ZNF592 STUB1 C16orf88 TP53 SCRN2 MIS18A-AS1
GOBPID Pvalue OddsRatio ExpCount Count Size Term
GO:0090398 3.88690786963359e-06 128.660714285714 0.0317625077849284

3 27 cellular senescence
Tissue: Whole_Blood=>Muscle_Skeletal Type: asymmetric
SourceGene: RP11-184M15.1(ENSG00000248187.1)
TargetGeneSet: RP3 ERI3 TAL1 GADD45A RD3 LPGAT1 RP11 ROCK2
OXSR1 ANKRD23 CDCA7 EPHA4 FAM132B LAMB2 SLC41A3 DCUN1D1 BHMT HUS1B
TCF21 ELMO1 CA1 KDM4C FTH1P12 BICC1 METTL7A BRI3BP SOCS6 CHRNA7
GCHFR DYX1C1-CCPG1 SOCS1 FBXL19 ABR NLE1 MED13 SLC25A19
ARHGAP28 SOGA2 GALNT1 ELAVL1 BBC3 BMP2 BCR TOM1 CASK
GOBPID Pvalue OddsRatio ExpCount Count Size Term
GO:0048708 2.23270655358904e-06 53.0175115207373 0.0944571309943949
4 39 astrocyte differentiation
Tissue: Muscle_Skeletal=>Whole_Blood Type: asymmetric
SourceGene: RP11-36C20.1(ENSG00000240376.1)
TargetGeneSet: CELA2A CELA3B CELA3A LAMP3 MUC21 CPA2 CPA1 PRSS1
PRSS3P2 PNLIP PNLIPRP2 TEX12 RP11 SNHG9 KREMEN2 CTRB2 POLR2A SYCN
OTUD5 PIN4
GOBPID Pvalue OddsRatio ExpCount Count Size Term
GO:0007586 1.28159346036606e-07 57.411858974359 0.128226420316933 5
109 digestion
Tissue: Muscle_Skeletal=>Whole_Blood Type: asymmetric
SourceGene: RP11-543B16.1(ENSG00000234004.2)
TargetGeneSet: RP11 MYT1L VSNL1 SCN2A DNER KIF1A SLC6A1 TAGLN3
TECRL GRIA2 CTNND2 GABRB2 GABRA1 GABRG2 ODZ2 SNCB NRSN1 BEND6
SNAP91 FABP7 MLLT4 STMN2 SH3GL2 DIRAS2 OPALIN BAD OPCML PRMT8
FAIM2 CTD CKMT1B ASPHD1 CBLN1 NPTX1 SYT4 ST8SIA3 HSBP1L1 TUBB4A
ELAVL3 UNC13A TCF15 SNAP25 GAL3ST1
GOBPID Pvalue OddsRatio ExpCount Count Size Term
GO:0051932 7.95299767355556e-07 70.5343137254902 0.0736281226212719
4 28 synaptic transmission, GABAergic
GO:0007270 3.19865250630032e-06 26.1591821832786 0.231402671095426
5 88 neuron-neuron synaptic transmission
GO:0007214 1.33208946821051e-05 82.2742857142857 0.0473323645422462
3 18 gamma-aminobutyric acid signaling pathway
Tissue: Whole_Blood=>Muscle_Skeletal Type: asymmetric
SourceGene: SLC6A1(ENSG00000157103.6)
TargetGeneSet: CACNA1A KCNJ9 CNTN2 RP11 MYT1L VSNL1 PTPRN ASB1 MOBP
CAMKV CTD SYNPR TAGLN3 GUF1 GRIA2 GPM6A SOWAHA CTB CPLX2
NRSN1 CTA MOG HCG17 7SK TMEM130 SDSL NEFL STMN2 DIRAS2
GRIN1 VPS26A OPALIN INA SLC1A2 NRGN KIF5A SYT1 GABRB3 FBXL16
LONP2 NEUROD2 RUNDC3A GFAP PNPO SYT4 SCARNA17 MAST1 PSMF1
SNAP25 SLC12A5 CBX6 APOBEC3H MLC1 ZCCHC12
GOBPID Pvalue OddsRatio ExpCount Count Size Term
GO:0006836 1.30577705071748e-06 20.5142310452045 0.35409314234309
6 119 neurotransmitter transport
GO:0001505 1.58500294118228e-06 19.8073458073458 0.365995432842018
6 123 regulation of neurotransmitter levels
GO:0007269 5.34763290410581e-06 23.2732293697206 0.255899245726939
5 86 neurotransmitter secretion
Tissue: Whole_Blood=>Muscle_Skeletal Type: asymmetric
SourceGene: SNAP91(ENSG00000065609.8)
TargetGeneSet: VPS13D STMN1 CACNA1A CELF3 RP11 MYT1L VSNL1 MZT2A ERMN
SCN2A PTPRN SLC16A14 ASB1 MOBP CTD TAGLN3 GUF1 GRIA2
CPLX2 CTA MOG RP1 TMEM130 NEFL STMN2 GRIN1 VPS26A OPALIN INA
NRAP CALY CEND1 SLC1A2 PRMT8 USP5 FAM186B SYT1 C14orf21
KLHDC1 SYNDIG1L TMEM179 SCG5 RPL5P3 GABARAPL3 FBXL16 TOM1L2 BLMH
NEUROD2 DUSP3 RUNDC3A GFAP C18orf34 SYT4 MAST1 TTYH1 SNAP25
SLC12A5 POLR3H SEPT3 LDOC1L
GOBPID Pvalue OddsRatio ExpCount Count Size Term
GO:0006836 1.72131677403843e-06 19.4594962559564 0.370562590824164
6 119 neurotransmitter transport
GO:0001505 2.08841173625885e-06 18.7889546351085 0.383018476230019
6 123 regulation of neurotransmitter levels
GO:0007269 6.72428868269139e-06 22.1064814814815 0.267801536225867

5	86	neurotransmitter secretion
Tissue: Whole_Blood=>Muscle_Skeletal	Type:	asymmetric
SourceGene:	ZNF276(ENSG00000158805.7)
TargetGeneSet:	PAFAH2	CERS2	PYGO2	SLAMF7	FCGR2A	PTPRC	PLEK	MGAT5
TMEFF2	RP11	GJA1	GIMAP2	RNF141	SPI1	CD3E	REP15	ARHGAP9	LCP1
LINC00441	RCBTB2	MCTP2	SPN	C16orf54	CORO1A	DUSP14	SOCS3
HMHA1	JAK3	TYROBP	SERTAD3	BCL3	C19orf76	ZNF628	HCK	ITGB2	RAC2
GOBPID	Pvalue	OddsRatio	ExpCount	Count	Size	Term
GO:0042102	1.54330267381976e-07	51.1831789023521	0.127880423500104
5	66	positive regulation of T cell proliferation
GO:0002252	4.68992648981497e-07	14.8624338624339	0.747906719258183
8	386	immune effector process
GO:0050671	1.01426167023644e-06	34.2379359770664	0.186007888727424
5	96	positive regulation of lymphocyte proliferation
GO:0032946	1.06796307774564e-06	33.8634215500945	0.187945470901668
5	97	positive regulation of mononuclear cell proliferation
GO:0046649	1.11743181211452e-06	13.1745882352941	0.838973081447651
8	433	lymphocyte activation
GO:0070665	1.24265332334512e-06	32.7871853546911	0.1937582174244 5
100	positive regulation of leukocyte proliferation
GO:0042129	1.30563684756582e-06	32.4433876811594	0.195695799598644
5	101	regulation of T cell proliferation
GO:0042110	1.90053653913798e-06	15.0758547008547	0.618088713583835
7	319	T cell activation
GO:0042098	2.81950870580034e-06	27.5298191612159	0.228634696560792
5	118	T cell proliferation
GO:0050865	3.07598683139619e-06	13.9751984126984	0.664590685765691
7	343	regulation of cell activation
GO:0050867	5.46715383363551e-06	16.537296037296 0.465019721818559	6
240	positive regulation of cell activation
GO:0050670	7.48770937435757e-06	22.3396934626212	0.279011833091136
5	144	regulation of lymphocyte proliferation
GO:0032944	7.74480916145515e-06	22.1785714285714	0.28094941526538
5	145	regulation of mononuclear cell proliferation
GO:0007229	8.43117066891122e-06	37.3932291666667	0.131755587848592
4	68	integrin-mediated signaling pathway
GO:0070663	9.13597438646053e-06	21.4062968515742	0.2906373261366 5
150	regulation of leukocyte proliferation
GO:0030217	1.10557334963855e-05	20.5470774546502	0.302262819182064
5	156	T cell differentiation
GO:0051249	1.40216944209456e-05	13.927797833935 0.548335755311051	6
283	regulation of lymphocyte activation
GO:0050870	1.72487052878309e-05	18.6707700366684	0.331326551795723
5	171	positive regulation of T cell activation
GO:0046651	1.77414845089915e-05	18.5576672741474	0.333264133969967
5	172	lymphocyte proliferation
GO:0032943	1.87599746165927e-05	18.3354772317983	0.337139298318455
5	174	mononuclear cell proliferation
Tissue: Skin_Sun_Exposed_Lower_leg=>Nerve_Tibial	Type:	asymmetric
SourceGene:	C11orf51(ENSG00000110200.4)
TargetGeneSet:	CROCCP3	RPL11	TMEM57	NASP	RPL5	TOMM20	RPL31	RPL15
RPL14	RPL24	RP11	ZNF718	GALNT7	FAM149A	KLKB1	EEF1A1P19	CAMLG
RPS14	EEF1A1	BCKDHB	MKRN1	SSPO	RPS20	RPL30	EIF3H	EEF1D	SCRIB	RPS6
TCEA1P1	CCL21	RPL12	LDB1	RPLP2	RPL27A	FAU	RPS25	TMEM136	ATP5G2
RPL41	RPL14P1	U8	RPL4	ALDH3A1	RPL23A	RPL17	RPL19	KPNA2	MUM1
CEP89	PTOV1	EIF3L	RANGAP1	TTLL12	RPS4X	RPL10
GOBPID	Pvalue	OddsRatio	ExpCount	Count	Size	Term
GO:0006414	3.93523516651412e-40	184.515297906602	0.341498858210504
24	105	translational elongation
GO:0006415	3.22333858429592e-37	182.823188405797	0.295965677115771
22	91	translational termination
GO:0006413	6.67234534614046e-36	116.380434782609	0.494360251885683
24	152	translational initiation

GO:0006614	1.09240280307253e-35	151.837108433735	0.341498858210504
22	105	SRP-dependent cotranslational protein targeting to membrane
GO:0006613	1.37621165623957e-35	150.019047619048	0.3447512282887	22
106	cotranslational protein targeting to membrane
GO:0045047	1.37621165623957e-35	150.019047619048	0.3447512282887	22
106	protein targeting to ER
GO:0072599	1.37621165623957e-35	150.019047619048	0.3447512282887	22
106	establishment of protein localization to endoplasmic reticulum
GO:0070972	1.84238264441215e-34	131.156666666667	0.383779669227043
22	118	protein localization to endoplasmic reticulum
GO:0000184	2.25647386992946e-34	129.795463917526	0.387032039305238
22	119	nuclear-transcribed mRNA catabolic process, nonsense-mediated decay
GO:0019080	6.40124123854654e-32	97.3798449612403	0.491107881807487
22	151	viral genome expression
GO:0019083	6.40124123854654e-32	97.3798449612403	0.491107881807487
22	151	viral transcription
GO:0006612	8.71228281547153e-32	95.8796946564886	0.497612621963878
22	153	protein targeting to membrane
GO:0043624	8.71228281547153e-32	95.8796946564886	0.497612621963878
22	153	cellular protein complex disassembly
GO:0043241	1.84769533836988e-31	92.3223529411765	0.513874472354854
22	158	protein complex disassembly
GO:0000956	8.85202173874874e-31	85.3480272108844	0.549650543215002
22	169	nuclear-transcribed mRNA catabolic process
GO:0072594	9.40156975132788e-31	75.3059392265193	0.663483495951837
23	204	establishment of protein localization to organelle
GO:0034623	1.7402654742189e-30	82.5115789473684	0.565912393605979
22	174	cellular macromolecular complex disassembly
GO:0006402	2.94429546497264e-30	80.3733333333333	0.57892187391876
22	178	mRNA catabolic process
GO:0032984	3.3512531851011e-30	79.855796178344	0.582174243996955	22
179	macromolecular complex disassembly
GO:0006401	7.56836305532536e-29	68.3851366120219	0.666735866030033
22	205	RNA catabolic process
GO:0019058	1.14795816151814e-27	59.7684210526316	0.75129748806311
22	231	viral infectious cycle
GO:0071845	2.42024313434875e-27	51.9299169859515	0.92367310220746
23	284	cellular component disassembly at cellular level
GO:0022411	3.36061533517501e-27	51.1316037735849	0.936682582520241
23	288	cellular component disassembly
GO:0022415	1.66022616153413e-26	52.378487394958	0.845616220330773	22
260	viral reproductive process
GO:0006605	3.2150454592583e-25	35.3183842883175	1.54162341706456
25	474	protein targeting
GO:0033365	1.03305378704065e-23	32.4314902682289	1.53837104698637
24	473	protein localization to organelle
GO:0016032	3.25821142747066e-21	28.6666666666667	1.46681890526607
22	451	viral reproduction
GO:0000028	6.40494922747351e-07	327.295454545455	0.0195142204691717
3	6	ribosomal small subunit assembly
GO:0042273	6.94949201671952e-06	109.05303030303	0.0390284409383434	3
12	ribosomal large subunit biogenesis
GO:0022613	7.48271332996681e-06	11.3878440366972	0.731783267593938
7	225	ribonucleoprotein complex biogenesis
GO:0042254	8.23103658715123e-06	14.5942350332594	0.484603141651097
6	149	ribosome biogenesis
GO:0071843	1.10960357636727e-05	10.6900862068966	0.777316448688672
7	239	cellular component biogenesis at cellular level
GO:0042255	1.75287177006372e-05	75.4772727272727	0.0520379212511245
3	16	ribosome assembly
Tissue: Skin_Sun_Exposed_Lower_leg=>Nerve_Tibial	Type:	asymmetric
SourceGene:	CIB2(ENSG00000136425.8)
TargetGeneSet:	RPL22	RPL11	RPS27	GAS5	ASTN1	UBC	RPL31	MLPH	XPC

```
RPL14   KPNA1   RP11    PLD1    RPL34   RPS3A   HOMER1  EIF4EBP3        EFCAB9
RPL10A  VN1R42P RPS20   RPL30   RPL27A  DDB2    PITPNM1 RPS25   NCKAP5L PFDN5
RPL41   NACA    CYP27B1 SSH1    RPS3AP6 SEC11A  RPS15A  NFAT5   RPL26   C17orf48
RPL23A  RPL17   RPL19   WIZ     UBA52   FTL     RPS4X   HAUS7   RPL10
GOBPID  Pvalue  OddsRatio       ExpCount        Count   Size    Term
GO:0006614      3.93238183764693e-40    236.418220946915        0.2906373261366 23
105     SRP-dependent cotranslational protein targeting to membrane
GO:0006613      5.01637705209728e-40    233.553508150248        0.293405300671234
23      106     cotranslational protein targeting to membrane
GO:0045047      5.01637705209728e-40    233.553508150248        0.293405300671234
23      106     protein targeting to ER
GO:0072599      5.01637705209728e-40    233.553508150248        0.293405300671234
23      106     establishment of protein localization to endoplasmic reticulum
GO:0006415      2.54414903770958e-39    254.045088566828        0.25188568265172
22      91      translational termination
GO:0070972      7.72410679129691e-39    203.881114551084        0.326620995086845
23      118     protein localization to endoplasmic reticulum
GO:0006414      8.67876567754578e-38    210.987951807229        0.2906373261366 22
105     translational elongation
GO:0000184      1.80445658011118e-36    180.359679266896        0.329388969621479
22      119     nuclear-transcribed mRNA catabolic process, nonsense-mediated decay
GO:0006413      4.31958891118867e-36    149.788417692658        0.420732129264411
23      152     translational initiation
GO:0006612      5.07803517317897e-36    148.625791855204        0.423500103799045
23      153     protein targeting to membrane
GO:0019080      5.19616739139964e-34    135.316106804479        0.417964154729776
22      151     viral genome expression
GO:0019083      5.19616739139964e-34    135.316106804479        0.417964154729776
22      151     viral transcription
GO:0043624      7.07876921231269e-34    133.23155216285 0.423500103799045       22
153     cellular protein complex disassembly
GO:0043241      1.50478167661709e-33    128.28839869281 0.437339976472216       22
158     protein complex disassembly
GO:0072594      5.6314287424571e-33     106.366590835229        0.564666805065393
23      204     establishment of protein localization to organelle
GO:0000956      7.24644259793017e-33    118.597127739985        0.467787696353194
22      169     nuclear-transcribed mRNA catabolic process
GO:0034623      1.42796062147389e-32    114.655701754386        0.481627569026365
22      174     cellular macromolecular complex disassembly
GO:0006402      2.42045501881131e-32    111.684472934473        0.492699467164902
22      178     mRNA catabolic process
GO:0032984      2.75630086898174e-32    110.965322009908        0.495467441699536
22      179     macromolecular complex disassembly
GO:0019058      1.1129298490132e-31     92.3837669683258        0.639402117500519
23      231     viral infectious cycle
GO:0006401      6.30120187678486e-31    95.0261080752884        0.567434779600028
22      205     RNA catabolic process
GO:0022415      1.86162517444779e-30    80.9138744105237        0.719673379004913
23      260     viral reproductive process
GO:0071845      1.50548546374765e-29    73.3491097588461        0.786104767836136
23      284     cellular component disassembly at cellular level
GO:0022411      2.09439452941768e-29    72.2215316315205        0.797176665974673
23      288     cellular component disassembly
GO:0006605      1.07853780536434e-27    51.826280623608 1.31201992941665        25
474     protein targeting
GO:0033365      4.97986219813e-26       46.6436525612472        1.30925195488202
24      473     protein localization to organelle
GO:0016032      7.30060770849561e-25    44.2013468938977        1.24835651512006
23      451     viral reproduction
GO:0042273      4.24829873259561e-06    129.747747747748        0.0332156944156114
3       12      ribosomal large subunit biogenesis
Tissue: Nerve_Tibial=>Skin_Sun_Exposed_Lower_leg        Type:   asymmetric
SourceGene:     EIF3M(ENSG00000149100.8)
```

TargetGeneSet: LAMTOR2 ACBD6 GLT25D2 KLHL12 RP11 ADCK3 NUP133 TOMM20 UBC
MZT2B WDR75 GTF3C3 CNPPD1 TMEM111 HDAC11 FNDC3B HAUS3 LIAS NOA1
NUP54 ZNF622 BTF3 RPS14 HNRNPC RPL10A C6orf130 PSMB1 MRPS24
BUD31 MKRN1 HOOK3 C9orf123 RPS6 FAM95B1 AKR1C1 MGEA5 POLR2G FAU
THYN1 C12orf57 PRKAG1 PWP1 SAP18 C14orf183 SPTB C14orf2
GCHFR LYSMD2 ANP32A RPS2 RPS15A NUPR1 BCKDK GGNBP2 FN3KRP ELP2
PRKCSH ZNF826P SNRPD2 RPL13A RPS5 NSFL1C CRYZL1 SAMM50 EDA IL13RA2
GOBPID Pvalue OddsRatio ExpCount Count Size Term
GO:0006612 1.28973883831421e-11 24.5356807511737 0.592900145318663
11 153 protein targeting to membrane
GO:0043241 1.8325166667218e-11 23.6928193499622 0.612275967061103
11 158 protein complex disassembly
GO:0006415 6.1771917725899e-11 33.4242345614946 0.352639955712407
9 91 translational termination
GO:0032984 7.10308950550242e-11 20.7006613756614 0.693654418379351
11 179 macromolecular complex disassembly
GO:0006414 2.27284005861797e-10 28.5219414893617 0.406892256591239
9 105 translational elongation
GO:0006614 2.27284005861797e-10 28.5219414893617 0.406892256591239
9 105 SRP-dependent cotranslational protein targeting to membrane
GO:0006613 2.47642470537799e-10 28.2259267383198 0.410767420939727
9 106 cotranslational protein targeting to membrane
GO:0045047 2.47642470537799e-10 28.2259267383198 0.410767420939727
9 106 protein targeting to ER
GO:0072599 2.47642470537799e-10 28.2259267383198 0.410767420939727
9 106 establishment of protein localization to endoplasmic reticulum
GO:0019080 2.71259836378899e-10 21.9765649090348 0.585149816621687
10 151 viral genome expression
GO:0019083 2.71259836378899e-10 21.9765649090348 0.585149816621687
10 151 viral transcription
GO:0072594 2.89516314673346e-10 17.9875647668394 0.790533527091551
11 204 establishment of protein localization to organelle
GO:0043624 3.08861291364601e-10 21.6661599270295 0.592900145318663
10 153 cellular protein complex disassembly
GO:0070972 6.50848000839002e-10 25.0974038649229 0.457269393121583
9 118 protein localization to endoplasmic reticulum
GO:0000184 7.02029640361192e-10 24.8675048355899 0.461144557470071
9 119 nuclear-transcribed mRNA catabolic process, nonsense-mediated decay
GO:0034623 1.0908891756791e-09 18.8639978791092 0.674278596636911
10 174 cellular macromolecular complex disassembly
GO:0006605 2.26412594476969e-09 10.0978260869565 1.83682790118331
14 474 protein targeting
GO:0006413 6.1746345430566e-09 19.0846600208302 0.589024980970175
9 152 translational initiation
GO:0022411 1.09352464497453e-08 12.4587244283995 1.11604733236454
11 288 cellular component disassembly
GO:0016032 1.24746289364511e-08 9.63369438249974 1.74769912116809
13 451 viral reproduction
GO:0000956 1.56302678744595e-08 17.0365691489362 0.654902774894471
9 169 nuclear-transcribed mRNA catabolic process
GO:0019058 1.67773738420351e-08 13.9425536100728 0.895162964500727
10 231 viral infectious cycle
GO:0033365 2.19498901115659e-08 9.15849342770475 1.83295273683482
13 473 protein localization to organelle
GO:0006402 2.45514522992741e-08 16.1190985773637 0.689779254030863
9 178 mRNA catabolic process
GO:0022415 5.14251305473823e-08 12.3 1.00754273060688 10 260
viral reproductive process
GO:0006401 8.30638180347452e-08 13.872231871472 0.794408691440039 9
205 RNA catabolic process
GO:0071845 1.17653160995401e-07 11.2035861631228 1.10054667497059
10 284 cellular component disassembly at cellular level
Tissue: Skin_Sun_Exposed_Lower_leg=>Nerve_Tibial Type: asymmetric

SourceGene: MMP27(ENSG00000137675.4)
TargetGeneSet: TMEM201 RPL11 CDC42SE1 CCT3 RP11 URB2 CDC42EP3
RTKN C2orf55 RPL31 RFX8 R3HDM1 HOXD8 RPL37A OTOS RPL32 CRTAP
RPL14 IFRD2 CCDC48 GPR160 PLD1 EIF4G1 RPL35A LETM1 SOD3 UBA6
RPL34 CCT5 RPL37 EPB41L4A EPB41L4A-AS1 CTD LYRM4 HLA-C
PAQR8 CD109 ICA1 RPS20 SCRIB LRRC14 KCNV2 RPS6 QSOX2 EGFL7
SNHG7 ABCA2 RPL13AP5 PTDSS2 RPLP2 RPS13 FAU CCL13 C2CD3 RPS3
CAPRIN2 KIF21A FMNL3 NCKAP5L MARS RPL14P1 GPR137C C14orf135 RPL3P4
TP53BP1 RPLP1 TMC7 U6 RPL17 RPL19 ACLY PRPSAP1 RPL18 RPS9
RPS21 NPTXR PPARA PIM2 NLGN3 RPL10 PLXNA3
GOBPID Pvalue OddsRatio ExpCount Count Size Term
GO:0006415 2.18531216577091e-32 113.068421052632 0.371531381911286
21 91 translational termination
GO:0000184 1.26188456293878e-31 87.6260796879354 0.485848730191682
22 119 nuclear-transcribed mRNA catabolic process, nonsense-mediated decay
GO:0006414 6.09772356402699e-31 94.1315789473684 0.428690056051484
21 105 translational elongation
GO:0006614 6.09772356402699e-31 94.1315789473684 0.428690056051484
21 105 SRP-dependent cotranslational protein targeting to membrane
GO:0006613 7.58500679264764e-31 93.0176470588235 0.43277281849007
21 106 cotranslational protein targeting to membrane
GO:0045047 7.58500679264764e-31 93.0176470588235 0.43277281849007
21 106 protein targeting to ER
GO:0072599 7.58500679264764e-31 93.0176470588235 0.43277281849007
21 106 establishment of protein localization to endoplasmic reticulum
GO:0070972 8.80646416025724e-30 81.4419424850787 0.481765967753097
21 118 protein localization to endoplasmic reticulum
GO:0006413 4.06936847496523e-29 65.2316008316008 0.620579890665006
22 152 translational initiation
GO:0000956 4.75552145898171e-28 57.6190476190476 0.68998685212096
22 169 nuclear-transcribed mRNA catabolic process
GO:0006402 1.57033952636964e-27 54.2605682605683 0.726731714068231
22 178 mRNA catabolic process
GO:0019080 2.21888062683604e-27 60.6279352226721 0.61649712822642
21 151 viral genome expression
GO:0019083 2.21888062683604e-27 60.6279352226721 0.61649712822642
21 151 viral transcription
GO:0006612 2.9690674028572e-27 59.700956937799 0.624662653103591 21
153 protein targeting to membrane
GO:0043624 2.9690674028572e-27 59.700956937799 0.624662653103591 21
153 cellular protein complex disassembly
GO:0043241 6.04079034146103e-27 57.5019208605455 0.645076465296519
21 158 protein complex disassembly
GO:0006401 3.9497866500228e-26 46.1671835770196 0.836966299910041
22 205 RNA catabolic process
GO:0034623 5.02452105141684e-26 51.4308565531476 0.710400664313888
21 174 cellular macromolecular complex disassembly
GO:0032984 9.32911505383716e-26 49.7858094603598 0.730814476506816
21 179 macromolecular complex disassembly
GO:0072594 1.59168676835374e-24 42.9089732528041 0.832883537471455
21 204 establishment of protein localization to organelle
GO:0019058 2.29831854276085e-23 37.321052631579 0.943118123313265 21
231 viral infectious cycle
GO:0022415 2.84789655475449e-22 32.7255009909711 1.06151823403225
21 260 viral reproductive process
GO:0071845 1.83803761985036e-21 29.6887132279368 1.1595045325583 21
284 cellular component disassembly at cellular level
GO:0022411 2.46690905591522e-21 29.235659373152 1.17583558231264 21
288 cellular component disassembly
GO:0016032 2.62847622369186e-17 17.9438188494492 1.84132585980209
21 451 viral reproduction
GO:0033365 6.90344421122322e-17 17.0435491383326 1.93114663345097
21 473 protein localization to organelle

GO:0006605	7.20480194752119e-17	17.00470547229	1.93522939588956	21
474	protein targeting
GO:0042273	1.38548381246585e-05	85.6130952380952	0.0489931492630268
3	12	ribosomal large subunit biogenesis
Tissue: Skin_Sun_Exposed_Lower_leg=>Nerve_Tibial	Type:	asymmetric
SourceGene:	PRG4(ENSG00000116690.7)
TargetGeneSet:	RPL22	SLC45A1	RP4	CROCCP3	RPL11	GTF2B	RPL5	VPS45
PLXNA2	RP11	FAM179A	PPM1B	THSD7B	STK39	SATB2	RPL32	RPL15	SPICE1
RPL34	CTD	EEF1A1P19	ARSK	CAMLG	SLU7	RPL10A	EEF1A1	BCKDHB	ELN
GNAI1	CPSF4	U6	RPL30	EIF3E	EIF3H	RPS6	RPL12	NUDT5	OR13A1
RPS13	FAU	RPS25	LGR5	RPS2P5	ZNF605	GPR137C	RSL24D1	RPS3AP6	RPL4
HEATR3	WSCD1	MAPK7	RNF112	FAM106B	RPL23A	RPL17	RPL19	TMEM104	SOCS6
SAMD1	ZNF233	FTL	GYS1	FER1L4	LSS	EIF3L	RPS4X	RPL10
GOBPID	Pvalue	OddsRatio	ExpCount	Count	Size	Term
GO:0006413	4.35570908347227e-31	86.2532967032967	0.525915161580513
22	152	translational initiation
GO:0006414	9.81471333792888e-31	112.282352941176	0.363296657670749
20	105	translational elongation
GO:0006415	5.3704315342553e-30	121.976254480287	0.31485710331465
19	91	translational termination
GO:0000184	1.48520467396305e-29	96.3097643097643	0.411736212026849
20	119	nuclear-transcribed mRNA catabolic process, nonsense-mediated decay
GO:0006614	1.0549507204945e-28	102.019879969993	0.363296657670749
19	105	SRP-dependent cotranslational protein targeting to membrane
GO:0006613	1.28270559702417e-28	100.840192806822	0.366756625839042
19	106	cotranslational protein targeting to membrane
GO:0045047	1.28270559702417e-28	100.840192806822	0.366756625839042
19	106	protein targeting to ER
GO:0072599	1.28270559702417e-28	100.840192806822	0.366756625839042
19	106	establishment of protein localization to endoplasmic reticulum
GO:0070972	1.15508207417614e-27	88.5428478331704	0.408276243858556
19	118	protein localization to endoplasmic reticulum
GO:0000956	2.52199054182393e-26	63.7673378076063	0.584734620441492
20	169	nuclear-transcribed mRNA catabolic process
GO:0006402	7.43531417677252e-26	60.0970464135021	0.615874333956128
20	178	mRNA catabolic process
GO:0019080	1.65882790846359e-25	66.2539100684262	0.522455193412221
19	151	viral genome expression
GO:0019083	1.65882790846359e-25	66.2539100684262	0.522455193412221
19	151	viral transcription
GO:0006612	2.15571449226425e-25	65.2558979297063	0.529375129748806
19	153	protein targeting to membrane
GO:0043624	2.15571449226425e-25	65.2558979297063	0.529375129748806
19	153	cellular protein complex disassembly
GO:0043241	4.08474764064686e-25	62.8865165931771	0.546674970590271
19	158	protein complex disassembly
GO:0006401	1.3805767162083e-24	51.2288288288288	0.709293474500035
20	205	RNA catabolic process
GO:0034623	2.75125290830841e-24	56.331737773153	0.602034461282956	19
174	cellular macromolecular complex disassembly
GO:0032984	4.80452761347157e-24	54.5522177419355	0.61933430212442
19	179	macromolecular complex disassembly
GO:0019058	1.59507992332374e-23	44.8341232227488	0.799252646875649
20	231	viral infectious cycle
GO:0072594	6.2000572913919e-23	47.0974716652136	0.705833506331742
19	204	establishment of protein localization to organelle
GO:0022415	1.76520224049109e-22	39.3361111111111	0.899591723756141
20	260	viral reproductive process
GO:0071845	3.62015978859154e-20	32.6943396226415	0.98263095979517
19	284	cellular component disassembly at cellular level
GO:0022411	4.7241771343403e-20	32.1990646360475	0.996470832468341
19	288	cellular component disassembly
GO:0016032	4.42119662935522e-19	23.5277465918204	1.56044564390008

21	451	viral reproduction
GO:0033365	2.54654235090876e-17	20.5268579838116	1.63656494360252
20	473	protein localization to organelle
GO:0006605	2.65331051473204e-17	20.4801762114537	1.64002491177081
20	474	protein targeting
GO:0022613	1.14224741638247e-05	10.5911030509921	0.778492837865892
7	225	ribonucleoprotein complex biogenesis
GO:0071843	1.68953775952073e-05	9.94216118684844	0.826932392221992
7	239	cellular component biogenesis at cellular level
Tissue: Nerve_Tibial=>Skin_Sun_Exposed_Lower_leg	Type:	asymmetric
SourceGene:	RP11-134P9.1(ENSG00000233791.1)
TargetGeneSet:	PADI3	PTGFRN	RP11	TCHH	S100A3	C1orf204	VSIG8
MYCNOS	LYG2	ALS2CL	RAP2B	RP1	TTYH3	LRRC4	CUL1	RP4	LZTS1	BNC2
KRTAP5-3	TMPRSS5	CCND2	GPRC5D	KRT81	KRT83	KRT85	KRT82	KRT71
BEST3	RARA	KRT25	KRT26	KRT27	KRT28	KRTAP3-3	KRTAP3-1
KRTAP1-3	KRTAP2-2	KRTAP4-9	KRTAP4-12	KRTAP4-6
KRTAP4-5	KRTAP4-3	KRTAP9-2	KRTAP9-3	KRTAP9-8
KRTAP16-1	KRTAP17-1	KRT33A	KRT34	KRT38	KRT32	KRT35	KRT36
TIMM21	TGM6	CST1	KRTAP24-1	KRTAP26-1	KRTAP13-2	KRTAP13-1
KRTAP19-1	KRTAP19-3	KRTAP19-5	KRTAP11-1	KRTAP10-1
KRTAP10-3	KRTAP10-5	KRTAP10-6	KRTAP10-7	KRTAP10-8
KRTAP10-9	KRTAP10-10	KRTAP12-3	KRTAP12-2	KRTAP12-1
KRTAP10-12	APOBEC3C
GOBPID	Pvalue	OddsRatio	ExpCount	Count	Size	Term
GO:0008544	1.21542717159529e-10	26.0802355539198	0.569095564320808
10	257	epidermis development
Tissue: Nerve_Tibial=>Skin_Sun_Exposed_Lower_leg	Type:	cluster
SourceGene:	RP11-134P9.1(ENSG00000233791.1)
TargetGeneSet:	PADI3	PTGFRN	RP11	TCHH	S100A3	C1orf204	VSIG8
MYCNOS	LYG2	ALS2CL	RAP2B	RP1	TTYH3	LRRC4	CUL1	RP4	LZTS1	BNC2
KRTAP5-3	TMPRSS5	CCND2	GPRC5D	KRT81	KRT83	KRT85	KRT82	KRT71
BEST3	RARA	KRT25	KRT26	KRT27	KRT28	KRTAP3-3	KRTAP3-1
KRTAP1-3	KRTAP2-2	KRTAP4-9	KRTAP4-12	KRTAP4-6
KRTAP4-5	KRTAP4-3	KRTAP9-2	KRTAP9-3	KRTAP9-8
KRTAP16-1	KRTAP17-1	KRT33A	KRT34	KRT38	KRT32	KRT35	KRT36
TIMM21	TGM6	CST1	KRTAP24-1	KRTAP26-1	KRTAP13-2	KRTAP13-1
KRTAP19-1	KRTAP19-3	KRTAP19-5	KRTAP11-1	KRTAP10-1
KRTAP10-3	KRTAP10-5	KRTAP10-6	KRTAP10-7	KRTAP10-8
KRTAP10-9	KRTAP10-10	KRTAP12-3	KRTAP12-2	KRTAP12-1
KRTAP10-12	APOBEC3C
GOBPID	Pvalue	OddsRatio	ExpCount	Count	Size	Term
GO:0008544	1.21542717159529e-10	26.0802355539198	0.569095564320808
10	257	epidermis development
Tissue: Skin_Sun_Exposed_Lower_leg=>Nerve_Tibial	Type:	asymmetric
SourceGene:	RP11-359P5.1(ENSG00000249996.1)
TargetGeneSet:	PLA2G2A	RP11	SORT1	C1orf9	TTC32	RPLP0P6	RPL15	GPR160
EIF4G1	HSD17B11	ADH1C	IL6ST	CTD	GNB2L1	PACSIN1	RPL10A	CTB
PNPLA8	SSPO	EIF3E	RPL7A	FAM208B	C10orf95	MICAL2	MICALCL	ABTB2
KLRF1	FAM19A2	RPL18AP3	PPTC7	RPLP0	OXA1L	ARID4A	TBC1D2B	GOLGA6L4
OR2C1	C16orf46	RPL26	RPL23A	COX11	TEX2	RPL13A	ZNF28	RPS5
CBR3-AS1	RPL10
GOBPID	Pvalue	OddsRatio	ExpCount	Count	Size	Term
GO:0000184	1.79752529619068e-15	63.3651368760064	0.279980624178258
11	119	nuclear-transcribed mRNA catabolic process, nonsense-mediated decay
GO:0006413	2.81102848889924e-14	48.4230650632131	0.357622309874749
11	152	translational initiation
GO:0006414	3.00209303319272e-14	62.8157894736842	0.24704172721611
10	105	translational elongation
GO:0000956	9.14113499326756e-14	43.1615299944964	0.397619541900215
11	169	nuclear-transcribed mRNA catabolic process
GO:0006402	1.62388034869741e-13	40.8096849778703	0.418794547090167
11	178	mRNA catabolic process
GO:0006415	4.78713812070416e-13	62.9341463414634	0.214102830253962

9	91	translational termination
GO:0006401	7.69623482128468e-13	35.0634244733303	0.482319562660024
11	205	RNA catabolic process
GO:0043624	1.38765298793194e-12	41.5909090909091	0.359975088229188
10	153	cellular protein complex disassembly
GO:0006614	1.79564410525806e-12	53.70375	0.24704172721611	9
105	SRP-dependent cotranslational protein targeting to membrane
GO:0043241	1.91798284449093e-12	40.1717342342342	0.371738980001384
10	158	protein complex disassembly
GO:0006613	1.95917933070476e-12	53.1463917525773	0.249394505570549
9	106	cotranslational protein targeting to membrane
GO:0045047	1.95917933070476e-12	53.1463917525773	0.249394505570549
9	106	protein targeting to ER
GO:0072599	1.95917933070476e-12	53.1463917525773	0.249394505570549
9	106	establishment of protein localization to endoplasmic reticulum
GO:0034623	5.04667242327994e-12	36.2118902439024	0.40938343367241
10	174	cellular macromolecular complex disassembly
GO:0070972	5.23481552223919e-12	47.2557798165138	0.277627845823818
9	118	protein localization to endoplasmic reticulum
GO:0032984	6.69863956678448e-12	35.1282051282051	0.421147325444606
10	179	macromolecular complex disassembly
GO:0019080	4.90384069790226e-11	36.1901408450704	0.35526953152031
9	151	viral genome expression
GO:0019083	4.90384069790226e-11	36.1901408450704	0.35526953152031
9	151	viral transcription
GO:0006612	5.5208947747555e-11	35.6825	0.359975088229188	9	153
protein targeting to membrane
GO:0071845	6.34576895101125e-10	21.50699513382	0.668189052660715	10
284	cellular component disassembly at cellular level
GO:0072594	7.21199214575709e-10	26.256	0.479966784305584	9	204
establishment of protein localization to organelle
GO:0022411	7.27008819346467e-10	21.1915467625899	0.677600166078472
10	288	cellular component disassembly
GO:0019058	2.16087239176098e-09	23.0189189189189	0.543491799875441
9	231	viral infectious cycle
GO:0022415	6.09193218574354e-09	20.3177689243028	0.611722372154176
9	260	viral reproductive process
GO:0016032	6.79301792375875e-07	11.3823529411765	1.06110303785205
9	451	viral reproduction
GO:0033365	1.01075029464476e-06	10.8256034482759	1.11286416164971
9	473	protein localization to organelle
GO:0006605	1.02867318047624e-06	10.8015483870968	1.11521694000415
9	474	protein targeting
GO:0071843	1.7535784145997e-05	13.04475781729	0.562314026710954	6
239	cellular component biogenesis at cellular level
Tissue: Skin_Sun_Exposed_Lower_leg=>Nerve_Tibial	Type:	asymmetric
SourceGene:	RP11-456P18.2(ENSG00000229808.1)
TargetGeneSet:	RP3	C1QC	C1QB	HMGCL	LAPTM5	CTSS	SLAMF8	CD84	CD48
ARHGAP30	CHIT1	TLR5	U4	GPX1	TRAT1	CD96	HCLS1	LRRC33
C4orf19	RBM47	MRPL1	CTD	FYB	EMB	ERBB2IP	CCDC112	ABLIM3	ARHGEF37
CD74	GAPDHP71	LY86	HLA-H	HLA-DRA	HLA-DMA	HLA-DOA	COL11A2	DEFA6	FGD2
BVES-AS1	AOAH	GUSB	NCF1C	TFEC	TBXAS1	RP11	EPB49	MYBL1
LRRCC1	SLA	SYK	FGD3	NKX2-1	FCN1	WDFY4	PRF1	PAN3	OR52N4
PLEKHA7	MS4A6A	MS4A7	SLCO2B1	IL10RA	SIAE	CACNA2D4	PTPN6	C3AR1
C12orf35	IRAK4	BIN2	NCKAP1L	YEATS4	P2RX4	ERCC5	GZMH	SERPINA1
SPG11	ITGAL	ACSF3	ZFP3	EVI2B	EVI2A	GHDC	HMHA1	C19orf35
TMPRSS9	TMIGD2	STXBP2	SUGP1	ZNF546	CEACAM21	NKG7	SIGLEC10
LAIR1	FCAR	MAFB	PLTP	RGS19	BRWD1-IT2	ITGB2	CECR6	APOBEC3G
GPR34	WAS	BTK	GAB3
GOBPID	Pvalue	OddsRatio	ExpCount	Count	Size	Term
GO:0002253	5.51180325839901e-09	10.0056818181818	1.59317694277213
13	299	activation of immune response
GO:0050778	6.43565678999881e-09	8.90412698412698	1.93951975641824

14	364	positive regulation of immune response
GO:0050851	1.7307211487588e-08	16.5557275541796	0.655387170438032
9	123	antigen receptor-mediated signaling pathway
GO:0002429	3.42978619868544e-08	15.2099146110057	0.708670680229742
9	133	immune response-activating cell surface receptor signaling pathway
GO:0002757	4.32779456435286e-08	10.5282738095238	1.25216248010518
11	235	immune response-activating signal transduction
GO:0002768	6.83749069799591e-08	13.9598039215686	0.767282541000623
9	144	immune response-regulating cell surface receptor signaling pathway
GO:0002764	7.50060801641483e-08	9.94163150492264	1.32143104283441
11	248	immune response-regulating signaling pathway
GO:0051251	2.0292658794333e-06	9.10279840091376	1.14559546052176
9	215	positive regulation of lymphocyte activation
GO:0019886	2.87593169441638e-06	194.202702702703	0.031970105875026
3	6	antigen processing and presentation of exogenous peptide antigen via
MHC class II
GO:0050870	3.42869900239267e-06	10.1082955454788	0.91114801743824
8	171	positive regulation of T cell activation
GO:0030593	3.47657926738284e-06	25.5252849002849	0.234447443083524
5	44	neutrophil chemotaxis
GO:0002696	3.92689344981484e-06	8.36068802521008	1.24150577814684
9	233	positive regulation of leukocyte activation
GO:0050867	4.99799814425011e-06	8.1033231474408 1.27880423500104	9
240	positive regulation of cell activation
GO:0002504	5.06717964097792e-06	43.7016742770167	0.117223721541762
4	22	antigen processing and presentation of peptide or polysaccharide
antigen via MHC class II
GO:0050900	5.16980516194615e-06	8.06782454361055	1.28413258598021
9	241	leukocyte migration
GO:0002252	5.8858160284775e-06	6.22177777777778	2.05674347796	11
386	immune effector process
GO:0042110	7.06599692442657e-06	6.79370139593296	1.69974396235555
10	319	T cell activation
GO:0050850	7.30147993351765e-06	39.3260273972603	0.127880423500104
4	24	positive regulation of calcium-mediated signaling
GO:0002694	7.46462572720965e-06	6.74905216681864	1.71040066431389
10	321	regulation of leukocyte activation
GO:0002495	7.99090519834147e-06	116.505405405405	0.0426268078333679
3	8	antigen processing and presentation of peptide antigen via MHC class
II
GO:0002250	1.01043864714539e-05	8.65537757437071	1.05501349387586
8	198	adaptive immune response
GO:0045579	1.19403604403125e-05	97.0810810810811	0.0479551588125389
3	9	positive regulation of B cell differentiation
GO:0050865	1.33103492590027e-05	6.29330823360674	1.82762438585565
10	343	regulation of cell activation
GO:0045619	1.42055282453058e-05	12.9768287142208	0.527506746937928
6	99	regulation of lymphocyte differentiation
GO:0050852	1.42055282453058e-05	12.9768287142208	0.527506746937928
6	99	T cell receptor signaling pathway
GO:0046649	1.73307415273254e-05	5.51026856240126	2.30717597398104
11	433	lymphocyte activation
GO:0051249	1.87708302529189e-05	6.81086303134392	1.50792332710539
9	283	regulation of lymphocyte activation
GO:0045621	1.92243727644204e-05	17.442738791423 0.330357760708601	5
62	positive regulation of lymphocyte differentiation
Tissue: Nerve_Tibial=>Skin_Sun_Exposed_Lower_leg	Type:	asymmetric
SourceGene:	RP11-626E13.1(ENSG00000235902.1)
TargetGeneSet:	RPL11	RPS8	RPL5	NBPF10	MIR765	JMJD4	RN5S18	RPL23AP23
SH3YL1	RPS7	RPL31	THUMPD3 RPL32	RPL15	RPL14	RPL24	RPL32P3 RP11
RPL35A	CAMLG	RPS6	EXOSC2	CTSD	C11orf9 RNF169	CASP4	RPS25	POU6F1
PFDN5	RPL14P1 RPL6	RSL24D1 RPS3AP6 RPS17L	FAM103A1		RPS15A	CTD	CTC1
RPL26	RPS7P1	RPL23A	RPL19	RPL27	ZNF529	PSG7	RPS11	KCNS1	FAM209B

CECR7 RP4 RBMX2

GOBPID	Pvalue	OddsRatio	ExpCount	Count	Size	Term
GO:0006415	6.92165224449662e-36	252.535211267606	0.226697114386548	20	91	translational termination
GO:0006414	1.64673108293849e-34	210.735294117647	0.26157359352294	20	105	translational elongation
GO:0006614	1.64673108293849e-34	210.735294117647	0.26157359352294	20	105	SRP-dependent cotranslational protein targeting to membrane
GO:0006613	2.02753692052414e-34	208.270348837209	0.26406477060411	20	106	cotranslational protein targeting to membrane
GO:0045047	2.02753692052414e-34	208.270348837209	0.26406477060411	20	106	protein targeting to ER
GO:0072599	2.02753692052414e-34	208.270348837209	0.26406477060411	20	106	establishment of protein localization to endoplasmic reticulum
GO:0070972	2.10251040588688e-33	182.614795918367	0.293958895578161	20	118	protein localization to endoplasmic reticulum
GO:0000184	2.5245746104842e-33	180.757575757576	0.296450072659332	20	119	nuclear-transcribed mRNA catabolic process, nonsense-mediated decay
GO:0000956	3.57071027686695e-32	134.958108108108	0.421008926717874	21	169	nuclear-transcribed mRNA catabolic process
GO:0006402	1.12637427027264e-31	127.141401273885	0.443429520448412	21	178	mRNA catabolic process
GO:0019080	4.16001389418109e-31	136.297709923664	0.376167739256799	20	151	viral genome expression
GO:0019083	4.16001389418109e-31	136.297709923664	0.376167739256799	20	151	viral transcription
GO:0006413	4.78521822791732e-31	135.255681818182	0.37865891633797	20	152	translational initiation
GO:0006612	5.4989379484775e-31	134.229323308271	0.381150093419141	20	153	protein targeting to membrane
GO:0043624	5.4989379484775e-31	134.229323308271	0.381150093419141	20	153	cellular protein complex disassembly
GO:0043241	1.08622902858703e-30	129.320652173913	0.393605978824995	20	158	protein complex disassembly
GO:0006401	2.51579554452287e-30	108.279347826087	0.510691301640025	21	205	RNA catabolic process
GO:0034623	8.28925107923251e-30	115.75487012987	0.433464812123728	20	174	cellular macromolecular complex disassembly
GO:0032984	1.50170622681188e-29	112.075471698113	0.445920697529583	20	179	macromolecular complex disassembly
GO:0072594	2.29719178236226e-28	96.6779891304348	0.508200124558854	20	204	establishment of protein localization to organelle
GO:0019058	3.01029488319043e-27	84.1469194312796	0.575461905750467	20	231	viral infectious cycle
GO:0022415	3.42319991022414e-26	73.828125	0.647706041104422	20	260	viral reproductive process
GO:0071845	2.08122307600338e-25	67.0028409090909	0.707494291052522	20	284	cellular component disassembly at cellular level
GO:0022411	2.7679832832455e-25	65.9841417910448	0.717458999377206	20	288	cellular component disassembly
GO:0016032	2.33832989655251e-21	40.5568445475638	1.12352086360805	20	451	viral reproduction
GO:0033365	6.03905485999849e-21	38.5264900662252	1.17832675939381	20	473	protein localization to organelle
GO:0006605	6.29823720749117e-21	38.4388766519824	1.18081793647498	20	474	protein targeting
GO:0022613	9.19556629956182e-17	42.8384317104696	0.560514843263442	14	225	ribonucleoprotein complex biogenesis
GO:0071843	2.15048052944305e-16	40.1333333333333	0.595391322399834	14	239	cellular component biogenesis at cellular level
GO:0042254	9.32638157733248e-16	52.1094890510949	0.371185385094457	12	149	ribosome biogenesis
GO:0006364	5.21097056447939e-14	58.5965630114566	0.259082416441769			

10	104	rRNA processing
GO:0016072	9.2709369723543e-14	55.0576923076923	0.274029478928794
10	110	rRNA metabolic process
GO:0034470	3.8257783274865e-11	28.7955465587045	0.498235416234171
10	200	ncRNA processing
GO:0042273	5.6183419832097e-11	331.981566820276	0.0298941249740502
5	12	ribosomal large subunit biogenesis
GO:0042274	6.01309559398377e-10	178.684863523573	0.0448411874610754
5	18	ribosomal small subunit biogenesis
GO:0034660	1.22928431002903e-09	19.7762237762238	0.709985468133693
10	285	ncRNA metabolic process
GO:0000028	2.82515477576935e-07	436.727272727273	0.0149470624870251
3	6	ribosomal small subunit assembly
GO:0022618	3.54261731008932e-06	25.6720430107527	0.236661822711231
5	95	ribonucleoprotein complex assembly
GO:0071826	4.56221489971051e-06	24.3123938879457	0.249117708117085
5	100	ribonucleoprotein complex subunit organization
GO:0042255	7.77602369431775e-06	100.713286713287	0.0398588332987337
3	16	ribosome assembly
Tissue: Skin_Sun_Exposed_Lower_leg=>Nerve_Tibial	Type:	asymmetric
SourceGene:	RP11-727A23.4(ENSG00000254676.1)
TargetGeneSet:	RPS6KA1	TMEM81	UXS1	CDK15	EPM2AIP1	GAP43	CLDN18	RP11
RBM47	BTC	SLC1A3	ERBB2IP	CD74	HLA-H	HLA-DRA	HLA-DMB	HLA-DMA	HLA-DOA
HLA-DPB1	KCNQ5	SNORD50A	FTSJ2	CLDN12	HR	SYK	C9orf152
WDFY4	KIAA1274	FGF8	KNDC1	RARRES3	ALKBH8	PTPRO	MED21	LACC1
ARL11	APBA2	KLHDC4	NUFIP2	IMPACT	HMHA1	TMIGD2	FSD1	TMEM59L	NUMBL	BCL3
BRWD1-IT2	SSR4P1	TMEM27	GPR34
GOBPID	Pvalue	OddsRatio	ExpCount	Count	Size	Term
GO:0002504	9.3239907501338e-12	186.206896551724	0.0532835097917099
6	22	antigen processing and presentation of peptide or polysaccharide antigen via MHC class II
GO:0019882	6.51431909511458e-08	24.6051724137931	0.368140613106359
7	152	antigen processing and presentation
GO:0050867	8.23210804775444e-08	18.1149425287356	0.581274652273199
8	240	positive regulation of cell activation
GO:0050870	1.46119192084503e-07	21.7256097560976	0.414158189744654
7	171	positive regulation of T cell activation
GO:0019886	2.59012915558126e-07	450.40625	0.01453186630683	3
6	antigen processing and presentation of exogenous peptide antigen via MHC class II
GO:0050851	4.46117382246977e-07	25.2855879752431	0.297903259290015
6	123	antigen receptor-mediated signaling pathway
GO:0051251	6.90765278446614e-07	17.0769230769231	0.520725209328074
7	215	positive regulation of lymphocyte activation
GO:0002429	7.0750114798147e-07	23.2783057290253	0.322123036468065
6	133	immune response-activating cell surface receptor signaling pathway
GO:0002495	7.22829210925623e-07	270.20625	0.01937582174244	3
8	antigen processing and presentation of peptide antigen via MHC class II
GO:0042110	7.23165851965889e-07	13.4381326664285	0.772610891979794
8	319	T cell activation
GO:0050863	8.31241752709878e-07	16.5911214953271	0.535257075634904
7	221	regulation of T cell activation
GO:0031294	9.45662089990647e-07	34.1571428571429	0.181648328835375
5	75	lymphocyte costimulation
GO:0031295	9.45662089990647e-07	34.1571428571429	0.181648328835375
5	75	T cell costimulation
GO:0002768	1.12819095447537e-06	21.4062968515742	0.348764791363919
6	144	immune response-regulating cell surface receptor signaling pathway
GO:0002696	1.18502447723853e-06	15.6969026548673	0.564320808248564
7	233	positive regulation of leukocyte activation
GO:0060333	1.22632538943352e-06	32.3018018018018	0.191336239706595
5	79	interferon-gamma-mediated signaling pathway
GO:0050865	1.24856728025995e-06	12.4541735765616	0.830738357207114

8	343	regulation of cell activation
GO:0002757	1.25477136142623e-06	15.5570175438596	0.569164763684174
7	235	immune response-activating signal transduction
GO:0002764	1.79747702408908e-06	14.704356846473 0.600650474015639	7
248	immune response-regulating signaling pathway
GO:0050778	1.94789146810557e-06	11.7020391177695	0.881599889281019
8	364	positive regulation of immune response
GO:0071346	2.91011042818244e-06	26.8295880149813	0.22766590547367
5	94	cellular response to interferon-gamma
GO:0050852	3.7591586958813e-06	25.3936170212766	0.239775794062695
5	99	T cell receptor signaling pathway
GO:0051249	4.31125997728482e-06	12.8079710144928	0.685419694138814
7	283	regulation of lymphocyte activation
GO:0002253	6.1892885825452e-06	12.0924657534247	0.724171337623694
7	299	activation of immune response
GO:0034341	6.59747971644911e-06	22.5	0.268839526676355	5	111
response to interferon-gamma
GO:0046649	7.03762282738726e-06	9.75407407407407	1.04871635180956
8	433	lymphocyte activation
GO:0002694	9.84499468954118e-06	11.2277070063694	0.777454847415404
7	321	regulation of leukocyte activation
GO:0019221	1.45796365421009e-05	10.5404191616766	0.825894401771504
7	341	cytokine-mediated signaling pathway
Tissue: Skin_Sun_Exposed_Lower_leg=>Nerve_Tibial	Type:	asymmetric
SourceGene:	RP11-83A16.1(ENSG00000235096.1)
TargetGeneSet:	C1QC	C1QB	LAPTM5	CD53	FCGR1A	CTSS	FCER1G	FCGR3A
ARL4C	MIR135A1	RP11	SIDT1	HCLS1	FYB	CSF1R	HAVCR2	LY86
GPSM3	TREM2	FAM26F	TRMT11	VNN2	TBXAS1	CTSB	MRC1	FAM86GP	LDHAL6A
MS4A7	FOLR2	KCNE3	SLCO2B1	PTPN6	C3AR1	P2RX4	LCP1	ARL11	GPR183
SERPINA1	GOLGA8J	DPEP3	DPEP2	ARRB2	CD300A	RHBDF2	DSC2	VAV1	EMR1
STXBP2	IL12RB1	IFI30	PDE4C	LRRC25	HCST	SIGLEC9	FPR3	SIRPB1	HCK
RPS3P2	ITGB2	NFAM1	TLR8	RP1	WAS
GOBPID	Pvalue	OddsRatio	ExpCount	Count	Size	Term
GO:0050778	9.68765605504039e-07	8.81481481481481	1.38537125458446
10	364	positive regulation of immune response
GO:0002252	1.64707071475623e-06	8.28605200945626	1.46910248425714
10	386	immune effector process
GO:0002250	9.48230441307929e-06	10.8458769633508	0.753581067054183
7	198	adaptive immune response
GO:0002443	1.43434834545748e-05	10.1454248366013	0.803058611860771
7	211	leukocyte mediated immunity
GO:0002253	1.60009015720984e-05	8.25034729838415	1.13798353055152
8	299	activation of immune response
Tissue: Skin_Sun_Exposed_Lower_leg=>Nerve_Tibial	Type:	asymmetric
SourceGene:	RP11-84C10.4(ENSG00000258471.1)
TargetGeneSet:	RPL11	GPR161	KRT18P12	C2orf63	RPL31	RPL32	RHOA
FAM162A	RPL37	IL6ST	CAMLG	PCDHB5	RPL10A	RPL39P3	SSPO	RPS20	RPL30	RP11
S1PR3	RPL12	ARFIP2	RPS25	PFDN5	RPL41	PARP2	DLST	RPS15A	RPL17
RPL19	ACLY	CTD	UBA52	IGBP1
GOBPID	Pvalue	OddsRatio	ExpCount	Count	Size	Term
GO:0006415	3.89347974264337e-24	173.878787878788	0.182617119922497
14	91	translational termination
GO:0006414	3.29879799035074e-23	146.984615384615	0.210712061449035
14	105	translational elongation
GO:0006614	3.29879799035074e-23	146.984615384615	0.210712061449035
14	105	SRP-dependent cotranslational protein targeting to membrane
GO:0006613	3.79708282379369e-23	145.376811594203	0.212718842986645
14	106	cotranslational protein targeting to membrane
GO:0045047	3.79708282379369e-23	145.376811594203	0.212718842986645
14	106	protein targeting to ER
GO:0072599	3.79708282379369e-23	145.376811594203	0.212718842986645
14	106	establishment of protein localization to endoplasmic reticulum
GO:0070972	1.85228473059208e-22	128.494871794872	0.236800221437963

14	118	protein localization to endoplasmic reticulum
GO:0000184	2.09720741435729e-22	127.262222222222	0.238807002975573
14	119	nuclear-transcribed mRNA catabolic process, nonsense-mediated decay
GO:0019080	6.78599757980201e-21	97.3187347931873	0.303024012179088
14	151	viral genome expression
GO:0019083	6.78599757980201e-21	97.3187347931873	0.303024012179088
14	151	viral transcription
GO:0006413	7.46712331646782e-21	96.6067632850242	0.305030793716698
14	152	translational initiation
GO:0006612	8.21117011725791e-21	95.905035971223 0.307037575254308	14
153	protein targeting to membrane
GO:0043624	8.21117011725791e-21	95.905035971223 0.307037575254308	14
153	cellular protein complex disassembly
GO:0043241	1.30761493981802e-20	92.5425925925926	0.317071482942357
14	158	protein complex disassembly
GO:0000956	3.45346379146708e-20	85.9088172043011	0.339146079856065
14	169	nuclear-transcribed mRNA catabolic process
GO:0034623	5.25409965248226e-20	83.195	0.349179987544115	14	174
cellular macromolecular complex disassembly
GO:0006402	7.28396567836956e-20	81.1430894308943	0.357207113694554
14	178	mRNA catabolic process
GO:0032984	7.89425949776488e-20	80.6456565656566	0.359213895232164
14	179	macromolecular complex disassembly
GO:0072594	5.12801761457407e-19	69.9115789473684	0.40938343367241
14	204	establishment of protein localization to organelle
GO:0006401	5.49849388107925e-19	69.5406631762653	0.41139021521002
14	205	RNA catabolic process
GO:0019058	3.00474331921092e-18	61.0967741935484	0.463566535187876
14	231	viral infectious cycle
GO:0022415	1.60062313346179e-17	53.7842818428184	0.521763199778562
14	260	viral reproductive process
GO:0071845	5.54797086712677e-17	48.9204938271605	0.569925956681198
14	284	cellular component disassembly at cellular level
GO:0022411	6.75234286321892e-17	48.192700729927 0.577953082831638	14
288	cellular component disassembly
GO:0033365	2.16924871329741e-15	32.666874610106 0.949207667289461	15
473	protein localization to organelle
GO:0006605	2.23804350974266e-15	32.593370681606 0.951214448827071	15
474	protein targeting
GO:0016032	3.44467018415915e-14	29.8688024408848	0.905058473462044
14	451	viral reproduction
Tissue: Nerve_Tibial=>Skin_Sun_Exposed_Lower_leg	Type:	cluster
SourceGene:	SPATA25(ENSG00000149634.4)
TargetGeneSet:	RP4	C1orf170	CDC20	KIF2C	ORC1	DEPDC1	FAM72D	MTX1
IQGAP3	NUF2	ASPM	CENPF	DPYSL5	NCAPH	BUB1	TUBA4A	SPHKAP	HJURP
ITPR1	CRELD1	KIF15	WNT5A	SETP14	EIF4A2	TACC3	NCAPG	CCNA2	PCDH10
HMGB2	BRCC3	CCNB1	ZFYVE16	FAM13B	KIF20A	CDC25C	KIAA1191	PBK
ZBTB34	ZWINT	CDK1	HNRNPH3	KIF11	FAM160B1	C10orf46	MKI67	E2F8
STIP1	CDCA5	YAP1	CACNA2D4	FKBP4	TROAP	MBD6	E2F7	ZNF891
DIAPH3	DLGAP5	SLC8A3	AHSA1	BUB1B	CASC5	NUSAP1	CCNB2	KIF23	NPTN
C15orf42	UNC45A	PRC1	SYNM	RP11	HBA2	HBA1	PLK1	AARS
AURKB	CDC6	NKIRAS2	PRPSAP1	BIRC5	RP13	NDC80	SKA1	GNG7	CADM4	TPX2
UBE2C	AURKA	C21orf58	GTSE1	KIF4A
GOBPID	Pvalue	OddsRatio	ExpCount	Count	Size	Term
GO:0000087	5.0847274738295e-32	29.5246559633028	2.01785343574839
33	360	M phase of mitotic cell cycle
GO:0000280	5.91476317426276e-31	28.8579129765114	1.96180195142205
32	350	nuclear division
GO:0007067	5.91476317426276e-31	28.8579129765114	1.96180195142205
32	350	mitosis
GO:0048285	3.55039377299656e-30	27.1117014853279	2.07390492007474
32	370	organelle fission
GO:0051301	4.47473727085696e-26	20.5424703087886	2.53352709155076

31	452	cell division
GO:0010564	9.39408493606754e-21	17.0352389878163	2.32053145111065
26	414	regulation of cell cycle process
GO:0007017	4.45654204773914e-20	16.9861218944099	2.20282333402533
25	393	microtubule-based process
GO:0007059	5.01608028890097e-20	33.367681498829 0.784720780568819	18
140	chromosome segregation
GO:0000226	2.09231203884606e-17	18.5937191388505	1.50778492837866
20	269	microtubule cytoskeleton organization
GO:0000236	2.5768957809755e-17	42.0824048770233	0.476437616773926
14	85	mitotic prometaphase
GO:0000075	1.01798615418588e-15	18.0433673469388	1.35644592069753
18	242	cell cycle checkpoint
GO:0007346	3.43616833472551e-15	15.19956837801	1.69835997508823	19
303	regulation of mitotic cell cycle
GO:0071156	4.69716445293593e-15	16.404181184669 1.47975918621549	18
264	regulation of cell cycle arrest
GO:0007051	1.82947026663233e-14	36.5780051150895	0.448411874610754
12	80	spindle organization
GO:0007050	1.48551154839294e-12	11.4114774114774	2.0682997716421 18
369	cell cycle arrest
GO:0007088	1.92177534431201e-12	29.169387755102 0.493253062071829	11
88	regulation of mitosis
GO:0051783	1.92177534431201e-12	29.169387755102 0.493253062071829	11
88	regulation of nuclear division
GO:0045786	2.50036875731423e-11	9.51312649164678	2.44944986506124
18	437	negative regulation of cell cycle
GO:0031577	6.91910766519952e-11	46.2078968573731	0.235416234170646
8	42	spindle checkpoint
GO:0000070	2.56165649153333e-10	38.300033411293 0.274652273199087	8
49	mitotic sister chromatid segregation
GO:0051329	2.5981955340301e-10	9.49828353464717	2.12435125596844
16	379	interphase of mitotic cell cycle
GO:0007093	2.98659727102064e-10	17.4845982142857	0.779115632136184
11	139	mitotic cell cycle checkpoint
GO:0051325	3.52951857411441e-10	9.28816089570807	2.16919244342952
16	387	interphase
GO:0000819	4.21721831072271e-10	35.681195516812 0.29146771849699	8
52	sister chromatid segregation
GO:0000086	4.37089679068325e-10	16.8213748657358	0.807141374299356
11	144	G2/M transition of mitotic cell cycle
GO:0007052	5.06895725025873e-10	52.1871101871102	0.184969898276936
7	33	mitotic spindle organization
GO:0090068	7.27458579217874e-10	15.9724489795918	0.846377413327797
11	151	positive regulation of cell cycle process
GO:0033043	2.34798653977427e-09	8.07674208144796	2.47187045879178
16	441	regulation of organelle organization
GO:0051983	3.19035394030749e-09	63.7866666666667	0.134523562383226
6	24	regulation of chromosome segregation
GO:0030071	3.6555912191931e-09	37.6644144144144	0.24102138260328
7	43	regulation of mitotic metaphase/anaphase transition
GO:0007091	5.10195485306002e-09	35.6770981507824	0.252231679468549
7	45	mitotic metaphase/anaphase transition
GO:0030261	1.37029512037098e-08	47.82	0.168154452979033	6	30
chromosome condensation
GO:0031145	1.9223468939349e-08	20.8876712328767	0.465227319908657
8	83	anaphase-promoting complex-dependent proteasomal ubiquitin-dependent
protein catabolic process
GO:0071173	3.04889055198969e-08	40.9771428571429	0.19057504670957
6	34	spindle assembly checkpoint
GO:0071174	4.37645745520425e-08	38.24	0.201785343574839	6	36
mitotic cell cycle spindle checkpoint
GO:0000910	5.16292071265058e-08	18.2019751513221	0.526883952667635

8 94 cytokinesis
GO:0050000 9.26031690678646e-08 59.0213815789474 0.117708117085323
5 21 chromosome localization
GO:0051303 9.26031690678646e-08 59.0213815789474 0.117708117085323
5 21 establishment of chromosome localization
GO:0034502 1.51789880148426e-07 52.4561403508772 0.128918413950592
5 23 protein localization to chromosome
GO:0051640 1.51824571807191e-07 12.6143617021277 0.840772264895163
9 150 organelle localization
GO:0006323 1.79885415492457e-07 12.3489583333333 0.857587710193066
9 153 DNA packaging
GO:0033205 5.21782332135302e-07 39.3256578947368 0.162549304546398
5 29 cell cycle cytokinesis
GO:0010389 6.23398208321624e-07 37.75 0.168154452979033 5 30
regulation of G2/M transition of mitotic cell cycle
GO:0007076 6.30228466653901e-07 82.8917748917749 0.0728669296242475
4 13 mitotic chromosome condensation
GO:0007094 1.02466434659373e-06 33.6983082706767 0.184969898276936
5 33 mitotic cell cycle spindle assembly checkpoint
GO:0010948 1.06582718270912e-06 15.1787124202855 0.538094249532904
7 96 negative regulation of cell cycle process
GO:0071103 1.07300540491188e-06 9.85416666666667 1.05937305376791
9 189 DNA conformation change
GO:0007018 1.25327483367243e-06 11.642608873441 0.795931077434088 8
142 microtubule-based movement
GO:0045841 1.38931399792082e-06 31.4473684210526 0.196180195142205
5 35 negative regulation of mitotic metaphase/anaphase transition
GO:0051656 1.5038836713339e-06 14.3663024726855 0.566119991696076
7 101 establishment of organelle localization
GO:0045787 1.60746686796036e-06 14.2140825035562 0.571725140128711
7 102 positive regulation of cell cycle
GO:0008608 2.06231748342511e-06 57.3706293706294 0.0952875233547851
4 17 attachment of spindle microtubules to kinetochore
GO:0045839 2.75499188362075e-06 26.9454887218045 0.224205937305377
5 40 negative regulation of mitosis
GO:0051784 2.75499188362075e-06 26.9454887218045 0.224205937305377
5 40 negative regulation of nuclear division
GO:0051313 3.33009793296381e-06 49.7142857142857 0.106497820220054
4 19 attachment of spindle microtubules to chromosome
GO:0034501 3.35230564627903e-06 184.192307692308 0.0336308905958065
3 6 protein localization to kinetochore
GO:0051439 4.78685095042972e-06 15.8866666666667 0.437201577745485
6 78 regulation of ubiquitin-protein ligase activity involved in mitotic
cell cycle
GO:0051488 5.84279665726836e-06 138.134615384615 0.0392360390284409
3 7 activation of anaphase-promoting complex activity
GO:0000079 6.87961998116735e-06 14.8498701298701 0.465227319908657
6 83 regulation of cyclin-dependent protein kinase activity
GO:0032886 1.17246328134062e-05 13.4447058823529 0.510068507369732
6 91 regulation of microtubule-based process
GO:0051438 1.5947979052682e-05 12.6933333333333 0.538094249532904
6 96 regulation of ubiquitin-protein ligase activity
Tissue: Skin_Sun_Exposed_Lower_leg=>Nerve_Tibial Type: asymmetric
SourceGene: TSC1(ENSG00000165699.6)
TargetGeneSet: C1QC C1QB RPS6KA1 PPCS FCGR1B FAM72D SEMA4A C1orf85
SLAMF8 CD84 ARHGAP30 TMEM81 OR2L3 GPN1 PKDCC ARHGAP25 GPX1
UBA7 STX18 TLR1 TLR6 LIAS DAPP1 ANKDD1B XRCC4 SLC25A46 SNX2
SLU7 RP11 DDX41 F13A1 LST1 AIF1 GPANK1 HSPA1B GPSM3 FGD2
CYB5R4 ZC3H12D C6orf97 MKRN1 MRPS33 DOK2 FAM49B SYK TRIM14 NDUFA8
FAM78A FCN1 ACBD5 PIK3AP1 COMMD9 MS4A6A LAMTOR1 SLCO2B1 VPS11 PTPN6
ARHGDIB PTPRO LRMP NR4A1 NCKAP1L TMEM194A ERP29 DENR LCP1
ARL11 NLRC3 NAGPA CORO1A DUS2L FANCA DBIL5P ADAP2 CDR2L TNFRSF11A
VPS4B EBI3 VAV1 STXBP2 C19orf40 POU2F2 SIGLEC10 CTD

LAIR1 SIRPG GINS1 HCK PLTP ITGB2 TXNRD2 C22orf25 BCR
SLC2A11 RAC2 C22orf40 TLR7 CYBB GPR34 WAS ARHGAP4
GOBPID Pvalue OddsRatio ExpCount Count Size Term
GO:0006909 1.56187313052746e-09 22.2701298701299 0.499896200954951
9 86 phagocytosis
GO:0050900 1.37690707673575e-08 10.2896652110626 1.40087191197841
12 241 leukocyte migration
GO:0042110 3.54707280255418e-08 8.41355979011323 1.85426614075151
13 319 T cell activation
GO:0002252 4.32196566570316e-08 7.5241935483871 2.24372015777455 14
386 immune effector process
GO:0046649 1.78450638055667e-07 6.65775656324582 2.51691924434295
14 433 lymphocyte activation
GO:0002250 2.12684226820067e-07 10.1919206440483 1.15092381150093
10 198 adaptive immune response
GO:0002263 5.56195940913319e-07 13.0453089244851 0.714967822296035
8 123 cell activation involved in immune response
GO:0002366 5.56195940913319e-07 13.0453089244851 0.714967822296035
8 123 leukocyte activation involved in immune response
GO:0060326 1.19580713977144e-06 11.7097039473684 0.790533527091551
8 136 cell chemotaxis
GO:0002253 1.20183616994373e-06 7.36629566210046 1.73801121029687
11 299 activation of immune response
GO:0050778 1.20317321759012e-06 6.63589015151515 2.11583973427444
12 364 positive regulation of immune response
GO:0002764 1.67589937093668e-06 8.02237111060641 1.44156113763753
10 248 immune response-regulating signaling pathway
GO:0050764 2.53995359159108e-06 27.4913693901036 0.220884367863816
5 38 regulation of phagocytosis
GO:0030595 3.62929556025758e-06 12.4676573426573 0.645214864023251
7 111 leukocyte chemotaxis
GO:0050865 4.53592914436223e-06 6.37006931836937 1.99377205729707
11 343 regulation of cell activation
GO:0050870 6.62202013862449e-06 9.17274782047142 0.99397965538717
8 171 positive regulation of T cell activation
GO:0002275 6.68814015747244e-06 22.1148502624267 0.267386340045672
5 46 myeloid cell activation involved in immune response
GO:0002886 8.64650380938989e-06 37.7578947368421 0.133693170022836
4 23 regulation of myeloid leukocyte mediated immunity
GO:0002757 8.70612819212225e-06 7.50849557522124 1.36599543284202
9 235 immune response-activating signal transduction
GO:0002460 1.00493545434512e-05 8.6364466078491 1.05210712061449 8
181 adaptive immune response based on somatic recombination of immune receptors built from immunoglobulin superfamily domains
GO:0050867 1.03118971907143e-05 7.34337662337662 1.39505916545568
9 240 positive regulation of cell activation
GO:0006954 1.38329207528784e-05 5.15444444444444 2.68548889350218
12 462 inflammatory response
GO:0001774 1.55193509925864e-05 88.6481481481482 0.0523147187045879
3 9 microglial cell activation
GO:0043312 1.55193509925864e-05 88.6481481481482 0.0523147187045879
3 9 neutrophil degranulation
GO:0002694 1.63789020496631e-05 6.10758668636482 1.86589163379697
10 321 regulation of leukocyte activation
Tissue: Skin_Sun_Exposed_Lower_leg=>Nerve_Tibial Type: asymmetric
SourceGene: WDR75(ENSG00000115368.5)
TargetGeneSet: RPL11 RP11 UBC RPL31 ASNSD1 RPL37A SCARNA6 RPL32
EXOSC7 FAM162A IFT80 RPL34 ODZ1 CTD RPL37 RPL10A RPL39P3 MRPS12 SSPO
RPL30 RPS6 RPL12 LCN2 RPS24 MIR1307 RPS13 EIF3M GANAB C11orf67
RPS25 PFDN5 RPL41 NACA RPS2P5 RPS29 RPL3P4 RPL4 CHD2 RPL17
RPL36 IFNGR2 C22orf32
GOBPID Pvalue OddsRatio ExpCount Count Size Term
GO:0006415 1.5680139250674e-34 270.410714285714 0.207805688187669

19	91	translational termination
GO:0006414	3.12909219563623e-33	226.169435215947	0.239775794062695
19	105	translational elongation
GO:0006614	3.12909219563623e-33	226.169435215947	0.239775794062695
19	105	SRP-dependent cotranslational protein targeting to membrane
GO:0006613	3.80892439741973e-33	223.554187192118	0.242059373053768
19	106	cotranslational protein targeting to membrane
GO:0045047	3.80892439741973e-33	223.554187192118	0.242059373053768
19	106	protein targeting to ER
GO:0072599	3.80892439741973e-33	223.554187192118	0.242059373053768
19	106	establishment of protein localization to endoplasmic reticulum
GO:0070972	3.47664674423231e-32	196.292207792208	0.269462320946647
19	118	protein localization to endoplasmic reticulum
GO:0006413	3.85299653365757e-32	166.503496503497	0.347104006643139
20	152	translational initiation
GO:0000184	4.13337274621525e-32	194.315714285714	0.271745899937721
19	119	nuclear-transcribed mRNA catabolic process, nonsense-mediated decay
GO:0000956	3.62877134318008e-31	147.330924109448	0.385924849491385
20	169	nuclear-transcribed mRNA catabolic process
GO:0006402	1.08080495844364e-30	138.851022395326	0.406477060411044
20	178	mRNA catabolic process
GO:0019080	5.18226236288466e-30	146.87987012987 0.344820427652066	19
151	viral genome expression
GO:0019083	5.18226236288466e-30	146.87987012987 0.344820427652066	19
151	viral transcription
GO:0006612	6.74979111260142e-30	144.667377398721	0.349387585634212
19	153	protein targeting to membrane
GO:0043624	6.74979111260142e-30	144.667377398721	0.349387585634212
19	153	cellular protein complex disassembly
GO:0043241	1.2862263367654e-29	139.414696813977	0.360805480589579
19	158	protein complex disassembly
GO:0006401	2.06927629276862e-29	118.361746361746	0.468133693170023
20	205	RNA catabolic process
GO:0034623	8.82139705076275e-29	124.883410138249	0.397342744446751
19	174	cellular macromolecular complex disassembly
GO:0032984	1.54922199540392e-28	120.938392857143	0.408760639402117
19	179	macromolecular complex disassembly
GO:0072594	2.05660090582034e-27	104.411969111969	0.46585011417895
19	204	establishment of protein localization to organelle
GO:0019058	2.36044711594199e-26	90.9413746630728	0.527506746937928
19	231	viral infectious cycle
GO:0022415	2.37057827528782e-25	79.834914048607 0.593730537679053	19
260	viral reproductive process
GO:0071845	1.31503124514101e-24	72.4816711590296	0.648536433464812
19	284	cellular component disassembly at cellular level
GO:0022411	1.72390005902751e-24	71.3836962294211	0.657670749429105
19	288	cellular component disassembly
GO:0016032	9.27379826671703e-21	43.9375 1.02989412497405	19	451
viral reproduction
GO:0033365	2.28597037358896e-20	41.7426054122089	1.08013286277766
19	473	protein localization to organelle
GO:0006605	2.37916861648841e-20	41.6478806907378	1.08241644176874
19	474	protein targeting
GO:0042274	8.6489391356826e-06	96.02	0.0411044218393191	3	18
ribosomal small subunit biogenesis
GO:0022613	1.03848533290597e-05	14.4079147640791	0.513805272991488
6	225	ribonucleoprotein complex biogenesis
GO:0071843	1.46423493164358e-05	13.5288507391512	0.545775378866514
6	239	cellular component biogenesis at cellular level
Tissue: Nerve_Tibial=>Skin_Sun_Exposed_Lower_leg	Type:	asymmetric
SourceGene:	ZNF426(ENSG00000130818.4)
TargetGeneSet:	WASF2	MAGOH	ENSA	BROX	GCC2	RPL37A	DCP1A	SEC62
SLC34A2 ADAMTS3 RP11	RPL39P3 TBP	GTF2IP1 RPS15AP24	ANP32B	FAU

RAB6A RPS3 SNRPF RPS2P5 RPS29 RPL3P4 HMGN2P5 C15orf41 CTD
SUZ12 NLE1 RPS16 RPS21 TCP10L NDUFB11 ZDHHC9

GOBPID	Pvalue	OddsRatio	ExpCount	Count	Size	Term
GO:0000184	3.10082702951292e-12	79.3846153846154	0.172929209051277	8	119	nuclear-transcribed mRNA catabolic process, nonsense-mediated decay
GO:0000956	5.2972725883944e-11	54.539894887721	0.245588540585427	8	169	nuclear-transcribed mRNA catabolic process
GO:0006402	8.03358941506834e-11	51.6199095022624	0.258667220261574	8	178	mRNA catabolic process
GO:0006613	9.99015863106583e-11	72.3787878787879	0.154037782852398	7	106	cotranslational protein targeting to membrane
GO:0072594	2.39006241070147e-10	44.690737833595	0.296450072659332	8	204	establishment of protein localization to organelle
GO:0006401	2.48511036922702e-10	44.4607575165951	0.297903259290015	8	205	RNA catabolic process
GO:0006612	1.33423773257923e-09	48.9178082191781	0.222337554494499	7	153	protein targeting to membrane
GO:0006415	2.65350413836654e-09	67.5058823529412	0.132239983392153	6	91	translational termination
GO:0006414	6.32691400295807e-09	57.9030303030303	0.152584596221715	6	105	translational elongation
GO:0006614	6.32691400295807e-09	57.9030303030303	0.152584596221715	6	105	SRP-dependent cotranslational protein targeting to membrane
GO:0045047	6.7005347479569e-09	57.32	0.154037782852398	6	106	protein targeting to ER
GO:0072599	6.7005347479569e-09	57.32	0.154037782852398	6	106	establishment of protein localization to endoplasmic reticulum
GO:0006605	8.43381150091961e-09	22.5241935483871	0.688810462943741	9	474	protein targeting
GO:0070972	1.28046562758319e-08	51.1357142857143	0.171476022420594	6	118	protein localization to endoplasmic reticulum
GO:0019080	5.61716063210416e-08	39.4068965517241	0.219431181233133	6	151	viral genome expression
GO:0019083	5.61716063210416e-08	39.4068965517241	0.219431181233133	6	151	viral transcription
GO:0006413	5.8427660726956e-08	39.1342465753425	0.220884367863816	6	152	translational initiation
GO:0043624	6.07580045226447e-08	38.865306122449	0.222337554494499	6	153	cellular protein complex disassembly
GO:0043241	7.35921978450444e-08	37.5736842105263	0.229603487647914	6	158	protein complex disassembly
GO:0034623	1.30566915846309e-07	33.9571428571429	0.252854473738842	6	174	cellular macromolecular complex disassembly
GO:0032984	1.54445931912642e-07	32.964161849711	0.260120406892257	6	179	macromolecular complex disassembly
GO:0033365	1.73630693240662e-07	18.4813895781638	0.687357276313058	8	473	protein localization to organelle
GO:0019058	6.9406123036348e-07	25.2533333333333	0.335686111687772	6	231	viral infectious cycle
GO:0022415	1.38494421022697e-06	22.3244094488189	0.377828523977579	6	260	viral reproductive process
GO:0016032	2.18983943464244e-06	15.75	0.655387170438032	7	451	viral reproduction
GO:0071845	2.31347185524326e-06	20.3625899280576	0.412705003113971	6	284	cellular component disassembly at cellular level
GO:0022411	2.50880477984474e-06	20.068085106383	0.418517749636703	6	288	cellular component disassembly

Tissue: Thyroid=>Adipose_Subcutaneous Type: asymmetric
SourceGene: C11orf94(ENSG00000234776.1)
TargetGeneSet: RPL11 RP4 RPS8 MAGOH RPL5 RWDD3 RP11 ATP5F1
HSD3BP2 SNRPE KIAA1383 IRF2BP2 RPS7 RPLP0P6 UBC SNRPG TPRKB
C2orf65 HAT1 EEF1B2 XRCC5 RPL37A RPL15 RPSA RPL14 LAMB2 RPL24 ATG3
RNF7 RPL22L1 DNAJC19 HNRPDL OSTC WDR70 CTD CWC27 TAF9 BTF3

HINT1 CTB SYNPO ZNF300 MRPL22 NUDCD2 RPL26L1 LYRM4 NOL7 TDP2
RPS10 RPL10A RPL39P3 MRPS12 PSMB1 SLC29A4 CBX3 ECD NAA38 SSBP1
RPS20 SNHG6 RPL7 C8orf59 RPL30 POLR2K KANK1 RPS6 NDUFB6 XPA
SEC61B FAM206A RPL12 RPL7A CDC123 RPS24 RPL13AP5 GSTO1 POLR2G FIBP
LAMTOR1 CLNS1A TMEM126A RPS25 PRR13 HNRNPA1 C12orf45 RPLP0
UNC119B HMGB1 F7 ERH COX16 RPSAP4 RPS3AP6 ANKDD1A RPLP1 CES2
DDX19B C17orf61-PLSCR3 PLSCR3 RPL26 RPS7P1 RPL23A SLC6A4 RPL19 RPL27
PSMG2 HNRNPA1P7 RPL17 NFIX RPS19 RPL13A RPL28 CHMP2A NAA20 DPM1
PFDN4 BRWD1-IT2 HIRA PRDX4 PDZD11 RPS23P8 CXorf26 RPL39 HPRT1
GOBPID Pvalue OddsRatio ExpCount Count Size Term
GO:0006414 4.58188832262933e-49 100.549295774648 0.741125181648329
34 105 translational elongation
GO:0006415 1.77973484293038e-47 110.721549636804 0.642308490761885
32 91 translational termination
GO:0006614 4.479817834146e-47 94.8351449275362 0.741125181648329
33 105 SRP-dependent cotranslational protein targeting to membrane
GO:0006613 6.47458769958061e-47 93.5294818344253 0.748183516711646
33 106 cotranslational protein targeting to membrane
GO:0045047 6.47458769958061e-47 93.5294818344253 0.748183516711646
33 106 protein targeting to ER
GO:0072599 6.47458769958061e-47 93.5294818344253 0.748183516711646
33 106 establishment of protein localization to endoplasmic reticulum
GO:0070972 3.9693343567914e-45 80.2578005115089 0.832883537471455
33 118 protein localization to endoplasmic reticulum
GO:0000184 5.46680105828962e-45 79.3190091001011 0.839941872534773
33 119 nuclear-transcribed mRNA catabolic process, nonsense-mediated decay
GO:0019080 6.33160869608941e-43 60.8205128205128 1.06580859456093
34 151 viral genome expression
GO:0019083 6.33160869608941e-43 60.8205128205128 1.06580859456093
34 151 viral transcription
GO:0006612 1.03896542454123e-42 59.7899159663866 1.07992526468756
34 153 protein targeting to membrane
GO:0000956 2.19268408537288e-39 49.9817774936061 1.19285862570064
33 169 nuclear-transcribed mRNA catabolic process
GO:0006413 2.67922162204485e-39 54.2057142857143 1.07286692962425
32 152 translational initiation
GO:0043624 3.3717066276433e-39 53.7539551357733 1.07992526468756
32 153 cellular protein complex disassembly
GO:0043241 1.03774293688346e-38 51.6027210884354 1.11521694000415
32 158 protein complex disassembly
GO:0006402 1.39576346096265e-38 46.8497751124438 1.2563836412705 33
178 mRNA catabolic process
GO:0072594 4.16417593864301e-38 41.7029411764706 1.43990035291675
34 204 establishment of protein localization to organelle
GO:0019058 9.72323431385238e-38 37.7212153518124 1.63047539962632
35 231 viral infectious cycle
GO:0034623 2.93436748613485e-37 45.7368209255533 1.22815030101723
32 174 cellular macromolecular complex disassembly
GO:0032984 7.76666320262037e-37 44.1655976676385 1.26344197633382
32 179 macromolecular complex disassembly
GO:0006401 2.02075855827984e-36 39.4203741152679 1.44695868798007
33 205 RNA catabolic process
GO:0022415 7.33747502724919e-36 32.792039800995 1.83516711646253 35
260 viral reproductive process
GO:0071845 1.43879461956619e-31 26.8626364108782 2.00456715798215
33 284 cellular component disassembly at cellular level
GO:0022411 2.30340311471369e-31 26.4337595907928 2.03280049823542
33 288 cellular component disassembly
GO:0016032 5.95522087315355e-30 19.159977703456 3.18330911355616 37
451 viral reproduction
GO:0006605 6.50904804752065e-28 17.3237858032379 3.34565082001246
36 474 protein targeting
GO:0033365 1.03401432052651e-26 16.5911878961358 3.33859248494914

35	473	protein localization to organelle
GO:0022613	1.34282864689106e-15	15.7162826061528	1.58812538924642
19	225	ribonucleoprotein complex biogenesis
GO:0071843	4.10075410879317e-15	14.7015881708653	1.68694208013286
19	239	cellular component biogenesis at cellular level
GO:0042254	1.25296005045935e-13	18.2900154400412	1.0516919244343 15
149	ribosome biogenesis
GO:0042274	2.11523361997678e-11	96.044019138756 0.127050031139714	7
18	ribosomal small subunit biogenesis
GO:0006364	1.61763612700701e-10	18.5295994328252	0.734066846585011
11	104	rRNA processing
GO:0016072	2.98620379108735e-10	17.3992673992674	0.776416856964916
11	110	rRNA metabolic process
GO:0042273	1.20855297190459e-08	105.611192930781	0.084700020759809
5	12	ribosomal large subunit biogenesis
GO:0034660	1.23136219132765e-08	8.26450855417645	2.01162549304546
14	285	ncRNA metabolic process
GO:0034470	1.61007550097505e-07	9.05634048491191	1.41166701266348
11	200	ncRNA processing
GO:0000377	1.73708903383027e-06	7.93086060062752	1.43990035291675
10	204	RNA splicing, via transesterification reactions with bulged adenosine as nucleophile
GO:0000398	1.73708903383027e-06	7.93086060062752	1.43990035291675
10	204	nuclear mRNA splicing, via spliceosome
GO:0000375	2.16127954638703e-06	7.72886169980336	1.47519202823334
10	209	RNA splicing, via transesterification reactions
GO:0022618	4.71284595855416e-06	11.941028708134 0.670541831015155	7
95	ribonucleoprotein complex assembly
GO:0071826	6.63361502968288e-06	11.2950764006791	0.705833506331742
7	100	ribonucleoprotein complex subunit organization
GO:0000028	6.72414408354161e-06	144.909090909091	0.0423500103799045
3	6	ribosomal small subunit assembly
GO:0000462	6.72414408354161e-06	144.909090909091	0.0423500103799045
3	6	maturation of SSU-rRNA from tricistronic rRNA transcript (SSU-rRNA, 5.8S rRNA, LSU-rRNA)
GO:0030490	1.17068313659372e-05	108.674242424242	0.0494083454432219
3	7	maturation of SSU-rRNA
Tissue: Thyroid=>Adipose_Subcutaneous	Type:	asymmetric
SourceGene:	C19orf43(ENSG00000123144.5)
TargetGeneSet:	PLEKHN1 PRKCZ	RP4	RP1	RPS8	CTB	ABCA4	RPS27
MEF2D	ZC3H11B UBC	EEF1B2	KCNE4	RPL29	GPR87	RP11	TP63	H3F3AP6
RPS3A	CTD	SKP1	RPS14	ZNF300	DSP	UBD	FTH1P5	SENP6	FOXK1	BZW2
IGFBP3	SLC26A4 EEF1D	RPL8	RPS6	RPL7A	BCCIP	RPL27A	SDHD	DDX6	NACA
RPL18AP3	RPL6	ZDHHC20 RPL21	EXOSC8	DNAJC3	RPL4	RPS15A	ESRP2
RPL13	GGT6	RPL26	RPL19	KRT35	NT5C	RPL36	HNRNPA1P10	NCCRP1
ZNF600	RPS5	SOGA1	TSHZ2	PLA2G3	RPL3	TNRC6B	WDR13	WDR45	POF1B
RPL39
GOBPID	Pvalue	OddsRatio	ExpCount	Count	Size	Term
GO:0006414	3.53396181174091e-35	114.687456445993	0.421424122898069
23	105	translational elongation
GO:0006415	1.41405068971443e-32	116.132432432432	0.365234239844993
21	91	translational termination
GO:0006614	3.94933669285342e-31	96.6824324324324	0.421424122898069
21	105	SRP-dependent cotranslational protein targeting to membrane
GO:0006613	4.91293861919361e-31	95.5383147853736	0.425437685973289
21	106	cotranslational protein targeting to membrane
GO:0045047	4.91293861919361e-31	95.5383147853736	0.425437685973289
21	106	protein targeting to ER
GO:0072599	4.91293861919361e-31	95.5383147853736	0.425437685973289
21	106	establishment of protein localization to endoplasmic reticulum
GO:0000956	4.85786966626846e-30	64.1254403131115	0.678292159712131
23	169	nuclear-transcribed mRNA catabolic process
GO:0070972	5.70866153285636e-30	83.6489272777933	0.473600442875926

21	118	protein localization to endoplasmic reticulum
GO:0000184	6.91487865576159e-30	82.7895752895753	0.47761400595114 5
21	119	nuclear-transcribed mRNA catabolic process, nonsense-mediated decay
GO:0006402	1.7043328369606e-29	60.3638709677419	0.714414227389108
23	178	mRNA catabolic process
GO:0006401	5.04637953237574e-28	51.3113029827316	0.82278043042004
23	205	RNA catabolic process
GO:0019080	1.44152805700065e-27	62.270893970894 0.606048024358176	21
151	viral genome expression
GO:0019083	1.44152805700065e-27	62.270893970894 0.606048024358176	21
151	viral transcription
GO:0006413	1.6684861258793e-27	61.7912110583866	0.610061587433396
21	152	translational initiation
GO:0006612	1.9291555246234e-27	61.3187960687961	0.614075150508615
21	153	protein targeting to membrane
GO:0043624	1.9291555246234e-27	61.3187960687961	0.614075150508615
21	153	cellular protein complex disassembly
GO:0043241	3.92632307364915e-27	59.0601696587098	0.634142965884714
21	158	protein complex disassembly
GO:0034623	3.26927523770176e-26	52.8245892951775	0.698359975088229
21	174	cellular macromolecular complex disassembly
GO:0032984	6.07215039653247e-26	51.134964078002 0.718427790464328	21
179	macromolecular complex disassembly
GO:0072594	1.03773523730116e-24	44.0717766947275	0.81876686734482
21	204	establishment of protein localization to organelle
GO:0019058	1.50115437852748e-23	38.3324324324324	0.927133070375753
21	231	viral infectious cycle
GO:0071845	3.762365063634e-23	32.9603477523325	1.1398519133624 22
284	cellular component disassembly at cellular level
GO:0022411	5.12908182491589e-23	32.4555137844612	1.15590616566328
22	288	cellular component disassembly
GO:0022415	1.86373894425895e-22	33.6123487504241	1.04352639955712
21	260	viral reproductive process
GO:0016032	8.98932556008988e-19	19.8917378917379	1.81011694692409
22	451	viral reproduction
GO:0033365	4.58332234219373e-17	17.50544128199	1.89841533457892	21
473	protein localization to organelle
GO:0006605	4.78372454065249e-17	17.4655450152139	1.90242889765414
21	474	protein targeting
GO:0022613	2.9915768230483e-06	10.4523502304147	0.903051691924434
8	225	ribonucleoprotein complex biogenesis
GO:0071843	4.67770726164057e-06	9.80917748917749	0.95924157497751
8	239	cellular component biogenesis at cellular level
GO:0007603	9.89560402546092e-06	98.0795454545455	0.0441491938274168
3	11	phototransduction, visible light
Tissue: Thyroid=>Adipose_Subcutaneous	Type:	asymmetric
SourceGene:	C6orf108(ENSG00000112667.8)
TargetGeneSet:	CLSTN1	HSPG2	PEF1	RPS8	RP4	CRYZ	CCBL2	RPL5	RP11
SRGAP2P2	JTB	RPS27	TKT	GORAB	PRRC2C	TPR	ZBTB41	FAM108A4P
RPS7	UBC	PRORSD1P	UGGT1	KLF7	BRK1	WDR48	LAMB2	APPL1
KCTD6	CCDC80	PHC3	DNAJC19 PPP1R2	BOD1L	MTHFD2L WDFY3-AS1	RPL34
RPS3A	CTD	SKP1	RPS14	HMGN4	HCG16	WDR11	IGF2R	STARD3NL
RPS3AP26	MTPN	LSM1	PPAPDC1B	HOOK3	PAG1	RNF19A	SMARCA2 RPS6
CDC26	RPL12	RPL7A	NUDT5	ANAPC16 RPS24	RPS3AP5 ARL3	EIF6	RPL27A
CKAP5	AHNAK	SLC22A25	NUMA1	TMEM126B	BACE1	UBE4A	CD163L1
SCAF11	LRP1	EEA1	ISCU	DNAJC3	CCNB1IP1	RPS27L	RPL4	IFT140
RPS15A	TERF2	RPL26	RPS7P1	RPL23A	TAOK1	RPL17	RPL27	RPS7P11 TNRC6C
CEP192	RPL12P4 EP300	C22orf32	MXRA5	RPS4X	RPL39
GOBPID	Pvalue	OddsRatio	ExpCount	Count	Size	Term
GO:0006415	2.10803275797202e-29	75.2789473684211	0.491177081170853
21	91	translational termination
GO:0006414	5.77875692373691e-28	62.6710526315789	0.566742785966369
21	105	translational elongation

GO:0006614	5.77875692373691e-28	62.6710526315789	0.566742785966369	21	105	SRP-dependent cotranslational protein targeting to membrane
GO:0006613	7.17914378665368e-28	61.9294117647059	0.572140336308906	21	106	cotranslational protein targeting to membrane
GO:0045047	7.17914378665368e-28	61.9294117647059	0.572140336308906	21	106	protein targeting to ER
GO:0072599	7.17914378665368e-28	61.9294117647059	0.572140336308906	21	106	establishment of protein localization to endoplasmic reticulum
GO:0070972	8.20948226222399e-27	54.2224633749322	0.636910940419348	21	118	protein localization to endoplasmic reticulum
GO:0000184	9.93085599893116e-27	53.6654135338346	0.642308490761885	21	119	nuclear-transcribed mRNA catabolic process, nonsense-mediated decay
GO:0019080	4.68301832880547e-26	43.3787375415282	0.815030101723064	22	151	viral genome expression
GO:0019083	4.68301832880547e-26	43.3787375415282	0.815030101723064	22	151	viral transcription
GO:0000956	6.23836773568815e-25	38.0189504373178	0.912186007888727	22	169	nuclear-transcribed mRNA catabolic process
GO:0006402	2.03651287174155e-24	35.8028846153846	0.960763960971559	22	178	mRNA catabolic process
GO:0006413	2.29291946577241e-24	40.053836882282	0.820427652065601	21	152	translational initiation
GO:0006612	2.647604090615e-24	39.7476076555024	0.825825202408138	21	153	protein targeting to membrane
GO:0043624	2.647604090615e-24	39.7476076555024	0.825825202408138	21	153	cellular protein complex disassembly
GO:0043241	5.3526565448962e-24	38.2835190165194	0.852812954120822	21	158	protein complex disassembly
GO:0034623	4.36256393431683e-23	34.2414860681115	0.939173759601412	21	174	cellular macromolecular complex disassembly
GO:0072594	4.43450340207823e-23	30.6320643642072	1.10110026987752	22	204	establishment of protein localization to organelle
GO:0006401	4.94897124682394e-23	30.4625292740047	1.10649782022005	22	205	RNA catabolic process
GO:0032984	8.04872623616844e-23	33.1462358427715	0.966161511314096	21	179	macromolecular complex disassembly
GO:0019058	7.11073970455692e-22	26.6240601503759	1.24683412912601	22	231	viral infectious cycle
GO:0033365	5.51543519836817e-21	16.5316539171722	2.55304131201993	27	473	protein localization to organelle
GO:0022415	9.68003615885008e-21	23.3320828331333	1.40336308905958	22	260	viral reproductive process
GO:0071845	6.67665909730729e-20	21.1588058887677	1.53290429728046	22	284	cellular component disassembly at cellular level
GO:0022411	9.0530347428169e-20	20.8347207303974	1.55449449865061	22	288	cellular component disassembly
GO:0006605	1.44216818696266e-18	14.6278942723873	2.55843886236247	25	474	protein targeting
GO:0016032	6.63950148420574e-18	14.5157429091855	2.43429520448412	24	451	viral reproduction
GO:0022613	3.17925908695173e-08	10.8626726182173	1.21444882707079	11	225	ribonucleoprotein complex biogenesis
GO:0042274	3.26261657760861e-08	75.6585879873551	0.0971559061656633	5	18	ribosomal small subunit biogenesis
GO:0071843	5.90216155929363e-08	10.1855852317361	1.29001453186631	11	239	cellular component biogenesis at cellular level
GO:0042254	1.0287403911049e-07	13.2605590062112	0.804235001037991	9	149	ribosome biogenesis
GO:0006364	1.4171483529461e-06	14.5102366777988	0.561345235623832	7	104	rRNA processing
GO:0016072	2.06869764851902e-06	13.6592369752496	0.593730537679053	7	110	rRNA metabolic process
GO:0030490	5.21301543614226e-06	143.69	0.0377828523977579	3	7	

maturation of SSU-rRNA
Tissue: Thyroid=>Adipose_Subcutaneous   Type: asymmetric
SourceGene: CTD-2034L19.1(ENSG00000248394.1)
TargetGeneSet: MTND1P23   MTND2P28   NMNAT1 DFFA   AIM1L   DNAJC8 RP11
RPS8   HBXIP   SPRR2D RPS27   CADM3   PFN1P1 GAS5   HMGN1P4 ST13P19 XDH
DGUOK-AS1   EIF5B   UGGT1   RHOQP3 HAT1   H3F3AP4 CASP10 TRANK1 RPL29
METTL15P1   SETD7   H3F3AP6 RPS3A   C5orf15 SKP1   CTB   MTND5P11
RPL15P3 PPIAP29 AGPAT1 FTH1P5 MTRNR2L9   KIAA1919   FOXK1   EEF1A1P6
RPS3AP26   POLR2J ARF5   CTAGE8 HMBOX1 HOOK3   GS1   ATP6V1G1
RPL7A   FUT11   EIF2S2P3   MRPL43 GLRX3   MTRNR2L8   TSG101 C11orf94
ALDH3B2 DDX6   BARX2   KRT6B   NACA   RPL6   COX6A1 SBNO1   GOLGA3 MED4-AS1
NDFIP2 PSMA6   TJP1   HERC2P10   C15orf40   CTD   MPG   HBQ1
SPSB3   RPS15A SULT1A4 NFATC3 VPS53   TLCD2   RPL26   C17orf48   TAF15
RPL19   RPS7P11 PRR15L RPS2P48 ROCK1P1 MYL12B RPL17   HMSD   HNRNPA1P10
SLC35E1 LGALS7 RPL18   RPL13A KLK7   RPS5   RPL41P1 KIF3B   RP4   RPL12P4
HMGB1P10   PRR14L RPL3   RBX1   USP9X   RP6   RPS4X   TTC3P1 RP1
HCFC1   RPL10   MT
GOBPID Pvalue OddsRatio   ExpCount   Count   Size   Term
GO:0006415   4.75045189170174e-21   54.4914285714286   0.453394228773095
16   91   translational termination
GO:0006414   5.42406537022357e-20   45.8747993579454   0.523147187045879
16   105   translational elongation
GO:0006614   5.42406537022357e-20   45.8747993579454   0.523147187045879
16   105   SRP-dependent cotranslational protein targeting to membrane
GO:0006613   6.36490887575167e-20   45.3619047619048   0.528129541208221
16   106   cotranslational protein targeting to membrane
GO:0045047   6.36490887575167e-20   45.3619047619048   0.528129541208221
16   106   protein targeting to ER
GO:0072599   6.36490887575167e-20   45.3619047619048   0.528129541208221
16   106   establishment of protein localization to endoplasmic reticulum
GO:0070972   3.84607719690325e-19   39.9915966386555   0.587917791156321
16   118   protein localization to endoplasmic reticulum
GO:0000184   4.42721450880039e-19   39.6005547850208   0.592900145318663
16   119   nuclear-transcribed mRNA catabolic process, nonsense-mediated decay
GO:0019080   6.98033579785191e-19   32.8582089552239   0.752335478513598
17   151   viral genome expression
GO:0019083   6.98033579785191e-19   32.8582089552239   0.752335478513598
17   151   viral transcription
GO:0006413   7.83085074124861e-19   32.6125252525253   0.757317832675939
17   152   translational initiation
GO:0022415   8.22254859659035e-19   22.6586538461538   1.29541208220884
20   260   viral reproductive process
GO:0019058   1.96121604843618e-18   23.9563011747953   1.15092381150093
19   231   viral infectious cycle
GO:0000956   4.9171950353796e-18   28.9305023923445   0.842017853435748
17   169   nuclear-transcribed mRNA catabolic process
GO:0006402   1.20074409114895e-17   27.2959909655562   0.886859040896824
17   178   mRNA catabolic process
GO:0016032   1.27258931473331e-17   15.3000667556742   2.24704172721611
23   451   viral reproduction
GO:0006612   2.7774367925768e-17   29.7017726798749   0.762300186838281
16   153   protein targeting to membrane
GO:0043624   2.7774367925768e-17   29.7017726798749   0.762300186838281
16   153   cellular protein complex disassembly
GO:0043241   4.68432741617164e-17   28.6458752515091   0.78721195764999
16   158   protein complex disassembly
GO:0006401   1.33614007288617e-16   23.3314313346228   1.02138260328005
17   205   RNA catabolic process
GO:0034623   2.22674282394987e-16   25.7160940325497   0.866929624247457
16   174   cellular macromolecular complex disassembly
GO:0032984   3.510842773563e-16   24.9184925503944   0.891841395059165
16   179   macromolecular complex disassembly
GO:0071845   1.78371966509068e-15   17.6854636591479   1.41498858210504

18	284	cellular component disassembly at cellular level
GO:0022411	2.27923242445049e-15	17.4185185185185	1.43491799875441
18	288	cellular component disassembly
GO:0072594	2.82815523203863e-15	21.5668693009119	1.01640024911771
16	204	establishment of protein localization to organelle
GO:0033365	1.10032896569639e-10	9.43744019138756	2.35665351878763
17	473	protein localization to organelle
GO:0006605	1.05853921576791e-09	8.68434185901435	2.36163587294997
16	474	protein targeting
Tissue: Adipose_Subcutaneous=>Thyroid	Type:	asymmetric
SourceGene:	ENSG00000179935.5
TargetGeneSet:	NOL9	DNAJC11	DNAJC16	TCEB3	ARID1A	NUDC	SNRNP40	TXLNA
ADPRHL2	GNL2	RP11	IPO13	POMGNT1	C1orf123	WDR3	GOLPH3L	ADAR
PYGO2	FLAD1	CCT3	METTL13	STX6	DHX9	RBBP5	FBXO28	ACBD3	LIN9	URB2
HEATR1	HNRNPU	TAF1B	PREB	SLC5A6	EIF2B4	PPM1G	ASS1P2	PNPT1	PAPOLG	CCT7
POLR1A	RPIA	LMAN2L	POLR1B	AMMECR1L	CREB1	ATIC	CLN5	ANO7
ZDHHC3	SCAP	SLC52A1	ACTR8	SHQ1	QTRTD1	TSC22D2	ACTL6A	ABCF3	ALG3
PSMD2	LETM1	FAM193A	GRPEL1	ANKRD17	SDAD1	CCT5	MARCH6	NUP155	SKIV2L2
MIER3	IPO11	C5orf48	HARS	TSPAN17	ZSCAN12P1	TRIM27	C6orf25	VARS
ZBTB9	BYSL	PPP2R5D	MCM3	RP3	LATS1	MRPL18	EIF3B	C7orf26	BAZ1B
MEPCE	METTL2A	TNPO3	CASP2	ZNF398	ABCF2	NEIL2	POLR3D	ELP3	INTS9	TTI2
TMEM68	SMU1	NOL6	ALG2	PAPPA	NUP188	EXOSC2	TPRN	NGB	BMS1
HNRNPF	ZFAND4	SEC24C	POLR3A	PDCD11	CTR9	NAT10	SSRP1	CCDC86	MTA2
GANAB	C11orf84	MARK2	STIP1	MRPL11	ANKRD42	DDX10	ZNF259	DDX6
TRAPPC4	FKBP4	RP4	DDX23	TARBP2	SUOX	OBFC2B	CCT2	UTP20	RNF34
DDX55	PUS1	PRMT5-AS1	PRMT5	AHSA1	PPP1R14D	TUBGCP4	GABPB1
RNF111	DIS3L	ASB7	SOLH	DNAJA3	GLYR1	CTD	CD2BP2	TBC1D10B
SALL1	TMCO7	CIRH1A	EXOSC6	SF3B3	DHODH	ZNF778	TSR1	TP53	ELAC2
TMEM11	TMEM199	ERAL1	UTP6	PSME3	TMEM101	EFTUD2	UBE2Z	DLX3	PPP1R9B
APPBP2	TACO1	FTSJ3	SRP68	CANT1	EIF4A3	MBD1	MOB3A	ELAVL1	HNRNPM
SYCE2	DNAJB1	MPV17L2	ZNF526	GRWD1	PRMT1	FIZ1	ZNF264	ZNF749	ZNF549
ZNF586	ZSCAN22	FAM110A	NOP56	CSRP2BP	SYS1	NCOA5	ZFP64	URB1	GART	CRKL
MORC2	PRR14L	ADSL	XPNPEP3	L3MBTL2	RRP7A	BCOR	GPKOW	SMC1A	FAM123B
TCEAL3-AS1	UTP14A
GOBPID	Pvalue	OddsRatio	ExpCount	Count	Size	Term
GO:0071843	1.73703127267487e-19	12.1937862032649	2.99349525984361
28	239	cellular component biogenesis at cellular level
GO:0022613	4.32132962375617e-19	12.4604486422668	2.81814407307453
27	225	ribonucleoprotein complex biogenesis
GO:0042254	1.69362791843912e-16	14.50107421875	1.8662376306138	21	149
ribosome biogenesis
GO:0034660	1.42909505026306e-13	8.20499304487883	3.56964915922774
24	285	ncRNA metabolic process
GO:0016072	6.8172733156039e-12	13.4828788839569	1.37775932461421
15	110	rRNA metabolic process
GO:0034470	6.97407048258037e-12	9.12935679694427	2.50501695384402
19	200	ncRNA processing
GO:0006364	4.20316621408951e-11	13.208250166334	1.30260881599889	14
104	rRNA processing
GO:0000377	2.80058731469138e-07	6.21241727072171	2.5551172929209	14
204	RNA splicing, via transesterification reactions with bulged adenosine as nucleophile
GO:0000398	2.80058731469138e-07	6.21241727072171	2.5551172929209	14
204	nuclear mRNA splicing, via spliceosome
GO:0022618	2.98387086649268e-07	9.75920192638459	1.18988305307591
10	95	ribonucleoprotein complex assembly
GO:0000375	3.76452682895004e-07	6.05097497313066	2.61774271676701
14	209	RNA splicing, via transesterification reactions
GO:0071826	4.84306528829035e-07	9.21377517868746	1.25250847692201
10	100	ribonucleoprotein complex subunit organization
GO:0006397	2.73444066408918e-06	4.02560262789237	4.99750882291883
18	399	mRNA processing
GO:0006457	3.29274085052668e-06	5.36215693173821	2.70541831015155

13	216	protein folding
GO:0006354	8.38605689660854e-06	7.4899577167019 1.35270915507577	9
108	DNA-dependent transcription, elongation
GO:0008380	1.36693574880991e-05	4.09618995462369	4.0456023804581 15
323	RNA splicing
Tissue: Thyroid=>Adipose_Subcutaneous	Type:	asymmetric
SourceGene:	ENSG00000239948.1
TargetGeneSet:	MTND2P28	RPL22	RPL11	ARID1A	KPNA7	RP1	RPS8
RPS15AP10	GTF2B	RPL5	RP11	RPS27	MTX1	UBC	EIF5B	MZT2B	BRK1
RPL15	RNF123	RPL29	DCP1A	PCNP	RPL24	FAM162A DNAJC19 ZBTB12B RPL34
RPS3A	FAM160A1	SKP1	CTB	SLC25A2 RPS14	RPL15P3 NOL7	RPS10
RPL10A	YIPF3	MTRNR2L9	RPL39P3 HSF2	MRPS12	EEF1A1P6	BAZ1B
RPS3AP26	TRIM56	HMBOX1	RPS20	MED30	RPS6	DCTN3	SETX	RPL7A
RPS24	PGAM1	RPL27A	BARX2	SLC38A4 PFDN5	NACA	STAT6	MRS2P2	RPL18AP3
MLXIP	ZC3H13	MBIP	SIVA1	RPL9P25 RPL4	C15orf40	SEC11A	RPS15A
RPL26	RPL23A	RPL17	RPL19	RPL27	SNORA76 ATF4P3	ROCK1P1 C18orf32
RPS15	ZBTB7A	RPL36	CTD	BRD4	RPSAP58 BCKDHA	RPL18	RPS5	RPL41P1
SOGA1	DPM1	HMGB1P10	RPL3	RP4	RP6	UXT	RPS4X	RPL10	MT
GOBPID	Pvalue	OddsRatio	ExpCount	Count	Size	Term
GO:0006415	1.67042444738663e-54	185.120689655172	0.48487993910456
33	91	translational termination
GO:0006614	3.20328827974753e-54	159.286603340976	0.559476852812954
34	105	SRP-dependent cotranslational protein targeting to membrane
GO:0006613	4.70222493290163e-54	157.06330749354 0.564805203792125	34
106	cotranslational protein targeting to membrane
GO:0045047	4.70222493290163e-54	157.06330749354 0.564805203792125	34
106	protein targeting to ER
GO:0072599	4.70222493290163e-54	157.06330749354 0.564805203792125	34
106	establishment of protein localization to endoplasmic reticulum
GO:0070972	3.428401168231e-52	134.512735326689	0.628745415542177
34	118	protein localization to endoplasmic reticulum
GO:0000184	4.78577855377913e-52	132.920930232558	0.634073766521348
34	119	nuclear-transcribed mRNA catabolic process, nonsense-mediated decay
GO:0006414	4.91924770692221e-52	148.979166666667	0.559476852812954
33	105	translational elongation
GO:0006413	6.14796341299922e-48	95.5270003941663	0.809909348833991
34	152	translational initiation
GO:0006612	7.88144496579286e-48	94.7176079734219	0.815237699813162
34	153	protein targeting to membrane
GO:0034623	1.18682145954065e-47	85.341726618705 0.927133070375753	35
174	cellular macromolecular complex disassembly
GO:0032984	3.52072157385659e-47	82.349537037037 0.953774825271608	35
179	macromolecular complex disassembly
GO:0072594	7.0207651895783e-47	74.2473867595819	1.08698359975088
36	204	establishment of protein localization to organelle
GO:0000956	3.31579657706314e-46	83.398105081826 0.900491315479898	34
169	nuclear-transcribed mRNA catabolic process
GO:0019080	4.47185534298828e-46	90.6101694915254	0.80458099785482
33	151	viral genome expression
GO:0019083	4.47185534298828e-46	90.6101694915254	0.80458099785482
33	151	viral transcription
GO:0043624	7.23932442857183e-46	89.0875 0.815237699813162	33	153
cellular protein complex disassembly
GO:0006402	2.28648566589893e-45	78.1363049095607	0.948446474292437
34	178	mRNA catabolic process
GO:0043241	2.34076064577948e-45	85.494	0.841879454709017	33	158
protein complex disassembly
GO:0006401	4.11909201221411e-43	65.6741466068272	1.09231195073005
34	205	RNA catabolic process
GO:0071845	1.06961756697119e-39	47.2724230254351	1.51325167808456
35	284	cellular component disassembly at cellular level
GO:0019058	1.7280090696582e-39	53.6969696969697	1.2308490761885 33
231	viral infectious cycle

GO:0022411 1.78061290578568e-39 46.5118577075099 1.53456508200125
35 288 cellular component disassembly
GO:0022415 1.03803681237724e-37 46.7411894273128 1.38537125458446
33 260 viral reproductive process
GO:0033365 1.09028316365855e-34 29.5702981651376 2.52031001314788
37 473 protein localization to organelle
GO:0006605 1.17930594278941e-34 29.5005148741419 2.52563836412705
37 474 protein targeting
GO:0016032 1.62166457839337e-32 27.9607371794872 2.40308629160612
35 451 viral reproduction
GO:0042274 3.05577230658442e-08 76.7147435897436 0.0959103176250778
5 18 ribosomal small subunit biogenesis
GO:0006364 7.65036914976698e-08 17.243961352657 0.554148501833783 8
104 rRNA processing
GO:0042254 9.18032191699592e-08 13.456512605042 0.793924295896478 9
149 ribosome biogenesis
GO:0016072 1.18618743017287e-07 16.2227905655016 0.586118607708809
8 110 rRNA metabolic process
GO:0022613 3.02358418324044e-07 9.82922596320722 1.19887897031347
10 225 ribonucleoprotein complex biogenesis
GO:0071843 5.27308797462763e-07 9.21918790327837 1.27347588402187
10 239 cellular component biogenesis at cellular level
GO:0030490 5.01355906814168e-06 145.641891891892 0.0372984568541969
3 7 maturation of SSU-rRNA
GO:0034470 1.08732419864739e-05 8.56400966183575 1.0656701958342 8
200 ncRNA processing
Tissue: Thyroid=>Adipose_Subcutaneous Type: asymmetric
SourceGene: FSCN3(ENSG00000106328.8)
TargetGeneSet: NMNAT1 DFFA SPEN RPL11 KPNA7 TXLNA RP3 RP1
RABGGTB RP4 RP11 HBXIP ATP5F1 HMGCS2 SRGAP2P1 RPRD2 LCE2B
RPS27 PYGO2 MEF2D ISG20L2 GORAB TTC32 RHOQ GPR75 UBC TIA1
DGUOK COX5B SLC9A2 SPOPL KCNE4 MAP4 RPL29 APPL1 PDCD10 RPL22L1
DNAJC19 RPL34 SETD7 WWC2 FRG1 RICTOR GRAMD3 HINT1 CTB RPS14
SYNPO ZNF300 C5orf25 GPLD1 PPP1R18 RPS10 C6orf162 TRMT11 URGCP
BAZ1B CLDN3 GTPBP10 TRIM56 NAA38 ZNF862 DCTN6 RPS20 SNHG6 RPL30
POLR2K MED30 TG LY6D EEF1D RPL8 RPS6 TUSC1 NDUFB6 CALML5
VDAC2 RPS24 SNCG AS3MT C10orf118 PIDD EIF3F RPL27A SPTY2D1 EXT2
NDUFS3 MRPL16 ALDH3B2 RSF1 TMEM126A SDHD BACE1 DDX6 KRT80
PFDN5 NACA AVIL SSH1 RPL21 MED4-AS1 PHF11 METTL17 PSME1
PSME2 OIP5-AS1 RPS3AP6 C15orf40 ECI1 RPS15A CES2 APRT
VPS53 TLCD2 KDM6B TMEM107 RPL26 TOM1L2 SUPT6H NUFIP2 RPL19 WIPF2 RARA
RPL27 UBTF ERN1 TNRC6C STRA13 CTD RPS15 OAZ1 RPL36 BRD4
UQCRFS1 KCNK6 RPL18 ZNF264 ZSCAN22 PCNA KIF3B SOGA1 ZNFX1 B4GALT5
PFDN4 ATP5O ICOSLG MPST STS BCOR NDUFB11 UXT RPS4X RPL39
BCORL1 MT
GOBPID Pvalue OddsRatio ExpCount Count Size Term
GO:0006414 1.72261507924495e-27 39.429560401523 0.951837243097364 24
105 translational elongation
GO:0006415 1.76542062947188e-27 44.6345315904139 0.824925610684382
23 91 translational termination
GO:0006614 6.65790942945432e-26 36.9776422764228 0.951837243097364
23 105 SRP-dependent cotranslational protein targeting to membrane
GO:0006613 8.44160722142093e-26 36.5295626952253 0.960902359698291
23 106 cotranslational protein targeting to membrane
GO:0045047 8.44160722142093e-26 36.5295626952253 0.960902359698291
23 106 protein targeting to ER
GO:0072599 8.44160722142093e-26 36.5295626952253 0.960902359698291
23 106 establishment of protein localization to endoplasmic reticulum
GO:0019080 7.26053316034125e-25 26.568583408206 1.36883260674002 25
151 viral genome expression
GO:0019083 7.26053316034125e-25 26.568583408206 1.36883260674002 25
151 viral transcription
GO:0006413 8.62794227092081e-25 26.3575248848611 1.37789772334095

25	152	translational initiation
GO:0070972	1.20810811690048e-24	31.8884015594542	1.06968375890942
23	118	protein localization to endoplasmic reticulum
GO:0000184	1.4867530081339e-24	31.554012345679 1.07874887551035	23
119	nuclear-transcribed mRNA catabolic process, nonsense-mediated decay
GO:0043624	2.63788859273469e-23	24.6746359487068	1.38696283994187
24	153	cellular protein complex disassembly
GO:0034623	2.85741956529368e-23	22.4309864505508	1.57733028856135
25	174	cellular macromolecular complex disassembly
GO:0043241	5.86700946048458e-23	23.7455712093737	1.43228842294651
24	158	protein complex disassembly
GO:0032984	5.90363735413787e-23	21.6950502327861	1.62265587156598
25	179	macromolecular complex disassembly
GO:0000956	3.09189014661989e-22	21.9271672574927	1.53200470555671
24	169	nuclear-transcribed mRNA catabolic process
GO:0006612	6.41441462649512e-22	23.2457264957265	1.38696283994187
23	153	protein targeting to membrane
GO:0006402	1.10239174416829e-21	20.632601043816 1.61359075496505	24
178	mRNA catabolic process
GO:0019058	2.13772651322195e-21	17.0494773519164	2.0940419348142 26
231	viral infectious cycle
GO:0072594	2.98938399073077e-20	17.619937694704 1.84928378658916	24
204	establishment of protein localization to organelle
GO:0006401	3.36142128074374e-20	17.5213507512779	1.85834890319009
24	205	RNA catabolic process
GO:0022415	4.45590464750292e-20	14.9058201058201	2.35693031624109
26	260	viral reproductive process
GO:0071845	4.17372903084162e-19	13.4961978589886	2.57449311466334
26	284	cellular component disassembly at cellular level
GO:0022411	5.93462448425613e-19	13.2863685932388	2.61075358106705
26	288	cellular component disassembly
GO:0016032	4.70061871788295e-17	9.36348852337143	4.0883675870182 29
451	viral reproduction
GO:0033365	7.65497108572481e-13	7.30289757412399	4.2878001522386 25
473	protein localization to organelle
GO:0006605	8.02267033340791e-13	7.28610749254108	4.29686526883953
25	474	protein targeting
GO:0022904	2.499126848136e-06	10.5188470066519	0.870251193689018
8	96	respiratory electron transport chain
GO:0022613	6.29876814588043e-06	6.04228971962617	2.03965123520864
11	225	ribonucleoprotein complex biogenesis
GO:0042254	8.39393584807817e-06	7.47189695550351	1.35070237353816
9	149	ribosome biogenesis
GO:0071843	1.11563267010338e-05	5.6656432748538 2.16656286762162	11
239	cellular component biogenesis at cellular level
GO:0042274	1.7884689744967e-05	32.1844769403825	0.163172098816691
4	18	ribosomal small subunit biogenesis
Tissue: Adipose_Subcutaneous=>Thyroid	Type:	asymmetric
SourceGene:	OSBP(ENSG00000110048.6)
TargetGeneSet:	NOL9	DFFA	DNAJC16	SPEN	KIAA0090	ALPL	TCEB3	NUDC
GPR3	EYA3	GMEB1	SNRNP40	KPNA7	TMEM234	TXLNA	EIF2C3	THRAP3	FHL3
IPO13	DPH2	TOE1	CCDC17	RPS15AP10	RP11	ST7L	WDR3	PIAS3
GOLPH3L	ADAR	PYGO2	CCT3	METTL13	URB2	HEATR1	KIF3C	EIF2B4	WDR43	MSH6
CCT4	TCEB1P21	POLR1A	ZNF2	LMAN2L	POU3F3	POLR1B	MYO7B	RBP4
AMMECR1L	CLN5	C3orf19	CMC1	MYD88	SCN5A	IP6K1	RRP9	ACTR8	GMPS
SH3BP2	NOP14	SRP72	NUP155	SKIV2L2	HSPA4	HARS	MFAP3	HRH2	NOP16	C4B
PFDN6	ZBTB9	BYSL	TAF8	RPL7L1	POLR1C	TRAM2	EIF3B	C7orf26	CCT6A	TBL2
POM121C	ZKSCAN5	ZKSCAN1	MEPCE	AGFG2	KLHDC10	CNOT4	ABCF2	NOM1	NEIL2	XPO7
POLR3D	ELP3	EXTL3	LSM12P1	PRKDC	SULF1	ZNF706	BAALC	PLAA	DNAJA1	NOL6
STOML2	TBC1D13	NUP188	EXOSC2	GTF3C4	QSOX2	NGB	BMS1	HNRNPF	FAM35B
POLR3A	SFTPD	ENTPD7	CHUK	C10orf2	BTRC	PPRC1	NOLC1	PDCD11	CTR9
SPTY2D1	CAPRIN1	NAT10	CTD	HARBI1	OSBP	MTA2	GNG3	STX5	WDR74
C11orf95	MARK2	STIP1	DPF2	U4	IL18BP	ANKRD42	MRE11A	ZNF259	DDX6

ZNF202 FKBP4 ZCRB1 SCAF11 DDX23 PA2G4 OBFC2B KIF5A CAND1 KRT8P22
SART3 ATXN2 GCN1L1 HSPH1 NUFIP1 RBM23 PRMT5-AS1 PRMT5 RNF31 RP4
NEK9 CYP46A1 C15orf57 TUBGCP4 MFAP1 DUOXA1 DUOX1 PIGB CSNK1G1 ULK3
CHRNA5 C16orf73 THUMPD1 PPP4C CD2BP2 ZNF785 ZNF629 DNAJA2 GFOD2
CIRH1A DHODH VPS53 TSR1 TP53 TMEM199 CTB TP53I13 GOSR1 UTP6 NLE1
PSME3 AOC2 DHX8 EFTUD2 GFAP SNX11 UBE2Z VEZF1 APPBP2 FTSJ3
PSMC5 CEP76 ZNF24 FAM108A1 ZNF77 TNFSF14 ZNF121 ZNF562 SYCE2
DNAJB1 ZNF790 ZNF585B FBL KCNJ14 JOSD2 ZNF613 ZNF614 ZNF347 ZNF542
ZNF582 ZNF543 ZNF17 ZNF749 ZNF587 ZNF417 ZNF8 NOP56 AHCY SRSF6
RIMS4 NCOA5 GTPBP5 C20orf11 GART COL18A1 ZNF70 BCRP3 TFIP11
TTC28 NF2 PES1 C22orf23 ADSL XPNPEP3 ZNF81 PQBP1 GNL3L
FRMPD3 UTP14A
GOBPID Pvalue OddsRatio ExpCount Count Size Term
GO:0071843 3.85252355044714e-22 12.39983436853 3.42350010379905 32
239 cellular component biogenesis at cellular level
GO:0022613 7.56635125155855e-22 12.7562675726336 3.22296034876479
31 225 ribonucleoprotein complex biogenesis
GO:0042254 2.02701796015587e-16 13.2187273888061 2.13431596429313
22 149 ribosome biogenesis
GO:0006364 1.53815483121709e-12 13.4754878629224 1.48972389454017
16 104 rRNA processing
GO:0016072 3.75266354500608e-12 12.6100033418737 1.57566950384056
16 110 rRNA metabolic process
GO:0034470 2.93591071729606e-08 6.40109264739358 2.86485364334648
16 200 ncRNA processing
GO:0022618 1.08997984511789e-07 9.46064139941691 1.36080548058958
11 95 ribonucleoprotein complex assembly
GO:0034660 1.49143407056048e-07 4.98555377207063 4.08241644176874
18 285 ncRNA metabolic process
GO:0071826 1.85738005062477e-07 8.92599174501261 1.43242682167324
11 100 ribonucleoprotein complex subunit organization
GO:0006397 4.77018739622153e-06 3.68723404255319 5.71538301847623
19 399 mRNA processing
GO:0000377 7.67200425713932e-06 4.93034490203487 2.92215071621341
13 204 RNA splicing, via transesterification reactions with bulged
adenosine as nucleophile
GO:0000398 7.67200425713932e-06 4.93034490203487 2.92215071621341
13 204 nuclear mRNA splicing, via spliceosome
GO:0000375 9.96691757170118e-06 4.80286135072586 2.99377205729707
13 209 RNA splicing, via transesterification reactions
GO:0000460 1.85010637569267e-05 35.064039408867 0.171891218600789 4
12 maturation of 5.8S rRNA
Tissue: Thyroid=>Adipose_Subcutaneous Type: asymmetric
SourceGene: PRINS(ENSG00000238115.1)
TargetGeneSet: MTND1P23 MTND2P28 RPL22 NMNAT1 UBE4B DFFA SPEN
LDLRAD2 HSPG2 RPL11 ARID1A WASF2 DNAJC8 EIF2C1 THRAP3 MFI2 RPS8
NEDD4 RP11 RPL5 HBXIP NUDT4P1 HIST2H2AC RPRD2 JTB RPS27
ASH1L MIR555 PIGM TKT PRRC2C ZBTB37 CEP350 ARPC5 PTPN14 ZC3H11B ADI1
RPS7 ASXL2 C2orf18 CALM2 RPL36AP15 UBC USP34 COMMD1 C1D AAK1
DGUOK-AS1 TET3 DCTN1 VAMP5 TMEM131 EIF5B UGGT1 MZT2B RHOQP3
H3F3AP4 ANKRD44 RAPH1 TRIP12 PSMD1 GIGYF2 HDLBP NR2C2 UBE2E2 RPL15
TRANK1 WDR48 HIGD1A MAP4 RAD54L2 RPL29 DCP1A NIT2 PCNP RPL24
FAM162A NME9 COMMD2 PDCD10 PHC3 PEX5L DNAJC19 HTT BOD1L RELL1
DANCR LAMTOR3 RPL34 SETD7 H3F3AP6 RPS3A MARCH6 NIPBL CTD SREK1IP1
MRPS36 TAF9 SERINC5 ERAP1 HINT1 C5orf15 SKP1 CTB MTND5P11
CDC23 RPS14 ATP6V0E1 GNB2L1 TXNDC5 TMEM14C RPL15P3 NOL7 PPIAP29
ZSCAN23 SYNGAP1 MNF1 RP1 UHRF1BP1 RPL10A MTRNR2L9 WDR11
BCKDHB C6orf162 KIAA1919 PHACTR2 IGF2R SFT2D1 FOXK1 EEF1A1P6
MPLKIP CHCHD2 DPY19L2P4 RPS3AP26 TRIM56 PIK3CG COG5 CAPZA2
METTL2A ZC3HAV1L HIPK2 MLL3 DRP2 HMBOX1 DCTN6 LSM1 HOOK3
RPS20 POLR2K UBR5 EIF3E MED30 TG RGP1 CLTA CBWD6 CBWD3
ATP6V1G1 GAPVD1 RPL12 NUP214 PRRC2B SETX RPL7A PRINS FUT11
RPS24 RPS3AP5 EIF6 RPL27A MTRNR2L8 TSG101 TRIM44 CKAP5 TMEM126B

SDHD BACE1 UBE4A DDX6 EEF1A1P33 SCAF11 MLL4 PFDN5 CBX5 NACA
LRP1 GNS EEA1 USP44 GIT2 RPL6 PEBP1 COX6A1 MLXIP SBNO1
GOLGA3 RPSAP54 MZT1 COMMD6 COL4A2 CCNB1IP1 C14orf21 ACTR10
MNAT1 C14orf142 CCNK TJP1 HERC2P10 SNAP23 B2M GTF2A2 RPL4
MYO9A C15orf40 SEC11A MPG SPSB3 NDUFB10 RPS15A SULT1A3 SRCAP
NFATC3 ATXN1L HSBP1 MNT RPL26 TOM1L2 USP22 RPL23A TAOK1 CPD RFFL
TAF15 RPL19 CDK12 WIPF2 RPL27 RPS7P11 RPS2P48 CLTC SUMO2 TNRC6C
ROCK1P1 MYL12A MYL12B ANKRD12 C18orf32 RPL17 NFIC NDUFA11 BRD4
SLC35E1 SDHAF1 ZNF585A KCNK6 RPL18 BCAT2 NUCB1-AS1 RPL13A KLK7
ZNF264 ZNF587 RPS5 TMEM230 KIF3B RP4 NCOA3 ADNP TSHZ2 RPL12P4 SON
VWFP1 THAP7 HMGB1P10 NF2 APOL6 RPL3 EP300 TCF20 RNU12-2P
USP9X RP6 HSD17B10 HUWE1 GNL3L RPS4X TTC3P1 COX7B PGAM4
ITM2A APOOL RPL39 HCFC1 RPL10 FUNDC2 MT
GOBPID Pvalue OddsRatio ExpCount Count Size Term
GO:0006415 7.33765796271195e-39 45.2089314194577 1.39166839665075
34 91 translational termination
GO:0006614 6.26152776488408e-38 38.0645161290323 1.60577122690471
35 105 SRP-dependent cotranslational protein targeting to membrane
GO:0006613 9.23046030882955e-38 37.525745873088 1.62106428620857 35
106 cotranslational protein targeting to membrane
GO:0045047 9.23046030882955e-38 37.525745873088 1.62106428620857 35
106 protein targeting to ER
GO:0072599 9.23046030882955e-38 37.525745873088 1.62106428620857 35
106 establishment of protein localization to endoplasmic reticulum
GO:0000184 2.99269513480796e-37 33.1678280690329 1.81987405715867
36 119 nuclear-transcribed mRNA catabolic process, nonsense-mediated decay
GO:0006414 2.34027871483817e-36 36.258642765685 1.60577122690471 34
105 translational elongation
GO:0070972 6.98358439932385e-36 32.0731312346159 1.80458099785482
35 118 protein localization to endoplasmic reticulum
GO:0000956 2.99556436540263e-35 23.2417582417582 2.58452702235139
39 169 nuclear-transcribed mRNA catabolic process
GO:0019080 1.69538391784688e-34 24.8995041952708 2.30925195488201
37 151 viral genome expression
GO:0019083 1.69538391784688e-34 24.8995041952708 2.30925195488201
37 151 viral transcription
GO:0006402 2.62958091883432e-34 21.7230215827338 2.72216455608608
39 178 mRNA catabolic process
GO:0006413 5.39434674311892e-33 23.6767940354147 2.32454501418587
36 152 translational initiation
GO:0006401 8.735982473712e-32 18.1549053356282 3.13507715729015
39 205 RNA catabolic process
GO:0006612 1.615353405929e-31 22.5041006014215 2.33983807348972
35 153 protein targeting to membrane
GO:0043624 1.615353405929e-31 22.5041006014215 2.33983807348972
35 153 cellular protein complex disassembly
GO:0022415 3.6452905487949e-31 15.0813387319973 3.97619541900215
42 260 viral reproductive process
GO:0043241 5.40034035051794e-31 21.5816504939243 2.416303370009 35
158 protein complex disassembly
GO:0034623 9.81317478602205e-31 19.8712103407756 2.66099231887067
36 174 cellular macromolecular complex disassembly
GO:0072594 1.29617062208105e-30 17.592731582066 3.1197840979863 38 204
establishment of protein localization to organelle
GO:0032984 2.8715111329249e-30 19.1696087696088 2.73745761538994
36 179 macromolecular complex disassembly
GO:0019058 1.05483846835524e-29 15.6674107142857 3.53269669919037
39 231 viral infectious cycle
GO:0016032 3.86713719436514e-28 9.79943306722203 6.89716974603834
49 451 viral reproduction
GO:0071845 3.32954327223926e-26 12.231778425656 4.34322884229465 39
284 cellular component disassembly at cellular level
GO:0022411 5.68518374756703e-26 12.0318416523236 4.40440107951007

39 288 cellular component disassembly
GO:0033365 1.74772767289841e-22 7.99710270896712 7.23361705072313
44 473 protein localization to organelle
GO:0006605 1.51247769006523e-21 7.7342605385959 7.24891011002699 43
474 protein targeting
GO:0006511 8.13314886944853e-07 4.01654518107139 5.566673586603 20
364 ubiquitin-dependent protein catabolic process
GO:0019941 1.09436102136724e-06 3.93445876032941 5.67372500172998
20 371 modification-dependent protein catabolic process
GO:0043632 1.23993118404523e-06 3.90027264805914 5.71960417964155
20 374 modification-dependent macromolecule catabolic process
GO:0051603 2.26947503386426e-06 3.73768016287128 5.94900006919936
20 389 proteolysis involved in cellular protein catabolic process
GO:0044257 3.73596844110694e-06 3.60709541298742 6.14780984014947
20 402 cellular protein catabolic process
GO:0022613 9.54713578926652e-06 4.49357785562195 3.44093834336724
14 225 ribonucleoprotein complex biogenesis
GO:0042254 1.81476997840804e-05 5.34893029675638 2.27866583627431
11 149 ribosome biogenesis
GO:0071843 1.88730436687949e-05 4.20976918947933 3.6550411736212 14
239 cellular component biogenesis at cellular level
Tissue: Thyroid=>Adipose_Subcutaneous Type: cluster
SourceGene: PRINS(ENSG00000238115.1)
TargetGeneSet: MTND1P23 MTND2P28 RPL22 NMNAT1 UBE4B DFFA SPEN
LDLRAD2 HSPG2 RPL11 ARID1A WASF2 DNAJC8 EIF2C1 THRAP3 MFI2 RPS8
NEDD4 RP11 RPL5 HBXIP NUDT4P1 HIST2H2AC RPRD2 JTB RPS27
ASH1L MIR555 PIGM TKT PRRC2C ZBTB37 CEP350 ARPC5 PTPN14 ZC3H11B ADI1
RPS7 ASXL2 C2orf18 CALM2 RPL36AP15 UBC USP34 COMMD1 C1D AAK1
DGUOK-AS1 TET3 DCTN1 VAMP5 TMEM131 EIF5B UGGT1 MZT2B RHOQP3
H3F3AP4 ANKRD44 RAPH1 TRIP12 PSMD1 GIGYF2 HDLBP NR2C2 UBE2E2 RPL15
TRANK1 WDR48 HIGD1A MAP4 RAD54L2 RPL29 DCP1A NIT2 PCNP RPL24
FAM162A NME9 COMMD2 PDCD10 PHC3 PEX5L DNAJC19 HTT BOD1L RELL1
DANCR LAMTOR3 RPL34 SETD7 H3F3AP6 RPS3A MARCH6 NIPBL CTD SREK1IP1
MRPS36 TAF9 SERINC5 ERAP1 HINT1 C5orf15 SKP1 CTB MTND5P11
CDC23 RPS14 ATP6V0E1 GNB2L1 TXNDC5 TMEM14C RPL15P3 NOL7 PPIAP29
ZSCAN23 SYNGAP1 MNF1 RP1 UHRF1BP1 RPL10A MTRNR2L9 WDR11
BCKDHB C6orf162 KIAA1919 PHACTR2 IGF2R SFT2D1 FOXK1 EEF1A1P6
MPLKIP CHCHD2 DPY19L2P4 RPS3AP26 TRIM56 PIK3CG COG5 CAPZA2
METTL2A ZC3HAV1L HIPK2 MLL3 DRP2 HMBOX1 DCTN6 LSM1 HOOK3
RPS20 POLR2K UBR5 EIF3E MED30 TG RGP1 CLTA CBWD6 CBWD3
ATP6V1G1 GAPVD1 RPL12 NUP214 PRRC2B SETX RPL7A PRINS FUT11
RPS24 RPS3AP5 EIF6 RPL27A MTRNR2L8 TSG101 TRIM44 CKAP5 TMEM126B
SDHD BACE1 UBE4A DDX6 EEF1A1P33 SCAF11 MLL4 PFDN5 CBX5 NACA
LRP1 GNS EEA1 USP44 GIT2 RPL6 PEBP1 COX6A1 MLXIP SBNO1
GOLGA3 RPSAP54 MZT1 COMMD6 COL4A2 CCNB1IP1 C14orf21 ACTR10
MNAT1 C14orf142 CCNK TJP1 HERC2P10 SNAP23 B2M GTF2A2 RPL4
MYO9A C15orf40 SEC11A MPG SPSB3 NDUFB10 RPS15A SULT1A3 SRCAP
NFATC3 ATXN1L HSBP1 MNT RPL26 TOM1L2 USP22 RPL23A TAOK1 CPD RFFL
TAF15 RPL19 CDK12 WIPF2 RPL27 RPS7P11 RPS2P48 CLTC SUMO2 TNRC6C
ROCK1P1 MYL12A MYL12B ANKRD12 C18orf32 RPL17 NFIC NDUFA11 BRD4
SLC35E1 SDHAF1 ZNF585A KCNK6 RPL18 BCAT2 NUCB1-AS1 RPL13A KLK7
ZNF264 ZNF587 RPS5 TMEM230 KIF3B RP4 NCOA3 ADNP TSHZ2 RPL12P4 SON
VWFP1 THAP7 HMGB1P10 NF2 APOL6 RPL3 EP300 TCF20 RNU12-2P
USP9X RP6 HSD17B10 HUWE1 GNL3L RPS4X TTC3P1 COX7B PGAM4
ITM2A APOOL RPL39 HCFC1 RPL10 FUNDC2 MT
GOBPID Pvalue OddsRatio ExpCount Count Size Term
GO:0006415 7.33765796271195e-39 45.2089314194577 1.39166839665075
34 91 translational termination
GO:0006614 6.26152776488408e-38 38.0645161290323 1.60577122690471
35 105 SRP-dependent cotranslational protein targeting to membrane
GO:0006613 9.23046030882955e-38 37.525745873088 1.62106428620857 35
106 cotranslational protein targeting to membrane
GO:0045047 9.23046030882955e-38 37.525745873088 1.62106428620857 35

106	protein targeting to ER
GO:0072599	9.23046030882955e-38	37.525745873088 1.62106428620857	35
106	establishment of protein localization to endoplasmic reticulum
GO:0000184	2.99269513480796e-37	33.1678280690329	1.81987405715867
36	119	nuclear-transcribed mRNA catabolic process, nonsense-mediated decay
GO:0006414	2.34027871483817e-36	36.258642765685 1.60577122690471	34
105	translational elongation
GO:0070972	6.98358439932385e-36	32.0731312346159	1.80458099785482
35	118	protein localization to endoplasmic reticulum
GO:0000956	2.99556436540263e-35	23.2417582417582	2.58452702235139
39	169	nuclear-transcribed mRNA catabolic process
GO:0019080	1.69538391784688e-34	24.8995041952708	2.30925195488201
37	151	viral genome expression
GO:0019083	1.69538391784688e-34	24.8995041952708	2.30925195488201
37	151	viral transcription
GO:0006402	2.62958091883432e-34	21.7230215827338	2.72216455608608
39	178	mRNA catabolic process
GO:0006413	5.39434674311892e-33	23.6767940354147	2.32454501418587
36	152	translational initiation
GO:0006401	8.735982473712e-32	18.1549053356282	3.13507715729015
39	205	RNA catabolic process
GO:0006612	1.615353405929e-31	22.5041006014215	2.33983807348972
35	153	protein targeting to membrane
GO:0043624	1.615353405929e-31	22.5041006014215	2.33983807348972
35	153	cellular protein complex disassembly
GO:0022415	3.6452905487949e-31	15.0813387319973	3.97619541900215
42	260	viral reproductive process
GO:0043241	5.40034035051794e-31	21.5816504939243	2.416303370009	35
158	protein complex disassembly
GO:0034623	9.81317478602205e-31	19.8712103407756	2.66099231887067
36	174	cellular macromolecular complex disassembly
GO:0072594	1.29617062208105e-30	17.592731582066 3.1197840979863 38	204
establishment of protein localization to organelle
GO:0032984	2.8715111329249e-30	19.1696087696088	2.73745761538994
36	179	macromolecular complex disassembly
GO:0019058	1.05483846835524e-29	15.6674107142857	3.53269669919037
39	231	viral infectious cycle
GO:0016032	3.86713719436514e-28	9.79943306722203	6.89716974603834
49	451	viral reproduction
GO:0071845	3.32954327223926e-26	12.231778425656 4.34322884229465	39
284	cellular component disassembly at cellular level
GO:0022411	5.68518374756703e-26	12.0318416523236	4.40440107951007
39	288	cellular component disassembly
GO:0033365	1.74772767289841e-22	7.99710270896712	7.23361705072313
44	473	protein localization to organelle
GO:0006605	1.51247769006523e-21	7.7342605385959 7.24891011002699	43
474	protein targeting
GO:0006511	8.13314886944853e-07	4.01654518107139	5.566673586603	20
364	ubiquitin-dependent protein catabolic process
GO:0019941	1.09436102136724e-06	3.93445876032941	5.67372500172998
20	371	modification-dependent protein catabolic process
GO:0043632	1.23993118404523e-06	3.90027264805914	5.71960417964155
20	374	modification-dependent macromolecule catabolic process
GO:0051603	2.26947503386426e-06	3.73768016287128	5.94900006919936
20	389	proteolysis involved in cellular protein catabolic process
GO:0044257	3.73596844110694e-06	3.60709541298742	6.14780984014947
20	402	cellular protein catabolic process
GO:0022613	9.54713578926652e-06	4.49357785562195	3.44093834336724
14	225	ribonucleoprotein complex biogenesis
GO:0042254	1.81476997840804e-05	5.34893029675638	2.27866583627431
11	149	ribosome biogenesis
GO:0071843	1.88730436687949e-05	4.20976918947933	3.6550411736212 14
239	cellular component biogenesis at cellular level

Tissue: Adipose_Subcutaneous=>Thyroid Type: asymmetric
SourceGene: RNU5B-3P(ENSG00000252410.1)
TargetGeneSet: GBP5 GAPDHP23 FCRL5 LAX1 IGKJ5 IGKC IGKJ4
IGKJ3 IGKJ2 IGKJ1 IGKV4-1 IGKV1-5 IGKV1-9 IGKV3-11 IGKV3-15
IGKV1-16 IGKV2-28 IGKV1-39 SP140 IGJ RP11 CPQ
TSPAN32 POU2AF1 PPIHP1 IGHG2 IGHGP IGHG1 IGHG3 KIAA0125 IGHV4-39
CHRFAM7A U6 FER1L4 IGLV1-47 IGLV3-19 IGLV2-14
IGLV3-10 IGLV3-1 IGLL5 IGLC1 IGLC3
GOBPID Pvalue OddsRatio ExpCount Count Size Term
GO:0006958 1.43760734354932e-16 342.690476190476 0.0553594906926856
8 50 complement activation, classical pathway
GO:0006956 1.03041429986511e-15 261.454545454545 0.0697529582727839
8 63 complement activation
GO:0002455 1.34156965685537e-15 252.245614035088 0.0719673379004913
8 65 humoral immune response mediated by circulating immunoglobulin
GO:0006959 1.75770439496887e-15 162.955752212389 0.135077157290153
9 122 humoral immune response
GO:0072376 9.39406184566582e-15 194.067567567568 0.0907895647360044
8 82 protein activation cascade
GO:0016064 1.14715514870305e-13 139.145631067961 0.122898069337762
8 111 immunoglobulin mediated immune response
GO:0019724 1.42774143786272e-13 135.179245283019 0.126219638779323
8 114 B cell mediated immunity
GO:0002449 3.1980049917134e-12 89.7861635220126 0.18490069891357
8 167 lymphocyte mediated immunity
GO:0002460 6.12848705203208e-12 82.4393063583815 0.200401356307522
8 181 adaptive immune response based on somatic recombination of immune receptors built from immunoglobulin superfamily domains
GO:0002250 1.26327648627516e-11 74.9736842105263 0.219223583143035
8 198 adaptive immune response
GO:0002443 2.10622277151748e-11 70.1083743842365 0.233617050723133
8 211 leukocyte mediated immunity
GO:0002253 3.41068508676345e-10 48.6048109965636 0.33104975434226
8 299 activation of immune response
GO:0050778 1.61994340149145e-09 39.5477528089888 0.403017092242751
8 364 positive regulation of immune response
GO:0002252 2.57343455361979e-09 37.1878306878307 0.427375268147533
8 386 immune effector process
Tissue: Thyroid=>Adipose_Subcutaneous Type: asymmetric
SourceGene: RP11-165M1.3(ENSG00000261394.1)
TargetGeneSet: RPL22 NMNAT1 ZMYM4 RPS8 GTF2B RPL5 RPS27 GAS5
ZBTB37 RP11 ZC3H11B RPS7 SNX17 CCT7 H3F3AP4 BRK1 RPL15 RPL14
LIMD1 PCNP RPL24 C3orf33 RPL7L1P8 KIAA1530 LAMTOR3 EEF1A1P9
H3F3AP6 RPS3A RWDD4 PDCD6 CTD SMN1 SKP1 CTB RPL15P3 PPIAP29 ABT1
RPS10 RPL10A YIPF3 FTH1P5 EEF1A1P6 RPS3AP26 TRIM56 CLTA
ATP6V1G1 RPL7A ADRA2A RPS3AP5 BCCIP RPL27A TSG101 MRE11A COPZ1 LRP1
RPL6 RPSAP54 COL4A2-AS1 SIVA1 RAB11A MYO9A RPS15A TUFM
SLX1B-SULT1A4 SULT1A4 HSBP1 TAOK1 RPS7P11 PPM1E SUMO2 RPL17 NFIC
RPL18A RPSAP58 RPL18 RPL13A RPL41P1 DPM1 RPL12P4 RP1 ZNF70 RP6
WASF4P RPS23P8 HK2P1 MT
GOBPID Pvalue OddsRatio ExpCount Count Size Term
GO:0006414 5.5318913889203e-29 86.316742081448 0.428690056051484 20
105 translational elongation
GO:0006415 2.36445172426491e-28 94.4722222222222 0.371531381911286
19 91 translational termination
GO:0006614 4.60603802964364e-27 79.0156976744186 0.428690056051484
19 105 SRP-dependent cotranslational protein targeting to membrane
GO:0006613 5.59710219405467e-27 78.1020114942529 0.43277281849007
19 106 cotranslational protein targeting to membrane
GO:0045047 5.59710219405467e-27 78.1020114942529 0.43277281849007
19 106 protein targeting to ER
GO:0072599 5.59710219405467e-27 78.1020114942529 0.43277281849007
19 106 establishment of protein localization to endoplasmic reticulum

GO:0070972	5.00424773490483e-26	68.5775252525252	0.481765967753097
19	118	protein localization to endoplasmic reticulum
GO:0000184	5.9392824035809e-26	67.887	0.485848730191682	19	119
nuclear-transcribed mRNA catabolic process, nonsense-mediated decay
GO:0019080	7.04633966105414e-24	51.3143939393939	0.61649712822642
19	151	viral genome expression
GO:0019083	7.04633966105414e-24	51.3143939393939	0.61649712822642
19	151	viral transcription
GO:0006413	8.0315888380199e-24	50.925	0.620579890665006	19	152
translational initiation
GO:0006612	9.14605855784254e-24	50.5414179104478	0.624662653103591
19	153	protein targeting to membrane
GO:0043624	9.14605855784254e-24	50.5414179104478	0.624662653103591
19	153	cellular protein complex disassembly
GO:0043241	1.72786162919261e-23	48.7062949640288	0.645076465296519
19	158	protein complex disassembly
GO:0000956	6.50560619729064e-23	45.0996666666667	0.68998685212096
19	169	nuclear-transcribed mRNA catabolic process
GO:0034623	1.15269674110565e-22	43.6295161290323	0.710400664313888
19	174	cellular macromolecular complex disassembly
GO:0006402	1.79869541711764e-22	42.5199685534591	0.726731714068231
19	178	mRNA catabolic process
GO:0032984	2.0069417338134e-22	42.25125	0.730814476506816	19
179	macromolecular complex disassembly
GO:0019058	8.33552572293967e-22	34.4659132336857	0.943118123313265
20	231	viral infectious cycle
GO:0072594	2.55136214309036e-21	36.4774324324324	0.832883537471455
19	204	establishment of protein localization to organelle
GO:0006401	2.80449918466187e-21	36.2787634408602	0.836966299910041
19	205	RNA catabolic process
GO:0022415	9.06468470910005e-21	30.2393162393162	1.06151823403225
20	260	viral reproductive process
GO:0016032	1.39265510665852e-18	19.3527373527374	1.84132585980209
22	451	viral reproduction
GO:0071845	1.41977872101893e-18	25.3219811320755	1.1595045325583 19
284	cellular component disassembly at cellular level
GO:0022411	1.84830042862882e-18	24.9383828996283	1.17583558231264
19	288	cellular component disassembly
GO:0033365	1.77300484115557e-14	14.5827092511013	1.93114663345097
19	473	protein localization to organelle
GO:0006605	1.84204569789139e-14	14.5496153846154	1.93522939588956
19	474	protein targeting
Tissue: Adipose_Subcutaneous=>Thyroid	Type:	cluster
SourceGene:	RP11-416A17.1(ENSG00000244540.1)
TargetGeneSet:	RP11	FCRL3	FCRL2	IGKJ5	IGKC	IGKJ4	IGKJ3	IGKJ2
IGKJ1	IGKV4-1 IGKV1-5 IGKV1-6 IGKV1-8 IGKV3-15	IGKV3D-15	EOMES
KLHL7	IGJ	GALNTL6 BLK	POU2AF1 GS1	GJB6	IGHA2	IGHG2	IGHGP
IGHA1	IGHG1	IGHG3	IGHM	IGHJ5	IGHV6-1 IGHV2-5 IGHV1-18	IGHV3-30
IGHV3-33	IGHV3-48	IGHV5-51	IGHV4-59	TNFRSF17
TBC1D27 IGLV4-69	IGLV1-47	IGLV5-45	IGLV1-44	IGLV1-40
ZNF280A IGLV3-25	IGLV2-11	IGLV3-1 IGLC1	IGLC2	IGLC3
GOBPID	Pvalue	OddsRatio	ExpCount	Count	Size	Term
GO:0006958	4.17272448412705e-17	210.526829268293	0.0830392360390284
9	50	complement activation, classical pathway
GO:0006956	3.89403027699151e-16	159.7	0.104629437409176	9	63
complement activation
GO:0002455	5.24961254567502e-16	153.975	0.107951006850737	9	65
humoral immune response mediated by circulating immunoglobulin
GO:0006959	2.24370981243159e-15	91.2946428571429	0.202615735935229
10	122	humoral immune response
GO:0072376	4.73636565626121e-15	117.978082191781	0.136184347104007
9	82	protein activation cascade
GO:0016064	7.94729347232045e-14	84.2647058823529	0.184347104006643

9	111	immunoglobulin mediated immune response
GO:0019724	1.01656580271012e-13	81.84	0.189329458168985	9	114
B cell mediated immunity
GO:0002449	3.33579003599497e-12	54.1860759493671	0.277351048370355
9	167	lymphocyte mediated immunity
GO:0002460	6.91206296908552e-12	49.7267441860465	0.300602034461283
9	181	adaptive immune response based on somatic recombination of immune receptors built from immunoglobulin superfamily domains
GO:0002250	1.55296624225031e-11	45.2	0.328835374714553	9	198
adaptive immune response
GO:0002253	1.87926130523349e-11	34.9431537320811	0.49657463151339
10	299	activation of immune response
GO:0002443	2.75033885290613e-11	42.2524752475247	0.3504255760847 9
211	leukocyte mediated immunity
GO:0050778	1.3027059467683e-10	28.3958837772397	0.604525638364127
10	364	positive regulation of immune response
GO:0002252	2.31323124500339e-10	26.6926291793313	0.6410629022213 10
386	immune effector process
Tissue: Thyroid=>Adipose_Subcutaneous	Type:	asymmetric
SourceGene:	RP11-95P2.3(ENSG00000263105.1)
TargetGeneSet:	FAM213B CEP104	DFFA	LUZP1	RPL11	RP4	RP1	RPS8	RPL5
MTF2	RWDD3	RP11	HBXIP	RPRD2	PI4KB	KIRREL	GORAB	ACBD6	UBC
PRORSD1P	CCDC104 COMMD1	TPRKB	DGUOK	ZEB2-AS1	ZNF662	SETD2
IP6K2	RPL10AP6	NIT2	RPL24	HEG1	TRIM42	RPL22L1 DNAJC19 HNRPDL
RPL34	SETD7	FRG1	C5orf55 NDUFAF2 TAF9	GRAMD3	CTB	NDST1	EHMT2
AGPAT1	MNF1	RPL10A	LINC00472	RPL39P3 COX7A2	SENP6	C6orf162
TRMT11	MRPS12	C7orf44 BAZ1B	FIS1	ST7-AS1 MRPS33	SSBP1	ZNF862	MLL3
KCTD9	DCTN6	RPS20	CHCHD7	C8orf59 RPL30	NDUFB9	ZNF572	RPS6	RPL12
PRRC2B	ARHGAP12	RPS24	SFR1	CYB5R2	RPL27A	FIBIN	INCENP	MRPL11
CLNS1A	TMEM126B	TMEM126A	SDHD	RPS25	CBL	PFDN5	SSH1	MMAB
RILPL1	MED4-AS1	NIN	MNAT1	EXD2	COX16	RPL15P2 PPP1R13B
ATPBD4	RN5S395 RPS27L	LRRC49	C15orf39	WDR61	MORF4L1 C15orf40
RPS15A	NFATC3	APRT	RPL26	C17orf48	NCOR1	RPL23A	RFFL	RPL17
RPL19	RPL27	SSTR2	CDC42EP4	TNRC6C	CTD	NDUFV2	C18orf32
RPS15	ZBTB7A	SLC35E1 ZNF224	SNRPD2	ARHGAP35	RPL18	ZNF264	SNRPB2
SOGA1	SNHG11	B4GALT5 SPATA2	DPM1	TSHZ2	PFDN4	ATP5O	KRTAP10-5
MRPL40	FAM211B EP300	FTLP2	RPS2P55 CXorf36 UXT	IQSEC2	COX7B	MID2
RPL39	CCDC160 MECP2	FUNDC2	MT
GOBPID	Pvalue	OddsRatio	ExpCount	Count	Size	Term
GO:0006415	3.6001244660263e-29	54.2354593522802	0.705279911424815
23	91	translational termination
GO:0006414	1.38215202205398e-27	44.9316251027679	0.813784513182479
23	105	translational elongation
GO:0006614	1.38215202205398e-27	44.9316251027679	0.813784513182479
23	105	SRP-dependent cotranslational protein targeting to membrane
GO:0006613	1.75467512630116e-27	44.3871666441045	0.821534841879455
23	106	cotranslational protein targeting to membrane
GO:0045047	1.75467512630116e-27	44.3871666441045	0.821534841879455
23	106	protein targeting to ER
GO:0072599	1.75467512630116e-27	44.3871666441045	0.821534841879455
23	106	establishment of protein localization to endoplasmic reticulum
GO:0019080	1.08115365886981e-26	32.4142492245941	1.17029963324337
25	151	viral genome expression
GO:0019083	1.08115365886981e-26	32.4142492245941	1.17029963324337
25	151	viral transcription
GO:0070972	2.54989901228049e-26	38.747723240686 0.914538786243167	23
118	protein localization to endoplasmic reticulum
GO:0000184	3.14202731221828e-26	38.34140917603	0.922289114940143	23
119	nuclear-transcribed mRNA catabolic process, nonsense-mediated decay
GO:0034623	1.18028220087103e-23	25.7981818181818	1.34855719327382
24	174	cellular macromolecular complex disassembly
GO:0006413	1.21010173272858e-23	28.4670324884592	1.17804996194035
23	152	translational initiation

GO:0006612	1.41571643707551e-23	28.2460674157303	1.18580029063733
23	153	protein targeting to membrane
GO:0043624	1.41571643707551e-23	28.2460674157303	1.18580029063733
23	153	cellular protein complex disassembly
GO:0032984	2.37440225082577e-23	24.9571847507331	1.3873088367587 24
179	macromolecular complex disassembly
GO:0019058	2.8929724382685e-23	20.8442427680091	1.79032592900145
26	231	viral infectious cycle
GO:0043241	3.05073149597029e-23	27.1903454015814	1.22455193412221
23	158	protein complex disassembly
GO:0000956	1.50675129089966e-22	25.1222872094813	1.30980554978894
23	169	nuclear-transcribed mRNA catabolic process
GO:0006402	5.11638036035902e-22	23.6485683218557	1.37955850806173
23	178	mRNA catabolic process
GO:0072594	5.79866532403971e-22	21.4530303030303	1.5810670541831 24
204	establishment of protein localization to organelle
GO:0022415	6.26001439040458e-22	18.2235142118863	2.01508546121376
26	260	viral reproductive process
GO:0006401	1.37336880572313e-20	20.1019261637239	1.58881738288008
23	205	RNA catabolic process
GO:0071845	9.85998518569059e-20	15.6215328629122	2.20109334994118
25	284	cellular component disassembly at cellular level
GO:0022411	1.38750251207979e-19	15.3795725711289	2.23209466472908
25	288	cellular component disassembly
GO:0016032	5.71737765680821e-17	10.424667036626 3.49539824233617	27
451	viral reproduction
GO:0033365	1.72757418304349e-14	8.90997023809524	3.66590547366964
25	473	protein localization to organelle
GO:0006605	1.81291181544045e-14	8.88948621457645	3.67365580236662
25	474	protein targeting
GO:0022613	1.69884318108812e-07	7.95830985915493	1.7438239568196 12
225	ribonucleoprotein complex biogenesis
GO:0042274	2.02089222131986e-07	51.4953271028037	0.139505916545568
5	18	ribosomal small subunit biogenesis
GO:0071843	3.26044016689171e-07	7.46008810572687	1.85232855857726
12	239	cellular component biogenesis at cellular level
GO:0006364	1.40535534033086e-06	11.4126602564103	0.806034184485503
8	104	rRNA processing
GO:0016072	2.15109764132722e-06	10.736802413273 0.852536156667359	8
110	rRNA metabolic process
GO:0042254	2.30284618741013e-06	8.8620665742025 1.15479897584942	9
149	ribosome biogenesis
GO:0018022	2.31647393217416e-06	58.9711934156379	0.100754273060688
4	13	peptidyl-lysine methylation
GO:0034660	1.30103427658037e-05	5.59062658090627	2.20884367863816
11	285	ncRNA metabolic process
Tissue: Thyroid=>Adipose_Subcutaneous	Type:	asymmetric
SourceGene:	SERPINB7(ENSG00000166396.8)
TargetGeneSet:	RP4	RPL11	SYNC	RP3	RPS8	PTGES3P1	RP11
RP11-165H20.1	SRGAP2P1	ISG20L2 C1orf9	UBC	COX5B	WDSUB1	RPL37A
PCOLCE2 NAALADL2	RPL34	NDUFC1	COX7C	CTD	HINT1	PAIP2	CTB
RPS14	GPLD1	PPP1R10 RPL24P4 RPL39P3 FILIP1	MRPS12	C7orf44 RPS20	EEF1D	RPL8
RPS6	ANKRD19P	SEC61B	RPL35	ITIH5	RPS24	NHP2P1	PIDD	RPLP2
STIM1	RPL27A	C11orf10	FAU	CDC42EP2	TMEM126A	RPS25
ZDHHC17 ACTR6	EIF4A1P7	RAD17P2 RPL7AP6 SNAP23	DUT	RPS3AP6 RPLP1
C16orf13	RPL13	RPL7AP64	RPL19	RPL27	BPTF	C18orf21
DAZAP1	RPS15	UQCR11	ZBTB7A	RPL36	RPS19	APOE	RPL18	SLC27A5 STS
EEF1B2P3	RPL39
GOBPID	Pvalue	OddsRatio	ExpCount	Count	Size	Term
GO:0006414	3.91760352999628e-39	131.544117647059	0.428690056051484
25	105	translational elongation
GO:0006614	3.91760352999628e-39	131.544117647059	0.428690056051484
25	105	SRP-dependent cotranslational protein targeting to membrane

GO:0006613 5.11506298933691e-39 129.91103848947 0.43277281849007 25
106 cotranslational protein targeting to membrane
GO:0045047 5.11506298933691e-39 129.91103848947 0.43277281849007 25
106 protein targeting to ER
GO:0072599 5.11506298933691e-39 129.91103848947 0.43277281849007 25
106 establishment of protein localization to endoplasmic reticulum
GO:0006415 1.01595607281427e-38 146.609808102345 0.371531381911286
24 91 translational termination
GO:0070972 1.01929864570169e-37 113.053447185326 0.481765967753097
25 118 protein localization to endoplasmic reticulum
GO:0000184 1.38503807196725e-35 103.196390977444 0.485848730191682
24 119 nuclear-transcribed mRNA catabolic process, nonsense-mediated decay
GO:0006413 1.01370724578367e-34 82.5903195924039 0.620579890665006
25 152 translational initiation
GO:0006612 1.20892872418513e-34 81.9393382352941 0.624662653103591
25 153 protein targeting to membrane
GO:0019080 6.81769414030768e-33 77.0213723284589 0.61649712822642
24 151 viral genome expression
GO:0019083 6.81769414030768e-33 77.0213723284589 0.61649712822642
24 151 viral transcription
GO:0043624 9.55721164742459e-33 75.8166112956811 0.624662653103591
24 153 cellular protein complex disassembly
GO:0043241 2.17778342448324e-32 72.9620469083156 0.645076465296519
24 158 protein complex disassembly
GO:0000956 1.210548569293e-31 67.3749753694581 0.68998685212096
24 169 nuclear-transcribed mRNA catabolic process
GO:0072594 2.45626616808616e-31 58.3839960565232 0.832883537471455
25 204 establishment of protein localization to organelle
GO:0034623 2.53647848095198e-31 65.1062857142857 0.710400664313888
24 174 cellular macromolecular complex disassembly
GO:0006402 4.50871690182668e-31 63.3974025974026 0.726731714068231
24 178 mRNA catabolic process
GO:0032984 5.19459579490162e-31 62.9839631336406 0.730814476506816
24 179 macromolecular complex disassembly
GO:0006401 1.56735702897842e-29 53.8380426203631 0.836966299910041
24 205 RNA catabolic process
GO:0019058 3.04852624346059e-28 46.9896480331263 0.943118123313265
24 231 viral infectious cycle
GO:0022415 5.60861453497785e-27 41.1312348668281 1.06151823403225
24 260 viral reproductive process
GO:0071845 4.85079485677874e-26 37.2712087912088 1.1595045325583 24
284 cellular component disassembly at cellular level
GO:0022411 6.81833763565149e-26 36.6961038961039 1.17583558231264
24 288 cellular component disassembly
GO:0033365 1.81251467797619e-23 24.5793505525049 1.93114663345097
26 473 protein localization to organelle
GO:0006605 1.91335697240479e-23 24.5227272727273 1.93522939588956
26 474 protein targeting
GO:0016032 3.14244848263736e-21 22.4262295081967 1.84132585980209
24 451 viral reproduction
GO:0042274 4.02163361774794e-13 175.991258741259 0.0734897238945402
7 18 ribosomal small subunit biogenesis
GO:0042254 8.37006330389339e-09 18.324 0.608331603349249 9 149
ribosome biogenesis
GO:0022613 2.21126551623303e-08 13.4570479354533 0.918621548681752
10 225 ribonucleoprotein complex biogenesis
GO:0071843 3.9182406551914e-08 12.6218697085821 0.97578022282195
10 239 cellular component biogenesis at cellular level
GO:0006364 2.04628754142937e-07 19.838421887391 0.424607293612899 7
104 rRNA processing
GO:0016072 3.00773334138548e-07 18.6749439880508 0.449103868244412
7 110 rRNA metabolic process
GO:0000028 1.28170735981912e-06 256.946428571429 0.0244965746315134

3	6	ribosomal small subunit assembly
GO:0030490	2.23647028926986e-06	192.696428571429	0.028579337070099
3	7	maturation of SSU-rRNA
GO:0034470	1.62448585712689e-05	9.90364687126345	0.816552487717113
7	200	ncRNA processing
Tissue: Thyroid=>Adipose_Subcutaneous	Type:	asymmetric
SourceGene:	STAC2(ENSG00000141750.5)
TargetGeneSet:	RPL11	SPATA1	GTF2B	RPL5	RP4	RP11	MRPL9	UFC1
GORAB	ZBTB37	RPS7	UBC	RPL37A	RPL14	RPL24	CD80	AADAC	ANKRD17
RPL34	EEF1A1P19	BTF3	CTB	RPS14	GTPBP10	BPGM	RPS20	RPL30	RPL8
RPS6	RPL35	GPSM1	RPS24	RPL13AP5	FAU	RPS3	RPL41	NACA
CSRP2	C12orf51	ST13P4	POU4F1	RPL7AP6	GANC	RPS3AP6	RPL4	GOLGA6L9
RPS15A	CES4A	RPL26	TOM1L2	RPS7P1	RPL23A	RPL17	RPL19	RPL27	CTD
RPS16	RPS19	SNRPD2	RPS11	RPS23P8	RPS4X
GOBPID	Pvalue	OddsRatio	ExpCount	Count	Size	Term
GO:0006415	1.45136449418672e-52	304.840175953079	0.321154245380943
29	91	translational termination
GO:0006414	1.89776590058902e-50	248.442583732057	0.370562590824164
29	105	translational elongation
GO:0006614	1.89776590058902e-50	248.442583732057	0.370562590824164
29	105	SRP-dependent cotranslational protein targeting to membrane
GO:0006613	2.60863724274437e-50	245.19893742621	0.374091758355823	29
106	cotranslational protein targeting to membrane
GO:0045047	2.60863724274437e-50	245.19893742621	0.374091758355823	29
106	protein targeting to ER
GO:0072599	2.60863724274437e-50	245.19893742621	0.374091758355823	29
106	establishment of protein localization to endoplasmic reticulum
GO:0070972	9.21040633400354e-49	211.960674157303	0.416441768735728
29	118	protein localization to endoplasmic reticulum
GO:0000184	1.21601407591743e-48	209.590909090909	0.419970936267386
29	119	nuclear-transcribed mRNA catabolic process, nonsense-mediated decay
GO:0019080	2.69986575030717e-45	154.270491803279	0.532904297280465
29	151	viral genome expression
GO:0019083	2.69986575030717e-45	154.270491803279	0.532904297280465
29	151	viral transcription
GO:0006413	3.33145999118763e-45	153.005543237251	0.536433464812124
29	152	translational initiation
GO:0006612	4.1044811073224e-45	151.760997067449	0.539962632343782
29	153	protein targeting to membrane
GO:0043624	4.1044811073224e-45	151.760997067449	0.539962632343782
29	153	cellular protein complex disassembly
GO:0043241	1.13940202102058e-44	145.827695560254	0.557608470002076
29	158	protein complex disassembly
GO:0000956	9.54110386490306e-44	134.266233766234	0.596429312850322
29	169	nuclear-transcribed mRNA catabolic process
GO:0034623	2.38428944348493e-43	129.590909090909	0.614075150508615
29	174	cellular macromolecular complex disassembly
GO:0006402	4.85955415276291e-43	126.076571079927	0.62819182063525
29	178	mRNA catabolic process
GO:0032984	5.79043246756825e-43	125.227272727273	0.631720988166909
29	179	macromolecular complex disassembly
GO:0072594	3.36770442063706e-41	107.149350649351	0.719950176458377
29	204	establishment of protein localization to organelle
GO:0006401	3.91675899148825e-41	106.53305785124	0.723479343990035	29
205	RNA catabolic process
GO:0019058	1.53514968848981e-39	92.6512151215122	0.815237699813162
29	231	viral infectious cycle
GO:0022415	5.61262883182794e-38	80.8541912632822	0.917583558231264
29	260	viral reproductive process
GO:0071845	8.07621455431916e-37	73.120320855615	1.00228357899107	29
284	cellular component disassembly at cellular level
GO:0022411	1.23024967229617e-36	71.9706914706915	1.01640024911771
29	288	cellular component disassembly

GO:0016032 1.62286670818752e-32 47.4346793349169 1.59165455677808
30 451 viral reproduction
GO:0033365 2.95274583043609e-30 41.4336609336609 1.66929624247457
29 473 protein localization to organelle
GO:0006605 3.14039544754146e-30 41.3375893769152 1.67282541000623
29 474 protein targeting
GO:0022613 6.30863781610371e-13 22.895233366435 0.794062694623209 13
225 ribonucleoprotein complex biogenesis
GO:0071843 1.36407422619961e-12 21.4557522123894 0.843471040066431
13 239 cellular component biogenesis at cellular level
GO:0006364 2.39628482267737e-12 37.1198754540737 0.367033423292506
10 104 rRNA processing
GO:0042254 3.22551214992942e-12 28.420652173913 0.525845962217148 11
149 ribosome biogenesis
GO:0016072 4.23907571398562e-12 34.8780487804878 0.388208428482458
10 110 rRNA metabolic process
GO:0042274 2.56231928117234e-11 159.866666666667 0.0635250155698568
6 18 ribosomal small subunit biogenesis
GO:0034470 1.60597167918263e-09 18.2413350449294 0.705833506331742
10 200 ncRNA processing
GO:0034660 3.42466960135217e-09 14.1775547445255 1.00581274652273
11 285 ncRNA metabolic process
GO:0042273 6.66989270388823e-08 153.106382978723 0.0423500103799045
4 12 ribosomal large subunit biogenesis
Tissue: Thyroid=>Adipose_Subcutaneous Type: asymmetric

SourceGene: TNFRSF14(ENSG00000157873.11)

TargetGeneSet: CEP104 UBR4 RP1 PPCS RP11 RPS27 GUK1 UBC
TPRKB ART4 EEF1B2 IL17RC FLNB-AS1 THOC7 TMEM45A FAM55C RPL22L1
WHSC1 BDH2 RPL34 RPS3A CTD TAF9 NDFIP1 CTB ANKS1A TRMT11
IGF2R TMEM120A FIS1 C7orf73 RPS20 RPL30 POLR2K EEF1D GPAA1 RPL8
RPS6 NOXA1 KIAA0913 RPS24 BTRC TRIM44 C11orf55 FIBP MLL
RPS25 B4GALNT3 ZNF384 NACA POC1B RPL18AP3 KIAA0247
ZC3H14 RPS3AP6 LRRC49 WDR61 APRT TAX1BP3 RPL26 C17orf103 HSD17B1P1
RPL27 MAP3K3 NT5C SEMA6B RPL36 ERCC1 C19orf68 B4GALT5 RPS2P55 UXT
RPL39

GOBPID Pvalue OddsRatio ExpCount Count Size Term

GO:0006414 3.09E-22 67.69012419 0.39236039 16 105
translational elongation

GO:0070972	1.27E-19	53.37565347	0.440938343	15	118
protein localization to endoplasmic reticulum					
GO:0006415	1.44E-19	65.09090909	0.340045672	14	91
translational termination					
GO:0006614	1.19E-18	55.02307692	0.39236039	14	105
SRP-dependent cotranslational protein targeting to membrane					
GO:0006613	1.37E-18	54.42119565	0.396097156	14	106
cotranslational protein targeting to membrane					
GO:0045047	1.37E-18	54.42119565	0.396097156	14	106
protein targeting to ER					
GO:0072599	1.37E-18	54.42119565	0.396097156	14	106
establishment of protein localization to endoplasmic reticulum					
GO:0019080	5.81E-18	40.33088235	0.564251609	15	151
viral genome expression					

GO:0019083	5.81E-18	40.33088235	0.564251609	15	151
viral transcription					
GO:0000184	7.40E-18	47.64	0.444675109	14	119
"nuclear-transcribed mRNA catabolic process, nonsense-mediated decay"					
GO:0006413	2.50E-16	36.16413043	0.567988375	14	152
translational initiation					
GO:0006612	2.74E-16	35.90143885	0.57172514	14	153
protein targeting to membrane					
GO:0043624	2.74E-16	35.90143885	0.57172514	14	153
cellular protein complex disassembly					
GO:0043241	4.33E-16	34.64270833	0.590408968	14	158
protein complex disassembly					
GO:0022415	8.70E-16	24.42277826	0.971559062	16	260
viral reproductive process					

GO:0000956	1.12E-15	32.15935484	0.63151339	14	169	nuclear-transcribed mRNA catabolic process
GO:0034623	1.70E-15	31.1434375	0.650197218	14	174	cellular macromolecular complex disassembly
GO:0006402	2.33E-15	30.37530488	0.665144281	14	178	mRNA catabolic process
GO:0032984	2.53E-15	30.18909091	0.668881046	14	179	macromolecular complex disassembly
GO:0019058	3.59E-15	25.25106838	0.863192859	15	231	viral infectious cycle
GO:0072594	1.58E-14	26.17078947	0.762300187	14	204	establishment of protein localization to organelle
GO:0006401	1.69E-14	26.03193717	0.766036952	14	205	RNA catabolic process

GO:0016032 1.94E-14 16.12471132 1.685281295 18 451
viral reproduction

GO:0071845 1.50E-12 18.31277778 1.061241437 14 284
cellular component disassembly at cellular level

GO:0022411 1.81E-12 18.04032847 1.076188499 14 288
cellular component disassembly

GO:0033365 1.14E-10 11.70557608 1.767490139 15 473
protein localization to organelle

GO:0006605 1.34E-09 10.60423913 1.771226905 14 474
protein targeting

Tissue: Artery_Tibial=>Thyroid Type: asymmetric
SourceGene: AMPD3(ENSG00000133805.10)
TargetGeneSet: RPL22 ICMT RP11 RP3 WNT4 RPL11 KDM4A RPS8 IPP
PGM1 FPGT GNG5 EVI5 RPL5 ABCD3 PSMA5 RPS27 RGS8 NAV1
IPO9-AS1 CICP13 ACBD3 B3GALNT2 TRIM58 RPS7 DDX1 CIB4
KCNK3 DHX57 SRBD1 UBC AAK1 PAX8 MAP3K2 METTL8 OLA1 NBEAL1
CREB1 PSMD1 GRM7 ATP2B2 TMPPE DCLK3 RPL14 CTNNB1 RPL29 CRYBG3
RPL24 HGD ZNF148 COPG1 METTL15P1 PSMD2 C3orf70 MB21D2 MIR943
TAPT1 SCFD2 THAP6 SEC31A AGPAT9 RPS3A TMA16 STOX2 SORBS2 ANKRA2 CTD
PRRC1 CAMLG MTND5P11 PCDHA14 GNB2L1 RP1 MUTED PHACTR1 AGPAT1
CCND3 FAXC PDE7B IFNGR1 LATS1 TIAM2 TULP4 FAM120B ELFN1 FBXL18
TMEM60 RPS3AP26 CAV1 CEP41 RPS20 YTHDF3 FAM82B EIF3E FER1L6

AK3P2 PUF60 RPS6 VCP TSTD2 TGFBR1 CTNNAL1 RPL7A SEC16A CCNY
HKDC1 HK1 TSPAN15 C10orf35 VDAC2 RPS3AP5 ALDH18A1 ENTPD7 GBF1
MXI1 SEC23IP RPL27A ST5 PLEKHA7 MUC15 METTL15 AMBRA1 ARHGAP1 CYBASC3
FADS2 U6 SNHG1 DRAP1 TSGA10IP NDUFV1 CCND1 CAPN5 PAK1
ARHGAP20 RPS12P21 RPS25 PKNOX2 NRIP2 COPS7A CYP27B1 CCDC65
PFDN5 CBX5 CAND1 NAP1L1 LTA4H SLC9A7P1 SYCP3 KCTD10 RPL6
NOC4L ANKRD26P4 N4BP2L1 TPT1 LMO7 GRK1 SALL2 CPNE6 TSSK6
LINC00517 MGAT2 GNPNAT1 VTI1B FAM181A-AS1 FAM181A WDR25 SEC11A DET1
GLYR1 BFAR RPS15A 7SK CHMP1A POLR2A RPL26 ZNF29P TOM1L2 RPS7P1
RPL23A RASL10B ZNHIT3 RPL17 RPL19 CNP UBTF RPS7P11 CD300C TNRC6C
BAHCC1 MAPRE2 MYO5B ADNP2 ZNF562 ZNF846 NOTCH3 SLC35E1 ARMC6 RPSAP58 OPA3
RSPH6A FPR3 ZNF578 AP5S1 RRBP1 PYGB COX4I2 FOXS1 PPIAP3 ZNFX1
SNAI1 ADNP PMEPA1 GNAS SLCO4A1 SOX18 WRB ZNF70 TTC28 SH3BP1
SLC35A2 IGBP1 CXCR3 NHSL2 RPS4X FGF16 LPAR4 FRMPD3 XIAP
GOBPID Pvalue OddsRatio ExpCount Count Size Term
GO:0006415 5.49707574372244e-25 33.6272110242698 1.04532558300464
23 91 translational termination
GO:0006614 6.885869606049e-25 29.6379760041732 1.20614490346689
24 105 SRP-dependent cotranslational protein targeting to membrane
GO:0006613 8.81679103035034e-25 29.2744761250429 1.21763199778562
24 106 cotranslational protein targeting to membrane
GO:0045047 8.81679103035034e-25 29.2744761250429 1.21763199778562
24 106 protein targeting to ER
GO:0072599 8.81679103035034e-25 29.2744761250429 1.21763199778562
24 106 establishment of protein localization to endoplasmic reticulum
GO:0070972 1.4048713517324e-23 25.5157326940366 1.35547712961041
24 118 protein localization to endoplasmic reticulum
GO:0000184 1.74307841946451e-23 25.2453669384729 1.36696422392914
24 119 nuclear-transcribed mRNA catabolic process, nonsense-mediated decay
GO:0006414 2.0059914759924e-23 27.8585195292512 1.20614490346689
23 105 translational elongation
GO:0006413 4.10438586134194e-22 19.7660132908918 1.7460383364473 25
152 translational initiation
GO:0006612 4.85961034177315e-22 19.6102061170213 1.75752543076604
25 153 protein targeting to membrane
GO:0019080 6.81456303131408e-21 18.8417433736276 1.73455124212857
24 151 viral genome expression
GO:0019083 6.81456303131408e-21 18.8417433736276 1.73455124212857
24 151 viral transcription
GO:0000956 1.06276498205865e-19 16.4817872753764 1.94131893986575
24 169 nuclear-transcribed mRNA catabolic process
GO:0043624 1.726210050884e-19 17.5129101667563 1.75752543076604
23 153 cellular protein complex disassembly
GO:0043241 3.65285913707503e-19 16.8583268583269 1.8149609023597 23
158 protein complex disassembly
GO:0006402 3.709521462815e-19 15.508688494604 2.04470278873434 24
178 mRNA catabolic process
GO:0034623 3.38665676251778e-18 15.0549715185477 1.99875441145941
23 174 cellular macromolecular complex disassembly
GO:0032984 6.47404941193852e-18 14.5672852788237 2.05618988305308
23 179 macromolecular complex disassembly
GO:0072594 9.45973896564975e-18 13.2441314553991 2.34336724102138
24 204 establishment of protein localization to organelle
GO:0006401 1.06119106486774e-17 13.1700256789355 2.35485433534011
24 205 RNA catabolic process
GO:0019058 1.71046442166792e-16 11.4945907328026 2.65351878762715
24 231 viral infectious cycle
GO:0022415 2.55124071533549e-15 10.0613511577942 2.98664452287039
24 260 viral reproductive process
GO:0071845 1.85440489406182e-14 9.11700975081257 3.26233478651996
24 284 cellular component disassembly at cellular level
GO:0022411 2.53208610317863e-14 8.97631241997439 3.30828316379489
24 288 cellular component disassembly

GO:0016032	1.60963128202544e-12	6.3500576896973 5.18067953774825	27
451	viral reproduction
GO:0006605	5.14730290142517e-12	6.01332625175028	5.44488270707909
27	474	protein targeting
GO:0033365	2.95186882341796e-11	5.7492489613295 5.43339561276036	26
473	protein localization to organelle
GO:0042273	7.73650233766217e-06	44.0648148148148	0.137845131824787
4	12	ribosomal large subunit biogenesis
GO:0042254	8.44832163588241e-06	6.52370411363217	1.71157705349111
10	149	ribosome biogenesis
GO:0022613	1.11906352074409e-05	5.1479787817816 2.58459622171476	12
225	ribonucleoprotein complex biogenesis
Tissue: Artery_Tibial=>Thyroid	Type:	cluster
SourceGene:	AMPD3(ENSG00000133805.10)
TargetGeneSet:	RPL22	ICMT	RP11	RP3	WNT4	RPL11	KDM4A	RPS8	IPP
PGM1	FPGT	GNG5	EVI5	RPL5	ABCD3	PSMA5	RPS27	RGS8	NAV1
IPO9-AS1		CICP13	ACBD3	B3GALNT2		TRIM58	RPS7	DDX1	CIB4
KCNK3	DHX57	SRBD1	UBC	AAK1	PAX8	MAP3K2	METTL8	OLA1	NBEAL1
CREB1	PSMD1	GRM7	ATP2B2	TMPPE	DCLK3	RPL14	CTNNB1	RPL29	CRYBG3
RPL24	HGD	ZNF148	COPG1	METTL15P1		PSMD2	C3orf70	MB21D2	MIR943
TAPT1	SCFD2	THAP6	SEC31A	AGPAT9	RPS3A	TMA16	STOX2	SORBS2	ANKRA2	CTD
PRRC1	CAMLG	MTND5P11		PCDHA14	GNB2L1	RP1	MUTED	PHACTR1	AGPAT1
CCND3	FAXC	PDE7B	IFNGR1	LATS1	TIAM2	TULP4	FAM120B	ELFN1	FBXL18
TMEM60	RPS3AP26		CAV1	CEP41	RPS20	YTHDF3	FAM82B	EIF3E	FER1L6
AK3P2	PUF60	RPS6	VCP	TSTD2	TGFBR1	CTNNAL1	RPL7A	SEC16A	CCNY
HKDC1	HK1	TSPAN15	C10orf35		VDAC2	RPS3AP5	ALDH18A1		ENTPD7	GBF1
MXI1	SEC23IP	RPL27A	ST5	PLEKHA7	MUC15	METTL15	AMBRA1	ARHGAP1	CYBASC3
FADS2	U6	SNHG1	DRAP1	TSGA10IP		NDUFV1	CCND1	CAPN5	PAK1
ARHGAP20		RPS12P21		RPS25	PKNOX2	NRIP2	COPS7A	CYP27B1	CCDC65
PFDN5	CBX5	CAND1	NAP1L1	LTA4H	SLC9A7P1		SYCP3	KCTD10	RPL6
NOC4L	ANKRD26P4		N4BP2L1	TPT1	LMO7	GRK1	SALL2	CPNE6	TSSK6
LINC00517		MGAT2	GNPNAT1	VTI1B	FAM181A-AS1		FAM181A	WDR25	SEC11A	DET1
GLYR1	BFAR	RPS15A	7SK	CHMP1A	POLR2A	RPL26	ZNF29P	TOM1L2	RPS7P1
RPL23A	RASL10B	ZNHIT3	RPL17	RPL19	CNP	UBTF	RPS7P11	CD300C	TNRC6C
BAHCC1	MAPRE2	MYO5B	ADNP2	ZNF562	ZNF846	NOTCH3	SLC35E1	ARMC6	RPSAP58	OPA3
RSPH6A	FPR3	ZNF578	AP5S1	RRBP1	PYGB	COX4I2	FOXS1	PPIAP3	ZNFX1
SNAI1	ADNP	PMEPA1	GNAS	SLCO4A1	SOX18	WRB	ZNF70	TTC28	SH3BP1
SLC35A2	IGBP1	CXCR3	NHSL2	RPS4X	FGF16	LPAR4	FRMPD3	XIAP
GOBPID	Pvalue	OddsRatio	ExpCount	Count	Size	Term
GO:0006415	5.49707574372244e-25	33.6272110242698	1.04532558300464
23	91	translational termination
GO:0006614	6.885869606049e-25	29.6379760041732	1.20614490346689
24	105	SRP-dependent cotranslational protein targeting to membrane
GO:0006613	8.81679103035034e-25	29.2744761250429	1.21763199778562
24	106	cotranslational protein targeting to membrane
GO:0045047	8.81679103035034e-25	29.2744761250429	1.21763199778562
24	106	protein targeting to ER
GO:0072599	8.81679103035034e-25	29.2744761250429	1.21763199778562
24	106	establishment of protein localization to endoplasmic reticulum
GO:0070972	1.4048713517324e-23	25.5157326940366	1.35547712961041
24	118	protein localization to endoplasmic reticulum
GO:0000184	1.74307841946451e-23	25.2453669384729	1.36696422392914
24	119	nuclear-transcribed mRNA catabolic process, nonsense-mediated decay
GO:0006414	2.0059914759924e-23	27.8585195292512	1.20614490346689
23	105	translational elongation
GO:0006413	4.10438586134194e-22	19.7660132908918	1.7460383364473 25
152	translational initiation
GO:0006612	4.85961034177315e-22	19.6102061170213	1.75752543076604
25	153	protein targeting to membrane
GO:0019080	6.81456303131408e-21	18.8417433736276	1.73455124212857
24	151	viral genome expression
GO:0019083	6.81456303131408e-21	18.8417433736276	1.73455124212857
24	151	viral transcription

GO:0000956	1.06276498205865e-19	16.4817872753764	1.94131893986575
24	169	nuclear-transcribed mRNA catabolic process
GO:0043624	1.726210050884e-19	17.5129101667563	1.75752543076604
23	153	cellular protein complex disassembly
GO:0043241	3.65285913707503e-19	16.8583268583269	1.8149609023597 23
158	protein complex disassembly
GO:0006402	3.709521462815e-19	15.508688494604 2.04470278873434	24
178	mRNA catabolic process
GO:0034623	3.38665676251778e-18	15.0549715185477	1.99875441145941
23	174	cellular macromolecular complex disassembly
GO:0032984	6.47404941193852e-18	14.5672852788237	2.05618988305308
23	179	macromolecular complex disassembly
GO:0072594	9.45973896564975e-18	13.2441314553991	2.34336724102138
24	204	establishment of protein localization to organelle
GO:0006401	1.06119106486774e-17	13.1700256789355	2.35485433534011
24	205	RNA catabolic process
GO:0019058	1.71046442166792e-16	11.4945907328026	2.65351878762715
24	231	viral infectious cycle
GO:0022415	2.55124071533549e-15	10.0613511577942	2.98664452287039
24	260	viral reproductive process
GO:0071845	1.85440489406182e-14	9.11700975081257	3.26233478651996
24	284	cellular component disassembly at cellular level
GO:0022411	2.53208610317863e-14	8.97631241997439	3.30828316379489
24	288	cellular component disassembly
GO:0016032	1.60963128202544e-12	6.3500576896973 5.18067953774825	27
451	viral reproduction
GO:0006605	5.14730290142517e-12	6.01332625175028	5.44488270707909
27	474	protein targeting
GO:0033365	2.95186882341796e-11	5.7492489613295 5.43339561276036	26
473	protein localization to organelle
GO:0042273	7.73650233766217e-06	44.0648148148148	0.137845131824787
4	12	ribosomal large subunit biogenesis
GO:0042254	8.44832163588241e-06	6.52370411363217	1.71157705349111
10	149	ribosome biogenesis
GO:0022613	1.11906352074409e-05	5.1479787817816 2.58459622171476	12
225	ribonucleoprotein complex biogenesis
Tissue: Artery_Tibial=>Thyroid	Type:	asymmetric
SourceGene:	C19orf66(ENSG00000130813.12)
TargetGeneSet:	RPL22	PNRC2	TMEM57	PTAFR	DNAJC8	TAF12	RPS8	NASP
CMPK1	ST6GALNAC3	NEDD4	GNG5	RPL5	RP11	JTB	RPS27	C1orf43
IVNS1ABP	SERTAD4	GGPS1	SH3YL1	SRSF7	UBC	CCDC104	CCT4	SSB	OLA1
HSPD1	CASP10	MFF	UBE2F	ING5	JAGN1	RPL15	RPL14	IFRD2	RPL29	NEK4
RPL24	SEMA5B	CNBP	PIK3CB	RPL22L1	LBP	MRFAP1	CC2D2A	CCNI	MAPK10
H2AFZ	ANK2	RPS3A	SORBS2	PLEKHG4B	NDUFAF2	SLC30A5	BTF3	NSA2
HINT1	PAIP2	CTB	BOD1	ZNF354B	BPHL	DEK	HLA-H	HIST1H2BK
RPS10	C6orf106	RPL10A	BRPF3	EEF1A1	PNISR	AKD1	MRPS12	C6orf70
HOXA-AS3	FKBP9	C7orf23	CDK14	RPS3AP26	FAM185A	HGSNAT	RPS20	E2F5
GS1	EIF3E	EIF3H	EEF1D	AK3	FREM1	RPS6	RFK	RBM18	RPL35
GARNL3	RPL12	SET	NKX2-1	EEF1A1P5	RPL7A	DCLRE1C	WAC	P4HA1
VDAC2	RPS3AP5	MINPP1	MRPL43	KAZALD1	TIAL1	HTRA1	RASSF7	OLFML1	RPL27A	IPO7
C11orf58	SERGEF	SNHG1	SLC25A45	CAPN5	C11orf75	TAF1D
SCN4B	RPS25	FAM118B	SLC6A12	SLC6A13	COPS7A	FGFR1OP2	MED21	MBD6
RPL14P1	NAP1L1	CHPT1	RPLP0	C12orf65	RP13	MRP63	C1QTNF9-AS1	TPT1
PIBF1	FAM155A	LRRC16B	BAZ1A	VTI1B	CDAN1	SLTM	RPS3AP6	FAM96A	RSL1D1
SNX29	BFAR	NPIP	RPS15A	BCKDK	KAT8	PLCG2	MNT	FXR2	RPL26
ZNF18	C17orf108	RPL23A	RPL19	ASB16	TMUB2	RPS7P11	UTP18	RPL17
ADNP2	RPS15	OAZ1	CAMSAP3	EIF3G	DHPS	RPL18	RPL13A	RPS9	RPS5
BFSP1	AHCY	C22orf39	SEPT5	EIF3L	PLXNB2	MED14	IGBP1	RPS4X
EIF4BP7	FAM3A
GOBPID	Pvalue	OddsRatio	ExpCount	Count	Size	Term
GO:0006414	2.36050348003758e-40	53.8825757575758	1.11895370562591
33	105	translational elongation
GO:0006415	3.48190877685576e-39	59.8031165311653	0.96975987820912

31	91	translational termination
GO:0006413	4.94510631537467e-39	37.2966101694915	1.61981869766798
36	152	translational initiation
GO:0000184	2.7413336646545e-38	45.0665961945032	1.2681475330427	33
119	nuclear-transcribed mRNA catabolic process, nonsense-mediated decay
GO:0006614	6.23447663693387e-37	48.4413315754779	1.11895370562591
31	105	SRP-dependent cotranslational protein targeting to membrane
GO:0006613	8.73826405145199e-37	47.7920867208672	1.12961040758425
31	106	cotranslational protein targeting to membrane
GO:0045047	8.73826405145199e-37	47.7920867208672	1.12961040758425
31	106	protein targeting to ER
GO:0072599	8.73826405145199e-37	47.7920867208672	1.12961040758425
31	106	establishment of protein localization to endoplasmic reticulum
GO:0070972	8.93874122208456e-37	43.3427373236752	1.25749083108435
32	118	protein localization to endoplasmic reticulum
GO:0000956	9.20926500736885e-33	28.3977272727273	1.8009826309598	33
169	nuclear-transcribed mRNA catabolic process
GO:0006402	5.67781830737457e-32	26.6181818181818	1.89689294858487
33	178	mRNA catabolic process
GO:0019080	1.67542810010713e-31	29.7755420054201	1.60916199570964
31	151	viral genome expression
GO:0019083	1.67542810010713e-31	29.7755420054201	1.60916199570964
31	151	viral transcription
GO:0006612	2.59571371316958e-31	29.2832866853259	1.63047539962632
31	153	protein targeting to membrane
GO:0043624	2.59571371316958e-31	29.2832866853259	1.63047539962632
31	153	cellular protein complex disassembly
GO:0034623	6.9257309933436e-31	26.1463865158162	1.85426614075151
32	174	cellular macromolecular complex disassembly
GO:0043241	7.53752324109567e-31	28.1204788425837	1.68375890941803
31	158	protein complex disassembly
GO:0032984	1.80086583217337e-30	25.2481320397011	1.90754965054322
32	179	macromolecular complex disassembly
GO:0072594	6.31783128145094e-30	22.5295055821372	2.17396719950176
33	204	establishment of protein localization to organelle
GO:0006401	7.46800371641796e-30	22.3969344608879	2.18462390146011
33	205	RNA catabolic process
GO:0019058	8.21654898857364e-27	18.5820907817777	2.461698152377	32
231	viral infectious cycle
GO:0022415	2.20499404397268e-26	16.9042851421706	2.77074250916892
33	260	viral reproductive process
GO:0071845	6.06660650136629e-24	14.6187874056727	3.02650335616912
32	284	cellular component disassembly at cellular level
GO:0022411	9.42250704533257e-24	14.3862704918033	3.06913016400249
32	288	cellular component disassembly
GO:0016032	9.6487085624725e-21	9.81405542986425	4.80617258321223
35	451	viral reproduction
GO:0033365	4.5441610743182e-20	9.30633897394574	5.04062002629576
35	473	protein localization to organelle
GO:0006605	4.49057635309671e-19	8.92306818181818	5.0512767282541	34
474	protein targeting
GO:0042254	5.49616685618282e-07	7.89241917502787	1.58784859179296
11	149	ribosome biogenesis
GO:0022613	5.18549968006423e-06	5.5877802023408	2.39775794062695	12
225	ribonucleoprotein complex biogenesis
GO:0071843	9.58602691291293e-06	5.2379475088416	2.54695176804373	12
239	cellular component biogenesis at cellular level
GO:0006364	1.50371048294149e-05	8.10559360730594	1.10829700366757
8	104	rRNA processing
Tissue: Artery_Tibial=>Thyroid	Type:	asymmetric
SourceGene:	C3orf37(ENSG00000183624.8)
TargetGeneSet:	ATAD3C	RPL22	RP11	C1orf100	CHMP3	MRPS25	NEK10
RPL10AP6	EIF4E3	SLC15A2	ATP13A4-AS1	WDR19	RAP1GDS1	CTB

RPL10A KLHDC3 MED23 RP1 INHBA RPS20 RPL7 RPL30 RPL13AP5 SSH3
RPS12P21 CREBL2 SIPA1L1 ANP32BP1 ODF3L1 JMJD5 MT1G ZDHHC1
C16orf7 RPL23A RPL17 RPL19 SDK2 KIAA1328 RPL18A RPS11 RPS5
C20orf196 ZC3H7B SHROOM2 MCF2 VDAC1P6
GOBPID Pvalue OddsRatio ExpCount Count Size Term
GO:0006415 8.40751245877888e-17 85.7103260869565 0.214102830253962
11 91 translational termination
GO:0006414 4.33612779750617e-16 72.8737280296022 0.24704172721611
11 105 translational elongation
GO:0006614 4.33612779750617e-16 72.8737280296022 0.24704172721611
11 105 SRP-dependent cotranslational protein targeting to membrane
GO:0006613 4.83110592468598e-16 72.1016018306636 0.249394505570549
11 106 cotranslational protein targeting to membrane
GO:0045047 4.83110592468598e-16 72.1016018306636 0.249394505570549
11 106 protein targeting to ER
GO:0072599 4.83110592468598e-16 72.1016018306636 0.249394505570549
11 106 establishment of protein localization to endoplasmic reticulum
GO:0043624 5.75205039824041e-16 55.2263056092843 0.359975088229188
12 153 cellular protein complex disassembly
GO:0043241 8.52251785682823e-16 53.3163138231631 0.371738980001384
12 158 protein complex disassembly
GO:0070972 1.63376664072382e-15 63.9618041446566 0.277627845823818
11 118 protein localization to endoplasmic reticulum
GO:0000184 1.79752529619068e-15 63.3651368760064 0.279980624178258
11 119 nuclear-transcribed mRNA catabolic process, nonsense-mediated decay
GO:0034623 2.75998083956882e-15 47.996632996633 0.40938343367241 12
174 cellular macromolecular complex disassembly
GO:0032984 3.89294897056301e-15 46.5432770821992 0.421147325444606
12 179 macromolecular complex disassembly
GO:0019080 2.61144042545839e-14 48.7723602484472 0.35526953152031
11 151 viral genome expression
GO:0019083 2.61144042545839e-14 48.7723602484472 0.35526953152031
11 151 viral transcription
GO:0006413 2.81102848889924e-14 48.4230650632131 0.357622309874749
11 152 translational initiation
GO:0006612 3.02432840322377e-14 48.0786895284752 0.359975088229188
11 153 protein targeting to membrane
GO:0000956 9.14113499326756e-14 43.1615299944964 0.397619541900215
11 169 nuclear-transcribed mRNA catabolic process
GO:0006402 1.62388034869741e-13 40.8096849778703 0.418794547090167
11 178 mRNA catabolic process
GO:0072594 7.29402674082471e-13 35.2475782833972 0.479966784305584
11 204 establishment of protein localization to organelle
GO:0006401 7.69623482128468e-13 35.0634244733303 0.482319562660024
11 205 RNA catabolic process
GO:0071845 9.82304026513999e-13 28.3656417112299 0.668189052660715
12 284 cellular component disassembly at cellular level
GO:0022411 1.15902680630502e-12 27.9466403162055 0.677600166078472
12 288 cellular component disassembly
GO:0019058 2.84176510755388e-12 30.8630434782609 0.543491799875441
11 231 viral infectious cycle
GO:0022415 1.02754949058485e-11 27.2128514056225 0.611722372154176
11 260 viral reproductive process
GO:0016032 3.62519974997775e-09 15.1923913043478 1.06110303785205
11 451 viral reproduction
GO:0033365 5.95747487152447e-09 14.4461697722567 1.11286416164971
11 473 protein localization to organelle
GO:0006605 6.08988502832755e-09 14.4139355808057 1.11521694000415
11 474 protein targeting
Tissue: Thyroid=>Artery_Tibial Type: asymmetric
SourceGene: CDC37P1(ENSG00000259982.1)
TargetGeneSet: RNASEL PPP1R15B CNIH3 RPS7 MSH2 MZT2B MZT2A
COL3A1 SATB2 ZNF860 CCR5 RPL29 ZNF721 BEND4 RPL34 RPS3A FYB

RPS14 EDN1 RPS18 RPS10 ZFAND2A COL1A2 RP11 ENY2 FAM69B SNHG7
TSSC4 RPL27A RPS13 CTD FAU CCDC85B CTSC YEATS4 CCDC59 RPL18AP3
GIT2 ARL6IP4 COL4A2 TRAV18 CATSPERB BNIP2 RPS3AP6 HMGN1P38
CDC37P1 ELMO3 ZFPM1 RPL13 RPL27 PLIN3 RPL36 ZNF358 C19orf43
FXYD1 ZNF567 DEDD2 RPS11 RRAS CACNG6 RPS9 PPP1R12C ZNF579
ZNF865 RPS5 MAP1LC3A PKIG PCIF1 NEFH

GOBPID	Pvalue	OddsRatio	ExpCount	Count	Size	Term
GO:0006415	2.65957599051324e-23	80.4042105263158	0.340045671579821	16	91	translational termination
GO:0006414	3.08734828789197e-22	67.6901241868717	0.392360390284409	16	105	translational elongation
GO:0006614	3.08734828789197e-22	67.6901241868717	0.392360390284409	16	105	SRP-dependent cotranslational protein targeting to membrane
GO:0006613	3.62715533822434e-22	66.9333333333333	0.396097155906166	16	106	cotranslational protein targeting to membrane
GO:0045047	3.62715533822434e-22	66.9333333333333	0.396097155906166	16	106	protein targeting to ER
GO:0072599	3.62715533822434e-22	66.9333333333333	0.396097155906166	16	106	establishment of protein localization to endoplasmic reticulum
GO:0070972	2.22306795689632e-21	59.0092879256966	0.440938343367241	16	118	protein localization to endoplasmic reticulum
GO:0000184	2.5619983674375e-21	58.4322943280531	0.444675108988997	16	119	nuclear-transcribed mRNA catabolic process, nonsense-mediated decay
GO:0019080	1.34729230239709e-19	44.4818713450292	0.564251608885198	16	151	viral genome expression
GO:0019083	1.34729230239709e-19	44.4818713450292	0.564251608885198	16	151	viral transcription
GO:0019058	1.38823242073223e-19	33.2957746478873	0.863192858625701	18	231	viral infectious cycle
GO:0006413	1.50203774440156e-19	44.1517027863777	0.567988374506954	16	152	translational initiation
GO:0006612	1.67326985126662e-19	43.8263542066846	0.571725140128711	16	153	protein targeting to membrane
GO:0043624	1.67326985126662e-19	43.8263542066846	0.571725140128711	16	153	cellular protein complex disassembly
GO:0043241	2.83883936612228e-19	42.2683469236471	0.590408968237492	16	158	protein complex disassembly
GO:0006401	5.8075238810556e-19	34.7258481886141	0.766036952460037	17	205	RNA catabolic process
GO:0000956	8.5466593718169e-19	39.1991744066047	0.631513390076811	16	169	nuclear-transcribed mRNA catabolic process
GO:0022415	1.1761615366002e-18	29.245867768595	0.971559061656633	18	260	viral reproductive process
GO:0034623	1.37529854420929e-18	37.9453697534977	0.650197218185593	16	174	cellular macromolecular complex disassembly
GO:0006402	1.99128888750454e-18	36.9980506822612	0.665144280672618	16	178	mRNA catabolic process
GO:0032984	2.18128476970743e-18	36.7684856312561	0.668881046294374	16	179	macromolecular complex disassembly
GO:0072594	1.81001713496122e-17	31.8230683090705	0.762300186838281	16	204	establishment of protein localization to organelle
GO:0071845	1.48037041826462e-16	24.315214090495	1.06124143657878	17	284	cellular component disassembly at cellular level
GO:0022411	1.87222646465371e-16	23.9495362521193	1.07618849906581	17	288	cellular component disassembly
GO:0016032	1.94146036038663e-14	16.1247113163972	1.68528129541208	18	451	viral reproduction
GO:0033365	4.40832093871633e-14	15.3208791208791	1.76749013909072	18	473	protein localization to organelle
GO:0006605	4.57120842919428e-14	15.2861842105263	1.77122690471248	18	474	protein targeting

Tissue: Thyroid=>Artery_Tibial Type: asymmetric
SourceGene: ENSG00000161149.7

TargetGeneSet: RP11 ZNF683 MIR4426 U6 RPL7AP11 SEC62 RPL7AP31
RPL34 RPL37 RPS23 CTD RPS14 RPS10 RP3 RPL24P4 RPL39P3 RP1
MRPS12 TOMM7 RP9 CTB INSIG1 STMN4 UQCRB LDHAP4 RPS6 EDF1 SNCG
NHP2P1 NDUFB8 USMG5 RPLP2 FAU KCNJ5 CYP27B1 RPS11P5 SAP18 RPS7P10 LMO7
RPS29 RPL7AP6 NDUFB1 RPL3P4 MIR1282 EMP2 FHOD1 CCL3L1 RPL27 RPL38
SNRPEP4 RPL36 C19orf79 RPS16 RPS19 SNRPD2 RPS11 NDUFA3 RPL28 RPS5
TOMM22 EEF1B2P3 RP13

GOBPID	Pvalue	OddsRatio	ExpCount	Count	Size	Term
GO:0006613	4.35162316180297e-28	117.163636363636	0.315410698221576	18	106	cotranslational protein targeting to membrane
GO:0006415	2.93000940157936e-27	126.651767151767	0.270777108850599	17	91	translational termination
GO:0006612	6.03872468444355e-27	84.330223880597	0.455262611583973	19	153	protein targeting to membrane
GO:0006414	4.08057203539776e-26	106.398601398601	0.312435125596844	17	105	translational elongation
GO:0006614	4.08057203539776e-26	106.398601398601	0.312435125596844	17	105	SRP-dependent cotranslational protein targeting to membrane
GO:0045047	4.85169505409732e-26	105.195764909248	0.315410698221576	17	106	protein targeting to ER
GO:0072599	4.85169505409732e-26	105.195764909248	0.315410698221576	17	106	establishment of protein localization to endoplasmic reticulum
GO:0070972	3.40182530046741e-25	92.6195734958111	0.351117569718359	17	118	protein localization to endoplasmic reticulum
GO:0000184	3.96200299049282e-25	91.7051282051282	0.35409314234309	17	119	nuclear-transcribed mRNA catabolic process, nonsense-mediated decay
GO:0072594	1.77855423505988e-24	60.8641891891892	0.607016815445298	19	204	establishment of protein localization to organelle
GO:0019080	2.8000847532111e-23	69.6492537313433	0.44931146633451	17	151	viral genome expression
GO:0019083	2.8000847532111e-23	69.6492537313433	0.44931146633451	17	151	viral transcription
GO:0006413	3.14728091390126e-23	69.12849002849	0.452287038959242	17	152	translational initiation
GO:0043624	3.53461810008498e-23	68.6153846153846	0.455262611583973	17	153	cellular protein complex disassembly
GO:0043241	6.24002465385604e-23	66.1590289143481	0.470140474707633	17	158	protein complex disassembly
GO:0000956	2.04168648612479e-22	61.3238866396761	0.502871773579683	17	169	nuclear-transcribed mRNA catabolic process
GO:0034623	3.40610328996313e-22	59.3500734933856	0.517749636703342	17	174	cellular macromolecular complex disassembly
GO:0006402	5.07242152605792e-22	57.859292881032	0.52965192720227	17	178	mRNA catabolic process
GO:0032984	5.59508439791447e-22	57.4981006647673	0.532627499827002	17	179	macromolecular complex disassembly
GO:0006401	5.94451087059195e-21	49.4558101472995	0.60999238807003	17	205	RNA catabolic process
GO:0019058	4.6818076760367e-20	43.3677210639827	0.687357276313058	17	231	viral infectious cycle
GO:0022415	3.56191690710266e-19	38.1141183918962	0.773648882430282	17	260	viral reproductive process
GO:0006605	6.6909496371469e-19	26.7266807125072	1.4104214241229	20	474	protein targeting
GO:0071845	1.60523228930624e-18	34.6293575338519	0.845062625423846	17	284	cellular component disassembly at cellular level
GO:0022411	2.03608818542751e-18	34.1085722395686	0.856964915922773	17	288	cellular component disassembly
GO:0033365	1.65005985964397e-17	24.3324155653451	1.40744585149817	19	473	protein localization to organelle
GO:0016032	3.74552213027413e-15	21.0526409074796	1.34198325375407	17	451	viral reproduction
GO:0042274	2.01711012928941e-07	105.450549450549	0.0535603072451733			

4	18	ribosomal small subunit biogenesis
GO:0042773	1.10436984124632e-05	34.2635658914729	0.139851913362397
4	47	ATP synthesis coupled electron transport
GO:0042775	1.10436984124632e-05	34.2635658914729	0.139851913362397
4	47	mitochondrial ATP synthesis coupled electron transport
Tissue: Artery_Tibial=>Thyroid	Type:	asymmetric
SourceGene:	FAM198A(ENSG00000144649.4)
TargetGeneSet:	MRPL20	TMEM52	PEX10	RPL11	RP11	PSMB4	UBC	RPL37A
RPL32	MUSTN1	FAM19A1	ALG1L	RPS14	C6orf48	TCTE1	TOMM7	RPL30	RPL13AP5
EIF3M	FAU	CTD	PHB2	ATP5G2	WDR90	TCEB2	POLR2C	RPS12P27
CENPV	RPL17	RPL27	RPS15	XAB2	SNRPD2	RPS11	RP4	CRYZL1	MRPL40
MAGT1
GOBPID	Pvalue	OddsRatio	ExpCount	Count	Size	Term
GO:0019080	6.1554212472483e-19	84.0996376811594	0.303024012179088
13	151	viral genome expression
GO:0019083	6.1554212472483e-19	84.0996376811594	0.303024012179088
13	151	viral transcription
GO:0006415	1.04293495901498e-17	109.556944444444	0.182617119922497
11	91	translational termination
GO:0072594	3.35013978170808e-17	60.5376308900524	0.40938343367241
13	204	establishment of protein localization to organelle
GO:0006413	5.25490439834157e-17	72.0100840336135	0.305030793716698
12	152	translational initiation
GO:0006414	5.40296037411533e-17	93.1489361702128	0.210712061449035
11	105	translational elongation
GO:0006614	5.40296037411533e-17	93.1489361702128	0.210712061449035
11	105	SRP-dependent cotranslational protein targeting to membrane
GO:0006613	6.02164137774053e-17	92.1619883040936	0.212718842986645
11	106	cotranslational protein targeting to membrane
GO:0045047	6.02164137774053e-17	92.1619883040936	0.212718842986645
11	106	protein targeting to ER
GO:0072599	6.02164137774053e-17	92.1619883040936	0.212718842986645
11	106	establishment of protein localization to endoplasmic reticulum
GO:0019058	1.71709935812202e-16	52.9392201834862	0.463566535187876
13	231	viral infectious cycle
GO:0070972	2.04419473587998e-16	81.757528556594	0.236800221437963	11
118	protein localization to endoplasmic reticulum
GO:0000184	2.2498105684425e-16	80.9948559670782	0.238807002975573
11	119	nuclear-transcribed mRNA catabolic process, nonsense-mediated decay
GO:0022415	8.05900844682899e-16	46.6282894736842	0.521763199778562
13	260	viral reproductive process
GO:0006612	3.82665601067179e-15	61.4553990610329	0.307037575254308
11	153	protein targeting to membrane
GO:0043624	3.82665601067179e-15	61.4553990610329	0.307037575254308
11	153	cellular protein complex disassembly
GO:0043241	5.48420494375765e-15	59.3442932728647	0.317071482942357
11	158	protein complex disassembly
GO:0000956	1.16255646827721e-14	55.1701828410689	0.339146079856065
11	169	nuclear-transcribed mRNA catabolic process
GO:0034623	1.60854144364843e-14	53.459100204499	0.349179987544115	11
174	cellular macromolecular complex disassembly
GO:0006402	2.0711872234438e-14	52.164005322688	0.357207113694554	11
178	mRNA catabolic process
GO:0032984	2.20425535631333e-14	51.8498677248677	0.359213895232164
11	179	macromolecular complex disassembly
GO:0016032	3.44467018415915e-14	29.8688024408848	0.905058473462044
14	451	viral reproduction
GO:0006401	9.9014971193746e-14	44.8190148911798	0.41139021521002
11	205	RNA catabolic process
GO:0033365	1.76897757278393e-12	24.6611413043478	0.949207667289461
13	473	protein localization to organelle
GO:0006605	1.81693803399836e-12	24.6058839479393	0.951214448827071
13	474	protein targeting

GO:0071845	3.5201848940134e-12	31.6725681725682	0.569925956681198
11	284	cellular component disassembly at cellular level
GO:0022411	4.09790316936296e-12	31.2063778580024	0.577953082831638
11	288	cellular component disassembly
Tissue: Thyroid=>Artery_Tibial	Type:	asymmetric
SourceGene:	HEBP2(ENSG00000051620.5)
TargetGeneSet:	TRNAU1AP	RPS8	RP11	HSPB11	RPS27	UBC	MZT2B
MZT2A	RPL15	DAG1	RPL29	CEP70	RPS3A	SAP30	CCNO	RPS14	RGL2
RPS10	FAM135A	RPL7A	RPS13	EHD1	FAU	NDUFA4L2	NUDT4	RPL18AP3
SIVA1	MT2A	RPL13	SNORD3A	RPL23A	RPL27	RPL17	RPL36	CTD	RPL18A
ZNF461	RPS16	ITPKC	RPS19	OPA3	RPS11	A1BG	RPS5	ZNF133	MICALL1
C22orf23	RPS19BP1	RPS23P8	RPS4X
GOBPID	Pvalue	OddsRatio	ExpCount	Count	Size	Term
GO:0006414	1.68042319824457e-40	251.211890243902	0.283371392983185
23	105	translational elongation
GO:0006415	1.15013307802615e-39	269.007672634271	0.245588540585427
22	91	translational termination
GO:0006614	3.9270726550027e-38	223.41459957477	0.283371392983185	22
105	SRP-dependent cotranslational protein targeting to membrane
GO:0006613	4.94997699629574e-38	220.739495798319	0.286070168154453
22	106	cotranslational protein targeting to membrane
GO:0045047	4.94997699629574e-38	220.739495798319	0.286070168154453
22	106	protein targeting to ER
GO:0072599	4.94997699629574e-38	220.739495798319	0.286070168154453
22	106	establishment of protein localization to endoplasmic reticulum
GO:0070972	6.66930706644978e-37	192.985294117647	0.318455470209674
22	118	protein localization to endoplasmic reticulum
GO:0000184	8.17265548794994e-37	190.98241358399	0.321154245380943	22
119	nuclear-transcribed mRNA catabolic process, nonsense-mediated decay
GO:0019080	2.3584611271864e-34	143.285909712722	0.407515050861532
22	151	viral genome expression
GO:0019083	2.3584611271864e-34	143.285909712722	0.407515050861532
22	151	viral transcription
GO:0006413	2.75445166416166e-34	142.173755656109	0.4102138260328	22
152	translational initiation
GO:0006612	3.21337533899315e-34	141.078581050741	0.412912601204069
22	153	protein targeting to membrane
GO:0043624	3.21337533899315e-34	141.078581050741	0.412912601204069
22	153	cellular protein complex disassembly
GO:0043241	6.83317452860222e-34	135.844290657439	0.426406477060411
22	158	protein complex disassembly
GO:0000956	3.29301516299787e-33	125.582232893157	0.456093003944364
22	169	nuclear-transcribed mRNA catabolic process
GO:0034623	6.49128334689652e-33	121.40866873065	0.469586879800706	22
174	cellular macromolecular complex disassembly
GO:0006402	1.10059555255609e-32	118.262443438914	0.48038198048578
22	178	mRNA catabolic process
GO:0032984	1.25339068946911e-32	117.500936680405	0.483080755657048
22	179	macromolecular complex disassembly
GO:0072594	2.56527150823439e-31	101.182934712346	0.550550134938759
22	204	establishment of protein localization to organelle
GO:0006401	2.87038799609198e-31	100.622950819672	0.553248910110027
22	205	RNA catabolic process
GO:0022415	8.03852694519248e-31	85.9770569620253	0.70168154452979
23	260	viral reproductive process
GO:0019058	4.41503190645081e-30	87.9442724458204	0.623417064563006
22	231	viral infectious cycle
GO:0071845	4.76474345512123e-28	69.892231701841	0.766452148640232	22
284	cellular component disassembly at cellular level
GO:0022411	6.52897562544952e-28	68.8217602830606	0.777247249325306
22	288	cellular component disassembly
GO:0016032	3.19356796554016e-25	46.9672897196262	1.21714760224206
23	451	viral reproduction

GO:0033365 9.57811070796263e-25 44.6008333333333 1.27652065600996
23 473 protein localization to organelle
GO:0006605 1.00555545696715e-24 44.4987527716186 1.27921943118123
23 474 protein targeting
GO:0042274 1.34855588817041e-07 117.534693877551 0.0485779530828316
4 18 ribosomal small subunit biogenesis
Tissue: Artery_Tibial=>Thyroid Type: asymmetric
SourceGene: HES1(ENSG00000114315.3)
TargetGeneSet: UBE2J2 DFFB ERRFI1 DNAJC16 EPHA2 ARHGEF19 ARHGEF19-AS1
NECAP2 UBXN10 SRRM1 CNKSR1 XKR8 PTAFR SNRNP40 TMEM39B FAM167B HDAC1 PHC2
ZMYM1 LSM10 SLFNL1 CCDC24 RNF220 C1orf123 TTC22 SYDE2 RP4
PSMA5 ATXN7L2 CEPT1 DDX20 RP11 LIX1L BOLA1 LYSMD1 CHTOP DENND4B SHE
PYGO2 FLAD1 CLK2 CCT3 USP21 SELP CACYBP MRPS14 SMG7 BAP1
PTGS2 BTG2 SOX13 IRF6 RP3 RPS6KC1 MARC1 HLX TP53BP2 PARP1
PYCR2 ZNF678 COG2 C1orf131 TOMM20 ARID4B PGBD2 SLC5A6 PPM1G EHD3
FAM98A PAPOLG RAB1A PCBP1-AS1 ART4 MRPL15 MRPL35 ZNF2 ARID5A
C2orf29 POLR1B SCTR CHN1 CALCRL MOB4 CREB1 IDH1 TMEM169 BCS1L
TTLL4 NHEJ1 SP100 PSMD1 EIF4E2 ASB1 DTYMK DHX8 CHCHD4 C3orf19
METTL6 ACAA1 GORASP1 ZNF619 ZNF620 NME6 NPRL2 DUSP7 PBRM1 SCT
ARL4D MINA ABHD10 QTRTD1 ARHGAP31 POGLUT1 TIMMDC1 ADPRH GTF2E1
PLXNA1 C3orf25 SRPRB SLCO2A1 MSL2 SLC25A36 RNF13 C3orf55 MYNN
ACTL6A ABCF3 ALG3 POLR2H EPHB3 CRYGS TBCCD1 LRRC33 LRCH3 TACC3 LYAR
MRFAP1L1 TBC1D1 NFXL1 SLC10A4 UTP3 COX18 IL8 FAM47E RASGEF1B
CASP6 ANKRD50 MFAP3L STOX2 KLKB1 BRIP1 CTD C5orf51 SNX18 PTCD2
HMGCR ZCCHC9 PHAX HARS2 DIAPH1 MED7 DOK3 RP1 RPP40 TRIM27
PFDN6 ZBTB9 PPIL1 TAF8 CAPN11 AARS2 MTO1 SH3BGRL2 UBE2J1
C6orf203 GOPC MCM9 MAP3K4 PSMG3 ELFN1 MIOS GLCCI1 VOPP1
ZNF107 BAZ1B RUNDC3B CNPY4 SLC12A9 CLDN15 CAV2 LRRC4 METTL2A TNPO3
TRIM24 PRSS37 OR9A3P ZNF786 ZNF398 SMARCD3 CLN8 FUT10 TTI2 CHMP4C
INTS8 GRHL2 TRIB1 ZNF623 EXOSC4 ZNF250 RANBP6 SNAPC3 KLHL9 TEK VCP
STOML2 FAM214B FXN CEP78 BICD2 FOXE1 RNF20 CTNNAL1 C9orf80 ZFP37
TRIM32 DOLPP1 EXOSC2 SLC2A6 SDCCAG3 C9orf142 NPDC1 TUBB4B NRARP
NUDT5 ZFAND4 SYT15 C10orf128 CSTF2T TMEM26 MSS51 AP3M1 ENTPD7 BTRC
SUFU MXI1 STK32C ZNF511 ZNF195 CHRNA10 RRP8 MRPL17 ZNF143 CTR9
MPPED2 CELF1 TMEM216 TTC9C ZBTB3 TMEM223 STIP1 PLCB3 RPS6KA4 MEN1 UCP3
PICALM FAM76B MSANTD4 RDX ARHGAP20 C11orf93 RNF214 DPAGT1
C2CD2L ZNF202 HYLS1 VPS26B RAD52 FKBP4 GNB3 YARS2 BCDIN3D MFSD5
TARBP2 HOXC6 SUOX TIMELESS PPM1H LEMD3 DYRK2 NT5DC3 C12orf34
ANAPC7 DYNLL1 SNRNP35 GOLGA3 SERTM1 KBTBD6 KBTBD7 ITM2B ARL11 SPRYD7
THSD1 LMO7 PCID2 TTC5 RNASE1 DAD1 AJUBA CDH24 ACIN1 MGAT2
SOCS6 BCRP2 DPF3 MLH3 POMT2 BTBD7 RP13 C15orf41 BUB1B
ZFYVE19 LCMT2 SLC24A5 AP4E1 RNF111 PARP16 PTPLAD1 DIS3L SMAD6 CALML4
ANP32A CSK SCAMP2 CHD2 MRPS34 SLC9A3R2 NTHL1 PGPEP1 ERCC4
C16orf88 UBFD1 NDUFAB1 MAZ TAOK2 GDPD3 SULT1A3 TBC1D10B
FBXL19 ZNF668 CNEP1R1 HEATR3 CAPNS2 C16orf57 EXOC3L1 E2F4 CTCF
ZNRF1 PLCG2 MAP1LC3B ZC3H18 CBFA2T3 ZNF276 CENPBD1 RTN4RL1 DERL2
SLC16A13 ARHGEF15 KRBA2 SCO1 ZSWIM7 ALKBH5 LLGL1 TMEM11
C17orf51 CRLF3 CDK5R1 MYO1D ZNF830 PEX12 DHRS11 PIP4K2B LEPREL4 ACLY
RUNDC1 UBTF DLX3 PPP1R9B RSAD1 MBTD1 DGKE TRIM25 SEPT4 DHX40
FAM104A ICT1 HN1 TRIM65 QRICH2 CBX8 SIRT7 LRRC45 ZNF397 MIER2
LMNB2 ZNF555 PIAS4 DPP9 CAPS ELAVL1 DNMT1 ZNF627 ZNF440 ZNF443
ZNF490 WDR83 LYL1 IL27RA SAMD1 SIN3B NR2F6 ANO8 GLT25D1 PIK3R2
RAB3A ZNF91 ANKRD27 KCTD15 LIN37 THAP8 ZFP82 ZNF571 ZNF781 MAP3K10
ZNF526 GEMIN7 OPA3 FBXO46 DMPK MYPOP BAX ALDH16A1 LINC00085
U2AF2 ZNF264 ZNF543 ZNF547 ZNF549 ZNF586 ZSCAN22 ZCCHC3 SOX12 FAM110A
NOP56 PCNA GZF1 C20orf160 COMMD7 DNMT3B RBL1 FITM2 SDC4
ZSWIM3 ZSWIM1 NCOA5 ELMO2 TMEM189 ADNP ZFP64 CSTF1 C20orf151
TCFL5 SOX18 RGS19 RBM11 SNORA81 DSTNP1 CECR5 DGCR8 ZNF74 CRKL HIC2
UBE2L3 ZNF280B ZNF70 SGSM1 RNF215 FBXO7 MGAT3 L3MBTL2 EFCAB6 ZBED4 TLR7
ZNF41 PPP1R3F WNK3 RPL23AP83 FAM123B MAGEC2 BEX4 TCEAL8 AIFM1
NAA10 LAGE3
GOBPID Pvalue OddsRatio ExpCount Count Size Term
GO:0031060 2.1966285289602e-06 22.2522427440633 0.426268078333679

6	16	regulation of histone methylation
Tissue: Artery_Tibial=>Thyroid	Type:	asymmetric
SourceGene:	IGSF6(ENSG00000140749.7)
TargetGeneSet:	SDF4	RPL22	RPL11	RP11	U6	UQCRH	RPL5	TARS2	RPS7
RPL31	OLA1	RPL37A	KCNE4	RPL32	RPL15	RPL14	RPL24	SLC15A2	TBC1D1	CCNI
PRLR	BTF3	FSTL4	CTB	GNB2L1	RPL10A	ELFN1	RPS20	RPL7	C8orf38
RPL30	EIF3E	EIF3H	RPS6	FAM214B	RPL7A	HK1	CLNS1A	ATP5G2	RPL14P1
NAP1L1	RPL6	CCNB1IP1		SEC11A	BFAR	RPS15A	INCA1	RPL26	RPL23A
RPL17	RPL19	RPS7P11	CTD	MAPRE2	RFX1	UBA52	RPSAP58	ALDH16A1
RPS11	SUV420H2		RPL28	ISOC2	GPX1P2	RPSAP9	PLXNB3	RPL10
GOBPID	Pvalue	OddsRatio	ExpCount	Count	Size	Term
GO:0006614	7.9668230342967e-45	183.576923076923	0.392360390284409
27	105	SRP-dependent cotranslational protein targeting to membrane
GO:0006613	1.06702684336943e-44	181.240506329114	0.396097155906166
27	106	cotranslational protein targeting to membrane
GO:0045047	1.06702684336943e-44	181.240506329114	0.396097155906166
27	106	protein targeting to ER
GO:0072599	1.06702684336943e-44	181.240506329114	0.396097155906166
27	106	establishment of protein localization to endoplasmic reticulum
GO:0006415	1.93958496782957e-44	204.742857142857	0.340045671579821
26	91	translational termination
GO:0070972	2.82321154560104e-43	157.208791208791	0.440938343367241
27	118	protein localization to endoplasmic reticulum
GO:0000184	3.64513225043217e-43	155.489130434783	0.444675108988997
27	119	nuclear-transcribed mRNA catabolic process, nonsense-mediated decay
GO:0006414	1.3930963837399e-42	168.294755877034	0.392360390284409
26	105	translational elongation
GO:0006413	4.5023842499722e-42	123.95905707196	0.567988374506954	28
152	translational initiation
GO:0006612	6.4663478484945e-40	113.261904761905	0.571725140128711
27	153	protein targeting to membrane
GO:0000956	1.17612228885946e-38	100.387323943662	0.631513390076811
27	169	nuclear-transcribed mRNA catabolic process
GO:0019080	4.84290716918424e-38	106.020571428571	0.564251608885198
26	151	viral genome expression
GO:0019083	4.84290716918424e-38	106.020571428571	0.564251608885198
26	151	viral transcription
GO:0006402	5.27938708898755e-38	94.3443708609272	0.665144280672618
27	178	mRNA catabolic process
GO:0043624	7.01178526608285e-38	104.33633295838	0.571725140128711	26
153	cellular protein complex disassembly
GO:0043241	1.72853211291102e-37	100.349025974026	0.590408968237492
26	158	protein complex disassembly
GO:0034623	2.5435747712546e-36	89.4000965250965	0.650197218185593
26	174	cellular macromolecular complex disassembly
GO:0072594	2.63709814775386e-36	80.3389830508475	0.762300186838281
27	204	establishment of protein localization to organelle
GO:0006401	3.03156197171403e-36	79.8820224719101	0.766036952460037
27	205	RNA catabolic process
GO:0032984	5.57634709354595e-36	86.4481792717087	0.668881046294374
26	179	macromolecular complex disassembly
GO:0019058	5.98952653037679e-33	64.2843205574913	0.863192858625701
26	231	viral infectious cycle
GO:0022415	1.45271594129644e-31	56.2023809523809	0.971559061656633
26	260	viral reproductive process
GO:0071845	1.54278392957137e-30	50.8878737541528	1.06124143657878
26	284	cellular component disassembly at cellular level
GO:0022411	2.24011454451842e-30	50.0967829880044	1.07618849906581
26	288	cellular component disassembly
GO:0033365	3.31416810940732e-26	31.280269058296	1.76749013909072	27
473	protein localization to organelle
GO:0006605	3.50782138071011e-26	31.2080536912752	1.77122690471248
27	474	protein targeting

GO:0016032	2.92028648718522e-25	30.5270588235294	1.68528129541208
26	451	viral reproduction
GO:0042273	4.67891000109376e-10	209.766763848396	0.0448411874610754
5	12	ribosomal large subunit biogenesis
GO:0022613	9.0147554393954e-09	14.9915433403806	0.840772264895163
10	225	ribonucleoprotein complex biogenesis
GO:0071843	1.60445580432567e-08	14.061135371179 0.893086983599751	10
239	cellular component biogenesis at cellular level
GO:0042254	7.36950037898148e-08	17.5837187789084	0.556778077641686
8	149	ribosome biogenesis
GO:0006364	1.09399988477819e-07	21.9565694231191	0.388623624662653
7	104	rRNA processing
GO:0016072	1.61093682834633e-07	20.6688700681677	0.411044218393191
7	110	rRNA metabolic process
GO:0034660	9.72266698510121e-07	10.2326086956522	1.06497820220054
9	285	ncRNA metabolic process
GO:0034470	8.94038674369838e-06	10.9610847756587	0.747353124351256
7	200	ncRNA processing
Tissue: Artery_Tibial=>Thyroid	Type:	asymmetric
SourceGene:	LYZ(ENSG00000090382.2)
TargetGeneSet:	VWA1	RPL22	RPL5	RP11	KCNC4-AS1	CSDE1	NGF
SPRR1B	RPS27	C1orf43	RGS2	TOMM20	RPS7	CCDC104	OLA1	IHH	C3orf32
RPL15	RPL14	IMPDH2	RPL24	POLQ	GATA2	WDR19	SLC10A4	CCNI	ELMOD2	HHIP
RPS3A	BTF3	CTB	GNB2L1	RPL15P3	HLA-H	RPL10A	RP1	EEF1A1	ELFN1
CYP3A5	LRRC4	CDCA2	DUSP26	RPL7	EIF3E	EIF3H	RPS6	RPL35	SET
EEF1A1P5		RPL7A	WAC	IPO7	RASSF10	TAF6L	EHD1	SAC3D1	MMP3
AMIGO2	RPL14P1	LEMD3	LYZ	NAP1L1	SLC9A7P1		RPL6	POMP	TPT1
CCNB1IP1		C14orf166		SEC11A	RSL1D1	BFAR	RPS15A	MT1F	RTN4RL1
INCA1	RPL26	RPL23A	RPL17	CTD	ATP8B3	LYL1	RPL18A	C19orf60		LIPE
EXOC3L2	FBXO46	PLEKHA4	RPS11	RPL28	RPS5	AHCY	SNX21	SOX18	EIF3L
C22orf46		SYP	RPS4X	RPSAP9	PLXNB3	FAM3A
GOBPID	Pvalue	OddsRatio	ExpCount	Count	Size	Term
GO:0006414	1.28219985360447e-32	70.5481481481481	0.610338384886859
24	105	translational elongation
GO:0006614	1.28219985360447e-32	70.5481481481481	0.610338384886859
24	105	SRP-dependent cotranslational protein targeting to membrane
GO:0006613	1.65082798032538e-32	69.6829268292683	0.616151131409591
24	106	cotranslational protein targeting to membrane
GO:0045047	1.65082798032538e-32	69.6829268292683	0.616151131409591
24	106	protein targeting to ER
GO:0072599	1.65082798032538e-32	69.6829268292683	0.616151131409591
24	106	establishment of protein localization to endoplasmic reticulum
GO:0006415	2.23213933103101e-32	79.2856798457088	0.528959933568611
23	91	translational termination
GO:0006413	8.4233156010459e-32	50.6658456486043	0.883537471455263
26	152	translational initiation
GO:0070972	2.81039982626144e-31	60.736170212766 0.685904089682375	24
118	protein localization to endoplasmic reticulum
GO:0000184	3.5062605041112e-31	60.0926315789474	0.691716836205107
24	119	nuclear-transcribed mRNA catabolic process, nonsense-mediated decay
GO:0006612	2.28515658252251e-28	44.1488372093023	0.889350217977995
24	153	protein targeting to membrane
GO:0000956	2.8176107971175e-27	39.2331034482759	0.982354162341706
24	169	nuclear-transcribed mRNA catabolic process
GO:0034623	5.85431298116393e-27	37.912	1.01141789495537	24	174
cellular macromolecular complex disassembly
GO:0019080	7.16972779958561e-27	41.9437756147541	0.877724724932531
23	151	viral genome expression
GO:0019083	7.16972779958561e-27	41.9437756147541	0.877724724932531
23	151	viral transcription
GO:0072594	9.2505363782843e-27	33.5858346747467	1.18580029063733
25	204	establishment of protein localization to organelle
GO:0043624	9.86168389136766e-27	41.2926860025221	0.889350217977995

23	153	cellular protein complex disassembly
GO:0006402	1.03365059022287e-26	36.9168831168831	1.03466888104629
24	178	mRNA catabolic process
GO:0032984	1.18888940060951e-26	36.6761290322581	1.04048162756903
24	179	macromolecular complex disassembly
GO:0043241	2.1451797018786e-26	39.7493624772313	0.918413950591655
23	158	protein complex disassembly
GO:0006401	3.43348159778401e-25	31.3502762430939	1.19161303716006
24	205	RNA catabolic process
GO:0071845	4.10172742036373e-23	23.0809501995943	1.65082001245589
25	284	cellular component disassembly at cellular level
GO:0022411	5.81602459443904e-23	22.7234645872269	1.67407099854681
25	288	cellular component disassembly
GO:0019058	1.71592017692334e-22	25.6665353089533	1.34274444675109
23	231	viral infectious cycle
GO:0022415	2.63356083777877e-21	22.4797675866362	1.51131409591032
23	260	viral reproductive process
GO:0033365	7.49727073773741e-19	13.9597315436242	2.74942910525223
26	473	protein localization to organelle
GO:0006605	7.90080609728781e-19	13.9275708128079	2.75524185177496
26	474	protein targeting
GO:0016032	5.83811772398419e-16	12.279646085491 2.62154868175213	23
451	viral reproduction
GO:0042273	5.07494776283997e-07	89.74375	0.0697529582727839	4
12	ribosomal large subunit biogenesis
Tissue: Artery_Tibial=>Thyroid	Type:	asymmetric
SourceGene:	MLX(ENSG00000108788.6)
TargetGeneSet:	UBE2J2	RP4	NOL9	DNAJC11 RP11	DFFA	DNAJC16 ARHGEF19
ARHGEF19-AS1	CROCCP3 ATP13A2 RCC2	KDM1A	TCEB3	LDLRAP1 SEPN1	XKR8	EYA3
MED18	RCC1	PRDX3P2 GMEB1	SNRNP40 HDAC1	ZMYM1	NCDN	RNF220	MMACHC
TTC22	GADD45A FRRS1	CYB561D1	AMIGO1	HSD3BP5 ANKRD34A	RPRD2
GOLPH3L LYSMD1	SNX27	THEM4	PYGO2	FLAD1	RUSC1-AS1	UBQLN4	RRNAD1
USP21	METTL13 MRPS14	BAP1	INTS7	TMEM206 PARP1	PYCR2	COG2	C1orf131
ZNF238	ADSS	PGBD2	SMC6	PGAM1P6 C2orf44 DNMT3A	C2orf18 TMEM214 PREB
SLC5A6	EIF2B4	PPM1G	CCDC121 EHD3	FAM98A	GEMIN6	PCBP1-AS1	CCT7
CCDC142 AUP1	ART4	MRPL15	GGCX	C2orf68 MRPL35	RMND5A	ZNF2	C2orf29
POLR1B	FOXD4L1 AMMECR1L	ORC2	TTLL4	EIF4E2	ASB1	DTYMK	RPUSD3
CHCHD4	ACAA1	MYD88	GORASP1 ZNF445	PTH1R	KLHL18	CTD	NME6	SLC25A20
NPRL2	ARL4D	ABHD10	WDR52	ADPRH	SRPRB	MSL2	SLC25A36	GRK7
UBQLN4P1	ABCC5	ABCF3	ALG3	PSMD2	MUC4	LRCH3	ZNF141	MYL5
MRFAP1L1	YIPF7	CLOCK	COX18	BRIP1	GTF2H2C ARSB	SERINC5 ZCCHC9
WDR36	PHAX	CDKN2AIPNL	SFXN1	C5orf25 SSR1	MAK	PFDN6	DAXX
SYNGAP1 ZBTB9	TAF8	RPL7L1	DLK2	AARS2	TDRD6	MCM3	VTA1	RSPH3	IQCE
C7orf26 MIOS	CDK13	URGCP	PSPH	SBDSP1	BAZ1B	ZKSCAN5 ZKSCAN1 TAF6
CNPY4	SLC12A9 METTL2A TNPO3	SMO	NFE2L1	C7orf49 ZNF398	ZNF862	LONRF1
CHMP7	EXTL3	INTS9	TTI2	ARMC1	DERL1	LRRC6	ZNF623	SNAPC3	KLHL9	PLAA
FAM122A FXN	C9orf40 CEP78	FOXE1	RNF20	TRIM32	PSMD5	ZBTB43	TRUB2
TBC1D13 CCBL1	TOR1B	EXOSC2	BRD3	NACC2	C9orf69 QSOX2	SDCCAG3 NUDT5
ZNF485	ZFAND4	ERCC6	CSTF2T	MSS51	AP3M1	POLR3A	IDE	MYOF	ENTPD7	BTRC
TAF5	SMC3	FAM175B CTR9	CAPRIN1 TTC9C	ZBTB3	MARK2	PLCB3	GPR137
RPS6KA4 MEN1	CPT1A	UCP3	C11orf30	FAM76B	DLAT	ZW10	PAFAH1B2
DDX6	DPAGT1	RNF26	ZNF202	HYLS1	APPL2	VPS26B	RAD52	NCAPD2	KIAA1467
REP15	BCDIN3D MFSD5	SMUG1	PDE1B	ZC3H10	OBFC2B	TIMELESS	KIF5A
TMEM19	TMPO	TRPV4	TCHP	ANAPC7	VPS29	ATXN2	RNF34	ORAI1	C12orf65
SBNO1	SNRNP35 BRI3BP	GOLGA3	KRR1P1	PDS5B	PROSER1 KBTBD6	KBTBD7	NAA16
ITM2B	BORA	TTC5	MRPL52	ACIN1	SYNJ2BP PROX2	NEK9	POMT2	CPSF2
BTBD7	GOLGA8B ZFYVE19 LCMT2	TUBGCP4 DUOX1	AP4E1	RNF111	PARP16	DIS3L
PIAS1	CALML4	ANP32A	HEXA	MPI	ZSCAN2	ZNF774	NR2F2	ALDH1A3 GLYR1	USP7
ERCC4	C16orf88	UBFD1	MAZ	TAOK2	GDPD3	TBC1D10B	CNEP1R1
HEATR3	CIAPIN1 GOT2	E2F4	CTCF	TMEM170A	VN2R10P MLYCD	USP10
GALNS	CENPBD1 VPS53	ANKFY1	ZFP3	DHX33	SLC16A13	GPS2	WRAP53
KRBA2	USP32P2 C17orf51	SPAG5-AS1	KIAA0100	CRLF3	TEFM
PEX12	DHRS11	PIP4K2B MED1	LEPREL4 CNP	FAM134C PSME3	RUNDC1	ZNF652

PPP1R9B RSAD1 ANKRD40 COX11 APPBP2 DCAF7 FTSJ3 PSMC5 POLG2 FAM104A
CD300A QRICH2 RP13 ZNF397 WDR7 ZADH2 LMNB2 ZNF555 ZBTB7A FEM1A
ZNF562 ZNF799 ZNF490 WDR83 SAMD1 TMEM221 GLT25D1 PIK3R2 LSM4 ZNF253
ZNF429 ANKRD27 NUDT19 ZNF792 LIN37 ZNF790 ZNF781 ZNF546 MIA BLOC1S3
MYPOP GRWD1 FUT2 BAX ZNF613 ZNF480 ZNF808 ZNF677 U2AF2 ZNF835
ZNF264 ZNF543 ZNF547 ZNF17 ZNF549 ZNF814 ZNF329 ZSCAN22 ZNF132 FASTKD5 MKKS
CSRP2BP CRNKL1 NANP CDK5RAP1 RBL1 FITM2 PI3 ZSWIM1 NCOA5
ELMO2 KRT18P4 ADNP ZFP64 VAPB RGS19 SON PRDM15 UBE2G2 CECR5
ZNF74 KRT18P5 CRKL UBE2L3 ZNF70 NF2 RNF215 PRR14L TOMM22 TNRC6B
XPNPEP3 L3MBTL2 TCF20 PPP2R3B-AS1 HDHD1 TLR8 ZNF41 ZNF81 RPL7P57
FTSJ1 GPKOW PPP1R3F FAM123B RBM41 AIFM1 NAA10 UBL4A
GOBPID Pvalue OddsRatio ExpCount Count Size Term
Tissue: Artery_Tibial=>Thyroid Type: asymmetric
SourceGene: RP1-92O14.6(ENSG00000229431.1)
TargetGeneSet: RUNX3 LCK CYP4A22 GBP5 RP4 CD2 ANKRD20A12P RP11
SLAMF6 CD84 CD48 LY9 CD247 XCL2 PTPN7 TRAF3IP3 IGKV1D-39
ZAP70 ITGA4 RUFY4 FBXO36 PDCD1 GPR15 PARP15 RHOH CXCL9 CXCL10
DAPP1 IL7R FYB ITK TAGAP CCR6 CARD11 IKZF1 TRBV29-1
TRBC2 CDKN2B-AS1 TSPAN32 CD5 AMICA1 CD3E CD3D LAG3 CLEC7A
TESPA1 TRAC PATL2 TPSB2 SEPT1 ITGAL ACAP1 C17orf66 CCL4L1
FMNL1 TMC8 DENND1C DNASE2 MAP4K1 NKG7 SIRPG UBASH3A CECR1 IGLV2-11
IL2RB KLHDC7B WWC3-AS1 IL2RG MTCP1
GOBPID Pvalue OddsRatio ExpCount Count Size Term
GO:0042110 7.91799160308697e-19 27.0586868686869 1.14787903951284
19 319 T cell activation
GO:0046649 1.14899298458457e-17 21.1652542372881 1.55809286554564
20 433 lymphocyte activation
GO:0050863 9.87449168117062e-16 27.9316452374705 0.795239083800429
15 221 regulation of T cell activation
GO:0050870 2.39477060932062e-14 30.0443037974684 0.615320739049201
13 171 positive regulation of T cell activation
GO:0051249 3.86222033178807e-14 21.3760588947156 1.01833783129195
15 283 regulation of lymphocyte activation
GO:0002696 5.57899666146866e-14 23.8548425859168 0.838419486540724
14 233 positive regulation of leukocyte activation
GO:0050867 8.3966696993133e-14 23.1045645086167 0.863608054805896
14 240 positive regulation of cell activation
GO:0002694 2.43773151427523e-13 18.6711711711712 1.15507577330289
15 321 regulation of leukocyte activation
GO:0051251 4.6343104045604e-13 23.4273927392739 0.773648882430282
13 215 positive regulation of lymphocyte activation
GO:0050865 6.37990227168931e-13 17.391644693474 1.23423984499343 15
343 regulation of cell activation
GO:0002429 1.15672041073631e-12 31.3968412634946 0.478582797038267
11 133 immune response-activating cell surface receptor signaling pathway
GO:0050852 1.78391606113461e-12 38.2825040128411 0.356238322607432
10 99 T cell receptor signaling pathway
GO:0002768 2.7847198420019e-12 28.7779204107831 0.518164832883537
11 144 immune response-regulating cell surface receptor signaling pathway
GO:0030098 5.69787603438948e-12 22.1984375 0.734066846585011 12
204 lymphocyte differentiation
GO:0030217 6.71253741383032e-12 26.3740958788898 0.561345235623832
11 156 T cell differentiation
GO:0050851 1.61161905646764e-11 30.1011378002528 0.442599128088022
10 123 antigen receptor-mediated signaling pathway
GO:0050778 3.46996677047776e-10 13.340930674264 1.30980554978894 13
364 positive regulation of immune response
GO:0002521 5.45838406235542e-10 14.5955172413793 1.08670680229742
12 302 leukocyte differentiation
GO:0002757 5.59221983302669e-10 16.9778963414634 0.845616220330773
11 235 immune response-activating signal transduction
GO:0050900 7.30755948021455e-10 16.5279957582185 0.86720642170092
11 241 leukocyte migration

GO:0002764 9.89526932126761e-10 16.0319028506741 0.892394989966092
11 248 immune response-regulating signaling pathway
GO:0030097 6.43672619411107e-09 10.3325925925926 1.66604387239637
13 463 hemopoiesis
GO:0002253 7.03563355743526e-09 13.1454098915989 1.07591170161235
11 299 activation of immune response
GO:0031294 8.45751436674455e-09 32.7833333333333 0.269877517126842
7 75 lymphocyte costimulation
GO:0031295 8.45751436674455e-09 32.7833333333333 0.269877517126842
7 75 T cell costimulation
GO:0048534 1.42906661055106e-08 9.62448132780083 1.78119161303716
13 495 hemopoietic or lymphoid organ development
GO:0045580 1.58926020043176e-08 29.709037037037 0.295066085392014 7
82 regulation of T cell differentiation
GO:0045619 5.94068377160562e-08 24.1905797101449 0.356238322607432
7 99 regulation of lymphocyte differentiation
GO:0033077 7.69733822378621e-08 34.0173913043478 0.219500380596499
6 61 T cell differentiation in thymus
GO:0045061 3.42675928321822e-07 92.2179487179487 0.0611722372154176
4 17 thymic T cell selection
GO:0045582 1.2475444866316e-06 31.8062943262411 0.190713445436302
5 53 positive regulation of T cell differentiation
GO:0045058 1.50173135517509e-06 59.9125 0.0863608054805896 4 24
T cell selection
GO:0045621 2.74531788601454e-06 26.7674505412467 0.223098747491523
5 62 positive regulation of lymphocyte differentiation
GO:0071350 3.63567199059503e-06 146.867346938776 0.0323853020552211
3 9 cellular response to interleukin-15
GO:0002252 8.39595002105175e-06 7.78471408303004 1.38896962147948
9 386 immune effector process
GO:0070672 9.4495803812895e-06 97.891156462585 0.0431804027402948 3
12 response to interleukin-15
Tissue: Artery_Tibial=>Thyroid Type: asymmetric
SourceGene: RP11-398A8.3(ENSG00000241163.1)
TargetGeneSet: RP1 RPL22 RPL11 RP4 GAPDHP32 RPS27 C1orf100
THPO LRPPRC PPM1B FAM161A RPL32 RPL15 RP11 HGD ABCC5 RPL35A
RPL34 LPCAT1 BTF3 NSA2 PCDHGB8P RPS14 OR2B6 RPL10A ZNF117
RPL30 POLR1E C9orf153 RPL35 RPL27A EIF3M RPS25 PHB2 RPL18AP3
GPN3 MLEC WDR90 NUDT21 NOL3 KLHDC4 DVL2 ADORA2B RPL17 RPL19
RPL27 MRPL10 UTP18 XAB2 ANO8 RPL18A SNRPD2 GLTSCR2 RPL18 SLC6A16
RPL13A RPS11 OTOR U6 ZNFX1-AS1 PMEPA1 EIF3D RPL3 EFCAB6
SSX2B LAS1L
GOBPID Pvalue OddsRatio ExpCount Count Size Term
GO:0006415 2.24254460945105e-33 130.245454545455 0.340045671579821
21 91 translational termination
GO:0006413 4.68572913134875e-32 82.0615153788447 0.567988374506954
23 152 translational initiation
GO:0006414 6.28665788110763e-32 108.431818181818 0.392360390284409
21 105 translational elongation
GO:0006614 6.28665788110763e-32 108.431818181818 0.392360390284409
21 105 SRP-dependent cotranslational protein targeting to membrane
GO:0006613 7.82262904892689e-32 107.148663101604 0.396097155906166
21 106 cotranslational protein targeting to membrane
GO:0045047 7.82262904892689e-32 107.148663101604 0.396097155906166
21 106 protein targeting to ER
GO:0072599 7.82262904892689e-32 107.148663101604 0.396097155906166
21 106 establishment of protein localization to endoplasmic reticulum
GO:0070972 9.11874317632008e-31 93.8144329896907 0.440938343367241
21 118 protein localization to endoplasmic reticulum
GO:0000184 1.10484427653315e-30 92.8506493506493 0.444675108988997
21 119 nuclear-transcribed mRNA catabolic process, nonsense-mediated decay
GO:0019080 2.32300005459506e-28 69.8384615384615 0.564251608885198
21 151 viral genome expression

GO:0019083	2.32300005459506e-28	69.8384615384615	0.564251608885198
21	151	viral transcription
GO:0006612	3.110465836935e-28	68.7706611570248	0.571725140128711
21	153	protein targeting to membrane
GO:0043624	3.110465836935e-28	68.7706611570248	0.571725140128711
21	153	cellular protein complex disassembly
GO:0043241	6.33905511835966e-28	66.2375580623756	0.590408968237492
21	158	protein complex disassembly
GO:0000956	2.79484968933089e-27	61.267199017199 0.631513390076811	21
169	nuclear-transcribed mRNA catabolic process
GO:0034623	5.30088093437068e-27	59.2442067736185	0.650197218185593
21	174	cellular macromolecular complex disassembly
GO:0006402	8.7213255440415e-27	57.7185871453387	0.665144280672618
21	178	mRNA catabolic process
GO:0032984	9.85870964498321e-27	57.349252013809 0.668881046294374	21
179	macromolecular complex disassembly
GO:0072594	1.69617848285117e-25	49.4277198211624	0.762300186838281
21	204	establishment of protein localization to organelle
GO:0006401	1.88559175218445e-25	49.1556324110672	0.766036952460037
21	205	RNA catabolic process
GO:0019058	2.47146800524597e-24	42.9909090909091	0.863192858625701
21	231	viral infectious cycle
GO:0022415	3.09241419212308e-23	37.6972232788132	0.971559061656633
21	260	viral reproductive process
GO:0071845	2.01202174463804e-22	34.1991012789492	1.06124143657878
21	284	cellular component disassembly at cellular level
GO:0022411	2.70405805785441e-22	33.6772216547497	1.07618849906581
21	288	cellular component disassembly
GO:0016032	3.04439881613852e-18	20.669978858351 1.68528129541208	21
451	viral reproduction
GO:0033365	8.05583481382574e-18	19.6329444891392	1.76749013909072
21	473	protein localization to organelle
GO:0006605	8.41035788686445e-18	19.5881998795906	1.77122690471248
21	474	protein targeting
GO:0022613	5.42881346003134e-10	16.9542490762878	0.840772264895163
11	225	ribonucleoprotein complex biogenesis
GO:0071843	1.02968832075048e-09	15.8974908200734	0.893086983599751
11	239	cellular component biogenesis at cellular level
GO:0042254	7.36950037898148e-08	17.5837187789084	0.556778077641686
8	149	ribosome biogenesis
GO:0006364	2.34516328586075e-06	18.2385204081633	0.388623624662653
6	104	rRNA processing
GO:0016072	3.2541389883178e-06	17.1790865384615	0.411044218393191
6	110	rRNA metabolic process
Tissue: Artery_Tibial=>Thyroid	Type:	asymmetric
SourceGene:	RP11-469M7.1(ENSG00000260006.1)
TargetGeneSet:	UBE2J2	RP4	TTC34	CEP104	DFFB	RP13	THAP3	ERRFI1	LZIC
RP11	DFFA	PLOD1	DNAJC16	ARHGEF19	ARHGEF19-AS1	CROCCP3	ATP13A2
HP1BP3	TCEB3	LDLRAP1	SH3BGRL3	XKR8	EYA3	MED18	RCC1	PRDX3P2
SNRNP40	TXLNA	FAM167B	HDAC1	ZMYM1	NCDN	C1orf216	LSM10	ZMPSTE24
SLFNL1	RNF220	MMACHC	ATRX	STIL	C1orf123	TTC22	OMA1	MYSM1
ATG4C	ALG6	GADD45A	HHLA3	SYDE2	CCBL2	FRRS1	PSMA5	ATXN7L2	CYB561D1
CEPT1	GDAP2	GAPDHP32	LIX1L	GNRHR2	BOLA1	TARS2	GOLPH3L	ARNT
GABPB1	LYSMD1	SNX27	INTS3	DENND4B	PYGO2	FLAD1	FAM189B	UBQLN4	BGLAP	CCT3
C1orf182	ARHGEF11	USP21	HSD17B7	POGK	METTL13	PIGC	CACYBP
MRPS14	SMG7	TRMT1L	PTGS2	C1orf98	KLHL12	BTG2	TMEM81	TMCC2	CD46
INTS7	MARC1	TP53BP2	PARP1	LBR	COG2	C1orf131	TOMM20	MTR	ADSS
HSD17B7P1	PGBD2	SMC6	TTC32	UBXN2A	DNMT3A	RAB10	PREB	SLC5A6
CCDC121	SUPT7L	EHD3	SLC30A6	FAM98A	GEMIN6	ARHGEF33	PLEKHH2	PAPOLG	XPO1
FAM161A	PCBP1-AS1	SPR	CCT7	AUP1	ART4	MRPL15	GGCX	C2orf68
MRPL35	RMND5A	PLGLB1	MRPS5	ZNF2	LMAN2L	TBC1D8	C2orf29	GPR45	POLR1B
FOXD4L1	STEAP3	STEAP3-AS1	TMEM37	LY75	BBS5	C2orf77	HSPD1	SATB2
CASP10	ICA1L	WDR12	KLF7-IT1	CREB1	IDH1	ATIC	RQCD1	BCS1L

TTLL4 NHEJ1 COL4A3 EIF4E2 RNPEPL1 DTYMK TTLL3 CRELD1 CHCHD4 C3orf19
METTL6 GORASP1 ZNF619 ZNF620 C3orf23 CTD NME6 PLXNB1 NPRL2 HEMK1
DUSP7 PHF7 SCT FLNB-AS1 SHQ1 ARL4D ABHD10 WDR52 KIAA1407
TIMMDC1 GTF2E1 PTPLB SEC61A1 CDV3 SRPRB MSL2 SLC25A36 ZBTB38 GRK7
GK5 TRPC1 UBQLN4P1 SIAH2 MLF1 SERPINI2 WDR49 ACTL6A
DCUN1D1 PARL ABCC5 ABCF3 ALG3 POLR2H EPHB3 CRYGS TBCCD1 DNAJB11 RFC4
U2 CEP19 LRCH3 ZNF732 ZNF141 MYL5 DGKQ CTBP1 TMEM128 ZNF518B
NFXL1 CLOCK COX18 ANKRD17 CXCL2 CXCL11 FAM47E THAP9 CASP6 CCNA2
KIAA1109 ANKRD50 IL15 TIGD4 MFAP3L IRF2 EXOC3 LPCAT1 BRIP1
MARCH6 FAM105B C5orf51 SNX18 GPX8 SLC38A9 IPO11 PTCD2 ARSB FAM151B
ZCCHC9 FAM81B FAM174A PPIP5K2 WDR36 EPB41L4A-AS1 APC XBP1P1 PHAX
P4HA2-AS1 P4HA2 SHROOM1 C5orf65 IGIP CTB HARS2 DIAPH1 SH3RF2
HMGXB3 IRGM MED7 TSPAN17 TRIM52 RP1 PXDC1 C6orf228 HIST1H2AC
HIST1H2BD HIST1H1PS1 HIST1H3E HIST1H2BH HIST1H3J
OR2B6 SCAND3 TRIM27 PPP1R11 GNL1 DDR1 CSNK2B C6orf25 HSPA1L PFDN6
SYNGAP1 ZBTB9 NUDT3 PPIL1 TAF8 CNPY3 AARS2 BAG2 LINC00472
MB21D1 MTO1 UBE2J1 PREP C6orf203 TRMT11 PEX7 VIP SERAC1
MRPL18 SUN1 RNF216P1 C7orf26 MIOS C7orf46 CDK13 URGCP CAMK2B
VOPP1 PSPH ZNF117 SBDS SBDSP1 BAZ1B CLIP2 STEAP4 RBM48 PPP1R9A
ZKSCAN5 FAM200A ZKSCAN1 CNPY4 SLC12A9 PLOD3 CLDN15 C7orf53 CAV2 CADPS2 GCC1
IMPDH1 METTL2A TNPO3 SMO TRPV6 ZNF786 ZNF398 ZNF862 GIMAP4 CDK5
ABCF2 SMARCD3 CTA FDFT1 LONRF1 FAM160B2 CHMP7 PPP2R2A EXTL3
INTS9 HMBOX1 FUT10 TTI2 C8orf40 CYP7A1 YTHDF3 ARMC1 GDAP1 INTS8
GAPDHP62 GRHL2 ENY2 DERL1 WDR67 ZHX1 LRRC6 ZFAT C8orf73
ZNF623 NRBP2 ZNF252 CBWD1 RANBP6 SNAPC3 KLHL9 C9orf11 DNAJA1 VCP
HMGB3P24 DCAF10 FXN SMC5 C9orf40 CEP78 C9orf103 LINC00476
FOXE1 NANS ALG2 RNF20 NIPSNAP3B TRIM32 TBC1D13 CCBL1 DOLPP1
TOR1B EXOSC2 SURF4 C9orf69 SDCCAG3 FAM69B MAMDC4 C9orf142 SSNA1
TUBB4B NOXA1 KLF6 FBXO18 NUDT5 CDNF BMI1 LINC00202 ZNF485
ZFAND4 ERCC6 CSTF2T ADO PBLD DNA2 P4HA1 MSS51 CHCHD1 AP3M1
MINPP1 BTAF1 IDE MYOF FRAT1 LINC00263 BTRC TMEM180 SUFU TAF5
ZDHHC6 METTL10 FAM175B CTAGE7P STK32C CYP2E1 AP2A2 IFITM10 ZNF195 CHRNA10
UBQLNL TRIM34 TRIM5 RRP8 MRPL17 RIC3 ZNF143 TPH1 MPPED2 SLC35C1 ETFA
CELF1 B3GAT3 TTC9C ZBTB3 C11orf84 MARK2 STIP1 FKBP2 PLCB3
GPR137 RPS6KA4 MEN1 PPP2R5B GPHA2 SAC3D1 EFEMP2 RBM14 7SK SUV420H1
KRTAP5-9 FOLR3 CLPB MRPL48 UCP3 C11orf30 SLC36A4 FAM76B
MSANTD4 RDX C11orf93 TIMM8B ZW10 USP28 RNF214 SETP16 H2AFX
DPAGT1 C2CD2L CBL HSPA8 ZNF202 STT3A HYLS1 APPL2 VPS26B RAD52
FKBP4 NCAPD2 RPL13AP20 KIAA1467 REP15 PUS7L ENDOU BCDIN3D
TARBP2 SMUG1 SUOX ZC3H10 TIMELESS ZBTB39 TMEM194A TBC1D30
LEMD3 DYRK2 TMEM19 CSRP2 TMPO CKAP4 POLR3B FICD C12orf34
ANAPC7 VPS29 DYNLL1 POP5 MLEC RNF34 C12orf65 SBNO1 SNRNP35 UBC
BRI3BP SFSWAP PUS1 GOLGA3 RPL23AP67 ZMYM5 KRR1P1 LNX2 PDS5B
NHLRC3 KBTBD6 KBTBD7 NAA16 ITM2B DLEU1 RPS4XP16 DHRS12 SUGT1 BORA
LMO7 DNAJC3-AS1 DNAJC3 UGGT2 RAP2A PROZ PCID2 TTC5 PARP2 DAD1
MRPL52 ACIN1 SFTA3 MGAT2 DNAAF2 NEMF TXNDC16 GNPNAT1 HNRPCP SYNJ2BP
MAP3K9 RBM25 ZNF410 PROX2 NEK9 POMT2 AHSA1 SPTLC2 RPS6KA5 CPSF2
BTBD7 PAPOLA RPS20P33 GOLGA8B ZFYVE19 PPP1R14D RPAP1 ZFP106
TP53BP1 PATL2 B2M MYEF2 HDC AP4E1 PIGB RNF111 NARG2 CSNK1G1
PARP16 DIS3L SMAD6 FEM1B ANP32A HEXA CSK SCAMP2 GOLGA6L5
ZSCAN2 CHD2 NR2F2 LRRC28 ASB7 ALDH1A3 SOLH FBXL16 CCDC154 MRPS34
NTHL1 ZNF205 ATF7IP2 ERCC4 UBFD1 PLK1 MAZ TAOK2 TBX6 GDPD3
SULT1A3 TBC1D10B FBXL19-AS1 FBXL19 CNEP1R1 HEATR3 PAPD5 SALL1
CCL22 DOK4 EXOC3L1 CTCF DDX19B TMEM170A C16orf46 MLYCD
ZC3H18 CENPBD1 VPS53 RAP1GAP2 ZMYND15 RNF167 ZFP3 DERL2 PITPNM3
SLC16A13 WRAP53 KRBA2 COX10-AS1 ZSWIM7 ALKBH5 LLGL1 C17orf51
WSB1 C17orf108 SUPT6H ABHD15 BLMH CRLF3 RNF135 CCT6B ZNF830
PEX12 DHRS11 PIP4K2B LEPREL4 ACLY KAT2A FAM134C RUNDC1 UBTF C17orf69
ZNF652 DLX4 PPP1R9B RSAD1 C17orf67 TRIM25 RAD51C HEATR6 APPBP2
DCAF7 CEP95 HN1 GGA3 UNK TRIM65 QRICH2 ENGASE CBX2 CBX8
SIRT7 PYCR1 LRRC45 RNMT RIOK3 ZNF397 MOCOS CCDC11 ONECUT2 BCL2
ZADH2 MIER2 C19orf21 CIRBP MOB3A DOT1L LMNB2 ZNF555 TLE6
PIAS4 ZBTB7A DPP9 SAFB CAPS ELAVL1 ZNF558 ZNF562 PIN1 ICAM1

ZGLP1 RAB3D ZNF627 ZNF440 ZNF433 ZNF844 ZNF442 ZNF799 ZNF490 WDR83 LYL1
STX10 SAMD1 SIN3B NR2F6 ANO8 TMEM221 GLT25D1 PIK3R2 JUND ZNF253
ZNF91 ANKRD27 NUDT19 KCTD15 ZNF792 LIN37 CAPNS1 ZNF565 ZNF529 ZNF790
ZNF781 MED29 ZFP36 ZNF546 MAP3K10 SHKBP1 MIA SMG9 ZNF283 ZNF284
GEMIN7 BLOC1S3 FBXO46 DMPK RSPH6A MYPOP KPTN CARD8 FUT2 PPP1R15A
BAX SLC6A16 PTOV1 NAPSA C19orf63 CTU1 ZNF615 ZNF808 ZNF816
ZNF347 ZNF761 U2AF2 ZNF835 ZNF264 ZNF543 ZNF547 ZNF749 ZNF549 ZNF586
ZNF814 ZNF329 ZSCAN22 ZNF132 ZCCHC3 FAM110A NOP56 FASTKD5 PCNA MKKS GZF1
ABHD12 NANP COMMD7 DNMT3B PXMP4 ZNF341 MMP24 NFS1 NDRG3 RBL1
IFT52 FITM2 ADA MATN4 SDC4 ZSWIM3 ZSWIM1 NCOA5 ELMO2 KRT18P4
TMEM189 ADNP ZFP64 VAPB C20orf151 TCFL5 DPH3P1 RGS19 PCMTD2
RBM11 RNGTTP1 TCP10L TMEM50B C2CD2 SUMO3 CECR5 ZNF74 KRT18P5 CRKL
UBE2L3 SDF2L1 ZNF280B ZNF70 ASPHD2 HPS4 GAS2L1 NF2 CCDC157 RNF215
PRR14L POLR2F TNRC6B SGSM3 XPNPEP3 L3MBTL2 EFCAB6 RP6 NCAPH2 CPT1B ACR
PPP2R3B-AS1 HDHD1 ARHGAP6 TCEANC REPS2 SCML1 ZNF673 ZNF41 ZNF81
ZNF630 RBM3 KCND1 PPP1R3F WNK3 FAM123B FAM155B CXorf65 ACRC DMRTC1
PABPC5 BEX4 PGRMC1 EEF1A1P30 ZBTB33 AIFM1 ZNF449 FHL1 IDS
NAA10 HCFC1 LAGE3 UBL4A MT
GOBPID Pvalue OddsRatio ExpCount Count Size Term
Tissue: Artery_Tibial=>Thyroid Type: cluster
SourceGene: RP11-469M7.1(ENSG00000260006.1)
TargetGeneSet: UBE2J2 RP4 TTC34 CEP104 DFFB RP13 THAP3 ERRFI1 LZIC
RP11 DFFA PLOD1 DNAJC16 ARHGEF19 ARHGEF19-AS1 CROCCP3 ATP13A2
HP1BP3 TCEB3 LDLRAP1 SH3BGRL3 XKR8 EYA3 MED18 RCC1 PRDX3P2
SNRNP40 TXLNA FAM167B HDAC1 ZMYM1 NCDN C1orf216 LSM10 ZMPSTE24
SLFNL1 RNF220 MMACHC ATRX STIL C1orf123 TTC22 OMA1 MYSM1
ATG4C ALG6 GADD45A HHLA3 SYDE2 CCBL2 FRRS1 PSMA5 ATXN7L2 CYB561D1
CEPT1 GDAP2 GAPDHP32 LIX1L GNRHR2 BOLA1 TARS2 GOLPH3L ARNT
GABPB1 LYSMD1 SNX27 INTS3 DENND4B PYGO2 FLAD1 FAM189B UBQLN4 BGLAP CCT3
C1orf182 ARHGEF11 USP21 HSD17B7 POGK METTL13 PIGC CACYBP
MRPS14 SMG7 TRMT1L PTGS2 C1orf98 KLHL12 BTG2 TMEM81 TMCC2 CD46
INTS7 MARC1 TP53BP2 PARP1 LBR COG2 C1orf131 TOMM20 MTR ADSS
HSD17B7P1 PGBD2 SMC6 TTC32 UBXN2A DNMT3A RAB10 PREB SLC5A6
CCDC121 SUPT7L EHD3 SLC30A6 FAM98A GEMIN6 ARHGEF33 PLEKHH2 PAPOLG XPO1
FAM161A PCBP1-AS1 SPR CCT7 AUP1 ART4 MRPL15 GGCX C2orf68
MRPL35 RMND5A PLGLB1 MRPS5 ZNF2 LMAN2L TBC1D8 C2orf29 GPR45 POLR1B
FOXD4L1 STEAP3 STEAP3-AS1 TMEM37 LY75 BBS5 C2orf77 HSPD1 SATB2
CASP10 ICA1L WDR12 KLF7-IT1 CREB1 IDH1 ATIC RQCD1 BCS1L
TTLL4 NHEJ1 COL4A3 EIF4E2 RNPEPL1 DTYMK TTLL3 CRELD1 CHCHD4 C3orf19
METTL6 GORASP1 ZNF619 ZNF620 C3orf23 CTD NME6 PLXNB1 NPRL2 HEMK1
DUSP7 PHF7 SCT FLNB-AS1 SHQ1 ARL4D ABHD10 WDR52 KIAA1407
TIMMDC1 GTF2E1 PTPLB SEC61A1 CDV3 SRPRB MSL2 SLC25A36 ZBTB38 GRK7
GK5 TRPC1 UBQLN4P1 SIAH2 MLF1 SERPINI2 WDR49 ACTL6A
DCUN1D1 PARL ABCC5 ABCF3 ALG3 POLR2H EPHB3 CRYGS TBCCD1 DNAJB11 RFC4
U2 CEP19 LRCH3 ZNF732 ZNF141 MYL5 DGKQ CTBP1 TMEM128 ZNF518B
NFXL1 CLOCK COX18 ANKRD17 CXCL2 CXCL11 FAM47E THAP9 CASP6 CCNA2
KIAA1109 ANKRD50 IL15 TIGD4 MFAP3L IRF2 EXOC3 LPCAT1 BRIP1
MARCH6 FAM105B C5orf51 SNX18 GPX8 SLC38A9 IPO11 PTCD2 ARSB FAM151B
ZCCHC9 FAM81B FAM174A PPIP5K2 WDR36 EPB41L4A-AS1 APC XBP1P1 PHAX
P4HA2-AS1 P4HA2 SHROOM1 C5orf65 IGIP CTB HARS2 DIAPH1 SH3RF2
HMGXB3 IRGM MED7 TSPAN17 TRIM52 RP1 PXDC1 C6orf228 HIST1H2AC
HIST1H2BD HIST1H1PS1 HIST1H3E HIST1H2BH HIST1H3J
OR2B6 SCAND3 TRIM27 PPP1R11 GNL1 DDR1 CSNK2B C6orf25 HSPA1L PFDN6
SYNGAP1 ZBTB9 NUDT3 PPIL1 TAF8 CNPY3 AARS2 BAG2 LINC00472
MB21D1 MTO1 UBE2J1 PREP C6orf203 TRMT11 PEX7 VIP SERAC1
MRPL18 SUN1 RNF216P1 C7orf26 MIOS C7orf46 CDK13 URGCP CAMK2B
VOPP1 PSPH ZNF117 SBDS SBDSP1 BAZ1B CLIP2 STEAP4 RBM48 PPP1R9A
ZKSCAN5 FAM200A ZKSCAN1 CNPY4 SLC12A9 PLOD3 CLDN15 C7orf53 CAV2 CADPS2 GCC1
IMPDH1 METTL2A TNPO3 SMO TRPV6 ZNF786 ZNF398 ZNF862 GIMAP4 CDK5
ABCF2 SMARCD3 CTA FDFT1 LONRF1 FAM160B2 CHMP7 PPP2R2A EXTL3
INTS9 HMBOX1 FUT10 TTI2 C8orf40 CYP7A1 YTHDF3 ARMC1 GDAP1 INTS8
GAPDHP62 GRHL2 ENY2 DERL1 WDR67 ZHX1 LRRC6 ZFAT C8orf73
ZNF623 NRBP2 ZNF252 CBWD1 RANBP6 SNAPC3 KLHL9 C9orf11 DNAJA1 VCP

HMGB3P24 DCAF10 FXN SMC5 C9orf40 CEP78 C9orf103 LINC00476
FOXE1 NANS ALG2 RNF20 NIPSNAP3B TRIM32 TBC1D13 CCBL1 DOLPP1
TOR1B EXOSC2 SURF4 C9orf69 SDCCAG3 FAM69B MAMDC4 C9orf142 SSNA1
TUBB4B NOXA1 KLF6 FBXO18 NUDT5 CDNF BMI1 LINC00202 ZNF485
ZFAND4 ERCC6 CSTF2T ADO PBLD DNA2 P4HA1 MSS51 CHCHD1 AP3M1
MINPP1 BTAF1 IDE MYOF FRAT1 LINC00263 BTRC TMEM180 SUFU TAF5
ZDHHC6 METTL10 FAM175B CTAGE7P STK32C CYP2E1 AP2A2 IFITM10 ZNF195 CHRNA10
UBQLNL TRIM34 TRIM5 RRP8 MRPL17 RIC3 ZNF143 TPH1 MPPED2 SLC35C1 ETFA
CELF1 B3GAT3 TTC9C ZBTB3 C11orf84 MARK2 STIP1 FKBP2 PLCB3
GPR137 RPS6KA4 MEN1 PPP2R5B GPHA2 SAC3D1 EFEMP2 RBM14 7SK SUV420H1
KRTAP5-9 FOLR3 CLPB MRPL48 UCP3 C11orf30 SLC36A4 FAM76B
MSANTD4 RDX C11orf93 TIMM8B ZW10 USP28 RNF214 SETP16 H2AFX
DPAGT1 C2CD2L CBL HSPA8 ZNF202 STT3A HYLS1 APPL2 VPS26B RAD52
FKBP4 NCAPD2 RPL13AP20 KIAA1467 REP15 PUS7L ENDOU BCDIN3D
TARBP2 SMUG1 SUOX ZC3H10 TIMELESS ZBTB39 TMEM194A TBC1D30
LEMD3 DYRK2 TMEM19 CSRP2 TMPO CKAP4 POLR3B FICD C12orf34
ANAPC7 VPS29 DYNLL1 POP5 MLEC RNF34 C12orf65 SBNO1 SNRNP35 UBC
BRI3BP SFSWAP PUS1 GOLGA3 RPL23AP67 ZMYM5 KRR1P1 LNX2 PDS5B
NHLRC3 KBTBD6 KBTBD7 NAA16 ITM2B DLEU1 RPS4XP16 DHRS12 SUGT1 BORA
LMO7 DNAJC3-AS1 DNAJC3 UGGT2 RAP2A PROZ PCID2 TTC5 PARP2 DAD1
MRPL52 ACIN1 SFTA3 MGAT2 DNAAF2 NEMF TXNDC16 GNPNAT1 HNRPCP SYNJ2BP
MAP3K9 RBM25 ZNF410 PROX2 NEK9 POMT2 AHSA1 SPTLC2 RPS6KA5 CPSF2
BTBD7 PAPOLA RPS20P33 GOLGA8B ZFYVE19 PPP1R14D RPAP1 ZFP106
TP53BP1 PATL2 B2M MYEF2 HDC AP4E1 PIGB RNF111 NARG2 CSNK1G1
PARP16 DIS3L SMAD6 FEM1B ANP32A HEXA CSK SCAMP2 GOLGA6L5
ZSCAN2 CHD2 NR2F2 LRRC28 ASB7 ALDH1A3 SOLH FBXL16 CCDC154 MRPS34
NTHL1 ZNF205 ATF7IP2 ERCC4 UBFD1 PLK1 MAZ TAOK2 TBX6 GDPD3
SULT1A3 TBC1D10B FBXL19-AS1 FBXL19 CNEP1R1 HEATR3 PAPD5 SALL1
CCL22 DOK4 EXOC3L1 CTCF DDX19B TMEM170A C16orf46 MLYCD
ZC3H18 CENPBD1 VPS53 RAP1GAP2 ZMYND15 RNF167 ZFP3 DERL2 PITPNM3
SLC16A13 WRAP53 KRBA2 COX10-AS1 ZSWIM7 ALKBH5 LLGL1 C17orf51
WSB1 C17orf108 SUPT6H ABHD15 BLMH CRLF3 RNF135 CCT6B ZNF830
PEX12 DHRS11 PIP4K2B LEPREL4 ACLY KAT2A FAM134C RUNDC1 UBTF C17orf69
ZNF652 DLX4 PPP1R9B RSAD1 C17orf67 TRIM25 RAD51C HEATR6 APPBP2
DCAF7 CEP95 HN1 GGA3 UNK TRIM65 QRICH2 ENGASE CBX2 CBX8
SIRT7 PYCR1 LRRC45 RNMT RIOK3 ZNF397 MOCOS CCDC11 ONECUT2 BCL2
ZADH2 MIER2 C19orf21 CIRBP MOB3A DOT1L LMNB2 ZNF555 TLE6
PIAS4 ZBTB7A DPP9 SAFB CAPS ELAVL1 ZNF558 ZNF562 PIN1 ICAM1
ZGLP1 RAB3D ZNF627 ZNF440 ZNF433 ZNF844 ZNF442 ZNF799 ZNF490 WDR83 LYL1
STX10 SAMD1 SIN3B NR2F6 ANO8 TMEM221 GLT25D1 PIK3R2 JUND ZNF253
ZNF91 ANKRD27 NUDT19 KCTD15 ZNF792 LIN37 CAPNS1 ZNF565 ZNF529 ZNF790
ZNF781 MED29 ZFP36 ZNF546 MAP3K10 SHKBP1 MIA SMG9 ZNF283 ZNF284
GEMIN7 BLOC1S3 FBXO46 DMPK RSPH6A MYPOP KPTN CARD8 FUT2 PPP1R15A
BAX SLC6A16 PTOV1 NAPSA C19orf63 CTU1 ZNF615 ZNF808 ZNF816
ZNF347 ZNF761 U2AF2 ZNF835 ZNF264 ZNF543 ZNF547 ZNF749 ZNF549 ZNF586
ZNF814 ZNF329 ZSCAN22 ZNF132 ZCCHC3 FAM110A NOP56 FASTKD5 PCNA MKKS GZF1
ABHD12 NANP COMMD7 DNMT3B PXMP4 ZNF341 MMP24 NFS1 NDRG3 RBL1
IFT52 FITM2 ADA MATN4 SDC4 ZSWIM3 ZSWIM1 NCOA5 ELMO2 KRT18P4
TMEM189 ADNP ZFP64 VAPB C20orf151 TCFL5 DPH3P1 RGS19 PCMTD2
RBM11 RNGTTP1 TCP10L TMEM50B C2CD2 SUMO3 CECR5 ZNF74 KRT18P5 CRKL
UBE2L3 SDF2L1 ZNF280B ZNF70 ASPHD2 HPS4 GAS2L1 NF2 CCDC157 RNF215
PRR14L POLR2F TNRC6B SGSM3 XPNPEP3 L3MBTL2 EFCAB6 RP6 NCAPH2 CPT1B ACR
PPP2R3B-AS1 HDHD1 ARHGAP6 TCEANC REPS2 SCML1 ZNF673 ZNF41 ZNF81
ZNF630 RBM3 KCND1 PPP1R3F WNK3 FAM123B FAM155B CXorf65 ACRC DMRTC1
PABPC5 BEX4 PGRMC1 EEF1A1P30 ZBTB33 AIFM1 ZNF449 FHL1 IDS
NAA10 HCFC1 LAGE3 UBL4A MT
GOBPID Pvalue OddsRatio ExpCount Count Size Term
Tissue: Artery_Tibial=>Thyroid Type: asymmetric
SourceGene: RP11-496I2.3(ENSG00000257898.1)
TargetGeneSet: B3GALT6 RPL22 PLEKHG5 RP3 RPL11 RP4 PABPC4 YBX1 RPS8
RPL5 RP11 TMEM167B RPS27 LRRN2 GALNT2 TOMM20 OR11L1 RPS7 UBC
CCDC104 INPP4A RPL31 FOXD4L1 STEAP3-AS1 RPL37A RPL32 RPL15 CCRL1
EIF1B RPL14 LARS2 SLC52A1 RPL10AP6 THOC7 RPL24 CNBP RPL35A

RPL34 RPS3A TLR3 RPL37 ITGA2 BTF3 NSA2 EPB41L4A-AS1 CCNI2 SKP1
CTB THG1L GNB2L1 EXOC2 BTN3A3 RPL10A RPL39P3 EEF1A1 RWDD1 MRPS12
ZDHHC4 VOPP1 PIK3CG RPS20 SNHG6 RPL7 RPL30 EIF3E EIF3H PARP10 RPS6
VCP SIT1 ANP32B U4 RPL12 SET ENDOG RPL7A SLC2A6 TUBB4B CCNY
TSPAN15 RPS3AP5 RPL13AP5 TRIM5 RPL27A RASSF10 C11orf58 CTD
INTS5 CLPB RPS25 CDON PFDN5 PPM1H RPL14P1 NAP1L1 FAM71C POLR3B GPN3
RPL6 MLEC BRI3BP TPT1 RCBTB2 CCNB1IP1 RPL36A RPL7AP6 BTBD7
RPS3AP6 CSK SCAMP2 ANP32BP1 NTHL1 RPS15A LYRM1 C16orf54
ADCY7 LPCAT2 GABARAP RPL26 RPL23A RPL17 RPL19 EIF1 RPL27 ITGB3
LUC7L3 TRIM25 CYB561 LRRC45 PHLPP1 BCL2 MED16 TMIGD2 VAV1 RFX1 ANO8
UBA52 ARMC6 LRFN1 SNRPD2 RPS11 RPS5 AP5S1 SEC23B SDC4 ZNFX1-AS1
ZFP64 POLR2CP RTN4R RIMBP3 ZNF70 EIF3D RPL3 TNRC6B UXT RPS23P8
RPS4X SLC16A2 LPAR4
GOBPID Pvalue OddsRatio ExpCount Count Size Term
GO:0006415 8.10773869219631e-57 123.329393043874 0.761954190021452
38 91 translational termination
GO:0006414 7.0171850235729e-56 102.789356984479 0.879177911563214
39 105 translational elongation
GO:0006413 8.52636321611516e-56 71.9246059750647 1.27271469102484
43 152 translational initiation
GO:0006614 7.02328282744798e-54 97.463405862255 0.879177911563214 38
105 SRP-dependent cotranslational protein targeting to membrane
GO:0006613 1.08863645954581e-53 96.0233876683203 0.887551034530482
38 106 cotranslational protein targeting to membrane
GO:0045047 1.08863645954581e-53 96.0233876683203 0.887551034530482
38 106 protein targeting to ER
GO:0072599 1.08863645954581e-53 96.0233876683203 0.887551034530482
38 106 establishment of protein localization to endoplasmic reticulum
GO:0000184 2.544223793924e-53 84.717987804878 0.996401633104975 39
119 nuclear-transcribed mRNA catabolic process, nonsense-mediated decay
GO:0070972 1.44053772536786e-51 81.5512048192771 0.988028510137707
38 118 protein localization to endoplasmic reticulum
GO:0019080 7.05958218321539e-47 57.6016632903295 1.26434156805757
38 151 viral genome expression
GO:0019083 7.05958218321539e-47 57.6016632903295 1.26434156805757
38 151 viral transcription
GO:0006612 1.23819243971341e-46 56.5919329491881 1.28108781399211
38 153 protein targeting to membrane
GO:0043624 1.23819243971341e-46 56.5919329491881 1.28108781399211
38 153 cellular protein complex disassembly
GO:0000956 1.62965664813558e-46 51.9512195121951 1.41505778146841
39 169 nuclear-transcribed mRNA catabolic process
GO:0043241 4.86135258011712e-46 54.214859437751 1.32295342882845 38
158 protein complex disassembly
GO:0034623 5.73253131166412e-46 50.009485094851 1.45692339630475 39
174 cellular macromolecular complex disassembly
GO:0006402 1.52190575946733e-45 48.5566766099316 1.49041588817383
39 178 mRNA catabolic process
GO:0032984 1.93497356766425e-45 48.2064459930314 1.4987890111411 39
179 macromolecular complex disassembly
GO:0006401 1.46130671957922e-44 42.3943135054246 1.71649020829008
40 205 RNA catabolic process
GO:0072594 4.95839958975132e-43 40.830376940133 1.70811708532281 39
204 establishment of protein localization to organelle
GO:0019058 3.03134643812145e-39 33.5355515325551 1.93419140543907
38 231 viral infectious cycle
GO:0022415 1.12270140301278e-38 30.3637015781923 2.17701197148986
39 260 viral reproductive process
GO:0071845 1.42567564632147e-38 28.508399109492 2.37796692270431 40
284 cellular component disassembly at cellular level
GO:0022411 2.54419471907449e-38 28.0406212664277 2.41145941457339
40 288 cellular component disassembly
GO:0006605 7.84613517573552e-31 16.4485277136259 3.96886028648536

41	474	protein targeting
GO:0016032	1.81630407034692e-30	16.7240395302034	3.77627845823818
40	451	viral reproduction
GO:0033365	1.17391236470769e-29	15.8492287514612	3.9604871635181 40
473	protein localization to organelle
GO:0042273	2.69734245956887e-10	124.55652173913 0.100477475607224	6
12	ribosomal large subunit biogenesis
GO:0022613	6.02907803749116e-10	9.51482479784367	1.88395266763546
15	225	ribonucleoprotein complex biogenesis
GO:0071843	1.39171583155575e-09	8.91130390835579	2.00117638917722
15	239	cellular component biogenesis at cellular level
GO:0042254	4.12671955524885e-09	11.4053438692828	1.24759532212304
12	149	ribosome biogenesis
GO:0006364	1.56556302110038e-08	13.64385662258	0.870804788595945	10
104	rRNA processing
GO:0016072	2.69705696344428e-08	12.8198198198198	0.921043526399557
10	110	rRNA metabolic process
GO:0034470	1.11109670401426e-07	8.2814756978333 1.67462459345374	12
200	ncRNA processing
GO:0034660	1.1214091803254e-07	6.7878056350657 2.38634004567158	14
285	ncRNA metabolic process
GO:0042274	1.30689592018947e-05	34.959706959707 0.150716213410837	4
18	ribosomal small subunit biogenesis
Tissue: Artery_Tibial=>Thyroid	Type:	asymmetric
SourceGene:	SERTAD2(ENSG00000179833.3)
TargetGeneSet:	UBE2J2	RP4	LZIC	DFFA	CLCN6	DNAJC16	ARHGEF19
ARHGEF19-AS1	CROCCP3	RP1	RCC2	TCEB3	SRRM1	NUDC	XKR8	MED18
PHACTR4	PRDX3P2	TAF12	GMEB1	SNRNP40	TINAGL1	PEF1	TMEM39B	TXLNA	HDAC1
ZMYM1	LSM10	INPP5B	ZMPSTE24	SLFNL1	RNF220	RP11	C1orf123
MYSM1	NFIA	ZNHIT6	CEPT1	GOLPH3L	ARNT	GABPB1	LYSMD1	SNX27	CHTOP	SHE
PYGO2	FLAD1	UBQLN4	BGLAP	CCT3	ARHGEF11	USP21	POGK	METTL13
MRPS14	SMG7	BAP1	TRMT1L	CDC73	KLHL12	SOX13	TMEM81	CD46	FBXO28
PYCR2	C1orf131	MTR	OR2L3	PGBD2	SMC6	DNMT3A	C2orf18	PREB
SLC5A6	EIF2B4	PPM1G	SLC30A6	FAM98A	GEMIN6	PLEKHH2	ABCG5	MSH6	PAPOLG
PPP3R1	SNRPG	SPR	CCT7	CCDC142	AUP1	ART4	MRPL15	C2orf68	MRPL35
RMND5A	RPIA	MRPS5	ZNF2	ANKRD36C	LMAN2L	POLR1B	AMMECR1L
DNAJC10	ICA1L	WDR12	CREB1	RQCD1	TTLL4	COL4A3	EIF4E2	DTYMK	CHCHD4
C3orf19	ACAA1	GORASP1	ZNF620	NME6	C3orf62	DUSP7	SCT	FLNB-AS1	SHQ1
CLCP1	ABHD10	KIAA1407	ARHGAP31	POGLUT1	GTF2E1	PTPLB	ZNF148
SRPRB	MSL2	SLC25A36	UBQLN4P1	MYNN	ACTL6A	DCUN1D1	ABCF3	ALG3
POLR2H	CRYGS	MUC4	LRCH3	MRFAP1L1	UBE2K	DCAF4L1	CORIN	CLOCK
GRSF1	THAP9	COQ2	KIAA1109	IL15	LSM6	TIGD4	MFAP3L	IRF2
BRIP1	CTD	MARCH6	C5orf51	SNX18	IPO11	MRPS27	DMGDH	ZCCHC9	WDR36	PHAX
P4HA2-AS1	IGIP	HARS	G3BP1	GEMIN5	CTB	C6orf228	HIST1H2BM
TRIM27	LY6G6C	LSM2	AGPAT1	RING1	PFDN6	ARMC12	TAF8	RPL7L1	XPO5
TDRD6	MTO1	TSPYL4	GOPC	VIP	SUN1	C7orf26	MIOS	TRGJP1	CDK13
URGCP	PSPH	BAZ1B	RBM48	CCDC132	ZKSCAN5	ZKSCAN1	CNPY4	SLC12A9	CLDN15
C7orf53	METTL2A	TNPO3	SMO	NFE2L1	PRSS37	OR9A3P	ZNF786	ZNF398	ABCF2
RPL23AP53	CTA	NEIL2	POLR3D	CHMP7	PPP2R2A	INTS9	TM2D2	YTHDF3
ARMC1	TERF1	INTS8	GRHL2	DERL1	TG	ZNF623	ZNF252	SNAPC3	KLHL9
DCAF10	FXN	C9orf40	CEP78	BICD2	FOXE1	MURC	RNF20	NIPSNAP3B
C9orf84	PKN3	TBC1D13	TOR1B	EXOSC2	BRD3	C9orf69	SDCCAG3	TAF3	NUDT5
C10orf140	BMI1	ZNF485	ZFAND4	ERCC6	CSTF2T	ADO	MSS51	FUT11
AP3M1	TEP1	BTAF1	MYOF	BTRC	TAF5	SMC3	FAM175B	AP2A2	CHRNA10	RRP8
ZNF143	RNF141	MPPED2	SLC35C1	CELF1	TTC9C	ZBTB3	MARK2	STIP1	PLCB3	MEN1
DPF2	MRPL21	KRTAP5-9	UCP3	PPME1	C11orf30	RSF1	FAM76B	RDX
C11orf93	ZW10	PAFAH1B2	DDX6	C2CD2L	ZNF202	HYLS1	VPS26B
RAD52	FKBP4	NCAPD2	KIAA1467	KIAA0528	REP15	BCDIN3D	TARBP2
SMUG1	DNAJC14	DYRK2	TMPO	SART3	ANAPC7	ATXN2	POP5	RNF34	C12orf65
SNRNP35	PUS1	GOLGA3	MRPS31P2	PSPC1-OT1	LNX2	NHLRC3	KBTBD6
KBTBD7	NAA16	RPS4XP16	DHRS12	RAP2A	PCID2	TTC5	PARP2	MRPL52
RBM23	AJUBA	ACIN1	ZFHX2	FITM1	NEMF	MAP3K9	PROX2	NEK9	AHSA1
CPSF2	BTBD7	TJP1	GOLGA8B	BUB1B	ZFYVE19	PPP1R14D	NDUFAF1	MYEF2	HDC

AP4E1 PIGB RNF111 NARG2 CSNK1G1 UBAP1L DIS3L SMAD6 FEM1B ANP32A HEXA
MPI ZSCAN2 WDR73 NR2F2 ASB7 FBXL16 CCDC154 ZNF205 USP7 ERCC4
UBFD1 MAZ TAOK2 GDPD3 SULT1A3 CD2BP2 TBC1D10B FBXL19-AS1
FBXL19 PAPD5 CTCF TMEM170A C16orf46 MLYCD USP10 ZC3H18
RNMTL1 SGSM2 RAP1GAP2 DHX33 DERL2 SLC16A13 ZSWIM7 PIGL
ALKBH5 C17orf51 SDF2 C17orf63 BLMH CRLF3 UTP6 ZNF830
PEX12 DHRS11 PIP4K2B MED1 CNP NAGLU FAM134C PSME3 RUNDC1 UBTF
C17orf69 UBE2Z ZNF652 PPP1R9B APPBP2 DCAF7 FTSJ3 SMARCD2 FAM104A HN1
UNK QRICH2 CANT1 ENGASE FSCN2 SIRT7 RP13 ZNF397 CCDC11 BCL2 DSEL
RTTN ZADH2 MIER2 LMNB2 ZNF555 PIAS4 ZBTB7A DPP9 SAFB PCP2
ELAVL1 PIN1 ZGLP1 ZNF627 ZNF441 ZNF799 ZNF490 WDR83 FARSA TRMT1
SAMD1 NR2F6 GLT25D1 SUGP1 ANKRD27 KCTD15 WTIP KRTDAP LIN37 ZNF790
ZNF571 ZNF781 ZNF546 MAP3K10 MIA IRGQ ZNF284 BLOC1S3 DMPK MYPOP
STRN4 KPTN KCNJ14 BAX SIGLEC16 NAPSA CTU1 ZNF615 ZNF808
ZNF347 ZNF677 ZNF761 U2AF2 ZNF264 ZNF543 ZNF547 ZNF749 ZNF549 ZNF586
ZNF587 ZNF814 ZNF417 ZNF329 ZSCAN22 ZNF132 FAM110A NOP56 NANP COMMD7
DNMT3B RBL1 FITM2 RP3 ZSWIM3 ZSWIM1 NCOA5 ELMO2 ARFGEF2 KRT18P4
TMEM189 ADNP ZFP64 CSTF1 VAPB TCFL5 DPH3P1 RBM11 TCP10L C21orf59
CECR5 KRT18P5 CRKL UBE2L3 ZNF70 HPS4 RNF215 PRR14L ANKRD54 POLR2F
XPNPEP3 L3MBTL2 HDHD1 SCML1 ATP6AP2 ZNF673 ZNF41 GPKOW PPP1R3F SHROOM4 PHF8
WNK3 FAM155B CXorf65 ACRC DMRTC1 PABPC5 UTP14A ZNF449 NAA10
GOBPID Pvalue OddsRatio ExpCount Count Size Term
GO:0071843 2.48233480270064e-06 3.45626671218069 7.06200262957581
22 239 cellular component biogenesis at cellular level
GO:0022613 3.37420959179176e-06 3.50405679513185 6.64832883537471
21 225 ribonucleoprotein complex biogenesis
GO:0034660 4.16704383617833e-06 3.14035538062234 8.42121652480797
24 285 ncRNA metabolic process
GO:0006353 4.24157232674311e-06 5.84808800419068 2.3933983807349 12
81 DNA-dependent transcription, termination
GO:0006397 7.8812546341673e-06 2.68889039793562 11.7897031347312
29 399 mRNA processing
GO:0000377 1.01642442008761e-05 3.48358505564388 6.02781814407307
19 204 RNA splicing, via transesterification reactions with bulged adenosine as nucleophile
GO:0000398 1.01642442008761e-05 3.48358505564388 6.02781814407307
19 204 nuclear mRNA splicing, via spliceosome
GO:0000375 1.43651180966434e-05 3.3906862745098 6.17555878485918 19
209 RNA splicing, via transesterification reactions
Tissue: Artery_Tibial=>Thyroid Type: asymmetric
SourceGene: TTI1(ENSG00000101407.8)
TargetGeneSet: DFFB ERRFI1 LZIC RP11 DFFA PLOD1 DNAJC16 ARHGEF19
ARHGEF19-AS1 CROCCP3 HP1BP3 TCEB3 RP1 XKR8 EYA3 MED18 PHACTR4
TXLNA SLFNL1 MMACHC C1orf123 MYSM1 GADD45A RP4 SLC35A3 ATXN7L2
CYB561D1 GDAP2 PIAS3 GOLPH3L LYSMD1 SNX27 INTS3 GBAP1 FAM189B CLK2
UBQLN4 SEMA4A VHLL CCT3 C1orf182 USP21 POGK VAMP4 SMG7
KLHL12 SOX13 TMEM81 SRGAP3 CD55 CD46 ATF3 PGBD2 ID2 TTC32
C2orf44 SLC5A6 CCDC121 UBASH3A EHD3 FAM98A GEMIN6 ARHGEF33 SNORA67
PAPOLG XPO1 DBIL5P2 PPP3R1 AUP1 MRPL35 RMND5A RPIA MRPS5 ZNF2
LMAN2L C2orf29 RBP4 AMMECR1L R3HDM1 C2orf77 ANKAR OSGEPL1 SATB2
ICA1L WDR12 SPAG16 BCS1L DTYMK CRELD1 C3orf19 RFTN1 GORASP1 ZNF619
ZDHHC3 NME6 DUSP7 ABHD6 SHQ1 ADPRH CDV3 SLC25A36 TIPARP-AS1
PIK3CA ABCC5 ABCF3 POLR2H CRYGS RFC4 TMEM128 DCAF4L1 YIPF7 REST
CENPC1 AREGB THAP9 CCNA2 IL15 BRIP1 MARCH6 C5orf51 CTD IPO11
ZNF366 ZCCHC9 FAM174A XBP1P1 P4HA2 SHROOM1 CTB HARS HARS2 PCDHGB8P
ARAP3 PCDH12 LSM11 CPEB4 TSPAN17 GNL1 PRR3 C6orf25 LSM2 NOTCH4
PFDN6 TAF8 GUSBP4 LINC00472 C6orf203 MIOS CDK13 URGCP PSPH
GS1 RHBDD2 RBM48 CCDC132 DLX5 ZKSCAN1 METTL2A TNPO3 CNOT4 ZNF398
ZNF862 GIMAP4 SLC35G5 HMBOX1 TTI2 C8orf40 ARMC1 INTS8 GAPDHP62
GRHL2 LRRC6 ZNF623 RANBP6 SNAPC3 KLHL9 FANCG C9orf103 SECISBP2
FAM22G ALG2 NR4A3 RNF20 TRIM32 LHX6 PRPS1P2 ZBTB43 SH2D3C CCBL1
EXOSC2 AK8 C9orf69 QSOX2 SDCCAG3 INPP5E NOXA1 FBXO18 CDNF BMI1
LINC00202 ZNF485 ERCC6 CSTF2T CHST3 MSS51 AP3M1 ENTPD7 C10orf2 BTRC

NSMCE4A	ZNF195	CHRNA10	UBQLNL	TRIM34	TRIM5	RRP8	ZNF143	SLC35C1	TTC9C	
ZBTB3	STIP1	PLCB3	RPS6KA4	MEN1	PPP2R5B	C11orf85		DPF2	RBM14	
SUV420H1		UCP3	C11orf30		FAM76B	C11orf34		ZW10	DPAGT1	
HSPA8	ZNF202	VPS26B	RAD52	FKBP4	KLRAP1	REP15	PUS7L	TUBA1C	BCDIN3D	
ACVRL1	KRT81	SMUG1	OBFC2B	TIMELESS		DYRK2	IL26	ALDH2	DYNLL1	
RNF34	GOLGA3	EFHA1	KBTBD6	KBTBD7	ITM2B	RPS4XP16		DHRS12	BORA	
DNAJC3-AS1		RAP2A	PROZ	PCID2	TTC5	PARP2	ACIN1	KLHDC1	HNRPCP	EXD2
MAP3K9	NEK9	AHSA1	SPTLC2	ZC3H14	RPS6KA5	BTBD7	GOLGA8B	THBS1	ZFYVE19	
PPP1R14D		ZFP106	CSNK1G1	PARP16	ZSCAN2	ZNF774	FBXL16	LMF1	ERCC4	
UBFD1	DCTN5	PLK1	MAZ	GDPD3	CNEP1R1	SALL1	DOK4	DDX19B	TMEM170A	
ZC3H18	GALNS	SRR	ZMYND15	DHX33	DERL2	SLC35G6	C17orf51		WSB1	
TMEM199	UTP6	CDK5R1	CCL2	CCT6B	DHRS11	PIP4K2B	WIPF2	LEPREL4	FAM134C	
HOXB-AS2		HOXB4	DLX4	PPP1R9B	PCTP	TRIM25	HEATR6	FTSJ3	HN1	
QRICH2	AFMID	CANT1	CBX2	RP13	HRH4	ZNF24	C18orf54		ONECUT2	
ZADH2	ZNF555	MRPL54	TNFSF9	PEX11G	ZGLP1	RAB3D	ZNF788	ZNF799	ZNF490	
WDR83	TRMT1	SAMD1	TMEM221	JUND	SUGP1	ZNF253	NUDT19	KCTD15	ZNF565	
ZNF260	HKR1	ZFP36	MIA	SMG9	ZNF284	ZNF225	GEMIN7	BLOC1S3	MYPOP	
CPT1C	NR1H2	NAPSA	CTU1	ZNF175	ZNF816	ZNF347	ZNF761	U2AF2	ZNF835	
ZNF264	ZNF543	ZNF547	ZNF749	ZNF549	ZNF550	ZNF814	ZNF329	ZSCAN22	ZNF132	
NOP56	CRNKL1	DNMT3B	ZNF341	EDEM2	NFS1	SCAND1	FITM2	ZSWIM3	ZSWIM1	
NCOA5	KRT18P4	ADNP	ZFP64	FAM209B	GTPBP5	ITSN1	HMGN1	PRDM15	DSTNP1	
CECR5	KRT18P5	CRKL	UBE2L3	NF2	RNF215	PRR14L	NCAPH2	SHANK3	PPP2R3B-AS1	
HDHD1	SCML1	CXorf36	ZNF673	ZNF41	NUS1P1	ZNF81	KCND1	SHROOM4	FAM123B	
BHLHB9	ZNF449									
GOBPID	Pvalue	OddsRatio		ExpCount		Count	Size	Term		
Tissue:	Thyroid=>Heart_Left_Ventricle				Type:	asymmetric				

SourceGene: APOM(ENSG00000204444.6) s

TargetGeneSet: HP1BP3 NUDC EYA3 RCC1 RP11 LEPROT CCBL2 RPAP2
ALG14 FAM46C APH1A GOLPH3L CCT3 USP21 HSD17B7 CENPL CACYBP TMEM81
INTS7 IRF2BP2 ZNF692 PQLC3 SLC5A6 HNRPLL XPO1 CCT4 C2orf42 CCT7 AUP1
LMAN2L LYG1 GPR17 ARL6IP6 DNAJC10 HSPD1 SATB2 SLC23A3 CRELD1 NUP210
NGLY1 STT3B TRANK1 ZNF619 CCK HEMK1 PPP4R2 CPOX TPRA1 MLF1 FXR1
SOX2 PPP1R2 TCTEX1D2 CEP19 ABCA11P HGFAC DCAF16 TBC1D19 PGM2
APBB2 GNPDA2 UBE2D3 TBCK PLK4 CTSO PPID SH3RF1 ANKRD37 C5orf55 CCT5
CTD GPBP1 KLHL35 FAM13B BRD8 WDR55 PCDHB16 ADRB2 SNRNP48 GNL1
CNPY3 RP1 HSP90AB1 AARS2 FBXO9 PM20D2 SESN1 MICAL1 RSPH4A TCP1
KLHL7-AS1 TRA2A STYXL1 SAP25 MOSPD3 NAPEPLD RBM28 C7orf29 GBX1
SLC39A14 GTF2E2 THAP1 PYCRL ZNF623 NRBP2 DNAJA1 RECK C9orf71
NCBP1 KIAA0368 ZBTB34 FPGS MAMDC4 WAC OR13A1 PBLD HNRNPH3
P4HA1 AP2A2 C11orf91 EML3 NXF1 STIP1 SLC25A45 EED
CHORDC1 SRSF8 PHLDB1 DPAGT1 HINFP HSPA8 RAD52 FKBP4 RP4 IFFO1
CDKN1B TM7SF3 DAZAP2 DNAJC14 PAN2 PTGES3 ARHGAP9 HSP90B1 SELPLG LINC00173
GCN1L1 CRYL1 HSPH1 LHFP RPS4XP16 SUGT1 GPR180 PCID2 ABHD4
RBM23 ACIN1 ZC2HC1C AHSA1 ZC3H14 PAPOLA VRK1 HSP90AA1 ATPBD4
TMEM87A PIGB ANKDD1A C15orf44 POLG DNASE1L2 TBC1D24 GDPD3
ZDHHC7 CDK10 MIR22HG C17orf108 UNC119 DHRS13 KAT2A NMT1 STXBP4
WIPI1 QRICH2 JMJD6 C17orf90 IMPACT ABCA7 CIRBP SAFB PNPLA6 RETN
ZNF788 ATP13A1 DPY19L3 MED29 DEDD2 KPTN ZNF580 TRIM28 PYGB TTI1
SNHG11 RNF114 DNAJC5 USP16 MORC3 TOP3B PRR14L TRIOBP RANGAP1 FAM118A
TBC1D22A GPR173 RP6

GOBPID	Pvalue	OddsRatio	ExpCount	Count	Size	Term
GO:0006457	5.05E-14	10.53814103	2.46626531	21	216	protein folding

GO:0006458 1.69E-06 14.34033372 0.582312643 7 51 'de novo' protein folding

GO:0051084 1.30E-05 13.43962264 0.525223168 6 46 'de novo' posttranslational protein folding

Tissue: Heart_Left_Ventricle=>Thyroid Type: asymmetric

SourceGene: ATP13A1(ENSG00000105726.11) ATPase type 13A1

TargetGeneSet: C1orf159 HP1BP3 TXLNA CTPS U2 CCT3 C1orf114 PREB SLC5A6 FAM98A CCT4 SPR CCT7 LMAN2L ARL6IP6 SSB STAT4 NEK4 KIAA1407 RP11 CCT5 RPP40 SNRNP48 HIST1H2BK APOM RP1 PEX6 HSP90AB1 TDRD6 MTO1 LCA5 CA3 TCEA1P1 DNAJA1 PTAR1 GTF3C5 DNA2 CTD IPO7 STIP1 C2CD3 EED CEP57 RNF214 FKBP4 RP4 GPR162 CCT2 AMDHD1 OR7E36P NEK9 AHSA1 HSP90AA1 EXOC3L4 FBXL19-AS1 CYB5D2 MED31 C17orf108 SDF2 MLLT6 KAT2A PHB ZNF441 CTA LARGE HDHD1 FAM45B KDM5D

GOBPID	Pvalue	OddsRatio	ExpCount	Count	Size	Term
GO:0006457	2.28E-10	18.57632362	0.777247249	11	216	protein folding
GO:0051084	6.07E-07	37.25480021	0.165524877	5	46	'de novo' posttranslational protein folding
GO:0006458	1.03E-06	33.19380204	0.183516712	5	51	'de novo' protein folding

Tissue: Thyroid=>Heart_Left_Ventricle Type: asymmetric

SourceGene: CRIPT(ENSG00000119878.5) cysteine-rich PDZ-binding protein

TargetGeneSet: RP11 DFFB EPHA2 MFAP2 HSPG2 NUDC EYA3 RCC1
PTPRU C1orf212 ZNF691 GPBP1L1 SLC35D1 CYR61 KIAA1107 RPAP2
FRRS1 SASS6 NOTCH2 ECM1 GOLPH3L BNIPL LYSMD1 CREB3L4 PYGO2 FAM189B CCT3
CRABP2 CD1C CASQ1 USP21 B4GALT3 HSD17B7 RGS5 METTL13 FAM163A LAMC1
TMEM81 RBBP5 ACTA1 TFB2M PXDN NBAS RHOB C2orf44 EMILIN1 SLC5A6 ASB3

XPO1	C2orf42	SPR	CCDC142	AUP1	HTRA2	LOXL3	SEMA4F	ELMOD3	LINC00152	
MRPS5	LMAN2L	FBLN7	STEAP3	TNFAIP6	ITGA4	COL3A1	COL5A2	SATB2	IDH1	FN1
BCS1L	SLC23A3	FAM134A	COL6A3	LRRFIP1	CRELD1	TRANK1	ZNF619	IP6K1	TNNC1	
EPHA3	CPOX	ABI3BP	CBLB	CCDC80	CHCHD6	TPRA1	IL20RB	ARID4A	SSR3	
PPM1L	FXR1	EIF4A2	HRASLS	PPP1R2	ABCA11P	SPON2	WDR1	APBB2	11-Sep	
BMP2K	TIGD2	UNC5C	ADH1A	UBE2D3	TBCK	ARSJ	SEC24D	PDE5A	CTSO	PPID
SH3RF1	LPCAT1	CTD	SLC38A9	KLHL35	LHFPL2	ARSK	FAM13B	BRD8	CYSTM1	SOX9
TCERG1	MIR143HG		CSF1R	PDGFRB	SPARC	C5orf4	PDLIM7	ZNF184	GNL1	
COL11A2	RGL2	TEAD3	TOMM6	SRF	HSP90AB1		FBXO9	EYS	MTO1	
COL12A1	KIAA1009		PM20D2	SESN1	RSPH4A	SLC35F1	TRMT11	BMPR1APS1		
TCF21	FNDC1	TCP1	TTYH3	ZNF890P	ACTB	C7orf26	TRA2A	RP9P	7-Sep	VWC2
GRB10	PSPH	SEMA3C	KIAA1324L		COL1A2	ZKSCAN5	FAM200A	TAF6	GCC1	ARF5
CALU	TNPO3	ZYX	CHPF2	PPP2R2A	GTF2E2	PLAT	THAP1	SDC2	UHRF2	
TYRP1	DNAJB5	TPM2	GLIPR2	GNE	RG9MTD3	NOL8	OGN	ECM2	COL15A1	
KIAA0368		ZBTB34	FPGS	C9orf114		COL5A1	COBRA1	WAC	ITGB1	
ZNF37BP	CSGALNACT2		EGR2	FRAT1	FRAT2	MARVELD1		KAZALD1	TMEM180	TAF5
FAM160B1		VENTX	AP2A2	HTATIP2	SLC39A13		LRRC55	PRG2	SLC43A1	
STIP1	MEN1	CFL1	EFEMP2	EIF1AD	RELT	RAB6A	PICALM	SCN4B	IFT46	
PHLDB1	DPAGT1	HINFP	SRPR	RAD52	FKBP4	RP4	TSPAN9	NTN4	EMP1	
REP15	FAR2	C12orf35		LIMA1	ZC3H10	PAN2	AVIL	CTDSP2	MSRB3	
BBS10	CMKLR1	SIRT4	CCDC169	LHFP	EBPL	SUGT1	LINC00346		ADPRHL1	
ARHGEF40		LRFN5	RPL32P29		FRMD6	DACT1	MAX	ZFYVE1	ABCD4	
C14orf118		VRK1	PLD4	FAN1	ACTC1	ATPBD4	GPR176	BAHD1	ZFYVE19	
MFAP1	CTDSPL2	FBN1	TPM1	ANKDD1A	CILP	IGDCC4	NPTN	LOXL1	COMMD4	
WDR61	ADAMTSL3		POLG	MRPL28	RHOT2	PGPEP1	TBC1D24	VASN	MAZ	
BCL7C	HERPUD1	HSD11B2	TSNAXIP1		CENPN	CDK10	DEF8	RNMTL1	ZMYND15	PFN1
SPAG7	DERL2	EIF5A	CTC1	C17orf39		SMCR7	SDF2	ZNF830	FBXL20	
KAT2A	FAM134C	NMT1	SCRN2	COPZ2	COL1A1	MRC2	FTSJ3	GGA3	RECQL5	
SRP68	QRICH2	SPHK1	ARHGAP28		CXXC1	POLRMT	MOB3A	PNPLA6	ZNF558	KRI1
CNN1	ZNF788	PRKACA	C19orf44		DDA1	DPY19L3	ZNF585B	PSMC4	LTBP4	
TMEM91	DEDD2	ZNF233	VASP	KPTN	LIN7B	ZNF473	SHANK1	ZNF577	ZNF816	
CCDC106	ZNF667	ZNF211	ZNF671	ZNF329	SNRPB	DDRGK1	SLC23A2	CRNKL1	PYGB	
ZNF337	TTI1	KIAA1755		PKIG	SYS1	PCMTD2	LINC00478		C21orf119	
RCAN1	C21orf2	COL6A1	COL6A2	MMP11	TFIP11	DUSP18	MYH9	MFNG	TRIOBP	
GALR3	RANGAP1	TOB2	POLDIP3	GPM6B	DMD	GPR173	SPIN4	RP6	ARMCX4	
RAB9B	NRK	KIAA1210		SRPK3	NLGN4Y					

GOBPID	Pvalue	OddsRatio	ExpCount	Count	Size	Term

GO:0030198	2.21E-10	6.896178093	3.431458031	20	161
extracellular matrix organization					
GO:0043062	2.47E-10	6.847124413	3.452771435	20	162
extracellular structure organization					

GO:0007411	1.27E-06	3.486063596	7.310497543	23	343	axon guidance
GO:0030199	4.95E-06	12.62701252	0.703342329	7	33	collagen fibril organization

GO:0030029	1.87E-05	2.844863297	9.207390492	24	432

actin filament-based process

Tissue: Thyroid=>Heart_Left_Ventricle Type: asymmetric

SourceGene: CXCL17(ENSG00000189377.3) chemokine (C-X-C motif) ligand 17 (no ESTs in heart or thyroid)

TargetGeneSet: WRAP73 DFFB HSPG2 ASAP3 PTPRU COL16A1 GPBP1L1 NRD1 RPAP2 CTSO BNIPL EFNA4 USP21 DPT TMEM81 C2orf44 ARHGEF33 SEMA4F PROM2 FBLN7 LYPD1 DCAF17 FRZB COL3A1 COL5A2 FN1 RBM6 ABI3BP COL6A6 ARID4A SPON2 11-Sep ARSJ PDE5A RAB33B ADAMTS12 KLHL35 LOX PHF15 SPARC RP11 ZFP62 DTNBP1 ZNF184 ZNRD1-AS1 MOCS1 TPBG KIAA1009 CASP8AP2 FNDC1 VWC2 COL1A2 CALU ZYX MATN2 COL14A1 GLIS3-AS1 TYRP1 RG9MTD3 OGN ASPN ECM2 TNFSF8 COL5A1 ITGB1 ZNF37BP SPON1 SLC39A13 DPAGT1 HINFP PUS3 CLEC4A EMP1 C12orf35 LUM SLC41A2 TMEM119 TMEM233 COL4A1 COL4A2 FRMD6 ABCD4 GPR176 FBN1 CILP LOXL1 VASN MMP2 TXNL4B CDK10 ASGR1 MFAP4 FLOT2 MLLT6 STAT3 COL1A1 GGA3 WDR45L NR2C2AP ZNF600 DDRGK1 PYGB FAM83D RP3 COL6A2 CTA GPM6B LAMP2

GOBPID	Pvalue	OddsRatio	ExpCount	Count	Size	Term
GO:0030198	5.75E-15	20.10539346	1.047263165	16	161	extracellular matrix organization

GO:0043062	6.34E-15	19.9662803	1.053767905	16	162
extracellular structure organization					
GO:0030199	4.78E-13	63.23382353	0.214656425	9	33
collagen fibril organization					
GO:0071230	2.28E-09	41.17528736	0.227665905	7	35
cellular response to amino acid stimulus					
GO:0071229	7.37E-09	33.89486139	0.266694346	7	41
cellular response to acid					
GO:0071418	3.12E-08	26.78374766	0.325237008	7	50
cellular response to amine stimulus					

GO:0071417	4.14E-08	25.58978289	0.338246488	7	52
cellular response to organic nitrogen					
GO:0043200	2.01E-07	19.83610781	0.42280811	7	65
response to amino acid stimulus					
GO:0001101	1.28E-06	14.72929561	0.552902913	7	85
response to acid					
GO:0014075	4.69E-06	11.95246648	0.669988236	7	103
response to amine stimulus					
GO:0001957	5.26E-06	157.7362637	0.039028441	3	6
intramembranous ossification					

GO:0036072	5.26E-06	157.7362637	0.039028441	3	6
direct ossification

GO:0007411	1.37E-05	5.598599216	2.231125874	11	343	axon
guidance

GO:0043588	1.94E-05	17.47801661	0.331741748	5	51	skin
development

Tissue: Thyroid=>Heart_Left_Ventricle	Type:	asymmetric

SourceGene:	DNAJA3(ENSG00000103423.9)	"DnaJ (Hsp40) homolog, subfamily A,
member 3"

TargetGeneSet:	KLHL21	HAX1	MEF2D	MRPL24	TAGLN2	ADCY10	MYL3	FTH1P23
NAP1L5	RPS23	RNF44	FGFR4	SSR1	LEMD2	TOMM7	BMPER	MRPS24	CTA
NDUFB2	RP11	MMP16	NDUFB9	GRIN3A	PSMB7	USP20	PPP1R26	RSU1	NDUFB8
NDUFS3	C11orf10	COX8A	ADRBK1	PRKRIR	C11orf67	ZBTB44	FOXJ2	FAR2
RHOJ	SLIRP	NDUFB1	YY1	AP4E1	TEPP	PSMB6	PSMB3	SAFB2	C19orf79
OLFM2	COX6B1	POLR2I	FBL	ETFB	PROCR	ROMO1	RPS21	HSPA13	NDUFV3	GGA1
NDUFB11	HSD17B10	COX7B

GOBPID	Pvalue	OddsRatio	ExpCount	Count	Size	Term

GO:0022904	2.98E-14	45.1804878	0.345443222	11	96	respiratory electron transport chain
GO:0022900	1.26E-12	31.13940115	0.482181164	11	134	electron transport chain
GO:0045333	2.39E-12	29.22137405	0.510968099	11	142	cellular respiration
GO:0006120	2.89E-09	62.47391304	0.129541208	6	36	"mitochondrial electron transport, NADH to ubiquinone"
GO:0015980	9.26E-09	12.78287739	1.104698637	11	307	energy derivation by oxidation of organic compounds
GO:0042773	1.55E-08	45.6776246	0.169123244	6	47	ATP synthesis coupled electron transport
GO:0042775	1.55E-08	45.6776246	0.169123244	6	47	mitochondrial ATP synthesis coupled electron transport
GO:0006119	5.66E-08	35.98745819	0.20870528	6	58	

oxidative phosphorylation

GO:0006091 2.74E-07 8.995847225 1.540101031 11 428
generation of precursor metabolites and energy

Tissue: Thyroid=>Heart_Left_Ventricle Type: asymmetric

SourceGene: ENOSF1(ENSG00000132199.11) enolase superfamily member 1

TargetGeneSet: KLHL17 CPSF3L TAS1R3 TNFRSF14 RP1 HP1BP3 NUDC RP11
GPBP1L1 RPAP2 PRPF38B CCT3 USP21 CACYBP TMEM183A ZC3H11A TMEM81
INTS7 MTR SMC6 SLC5A6 ASB3 CCT4 AFTPH C2orf42 SPR PCGF1 AUP1
MRPS5 ARL6IP6 DNAJC10 HSPD1 ILKAP CRELD1 GHRLOS CHCHD4 NGLY1 ABHD14A-ACY1
CBLB SLC9C1 TPRA1 FXR1 DCUN1D1 PGM2 NUDT10 PLK4 CTSO PPID CCT5
MEF2C BRD8 WDR55 PCDHB16 ZNF300P1 SNRNP48 GNL1 CNPY3 HSP90AB1
VIP TCP1 PARK2 NUPL2 RP9 PSPH STYXL1 MAGI2 FAM133B THAP1
C8orf44 PYCRL DNAJA1 GNE FAM95B1 GKAP1 NCBP1 FAM73B RP11-88G17 WAC
HNRNPH3 DNA2 ANXA7 KIAA0913 C10orf131 PPP2R2D HTATIP2 NXF1
STIP1 EIF1AD ANO1 CAPN5 CHORDC1 MED17 ZBTB44 RAD52 FKBP4 RP4
IFFO1 REP15 PTGES3 SIRT4 ZCCHC8 C12orf65 HSPH1 SUGT1 PCID2
ACIN1 DHRS1 TRIM9 ZC2HC1C AHSA1 SMEK1 PAPOLA HSP90AA1 EHD4
DTWD1 ANKDD1A DPP8 C15orf42 SPNS3 C17orf108 COASY HOXB-AS1
MRC2 QRICH2 JMJD6 CIRBP SAFB PEX11G SAMD1 ZNF91 DPY19L3 DEDD2 KPTN
TRPM4 TFPT SLC23A2 RRBP1 DDX27 RNF114 STMN3 MORC3 THAP7-AS1
TRIOBP RANGAP1 CYB5R3 ARSD GPR173 GDPD2

GOBPID Pvalue OddsRatio ExpCount Count Size Term

GO:0006457 1.86E-10 10.44332791 1.733859248 15 216 protein folding

GO:0006458 3.15E-06 17.32121212 0.409383434 6 51 'de novo' protein folding

Tissue: Heart_Left_Ventricle=>Thyroid Type: asymmetric
SourceGene: ENSG00000233038.1
TargetGeneSet: FCRL5 LIN9 IGKJ5 IGKC IGKJ3 IGKJ2 IGKV1-16 IGKV2-28 IGKV1-39 MST1 RP11 KCNMB2-IT1 TMPRSS11CP IGJ C7orf50 BMPER PCLO IL18 MEG3 IGHA2 IGHG2 IGHGP IGHG1 IGHG3 IGHV3-43 SNORA10 TGIF1 IGLV3-21 IGLV2-8 IGLC3 ASMT MAMLD1

GOBPID	Pvalue	OddsRatio	ExpCount	Count	Size	Term
GO:0006958	1.71372213274009e-09	145.353535353535	0.0553594906926856	5	50	complement activation, classical pathway
GO:0006956	5.63834339914804e-09	112.672413793103	0.0697529582727839	5	63	complement activation
GO:0002455	6.61742347366508e-09	108.901515151515	0.0719673379004913	5	65	humoral immune response mediated by circulating immunoglobulin
GO:0072376	2.16244266875052e-08	84.7579693034238	0.0907895647360044	5	82	protein activation cascade
GO:0002460	2.5644627071602e-08	48.8914285714286	0.200401356307522			

6	181	adaptive immune response based on somatic recombination of immune receptors built from immunoglobulin superfamily domains
GO:0002250	4.38173798530615e-08	44.509375	0.219223583143035	6
198	adaptive immune response
GO:0016064	9.97147565448117e-08	61.4451114922813	0.122898069337762
5	111	immunoglobulin mediated immune response
GO:0019724	1.13997696105075e-07	59.7414512093411	0.126219638779323
5	114	B cell mediated immunity
GO:0006959	1.60147054710837e-07	55.6254856254856	0.135077157290153
5	122	humoral immune response
GO:0002449	7.64940235407775e-07	40.0476992143659	0.18490069891357
5	167	lymphocyte mediated immunity
GO:0002443	2.42520447463488e-06	31.3967343336275	0.233617050723133
5	211	leukocyte mediated immunity
GO:0002253	1.32836322946249e-05	21.8630179344465	0.33104975434226
5	299	activation of immune response
Tissue: Thyroid=>Heart_Left_Ventricle	Type:	asymmetric

SourceGene:	FAAH2(ENSG00000165591.6)	fatty acid amide hydrolase 2

TargetGeneSet:	PLA2G2A	ASAP3	PDIK1L	PTPRU	GPBP1L1	YIPF1	GNG12	CYR61	RP11
BNIPL	CREB3L4	PYGO2	FAM189B	USP21	HSD17B7	DPT	PRRX1	LAMC1	ZC3H11A
ITPKB-IT1	KLHL29	C2orf44	ARHGEF33	HTRA2	LOXL3	SEMA4F	PROM2
FBLN7	COL3A1	COL5A2	FN1	B3GNT7	CRELD1	C3orf45	TNNC1	VGLL3	EPHA3
ABI3BP	BZW1P2	COL6A6	ECT2	ST6GAL1	BCL6	SPON2	11-Sep	UNC5C	CXXC4	ARSJ
LPCAT1	SLC38A9	LHFPL2	LOX	GRAMD3	PHF15	H2AFJ	ZNF354B	ID4	ZNRD1-AS1
TMEM63B	TDRD6	EYS	KIAA1009	PM20D2	CASP8AP2	TCF21	FNDC1
WDR27	LFNG	VWC2	GS1	COL1A2	SLC12A9	ZYX	INTS9	PPP2CB	PLAT	GDF6
IGHEP2	TYRP1	IL11RA	RG9MTD3	OGN	OMD	ECM2	KIAA1958	COL5A1
ITGB1	NAMPTL	ZNF37BP	DNAJC12	INSC	SLC39A13	MEN1	EFEMP2	PICALM
AMOTL1	KBTBD3	TAGLN	DPAGT1	PUS3	RAD52	EMP1	FAR2	C12orf35

LIMA1 GLI1 GLIPR1 NUDT4 CMKLR1 TMEM119 DTX1 CCDC169 RPS4XP16
GPR180 ADPRHL1 ARHGEF40 LRFN5 FRMD6 ABCD4 GPR176 BAHD1 GLDN CTD
ANKDD1A LRRK1 VASN CDH11 HSD11B2 FTSJD1 TXNL4B CENPN CDK10 SERPINF2
NLGN2 MFAP4 RAB34 MLLT6 STAT3 PTRF C17orf53 COPZ2 COL1A1
RNF43 KCNJ2 ENGASE ACTG1 WDR45L ARHGAP28 ZADH2 POLR2E PNPLA6
ZNF558 OLFM2 COL5A3 TPM4 TSHZ3 ANKRD27 PSMC4 LYPD5 ZNF233 ZFP112
ZNF577 ZNF211 DDRGK1 SLC24A3 EMILIN3 PCMTD2 C21orf2 COL6A1 COL6A2 GPM6B
PPP1R3F SPIN4 PLS3 KIAA1210 PGRMC1 LAMP2 SRPK3 VAMP7

GOBPID	Pvalue	OddsRatio	ExpCount	Count	Size	Term
GO:0030199	1.51E-11	41.86523438	0.312850322	9	33	collagen fibril organization
GO:0030198	3.15E-11	11.93128228	1.526330358	15	161	extracellular matrix organization

GO:0043062	3.44E-11	11.8492807	1.535810671	15	162
extracellular structure organization					
GO:0031589	6.19E-08	7.835168117	1.915023182	13	202
cell-substrate adhesion					
GO:0010810	2.55E-07	11.7703125	0.891149401	9	94
regulation of cell-substrate adhesion					
GO:0070208	1.55E-06	71.71929825	0.094803128	4	10
protein heterotrimerization					

GO:0043588	8.25E-06	14.52315522	0.483495952	6	51	skin development
GO:0071230	1.84E-05	18.03535354	0.331810947	5	35	cellular response to amino acid stimulus

Tissue: Thyroid=>Heart_Left_Ventricle Type: asymmetric

SourceGene: HIST1H2BD(ENSG00000158373.7) "histone cluster 1, H2bd"

TargetGeneSet: KLHL17 MXRA8 TNFRSF14 RP13 RP11 ZBTB17 HP1BP3 RCC1
IPO13 GPBP1L1 LEPROT SLC35D1 RPAP2 CEPT1 RP4 APH1A GOLPH3L BNIPL
S100A9 DENND4B FAM189B CCT3 KIRREL USP21 B4GALT3 HSD17B7 CACYBP TMEM183A
ZC3H11A TMEM81 INTS7 CAPN2 ARF1 MTR ZNF672 ZNF692 SMC6 CCT4
AFTPH C2orf42 SPR PCGF1 LMAN2L DNAJC10 COL5A2 HSPD1 ICA1L IKZF2
SLC23A3 FBXO36 COL6A3 CRELD1 CHCHD4 PLCL2 NGLY1 ZNF619 RBM6 MANF
PPP4R2 CPOX MSL2 MYNN FXR1 EIF4A2 PPP1R2 APBB2 SRP72 TBCK GLRB
PPID SH3RF1 LPCAT1 CCT5 FAM105B GPBP1 P4HA2-AS1 P4HA2 WDR55
PCDHB16 ZNF300P1 PDLIM7 ZNF354A ZNF879 SNRNP48 ZNF184 SCAND3 GNL1
SLC39A7 RP1 TOMM6 CNPY3 HSP90AB1 TDRD6 BAI3 RARS2 PM20D2
C6orf203 VNN2 VIP SERAC1 FNDC1 PNLDC1 ZNF92 STYXL1 RBM48
SLC25A13 GIMAP4 SLC39A14 KIF13B GTF2E2 GINS4 THAP1 GSDMD
PYCRL ZNF623 NRBP2 DNAJA1 NOL6 RECK GKAP1 OGN NCBP1 COL15A1 COQ4
C9orf114 CCBL1 RP11-88G17 COL5A1 MAMDC4 NUDT5 WAC ITGB1 PBLD
DNA2 C10orf32 DCHS1 ZNF143 HTATIP2 C11orf46 STIP1 GPR137
SLC25A45 RBM14 RBM4B EED CHORDC1 MED17 SRSF8 DPAGT1 HSPA8
ZBTB44 RAD52 FKBP4 EMP1 REP15 C12orf35 LIMA1 DAZAP2 PAN2
PTGES3 ARHGAP9 MRPL42 HSP90B1 SELPLG C12orf65 HSPH1 RPS4XP16
SUGT1 COL4A2 PCID2 ACIN1 LRFN5 MLH3 ZC2HC1C AHSA1 SERPINA1
PAPOLA HSP90AA1 ZFYVE19 TRIM69 PIGB ANKDD1A IGDCC4 LOXL1 WDR61
DNASE1L2 TBC1D24 PRKCB FUS C16orf58 CENPN CDK10 RNF167
C17orf108 SDF2 RAB34 DHRS13 CCT6B ARHGAP23 KAT2A SCRN2
KCNJ2 HN1 QRICH2 JMJD6 HEXDC VPS4B ATP9B ABCA7 CIRBP GIPC3
C19orf59 WDR83 SAMD1 PRKACA DPY19L3 DEDD2 CD177 ZNF233 ZFP112 KPTN
PIH1D1 CTU1 FPR2 ZNF577 ZNF761 ZNF580 TRIM28 PTPRA DDRGK1 PYGB
IFT52 RP3 RNF114 STMN3 MORC3 THAP7-AS1 PRR14L MYH9 TRIOBP

RANGAP1 LMF2 ASMTL PPP1R3F GPR173 KIAA1210 HPRT1

GOBPID	Pvalue	OddsRatio	ExpCount	Count	Size	Term
GO:0006457	1.36E-09	6.942193676	3.019306622	18	216	protein folding

Tissue: Thyroid=>Heart_Left_Ventricle Type: asymmetric

SourceGene: MIA(ENSG00000261857.1) melanoma inhibitory activity

TargetGeneSet: FAM41C KLHL17 UBE2J2 CPSF3L RP11 TNFRSF14 HP1BP3 NUDC
EYA3 SLFNL1 RNF220 GPBP1L1 RP1 LEPROT SLC35D1 FPGT-TNNI3K TYW3
WDR63 CCBL2 RPAP2 CNN3 CEPT1 PTGFRN GDAP2 RP4 APH1A BNIPL
SNX27 FAM189B CLK2 PMF1 TMEM79 CCT3 USP21 HSD17B7 ADCY10 CACYBP
LAMC1 SMG7 SWT1 TMEM81 INTS7 JMJD4 TFB2M ZNF692 SMC6 C2orf44
SLC5A6 LTBP1 CDC42EP3 EML4 CCDC88A XPO1 CCT4 AFTPH C2orf42 SPR
PCGF1 ELMOD3 MRPS5 LMAN2L RFX8 METAP1D HSPD1 ALS2CR12 BCS1L
SLC23A3 RBM44 ILKAP CRELD1 C3orf19 NGLY1 SLC25A38 CPOX KIAA1524
WDR52 SEMA5B SIAH2 FXR1 PARL POLR2H MAGEF1 EIF4A2 BCL6 PPP1R2
CEP19 ABCA11P NOP14 DCAF16 PARM1 HELQ UBE2D3 TBCK SEC24D CTSO PPID
CPE SH3RF1 DCTD ENPP6 CCT5 CTD PPWD1 BRD8 SOX9 PCDHB16

PCDHGA2 NDST1 FNDC9 ADRA1B ZNRD1-AS1 GNL1 VWA7 HLA-DRA HLA-DMB
HLA-DMA SLC39A7 SPDEF HSP90AB1 SLC35B2 BAI3 COL12A1 SENP6 SNX3 RP3
RSPH4A FUCA2 C6orf97 TCP1 PNLDC1 CDK13 PSPH STYXL1 MAGI2 RBM48 TAF6
STAG3 CLDN15 TMEM168 ST7-AS1 DYNLL1 LZTS1 POLR3D ADAM28 KIF13B DCTN6
GTF2E2 TTI2 THAP1 RB1CC1 ZNF623 SCRIB CYHR1 MTND4P14 DNAJA1 GNE
GKAP1 NOL8 NCBP1 COL15A1 NIPSNAP3B DAB2IP PRPS1P2 ZBTB34 FPGS
RP11-88G17 QSOX2 CDNF ZNF37BP ERCC6 ANXA7 FGFBP3 C10orf131
FRAT2 C10orf62 C10orf2 C10orf32 TAF5 BAG3 C10orf125
AP2A2 PRKCDBP WT1-AS PIGCP1 NXF1 STIP1 SLC25A45 CFL1 EFEMP2
EIF1AD ZDHHC24 RBM14 RBM4B ANO1-AS1 CAPN5 ANKRD42 EED CHORDC1
MED17 TMPRSS5 PHLDB1 HINFP ZNF202 SRPR KCNJ5 RAD52 FKBP4 TAPBPL
C12orf71 REP15 PAN2 PTGES3 AVIL CRADD APPL2 PLBD2 SIRT4
HSPH1 FAM48A LHFP RPS4XP16 DHRS12 SUGT1 ACIN1 NEDD8 CTSG
RPL32P29 SLC38A6 SNAPC1 EIF2B2 POMT2 AHSA1 ZC3H14 PAPOLA VRK1
HSP90AA1 ATPBD4 EHD4 TMEM87A PIGB POLR2M DPP8 NPTN WDR61 PRC1
SOLH TPSAB1 TBC1D24 PDPK1 CLDN9 NPIP NPIPP1 ATXN2L GDPD3 CTF1
HERPUD1 TSNAXIP1 SLC7A6OS AARS BANP CDK10 DERL2 TRIM16
TRPV2 LGALS9 NUFIP2 ANKRD13B CCT6B TBC1D3 KAT2A NAGLU COASY NMT1
CUEDC1 DDX42 HELZ NT5C RECQL5 QRICH2 JMJD6 LRRC45 CXXC1 ATP9B
APBA3 SAFB2 SAFB PNPLA6 ZNF558 TRMT1 SAMD1 DEDD2 ZNF284 PPP1R37 KPTN
KCNJ14 IRF3 ZNF137P MYADM ZNF580 ZNF667 SLC23A2 CRNKL1 ZNF337 MT1P3
EPB41L1 PLCG1 PKIG PABPC1L DDX27 RNF114 C20orf11 C21orf2 C21orf67
THAP7-AS1 TFIP11 TPST2 PRR14L TRIOBP ST13 CYB5R3 ACR ARSD
HDHD1 ZNF673 RP6 ACRC RAB9B SLC6A8

GOBPID	Pvalue	OddsRatio	ExpCount	Count	Size	Term
GO:0006457	1.63E-10	6.586630836	3.736765622	21	216	protein folding

GO:0006986	1.47E-05	5.492979221	2.231679469	11	129

response to unfolded protein

Tissue: Thyroid=>Heart_Left_Ventricle   Type: asymmetric

SourceGene: NUDT3(ENSG00000112664.4) nudix (nucleoside diphosphate linked moiety X)-type motif 3

TargetGeneSet: FAM41C KLHL17 C1orf159 CPSF3L TAS1R3 DVL1 TNFRSF14
RP13 KLHL21 RP11 NMNAT1 ZBTB17 RSG1 HP1BP3 CDC42 ZBTB40 FUCA1
UBXN11 PIGV NUDC EYA3 RCC1 CITED4 RNF220 GPBP1L1 RP1 LEPROT
SLC35D1 TYW3 CCBL2 KIAA1107 RPAP2 ALG14 RP4 FRRS1 C1orf194
CEPT1 APH1A GOLPH3L BNIPL PSMB4 SNX27 FAM189B SMG5 CCT3 USP21
B4GALT3 HSD17B7 METTL13 CACYBP CEP350 TRMT1L GS1 TMEM183A ZC3H11A
TMEM81 INTS7 TARBP1 IRF2BP2 MTR ZNF692 SMC6 C2orf44 SLC5A6 LTBP1
ARHGEF33 EML4 CCDC88A BCL11A XPO1 CCT4 AFTPH C2orf42 SPR
PCGF1 AUP1 HTRA2 GCFC2 ELMOD3 RNF181 RNF103 RMND5A MRPS5 LMAN2L RFX8
HS6ST1 ARL6IP6 DNAJC10 HIBCH HSPD1 SATB2 ALS2CR12 TRAK2 IKZF2
XRCC5 SLC23A3 RBM44 CRELD1 CHCHD4 C3orf19 CAPN7 PLCL2-AS1 NGLY1
ZNF619 ZNF852 KIAA1143 RBM6 MANF PPP4R2 ZNF717 CGGBP1 CPOX CBLB
KIAA1524 SLC9C1 WDR52 KLF15 TPRA1 PPM1L FXR1 ATP11B DCUN1D1 PARL
MAGEF1 TBCCD1 BCL6 PPP1R2 UBXN7 ABCA11P HGFAC DCAF16 CTD RASGEF1B
NUDT10 RG9MTD2 UBE2D3 SLC9B1 TBCK PLK4 GAB1 CTSO PPID ANKRD37
LPCAT1 FAM134B NIPBL C5orf51 TRIM23 KLHL35 BRD8 SOX9 WDR55 PCDHB16
TCERG1 ZNF300P1 RANBP17 CREBRF PDLIM7 ZNF354A SNRNP48 GNL1 DDX39B
HLA-DRA HLA-DMB HLA-DMA SLC39A7 SPDEF ARMC12 TOMM6 HSP90AB1 TDRD6 BAI3
MTO1 RARS2 PM20D2 UFL1 SESN1 RP3 RSPH4A TRMT11 HECA VIP
SERAC1 FNDC1 PNLDC1 WDR27 C7orf50 KLHL7-AS1 ADCYAP1R1 7-Sep
CDK13 LANCL2 PSPH STYXL1 MAGI2 CLDN12 RBM48 FAM200A STAG3 ZCWPW1
PRKAR2B ARF5 ZYX C7orf29 GBX1 UBE3C ZNF596 SLC39A14 CHMP7
ADAM28 KIF13B GTF2E2 TTI2 GINS4 THAP1 MSC ZBTB10 ENY2 C8orf76
PYCRL ZNF623 NRBP2 CYHR1 ZNF250 KDM4C TYRP1 TOPORS DNAJA1 GNE
RG9MTD3 GKAP1 NOL8 NCBP1 GRIN3A CDC26 ZBTB34 FPGS C9orf114
RP11-88G17 RXRA FAM166A EXD3 NOXA1 USP6NL WAC ITGB1 NELL1
ZNF37BP ERCC6 HNRNPH3 DNA2 P4HA1 ANXA7 KIAA0913 KAT6B C10orf131
FRAT2 C10orf32 TAF5 MXI1 FAM160B1 ZRANB1 C10orf125
PRKCDBP ZNF143 HTATIP2 DNAJC24 PIGCP1 ALKBH3 CELF1 FADS3 NXF1 MARK2
STIP1 TRPT1 GPR137 MEN1 SLC25A45 CFL1 EFEMP2 EIF1AD CD248
RBM14 RBM4B CAPN5 EED CHORDC1 MED17 IFT46 PHLDB1 DPAGT1 HINFP OAF
SRPR PRDM10 ZBTB44 RAD52 CACNA2D4 FKBP4 IFFO1 CDKN1B WBP11
REP15 FAR2 C12orf35 SAT2 LIMA1 DAZAP2 PAN2 PTGES3 AVIL
CTDSP2 BBS10 SELPLG ALKBH2 DTX1 SIRT4 C12orf65 ZNF605 CRYL1
PTPN2P2 HSPH1 RPS4XP16 DHRS12 SUGT1 UGGT2 PCID2 ACIN1 SEC23A
FBXO33 LRFN5 RPL32P29 MAPK1IP1L MAX ABCD4 ZC2HC1C C14orf118
AHSA1 PAPOLA VRK1 HERC2P3 FAN1 ZFYVE19 EHD4 HAUS2 PIGB POLR2M
ANKDD1A IGDCC4 LOXL1 COMMD4 WDR61 POLG BLM WASH4P RHOT2 TBC1D24 NPIP
NPIPP1 CLN3 MAZ FUS C16orf58 FTO HERPUD1 HSD11B2 TSNAXIP1

TMEM231 CENPN KLHDC4 BANP CDK10 RNMTL1 SGSM2 SPNS3 PFN1 DERL2
EIF5A COX10-AS1 MEIS3 C17orf39 MEIS3P2 C17orf108 SDF2
RAB34 RHOT1 ARHGAP23 KAT2A CCR10 NMT1 SCRN2 SP2 DDX42 NT5C
RECQL5 QRICH2 JMJD6 CHMP6 ARHGDIA LRRC45 ARHGAP28 CABYR HNRNPA1P7
ASXL3 ZNF397 RNF165 CXXC1 ZNF236 ABCA7 CIRBP SAFB2 SAFB PRR22
PNPLA6 ZNF558 COL5A3 ZGLP1 KEAP1 WDR83 SAMD1 PRKACA SLC27A1 ZNF91
DPY19L3 ZNF571 FAM98C MED29 EID2B DEDD2 ZNF284 ZNF233 ZFP112 TMEM160 KPTN
FUT2 TSKS ZNF577 ZNF615 ZNF808 ZNF580 ZNF667 NSFL1C SNRPB PTPRA
DDRGK1 SLC23A2 PYGB MT1P3 MMP24 TTI1 IFT52 C20orf111 PKIG SYS1
NCOA5 RNF114 C20orf166-AS1 PCMTD2 HSPA13 LINC00478 MORC3 C21orf2
SCARF2 THAP7-AS1 TFIP11 TPST2 PRR14L TRIOBP H1F0 RANGAP1 PPPDE2
ASMTL PPP1R3F MAGED1 GPR173 RP6 MAGEC2 ARMCX4 BEX4 KIAA1210
FAM127C VAMP7 TMSB4Y

GOBPID	Pvalue	OddsRatio	ExpCount	Count	Size	Term
GO:0035511	1.67E-05	Inf	0.076811293	3	3	oxidative DNA demethylation

Tissue: Thyroid=>Heart_Left_Ventricle Type: asymmetric

SourceGene: PLD5(ENSG00000180287.11) "phospholipase D family, member 5"

TargetGeneSet: PODN RP11 BNIPL TKT FMOD NID1 RHOB IL1R1
SCN7A COL5A2 COL4A4 COL6A3 CLSTN2 PDGFRA 11-Sep SPOCK1 C5orf62 SNX14
EPB41L2 SASH1 KIAA1324L INTS9 RECK SVEP1 OR2K2 SPON1 EMP1
PTPRQ LIG4 AKAP5 FBLN5 MMP2 SF3B3 CRISPLD2 ASGR1 KCNJ2
SETBP1 DSEL MN1 CHRDL1

GOBPID	Pvalue	OddsRatio	ExpCount	Count	Size	Term

GO:0030198	8.73E-11	33.78552632	0.378797315	9	161
extracellular matrix organization

GO:0043062	9.23E-11	33.56235294	0.381150093	9	162
extracellular structure organization

Tissue: Thyroid=>Heart_Left_Ventricle	Type:	asymmetric

SourceGene:	PTPLB(ENSG00000206527.5)	"protein tyrosine phosphatase-like (proline instead of catalytic arginine), member b"

TargetGeneSet:	FAM41C	TAS1R3	TNFRSF14	RP1	WRAP73	ZBTB17	FBXO42
HP1BP3	FUCA1	PNRC2	NUDC	EYA3	RCC1	RP11	GPBP1L1	LEPROT	TYW3
CCBL2	RP4	ZNF326	RPAP2	ALG14	PRPF38B	SNX27	JTB	CCT3	USP21

HSD17B7 CACYBP TMEM183A TMEM81 INTS7 MTR ZNF672 ZNF692 THADA
CCDC88A CCT4 AFTPH C2orf42 PCGF1 AUP1 ELMOD3 MRPS5 ANKRD36C
LMAN2L MTND3P10 WDSUB1 HSPD1 SLC23A3 CRELD1 CHCHD4 PLCL2-AS1
NGLY1 DHX30 MANF FRMD4B KIAA1524 WDR52 TPRA1 MYNN FXR1
EIF2B5 EIF4A2 ABCA11P NOP14 DCAF16 CHIC2 NUDT10 UBE2D3 TBCK PLK4
GUSBP5 PPID CCT5 SOX9 WDR55 PCDHB16 PCDHGA2 ZNF300P1 FNDC9
ZNF184 GNL1 SLC39A7 SPDEF HSP90AB1 SLC35B2 RP3 RSPH4A C6orf97 VIP
TCP1 PNLDC1 C6orf70 HOXA10 STYXL1 MAGI2 RBM48 FAM133B SLC25A13
TMEM168 GBX1 LZTS1 TTI2 GOLGA7 GINS4 THAP1 EBAG9 PYCRL ZNF623
CYHR1 UHRF2 KDM4C DNAJA1 NOL6 C9orf100 HMGB3P24 GKAP1
NCBP1 RAB14 FPGS RP11-88G17 EXD3 TAF3 WAC ZNF32-AS2 PBLD
HNRNPH3 C10orf131 FRAT2 TAF5 BAG3 BUB3 PPP2R2D C10orf125 CTD
SLC22A18AS PRG2 FADS3 NXF1 STIP1 SLC25A45 EIF1AD ZDHHC24
RBM14 EED CHORDC1 MED17 HINFP HSPA8 KCNJ5 FKBP4 ATF1 PAN2
PTGES3 ALKBH2 PLBD2 HSPH1 FANCB INTS6 DHRS12 SUGT1 PCID2 RPL32P29
MAX EIF2B2 POMT2 AHSA1 PAPOLA HSP90AA1 TUBGCP5 TMEM87A MFAP1 PIGB
ANKDD1A PRC1 DNASE1L2 TBC1D24 MGRN1 NPIP NPIPP1 PKD1P6 BANP
SGSM2 DERL2 CNTROB MEIS3 MEIS3P2 C17orf108 UTP6 CCT6B KRT19
KAT2A NAGLU QRICH2 JMJD6 IMPACT ASXL3 CIRBP SAFB YJEFN3 DEDD2
TRPM4 NOSIP TSKS TFPT ZNF580 TRIM28 RRBP1 POFUT1 DDX27 RNF114
C20orf11 STMN3 MORC3 C21orf67 TPST2 PRR14L TRIOBP RANGAP1
C22orf26 GTSE1 ARSD GPR173

GOBPID	Pvalue	OddsRatio	ExpCount	Count	Size	Term
GO:0006457	8.80E-11	8.367201426	2.555947685	18	216	protein folding

GO:0000226	1.90E-05	4.507318038	3.183101515	13	269
microtubule cytoskeleton organization

Tissue: Heart_Left_Ventricle=>Thyroid	Type:	asymmetric
SourceGene:	RP11-135J2.4(ENSG00000228063.1)
TargetGeneSet:	FCRL5	IGKJ5	IGKC	IGKJ2	IGKV1-17	IGKV3D-20	AGA
UBTD2	MRPS17	ANKRD20A19P	SLC39A2	RP11	IGHG1	IGHG3	IGHJ5	IGHJ3
IGHV3-73	SBK1	IGLV1-40	IGLV2-11	IGLV2-8	IGLL5	IGLC1
IGLC3

GOBPID	Pvalue	OddsRatio	ExpCount	Count	Size	Term
GO:0006958	5.73461135878179e-13	392.618181818182	0.0380596498512214	6	50	complement activation, classical pathway
GO:0006956	2.44254125353039e-12	302.8	0.0479551588125389	6	63	complement activation
GO:0002455	2.96754069301776e-12	292.494915254237	0.0494775448065878	6	65	humoral immune response mediated by circulating immunoglobulin
GO:0072376	1.25167763479958e-11	226.8	0.062417825756003	6	82	protein activation cascade
GO:0016064	8.02400420274271e-11	163.828571428571	0.0844924226697114	6	111	immunoglobulin mediated immune response
GO:0019724	9.44257001614251e-11	159.244444444444	0.0867760016607847	6	114	B cell mediated immunity
GO:0006959	1.42774536701093e-10	148.179310344828	0.0928655456369801	6	122	humoral immune response
GO:0002449	9.58785477132851e-10	106.427329192547	0.127119230503079	6	167	lymphocyte mediated immunity
GO:0002460	1.55867569635427e-09	97.8171428571429	0.137775932461421	6	181	adaptive immune response based on somatic recombination of immune receptors built from immunoglobulin superfamily domains
GO:0002250	2.67678847039623e-09	89.05	0.150716213410837	6	198	adaptive immune response
GO:0002443	3.92363245231903e-09	83.3268292682927	0.160611722372154	6	211	leukocyte mediated immunity
GO:0002253	3.16044752134614e-08	57.9399317406143	0.227596706110304	6	299	activation of immune response
GO:0050778	1.01797558856702e-07	47.2022346368715	0.277074250916892	6	364	positive regulation of immune response
GO:0002252	1.44141824975688e-07	44.4	0.293820496851429	6	386	immune effector process

Tissue: Heart_Left_Ventricle=>Thyroid Type: asymmetric
SourceGene: RP11-175D17.3(ENSG00000236643.1)
TargetGeneSet: PLA2G2D CCDC163P AMPD1 RP11 FCRL5 IGKJ5 IGKC
IGKJ4 IGKJ3 IGKJ2 IGKJ1 IGKV4-1 IGKV1-5 IGKV1-12 IGKV1-16
IGKV3-20 IGKV2-24 SP140 EOMES CCR4 PARP15 IGJ RP3
NCF1C BLK MS4A1 IGHA2 IGHG2 IGHGP IGHA1 IGHG1 IGHG3 IGHD IGHM
IGHJ5 IGHV3-7 IGHV3-33 IGHV3-48 IGHV4-59 SCARNA15 CD19
P2RX5 TBC1D27 CCL4 CD79A IGLV1-51 IGLV1-47 IGLV1-44
IGLV1-40 IGLV3-25 IGLV2-23 IGLV3-21 IGLV2-14
IGLV2-11 IGLV2-8 IGLV3-1 IGLL5 IGLC1 IGLC2 IGLC3 IGLC7 WAS
GOBPID Pvalue OddsRatio ExpCount Count Size Term
GO:0006958 3.1938931138853e-20 193.137973137973 0.110718981385371
11 50 complement activation, classical pathway
GO:0006956 5.17450829290118e-19 144.722527472527 0.139505916545568
11 63 complement activation
GO:0002455 7.50120043233589e-19 139.3430335097 0.143934675800983 11
65 humoral immune response mediated by circulating immunoglobulin
GO:0072376 1.14709519640839e-17 105.853789403085 0.181579129472009
11 82 protein activation cascade
GO:0016064 3.72901390844983e-16 75.0042857142857 0.245796138675524
11 111 immunoglobulin mediated immune response
GO:0019724 5.04997115378365e-16 72.8044382801664 0.252439277558646
11 114 B cell mediated immunity
GO:0002253 6.56781311201327e-16 38.5723196881092 0.66209950868452
14 299 activation of immune response
GO:0006959 1.08954706873963e-15 67.5195195195195 0.270154314580306
11 122 humoral immune response
GO:0050778 1.00974770116133e-14 31.2644444444444 0.806034184485503
14 364 positive regulation of immune response
GO:0002449 3.68503467947307e-14 47.8916361416361 0.36980139782714
11 167 lymphocyte mediated immunity
GO:0002460 9.00267805846482e-14 43.9044817927171 0.400802712615044
11 181 adaptive immune response based on somatic recombination of immune receptors built from immunoglobulin superfamily domains
GO:0002250 2.42687592582153e-13 39.8655462184874 0.43844716628607
11 198 adaptive immune response
GO:0002443 4.8856649306588e-13 37.2402380952381 0.467234101446267
11 211 leukocyte mediated immunity
GO:0002252 1.5521457949534e-11 22.5320855614973 0.854750536295066
12 386 immune effector process
Tissue: Heart_Left_Ventricle=>Thyroid Type: cluster
SourceGene: RP11-236B18.3(ENSG00000225525.1)
TargetGeneSet: PLA2G2D FCRL5 CD84 CHIT1 IGKJ5 IGKC IGKJ3 IGKJ2
IGKJ1 IGKV4-1 IGKV1-5 IGKV1-6 IGKV3-7 IGKV1-9 IGKV3-15 IGKV1-17
IGKV3-20 IGKV2-24 IGKV2-28 IGKV1-39 IGKV2D-28
IGKV3D-20 LAP3 ICOS CCDC51 KLHL7 IGJ RASSF6 CXCL13 GZMK RP11
TSPAN33 KIAA0196 ZBTB43 TAF1D POU2AF1 GS1 FLT3 TRAV12-1
IGHG1 IGHG3 IGHM IGHV1-2 IGHV2-5 IGHV3-7 IGHV3-15 IGHV1-18
IGHV3-21 IGHV3-22 IGHV3-23 IGHV1-24 IGHV3-30
IGHV1-46 IGHV3-48 IGHV5-51 IGHV3-53 IGHV4-55
IGHV4-59 IGHV4-61 IGHV2-70 KREMEN2 CCL4L1 MFSD11 ANGPTL4
FFAR2 CD37 IGLV1-51 IGLV7-46 IGLV1-44 IGLV1-40
IGLV2-23 IGLV3-19 IGLV2-18 IGLV2-11 IGLV3-10
IGLV3-9 IGLV2-8 IGLV3-1 IGLL5 IGLC1 IGLC2 IGLC3 IGLC5 IGLC6 IGLC7
APOBEC3C
GOBPID Pvalue OddsRatio ExpCount Count Size Term
GO:0006959 4.5849197390771e-24 98.1612349914237 0.320808248564113
16 122 humoral immune response
GO:0006958 9.45629359658127e-24 202.041081081081 0.131478790395128
13 50 complement activation, classical pathway
GO:0006956 2.73173090141433e-22 149.3752 0.165663275897862 13
63 complement activation
GO:0002455 4.27126034024078e-22 143.61 0.170922427513667 13 65

humoral immune response mediated by circulating immunoglobulin
GO:0072376	1.128945768015e-20	108.099710144928	0.215625216248011
13	82	protein activation cascade
GO:0016064	7.25210199108449e-19	75.9571428571429	0.291882914677185
13	111	immunoglobulin mediated immune response
GO:0019724	1.04135872730982e-18	73.6855445544554	0.299771642100893
13	114	B cell mediated immunity
GO:0002460	1.04693841228951e-17	49.7614770459082	0.475953221230365
14	181	adaptive immune response based on somatic recombination of immune receptors built from immunoglobulin superfamily domains
GO:0002250	3.74836676948325e-17	45.1100543478261	0.520656009964708
14	198	adaptive immune response
GO:0002449	1.72341146454477e-16	48.1472727272727	0.439139159919729
13	167	lymphocyte mediated immunity
GO:0002443	3.71519761846452e-15	37.3323232323232	0.554840495467442
13	211	leukocyte mediated immunity
GO:0002253	1.20494452304226e-14	28.9169590643275	0.786243166562868
14	299	activation of immune response
GO:0050778	1.80555239275062e-13	23.4383333333333	0.957165594076534
14	364	positive regulation of immune response
GO:0002252	4.02349071322156e-13	22.0176971326165	1.01501626185039
14	386	immune effector process
Tissue: Heart_Left_Ventricle=>Thyroid	Type:	asymmetric
SourceGene:	RP11-764K9.1(ENSG00000225411.1)
TargetGeneSet:	RP4	GBP4	GBP6	AMY2A	SLAMF1	RPL31	IL1A	RPL32
RPL14	RPL24	RPL35A	TET2	AGA	BTF3	CTD	NPM1	LTB	RPL10A
MRPS12	AHR	TRGV4	TRBV19	RPS20	GRIP1	RPL7	HSD17B3	RP13	FAM13C	RP11
SLC6A13	RPL14P1	IRAK3	PGAM1P5	KDM2B	TM9SF2	TRAV3	RPL7AP6	NUMB	U6
RPS3AP6	RSL1D1	RPL17	ICAM4	RFX1	IL4I1	CXADR	RBX1	ALG12
GOBPID	Pvalue	OddsRatio	ExpCount	Count	Size	Term
GO:0006415	6.41128520091338e-13	60.5093808630394	0.220399972320255
9	91	translational termination
GO:0006414	2.40275164761738e-12	51.6346153846154	0.254307660369525
9	105	translational elongation
GO:0006614	2.40275164761738e-12	51.6346153846154	0.254307660369525
9	105	SRP-dependent cotranslational protein targeting to membrane
GO:0006613	2.62141405736306e-12	51.0987311657415	0.25672963808733
9	106	cotranslational protein targeting to membrane
GO:0045047	2.62141405736306e-12	51.0987311657415	0.25672963808733
9	106	protein targeting to ER
GO:0072599	2.62141405736306e-12	51.0987311657415	0.25672963808733
9	106	establishment of protein localization to endoplasmic reticulum
GO:0043241	2.66016827471332e-12	38.5621621621622	0.382672479413189
10	158	protein complex disassembly
GO:0070972	6.99900852258675e-12	45.4350741002117	0.28579337070099
9	118	protein localization to endoplasmic reticulum
GO:0000184	7.55932440136553e-12	45.0188811188811	0.288215348418795
9	119	nuclear-transcribed mRNA catabolic process, nonsense-mediated decay
GO:0032984	9.2783898894966e-12	33.7207100591716	0.433534011487094
10	179	macromolecular complex disassembly
GO:0019080	6.54295004255737e-11	34.7957746478873	0.365718635388554
9	151	viral genome expression
GO:0019083	6.54295004255737e-11	34.7957746478873	0.365718635388554
9	151	viral transcription
GO:0006413	6.94340123210026e-11	34.5500268961807	0.368140613106359
9	152	translational initiation
GO:0006612	7.36533111351605e-11	34.3076923076923	0.370562590824164
9	153	protein targeting to membrane
GO:0043624	7.36533111351605e-11	34.3076923076923	0.370562590824164
9	153	cellular protein complex disassembly
GO:0000956	1.79734922251437e-10	30.8423076923077	0.409314234309044
9	169	nuclear-transcribed mRNA catabolic process
GO:0034623	2.33236914653626e-10	29.8972027972028	0.421424122898069

9	174	cellular macromolecular complex disassembly
GO:0006402	2.85681397983336e-10	29.1813837050523	0.431112033769289
9	178	mRNA catabolic process
GO:0072594	9.59066382228669e-10	25.2443786982249	0.494083454432219
9	204	establishment of protein localization to organelle
GO:0022411	1.00003837357733e-09	20.3424460431655	0.697529582727839
10	288	cellular component disassembly
GO:0006401	1.00146514526641e-09	25.1138147566719	0.496505432150024
9	205	RNA catabolic process
GO:0019058	2.86870699769166e-09	22.1320166320166	0.559476852812954
9	231	viral infectious cycle
GO:0022415	8.07273389821069e-09	19.5349371743794	0.629714206629299
9	260	viral reproductive process
GO:0071845	1.73863495664393e-08	17.7998601398601	0.687841671856619
9	284	cellular component disassembly at cellular level
GO:0016032	7.23299505154884e-08	12.6757369614512	1.09231195073005
10	451	viral reproduction
GO:0033365	1.3215335997197e-06	10.4084880636605	1.14559546052176
9	473	protein localization to organelle
GO:0006605	1.3448823802972e-06	10.3853598014888	1.14801743823957
9	474	protein targeting
GO:0042273	2.82087160676787e-06	150.072916666667	0.02906373261366
3	12	ribosomal large subunit biogenesis
Tissue: Heart_Left_Ventricle=>Thyroid	Type:	asymmetric

SourceGene:	TCFL5(ENSG00000101190.8)	transcription factor-like 5 (basic helix-loop-helix)

TargetGeneSet:	RP11	ZMPSTE24	UQCRH	URB2	HEATR1	SLC52A1	ALG3
NOP14	TMEM33	TIGD4	WDR36	SRFBP1	GEMIN5	BYSL	RN5S223	SP4	ABCF2	TTI2
NUDCD1	ZC3H3	CYHR1	NCBP1	NGB	PDZD7	TARBP2	IRF9	SETD1A	ARMC5	NIP7
ZC3H18	CANT1	PTBP1	RAVER1	UPF1	ZNF793	ZNF571	ZNF576	GRWD1	PRMT1
ZNF417	TRIM28	TOMM34	URB1	GART

GOBPID	Pvalue	OddsRatio	ExpCount	Count	Size	Term

GO:0022613	4.64E-11	28.72194135	0.513805273	10	225
ribonucleoprotein complex biogenesis

GO:0071843	8.41E-11	26.93943421	0.545775379	10	239
cellular component biogenesis at cellular level

GO:0042254	9.56E-07	22.18337218	0.34025327	6	149
ribosome biogenesis

GO:0022618 2.27E-06 28.42857143 0.216940004 5 95
ribonucleoprotein complex assembly

GO:0071826 2.92E-06 26.92293233 0.228357899 5 100
ribonucleoprotein complex subunit organization

Tissue: Thyroid=>Heart_Left_Ventricle Type: asymmetric

SourceGene: USP35(ENSG00000118369.8) ubiquitin specific peptidase 35

TargetGeneSet: MXRA8 CTNNBIP1 RP11 CMPK1 PODN NEGR1 GSTM5
BNIPL KIRREL TKT RGS5 DPT LAMC1 CFH FMOD AKT3 ZNF692
GRHL1 RHOB KLHL29 C2orf56 EFEMP1 SPRED2 ANTXR1 IL1R1 CYBRD1 COL5A2
SLC23A3 SCARNA6 COL6A3 ITPR1 FBLN2 HEMK1 C3orf78 VGLL3 EPHA3 ABI3BP
CCDC80 CLSTN2 IGSF10 SPON2 EVC AFAP1 APBB2 GABRB1 ATP10D PDGFRA
11-Sep PTPN13 PKD2 U6 UNC5C GLRB SH3RF1 SPOCK1 PDGFRB C5orf62 EBF1
GNL1 TNFRSF21 RP3 PM20D2 MAN1A1 EPB41L2 HBS1L SASH1 FNDC1
COL28A1 IGFBP3 EGFR EBF2 BNIP3L INTS9 KIF13B CTD GDF6 OGN OMD
ASPN ECM2 SVEP1 SLC27A4 COL5A1 VIM KIAA1462 DNAJC12 MARVELD1
SPON1 GPR137 SLCO2B1 DPAGT1 KCNA5 EMP1 RERG EPS8 DCN ANAPC7
HMGB1 LHFP ITGBL1 ADPRHL1 MMP14 LRFN5 MAP3K9 FBLN5 FBN1 ALDH1A2
PTPN9 TPSAB1 ZNF423 FTO MMP2 CDH11 SF3B3 FTSJD1 CHD3 COX10-AS1
TLCD1 NUFIP2 CCT6B KCNJ2 SETBP1 DSEL COL5A3 CARM1 NFIX LTBP4
ZFP112 PTPRA EPB41L1 APCDD1L ICOSLG C21orf2 COL6A2 MN1 GALR3 FBLN1 CTA
OFD1 CHRDL1 GPC3 ZNF275

GOBPID Pvalue OddsRatio ExpCount Count Size Term

GO:0030198	3.46E-13	14.90706076	1.348072798	16	161
extracellular matrix organization					
GO:0043062	3.82E-13	14.80391389	1.356445921	16	162
extracellular structure organization					
GO:0031589	1.01E-06	7.402617801	1.691370839	11	202
cell-substrate adhesion					
GO:0030199	7.45E-06	22.01662562	0.276313058	5	33
collagen fibril organization					

GO:0061438 1.12E-05 121.4152542 0.050238738 3 6
renal system vasculature morphogenesis

GO:0061439 1.12E-05 121.4152542 0.050238738 3 6
kidney vasculature morphogenesis

GO:0022617 1.16E-05 19.88181313 0.301432427 5 36
extracellular matrix disassembly

Tissue: Muscle_Skeletal=>Thyroid Type: asymmetric

SourceGene: C11orf54(ENSG00000182919.9) ester hydrolase C11orf54

TargetGeneSet: RUNX3 CD2 FAM46C FCRL5 LY9 PTPN7 LAX1 MAL CD8B
IGKJ5 IGKC IGKJ4 IGKJ1 IGKV4-1 IGKV5-2 IGKV1-6 IGKV1-9 IGKV3-11
IGKV6-21 IGKV1D-13 IGKV3D-11 CD96 TIGIT RHOH IGJ RP11
GZMA CTD FAM26F TRGV5 U3 FAM27E1 FAM22A SNORD14A POLA2
C11orf54 POU2AF1 REXO2 CD3E CD3D PRKAG1 LCP1 TRAV12-2
HAUS4 PLD4 IGHG3 IGHJ6 IGHJ5 IGHV6-1 IGHV1-2 IGHV2-5 IGHV3-15
IGHV1-18 IGHV3-21 IGHV3-23 IGHV3-30 IGHV3-33
IGHV4-34 IGHV4-39 IGHV1-46 IGHV3-48 IGHV3-49
IGHV5-51 IGHV3-53 IGHV4-59 IGHV4-61 IGHV2-70
1-Sep GPR114 IKZF3 TMEM106A VMAC ZNF83 FER1L4 IGLV4-69
IGLV1-51 IGLV9-49 IGLV1-44 IGLV7-43 IGLV3-25
IGLV3-19 IGLV2-11 IGLV3-1 IGLC2 IGLC3 IGLC6 RAC2 CBX7 RP4
PIM2 IL2RG

GOBPID	Pvalue	OddsRatio	ExpCount	Count	Size	Term
GO:0006958	1.07E-14	98.56783537	0.141858695	9	50	"complement activation, classical pathway"
GO:0006956	9.85E-14	74.77083333	0.178741956	9	63	complement activation
GO:0002455	1.33E-13	72.09040179	0.184416303	9	65	humoral immune response mediated by circulating immunoglobulin

GO:0016064	4.35E-13	45.70105398	0.314926303	10	111	
immunoglobulin mediated immune response						
GO:0019724	5.70E-13	44.37344913	0.323437824	10	114	B
cell mediated immunity						
GO:0002449	8.25E-13	33.50299145	0.473808041	11	167	
lymphocyte mediated immunity						
GO:0006959	1.14E-12	41.18087558	0.346135216	10	122	
humoral immune response						
GO:0072376	1.17E-12	55.23672945	0.23264826	9	82	
protein activation cascade						
GO:0002460	2.00E-12	30.71372549	0.513528476	11	181	
adaptive immune response based on somatic recombination of immune receptors built from immunoglobulin superfamily domains						

GO:0002250	5.34E-12	27.88823529	0.561760432	11	198	
adaptive immune response						
GO:0002443	1.07E-11	26.05166667	0.598643692	11	211	
leukocyte mediated immunity						
GO:0002252	2.58E-11	17.47232861	1.095149125	13	386	
immune effector process						
GO:0002253	4.51E-10	17.97939815	0.848314996	11	299	
activation of immune response						
GO:0050778	3.58E-09	14.60122757	1.032731299	11	364	
positive regulation of immune response						
GO:0042110	2.09E-07	12.79233871	0.905058473	9	319	T
cell activation						

GO:0046649	2.56E-07	10.66651415	1.228496298	10	433
lymphocyte activation

Tissue: Muscle_Skeletal=>Thyroid	Type:	cluster
SourceGene:	C11orf54(ENSG00000182919.9)
TargetGeneSet:	RUNX3	CD2	FAM46C	FCRL5	LY9	PTPN7	LAX1	MAL	CD8B
IGKJ5	IGKC	IGKJ4	IGKJ1	IGKV4-1	IGKV5-2	IGKV1-6	IGKV1-9	IGKV3-11
IGKV6-21	IGKV1D-13	IGKV3D-11	CD96	TIGIT	RHOH	IGJ	RP11
GZMA	CTD	FAM26F	TRGV5	U3	FAM27E1	FAM22A	SNORD14A	POLA2
C11orf54	POU2AF1	REXO2	CD3E	CD3D	PRKAG1	LCP1	TRAV12-2
HAUS4	PLD4	IGHG3	IGHJ6	IGHJ5	IGHV6-1	IGHV1-2	IGHV2-5	IGHV3-15
IGHV1-18	IGHV3-21	IGHV3-23	IGHV3-30	IGHV3-33
IGHV4-34	IGHV4-39	IGHV1-46	IGHV3-48	IGHV3-49
IGHV5-51	IGHV3-53	IGHV4-59	IGHV4-61	IGHV2-70
SEPT1	GPR114	IKZF3	TMEM106A	VMAC	ZNF83	FER1L4	IGLV4-69
IGLV1-51	IGLV9-49	IGLV1-44	IGLV7-43	IGLV3-25
IGLV3-19	IGLV2-11	IGLV3-1	IGLC2	IGLC3	IGLC6	RAC2	CBX7	RP4
PIM2	IL2RG

GOBPID	Pvalue	OddsRatio	ExpCount	Count	Size	Term
GO:0006958	1.07045074677632e-14	98.5678353658537	0.141858694900007	9	50	complement activation, classical pathway
GO:0006956	9.852512107991e-14	74.7708333333333	0.178741955574009	9	63	complement activation
GO:0002455	1.32541446159318e-13	72.0904017857143	0.184416303370009	9	65	humoral immune response mediated by circulating immunoglobulin
GO:0016064	4.34557281079881e-13	45.7010539763654	0.314926302678015	10	111	immunoglobulin mediated immune response
GO:0019724	5.70453571225452e-13	44.3734491315136	0.323437824372016	10	114	B cell mediated immunity
GO:0002449	8.25067236558966e-13	33.5029914529915	0.473808040966023	11	167	lymphocyte mediated immunity
GO:0006959	1.13719065670266e-12	41.1808755760369	0.346135215556017	10	122	humoral immune response
GO:0072376	1.17441085855504e-12	55.2367294520548	0.232648259636011	9	82	protein activation cascade
GO:0002460	1.99948012484219e-12	30.7137254901961	0.513528475538025	11	181	adaptive immune response based on somatic recombination of immune receptors built from immunoglobulin superfamily domains
GO:0002250	5.33749431796508e-12	27.8882352941176	0.561760431804027	11	198	adaptive immune response
GO:0002443	1.06649145406103e-11	26.0516666666667	0.598643692478029	11	211	leukocyte mediated immunity
GO:0002252	2.57978683847137e-11	17.4723286097281	1.09514912462805	13	386	immune effector process
GO:0002253	4.50582818573953e-10	17.9793981481481	0.848314995502041	11	299	activation of immune response
GO:0050778	3.57602950676341e-09	14.6012275731822	1.03273129887205	11	364	positive regulation of immune response

GO:0042110	2.09246316077927e-07	12.7923387096774	0.905058473462044
9	319	T cell activation
GO:0046649	2.56462609546679e-07	10.6665141462671	1.22849629783406
10	433	lymphocyte activation
Tissue: Muscle_Skeletal=>Thyroid	Type:	cluster
SourceGene:	ENSG00000182648.7
TargetGeneSet:	RP4	PLOD1	GBP5	CD2	RAB13	CD84	SLAMF1	CD48
SLAMF7	LY9	PTPN7	LAX1	RASSF3	FAIM3	TRAF3IP3	CHRM3-AS2	RP11
RBKS	MAL	IGKJ5	IGKC	IGKJ4	IGKJ2	IGKJ1	IGKV4-1 IGKV1-5 IGKV1-9
IGKV3-11	IGKV1-16	IGKV1-17	IGKV3-20	IGKV1-27
IGKV2-30	IGKV1-33	IGKV1-39	IGKV1D-27	PAX8	ARL6IP6
ITGA4	RUFY4	SP140	PDCD1	ATG7	PPARG	DNAH1	FAM86DP CD96	SIDT1
ACTG1P1 HLTF-AS1	MCF2L2	RHOH	IGJ	CXCL10	EXOSC9	GZMK	GZMA
ATG12	OR2I1P	RP1	TAGAP	TRGV9	NCF1	STAG3L1 HNRNPA1P8	TRBV29-1
CSPP1	FAM92A1 ZHX1	PAICSP1 CDNF	P4HA1	ANXA7	FAM22A	CWF19L1 MS4A1	CD5
NUMA1	CD3E	CD3D	CD3G	LAG3	OLR1	REP15	H1FNT	KRR1P1	TRAC
BCL2L2	HNRPCP	DDX24	EXOC3L4 PLD4	IGHG2	IGHG1	IGHG3	IGHJ6	IGHJ5
IGHV1-2 IGHV3-7 IGHV3-9 IGHV3-11	IGHV3-15	IGHV1-18	IGHV3-23
IGHV4-31	IGHV3-33	IGHV4-34	IGHV4-39	IGHV3-49
IGHV5-51	IGHV4-59	IGHV3-66	IGHV1-69	IL16	TCEB2
TNFRSF17	SEPT1	ITGAL	NLRC5	KIF1C	CCL5	ACACA	RAB37	CD7
THOC1	GZMM	CCDC94	CYP4F8	MAP4K1	MED29	SELV	CD79A	ESF1	FER1L4	MX1
UBASH3A PDXK	C21orf67	IGLV10-54	IGLV1-47	IGLV1-40
IGLV2-23	IGLV3-21	IGLV3-19	IGLV2-14	IGLV2-8 IGLV3-1
IGLC2	SSTR3	DDX17	SPIN2B	IL2RG	BEX2	MTCP1	SASH3
GOBPID	Pvalue	OddsRatio	ExpCount	Count	Size	Term
GO:0002449	1.66142026815146e-13	17.9731781376518	1.07473531243513
15	167	lymphocyte mediated immunity
GO:0002443	3.41187240157216e-13	15.0921078921079	1.35789910732821
16	211	leukocyte mediated immunity
GO:0046649	3.12441095274885e-12	9.25072141696242	2.7865891633797 20
433	lymphocyte activation
GO:0002252	3.88485481421508e-12	9.78823919287134	2.48411874610754
19	386	immune effector process
GO:0050778	1.42827202853624e-11	9.7193063583815 2.34253684866099	18
364	positive regulation of immune response
GO:0006958	2.57203013223223e-11	37.4137630662021	0.321777039651235
9	50	complement activation, classical pathway
GO:0006956	2.26936872040733e-10	28.3809523809524	0.405439069960556
9	63	complement activation
GO:0002455	3.03310069282267e-10	27.3635204081633	0.418310151546606
9	65	humoral immune response mediated by circulating immunoglobulin
GO:0006959	3.34336945914543e-10	17.2178642056691	0.785135976749014
11	122	humoral immune response
GO:0042110	1.7145708730557e-09	8.89043522267206	2.05293751297488
15	319	T cell activation
GO:0002460	1.78298176662382e-09	12.4383081306158	1.16483288353747
12	181	adaptive immune response based on somatic recombination of immune receptors built from immunoglobulin superfamily domains
GO:0016064	2.24733764799014e-09	17.0070380532029	0.714345028025742
10	111	immunoglobulin mediated immune response
GO:0072376	2.54310053819614e-09	20.9662426614481	0.527714345028026
9	82	protein activation cascade
GO:0019724	2.92119921605593e-09	16.5129749768304	0.733651650404816
10	114	B cell mediated immunity
GO:0002250	4.9501471041309e-09	11.2879330943847	1.27423707701889
12	198	adaptive immune response
GO:0002253	6.65456799691893e-09	8.75069953364424	1.92422669711439
14	299	activation of immune response
GO:0050863	1.6946632877093e-07	9.03763066202091	1.42225451525846
11	221	regulation of T cell activation
GO:0051249	2.5589078126252e-07	7.70097034303676	1.82125804442599
12	283	regulation of lymphocyte activation

GO:0002694 9.78514014293024e-07 6.73570658036677 2.06580859456093
12 321 regulation of leukocyte activation
GO:0050865 1.95808857459254e-06 6.27816940807877 2.20739049200747
12 343 regulation of cell activation
GO:0050870 1.41352685719421e-05 8.19631901840491 1.10047747560722
8 171 positive regulation of T cell activation
GO:0002696 1.87270457946094e-05 6.76052295918367 1.49948100477476
9 233 positive regulation of leukocyte activation
Tissue: Muscle_Skeletal=>Thyroid Type: asymmetric
SourceGene: ENSG00000244256.1
TargetGeneSet: RP11 C1orf159 RP4 CEP104 LZIC DFFA PLOD1
DNAJC16 HP1BP3 LDLRAP1 EYA3 PHACTR4 RCC1 PEF1 TXLNA TMEM234 NCDN
FOXO6 PPCS CCDC24 DNAJC6 RBMXL1 CYB561D1 GOLPH3L SNX27 INTS3
SLC39A1 TDRD10 MSTO1 UBQLN4 CCT3 POGK MYBPH ZNF238 ID2 C2orf48
PDIA6 SLC5A6 PPM1G ARHGEF33 CCT4 PCBP1-AS1 SPR MOB1A DQX1
AUP1 SEMA4F C2orf68 MRPS5 LMAN2L NOSTRIN KRT18P19 HSPD1 ALS2CR12
FZD5 ASB1 HDLBP DTYMK TTLL3 CRELD1 SLC6A1-AS1 NME6 WDR6
FAM116A SLC25A36 CPA3 SLC33A1 SSR3 LRCH3 TMEM128 UGDH-AS1
DCAF4L1 ANKRD17 CDKL2 CTSO DNAJC21 FBXO4 KIF2A PAPD4 PCDHGA1 TCOF1
TMED9 SSR1 HCG17 DAXX SYNGAP1 NUDT3 FANCE BRPF3 NFYA RP1
AARS2 BAG2 SYNCRIP PEX7 KIAA0895 URGCP HSPB1 CYP3A43 TNPO3
CNOT4 HIPK2 XKR6 NEIL2 POLR3D CHMP7 CTD C8orf40 STMN2 GAPDHP62
GRHL2 LRRC6 ZNF623 PLAA VCP ATP8B5P MCART1 FAM22G NANS ALG2
PRPS1P2 CCBL1 TOR1B PRRC2B SURF4 MAMDC4 C9orf169 TUBB4B NOXA1
KRT8P37 CDNF SUV39H2 C10orf114 ZDHHC16 CYP2E1 RP13 UBQLNL SLC35C1
CELF1 PATL1 GANAB MARK2 STIP1 FKBP2 GPR137 SYVN1 RBM14 7SK
C2CD3 CHORDC1 ATM RNF214 DPAGT1 RNF26 HSPA8 STT3A RAD52 CBX3P4
FKBP4 TEAD4 NCAPD2 PEX5 RPL13AP20 KIAA1467 REP15 PUS7L
H1FNT TUBA1C RAB5B RNF41 OS9 TSPAN31 TBC1D30 TSPAN8 ALDH2 C12orf65
GOLGA3 ITM2B DNAJC3-AS1 DNAJC3 PCID2 OR11H7 MRPL52 AJUBA ACIN1
NEDD8 FANCM GNPNAT1 HNRPCP MAP3K9 AHSA1 ZC3H14 HSP90AA1 GOLGA8B
ZFYVE19 PPP1R14D ZFP106 TP53BP1 PPIP5K1 PPIB MAN2C1 DNAJA4 SELS HN1L
SULT1A3 TBC1D10B FBXL19-AS1 SALL1 CMTM4 C16orf46 ZC3H18
RNF167 KCTD11 FLOT2 CCT6B WIPF2 UBE2Z ZNF652 SRP68 QRICH2 CANT1
ENGASE CSNK1D TMEM241 ZNF397 MBD1 AP3D1 ZNF555 ELAVL1 ZGLP1 ZNF441 JAK3
PGPEP1 MIA SMG9 ZNF284 BLOC1S3 KPTN PRMT1 ZNF347 ZNF761 ZNF329 MCM8
HM13 TOMM34 STK4 ADNP C20orf11 PDXK UBE2G2 KRT18P5 UBE2L3
SDF2L1 ZNF70 MORC2 PRR14L CTA RANGAP1 RP6 ZBED4 ALG12 CRELD2
CXorf58 FAM123B KIAA2022 MAGT1 TIMM8A MID2 LONRF3 ZNF449 FHL1
GOBPID Pvalue OddsRatio ExpCount Count Size Term
GO:0006457 4.70322619491241e-14 9.20170244263509 3.06414780984015
23 216 protein folding
GO:0006986 2.21067554751856e-06 6.78874716058012 1.82997716421009
11 129 response to unfolded protein
GO:0035966 3.71122598459935e-06 6.40540206185567 1.92927825064009
11 136 response to topologically incorrect protein
Tissue: Muscle_Skeletal=>Thyroid Type: cluster
SourceGene: ENSG00000244256.1
TargetGeneSet: RP11 C1orf159 RP4 CEP104 LZIC DFFA PLOD1
DNAJC16 HP1BP3 LDLRAP1 EYA3 PHACTR4 RCC1 PEF1 TXLNA TMEM234 NCDN
FOXO6 PPCS CCDC24 DNAJC6 RBMXL1 CYB561D1 GOLPH3L SNX27 INTS3
SLC39A1 TDRD10 MSTO1 UBQLN4 CCT3 POGK MYBPH ZNF238 ID2 C2orf48
PDIA6 SLC5A6 PPM1G ARHGEF33 CCT4 PCBP1-AS1 SPR MOB1A DQX1
AUP1 SEMA4F C2orf68 MRPS5 LMAN2L NOSTRIN KRT18P19 HSPD1 ALS2CR12
FZD5 ASB1 HDLBP DTYMK TTLL3 CRELD1 SLC6A1-AS1 NME6 WDR6
FAM116A SLC25A36 CPA3 SLC33A1 SSR3 LRCH3 TMEM128 UGDH-AS1
DCAF4L1 ANKRD17 CDKL2 CTSO DNAJC21 FBXO4 KIF2A PAPD4 PCDHGA1 TCOF1
TMED9 SSR1 HCG17 DAXX SYNGAP1 NUDT3 FANCE BRPF3 NFYA RP1
AARS2 BAG2 SYNCRIP PEX7 KIAA0895 URGCP HSPB1 CYP3A43 TNPO3
CNOT4 HIPK2 XKR6 NEIL2 POLR3D CHMP7 CTD C8orf40 STMN2 GAPDHP62
GRHL2 LRRC6 ZNF623 PLAA VCP ATP8B5P MCART1 FAM22G NANS ALG2
PRPS1P2 CCBL1 TOR1B PRRC2B SURF4 MAMDC4 C9orf169 TUBB4B NOXA1
KRT8P37 CDNF SUV39H2 C10orf114 ZDHHC16 CYP2E1 RP13 UBQLNL SLC35C1

CELF1 PATL1 GANAB MARK2 STIP1 FKBP2 GPR137 SYVN1 RBM14 7SK
C2CD3 CHORDC1 ATM RNF214 DPAGT1 RNF26 HSPA8 STT3A RAD52 CBX3P4
FKBP4 TEAD4 NCAPD2 PEX5 RPL13AP20 KIAA1467 REP15 PUS7L
H1FNT TUBA1C RAB5B RNF41 OS9 TSPAN31 TBC1D30 TSPAN8 ALDH2 C12orf65
GOLGA3 ITM2B DNAJC3-AS1 DNAJC3 PCID2 OR11H7 MRPL52 AJUBA ACIN1
NEDD8 FANCM GNPNAT1 HNRPCP MAP3K9 AHSA1 ZC3H14 HSP90AA1 GOLGA8B
ZFYVE19 PPP1R14D ZFP106 TP53BP1 PPIP5K1 PPIB MAN2C1 DNAJA4 SELS HN1L
SULT1A3 TBC1D10B FBXL19-AS1 SALL1 CMTM4 C16orf46 ZC3H18
RNF167 KCTD11 FLOT2 CCT6B WIPF2 UBE2Z ZNF652 SRP68 QRICH2 CANT1
ENGASE CSNK1D TMEM241 ZNF397 MBD1 AP3D1 ZNF555 ELAVL1 ZGLP1 ZNF441 JAK3
PGPEP1 MIA SMG9 ZNF284 BLOC1S3 KPTN PRMT1 ZNF347 ZNF761 ZNF329 MCM8
HM13 TOMM34 STK4 ADNP C20orf11 PDXK UBE2G2 KRT18P5 UBE2L3
SDF2L1 ZNF70 MORC2 PRR14L CTA RANGAP1 RP6 ZBED4 ALG12 CRELD2
CXorf58 FAM123B KIAA2022 MAGT1 TIMM8A MID2 LONRF3 ZNF449 FHL1
GOBPID Pvalue OddsRatio ExpCount Count Size Term
GO:0006457 4.70322619491241e-14 9.20170244263509 3.06414780984015
23 216 protein folding
GO:0006986 2.21067554751856e-06 6.78874716058012 1.82997716421009
11 129 response to unfolded protein
GO:0035966 3.71122598459935e-06 6.40540206185567 1.92927825064009
11 136 response to topologically incorrect protein
Tissue: Muscle_Skeletal=>Thyroid Type: asymmetric
SourceGene: GAPVD1(ENSG00000165219.14)
TargetGeneSet: MTND1P23 NUDC WASF2 EYA3 MED18 PHACTR4 GNG5 ETV3
TROVE2 ZNF281 RP11 ADAM18 C2orf18 MOB1A PRKAR2A IP6K1 KIAA2018
RNF168 ZNF141 RFC1 SETD7 MTMR12 PHAX CTB MTND5P11 RBM22
PPP1R18 BTBD9 MTRNR2L9 ARID1B FOXK1 PURB MEPCE CBLL1 GCC1
NEIL2 HR CTD KAT6A C9orf102 NIPSNAP3A ZBTB43 GTF3C4
NCLP1 NRBF2 POLR3A TSSC4 MTRNR2L8 SPTY2D1 ATG13 HEXIM1 OSBP
MARK2 RSF1 DDX6 RNF26 ERC1 ZBTB39 GCN1L1 SBNO1 RNF6 N4BP2L2-IT1
RBM23 SOCS6 DLST GOLGA8IP TJP1 EMP2 FTO-IT1 NFATC3 VPS4A
ATXN1L METTL16 NCOR1 SMCR8 MED1 WIPF2 PSME3 ZNF652 RPS2P48 MAP3K3
CANT1 MYL12B NFIC FEM1A ZNF557 ZNF121 ZNF562 ZNF791 DNAJB1 ZNF429
ZFP82 ARHGAP35 KCNJ14 ZNF480 ZNF264 ZNF543 ZNF749 ZNF329 FASTKD5
CRNKL1 PLAGL2 PHF20 ZSWIM1 ZNFX1 TSHZ2 C20orf11 PRR14L SMCR7L
RRP7A PPARA CERK USP9X GNL3L
GOBPID Pvalue OddsRatio ExpCount Count Size Term
Tissue: Muscle_Skeletal=>Thyroid Type: cluster
SourceGene: KLF4(ENSG00000136826.9)
TargetGeneSet: RP4 CTNNBIP1 PLOD1 HP1BP3 TCEB3 LYPLA2 EYA3
TXLNA MRPS15 ZMPSTE24 CTPS LEPRE1 CCDC24 EIF2B3 CYP4A11 OMA1
SRSF11 BCL10 FNBP1L RP11 CYB561D1 AMPD2 MAN1A2 SEC22B NOTCH2NL
GOLPH3L CERS2 INTS3 DENND4B CREB3L4 FLAD1 SLC50A1 MSTO1 UBQLN4 CCT3
C1orf182 USP21 HSD17B7 POGK RNASEL ZC3H11A TMEM81 CDK18 ELK4
C1orf96 SIPA1L2 OR11L1 NOL10 PDIA6 TTC32 RAB10 SLC5A6 XPO1 CCT4
PPP3R1 PCBP1-AS1 PCYOX1 PCGF1 AUP1 MRPS5 RALB HNRNPA3 HSPD1
C2orf69 WDR12 PARD3B METTL21A RPE RQCD1 TTLL4 ABCB6 ATG4B
CRELD1 CHCHD4 SEMA3F SLC38A3 IFRD2 HEMK1 RAD54L2 DUSP7 POC1A PHF7 ARF4
TFG TMEM39A COPG1 NUDT16 UBA5 ACTG1P1 SLC25A36 SLC33A1 SSR3
MFSD1 MCCC1 ALG3 TNIP2 GRPEL1 UGDH-AS1 DCAF4L1 TEC SEC31A
THAP9 TMSB4XP8 EIF4E C4orf32 SEC24D PPP1R14BP3 SLC10A7 STOX2 SDHA
MTRR CTD MARCH6 FAM105A IPO11 PIK3R1 ANKRA2 ZCCHC9 TMEM167A
EPB41L4A-AS1 XBP1P1 CSNK1G3 CHSY3 SMAD5 CTB ARAP3 PCDH12 ADRB2
FAM114A2 MFAP3 TMED9 B4GALT7 ZFP62 TRIM52 SSR1 C6orf228
ZSCAN12P1 TRIM27 TUBB C6orf25 VARS NUDT3 PPIL1 BYSL GPR110
BEND6 LINC00472 PGM3 LATS1 MRPL18 MICALL2 TSPAN13 C7orf46 STK31 CYCS
RALA PURB SNORA5A IGFBP3 LANCL2 PSPH C7orf53 AASS CEP41 LUC7L2
OR2A7 ABCB8 SMARCD3 PINX1 NEIL2 FAM160B2 POLR3D R3HCC1 PPP2R2A
GTF2E2 PPAPDC1B AP3M2 C8orf46 RNF19A GAPDHP62 GRHL2 BAALC
NUDCD1 ATAD2 LRRC6 BOP1 KLHL9 DNAJA1 VCP MCART1 C9orf41 RFK OMD
C9orf156 NANS ALG2 FSD1L HDHD3 RBM18 ZBTB26 PRPS1P2 CCBL1
METTL11A SURF4 INPP5E SLC6A2 FBXO18 CELF2 SUV39H2 PBLD AP3M1
CYP2C9 ZDHHC16 OCRL TACC2 TRIM68 UBQLNL MRPL17 NLRP14 COPB1 CAPRIN1

SLC35C1 B3GAT3 C11orf84 STIP1 FKBP2 GPR137 PPP2R5B SYVN1 RBM14
PITPNM1 NUMA1 CLPB CHCHD8 KCNE3 CHORDC1 PAFAH1B2 HMBS DPAGT1
TBCEL HSPA8 MSANTD2 STT3A HYLS1 RAD52 FKBP4 TEAD4 HIN1L NOP2
CLEC1A KIAA1467 REP15 PPHLN1 PUS7L H1FNT TUBA1C FMNL3 TARBP2 NPFF
RAB5B CNPY2 PAN2 METTL1 METTL21B TBC1D30 TSPAN8 DCN NR2C1
IKBIP MTERFD3 C12orf34 ANAPC7 ATXN2 ALDH2 DYNLL1 PUS1 GOLGA3 U6
GTF3A FANCB RFC3 KBTBD6 DHRS12 SUGT1 DNAJC3-AS1 DNAJC3 RAP2A
SLC15A1 TMTC4 CARKD PROZ PCID2 FAM70B DAD1 MRPL52 PRMT5-AS1
PRMT5 PSME2 NEDD8 EAPP GNPNAT1 CDKN3 SOCS6 AHSA1 C14orf142
HSP90AA1 ZFYVE19 TP53BP1 FBN1 GABPB1 NEDD4 NARG2 RORA PARP16
SKOR1 IL16 PRC1 SELS TMEM8A SOLH HN1L IGFALS TRAF7 ATF7IP2
USP31 UBFD1 TBX6 PHKG2 ZNF267 ABCC11 SALL1 BBS2 GPR56 C16orf57
E2F4 NUTF2 FTLP14 CYB5B CHST4 BCAR1 C16orf46 AFG3L1P GAS8
CYB5D2 RPAIN COX10-AS1 SLC47A2 MAP2K3 KSR1 PIPOX UTP6 SLFN11
MMP28 CCL16 LEPREL4 LSM12 ATXN7L3 SLC25A39 PNPO CDK5RAP3 DDX5
HN1 QRICH2 AFMID CBX8 SIRT7 PYCR1 CSNK1D C17orf62 NARF FN3K
TBCD TMEM241 RPRD1A MBD1 ZADH2 ZNF555 TLE2 NCLN ELAVL1 ZNF562 PPAN
ZGLP1 ELOF1 ZNF440 DNASE2 TRMT1 JAK3 MPV17L2 LSM4 YJEFN3 KCTD15
FXYD1 ZNF146 ZNF260 MIA SMG9 BLOC1S3 VASP MYPOP NOVA2 PTGIR
DHX34 PRMT1 PTOV1 CTU1 ZNF761 ISOC2 ZNF550 ZNF814 FKBP1A FOXA2 GZF1
ENTPD6 NANP HM13 ZNF341 EDEM2 RPN2 EMILIN3 YWHAB TOMM34 KRT18P4
DPH3P1 TMEM50B UBE2L3 SDF2L1 ZNF70 UPB1 GAL3ST1 SGSM3 RP6 ALG12
CRELD2 TRABD TUBGCP6 HDHD1 RAI2 MAOB ZNF673 ZNF81 ZNF630 WDR13
RRAGB MAGT1 PGK1 ARMCX4 ARMCX2 NKRF PHF6 MOSPD1 FAM50A
GOBPID Pvalue OddsRatio ExpCount Count Size Term
GO:0006487 4.10627332612179e-09 8.30734614929144 2.19002145180264
15 92 protein N-linked glycosylation
GO:0018279 1.31551960315768e-07 7.44790560953703 2.07099854681337
13 87 protein N-linked glycosylation via asparagine
GO:0018196 1.51082844816065e-07 7.34807653575025 2.09480312781122
13 88 peptidyl-asparagine modification
GO:0006457 2.30035398870351e-07 4.38114134542706 5.14178949553664
20 216 protein folding
GO:0006986 2.37363259406325e-06 5.16173913043478 3.07079094872327
14 129 response to unfolded protein
GO:0035966 4.4510566391263e-06 4.86313959264779 3.23742301570826
14 136 response to topologically incorrect protein
GO:0070085 1.16383774428643e-05 3.43610169491525 6.07016815445298
19 255 glycosylation
Tissue: Muscle_Skeletal=>Thyroid Type: asymmetric
SourceGene: LMOD3(ENSG00000163380.11)
TargetGeneSet: MEGF6 ANGPTL7 FAM46B COL16A1 CYP4B1 NEXN RP11 OLFML3 NGF
FCGR1A S100A10 HAPLN2 ATP1A2 ANGPTL1 GLT25D2 DISC1 RP4 LYST NID1 RHOB
ITSN2 VIT EHBP1 FIGN PDE11A SESTD1 ZNF385B PID1 EFHD1 CXCR7
PGAM1P4 CIDEC PPARG VIPR1 CLEC3B TNNC1 ALDH1L1 MBNL1 MME MME-AS1
ADIPOQ ADIPOQ-AS1 KLB CORIN SEPT11 USP53 SH3D19 NPY1R PDLIM3
SLC12A7 PIK3R1 GPR98 LVRN LOX SNCAIP SPOCK1 ADRB2 RP1 MYO1G EGFR
CTB AZGP1 NYAP1 CAV1 WNT2 LEP LCP1 OSR2 MAMDC2 OMD
HDHD3 GSN ARHGAP21 SFRP5 ADD3 EBF3 INPP5A TNNT3 H19
PDE3B CAPRIN1 CD44 IL18BP POU2F3 IGFBP6 TSPAN8 DCN MLXIP RXFP2
DCLK1 CAB39L RN7SKP7 PRKD1 ZFYVE21 TPM1 PML MFGE8 ANPEP NDRG4
MMP28 PNMT CD300LG C17orf62 CABLES1 CYB5A COL5A3 CACNA1A JAK3
FOXA2 CST3 EXOSC3P1 MTND1P10 RP3 PMM1 WNT7B MIR221
FAAH2 ITM2A TBX22 DRP2 ZBTB33 AFF2 MAMLD1 PDZD4
GOBPID Pvalue OddsRatio ExpCount Count Size Term
GO:0044057 1.48109479301536e-06 5.44316502634734 2.97218185592693
14 417 regulation of system process
GO:0008217 3.37093466593401e-06 10.0691729323308 0.905196872188776
8 127 regulation of blood pressure
Tissue: Muscle_Skeletal=>Thyroid Type: cluster
SourceGene: MTND1P23(ENSG00000225972.1)
TargetGeneSet: WASH7P MTND1P23 MTND2P28 AURKAIP1 RP13 LZIC
DFFA SNORA59A DNAJC16 RP11 PAQR7 RP1 WASF2 EYA3 PHACTR4

SNRNP40 MFI2 NSUN4 SELRC1 NFIA NSRP1P1 RP4 U6 RBMXL1 TAF13
MRPL53P1 BCAS2 BCL9 LCE2B GATAD2B JTB MIR555 ETV3 KIRREL-IT1
HMGN1P4 TROVE2 KRT8P29 FAM108A4P ZC3H11B FTH1P2 HMGN2P19 AKT3-IT1
C2orf18 PPP1CB WDR43 AAK1 C2orf68 EIF5B MZT2B H3F3AP4 KRT18P29
CYP20A1 RQCD1 CLN5 CAPN10 ARPC4 RPL29 PSMC1P1 KIAA2018 ZBTB20
POU5F1P6 ATP5G1P3 GMPS RNF168 SENP5 PSAPL1 HSP90AB2P
RELL1 RAC1P2 ANKRD17 EIF4E CTD NUP155 EMB SREK1IP1 CENPK
ANKRA2 PHAX C5orf15 CTB MTND5P11 KDM3B ZMAT2 RBM27 RANP1
C6orf47 AGPAT1 RXRB TAF8 RPL7L1 CDC5L RP3 MTRNR2L9 FKBP1C
PHACTR2 ARID1B WTAP BRAT1 FOXK1 AP5Z1 POLR2J4 RPL35P5 RPS3AP26
ZKSCAN5 RPL19P12 CBLL1 RN5S245 ZNF212 LSM12P1 FAM150A JPH1 KIAA1429
ST13P6 PTK2 LRRC19 FAM22G ZNF462 SKA2L DPM2 PRRC2B FRMD4A DCLRE1C
YWHAZP3 PRINS POLR3A RPS3AP5 NHP2P1 EIF2S2P3 FBXL15 PRDX3 OR52E8
TMEM9B MTRNR2L8 MRVI1-AS1 ATG13 ARHGAP1 C11orf31 HNRNPUL2
MARK2 BAD SF1 RPL37P2 ACER3 RSF1 FAM76B CWF19L2 SDHD PAFAH1B2
DDX6 ERC1 KIAA0528 ARID2 SMARCD1 CERS5 SLC11A2 CBX5 MRS2P2
LRRIQ1 BRAP PARP4-AS1 RBM22P2 MFAP1P1 N4BP2L2-IT1 TDRD3 ARGLU1
RNASE4 ACIN1 PRPF39 GNPNAT1 DLST ZFYVE21 RPS26P49 GOLGA8IP TJP1
HMGN2P5 MGA VPS39 HMGN2P46 FAM96A MTFMT IDH3A IQGAP1 CRTC3
CHTF18 EMP2 PKD1P1 DCTN5 C16orf53 SULT1A3 CD2BP2 UBE2MP1 DNAJA2
FTO-IT1 NOL3 NFATC3 CIRH1A VPS4A YWHAE METTL16 RNASEK VAMP2 NCOR1
TP53I13 TAF15 C17orf96 FBXL20 MED1 WIPF2 IGFBP4 FAM134C PSME3
KANSL1 KPNB1 ZNF652 KAT7 RPS2P48 SCARNA20 MED13 GH1 SLC38A10
ANAPC11 MYL12B MPPE1 SERPINB7 ZNF407 C19orf24 NFIC CRB3
ZNF562 FBXL12 ZNF791 BRD4 ZNF429 ZNF568 NOVA2 ZNF480 ZNF813 ZNF543
ZNF544 ZSCAN22 CRNKL1 SCAND1 TSHZ2 RPL12P4 RAB22A NDUFV3 FAM108A5P CTA
TTC28 SF3A1 MYH9 TOMM22 RPS19BP1 TRABD ARSA PRDX4 USP9X RP6
OTUD5 GNL3L TLE1P1 RPSAP14 HK2P1 MTND4P24 MMGT1
GOBPID Pvalue OddsRatio ExpCount Count Size Term
GO:0006397 5.91291960082991e-07 4.53119739342927 4.50051899522524
18 399 mRNA processing
GO:0008380 3.81086233695306e-06 4.60029835029835 3.64327728184901
15 323 RNA splicing
Tissue: Muscle_Skeletal=>Thyroid Type: cluster
SourceGene: NNAT(ENSG00000053438.7)
TargetGeneSet: UBE2J2 RP11 DFFA DNAJC16 RSG1 RCC2 LDLRAP1 YTHDF2
SNRNP40 TXLNA NCDN AKIRIN1 KIF2C LDLRAD1 PGM1 CCBL2 KCND3 SEC22B
GOLPH3L PYGO2 MIR555 UBQLN4 CCT3 CDC73 KRT8P29 ACBD3 ZNF678 URB2
ZNF238 DNMT3A DPYSL5 SLC5A6 PPM1G ARHGEF33 MSH6 FBXO11 REL
PCBP1-AS1 PCBP1 MOB1A SH2D6 MRPS5 TBC1D8 MAP4K4 MFSD9 TMEM182
POLR1B MYO7B SMPD4 GORASP2 HSPD1 C2orf69 CREB1 METTL21A FZD5
TTLL4 NHEJ1 HMGB1P3 PRRT3-AS1 LARS2 TMEM89 WDR6 ZBTB11 ZBTB20
TMEM39A COPG1 H1FX-AS1 MED12L SLC33A1 GMPS MCCC1 ZNF141 TMEM128
TADA2B EXOC7 ANKRD17 EIF4E COL25A1 SEC24D IRF2 SLC12A7 FAM105B SNX18 CCNO
KIF2A PAPD4 EPB41L4A-AS1 DTWD2 SRFBP1 SEC24A CTB ADRB2 CAMK2A
FAM114A2 MFAP3 FBXW11 TMED9 ZSCAN12P1 TRIM27 HCG18 SNORD48
SRPK1 BRPF3 BYSL PGM3 UBE2J1 TSPYL4 LATS1 RSPH3 FAM120B HUS1
BAZ1B ZKSCAN1 SH2B2 METTL2A MTPN EPHA1 ZNF786 NEIL2 LCP1 POLR3D CTD
CHMP7 EXTL3 LSM12P1 WHSC1L1 KAT6A LRRC6 EIF2C2 EEF1D NRBP2 BOP1
DNAJA1 ATP8B5P DCAF10 FAM22G NANS ALG2 MRPL50 TTLL11 NEK6 OLFML2A
METTL11A SURF4 TUBB4B OPTN AP3M1 ALDH18A1 CC2D2B RRP12
ENTPD7 ZDHHC6 NHLRC2 MRPL17 SPTY2D1 LIN7C CAPRIN1 TRIM44 ZBTB3 C11orf84
MARK2 STIP1 GPR137 GPHA2 SYVN1 RBM14 CLPB PRSS23 CHORDC1 DDX6
HSPA8 FKBP4 RP4 GNB3 ASUN PUS7L DDX23 TIMELESS TMEM194A
METTL1 SLC16A7 TBC1D30 HELB C12orf34 ATXN2 C12orf65 SBNO1
GOLGA3 LNX2 COG3 TPTE2P3 DNAJC3 TTC5 RBM23 ARHGAP5 HMGB1P14
SYNJ2BP MAP3K9 ZC3H14 BTBD7 CYP46A1 WDR25 HSP90AA1 TRAF3 NIPA2 MGA
TP53BP1 PIGB PARP16 IDH3A C15orf38 SOLH CLDN9 MTRNR2L4
EARS2 DCTN5 TAOK2 SRCAP DNAJA2 BCAR1 C16orf46 ZC3H18 VPS53
PELP1 SHISA6 ALKBH5 C17orf108 MMP28 CNTNAP1 PSME3 UBTF UBE2Z
PPP1R9B DCAF7 CANT1 CSNK1D FOXK2 RP13 GNAL ZNF397 TPGS2 MBD1
AP3D1 ZNF555 SEMA6B TNFSF14 ZNF121 ZNF562 ZNF441 BRD4 JAK3 PIK3R2
MPV17L2 ARMC6 GATAD2A ZNF91 MAP3K10 SMG9 DHX34 PRMT1 ZNF347 PEG3

ZNF587 ZNF417 SEC23B FOXA2 ZNF341 NCOA5 ADNP C20orf201 FAM207CP
URB1 C21orf59 OLIG1 RPS5P2 PRDM15 UBE2G2 KRT18P5 HIC2 ZNF70 XBP1
PRR14L GGA1 TCF20 RP6 TMSB4X ZNF81 SMC1A MAGEH1 KIAA2022 MID2
ZBTB33 CUL4B XIAP HCFC1
GOBPID Pvalue OddsRatio ExpCount Count Size Term
Tissue: Muscle_Skeletal=>Thyroid Type: asymmetric
SourceGene: NR1I3(ENSG00000143257.7)
TargetGeneSet: SEPT2P1 KCNA3 FAM46C SLAMF7 PTPRC IGKV1OR2-118 EOMES
TRAT1 CD96 GZMA GPSM3 RP1 TAGAP CARD11 ARL4A TRGC1 TRGV4
PLEKHA2 CHRNA6 RP11 APBB1IP PRF1 CD59 CD3E CD3D SPIC LCP1
TRAV2 IGHV6-1 IGHV3-49 IGHV4OR15-8 RASGRP1 ITGAL TNK1 CCL5
TMIGD2 MYO1F ADAM33 IL2RB CENPM PIM2 IL2RG MTCP1
GOBPID Pvalue OddsRatio ExpCount Count Size Term
GO:0046649 3.64642720047823e-07 12.3767688679245 0.988789703134731
9 433 lymphocyte activation
GO:0042102 3.66187242958608e-07 42.0286885245902 0.150716213410837
5 66 positive regulation of T cell proliferation
GO:0042110 4.43334223453641e-07 14.5152411575563 0.728461698152377
8 319 T cell activation
GO:0002252 1.86362804014922e-06 11.8857142857143 0.881461490554287
8 386 immune effector process
GO:0050870 2.13674031793135e-06 19.1959595959596 0.390492007473531
6 171 positive regulation of T cell activation
GO:0050671 2.38586772474431e-06 28.1142072213501 0.219223583143035
5 96 positive regulation of lymphocyte proliferation
GO:0032946 2.51146686554061e-06 27.8066770186335 0.221507162134108
5 97 positive regulation of mononuclear cell proliferation
GO:0050852 2.77825602583929e-06 27.2112462006079 0.226074320116255
5 99 T cell receptor signaling pathway
GO:0051249 2.8322941466464e-06 13.7951505016722 0.646252854473739
7 283 regulation of lymphocyte activation
GO:0070665 2.91975102891644e-06 26.9229323308271 0.228357899107328
5 100 positive regulation of leukocyte proliferation
GO:0042129 3.06685408569195e-06 26.640625 0.230641478098401 5
101 regulation of T cell proliferation
GO:0002694 6.49763083231527e-06 12.093091621754 0.733028856134524 7
321 regulation of leukocyte activation
GO:0042098 6.59049025861439e-06 22.6058786346397 0.269462320946647
5 118 T cell proliferation
GO:0045061 7.21868328547381e-06 102.885714285714 0.0388208428482458
3 17 thymic T cell selection
GO:0051251 8.00881384652893e-06 15.1079213184476 0.490969483080756
6 215 positive regulation of lymphocyte activation
GO:0050851 8.07333000487323e-06 21.6404358353511 0.280880215902014
5 123 antigen receptor-mediated signaling pathway
GO:0050863 9.37404367992686e-06 14.6801033591731 0.504670957027195
6 221 regulation of T cell activation
GO:0050865 1.00205734608208e-05 11.2836538461538 0.783267593938136
7 343 regulation of cell activation
GO:0002429 1.1816084699071e-05 19.9358258928571 0.303716005812747
5 133 immune response-activating cell surface receptor signaling pathway
GO:0002696 1.26720978076269e-05 13.8923152227117 0.532073904920075
6 233 positive regulation of leukocyte activation
GO:0050778 1.47416451699015e-05 10.60407239819 0.831222752750675 7
364 positive regulation of immune response
GO:0050867 1.49935907178376e-05 13.4700854700855 0.548058957857588
6 240 positive regulation of cell activation
GO:0038110 1.51494826074357e-05 930.129032258065 0.00685073697321985
2 3 interleukin-2-mediated signaling pathway
GO:0002768 1.73717388402897e-05 18.3440390544707 0.328835374714553
5 144 immune response-regulating cell surface receptor signaling pathway
GO:0050670 1.73717388402897e-05 18.3440390544707 0.328835374714553
5 144 regulation of lymphocyte proliferation

GO:0032944	1.7963050440714e-05	18.2117346938776	0.331118953705626
5	145	regulation of mononuclear cell proliferation
Tissue: Muscle_Skeletal=>Thyroid	Type:	cluster
SourceGene:	RP11-55L3.2(ENSG00000213493.3)
TargetGeneSet:	DNAJC11	UBIAD1	RP1	PAQR7	EYA3	PHACTR4	SNRNP40	HDAC1
PRPF38A	TARS2	METTL13	DHX9	PPM1G	POLR1B	CLN5	TEX264	WDR82	COPG1
KDM5A	PSMD2	BOD1L	FIP1L1	KIT	GARS	SLCO4C1	AP3S1	IK	HAVCR2	RP3
TMEM151B		POLR2J4	SUMF2	COPS6	CASP2	ADRA1D	ELP3	TEX10	RNF20
FKBP15	ATP6V1G1		EXOSC2	POLR3A	NAT10	RP11	NUP160	GANAB	TMEM179B
VPS11	NCKAP1L	SUOX	OBFC2B	CENPC1P1		SART3	SUPT16H	YLPM1	MGA	MPI
HIRIP3	RNF40	CTCF	TMCO7	SF3B3	DHX38	RP13	RPA1	TSR1	RNF167	TP53
PFAS	ERAL1	AP2B1	CDK12	PSME3	GPATCH8	EFTUD2	BCAS3	DCAF7	EIF4A3	MAU2
IDH3B	DDX27	PRPF6	LINC00515		ITGB2	L3MBTL2	XRCC6	SLC35A2	SMC1A
GNL3L	RBM41
GOBPID	Pvalue	OddsRatio	ExpCount	Count	Size	Term
GO:0008380	9.38632128306859e-08	8.61430102587659	1.67635457753789
12	323	RNA splicing
GO:0000377	9.40777010348585e-08	11.2466296590008	1.05875025949761
10	204	RNA splicing, via transesterification reactions with bulged adenosine as nucleophile
GO:0000398	9.40777010348585e-08	11.2466296590008	1.05875025949761
10	204	nuclear mRNA splicing, via spliceosome
GO:0000375	1.18048959291484e-07	10.9601855431001	1.08470002075981
10	209	RNA splicing, via transesterification reactions
GO:0006397	1.19572460637369e-07	7.59944843723884	2.07079094872327
13	399	mRNA processing
Tissue: Muscle_Skeletal=>Thyroid	Type:	asymmetric
SourceGene:	RP11-629O1.2(ENSG00000261220.1)
TargetGeneSet:	NOC2L	UBE2J2	DNAJC16	TCEB3	LYPLA2	TXLNA	MARCKSL1
S100PBP	SF3A3	CTPS	EIF2B3	RP11	CDC7	ATXN7L2	AMPD2	KCND3	SLC39A1
FLAD1	UBQLN4	CCT3	HAPLN2	PFKFB2	NOL10	ITSN2	DNMT3A	SLC5A6	MRPS5
SEMA4C	MAP4K4	MTND5P28		IWS1	GORASP2	HSPD1	TTLL4	HMGB1P3	EFHD1	DPH3
SLC25A38		ZDHHC3	CDCP1	SEMA3F	IFRD2	ARF4	PXK	COPG1	H1FX-AS1
MED12L	GMPS	MCCC1	SLC12A7	ARSB	GPR98	FSTL4	ADRB2	MAK	TRIM27
RANP1	PPP1R18	DAXX	ITPR3	BRPF3	BYSL	GPR110	SYTL3	HEATR2	CCZ1
IGFBP3	PRKAG2	PINX1	GATA4	POLR3D	INTS9	KLF10	NUDCD1	DERL1	EEF1D	PLAA
DNAJA1	DCAF12	CCL19	DCAF10	FXN	NCBP1	CDK5RAP2		OLFML2A	NUP188
METTL11A		SURF4	GRIN1	NGB	NUDT5	RRP12	ENTPD7	TMEM123	SPTY2D1
CAPRIN1	CHST1	PATL1	CCDC86	C11orf84		MARK2	STIP1	IL18BP	CLPB
P2RY6	DDX10	ARCN1	HSPA8	FKBP4	RP4	NOP2	DDX23	METTL1	UTP20
ATXN2	DHX37	PUS1	GOLGA3	RGCC	ATP11A	RBM23	EIF2B2	AHSA1	ZC3H14	CTD
DUOXA1	DUOX1	SLTM	PARP16	IDH3A	SOLH	CARHSP1	UBFD1	DCTN5	CD2BP2	MT1F
CIRH1A	BCAR1	COX4NB	ZC3H18	TSR1	CYB5D2	C17orf51		C17orf108		UTP6
CDK12	LEPREL4	CNTNAP1	PSME3	UBE2Z	PPP1R9B	FAM20A	HN1	MRPL38	PTPN2
ZNF397	MBD1	PTBP1	TNFSF14	ANGPTL4	ZNF121	ZNF441	EMR2	ANKLE1	JAK3
CCDC124	MPV17L2	MAP3K10	ETHE1	BCL3	PRMT1	ZNF587	PAK7	TOMM34	RPL12P9
EXOSC3P1		RUNX1	PRDM15	UBE2L3	HPS4	MORC2	GGA1	TCF20	ZNF81
TSC22D3	RP13	TMSB4Y
GOBPID	Pvalue	OddsRatio	ExpCount	Count	Size	Term
GO:0022613	8.33411188747106e-08	6.92553975776725	2.31990865684036
14	225	ribonucleoprotein complex biogenesis
GO:0071843	1.7581208002344e-07	6.4881646090535	2.46425852882153	14
239	cellular component biogenesis at cellular level
GO:0022618	6.03014277432934e-06	9.27040026086248	0.979516988443706
8	95	ribonucleoprotein complex assembly
GO:0071826	8.84007004973229e-06	8.76349059512797	1.03107051415127
8	100	ribonucleoprotein complex subunit organization
Tissue: Muscle_Skeletal=>Thyroid	Type:	asymmetric
SourceGene:	RP11-734I18.1(ENSG00000251129.1)
TargetGeneSet:	SDHC	IFT172	CCDC104	RPL15	RPL29	MLF1	RP11	CC2D2A
LAMTOR3	MTND5P11		CTB	GNB2L1	EIF3E	EIF3H	ZNF618	RPL12	EEF1A1P5
COX15	MRPL16	SLC48A1	RPL6	ASB16	RPL12P4	IFNGR2	EIF3L	RPL3	RPS4X
GOBPID	Pvalue	OddsRatio	ExpCount	Count	Size	Term

GO:0006413	8.94682028637923e-13	64.2182817182817	0.241920974327036
9	152	translational initiation
GO:0000184	4.72339942275701e-10	55.921875	0.189398657532351	7
119	nuclear-transcribed mRNA catabolic process, nonsense-mediated decay
GO:0006415	4.88666401663353e-09	59.5557093425606	0.144834267524739
6	91	translational termination
GO:0000956	5.53154659155372e-09	38.5270061728395	0.268977925403086
7	169	nuclear-transcribed mRNA catabolic process
GO:0006402	7.93522129315348e-09	36.4762426900585	0.283302193619819
7	178	mRNA catabolic process
GO:0006414	1.16321782613037e-08	51.0837789661319	0.167116462528545
6	105	translational elongation
GO:0006614	1.16321782613037e-08	51.0837789661319	0.167116462528545
6	105	SRP-dependent cotranslational protein targeting to membrane
GO:0006613	1.23176224574141e-08	50.5694117647059	0.168708047885959
6	106	cotranslational protein targeting to membrane
GO:0045047	1.23176224574141e-08	50.5694117647059	0.168708047885959
6	106	protein targeting to ER
GO:0072599	1.23176224574141e-08	50.5694117647059	0.168708047885959
6	106	establishment of protein localization to endoplasmic reticulum
GO:0006401	2.11025172744099e-08	31.4425505050505	0.326274998270016
7	205	RNA catabolic process
GO:0070972	2.35052545672698e-08	45.1134453781513	0.187807072174936
6	118	protein localization to endoplasmic reticulum
GO:0019080	1.02708662569541e-07	34.7659229208925	0.240329388969621
6	151	viral genome expression
GO:0019083	1.02708662569541e-07	34.7659229208925	0.240329388969621
6	151	viral transcription
GO:0006612	1.11068333584869e-07	34.2881152460984	0.243512559684451
6	153	protein targeting to membrane
GO:0043624	1.11068333584869e-07	34.2881152460984	0.243512559684451
6	153	cellular protein complex disassembly
GO:0043241	1.34449694415459e-07	33.1486068111455	0.251470486471524
6	158	protein complex disassembly
GO:0034623	2.38085620590658e-07	29.9579831932773	0.27693585219016
6	174	cellular macromolecular complex disassembly
GO:0032984	2.81460650567542e-07	29.081944916695	0.284893778977233	6
179	macromolecular complex disassembly
GO:0072594	6.07562117312445e-07	25.3654188948307	0.324683412912601
6	204	establishment of protein localization to organelle
GO:0019058	1.25703115685503e-06	22.2792156862745	0.367656217562798
6	231	viral infectious cycle
GO:0022415	2.49964104162857e-06	19.6952292728115	0.413812192927825
6	260	viral reproductive process
GO:0071845	4.16356426373618e-06	17.9644519678375	0.452010241505778
6	284	cellular component disassembly at cellular level
GO:0022411	4.5129496406714e-06	17.7046307884856	0.458376582935437
6	288	cellular component disassembly
Tissue: Muscle_Skeletal=>Thyroid	Type:	asymmetric
SourceGene:	SMAD7(ENSG00000101665.3)
TargetGeneSet:	RP11	SEMA4A	TTC24	FCRL5	FCRL2	KIF21B	STAMBP	IGKJ5	IGKC
IGKJ2	IGKJ1	IGKV3-11	IGKV1-12	IGKV3-15	SP140	OR2A5
CNTNAP2	SYT12	CRTAM	ARHGAP9	GS1	TRDC	IGHA2	IGHG2	IGHG1	GPR114
IFT20	FAM209A	IGLV3-10	IGLV2-8	IGLC2	IGLC3	MIAT
GOBPID	Pvalue	OddsRatio	ExpCount	Count	Size	Term
GO:0006958	3.34410537044235e-09	122.974358974359	0.0622794270292713
5	50	complement activation, classical pathway
GO:0006956	1.09859748955005e-08	95.3249336870027	0.0784720780568819
5	63	complement activation
GO:0002455	1.28906736371292e-08	92.1346153846154	0.0809632551380527
5	65	humoral immune response mediated by circulating immunoglobulin
GO:0002449	3.60247991194033e-08	44.3229813664596	0.208013286277766
6	167	lymphocyte mediated immunity

GO:0072376 4.20414596204078e-08 71.7082917082917 0.102138260328005
5 82 protein activation cascade
GO:0002460 5.8222716843612e-08 40.7371428571429 0.225451525845962
6 181 adaptive immune response based on somatic recombination of immune receptors built from immunoglobulin superfamily domains
GO:0002250 9.92797013957539e-08 37.0859375 0.246626531035914 6
198 adaptive immune response
GO:0002443 1.44733874159179e-07 34.7024390243902 0.262819182063525
6 211 leukocyte mediated immunity
GO:0016064 1.93212855523158e-07 51.9847605224964 0.138260328004982
5 111 immunoglobulin mediated immune response
GO:0019724 2.20811624721492e-07 50.5434015525759 0.141997093626739
5 114 B cell mediated immunity
GO:0002252 2.2681262098458e-07 23.5975053969777 0.480797176665975
7 386 immune effector process
GO:0006959 3.09915170958394e-07 47.0611439842209 0.151961801951422
5 122 humoral immune response
GO:0050778 3.52128674649164e-06 19.6578212290503 0.453394228773095
6 364 positive regulation of immune response
Tissue: Muscle_Skeletal=>Thyroid Type: asymmetric
SourceGene: TBCAP3(ENSG00000249055.1)
TargetGeneSet: ACAP3 ARHGEF10L RPL11 LRRC8B SLC35A3 MAN1A2 OTUD7B LMNA
RGS4 KCNT2 TSSC1 RP11 CCDC88A NR4A2 NPPC ARF4 PPIAP16 MAGI1-IT1
TFG ATP6V1A PFN2 DEFB131 TAPT1 MOB1B SEC31A SEC24D STOX2 C5orf27
STARD4 PHF15 CAMLG RPS14 CNOT8 THG1L CPEB4 ELOVL2 RP3 CCND3
KLHDC3 UFL1 FOXO3 C7orf50 SLC29A4 ZCWPW1 TMEM168 MKRN1 ZYX FAM82B
DGAT1 CTD GOLGA2 RPL7A IL15RA STOX1 PTPRE PRMT3 DRAP1 NDUFS8 RPS3
CALCOCO1 RPL41 ARL1 TCP11L2 VSIG10 RNU4-2 CHFR SCFD1 RPL32P29
SIPA1L1 TGFB3 FOXN3 TELO2 ECI1 CLUAP1 RPS15A C16orf58 GPR56
BCAR1 C16orf7 TP53I13 RPL17 PLEKHH3 SUPT4H1 BTBD17 FAM100B C17orf70
ARHGDIA ATP8B1 FBN3 ILVBL COPE RPS11 ISOC2 NECAB3 UCKL1 LINC00158
ARVCF APOL2 TRABD CCDC22 MAGED2 FOXO4 RP4 FAM199X
GOBPID Pvalue OddsRatio ExpCount Count Size Term
GO:0006415 2.4029949032464e-08 20.2579730687456 0.478582797038267
8 91 translational termination
GO:0006414 7.43372609414547e-08 17.3171619163129 0.552210919659539
8 105 translational elongation
GO:0006614 7.43372609414547e-08 17.3171619163129 0.552210919659539
8 105 SRP-dependent cotranslational protein targeting to membrane
GO:0006613 8.00688636884317e-08 17.1392557022809 0.557470071275344
8 106 cotranslational protein targeting to membrane
GO:0045047 8.00688636884317e-08 17.1392557022809 0.557470071275344
8 106 protein targeting to ER
GO:0072599 8.00688636884317e-08 17.1392557022809 0.557470071275344
8 106 establishment of protein localization to endoplasmic reticulum
GO:0019080 9.17468376821776e-08 13.4640529745638 0.794131893986575
9 151 viral genome expression
GO:0019083 9.17468376821776e-08 13.4640529745638 0.794131893986575
9 151 viral transcription
GO:0070972 1.84678499912722e-07 15.2566844919786 0.620579890665006
8 118 protein localization to endoplasmic reticulum
GO:0000184 1.97159417954415e-07 15.1181770005299 0.625839042280811
8 119 nuclear-transcribed mRNA catabolic process, nonsense-mediated decay
GO:0000956 2.40668033314045e-07 11.9342350746269 0.888796623071068
9 169 nuclear-transcribed mRNA catabolic process
GO:0006402 3.73811607986339e-07 11.2915305131149 0.936128987613314
9 178 mRNA catabolic process
GO:0006401 1.22282571351335e-06 9.71756015839171 1.07812608124005
9 205 RNA catabolic process
GO:0006413 1.28258848919235e-06 11.6266339869281 0.799391045602381
8 152 translational initiation
GO:0006612 1.3476829986127e-06 11.5456389452333 0.804650197218186
8 153 protein targeting to membrane

GO:0043624 1.3476829986127e-06 11.5456389452333 0.804650197218186
8 153 cellular protein complex disassembly
GO:0043241 1.71710525455108e-06 11.156862745098 0.830945955297211 8
158 protein complex disassembly
GO:0019058 3.27711101709493e-06 8.56373537716821 1.21486402325099
9 231 viral infectious cycle
GO:0034623 3.53234654863319e-06 10.070163004961 0.915092381150093 8
174 cellular macromolecular complex disassembly
GO:0032984 4.35900428544295e-06 9.77227382180943 0.941388139229119
8 179 macromolecular complex disassembly
GO:0022415 8.56194963266732e-06 7.55877980614854 1.36737942010933
9 260 viral reproductive process
GO:0072594 1.13913791253346e-05 8.51080432172869 1.07286692962425
8 204 establishment of protein localization to organelle
GO:0071845 1.73410816782255e-05 6.88738127544098 1.49359905888866
9 284 cellular component disassembly at cellular level
GO:0022411 1.93742077695957e-05 6.78671160327395 1.51463566535188
9 288 cellular component disassembly
Tissue: Muscle_Skeletal=>Thyroid Type: cluster
SourceGene: TBCAP3(ENSG00000249055.1)
TargetGeneSet: ACAP3 ARHGEF10L RPL11 LRRC8B SLC35A3 MAN1A2 OTUD7B LMNA
RGS4 KCNT2 TSSC1 RP11 CCDC88A NR4A2 NPPC ARF4 PPIAP16 MAGI1-IT1
TFG ATP6V1A PFN2 DEFB131 TAPT1 MOB1B SEC31A SEC24D STOX2 C5orf27
STARD4 PHF15 CAMLG RPS14 CNOT8 THG1L CPEB4 ELOVL2 RP3 CCND3
KLHDC3 UFL1 FOXO3 C7orf50 SLC29A4 ZCWPW1 TMEM168 MKRN1 ZYX FAM82B
DGAT1 CTD GOLGA2 RPL7A IL15RA STOX1 PTPRE PRMT3 DRAP1 NDUFS8 RPS3
CALCOCO1 RPL41 ARL1 TCP11L2 VSIG10 RNU4-2 CHFR SCFD1 RPL32P29
SIPA1L1 TGFB3 FOXN3 TELO2 ECI1 CLUAP1 RPS15A C16orf58 GPR56
BCAR1 C16orf7 TP53I13 RPL17 PLEKHH3 SUPT4H1 BTBD17 FAM100B C17orf70
ARHGDIA ATP8B1 FBN3 ILVBL COPE RPS11 ISOC2 NECAB3 UCKL1 LINC00158
ARVCF APOL2 TRABD CCDC22 MAGED2 FOXO4 RP4 FAM199X
GOBPID Pvalue OddsRatio ExpCount Count Size Term
GO:0006415 2.4029949032464e-08 20.2579730687456 0.478582797038267
8 91 translational termination
GO:0006414 7.43372609414547e-08 17.3171619163129 0.552210919659539
8 105 translational elongation
GO:0006614 7.43372609414547e-08 17.3171619163129 0.552210919659539
8 105 SRP-dependent cotranslational protein targeting to membrane
GO:0006613 8.00688636884317e-08 17.1392557022809 0.557470071275344
8 106 cotranslational protein targeting to membrane
GO:0045047 8.00688636884317e-08 17.1392557022809 0.557470071275344
8 106 protein targeting to ER
GO:0072599 8.00688636884317e-08 17.1392557022809 0.557470071275344
8 106 establishment of protein localization to endoplasmic reticulum
GO:0019080 9.17468376821776e-08 13.4640529745638 0.794131893986575
9 151 viral genome expression
GO:0019083 9.17468376821776e-08 13.4640529745638 0.794131893986575
9 151 viral transcription
GO:0070972 1.84678499912722e-07 15.2566844919786 0.620579890665006
8 118 protein localization to endoplasmic reticulum
GO:0000184 1.97159417954415e-07 15.1181770005299 0.625839042280811
8 119 nuclear-transcribed mRNA catabolic process, nonsense-mediated decay
GO:0000956 2.40668033314045e-07 11.9342350746269 0.888796623071068
9 169 nuclear-transcribed mRNA catabolic process
GO:0006402 3.73811607986339e-07 11.2915305131149 0.936128987613314
9 178 mRNA catabolic process
GO:0006401 1.22282571351335e-06 9.71756015839171 1.07812608124005
9 205 RNA catabolic process
GO:0006413 1.28258848919235e-06 11.6266339869281 0.799391045602381
8 152 translational initiation
GO:0006612 1.3476829986127e-06 11.5456389452333 0.804650197218186
8 153 protein targeting to membrane
GO:0043624 1.3476829986127e-06 11.5456389452333 0.804650197218186

8	153	cellular protein complex disassembly
GO:0043241	1.71710525455108e-06	11.156862745098 0.830945955297211	8
158	protein complex disassembly
GO:0019058	3.27711101709493e-06	8.56373537716821	1.21486402325099
9	231	viral infectious cycle
GO:0034623	3.53234654863319e-06	10.070163004961 0.915092381150093	8
174	cellular macromolecular complex disassembly
GO:0032984	4.35900428544295e-06	9.77227382180943	0.941388139229119
8	179	macromolecular complex disassembly
GO:0022415	8.56194963266732e-06	7.55877980614854	1.36737942010933
9	260	viral reproductive process
GO:0072594	1.13913791253346e-05	8.51080432172869	1.07286692962425
8	204	establishment of protein localization to organelle
GO:0071845	1.73410816782255e-05	6.88738127544098	1.49359905888866
9	284	cellular component disassembly at cellular level
GO:0022411	1.93742077695957e-05	6.78671160327395	1.51463566535188
9	288	cellular component disassembly
Tissue: Muscle_Skeletal=>Thyroid	Type:	asymmetric
SourceGene:	U2(ENSG00000222389.1)
TargetGeneSet:	MAN1C1	RPS7	MFF	JAGN1	BMP2K	RP11	CTD	PLEKHG4B
ATP6V0E1	CPEB4	RPL10A	RPS3AP26	RPL35	RPL7A	P2RX3	RPL6	CHKB
RPS15A	FSCN2	TP53INP2
GOBPID	Pvalue	OddsRatio	ExpCount	Count	Size	Term
GO:0006415	4.01611449165716e-10	101.294117647059	0.100754273060688
6	91	translational termination
GO:0006414	9.61586459350121e-10	86.8848484848485	0.11625493045464
6	105	translational elongation
GO:0006614	9.61586459350121e-10	86.8848484848485	0.11625493045464
6	105	SRP-dependent cotranslational protein targeting to membrane
GO:0006613	1.01867392935224e-09	86.01	0.117362120268494	6	106
cotranslational protein targeting to membrane
GO:0045047	1.01867392935224e-09	86.01	0.117362120268494	6	106
protein targeting to ER
GO:0072599	1.01867392935224e-09	86.01	0.117362120268494	6	106
establishment of protein localization to endoplasmic reticulum
GO:0072594	1.03536178759999e-09	56.2131979695431	0.225866722026157
7	204	establishment of protein localization to organelle
GO:0070972	1.95364559544598e-09	76.7303571428571	0.130648398034738
6	118	protein localization to endoplasmic reticulum
GO:0000184	2.05615156802194e-09	76.046017699115 0.131755587848592	6
119	nuclear-transcribed mRNA catabolic process, nonsense-mediated decay
GO:0019080	8.65497447098544e-09	59.1310344827586	0.167185661891911
6	151	viral genome expression
GO:0019083	8.65497447098544e-09	59.1310344827586	0.167185661891911
6	151	viral transcription
GO:0006413	9.00527408901662e-09	58.7219178082192	0.168292851705764
6	152	translational initiation
GO:0006612	9.36723534542493e-09	58.3183673469388	0.169400041519618
6	153	protein targeting to membrane
GO:0043624	9.36723534542493e-09	58.3183673469388	0.169400041519618
6	153	cellular protein complex disassembly
GO:0043241	1.13628486543256e-08	56.3802631578947	0.174935990588887
6	158	protein complex disassembly
GO:0000956	1.70119170987948e-08	52.5349693251534	0.187115078541277
6	169	nuclear-transcribed mRNA catabolic process
GO:0034623	2.0256353412196e-08	50.9535714285714	0.192651027610546
6	174	cellular macromolecular complex disassembly
GO:0006402	2.32062780153623e-08	49.7546511627907	0.197079786865961
6	178	mRNA catabolic process
GO:0032984	2.39967536614885e-08	49.4635838150289	0.198186976679815
6	179	macromolecular complex disassembly
GO:0006401	5.38891409876322e-08	42.9226130653266	0.226973911840011
6	205	RNA catabolic process

GO:0019058 1.09526014563922e-07 37.8933333333333 0.255760847000208
6 231 viral infectious cycle
GO:0022415 2.20453366736355e-07 33.4984251968504 0.287869351601965
6 260 viral reproductive process
GO:0033365 3.40946410365617e-07 23.3149737720553 0.523700781952806
7 473 protein localization to organelle
GO:0006605 3.45863552375854e-07 23.2633832976445 0.52480797176666
7 474 protein targeting
GO:0071845 3.70908453898079e-07 30.5546762589928 0.314441907134454
6 284 cellular component disassembly at cellular level
GO:0022411 4.02707066625748e-07 30.1127659574468 0.318870666389869
6 288 cellular component disassembly
GO:0016032 5.48524295696051e-06 18.8629213483146 0.499342606048024
6 451 viral reproduction
Tissue: Nerve_Tibial=>Thyroid Type: asymmetric
SourceGene: AK3(ENSG00000147853.10)
TargetGeneSet: RPL22 RP3 CNKSR1 JTB RPS27 FAM78B NDUFB1P2
RPL14 RPL29 METTL15P1 RP11 RPS3A STK32A PCYOX1L PRELID1 DEK
EEF1A1 CASD1 RPS3AP26 PCOLCE RPL7 EEF1A1P5 RPL7A RPS3AP5
RPL27A CTD COPS7A TFCP2 NACA RPL18AP3 SPG20OS LINC00571 FHP1
POLE2 CES3 LGALS9 RPL19 VPS25 EPX TCAM1 LRRC37A3 RPS15 DHPS
GLTSCR2 RPS5 MAFB RP4 PISD RPL3 RPS4X SPRY3
GOBPID Pvalue OddsRatio ExpCount Count Size Term
GO:0006414 9.52400953644563e-22 104.945054945055 0.254307660369525
14 105 translational elongation
GO:0006415 1.3235913401609e-20 108.621212121212 0.220399972320255
13 91 translational termination
GO:0006614 9.4483628916079e-20 92.001976284585 0.254307660369525 13
105 SRP-dependent cotranslational protein targeting to membrane
GO:0006613 1.07537836841364e-19 91.0063538611926 0.25672963808733
13 106 cotranslational protein targeting to membrane
GO:0045047 1.07537836841364e-19 91.0063538611926 0.25672963808733
13 106 protein targeting to ER
GO:0072599 1.07537836841364e-19 91.0063538611926 0.25672963808733
13 106 establishment of protein localization to endoplasmic reticulum
GO:0070972 4.62144617185643e-19 80.5380952380952 0.28579337070099
13 118 protein localization to endoplasmic reticulum
GO:0000184 5.18084198822889e-19 79.7727272727273 0.288215348418795
13 119 nuclear-transcribed mRNA catabolic process, nonsense-mediated decay
GO:0019080 1.2693059713826e-17 61.1376811594203 0.365718635388554
13 151 viral genome expression
GO:0019083 1.2693059713826e-17 61.1376811594203 0.365718635388554
13 151 viral transcription
GO:0006413 1.38603847599456e-17 60.6935905820798 0.368140613106359
13 152 translational initiation
GO:0006612 1.51258162845701e-17 60.2558441558442 0.370562590824164
13 153 protein targeting to membrane
GO:0043624 1.51258162845701e-17 60.2558441558442 0.370562590824164
13 153 cellular protein complex disassembly
GO:0043241 2.32048493093861e-17 58.1576802507837 0.382672479413189
13 158 protein complex disassembly
GO:0000956 5.66828256766503e-17 54.0151515151515 0.409314234309044
13 169 nuclear-transcribed mRNA catabolic process
GO:0019058 8.25737690205258e-17 43.6221198156682 0.559476852812954
14 231 viral infectious cycle
GO:0034623 8.33715556476071e-17 52.3193111236589 0.421424122898069
13 174 cellular macromolecular complex disassembly
GO:0006402 1.12576743050793e-16 51.0366391184573 0.431112033769289
13 178 mRNA catabolic process
GO:0032984 1.21219596404493e-16 50.7256297918949 0.433534011487094
13 179 macromolecular complex disassembly
GO:0022415 4.34881362463966e-16 38.4010840108401 0.629714206629299
14 260 viral reproductive process

GO:0072594	6.76709447762259e-16	44.0088053307949	0.494083454432219
13	204	establishment of protein localization to organelle
GO:0006401	7.21494711640007e-16	43.7765151515151	0.496505432150024
13	205	RNA catabolic process
GO:0071845	4.97717584360643e-14	30.8428379738343	0.687841671856619
13	284	cellular component disassembly at cellular level
GO:0022411	5.95874574839357e-14	30.3856198347107	0.69752958272783
13	288	cellular component disassembly
GO:0016032	8.67784210839148e-13	21.3257055682685	1.09231195073005
14	451	viral reproduction
GO:0033365	3.21483071021847e-11	17.9276679841897	1.14559546052176
13	473	protein localization to organelle
GO:0006605	3.30068489648991e-11	17.887497535003 1.14801743823957	13
474	protein targeting
Tissue: Nerve_Tibial=>Thyroid	Type:	asymmetric
SourceGene:	ATP6V0CP1(ENSG00000230201.4)
TargetGeneSet:	IGSF21	AKR7A2	C1QA	C1QC	C1QB	MACF1	CD53	FCER1G
FCGR3A	CCL15	TMEM177	TTC30B	TRANK1	GOLGA4	SLC9A9	FAM53A	AASDH	ODAM	RP11
HERC5	CFI	MARCH1	CCDC111	TLR3	FAM105A	PCDHGA1	CTB	AIF1	HLA-DRB6
RP1	CCNC	FAM26F	SNX10	TBXAS1	ZNF282	NUB1	CTD	DOK2	MS4A6A
MS4A7	PTPRCAP	C3AR1	KIF21A	SELPLG	CLIP1	TDRD3	BATF	SAMD15	GALC
EFCAB11	MPI	TTC23	CORO1A	MLYCD	CAMTA2	DHX58	VPS25	MKS1	SMURF2
C19orf35		GNA15	EBI3	CD209	DOCK6	HCST	SIGLEC16		CD33
SIGLEC8	PCNA	HCK	PXMP4	ASIP	CSTF1	CASS4	PWP2	ITGB2	C22orf28
ATF4	ARSA	GTPBP6	CYBB	VSIG4	BTK	MAGEA5
GOBPID	Pvalue	OddsRatio	ExpCount	Count	Size	Term
GO:0002253	1.08199749417542e-06	8.55642566624173	1.38627084630821
10	299	activation of immune response
GO:0002252	1.4377197720213e-06	7.33804761904762	1.78963393536779
11	386	immune effector process
GO:0002460	1.8017739586064e-06	11.1382384637994	0.839180679537748
8	181	adaptive immune response based on somatic recombination of immune receptors built from immunoglobulin superfamily domains
GO:0002250	3.52042987973701e-06	10.1295272078501	0.917998754411459
8	198	adaptive immune response
GO:0050778	6.28010223781468e-06	6.95311725641788	1.68763407376652
10	364	positive regulation of immune response
GO:0006956	1.0510244848342e-05	19.9193548387097	0.292090512767283
5	63	complement activation
GO:0019724	1.42428302192121e-05	13.0018214936248	0.528544737388416
6	114	B cell mediated immunity
GO:0050900	1.48767819098159e-05	8.23510584127446	1.11736212026849
8	241	leukocyte migration
Tissue: Thyroid=>Nerve_Tibial	Type:	asymmetric
SourceGene:	CCT8L1P(ENSG00000020219.8)
TargetGeneSet:	C1QC	C1QB	CD52	DHDDS	RPS6KA1	ERMAP	CPT2	IFI44	CD84
ARHGAP30		PTPRC	TMEM81	PTRHD1	DNAJC27	QPCT	CCDC88A	FBXO41	LOXL3	PROC
COL3A1	CLK1	CASP8	EBP	STAB1	PDHB	ACPP	P2RY13	FAM131A	DAPP1
CCNA2	APC	CSF1R	RP11	LY86	GPSM3	DEFA6	AKIRIN2	FAM54A	COL1A2
PIK3CG	IRF5	TAS2R5	DOK2	ADAM28	PPP2R2A	LYN	KIAA0196		FAM49B
FAM83H	C9orf40	SYK	SUSD3	PTGS1	FAM78A	PRKCQ	CDC123	APBB1IP	WDFY4
HELLS	BLNK	NDUFS3	MRPL16	MS4A6A	FEN1	FERMT3	SF1	SLCO2B1	IL10RA
SORL1	NDUFA9	LPAR5	CD4	PTPN6	PLBD1	LRMP	MED21	LARP4	BIN2
NCKAP1L	HVCN1	ERP29	DTX1	LCP1	ARL11	CKAP2	RNASE1	MAX	BCL11B	CTD
PLCB2	VPS18	PARP16	CORO1A	ITGAM	IRF8	ZNF469	CYBA	SNORA67	NDEL1
AKAP10	ADAP2	C17orf75		DDX52	MRPS23	DSC2	VAV1	EMR1	STXBP2
MYO1F	GMIP	ZNF506	TYROBP	ZNF567	RASGRP4	KCNN4	CD33	LAIR1	ZNF548
ERGIC3	ZNF295	ITGB2	CECR6	PITPNB	CYTH4	JOSD1	CBX7	RP1	KLHDC7B	TLR7
PHEX	CYBB	GPR34	WAS	BTK	ARHGAP4
GOBPID	Pvalue	OddsRatio	ExpCount	Count	Size	Term
GO:0046649	7.58581529715495e-09	6.29006024096386	3.41581897446543
18	433	lymphocyte activation
GO:0042110	3.01210299330702e-08	6.99411881977671	2.51650404816276

15	319	T cell activation
GO:0002275	6.55562844714552e-08	23.9841840402588	0.362881461490554
7	46	myeloid cell activation involved in immune response
GO:0002253	9.50110401902084e-08	6.90273684210526	2.3587294996886 14
299	activation of immune response
GO:0070527	2.97791658404737e-07	46.9298820445609	0.149885821050446
5	19	platelet aggregation
GO:0002757	3.30392783566779e-07	7.44605644948562	1.85385094457131
12	235	immune response-activating signal transduction
GO:0007596	3.5152353649221e-07	5.0485886362175 3.92858625700644	17
498	blood coagulation
GO:0050900	4.32944640858016e-07	7.24788081171333	1.90118330911356
12	241	leukocyte migration
GO:0050851	5.3431215864553e-07	10.693984962406 0.970313473116047	9
123	antigen receptor-mediated signaling pathway
GO:0002764	5.87667026407685e-07	7.02941176470588	1.95640440107951
12	248	immune response-regulating signaling pathway
GO:0002521	7.33830439665032e-07	6.25660351502278	2.38239568195973
13	302	leukocyte differentiation
GO:0050778	1.03071600127173e-06	5.5948	2.8714967822296 14	364
positive regulation of immune response
GO:0002429	1.0347493362279e-06	9.82465437788018	1.04920074735312
9	133	immune response-activating cell surface receptor signaling pathway
GO:0002283	1.1616190191301e-06	74.4415584415584	0.0867760016607847
4	11	neutrophil activation involved in immune response
GO:0045730	1.61200452732009e-06	31.2712975098296	0.2051069130164 5
26	respiratory burst
GO:0002768	2.01128258708995e-06	9.01714285714286	1.13597674901391
9	144	immune response-regulating cell surface receptor signaling pathway
GO:0050853	2.85580633788103e-06	27.3566513761468	0.228773095287523
5	29	B cell receptor signaling pathway
GO:0050867	3.01409784877097e-06	6.5793869504388 1.89329458168985	11
240	positive regulation of cell activation
GO:0050865	3.02778961756455e-06	5.46327632763276	2.70583350633174
13	343	regulation of cell activation
GO:0030097	3.48796668194308e-06	4.6973079004329 3.65248079717667	15
463	hemopoiesis
GO:0030155	4.46380198729616e-06	6.29963033675915	1.97218185592693
11	250	regulation of cell adhesion
GO:0002263	5.67745663290834e-06	9.33355209187859	0.970313473116047
8	123	cell activation involved in immune response
GO:0002366	5.67745663290834e-06	9.33355209187859	0.970313473116047
8	123	leukocyte activation involved in immune response
GO:0033628	7.51772815983642e-06	21.8761467889908	0.27610545982977
5	35	regulation of cell adhesion mediated by integrin
GO:0051251	7.62662529661527e-06	6.62851782363977	1.69607639609716
10	215	positive regulation of lymphocyte activation
GO:0048534	7.82275545149041e-06	4.37405303030303	3.90492007473531
15	495	hemopoietic or lymphoid organ development
GO:0050870	8.24329624974661e-06	7.5	1.34897238945402	9	171
positive regulation of T cell activation
GO:0002694	8.55643832069019e-06	5.34094802969732	2.53228150301017
12	321	regulation of leukocyte activation
GO:0050863	9.71160843328381e-06	6.4372949325556 1.7434087606394 10	221
regulation of T cell activation
GO:0002252	1.08749423599764e-05	4.81862341730152	3.04504878555117
13	386	immune effector process
GO:0034109	1.14067743349607e-05	19.883236030025 0.299771642100893	5
38	homotypic cell-cell adhesion
GO:0043299	1.14067743349607e-05	19.883236030025 0.299771642100893	5
38	leukocyte degranulation
GO:0042119	1.29942252501971e-05	34.72	0.149885821050446	4	19
neutrophil activation

GO:0051249	1.44271115055245e-05	5.52238006853227	2.23250986090928
11	283	regulation of lymphocyte activation
GO:0002696	1.53947515461982e-05	6.0857192135219 1.83807348972389	10
233	positive regulation of leukocyte activation
GO:0007229	1.55215477456752e-05	12.7912186379928	0.536433464812124
6	68	integrin-mediated signaling pathway
GO:0090330	1.63543084703709e-05	96.8445945945946	0.0552210919659539
3	7	regulation of platelet aggregation
Tissue: Nerve_Tibial=>Thyroid	Type:	asymmetric
SourceGene:	CTD-3074O7.7(ENSG00000254596.1)
TargetGeneSet:	CSDE1	INTS3	TOMM20	PDIA6	DTYMK	EIF1B	RP11	CNBP
UGDH-AS1	PAPSS1	LARP7	NUDT6	AMACR	APBB3	SH3RF2	NPM1	WTAP
PLEKHA8	CROT	TECPR1	LUC7L2	RP4	TRPV6	SGK3	EIF3H	ERP44	TUBB4B	TET1
EIF3M	PDHX	FKBP2	C11orf85	STT3A	PUS7L	PRR13	HSP90B1	NUS1P2
EIF4A1P7	ENTPD5	MOAP1	CKMT1A	PDIA3	PPIB	TMC3	EIF1	CLTC
RAB12	TMEM241	TLE6	FKBP8	RPN2	C20orf151	SDF2L1	CCDC134	ALG12
GOBPID	Pvalue	OddsRatio	ExpCount	Count	Size	Term
GO:0006457	5.63442705220163e-10	20.8914386584289	0.64272368694208
10	216	protein folding
GO:0006487	7.45249247180947e-06	21.6591046581972	0.27375268147533
5	92	protein N-linked glycosylation
Tissue: Thyroid=>Nerve_Tibial	Type:	asymmetric
SourceGene:	CYP1B1(ENSG00000138061.7)
TargetGeneSet:	PHF13	RPS6KA1	ZC3H12A	LYST-IT1	FOSL2	RP11	LOXL3
UNC50	BAZ2B	EBP	TRAM1L1	POC5	CD74	SLC36A2	RBM24	HLA-DRA	HLA-DMA
HLA-DOA	HLA-DPA1	HLA-DPB1	PPARD	VEGFA	GS1	ABHD11	ABHD11-AS2
CLDN12	IRF5	SYK	NR4A3	ARL5B	APBB1IP	SWAP70	CAPN5	PTPN6	FAR2
CDK17	ARL11	DOCK9	RPGRIP1	CTD	PRSS27	CDRT4	TMEM101	RNFT1	PRR22
FAM125A	GMIP	ZNF85	PVR	BRSK1	ZIK1	SNTA1	MAFB	CECR6	FBXW4P1
JOSD1	GPR34	PNMA6A	RENBP
GOBPID	Pvalue	OddsRatio	ExpCount	Count	Size	Term
GO:0002504	8.99884222033228e-11	122.599431818182	0.0761192997024427
6	22	antigen processing and presentation of peptide or polysaccharide antigen via MHC class II
GO:0060333	9.20173883057114e-09	32.3976098191214	0.273337485295135
7	79	interferon-gamma-mediated signaling pathway
GO:0071346	3.12439656035624e-08	26.7837476610532	0.325237007819528
7	94	cellular response to interferon-gamma
GO:0034341	9.91663303037464e-08	22.3790250447227	0.384056466680507
7	111	response to interferon-gamma
GO:0050867	1.1458642519952e-07	13.4653151726322	0.830392360390284
9	240	positive regulation of cell activation
GO:0050870	1.14730045992857e-07	16.638036809816 0.591654556778078	8
171	positive regulation of T cell activation
GO:0031294	2.11424996885467e-07	28.3241106719368	0.259497612621964
6	75	lymphocyte costimulation
GO:0031295	2.11424996885467e-07	28.3241106719368	0.259497612621964
6	75	T cell costimulation
GO:0051251	6.61372722724634e-07	13.0609615827007	0.743893156182963
8	215	positive regulation of lymphocyte activation
GO:0019886	7.73840549732255e-07	306.340425531915	0.0207598090097571
3	6	antigen processing and presentation of exogenous peptide antigen via MHC class II
GO:0050863	8.1445014274641e-07	12.6876816454281	0.76465296519272
8	221	regulation of T cell activation
GO:0002696	1.21311071480848e-06	12.0008465608466	0.806172583212234
8	233	positive regulation of leukocyte activation
GO:0002495	2.15619759798687e-06	183.778723404255	0.0276797453463428
3	8	antigen processing and presentation of peptide antigen via MHC class II
GO:0050865	2.28697402150652e-06	9.24514385862422	1.18676908172445
9	343	regulation of cell activation
GO:0050778	3.72036959373266e-06	8.68526279628994	1.2594284132586 9

364	positive regulation of immune response
GO:0050851	3.93543317118726e-06	16.6480186480186	0.425576084700021
6	123	antigen receptor-mediated signaling pathway
GO:0051249	5.15417293342853e-06	9.78424242424243	0.979170991626877
8	283	regulation of lymphocyte activation
GO:0002429	6.18604377546172e-06	15.3264137437366	0.460175766382949
6	133	immune response-activating cell surface receptor signaling pathway
GO:0002768	9.76847894324836e-06	14.0938735177866	0.498235416234171
6	144	immune response-regulating cell surface receptor signaling pathway
GO:0042110	1.23690374992472e-05	8.62961261675088	1.10372984568542
8	319	T cell activation
GO:0002694	1.29427009876945e-05	8.57325422181652	1.11064978202201
8	321	regulation of leukocyte activation
GO:0019882	1.33066251569055e-05	13.3141344956413	0.525915161580513
6	152	antigen processing and presentation
GO:0046649	1.50768866598216e-05	7.23613667740451	1.4981662168708 9
433	lymphocyte activation
GO:0002757	1.51476330399005e-05	10.1194410444716	0.81309251954882
7	235	immune response-activating signal transduction
Tissue: Nerve_Tibial=>Thyroid	Type:	asymmetric
SourceGene:	ENSG00000206651.1
TargetGeneSet:	CLSTN1	C1QA	C1QC	C1QB	CMPK1	CD53	CTSS	CD84
FCGR2A	FCGR2B	7SK	ARL4C	BTD	CCR5	RP11	CD200R1	CD86	UBA5
P2RY13	TM4SF19-AS1	CSF1R	HAVCR2	DOCK2	HOXA10	ERV3-1	NCF1C	TFEC
TRBV30	PIGO	TRIM14	SMC2	ALOX5	PSAP	CTSD	FOLR2	IL10RA	CD3E	M6PR
NCKAP1L	LCP1	SUCLA2	RNASE6	NOMO2	SPN	CORO1A	SMCR8	VAV1	ACP5	CD37
LAIR1	CRLS1	PLTP	MX1	ITGB2	RAC2	CYBB	WAS
GOBPID	Pvalue	OddsRatio	ExpCount	Count	Size	Term
GO:0042110	1.05061286416583e-06	10.228064516129	1.08165524877171	9
319	T cell activation
GO:0045060	5.93078768896842e-06	117.342391304348	0.0372984568541969
3	11	negative thymic T cell selection
GO:0043383	7.8888501007261e-06	104.297101449275	0.0406892256591239
3	12	negative T cell selection
GO:0046649	1.26953278577271e-05	7.41757075471698	1.46820289253339
9	433	lymphocyte activation
Tissue: Thyroid=>Nerve_Tibial	Type:	asymmetric
SourceGene:	HIST1H2BI(ENSG00000168242.3)
TargetGeneSet:	RPL11	IL22RA1	PUM1	RP11	RPS27	CAMSAP2	UBC	MBNL1
RANBP3L	RPL37	CTB	RPL10A	HEY2	RPL30	ASTN2	LCN2	RPS24	ACTR1A
KRTAP5-9		RPS25	COMMD6	U6	RPL7AP6	KDM6B	RPL26	RPL17	ZNF14
C19orf18		FOXRED2	SMC1A	CHIC1
GOBPID	Pvalue	OddsRatio	ExpCount	Count	Size	Term
GO:0006415	1.78325549682361e-16	118.065843621399	0.157428551657325
10	91	translational termination
GO:0006414	7.89882819762901e-16	100.568421052632	0.181648328835375
10	105	translational elongation
GO:0006614	7.89882819762901e-16	100.568421052632	0.181648328835375
10	105	SRP-dependent cotranslational protein targeting to membrane
GO:0006613	8.7133434798242e-16	99.5138888888889	0.183378312919521
10	106	cotranslational protein targeting to membrane
GO:0045047	8.7133434798242e-16	99.5138888888889	0.183378312919521
10	106	protein targeting to ER
GO:0072599	8.7133434798242e-16	99.5138888888889	0.183378312919521
10	106	establishment of protein localization to endoplasmic reticulum
GO:0070972	2.63560516523313e-15	88.3827160493827	0.204138121929278
10	118	protein localization to endoplasmic reticulum
GO:0000184	2.87467034349544e-15	87.565749235474 0.205868106013425	10
119	nuclear-transcribed mRNA catabolic process, nonsense-mediated decay
GO:0019080	3.28262315323143e-14	67.5413711583924	0.26122759670611
10	151	viral genome expression
GO:0019083	3.28262315323143e-14	67.5413711583924	0.26122759670611
10	151	viral transcription

GO:0006413 3.51044963663819e-14 67.0610328638498 0.262957580790257
10 152 translational initiation
GO:0006612 3.75236081255074e-14 66.5874125874126 0.264687564874403
10 153 protein targeting to membrane
GO:0043624 3.75236081255074e-14 66.5874125874126 0.264687564874403
10 153 cellular protein complex disassembly
GO:0043241 5.20125158025491e-14 64.3153153153153 0.273337485295135
10 158 protein complex disassembly
GO:0000956 1.02855993134244e-13 59.8197064989518 0.292367310220746
10 169 nuclear-transcribed mRNA catabolic process
GO:0034623 1.38113512859077e-13 57.9756097560976 0.301017230641478
10 174 cellular macromolecular complex disassembly
GO:0006402 1.73742476558946e-13 56.5793650793651 0.307937166978064
10 178 mRNA catabolic process
GO:0032984 1.83847693355907e-13 56.2406311637081 0.30966715106221
10 179 macromolecular complex disassembly
GO:0072594 6.85030496520499e-13 48.9072164948454 0.352916753165871
10 204 establishment of protein localization to organelle
GO:0006401 7.19472842217583e-13 48.6529914529915 0.354646737250017
10 205 RNA catabolic process
GO:0019058 2.37606065091673e-12 42.8506787330317 0.399626323437824
10 231 viral infectious cycle
GO:0022415 7.71088005492001e-12 37.8026666666667 0.449795861878071
10 260 viral reproductive process
GO:0071845 1.849551847592e-11 34.4330900243309 0.491315479897585
10 284 cellular component disassembly at cellular level
GO:0022411 2.12382569663949e-11 33.9280575539568 0.498235416234171
10 288 cellular component disassembly
GO:0016032 1.70492184763235e-09 21.1413454270597 0.780222821950038
10 451 viral reproduction
GO:0033365 2.69983390067445e-09 20.1051115910727 0.818282471801259
10 473 protein localization to organelle
GO:0006605 2.75534740067316e-09 20.0603448275862 0.820012455885406
10 474 protein targeting
GO:0022613 1.84274587978072e-06 20.4859408795962 0.389246418932946
6 225 ribonucleoprotein complex biogenesis
GO:0071843 2.61556716958861e-06 19.2360515021459 0.413466196110996
6 239 cellular component biogenesis at cellular level
Tissue: Nerve_Tibial=>Thyroid Type: asymmetric
SourceGene: IQCK(ENSG00000174628.12)
TargetGeneSet: KDM1A RPL11 PRPF38A SETDB1 WNT9A RPL31 RPL32 GRK7
RPL35A RPL34 EXOSC9 RPL37 RPS23 ZSCAN12 RPS18 RPS10 MEA1 RP11
PDCD2 AQP1 CTD RPL30 ANKRD20A3 NDST2 FAM175B SAAL1 WDR74
TRMT112 FAU RPS3 PA2G4 ZSCAN29 UBE2I POLR2C TRIM16 RPL17 RPL27
RPL38 RPL36 POLR2I ZNF606 FKBP1A-SDCBP2 RPS21 ERVH48-1 TSPEAR
C22orf23 GPKOW
GOBPID Pvalue OddsRatio ExpCount Count Size Term
GO:0006415 1.06484340271148e-27 137.225225225225 0.258182824718013
17 91 translational termination
GO:0019080 1.44397720200429e-27 93.4166666666667 0.428413258598021
19 151 viral genome expression
GO:0019083 1.44397720200429e-27 93.4166666666667 0.428413258598021
19 151 viral transcription
GO:0006414 1.4857246303972e-26 115.28125 0.297903259290015 17
105 translational elongation
GO:0006614 1.4857246303972e-26 115.28125 0.297903259290015 17
105 SRP-dependent cotranslational protein targeting to membrane
GO:0006613 1.76672101846751e-26 113.977996254682 0.300740433188015
17 106 cotranslational protein targeting to membrane
GO:0045047 1.76672101846751e-26 113.977996254682 0.300740433188015
17 106 protein targeting to ER
GO:0072599 1.76672101846751e-26 113.977996254682 0.300740433188015
17 106 establishment of protein localization to endoplasmic reticulum

GO:0070972 1.24071833493747e-25 100.351897689769 0.334786519964016
17 118 protein localization to endoplasmic reticulum
GO:0000184 1.44521759105512e-25 99.3611111111111 0.337623693862016
17 119 nuclear-transcribed mRNA catabolic process, nonsense-mediated decay
GO:0000956 1.06403217343371e-24 73.9021019291679 0.479482388762023
18 169 nuclear-transcribed mRNA catabolic process
GO:0006402 2.80491330611982e-24 69.7010869565217 0.505016953844025
18 178 mRNA catabolic process
GO:0019058 6.30270425777528e-24 57.8391938250429 0.655387170438032
19 231 viral infectious cycle
GO:0006413 1.15304133202581e-23 74.8996913580247 0.431250432496021
17 152 translational initiation
GO:0006612 1.29511764163764e-23 74.34375 0.434087606394021 17
153 protein targeting to membrane
GO:0043624 1.29511764163764e-23 74.34375 0.434087606394021 17
153 cellular protein complex disassembly
GO:0043241 2.2879148116028e-23 71.682328605201 0.448273475884022 17
158 protein complex disassembly
GO:0006401 3.85853626245071e-23 59.5242966751918 0.581620649090028
18 205 RNA catabolic process
GO:0022415 6.23240340013473e-23 50.7753677857412 0.737665213480036
19 260 viral reproductive process
GO:0034623 1.25149674302523e-22 64.3049363057325 0.493668258252024
17 174 cellular macromolecular complex disassembly
GO:0032984 2.05714880735212e-22 62.298353909465 0.507854127742025 17
179 macromolecular complex disassembly
GO:0072594 2.01448505144395e-21 53.875 0.578783475192028 17 204
establishment of protein localization to organelle
GO:0016032 2.2914474365241e-21 34.1372093023256 1.27956542799806
21 451 viral reproduction
GO:0071845 5.98474503630991e-19 37.520443196005 0.805757387032039 17
284 cellular component disassembly at cellular level
GO:0022411 7.59513456674918e-19 36.9561808118081 0.81710608262404
17 288 cellular component disassembly
GO:0033365 3.14167204277754e-15 21.6756213450292 1.34198325375407
17 473 protein localization to organelle
GO:0006605 3.25327734533402e-15 21.6266411378556 1.34482042765207
17 474 protein targeting
GO:0022613 2.89559201436361e-06 13.4031300593632 0.638364127050031
7 225 ribonucleoprotein complex biogenesis
GO:0042254 3.62671779673266e-06 17.1032967032967 0.422738910802021
6 149 ribosome biogenesis
GO:0071843 4.31571459631471e-06 12.5818965517241 0.678084561622033
7 239 cellular component biogenesis at cellular level
GO:0006364 1.06890887301401e-05 20.0771604938272 0.295066085392014
5 104 rRNA processing
GO:0016072 1.40496412678571e-05 18.9219576719577 0.312089128780015
5 110 rRNA metabolic process
Tissue: Nerve_Tibial=>Thyroid Type: asymmetric
SourceGene: KLF15(ENSG00000163884.3)
TargetGeneSet: TNFRSF25 HP1BP3 TXLNA GPBP1L1 TAL1 RP1 GDAP2
UBQLN4 CCT3 PTPRVP URB2 SLC5A6 CCT4 PCBP1-AS1 AUP1 ART4
MRPS5 RBM43 HSPD1 HMGB1P3 CGGBP1 PVRL3-AS1 POGLUT1 KIAA0226
TNIP2 TMEM128 ARHGAP24 TRIM2 CCT5 DNAJC21 RP11 C6orf228
ZNF193 NUDT3 BRPF3 RPL7L1 LRRC73 STX1A PINX1 POLR3D CHMP7 TMEM68
CYHR1 DNAJA1 C9orf80 C9orf114 METTL11A FAM69B FBXO18 HNRNPF
P4HA1 TRIM68 UBQLNL GVINP1 SLC35C1 C11orf84 STIP1 GPR137 C11orf30
EED ATM HSPA8 SLC6A12 FKBP4 RP4 C12orf33 REP15 CCT2
C12orf65 RAN HSPH1 PCID2 TEP1 SOCS6 AHSA1 ZC3H14 HSP90AA1
HERC2P2 ZFYVE19 PPP1R14D TP53BP1 CSK C16orf57 NIP7 TMEM170A
MIR423 CTD ZNF441 ZNF702P ZNF761 ZNF71 ZNF329 SNHG11 TOP1 TOMM34 CTA
RANGAP1 ZNF630
GOBPID Pvalue OddsRatio ExpCount Count Size Term

GO:0006457	5.40303927786045e-10	14.1335992023928	1.06124143657878
12	216	protein folding
GO:0006458	1.71557085096681e-07	29.4051282051282	0.250570894747768
6	51	'de novo' protein folding
GO:0051084	2.90875758793648e-06	26.4948263118995	0.226005120752889
5	46	'de novo' posttranslational protein folding
Tissue: Thyroid=>Nerve_Tibial	Type:	asymmetric
SourceGene:	MAGI2(ENSG00000187391.12)
TargetGeneSet:	C1orf159	ZBTB8OS	PPCS	ATP5F1	RP11	CFLAR	CAMP
PARP3	UBA3	ATP6V1A	NDUFB5	NDUFC1	GUSBP5	PDCD6	UBLCP1	RP1	MRPS18B
SENP6	LSM5	MPLKIP	DUS4L	POT1	LACTB2	U1	C9orf133	NDUFB6
SURF1	C10orf125	MRPL23	IMMP1L	CSTF3	CUL5	C11orf1	ATP5L	CACNA2D4
IFFO1	MAP3K12	ERH	NDUFB1	COX5A	COMMD4	RHOT2	NDUFB10	LCMT1	TXNDC17	SNF8
SKA2	SPHK1	SNRPD1	UQCR11	C19orf70	LIG1	RN5S493	SLC2A11	RBM3
GOBPID	Pvalue	OddsRatio	ExpCount	Count	Size	Term
GO:0022904	8.04425735051335e-12	43.5730223123732	0.285654971974258
9	96	respiratory electron transport chain
GO:0045333	8.86289230077006e-12	32.77318640955	0.422531312711923	10
142	cellular respiration
GO:0022900	1.66916152454155e-10	30.2463529411765	0.398726731714068
9	134	electron transport chain
GO:0015980	1.66753391637132e-08	14.3975104581165	0.913500795792679
10	307	energy derivation by oxidation of organic compounds
GO:0006119	1.74184387725757e-08	44.7692307692308	0.172583212234447
6	58	oxidative phosphorylation
GO:0006120	6.45991136676516e-08	61.0229202037351	0.107120614490347
5	36	mitochondrial electron transport, NADH to ubiquinone
GO:0042773	2.56578666390117e-07	45.0062656641604	0.139851913362397
5	47	ATP synthesis coupled electron transport
GO:0042775	2.56578666390117e-07	45.0062656641604	0.139851913362397
5	47	mitochondrial ATP synthesis coupled electron transport
GO:0006091	3.7358954967509e-07	10.1420907641003	1.27354508338523
10	428	generation of precursor metabolites and energy
Tissue: Thyroid=>Nerve_Tibial	Type:	asymmetric
SourceGene:	RP11-1007G5.2(ENSG00000254588.1)
TargetGeneSet:	IL22RA1	KPNA7	RP11	ILF3	IMPDH2	ANKRD37	CTD	RPL37
RPL10A	RPL39P3	EEF1A1	MRPS12	FBXO43	RPS24	VWA5A	RPS29	RPL7AP6	RPS3AP6
ALKBH5	HILS1	RPS19	SNRPD2	RPL28	C19orf18	C21orf7	EEF1B2P3
RPS4Y1
GOBPID	Pvalue	OddsRatio	ExpCount	Count	Size	Term
GO:0006414	1.364216758407e-13	131.381443298969	0.123520863608055
8	105	translational elongation
GO:0006415	5.73667225715822e-12	119.583333333333	0.107051415126981
7	91	translational termination
GO:0006614	1.59949056608047e-11	102.4	0.123520863608055	7	105
SRP-dependent cotranslational protein targeting to membrane
GO:0006613	1.71154309927941e-11	101.358585858586	0.124697252785274
7	106	cotranslational protein targeting to membrane
GO:0045047	1.71154309927941e-11	101.358585858586	0.124697252785274
7	106	protein targeting to ER
GO:0072599	1.71154309927941e-11	101.358585858586	0.124697252785274
7	106	establishment of protein localization to endoplasmic reticulum
GO:0070972	3.67578705037832e-11	90.3252252252252	0.138813922911909
7	118	protein localization to endoplasmic reticulum
GO:0000184	3.90314413768745e-11	89.5125	0.139990312089129	7	119
nuclear-transcribed mRNA catabolic process, nonsense-mediated decay
GO:0019080	2.10735439400921e-10	69.4652777777778	0.177634765760155
7	151	viral genome expression
GO:0019083	2.10735439400921e-10	69.4652777777778	0.177634765760155
7	151	viral transcription
GO:0006413	2.20774045242664e-10	68.9813793103448	0.178811154937375
7	152	translational initiation
GO:0006612	2.31217887521436e-10	68.5041095890411	0.179987544114594

7	153	protein targeting to membrane
GO:0043624	2.31217887521436e-10	68.5041095890411	0.179987544114594
7	153	cellular protein complex disassembly
GO:0043241	2.89998430384273e-10	66.212582781457	0.185869490000692	7
158	protein complex disassembly
GO:0000956	4.65526458386655e-10	61.6691358024691	0.198809770950107
7	169	nuclear-transcribed mRNA catabolic process
GO:0034623	5.71276192231528e-10	59.8017964071856	0.204691716836205
7	174	cellular macromolecular complex disassembly
GO:0006402	6.70019047479182e-10	58.3865497076023	0.209397273545083
7	178	mRNA catabolic process
GO:0032984	6.9686108506441e-10	58.043023255814	0.210573662722303	7
179	macromolecular complex disassembly
GO:0072594	1.73913476203622e-09	50.5883248730964	0.239983392152792
7	204	establishment of protein localization to organelle
GO:0006401	1.79951730198254e-09	50.3292929292929	0.241159781330012
7	205	RNA catabolic process
GO:0019058	4.13384965419902e-09	44.40625	0.271745899937721	7
231	viral infectious cycle
GO:0022415	9.38945783179033e-09	39.2359683794466	0.305861186077088
7	260	viral reproductive process
GO:0071845	1.72847995398527e-08	35.7758122743682	0.334094526330358
7	284	cellular component disassembly at cellular level
GO:0022411	1.90356726981663e-08	35.2565836298932	0.338800083039236
7	288	cellular component disassembly
GO:0033365	2.32883793308273e-08	26.7029868578256	0.556432080824856
8	473	protein localization to organelle
GO:0006605	2.3674714037337e-08	26.6437768240343	0.557608470002076
8	474	protein targeting
GO:0016032	4.0825539604407e-07	22.0563063063063	0.530551518926026
7	451	viral reproduction
Tissue: Thyroid=>Nerve_Tibial	Type:	asymmetric
SourceGene:	RP11-417O11.6(ENSG00000226458.1)
TargetGeneSet:	NADK	HP1BP3	RPL11	PPP1R8	XKR8	7SK	PPCS	UQCRH
BEND5	RP11	GTF2B	WARS2	S100A6	ACBD6	DHX9	SLC5A6	MRPL33	MORN2
PPM1B	PSME4	NONOP2	TMEM17	NFU1	DCTN1	CCDC115	U6	SETD5	UBE2E1
CCDC72	RPL24	FNDC3B	KCNMB3	RPL39L	SENP5	LETM1	NAA11	MRPS18C	NDUFC1
HADHAP1	FRG1	RPL37	EEF1A1P19	CTD	UQCRQ	TMEM14C	TMEM14B	GABBR1	APOM
C6orf48	COX6A1P2	C6orf226	RPL39P3	LINC00222	MRPS12	SUMO4
PHF14	TOMM7	YAE1D1	MRPS24	GS1	MKLN1	C8orf40	C8orf59	RPL30	COX6C
POLR2K	C9orf46	MTAP	GBA2	FAM27D1	SEC61B	ATP5C1	CDNF	RPS24	C10orf116
AGAP11	IFIT2	IFIT3	ENTPD1	RPLP2	PRKCDBP	RPL27A	LMO2	ALKBH3	CELF1
C11orf10	MTA2	FAU	NADSYN1	ATP5L	RPS25	C12orf57	KLRG1	FGD4
PFDN5	ALKBH2	C12orf51	RPS2P5	DOCK9	EAPP	RPS29	CGRRF1	LGALS3	ERH
RPL7AP6	NEK9	NDUFB1	RPL3P4	HERC2P3	RSL24D1	RPLP1	NDUFB10	DNASE1L2
ERVK13-1	SRRM2	TNFRSF17	USP31	NUDT21	C17orf67	STARD6
RAB27B	PIP5K1C	RPL36	INSR	PIN1	ZNF208	COX6B1	RPS19	ETHE1	SNRPD2
ZNF845	PTPRA	LINC00493	RP3	IFT52	PFDN4	ATP5J	N6AMT1	KCNE1
PLA2G6	EEF1B2P3	PHF8	NDUFA1
GOBPID	Pvalue	OddsRatio	ExpCount	Count	Size	Term
GO:0006614	2.85187993110751e-14	23.8361204013378	0.770188914261989
14	105	SRP-dependent cotranslational protein targeting to membrane
GO:0006613	3.2662520390078e-14	23.5753780718336	0.77752404677877
14	106	cotranslational protein targeting to membrane
GO:0045047	3.2662520390078e-14	23.5753780718336	0.77752404677877
14	106	protein targeting to ER
GO:0072599	3.2662520390078e-14	23.5753780718336	0.77752404677877
14	106	establishment of protein localization to endoplasmic reticulum
GO:0006415	9.97513105924338e-14	25.5681003584229	0.667497059027057
13	91	translational termination
GO:0070972	1.50034564504427e-13	20.8375836120401	0.86554563698014
14	118	protein localization to endoplasmic reticulum
GO:0006414	6.67721192852802e-13	21.65603085554	0.770188914261989	13

105	translational elongation
GO:0000184	3.43323659244544e-12	18.7773382024751	0.872880769496921
13	119	nuclear-transcribed mRNA catabolic process, nonsense-mediated decay
GO:0019080	4.65830556068635e-12	15.781656616947 1.10760501003391	14
151	viral genome expression
GO:0019083	4.65830556068635e-12	15.781656616947 1.10760501003391	14
151	viral transcription
GO:0006612	5.58034789813552e-12	15.552392868314 1.12227527506747	14
153	protein targeting to membrane
GO:0072594	2.20564378957146e-11	12.3460666317809	1.49636703342329
15	204	establishment of protein localization to organelle
GO:0006413	7.89217215308798e-11	14.2862226347954	1.11494014255069
13	152	translational initiation
GO:0043624	8.57319919396345e-11	14.1831797235023	1.12227527506747
13	153	cellular protein complex disassembly
GO:0022904	1.02303691109126e-10	19.425386996904 0.704172721610961	11
96	respiratory electron transport chain
GO:0043241	1.28533505959621e-10	13.689284390063 1.15895093765137	13
158	protein complex disassembly
GO:0006401	2.87805973677657e-10	11.2768040063738	1.50370216594007
14	205	RNA catabolic process
GO:0000956	2.98479905878361e-10	12.7141577060932	1.23963739533596
13	169	nuclear-transcribed mRNA catabolic process
GO:0034623	4.29033958791117e-10	12.3149669404929	1.27631305791987
13	174	cellular macromolecular complex disassembly
GO:0006402	5.68761026823276e-10	12.0130335614207	1.30565358798699
13	178	mRNA catabolic process
GO:0032984	6.09615116983799e-10	11.9398238113745	1.31298872050377
13	179	macromolecular complex disassembly
GO:0022415	6.73842771840518e-10	9.48643193541153	1.90713445436302
15	260	viral reproductive process
GO:0019058	1.38006469738166e-09	9.90743338008415	1.69441561137638
14	231	viral infectious cycle
GO:0022900	3.78745454984021e-09	13.3882755669662	0.982907757248633
11	134	electron transport chain
GO:0045333	6.99654227157378e-09	12.5635998392929	1.04158881738288
11	142	cellular respiration
GO:0016032	2.83744530199301e-08	6.12248226583131	3.30814476506816
17	451	viral reproduction
GO:0071845	1.54326233186212e-07	7.25953259532595	2.08317763476576
13	284	cellular component disassembly at cellular level
GO:0022411	1.81385744427727e-07	7.15190615835777	2.11251816483288
13	288	cellular component disassembly
GO:0033365	1.78833529976849e-06	4.99796055472911	3.46951768043734
15	473	protein localization to organelle
GO:0006605	1.83567570389682e-06	4.98671263377146	3.47685281295412
15	474	protein targeting
GO:0015980	2.52252795364082e-06	6.0800576992427 2.25188568265172	12
307	energy derivation by oxidation of organic compounds
GO:0006119	4.01204607330287e-06	16.4919230769231	0.425437685973289
6	58	oxidative phosphorylation
Tissue: Thyroid=>Nerve_Tibial	Type:	asymmetric
SourceGene:	RP11-757C15.3(ENSG00000254593.1)
TargetGeneSet:	CAPZB	KHDRBS1	PSMB2	SLFNL1	RP1	C1orf123	CCDC76
GDAP2	RP4	S100A6	PFN1P1	MORF4L1P1	PTRHD1	DDX50P1	PAPOLG	NFU1
UBE2E3	ING5	TOP2B	IQCF1	PSMD6	ST3GAL6-AS1	TTC14	MAGEF1	RAC1P2
RAPGEF2	RP11	TRIM27	KCTD20	COX6A1P2	C6orf226	YWHAZP4	NDUFA4
MALSU1	NPC1L1	PILRB	PSMC2	POLR3D	TOX	C8orf59	COX6C	MTAP	SKA2L
ATP5C1	IFIT3	IFIT1	TAF5	BNIP3	C11orf10	NADSYN1	SNORA25	SNORA32
TAF1D	ATP5L	OAF	DAZAP2	IRAK3	CKAP2	RHOJ	NEK9	NDUFB1	SRP14
PLA2G4B	SENP8	NDUFB10	ZNF263	SMYD4	PSMB6	ZFP161	EPG5	ATP5A1	ZNF812
COX6B1	EID2	ZNF28	ZSCAN22	ROMO1	MRPL39	N6AMT1	KCNE1	CHAF1B	HMGB1P10
LGALS1	TCEAL8	NDUFA1

GOBPID	Pvalue	OddsRatio	ExpCount	Count	Size	Term
GO:0022904	2.60977985895883e-10	27.9154196486662	0.411874610753581
9	96	respiratory electron transport chain
GO:0022900	5.17620117142398e-09	19.3775094339623	0.57490831084354
9	134	electron transport chain
GO:0045333	8.62946122562942e-09	18.2017307419492	0.609231195073005
9	142	cellular respiration
GO:0006119	4.74561742215685e-06	23.7272426348891	0.248840910663622
5	58	oxidative phosphorylation
GO:0015980	5.91764803658849e-06	8.02956819045207	1.31714068230572
9	307	energy derivation by oxidation of organic compounds
GO:0006120	1.6323489044894e-05	30.9418103448276	0.154452979032593
4	36	mitochondrial electron transport, NADH to ubiquinone
GO:0006200	1.69040885833644e-05	17.9436090225564	0.321777039651235
5	75	ATP catabolic process
Tissue: Thyroid=>Nerve_Tibial	Type:	asymmetric
SourceGene:	SEPSECS(ENSG00000109618.7)
TargetGeneSet:	CTNNBIP1	HP1BP3	ZDHHC18	PSMB2	EIF2C3	INPP5B	RP3	RP4
UQCRH	MOB3C	CPT2	LRRC40	PIGK	IFI44	ATP5F1	GDAP2	MRPS21	S100A6
S100A4	INTS3	CRABP2	B4GALT3	PIGC	RP11	ACBD6	DENND1B	TIMM17A	PTPN14
KIAA1383	LYST-IT1	FH	CPSF3	DDX1	TTC32	PUM2	FOSL2
DDX50P1	ATL2	ASS1P2	ETAA1	NFU1	CAPG	CNNM3	AMMECR1L	UBE2E3
KCTD18	CFLAR	IMPDH1P10	CASP8	LANCL1	SLC23A3	AGAP1-IT1	SETD5
ZNF385D	EBP	CSRNP1	QARS	RHOA	RBM5	SPCS1	PDHB	PSMD6	ADAMTS9-AS2
UBA3	ARL6IP5	ATG3	ATP6V1A	ZBTB20	KALRN	ARID4A	KPNA4	FAM131A	MAGEF1
SENP5	RELL1	ANKRD17	DNAJB14	PPA2	ANXA5	NUDT6	INTU	OTUD4	NDUFS6	CTD
TXNDC15	SOX9	NDFIP1	PRRC2A	APOM	HNRNPC	DEFA6	FANCE	C6orf226
GTPBP2	MAD2L1BP	VEGFA	GCM1	HACE1	LINC00222	ZNF259P1
FAM54A	NUDT1	YAE1D1	URGCP	HUS1	GS1	ZKSCAN1	GPC2	PSMC2	ST7-AS1
NDUFA5	OPN1SW	GSTK1	CASP2	POLR3D	TNFRSF10A	LOXL2	RNF122	KAT6A
C8orf40	HOOK3	LACTB2	RPS20P21	C8orf59	DECR1	COX6C	NDUFB9	KIAA0196
RP13	HSF1	CD274	LINC00032	HRCT1	C9orf40	LINC00475	STXBP1
ZDHHC12	C9orf169	ATP5C1	CDC123	C10orf102	MARVELD1	PPRC1
DMBT1	NUP98	SMPD1	COMMD9	ALKBH3	NDUFS3	MRPL16	FEN1	C11orf84	RCE1
SNORA32	SNORA8	SNORA18	ATP5L	TBCEL	HIN1L	NDUFA9	PTPN6	SCAF11	H1FNT
FMNL3	ITGA5	ATXN2	RFC5	MPHOSPH8	SACS	RFC3	TUBGCP3	PARP2
LGALS3	CHMP4BP1	MAX	NEK9	ANGEL1	NDUFB1	TMEM85	TMEM62	NARG2	PKM2
LOXL1	COX5A	IMP3	SCAPER	PSMA4	NPRL3	NDUFB10	HCFC1R1	CTA	DCUN1D3
USP31	C16orf54	PYCARD	LPCAT2	NAE1	RANBP10	ZNF821	C16orf46
MIR4720	ZNF469	CYBA	NDEL1	C17orf75	SMARCE1	HEXIM1	HILS1	RNF43	SKA2
DNAH17	USP36	C17orf90	MYADML2	MYL12B	ZFP161	ATP5A1	DYM	ELAC1
TICAM1	C19orf70	HSD11B1L	MAN2B1	NDUFB7	ZNF506	UQCRFS1	COX6B1
WDR62	EID2	KCNN4	ZNF404	PVR	CACNG8	ZIK1	ZSCAN22	ZNF446	PCNA
RALGAPA2	CBFA2T2	MYH7B	ERGIC3	ROMO1	SNHG11	IFT52	GDAP1L1	KRT18P4
SLCO4A1	RPL10P1	MORC3	MRPL40	CRYBB1	MAFF	JOSD1	PRDX3P1	SERHL	CA5BP1
ATP6AP2	ZNF630	PHF8	RP6	CXorf65	CSTF2	TCEAL8	MID2	C1GALT1C1	SSR4
ARHGAP4	VAMP7
GOBPID	Pvalue	OddsRatio	ExpCount	Count	Size	Term
GO:0045333	5.95369350768205e-15	12.9407454895137	1.95543560999239
20	142	cellular respiration
GO:0022904	1.39047326264504e-14	16.7576158019196	1.32198463774133
17	96	respiratory electron transport chain
GO:0022900	3.3191026465857e-13	12.118879786626	1.84527022351394	18
134	electron transport chain
GO:0015980	2.52703603108403e-10	6.09126771731589	4.2275967061103	22
307	energy derivation by oxidation of organic compounds
GO:0006091	5.22707937323311e-09	4.70087694483734	5.89384817659678
24	428	generation of precursor metabolites and energy
GO:0006119	5.74537505179864e-09	15.6569664902998	0.798699051968722
10	58	oxidative phosphorylation
GO:0042773	1.28826821569728e-08	17.7182825484765	0.647221645560861
9	47	ATP synthesis coupled electron transport
GO:0042775	1.28826821569728e-08	17.7182825484765	0.647221645560861

9	47	mitochondrial ATP synthesis coupled electron transport
GO:0006120	2.44259415397913e-08	21.2774869109948	0.495744239153	8
36	mitochondrial electron transport, NADH to ubiquinone
Tissue: Nerve_Tibial=>Thyroid	Type:	asymmetric
SourceGene:	TRMT2A(ENSG00000099899.9)
TargetGeneSet:	PARK7	MYOM3	ZNF642	TDPX2	AKR1A1	MKNK1	PSRC1	VPS45
TARS2	PSMD4	CHTOP	RUSC1-AS1	CCT3	CCL15	DARS2	NEK7	ATF3
C1orf131	FH	C2orf43	SLC4A1AP	PCBP1-AS1	CCDC142	C2orf68
TMEM177	PSMD14	PMS1	WDR12	ATIC	BCS1L	TTLL4	SLC16A14	PSMD1
DTYMK	MLH1	RUVBL1	ASTE1	CEP63	EIF2B5	ALG3	SLC10A7	MARCH1	BRIP1
NDUFS6	SKP2	GTF2H2C	PTCD2	HARS2	RP11	SLC36A1	TTC1	RPL26L1	PRR7
ACOT13	ZKSCAN3	ZNRD1-AS1	VPS52	MNF1	ABCC10	AARS2	MCM3	RP13	PREP
RTN4IP1	ANKRD61	COPS6	SLC12A9	CTA	KLHDC10	CDK5	COPS5	NUDT2	EXOSC3
FAM108B1	FKBP15	PSMB7	TRUB2	SLC2A6	ASAH2B	DNAJC12	SIRT1	SLC25A16
MRPS16	EXOSC1	ENTPD7	COX15	PDE3B	TRMT112	HMBS	APPL2	NDUFA9	COPS7A	USP5
PRKAG1	ATF7	STAT2	NUP37	ANAPC5	BRD7P5	PSMB5	PSMA3	ANGEL1	ZNF280D	MPI
KIAA1024	DET1	TCEB2	ZNF174	ARL6IP1	CDC37P1	EXOC3L1	TUBB3	WRAP53
USP32P3	TMEM199	ALDOC	TEFM	PSMB3	ATP5G1	SNF8	INTS2	C17orf80	FDXR
ANAPC11	SMCHD1	ZNF397	HAUS1	PALM	YIF1B	EID2B	ZNF780A	EXOSC5	ZNF225
ZNF135	TRIB3	C20orf203	ZNF341	RBL1	APP	MIS18A-AS1	ZNF70
ZMAT5	ATF4	ZNF81	NAA10
GOBPID	Pvalue	OddsRatio	ExpCount	Count	Size	Term
GO:0051443	6.97727338314707e-10	19.42674291939	0.635526953152031	10
82	positive regulation of ubiquitin-protein ligase activity
GO:0031145	7.88209797771786e-10	19.1592801504163	0.643277281849007
10	83	anaphase-promoting complex-dependent proteasomal ubiquitin-dependent protein catabolic process
GO:0051351	1.12547124189759e-09	18.3991228070175	0.666528267939935
10	86	positive regulation of ligase activity
GO:0051436	2.140548360725e-09	21.5147304988283	0.519272022697391
9	67	negative regulation of ubiquitin-protein ligase activity involved in mitotic cell cycle
GO:0031397	2.2049634277003e-09	17.0456719273075	0.713030240121791
10	92	negative regulation of protein ubiquitination
GO:0051438	3.36151528468684e-09	16.2482900136799	0.744031554909695
10	96	regulation of ubiquitin-protein ligase activity
GO:0051352	4.12598286118423e-09	19.8002773925104	0.558023666182271
9	72	negative regulation of ligase activity
GO:0051437	4.12598286118423e-09	19.8002773925104	0.558023666182271
9	72	positive regulation of ubiquitin-protein ligase activity involved in mitotic cell cycle
GO:0051444	4.12598286118423e-09	19.8002773925104	0.558023666182271
9	72	negative regulation of ubiquitin-protein ligase activity
GO:0051340	5.02731708972011e-09	15.5217864923747	0.775032869697599
10	100	regulation of ligase activity
GO:0051439	8.5054595135404e-09	18.0709159983115	0.604525638364127
9	78	regulation of ubiquitin-protein ligase activity involved in mitotic cell cycle
GO:0000209	4.64095777366706e-08	10.3022442244224	1.24780292021313
11	161	protein polyubiquitination
GO:0031398	5.10327031114352e-08	11.917211328976	0.98429174451595	10
127	positive regulation of protein ubiquitination
GO:0031396	7.19090893205085e-08	9.83805259506842	1.30205522109197
11	168	regulation of protein ubiquitination
GO:0043161	7.59228779160501e-08	8.61441624365482	1.61981869766798
12	209	proteasomal ubiquitin-dependent protein catabolic process
GO:0010498	1.08915664639264e-07	8.31470588235294	1.67407099854681
12	216	proteasomal protein catabolic process
GO:0042770	1.2873709888606e-07	10.7156862745098	1.08504601757664
10	140	signal transduction in response to DNA damage
GO:0000216	1.67078785870657e-07	15.4582881906826	0.612275967061103
8	79	M/G1 transition of mitotic cell cycle
GO:0006521	2.67078153697813e-07	19.052	0.441768735727631	7	57

regulation of cellular amino acid metabolic process
GO:0010565	3.14558599833867e-07	9.66435185185185	1.1935506193343	10
154	regulation of cellular ketone metabolic process
GO:0007346	6.2001778638116e-07	6.36144200626959	2.34834959518372
13	303	regulation of mitotic cell cycle
GO:0006977	7.42913194607956e-07	16.135593220339	0.511521694000415	7
66	DNA damage response, signal transduction by p53 class mediator resulting in cell cycle arrest
GO:0072413	7.42913194607956e-07	16.135593220339	0.511521694000415	7
66	signal transduction involved in mitotic cell cycle checkpoint
GO:0072431	7.42913194607956e-07	16.135593220339	0.511521694000415	7
66	signal transduction involved in mitotic cell cycle G1/S transition DNA damage checkpoint
GO:0072474	7.42913194607956e-07	16.135593220339	0.511521694000415	7
66	signal transduction involved in mitotic cell cycle G1/S checkpoint
GO:0072401	8.24323360175333e-07	15.8655555555556	0.519272022697391
7	67	signal transduction involved in DNA integrity checkpoint
GO:0072404	8.24323360175333e-07	15.8655555555556	0.519272022697391
7	67	signal transduction involved in G1/S transition checkpoint
GO:0072422	8.24323360175333e-07	15.8655555555556	0.519272022697391
7	67	signal transduction involved in DNA damage checkpoint
GO:0072395	9.13088353527624e-07	15.6043715846995	0.527022351394367
7	68	signal transduction involved in cell cycle checkpoint
GO:0071156	9.40758134296031e-07	6.70809523809524	2.04608677600166
12	264	regulation of cell cycle arrest
GO:0000084	9.48851097160211e-07	9.936	1.03854404539478	9	134
S phase of mitotic cell cycle
GO:0007093	1.29061935829855e-06	9.5504854368932	1.07729568887966	9
139	mitotic cell cycle checkpoint
GO:0033238	1.35260516890109e-06	14.64	0.558023666182271	7	72
regulation of cellular amine metabolic process
GO:0031571	1.48657480361926e-06	14.4171717171717	0.565773994879247
7	73	mitotic cell cycle G1/S transition DNA damage checkpoint
GO:0051320	1.63603628759187e-06	9.26278800173888	1.10829700366757
9	143	S phase
GO:0002479	1.78801043514461e-06	13.9911764705882	0.581274652273199
7	75	antigen processing and presentation of exogenous peptide antigen via MHC class I, TAP-dependent
GO:0048002	1.87208952342203e-06	10.9530769230769	0.837035499273407
8	108	antigen processing and presentation of peptide antigen
GO:0042590	2.54570325230481e-06	13.2101851851852	0.612275967061103
7	79	antigen processing and presentation of exogenous peptide antigen via MHC class I
GO:0000075	2.74253804769764e-06	6.65157944365865	1.87557954466819
11	242	cell cycle checkpoint
GO:0071158	2.7721241533322e-06	13.0283105022831	0.620026295758079
7	80	positive regulation of cell cycle arrest
GO:0031575	3.27562767974461e-06	12.6791111111111	0.635526953152031
7	82	mitotic cell cycle G1/S transition checkpoint
GO:0030330	3.42337532673402e-06	10.0423429781228	0.906788457546191
8	117	DNA damage response, signal transduction by p53 class mediator
GO:0002478	3.55465313198523e-06	12.5114035087719	0.643277281849007
7	83	antigen processing and presentation of exogenous peptide antigen
GO:0031400	3.56660021202779e-06	5.85458333333333	2.3250986090928	12
300	negative regulation of protein modification process
GO:0019884	4.17240953611961e-06	12.1888888888889	0.658777939242959
7	85	antigen processing and presentation of exogenous antigen
GO:0000959	4.36971911079224e-06	48.2424242424242	0.11625493045464
4	15	mitochondrial RNA metabolic process
GO:0070127	4.48128249109726e-06	197.298165137615	0.0387516434848799
3	5	tRNA aminoacylation for mitochondrial protein translation
GO:0071779	4.51332815998386e-06	12.0337552742616	0.666528267939935
7	86	G1/S transition checkpoint

GO:0072331 4.97523235956747e-06 9.51438127090301 0.953290429728046
8 123 signal transduction by p53 class mediator
GO:0007050 5.50619454687212e-06 5.15772897514471 2.85987128918414
13 369 cell cycle arrest
GO:0032269 7.14397009421443e-06 5.02731423827314 2.92962424745692
13 378 negative regulation of cellular protein metabolic process
GO:0051329 7.35017499124349e-06 5.01321962797373 2.9373745761539 13
379 interphase of mitotic cell cycle
GO:0000077 7.50237979458588e-06 8.9640605296343 1.00754273060688 8
130 DNA damage checkpoint
GO:0051325 9.19849866292754e-06 4.90317614649165 2.99937720572971
13 387 interphase
GO:0031570 1.04609790727953e-05 8.54026442307692 1.05404470278873
8 136 DNA integrity checkpoint
GO:0002474 1.15129659673872e-05 10.3239130434783 0.767282541000623
7 99 antigen processing and presentation of peptide antigen via MHC class
I
GO:2000045 1.15129659673872e-05 10.3239130434783 0.767282541000623
7 99 regulation of G1/S transition of mitotic cell cycle
GO:0034660 1.30103427658037e-05 5.59062658090627 2.20884367863816
11 285 ncRNA metabolic process
GO:0000082 1.40440791450296e-05 6.99127859141023 1.44156113763753
9 186 G1/S transition of mitotic cell cycle
GO:0010564 1.88225320034168e-05 4.56419557167687 3.20863608054806
13 414 regulation of cell cycle process
Tissue: Nerve_Tibial=>Thyroid Type: asymmetric
SourceGene: VNN2(ENSG00000112303.9)
TargetGeneSet: C1QC C1QB RP1 CHI3L2 SLAMF8 FCER1G FCGR2A FCGR3A
FCGR2B GALNT14 POLQ P2RY13 PDE6B CFI NDNF CD180 LY86 LST1 AIF1
HLA-DRA HLA-DOA SNX10 TFEC TBXAS1 SPI1 MS4A6A MS4A7 CTSC RAD51AP1
C3AR1 RP11 SLC26A10 BATF SAMD15 CD209 KANK2 ASF1B TYROBP
CYP2S1 CXCL17 SIGLEC10 CTD SIGLEC5 LAIR1 MIR1302-5 RGS19
ITGB2 GPR34 BTK
GOBPID Pvalue OddsRatio ExpCount Count Size Term
GO:0002460 5.66274072390625e-07 17.3561476837339 0.501003390768805
7 181 adaptive immune response based on somatic recombination of immune
receptors built from immunoglobulin superfamily domains
GO:0002250 1.03635928815936e-06 15.7924797715374 0.548058957857588
7 198 adaptive immune response
GO:0002253 1.32280493150263e-06 12.1305841924399 0.82762438585565
8 299 activation of immune response
GO:0050778 5.70180234759249e-06 9.87008426966292 1.00754273060688
8 364 positive regulation of immune response
GO:0002449 6.03478962875416e-06 15.6192911947388 0.462251747283925
6 167 lymphocyte mediated immunity
GO:0019724 1.47667513385958e-05 18.7444298820446 0.315549096948308
5 114 B cell mediated immunity
Tissue: Nerve_Tibial=>Thyroid Type: asymmetric
SourceGene: ZNF214(ENSG00000149050.5)
TargetGeneSet: NECAP2 RP4 HP1BP3 CEP85 HDAC1 SLFNL1 HHLA3 HSD3BP5
INTS3 RP11 TDRD10 RUSC1-AS1 UBQLN4 CCT3 NEK7 KLHL12 RN5S78
PDIA6 C2orf43 SLC5A6 CAD TRMT61B THADA MSH2 PCBP1-AS1 CCT7
HTRA2 SEMA4F C2orf68 POLR1B TMEM177 ATIC BCS1L TTLL4 DTYMK TRANK1
LARS2 USP4 CBX5P1 CCDC14 PLXNA1 ATP2C1 ABCC5 ALG3 TBCCD1 LRCH3
ZNF141 TBC1D19 UGDH-AS1 TBCK CPE LPCAT1 CCT5 PAPD4 ZCCHC9
PCDHGC4 CTB RPL26L1 RPP40 ZSCAN23 C6orf25 AARS2 MCM3 MTO1 C6orf162
RP3 MRPL18 COL28A1 MIOS C7orf42 SGCE ZKSCAN1 SLC12A9 PLOD3 SMO
TAS2R5 POLR3D CHMP7 TTI2 C8orf40 LRRC6 JRK ZNF623 EXOSC4 SNAPC3
FAM205A VCP C9orf103 BICD2 TRIM32 TRAF1 TUBB4B ZNF239 ERCC6
AP3M1 EXOSC1 MORN4 ENTPD7 CYP2E1 POLR2L RP13 TCP11L1 TTC9C STIP1
GPR137 RBM14 RBM4B C2CD3 POLD3 ALG8 EED ATM DPAGT1 TMEM218
RAD52 DCP1B FKBP4 NCAPD2 KIAA1467 REP15 PAN2 STAT2 TIMELESS
NUP37 ALDH2 DYNLL1 RAN ST6GALNAC4P1 ZMYM2 ITM2B PCID2 TTC5 DAD1

PSMB5	AHSA1	ZFYVE19 RPAP1	TP53BP1 UBFD1	ZNF48	RPGRIP1L		ZNF319
C16orf57		C16orf70		TMEM170A		SDR42E1 GALNS	CTNS	KRBA2
C17orf51		SDF2	NUFIP2	CRLF3	TEFM	PIP4K2B TMEM101 GNG8	HEATR6
POLG2	HN1	ZNF397	ZADH2	MAST3	ZNF790	YIF1B	SMG9	FUT2	ZNF841
ZNF808	ZNF137P ZNF17	ZNF132	VN1R108P		ZNF341	NFATC2	APP	SON
TOP3B	IGLV1-50		ZNF70	ZBED4	ALG12	GEMIN8	ZNF81	RPS7P14 TTC3P1
PNMA6C
GOBPID	Pvalue	OddsRatio		ExpCount		Count	Size	Term
Tissue: Skin_Sun_Exposed_Lower_leg=>Thyroid		Type:	asymmetric
SourceGene:	ANKRD13B(ENSG00000198720.7)
TargetGeneSet:	YTHDF2	KCND3	MAN1A2	GJA5	RP11	MNDA	DARC	FCER1G
FCGR3A	CR1	PLEK	STT3B	MYD88	CCRL1	PROS1	PLCXD2-AS1		TLR1
MOCS2	PCDHGA6 F13A1	LY86	LST1	AIF1	CFB	DDO	GPR146	MSR1	LY96
GBGT1	FCN1	NCOA4	SPI1	CLNS1A	CASP4	IQSEC3	ATP5G2	LCP1	U6
MRPL46	PABPN1L GAS8	CD68	VPS25	ABCA7	GNA15	TNFSF14 C3	C19orf38
LRRC25	TYROBP	SIGLEC7 LAIR1	HCK	SPATA25 RUNX1	ITGB2	PKDREJ	TLR8	CYBB
INE1
GOBPID	Pvalue	OddsRatio		ExpCount		Count	Size	Term
GO:0006954	5.5734770359018e-10	11.4728618421053		1.66244550550135
14	462	inflammatory response
GO:0002252	7.12235579674237e-10	12.5344057193923		1.38896962147948
13	386	immune effector process
GO:0050778	4.5365958326799e-09	11.971875	1.30980554978894		12
364	positive regulation of immune response
GO:0002253	7.03563355743526e-09	13.1454098915989		1.07591170161235
11	299	activation of immune response
GO:0031347	1.495622186636e-07	9.5866724738676 1.4501418586949 11	403
regulation of defense response
GO:0030449	3.42675928321822e-07	92.2179487179487		0.0611722372154176
4	17	regulation of complement activation
GO:2000257	3.42675928321822e-07	92.2179487179487		0.0611722372154176
4	17	regulation of protein activation cascade
GO:0006909	6.08708689018233e-07	23.3461956521739		0.309459552972113
6	86	phagocytosis
GO:0071219	8.51283009204456e-07	21.9652173913043		0.327451387447236
6	91	cellular response to molecule of bacterial origin
GO:0031349	8.72426768161208e-07	12.5269196822595		0.770050515535257
8	214	positive regulation of defense response
GO:0002673	1.02676702691067e-06	33.1938020351526		0.183516711646253
5	51	regulation of acute inflammatory response
GO:0071216	1.66876279571164e-06	19.4334239130435		0.367033423292506
6	102	cellular response to biotic stimulus
GO:0050727	2.8628699268569e-06	13.1768518518519		0.629714206629299
7	175	regulation of inflammatory response
GO:0001774	3.63567199059503e-06	146.867346938776		0.0323853020552211
3	9	microglial cell activation
GO:0002920	3.81175880456876e-06	46.0673076923077		0.107951006850737
4	30	regulation of humoral immune response
GO:0007229	4.35105782734731e-06	24.2080378250591		0.24468894886167
5	68	integrin-mediated signaling pathway
GO:0032101	4.56601747025234e-06	8.42606783501033		1.28821534841879
9	358	regulation of response to external stimulus
GO:0006959	4.7428214144455e-06	16.0603448275862		0.439000761192997
6	122	humoral immune response
GO:0070613	5.64636154537336e-06	41.2931034482759		0.118746107535811
4	33	regulation of protein processing
GO:0002281	7.10524156150625e-06	110.135204081633		0.0395820358452702
3	11	macrophage activation involved in immune response
GO:0031663	8.06349374928719e-06	37.4140625	0.129541208220884	4
36	lipopolysaccharide-mediated signaling pathway
GO:0042116	1.00509720627651e-05	35.2083333333333		0.136737942010934
4	38	macrophage activation
GO:0045088	1.10908638510616e-05	10.6129273504274		0.773648882430282

7	215	regulation of innate immune response
GO:0071222	1.38442755006828e-05	18.8048332019963	0.309459552972113
5	86	cellular response to lipopolysaccharide
GO:0006957	1.55554109967332e-05	80.0816326530612	0.0503771365303439
3	14	complement activation, alternative pathway
GO:0002757	1.9736253071039e-05	9.66832358674464	0.845616220330773
7	235	immune response-activating signal transduction
GO:0045089	2.01351993913003e-05	12.3075151166139	0.564943602518857
6	157	positive regulation of innate immune response
GO:0002275	2.1752826216248e-05	28.4861111111111	0.16552487717113
4	46	myeloid cell activation involved in immune response
GO:0002764	2.78970365820371e-05	9.13840479483633	0.892394989966092
7	248	immune response-regulating signaling pathway
GO:0002526	3.64588615020768e-05	15.2117021276596	0.377828523977579
5	105	acute inflammatory response
GO:0001794	3.80124591832027e-05	575.92	0.0107951006850737	2	3
type IIa hypersensitivity
GO:0001796	3.80124591832027e-05	575.92	0.0107951006850737	2	3
regulation of type IIa hypersensitivity
GO:0001798	3.80124591832027e-05	575.92	0.0107951006850737	2	3
positive regulation of type IIa hypersensitivity
GO:0002445	3.80124591832027e-05	575.92	0.0107951006850737	2	3
type II hypersensitivity
GO:0002888	3.80124591832027e-05	575.92	0.0107951006850737	2	3
positive regulation of myeloid leukocyte mediated immunity
GO:0002892	3.80124591832027e-05	575.92	0.0107951006850737	2	3
regulation of type II hypersensitivity
GO:0002894	3.80124591832027e-05	575.92	0.0107951006850737	2	3
positive regulation of type II hypersensitivity
GO:0002274	3.9922535995047e-05	14.9113475177305	0.385025257767629
5	107	myeloid leukocyte activation
GO:0016064	4.76115698302669e-05	14.3446407065436	0.399418725347727
5	111	immunoglobulin mediated immune response
GO:0019724	5.4094204510524e-05	13.9469061097013	0.4102138260328	5
114	B cell mediated immunity
GO:0006968	6.26313193173497e-05	21.34375	0.215902013701474	4
60	cellular defense response
GO:0006956	7.59000707383041e-05	20.2542372881356	0.226697114386548
4	63	complement activation
GO:0002263	7.77010228724405e-05	12.8750450775334	0.442599128088022
5	123	cell activation involved in immune response
GO:0002366	7.77010228724405e-05	12.8750450775334	0.442599128088022
5	123	leukocyte activation involved in immune response
GO:0002697	8.03121432507186e-05	9.5010033444816	0.723271745899938	6
201	regulation of immune effector process
GO:0002758	9.3860821222551e-05	12.3473447500432	0.460590962563144
5	128	innate immune response-activating signal transduction
GO:0002218	0.000101002938279896	12.148085106383	0.467787696353194	5
130	activation of innate immune response
GO:0032640	0.000108416217172059	18.3769230769231	0.248287315756695
4	69	tumor necrosis factor production
GO:0032680	0.000108416217172059	18.3769230769231	0.248287315756695
4	69	regulation of tumor necrosis factor production
GO:0002282	0.000126124456521837	191.946666666667	0.0179918344751228
2	5	microglial cell activation involved in immune response
GO:0002237	0.000131959095993139	8.64587566030069	0.791640716905404
6	220	response to molecule of bacterial origin
GO:0071706	0.000135058853366826	17.3067632850242	0.262680783336793
4	73	tumor necrosis factor superfamily cytokine production
GO:0002885	0.000188750548594402	143.95	0.0215902013701474	2	6
positive regulation of hypersensitivity
GO:0002889	0.000202487136379226	30.3377902885292	0.115147740640786
3	32	regulation of immunoglobulin mediated immune response

GO:0009617	0.000209391343195116	6.53054726368159	1.2306414780984 7
342	response to bacterium
GO:0072376	0.000211917767969644	15.3002136752137	0.295066085392014
4	82	protein activation cascade
GO:0050900	0.000216623847505622	7.86160962072155	0.86720642170092
6	241	leukocyte migration
GO:0006897	0.000220888078517393	6.47120315581854	1.24143657878348
7	345	endocytosis
GO:0002576	0.000222058612382156	15.1054852320675	0.298664452287039
4	83	platelet degranulation
GO:0002712	0.000222171213862611	29.3244897959184	0.118746107535811
3	33	regulation of B cell mediated immunity
GO:0002866	0.000263641729934762	115.152 0.025188568265172	2	7
positive regulation of acute inflammatory response to antigenic stimulus
GO:0002449	0.00032529170250609	9.34922511163646	0.600927271469103
5	167	lymphocyte mediated immunity
GO:0006887	0.000332520106185679	7.23478260869565	0.939173759601412
6	261	exocytosis
GO:0050764	0.000339187854369615	25.1265306122449	0.136737942010934
3	38	regulation of phagocytosis
GO:0002524	0.000350712320370144	95.9533333333333	0.0287869351601965
2	8	hypersensitivity
GO:0002883	0.000350712320370144	95.9533333333333	0.0287869351601965
2	8	regulation of hypersensitivity
GO:0032490	0.000350712320370144	95.9533333333333	0.0287869351601965
2	8	detection of molecule of bacterial origin
GO:0002460	0.00047064011604471	8.59707446808511	0.651304407999446
5	181	adaptive immune response based on somatic recombination of immune receptors built from immunoglobulin superfamily domains
GO:0002863	0.0005610511925052	71.955	0.0359836689502457	2	10
positive regulation of inflammatory response to antigenic stimulus
GO:0002864	0.0005610511925052	71.955	0.0359836689502457	2	10
regulation of acute inflammatory response to antigenic stimulus
GO:0016485	0.000564831236692845	11.6805555555556	0.381426890872604
4	106	protein processing
GO:0002224	0.000672007986874714	11.1308411214953	0.399418725347727
4	111	toll-like receptor signaling pathway
GO:0030595	0.000672007986874714	11.1308411214953	0.399418725347727
4	111	leukocyte chemotaxis
GO:0002283	0.000684150292714912	63.9555555555556	0.0395820358452702
2	11	neutrophil activation involved in immune response
GO:0002438	0.000684150292714912	63.9555555555556	0.0395820358452702
2	11	acute inflammatory response to antigenic stimulus
GO:0002250	0.000707554587946299	7.83044868261493	0.712476645214864
5	198	adaptive immune response
GO:0051604	0.000818774151314756	10.5353982300885	0.421008926717874
4	117	protein maturation
GO:0045351	0.000819090435181635	57.556	0.0431804027402948	2	12
type I interferon biosynthetic process
GO:0071260	0.000857603344496901	17.930029154519 0.187115078541277	3
52	cellular response to mechanical stimulus
GO:0032496	0.000883257672061884	7.43947175348496	0.74846031416511
5	208	response to lipopolysaccharide
GO:0002443	0.000941813331973893	7.32958066515183	0.759255414850183
5	211	leukocyte mediated immunity
GO:0002714	0.000965788107013885	52.32	0.0467787696353194	2	13
positive regulation of B cell mediated immunity
GO:0002891	0.000965788107013885	52.32	0.0467787696353194	2	13
positive regulation of immunoglobulin mediated immune response
GO:0002221	0.00101707625361233	9.91597222222222	0.446197494983046
4	124	pattern recognition receptor signaling pathway
GO:0030168	0.00106755609284447	7.11912886391008	0.780845616220331
5	217	platelet activation

GO:0042108 0.00123937544339854 15.6811224489796 0.212303646806449
3 59 positive regulation of cytokine biosynthetic process
GO:0006911 0.00129412412379021 44.2646153846154 0.0539755034253685
2 15 phagocytosis, engulfment
GO:0050865 0.00138844014391652 5.44265256095988 1.23423984499343
6 343 regulation of cell activation
GO:0060326 0.00143200011832707 9.00694444444444 0.489377897723341
4 136 cell chemotaxis
GO:0030193 0.00149870439702866 14.6316326530612 0.226697114386548
3 63 regulation of blood coagulation
GO:0032675 0.00149870439702866 14.6316326530612 0.226697114386548
3 63 regulation of interleukin-6 production
GO:1900046 0.00149870439702866 14.6316326530612 0.226697114386548
3 63 regulation of hemostasis
GO:0042742 0.00163539136201152 8.67518248175183 0.507369732198464
4 141 defense response to bacterium
GO:0032635 0.00164018012153783 14.1576695194207 0.233893848176597
3 65 interleukin-6 production
GO:0050867 0.00166773326873936 6.41195110909914 0.863608054805896
5 240 positive regulation of cell activation
GO:0010324 0.00166849875966769 38.3573333333333 0.0611722372154176
2 17 membrane invagination
GO:0001817 0.00175072619479345 5.19004803547235 1.29181371531382
6 359 regulation of cytokine production
GO:0050818 0.00186763486545887 13.5014128728414 0.24468894886167
3 68 regulation of coagulation
GO:0002861 0.00187274628793963 35.9575 0.0647706041104422 2 18
regulation of inflammatory response to antigenic stimulus
GO:0051017 0.00194759934947191 13.2959183673469 0.248287315756695
3 69 actin filament bundle assembly
GO:0042119 0.00208825918356867 33.84 0.0683689710054668 2 19
neutrophil activation
GO:0002706 0.00228871577238622 12.5326530612245 0.262680783336793
3 73 regulation of lymphocyte mediated immunity
GO:0042994 0.00231495688787468 31.9577777777778 0.0719673379004913
2 20 cytoplasmic sequestering of transcription factor
GO:0031529 0.00255275925672425 30.2736842105263 0.0755657047955159
2 21 ruffle organization
GO:0045766 0.00276435842778494 11.6930612244898 0.280672617811916
3 78 positive regulation of angiogenesis
GO:0010543 0.00280158655868265 28.758 0.0791640716905404 2 22
regulation of platelet activation
GO:0001816 0.00282647226772585 4.69766402145971 1.4213549235347 6
395 cytokine production
GO:0061041 0.00286623451783342 11.5383995703545 0.284270984706941
3 79 regulation of wound healing
GO:0002822 0.00297040038127584 11.3877551020408 0.287869351601965
3 80 regulation of adaptive immune response based on somatic
recombination of immune receptors built from immunoglobulin superfamily domains
GO:0002886 0.00306135947317336 27.3866666666667 0.082762438585565
2 23 regulation of myeloid leukocyte mediated immunity
GO:0002675 0.00333199908864514 26.14 0.0863608054805896 2 24
positive regulation of acute inflammatory response
GO:0051220 0.00333199908864514 26.14 0.0863608054805896 2 24
cytoplasmic sequestering of protein
GO:0046651 0.00336308482564778 7.05902777777778 0.618919105944225
4 172 lymphocyte proliferation
GO:0032943 0.00350517443624208 6.975 0.626115839734274 4 174
mononuclear cell proliferation
GO:0002819 0.0037650599096333 10.4336734693878 0.313057919867137
3 87 regulation of adaptive immune response
GO:0009595 0.00390556481050803 23.9583333333333 0.0935575392706387
2 26 detection of biotic stimulus

GO:0045730 0.00390556481050803 23.9583333333333 0.0935575392706387
2 26 respiratory burst
GO:0070661 0.00411449750560518 6.65777153558052 0.654902774894471
4 182 leukocyte proliferation
GO:0042035 0.00427199267749964 9.95663265306122 0.327451387447236
3 91 regulation of cytokine biosynthetic process
GO:0002703 0.00440487920668883 9.84407246044485 0.33104975434226
3 92 regulation of leukocyte mediated immunity
GO:0043647 0.00452166054318574 22.1123076923077 0.100754273060688
2 28 inositol phosphate metabolic process
GO:0050766 0.00484546417881378 21.2918518518519 0.104352639955712
2 29 positive regulation of phagocytosis
GO:0070098 0.00517966953394122 20.53 0.107951006850737 2 30
chemokine-mediated signaling pathway
GO:0007015 0.0052514727330191 6.19895287958115 0.70168154452979
4 195 actin filament organization
GO:0000387 0.00552420050949671 19.8206896551724 0.111549373745762
2 31 spliceosomal snRNP assembly
GO:0032755 0.00552420050949671 19.8206896551724 0.111549373745762
2 31 positive regulation of interleukin-6 production
GO:0042089 0.00555871085164821 9.0271407532085 0.359836689502457 3
100 cytokine biosynthetic process
GO:0043900 0.00571447498593333 8.93440233236152 0.363435056397481
3 101 regulation of multi-organism process
GO:0002694 0.00579991613209956 4.74111230810665 1.15507577330289
5 321 regulation of leukocyte activation
GO:0060627 0.00584089060823485 6.00761421319797 0.723271745899938
4 201 regulation of vesicle-mediated transport
GO:0042107 0.00587284324787367 8.84353741496599 0.367033423292506
3 102 cytokine metabolic process
GO:0002437 0.00587898140103788 19.1586666666667 0.115147740640786
2 32 inflammatory response to antigenic stimulus
GO:0032760 0.00587898140103788 19.1586666666667 0.115147740640786
2 32 positive regulation of tumor necrosis factor production
GO:0042992 0.00587898140103788 19.1586666666667 0.115147740640786
2 32 negative regulation of transcription factor import into nucleus
GO:0008154 0.00705528491353845 8.25548710050058 0.392221991557678
3 109 actin polymerization or depolymerization
GO:0007249 0.00726627631974132 5.63055555555556 0.770050515535257
4 214 I-kappaB kinase/NF-kappaB cascade
GO:0050830 0.00739910375149829 16.9 0.129541208220884 2 36
defense response to Gram-positive bacterium
GO:0043299 0.0082187248167956 15.9588888888889 0.136737942010934
2 38 leukocyte degranulation
GO:0007596 0.00864094515181872 3.68690349946978 1.79198671372223
6 498 blood coagulation
GO:0048661 0.00864316817551764 15.5264864864865 0.140336308905958
2 39 positive regulation of smooth muscle cell proliferation
GO:0009612 0.00876971944583186 7.60461401952085 0.424607293612899
3 118 response to mechanical stimulus
GO:0030100 0.00876971944583186 7.60461401952085 0.424607293612899
3 118 regulation of endocytosis
GO:0071496 0.0094543803305764 5.20264317180617 0.831222752750675
4 231 cellular response to external stimulus
GO:0002696 0.00973707704665 5.15647743813683 0.838419486540724
4 233 positive regulation of leukocyte activation
GO:0010951 0.00981881947496151 7.28520408163265 0.442599128088022
3 123 negative regulation of endopeptidase activity
GO:0002824 0.00997415936298272 14.359 0.151131409591032 2 42
positive regulation of adaptive immune response based on somatic recombination of immune receptors built from immunoglobulin superfamily domains
Tissue: Skin_Sun_Exposed_Lower_leg=>Thyroid Type: asymmetric
SourceGene: C2orf68(ENSG00000168887.6)

TargetGeneSet: MRPL20 RBMXL1 HAX1 ADAR ISG20L2 ATF6 RP11 DHX9
MIR4426 QARS HNRNPU RNASEH1 DDX1 POTEE XRCC5 TRIP12 CIDECP FANCD2
CTNNB1 SPCS1 POLR2B NUP155 SKIV2L2 ANKRA2 CTD AP3S1 DDX46 IK
LARP1 C6orf47 FTSJD2 UNC5CL RPL24P4 LYRM2 RPL23P8 POLR2J3 ASH2L JPH1
ST13P6 SMU1 SMC3 NUP160 STX5 DLAT PRKAG1 BAZ2A CHST11 RPS11P5
ATP11A TOX4 DCAF11 DLST HMGN2P5 ARHGAP11B AQR HMGN2P46
IDH3A C15orf38 SV2B FUS UBE2MP1 CIRH1A SF3B3 TSR1 TP53
VAMP2 TMEM199 CTB PIGS FAM134C PSME3 DHX8 KPNB1 ARMC7 MRPS7
CEP76 PIK3C3 CTIF ZNF407 C19orf38 EMR2 UBE2M RPS10L RP4
DDX27 TPTE SON APOL6 TOMM22 ADSL PPPDE2 TCF20 USP9X RP6
SLC35A2 NONO EIF4A1P2

GOBPID	Pvalue	OddsRatio	ExpCount	Count	Size	Term
GO:0006397	8.48042610623201e-11	10.4408802685565	1.98795931077434	16	399	mRNA processing
GO:0008380	5.45111296043078e-10	10.9909608302645	1.60930039443637	14	323	RNA splicing
GO:0000377	6.31346020257997e-08	11.7933155969405	1.01640024911771	10	204	RNA splicing, via transesterification reactions with bulged adenosine as nucleophile
GO:0000398	6.31346020257997e-08	11.7933155969405	1.01640024911771	10	204	nuclear mRNA splicing, via spliceosome
GO:0000375	7.92964531613603e-08	11.4929486140379	1.04131201992942	10	209	RNA splicing, via transesterification reactions
GO:0070934	0.00024244194982661	136.914285714286	0.0249117708117085	2	5	CRD-mediated mRNA stabilization
GO:0016556	0.000505853434055779	82.1371428571429	0.0348764791363919	2	7	mRNA modification
GO:0006734	0.00067229672626930l	68.4428571428571	0.0398588332987337	2	8	NADH metabolic process
GO:0009060	0.0011843179228636	15.9866220735786	0.209258874818352	3	42	aerobic respiration
GO:0006084	0.0020806586287254l	12.9809782608696	0.254100062279427	3	51	acetyl-CoA metabolic process
GO:0006281	0.0029163886675157	4.01067921440262	1.90824164417687	7	383	DNA repair
GO:0006103	0.0039628285592237	24.1378151260504	0.0946647290844924	2	19	2-oxoglutarate metabolic process
GO:0031023	0.0041524956649226	10.0399719495091	0.323853020552211	3	65	microtubule organizing center organization
GO:0022613	0.0052554513493684	4.80291723202171	1.12102968652688	5	225	ribonucleoprotein complex biogenesis
GO:0006091	0.0053507924790887	3.57047323223095	2.13244758148225	7	428	generation of precursor metabolites and energy
GO:0071843	0.006755172069383	4.51109835438194	1.19078264479967	5	239	cellular component biogenesis at cellular level
GO:0019674	0.0068196098334614	17.8335403726708	0.124558854058543	2	25	NAD metabolic process
GO:0043489	0.0068196098334614	17.8335403726708	0.124558854058543	2	25	RNA stabilization
GO:0048255	0.0068196098334614	17.8335403726708	0.124558854058543	2	25	mRNA stabilization
GO:0006099	0.0073642455264579	17.0892857142857	0.129541208220884	2	26	tricarboxylic acid cycle
GO:0009267	0.0079274031794264	7.87011557512383	0.40855304131202	3	82	cellular response to starvation
GO:0046356	0.0079279179831569	16.4045714285714	0.134523562383226	2	27	acetyl-CoA catabolic process
GO:0006370	0.0097311853662280	14.6438775510204	0.149470624870251	2	30	7-methylguanosine mRNA capping

Tissue: Skin_Sun_Exposed_Lower_leg=>Thyroid Type: cluster
SourceGene: C2orf68(ENSG00000168887.6)
TargetGeneSet: MRPL20 RBMXL1 HAX1 ADAR ISG20L2 ATF6 RP11 DHX9
MIR4426 QARS HNRNPU RNASEH1 DDX1 POTEE XRCC5 TRIP12 CIDECP FANCD2

CTNNB1 SPCS1 POLR2B NUP155 SKIV2L2 ANKRA2 CTD AP3S1 DDX46 IK
LARP1 C6orf47 FTSJD2 UNC5CL RPL24P4 LYRM2 RPL23P8 POLR2J3 ASH2L JPH1
ST13P6 SMU1 SMC3 NUP160 STX5 DLAT PRKAG1 BAZ2A CHST11 RPS11P5
ATP11A TOX4 DCAF11 DLST HMGN2P5 ARHGAP11B AQR HMGN2P46
IDH3A C15orf38 SV2B FUS UBE2MP1 CIRH1A SF3B3 TSR1 TP53
VAMP2 TMEM199 CTB PIGS FAM134C PSME3 DHX8 KPNB1 ARMC7 MRPS7
CEP76 PIK3C3 CTIF ZNF407 C19orf38 EMR2 UBE2M RPS10L RP4
DDX27 TPTE SON APOL6 TOMM22 ADSL PPPDE2 TCF20 USP9X RP6
SLC35A2 NONO EIF4A1P2

GOBPID	Pvalue	OddsRatio	ExpCount	Count	Size	Term
GO:0006397	8.48042610623201e-11	10.4408802685565	1.98795931077434	16	399	mRNA processing
GO:0008380	5.45111296043078e-10	10.9909608302645	1.60930039443637	14	323	RNA splicing
GO:0000377	6.31346020257997e-08	11.7933155969405	1.01640024911771	10	204	RNA splicing, via transesterification reactions with bulged adenosine as nucleophile
GO:0000398	6.31346020257997e-08	11.7933155969405	1.01640024911771	10	204	nuclear mRNA splicing, via spliceosome
GO:0000375	7.92964531613603e-08	11.4929486140379	1.04131201992942	10	209	RNA splicing, via transesterification reactions
GO:0070934	0.000242444194982661	136.914285714286	0.0249117708117085	2	5	CRD-mediated mRNA stabilization
GO:0016556	0.000505853434055779	82.1371428571429	0.0348764791363919	2	7	mRNA modification
GO:0006734	0.000672296726269301	68.4428571428571	0.0398588332987337	2	8	NADH metabolic process
GO:0009060	0.00118431792286366	15.9866220735786	0.209258874818352	3	42	aerobic respiration
GO:0006084	0.00208065862872541	12.9809782608696	0.254100062279427	3	51	acetyl-CoA metabolic process
GO:0006281	0.00291638866751573	4.01067921440262	1.90824164417687	7	383	DNA repair
GO:0006103	0.00396282855922372	24.1378151260504	0.0946647290844924	2	19	2-oxoglutarate metabolic process
GO:0031023	0.00415249566492265	10.0399719495091	0.323853020552211	3	65	microtubule organizing center organization
GO:0022613	0.00525545134936841	4.80291723202171	1.12102968652688	5	225	ribonucleoprotein complex biogenesis
GO:0006091	0.00535079247908876	3.57047323223095	2.13244758148225	7	428	generation of precursor metabolites and energy
GO:0071843	0.006755172069383	4.51109835438194	1.19078264479967	5	239	cellular component biogenesis at cellular level
GO:0019674	0.00681960983346149	17.8335403726708	0.124558854058543	2	25	NAD metabolic process
GO:0043489	0.00681960983346149	17.8335403726708	0.124558854058543	2	25	RNA stabilization
GO:0048255	0.00681960983346149	17.8335403726708	0.124558854058543	2	25	mRNA stabilization
GO:0006099	0.00736424552645795	17.0892857142857	0.129541208220884	2	26	tricarboxylic acid cycle
GO:0009267	0.00792740317942642	7.87011557512383	0.40855304131202	3	82	cellular response to starvation
GO:0046356	0.00792791798315694	16.4045714285714	0.134523562383226	2	27	acetyl-CoA catabolic process
GO:0006370	0.00973118536622807	14.6438775510204	0.149470624870251	2	30	7-methylguanosine mRNA capping

Tissue: Skin_Sun_Exposed_Lower_leg=>Thyroid Type: asymmetric
SourceGene: CBX8(ENSG00000141570.6)
TargetGeneSet: RP11 ACTA1 RAB10 CHAC2 XIRP2 MYL1 SUMF1 RFC1
PPP1R3A FAM90A11P MYPN ANKRD2 SLN MYF6 MYBPC1 C12orf73
ACA64 MYH2 TCAP PPP1R27 CKM TNNT1 KRTAP11-1 PVALB FAM45B
GOBPID Pvalue OddsRatio ExpCount Count Size Term

GO:0030049	6.82931318946151e-12	201.384615384615	0.0512767282541001	6	39	muscle filament sliding
GO:0033275	6.82931318946151e-12	201.384615384615	0.0512767282541001	6	39	actin-myosin filament sliding
GO:0070252	1.09550346103916e-11	184.564102564103	0.0552210919659539	6	42	actin-mediated cell contraction
GO:0030048	9.28670025034424e-11	125.216255442671	0.0775724863331257	6	59	actin filament-based movement
GO:0006936	3.21718692437347e-10	44.7099567099567	0.314234309044357	8	239	muscle contraction
GO:0003012	7.52608626815069e-10	39.9548978153629	0.349733582451041	8	266	muscle system process
GO:0030029	3.38449798682131e-08	24.0274442538593	0.567988374506954	8	432	actin filament-based process
GO:0003009	1.84255948873129e-06	168.9375	0.0249809701750744	3	19	skeletal muscle contraction
GO:0006941	2.44499268606635e-06	53.9380281690141	0.0986090927963463	4	75	striated muscle contraction
GO:0050879	4.91524401869724e-06	117.464673913043	0.0341844855027334	3	26	multicellular organismal movement
GO:0050881	4.91524401869724e-06	117.464673913043	0.0341844855027334	3	26	musculoskeletal movement
GO:0003010	9.81137925112146e-06	848.823529411765	0.00525915161580513	2	4	voluntary skeletal muscle contraction
GO:0014721	9.81137925112146e-06	848.823529411765	0.00525915161580513	2	4	twitch skeletal muscle contraction
GO:0061061	9.83140057717353e-06	16.066806642489	0.537748252716075	6	409	muscle structure development
GO:0030239	1.1237674975672e-05	87.1028225806452	0.0447027887343436	3	34	myofibril assembly
GO:0030240	1.63394737183147e-05	565.843137254902	0.00657393951975642	2	5	skeletal muscle thin filament assembly
GO:0014866	2.44899890051702e-05	424.352941176471	0.0078887274237077	2	6	skeletal myofibril assembly
GO:0031032	2.64057118044168e-05	64.2410714285714	0.0591654556778078	3	45	actomyosin structure organization
GO:0007517	3.58101005777371e-05	16.9391179290508	0.398380734897239	5	303	muscle organ development
GO:0042692	0.000251743550383548	15.7688888888889	0.320808248564113	4	244	muscle cell differentiation
GO:0055002	0.000350809358083889	25.8317307692308	0.140682305722787	3	107	striated muscle cell development
GO:0045214	0.000372442933368624	84.7764705882353	0.0289253338869282	2	22	sarcomere organization
GO:0010927	0.000527800161591498	22.3625	0.161718912186008	3	123	cellular component assembly involved in morphogenesis
GO:0055001	0.000606537671670912	21.2886904761905	0.169607639609716	3	129	muscle cell development
GO:0051146	0.0013265864772265	16.1137048192771	0.222199155767767	3	169	striated muscle cell differentiation
GO:0060048	0.00143215215855968	41.2941176470588	0.0565358798699052	2	43	cardiac muscle contraction
GO:0030036	0.00145388941752792	9.72952380952381	0.511452494637049	4	389	actin cytoskeleton organization
GO:0060415	0.00193222176422744	35.2549019607843	0.0657393951975642	2	50	muscle tissue morphogenesis
GO:0001756	0.00216845350920103	33.1741637831603	0.069683758909418	2	53	somitogenesis
GO:0048644	0.00241769650587859	31.3246187363834	0.0736281226212719	2	56	muscle organ morphogenesis
GO:0061053	0.00295483586424565	28.1803921568627	0.0815168500449796	2	62	somite development
GO:0030241	0.00393945224456549	400.833333333333	0.00394436371185385			

1	3	skeletal muscle myosin thick filament assembly
GO:0031034	0.00393945224456549	400.833333333333	0.00394436371185385
1	3	myosin filament assembly
GO:0042396	0.00393945224456549	400.833333333333	0.00394436371185385
1	3	phosphagen biosynthetic process
GO:0048769	0.00393945224456549	400.833333333333	0.00394436371185385
1	3	sarcomerogenesis
GO:0071688	0.00393945224456549	400.833333333333	0.00394436371185385
1	3	striated muscle myosin thick filament assembly
GO:0014706	0.00460462018587363	10.2603764478764	0.344474430835236
3	262	striated muscle tissue development
GO:0035282	0.00463406501533718	22.2229102167183	0.1025534565082 2
78	segmentation
GO:0048747	0.00475071458473899	21.9327731092437	0.103868244412151
2	79	muscle fiber development
GO:0060537	0.00516431657442708	9.83472222222222	0.3589370977787 3
273	muscle tissue development
GO:0031033	0.00524933253300364	267.203703703704	0.00525915161580513
1	4	myosin filament organization
GO:0001778	0.00655758079782431	200.388888888889	0.00657393951975642
1	5	plasma membrane repair
GO:0000244	0.00786419895958823	160.3	0.0078887274237077	1	6
assembly of spliceosomal tri-snRNP
GO:0043353	0.00786419895958823	160.3	0.0078887274237077	1	6
enucleate erythrocyte differentiation
GO:0048739	0.00916918893672691	133.574074074074	0.00920351532765899
1	7	cardiac muscle fiber development
GO:0003015	0.00999174999097359	14.7760577915377	0.152515396858349
2	116	heart process
GO:0060047	0.00999174999097359	14.7760577915377	0.152515396858349
2	116	heart contraction
Tissue: Skin_Sun_Exposed_Lower_leg=>Thyroid	Type:	cluster
SourceGene:	CORO7(ENSG00000262246.1)
TargetGeneSet:	RP11	MFI2	TMSB4XP1	DPH2	KCND3	RBBP5	RRP15
HEATR1	DDIT3	POLR1B	DDX18	HNRNPKP2	USP37	GIGYF2	BRPF1	MYNN
OCIAD1-AS1	ZNF131	RIOK1	PAK1IP1	RPL7L1	CDC5L	CCNC	ZNF680	BAZ1B
TRIM4	TGS1	TG	FXN	EXOSC2	NGB	BMS1	SPTY2D1	NAT10	RPS3
PA2G4	RPL41	UTP20	SART3	NUFIP1	SUPT16H	RSL1D1	SRCAP	ZNF778	NEURL4	DHX8
NOL11	C17orf101	HMG20B	ZNF121	SYCE2	ZNF576	ZNF749	ZSCAN22	CSRP2BP	AHCY
TFIP11	RP4	GAPDHP1	ATP6AP2	TAF1	UTP14A	RAP2C
GOBPID	Pvalue	OddsRatio	ExpCount	Count	Size	Term
GO:0071843	8.31956408197553e-10	16.2771512113617	0.876548335755311
11	239	cellular component biogenesis at cellular level
GO:0022613	7.44588986654496e-09	15.3412655489454	0.825202408137845
10	225	ribonucleoprotein complex biogenesis
GO:0042254	1.11473650659671e-06	15.2774035517453	0.546467372500173
7	149	ribosome biogenesis
GO:0006364	3.82252748338727e-05	15.0452441077441	0.381426890872604
5	104	rRNA processing
GO:0016072	5.00360401007735e-05	14.1795634920635	0.403432288422946
5	110	rRNA metabolic process
GO:0016570	0.000369369644579804	7.08035043804756	0.9572347934399 6
261	histone modification
GO:0022618	0.000401071785835457	12.8342677730433	0.34841879454709
4	95	ribonucleoprotein complex assembly
GO:0016569	0.000416853690713839	6.9146490584495 0.979240190990243	6
267	covalent chromatin modification
GO:0071826	0.000487377959773485	12.1615646258503	0.366756625839042
4	100	ribonucleoprotein complex subunit organization
GO:0034660	0.000588487198610036	6.4603065660032 1.04525638364127	6
285	ncRNA metabolic process
GO:0016573	0.000651857061619797	11.2197802197802	0.396097155906166
4	108	histone acetylation

GO:0018393	0.000698443637537373	11.0065460146323	0.403432288422946
4	110	internal peptidyl-lysine acetylation
GO:0018394	0.000772680266855757	10.7013667852462	0.414434987198118
4	113	peptidyl-lysine acetylation
GO:0016568	0.000798470522773041	5.16578725200507	1.53671026226559
7	419	chromatin modification
GO:0034470	0.000808439723585181	7.58707264957265	0.733513251678085
5	200	ncRNA processing
GO:0006475	0.000825168423407011	10.5070785070785	0.421770119714899
4	115	internal protein amino acid acetylation
GO:0000377	0.00088366756234403	7.43247487437186	0.748183516711646
5	204	RNA splicing, via transesterification reactions with bulged adenosine as nucleophile
GO:0000398	0.00088366756234403	7.43247487437186	0.748183516711646
5	204	nuclear mRNA splicing, via spliceosome
GO:0000375	0.000984912130114098	7.24775326797386	0.766521348003598
5	209	RNA splicing, via transesterification reactions
GO:0006473	0.00122944119370842	9.39697169190257	0.469448481073974
4	128	protein acetylation
GO:0006337	0.001532475065685	40.2913165266106	0.0586810601342468
2	16	nucleosome disassembly
GO:0031498	0.001532475065685	40.2913165266106	0.0586810601342468
2	16	chromatin disassembly
GO:0032986	0.001532475065685	40.2913165266106	0.0586810601342468
2	16	protein-DNA complex disassembly
GO:0018205	0.00175565072825971	8.49754208252644	0.51712684243305
4	141	peptidyl-lysine modification
GO:0043543	0.00189624101207078	8.31370262390671	0.528129541208221
4	144	protein acylation
GO:0034623	0.00375623821690118	6.83217286914766	0.638156528959934
4	174	cellular macromolecular complex disassembly
GO:0032984	0.00415486758017016	6.63463556851312	0.656494360251886
4	179	macromolecular complex disassembly
GO:0000387	0.00573319951906942	19.4306964164976	0.113694554010103
2	31	spliceosomal snRNP assembly
GO:0008380	0.00645433658226166	4.61215932914046	1.18462390146011
5	323	RNA splicing
GO:0006354	0.00725196574203883	8.16742857142857	0.396097155906166
3	108	DNA-dependent transcription, elongation
Tissue: Skin_Sun_Exposed_Lower_leg=>Thyroid	Type:	asymmetric
SourceGene:	DYNLL1(ENSG00000088986.6)
TargetGeneSet:	RP4	SELL	TNNI1	XIRP2	GFRA3	RP1	MOG	LMOD2	NRAP	CSRP3	ANGPTL5	MYBPC1	MYL2	MYH7	CTD	MMP2	MYH1	TCAP	CKM	PVALB	RPSAP8	FAM127C
GOBPID	Pvalue	OddsRatio	ExpCount	Count	Size	Term
GO:0030049	6.63055833692767e-10	176.470588235294	0.0458791779115632
5	39	muscle filament sliding
GO:0033275	6.63055833692767e-10	176.470588235294	0.0458791779115632
5	39	actin-myosin filament sliding
GO:0070252	9.77614220520049e-10	162.128378378378	0.0494083454432219
5	42	actin-mediated cell contraction
GO:0030048	5.68604279134456e-09	110.956790123457	0.0694069614559546
5	59	actin filament-based movement
GO:0048738	1.6766067820844e-07	54.2575757575758	0.135284755380251
5	115	cardiac muscle tissue development
GO:0055008	1.89547834193003e-07	108.015009380863	0.0529375129748806
4	45	cardiac muscle tissue morphogenesis
GO:0006936	2.04235339756834e-07	33.244635193133 0.281157013355477	6
239	muscle contraction
GO:0060415	2.91927812134081e-07	96.2408026755853	0.0588194588609785
4	50	muscle tissue morphogenesis
GO:0003012	3.83791962639148e-07	29.7356643356643	0.312919521140405
6	266	muscle system process

GO:0048644 4.63566068315803e-07 85.1005917159763 0.0658777939242959
4 56 muscle organ morphogenesis
GO:0006941 1.51316902074374e-06 62.2448537378115 0.0882291882914677
4 75 striated muscle contraction
GO:0007507 1.66962570045951e-06 22.8863636363636 0.402325098609093
6 342 heart development
GO:0030029 6.44751475380701e-06 17.9359795134443 0.508200124558854
6 432 actin filament-based process
GO:0055010 7.21868328547381e-06 102.885714285714 0.0388208428482458
3 33 ventricular cardiac muscle tissue morphogenesis
GO:0003015 8.65413335701117e-06 39.3461538461538 0.13646114455747
4 116 heart process
GO:0060047 8.65413335701117e-06 39.3461538461538 0.13646114455747
4 116 heart contraction
GO:0003229 9.4261519298055e-06 93.512987012987 0.0423500103799045 3
36 ventricular cardiac muscle tissue development
GO:0014706 9.76150598372339e-06 22.9847600518807 0.308213964431527
5 262 striated muscle tissue development
GO:0060537 1.1916725202437e-05 22.0242537313433 0.321154245380943
5 273 muscle tissue development
GO:0007517 1.97254232303307e-05 19.7651006711409 0.35644592069753
5 303 muscle organ development
GO:0003007 2.85819821851923e-05 28.7199597787833 0.184693100823472
4 157 heart morphogenesis
GO:0003208 3.05467779594253e-05 61.6457142857143 0.0623486263926372
3 53 cardiac ventricle morphogenesis
GO:0009582 6.75297557433346e-05 46.6493506493507 0.0811708532281503
3 69 detection of abiotic stimulus
GO:0007603 7.11967467526906e-05 213.703703703704 0.0129402809494153
2 11 phototransduction, visible light
GO:0061061 8.27676600816249e-05 14.4698844884488 0.481143173482804
5 409 muscle structure development
GO:0003231 9.01483592865506e-05 42.1555772994129 0.0894055774686873
3 76 cardiac ventricle development
GO:0007512 0.000100830265848504 174.824242424242 0.0152930593038544
2 13 adult heart development
GO:0003206 0.00010901190307642 39.4395604395604 0.0952875233547851
3 81 cardiac chamber morphogenesis
GO:0055003 0.000117553927184637 160.244444444444 0.016469448481074
2 14 cardiac myofibril assembly
GO:0009581 0.000130283379331782 37.0507745266781 0.101169469240883
3 86 detection of external stimulus
GO:0003205 0.000164358884169156 34.152380952381 0.10940419348142 3
93 cardiac chamber development
GO:0003009 0.000220135062408993 113.074509803922 0.0223513943671718
2 19 skeletal muscle contraction
GO:0009584 0.00024442533295198 106.785185185185 0.0235277835443914
2 20 detection of visible light
GO:0008015 0.000363425273176549 14.5459223051196 0.35644592069753
4 303 blood circulation
GO:0003013 0.000368003727025375 14.4964102564103 0.357622309874749
4 304 circulatory system process
GO:0050879 0.000416364046374626 80.0555555555556 0.0305861186077088
2 26 multicellular organismal movement
GO:0050881 0.000416364046374626 80.0555555555556 0.0305861186077088
2 26 musculoskeletal movement
GO:0055006 0.000555747467346677 68.6 0.0352916753165871 2 30
cardiac cell development
GO:0055013 0.000555747467346677 68.6 0.0352916753165871 2 30
cardiac muscle cell development
GO:0071482 0.000632803923949525 64.0177777777778 0.0376444536710262
2 32 cellular response to light stimulus
GO:0007602 0.000673164262056883 61.9483870967742 0.0388208428482458

2	33	phototransduction
GO:0030239	0.000714742559025276	60.0083333333333	0.0399972320254654
2	34	myofibril assembly
GO:0006942	0.000893183771772659	53.3259259259259	0.0447027887343436
2	38	regulation of striated muscle contraction
GO:0009583	0.000989645204184707	50.5122807017544	0.0470555670887828
2	40	detection of light stimulus
GO:0048729	0.00107264463754218	10.7953846153846	0.475261227596706
4	404	tissue morphogenesis
GO:0044057	0.00120661265491822	10.4458930899609	0.490554286900561
4	417	regulation of system process
GO:0051606	0.00124909903274545	16.6873536299766	0.21880838696284
3	186	detection of stimulus
GO:0031032	0.00125175363671356	44.6232558139535	0.0529375129748806
2	45	actomyosin structure organization
GO:0055007	0.00193242469401933	35.5061728395062	0.0658777939242959
2	56	cardiac muscle cell differentiation
GO:0071478	0.00207135493513362	34.2333333333333	0.068230572278735
2	58	cellular response to radiation
GO:0016056	0.00352526115515339	451	0.00352916753165871	1	3
rhodopsin mediated signaling pathway
GO:0030241	0.00352526115515339	451	0.00352916753165871	1	3
skeletal muscle myosin thick filament assembly
GO:0031034	0.00352526115515339	451	0.00352916753165871	1	3
myosin filament assembly
GO:0034124	0.00352526115515339	451	0.00352916753165871	1	3
regulation of MyD88-dependent toll-like receptor signaling pathway
GO:0035845	0.00352526115515339	451	0.00352916753165871	1	3
photoreceptor cell outer segment organization
GO:0042396	0.00352526115515339	451	0.00352916753165871	1	3
phosphagen biosynthetic process
GO:0048769	0.00352526115515339	451	0.00352916753165871	1	3
sarcomerogenesis
GO:0071688	0.00352526115515339	451	0.00352916753165871	1	3
striated muscle myosin thick filament assembly
GO:0035051	0.00352717337339985	25.8738738738739	0.0894055774686873
2	76	cardiac cell differentiation
GO:0003010	0.00469774665905387	300.645833333333	0.00470555670887828
1	4	voluntary skeletal muscle contraction
GO:0014721	0.00469774665905387	300.645833333333	0.00470555670887828
1	4	twitch skeletal muscle contraction
GO:0031033	0.00469774665905387	300.645833333333	0.00470555670887828
1	4	myosin filament organization
GO:0006937	0.00480315999875744	21.9877394636015	0.104698636772542
2	89	regulation of muscle contraction
GO:0060041	0.00534277161637755	20.7855072463768	0.11058058265864
2	94	retina development in camera-type eye
GO:0008016	0.00556610375852272	20.3404255319149	0.112933361013079
2	96	regulation of heart contraction
GO:0001955	0.00586893363952024	225.46875	0.00588194588609785	1
5	blood vessel maturation
GO:0030240	0.00586893363952024	225.46875	0.00588194588609785	1
5	skeletal muscle thin filament assembly
GO:0090257	0.00674614320171622	18.3717948717949	0.124697252785274
2	106	regulation of muscle system process
GO:0055002	0.00686990117416381	18.1955555555556	0.125873641962494
2	107	striated muscle cell development
GO:0001957	0.00703882344487339	180.3625	0.00705833506331742	1
6	intramembranous ossification
GO:0009586	0.00703882344487339	180.3625	0.00705833506331742	1
6	rhodopsin mediated phototransduction
GO:0014866	0.00703882344487339	180.3625	0.00705833506331742	1
6	skeletal myofibril assembly

GO:0036072 0.00703882344487339 180.3625 0.00705833506331742 1
6 direct ossification
GO:0043353 0.00703882344487339 180.3625 0.00705833506331742 1
6 enucleate erythrocyte differentiation
GO:0046548 0.00703882344487339 180.3625 0.00705833506331742 1
6 retinal rod cell development
GO:0000302 0.00750419816453199 17.3624242424242 0.131755587848592
2 112 response to reactive oxygen species
GO:0048739 0.00820741742212772 150.291666666667 0.00823472424053699
1 7 cardiac muscle fiber development
GO:0010927 0.00898948094198444 15.7719008264463 0.144695868798007
2 123 cellular component assembly involved in morphogenesis
GO:0042670 0.00937471691699199 128.8125 0.00941111341775656 1
8 retinal cone cell differentiation
GO:0046549 0.00937471691699199 128.8125 0.00941111341775656 1
8 retinal cone cell development
GO:0055001 0.00985084345743121 15.0204724409449 0.151754203861324
2 129 muscle cell development
GO:0030036 0.00994051330598178 7.79866765358993 0.457615389938413
3 389 actin cytoskeleton organization
Tissue: Skin_Sun_Exposed_Lower_leg=>Thyroid Type: asymmetric
SourceGene: ENSG00000237411.1
TargetGeneSet: RP4 NSRP1P1 TNNI1 ACTA1 LBH XIRP2 TLK1 RP11
SH3PXD2B GCNT6 TRDN NRAP RNU2-2 MYBPC1 MYH7 CTD COX16 MYH2
MYH1 CKM TNNT1 PRMT2 PVALB
GOBPID Pvalue OddsRatio ExpCount Count Size Term
GO:0030049 6.82931318946151e-12 201.384615384615 0.0512767282541001
6 39 muscle filament sliding
GO:0033275 6.82931318946151e-12 201.384615384615 0.0512767282541001
6 39 actin-myosin filament sliding
GO:0070252 1.09550346103916e-11 184.564102564103 0.0552210919659539
6 42 actin-mediated cell contraction
GO:0030048 9.28670025034424e-11 125.216255442671 0.0775724863331257
6 59 actin filament-based movement
GO:0006936 3.21718692437347e-10 44.7099567099567 0.314234309044357
8 239 muscle contraction
GO:0003012 7.52608626815069e-10 39.9548978153629 0.349733582451041
8 266 muscle system process
GO:0030029 7.50211871241557e-07 19.2252941176471 0.567988374506954
7 432 actin filament-based process
GO:0003010 9.81137925112146e-06 848.823529411765 0.00525915161580513
2 4 voluntary skeletal muscle contraction
GO:0014721 9.81137925112146e-06 848.823529411765 0.00525915161580513
2 4 twitch skeletal muscle contraction
GO:0006941 0.00012256166736417 37.3958333333333 0.0986090927963463
3 75 striated muscle contraction
GO:0003009 0.000276353744574814 99.757785467128 0.0249809701750744 2
19 skeletal muscle contraction
GO:0050879 0.0005223593393242 70.6274509803922 0.0341844855027334
2 26 multicellular organismal movement
GO:0050881 0.0005223593393242 70.6274509803922 0.0341844855027334
2 26 musculoskeletal movement
GO:0055010 0.000843990329769391 54.6527514231499 0.0433880008303924
2 33 ventricular cardiac muscle tissue morphogenesis
GO:0007507 0.000901575672369832 11.1195266272189 0.449657463151339
4 342 heart development
GO:0003229 0.00100466965926752 49.8200692041522 0.0473323645422462
2 36 ventricular cardiac muscle tissue development
GO:0055008 0.00156767622957267 39.3679890560876 0.0591654556778078
2 45 cardiac muscle tissue morphogenesis
GO:0060415 0.00193222176422744 35.2549019607843 0.0657393951975642
2 50 muscle tissue morphogenesis
GO:0003208 0.0021684535092010З 33.1741637831603 0.069683758909418

2	53	cardiac ventricle morphogenesis
GO:0048644	0.00241769650587859	31.3246187363834	0.0736281226212719
2	56	muscle organ morphogenesis
GO:0016056	0.00393945224456549	400.833333333333	0.00394436371185385
1	3	rhodopsin mediated signaling pathway
GO:0042396	0.00393945224456549	400.833333333333	0.00394436371185385
1	3	phosphagen biosynthetic process
GO:0003231	0.00440484670302872	22.8267090620032	0.0999238807002976
2	76	cardiac ventricle development
GO:0014706	0.00460462018587363	10.2603764478764	0.344474430835236
3	262	striated muscle tissue development
GO:0003206	0.00498807761938354	21.3745346239762	0.106497820220054
2	81	cardiac chamber morphogenesis
GO:0060537	0.00516431657442708	9.83472222222222	0.3589370977787 3
273	muscle tissue development
GO:0001672	0.00524933253300364	267.203703703704	0.00525915161580513
1	4	regulation of chromatin assembly or disassembly
GO:0006937	0.00599122113800982	19.3982420554429	0.117016123451664
2	89	regulation of muscle contraction
GO:0003205	0.00652463296512004	18.5404007756949	0.122275275067469
2	93	cardiac chamber development
GO:0001778	0.00655758079782431	200.388888888889	0.00657393951975642
1	5	plasma membrane repair
GO:0030240	0.00655758079782431	200.388888888889	0.00657393951975642
1	5	skeletal muscle thin filament assembly
GO:0060315	0.00655758079782431	200.388888888889	0.00657393951975642
1	5	negative regulation of ryanodine-sensitive calcium-release channel activity
GO:0071800	0.00655758079782431	200.388888888889	0.00657393951975642
1	5	podosome assembly
GO:0007517	0.00689463365614857	8.8325	0.398380734897239	3	303
muscle organ development
GO:0000244	0.00786419895958823	160.3	0.0078887274237077	1	6
assembly of spliceosomal tri-snRNP
GO:0009586	0.00786419895958823	160.3	0.0078887274237077	1	6
rhodopsin mediated phototransduction
GO:0014866	0.00786419895958823	160.3	0.0078887274237077	1	6
skeletal myofibril assembly
GO:0043353	0.00786419895958823	160.3	0.0078887274237077	1	6
enucleate erythrocyte differentiation
GO:0090257	0.00840177241788033	16.2081447963801	0.139367517818836
2	106	regulation of muscle system process
GO:1901020	0.00916918893672691	133.574074074074	0.00920351532765899
1	7	negative regulation of calcium ion transmembrane transporter activity
GO:0000302	0.00934076689784703	15.3176470588235	0.147256245242544
2	112	response to reactive oxygen species
GO:0048738	0.00982714459386267	14.9078604893285	0.151200608954398
2	115	cardiac muscle tissue development
Tissue: Skin_Sun_Exposed_Lower_leg=>Thyroid	Type:	asymmetric
SourceGene:	MADD(ENSG00000110514.13)
TargetGeneSet:	TNFRSF14	RPL11	RP4	RPL5	RP11	RPL32	PLCL2-AS1
RPL37	EEF1A1P19	BTF3	RP1	RPL10A	SAMD3	TOMM7	EIF4BP6	RPL30
BBIP1	FAU	RPS25	KRT4	ATP5G2	RPL41	RPL14P1	NAP1L1	RPL7AP6	UBE2I
TP53I13	TBC1D3	RPL17	CASC3	STAT5B	CCDC102B	POLR2I	ISOC2	ZNF8
RPS21	EIF3D
GOBPID	Pvalue	OddsRatio	ExpCount	Count	Size	Term
GO:0000184	1.50047049188817e-18	100.345794392523	0.230572278735036
12	119	nuclear-transcribed mRNA catabolic process, nonsense-mediated decay
GO:0006415	6.50644801972714e-18	116.009558823529	0.176319977856204
11	91	translational termination
GO:0019080	2.865849493093e-17	77.0719424460432	0.292574908310844
12	151	viral genome expression

GO:0019083 2.865849493093e-17 77.0719424460432 0.292574908310844
12 151 viral transcription
GO:0006413 3.10828516241801e-17 76.5160714285714 0.294512490485088
12 152 translational initiation
GO:0006414 3.37370061889609e-17 98.6351689612015 0.20344612829562
11 105 translational elongation
GO:0006614 3.37370061889609e-17 98.6351689612015 0.20344612829562
11 105 SRP-dependent cotranslational protein targeting to membrane
GO:0006613 3.76025564035194e-17 97.590092879257 0.205383710469864 11
106 cotranslational protein targeting to membrane
GO:0045047 3.76025564035194e-17 97.590092879257 0.205383710469864 11
106 protein targeting to ER
GO:0072599 3.76025564035194e-17 97.590092879257 0.205383710469864 11
106 establishment of protein localization to endoplasmic reticulum
GO:0000956 1.14116022391456e-16 68.1496815286624 0.327451387447236
12 169 nuclear-transcribed mRNA catabolic process
GO:0070972 1.27749015390823e-16 86.5728422210006 0.228634696560792
11 118 protein localization to endoplasmic reticulum
GO:0006402 2.15121092431852e-16 64.414156626506 0.344889627015431 12
178 mRNA catabolic process
GO:0072594 1.12958423475647e-15 55.58984375 0.395266763545775 12
204 establishment of protein localization to organelle
GO:0006401 1.19857635529966e-15 55.2979274611399 0.397204345720019
12 205 RNA catabolic process
GO:0006612 2.39677087220734e-15 65.0749792874896 0.296450072659332
11 153 protein targeting to membrane
GO:0043624 2.39677087220734e-15 65.0749792874896 0.296450072659332
11 153 cellular protein complex disassembly
GO:0043241 3.436052031534e-15 62.8395358143257 0.306137983530551
11 158 protein complex disassembly
GO:0019058 5.0767250836969e-15 48.6438356164384 0.447581482250363
12 231 viral infectious cycle
GO:0034623 1.0088421326474e-14 56.6077228437387 0.337139298318455
11 174 cellular macromolecular complex disassembly
GO:0032984 1.38290377756684e-14 54.9037114845938 0.346827209189675
11 179 macromolecular complex disassembly
GO:0022415 2.10447537193709e-14 42.8679435483871 0.503771365303439
12 260 viral reproductive process
GO:0071845 2.22343453693889e-12 33.5380305968541 0.550273337485295
11 284 cellular component disassembly at cellular level
GO:0022411 2.58900225122003e-12 33.0443830961988 0.558023666182271
11 288 cellular component disassembly
GO:0016032 1.42814313194492e-11 23.8906605922551 0.873849560584043
12 451 viral reproduction
GO:0033365 2.48859198781389e-11 22.7147505422993 0.916476368417411
12 473 protein localization to organelle
GO:0006605 2.55054753927791e-11 22.663961038961 0.918413950591655 12
474 protein targeting
GO:0022613 3.76983542061772e-06 17.6886674968867 0.435955989204899
6 225 ribonucleoprotein complex biogenesis
GO:0071843 5.33749530884311e-06 16.6094420600858 0.463082139644315
6 239 cellular component biogenesis at cellular level
GO:0042254 0.000183306887678686 16.4114942528736 0.288699743962356
4 149 ribosome biogenesis
GO:0007603 0.00019698305497146 123.196581196581 0.021313403916684
2 11 phototransduction, visible light
GO:0042273 0.000236096351242935 110.869230769231 0.023250986090928
2 12 ribosomal large subunit biogenesis
GO:0042274 0.00054339147293453 69.2644230769231 0.0348764791363919
2 18 ribosomal small subunit biogenesis
GO:0009584 0.000673184465959876 61.5598290598291 0.0387516434848799
2 20 detection of visible light
GO:0022618 0.000800324303079238 18.6926086956522 0.18407030655318

3	95	ribonucleoprotein complex assembly
GO:0071826	0.000928927504268237	17.7228865979381	0.1937582174244 3
100	ribonucleoprotein complex subunit organization
GO:0006364	0.00104074135129846	17.0162376237624	0.201508546121376
3	104	rRNA processing
GO:0016072	0.00122389145234099	16.0553271028037	0.21313403916684
3	110	rRNA metabolic process
GO:0071482	0.0017322920285817	36.9051282051282	0.0620026295758079
2	32	cellular response to light stimulus
GO:0007602	0.0018418472247155	35.712158808933 0.0639402117500519	2
33	phototransduction
GO:0009583	0.0026982209398674	29.1194331983806	0.0775032869697599
2	40	detection of light stimulus
GO:0071478	0.00559649222465406	19.7348901098901	0.112379766106152
2	58	cellular response to radiation
GO:0016056	0.00580189185011537	267.055555555556	0.00581274652273199
1	3	rhodopsin mediated signaling pathway
GO:0031125	0.00580189185011537	267.055555555556	0.00581274652273199
1	3	rRNA 3'-end processing
GO:0035845	0.00580189185011537	267.055555555556	0.00581274652273199
1	3	photoreceptor cell outer segment organization
GO:0046544	0.00580189185011537	267.055555555556	0.00581274652273199
1	3	development of secondary male sexual characteristics
GO:0034470	0.00663066539227953	8.66558375634518	0.387516434848799
3	200	ncRNA processing
GO:0000255	0.00772863236978594	178.024691358025	0.00775032869697599
1	4	allantoin metabolic process
GO:0000447	0.00772863236978594	178.024691358025	0.00775032869697599
1	4	endonucleolytic cleavage in ITS1 to separate SSU-rRNA from 5.8S rRNA and LSU-rRNA from tricistronic rRNA transcript (SSU-rRNA, 5.8S rRNA, LSU-rRNA)
GO:0000469	0.00772863236978594	178.024691358025	0.00775032869697599
1	4	cleavage involved in rRNA processing
GO:0000478	0.00772863236978594	178.024691358025	0.00775032869697599
1	4	endonucleolytic cleavage involved in rRNA processing
GO:0000479	0.00772863236978594	178.024691358025	0.00775032869697599
1	4	endonucleolytic cleavage of tricistronic rRNA transcript (SSU-rRNA, 5.8S rRNA, LSU-rRNA)
GO:0006549	0.00772863236978594	178.024691358025	0.00775032869697599
1	4	isoleucine metabolic process
GO:0046449	0.00772863236978594	178.024691358025	0.00775032869697599
1	4	creatinine metabolic process
GO:0046642	0.00772863236978594	178.024691358025	0.00775032869697599
1	4	negative regulation of alpha-beta T cell proliferation
GO:0070669	0.00772863236978594	178.024691358025	0.00775032869697599
1	4	response to interleukin-2
GO:0009582	0.00783920174745108	16.4822043628014	0.133693170022836
2	69	detection of abiotic stimulus
GO:0008298	0.00965177200387202	133.509259259259	0.00968791087121998
1	5	intracellular mRNA localization
GO:0072338	0.00965177200387202	133.509259259259	0.00968791087121998
1	5	cellular lactam metabolic process
Tissue: Skin_Sun_Exposed_Lower_leg=>Thyroid	Type:	asymmetric
SourceGene:	PTDSS2(ENSG00000174915.7)
TargetGeneSet:	TNNI1	C1orf186	RP11	ACTA1	FAHD2A	MYL1	ANXA5
ERCC4	OR1F12P LY6G6E	STX7	SLC26A4 LZTS2	TNNI2	AGBL2	FADS3	MYBPC1
STK24-AS1	DDX18P1 SLC25A29	SLC24A1 CTD	MYLPF	MYH2	DBF4B
C19orf24	CKM	TNNT1	BPIFA2	SALL4
GOBPID	Pvalue	OddsRatio	ExpCount	Count	Size	Term
GO:0030049	4.45877744890921e-14	242.290865384615	0.0539755034253685
7	39	muscle filament sliding
GO:0033275	4.45877744890921e-14	242.290865384615	0.0539755034253685
7	39	actin-myosin filament sliding
GO:0070252	7.80225094085748e-14	221.476923076923	0.0581274652273199

7	42	actin-mediated cell contraction	
GO:0030048	9.73465781956882e-13	148.894970414201	0.0816552487717113
7	59	actin filament-based movement	
GO:0006936	2.00080286424127e-08	32.9552387267904	0.330772956888797
7	239	muscle contraction	
GO:0003012	4.18005688822706e-08	29.4636174636175	0.368140613106359
7	266	muscle system process	
GO:0030029	1.12458856263389e-06	17.7451583710407	0.597882499481005
7	432	actin filament-based process	
GO:0006941	3.04424438900589e-06	50.5633802816901	0.103799045048786
4	75	striated muscle contraction	
GO:0003009	0.0003068190587918	94.2091503267974	0.0262957580790257
2	19	skeletal muscle contraction	
GO:0050879	0.000579757517178671	66.6990740740741	0.0359836689502457
2	26	multicellular organismal movement	
GO:0050881	0.000579757517178671	66.6990740740741	0.0359836689502457
2	26	musculoskeletal movement	
GO:0007517	0.00070401627756025	11.8160535117057	0.419348141997094
4	303	muscle organ development	
GO:0061061	0.00213735663531172	8.65802469135802	0.56605079233271
4	409	muscle structure development	
GO:0006295	0.00414650474202016	379.710526315789	0.00415196180195142
1	3	nucleotide-excision repair, DNA incision, 3'-to lesion	
GO:0006296	0.00414650474202016	379.710526315789	0.00415196180195142
1	3	nucleotide-excision repair, DNA incision, 5'-to lesion	
GO:0010834	0.00414650474202016	379.710526315789	0.00415196180195142
1	3	telomere maintenance via telomere shortening	
GO:0042396	0.00414650474202016	379.710526315789	0.00415196180195142
1	3	phosphagen biosynthetic process	
GO:0003231	0.0048767138278522	21.5570570570571	0.105183032316103
2	76	cardiac ventricle development	
GO:0014706	0.00534538779923922	9.65614353849648	0.362604664037091
3	262	striated muscle tissue development	
GO:0000710	0.00552503948074912	253.122807017544	0.00553594906926856
1	4	meiotic mismatch repair	
GO:0003010	0.00552503948074912	253.122807017544	0.00553594906926856
1	4	voluntary skeletal muscle contraction	
GO:0009642	0.00552503948074912	253.122807017544	0.00553594906926856
1	4	response to light intensity	
GO:0014721	0.00552503948074912	253.122807017544	0.00553594906926856
1	4	twitch skeletal muscle contraction	
GO:0015705	0.00552503948074912	253.122807017544	0.00553594906926856
1	4	iodide transport	
GO:0060537	0.00599176048850142	9.25555555555556	0.377828523977579
3	273	muscle tissue development	
GO:0006937	0.0066303979558913	18.3192848020434	0.123174866791226
2	89	regulation of muscle contraction	
GO:0001778	0.00690176123684949	189.828947368421	0.0069199363365857
1	5	plasma membrane repair	
GO:0030240	0.00690176123684949	189.828947368421	0.0069199363365857
1	5	skeletal muscle thin filament assembly	
GO:0003205	0.00721940175550811	17.5091575091575	0.128710815860494
2	93	cardiac chamber development	
GO:0000244	0.00827667226933348	151.852631578947	0.00830392360390284
1	6	assembly of spliceosomal tri-snRNP	
GO:0014866	0.00827667226933348	151.852631578947	0.00830392360390284
1	6	skeletal myofibril assembly	
GO:0090257	0.00929093127083658	15.3066239316239	0.146702650335617
2	106	regulation of muscle system process	
GO:0000712	0.00964977483455431	126.535087719298	0.00968791087121998
1	7	resolution of meiotic recombination intermediates	
GO:0033683	0.00964977483455431	126.535087719298	0.00968791087121998
1	7	nucleotide-excision repair, DNA incision	

GO:0051307	0.00964977483455431	126.535087719298	0.00968791087121998
1	7	meiotic chromosome separation
Tissue: Skin_Sun_Exposed_Lower_leg=>Thyroid	Type:	asymmetric
SourceGene:	RP11-1084E5.1(ENSG00000253796.1)
TargetGeneSet:	TNFRSF18	PLA2G2D GBP4	HSD3BP3 FCRL5	FCRL3	FCRLA	CD84
SLAMF7	LAX1	GPR113	IGKJ4	IGKJ2	IGKJ1	IGKV1-5 IGKV1-12	IGKV3-15
IGKV6-21	IGKV2-30	CXCR2P1 TIGIT	PARP15	IGJ	HAVCR1	LTB	RP11
ZC3H12D TAGAP	TRGC1	TRGV7	BLK	PTK2B	PNOC	PAX5	MS4A1	POU2AF1
CRTAM	CLECL1	BIN2	GS1	FLT3	CYSLTR2 TRAV1-2 TRAV27	IGHG2	IGHM
IGHJ5	IGHJ3	IGHV6-1 IGHV2-5 IGHV3-11	IGHV4-31	IGHV3-33
IGHV3-66	IGHV1-69	IGHV3-74	ARRDC4	TNFRSF17	ITGAL
GPR114	SNORD68 P2RX5	TBC1D27 TNFRSF13B	RAB37	CD79A	NLRP7	GTSF1L	ZBP1
RSPH1	IGLV6-57	IGLV1-51	IGLV9-49	IGLV1-36	IGLV3-25
IGLV2-23	IGLV2-8 IGLL5	IGLC1	IL2RB	MTCP1	RP4	CSAG1
GOBPID	Pvalue	OddsRatio	ExpCount	Count	Size	Term
GO:0006959	1.30915259897009e-06	20.5359195402299	0.354577537886651
6	122	humoral immune response
GO:0046649	3.31811191594273e-06	8.9954974271012 1.25845962217148	9
433	lymphocyte activation
GO:0042113	4.88940263576622e-06	16.1700680272109	0.444675108988997
6	153	B cell activation
GO:0002449	8.09150784582112e-06	14.7494824016563	0.485364334648121
6	167	lymphocyte mediated immunity
GO:0002252	1.2838448008193e-05	8.73389355742297	1.12186007888727
8	386	immune effector process
GO:0002443	3.05321979791158e-05	11.5479674796748	0.613244758148225
6	211	leukocyte mediated immunity
GO:0042267	7.10970809712616e-05	44.2584615384615	0.0813784513182479
3	28	natural killer cell mediated cytotoxicity
GO:0050778	7.66078716559701e-05	7.87226890756302	1.05791986713722
7	364	positive regulation of immune response
GO:0002228	7.914037128239e-05	42.5532544378698	0.0842848245796139
3	29	natural killer cell mediated immunity
GO:0051250	0.00010048412649917	18.8539473684211	0.244135353954744
4	84	negative regulation of lymphocyte activation
GO:0033209	0.000152468956192974	33.5104895104895	0.104629437409176
3	36	tumor necrosis factor-mediated signaling pathway
GO:0051249	0.000155125843090672	8.50300842358604	0.822503632966577
6	283	regulation of lymphocyte activation
GO:0002695	0.00017558057522173	16.2037351443124	0.281918206352502
4	97	negative regulation of leukocyte activation
GO:0050866	0.000238202804191413	14.911933298593 0.305169192443429	4
105	negative regulation of cell activation
GO:0002694	0.000306434352846846	7.45714285714286	0.932945816898485
6	321	regulation of leukocyte activation
GO:0001909	0.000338648297043203	25.1136363636364	0.136599543284202
3	47	leukocyte mediated cytotoxicity
GO:0006958	0.00040687161867526	23.505728314239 0.1453186630683 3	50
complement activation, classical pathway
GO:0050865	0.000436394388543592	6.95944609297725	0.996886028648536
6	343	regulation of cell activation
GO:0006956	0.000803009247930836	18.3961538461538	0.183101515466058
3	63	complement activation
GO:0045954	0.000845353877431116	55.3692307692308	0.0435955989204899
2	15	positive regulation of natural killer cell mediated cytotoxicity
GO:0002455	0.000879712823769607	17.8002481389578	0.18891426198879
3	65	humoral immune response mediated by circulating immunoglobulin
GO:0002717	0.000964339849869892	51.4107142857143	0.0465019721818559
2	16	positive regulation of natural killer cell mediated immunity
GO:0030183	0.00100324756452195	16.9751479289941	0.197633381772888
3	68	B cell differentiation
GO:0001906	0.00104673776673337	16.7167832167832	0.200539755034254
3	69	cell killing

GO:0002683	0.00116752255233183	9.61740890688259	0.465019721818559
4	160	negative regulation of immune system process
GO:0071356	0.00133281622931245	15.3173076923077	0.21797799460245
3	75	cellular response to tumor necrosis factor
GO:0042269	0.00136661034477667	42.3294117647059	0.0552210919659539
2	19	regulation of natural killer cell mediated cytotoxicity
GO:0002715	0.00151566211163264	39.975	0.0581274652273199	2	20
regulation of natural killer cell mediated immunity
GO:0046651	0.00152453015269653	8.92293233082707	0.499896200954951
4	172	lymphocyte proliferation
GO:0032943	0.00159065866290391	8.81671826625387	0.505708947477683
4	174	mononuclear cell proliferation
GO:0002253	0.00166625680359298	6.48786541643685	0.869005605148433
5	299	activation of immune response
GO:0072376	0.00172353849898644	13.9532619279455	0.238322607432012
3	82	protein activation cascade
GO:0070661	0.00187524961800535	8.41573033707865	0.528959933568611
4	182	leukocyte proliferation
GO:0043552	0.00200710821962668	34.2571428571429	0.0668465850114179
2	23	positive regulation of phosphatidylinositol 3-kinase activity
GO:0001912	0.00218554691840735	32.6977272727273	0.0697529582727839
2	24	positive regulation of leukocyte mediated cytotoxicity
GO:0043551	0.00218554691840735	32.6977272727273	0.0697529582727839
2	24	regulation of phosphatidylinositol 3-kinase activity
GO:0050850	0.00218554691840735	32.6977272727273	0.0697529582727839
2	24	positive regulation of calcium-mediated signaling
GO:0090218	0.00218554691840735	32.6977272727273	0.0697529582727839
2	24	positive regulation of lipid kinase activity
GO:0007218	0.00224985752505742	12.6631299734748	0.26157359352294
3	90	neuropeptide signaling pathway
GO:0050869	0.00256410862520172	29.96875	0.0755657047955159	2
26	negative regulation of B cell activation
GO:0034612	0.00262425819952185	11.9707357859532	0.27610545982977
3	95	response to tumor necrosis factor
GO:0019221	0.00295183107394798	5.65999034749035	0.991073282125804
5	341	cytokine-mediated signaling pathway
GO:0031343	0.00318554101184146	26.6333333333333	0.0842848245796139
2	29	positive regulation of cell killing
GO:0043550	0.00318554101184146	26.6333333333333	0.0842848245796139
2	29	regulation of lipid kinase activity
GO:0050853	0.00318554101184146	26.6333333333333	0.0842848245796139
2	29	B cell receptor signaling pathway
GO:0001910	0.00340681136275634	25.6803571428571	0.0871911978409799
2	30	regulation of leukocyte mediated cytotoxicity
GO:0016064	0.00407240445033978	10.1858974358974	0.322607432011626
3	111	immunoglobulin mediated immune response
GO:0050848	0.00411243099306514	23.1903225806452	0.0959103176250778
2	33	regulation of calcium-mediated signaling
GO:0019724	0.00438827834974687	9.90852390852391	0.331326551795723
3	114	B cell mediated immunity
GO:0032655	0.00461728111549336	21.7818181818182	0.10172306414781
2	35	regulation of interleukin-12 production
GO:0031341	0.00487991712486397	21.1397058823529	0.104629437409176
2	36	regulation of cell killing
GO:0032615	0.00514930741245981	20.5342857142857	0.107535810670542
2	37	interleukin-12 production
GO:0071345	0.00650112841672883	4.66083078152044	1.19451941042142
5	411	cellular response to cytokine stimulus
GO:0002705	0.00722085666126351	17.1035714285714	0.127880423500104
2	44	positive regulation of leukocyte mediated immunity
GO:0002708	0.00722085666126351	17.1035714285714	0.127880423500104
2	44	positive regulation of lymphocyte mediated immunity
GO:0010522	0.00722085666126351	17.1035714285714	0.127880423500104

2 44 regulation of calcium ion transport into cytosol
GO:0051924 0.00837453187443606 7.78396072013093 0.418517749636703
3 144 regulation of calcium ion transport
GO:0002291 0.00869440324222703 175.69512195122 0.00871911978409799 1
3 T cell activation via T cell receptor contact with antigen bound to MHC molecule on antigen presenting cell
GO:0016056 0.00869440324222703 175.69512195122 0.00871911978409799 1
3 rhodopsin mediated signaling pathway
GO:0031585 0.00869440324222703 175.69512195122 0.00871911978409799 1
3 regulation of inositol 1,4,5-trisphosphate-sensitive calcium-release channel activity
GO:0031987 0.00869440324222703 175.69512195122 0.00871911978409799 1
3 locomotion involved in locomotory behavior
GO:0038110 0.00869440324222703 175.69512195122 0.00871911978409799 1
3 interleukin-2-mediated signaling pathway
GO:0051643 0.00869440324222703 175.69512195122 0.00871911978409799 1
3 endoplasmic reticulum localization
GO:0001776 0.00889458699134167 15.2787234042553 0.142412289806934
2 49 leukocyte homeostasis
GO:0046427 0.00960811169746031 14.6530612244898 0.148225036329666
2 51 positive regulation of JAK-STAT cascade
Tissue: Skin_Sun_Exposed_Lower_leg=>Thyroid Type: cluster
SourceGene: RP11-1084E5.1(ENSG00000253796.1)
TargetGeneSet: TNFRSF18 PLA2G2D GBP4 HSD3BP3 FCRL5 FCRL3 FCRLA CD84 SLAMF7 LAX1 GPR113 IGKJ4 IGKJ2 IGKJ1 IGKV1-5 IGKV1-12 IGKV3-15 IGKV6-21 IGKV2-30 CXCR2P1 TIGIT PARP15 IGJ HAVCR1 LTB RP11 ZC3H12D TAGAP TRGC1 TRGV7 BLK PTK2B PNOC PAX5 MS4A1 POU2AF1 CRTAM CLECL1 BIN2 GS1 FLT3 CYSLTR2 TRAV1-2 TRAV27 IGHG2 IGHM IGHJ5 IGHJ3 IGHV6-1 IGHV2-5 IGHV3-11 IGHV4-31 IGHV3-33 IGHV3-66 IGHV1-69 IGHV3-74 ARRDC4 TNFRSF17 ITGAL GPR114 SNORD68 P2RX5 TBC1D27 TNFRSF13B RAB37 CD79A NLRP7 GTSF1L ZBP1 RSPH1 IGLV6-57 IGLV1-51 IGLV9-49 IGLV1-36 IGLV3-25 IGLV2-23 IGLV2-8 IGLL5 IGLC1 IL2RB MTCP1 RP4 CSAG1
GOBPID Pvalue OddsRatio ExpCount Count Size Term
GO:0006959 1.30915259897009e-06 20.5359195402299 0.354577537886651
6 122 humoral immune response
GO:0046649 3.31811191594273e-06 8.9954974271012 1.25845962217148 9
433 lymphocyte activation
GO:0042113 4.88940263576622e-06 16.1700680272109 0.444675108988997
6 153 B cell activation
GO:0002449 8.09150784582112e-06 14.7494824016563 0.485364334648121
6 167 lymphocyte mediated immunity
GO:0002252 1.2838448008193e-05 8.73389355742297 1.12186007888727
8 386 immune effector process
GO:0002443 3.05321979791158e-05 11.5479674796748 0.613244758148225
6 211 leukocyte mediated immunity
GO:0042267 7.10970809712616e-05 44.2584615384615 0.0813784513182479
3 28 natural killer cell mediated cytotoxicity
GO:0050778 7.66078716559701e-05 7.87226890756302 1.05791986713722
7 364 positive regulation of immune response
GO:0002228 7.914037128239e-05 42.5532544378698 0.0842848245796139
3 29 natural killer cell mediated immunity
GO:0051250 0.00010048412649917 18.8539473684211 0.244135353954744
4 84 negative regulation of lymphocyte activation
GO:0033209 0.000152468956192974 33.5104895104895 0.104629437409176
3 36 tumor necrosis factor-mediated signaling pathway
GO:0051249 0.000155125843090672 8.50300842358604 0.822503632966577
6 283 regulation of lymphocyte activation
GO:0002695 0.00017558057522173 16.2037351443124 0.281918206352502
4 97 negative regulation of leukocyte activation
GO:0050866 0.000238202804191413 14.911933298593 0.305169192443429 4
105 negative regulation of cell activation
GO:0002694 0.000306434352846846 7.45714285714286 0.932945816898485

6 321 regulation of leukocyte activation
GO:0001909 0.000338648297043203 25.1136363636364 0.136599543284202
3 47 leukocyte mediated cytotoxicity
GO:0006958 0.00040687161867526 23.505728314239 0.1453186630683 3 50
complement activation, classical pathway
GO:0050865 0.000436394388543592 6.95944609297725 0.996886028648536
6 343 regulation of cell activation
GO:0006956 0.000803009247930836 18.3961538461538 0.18310151546605 8
3 63 complement activation
GO:0045954 0.000845353877431116 55.3692307692308 0.0435955989204899
2 15 positive regulation of natural killer cell mediated cytotoxicity
GO:0002455 0.000879712823769607 17.8002481389578 0.18891426198879
3 65 humoral immune response mediated by circulating immunoglobulin
GO:0002717 0.000964339849869892 51.4107142857143 0.0465019721818559
2 16 positive regulation of natural killer cell mediated immunity
GO:0030183 0.00100324756452195 16.9751479289941 0.197633381772888
3 68 B cell differentiation
GO:0001906 0.00104673776673337 16.7167832167832 0.200539755034254
3 69 cell killing
GO:0002683 0.00116752255233183 9.61740890688259 0.465019721818559
4 160 negative regulation of immune system process
GO:0071356 0.00133281622931245 15.3173076923077 0.21797799460245
3 75 cellular response to tumor necrosis factor
GO:0042269 0.00136661034477667 42.3294117647059 0.0552210919659539
2 19 regulation of natural killer cell mediated cytotoxicity
GO:0002715 0.0015156621116326 4 39.975 0.0581274652273199 2 20
regulation of natural killer cell mediated immunity
GO:0046651 0.00152453015269653 8.92293233082707 0.499896200954951
4 172 lymphocyte proliferation
GO:0032943 0.00159065866290391 8.81671826625387 0.505708947477683
4 174 mononuclear cell proliferation
GO:0002253 0.00166625680359298 6.48786541643685 0.869005605148433
5 299 activation of immune response
GO:0072376 0.00172353849898644 13.9532619279455 0.238322607432012
3 82 protein activation cascade
GO:0070661 0.00187524961800535 8.41573033707865 0.528959933568611
4 182 leukocyte proliferation
GO:0043552 0.00200710821962668 34.2571428571429 0.0668465850114179
2 23 positive regulation of phosphatidylinositol 3-kinase activity
GO:0001912 0.00218554691840735 32.6977272727273 0.0697529582727839
2 24 positive regulation of leukocyte mediated cytotoxicity
GO:0043551 0.00218554691840735 32.6977272727273 0.0697529582727839
2 24 regulation of phosphatidylinositol 3-kinase activity
GO:0050850 0.00218554691840735 32.6977272727273 0.0697529582727839
2 24 positive regulation of calcium-mediated signaling
GO:0090218 0.00218554691840735 32.6977272727273 0.0697529582727839
2 24 positive regulation of lipid kinase activity
GO:0007218 0.00224985752505742 12.6631299734748 0.26157359352294
3 90 neuropeptide signaling pathway
GO:0050869 0.00256410862520172 29.96875 0.0755657047955159 2
26 negative regulation of B cell activation
GO:0034612 0.00262425819952185 11.9707357859532 0.27610545982977
3 95 response to tumor necrosis factor
GO:0019221 0.00295183107394798 5.65999034749035 0.991073282125804
5 341 cytokine-mediated signaling pathway
GO:0031343 0.00318554101184146 26.6333333333333 0.0842848245796139
2 29 positive regulation of cell killing
GO:0043550 0.00318554101184146 26.6333333333333 0.0842848245796139
2 29 regulation of lipid kinase activity
GO:0050853 0.00318554101184146 26.6333333333333 0.0842848245796139
2 29 B cell receptor signaling pathway
GO:0001910 0.00340681136275634 25.6803571428571 0.0871911978409799
2 30 regulation of leukocyte mediated cytotoxicity

GO:0016064 0.00407240445033978 10.1858974358974 0.322607432011626
3 111 immunoglobulin mediated immune response
GO:0050848 0.00411243099306514 23.1903225806452 0.0959103176250778
2 33 regulation of calcium-mediated signaling
GO:0019724 0.00438827834974687 9.90852390852391 0.331326551795723
3 114 B cell mediated immunity
GO:0032655 0.00461728111549336 21.7818181818182 0.10172306414781
2 35 regulation of interleukin-12 production
GO:0031341 0.00487991712486397 21.1397058823529 0.104629437409176
2 36 regulation of cell killing
GO:0032615 0.00514930741245981 20.5342857142857 0.107535810670542
2 37 interleukin-12 production
GO:0071345 0.00650112841672883 4.66083078152044 1.19451941042142
5 411 cellular response to cytokine stimulus
GO:0002705 0.00722085666126351 17.1035714285714 0.127880423500104
2 44 positive regulation of leukocyte mediated immunity
GO:0002708 0.00722085666126351 17.1035714285714 0.127880423500104
2 44 positive regulation of lymphocyte mediated immunity
GO:0010522 0.00722085666126351 17.1035714285714 0.127880423500104
2 44 regulation of calcium ion transport into cytosol
GO:0051924 0.00837453187443606 7.78396072013093 0.418517749636703
3 144 regulation of calcium ion transport
GO:0002291 0.00869440324222703 175.69512195122 0.00871911978409799 1
3 T cell activation via T cell receptor contact with antigen bound to MHC molecule on antigen presenting cell
GO:0016056 0.00869440324222703 175.69512195122 0.00871911978409799 1
3 rhodopsin mediated signaling pathway
GO:0031585 0.00869440324222703 175.69512195122 0.00871911978409799 1
3 regulation of inositol 1,4,5-trisphosphate-sensitive calcium-release channel activity
GO:0031987 0.00869440324222703 175.69512195122 0.00871911978409799 1
3 locomotion involved in locomotory behavior
GO:0038110 0.00869440324222703 175.69512195122 0.00871911978409799 1
3 interleukin-2-mediated signaling pathway
GO:0051643 0.00869440324222703 175.69512195122 0.00871911978409799 1
3 endoplasmic reticulum localization
GO:0001776 0.00889458699134167 15.2787234042553 0.142412289806934
2 49 leukocyte homeostasis
GO:0046427 0.00960811169746031 14.6530612244898 0.148225036329666
2 51 positive regulation of JAK-STAT cascade
Tissue: Skin_Sun_Exposed_Lower_leg=>Thyroid Type: asymmetric
SourceGene: RP11-293B20.1(ENSG00000260518.1)
TargetGeneSet: CD53 FCGR2A FCGR3A RP11 CD86 PDE6B RHOH MOCS2 GZMA DOCK2 LY86 GPSM3 RP1 DOK2 FAM203A TNFSF8 MRRF AMPD3 CD3E CD3D BIN2 NCKAP1L SELPLG LCP1 TRAV21 SLC7A7 CORO1A CFDP1 CCL4L2 IKZF3 TNS4 SKAP1 ICAM2 CBX4 VAV1 CD37 LAIR1 C20orf196 TH1L ITGB2 APOBEC3F FAM127A
GOBPID Pvalue OddsRatio ExpCount Count Size Term
GO:0042110 2.68668050517739e-09 18.2614886731392 0.772610891979794
10 319 T cell activation
GO:0046649 3.35977389149501e-09 15.1988546603476 1.04871635180956
11 433 lymphocyte activation
GO:0030098 2.33314803451438e-08 21.4965986394558 0.494083454432219
8 204 lymphocyte differentiation
GO:0030217 7.78853087415086e-08 23.9379194630872 0.377828523977579
7 156 T cell differentiation
GO:0002521 4.77767431944924e-07 14.2323003275384 0.731437270777109
8 302 leukocyte differentiation
GO:0046631 1.47696702907015e-06 31.0367965367965 0.19860217286001
5 82 alpha-beta T cell activation
GO:0050870 3.07066414450078e-06 17.869592476489 0.414158189744654 6
171 positive regulation of T cell activation
GO:0051249 4.31125997728482e-06 12.8079710144928 0.685419694138814

7	283	regulation of lymphocyte activation
GO:0045061	8.64692470749186e-06	96.4419642857143	0.0411736212026849
3	17	thymic T cell selection
GO:0002694	9.84499468954118e-06	11.2277070063694	0.777454847415404
7	321	regulation of leukocyte activation
GO:0051251	1.14494044477565e-05	14.0640158389705	0.520725209328074
6	215	positive regulation of lymphocyte activation
GO:0030097	1.1480459922469e-05	9.09141229141229	1.12137568334371
8	463	hemopoiesis
GO:0050863	1.33916112296405e-05	13.665757818765 0.535257075634904	6
221	regulation of T cell activation
GO:0050865	1.51423074279706e-05	10.4761904761905	0.830738357207114
7	343	regulation of cell activation
GO:0002696	1.80774351286182e-05	12.9324016405894	0.564320808248564
6	233	positive regulation of leukocyte activation
GO:0048534	1.86345632831662e-05	8.4745608031029 1.19887897031347	8
495	hemopoietic or lymphoid organ development
GO:0050867	2.13714392741034e-05	12.5393457117595	0.581274652273199
6	240	positive regulation of cell activation
GO:0050900	2.18791773820163e-05	12.4851063829787	0.583696629991004
6	241	leukocyte migration
GO:0045058	2.54398158559894e-05	64.2633928571429	0.0581274652273199
3	24	T cell selection
GO:0031294	3.10160964812008e-05	26.0699681962744	0.181648328835375
4	75	lymphocyte costimulation
GO:0031295	3.10160964812008e-05	26.0699681962744	0.181648328835375
4	75	T cell costimulation
GO:0046651	5.47850058892634e-05	14.2205588822355	0.416580167462459
5	172	lymphocyte proliferation
GO:0032943	5.78834884852929e-05	14.0502958579882	0.421424122898069
5	174	mononuclear cell proliferation
GO:0070661	7.16565160147345e-05	13.4077212806026	0.440799944640509
5	182	leukocyte proliferation
GO:0043374	0.000118766137090523	174.678787878788	0.016953844024635
2	7	CD8-positive, alpha-beta T cell differentiation
GO:0046635	0.000150299917042784	33.69375	0.104145041865615	3
43	positive regulation of alpha-beta T cell activation
GO:0045059	0.000158113906383722	145.555555555556	0.01937582174244
2	8	positive thymic T cell selection
GO:0042098	0.000181896641323896	16.1878890775325	0.28579337070099
4	118	T cell proliferation
GO:0036037	0.000202980038708031	124.753246753247	0.021797799460245
2	9	CD8-positive, alpha-beta T cell activation
GO:0050851	0.00021335795684026	15.5023041474654	0.297903259290015
4	123	antigen receptor-mediated signaling pathway
GO:0002757	0.000237560414639501	10.2797101449275	0.569164763684174
5	235	immune response-activating signal transduction
GO:0002429	0.000287787005463801	14.2905726431608	0.322123036468065
4	133	immune response-activating cell surface receptor signaling pathway
GO:0002764	0.000304705132251831	9.72085048010974	0.600650474015639
5	248	immune response-regulating signaling pathway
GO:0007603	0.000309165754825163	97.016835016835 0.026641754895855	2
11	phototransduction, visible light
GO:0043368	0.000309165754825163	97.016835016835 0.026641754895855	2
11	positive T cell selection
GO:0045060	0.000309165754825163	97.016835016835 0.026641754895855	2
11	negative thymic T cell selection
GO:0046634	0.000313218738697508	25.8966346153846	0.133208774479275
3	55	regulation of alpha-beta T cell activation
GO:0043383	0.000370434731468843	87.3090909090909	0.02906373261366
2	12	negative T cell selection
GO:0002768	0.000389446373958234	13.1576036866359	0.348764791363919
4	144	immune response-regulating cell surface receptor signaling pathway

GO:0050670 0.000389446373958234 13.1576036866359 0.348764791363919
4 144 regulation of lymphocyte proliferation
GO:0032944 0.000399808628339826 13.0633722260352 0.351186769081724
4 145 regulation of mononuclear cell proliferation
GO:0033077 0.000425431799924838 23.207974137931 0.147740640786105 3
61 T cell differentiation in thymus
GO:0045086 0.000437120856360053 79.366391184573 0.031485710331465 2
13 positive regulation of interleukin-2 biosynthetic process
GO:0070663 0.000454602824247297 12.6115775519222 0.363296657670749
4 150 regulation of leukocyte proliferation
GO:0046632 0.000512923439393237 21.7046370967742 0.157428551657325
3 65 alpha-beta T cell differentiation
GO:0042102 0.000536458947008329 21.358630952381 0.15985052937513 3
66 positive regulation of T cell proliferation
GO:0002253 0.000715435393470298 8.00566893424036 0.724171337623694
5 299 activation of immune response
GO:0043372 0.000757536233832414 58.1858585858586 0.0411736212026849
2 17 positive regulation of CD4-positive, alpha-beta T cell
differentiation
GO:0045577 0.000757536233832414 58.1858585858586 0.0411736212026849
2 17 regulation of B cell differentiation
GO:0045076 0.000949598362949957 51.3333333333333 0.0460175766382949
2 19 regulation of interleukin-2 biosynthetic process
GO:2000516 0.000949598362949957 51.3333333333333 0.0460175766382949
2 19 positive regulation of CD4-positive, alpha-beta T cell activation
GO:0009584 0.00105350659720668 48.4781144781145 0.0484395543560999
2 20 detection of visible light
GO:0007596 0.00112639128863686 5.85534062237174 1.20614490346689
6 498 blood coagulation
GO:0006909 0.00116068996689376 16.1893825301205 0.20829008373123
3 86 phagocytosis
GO:0042094 0.00116263356851686 45.9234449760766 0.0508615320739049
2 21 interleukin-2 biosynthetic process
GO:0046633 0.00116263356851686 45.9234449760766 0.0508615320739049
2 21 alpha-beta T cell proliferation
GO:0002697 0.00135700176970707 9.31324709349926 0.486817521278804
4 201 regulation of immune effector process
GO:0032673 0.00139644608680904 41.5440115440115 0.0557054875095149
2 23 regulation of interleukin-4 production
GO:0043029 0.00139644608680904 41.5440115440115 0.0557054875095149
2 23 T cell homeostasis
GO:0050671 0.00159389247516337 14.4385080645161 0.23250986090928
3 96 positive regulation of lymphocyte proliferation
GO:0032946 0.00164204411823343 14.2839095744681 0.234931838627085
3 97 positive regulation of mononuclear cell proliferation
GO:0009167 0.00165084146432016 37.9262187088274 0.0605494429451249
2 25 purine ribonucleoside monophosphate metabolic process
GO:0050778 0.00171932957720419 6.52599814298979 0.881599889281019
5 364 positive regulation of immune response
GO:0008360 0.00174106597559996 13.984375 0.239775794062695 3
99 regulation of cell shape
GO:0045619 0.00174106597559996 13.984375 0.239775794062695 3
99 regulation of lymphocyte differentiation
GO:0050852 0.00174106597559996 13.984375 0.239775794062695 3
99 T cell receptor signaling pathway
GO:0009126 0.00178569731497192 36.3434343434343 0.0629714206629299
2 26 purine nucleoside monophosphate metabolic process
GO:0070665 0.00179194860229972 13.8392396907216 0.2421977717805 3
100 positive regulation of leukocyte proliferation
GO:0042129 0.00184375387589325 13.6970663265306 0.244619749498305
3 101 regulation of T cell proliferation
GO:0032633 0.00192562655023593 34.8872727272727 0.0653933983807349
2 27 interleukin-4 production

GO:0043370 0.00192562655023593 34.8872727272727 0.0653933983807349
2 27 regulation of CD4-positive, alpha-beta T cell differentiation
GO:2000514 0.00222060948894769 32.2985409652076 0.0702373538163449
2 29 regulation of CD4-positive, alpha-beta T cell activation
GO:0002252 0.00222277992826332 6.13954505686789 0.934883399072729
5 386 immune effector process
GO:0046638 0.00237561555054854 31.1428571428571 0.0726593315341499
2 30 positive regulation of alpha-beta T cell differentiation
GO:0030595 0.00241385960743403 12.4201388888889 0.268839526676355
3 111 leukocyte chemotaxis
GO:0051607 0.00253953689296051 12.1926136363636 0.273683482111965
3 113 defense response to virus
GO:0030838 0.00270053835413189 29.0626262626263 0.0775032869697599
2 32 positive regulation of actin filament polymerization
GO:0007602 0.00287040793370604 28.1231671554252 0.0799252646875649
2 33 phototransduction
GO:0007159 0.00359872757580016 24.9021645021645 0.0896131755587849
2 37 leukocyte cell-cell adhesion
GO:0009161 0.0039918667573981 23.5528255528256 0.0944571309943949
2 39 ribonucleoside monophosphate metabolic process
GO:0032663 0.0039918667573981 23.5528255528256 0.0944571309943949
2 39 regulation of interleukin-2 production
GO:0002260 0.00419560037852698 22.9314194577352 0.0968791087121998
2 40 lymphocyte homeostasis
GO:0009583 0.00419560037852698 22.9314194577352 0.0968791087121998
2 40 detection of light stimulus
GO:0046637 0.00419560037852698 22.9314194577352 0.0968791087121998
2 40 regulation of alpha-beta T cell differentiation
GO:0060326 0.00428179556092064 10.0679041353383 0.329388969621479
3 136 cell chemotaxis
GO:0043367 0.00440407944310129 22.3418803418803 0.0993010864300048
2 41 CD4-positive, alpha-beta T cell differentiation
GO:0035710 0.004835182637369 21.2490761271249 0.104145041865615
2 43 CD4-positive, alpha-beta T cell activation
GO:0030593 0.00505776132746409 20.7417027417027 0.10656701958342
2 44 neutrophil chemotaxis
GO:0032623 0.00505776132746409 20.7417027417027 0.10656701958342
2 44 interleukin-2 production
GO:0032273 0.00528499458136162 20.2579281183932 0.108988997301225
2 45 positive regulation of protein polymerization
GO:0002286 0.00551685987126941 19.7961432506887 0.11141097501903
2 46 T cell activation involved in immune response
GO:0009123 0.00551685987126941 19.7961432506887 0.11141097501903
2 46 nucleoside monophosphate metabolic process
GO:0042113 0.00594279085236457 8.91625 0.370562590824164 3 153
B cell activation
GO:0001776 0.00624002382385215 18.528691166989 0.118676908172445 2
49 leukocyte homeostasis
GO:0030888 0.0064901935046265 3 18.1414141414141 0.12109888589025
2 50 regulation of B cell proliferation
GO:0001766 0.00724884985550822 211.970588235294 0.00726593315341499
1 3 membrane raft polarization
GO:0002669 0.00724884985550822 211.970588235294 0.00726593315341499
1 3 positive regulation of T cell anergy
GO:0002913 0.00724884985550822 211.970588235294 0.00726593315341499
1 3 positive regulation of lymphocyte anergy
GO:0006922 0.00724884985550822 211.970588235294 0.00726593315341499
1 3 cleavage of lamin
GO:0009158 0.00724884985550822 211.970588235294 0.00726593315341499
1 3 ribonucleoside monophosphate catabolic process
GO:0009169 0.00724884985550822 211.970588235294 0.00726593315341499
1 3 purine ribonucleoside monophosphate catabolic process
GO:0031580 0.00724884985550822 211.970588235294 0.00726593315341499

1 3 membrane raft distribution
GO:0032071 0.00724884985550822 211.970588235294 0.00726593315341499
1 3 regulation of endodeoxyribonuclease activity
GO:0032641 0.00724884985550822 211.970588235294 0.00726593315341499
1 3 lymphotoxin A production
GO:0032790 0.00724884985550822 211.970588235294 0.00726593315341499
1 3 ribosome disassembly
GO:0035507 0.00724884985550822 211.970588235294 0.00726593315341499
1 3 regulation of myosin-light-chain-phosphatase activity
GO:0035845 0.00724884985550822 211.970588235294 0.00726593315341499
1 3 photoreceptor cell outer segment organization
GO:0042109 0.00724884985550822 211.970588235294 0.00726593315341499
1 3 lymphotoxin A biosynthetic process
GO:0045404 0.00724884985550822 211.970588235294 0.00726593315341499
1 3 positive regulation of interleukin-4 biosynthetic process
GO:0060099 0.00724884985550822 211.970588235294 0.00726593315341499
1 3 regulation of phagocytosis, engulfment
GO:0060100 0.00724884985550822 211.970588235294 0.00726593315341499
1 3 positive regulation of phagocytosis, engulfment
GO:0045582 0.0072677375634005 17.0707070707071 0.128364819043665
2 53 positive regulation of T cell differentiation
GO:0042100 0.00836672552680524 15.8247933884298 0.138052729914885
2 57 B cell proliferation
GO:0050688 0.00836672552680524 15.8247933884298 0.138052729914885
2 57 regulation of defense response to virus
GO:0042108 0.00894251186372708 15.2674109516215 0.142896685350495
2 59 positive regulation of cytokine biosynthetic process
GO:0001771 0.00965377027737002 141.303921568627 0.00968791087121998
1 4 immunological synapse formation
GO:0002249 0.00965377027737002 141.303921568627 0.00968791087121998
1 4 lymphocyte anergy
GO:0002667 0.00965377027737002 141.303921568627 0.00968791087121998
1 4 regulation of T cell anergy
GO:0002870 0.00965377027737002 141.303921568627 0.00968791087121998
1 4 T cell anergy
GO:0002911 0.00965377027737002 141.303921568627 0.00968791087121998
1 4 regulation of lymphocyte anergy
GO:0006923 0.00965377027737002 141.303921568627 0.00968791087121998
1 4 cleavage of cytoskeletal proteins involved in apoptosis
GO:0009128 0.00965377027737002 141.303921568627 0.00968791087121998
1 4 purine nucleoside monophosphate catabolic process
GO:0032070 0.00965377027737002 141.303921568627 0.00968791087121998
1 4 regulation of deoxyribonuclease activity
GO:0032264 0.00965377027737002 141.303921568627 0.00968791087121998
1 4 IMP salvage
GO:0042097 0.00965377027737002 141.303921568627 0.00968791087121998
1 4 interleukin-4 biosynthetic process
GO:0045402 0.00965377027737002 141.303921568627 0.00968791087121998
1 4 regulation of interleukin-4 biosynthetic process
GO:0051665 0.00965377027737002 141.303921568627 0.00968791087121998
1 4 membrane raft localization
GO:2001185 0.00965377027737002 141.303921568627 0.00968791087121998
1 4 regulation of CD8-positive, alpha-beta T cell activation
GO:0045621 0.00983857231101985 14.5010101010101 0.15016261850391
2 62 positive regulation of lymphocyte differentiation
Tissue: Skin_Sun_Exposed_Lower_leg=>Thyroid Type: cluster
SourceGene: RP11-320N21.1(ENSG00000245080.3)
TargetGeneSet: NOC2L KIAA0090 PSMB2 CYB5RL MRPL37 CCDC76 AMY2A
SEC22B PIAS3 RUSC1-AS1 METTL13 HEATR1 RNASEH1 CCT7 C2orf55 TMEM177 RP11
ATG7 XYLB U4 RUVBL1 PSMD2 LMLN CRMP1 CNGA1 CCT5 NHP2
ZSCAN23 ZNRD1-AS1 ATAT1 AARS2 BEND6 PLEKHA8 IGFBP1 COPS6 FAM115B GS1
EXOSC4 TUB RPS6KA4 DLAT DCPS COPS7A TAS2R20 PFKM NUP37 PSMB5
RPAP1 PKM2 CCDC113 DPH1 PSMB6 TP53 PFAS ZNF29P ERAL1 PSMD11 LIG3

PSMB3 TUBG1 PHB C19orf55 PSMD8 GRWD1 RUVBL2 CBR1 PISD
XRCC6 ATXN10 POLA1 APEX2 PGK1 G6PD
GOBPID Pvalue OddsRatio ExpCount Count Size Term
GO:0006977 1.69180334633732e-10 40.3546798029557 0.260328004982354
8 66 DNA damage response, signal transduction by p53 class mediator
resulting in cell cycle arrest
GO:0072413 1.69180334633732e-10 40.3546798029557 0.260328004982354
8 66 signal transduction involved in mitotic cell cycle checkpoint
GO:0072431 1.69180334633732e-10 40.3546798029557 0.260328004982354
8 66 signal transduction involved in mitotic cell cycle G1/S transition
DNA damage checkpoint
GO:0072474 1.69180334633732e-10 40.3546798029557 0.260328004982354
8 66 signal transduction involved in mitotic cell cycle G1/S checkpoint
GO:0072401 1.91539867176746e-10 39.6679349705984 0.264272368694208
8 67 signal transduction involved in DNA integrity checkpoint
GO:0072404 1.91539867176746e-10 39.6679349705984 0.264272368694208
8 67 signal transduction involved in G1/S transition checkpoint
GO:0072422 1.91539867176746e-10 39.6679349705984 0.264272368694208
8 67 signal transduction involved in DNA damage checkpoint
GO:0072395 2.16422956295763e-10 39.0040816326531 0.268216732406062
8 68 signal transduction involved in cell cycle checkpoint
GO:0031571 3.87650819970675e-10 35.9912087912088 0.287938550965331
8 73 mitotic cell cycle G1/S transition DNA damage checkpoint
GO:0000216 7.38828971619158e-10 32.9359011210118 0.311604733236454
8 79 M/G1 transition of mitotic cell cycle
GO:0071158 8.18441281880951e-10 32.4761904761905 0.315549096948308
8 80 positive regulation of cell cycle arrest
GO:0031575 1.00023942202098e-09 31.5940430226144 0.323437824372016
8 82 mitotic cell cycle G1/S transition checkpoint
GO:0071779 1.47116870827763e-09 29.9654631083203 0.339215279219431
8 86 G1/S transition checkpoint
GO:0006521 2.30334928262346e-09 40.1632 0.22482873157567 7 57
regulation of cellular amino acid metabolic process
GO:2000045 4.56365470640182e-09 25.6613590491142 0.390492007473531
8 99 regulation of G1/S transition of mitotic cell cycle
GO:0051436 7.34990694493526e-09 33.446 0.264272368694208 7 67
negative regulation of ubiquitin-protein ligase activity involved in mitotic cell
cycle
GO:0033238 1.22625579913569e-08 30.8624615384615 0.283994187253477
7 72 regulation of cellular amine metabolic process
GO:0051352 1.22625579913569e-08 30.8624615384615 0.283994187253477
7 72 negative regulation of ligase activity
GO:0051437 1.22625579913569e-08 30.8624615384615 0.283994187253477
7 72 positive regulation of ubiquitin-protein ligase activity involved in
mitotic cell cycle
GO:0051444 1.22625579913569e-08 30.8624615384615 0.283994187253477
7 72 negative regulation of ubiquitin-protein ligase activity
GO:0002479 1.63724525491548e-08 29.4947058823529 0.295827278389039
7 75 antigen processing and presentation of exogenous peptide antigen via
MHC class I, TAP-dependent
GO:0030330 1.7213929464672e-08 21.3967421831118 0.461490554286901
8 117 DNA damage response, signal transduction by p53 class mediator
GO:0051439 2.1594916278439e-08 28.2425352112676 0.3076603695246 7
78 regulation of ubiquitin-protein ligase activity involved in mitotic cell
cycle
GO:0042590 2.36225914332687e-08 27.8483333333333 0.311604733236454
7 79 antigen processing and presentation of exogenous peptide antigen via
MHC class I
GO:0072331 2.55290897424942e-08 20.2718722271517 0.485156736558024
8 123 signal transduction by p53 class mediator
GO:0051443 3.07004488772205e-08 26.7288 0.323437824372016 7 82
positive regulation of ubiquitin-protein ligase activity
GO:0002478 3.34264755705798e-08 26.3752631578947 0.32738218808387

7	83	antigen processing and presentation of exogenous peptide antigen
GO:0031145	3.34264755705798e-08	26.3752631578947	0.32738218808387
7	83	anaphase-promoting complex-dependent proteasomal ubiquitin-dependent protein catabolic process
GO:0000077	3.94165223336479e-08	19.0993643358983	0.512767282541001
8	130	DNA damage checkpoint
GO:0019884	3.94971858815973e-08	25.6953846153846	0.335270915507577
7	85	antigen processing and presentation of exogenous antigen
GO:0000082	4.22080493396771e-08	15.0603813559322	0.733651650404816
9	186	G1/S transition of mitotic cell cycle
GO:0051351	4.28665798083472e-08	25.3683544303797	0.339215279219431
7	86	positive regulation of ligase activity
GO:0000084	4.99586841258498e-08	18.4878522837706	0.528544737388416
8	134	S phase of mitotic cell cycle
GO:0031570	5.60840019695119e-08	18.1964285714286	0.536433464812124
8	136	DNA integrity checkpoint
GO:0007093	6.64817448853105e-08	17.7759775665992	0.548266555947685
8	139	mitotic cell cycle checkpoint
GO:0031397	6.86285717730111e-08	23.5677647058824	0.362881461490554
7	92	negative regulation of protein ubiquitination
GO:0042770	7.02976646102498e-08	17.6400742115028	0.552210919659539
8	140	signal transduction in response to DNA damage
GO:0051320	8.28985944607622e-08	17.2444444444444	0.564044010795101
8	143	S phase
GO:0051438	9.22454663005284e-08	22.5022471910112	0.37865891633797
7	96	regulation of ubiquitin-protein ligase activity
GO:2000602	1.02690770372898e-07	16.7435031566583	0.579821465642516
8	147	regulation of interphase of mitotic cell cycle
GO:0002474	1.14169414322057e-07	21.7639130434783	0.390492007473531
7	99	antigen processing and presentation of peptide antigen via MHC class I
GO:0051340	1.2239056893103e-07	21.5283870967742	0.394436371185385
7	100	regulation of ligase activity
GO:0090068	1.26419058489461e-07	16.2705865563008	0.595598920489931
8	151	positive regulation of cell cycle process
GO:0010565	1.47176005622437e-07	15.9329046687168	0.607432011625493
8	154	regulation of cellular ketone metabolic process
GO:0048002	2.0807229048262e-07	19.8120792079208	0.425991280880216
7	108	antigen processing and presentation of peptide antigen
GO:0051329	2.18365624736862e-07	9.11424858223062	1.49491384679261
11	379	interphase of mitotic cell cycle
GO:0051325	2.69142243053328e-07	8.91524051803885	1.52646875648744
11	387	interphase
GO:0031396	2.87416013552962e-07	14.5244897959184	0.662653103591447
8	168	regulation of protein ubiquitination
GO:0031398	6.29410745103251e-07	16.653	0.500934191405439	7	127
positive regulation of protein ubiquitination
GO:0006520	8.97584506929342e-07	7.8407838334354	1.72368694208013	11
437	cellular amino acid metabolic process
GO:0046365	1.79097767566084e-06	19.1256684491979	0.370770188914262
6	94	monosaccharide catabolic process
GO:0019882	2.11068667280847e-06	13.7576551724138	0.599543284201785
7	152	antigen processing and presentation
GO:0031400	2.38366102018076e-06	9.08698453608247	1.18330911355616
9	300	negative regulation of protein modification process
GO:0016052	2.73240688138478e-06	13.2054304635762	0.623209466472908
7	158	carbohydrate catabolic process
GO:0000209	3.09651986475303e-06	12.9454545454545	0.63504255760847
7	161	protein polyubiquitination
GO:0000075	4.48521167920036e-06	9.87964416535845	0.954536018268632
8	242	cell cycle checkpoint
GO:0044106	7.21446604962585e-06	11.3097727272727	0.721818559269255
7	183	cellular amine metabolic process

GO:0071156	8.49875948306201e-06	9.01658163265306	1.04131201992942	8	264	regulation of cell cycle arrest
GO:0016032	9.19777860880485e-06	6.73179910261977	1.77890803404609	10	451	viral reproduction
GO:0006007	1.11607128859528e-05	19.6758241758242	0.295827278389039	5	75	glucose catabolic process
GO:0009308	1.37475198092014e-05	10.1941538461538	0.796761469794478	7	202	amine metabolic process
GO:0032269	1.54541541720387e-05	7.1265243902439	1.49096948308076	9	378	negative regulation of cellular protein metabolic process
GO:0043161	1.71432678751984e-05	9.8360396039604	0.82437201577455	7	209	proteasomal ubiquitin-dependent protein catabolic process
GO:0010498	2.12055317973677e-05	9.50191387559809	0.851982561760432	7	216	proteasomal protein catabolic process
GO:0007346	2.30488984137643e-05	7.80297474922172	1.19514220469172	8	303	regulation of mitotic cell cycle
GO:0019320	2.71701511188135e-05	16.1866515837104	0.354992734066847	5	90	hexose catabolic process
GO:0051248	3.54646841588079e-05	6.37910583941606	1.65663275897862	9	420	negative regulation of protein metabolic process
GO:0007050	9.27819170873055e-05	6.3465430493527	1.45547020967407	8	369	cell cycle arrest
GO:0006096	0.000102335888881641	18.6545217957059	0.244550550134939	4	62	glycolysis
GO:0034644	0.000159123490207166	33.2638888888889	0.106497820220054	3	27	cellular response to UV
GO:0006310	0.000167194038994796	8.22428281657491	0.824372015777455	6	209	DNA recombination
GO:0010564	0.000204841947669952	5.625012566603	1.63296657670749	8	414	regulation of cell cycle process
GO:0071482	0.000266082225714309	27.5191570881226	0.126219638779323	3	32	cellular response to light stimulus
GO:0032392	0.000291873188185559	26.6	0.130164002491177	3	33	DNA geometric change
GO:0045786	0.000295410128982287	5.31468531468532	1.72368694208013	8	437	negative regulation of cell cycle
GO:0006273	0.000421539838176437	87.2	0.0315549096948308	2	8	lagging strand elongation
GO:0005996	0.000486257574063144	6.68320340184266	1.00581274652273	6	255	monosaccharide metabolic process
GO:0000338	0.000540606632178134	74.7376623376623	0.0354992734066847	2	9	protein deneddylation
GO:0010388	0.000540606632178134	74.7376623376623	0.0354992734066847	2	9	cullin deneddylation
GO:0006511	0.000543259288114523	5.50470588235294	1.4357483911148	7	364	ubiquitin-dependent protein catabolic process
GO:0016570	0.000549774824252818	6.52318339100346	1.02947892879386	6	261	histone modification
GO:0019941	0.000608344237296959	5.39615384615385	1.46335893709778	7	371	modification-dependent protein catabolic process
GO:0016569	0.000619583299297766	6.37052062204192	1.05314511106498	6	267	covalent chromatin modification
GO:0043632	0.000638074129706616	5.35089918256131	1.47519202823334	7	374	modification-dependent macromolecule catabolic process
GO:0006266	0.000674046648170741	65.3909090909091	0.0394436371185385	2	10	DNA ligation
GO:0030163	0.000676079825851976	4.66228051795667	1.95246003736766	8	495	protein catabolic process
GO:0006281	0.000734270793197293	5.21946808510638	1.51069130164002	7	383	DNA repair
GO:0051603	0.000804563710224911	5.13528795811518	1.53435748391115	7	389	proteolysis involved in cellular protein catabolic process
GO:0016573	0.000859442933124756	10.3701015965167	0.425991280880216			

4	108	histone acetylation
GO:0044419	0.000867131900157234	5.06713178294574	1.55407930247042
7	394	interspecies interaction between organisms
GO:0018393	0.000920464419094877	10.1730153079388	0.433880008303924
4	110	internal peptidyl-lysine acetylation
GO:0031056	0.00094253406036512	17.3285024154589	0.193273821880839
3	49	regulation of histone modification
GO:0044257	0.000975204959013457	4.96167088607595	1.58563421216525
7	402	cellular protein catabolic process
GO:0006303	0.000983602004949684	52.3054545454545	0.0473323645422462
2	12	double-strand break repair via nonhomologous end joining
GO:0019321	0.000983602004949684	52.3054545454545	0.0473323645422462
2	12	pentose metabolic process
GO:0043968	0.000983602004949684	52.3054545454545	0.0473323645422462
2	12	histone H2A acetylation
GO:0018394	0.00101763507377363	9.89094685823092	0.445713099439485
4	113	peptidyl-lysine acetylation
GO:0006006	0.00107870204397061	7.07502989238741	0.780984014947062
5	198	glucose metabolic process
GO:0006475	0.00108629059233314	9.7113717491076 0.453601826863193	4
115	internal protein amino acid acetylation
GO:0071478	0.00153941452161595	14.4838383838384	0.228773095287523
3	58	cellular response to radiation
GO:0000726	0.00155297347453807	40.2265734265734	0.0591654556778078
2	15	non-recombinational repair
GO:0006473	0.00161393407662699	8.68533171028606	0.504878555117293
4	128	protein acetylation
GO:0022613	0.00189749896939257	6.19493006993007	0.887481835167116
5	225	ribonucleoprotein complex biogenesis
GO:0019318	0.00225138991968919	5.94768223043332	0.922981108573801
5	234	hexose metabolic process
GO:0018205	0.00229823412393509	7.85401459854015	0.556155283371393
4	141	peptidyl-lysine modification
GO:0071843	0.00246765333928066	5.81854043392505	0.94270292713307
5	239	cellular component biogenesis at cellular level
GO:0043543	0.00248066554433373	7.68409703504043	0.567988374506954
4	144	protein acylation
GO:0042254	0.00280649402421377	7.41652569941444	0.587710193066224
4	149	ribosome biogenesis
GO:0006260	0.0040314535482301	5.16634980988593	1.05708947477683
5	268	DNA replication
GO:0033044	0.00427607512846763	9.94027777777778	0.32738218808387
3	83	regulation of chromosome organization
GO:0035065	0.0043262629639112	22.7209486166008	0.0986090927963463
2	25	regulation of histone acetylation
GO:0000291	0.00467496492120553	21.7727272727273	0.1025534565082 2
26	nuclear-transcribed mRNA catabolic process, exonucleolytic
GO:0043928	0.00467496492120553	21.7727272727273	0.1025534565082 2
26	exonucleolytic nuclear-transcribed mRNA catabolic process involved in deadenylation-dependent decay
GO:0070228	0.0050362322356854	20.9003636363636	0.106497820220054
2	27	regulation of lymphocyte apoptotic process
GO:2000756	0.0050362322356854	20.9003636363636	0.106497820220054
2	27	regulation of peptidyl-lysine acetylation
GO:0009411	0.0050366602528623	9.35228758169935	0.347104006643139
3	88	response to UV
GO:0034660	0.00522435135594807	4.84684065934066	1.12414365787835
5	285	ncRNA metabolic process
GO:0032508	0.00540996391986441	20.0951048951049	0.110442183931908
2	28	DNA duplex unwinding
GO:0006261	0.00587398483150878	8.82962962962963	0.366825825202408
3	93	DNA-dependent DNA replication
GO:0016568	0.00597030732275107	3.98262355789774	1.65268839526676

6	419	chromatin modification
GO:0031058	0.00619441932858303	18.6571428571429	0.118330911355616
2	30	positive regulation of histone modification
GO:0016575	0.00660494395082142	18.012539184953 0.122275275067469	2
31	histone deacetylation
GO:0006302	0.00758079190010149	8.02188552188552	0.402325098609093
3	102	double-strand break repair
GO:0006271	0.00790852280964366	16.3204545454545	0.134108366203031
2	34	DNA strand elongation involved in DNA replication
GO:0034470	0.00793936756122365	5.46707739699653	0.78887274237077
4	200	ncRNA processing
GO:0006364	0.00799575748262253	7.86193619361936	0.4102138260328 3
104	rRNA processing
GO:0070227	0.00883660524121672	15.3582887700535	0.141997093626739
2	36	lymphocyte apoptotic process
GO:0022616	0.00931806605517473	14.9184415584416	0.145941457338592
2	37	DNA strand elongation
GO:0016072	0.00932016759081294	7.41796469366563	0.433880008303924
3	110	rRNA metabolic process
GO:2001252	0.00981101262259769	14.5030303030303	0.149885821050446
2	38	positive regulation of chromosome organization
Tissue: Skin_Sun_Exposed_Lower_leg=>Thyroid	Type:	cluster
SourceGene:	RP11-323D18.4(ENSG00000224625.2)
TargetGeneSet:	ZBTB48	LZIC	RP1	TCEB3	EYA3	EBNA1BP2	DPH2
PRKAA2	THEM4	PYGO2	FLAD1	HEATR1	SLC5A6	ASS1P2	CCDC142	POLR1A	POLR1B
CNOT10	TMEM39A	TMEM41A	GFM2	HARS	LARS	EEF1E1	PAK1IP1	RPL7L1	POLR1C	VTA1
MIOS	STOML2	FXN	RABEPK	ZNF79	TBC1D13	EXOSC2	NGB	ADO	POLR3A
ENTPD7	C10orf2	LZTS2	RP11	PDCD11	SPTY2D1	TMEM216	ZNF259	ZNF202	NOP2
PA2G4	UTP20	ATP6V0A2	PUS1	TTC5	PRMT5	LEO1	C15orf26
POLR3K	UBFD1	CTD	TSR1	TMEM199	CTB	PSME3	SNX11	METTL2A	SIRT7	KRI1
ZNF433	NUDT19	ZNF461	ZNF576	OPA3	ZNF432	ZNF749	NOP56	RRP1B	PES1	GCAT
L3MBTL2	PPPDE2	UTP14A
GOBPID	Pvalue	OddsRatio	ExpCount	Count	Size	Term
GO:0071843	7.77522004347637e-15	20.3221524663677	1.09155075773303
16	239	cellular component biogenesis at cellular level
GO:0022613	6.37926985798725e-14	19.8529411764706	1.02761054598298
15	225	ribonucleoprotein complex biogenesis
GO:0042254	1.12063449901627e-13	25.6988068812431	0.680506539339838
13	149	ribosome biogenesis
GO:0034660	2.00245516060066e-12	15.3758169934641	1.30164002491177
15	285	ncRNA metabolic process
GO:0006364	3.61943461078362e-11	27.148556231003 0.474984430143243	10
104	rRNA processing
GO:0016072	6.36653164247794e-11	25.5089285714286	0.502387378036122
10	110	rRNA metabolic process
GO:0034470	1.49773832207879e-09	15.0222222222222	0.913431596429313
11	200	ncRNA processing
GO:0006354	5.80041871081762e-07	16.7793253901661	0.493253062071829
7	108	DNA-dependent transcription, elongation
GO:0006353	3.33910160873591e-05	15.4324849007765	0.369939796553872
5	81	DNA-dependent transcription, termination
GO:0006385	7.06983285707816e-05	45.6190476190476	0.0822088436786382
3	18	transcription elongation from RNA polymerase III promoter
GO:0006386	7.06983285707816e-05	45.6190476190476	0.0822088436786382
3	18	termination of RNA polymerase III transcription
GO:0006362	8.36803846565564e-05	42.7648809523809	0.0867760016607847
3	19	transcription elongation from RNA polymerase I promoter
GO:0009303	8.36803846565564e-05	42.7648809523809	0.0867760016607847
3	19	rRNA transcription
GO:0006363	0.000114107463271234	38.0079365079365	0.0959103176250778
3	21	termination of RNA polymerase I transcription
GO:0006361	0.000171956454651492	32.5714285714286	0.109611791571518
3	24	transcription initiation from RNA polymerase I promoter

GO:0007000 0.000203629069863812 149.8125 0.0228357899107328 2
5 nucleolus organization
GO:0000466 0.000304542518136291 112.3515625 0.0274029478928794 2
6 maturation of 5.8S rRNA from tricistronic rRNA transcript (SSU-rRNA, 5.8S rRNA, LSU-rRNA)
GO:0006360 0.000410539573718707 23.5730706075534 0.14614905542869
3 32 transcription from RNA polymerase I promoter
GO:0071826 0.00112000369305845 9.60282258064516 0.456715798214656
4 100 ribonucleoprotein complex subunit organization
GO:0000460 0.00131646899746473 44.921875 0.0548058957857588 2
12 maturation of 5.8S rRNA
GO:0006418 0.00135892751961416 15.1746031746032 0.219223583143035
3 48 tRNA aminoacylation for protein translation
GO:0043038 0.00162032020688437 14.2232142857143 0.232925057089475
3 51 amino acid activation
GO:0043039 0.00162032020688437 14.2232142857143 0.232925057089475
3 51 tRNA aminoacylation
GO:0006383 0.0017139455188221 13.9319727891156 0.237492215071621
3 52 transcription from RNA polymerase III promoter
GO:0006399 0.00254227009695544 7.60543854972007 0.57089474776832
4 125 tRNA metabolic process
GO:0016570 0.00674626543153756 4.52388575819672 1.19202823334025
5 261 histone modification
GO:0016569 0.00740867475787636 4.41840820923539 1.21943118123313
5 267 covalent chromatin modification
GO:0000387 0.00877135240453798 15.4698275862069 0.141581897446543
2 31 spliceosomal snRNP assembly
GO:0022618 0.00934004599905399 7.398033126294 0.433880008303924 3
95 ribonucleoprotein complex assembly
Tissue: Thyroid=>Skin_Sun_Exposed_Lower_leg Type: asymmetric
SourceGene: RP11-473O4.3(ENSG00000253720.1)
TargetGeneSet: TRIT1 RPS8 PRPF38A NEDD4 RPL5 BOLA1 DCST2 PAQR6 COPA LYPLAL1 SRP9 C1orf35 RPS7 RP11 TMEM214 DDIT3 UBC FIGLA TPRKB COA5 ARL6IP6 ASNSD1 SENP2 NOP58 EEF1B2 USP40 CRBN GRM2 THOC7 RPL24 RYK SNORA63 MRFAP1L1 COMMD8 EXOC7 RPL34 CDKN2AIP PPWD1 TAF9 CTD MAN2A1 NPM1 BOD1 TMEM14B TDP2 HIST1H1B PRRT1 AGER MAD2L1BP COQ3 RP3 AIM1 ASF1A GTF2H5 C6orf70 KLHL7 TAX1BP1 SBDS C8orf40 FNTA SGK3 PAF1 CKS1BP7 CA3 TTC35 C8orf76 C9orf123 RPS6 NDUFB6 CHMP5 C10orf31 COMMD3 CUL2 CHCHD1 RPS24 C10orf32 HRAS C11orf58 C11orf46 IMMP1L CCDC90B ZCRB1 NACA C12orf47 MRP63 EFHA1 HMGB1 FRY-AS1 UFM1 WBP4 UPF3A METTL17 EAPP SNX6 SRSF5 RSL24D1 UBAP1L MTHFS WFIKKN1 RHBDL1 NUDT21 EDC4 FTLP14 PSMD7 MED11 RPL26 RPS7P1 C17orf75 ARHGAP27 CEP95 NOL11 RPL17 CIRBP XAB2 CCDC130 SLC25A42 SUGP1 FAM98C ZNF773 C21orf119 SPATA20P1 CTA TBC1D8B VBP1
GOBPID Pvalue OddsRatio ExpCount Count Size Term
GO:0006414 9.63494194805802e-13 25.2284577993814 0.617604318040274
12 105 translational elongation
GO:0006614 2.38224892509629e-11 22.5692926969523 0.617604318040274
11 105 SRP-dependent cotranslational protein targeting to membrane
GO:0006613 2.64556672883813e-11 22.3301564722617 0.623486263926372
11 106 cotranslational protein targeting to membrane
GO:0045047 2.64556672883813e-11 22.3301564722617 0.623486263926372
11 106 protein targeting to ER
GO:0072599 2.64556672883813e-11 22.3301564722617 0.623486263926372
11 106 establishment of protein localization to endoplasmic reticulum
GO:0070972 8.604099912405e-11 19.8091689820662 0.694069614559546
11 118 protein localization to endoplasmic reticulum
GO:0000184 9.43576716288022e-11 19.6243743743744 0.699951560445644
11 119 nuclear-transcribed mRNA catabolic process, nonsense-mediated decay
GO:0006415 1.2558277564126e-10 23.5144032921811 0.535257075634904
10 91 translational termination
GO:0072594 1.82545487122759e-10 13.3998691099476 1.19991696076396

13	204	establishment of protein localization to organelle
GO:0000956	2.77698857692242e-10	14.8772358432947	0.994048854750536
12	169	nuclear-transcribed mRNA catabolic process
GO:0006402	5.06479339728e-10	14.0617263574847	1.04698636772542
12	178	mRNA catabolic process
GO:0022613	6.13784041834554e-10	12.0546383647799	1.32343782437202
13	225	ribonucleoprotein complex biogenesis
GO:0071843	1.28677545195209e-09	11.2967059980334	1.40578506677739
13	239	cellular component biogenesis at cellular level
GO:0006612	1.42124265995181e-09	14.8899885801294	0.899937720572971
11	153	protein targeting to membrane
GO:0043624	1.42124265995181e-09	14.8899885801294	0.899937720572971
11	153	cellular protein complex disassembly
GO:0043241	2.00077200951795e-09	14.3784703070417	0.92934745000346
11	158	protein complex disassembly
GO:0006401	2.55551489636779e-09	12.0715451770885	1.20579890665006
12	205	RNA catabolic process
GO:0034623	5.5419676658147e-09	12.9524954402255	1.02345858418103
11	174	cellular macromolecular complex disassembly
GO:0032984	7.45977842054639e-09	12.5625804375804	1.05286831361151
11	179	macromolecular complex disassembly
GO:0071845	1.03535434187357e-08	9.39088765887659	1.67047263165179
13	284	cellular component disassembly at cellular level
GO:0022411	1.22352049119903e-08	9.25166666666667	1.69400041519618
13	288	cellular component disassembly
GO:0042254	1.61989775956199e-08	13.6470023980815	0.876409937028579
10	149	ribosome biogenesis
GO:0019080	1.84113478799433e-08	13.451536643026 0.888173828800775	10
151	viral genome expression
GO:0019083	1.84113478799433e-08	13.451536643026 0.888173828800775	10
151	viral transcription
GO:0006413	1.96145897607643e-08	13.3558685446009	0.894055774686873
10	152	translational initiation
GO:0022415	3.66556342556424e-08	9.35793194874061	1.52930593038544
12	260	viral reproductive process
GO:0006364	1.66876313981007e-07	15.4437229437229	0.611722372154176
8	104	rRNA processing
GO:0016072	2.57978972112315e-07	14.5291571173924	0.647014047470763
8	110	rRNA metabolic process
GO:0016032	3.40853072402775e-07	6.28504206014117	2.65275759463013
14	451	viral reproduction
GO:0033365	6.04687279925659e-07	5.97434717235877	2.78216040412428
14	473	protein localization to organelle
GO:0006605	6.20176219177771e-07	5.96093080220453	2.78804235001038
14	474	protein targeting
GO:0019058	9.8363073351747e-07	8.53393665158371	1.3587294996886 10
231	viral infectious cycle
GO:0034470	2.58422186747729e-06	8.78857812069441	1.17638917721957
9	200	ncRNA processing
GO:0042274	3.20324942668156e-06	50.6243386243386	0.105875025949761
4	18	ribosomal small subunit biogenesis
GO:0008380	1.92025972464917e-05	5.9863684771033 1.8998685212096 10	323
RNA splicing
GO:0022618	1.98158317671353e-05	12.1834731901579	0.558784859179296
6	95	ribonucleoprotein complex assembly
GO:0071826	2.65503682587538e-05	11.5313762456235	0.588194588609785
6	100	ribonucleoprotein complex subunit organization
GO:0042273	4.15850860595777e-05	58.3617886178862	0.0705833506331742
3	12	ribosomal large subunit biogenesis
GO:0034660	4.41491830450558e-05	6.04548054919908	1.67635457753789
9	285	ncRNA metabolic process
GO:0042255	0.000104066430391483	40.3930581613508	0.0941111341775656
3	16	ribosome assembly

GO:0006397 0.000113667369892079 4.79074550128535 2.34689640855304
10 399 mRNA processing
GO:0000377 0.000196098658897105 6.45470519328387 1.19991696076396
7 204 RNA splicing, via transesterification reactions with bulged adenosine as nucleophile
GO:0000398 0.000196098658897105 6.45470519328387 1.19991696076396
7 204 nuclear mRNA splicing, via spliceosome
GO:0000375 0.000227478789806445 6.29271388677329 1.22932669019445
7 209 RNA splicing, via transesterification reactions
GO:0000462 0.000505082847272706 86.5180722891566 0.0352916753165871
2 6 maturation of SSU-rRNA from tricistronic rRNA transcript (SSU-rRNA, 5.8S rRNA, LSU-rRNA)
GO:0030490 0.000704412693740366 69.2096385542169 0.0411736212026849
2 7 maturation of SSU-rRNA
GO:0006511 0.00133476465735811 4.08872026849555 2.14102830253962
8 364 ubiquitin-dependent protein catabolic process
GO:0019941 0.00150602053313423 4.00787091696183 2.1822019237423 8
371 modification-dependent protein catabolic process
GO:0043632 0.00158456918102932 3.97416790859414 2.1998477614006 8
374 modification-dependent macromolecule catabolic process
GO:0006281 0.00183993437902993 3.87629437229437 2.25278527437548
8 383 DNA repair
GO:0051603 0.00202755986881313 3.81361420731499 2.28807694969206
8 389 proteolysis involved in cellular protein catabolic process
GO:0050930 0.00217192689908843 34.5927710843374 0.0705833506331742
2 12 induction of positive chemotaxis
GO:0030163 0.0024694876358983 3.38206627680312 2.91156321361843
9 495 protein catabolic process
GO:0044257 0.00248597939151822 3.68435625288417 2.36454224621134
8 402 cellular protein catabolic process
GO:0043620 0.00492094639463268 21.6114457831325 0.105875025949761
2 18 regulation of DNA-dependent transcription in response to stress
GO:0050927 0.00606459675869567 19.2074966532798 0.117638917721957
2 20 positive regulation of positive chemotaxis
GO:0050926 0.0066775058852177 18.1953075459734 0.123520863608055
2 21 regulation of positive chemotaxis
GO:0043161 0.00776631411310921 4.33884803921569 1.22932669019445
5 209 proteasomal ubiquitin-dependent protein catabolic process
GO:0065004 0.00823021901095836 5.36612948826689 0.794062694623209
4 135 protein-DNA complex assembly
GO:0007346 0.00885705081886503 3.59774964838256 1.78222960348765
6 303 regulation of mitotic cell cycle
GO:0010498 0.00888459433957299 4.1928317535545 1.27050031139714 5
216 proteasomal protein catabolic process
GO:0007093 0.00910002199762345 5.20566986739826 0.817590478167601
4 139 mitotic cell cycle checkpoint
Tissue: Thyroid=>Skin_Sun_Exposed_Lower_leg Type: cluster
SourceGene: RP11-473O4.3(ENSG00000253720.1)
TargetGeneSet: TRIT1 RPS8 PRPF38A NEDD4 RPL5 BOLA1 DCST2 PAQR6 COPA
LYPLAL1 SRP9 C1orf35 RPS7 RP11 TMEM214 DDIT3 UBC FIGLA TPRKB COA5
ARL6IP6 ASNSD1 SENP2 NOP58 EEF1B2 USP40 CRBN GRM2 THOC7 RPL24 RYK
SNORA63 MRFAP1L1 COMMD8 EXOC7 RPL34 CDKN2AIP PPWD1 TAF9 CTD
MAN2A1 NPM1 BOD1 TMEM14B TDP2 HIST1H1B PRRT1 AGER MAD2L1BP
COQ3 RP3 AIM1 ASF1A GTF2H5 C6orf70 KLHL7 TAX1BP1 SBDS C8orf40 FNTA
SGK3 PAF1 CKS1BP7 CA3 TTC35 C8orf76 C9orf123 RPS6 NDUFB6
CHMP5 C10orf31 COMMD3 CUL2 CHCHD1 RPS24 C10orf32 HRAS
C11orf58 C11orf46 IMMP1L CCDC90B ZCRB1 NACA C12orf47
MRP63 EFHA1 HMGB1 FRY-AS1 UFM1 WBP4 UPF3A METTL17 EAPP SNX6
SRSF5 RSL24D1 UBAP1L MTHFS WFIKKN1 RHBDL1 NUDT21 EDC4 FTLP14 PSMD7
MED11 RPL26 RPS7P1 C17orf75 ARHGAP27 CEP95 NOL11 RPL17
CIRBP XAB2 CCDC130 SLC25A42 SUGP1 FAM98C ZNF773 C21orf119
SPATA20P1 CTA TBC1D8B VBP1
GOBPID Pvalue OddsRatio ExpCount Count Size Term

GO:0006414	9.63494194805802e-13	25.2284577993814	0.617604318040274
12	105	translational elongation
GO:0006614	2.38224892509629e-11	22.5692926969523	0.617604318040274
11	105	SRP-dependent cotranslational protein targeting to membrane
GO:0006613	2.64556672883813e-11	22.3301564722617	0.623486263926372
11	106	cotranslational protein targeting to membrane
GO:0045047	2.64556672883813e-11	22.3301564722617	0.623486263926372
11	106	protein targeting to ER
GO:0072599	2.64556672883813e-11	22.3301564722617	0.623486263926372
11	106	establishment of protein localization to endoplasmic reticulum
GO:0070972	8.604099912405e-11	19.8091689820662	0.694069614559546
11	118	protein localization to endoplasmic reticulum
GO:0000184	9.43576716288022e-11	19.6243743743744	0.699951560445644
11	119	nuclear-transcribed mRNA catabolic process, nonsense-mediated decay
GO:0006415	1.2558277564126e-10	23.5144032921811	0.535257075634904
10	91	translational termination
GO:0072594	1.82545487122759e-10	13.3998691099476	1.19991696076396
13	204	establishment of protein localization to organelle
GO:0000956	2.77698857692242e-10	14.8772358432947	0.994048854750536
12	169	nuclear-transcribed mRNA catabolic process
GO:0006402	5.06479339728e-10	14.0617263574847	1.04698636772542
12	178	mRNA catabolic process
GO:0022613	6.13784041834554e-10	12.0546383647799	1.32343782437202
13	225	ribonucleoprotein complex biogenesis
GO:0071843	1.28677545195209e-09	11.2967059980334	1.40578506677739
13	239	cellular component biogenesis at cellular level
GO:0006612	1.42124265995181e-09	14.8899885801294	0.899937720572971
11	153	protein targeting to membrane
GO:0043624	1.42124265995181e-09	14.8899885801294	0.899937720572971
11	153	cellular protein complex disassembly
GO:0043241	2.00077200951795e-09	14.3784703070417	0.92934745000346
11	158	protein complex disassembly
GO:0006401	2.55551489636779e-09	12.0715451770885	1.20579890665006
12	205	RNA catabolic process
GO:0034623	5.5419676658147e-09	12.9524954402255	1.02345858418103
11	174	cellular macromolecular complex disassembly
GO:0032984	7.45977842054639e-09	12.5625804375804	1.05286831361151
11	179	macromolecular complex disassembly
GO:0071845	1.03535434187357e-08	9.39088765887659	1.67047263165179
13	284	cellular component disassembly at cellular level
GO:0022411	1.22352049119903e-08	9.25166666666667	1.69400041519618
13	288	cellular component disassembly
GO:0042254	1.61989775956199e-08	13.6470023980815	0.876409937028579
10	149	ribosome biogenesis
GO:0019080	1.84113478799433e-08	13.451536643026 0.888173828800775	10
151	viral genome expression
GO:0019083	1.84113478799433e-08	13.451536643026 0.888173828800775	10
151	viral transcription
GO:0006413	1.96145897607643e-08	13.3558685446009	0.894055774686873
10	152	translational initiation
GO:0022415	3.66556342556424e-08	9.35793194874061	1.52930593038544
12	260	viral reproductive process
GO:0006364	1.66876313981007e-07	15.4437229437229	0.611722372154176
8	104	rRNA processing
GO:0016072	2.57978972112315e-07	14.5291571173924	0.647014047470763
8	110	rRNA metabolic process
GO:0016032	3.40853072402775e-07	6.28504206014117	2.65275759463013
14	451	viral reproduction
GO:0033365	6.04687279925659e-07	5.97434717235877	2.78216040412428
14	473	protein localization to organelle
GO:0006605	6.20176219177771e-07	5.96093080220453	2.78804235001038
14	474	protein targeting
GO:0019058	9.8363073351747e-07	8.53393665158371	1.3587294996886 10

231	viral infectious cycle
GO:0034470	2.58422186747729e-06	8.78857812069441	1.17638917721957
9	200	ncRNA processing
GO:0042274	3.20324942668156e-06	50.6243386243386	0.105875025949761
4	18	ribosomal small subunit biogenesis
GO:0008380	1.92025972464917e-05	5.9863684771033 1.8998685212096 10	323
RNA splicing
GO:0022618	1.98158317671353e-05	12.1834731901579	0.558784859179296
6	95	ribonucleoprotein complex assembly
GO:0071826	2.65503682587538e-05	11.5313762456235	0.588194588609785
6	100	ribonucleoprotein complex subunit organization
GO:0042273	4.15850860595777e-05	58.3617886178862	0.0705833506331742
3	12	ribosomal large subunit biogenesis
GO:0034660	4.41491830450558e-05	6.04548054919908	1.67635457753789
9	285	ncRNA metabolic process
GO:0042255	0.000104066430391483	40.3930581613508	0.0941111341775656
3	16	ribosome assembly
GO:0006397	0.000113667369892079	4.79074550128535	2.34689640855304
10	399	mRNA processing
GO:0000377	0.000196098658897105	6.45470519328387	1.19991696076396
7	204	RNA splicing, via transesterification reactions with bulged adenosine as nucleophile
GO:0000398	0.000196098658897105	6.45470519328387	1.19991696076396
7	204	nuclear mRNA splicing, via spliceosome
GO:0000375	0.000227478789806445	6.29271388677329	1.22932669019445
7	209	RNA splicing, via transesterification reactions
GO:0000462	0.000505082847272706	86.5180722891566	0.0352916753165871
2	6	maturation of SSU-rRNA from tricistronic rRNA transcript (SSU-rRNA, 5.8S rRNA, LSU-rRNA)
GO:0030490	0.000704412693740366	69.2096385542169	0.0411736212026849
2	7	maturation of SSU-rRNA
GO:0006511	0.00133476465735811	4.08872026849555	2.14102830253962
8	364	ubiquitin-dependent protein catabolic process
GO:0019941	0.00150602053313423	4.00787091696183	2.1822019237423 8
371	modification-dependent protein catabolic process
GO:0043632	0.00158456918102932	3.97416790859414	2.1998477614006 8
374	modification-dependent macromolecule catabolic process
GO:0006281	0.00183993437902993	3.87629437229437	2.25278527437548
8	383	DNA repair
GO:0051603	0.00202755986881313	3.81361420731499	2.28807694969206
8	389	proteolysis involved in cellular protein catabolic process
GO:0050930	0.00217192689908843	34.5927710843374	0.0705833506331742
2	12	induction of positive chemotaxis
GO:0030163	0.0024694876358983	3.38206627680312	2.91156321361843
9	495	protein catabolic process
GO:0044257	0.00248597939151822	3.68435625288417	2.36454224621134
8	402	cellular protein catabolic process
GO:0043620	0.00492094639463268	21.6114457831325	0.105875025949761
2	18	regulation of DNA-dependent transcription in response to stress
GO:0050927	0.00606459675869567	19.2074966532798	0.117638917721957
2	20	positive regulation of positive chemotaxis
GO:0050926	0.0066775058852177	18.1953075459734	0.123520863608055
2	21	regulation of positive chemotaxis
GO:0043161	0.00776631411310921	4.33884803921569	1.22932669019445
5	209	proteasomal ubiquitin-dependent protein catabolic process
GO:0065004	0.00823021901095836	5.36612948826689	0.794062694623209
4	135	protein-DNA complex assembly
GO:0007346	0.00885705081886503	3.59774964838256	1.78222960348765
6	303	regulation of mitotic cell cycle
GO:0010498	0.00888459433957299	4.1928317535545 1.27050031139714	5
216	proteasomal protein catabolic process
GO:0007093	0.0091002199762345	5.20566986739826	0.817590478167601
4	139	mitotic cell cycle checkpoint

Tissue: Skin_Sun_Exposed_Lower_leg=>Thyroid Type: asymmetric
SourceGene: SLC26A10(ENSG00000135502.12)
TargetGeneSet: RP11 CACNA1S C1orf147 ACTA1 ACTN2 XIRP2 MYL1
VGLL2 TRDN BPGM MYPN TNNI2 OR7E13P TBRG1 RP3 MYBPC1 C12orf73
TCHP CATSPERB MYH2 CKM TNNT1 PCNT MYO18B
GOBPID Pvalue OddsRatio ExpCount Count Size Term
GO:0030049 4.45877744890921e-14 242.290865384615 0.0539755034253685
7 39 muscle filament sliding
GO:0033275 4.45877744890921e-14 242.290865384615 0.0539755034253685
7 39 actin-myosin filament sliding
GO:0070252 7.80225094085748e-14 221.476923076923 0.0581274652273199
7 42 actin-mediated cell contraction
GO:0030048 9.73465781956882e-13 148.894970414201 0.0816552487717113
7 59 actin filament-based movement
GO:0006936 1.14193843371407e-11 50.5173913043478 0.330772956888797
9 239 muscle contraction
GO:0003012 2.98289607788242e-11 45.1241598868058 0.368140613106359
9 266 muscle system process
GO:0030029 2.20114025828332e-09 27.0947775628627 0.597882499481005
9 432 actin filament-based process
GO:0006941 3.04424438900589e-06 50.5633802816901 0.103799045048786
4 75 striated muscle contraction
GO:0010927 2.17896209326086e-05 30.0672268907563 0.170230433880008
4 123 cellular component assembly involved in morphogenesis
GO:0043501 6.51320731303245e-05 228.952380952381 0.0124558854058543
2 9 skeletal muscle adaptation
GO:0014888 0.000140651479709995 145.656565656566 0.0179918344751228
2 13 striated muscle adaptation
GO:0061061 0.000193326293867678 11.5734323432343 0.56605079233271
5 409 muscle structure development
GO:0003009 0.0003068190587918 94.2091503267974 0.0262957580790257
2 19 skeletal muscle contraction
GO:0055002 0.000410499840858156 24.3105203619909 0.148086637602934
3 107 striated muscle cell development
GO:0050879 0.000579757517178671 66.6990740740741 0.0359836689502457
2 26 multicellular organismal movement
GO:0050881 0.000579757517178671 66.6990740740741 0.0359836689502457
2 26 musculoskeletal movement
GO:0007517 0.00070401627756025 11.8160535117057 0.419348141997094
4 303 muscle organ development
GO:0055001 0.000708935535931096 20.0350140056022 0.178534357483911
3 129 muscle cell development
GO:0030239 0.000994130463345698 49.9965277777778 0.0470555670887828
2 34 myofibril assembly
GO:0007519 0.00109773164116792 17.1476590636255 0.207598090097571
3 150 skeletal muscle tissue development
GO:0043500 0.00117726471214678 45.7015873015873 0.0512075288907342
2 37 muscle adaptation
GO:0060538 0.00127518523706249 16.2535104364326 0.218669988236108
3 158 skeletal muscle organ development
GO:0051146 0.0015473561962559 15.1647767540751 0.233893848176597
3 169 striated muscle cell differentiation
GO:0031032 0.0017384182648659 37.1782945736434 0.0622794270292713
2 45 actomyosin structure organization
GO:0030031 0.00209851528895702 13.5891891891892 0.260189606255622
3 188 cell projection assembly
GO:0042384 0.00370160561663533 24.9427083333333 0.0913431596429313
2 66 cilium assembly
GO:0030035 0.00414650474202016 379.710526315789 0.00415196180195142
1 3 microspike assembly
GO:0032066 0.00414650474202016 379.710526315789 0.00415196180195142
1 3 nucleolus to nucleoplasm transport
GO:0042396 0.00414650474202016 379.710526315789 0.00415196180195142

1	3	phosphagen biosynthetic process
GO:0048741	0.0041537626724066	23.468954248366 0.0968791087121998	2
70	skeletal muscle fiber development
GO:0042692	0.00438294808678494	10.3905296558457	0.337692893225382
3	244	muscle cell differentiation
GO:0048747	0.00525994507921223	20.7128427128427	0.109334994118054
2	79	muscle fiber development
GO:0014706	0.00534538779923922	9.65614353849648	0.362604664037091
3	262	striated muscle tissue development
GO:0003010	0.0055250394807 4912	253.122807017544	0.00553594906926856
1	4	voluntary skeletal muscle contraction
GO:0014721	0.00552503948074912	253.122807017544	0.00553594906926856
1	4	twitch skeletal muscle contraction
GO:0060537	0.00599176048850142	9.25555555555556	0.377828523977579
3	273	muscle tissue development
GO:0060271	0.00634458151023876	18.7529411764706	0.120406892256591
2	87	cilium morphogenesis
GO:0001778	0.00690176123684949	189.828947368421	0.0069199363365857
1	5	plasma membrane repair
GO:0030240	0.00690176123684949	189.828947368421	0.0069199363365857
1	5	skeletal muscle thin filament assembly
GO:0060315	0.00690176123684949	189.828947368421	0.0069199363365857
1	5	negative regulation of ryanodine-sensitive calcium-release channel activity
GO:0032412	0.00798790870288432	16.5914351851852	0.13563075219708
2	98	regulation of ion transmembrane transporter activity
GO:0000244	0.00827667226933348	151.852631578947	0.00830392360390284
1	6	assembly of spliceosomal tri-snRNP
GO:0014866	0.00827667226933348	151.852631578947	0.00830392360390284
1	6	skeletal myofibril assembly
GO:0022898	0.0087918046237 9007	15.7645764576458	0.142550688533665
2	103	regulation of transmembrane transporter activity
GO:1901020	0.00964977483455431	126.535087719298	0.00968791087121998
1	7	negative regulation of calcium ion transmembrane transporter activity
GO:0034765	0.0098025252408953	14.8743509865005	0.150854612137568
2	109	regulation of ion transmembrane transport
Tissue: Skin_Sun_Exposed_Lower_leg=>Thyroid	Type:	asymmetric
SourceGene:	TCF4(ENSG00000196628.7)
TargetGeneSet:	KCNAB2	WBP1	FTH1P22 RP4	IGKJ4	IGKV1-8 IGKV1-17
IGKV3-20	IGKV1-27	CD28	ICOS	ARL4C	PDCD1	SIDT1	DTHD1	U6
IGBP1P4 RP11	LHFPL3	PNOC	TCTN1	IGHG2	IGHGP	IGHG1	IGHV2-26
IGHV4-31	IGHV3-33	IGHV4-61	IGHV3-74	JMJD8	TBL3	GCSH
TNFRSF13B	PLD6	ATP9B	ZSCAN18 ZBP1	IGLV3-25	IGLV2-14
IGLC2	IGLC3	MIAT	TNFRSF13C	BEX2	MTCP1
GOBPID	Pvalue	OddsRatio	ExpCount	Count	Size	Term
GO:0006959	3.97227453570534e-11	52.8531855955679	0.227942702927133
8	122	humoral immune response
GO:0050778	1.16111174280658e-08	19.8154929577465	0.680091343159643
9	364	positive regulation of immune response
GO:0002449	1.77391227425219e-08	31.2025 0.312019929416649	7	167
lymphocyte mediated immunity
GO:0006958	3.0780705661675e-08	72.6212121212121	0.093419140543907
5	50	complement activation, classical pathway
GO:0002460	3.09440229672954e-08	28.6637931034483	0.338177288768943
7	181	adaptive immune response based on somatic recombination of immune receptors built from immunoglobulin superfamily domains
GO:0016064	4.65226895787969e-08	38.9632653061225	0.207390492007474
6	111	immunoglobulin mediated immune response
GO:0019724	5.45911568725853e-08	37.8730158730159	0.212995640440108
6	114	B cell mediated immunity
GO:0002250	5.73973811363212e-08	26.0814136125654	0.369939796553872
7	198	adaptive immune response

GO:0002443	8.87587889384766e-08	24.3970588235294	0.394228773095288	7	211	leukocyte mediated immunity
GO:0006956	1.00439134449161e-07	56.2931034482759	0.117708117085323	5	63	complement activation
GO:0002455	1.1773038054287e-07	54.4090909090909	0.121444882707079	5	65	humoral immune response mediated by circulating immunoglobulin
GO:0072376	3.80586713500269e-07	42.3465171192444	0.153207390492007	5	82	protein activation cascade
GO:0002252	5.14043064091848e-06	12.9703166226913	0.721195764998962	7	386	immune effector process
GO:0031294	1.07278252023149e-05	35.1573790569504	0.140128710815861	4	75	lymphocyte costimulation
GO:0031295	1.07278252023149e-05	35.1573790569504	0.140128710815861	4	75	T cell costimulation
GO:0050864	1.45726414017468e-05	32.4042913608131	0.151339007681129	4	81	regulation of B cell activation
GO:0002253	1.53118039550086e-05	13.7796196977084	0.558646460452564	6	299	activation of immune response
GO:0030888	0.000107522759101617	38.2367021276596	0.093419140543907	3	50	regulation of B cell proliferation
GO:0046649	0.00012146566979257	9.36567413850786	0.809009757110235	6	433	lymphocyte activation
GO:0050670	0.000138856518445971	17.744099378882	0.269047124766452	4	144	regulation of lymphocyte proliferation
GO:0032944	0.000142613770045866	17.6170212765957	0.27091550757733	4	145	regulation of mononuclear cell proliferation
GO:0051249	0.000157604446247797	11.5647482014388	0.528752335478514	5	283	regulation of lymphocyte activation
GO:0042100	0.000159123490207166	33.2638888888889	0.106497820220054	3	57	B cell proliferation
GO:0070663	0.000162515447931474	17.0077427039905	0.280257421631721	4	150	regulation of leukocyte proliferation
GO:0042113	0.00017538004703184	16.6618033265247	0.285862570064355	4	153	B cell activation
GO:0045066	0.000219333509983732	115.312	0.0224205937305377	2	12	regulatory T cell differentiation
GO:0030889	0.000258913610471625	104.821818181818	0.0242889765414158	2	13	negative regulation of B cell proliferation
GO:0050870	0.000268566108079728	14.8471752147878	0.319493460660162	4	171	positive regulation of T cell activation
GO:0046651	0.000274610196078045	14.7577639751553	0.32136184347104	4	172	lymphocyte proliferation
GO:0042110	0.000275018514558874	10.2127967573827	0.596014116670127	5	319	T cell activation
GO:0002694	0.000283079927967113	10.1467203682394	0.599750882291883	5	321	regulation of leukocyte activation
GO:0032943	0.000286991449531745	14.5820971867008	0.325098609092796	4	174	mononuclear cell proliferation
GO:0070661	0.000340560941099582	13.9189057156815	0.340045671579821	4	182	leukocyte proliferation
GO:0050865	0.000384162560432835	9.47149004841313	0.640855304131202	5	343	regulation of cell activation
GO:0001782	0.00050495018454502	72.04	0.0336308905958065	2	18	B cell homeostasis
GO:0042035	0.000633305008731961	20.3636363636364	0.170022835789911	3	91	regulation of cytokine biosynthetic process
GO:0051251	0.000639167222562705	11.714815577993	0.4017023043388	4	215	positive regulation of lymphocyte activation
GO:0050863	0.000708663573971892	11.3860949709477	0.412912601204069	4	221	regulation of T cell activation
GO:0042089	0.000833567862006689	18.4626288659794	0.186838281087814	3	100	cytokine biosynthetic process
GO:0002696	0.000863502941305667	10.780330358838	0.435333194934607	4		

233	positive regulation of leukocyte activation
GO:0042107	0.000882920450586887	18.0871212121212	0.19057504670957
3	102	cytokine metabolic process
GO:0050867	0.00096413679721674	10.4554163596168	0.448411874610754
4	240	positive regulation of cell activation
GO:0050869	0.00106276642474843	48	0.0485779530828316	2	26
negative regulation of B cell activation
GO:0002260	0.00250987280221428	30.2863157894737	0.0747353124351256
2	40	lymphocyte homeostasis
GO:0001776	0.00374516579797624	24.4714893617021	0.0915507577330289
2	49	leukocyte homeostasis
GO:0032945	0.00374516579797624	24.4714893617021	0.0915507577330289
2	49	negative regulation of mononuclear cell proliferation
GO:0050672	0.00374516579797624	24.4714893617021	0.0915507577330289
2	49	negative regulation of lymphocyte proliferation
GO:0070664	0.00420833512253117	22.9984 0.0971559061656633	2	52
negative regulation of leukocyte proliferation
GO:0050871	0.00453115282243661	22.1107692307692	0.10089267178742
2	54	positive regulation of B cell activation
GO:0042108	0.00538673529853182	20.1642105263158	0.11023458584181
2	59	positive regulation of cytokine biosynthetic process
GO:0002636	0.00559506886110994	277.346153846154	0.00560514843263442
1	3	positive regulation of germinal center formation
GO:0015917	0.00559506886110994	277.346153846154	0.00560514843263442
1	3	aminophospholipid transport
GO:0016056	0.00559506886110994	277.346153846154	0.00560514843263442
1	3	rhodopsin mediated signaling pathway
GO:0045590	0.00559506886110994	277.346153846154	0.00560514843263442
1	3	negative regulation of regulatory T cell differentiation
GO:0042102	0.00669906381624608	17.95	0.123313265517957	2	66
positive regulation of T cell proliferation
GO:0002706	0.00814189961126811	16.1723943661972	0.136391945194104
2	73	regulation of lymphocyte mediated immunity
GO:0007028	0.00930835421207143	138.653846153846	0.0093419140543907
1	5	cytoplasm organization
GO:0070934	0.00930835421207143	138.653846153846	0.0093419140543907
1	5	CRD-mediated mRNA stabilization
GO:0002822	0.00971188231026789	14.7138461538462	0.149470624870251
2	80	regulation of adaptive immune response based on somatic
recombination of immune receptors built from immunoglobulin superfamily domains
Tissue: Skin_Sun_Exposed_Lower_leg=>Thyroid	Type:	cluster
SourceGene:	TCF4(ENSG00000196628.7)
TargetGeneSet:	KCNAB2	WBP1	FTH1P22 RP4	IGKJ4	IGKV1-8 IGKV1-17
IGKV3-20	IGKV1-27	CD28	ICOS	ARL4C	PDCD1	SIDT1	DTHD1	U6
IGBP1P4 RP11	LHFPL3	PNOC	TCTN1	IGHG2	IGHGP	IGHG1	IGHV2-26
IGHV4-31	IGHV3-33	IGHV4-61	IGHV3-74	JMJD8	TBL3	GCSH
TNFRSF13B	PLD6	ATP9B	ZSCAN18 ZBP1	IGLV3-25	IGLV2-14
IGLC2	IGLC3	MIAT	TNFRSF13C	BEX2	MTCP1
GOBPID	Pvalue	OddsRatio	ExpCount	Count	Size	Term
GO:0006959	3.97227453570534e-11	52.8531855955679	0.227942702927133
8	122	humoral immune response
GO:0050778	1.16111174280658e-08	19.8154929577465	0.680091343159643
9	364	positive regulation of immune response
GO:0002449	1.77391227425219e-08	31.2025 0.312019929416649	7	167
lymphocyte mediated immunity
GO:0006958	3.0780705661675e-08	72.6212121212121	0.093419140543907
5	50	complement activation, classical pathway
GO:0002460	3.09440229672954e-08	28.6637931034483	0.338177288768943
7	181	adaptive immune response based on somatic recombination of immune
receptors built from immunoglobulin superfamily domains
GO:0016064	4.65226895787969e-08	38.9632653061225	0.207390492007474
6	111	immunoglobulin mediated immune response
GO:0019724	5.45911568725853e-08	37.8730158730159	0.212995640440108

6	114	B cell mediated immunity
GO:0002250	5.73973811363212e-08	26.0814136125654	0.369939796553872
7	198	adaptive immune response
GO:0002443	8.87587889384766e-08	24.3970588235294	0.394228773095288
7	211	leukocyte mediated immunity
GO:0006956	1.00439134449161e-07	56.2931034482759	0.117708117085323
5	63	complement activation
GO:0002455	1.1773038054287e-07	54.4090909090909	0.121444882707079
5	65	humoral immune response mediated by circulating immunoglobulin
GO:0072376	3.80586713500269e-07	42.3465171192444	0.153207390492007
5	82	protein activation cascade
GO:0002252	5.14043064091848e-06	12.9703166226913	0.721195764998962
7	386	immune effector process
GO:0031294	1.07278252023149e-05	35.1573790569504	0.140128710815861
4	75	lymphocyte costimulation
GO:0031295	1.07278252023149e-05	35.1573790569504	0.140128710815861
4	75	T cell costimulation
GO:0050864	1.45726414017468e-05	32.4042913608131	0.151339007681129
4	81	regulation of B cell activation
GO:0002253	1.53118039550086e-05	13.7796196977084	0.558646460452564
6	299	activation of immune response
GO:0030888	0.000107522759101617	38.2367021276596	0.093419140543907
3	50	regulation of B cell proliferation
GO:0046649	0.00012146566979257	9.36567413850786	0.809009757110235
6	433	lymphocyte activation
GO:0050670	0.000138856518445971	17.744099378882 0.269047124766452	4
144	regulation of lymphocyte proliferation
GO:0032944	0.000142613770045866	17.6170212765957	0.27091550757733
4	145	regulation of mononuclear cell proliferation
GO:0051249	0.000157604446247797	11.5647482014388	0.528752335478514
5	283	regulation of lymphocyte activation
GO:0042100	0.000159123490207166	33.2638888888889	0.106497820220054
3	57	B cell proliferation
GO:0070663	0.000162515447931474	17.0077427039905	0.280257421631721
4	150	regulation of leukocyte proliferation
GO:0042113	0.00017538004703184	16.6618033265247	0.285862570064355
4	153	B cell activation
GO:0045066	0.000219333509983732	115.312 0.0224205937305377	2	12
regulatory T cell differentiation
GO:0030889	0.000258913610471625	104.821818181818	0.0242889765414158
2	13	negative regulation of B cell proliferation
GO:0050870	0.000268566108079728	14.8471752147878	0.319493460660162
4	171	positive regulation of T cell activation
GO:0046651	0.000274610196078045	14.7577639751553	0.32136184347104
4	172	lymphocyte proliferation
GO:0042110	0.000275018514558874	10.2127967573827	0.596014116670127
5	319	T cell activation
GO:0002694	0.000283079927967113	10.1467203682394	0.599750882291883
5	321	regulation of leukocyte activation
GO:0032943	0.000286991449531745	14.5820971867008	0.325098609092796
4	174	mononuclear cell proliferation
GO:0070661	0.000340560941099582	13.9189057156815	0.340045671579821
4	182	leukocyte proliferation
GO:0050865	0.000384162560432835	9.47149004841313	0.640855304131202
5	343	regulation of cell activation
GO:0001782	0.00050495018454502	72.04	0.0336308905958065	2	18
B cell homeostasis
GO:0042035	0.000633305008731961	20.3636363636364	0.170022835789911
3	91	regulation of cytokine biosynthetic process
GO:0051251	0.000639167222562705	11.714815577993 0.4017023043388 4	215
positive regulation of lymphocyte activation
GO:0050863	0.000708663573971892	11.3860949709477	0.412912601204069
4	221	regulation of T cell activation

GO:0042089 0.000833567862006689 18.4626288659794 0.186838281087814
3 100 cytokine biosynthetic process
GO:0002696 0.000863502941305667 10.780330358838 0.435333194934607 4
233 positive regulation of leukocyte activation
GO:0042107 0.000882920450586887 18.0871212121212 0.19057504670957
3 102 cytokine metabolic process
GO:0050867 0.00096413679721674 10.4554163596168 0.448411874610754
4 240 positive regulation of cell activation
GO:0050869 0.00106276642474843 48 0.0485779530828316 2 26
negative regulation of B cell activation
GO:0002260 0.00250987280221428 30.2863157894737 0.0747353124351256
2 40 lymphocyte homeostasis
GO:0001776 0.00374516579797624 24.4714893617021 0.0915507577330289
2 49 leukocyte homeostasis
GO:0032945 0.00374516579797624 24.4714893617021 0.0915507577330289
2 49 negative regulation of mononuclear cell proliferation
GO:0050672 0.00374516579797624 24.4714893617021 0.0915507577330289
2 49 negative regulation of lymphocyte proliferation
GO:0070664 0.00420833512253117 22.9984 0.0971559061656633 2 52
negative regulation of leukocyte proliferation
GO:0050871 0.00453115282243661 22.1107692307692 0.10089267178742
2 54 positive regulation of B cell activation
GO:0042108 0.00538673529853182 20.1642105263158 0.11023458584181
2 59 positive regulation of cytokine biosynthetic process
GO:0002636 0.00559506886110994 277.346153846154 0.00560514843263442
1 3 positive regulation of germinal center formation
GO:0015917 0.00559506886110994 277.346153846154 0.00560514843263442
1 3 aminophospholipid transport
GO:0016056 0.00559506886110994 277.346153846154 0.00560514843263442
1 3 rhodopsin mediated signaling pathway
GO:0045590 0.00559506886110994 277.346153846154 0.00560514843263442
1 3 negative regulation of regulatory T cell differentiation
GO:0042102 0.00669906381624608 17.95 0.123313265517957 2 66
positive regulation of T cell proliferation
GO:0002706 0.00814189961126811 16.1723943661972 0.136391945194104
2 73 regulation of lymphocyte mediated immunity
GO:0007028 0.00930835421207143 138.653846153846 0.0093419140543907
1 5 cytoplasm organization
GO:0070934 0.00930835421207143 138.653846153846 0.0093419140543907
1 5 CRD-mediated mRNA stabilization
GO:0002822 0.00971188231026789 14.7138461538462 0.149470624870251
2 80 regulation of adaptive immune response based on somatic recombination of immune receptors built from immunoglobulin superfamily domains
Tissue: Whole_Blood=>Thyroid Type: asymmetric
SourceGene: 7SK(ENSG00000223039.1)
TargetGeneSet: RP11 ERRFI1 NMNAT1 DFFA DNAJC16 OTUD3 XKR8 MED18
PRDX3P2 FAM167B HDAC1 ZMYM1 RNF220 HHLA3 DDAH1 FRRS1 CYB561D1
DDX20 BCL9 GJA5 BOLA1 GOLPH3L ARNT VPS72 SHE LAMTOR2 MNDA
SLAMF8 USP21 FCGR3A RNASEL SMG7 TRMT1L ZNF281 DISP1 MIXL1 C2orf44 PREB
EHD3 ARHGEF33 MSH6 PAPOLG SPR MRPL35 ZNF2 C2orf29 POLR1B ORC2
ICA1L PARD3B IDH1 SPAG16 TMEM169 TNP1 MRPL44 EIF4E2 RNPEPL1 RPUSD3
CCRL1 ZNF619 ZNF620 CCR2 DUSP7 SCT ABHD10 WDR52 ADPRH MSL2
SLC25A36 ZBTB38 TRPC1 SIAH2 P2RY13 TACC3 SH3BP2 TMEM128 ZNF518B
NFXL1 UTP3 IL8 CASP6 ANKRD50 TIGD4 CCDC127 LPCAT1 BRIP1 DAP
C5orf51 SLC38A9 TRIM23 CD180 CETN3 SLC22A5 AFF4 SH3RF2 TCERG1 HMGXB3
C5orf54 NEURL1B DOK3 CTB HIST1H3E ZKSCAN4 ZKSCAN3 SCAND3 MICB APOM
PFDN6 ZBTB9 NFYA TAF8 C6orf203 TSPYL4 AKAP12 FAM103A2P
ELFN1 RNF216P1 COL28A1 TRA2A VOPP1 LAT2 TMEM60 SLC12A9 GCC1
TNPO3 FAM131B EPHA1 ZNF786 ZNF398 GIMAP8 GIMAP7 GIMAP4 ESYT2 CTA
SH2D4A CTD INTS9 PDP1 WDR67 KLHL38 ZNF623 TONSL ZNF250 RANBP6
KLHL9 DCAF10 C9orf85 C9orf40 RMI1 FOXE1 RNF20 C9orf80 ATP6V1G1
TRIM32 PSMD5 LHX6 ZBTB34 NAIF1 ABL1 GTF3C4 QSOX2 NUDT5 C10orf128
ERCC6 ADO MSS51 AP3M1 ARHGAP19 HIF1AN C10orf95 SUFU

DUSP5 SMC3 CASC2 DUSP8 TPH1 SLC35C1 FAM180B HEXIM1 TMEM132A
TTC9C ZBTB3 MEN1 EIF1AD C11orf82 ANKRD42 PICALM RDX DPAGT1
ZNF202 HYLS1 KCNJ1 RAD52 NCAPD2 BCDIN3D SMUG1 ZC3H10 ZBTB39 DYRK2
ATXN7L3B CSRP2 TMPO RFC5 SRRM4 RNF34 ORAI1 RNF6 FAM48A
KBTBD6 KBTBD7 ARL11 SUGT1 FARP1-IT1 LIG4 RNASE2 ACIN1 C14orf119
SYNJ2BP MAP3K9 PROX2 VASH1 ZC3H14 RPS6KA5 BTBD7 PGBD4 ZFYVE19 ZFP106
ZSCAN29 HDC RNF111 LACTB DIS3L ANP32A PEX11A C15orf38 NR2F2
LRRC28 ASB7 ZNF434 TFAP4 C16orf88 SPN TAOK2 DDX19B PLCG2
SLC16A13 TNK1 SNORA48 KRBA2 PIGL BLMH RNF135 CCT6B PEX12
FAM134C GJC1 HIGD1B TBKBP1 DLX3 PPP1R9B CHAD HEATR6 APPBP2 FTSJ3
CD79B QRICH2 CBX2 LIPG ZNF555 PIAS4 TNFSF9 ZNF557 ZNF799 ZNF443
ZNF490 LYL1 JUND LRRC25 SUGP1 ZNF90 ZNF486 KCTD15 ZNF792 U2AF1L4
LIN37 CAPNS1 ZFP82 ZNF260 ZNF420 ZFP36 EID2 LTBP4 MIA ZNF526
ZNF284 ZNF225 BLOC1S3 RSPH6A IGFL4 PPP1R15A NAPSA ZNF616 ZNF816
ZNF677 ZNF765 ZNF835 ZNF264 ZNF547 ZNF17 ZNF549 ZNF550 FAM110A ABHD12 NANP
DNMT3B PXMP4 LBP ZSWIM3 ZSWIM1 ZFP64 CSTF1 C20orf151 TCFL5
SOX18 RCAN1 C2CD2 C22orf29 CRKL PPM1F ZNF70 PRR14L MFNG TLR7
TLR8 SCML1 EIF1AX ZFX RBM3 FAM123B VSIG4 FHL1 IDS
GOBPID Pvalue OddsRatio ExpCount Count Size Term
GO:0006469 0.000279990173208074 4.19201861130995 2.59414573385925
10 142 negative regulation of protein kinase activity
GO:0010332 0.000353325705541072 9.4248435627746 0.621133485571933 5
34 response to gamma radiation
GO:0033673 0.000457566102139861 3.92192997151952 2.75856342121652
10 151 negative regulation of kinase activity
GO:0033233 0.000466732731923299 27.1666666666667 0.164417687357276
3 9 regulation of protein sumoylation
GO:0071901 0.000742358201208229 5.12498054474708 1.49802781814407
7 82 negative regulation of protein serine/threonine kinase activity
GO:0051348 0.000831268473669817 3.61123977149915 2.97778700435956
10 163 negative regulation of transferase activity
GO:0045356 0.00194710386073015 54.1412213740458 0.073074527714345
2 4 positive regulation of interferon-alpha biosynthetic process
GO:0001933 0.00223963189042068 3.13417143879742 3.39796553871704
10 186 negative regulation of protein phosphorylation
GO:0000122 0.00240816445650831 2.29541912146729 7.85551172929209
17 430 negative regulation of transcription from RNA polymerase II promoter
GO:0002282 0.00320605995974029 36.0916030534351 0.0913431596429313
2 5 microglial cell activation involved in immune response
GO:0045349 0.00320605995974029 36.0916030534351 0.0913431596429313
2 5 interferon-alpha biosynthetic process
GO:0045354 0.00320605995974029 36.0916030534351 0.0913431596429313
2 5 regulation of interferon-alpha biosynthetic process
GO:0042326 0.00351530233284685 2.9316049589546 3.61718912186008 10
198 negative regulation of phosphorylation
GO:0010212 0.0036502089730963 3.7986670262357 1.97301224828732 7
108 response to ionizing radiation
GO:0045736 0.0040144883875776 10.8597701149425 0.328835374714553
3 18 negative regulation of cyclin-dependent protein kinase activity
GO:0010563 0.00419302704988163 2.85463669372935 3.70853228150301
10 203 negative regulation of phosphorus metabolic process
GO:0045936 0.00419302704988163 2.85463669372935 3.70853228150301
10 203 negative regulation of phosphate metabolic process
GO:0000244 0.00475121374870953 27.0667938931298 0.109611791571518
2 6 assembly of spliceosomal tri-snRNP
GO:0007256 0.00475121374870953 27.0667938931298 0.109611791571518
2 6 activation of JNKK activity
GO:0008630 0.00493330491000203 6.40407239819005 0.694208013286278
4 38 DNA damage response, signal transduction resulting in induction of
apoptosis
GO:0033235 0.00657176753387678 21.6519083969466 0.127880423500104
2 7 positive regulation of protein sumoylation
GO:0035970 0.00657176753387678 21.6519083969466 0.127880423500104

2	7	peptidyl-threonine dephosphorylation
GO:0045359	0.00657176753387678	21.6519083969466	0.127880423500104
2	7	positive regulation of interferon-beta biosynthetic process
GO:0002263	0.00739237613492882	3.30393801153898	2.24704172721611
7	123	cell activation involved in immune response
GO:0002366	0.00739237613492882	3.30393801153898	2.24704172721611
7	123	leukocyte activation involved in immune response
GO:0000012	0.00865722004551591	18.0419847328244	0.14614905542869
2	8	single strand break repair
GO:0045350	0.00865722004551591	18.0419847328244	0.14614905542869
2	8	interferon-beta biosynthetic process
GO:0045357	0.00865722004551591	18.0419847328244	0.14614905542869
2	8	regulation of interferon-beta biosynthetic process
GO:0045416	0.00865722004551591	18.0419847328244	0.14614905542869
2	8	positive regulation of interleukin-8 biosynthetic process
GO:0031400	0.00926095629157091	2.29811507936508	5.48058957857588
12	300	negative regulation of protein modification process
GO:0002275	0.00973668034681133	5.18131868131868	0.840357068714968
4	46	myeloid cell activation involved in immune response
Tissue: Whole_Blood=>Thyroid	Type:	cluster
SourceGene:	7SK(ENSG00000223039.1)
TargetGeneSet:	RP11	ERRFI1	NMNAT1	DFFA	DNAJC16	OTUD3	XKR8	MED18
PRDX3P2	FAM167B	HDAC1	ZMYM1	RNF220	HHLA3	DDAH1	FRRS1	CYB561D1
DDX20	BCL9	GJA5	BOLA1	GOLPH3L	ARNT	VPS72	SHE	LAMTOR2	MNDA
SLAMF8	USP21	FCGR3A	RNASEL	SMG7	TRMT1L	ZNF281	DISP1	MIXL1	C2orf44	PREB
EHD3	ARHGEF33		MSH6	PAPOLG	SPR	MRPL35	ZNF2	C2orf29	POLR1B	ORC2
ICA1L	PARD3B	IDH1	SPAG16	TMEM169	TNP1	MRPL44	EIF4E2	RNPEPL1	RPUSD3
CCRL1	ZNF619	ZNF620	CCR2	DUSP7	SCT	ABHD10	WDR52	ADPRH	MSL2
SLC25A36		ZBTB38	TRPC1	SIAH2	P2RY13	TACC3	SH3BP2	TMEM128	ZNF518B
NFXL1	UTP3	IL8	CASP6	ANKRD50	TIGD4	CCDC127	LPCAT1	BRIP1	DAP
C5orf51	SLC38A9	TRIM23	CD180	CETN3	SLC22A5	AFF4	SH3RF2	TCERG1	HMGXB3
C5orf54	NEURL1B	DOK3	CTB	HIST1H3E		ZKSCAN4	ZKSCAN3	SCAND3	MICB	APOM
PFDN6	ZBTB9	NFYA	TAF8	C6orf203		TSPYL4	AKAP12	FAM103A2P
ELFN1	RNF216P1		COL28A1	TRA2A	VOPP1	LAT2	TMEM60	SLC12A9	GCC1
TNPO3	FAM131B	EPHA1	ZNF786	ZNF398	GIMAP8	GIMAP7	GIMAP4	ESYT2	CTA
SH2D4A	CTD	INTS9	PDP1	WDR67	KLHL38	ZNF623	TONSL	ZNF250	RANBP6
KLHL9	DCAF10	C9orf85	C9orf40	RMI1	FOXE1	RNF20	C9orf80	ATP6V1G1
TRIM32	PSMD5	LHX6	ZBTB34	NAIF1	ABL1	GTF3C4	QSOX2	NUDT5	C10orf128
ERCC6	ADO	MSS51	AP3M1	ARHGAP19		HIF1AN	C10orf95		SUFU
DUSP5	SMC3	CASC2	DUSP8	TPH1	SLC35C1	FAM180B	HEXIM1	TMEM132A
TTC9C	ZBTB3	MEN1	EIF1AD	C11orf82		ANKRD42	PICALM	RDX	DPAGT1
ZNF202	HYLS1	KCNJ1	RAD52	NCAPD2	BCDIN3D	SMUG1	ZC3H10	ZBTB39	DYRK2
ATXN7L3B		CSRP2	TMPO	RFC5	SRRM4	RNF34	ORAI1	RNF6	FAM48A
KBTBD6	KBTBD7	ARL11	SUGT1	FARP1-IT1		LIG4	RNASE2	ACIN1	C14orf119
SYNJ2BP	MAP3K9	PROX2	VASH1	ZC3H14	RPS6KA5	BTBD7	PGBD4	ZFYVE19	ZFP106
ZSCAN29	HDC	RNF111	LACTB	DIS3L	ANP32A	PEX11A	C15orf38		NR2F2
LRRC28	ASB7	ZNF434	TFAP4	C16orf88		SPN	TAOK2	DDX19B	PLCG2
SLC16A13		TNK1	SNORA48	KRBA2	PIGL	BLMH	RNF135	CCT6B	PEX12
FAM134C	GJC1	HIGD1B	TBKBP1	DLX3	PPP1R9B	CHAD	HEATR6	APPBP2	FTSJ3
CD79B	QRICH2	CBX2	LIPG	ZNF555	PIAS4	TNFSF9	ZNF557	ZNF799	ZNF443
ZNF490	LYL1	JUND	LRRC25	SUGP1	ZNF90	ZNF486	KCTD15	ZNF792	U2AF1L4
LIN37	CAPNS1	ZFP82	ZNF260	ZNF420	ZFP36	EID2	LTBP4	MIA	ZNF526
ZNF284	ZNF225	BLOC1S3	RSPH6A	IGFL4	PPP1R15A		NAPSA	ZNF616	ZNF816
ZNF677	ZNF765	ZNF835	ZNF264	ZNF547	ZNF17	ZNF549	ZNF550	FAM110A	ABHD12	NANP
DNMT3B	PXMP4	LBP	ZSWIM3	ZSWIM1	ZFP64	CSTF1	C20orf151		TCFL5
SOX18	RCAN1	C2CD2	C22orf29		CRKL	PPM1F	ZNF70	PRR14L	MFNG	TLR7
TLR8	SCML1	EIF1AX	ZFX	RBM3	FAM123B	VSIG4	FHL1	IDS
GOBPID	Pvalue	OddsRatio	ExpCount	Count	Size	Term
GO:0006469	0.000279990173208074	4.19201861130995	2.59414573385925
10	142	negative regulation of protein kinase activity
GO:0010332	0.000353325705541072	9.4248435627746	0.621133485571933	5
34	response to gamma radiation
GO:0033673	0.000457566102139861	3.92192997151952	2.75856342121652

10	151	negative regulation of kinase activity
GO:0033233	0.000466732731923299	27.1666666666667	0.164417687357276
3	9	regulation of protein sumoylation
GO:0071901	0.000742358201208229	5.12498054474708	1.49802781814407
7	82	negative regulation of protein serine/threonine kinase activity
GO:0051348	0.000831268473669817	3.61123977149915	2.97778700435956
10	163	negative regulation of transferase activity
GO:0045356	0.00194710386073015	54.1412213740458	0.073074527714345
2	4	positive regulation of interferon-alpha biosynthetic process
GO:0001933	0.00223963189042068	3.13417143879742	3.39796553871704
10	186	negative regulation of protein phosphorylation
GO:0000122	0.00240816445650831	2.29541912146729	7.85551172929209
17	430	negative regulation of transcription from RNA polymerase II promoter
GO:0002282	0.00320605995974029	36.0916030534351	0.0913431596429313
2	5	microglial cell activation involved in immune response
GO:0045349	0.00320605995974029	36.0916030534351	0.0913431596429313
2	5	interferon-alpha biosynthetic process
GO:0045354	0.00320605995974029	36.0916030534351	0.0913431596429313
2	5	regulation of interferon-alpha biosynthetic process
GO:0042326	0.00351530233284685	2.9316049589546 3.61718912186008	10
198	negative regulation of phosphorylation
GO:0010212	0.0036502089730963	3.7986670262357 1.97301224828732	7
108	response to ionizing radiation
GO:0045736	0.0040144883875776	10.8597701149425	0.328835374714553
3	18	negative regulation of cyclin-dependent protein kinase activity
GO:0010563	0.00419302704988163	2.85463669372935	3.70853228150301
10	203	negative regulation of phosphorus metabolic process
GO:0045936	0.00419302704988163	2.85463669372935	3.70853228150301
10	203	negative regulation of phosphate metabolic process
GO:0000244	0.00475121374870953	27.0667938931298	0.109611791571518
2	6	assembly of spliceosomal tri-snRNP
GO:0007256	0.00475121374870953	27.0667938931298	0.109611791571518
2	6	activation of JNKK activity
GO:0008630	0.00493330491000203	6.40407239819005	0.694208013286278
4	38	DNA damage response, signal transduction resulting in induction of apoptosis
GO:0033235	0.00657176753387678	21.6519083969466	0.127880423500104
2	7	positive regulation of protein sumoylation
GO:0035970	0.00657176753387678	21.6519083969466	0.127880423500104
2	7	peptidyl-threonine dephosphorylation
GO:0045359	0.00657176753387678	21.6519083969466	0.127880423500104
2	7	positive regulation of interferon-beta biosynthetic process
GO:0002263	0.00739237613492882	3.30393801153898	2.24704172721611
7	123	cell activation involved in immune response
GO:0002366	0.00739237613492882	3.30393801153898	2.24704172721611
7	123	leukocyte activation involved in immune response
GO:0000012	0.00865722004551591	18.0419847328244	0.14614905542869
2	8	single strand break repair
GO:0045350	0.00865722004551591	18.0419847328244	0.14614905542869
2	8	interferon-beta biosynthetic process
GO:0045357	0.00865722004551591	18.0419847328244	0.14614905542869
2	8	regulation of interferon-beta biosynthetic process
GO:0045416	0.00865722004551591	18.0419847328244	0.14614905542869
2	8	positive regulation of interleukin-8 biosynthetic process
GO:0031400	0.00926095629157091	2.29811507936508	5.48058957857588
12	300	negative regulation of protein modification process
GO:0002275	0.00973668034681133	5.18131868131868	0.840357068714968
4	46	myeloid cell activation involved in immune response
Tissue: Whole_Blood=>Thyroid	Type:	asymmetric
SourceGene:	AMFR(ENSG00000159461.9)
TargetGeneSet:	AKR7A2	C1QA	C1QC	C1QB	LAPTM5	AK2	ADORA3	RPRD2	RP11
SLAMF1	RRM2	CAPN14	PLEK	ZNF638-IT1	UBTFL6	WDR75	C3orf38	C3orf26
ILDR1	CD86	TFDP2	GP5	TADA2B	CTD	CD180	SERINC5	STARD4	APC

CSF1R HAVCR2 C5orf58 F13A1 C6orf47 ZBTB22 TBCC SGK1 SRP72P2 CCZ1
NCF1B TFEC GCC1 TBXAS1 ZNF767 INSIG1 NAIF1 SETP5 IL2RA HSD17B7P2
CEP55 O3FAR1 RHOG MTRNR2L8 FAM111B MS4A6A MS4A4E MS4A7 YIF1A
FOLR2 CWF19L2 IL18 MCAM CD163 ARID2 NCKAP1L C12orf56 KRT8P22
SELPLG HVCN1 EXOSC8 RNASE6 ALDH6A1 PSTPIP1 C15orf58 IGSF6 APOBR
ITGAM SHCBP1 AMFR MT3 CTB MLYCD PIK3R5 CDK5RAP3 PDK2
GNA15 EBI3 VAV1 MYO1F LRRC25 POU2F2 CD33 LILRA2 ZNF8 FKBP1A
CRLS1 7SK NCOA3 ITGB2 TOB2 GPR34 RP6 WAS ACTG1P10 ZXDB
VSIG4 RPSAP14 XIAP SASH3
GOBPID Pvalue OddsRatio ExpCount Count Size Term
GO:0046649 5.72667249465935e-07 6.54314574314574 2.36710262265587
13 433 lymphocyte activation
GO:0042110 1.21150996426741e-06 7.38655462184874 1.74389315618296
11 319 T cell activation
GO:0046635 3.51422065811752e-06 25.4871977240398 0.235070237353816
5 43 positive regulation of alpha-beta T cell activation
GO:0046634 1.20664215297118e-05 19.3540540540541 0.300671233824649
5 55 regulation of alpha-beta T cell activation
GO:0050865 1.67618630246928e-05 6.11002306654481 1.87509514912463
10 343 regulation of cell activation
GO:0042129 1.84692614533867e-05 12.3521268925739 0.552141720296173
6 101 regulation of T cell proliferation
GO:0050863 2.70602554461943e-05 7.49004827084573 1.2081516850045 8
221 regulation of T cell activation
GO:0042102 2.95788295713274e-05 15.8517944173682 0.360805480589579
5 66 positive regulation of T cell proliferation
GO:0002521 3.85262040908757e-05 6.17801072647489 1.65095841118262
9 302 leukocyte differentiation
GO:0050870 4.06885179853054e-05 8.42276422764228 0.934814199709363
7 171 positive regulation of T cell activation
GO:0042098 4.46166255895924e-05 10.4647749510763 0.645076465296519
6 118 T cell proliferation
GO:0002252 4.58983233867896e-05 5.39469626888683 2.11016538647844
10 386 immune effector process
GO:0050867 4.86618942058087e-05 6.86741136474016 1.31201992941665
8 240 positive regulation of cell activation
GO:0006959 5.3792325400273e-05 10.1010864430798 0.66694346412013
6 122 humoral immune response
GO:0002460 5.83890837826231e-05 7.9331098339719 0.98948169676839 7
181 adaptive immune response based on somatic recombination of immune receptors built from immunoglobulin superfamily domains
GO:0007596 7.9140657730722e-05 4.6121210291098 2.72244135353955 11
498 blood coagulation
GO:0046631 8.43596319850862e-05 12.543875043875 0.448273475884022 5
82 alpha-beta T cell activation
GO:0072376 8.43596319850862e-05 12.543875043875 0.448273475884022 5
82 protein activation cascade
GO:0032623 9.51104621521364e-05 19.1093333333333 0.240536987059719
4 44 interleukin-2 production
GO:0043372 0.000101184972910591 40.4830827067669 0.092934745000346
3 17 positive regulation of CD4-positive, alpha-beta T cell differentiation
GO:0002250 0.000102678435140264 7.21836823734729 1.08241644176874
7 198 adaptive immune response
GO:0050670 0.000134847218798117 8.47766527695057 0.78721195764999
6 144 regulation of lymphocyte proliferation
GO:0032944 0.000140061736785277 8.41608357149897 0.792678707355892
6 145 regulation of mononuclear cell proliferation
GO:2000516 0.000143056593367425 35.4177631578947 0.103868244412151
3 19 positive regulation of CD4-positive, alpha-beta T cell activation
GO:0002443 0.000152438472788205 6.75217864923747 1.15348418794547
7 211 leukocyte mediated immunity
GO:0051249 0.000153408109780161 5.7759795134443 1.54709016677047 8

283	regulation of lymphocyte activation
GO:0050778	0.000160843711861014	5.07657947686117	1.98989689294858
9	364	positive regulation of immune response
GO:0070663	0.000168584536111386	8.12100456621005	0.820012455885406
6	150	regulation of leukocyte proliferation
GO:0051251	0.00017117417106622	6.6204594017094 1.17535118676908	7
215	positive regulation of lymphocyte activation
GO:0050671	0.000178056376220367	10.6036531036531	0.52480797176666
5	96	positive regulation of lymphocyte proliferation
GO:0030168	0.000181218179527848	6.55648148148148	1.18628468618089
7	217	platelet activation
GO:0032946	0.000186937031774168	10.4876615746181	0.530274721472562
5	97	positive regulation of mononuclear cell proliferation
GO:0045582	0.000197657353137294	15.5896598639456	0.289737734412843
4	53	positive regulation of T cell differentiation
GO:0006954	0.000202253503132725	4.46325509811466	2.52563836412705
10	462	inflammatory response
GO:0030097	0.000205812297407556	4.45308250951787	2.53110511383295
10	463	hemopoiesis
GO:0070665	0.000215617880731047	10.1543385490754	0.546674970590271
5	100	positive regulation of leukocyte proliferation
GO:0002253	0.000223274281062664	5.45220463675524	1.63455816206491
8	299	activation of immune response
GO:0002696	0.000279994646039632	6.08542281219272	1.27375268147533
7	233	positive regulation of leukocyte activation
GO:0048534	0.000351097338747098	4.14970865082922	2.70604110442184
10	495	hemopoietic or lymphoid organ development
GO:0046651	0.000353095449693844	7.03383396600099	0.940280949415265
6	172	lymphocyte proliferation
GO:0002694	0.000360174358177522	5.0610628627998 1.75482665559477	8
321	regulation of leukocyte activation
GO:0045621	0.000362790922793897	13.1622988505747	0.338938481765968
4	62	positive regulation of lymphocyte differentiation
GO:0032943	0.000375591082620152	6.94911937377691	0.951214448827071
6	174	mononuclear cell proliferation
GO:0006956	0.000385789434511847	12.9383050847458	0.34440523147187
4	63	complement activation
GO:0043370	0.00041843949134889	23.5986842105263	0.147602242059373
3	27	regulation of CD4-positive, alpha-beta T cell differentiation
GO:0070661	0.000477030694398031	6.62951432129514	0.994948446474292
6	182	leukocyte proliferation
GO:0007229	0.000516664230974918	11.9233333333333	0.371738980001384
4	68	integrin-mediated signaling pathway
GO:2000514	0.000518632478943084	21.7803643724696	0.158535741471178
3	29	regulation of CD4-positive, alpha-beta T cell activation
GO:0046638	0.000573997426413046	20.9722222222222	0.164002491177081
3	30	positive regulation of alpha-beta T cell differentiation
GO:0032515	0.000608769608858003	74.6337662337662	0.0382672479413189
2	7	negative regulation of phosphoprotein phosphatase activity
GO:0032101	0.000739919289285456	4.51412474849095	1.95709639471317
8	358	regulation of response to external stimulus
GO:0001817	0.00075347948560816	4.50094297981622	1.96256314441907
8	359	regulation of cytokine production
GO:0030098	0.000868287961127102	5.88376919883769	1.11521694000415
6	204	lymphocyte differentiation
GO:0045580	0.00104736885590511	9.77367521367521	0.448273475884022
4	82	regulation of T cell differentiation
GO:0032663	0.00124719428053958	15.719298245614 0.213203238530206	3
39	regulation of interleukin-2 production
GO:0046637	0.00134303976147446	15.2933854907539	0.218669988236108
3	40	regulation of alpha-beta T cell differentiation
GO:0001816	0.00139261489937974	4.07176911598792	2.15936613383157
8	395	cytokine production

GO:0043367 0.00144339767295301 14.8898891966759 0.224136737942011
3 41 CD4-positive, alpha-beta T cell differentiation
GO:0030217 0.0016299378764811 6.36343296939323 0.852812954120822
5 156 T cell differentiation
GO:0006801 0.00165795990725779 14.1434210526316 0.235070237353816
3 43 superoxide metabolic process
GO:0035710 0.00165795990725779 14.1434210526316 0.235070237353816
3 43 CD4-positive, alpha-beta T cell activation
GO:0045619 0.00210180443756686 8.01515789473684 0.541208220884368
4 99 regulation of lymphocyte differentiation
GO:0002449 0.00219684706663474 5.92676009342676 0.912947200885752
5 167 lymphocyte mediated immunity
GO:0006958 0.0025619684878847 12.0310750279955 0.273337485295135
3 50 complement activation, classical pathway
GO:0006085 0.00257329522717421 31.0822510822511 0.0765344958826379
2 14 acetyl-CoA biosynthetic process
GO:0046641 0.00257329522717421 31.0822510822511 0.0765344958826379
2 14 positive regulation of alpha-beta T cell proliferation
GO:0030316 0.00286623451783341 11.5383995703545 0.284270984706941
3 52 osteoclast differentiation
GO:0016064 0.00318303113197412 7.11028037383178 0.6068092173552 4
111 immunoglobulin mediated immune response
GO:0019724 0.00350309245351609 6.91490909090909 0.623209466472908
4 114 B cell mediated immunity
GO:0030866 0.0038051697756915 24.8606060606061 0.092934745000346
2 17 cortical actin cytoskeleton organization
GO:0042095 0.0038051697756915 24.8606060606061 0.092934745000346
2 17 interferon-gamma biosynthetic process
GO:0045637 0.00396300212636255 6.67040935672515 0.645076465296519
4 118 regulation of myeloid cell differentiation
GO:0015949 0.0042656979540683 23.3051948051948 0.0984014947062487
2 18 nucleobase-containing small molecule interconversion
GO:0032620 0.0042656979540683 23.3051948051948 0.0984014947062487
2 18 interleukin-17 production
GO:0032660 0.0042656979540683 23.3051948051948 0.0984014947062487
2 18 regulation of interleukin-17 production
GO:0042104 0.0042656979540683 23.3051948051948 0.0984014947062487
2 18 positive regulation of activated T cell proliferation
GO:0046640 0.0042656979540683 23.3051948051948 0.0984014947062487
2 18 regulation of alpha-beta T cell proliferation
GO:0032649 0.00471707493739376 9.57604817127565 0.338938481765968
3 62 regulation of interferon-gamma production
GO:0030865 0.00475071458473899 21.9327731092437 0.103868244412151
2 19 cortical cytoskeleton organization
GO:0002573 0.00486334739263922 6.28143250688705 0.683343713237838
4 125 myeloid leukocyte differentiation
GO:0002455 0.00538456951437865 9.11078098471986 0.355338730883676
3 65 humoral immune response mediated by circulating immunoglobulin
GO:0046632 0.00538456951437865 9.11078098471986 0.355338730883676
3 65 alpha-beta T cell differentiation
GO:0002446 0.00579311705311845 19.6213260423787 0.114801743823957
2 21 neutrophil mediated immunity
GO:0031529 0.00579311705311845 19.6213260423787 0.114801743823957
2 21 ruffle organization
GO:0042094 0.00579311705311845 19.6213260423787 0.114801743823957
2 21 interleukin-2 biosynthetic process
GO:0046633 0.00579311705311845 19.6213260423787 0.114801743823957
2 21 alpha-beta T cell proliferation
GO:0032609 0.00585952701430043 8.82483552631579 0.366272230295481
3 67 interferon-gamma production
GO:0010543 0.00634996031081422 18.638961038961 0.12026849352986 2
22 regulation of platelet activation
GO:0030225 0.00634996031081422 18.638961038961 0.12026849352986 2

22	macrophage differentiation
GO:0032673	0.00693020683008556	17.7501546072975	0.125735243235762
2	23	regulation of interleukin-4 production
GO:0042554	0.00693020683008556	17.7501546072975	0.125735243235762
2	23	superoxide anion generation
GO:0043029	0.00693020683008556	17.7501546072975	0.125735243235762
2	23	T cell homeostasis
GO:0002761	0.00743179406559731	8.06503759398496	0.399072728530898
3	73	regulation of myeloid leukocyte differentiation
GO:0071706	0.00743179406559731	8.06503759398496	0.399072728530898
3	73	tumor necrosis factor superfamily cytokine production
GO:0046006	0.00753359074695003	16.9421487603306	0.131201992941665
2	24	regulation of activated T cell proliferation
GO:0030099	0.00804466067507511	4.30667153640127	1.24095218323991
5	227	myeloid cell differentiation
GO:0030858	0.00815984834055658	16.2044042913608	0.136668742647568
2	25	positive regulation of epithelial cell differentiation
GO:0032855	0.00815984834055658	16.2044042913608	0.136668742647568
2	25	positive regulation of Rac GTPase activity
GO:0042092	0.00815984834055658	16.2044042913608	0.136668742647568
2	25	type 2 immune response
GO:0043666	0.00815984834055658	16.2044042913608	0.136668742647568
2	25	regulation of phosphoprotein phosphatase activity
GO:0050798	0.00880871801818296	15.5281385281385	0.14213549235347
2	26	activated T cell proliferation
GO:0051004	0.00880871801818296	15.5281385281385	0.14213549235347
2	26	regulation of lipoprotein lipase activity
GO:0032633	0.00947994030032968	14.905974025974	0.147602242059373	2
27	interleukin-4 production
GO:0032722	0.00947994030032968	14.905974025974	0.147602242059373	2
27	positive regulation of chemokine production
GO:0042113	0.00982247367293868	5.0910067114094	0.836412705003114	4
153	B cell activation
Tissue: Whole_Blood=>Thyroid	Type:	cluster
SourceGene:	AMFR(ENSG00000159461.9)
TargetGeneSet:	AKR7A2	C1QA	C1QC	C1QB	LAPTM5	AK2	ADORA3	RPRD2	RP11
SLAMF1	RRM2	CAPN14	PLEK	ZNF638-IT1	UBTFL6	WDR75	C3orf38	C3orf26
ILDR1	CD86	TFDP2	GP5	TADA2B	CTD	CD180	SERINC5	STARD4	APC
CSF1R	HAVCR2	C5orf58	F13A1	C6orf47	ZBTB22	TBCC	SGK1	SRP72P2	CCZ1
NCF1B	TFEC	GCC1	TBXAS1	ZNF767	INSIG1	NAIF1	SETP5	IL2RA	HSD17B7P2
CEP55	O3FAR1	RHOG	MTRNR2L8	FAM111B	MS4A6A	MS4A4E	MS4A7	YIF1A
FOLR2	CWF19L2	IL18	MCAM	CD163	ARID2	NCKAP1L	C12orf56	KRT8P22
SELPLG	HVCN1	EXOSC8	RNASE6	ALDH6A1	PSTPIP1	C15orf58	IGSF6	APOBR
ITGAM	SHCBP1	AMFR	MT3	CTB	MLYCD	PIK3R5	CDK5RAP3	PDK2
GNA15	EBI3	VAV1	MYO1F	LRRC25	POU2F2	CD33	LILRA2	ZNF8	FKBP1A
CRLS1	7SK	NCOA3	ITGB2	TOB2	GPR34	RP6	WAS	ACTG1P10	ZXDB
VSIG4	RPSAP14	XIAP	SASH3
GOBPID	Pvalue	OddsRatio	ExpCount	Count	Size	Term
GO:0046649	5.72667249465935e-07	6.54314574314574	2.36710262265587
13	433	lymphocyte activation
GO:0042110	1.21150996426741e-06	7.38655462184874	1.74389315618296
11	319	T cell activation
GO:0046635	3.51422065811752e-06	25.4871977240398	0.235070237353816
5	43	positive regulation of alpha-beta T cell activation
GO:0046634	1.20664215297118e-05	19.3540540540541	0.300671233824649
5	55	regulation of alpha-beta T cell activation
GO:0050865	1.67618630246928e-05	6.11002306654481	1.87509514912463
10	343	regulation of cell activation
GO:0042129	1.84692614533867e-05	12.3521268925739	0.552141720296173
6	101	regulation of T cell proliferation
GO:0050863	2.70602554461943e-05	7.49004827084573	1.2081516850045 8
221	regulation of T cell activation
GO:0042102	2.95788295713274e-05	15.8517944173682	0.360805480589579

5	66	positive regulation of T cell proliferation
GO:0002521	3.85262040908757e-05	6.17801072647489	1.65095841118262
9	302	leukocyte differentiation
GO:0050870	4.06885179853054e-05	8.42276422764228	0.934814199709363
7	171	positive regulation of T cell activation
GO:0042098	4.46166255895924e-05	10.4647749510763	0.645076465296519
6	118	T cell proliferation
GO:0002252	4.58983233867896e-05	5.39469626888683	2.11016538647844
10	386	immune effector process
GO:0050867	4.86618942058087e-05	6.86741136474016	1.31201992941665
8	240	positive regulation of cell activation
GO:0006959	5.3792325400273e-05	10.1010864430798	0.66694346412013
6	122	humoral immune response
GO:0002460	5.83890837826231e-05	7.9331098339719 0.98948169676839	7
181	adaptive immune response based on somatic recombination of immune receptors built from immunoglobulin superfamily domains
GO:0007596	7.9140657730722e-05	4.6121210291098 2.72244135353955	11
498	blood coagulation
GO:0046631	8.43596319850862e-05	12.543875043875 0.448273475884022	5
82	alpha-beta T cell activation
GO:0072376	8.43596319850862e-05	12.543875043875 0.448273475884022	5
82	protein activation cascade
GO:0032623	9.51104621521364e-05	19.1093333333333	0.240536987059719
4	44	interleukin-2 production
GO:0043372	0.000101184972910591	40.4830827067669	0.092934745000346
3	17	positive regulation of CD4-positive, alpha-beta T cell differentiation
GO:0002250	0.000102678435140264	7.21836823734729	1.08241644176874
7	198	adaptive immune response
GO:0050670	0.000134847218798117	8.47766527695057	0.78721195764999
6	144	regulation of lymphocyte proliferation
GO:0032944	0.000140061736785277	8.41608357149897	0.792678707355892
6	145	regulation of mononuclear cell proliferation
GO:2000516	0.000143056593367425	35.4177631578947	0.103868244412151
3	19	positive regulation of CD4-positive, alpha-beta T cell activation
GO:0002443	0.000152438472788205	6.75217864923747	1.15348418794547
7	211	leukocyte mediated immunity
GO:0051249	0.000153408109780161	5.7759795134443 1.54709016677047	8
283	regulation of lymphocyte activation
GO:0050778	0.000160843711861014	5.07657947686117	1.98989689294858
9	364	positive regulation of immune response
GO:0070663	0.000168584536111386	8.12100456621005	0.820012455885406
6	150	regulation of leukocyte proliferation
GO:0051251	0.00017117417106622	6.6204594017094 1.17535118676908	7
215	positive regulation of lymphocyte activation
GO:0050671	0.000178056376220367	10.6036531036531	0.52480797176666
5	96	positive regulation of lymphocyte proliferation
GO:0030168	0.000181218179527848	6.55648148148148	1.18628468618089
7	217	platelet activation
GO:0032946	0.000186937031774168	10.4876615746181	0.530274721472562
5	97	positive regulation of mononuclear cell proliferation
GO:0045582	0.000197657353137294	15.5896598639456	0.289737734412843
4	53	positive regulation of T cell differentiation
GO:0006954	0.000202253503132725	4.46325509811466	2.52563836412705
10	462	inflammatory response
GO:0030097	0.000205812297407556	4.45308250951787	2.53110511383295
10	463	hemopoiesis
GO:0070665	0.000215617880731047	10.1543385490754	0.546674970590271
5	100	positive regulation of leukocyte proliferation
GO:0002253	0.000223274281062664	5.45220463675524	1.63455816206491
8	299	activation of immune response
GO:0002696	0.000279994646039632	6.08542281219272	1.27375268147533
7	233	positive regulation of leukocyte activation

GO:0048534 0.000351097338747098 4.14970865082922 2.70604110442184
10 495 hemopoietic or lymphoid organ development
GO:0046651 0.000353095449693844 7.03383396600099 0.940280949415265
6 172 lymphocyte proliferation
GO:0002694 0.000360174358177522 5.0610628627998 1.75482665559477 8
321 regulation of leukocyte activation
GO:0045621 0.000362790922793897 13.1622988505747 0.338938481765968
4 62 positive regulation of lymphocyte differentiation
GO:0032943 0.000375591082620152 6.94911937377691 0.951214448827071
6 174 mononuclear cell proliferation
GO:0006956 0.000385789434511847 12.9383050847458 0.34440523147187
4 63 complement activation
GO:0043370 0.00041843949134889 23.5986842105263 0.147602242059373
3 27 regulation of CD4-positive, alpha-beta T cell differentiation
GO:0070661 0.000477030694398031 6.62951432129514 0.994948446474292
6 182 leukocyte proliferation
GO:0007229 0.000516664230974918 11.9233333333333 0.371738980001384
4 68 integrin-mediated signaling pathway
GO:2000514 0.000518632478943084 21.7803643724696 0.158535741471178
3 29 regulation of CD4-positive, alpha-beta T cell activation
GO:0046638 0.000573997426413046 20.9722222222222 0.164002491177081
3 30 positive regulation of alpha-beta T cell differentiation
GO:0032515 0.000608769608858003 74.6337662337662 0.0382672479413189
2 7 negative regulation of phosphoprotein phosphatase activity
GO:0032101 0.000739919289285456 4.51412474849095 1.95709639471317
8 358 regulation of response to external stimulus
GO:0001817 0.00075347948560816 4.50094297981622 1.96256314441907
8 359 regulation of cytokine production
GO:0030098 0.000868287961127102 5.88376919883769 1.11521694000415
6 204 lymphocyte differentiation
GO:0045580 0.00104736885590511 9.77367521367521 0.448273475884022
4 82 regulation of T cell differentiation
GO:0032663 0.00124719428053958 15.719298245614 0.213203238530206 3
39 regulation of interleukin-2 production
GO:0046637 0.00134303976147446 15.2933854907539 0.218669988236108
3 40 regulation of alpha-beta T cell differentiation
GO:0001816 0.00139261489937974 4.07176911598792 2.15936613383157
8 395 cytokine production
GO:0043367 0.00144339767295301 14.8898891966759 0.224136737942011
3 41 CD4-positive, alpha-beta T cell differentiation
GO:0030217 0.0016299378764811 6.36343296939323 0.852812954120822
5 156 T cell differentiation
GO:0006801 0.00165795990725779 14.1434210526316 0.235070237353816
3 43 superoxide metabolic process
GO:0035710 0.00165795990725779 14.1434210526316 0.235070237353816
3 43 CD4-positive, alpha-beta T cell activation
GO:0045619 0.00210180443756686 8.01515789473684 0.541208220884368
4 99 regulation of lymphocyte differentiation
GO:0002449 0.00219684706663474 5.92676009342676 0.912947200885752
5 167 lymphocyte mediated immunity
GO:0006958 0.0025619684878847 12.0310750279955 0.273337485295135
3 50 complement activation, classical pathway
GO:0006085 0.00257329522717421 31.0822510822511 0.0765344958826379
2 14 acetyl-CoA biosynthetic process
GO:0046641 0.00257329522717421 31.0822510822511 0.0765344958826379
2 14 positive regulation of alpha-beta T cell proliferation
GO:0030316 0.00286623451783341 11.5383995703545 0.284270984706941
3 52 osteoclast differentiation
GO:0016064 0.00318303113197412 7.11028037383178 0.6068092173552 4
111 immunoglobulin mediated immune response
GO:0019724 0.00350309245351609 6.91490909090909 0.623209466472908
4 114 B cell mediated immunity
GO:0030866 0.0038051697756915 24.8606060606061 0.092934745000346

2 17 cortical actin cytoskeleton organization
GO:0042095 0.0038051697756915 24.8606060606061 0.092934745000346
2 17 interferon-gamma biosynthetic process
GO:0045637 0.00396300212636255 6.67040935672515 0.645076465296519
4 118 regulation of myeloid cell differentiation
GO:0015949 0.0042656979540683 23.3051948051948 0.0984014947062487
2 18 nucleobase-containing small molecule interconversion
GO:0032620 0.0042656979540683 23.3051948051948 0.0984014947062487
2 18 interleukin-17 production
GO:0032660 0.0042656979540683 23.3051948051948 0.0984014947062487
2 18 regulation of interleukin-17 production
GO:0042104 0.0042656979540683 23.3051948051948 0.0984014947062487
2 18 positive regulation of activated T cell proliferation
GO:0046640 0.0042656979540683 23.3051948051948 0.0984014947062487
2 18 regulation of alpha-beta T cell proliferation
GO:0032649 0.00471707493739376 9.57604817127565 0.338938481765968
3 62 regulation of interferon-gamma production
GO:0030865 0.00475071458473899 21.9327731092437 0.103868244412151
2 19 cortical cytoskeleton organization
GO:0002573 0.00486334739263922 6.28143250688705 0.683343713237838
4 125 myeloid leukocyte differentiation
GO:0002455 0.00538456951437865 9.11078098471986 0.355338730883676
3 65 humoral immune response mediated by circulating immunoglobulin
GO:0046632 0.00538456951437865 9.11078098471986 0.355338730883676
3 65 alpha-beta T cell differentiation
GO:0002446 0.00579311705311845 19.6213260423787 0.114801743823957
2 21 neutrophil mediated immunity
GO:0031529 0.00579311705311845 19.6213260423787 0.114801743823957
2 21 ruffle organization
GO:0042094 0.00579311705311845 19.6213260423787 0.114801743823957
2 21 interleukin-2 biosynthetic process
GO:0046633 0.00579311705311845 19.6213260423787 0.114801743823957
2 21 alpha-beta T cell proliferation
GO:0032609 0.00585952701430043 8.82483552631579 0.366272230295481
3 67 interferon-gamma production
GO:0010543 0.00634996031081422 18.638961038961 0.12026849352986 2
22 regulation of platelet activation
GO:0030225 0.00634996031081422 18.638961038961 0.12026849352986 2
22 macrophage differentiation
GO:0032673 0.00693020683008556 17.7501546072975 0.125735243235762
2 23 regulation of interleukin-4 production
GO:0042554 0.00693020683008556 17.7501546072975 0.125735243235762
2 23 superoxide anion generation
GO:0043029 0.00693020683008556 17.7501546072975 0.125735243235762
2 23 T cell homeostasis
GO:0002761 0.00743179406559731 8.06503759398496 0.399072728530898
3 73 regulation of myeloid leukocyte differentiation
GO:0071706 0.00743179406559731 8.06503759398496 0.399072728530898
3 73 tumor necrosis factor superfamily cytokine production
GO:0046006 0.00753359074695003 16.9421487603306 0.131201992941665
2 24 regulation of activated T cell proliferation
GO:0030099 0.00804466067507511 4.30667153640127 1.24095218323991
5 227 myeloid cell differentiation
GO:0030858 0.00815984834055658 16.2044042913608 0.136668742647568
2 25 positive regulation of epithelial cell differentiation
GO:0032855 0.00815984834055658 16.2044042913608 0.136668742647568
2 25 positive regulation of Rac GTPase activity
GO:0042092 0.00815984834055658 16.2044042913608 0.136668742647568
2 25 type 2 immune response
GO:0043666 0.00815984834055658 16.2044042913608 0.136668742647568
2 25 regulation of phosphoprotein phosphatase activity
GO:0050798 0.00880871801818296 15.5281385281385 0.14213549235347
2 26 activated T cell proliferation

GO:0051004 0.00880871801818296 15.5281385281385 0.14213549235347
2 26 regulation of lipoprotein lipase activity
GO:0032633 0.00947994030032968 14.905974025974 0.147602242059373 2
27 interleukin-4 production
GO:0032722 0.00947994030032968 14.905974025974 0.147602242059373 2
27 positive regulation of chemokine production
GO:0042113 0.00982247367293868 5.0910067114094 0.836412705003114 4
153 B cell activation
Tissue: Whole_Blood=>Thyroid Type: asymmetric
SourceGene: AMY2A(ENSG00000243480.3)
TargetGeneSet: CELA3B CELA3A CRNN SPRR3 CPB1 PRL CLPS CGA CPA2
CPA1 PRSS1 PNLIP PNLIPRP2 KRT4 PLA2G1B RP11 RHCG GP2
CTRB2 CTRB1 KRTAP3-2 KRTAP11-1 BRD1
GOBPID Pvalue OddsRatio ExpCount Count Size Term
GO:0007586 1.51426135092439e-12 87.3054074638233 0.158397342744447
8 109 digestion
GO:0044241 4.45140475278301e-09 308.427807486631 0.021797799460245
4 15 lipid digestion
GO:0022617 1.83105550247234e-05 72.7121212121212 0.0523147187045879
3 36 extracellular matrix disassembly
GO:0016042 0.000366898473453374 14.0306475531389 0.351671164625285
4 242 lipid catabolic process
GO:0008202 0.000586693720234097 12.3398692810458 0.398173136807141
4 274 steroid metabolic process
GO:0001523 0.0014428580814799 40.9473684210526 0.0566742785966369
2 39 retinoid metabolic process
GO:0030198 0.00155770974328471 15.0548523206751 0.233963047539963
3 161 extracellular matrix organization
GO:0043062 0.00158561961605076 14.9591194968553 0.235416234170646
3 162 extracellular structure organization
GO:0016101 0.00159389296614628 38.8421052631579 0.0595806518580029
2 41 diterpenoid metabolic process
GO:0006721 0.0023624467699341 31.5394736842105 0.0726593315341499
2 50 terpenoid metabolic process
GO:0032275 0.00435352854395898 360.7 0.00435955989204899 1 3
luteinizing hormone secretion
GO:0044240 0.00435352854395898 360.7 0.00435955989204899 1 3
multicellular organismal lipid catabolic process
GO:0070634 0.00435352854395898 360.7 0.00435955989204899 1 3
transepithelial ammonium transport
GO:0072488 0.00435352854395898 360.7 0.00435955989204899 1 3
ammonium transmembrane transport
GO:0006720 0.00483544160740886 21.593984962406 0.104629437409176 2
72 isoprenoid metabolic process
GO:0009791 0.00551129037912381 20.1473684210526 0.111895370562591
2 77 post-embryonic development
GO:0032431 0.00580068911293552 240.45 0.00581274652273199 1 4
activation of phospholipase A2 activity
GO:0006066 0.00641700421826788 8.97782002534854 0.386547643761677
3 266 alcohol metabolic process
GO:0032430 0.00724584627557323 180.325 0.00726593315341499 1 5
positive regulation of phospholipase A2 activity
GO:0030855 0.00753766248507537 8.45340501792115 0.409798629852605
3 282 epithelial cell differentiation
GO:0071845 0.0076854182153757 8.3920521945433 0.412705003113971 3
284 cellular component disassembly at cellular level
GO:0022411 0.00798610278699269 8.2719298245614 0.418517749636703 3
288 cellular component disassembly
GO:0000244 0.00868900266683825 144.25 0.00871911978409799 1 6
assembly of spliceosomal tri-snRNP
GO:0002385 0.00868900266683825 144.25 0.00871911978409799 1 6
mucosal immune response
GO:0015696 0.00868900266683825 144.25 0.00871911978409799 1 6

ammonium transport
GO:0019374 0.00868900266683825 144.25 0.00871911978409799 1 6
galactolipid metabolic process
GO:0045056 0.00868900266683825 144.25 0.00871911978409799 1 6
transcytosis
Tissue: Whole_Blood=>Thyroid Type: asymmetric
SourceGene: CASP10(ENSG00000003400.10)
TargetGeneSet: SF3A3 SELRC1 SETDB1 ADAR RP11 URB2 RHOB WDR43
POLR1B VPRBP RP13 NOP14 SRP72 CCT6P1 MAK16 NCBP1 NGB HNRNPF
NOLC1 BET1L NAT10 WDR74 RERG NUFIP1 EXOC5 NIP7 USP10 DPH1 TSR1
SEZ6 CCDC43 EFTUD2 KPNB1 ATCAY TNFSF14 ZNF317 BRD4 AKAP8 ZNF8
BACH1 BRWD1-IT2 COL18A1 EP300
GOBPID Pvalue OddsRatio ExpCount Count Size Term
GO:0071843 2.21202167880767e-10 23.8259993281827 0.595391322399834
10 239 cellular component biogenesis at cellular level
GO:0022613 2.99435719193124e-09 21.912037037037 0.560514843263442 9
225 ribonucleoprotein complex biogenesis
GO:0022618 1.14307459859597e-07 32.1932584269663 0.236661822711231
6 95 ribonucleoprotein complex assembly
GO:0071826 1.55388656106245e-07 30.4702127659574 0.249117708117085
6 100 ribonucleoprotein complex subunit organization
GO:0000377 1.00603769758897e-05 14.3606060606061 0.508200124558854
6 204 RNA splicing, via transesterification reactions with bulged adenosine as nucleophile
GO:0000398 1.00603769758897e-05 14.3606060606061 0.508200124558854
6 204 nuclear mRNA splicing, via spliceosome
GO:0000375 1.15505591914944e-05 14.0019704433498 0.520656009964708
6 209 RNA splicing, via transesterification reactions
GO:0042254 3.17423559617724e-05 15.9845430107527 0.371185385094457
5 149 ribosome biogenesis
GO:0006397 4.84742666700223e-05 8.63485221674877 0.99397965538717
7 399 mRNA processing
GO:0007000 6.0056443209649e-05 282.588235294118 0.0124558854058543
2 5 nucleolus organization
GO:0008380 0.000131646102897489 8.89463722397476 0.804650197218186
6 323 RNA splicing
GO:0090305 0.000508815473285125 21.75 0.156944156113764 3 63
nucleic acid phosphodiester bond hydrolysis
GO:0042255 0.000708352526025667 60.5084033613445 0.0398588332987337
2 16 ribosome assembly
GO:0006913 0.00107660461177516 7.2668246933938 0.792194311812331 5
318 nucleocytoplasmic transport
GO:0051169 0.00112263877896694 7.19630461412822 0.799667843055844
5 321 nuclear transport
GO:0006606 0.00135078207165987 9.30890052356021 0.485779530828316
4 195 protein import into nucleus
GO:0051170 0.0014289298286985 9.16301546391753 0.493253062071829
4 198 nuclear import
GO:0043388 0.00203655412226881 33.8588235294118 0.067261781191613
2 27 positive regulation of DNA binding
GO:0034504 0.00217167266002722 8.14048165137615 0.553041312019929
4 222 protein localization to nucleus
GO:0006354 0.00242256207847983 12.3896103896104 0.269047124766452
3 108 DNA-dependent transcription, elongation
GO:0000387 0.00268117418933943 29.1805273833671 0.0772264895162964
2 31 spliceosomal snRNP assembly
GO:0042306 0.00296692122864391 11.5060337892196 0.288976541415819
3 116 regulation of protein import into nucleus
GO:1900180 0.00296692122864391 11.5060337892196 0.288976541415819
3 116 regulation of protein localization to nucleus
GO:0000060 0.00380429496468588 24.1680672268908 0.0921735520033216
2 37 protein import into nucleus, translocation
GO:0017038 0.00405042757761231 6.80528846153846 0.657670749429105

4	264	protein import
GO:0046822	0.00473504233258129	9.68860244233379	0.341291260120407
3	137	regulation of nucleocytoplasmic transport
GO:0033157	0.00502965251544117	9.47445255474453	0.348764791363919
3	140	regulation of intracellular protein transport
GO:0070301	0.0051099802034095 5	20.622668579627 0.107120614490347	2
43	cellular response to hydrogen peroxide
GO:0006997	0.00558484740037423	19.6607387140903	0.112102968652688
2	45	nucleus organization
GO:0043967	0.00582960250515155	19.2125668449198	0.114594145733859
2	46	histone H4 acetylation
GO:0033365	0.00599990723060243	4.80665839536807	1.17832675939381
5	473	protein localization to organelle
GO:0006605	0.00605302286071859	4.79606575417842	1.18081793647498
5	474	protein targeting
GO:0031047	0.00607920608121654	18.7843137254902	0.11708532281503
2	47	gene silencing by RNA
GO:0051098	0.00727676494327996	8.25593514765489	0.398588332987337
3	160	regulation of binding
GO:0000491	0.00745544346333527	205.9	0.00747353124351256	1	3
small nucleolar ribonucleoprotein complex assembly
GO:0000492	0.00745544346333527	205.9	0.00747353124351256	1	3
box C/D snoRNP assembly
GO:0044154	0.00745544346333527	205.9	0.00747353124351256	1	3
histone H3-K14 acetylation
GO:0050434	0.00824768231140087	15.9400665926748	0.137014739464397
2	55	positive regulation of viral transcription
GO:0032386	0.00973668760545556	7.3974025974026 0.443429520448412	3
178	regulation of intracellular transport
GO:0000447	0.00992856112912421	137.257142857143	0.00996470832468341
1	4	endonucleolytic cleavage in ITS1 to separate SSU-rRNA from 5.8S rRNA and LSU-rRNA from tricistronic rRNA transcript (SSU-rRNA, 5.8S rRNA, LSU-rRNA)
GO:0000469	0.00992856112912421	137.257142857143	0.00996470832468341
1	4	cleavage involved in rRNA processing
GO:0000478	0.00992856112912421	137.257142857143	0.00996470832468341
1	4	endonucleolytic cleavage involved in rRNA processing
GO:0000479	0.00992856112912421	137.257142857143	0.00996470832468341
1	4	endonucleolytic cleavage of tricistronic rRNA transcript (SSU-rRNA, 5.8S rRNA, LSU-rRNA)
GO:0000966	0.00992856112912421	137.257142857143	0.00996470832468341
1	4	RNA 5'-end processing
GO:0006610	0.00992856112912421	137.257142857143	0.00996470832468341
1	4	ribosomal protein import into nucleus
GO:0008588	0.00992856112912421	137.257142857143	0.00996470832468341
1	4	release of cytoplasmic sequestered NF-kappaB
GO:0018076	0.00992856112912421	137.257142857143	0.00996470832468341
1	4	N-terminal peptidyl-lysine acetylation
GO:0060298	0.00992856112912421	137.257142857143	0.00996470832468341
1	4	positive regulation of sarcomere organization
GO:0061418	0.00992856112912421	137.257142857143	0.00996470832468341
1	4	regulation of transcription from RNA polymerase II promoter in response to hypoxia
GO:2000171	0.00992856112912421	137.257142857143	0.00996470832468341
1	4	negative regulation of dendrite development
Tissue: Thyroid=>Whole_Blood	Type:	asymmetric
SourceGene:	CLPP(ENSG00000125656.3)
TargetGeneSet:	ATRX	KCNN3	TOR3A	BUB1	KIAA1524	NCAPG	CCNA2	RANBP3L	TTK	RP11	FBXO5	EZH2	GINS4	MCM4	MCM10	RRM1	CHEK1	CCNB2	SHCBP1	C2CD2
GOBPID	Pvalue	OddsRatio	ExpCount	Count	Size	Term
GO:0000075	5.71053787162181e-09	42.2948936170213	0.284686180887136
7	242	cell cycle checkpoint
GO:0010564	8.22417850551875e-09	30.7126436781609	0.487025119368902

8	414	regulation of cell cycle process
GO:0071156	1.04358611884653e-08	38.6143968871595	0.310566742785966
7	264	regulation of cell cycle arrest
GO:0045786	1.25557048159008e-08	29.018389018389 0.514082070444952	8
437	negative regulation of cell cycle
GO:0000280	7.26777836398913e-08	28.7571428571429	0.411736212026849
7	350	nuclear division
GO:0007067	7.26777836398913e-08	28.7571428571429	0.411736212026849
7	350	mitosis
GO:0000087	8.81243477332554e-08	27.9226628895184	0.423500103799045
7	360	M phase of mitotic cell cycle
GO:0007050	1.04321868816989e-07	27.2110497237569	0.434087606394021
7	369	cell cycle arrest
GO:0048285	1.06267944644013e-07	27.1341597796143	0.435263995571241
7	370	organelle fission
GO:0045839	1.16683968809599e-07	123.059829059829	0.0470555670887828
4	40	negative regulation of mitosis
GO:0051784	1.16683968809599e-07	123.059829059829	0.0470555670887828
4	40	negative regulation of nuclear division
GO:0051329	1.25211051332442e-07	26.460752688172 0.445851498166217	7
379	interphase of mitotic cell cycle
GO:0051325	1.44378048516681e-07	25.8889473684211	0.455262611583973
7	387	interphase
GO:0007088	2.87596821198805e-06	52.5641025641026	0.103522247595322
4	88	regulation of mitosis
GO:0051783	2.87596821198805e-06	52.5641025641026	0.103522247595322
4	88	regulation of nuclear division
GO:0010948	4.07345040441317e-06	47.9665551839465	0.112933361013079
4	96	negative regulation of cell cycle process
GO:0045841	8.64692470749185e-06	96.4419642857143	0.0411736212026849
3	35	negative regulation of mitotic metaphase/anaphase transition
GO:0006260	1.08952418588103e-05	22.4508871989861	0.315272299494845
5	268	DNA replication
GO:0030071	1.62097344171137e-05	77.1107142857143	0.0505847346204415
3	43	regulation of mitotic metaphase/anaphase transition
GO:0007091	1.86112825578916e-05	73.4285714285714	0.0529375129748806
3	45	mitotic metaphase/anaphase transition
GO:0007346	1.97254232303307e-05	19.7651006711409	0.35644592069753
5	303	regulation of mitotic cell cycle
GO:0010639	2.85819821851923e-05	28.7199597787833	0.184693100823472
4	157	negative regulation of organelle organization
GO:0051301	0.000132680453138237	13.0378448918717	0.531727908103245
5	452	cell division
GO:0051129	0.000341146021950791	14.7985347985348	0.350563974811432
4	298	negative regulation of cellular component organization
GO:0007093	0.000536490187489596	22.5283613445378	0.16351809563352
3	139	mitotic cell cycle checkpoint
GO:0051320	0.000582809827051627	21.8785714285714	0.168223652342398
3	143	S phase
GO:0031572	0.000593664129512498	66.2298850574713	0.0364680644938067
2	31	G2/M transition DNA damage checkpoint
GO:0000086	0.000594777118110322	21.7218844984802	0.169400041519618
3	144	G2/M transition of mitotic cell cycle
GO:0007094	0.000673164262056883	61.9483870967742	0.0388208428482458
2	33	mitotic cell cycle spindle assembly checkpoint
GO:0006271	0.000714742559025276	60.0083333333333	0.0399972320254654
2	34	DNA strand elongation involved in DNA replication
GO:0071173	0.000714742559025276	60.0083333333333	0.0399972320254654
2	34	spindle assembly checkpoint
GO:0071174	0.000801542709238449	56.4705882352941	0.0423500103799045
2	36	mitotic cell cycle spindle checkpoint
GO:0022616	0.000846759411781415	54.8533333333333	0.0435263995571241
2	37	DNA strand elongation

GO:0031576	0.000989645204184707	50.5122807017544	0.0470555670887828
2	40	G2/M transition checkpoint
GO:0031577	0.00109090652425899	47.98	0.0494083454432219	2	42
spindle checkpoint
GO:0000082	0.00124909903274545	16.6873536299766	0.21880838696284
3	186	G1/S transition of mitotic cell cycle
GO:0033043	0.00148433095390793	9.85530716423165	0.51878762715383
4	441	regulation of organelle organization
GO:0046602	0.00352526115515339	451	0.00352916753165871	1	3
regulation of mitotic centrosome separation
GO:0000226	0.00357483858317044	11.4135338345865	0.316448688672064
3	269	microtubule cytoskeleton organization
GO:0000216	0.0038051697756915	24.8606060606061	0.0929347450003446
2	79	M/G1 transition of mitotic cell cycle
GO:0007051	0.00390004462103452	24.5401709401709	0.0941111341775656
2	80	spindle organization
GO:0000079	0.00419126352725067	23.6263374485597	0.0976403017092243
2	83	regulation of cyclin-dependent protein kinase activity
GO:0033044	0.00419126352725067	23.6263374485597	0.0976403017092243
2	83	regulation of chromosome organization
GO:0000212	0.00469774665905387	300.645833333333	0.00470555670887828
1	4	meiotic spindle organization
GO:0007144	0.00469774665905387	300.645833333333	0.00470555670887828
1	4	female meiosis I
GO:0060564	0.00469774665905387	300.645833333333	0.00470555670887828
1	4	negative regulation of mitotic anaphase-promoting complex activity
GO:0006261	0.00523270528103615	21.0153846153846	0.10940419348142
2	93	DNA-dependent DNA replication
GO:0000089	0.00586893363952024	225.46875	0.00588194588609785	1
5	mitotic metaphase
GO:0007100	0.00586893363952024	225.46875	0.00588194588609785	1
5	mitotic centrosome separation
GO:0035405	0.00586893363952024	225.46875	0.00588194588609785	1
5	histone-threonine phosphorylation
GO:0000244	0.00703882344487339	180.3625	0.00705833506331742	1
6	assembly of spliceosomal tri-snRNP
GO:0006975	0.00703882344487339	180.3625	0.00705833506331742	1
6	DNA damage induced protein phosphorylation
GO:0048096	0.00703882344487339	180.3625	0.00705833506331742	1
6	chromatin-mediated maintenance of transcription
GO:0051299	0.00703882344487339	180.3625	0.00705833506331742	1
6	centrosome separation
GO:0071313	0.00703882344487339	180.3625	0.00705833506331742	1
6	cellular response to caffeine
GO:0007095	0.00820741742212772	150.291666666667	0.00823472424053699
1	7	mitotic cell cycle G2/M transition DNA damage checkpoint
GO:0051290	0.00820741742212772	150.291666666667	0.00823472424053699
1	7	protein heterotetramerization
GO:0071415	0.00820741742212772	150.291666666667	0.00823472424053699
1	7	cellular response to purine-containing compound
GO:0043414	0.00829916638042183	16.4574712643678	0.138813922911909
2	118	macromolecule methylation
GO:0040029	0.00884940838944783	15.9044444444444	0.143519479620787
2	122	regulation of gene expression, epigenetic
GO:0000077	0.00999787074467132	14.9020833333333	0.152930593038544
2	130	DNA damage checkpoint
Tissue: Whole_Blood=>Thyroid	Type:	cluster
SourceGene:	ENSG00000238943.1
TargetGeneSet:	ERRFI1	SPSB1	NMNAT1	DNAJC16	ARHGEF19-AS1	NECAP2	OTUD3
LDLRAP1	XKR8	MED18	PRDX3P2	FAM167B	HDAC1	ZMYM1	RNF220	FRRS1	CYB561D1
POLR3GL	BCL9	BOLA1	GOLPH3L	VPS72	U6	SHE	USP21	DUSP27	SELP
DARS2	RNASEL	SMG7	TRMT1L	PTGS2	SOX13	RP11	INTS7	ATF3	DISP1	SMC6
C2orf44	EHD3	CCDC75	PAPOLG	SPR	MRPL35	ZNF2	C2orf29	POLR1B	CHN1

CASP10 CREB1 IDH1 TMEM169 EIF4E2 MSL3P1 COPS8 RNPEPL1 RPL15 TRANK1
ZNF619 ZNF620 NME6 NPRL2 DUSP7 SCT ABHD10 ADPRH GATA2 MSL2
SLC25A36 ZBTB38 HPS3 P2RY13 SERPINI2 TNFSF10 NAALADL2
GCNT1P3 TACC3 SORCS2 COX18 CASP6 ANKRD50 CCDC127 LPCAT1 DAP C5orf51
SLC38A9 PTCD2 TNFAIP8 AFF4 HARS2 HMGXB3 DOK3 HIST1H3E ZKSCAN4
ZKSCAN3 SCAND3 ZBTB9 TAF8 CAPN11 MCM3 DBIP1 UBE2J1 C6orf203
ASF1A AKAP12 ELFN1 RNF216P1 VOPP1 ZNF107 TMEM60 CROT SLC12A9
PIK3CG TNPO3 FAM131B ZNF786 ZNF398 GIMAP8 GIMAP7 GIMAP4 SMARCD3 CTA
FUT10 PDP1 WDR67 KLHL38 ZNF696 ZNF623 TONSL RANBP6 KLHL9 DCAF10
C9orf85 C9orf40 CEP78 RMI1 FOXE1 RNF20 C9orf80 LHX6 ZBTB34 NAIF1 GLE1
ABL1 NUDT5 C10orf136 C10orf128 ADO MSS51 AP3M1 ARHGAP19
SUFU SMC3 C10orf88 TTC9C ZBTB3 MEN1 FOSL1 C11orf82
SLC36A4 RDX RNF214 ABCG4 ZNF202 HYLS1 FLI1 RAD52 CD9 LRMP
BCDIN3D FAM186A KRT7 RP3 SMUG1 SUOX TIMELESS ZBTB39 ATXN7L3B
TMPO ANAPC7 COQ5 RNF34 FAM48A KBTBD6 KBTBD7 LPAR6 ARL11 TTC5
RNASE1 ACIN1 C14orf119 PROX2 VASH1 MIR656 ZFP106 ZSCAN29 CTD
CCNB2 DIS3L SMAD6 IQCH FEM1B ANP32A PEX11A CHD2 NR2F2 ASB7
TFAP4 C16orf88 PLK1 TAOK2 HIRIP3 PLCG2 SPNS2 SLC16A13
WRAP53 ARHGEF15 PIGL LLGL1 BLMH CRLF3 RNF135 PEX12 PIP4K2B
FAM134C RUNDC1 GJC1 DLX3 PPP1R9B HEATR6 APPBP2 CD79B QRICH2 USP36 CBX2
SIRT7 RNU5F-3P ZNF555 PIAS4 ZNF627 ZNF625 ZNF799 ZNF443 ZNF490 LYL1
IL27RA ANO8 RAB3A SUGP1 KCTD15 ZNF792 THAP8 CAPNS1 ZNF420 ZNF573
SAMD4B EID2 ZNF780A MAP3K10 LTBP4 MIA ZNF526 ZNF221 ZNF284 ZNF225
PVRL2 GEMIN7 BLOC1S3 FBXO46 PPP1R15A C19orf63 ZNF616 ZNF816
ZNF677 ZNF765 TMC4 ZNF264 ZNF547 ZNF549 SLC52A3 FAM110A PANK2 ANKRD5 NANP
C20orf160 RP1 DNMT3B PXMP4 RBL1 SDC4 ZSWIM3 ZSWIM1 ZFP64
AURKA CSTF1 TAF4 C20orf151 SOX18 RGS19 CYYR1 BACE2 C2CD2
C22orf29 CRKL ZNF70 GTPBP1 EFCAB6 TLR8 TCEANC SCML1 ZFX
GPR34 CXorf24 RPL23AP83 FAM123B VSIG4 MAGEC2

GOBPID	Pvalue	OddsRatio	ExpCount	Count	Size	Term
GO:0033261	0.00158554766356836	9.01398601398601	0.510691301640025	4	30	regulation of S phase
GO:0045736	0.00329117860132599	11.679012345679	0.306414780984015	3	18	negative regulation of cyclin-dependent protein kinase activity
GO:0010564	0.00494738674276267	2.24685089346743	7.04753996263234	15	414	regulation of cell cycle process
GO:0021700	0.00567986020378686	3.14957983193277	2.68964085530413	8	158	developmental maturation
GO:0007090	0.00590783962912106	9.21767381416504	0.374506954536018	3	22	regulation of S phase of mitotic cell cycle
GO:0045786	0.00800200296448542	2.12085308056872	7.43906996055636	15	437	negative regulation of cell cycle
GO:0033233	0.00960200448278314	16.6252927400468	0.153207390492007	2	9	regulation of protein sumoylation

Tissue: Whole_Blood=>Thyroid Type: cluster
SourceGene: ING4(ENSG00000111653.15)
TargetGeneSet: UBE2J2 DNAJC16 TXLNA SF3A3 TOE1 USP1 RP11 KCND3
SCNM1 CELF3 CCT3 IQGAP3 CDC73 ASPM URB2 HEATR1 EIF2B4 MSH6
PCBP1 NCAPH POU3F3 SSB NUP35 PTPRN CLN5 CHCHD4 IFRD2 ACTR8
ZBTB11 GRPEL1 WDR36 RBM22 FBXW11 HRH2 BYSL HDAC2 NKAIN2 FBXO5
SNORA29 EIF3B RNF216P1 THAP5 CNOT4 U1 ABCF2 NEIL2 PPP2R2A
BAALC PLAA DCAF10 FRMD3 METTL11A SURF6 NGB BMS1 HNRNPF
NOLC1 ADRA2A TSSC4 C11orf46 STIP1 NCAM1 MPP2 NOP2 DDX23
ZC3H10 INHBC TBK1 C12orf29 SART3 NAA16 TPTE2P3 FAM70B RBM23
SNORD116-15 BUB1B GABPB1 IDH3A FAM103A1 CTD E2F4 CIRH1A
DHODH USP10 CYBA TSR1 COIL FTSJ3 MRPL38 DOT1L ZNF77 KHSRP
GATAD2A ZFP82 GRWD1 PRMT1 PEG3 NOP56 MCM3AP-AS1 PARP4P3 UBE2L3
MIR221 MAGEH1 EDA FAM122C MAGEA8

GOBPID	Pvalue	OddsRatio	ExpCount	Count	Size	Term
GO:0022613	1.90176179361749e-12	15.0948827292111	1.27672825410006	15	225	ribonucleoprotein complex biogenesis
GO:0071843	4.54980692536357e-12	14.137460021322	1.35616912324407	15	239	cellular component biogenesis at cellular level

GO:0042254 7.21942106151792e-10 15.9768320065319 0.845477821604041
11 149 ribosome biogenesis
GO:0006397 4.75183889232843e-08 7.47807486631016 2.26406477060411
14 399 mRNA processing
GO:0008380 1.3928037721635e-05 6.23713170039049 1.83281433810809
10 323 RNA splicing
GO:0022618 1.6118246502793e-05 12.6670609107037 0.539063040620026
6 95 ribonucleoprotein complex assembly
GO:0071826 2.16151964470521e-05 11.9890817469205 0.567434779600028
6 100 ribonucleoprotein complex subunit organization
GO:0000375 2.38435884093652e-05 7.62027699341132 1.18593868936406
8 209 RNA splicing, via transesterification reactions
GO:0006364 2.70231486534987e-05 11.4965091299678 0.590132170784029
6 104 rRNA processing
GO:0016072 3.71277901418746e-05 10.8286943319838 0.62417825756003
6 110 rRNA metabolic process
GO:0032259 9.85669348900182e-05 8.99621052631579 0.743339561276036
6 131 methylation
GO:0034470 0.000138461507791272 6.85540587219344 1.13486955920006
7 200 ncRNA processing
GO:0000377 0.000156517181096764 6.71431472081218 1.15756695038406
7 204 RNA splicing, via transesterification reactions with bulged adenosine as nucleophile
GO:0000398 0.000156517181096764 6.71431472081218 1.15756695038406
7 204 nuclear mRNA splicing, via spliceosome
GO:0060789 0.000189440581023968 179.5875 0.0226973911840011 2
4 hair follicle placode formation
GO:0043414 0.000551193127756544 8.1921618204804 0.669573039928033 5
118 macromolecule methylation
GO:0033120 0.00065573595463257 71.82 0.0397204345720019 2 7
positive regulation of RNA splicing
GO:0045292 0.00065573595463257 71.82 0.0397204345720019 2 7
nuclear mRNA cis splicing, via spliceosome
GO:0034660 0.00116037215425537 4.73079136690647 1.61718912186008
7 285 ncRNA metabolic process
GO:0050685 0.0016922542545246 39.8888888888889 0.062417825756003
2 11 positive regulation of mRNA processing
GO:0060788 0.00318343603243353 27.6076923076923 0.0851152169400042
2 15 ectodermal placode formation
GO:0071696 0.00318343603243353 27.6076923076923 0.0851152169400042
2 15 ectodermal placode development
GO:0071697 0.00318343603243353 27.6076923076923 0.0851152169400042
2 15 ectodermal placode morphogenesis
GO:0009749 0.00329993944260055 7.03402366863905 0.61282956196803
4 108 response to glucose stimulus
GO:0042255 0.00362485571722543 25.6339285714286 0.0907895647360044
2 16 ribosome assembly
GO:0017038 0.00385904213214055 4.31793145654835 1.49802781814407
6 264 protein import
GO:0009746 0.00388106150564039 6.70900964478946 0.641201300948031
4 113 response to hexose stimulus
GO:0034284 0.0041317453096702 6.58720258720259 0.652549996540032
4 115 response to monosaccharide stimulus
GO:0043484 0.00413297682835745 10.0668073136428 0.323437824372016
3 57 regulation of RNA splicing
GO:0045862 0.00434075830036303 9.88308400460299 0.329112172168016
3 58 positive regulation of proteolysis
GO:0048285 0.0049975243521611 3.6011753902663 2.0995086845201 7 370
organelle fission
GO:0031365 0.00510878432960978 21.1058823529412 0.107812608124005
2 19 N-terminal protein amino acid modification
GO:0031648 0.00510878432960978 21.1058823529412 0.107812608124005
2 19 protein destabilization

GO:0009743	0.00671697810417207	5.70552884615385	0.749013909072037
4	132	response to carbohydrate stimulus
GO:0000083	0.00682576729509103	17.93625	0.124835651512006	2
22	regulation of transcription involved in G1/S phase of mitotic cell cycle
GO:0007090	0.00682576729509103	17.93625	0.124835651512006	2
22	regulation of S phase of mitotic cell cycle
GO:0030163	0.00694191987368296	3.08163605083523	2.80880215902014
8	495	protein catabolic process
GO:0033157	0.00823705393207931	5.36689291101056	0.794408691440039
4	140	regulation of intracellular protein transport
GO:0030162	0.00864950084488337	5.28836863619472	0.805757387032039
4	142	regulation of proteolysis
GO:0006479	0.00952863464021312	7.33578515224085	0.436924780292021
3	77	protein methylation
GO:0008213	0.00952863464021312	7.33578515224085	0.436924780292021
3	77	protein alkylation
Tissue: Thyroid=>Whole_Blood	Type:	asymmetric
SourceGene:	KRTAP3-2(ENSG00000212900.1)
TargetGeneSet:	CELA2B	CELA3A	AMY2A	SMCP	CPB1	SPINK1	CLPS	RP11	CPA2
CPA1	PRSS1	PNLIP	PNLIPRP2		KRT6A	KRT4	GP2	CTRB2	CTRB1
KRT13
GOBPID	Pvalue	OddsRatio	ExpCount	Count	Size	Term
GO:0007586	5.69496585169008e-13	103.193519351935	0.14331188153069
8	109	digestion
GO:0044241	8.68065653198509e-07	225.3125	0.0197218185592693	3
15	lipid digestion
GO:0022617	1.33863255514118e-05	81.8125	0.0473323645422462	3	36
extracellular matrix disassembly
GO:0030198	0.00115359192296497	16.939082278481	0.211680852536157	3
161	extracellular matrix organization
GO:0043062	0.00117438184385728	16.8313679245283	0.212995640440108
3	162	extracellular structure organization
GO:0001523	0.00117890899156601	45.7710651828299	0.0512767282541001
2	39	retinoid metabolic process
GO:0016101	0.00130255347491052	43.4177978883861	0.0539063040620026
2	41	diterpenoid metabolic process
GO:0006721	0.00193222176422744	35.2549019607843	0.0657393951975642
2	50	terpenoid metabolic process
GO:0016042	0.0036858273964719	11.1346757322176	0.318178672756211
3	242	lipid catabolic process
GO:0006720	0.00396282855922372	24.1378151260504	0.0946647290844924
2	72	isoprenoid metabolic process
GO:0009791	0.00451877459080465	22.5207843137255	0.101238668604249
2	77	post-embryonic development
GO:0008202	0.00521715255170248	9.79773985239852	0.360251885682652
3	274	steroid metabolic process
GO:0071845	0.00576367505250986	9.44239323843416	0.373399764722165
3	284	cellular component disassembly at cellular level
GO:0022411	0.00599161296939949	9.30723684210526	0.37865891633797
3	288	cellular component disassembly
GO:0007341	0.00655758079782431	200.388888888889	0.00657393951975642
1	5	penetration of zona pellucida
GO:0044245	0.00655758079782431	200.388888888889	0.00657393951975642
1	5	polysaccharide digestion
GO:0000244	0.00786419895958823	160.3	0.0078887274237077	1	6
assembly of spliceosomal tri-snRNP
GO:0002385	0.00786419895958823	160.3	0.0078887274237077	1	6
mucosal immune response
GO:0019374	0.00786419895958823	160.3	0.0078887274237077	1	6
galactolipid metabolic process
GO:0045056	0.00786419895958823	160.3	0.0078887274237077	1	6
transcytosis
Tissue: Whole_Blood=>Thyroid	Type:	asymmetric

SourceGene:	NCAN(ENSG00000130287.5)
TargetGeneSet:	NOC2L	RBP7	RP1	NUDC	NCDN	LRRC41	HIPK1	C1orf61
TROVE2	CDC73	RP11	ACBD3	URB2	SMPD4	GORASP2	MOBP	C3orf39	TMF1
GAP43	TMEM39A	ZIC1	LETM1	TBC1D1	ANKRD17	SEC24D	ZBTB8OSP1		WDR36
SAR1B	SEC24A	CAMK2A	CPLX2	FAF2	SNCB	SSR1	PPARD	PGM3	SF3B5
EIF3B	C7orf26	ABCF2	SLC35G5	NEIL2	PRKDC	STMN2	UBQLN1	SURF4	SEC16A
SEC24C	NOLC1	GBF1	SEC23IP	TSSC4	COPB1	SLC1A2	GANAB	STIP1	SYVN1
SLC22A20		ARCN1	RNF26	LEPREL2	CBX5	KIF5A	CAND1	SART3	GCN1L1
ATP6V0A2		STON2	RNF40	DNAJA2	EDC4	PRDM7	DPH1	ARHGDIA	FOXK2
ST8SIA3	AP3D1	ELAVL1	P2RY11	RAVER1	ELAVL3	USE1	GATAD2A	PSMC4	SMG9
GRWD1	PRMT1	CNOT3	FIZ1	PEG3	RPS18P1	SNAP25	SEC23B	RIMS4	RP4	PRR5
APEX2	MAGEH1	RP13	HCFC1
GOBPID	Pvalue	OddsRatio	ExpCount	Count	Size	Term
GO:0048199	2.75977668969244e-12	75.4947368421053	0.164417687357276
8	27	vesicle targeting, to, from or within Golgi
GO:0006901	2.81246575646194e-11	53.0962962962963	0.21313403916684
8	35	vesicle coating
GO:0006900	4.56823424919053e-11	49.4275862068965	0.225313127119231
8	37	membrane budding
GO:0006903	4.56823424919053e-11	49.4275862068965	0.225313127119231
8	37	vesicle targeting
GO:0051650	4.22897738743914e-10	35.8075	0.29229811085738	8	48
establishment of vesicle localization
GO:0051648	5.20139281349438e-09	25.0982456140351	0.395820358452702
8	65	vesicle localization
GO:0051656	1.00269660814096e-08	17.6718492019813	0.615043941595737
9	101	establishment of organelle localization
GO:0048207	2.13662666113678e-08	86.4638554216867	0.0913431596429313
5	15	vesicle targeting, rough ER to cis-Golgi
GO:0048208	2.13662666113678e-08	86.4638554216867	0.0913431596429313
5	15	COPII vesicle coating
GO:0090114	2.13662666113678e-08	86.4638554216867	0.0913431596429313
5	15	COPII-coated vesicle budding
GO:0006888	3.90193650725047e-08	25.7728909465021	0.334924918690748
7	55	ER to Golgi vesicle-mediated transport
GO:0016050	7.70335352135787e-08	17.2048192771084	0.554148501833783
8	91	vesicle organization
GO:0048193	1.04763838398002e-07	11.031857031857	1.0656701958342	10	175
Golgi vesicle transport
GO:0051640	3.12625900674405e-07	11.4909776461083	0.913431596429313
9	150	organelle localization
GO:0018279	9.60119573805604e-07	15.4291666666667	0.529790325929001
7	87	protein N-linked glycosylation via asparagine
GO:0018196	1.03798281649526e-06	15.2376162170401	0.535879869905197
7	88	peptidyl-asparagine modification
GO:0006487	1.40452995019808e-06	14.5164851125635	0.560238045809979
7	92	protein N-linked glycosylation
GO:0035964	5.9714342159921e-05	50.6576470588235	0.0791640716905404
3	13	COPI-coated vesicle budding
GO:0048194	5.9714342159921e-05	50.6576470588235	0.0791640716905404
3	13	Golgi vesicle budding
GO:0048200	5.9714342159921e-05	50.6576470588235	0.0791640716905404
3	13	Golgi transport vesicle coating
GO:0048205	5.9714342159921e-05	50.6576470588235	0.0791640716905404
3	13	COPI coating of Golgi vesicle
GO:0016044	9.99787248871515e-05	4.86206656938364	2.30793716697806
10	379	cellular membrane organization
GO:0061024	0.000106687222457645	4.82182244279019	2.32620579890665
10	382	membrane organization
GO:0043687	0.00011634485631479	7.04718319852419	1.10220745969137
7	181	post-translational protein modification
GO:0019882	0.000326942268488207	7.12512529234881	0.925610684381704
6	152	antigen processing and presentation

GO:0002474	0.000340633540317546	9.14445014098949	0.602864853643347
5	99	antigen processing and presentation of peptide antigen via MHC class I
GO:0006890	0.0004026008062532	24.1042016806723	0.14614905542869
3	24	retrograde vesicle-mediated transport, Golgi to ER
GO:0048002	0.0005086228600119	8.34015674347877	0.657670749429105
5	108	antigen processing and presentation of peptide antigen
GO:0006486	0.00086191015564211 3	4.97989417989418	1.53456508200125
7	252	protein glycosylation
GO:0043413	0.000861910155642113	4.97989417989418	1.53456508200125
7	252	macromolecule glycosylation
GO:0070085	0.000923652236374787	4.91860812425329	1.55283371392983
7	255	glycosylation
GO:0071482	0.000952519709758408	17.4450304259635	0.194865407238253
3	32	cellular response to light stimulus
GO:0033523	0.00128371324468993	47.6943521594684	0.0548058957857588
2	9	histone H2B ubiquitination
GO:0007603	0.0019457472888171	37.0904392764858	0.0669849837381496
2	11	phototransduction, visible light
GO:0007030	0.00196791157521203	13.3049535603715	0.249671303024012
3	41	Golgi organization
GO:0009101	0.0024766634915905 2	4.10698198198198	1.84513182478721
7	303	glycoprotein biosynthetic process
GO:0010390	0.0052649413928623 6	20.8531976744186	0.109611791571518
2	18	histone monoubiquitination
GO:0071478	0.0052882676485701 3	9.18160427807487	0.353193550619334
3	58	cellular response to radiation
GO:0009100	0.0055412324530108 7	3.52185615848407	2.13742993564459
7	351	glycoprotein metabolic process
GO:0031648	0.0058611654109295 8	19.625170998632 0.115701335547713	2
19	protein destabilization
GO:0009584	0.0064867627159179 2	18.5335917312661	0.121790879523908
2	20	detection of visible light
GO:1901137	0.0075408887867614 3	3.3142567224759 2.2653103591447 7	372
carbohydrate derivative biosynthetic process
Tissue: Thyroid=>Whole_Blood	Type:	asymmetric
SourceGene:	OLIG1(ENSG00000184221.8)
TargetGeneSet:	BCAN	ATP1A4	CNTN2	KIF1A	MOBP	IMPG2	SOX2-OT SORCS2
CTNND2	TMEM130 PTPRZ1	OR2A9P	STMN2	NTRK2	OLFM1	GRIN1	SLCO1A2 FAIM2
PPM1H	CLIC1P1 FAM123A CYP46A1 C16orf13	MT3	AQP4	TUBB4A	MAG
LRRC4B	SHANK1	RP11	BCAS1	GPM6B	PLP1
GOBPID	Pvalue	OddsRatio	ExpCount	Count	Size	Term
GO:0007612	2.42177573625753e-07	46.8101761252446	0.140336308905958
5	78	learning
GO:0007611	4.5936283323766e-06	25.015756302521 0.253684866099232	5
141	learning or memory
GO:0050890	7.0705225750299e-06	22.8124001278364	0.277074250916892
5	154	cognition
GO:0010975	1.86149100770493e-05	18.5297944314338	0.338246488132309
5	188	regulation of neuron projection development
GO:0050770	2.44408904771926e-05	28.3260869565217	0.172721610961179
4	96	regulation of axonogenesis
GO:0031344	4.89526522222706e-05	15.026455026455 0.413812192927825	5
230	regulation of cell projection organization
GO:0051960	5.56979163054521e-05	10.9111398963731	0.705279911424815
6	392	regulation of nervous system development
GO:0045664	0.000153540336941953	11.6873346560847	0.527160750121099
5	293	regulation of neuron differentiation
GO:0021782	0.000157213624916511	33.4681677018634	0.106151823403225
3	59	glial cell development
GO:0006707	0.000169504482655436	133.481481481481	0.0197910179226351
2	11	cholesterol catabolic process
GO:0016127	0.000169504482655436	133.481481481481	0.0197910179226351

2	11	sterol catabolic process
GO:0022010	0.000169504482655436	133.481481481481	0.0197910179226351
2	11	central nervous system myelination
GO:0032291	0.000169504482655436	133.481481481481	0.0197910179226351
2	11	axon ensheathment in central nervous system
GO:2000463	0.000169504482655436	133.481481481481	0.0197910179226351
2	11	positive regulation of excitatory postsynaptic membrane potential
GO:0010769	0.000215767505636689	15.810421286031 0.302262819182064	4
168	regulation of cell morphogenesis involved in differentiation
GO:0031345	0.000229468490445889	29.2683423913043	0.120545290983323
3	67	negative regulation of cell projection organization
GO:0050804	0.000241400759633476	15.3372781065089	0.311258736419625
4	173	regulation of synaptic transmission
GO:0051969	0.000324821799205402	14.1500248385494	0.336447304684797
4	187	regulation of transmission of nerve impulse
GO:0045838	0.000367786810607516	85.7797619047619	0.0287869351601965
2	16	positive regulation of membrane potential
GO:0050767	0.000378772103909581	9.54687288020621	0.640509307314373
5	356	regulation of neurogenesis
GO:0048167	0.000401922738021126	23.9916387959866	0.145733859248495
3	81	regulation of synaptic plasticity
GO:0031644	0.000411323128239673	13.2680652680653	0.358037506054944
4	199	regulation of neurological system process
GO:0042391	0.000522441754386378	12.4274475524476	0.381426890872604
4	212	regulation of membrane potential
GO:0006706	0.000769435401192477	57.1587301587302	0.0413812192927825
2	23	steroid catabolic process
GO:0021953	0.000882121917511441	18.1367665681722	0.190713445436302
3	106	central nervous system neuron differentiation
GO:0060284	0.00102179355141063	7.6032833224614 0.797038267247941	5
443	regulation of cell development
GO:0014003	0.0011432530838012	46.150641025641 0.0503771365303439	2
28	oligodendrocyte development
GO:0007610	0.00128095959583095	7.21206486933168	0.838419486540724
5	466	behavior
GO:0010001	0.00138795476634237	15.4193316564858	0.223098747491523
3	124	glial cell differentiation
GO:0022604	0.00143497775342852	9.39017916390179	0.500172998408415
4	278	regulation of cell morphogenesis
GO:0043523	0.00145330451763101	15.1664899257688	0.226697114386548
3	126	regulation of neuron apoptotic process
GO:1901214	0.00145330451763101	15.1664899257688	0.226697114386548
3	126	regulation of neuron death
GO:0007409	0.00165913525184291	6.78547083455059	0.888796623071068
5	494	axonogenesis
GO:0060079	0.00178569731497192	36.3434343434343	0.0629714206629299
2	35	regulation of excitatory postsynaptic membrane potential
GO:0051129	0.00185168168737784	8.73902288188003	0.53615666735866
4	298	negative regulation of cellular component organization
GO:0051402	0.00188620277565234	13.8067632850242	0.248287315756695
3	138	neuron apoptotic process
GO:0050771	0.00188865069289165	35.2720588235294	0.0647706041104422
2	36	negative regulation of axonogenesis
GO:0044087	0.00196773891443697	8.58984493767102	0.545152584596222
4	303	regulation of cellular component biogenesis
GO:0070997	0.00204652761729825	13.4056928370347	0.255484049546744
3	142	neuron death
GO:0042063	0.00217219112194648	13.1197183098592	0.260881599889281
3	145	gliogenesis
GO:0048708	0.00221406413008979	32.4054054054054	0.070168154452979
2	39	astrocyte differentiation
GO:0021537	0.0022585828669325l	12.9356884057971	0.264479966784306
3	147	telencephalon development

GO:0048168	0.00232802143443588	31.5504385964912	0.0719673379004913
2	40	regulation of neuronal synaptic plasticity
GO:0060078	0.00232802143443588	31.5504385964912	0.0719673379004913
2	40	regulation of postsynaptic membrane potential
GO:0021954	0.0026862197063143б	29.2357723577236	0.0773648882430282
2	43	central nervous system neuron development
GO:0008206	0.0028110299456б741	28.5376984126984	0.0791640716905404
2	44	bile acid metabolic process
GO:0008306	0.00293852999747532	27.8720930232558	0.0809632551380527
2	45	associative learning
GO:0007215	0.00320156226858409	26.6296296296296	0.0845616220330773
2	47	glutamate receptor signaling pathway
GO:0010976	0.0037594958556273	24.4489795918367	0.0917583558231264
2	51	positive regulation of neuron projection development
GO:0048709	0.00405424962355107	23.4869281045752	0.095356722718151
2	53	oligodendrocyte differentiation
GO:0007017	0.00501252730018951	6.56041131105398	0.707079094872327
4	393	microtubule-based process
GO:0031117	0.00538821719050797	288.46	0.00539755034253685	1	3
positive regulation of microtubule depolymerization
GO:0031133	0.00538821719050797	288.46	0.00539755034253685	1	3
regulation of axon diameter
GO:0036003	0.00538821719050797	288.46	0.00539755034253685	1	3
positive regulation of transcription from RNA polymerase II promoter in response to stress
GO:0045163	0.00538821719050797	288.46	0.00539755034253685	1	3
clustering of voltage-gated potassium channels
GO:0050893	0.00538821719050797	288.46	0.00539755034253685	1	3
sensory processing
GO:0051581	0.00538821719050797	288.46	0.00539755034253685	1	3
negative regulation of neurotransmitter uptake
GO:0051611	0.00538821719050797	288.46	0.00539755034253685	1	3
regulation of serotonin uptake
GO:0051612	0.00538821719050797	288.46	0.00539755034253685	1	3
negative regulation of serotonin uptake
GO:0071205	0.00538821719050797	288.46	0.00539755034253685	1	3
protein localization to juxtaparanode region of axon
GO:2000009	0.00538821719050797	288.46	0.00539755034253685	1	3
negative regulation of protein localization to cell surface
GO:0044057	0.00617586384696138	6.16861104996698	0.750259497612622
4	417	regulation of system process
GO:0042552	0.00659225990226509	18.1300505050505	0.122344474430835
2	68	myelination
GO:0032536	0.00717807781848756	192.293333333333	0.00719673379004913
1	4	regulation of cell projection size
GO:0042759	0.00717807781848756	192.293333333333	0.00719673379004913
1	4	long-chain fatty acid biosynthetic process
GO:0051610	0.00717807781848756	192.293333333333	0.00719673379004913
1	4	serotonin uptake
GO:0072592	0.00717807781848756	192.293333333333	0.00719673379004913
1	4	oxygen metabolic process
GO:0007272	0.00736424552645795	17.0892857142857	0.129541208220884
2	72	ensheathment of neurons
GO:0008366	0.00736424552645795	17.0892857142857	0.129541208220884
2	72	axon ensheathment
GO:0051899	0.00756335669081938	16.8474178403756	0.131340391668397
2	73	membrane depolarization
GO:0051494	0.00776489631219968	16.6122685185185	0.133139575115909
2	74	negative regulation of cytoskeleton organization
GO:0007420	0.00793004676512219	5.72522522522523	0.806034184485503
4	448	brain development
GO:0001764	0.00838399916751115	15.9444444444444	0.138537125458446
2	77	neuron migration

GO:0097285 0.00847345213804796 7.94476581451763 0.424607293612899
3 236 cell-type specific apoptotic process
GO:0001973 0.00896484115874663 144.21 0.00899591723756141 1 5
adenosine receptor signaling pathway
GO:0021826 0.00896484115874663 144.21 0.00899591723756141 1 5
substrate-independent telencephalic tangential migration
GO:0021830 0.00896484115874663 144.21 0.00899591723756141 1 5
interneuron migration from the subpallium to the cortex
GO:0021843 0.00896484115874663 144.21 0.00899591723756141 1 5
substrate-independent telencephalic tangential interneuron migration
GO:0021853 0.00896484115874663 144.21 0.00899591723756141 1 5
cerebral cortex GABAergic interneuron migration
GO:0021894 0.00896484115874663 144.21 0.00899591723756141 1 5
cerebral cortex GABAergic interneuron development
GO:0031115 0.00896484115874663 144.21 0.00899591723756141 1 5
negative regulation of microtubule polymerization
GO:0033210 0.00896484115874663 144.21 0.00899591723756141 1 5
leptin-mediated signaling pathway
GO:0035418 0.00896484115874663 144.21 0.00899591723756141 1 5
protein localization to synapse
GO:0071625 0.00896484115874663 144.21 0.00899591723756141 1 5
vocalization behavior
GO:0043524 0.00924294353506946 15.1329113924051 0.145733859248495
2 81 negative regulation of neuron apoptotic process
GO:0050768 0.00924294353506946 15.1329113924051 0.145733859248495
2 81 negative regulation of neurogenesis
GO:1901215 0.00924294353506946 15.1329113924051 0.145733859248495
2 81 negative regulation of neuron death
GO:0030900 0.00959389310905019 7.58071988595866 0.444398311535534
3 247 forebrain development
GO:0051493 0.0096998565238275 7.54924578527063 0.446197494983046
3 248 regulation of cytoskeleton organization
GO:0019228 0.00991195731059808 14.5762195121951 0.151131409591032
2 84 regulation of action potential in neuron
Tissue: Whole_Blood=>Thyroid Type: cluster
SourceGene: RP11-476C8.3(ENSG00000250163.1)
TargetGeneSet: FNDC5 RP11 GPSM2 HMGB1P11 SIX2 IGKJ5 IGKC
IGKJ2 IGKV4-1 IGKV5-2 IGKV3-15 IGKV3-20 IGKV2-24 IGKV2-29
IGKV2D-24 MFSD6 SERPINE2 CCR4 BCHE TXK IGJ TRIM2
ANXA10 NNT PPP2R2A PNOC POLR2K PIP4K2A RPS24 ROBO3 APAF1 SPIC
IGHA2 IGHA1 IGHV3-13 IGHV1-46 IGHV1-69 IGHV3-73
LINC00324 PIEZO2 PNMAL2 DPM1 IGLV7-43 IGLV2-11 IGLL5
IGLC1 IGLC3 RN5-8S6
GOBPID Pvalue OddsRatio ExpCount Count Size Term
GO:0006958 7.01309979723971e-10 81.6875 0.103799045048786 6 50
complement activation, classical pathway
GO:0006956 2.94353652635558e-09 63 0.13078679676147 6 63
complement activation
GO:0002455 3.56814685279044e-09 60.8559322033898 0.134938758563421
6 65 humoral immune response mediated by circulating immunoglobulin
GO:0072376 1.47636597233999e-08 47.1875 0.170230433880008 6 82
protein activation cascade
GO:0016064 9.15904609930835e-08 34.0857142857143 0.230433880008304
6 111 immunoglobulin mediated immune response
GO:0019724 1.07417590743408e-07 33.1319444444444 0.236661822711231
6 114 B cell mediated immunity
GO:0002250 1.27080174854711e-07 22.6747097655361 0.411044218393191
7 198 adaptive immune response
GO:0006959 1.60955212150291e-07 30.8297413793103 0.253269669919037
6 122 humoral immune response
GO:0002449 1.02719060191915e-06 22.1428571428571 0.346688810462944
6 167 lymphocyte mediated immunity
GO:0002460 1.64360542847173e-06 20.3514285714286 0.375752543076604

6	181	adaptive immune response based on somatic recombination of immune receptors built from immunoglobulin superfamily domains
GO:0002253	2.0475684111775e-06	14.7264740917213	0.620718289391738
7	299	activation of immune response
GO:0002443	3.99911101796174e-06	17.3365853658537	0.438031970105875
6	211	leukocyte mediated immunity
GO:0050778	7.49754686067186e-06	11.9897698209719	0.755657047955159
7	364	positive regulation of immune response
GO:0002252	0.000120432446390825	9.2375	0.801328627776625	6	386
immune effector process
GO:0090331	0.00621545179129268	248.603448275862	0.00622794270292713
1	3	negative regulation of platelet aggregation
GO:0010757	0.00827895791298872	165.724137931034	0.00830392360390284
1	4	negative regulation of plasminogen activation
GO:0035268	0.00827895791298872	165.724137931034	0.00830392360390284
1	4	protein mannosylation
GO:0035269	0.00827895791298872	165.724137931034	0.00830392360390284
1	4	protein O-linked mannosylation
GO:0060332	0.00827895791298872	165.724137931034	0.00830392360390284
1	4	positive regulation of response to interferon-gamma
GO:0060335	0.00827895791298872	165.724137931034	0.00830392360390284
1	4	positive regulation of interferon-gamma-mediated signaling pathway
Tissue: Whole_Blood=>Thyroid	Type:	cluster
SourceGene:	RP11-890B15.3(ENSG00000255455.1)
TargetGeneSet:	CEP104	RP4	DFFA	MED18	MFI2	AKIRIN1	MOB3C	ZFYVE9	USP1
ITGB3BP	U6	ZNF644	AMIGO1	HIPK1	DENND4B	UBQLN4	SMG5	RRNAD1	B4GALT3	POGK
GS1	CDC73	RP11	SLC5A6	MSH6	AMMECR1L	NDUFA10	CMC1	PRKAR2A	NEK4
SLC25A36	MYNN	SENP2	CEP19	CCDC149	MTRR	ZFR	NIPBL	SEC24A
FBXW11	C5orf25	SRPK1	BRPF3	TAF8	RPL7L1	RARS2	FBXO5	C7orf26	PURB
TRIM56	CUL1	ZNF398	ABCF2	POLR3D	CHMP7	KAT6A	CTD	COX6C	DNAJA1	RGP1
BICD2	NUP188	METTL11A	TOR1B	FBXO18	TAF3	PHRF1	NUP98	BDNF-AS1
ETFA	MARK2	STIP1	TEX12	PAFAH1B2	SETP16	CBL	HYLS1	TMEM198B
RNF41	GCN1L1	RP13	MPHOSPH8	RANP8	PDS5B	NIPA2	HERC2	PLEKHO2
GOLGA6L5	ZNF592	SEC14L5	TBC1D10B	SRCAP	SETD1A	DNAJA2	NFATC3	TAT
GAN	VPS53	ALKBH5	LEPREL4	STAT5B	RUNDC1	NMT1	PPM1D	METTL2A	FOXK2
ZBTB7A	FEM1A	ELAVL1	ZNF441	ZNF791	BRD4	U2AF2	ZNF264	ZNF587	ZSCAN22
TRIM28	RP5-1187M17.10	PHF20	TP53RK	GNAS-AS1	C22orf29	KRT18P5	CRKL
PPARA	DDX3X	SMC1A	MED12	RGAG4	MID2	CUL4B	XIAP
GOBPID	Pvalue	OddsRatio	ExpCount	Count	Size	Term
GO:0007064	4.94507727089692e-05	56.03125	0.0753581067054183	3
11	mitotic sister chromatid cohesion
GO:0007059	0.000397550402121733	6.84545016851228	0.959103176250778
6	140	chromosome segregation
GO:0016570	0.000422029338857773	4.8991008991009	1.78804235001038	8
261	histone modification
GO:0043543	0.000461953799762098	6.64516129032258	0.986506124143658
6	144	protein acylation
GO:0016569	0.000490697853596793	4.7835716407145	1.8291467718497	8	267
covalent chromatin modification
GO:0016573	0.000870290617365219	7.35850030985334	0.739879593107743
5	108	histone acetylation
GO:0018393	0.000945346314671262	7.21732522796353	0.753581067054183
5	110	internal peptidyl-lysine acetylation
GO:0018394	0.00106682760768211	7.01536643026005	0.774133277973843
5	113	peptidyl-lysine acetylation
GO:0006475	0.00115401297137377	6.88684719535783	0.787834751920282
5	115	internal protein amino acid acetylation
GO:0007062	0.00133918451408605	15.4342672413793	0.219223583143035
3	32	sister chromatid cohesion
GO:0006473	0.0018552255678164	6.15334717176959	0.87689433257214
5	128	protein acetylation
GO:0032968	0.00201721214730941	36.9690721649485	0.0685073697321985
2	10	positive regulation of transcription elongation from RNA polymerase

II promoter
GO:0016568 0.00230075228150553 3.40048780487805 2.87045879177912
9 419 chromatin modification
GO:0001833 0.00245449245277618 32.8591065292096 0.0753581067054183
2 11 inner cell mass cell proliferation
GO:0050684 0.0027527937845859 11.7713815789474 0.280880215902014
3 41 regulation of mRNA processing
GO:0018205 0.00282738089969911 5.56007509386733 0.965953913223998
5 141 peptidyl-lysine modification
GO:0034243 0.00293226963861002 29.5711340206186 0.0822088436786382
2 12 regulation of transcription elongation from RNA polymerase II promoter
GO:0043966 0.00336976613982241 10.9077743902439 0.301432426821673
3 44 histone H3 acetylation
GO:0001832 0.00344997965876219 26.8809746954077 0.089059580651858
2 13 blastocyst growth
GO:0006511 0.00350669296113077 3.45622916409433 2.49366825825202
8 364 ubiquitin-dependent protein catabolic process
GO:0008629 0.00365319924625485 6.82488038277512 0.630267801536226
4 92 induction of apoptosis by intracellular signals
GO:0019941 0.00393365268343782 3.3878848424303 2.54162341706456 8
371 modification-dependent protein catabolic process
GO:0051457 0.00400706415907981 24.639175257732 0.0959103176250778 2
14 maintenance of protein location in nucleus
GO:0043632 0.00412851483988718 3.359394703657 2.56217562798422 8
374 modification-dependent macromolecule catabolic process
GO:0032434 0.00431473509296612 9.93541666666667 0.328835374714553
3 48 regulation of proteasomal ubiquitin-dependent protein catabolic process
GO:0000070 0.0045735241388741 9.71875 0.335686111687772 3 49
mitotic sister chromatid segregation
GO:0031146 0.00460297042707586 22.7422680412371 0.102761054598298
2 15 SCF-dependent proteasomal ubiquitin-dependent protein catabolic process
GO:0051603 0.00521737929362048 3.22367396383144 2.66493668258252
8 389 proteolysis involved in cellular protein catabolic process
GO:0032786 0.00523715134210935 21.1163475699558 0.109611791571518
2 16 positive regulation of DNA-dependent transcription, elongation
GO:0000819 0.00540553901069568 9.1218112244898 0.356238322607432 3
52 sister chromatid segregation
GO:0006917 0.00570987006360094 3.17233154442457 2.70604110442184
8 395 induction of apoptosis
GO:0012502 0.00596907556326967 3.14725274725275 2.7265933153415 8
398 induction of programmed cell death
GO:0044257 0.00632855386116627 3.11440843420539 2.75399626323438
8 402 cellular protein catabolic process
GO:0061136 0.00698286457656797 8.27430555555556 0.390492007473531
3 57 regulation of proteasomal protein catabolic process
GO:0051028 0.00709591086384157 5.60550909985243 0.760431804027403
4 111 mRNA transport
GO:0006103 0.00736395360587303 17.3862947240752 0.130164002491177
2 19 2-oxoglutarate metabolic process
GO:0031365 0.00736395360587303 17.3862947240752 0.130164002491177
2 19 N-terminal protein amino acid modification
GO:0031648 0.00736395360587303 17.3862947240752 0.130164002491177
2 19 protein destabilization
GO:0045637 0.0087689923227001 5.25872576177285 0.808386962839942
4 118 regulation of myeloid cell differentiation
GO:0051052 0.00903878426481114 4.16451157869989 1.27423707701889
5 186 regulation of DNA metabolic process
Tissue: Whole_Blood=>Thyroid Type: asymmetric
SourceGene: SREBF2(ENSG00000198911.6)
TargetGeneSet: RPL22 C1QA C1QC C1QB RP4 RPS27 HMGN2P18

FCGR2B RPS7 RNF181 RPL31 RPL32 TBC1D5 RPSA RPL24 CD200R1 CD86
CHST13 TET2 AMACR ERAP1 SKP1 CD14 CSF1R CTB RP11 RPL10A
GPR63 HMGB1P17 GATA4 RPL7 RPL35 SET CARD9 RPL13AP5
FAM204A VENTX MS4A6A FOLR2 RPS25 NCKAP1L NACA RNASE6 PLCB2 CTD
GTF2A2 PSTPIP1 SHCBP1 RPL17 RPL19 PHLPP1 VAV1 RPS11 RPL28 CRLS1
PXMP4 FAM176C ITGB2 WAS

GOBPID	Pvalue	OddsRatio	ExpCount	Count	Size	Term
GO:0006415	5.46155160304568e-22	80.7800751879699	0.31485710331465	15	91	translational termination
GO:0006414	5.36924710258027e-21	68.147619047619	0.363296657670749	15	105	translational elongation
GO:0006614	5.36924710258027e-21	68.147619047619	0.363296657670749	15	105	SRP-dependent cotranslational protein targeting to membrane
GO:0006613	6.23999991538573e-21	67.3940345368917	0.366756625839042	15	106	cotranslational protein targeting to membrane
GO:0045047	6.23999991538573e-21	67.3940345368917	0.366756625839042	15	106	protein targeting to ER
GO:0072599	6.23999991538573e-21	67.3940345368917	0.366756625839042	15	106	establishment of protein localization to endoplasmic reticulum
GO:0070972	3.38727394618744e-20	59.4923717059639	0.408276243858556	15	118	protein localization to endoplasmic reticulum
GO:0000184	3.86696228041177e-20	58.9162087912088	0.411736212026849	15	119	nuclear-transcribed mRNA catabolic process, nonsense-mediated decay
GO:0034623	3.34592963438323e-19	42.421444527178	0.602034461282956	16	174	cellular macromolecular complex disassembly
GO:0032984	5.31378881271477e-19	41.1057380007218	0.61933430212442	16	179	macromolecular complex disassembly
GO:0019080	1.56537407444312e-18	44.952731092437	0.522455193412221	15	151	viral genome expression
GO:0019083	1.56537407444312e-18	44.952731092437	0.522455193412221	15	151	viral transcription
GO:0006413	1.73278747921261e-18	44.621480709072	0.525915161580513	15	152	translational initiation
GO:0006612	1.91673349877618e-18	44.2950310559006	0.529375129748806	15	153	protein targeting to membrane
GO:0043624	1.91673349877618e-18	44.2950310559006	0.529375129748806	15	153	cellular protein complex disassembly
GO:0043241	3.14143990266426e-18	42.7312687312687	0.546674970590271	15	158	protein complex disassembly
GO:0006401	4.80346781339796e-18	35.3862433862434	0.709293474500035	16	205	RNA catabolic process
GO:0000956	8.8021397121475e-18	39.6484230055659	0.584734620441492	15	169	nuclear-transcribed mRNA catabolic process
GO:0006402	1.9411925709822e-17	37.4355828220859	0.615874333956128	15	178	mRNA catabolic process
GO:0072594	1.52997242797413e-16	32.2267573696145	0.705833506331742	15	204	establishment of protein localization to organelle
GO:0071845	8.77764533169957e-16	24.8165057067603	0.98263095979517	16	284	cellular component disassembly at cellular level
GO:0019058	9.88046726504687e-16	28.1448412698413	0.799252646875649	15	231	viral infectious cycle
GO:0022411	1.09469512259589e-15	24.4446366782007	0.996470832468341	16	288	cellular component disassembly
GO:0022415	5.74240783472818e-15	24.7626822157434	0.899591723756141	15	260	viral reproductive process
GO:0016032	7.15927499949212e-14	16.5786203044268	1.56044564390008	17	451	viral reproduction
GO:0033365	3.34230472782977e-11	13.0470991890206	1.63656494360252	15	473	protein localization to organelle
GO:0006605	3.4434807145423e-11	13.0177404295051	1.64002491177081	15	474	protein targeting
GO:0042274	3.06614596131556e-05	61.2170212765957	0.0622794270292713	3	18	ribosomal small subunit biogenesis

GO:0002253	7.05025891710982e-05	7.86580121057662	1.03453048231956
7	299	activation of immune response
GO:0000028	0.000174438093056294	149.96875	0.0207598090097571	2
6	ribosomal small subunit assembly
GO:0050778	0.000238634397527419	6.40401276789786	1.2594284132586 7
364	positive regulation of immune response
GO:0006958	0.000681278069094101	19.4938886373925	0.172998408414643
3	50	complement activation, classical pathway
GO:0022613	0.00104845951084384	7.16212121212121	0.778492837865892
5	225	ribonucleoprotein complex biogenesis
GO:0006956	0.00133742369874619	15.2563829787234	0.21797799460245
3	63	complement activation
GO:0042255	0.00136496319274614	42.8184523809524	0.0553594906926856
2	16	ribosome assembly
GO:0071843	0.00137103252344914	6.72697056030389	0.826932392221992
5	239	cellular component biogenesis at cellular level
GO:0001817	0.00142445078473875	5.42673190831831	1.24212857241713
6	359	regulation of cytokine production
GO:0002455	0.00146397411224734	14.7621825669183	0.224897930939035
3	65	humoral immune response mediated by circulating immunoglobulin
GO:0046632	0.00146397411224734	14.7621825669183	0.224897930939035
3	65	alpha-beta T cell differentiation
GO:0043372	0.00154354313670416	39.9611111111111	0.0588194588609785
2	17	positive regulation of CD4-positive, alpha-beta T cell differentiation
GO:0042254	0.00172982013550401	8.54932533733133	0.515535257075635
4	149	ribosome biogenesis
GO:0045076	0.00193222176422743	35.2549019607843	0.0657393951975642
2	19	regulation of interleukin-2 biosynthetic process
GO:2000516	0.00193222176422743	35.2549019607843	0.0657393951975642
2	19	positive regulation of CD4-positive, alpha-beta T cell activation
GO:0071706	0.00204450628494989	13.0677811550152	0.252577676285378
3	73	tumor necrosis factor superfamily cytokine production
GO:0001816	0.00230931400200473	4.9118953026408 1.36668742647568	6
395	cytokine production
GO:0042094	0.00236244676993409	31.5394736842105	0.0726593315341499
2	21	interleukin-2 biosynthetic process
GO:0030225	0.00259296101324586	29.9604166666667	0.0761192997024427
2	22	macrophage differentiation
GO:0046631	0.00284836220456694	11.5717748451387	0.283717389800014
3	82	alpha-beta T cell activation
GO:0072376	0.00284836220456694	11.5717748451387	0.283717389800014
3	82	protein activation cascade
GO:0006909	0.00325985358161567	11.0110228146629	0.297557262473185
3	86	phagocytosis
GO:0002521	0.0037865254952186	5.2764683875795 1.04491038682444	5
302	leukocyte differentiation
GO:0043370	0.00389673425792935	23.96	0.093419140543907	2	27
regulation of CD4-positive, alpha-beta T cell differentiation
GO:0001819	0.00393115330481027	6.7559990496555 0.647014047470763	4
187	positive regulation of cytokine production
GO:0022618	0.00431401968990256	9.92761332099908	0.328696975987821
3	95	ribonucleoprotein complex assembly
GO:2000514	0.00448750684712852	22.1820987654321	0.100339076880493
2	29	regulation of CD4-positive, alpha-beta T cell activation
GO:0042110	0.00477401966054854	4.98478414720453	1.10372984568542
5	319	T cell activation
GO:0046638	0.00479746029912768	21.3883928571429	0.103799045048786
2	30	positive regulation of alpha-beta T cell differentiation
GO:0071826	0.00497922049308118	9.4125904803685 0.345996816829285	3
100	ribonucleoprotein complex subunit organization
GO:0030097	0.00504328122791284	4.16073204694649	1.60196526191959
6	463	hemopoiesis

GO:0032760	0.0054461620521191	19.9597222222222	0.110718981385371
2	32	positive regulation of tumor necrosis factor production
GO:0006364	0.00555360540278177	9.03728670739414	0.359836689502457
3	104	rRNA processing
GO:0016072	0.0064872011323432	8.52694372638696	0.380596498512214
3	110	rRNA metabolic process
GO:0016064	0.0066513335552234	8.44739952718676	0.384056466680507
3	111	immunoglobulin mediated immune response
GO:0048534	0.00694240379473592	3.87953151143335	1.71268424330496
6	495	hemopoietic or lymphoid organ development
GO:0019724	0.0071585503246588l	8.21736630247269	0.394436371185385
3	114	B cell mediated immunity
GO:0030099	0.0077615939744837	5.52856307272373	0.785412774202477
4	227	myeloid cell differentiation
GO:0032663	0.00801194098606083	16.1756756756757	0.134938758563421
2	39	regulation of interleukin-2 production
GO:0046637	0.00841510248029055	15.7489035087719	0.138398726731714
2	40	regulation of alpha-beta T cell differentiation
GO:0006959	0.00862134695839894	7.66064723761845	0.422116116531728
3	122	humoral immune response
GO:0002757	0.0087452078709616	5.33408620365142	0.81309251954882
4	235	immune response-activating signal transduction
GO:0043367	0.00882722417092368	15.3440170940171	0.141858694900007
2	41	CD4-positive, alpha-beta T cell differentiation
GO:0035710	0.00967809142555777	14.5934959349593	0.148778631236593
2	43	CD4-positive, alpha-beta T cell activation
GO:0046635	0.00967809142555777	14.5934959349593	0.148778631236593
2	43	positive regulation of alpha-beta T cell activation
Tissue: Whole_Blood=>Thyroid	Type:	cluster
SourceGene:	SST(ENSG00000157005.3)
TargetGeneSet:	LRRC47	KIAA0090		MFI2	UQCRH	RP4	GSTM3	NBPF10
PIAS3	ADAR	ZBTB7B	METTL13	QSOX1	DHX9	URB2	HEATR1	MORN2	MSH6
HMGA1P8	POLR1B	TMEM185B		ITGB6	GORASP2	7SK	FARP2	XCR1	RP11
LETM1	CNGA1	NUP155	CTD	SKIV2L2	PHAX	SEC24A	AFAP1L1	PRRC2A	ZBTB9	PGM3
LATS1	PRR18	HEATR2	C7orf26	POM121C	MEPCE	ABCF2	MTMR9	PRKDC	UQCRB
ZFP41	STOML2	TLN1	UBQLN1	SEC16A	BMS1P7	SEC24C	SEC23IP	NUP98	SPTY2D1
PRPF19	UBE4A	ARCN1	DDX6	CBX5	ANKRD52	CAND1	GCN1L1	ZIC2	PRMT5-AS1
PRMT5	TUBGCP4	TNFAIP8L3		RNF111	RNF40	POLR2C	EDC4	PIEZO1	TIMM22	RP13
DPH1	TP53	GRAP	LIG3	TEX34	CA10	GNA11	FEM1A	ZNF562	RAVER1
CHERP	MPV17L2	TOMM40	GRWD1	TRIM28	CDH22	SLC2A10	ADNP	GCNT7	PRPF6	PWP2
CRKL	SF3A1	BCOR	SMC1A	APEX2	HCFC1
GOBPID	Pvalue	OddsRatio	ExpCount	Count	Size	Term
GO:0000377	1.15365543243125e-08	12.0624854999613	1.10110026987752
11	204	RNA splicing, via transesterification reactions with bulged adenosine as nucleophile
GO:0000398	1.15365543243125e-08	12.0624854999613	1.10110026987752
11	204	nuclear mRNA splicing, via spliceosome
GO:0000375	1.48367893969954e-08	11.7537313432836	1.1280880215902 11
209	RNA splicing, via transesterification reactions
GO:0071843	5.96174559502445e-07	9.08296943231441	1.29001453186631
10	239	cellular component biogenesis at cellular level
GO:0022618	7.66284453746082e-07	16.0043213828425	0.51276728254100l
7	95	ribonucleoprotein complex assembly
GO:0071826	1.08642122355679e-06	15.1385733757383	0.539755034253685
7	100	ribonucleoprotein complex subunit organization
GO:0008380	1.20153249783804e-06	7.39911021814007	1.7434087606394 11
323	RNA splicing
GO:0006397	1.39444324278229e-06	6.5708245243129 2.1536225866722 12	399
mRNA processing
GO:0022613	3.29035265921355e-06	8.54891304347826	1.21444882707079
9	225	ribonucleoprotein complex biogenesis
GO:0048199	1.2541880371997e-05	33.7250293772033	0.145733859248495
4	27	vesicle targeting, to, from or within Golgi

GO:0000387	2.21207823724036e-05	28.7207207207207	0.167324060618642
4	31	spliceosomal snRNP assembly
GO:0006901	3.62128744856227e-05	25.0078465562337	0.18891426198879
4	35	vesicle coating
GO:0006900	4.53053923311206e-05	23.4889434889435	0.199709362673863
4	37	membrane budding
GO:0006903	4.53053923311206e-05	23.4889434889435	0.199709362673863
4	37	vesicle targeting
GO:0048207	6.56901663562067e-05	47.87	0.0809632551380527	3	15
vesicle targeting, rough ER to cis-Golgi
GO:0048208	6.56901663562067e-05	47.87	0.0809632551380527	3	15
COPII vesicle coating
GO:0090114	6.56901663562067e-05	47.87	0.0809632551380527	3	15
COPII-coated vesicle budding
GO:0051650	0.000127608563505716	17.6031941031941	0.259082416441769
4	48	establishment of vesicle localization
GO:0006122	0.000171364275136886	189.092105263158	0.0215902013701474
2	4	mitochondrial electron transport, ubiquinol to cytochrome c
GO:0006281	0.000213198878765418	4.88223668914206	2.06726178119161
9	383	DNA repair
GO:0002326	0.000284606396846764	126.052631578947	0.0269877517126842
2	5	B cell lineage commitment
GO:0051648	0.000414255240652964	12.6823216659282	0.350840772264895
4	65	vesicle localization
GO:0000244	0.000425414344459507	94.5328947368421	0.0323853020552211
2	6	assembly of spliceosomal tri-snRNP
GO:0007006	0.00073451305538224	19.124	0.178119161303716	3	33
mitochondrial membrane organization
GO:0051095	0.000788557041987128	63.0131578947368	0.0431804027402948
2	8	regulation of helicase activity
GO:0006403	0.000835014385006894	7.44640803095263	0.734066846585011
5	136	RNA localization
GO:0016044	0.000983353545347316	4.31328455910666	2.04567157982147
8	379	cellular membrane organization
GO:0000245	0.00102994617045959	16.8694117647059	0.199709362673863
3	37	spliceosomal complex assembly
GO:0061024	0.00103451672924188	4.277769289534	2.06186423084908	8
382	membrane organization
GO:0016050	0.00147111728672856	8.87604846225536	0.491177081170853
4	91	vesicle organization
GO:0006997	0.00182360238199878	13.6485714285714	0.242889765414158
3	45	nucleus organization
GO:0007007	0.00183288240692901	37.7973684210526	0.0647706041104422
2	12	inner mitochondrial membrane organization
GO:0008340	0.00183288240692901	37.7973684210526	0.0647706041104422
2	12	determination of adult lifespan
GO:0051656	0.00215795043950319	7.95541933686264	0.545152584596222
4	101	establishment of organelle localization
GO:0002360	0.00215856763415339	34.3588516746412	0.070168154452979
2	13	T cell lineage commitment
GO:0006486	0.00238648340456517	4.78556910569106	1.36018268631929
6	252	protein glycosylation
GO:0043413	0.00238648340456517	4.78556910569106	1.36018268631929
6	252	macromolecule glycosylation
GO:0070085	0.0025315329577764	4.72690763052209	1.3763753373469 6
255	glycosylation
GO:0006888	0.00324380592768268	11.0161538461538	0.296865268839527
3	55	ER to Golgi vesicle-mediated transport
GO:0051053	0.00377127759435451	10.4130909090909	0.313057919867137
3	58	negative regulation of DNA metabolic process
GO:0010390	0.00416076748304574	23.6134868421053	0.0971559061656633
2	18	histone monoubiquitination
GO:0009303	0.00463406501533718	22.2229102167183	0.1025534565082 2

19	rRNA transcription
GO:0050657	0.00519506983914373	6.1612972972973 0.696283994187254	4
129	nucleic acid transport
GO:0050658	0.00519506983914373	6.1612972972973 0.696283994187254	4
129	RNA transport
GO:0051236	0.00519506983914373	6.1612972972973 0.696283994187254	4
129	establishment of RNA localization
GO:0006310	0.00541934970834893	4.75725221595487	1.1280880215902 5
209	DNA recombination
GO:0009101	0.00587069167689304	3.94949494949495	1.63545775378867
6	303	glycoprotein biosynthetic process
GO:0006471	0.0061948900365192	18.8855263157895	0.118746107535811
2	22	protein ADP-ribosylation
GO:0010259	0.00735028424056083	17.1662679425837	0.129541208220884
2	24	multicellular organismal aging
GO:0006289	0.00801726283746006	7.83561643835616	0.4102138260328 3
76	nucleotide-excision repair
GO:0000291	0.00859516597238188	15.7335526315789	0.140336308905958
2	26	nuclear-transcribed mRNA catabolic process, exonucleolytic
GO:0043928	0.00859516597238188	15.7335526315789	0.140336308905958
2	26	exonucleolytic nuclear-transcribed mRNA catabolic process involved in deadenylation-dependent decay
GO:0015931	0.00878355991970019	5.26730840429471	0.809632551380527
4	150	nucleobase-containing compound transport
GO:0051640	0.00878355991970019	5.26730840429471	0.809632551380527
4	150	organelle localization
GO:0016925	0.0099275250191223	14.5212550607287	0.151131409591032
2	28	protein sumoylation
Tissue: Whole_Blood=>Thyroid	Type:	cluster
SourceGene:	TMEM191A(ENSG00000226287.3)
TargetGeneSet:	NOC2L	UBE2J2	SPEN	NUDC	EYA3	TXLNA	EIF2C3	STK40
AKIRIN1 TOE1	SELRC1	RP4	PYGO2	UBQLN4	CCT3	ETV3	TTC32	ERLEC1
PCBP1	TMEM127 RBP4	HSPD1	C2orf69 INHA	CLN5	NKIRAS1 TMEM158 TMEM115
FYTTD1	LETM1	GRPEL1	RP11	ANKRD17 EIF4E	CCRN4L	ANKRA2	PHAX	SLC35A4
RBM22	ABHD16A LY6G6E	DAXX	BRPF3	TBC1D22B	RPL7L1	RP13	ARID1B
TULP4	USP42	PURB	HUS1	BAZ1B	MEPCE	ABCF2	PINX1	LSM12P1 KAT6A
DNAJA1	TEX10	TBC1D13 TOR1B	RNLS	LZTS2	NOLC1	AFAP1L2 TSSC4	NUP98	NXF1
MARK2	STIP1	SF1	C11orf68	PAFAH1B2	C2CD2L	SC5DL	HSPA8
PKNOX2	FKBP4	NOP2	FMNL3	AVIL	GCN1L1	GPR133	PUS1	PDS5B	TARDBPP2
FAM70B	SALL2	RBM23	PRMT5	EIF2B2	DIO2	IDH3A	DNAJA4	CHRNA3	CD2BP2
TBC1D10B	RNF40	SETD1A	E2F4	CIRH1A	USP10	VPS53	TSR1	ALKBH5
USP22	GIT1	PPP1R9B MED13	SIRT7	RBBP8	NFIC	MAP2K2	FEM1A	ZNF562
ELOF1	DNAJB1	BRD4	ARMC6	GATAD2A IRF2BP1 GRWD1	KCNJ14	FIZ1	TRIM28
ZNF337	RP3	LINC00478	IGLC5	ST13	L3MBTL2 SMC1A
GOBPID	Pvalue	OddsRatio	ExpCount	Count	Size	Term
GO:0016570	3.6230625639836e-06	6.45975 1.93253062071829	11	261
histone modification
GO:0006457	4.49158677109177e-06	7.07536783104794	1.59933568611169
10	216	protein folding
GO:0016569	4.50585519221402e-06	6.3056640625	1.97695661199917	11
267	covalent chromatin modification
GO:0016568	1.29774902123674e-05	4.74777277014988	3.10241505778147
13	419	chromatin modification
GO:0000377	2.02722703576904e-05	6.66357927786499	1.51048370354993
9	204	RNA splicing, via transesterification reactions with bulged adenosine as nucleophile
GO:0000398	2.02722703576904e-05	6.66357927786499	1.51048370354993
9	204	nuclear mRNA splicing, via spliceosome
GO:0000375	2.45554717631266e-05	6.49469387755102	1.54750536295066
9	209	RNA splicing, via transesterification reactions
GO:0000087	7.19801848441197e-05	4.59482449856733	2.66555947685281
11	360	M phase of mitotic cell cycle
GO:0000387	7.663344165069e-05	20.5925925925926	0.229534288284548

4	31	spliceosomal snRNP assembly
GO:0042026	8.27114288575499e-05	45.9455128205128	0.0888519825617604
3	12	protein refolding
GO:0008380	0.00013985244034312	4.62138928230295	2.39159919728738
10	323	RNA splicing
GO:0006397	0.000178321613870727	4.12145618556701	2.95432842017853
11	399	mRNA processing
GO:0006986	0.000389673072422535	6.86838927795219	0.955158812538925
6	129	response to unfolded protein
GO:0061077	0.000476344630755438	22.9583333333333	0.155490969483081
3	21	chaperone-mediated protein folding
GO:0035966	0.000516382493018023	6.4953541507997 1.00698913569995	6
136	response to topologically incorrect protein
GO:0006458	0.000543270386402793	11.8132617227845	0.377620925887482
4	51	'de novo' protein folding
GO:0022618	0.000691968692341552	7.7636165577342 0.703411528613937	5
95	ribonucleoprotein complex assembly
GO:0000244	0.00079907177952278	68.2857142857143	0.0444259912808802
2	6	assembly of spliceosomal tri-snRNP
GO:0048096	0.00079907177952278	68.2857142857143	0.0444259912808802
2	6	chromatin-mediated maintenance of transcription
GO:0070389	0.00079907177952278	68.2857142857143	0.0444259912808802
2	6	chaperone cofactor-dependent protein refolding
GO:0071826	0.000873285052963591	7.35242518059855	0.74043318801467
5	100	ribonucleoprotein complex subunit organization
GO:0030521	0.00113948759308705	9.56545028456645	0.459068576569096
4	62	androgen receptor signaling pathway
GO:0000280	0.00115389361514591	3.77123107307439	2.59151615805135
9	350	nuclear division
GO:0007067	0.00115389361514591	3.77123107307439	2.59151615805135
9	350	mitosis
GO:0016573	0.00123404834008591	6.77755568246716	0.799667843055844
5	108	histone acetylation
GO:0018393	0.00133927048885431	6.64752567693744	0.814476506816137
5	110	internal peptidyl-lysine acetylation
GO:0022613	0.00141082096378194	4.53587155963303	1.66597467303301
7	225	ribonucleoprotein complex biogenesis
GO:0018394	0.00150933750578905	6.46151053013798	0.836689502456577
5	113	peptidyl-lysine acetylation
GO:0006475	0.00163122169908091	6.34313725490196	0.851498166216871
5	115	internal protein amino acid acetylation
GO:0048285	0.00169383049192516	3.55721069591271	2.73960279565428
9	370	organelle fission
GO:0006405	0.0016973727916986	8.531142643764	0.510898899730122	4
69	RNA export from nucleus
GO:0045931	0.00183206250357425	13.7634615384615	0.244342952044841
3	33	positive regulation of mitotic cell cycle
GO:0071843	0.00199011420128346	4.25793103448276	1.76963531935506
7	239	cellular component biogenesis at cellular level
GO:0051085	0.00235123801475254	34.1333333333333	0.074043318801467
2	10	chaperone mediated protein folding requiring cofactor
GO:0044419	0.00259200549074806	3.32973760932945	2.9173067607778 9
394	interspecies interaction between organisms
GO:0006473	0.00260718603424917	5.66754344014028	0.947754480658778
5	128	protein acetylation
GO:0051169	0.00263737072435057	3.62242230612838	2.37679053352709
8	321	nuclear transport
GO:0050657	0.00269660187948411	5.62144212523719	0.955158812538925
5	129	nucleic acid transport
GO:0050658	0.00269660187948411	5.62144212523719	0.955158812538925
5	129	RNA transport
GO:0051236	0.00269660187948411	5.62144212523719	0.955158812538925
5	129	establishment of RNA localization

GO:0007064	0.00285987547293078	30.3386243386243	0.0814476506816137
2	11	mitotic sister chromatid cohesion
GO:0006403	0.00338597480719715	5.31844035324053	1.00698913569995
5	136	RNA localization
GO:0018205	0.00395043290127815	5.12110726643599	1.04401079510069
5	141	peptidyl-lysine modification
GO:0043966	0.00419286621707235	10.06308630394	0.325790602726455	3
44	histone H3 acetylation
GO:0043543	0.00431979414912155	5.00952179432924	1.06622379074113
5	144	protein acylation
GO:0045815	0.00466373673300363	22.7492063492064	0.103660646322054
2	14	positive regulation of gene expression, epigenetic
GO:0051084	0.0047547071160943	9.59369409660107	0.340599266486748
3	46	'de novo' posttranslational protein folding
GO:0015931	0.00513145534617908	4.80020283975659	1.11064978202201
5	150	nucleobase-containing compound transport
GO:0000070	0.00567913236572984	8.96613712374582	0.362812262127188
3	49	mitotic sister chromatid segregation
GO:0010458	0.00609095393581712	19.4965986394558	0.118469310082347
2	16	exit from mitosis
GO:0030518	0.00669499024630151	5.70393354018617	0.747837519894817
4	101	intracellular steroid hormone receptor signaling pathway
GO:0000819	0.0067041453513425l	8.41542386185243	0.385025257767629
3	52	sister chromatid segregation
GO:0006302	0.00692927868272914	5.64533386170002	0.755241851774964
4	102	double-strand break repair
GO:0007346	0.00727626059367854	3.32216216216216	2.24351255968445
7	303	regulation of mitotic cell cycle
GO:0006281	0.00755558402172471	3.01015488215488	2.83585911009619
8	383	DNA repair
GO:0051168	0.00872236616921661	5.26638927415626	0.807072174935991
4	109	nuclear export
GO:0071478	0.00906605014910685	7.49423076923077	0.429451249048509
3	58	cellular response to radiation
GO:0051028	0.00928533524098818	5.16722620451865	0.821880838696284
4	111	mRNA transport
GO:0006913	0.00936147059222981	3.15855305466238	2.35457753788665
7	318	nucleocytoplasmic transport
GO:0016571	0.00950107513571365	7.35989010989011	0.436855580928655
3	59	histone methylation
GO:0006406	0.00994811026184323	7.23026315789474	0.444259912808802
3	60	mRNA export from nucleus
Tissue: Whole_Blood=>Thyroid	Type:	cluster
SourceGene:	TMEM64(ENSG00000180694.9)
TargetGeneSet:	FCRL3	RP11	FCRL2	IGKJ5	IGKC	IGKJ4	IGKJ3	IGKJ2
IGKV1-6	IGKV3-7	IGKV1-9	IGKV1-13	IGKV1-17	IGKV2-26	IGKV1-27
IGKV2-28	IGKV2-29	IGKV1-33	IGKV1-39	IGKV2-40
IGKV1D-33	IGKV2D-29	IGKV2D-28	IGKV3D-20	IGKV1D-16
IGKV1D-13	IGKV3D-7	IGJ	CTD	CTB	DNAH8	RGS17	ARHGEF10
CENPP	RIN1	AMOTL1	GS1	IGHG3	KIAA0125	IGHV1-3	IGHV4-4	IGHV3-13
IGHV1-18	IGHV4-28	IGHV4-31	IGHV3-33	IGHV3-43
IGHV1-46	IGHV5-51	IGHV4-55	IGHV2-70	IGHV3-73
IGHV3-74	IGHV5-78	DOC2B	TNFRSF13B	CD79A	LINC00176
PPM1F	IGLV9-49	IGLV1-47	IGLV5-45	IGLV1-40	IGLV3-25
IGLV3-21	IGLV3-1	IGLL5	IGLC1	IGLC3	IGLC7	PHF21B
GOBPID	Pvalue	OddsRatio	ExpCount	Count	Size	Term
GO:0006958	4.17272448412705e-17	210.526829268293	0.0830392360390284
9	50	complement activation, classical pathway
GO:0006956	3.89403027699151e-16	159.7	0.104629437409176	9	63
complement activation
GO:0002455	5.24961254567502e-16	153.975	0.107951006850737	9	65
humoral immune response mediated by circulating immunoglobulin
GO:0072376	4.73636565626121e-15	117.978082191781	0.136184347104007

9	82	protein activation cascade
GO:0016064	7.94729347232045e-14	84.2647058823529	0.184347104006643
9	111	immunoglobulin mediated immune response
GO:0019724	1.01656580271012e-13	81.84	0.189329458168985	9	114
B cell mediated immunity
GO:0006959	1.89850781575545e-13	76.0035398230089	0.202615735935229
9	122	humoral immune response
GO:0002449	3.33579003599497e-12	54.1860759493671	0.277351048370355
9	167	lymphocyte mediated immunity
GO:0002460	6.91206296908552e-12	49.7267441860465	0.300602034461283
9	181	adaptive immune response based on somatic recombination of immune receptors built from immunoglobulin superfamily domains
GO:0002250	1.55296624225031e-11	45.2	0.328835374714553	9	198
adaptive immune response
GO:0002253	1.87926130523349e-11	34.9431537320811	0.49657463151339
10	299	activation of immune response
GO:0002443	2.75033885290613e-11	42.2524752475247	0.3504255760847 9
211	leukocyte mediated immunity
GO:0050778	1.3027059467683e-10	28.3958837772397	0.604525638364127
10	364	positive regulation of immune response
GO:0002252	5.78430500835518e-09	22.3607427055703	0.6410629022213 9
386	immune effector process
GO:0051496	0.00083845629162471б	54.5568181818182	0.0431804027402948
2	26	positive regulation of stress fiber assembly
GO:0032233	0.0011177006176441	46.75	0.0498235416234171	2	30
positive regulation of actin filament bundle assembly
GO:0051492	0.00169914447007782	37.3818181818182	0.061449034668881
2	37	regulation of stress fiber assembly
GO:0032231	0.00228983935999548	31.8980044345898	0.0714137429935645
2	43	regulation of actin filament bundle assembly
GO:0043149	0.00239645191615069	31.1363636363636	0.073074527714345
2	44	stress fiber assembly
GO:0010638	0.00332423105454637	11.3071428571429	0.303923603902844
3	183	positive regulation of organelle organization
GO:0042100	0.0039902279309635б	23.7553719008264	0.0946647290844924
2	57	B cell proliferation
GO:0031585	0.00497442779656243	313.586956521739	0.00498235416234171
1	3	regulation of inositol 1,4,5-trisphosphate-sensitive calcium-release channel activity
GO:0031987	0.00497442779656243	313.586956521739	0.00498235416234171
1	3	locomotion involved in locomotory behavior
GO:0048791	0.00497442779656243	313.586956521739	0.00498235416234171
1	3	calcium ion-dependent exocytosis of neurotransmitter
GO:0051643	0.00497442779656243	313.586956521739	0.00498235416234171
1	3	endoplasmic reticulum localization
GO:0042552	0.00563234430661618	19.7809917355372	0.112933361013079
2	68	myelination
GO:0051017	0.00579461598130192	19.4843962008141	0.114594145733859
2	69	actin filament bundle assembly
GO:0007272	0.00629421014504686	18.6454545454545	0.119576499896201
2	72	ensheathment of neurons
GO:0008366	0.00629421014504686	18.6454545454545	0.119576499896201
2	72	axon ensheathment
GO:0009313	0.0066272941955714	209.04347826087 0.00664313888312228	1
4	oligosaccharide catabolic process
GO:0031340	0.0066272941955714	209.04347826087 0.00664313888312228	1
4	positive regulation of vesicle fusion
GO:0042759	0.0066272941955714	209.04347826087 0.00664313888312228	1
4	long-chain fatty acid biosynthetic process
GO:0019228	0.0084809763532936	15.9035476718404	0.139505916545568
2	84	regulation of action potential in neuron
GO:0051495	0.00927550456992079	15.1596194503171	0.14614905542869
2	88	positive regulation of cytoskeleton organization

GO:0000244	0.00992513678201701	125.408695652174	0.00996470832468341
1	6	assembly of spliceosomal tri-snRNP
Tissue: Whole_Blood=>Nerve_Tibial	Type:	asymmetric
SourceGene:	CTD-2313J17.1(ENSG00000258922.1)
TargetGeneSet:	CHCHD2P6	HSPB7	CCT8P1	RP11	CHRNB2	DPT	ACVR1C
KRT8P15	GPBAR1	DES	SLC19A3	TIMP4	ADIPOQ	ADIPOQ-AS1	LVRN	PPARGC1B
MLXIPL	AQP9	LRRC37A5P	AGPAT2	C10orf71	RBP4	GPAM	ACSL5
LGALS12	RPS3AP40	MYEOV	GPD1	PPP1R1A	ACACB	MYH7	PLIN1	TUSC5
STAT5A	CA4	SCN4A	CIDEA	PLIN4	PLIN5	SLC7A10	LIPE	FTCD-AS1
SULT4A1	GYG2	PFKFB1
GOBPID	Pvalue	OddsRatio	ExpCount	Count	Size	Term
GO:0006641	7.95965961852856e-08	34.6038647342995	0.223790741125182
6	98	triglyceride metabolic process
GO:0006639	1.07255759533129e-07	32.8087056128293	0.235208636080548
6	103	acylglycerol metabolic process
GO:0006638	1.13639611300147e-07	32.4716553287982	0.237492215071621
6	104	neutral lipid metabolic process
GO:0019216	2.24183935298213e-07	20.4888934594817	0.443014324268217
7	194	regulation of lipid metabolic process
GO:0009749	4.26731426718578e-06	24.8179611650485	0.246626531035914
5	108	response to glucose stimulus
GO:0009746	5.33018893176607e-06	23.6607142857143	0.258044425991281
5	113	response to hexose stimulus
GO:0034284	5.80925195704711e-06	23.2272727272727	0.262611583973427
5	115	response to monosaccharide stimulus
GO:0019432	6.09748631581001e-06	40.4475721323012	0.121029686526884
4	53	triglyceride biosynthetic process
GO:0046460	7.07899897634248e-06	38.8559837728195	0.125596844509031
4	55	neutral lipid biosynthetic process
GO:0046463	7.07899897634248e-06	38.8559837728195	0.125596844509031
4	55	acylglycerol biosynthetic process
GO:0010876	8.00881384652893e-06	15.1079213184476	0.490969483080756
6	215	lipid localization
GO:0009743	1.13902959533377e-05	20.0942069741282	0.301432426821673
5	132	response to carbohydrate stimulus
GO:0043434	2.01369832473102e-05	10.0821538461538	0.872327174589994
7	382	response to peptide hormone stimulus
GO:0046486	2.30751876697959e-05	12.4418093983311	0.59144695868798
6	259	glycerolipid metabolic process
GO:0006112	3.25040083304447e-05	16.0141509433962	0.374506954536018
5	164	energy reserve metabolic process
GO:0006091	4.17181105858948e-05	8.95112369815458	0.977371808179365
7	428	generation of precursor metabolites and energy
GO:0006631	4.18734110600985e-05	11.1394799054374	0.657670749429105
6	288	fatty acid metabolic process
GO:0045834	5.46501790211906e-05	22.4608150470219	0.210089267178742
4	92	positive regulation of lipid metabolic process
GO:0044262	7.06567202148421e-05	13.5163373860182	0.440730745277143
5	193	cellular carbohydrate metabolic process
GO:0006006	7.97540034018014e-05	13.1615470022206	0.45214864023251
5	198	glucose metabolic process
GO:0046889	0.00013480857295596	35.0658536585366	0.100477475607224
3	44	positive regulation of lipid biosynthetic process
GO:0019318	0.00017482274792078	11.0644104803493	0.534357483911148
5	234	hexose metabolic process
GO:0009744	0.000180239911948168	132.820276497696	0.0205522109196595
2	9	response to sucrose stimulus
GO:0034285	0.000180239911948168	132.820276497696	0.0205522109196595
2	9	response to disaccharide stimulus
GO:0055089	0.000180239911948168	132.820276497696	0.0205522109196595
2	9	fatty acid homeostasis
GO:0019915	0.000186082374057382	31.2434782608696	0.111895370562591
3	49	lipid storage

GO:0010867	0.00022497792689792	116.209677419355	0.0228357899107328
2	10	positive regulation of triglyceride biosynthetic process
GO:0032868	0.000246715170641447	10.2450838635049	0.575461905750467
5	252	response to insulin stimulus
GO:0032787	0.000257958470179801	7.88917018284107	0.915715175420386
6	401	monocarboxylic acid metabolic process
GO:0005996	0.000260608018732448	10.12	0.582312642723687	5	255
monosaccharide metabolic process
GO:0033032	0.000274580123993068	103.290322580645	0.0251193689018061
2	11	regulation of myeloid cell apoptotic process
GO:0006094	0.000307315513526828	26.1145454545455	0.13244758148225
3	58	gluconeogenesis
GO:0016024	0.000329025405415394	92.9548387096774	0.0274029478928794
2	12	CDP-diacylglycerol biosynthetic process
GO:0019319	0.000374340354630484	24.3372881355932	0.141581897446543
3	62	hexose biosynthetic process
GO:0033028	0.000388292739320376	84.4985337243402	0.0296865268839527
2	13	myeloid cell apoptotic process
GO:0046341	0.000388292739320376	84.4985337243402	0.0296865268839527
2	13	CDP-diacylglycerol metabolic process
GO:0045017	0.000418976347448355	12.94555353902	0.356238322607432	4
156	glycerolipid biosynthetic process
GO:0040014	0.000430331682137595	23.1548387096774	0.148432634419763
3	65	regulation of multicellular organism growth
GO:0046364	0.000450123790556485	22.7857142857143	0.150716213410837
3	66	monosaccharide biosynthetic process
GO:0010866	0.000452361159660209	77.4516129032258	0.031970105875026
2	14	regulation of triglyceride biosynthetic process
GO:0070293	0.000452361159660209	77.4516129032258	0.031970105875026
2	14	renal absorption
GO:0003014	0.000470491835915483	22.428125	0.15299979240191	3
67	renal system process
GO:0010906	0.000470491835915483	22.428125	0.15299979240191	3
67	regulation of glucose metabolic process
GO:0019217	0.000470491835915483	22.428125	0.15299979240191	3
67	regulation of fatty acid metabolic process
GO:0051240	0.000506655532519243	6.91363523880228	1.03902844093834
6	455	positive regulation of multicellular organismal process
GO:0090208	0.000594817723377894	66.3778801843318	0.0365372638571725
2	16	positive regulation of triglyceride metabolic process
GO:0002761	0.000605182306922169	20.4971428571429	0.16670126634835
3	73	regulation of myeloid leukocyte differentiation
GO:0015980	0.000609943471952853	8.34673604541154	0.701058750259498
5	307	energy derivation by oxidation of organic compounds
GO:0016051	0.000718327161609398	11.1614420062696	0.411044218393191
4	180	carbohydrate biosynthetic process
GO:0042104	0.000756228677422032	58.0725806451613	0.0411044218393191
2	18	positive regulation of activated T cell proliferation
GO:0010675	0.000880251032037665	17.9225	0.189537056259082	3	83
regulation of cellular carbohydrate metabolic process
GO:0042493	0.000916446693937499	7.59980578334053	0.767282541000623
5	336	response to drug
GO:0006109	0.00100918327127551	17.0642857142857	0.198671372223376
3	87	regulation of carbohydrate metabolic process
GO:0090207	0.00113525328177882	46.4451612903226	0.0502387378036122
2	22	regulation of triglyceride metabolic process
GO:0031667	0.0011718462077691	7.17755102040816	0.810670541831015
5	355	response to nutrient levels
GO:0006732	0.00118699078097754	9.70706725844998	0.470417272161096
4	206	coenzyme metabolic process
GO:0046890	0.00122405919835558	15.92	0.212372846169815	3	93
regulation of lipid biosynthetic process
GO:0001817	0.00123174727611219	7.09443099273608	0.819804857795308

5 359 regulation of cytokine production
GO:0019433 0.00124159877728433 44.2304147465438 0.0525223167946855
2 23 triglyceride catabolic process
GO:0032370 0.00124159877728433 44.2304147465438 0.0525223167946855
2 23 positive regulation of lipid transport
GO:0043029 0.00124159877728433 44.2304147465438 0.0525223167946855
2 23 T cell homeostasis
GO:0045471 0.00126245280254452 15.743956043956 0.214656425160889 3
94 response to ethanol
GO:0097305 0.00130159908001424 15.5717391304348 0.216940004151962
3 95 response to alcohol
GO:0008016 0.00134150364896893 15.4032258064516 0.219223583143035
3 96 regulation of heart contraction
GO:0050671 0.00134150364896893 15.4032258064516 0.219223583143035
3 96 positive regulation of lymphocyte proliferation
GO:0046006 0.00135253927893591 42.217008797654 0.0548058957857588 2
24 regulation of activated T cell proliferation
GO:0046461 0.00135253927893591 42.217008797654 0.0548058957857588 2
24 neutral lipid catabolic process
GO:0046464 0.00135253927893591 42.217008797654 0.0548058957857588 2
24 acylglycerol catabolic process
GO:0032946 0.00138217208181276 15.2382978723404 0.221507162134108
3 97 positive regulation of mononuclear cell proliferation
GO:0070665 0.00150881560490668 14.7639175257732 0.228357899107328
3 100 positive regulation of leukocyte proliferation
GO:0009991 0.00154789068584402 6.7239563385676 0.863192858625701 5
378 response to extracellular stimulus
GO:0040018 0.00158812434901315 38.6935483870968 0.0593730537679053
2 26 positive regulation of multicellular organism growth
GO:0050798 0.00158812434901315 38.6935483870968 0.0593730537679053
2 26 activated T cell proliferation
GO:0045923 0.0017127286007007 37.1432258064516 0.0616566327589786
2 27 positive regulation of fatty acid metabolic process
GO:0032844 0.00172440672777148 8.74014778325123 0.520656009964708
4 228 regulation of homeostatic process
GO:0032720 0.00184184722471557 35.712158808933 0.0639402117500519 2
28 negative regulation of tumor necrosis factor production
GO:0033865 0.00184184722471557 35.712158808933 0.0639402117500519 2
28 nucleoside bisphosphate metabolic process
GO:0001816 0.00187841568405204 6.42307692307692 0.902013701473946
5 395 cytokine production
GO:0006936 0.00204818652604451 8.32457813646368 0.545775378866514
4 239 muscle contraction
GO:0051048 0.00208751363034533 13.1275229357798 0.255760847000208
3 112 negative regulation of secretion
GO:0016042 0.00214332846222422 8.21790785279629 0.552626115839734
4 242 lipid catabolic process
GO:0051186 0.00224149476476971 8.11389326083846 0.559476852812954
4 245 cofactor metabolic process
GO:0051046 0.00228223744714 8 6.13182773109244 0.943118123313265
5 413 regulation of secretion
GO:0003015 0.00230708936296899 12.6592920353982 0.264895162964501
3 116 heart process
GO:0060047 0.00230708936296899 12.6592920353982 0.264895162964501
3 116 heart contraction
GO:0044057 0.00237998888247377 6.07056171983356 0.952252439277559
5 417 regulation of system process
GO:0046503 0.00240306638746981 30.941935483871 0.073074527714345 2
32 glycerolipid catabolic process
GO:0045637 0.00242205427147634 12.4373913043478 0.269462320946647
3 118 regulation of myeloid cell differentiation
GO:0002763 0.00271024518725041 29.0040322580645 0.0776416856964916
2 34 positive regulation of myeloid leukocyte differentiation

GO:0002573	0.00285223938865716	11.7180327868852	0.28544737388416	3	125	myeloid leukocyte differentiation
GO:0003012	0.00301746110844096	7.45248749670966	0.607432011625493	4	266	muscle system process
GO:0051384	0.00339833948917098	10.9907692307692	0.303716005812747	3	133	response to glucocorticoid stimulus
GO:0071375	0.0034896220308333	7.14664645699128	0.632551380527299	4	277	cellular response to peptide hormone stimulus
GO:0015909	0.00355442549303487	25.075850043592	0.089059580651858	2	39	long-chain fatty acid transport
GO:0030049	0.00355442549303487	25.075850043592	0.089059580651858	2	39	muscle filament sliding
GO:0033275	0.00355442549303487	25.075850043592	0.089059580651858	2	39	actin-myosin filament sliding
GO:0044242	0.00369380886083433	10.6597014925373	0.31285032177704	3	137	cellular lipid catabolic process
GO:0002260	0.00373617448509086	24.4142614601019	0.0913431596429313	2	40	lymphocyte homeostasis
GO:0006637	0.00373617448509086	24.4142614601019	0.0913431596429313	2	40	acyl-CoA metabolic process
GO:0035383	0.00373617448509086	24.4142614601019	0.0913431596429313	2	40	thioester metabolic process
GO:0007565	0.00392542749391757	10.4240875912409	0.31970105875026	3	140	female pregnancy
GO:0031960	0.00408466676056487	10.2726618705036	0.324268216732406	3	142	response to corticosteroid stimulus
GO:0070252	0.00411243099306515	23.1903225806452	0.0959103176250778	2	42	actin-mediated cell contraction
GO:0050670	0.00424780202510783	10.1255319148936	0.328835374714553	3	144	regulation of lymphocyte proliferation
GO:0046888	0.00430690018207738	22.6231313926043	0.0981938966161511	2	43	negative regulation of hormone secretion
GO:0032944	0.00433083971768706	10.0535211267606	0.331118953705626	3	145	regulation of mononuclear cell proliferation
GO:0048871	0.00433083971768706	10.0535211267606	0.331118953705626	3	145	multicellular organismal homeostasis
GO:0032623	0.00450557121108316	22.0829493087558	0.100477475607224	2	44	interleukin-2 production
GO:0070663	0.00476087008678795	9.70816326530612	0.342536848660992	3	150	regulation of leukocyte proliferation
GO:0008015	0.00480145424207656	6.51320493599354	0.691924434295204	4	303	blood circulation
GO:0048545	0.00480145424207656	6.51320493599354	0.691924434295204	4	303	response to steroid hormone stimulus
GO:0003013	0.00485769549577889	6.49103448275862	0.694208013286278	4	304	circulatory system process
GO:0010565	0.0051229100949274	9.44834437086093	0.351671164625285	3	154	regulation of cellular ketone metabolic process
GO:0015850	0.00534189439482383	20.1570827489481	0.109611791571518	2	48	organic alcohol transport
GO:0016052	0.00550119277722173	9.20193548387097	0.360805480589579	3	158	carbohydrate catabolic process
GO:0001776	0.00556129065102666	19.7268359643102	0.111895370562591	2	49	leukocyte homeostasis
GO:0015908	0.00647957616206189	18.1745730550285	0.121029686526884	2	53	fatty acid transport
GO:0044275	0.00671922984530893	17.8238213399504	0.123313265517957	2	54	cellular carbohydrate catabolic process
GO:0045638	0.00671922984530893	17.8238213399504	0.123313265517957	2	54	negative regulation of myeloid cell differentiation
GO:0050871	0.00671922984530893	17.8238213399504	0.123313265517957	2	54	positive regulation of B cell activation
GO:0009756	0.00683557664080414	225.25	0.00685073697321985	1	3	

carbohydrate mediated signaling
GO:0015825 0.00683557664080414 225.25 0.00685073697321985 1 3
L-serine transport
GO:0021562 0.00683557664080414 225.25 0.00685073697321985 1 3
vestibulocochlear nerve development
GO:0032226 0.00683557664080414 225.25 0.00685073697321985 1 3
positive regulation of synaptic transmission, dopaminergic
GO:0033026 0.00683557664080414 225.25 0.00685073697321985 1 3
negative regulation of mast cell apoptotic process
GO:0033034 0.00683557664080414 225.25 0.00685073697321985 1 3
positive regulation of myeloid cell apoptotic process
GO:0033211 0.00683557664080414 225.25 0.00685073697321985 1 3
adiponectin-mediated signaling pathway
GO:0045715 0.00683557664080414 225.25 0.00685073697321985 1 3
negative regulation of low-density lipoprotein particle receptor biosynthetic process
GO:0045759 0.00683557664080414 225.25 0.00685073697321985 1 3
negative regulation of action potential
GO:0046544 0.00683557664080414 225.25 0.00685073697321985 1 3
development of secondary male sexual characteristics
GO:0060084 0.00683557664080414 225.25 0.00685073697321985 1 3
synaptic transmission involved in micturition
GO:0060375 0.00683557664080414 225.25 0.00685073697321985 1 3
regulation of mast cell differentiation
GO:0070666 0.00683557664080414 225.25 0.00685073697321985 1 3
regulation of mast cell proliferation
GO:0070668 0.00683557664080414 225.25 0.00685073697321985 1 3
positive regulation of mast cell proliferation
GO:0071639 0.00683557664080414 225.25 0.00685073697321985 1 3
positive regulation of monocyte chemotactic protein-1 production
GO:0072244 0.00683557664080414 225.25 0.00685073697321985 1 3
metanephric glomerular epithelium development
GO:0072248 0.00683557664080414 225.25 0.00685073697321985 1 3
metanephric glomerular visceral epithelial cell differentiation
GO:0072249 0.00683557664080414 225.25 0.00685073697321985 1 3
metanephric glomerular visceral epithelial cell development
GO:0072312 0.00683557664080414 225.25 0.00685073697321985 1 3
metanephric glomerular epithelial cell differentiation
GO:0072313 0.00683557664080414 225.25 0.00685073697321985 1 3
metanephric glomerular epithelial cell development
GO:1901164 0.00683557664080414 225.25 0.00685073697321985 1 3
negative regulation of trophoblast cell migration
GO:2000467 0.00683557664080414 225.25 0.00685073697321985 1 3
positive regulation of glycogen (starch) synthase activity
GO:0006790 0.00695592397448554 8.43136094674556 0.392775586464605
3 172 sulfur compound metabolic process
GO:0046651 0.00695592397448554 8.43136094674556 0.392775586464605
3 172 lymphocyte proliferation
GO:0032368 0.00696287953114553 17.4863055386488 0.125596844509031
2 55 regulation of lipid transport
GO:0045639 0.00696287953114553 17.4863055386488 0.125596844509031
2 55 positive regulation of myeloid cell differentiation
GO:0032943 0.00718064994085068 8.33157894736842 0.397342744446751
3 174 mononuclear cell proliferation
GO:0030048 0.00797707204132772 16.2546689303905 0.134731160473324
2 59 actin filament-based movement
GO:0046942 0.008001113998289 8 0.413327797384264 3 181
carboxylic acid transport
GO:0070661 0.00812266346310296 7.95474860335196 0.415611376375337
3 182 leukocyte proliferation
GO:0015849 0.00824530508517664 7.91 0.417894955366411 3 183
organic acid transport
GO:0030856 0.00850766980920513 15.7014762165118 0.13929831845547

2	61	regulation of epithelial cell differentiation
GO:0005977	0.00877878079102428	15.4387096774194	0.141581897446543
2	62	glycogen metabolic process
GO:0006096	0.00877878079102428	15.4387096774194	0.141581897446543
2	62	glycolysis
GO:0006869	0.00887497436532377	7.69351351351351	0.429312850321777
3	188	lipid transport
GO:0006073	0.00905374255208205	15.1845584346906	0.143865476437617
2	63	cellular glucan metabolic process
GO:0007588	0.00905374255208205	15.1845584346906	0.143865476437617
2	63	excretion
GO:0044042	0.00905374255208205	15.1845584346906	0.143865476437617
2	63	glucan metabolic process
GO:0000255	0.00910401697654961	150.15625	0.00913431596429313	1
4	allantoin metabolic process
GO:0001661	0.00910401697654961	150.15625	0.00913431596429313	1
4	conditioned taste aversion
GO:0006003	0.00910401697654961	150.15625	0.00913431596429313	1
4	fructose 2,6-bisphosphate metabolic process
GO:0006549	0.00910401697654961	150.15625	0.00913431596429313	1
4	isoleucine metabolic process
GO:0010694	0.00910401697654961	150.15625	0.00913431596429313	1
4	positive regulation of alkaline phosphatase activity
GO:0015793	0.00910401697654961	150.15625	0.00913431596429313	1
4	glycerol transport
GO:0021631	0.00910401697654961	150.15625	0.00913431596429313	1
4	optic nerve morphogenesis
GO:0032329	0.00910401697654961	150.15625	0.00913431596429313	1
4	serine transport
GO:0033023	0.00910401697654961	150.15625	0.00913431596429313	1
4	mast cell homeostasis
GO:0033024	0.00910401697654961	150.15625	0.00913431596429313	1
4	mast cell apoptotic process
GO:0033025	0.00910401697654961	150.15625	0.00913431596429313	1
4	regulation of mast cell apoptotic process
GO:0035788	0.00910401697654961	150.15625	0.00913431596429313	1
4	cell migration involved in metanephros development
GO:0035789	0.00910401697654961	150.15625	0.00913431596429313	1
4	metanephric mesenchymal cell migration
GO:0046449	0.00910401697654961	150.15625	0.00913431596429313	1
4	creatinine metabolic process
GO:0061450	0.00910401697654961	150.15625	0.00913431596429313	1
4	trophoblast cell migration
GO:0070662	0.00910401697654961	150.15625	0.00913431596429313	1
4	mast cell proliferation
GO:0070970	0.00910401697654961	150.15625	0.00913431596429313	1
4	interleukin-2 secretion
GO:1901163	0.00910401697654961	150.15625	0.00913431596429313	1
4	regulation of trophoblast cell migration
GO:2000589	0.00910401697654961	150.15625	0.00913431596429313	1
4	regulation of metanephric mesenchymal cell migration
GO:0048511	0.00913456471449922	7.61016042780749	0.433880008303924
3	190	rhythmic process
GO:0042102	0.00990155387085299	14.4697580645161	0.150716213410837
2	66	positive regulation of T cell proliferation
Tissue: Whole_Blood=>Nerve_Tibial	Type:	cluster
SourceGene:	CTD-2313J17.1(ENSG00000258922.1)
TargetGeneSet:	CHCHD2P6	HSPB7	CCT8P1	RP11	CHRNB2	DPT	ACVR1C
KRT8P15	GPBAR1	DES	SLC19A3	TIMP4	ADIPOQ	ADIPOQ-AS1	LVRN	PPARGC1B
MLXIPL	AQP9	LRRC37A5P	AGPAT2	C10orf71	RBP4	GPAM	ACSL5
LGALS12	RPS3AP40	MYEOV	GPD1	PPP1R1A	ACACB	MYH7	PLIN1	TUSC5
STAT5A	CA4	SCN4A	CIDEA	PLIN4	PLIN5	SLC7A10	LIPE	FTCD-AS1
SULT4A1	GYG2	PFKFB1

GOBPID	Pvalue	OddsRatio	ExpCount	Count	Size	Term
GO:0006641	7.95965961852856e-08	34.6038647342995	0.223790741125182	6	98	triglyceride metabolic process
GO:0006639	1.07255759533129e-07	32.8087056128293	0.235208636080548	6	103	acylglycerol metabolic process
GO:0006638	1.13639611300147e-07	32.4716553287982	0.237492215071621	6	104	neutral lipid metabolic process
GO:0019216	2.24183935298213e-07	20.4888934594817	0.443014324268217	7	194	regulation of lipid metabolic process
GO:0009749	4.26731426718578e-06	24.8179611650485	0.246626531035914	5	108	response to glucose stimulus
GO:0009746	5.33018893176607e-06	23.6607142857143	0.258044425991281	5	113	response to hexose stimulus
GO:0034284	5.80925195704711e-06	23.2272727272727	0.262611583973427	5	115	response to monosaccharide stimulus
GO:0019432	6.09748631581001e-06	40.4475721323012	0.121029686526884	4	53	triglyceride biosynthetic process
GO:0046460	7.07899897634248e-06	38.8559837728195	0.125596844509031	4	55	neutral lipid biosynthetic process
GO:0046463	7.07899897634248e-06	38.8559837728195	0.125596844509031	4	55	acylglycerol biosynthetic process
GO:0010876	8.00881384652893e-06	15.1079213184476	0.490969483080756	6	215	lipid localization
GO:0009743	1.13902959533377e-05	20.0942069741282	0.301432426821673	5	132	response to carbohydrate stimulus
GO:0043434	2.01369832473102e-05	10.0821538461538	0.872327174589994	7	382	response to peptide hormone stimulus
GO:0046486	2.30751876697959e-05	12.4418093983311	0.59144695868798	6	259	glycerolipid metabolic process
GO:0006112	3.25040083304447e-05	16.0141509433962	0.374506954536018	5	164	energy reserve metabolic process
GO:0006091	4.17181105858948e-05	8.95112369815458	0.977371808179365	7	428	generation of precursor metabolites and energy
GO:0006631	4.18734110600985e-05	11.1394799054374	0.657670749429105	6	288	fatty acid metabolic process
GO:0045834	5.46501790211906e-05	22.4608150470219	0.210089267178742	4	92	positive regulation of lipid metabolic process
GO:0044262	7.06567202148421e-05	13.5163373860182	0.440730745277143	5	193	cellular carbohydrate metabolic process
GO:0006006	7.97540034018014e-05	13.1615470022206	0.45214864023251	5	198	glucose metabolic process
GO:0046889	0.00013480857295596	35.0658536585366	0.100477475607224	3	44	positive regulation of lipid biosynthetic process
GO:0019318	0.00017482274792078	11.0644104803493	0.534357483911148	5	234	hexose metabolic process
GO:0009744	0.000180239911948168	132.820276497696	0.0205522109196595	2	9	response to sucrose stimulus
[truncated: 223,961 more chars]
